# Gene coexpression networks reveal key drivers of phenotypic divergence in porcine muscle

**DOI:** 10.1186/s12864-015-1238-5

**Published:** 2015-02-05

**Authors:** Xiao Zhao, Zhao-Yang Liu, Qing-Xin Liu

**Affiliations:** Laboratory of Developmental Genetics, Shandong Agricultural University, Tai’an, Shandong China

**Keywords:** Muscle, Modules, Weighted gene coexpression network analysis, Phenotype variation, Artificial selection

## Abstract

**Background:**

Domestication of the wild pig has led to obese and lean phenotype breeds, and evolutionary genome research has sought to identify the regulatory mechanisms underlying this phenotypic diversity. However, revealing the molecular mechanisms underlying muscle phenotype variation based on differentially expressed genes has proved to be difficult. To characterize the mechanisms regulating muscle phenotype variation under artificial selection, we aimed to provide an integrated view of genome organization by weighted gene coexpression network analysis.

**Results:**

Our analysis was based on 20 publicly available next-generation sequencing datasets of lean and obese pig muscle generated from 10 developmental stages. The evolution of the constructed coexpression modules was examined using the genome resequencing data of 37 domestic pigs and 11 wild boars. Our results showed the regulation of muscle development might be more complex than had been previously acknowledged, and is regulated by the coordinated action of muscle, nerve and immunity related genes. Breed-specific modules that regulated muscle phenotype divergence were identified, and hundreds of hub genes with major roles in muscle development were determined to be responsible for key functional distinctions between breeds. Our evolutionary analysis showed that the role of changes in the coding sequence under positive selection in muscle phenotype divergence was minor.

**Conclusions:**

Muscle phenotype divergence was found to be regulated by the divergence of coexpression network modules under artificial selection, and not by changes in the coding sequence of genes. Our results present multiple lines of evidence suggesting links between modules and muscle phenotypes, and provide insights into the molecular bases of genome organization in muscle development and phenotype variation.

**Electronic supplementary material:**

The online version of this article (doi:10.1186/s12864-015-1238-5) contains supplementary material, which is available to authorized users.

## Background

Wild ancestors of the pig (*Sus scrofa*) are still alive, providing an excellent model for tracing their evolutionary history and for defining the evolutionary mechanism driven by artificial selection during domestication [1]. Pigs were first domesticated approximately 9,000 years ago [1-3]. The domestication of pigs occurred independently in various parts of the word [2-7] and historically, Europe and China are the two major areas of pig breeding [8]. More than 730 pig breeds or lines have undergone natural and artificial selection in different environments, especially catering to the distinct needs of humans, which has provided the large diversity of morphological and physiological characteristics that currently exist worldwide [5,9,10]. For example, the lean and muscular Landrace (Lde) type in Europe and the high fat deposition and thin muscle fibers of the Lantang (LT) type in China [11]. The lean (Lde) and obese (LT) pig breeds have been found to have significant differences in their genetic of muscle growth rate and fatness [11]. Lde is characterized by a high lean meat percentage, fast-growing muscle and high body weight [12,13], while LT, an obese pig breed indigenous to China, is characterized by high intramuscular fat content, slow-growing muscle, and low body weight [11]. Significant genome and transcriptome differences have been revealed by comparative genomic studies [2,11,13]. However, the mechanisms underlying the morphological variations in muscle among pig breeds are still unclear. Generally, it is has been reported that changes in gene expression and regulatory interaction networks rather than genetic changes that result in changes to the amino acid sequences of proteins that account for the phenotype differences among species [14]. Therefore, the identification of gene expression regulatory networks in pig breeds with distinct muscle phenotypes is necessary to understand how muscle has been modified during pig domestication.

Strong selective pressures though the artificial selection of domestication have caused rapid phenotype evolution and major changes in the morphological architectures of pig muscle [1,2,7,15]. The Lde and LT breeds, which have distinct muscle phenotypes, were domesticated under different breeding goals in Europe and Asia [2,5,11]. Thus, artificial selection was probably critical in modifying the gene expression regulatory networks that resulted in muscle phenotype divergence. To better understand gene expression network differences in muscle development between the Lde and LT breeds, we applied a global network approach using weighted gene coexpression network analysis (WGCNA) [14,16-20]. WGCNA elucidates the higher-order relationships between groups of genes coexpressed with high topological overlap across samples, which are termed “modules”. A module is a pairwise measure of the similarity of the coexpression relationships of two genes with all other genes in a network. The topological overlap of paired proteins in gene coexpression networks was significantly higher for physically interacting protein pairs compared with pairs that did not interact. Thus, WGCNA screens for the core functional units of transcriptional networks. WGCNA also identifies the statistical significant enrichments of genes with the highest degree of connectivity within each module, referred to as “hub genes”. Hub genes are expected to play critical roles in the coexpression network of each module [14,16-21]. Thus, a comprehensive analysis of gene coexpression relationships in different muscle phenotypes provides an efficient way of exploring the genetic basis of phenotype variation. In this way, we used this approach to identify and visualize modules of coexpressed genes, which were organized into modules of coexpressed genes with clear functional interpretations, and to explore module differences between breeds. We identified modules of coexpressed genes, which corresponded to muscle phenotypes, and determined the hub genes responsible for the key functional distinctions between breeds. Our results demonstrated that the molecular mechanism underlying phenotype divergence between breeds cannot be robustly explained by differential gene expression alone but can be explained by coexpression network modules. We also showed that muscle phenotype differences between the Lde and LT breeds were not regulated by the muscle genes alone but by the coordinated action of muscle, nerve, and immunity genes. Thus, our results indicated that the regulation of muscle development were more complex than previously acknowledged. The evolutionary rates of most modules were accelerated, implying that complex species-specific coexpression networks underlie artificial selection during domestication. These findings are important in elucidating the molecular mechanisms that underlie muscle development and phenotype variation, and reveal the potential impact of evolutionary changes at the coexpression network level.

## Results

### Gene coexpression networks in lean and obese pig muscle

**Figure 1:**
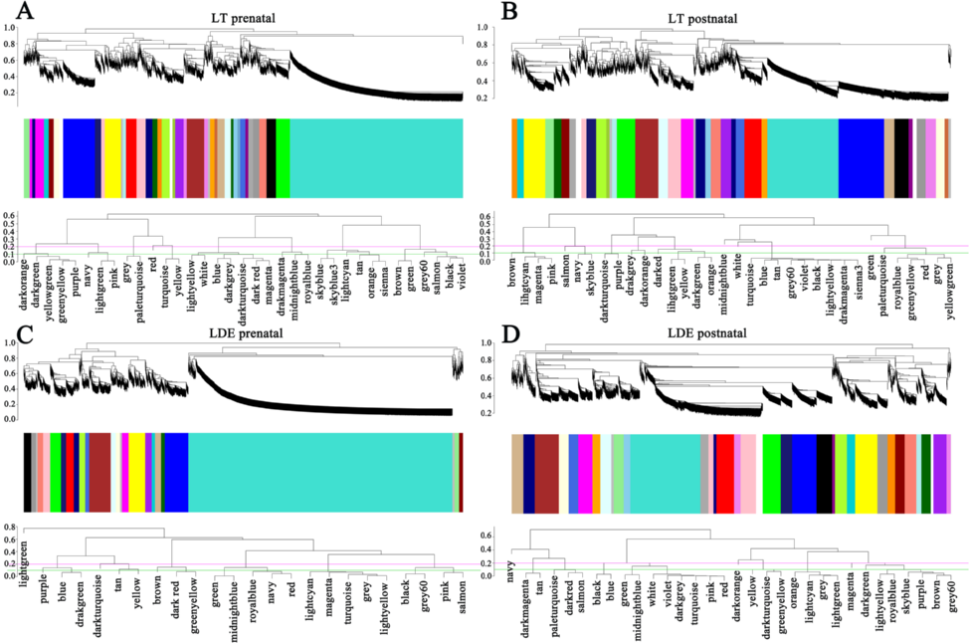
**Gene coexpression networks in lean (Lde) and obese (LT) pig muscle.** The Lde prenatal network **(A)**, Lde postnatal network **(B)**, LT prenatal network **(C)**, and LT postnatal network **(D)** are shown. The dendrograms were produced by average linkage hierarchical clustering of genes on the basis of topological overlap. The y axes correspond to co-expression distance and the x axis to genes. Dynamic tree cutting was used to determine modules, generally by dividing the dendrogram at significant branch points. The modules of coexpressed genes were assigned colors and numbers as indicated by the horizontal bar beneath each dendrogram. The y-axes correspond to co-expression distance and the x axis to genes. See also Additional file 1: Table S1.

To investigate coexpression networks that comprehensively represent muscle transcription during pig development, we constructed gene coexpression networks from 20 next-generation sequencing data sets generated by Solexa/Illumina’s genome sequencing technology [11]. The 20 data sets comprised 10 LT and 10 Lde data sets, each of which contained muscle transcriptomes data at 35, 49, 63, 77, 91 days post-coitus and at 2, 28, 90, 120, 180 days post-natum [11]. The gene expression levels in each sample were assessed using 3’ digital gene expression tag-based profiling [11]. A total of 3652 and 3404 temporally differentially expressed genes (DEGs) were identified during LT prenatal and postnatal muscle development, respectively. Similarly, 3649 and 3408 DEGs were identified from Lde prenatal and postnatal muscle, respectively. Weighted Pearson correlations were calculated for all 3652 and 3404 DEGs in LT, and for all 3649 and 3408 DEGs in Lde. All the weighted Pearson correlations were converted into matrices of connection strength by a power function [22]. The topological overlaps between genes were then calculated using these connection strengths. Topological overlaps values were used to assess the similarity of the coexpression relationship of two genes with all the other genes in the network in a robust and biologically meaningful way [22,23]. Average linkage hierarchical clustering was used to cluster coexpressed genes with similar patterns of connection strengths or with high topological overlaps into modules. In all, we identified 24 modules in the Lde prenatal network (Figure 1A), 32 in the Lde postnatal network (Figure 1B), 35 in the LT prenatal network (Figure 1C) and 34 in the LT postnatal network (Figure 1D) (Additional file 1: Table S1 and Table S2).

### Coexpression network modules between lean and obese breeds are more different in postnatal animals than in prenatal animals

**Figure 2:**
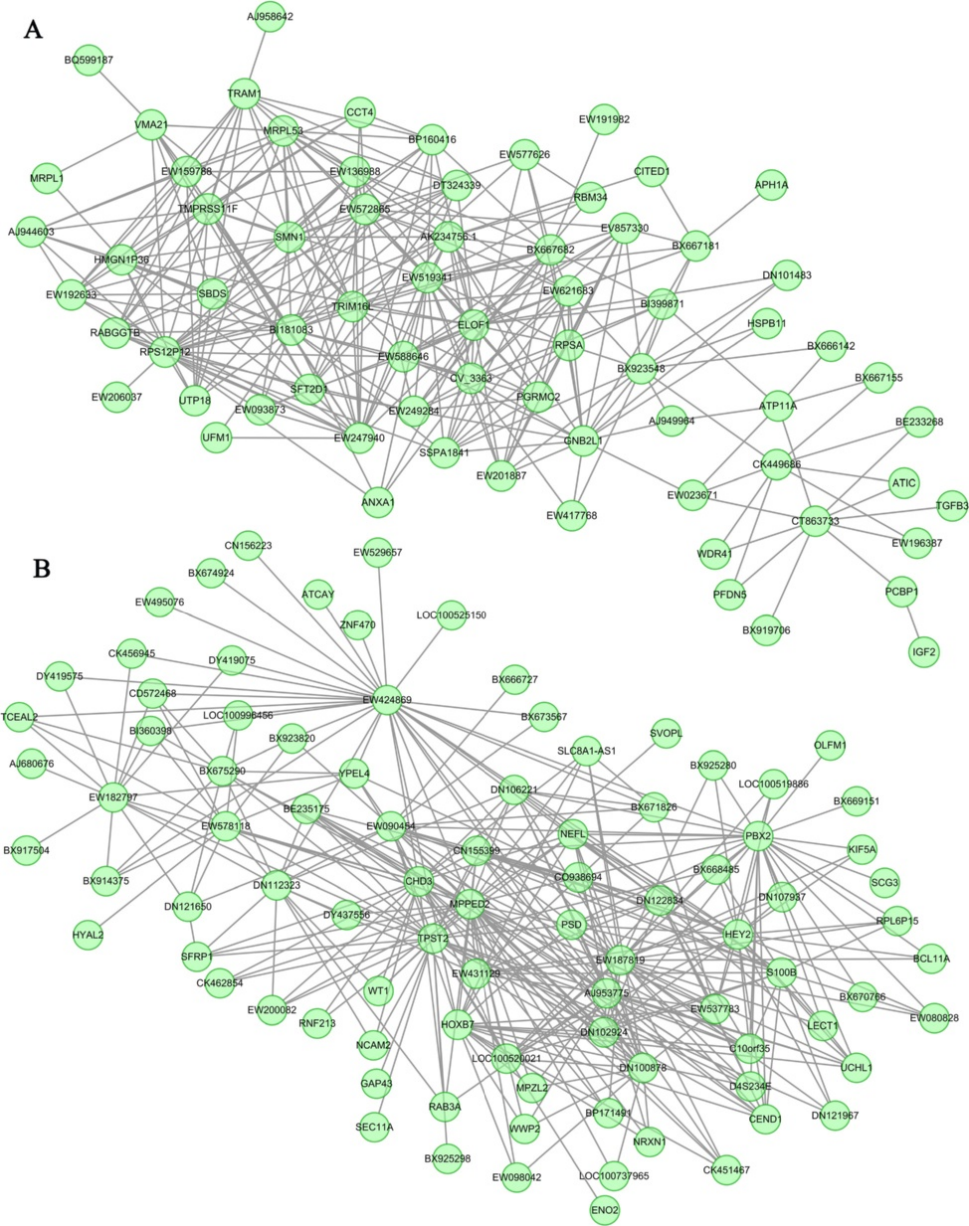
**Visualization of the common gene coexpression network modules to identify hub genes.** The eigengene in the common modules between the LT-blue and Lde-brown **(A)** and between LT-turquoise and Lde-turquoise **(B)** modules are shown. The top 300 connections are shown for each module. Dots correspond to genes and lines to connections; hubs genes have at least 15 connections. Where the gene symbols are unknown, gene IDs are shown (e.g., WE424869). See also Additional file 1: Table S4.

To determine the preservation of coexpression network modules between different muscle types, we assessed whether different modules were composed of the same genes on a module-by-module basis. A high degree of module preservation between the prenatal animals was observed by calculating the overlap for each possible pair of modules (Additional file 1: Table S3). Two pairs of modules were deemed to show significant preservation when the gene coexpression relationships were > 50% overlap. In the LT-turquoise and Lde-turquoise module pair (P < 0.001), we identified about 1021 overlapping genes: 47% (1021/2183) in Lde-turquoise; and 72% (1021/1417) in LT-turquoise. In the LT-blue and Lde-brown module pair (P < 0.001), we identified about 100 overlapping genes: 38% (100/261) in LT-blue and 56% (100/177) in Lde-brown. Overalll, these two coexpression network modules with 1121 genes (31% of all module genes) were highly preserved in the LT and Lde prenatal animals (Additional file 1: Table S4). The Gene Ontology (GO) annotations assigned to the genes indicated that most of the 1121 genes were involved in cell differentiation and growth, muscle and skeletal system development, neuron development, and cellular response (Additional file 1: Table S5). Because the “hub genes” have the highest degree of within-module connectivity, they were expected to play critical roles in the coexpression network modules and were therefore considered to be a primary indicator of the module function [14,16-21]. We identified the hub genes by visualizing the preserved coexpression network modules (Figure 2). In the blue-brown module (Figure 2A), the hub genes included *SMN1*, which has been shown to be crucial in neurite outgrowth and neuromuscular maturation during the differentiation and development of neurons and muscle [24]; *GNB2L1* [25] and *SBDS* [26], which may be involved in cell division and growth (Additional file 1: Table S5); and *ELOF1*, a conserved transcription elongation factor [27]. In the turquoise-turquoise module (Figure 2B), the hub genes included *HOXB7*, *HEY2* and *PBX2*, which have been reported to regulate muscle development [28-30]; and *MPPED2* and *NEFL*, which may play roles in neuronal differentiation [31,32]. Between the postnatal animals the degree of module preservation was much lower than was found between the prenatal pigs. Indeed, only two modules containing a total of 101 genes (1.5% of all module genes) were common between the LT and Lde postnatal animals (Additional file 1: Table S4). Thus, the coexpression network modules were more conserved in prenatal than in postnatal animals, and muscle related genes were found to play key roles in most of the preserved coexpression network modules.

### Differences in prenatal modules between lean and obese breeds provide insight into prenatal muscle development differences in fiber number and muscle fiber composition

**Figure 3:**
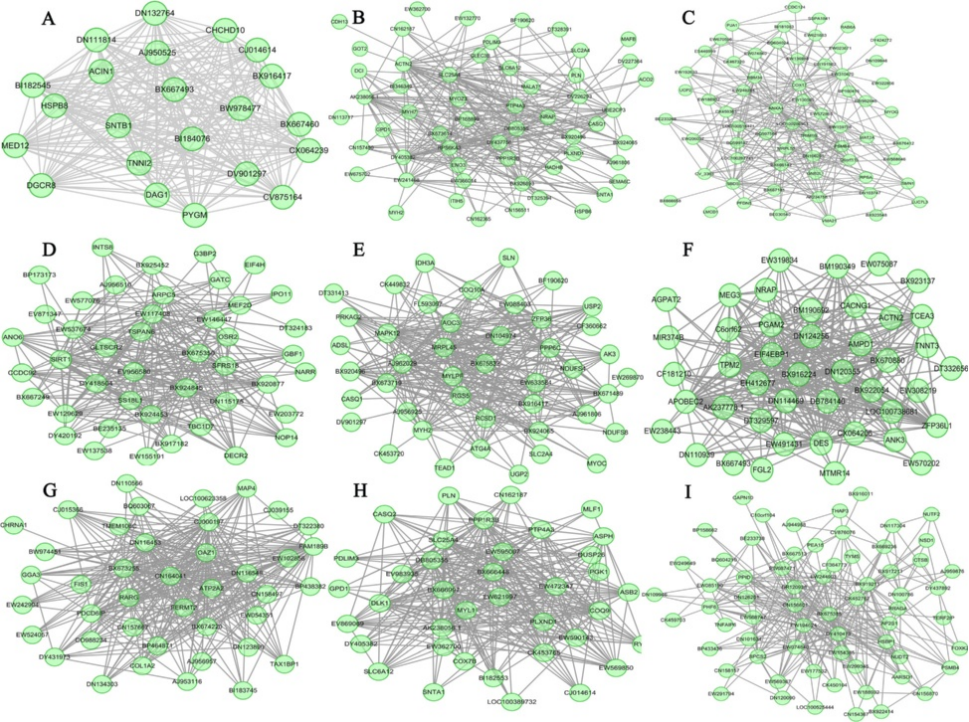
**Visualization of breed-specific gene coexpression networks in prenatal animals. (A)** LT-yellow green **(B)** LT-purple **(C)** LT-blue **(D)** Lde-tan **(E)** LDE-red **(F)** Lde-pink **(G)** Lde-turquoise **(H)** Lde-midnight blue **(I)** Lde-blue. The top 300 connections are shown for each module. Dots correspond to genes and lines to connections; hubs genes have at least 15 connections. Where the gene symbols are unknown, gene IDs are shown.

Differences in transcriptional levels are important for studying the evolutionary basis of phenotypic differences at the molecular level [18]. Differences in network modules could provide a basis for better understanding of the differences in muscle development between lean and obese pigs. In this study, we identified six highly lean-specific modules and five highly obese-specific modules in the prenatal animals (Additional file 1: Table S6 and Table S7). A GO analysis of these module genes revealed that nine of these modules were involved in muscle development, neuron development and cellular response (Additional file 1: Table S8 and Table S9). Hub genes involved in muscle development were enriched in six lean-specific modules (*HSBP1* in Lde-blue; *MYL1* and *DLK1* in Lde-midnight blue; *MAP4* and *FERMT2* in Lde-only-turquoise; *TPM2*, *TCEA3*, *ZFP36L1*, *DES*, *TNNT3,* and *ANK3* in Lde-pink; *MAPK12*, *MYLPF*, and *MYH2* in Lde-red; and *SIRT1*, *OSR2,* and *MEF2D* in Lde-tan) and in three obese-specific modules (*GNB2L1* in LT-blue; *ACTN2*, *MYH7*, *MYOZ3*, *MALAT1*, *PTP4A3,* and *ENO3* in LT-purple, and *TNNI2* and *DAG1* in LT-yellow green) (Figure 3). The hub genes in the LT-dark red module were significantly enriched for genes involved in cellular response (*RRAGD*, *EPHX1*, *TPD52* and *PSMA2*). Overall, a greater number of muscle development-related modules that regulate fiber number and muscle fiber composition were identified in lean Lde animals than in obese LT animals.

### Differences in postnatal modules between lean and obese breeds provide insight into differences in postnatal muscle growth and fat deposition

**Figure 4:**
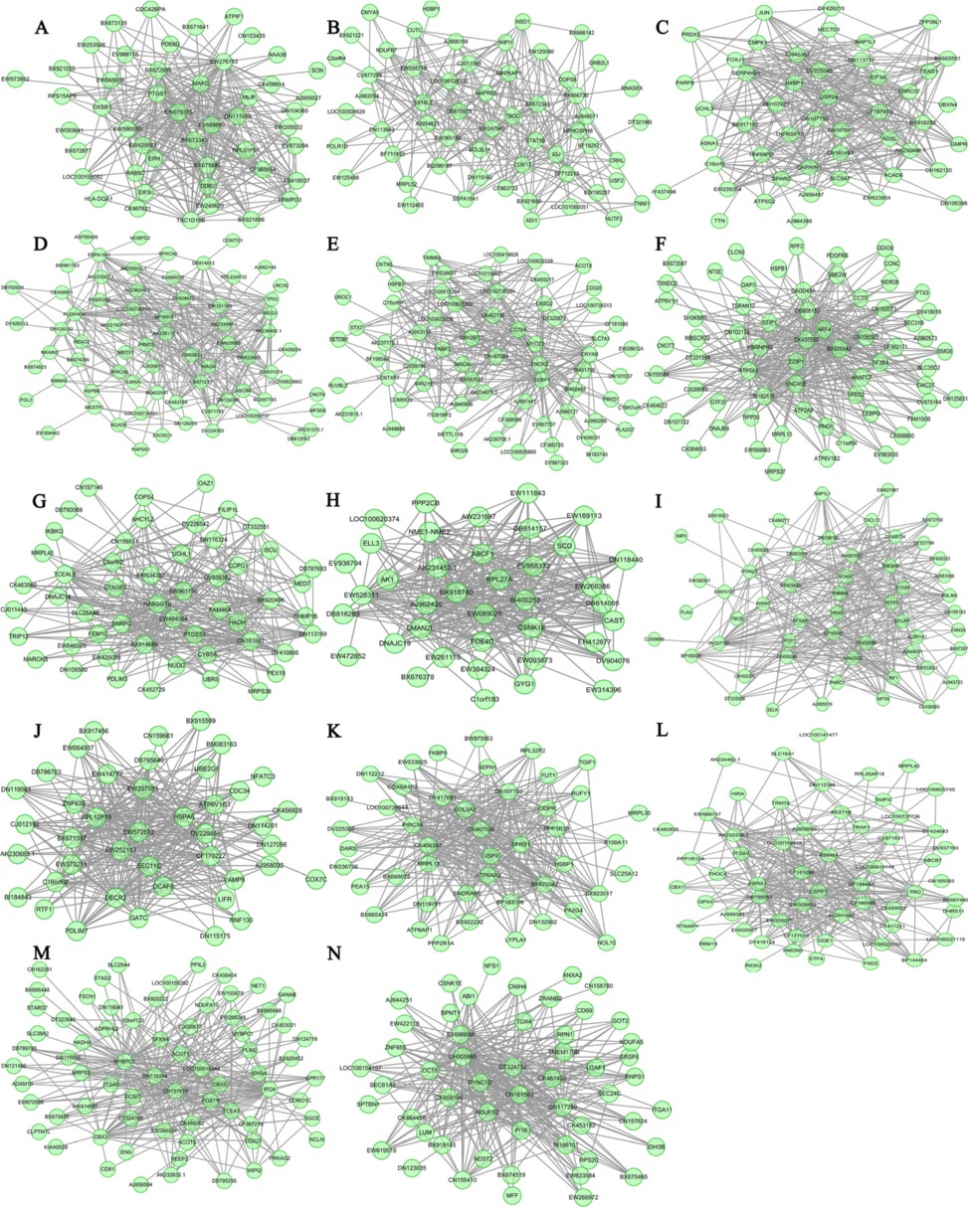
**Visualization of Lde-specific gene coexpression networks in postnatal animals. (A)** Lde-yellow **(B)** Lde-salmon **(C)** Lde-sky blue **(D)** Lde-red **(E)** Lde-pink **(F)** Lde-midnight blue **(G)** Lde-royal blue **(H)** Lde-purple **(I)** Lde-light yellow **(J)** Lde-grey60 **(K)** Lde-green **(L)** Lde-dark grey **(M)** Lde-blue **(N)** Lde-black. The top 300 connections are shown for each module. Dots correspond to genes and lines to connections; hubs genes have at least 15 connections. Where the gene symbols are unknown, gene IDs are shown.

**Figure 5:**
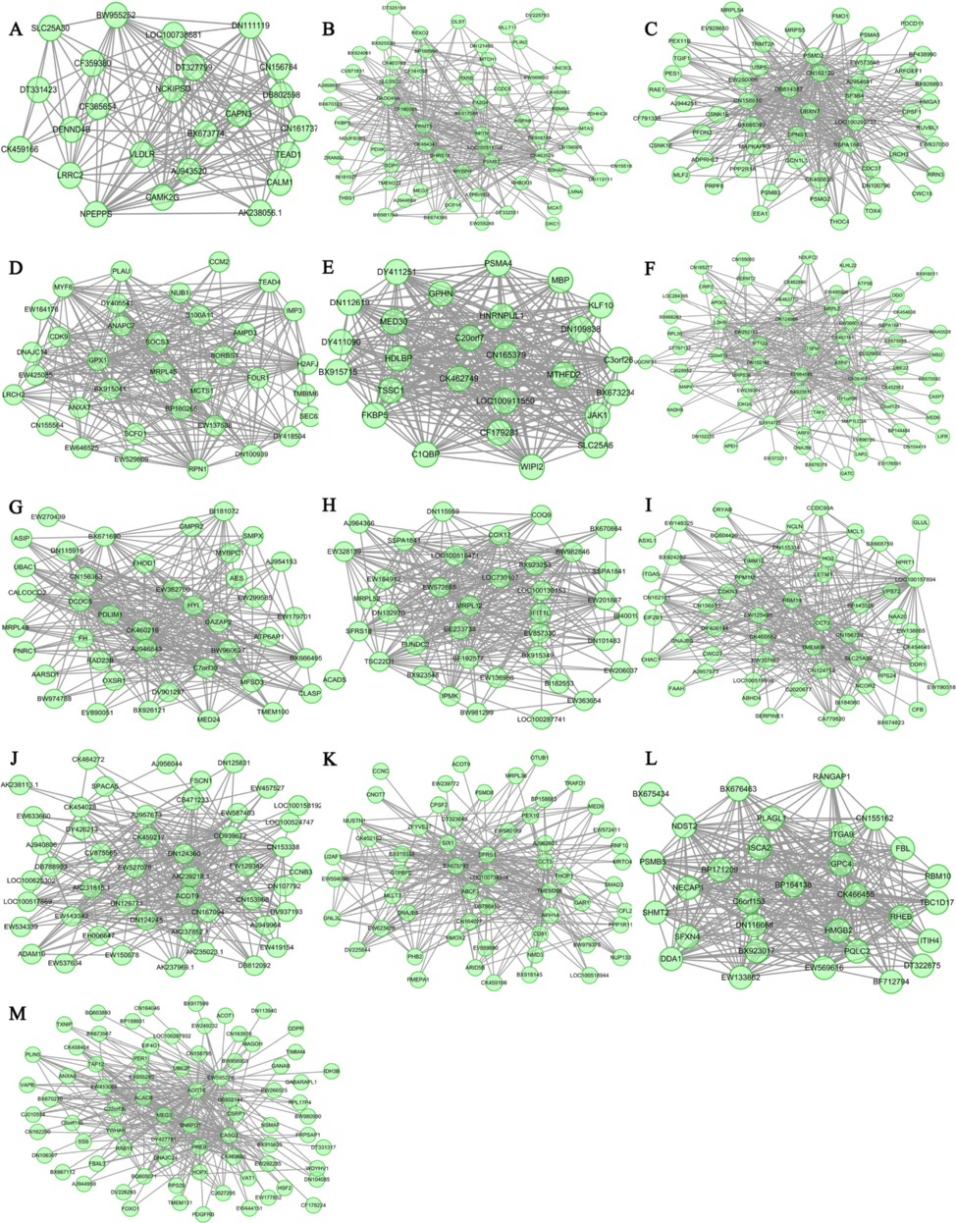
**Visualization of the LT-specific gene coexpression networks in postnatal animals. (A)** LT-yellow green **(B)** LT-violet **(C)** LT-tan **(D)** LT-sky blue **(E)** LT-sienna3 **(F)** LT-red **(G)** LT-orange **(H)** LT-magenta **(I)** LT-light yellow **(J)** LT-light green **(K)** LT-grey60 **(L)** LT-dark magenta **(M)** LT-black. The top 300 connections are shown for each module. Dots correspond to genes and lines to connections; hubs genes have at least 15 connections. Where the gene symbols are unknown, gene IDs are shown.

Only two modules were common between lean and obese postnatal animals; however, about 15 highly lean-specific modules and 13 highly obese-specific modules were identified (Additional file 1: Table S10 and Table S11). GO analysis of these module genes revealed that 18 of these modules were involved in muscle development, neuron development, and cellular response, and three were enriched in cellular response and metabolism (Additional file 1: Table S12 and Table S13). Hub genes involved in muscle development were enriched in 13 lean-specific modules (*MYBPC1* and *CBX3* in Lde-blue; *PRRX1* in Lde-dark grey; *USP2* in Lde-green; *PDLIM7* in Lde-grey60; *VCAM1*, *CXCL12*, *HRAS*, *SETD3,* and *MYLPF* in Lde-light yellow; *UNC45B* and *DZIP1* in Lde-midnight blue; *MYOZ2* and *FABP3* in Lde-pink; *LMNA* and *PRMT5* in LDE-red; *UBR5* in Lde-royal blue; *JUN, SPARC,* and *TEAD1* in Lde-sky blue; *STAT5B* and *GNB2L1* in Lde-salmon; *MLIP* in Lde-yellow; and *ELL3* and *RPL27A* in Lde-purple). The hub genes in the Lde-black module were significantly enriched for genes involved in the regulation of alternative splicing (*ZRANB2*, *RNPS1*, and *SRSF6*) (Figure 4). In addition, 18 muscle development hub genes were identified in the 11 obese-specific modules (*RHEB* in LT-dark magenta;; *SIX1*, *MUSTN1,* and *SFRS1* in LT-grey60; *FHOD1* and *SMPX* in LT-orange; *KLF10*, *HDLBP,* and *JAK1* in LT-sienna3; *MYF6*, *CDK9*, *TEAD4,* and *S100A11* in LT-sky blue; *GADD45A* and *PRMT5* in LT-violet; *TEAD1* in LT-yellow green; *SFRS18* in LT-magenta; *ATP5B* in LT-red; *MCL1*, *CDKN3,* and *RBM19* in LT-light yellow; and *SPNS1* in LT-tan). In particular, hub genes involved in intramuscular fat deposition and meat quality were significantly enriched in five obese-specific modules (*SFRS18* in LT-magenta; *ATP5B* in LT-red; *ACOT9* in LT-light green; *ACOT8* and *CSRP1* in LT-black; and *HDLBP* in LT-sienna3) (Figure 5). Thus, difference between lean- and obese- specific modules in the postnatal animals provided insights into differences in postnatal muscle growth and fat deposition in the LT and Lde pigs.

### Regulation of muscle development is coordinated by muscle, nerve, and immunity genes

Among the 42 modules mentioned above (i.e., the three common modules and the 39 breed-specific modules), 24 contained genes related to muscle development, nervous system development, and immune response, seven contained genes related to muscle development and immune response, and another three contained genes related to muscle development and nervous system development (Additional file 1: Table S5, Table S8, Table S9, Table S12 and Table S13). Many neuron and immune response genes played crucial roles in the coexpression network modules of muscle (Figures 2, 3, 4 and 5). This finding suggests that the regulation of muscle development might be more complex than previously acknowledged, because our results suggest that the muscle development process may be regulated not only by muscle genes but by the coordinated action of muscle, nerve, and immunity genes.

### Detection of positive selection pressure

**Figure 6:**
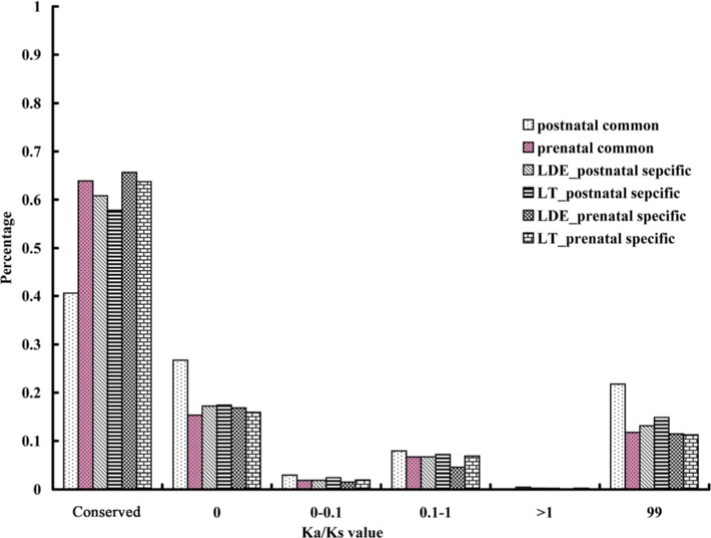
**Detection of selection pressure on all module genes.** Ka/Ks valuesare the nonsynonymous/synonymous substitution rate ratios; Conserved indicates gene sequences that are conserved and none snp detected; 0, Ka/Ks = 0; 0–0.1, 0 < Ka/Ks < 0.1; 0.1-1, 0.1 < Ka/Ks < 1; >1, Ka/Ks > 1; 99, Ka/Ks = 99. Genes with Ka/Ks ratios equal to 99 were not included in the 7% of genes with Ka/Ks ratios > 0.1 because estimates of omega equal to 99 are not reliable.

To examine the genes that showed accelerated evolution in the 42 modules, we obtained the ortholog sequences of the 4597 genes in these modules from whole-genome resequencing data of 37 individual pigs and 11 wild boars. Evolutionary rates (Ka/Ks values, nonsynonymous/synonymous substitution rate ratio) were inferred form the filtered alignments of these 4597 module genes (Additional file 1: Table S4-S7, Table S10 and Table S11). We found that 80% of these genes had Ka/Ks ratios < 0.1, indicating a high level of purifying selection pressure in these genes (Figure 6), and approximately 7% of the genes had Ka/Ks ratios >0.1 (Figure 6). Five genes under strong positive selection were identified in the prenatal common modules (Table 1), while only one of the genes under positive selection in the LT-blue module was identified in the 11 prenatal breed-specific modules (Table 1). In the postnatal modules, five genes from six breed-specific modules were found to be under strong positive selection, while no positively selected genes were found in the postnatal common modules. These genes could be involved in the regulation the basic cell biological processes, such as cell migration (*CDC42BPA*), transport (*PLTP*), proteolysis (*PLAU*), and RNA process (*SART3*) (Table 1). In particular, *CMYA5* and *FHOD1* have been reported to regulate the muscle cell phenotype and meat quality [3,33]. These results suggested that the genes under positive selection may have played a role in the muscle phenotype divergence among pig breeds. However, among these positively selected genes, only *FHOD1* was a hub gene in the postnatal LT-orange and Lde-purple modules (Table 1). A high level of purifying selection pressure was identified in 94 other muscle related hub genes. Therefore, although coding sequences changes under positive selection have a role in the evolution of gene function, their role in the muscle phenotype divergence among pig breeds seemed to be minor. The divergent of coexpression modules among breeds might regulate the muscle phenotype divergence during domestication.

**Table 1:** **Module genes under positive selection**

**Accession number**	**Gene name**	**Module name**	**GO annotation**	**Ka/Ks**
Prenatal common modules				
AK239624.1	*YWHAE*	brown-blue	interspecies interaction between organisms	4.6399
XM_001929007.1	*CDC42BPA*	turquoise	cell migration	1.0739
XM_001926685.1	*SART3*	turquoise	RNA-binding nuclear protein	1.0246
XM_001925245.1	*FAM149A*	turquoise	family with sequence similarity 149	1.0426
AK235649	*PLTP*	turquoise	transport	1.2134
Postnatal sepcific modules				
BX915506	*CMYA5*	Lde-grey60	Cardiomyopathy associated 5	1.6222
NM_213945.1	*PLAU*	Lde-lightyellow	proteolysis	1.8044
XM_001927929.1	*GBP4*	Lde-magenta	Guanylate-binding proteins	1.2581
AK235913.1	*FHOD1*	Lde-purple	the formin/diaphanous family proteins	1.2135
AK235913.1	*FHOD1*	LT-orange	the formin/diaphanous family proteins	1.2135
NM_213945.1	*PLAU*	LT-skyblue	proteolysis	1.8044
Prenatal specific modules				
AK239624.1	*YWHAE*	LT-blue	interspecies interaction between organisms	4.6399

## Discussion

During the domestication of wild boar, dramatic phenotype changes were generated in domestic pigs under artificial selection with different breeding goals. For example, the lean (Lde) and obese (LT) pig breeds have significant genetic differences in the processes associated with muscle growth rate and fatness [11]. The pig genome has been sequenced and resequenced, which has made it easier to investigate the regulatory mechanism that underlie the phenotype diversity in domestic pigs. Using the genome resequence methods, Rubin et al. [7] identified a few genes related to pig domestication that were under positive selection; however, none of these genes were related to muscle phenotype. Most previous studies have focused on changes in gene expression, while several studies have reported that connectivity was a more sensitive measure of evolutionary divergence compared with gene expression changes alone [14,16,18,19,21]. Therefore, we used WGCNA to reveal molecular and evolutionary mechanisms associated with the coordination of gene expression patterns in different pig breeds with distinct muscle phenotypes. In this study, we showed that the transcriptional diversity of different muscle phenotypes was regulated at the genome level by distinct gene coexpression networks.

Comparison of the coexpression network modules between prenatal LT and Lde animals, which were constructed using transcriptome data of five developmental stages, revealed that 1121 genes in two modules were also conserved in the LT and Lde pigs. We have highlighted seven hub genes (*SMN1*, *GNB2L1*, *SBDS*, *ELOF1*, *HOXB7*, *HEY2* and *PBX2*) that were predicted to play key roles in muscle development. SMN1 encodes a protein that is crucial in neuromuscular maturation [24]. *GNB2L1* [25] and *SBDS* [26] encode proteins that are involved in cell division and growth, which may contribute to the proliferation of muscle cells. *HOXB7*, *HEY2* and *PBX2* encode proteins that directly regulate muscle development [28-30], and *ELOF1* encodes a conserved transcription elongation factor, which might regulate the basic transcription process of muscle genes [27]. These results suggested that the conserved coexpression network modules contained genes that were associated with the regulation of basic muscle development; implying that the key processes that regulate muscle development are similar in the two breeds. Nonetheless, six highly lean-specific modules and five highly obese-specific modules were identified in the prenatal LT and Lde animals, indicating that prenatal myogenesis was significantly different in the two breeds. Most of these breed-specific modules were involved in muscle development, neuron development, and cellular response. Among the genes with known functions, 17 hub genes related to muscle development were found to play major roles in the six lean-specific modules. As an essential myogenesis regulator in many diverse species, *MEF2D* directly regulates muscle genes at all developmental stages [34]. *SIRT1* has been found to increase the cell proliferation of myoblasts [35]. *FERMT2* regulates myogenic differentiation by the myogenic factor, myogenin, via canonical Wnt signaling [36]. *DES* [37], *MAPK12* [38], *DLK1* [39] and *MAP4* [40] were reported to play essential roles in myoblast fusion, myotube formation, and maintenance of the structural and functional integrity of muscle during myogenesis. *OSR2* [41] and *TCEA3* [42] encode proteins that regulate proliferation and development genes. *ANK3* [43] and *HSBP1* [44] have been found to play critical roles in myogenesis. In the obese-specific modules, only seven key muscle related hub genes were found. Among these genes, *MALAT1* encodes a protein that was reported to regulate myoblast proliferation [45], and *ENO3* and *DAG1* [46] have both been shown to regulate myogenesis [47]. A greater number of hub genes related to myogenesis were detected in the lean-specific modules compared with in the obese-specific modules.

This might have resulted in the formation of more muscle fibers in Lde pig during embryonic development, which may explain the main phenotype difference in prenatal muscle development between the LT and Lde breeds [11]. In addition to the genes that were directly associated with myogenesis, numerous muscle fiber type related genes in the coexpression network modules were different between the LT and Lde pigs. For example, the hub genes *MYL1* [48], *TNNT3* [49], and *MYH2* [50] in the lean-specific modules, encode proteins that are critical for fast fiber differentiation, and *TPM2* [51] and *MYLPF* [52] have been reported to be critically important for fast and slow skeletal muscle development. In the obese-specific modules, the hub gene *MYH7* encodes a protein that regulates slow skeletal muscle fiber [53], while *ACTN2* [54], *TNNI2* [49], and *MYOZ3* [55] have been found to be highly expressed in fast skeletal muscle fibers, and *MYOZ3* is closely related to meat quality [55]. Total fiber number and muscle fiber composition between fast and slow muscle fibers are associated with different muscle phenotypes [56]. All these modules contain genes that can regulate differences in development of muscle fiber number, size, and fiber composition between the two breeds. Thus, these modules may be responsible for the different muscle features and meat quality in the LT and Lde pigs [11].

Previous studies have shown that muscle phenotype is determined during embryonic development and that postnatal muscle growth is not critical [11]. However, in our by coexpression network module analysis, we found that differences in transcriptional profiling between LT and Lde were more significant in postnatal animals than in prenatal animals. Only two modules that contained 101 module genes (1.5% of all module genes) were conserved in both breeds, and none of the genes were related to muscle regulation. In contrast, our analysis of 15 lean-specific modules and 13 obese-specific modules containing 2504 genes revealed a molecular regulation mechanism that was associated with the different muscle phenotype in the two breeds. Although muscle phenotype was found to be determined during embryonic development, several hub genes related to muscle development were identified in these modules. In lean-specific modules, a hub gene in Lde-salmon, *STAT5B,* was reported to be critical for normal postnatal growth [57]. *STAT5B* encodes a transcription factor that can regulate skeletal muscle growth and fiber composition. The absence of *STAT5B* has been shown to increase the expression levels of several genes that regulate type I fibers, which resulted in muscle composed almost exclusively of type II fibers [57]. Thus, *STAT5B* and *MYLPF* [52], a hub gene in the Lde-light yellow module, might be critical for muscle growth and fiber composition in postnatal development. Other hub genes, *VCAM1* [58] and *CXCL12* [59], in the Lde-light yellow module have been reported to play roles in the control of secondary muscle growth. Thus, although muscle phenotype is determined mainly during embryonic development, we found that secondary muscle growth during postnatal development was also critical for the muscle phenotype difference between LT and Lde. The hub genes *SETD3* [60], *DZIP1* [61], *LMNA* [62], *PRMT5* [63], *JUN* [64], *TEAD1* [65], *ELL3* [66] and *SPARC* [67] have been shown to control muscle cell proliferation and differentiation and regulate muscle development. Some of the hub genes that we identified have been reported to be involved in proliferation and differentiation of vascular smooth muscle cells and cardiac myocytes; for example, *CBX3* [68], *PRRX1* [69], *PDLIM7* [70], *FABP3* [71], and *UBR5* [72]. The coexpression network modules that contain these genes may regulate the development of the vascular and circulatory system. In addition, the hub genes, *MYBPC1* [73], *UNC45B* [74] and *MYOZ2* [75], have been shown to be are required for skeletal muscle function, such as muscle contraction. These results suggest that the lean-specific modules cover all the main processes of postnatal muscle development, including muscle cell proliferation and differentiation, secondary muscle growth, postnatal muscle growth for fiber composition, development of the vascular and circulatory system, and muscle function regulation. All these coexpression network modules seem to be associated with the mechanisms that regulate the high lean meat percentage muscle phenotype in Lde.

In addition to the numerous genes that were found to positively regulate muscle development in the coexpression network modules of Lde, we identified hub genes that negatively regulate muscle development in the postnatal LT modules. *RHEB* was found to negatively regulate skeletal myogenesis by repression of insulin receptor substrate 1 (*IRS1*) [76], and *KLF10* was reported to inhibit myoblast proliferation by suppression of the promoter activity of fibroblast growth factor receptor 1 [77]. Although *JAK1* was found to be critical in promoting proliferation, it was also found to prevent the premature differentiation of myoblasts [78]. In contrast, *MUSTN1* was shown to have no effect on myoblast proliferation, but was found to significantly impairs myoblast differentiation and prevent myofusion [79]. These negative coexpression network modules associated with muscle development might control the muscle phenotype in LT, which features low lean meat percentage, slow-growing muscle and low body weight. These characteristics facilitate the deposition of high levels of intramuscular fat. Besides these negative regulation modules, several positive coexpression network modules were also identified in LT. *TEAD1* was shown to regulate the fast-to-slow fiber-type transition and overexpression of *TEAD1* was found to produce a slower skeletal muscle contractile phenotype [80]. The hub genes *SIX1* [81], *MYF6* [82], *CDK9* [83], *TEAD4* [84], and *PRMT5* [63] in LT are myogenesis genes that regulate myogenic differentiation and muscle development. *FHOD1* [33] and *S100A11* [85] regulate smooth muscle cell migration, vesicular exocytosis, and smooth muscle cell phenotype, processes that are related to vascular and circulatory system development. *SMPX* [86] and *GADD45A* [87] are LT-specific muscle function genes. In particular, we have identified coexpression network modules related to intramuscular fat deposition and meat quality. For example, the hub genes *HDLBP* [88], *SFRS1* and *SFRS18* [89] can regulate the deposition of intramuscular fat, while *ACOT8* and *ACOT9* [90] can regulate lipid and amino acid metabolism. It has been suggested that fat deposition and fatty acid composition are the determining factors for meat quality [91]. *ATP5B* [92] and *CSRP1* [56] were shown to play key roles in muscle fiber development and may be responsible for breed-specific differences in meat quality.

It has been suggested that muscle fiber composition, size, and total fiber number are critical for meat quality, and that slow fibers contribute to both juiciness and tenderness [56]. These muscle fiber features also define muscle phenotypes. In our comparative transcriptome analysis, we detected a greater number of coexpression network modules related to myogenesis and muscle growth, secondary postnatal muscle growth, fast fiber differentiation, and fiber composition in the Lde transcriptome compared with in the LT transcriptome. Although fewer coexpression network modules related to myogenesis and muscle growth were identified in LT, more modules related to negative regulation of postnatal muscle and slow skeletal muscle fiber development were identified compared with Lde. In particular, coexpression network modules related to negative regulation of intramuscular fat deposition and meat quality were identified in LT. Thus, the differences in coexpression network modules between Lde and LT described above are likely to have resulted in the high lean meat percentage, fast-growing muscle, and high body weight characteristics in Lde, and the high intramuscular fat content, slow-growing muscle, and low body weight characteristics in LT. However, our results showed that the muscle phenotype differences between the two breeds were not only regulated by muscle genes but were coordinated by muscle, nerve, and immunity related genes. The complex coexpression networks responsible for the different muscle phenotypes are likely to have been generated by artificial selection during the domestication process. The evolutionary analysis showed that the coding sequences of most of the module genes in the coexpression network modules were conserved among pig breeds under artificial selection. Therefore, the role of changes in coding sequence under positive selection in the divergence of muscle phenotype among pig breeds was found to be minor. We propose that the divergence of coexpression modules among breeds under positive selection eventually regulated the muscle phenotype divergence during domestication. Previous studies have usually focused on the effect of selection pressure on gene function. In this study, we have shifted the emphasis to the role of selection in the divergence of coexpression networks between breeds during the domestication process.

## Conclusions

Here, we have carried out the first comprehensive analysis of gene coexpression relationships in muscle development in two pig breeds from embryo to adult. We identified significant differences in coexpression networks modules between the Lde and LT breeds, which may be responsible for divergence of the muscle phenotypes. A greater number of coexpression network modules related to myogenesis, postnatal muscle growth, and fast fiber differentiation were found in Lde compared with in LT. However, although fewer modules of myogenesis and muscle growth were identified in LT, more modules related to slow muscle fiber and negative regulation of muscle development were found. In particular, we identified five modules related to intramuscular fat deposition and meat quality in LT. We showed that positive selection played a key role in the divergence of the breed-specific modules, while changes in the gene coding sequence among breeds played only a minor role. Our results demonstrate that the molecular mechanism underlying phenotype divergence between breeds cannot be robustly explained by differential gene expression alone, but can be explained by coexpression network modules. The elucidation of gene coexpression network divergence in the developmental processes of different breeds provides a new foundation for understanding the functional organization of transcriptomes in phenotype variation.

## Methods

### Ethics statement

All animal procedures were performed according to the guidelines developed by the China Council on Animal Care and the protocols were approved by the Animal Care and Use Committee of Guangdong Province, China. The approval ID or permit numbers are SCXK (Guangdong) 2004–0011 and SYXK (Guangdong) 2007–0081.

### Selection of genes for network analysis

The transcriptome sequence data from 20 pig (*Sus scrofa*) muscle samples were downloaded from the National Center for Biotechnology Information (NCBI) Gene Expression Omnibus (http://www.ncbi.nlm.nih.gov/geo/query/acc.cgi?acc=GSE25406). These 20 datasets contain the sequenced transcriptomes of LT and Lde at prenatal days 35, 49, 63, 77, 91 and postnatal days 2, 28, 90, 120, 180. All possible CATG + 17-nt tag sequences were created from the *Sus scrofa* genome sequence (Sscrofa9.2) and UniGene (NCBI36.1, 20090827) databases and used as reference sequences to align and identify the sequencing tags. (The “CATC site” is a digestion site of the NlaIII restriction enzyme. The *NlaIII* digestion site was selected to produce the Solexa sequencing tags which were 21 bp long (i.e., CATG + 17 tags) because most the mRNA sequences (99%) have *NlaIII* digestion sites). All clean tags were aligned to the reference database, and unambiguous tags were annotated. Each alignment was allowed one mismatch to allow for polymorphisms across samples. Mismatches can be caused by sequencing errors, but the frequency of such errors is generally very low (1 or 2 per million).To compare the differential expression of genes across samples, the number of raw clean tags in each sample was normalized to tags per million (TPM) to obtain normalized gene expression levels. Differential expression of genes or tags across samples was detected according to methods described previously [93]. The DEGs with a log2 ratio > 0.5 (P < 0.009, false discovery rate (FDR) < 0.02) between libraries were identified. To construct the coexpression network modules, 7057 DEGs genes in Lde pigs and 7056 DEGs genes in LT pigs were used.

### Methodology used to construct the gene coexpression networks

WGCNA [14,16-20] was carried out using the R software (http://www.r-project.org). Breed and time were analyzed separately. The absolute values of the Pearson correlation coefficients were calculated for all pairwise comparisons of gene-expression values across the LT and Lde samples. The correlation matrix for each breed was then transformed into a matrix of connection strengths (i.e., an “adjacency” matrix) using a power function (connection strength = |correlation|b), which resulted in a “weighted” network. To make meaningful comparisons across data sets, a power of b = 10 was chosen for all analyses. The function TOMdist1 in R was used to compute dissimilarity based on the topological overlap matrix. To group nodes with high topological overlap into modules (clusters), we typically used the average linkage hierarchical clustering coupled with the TOM distance measure. We choose a height cutoff with a threshold of 0.995 to create the clusters. Modules that had at least 30 genes that corresponded to the branches of the dendrogram were selected for analysis. The modules were visualized by classical multidimensional scaling in three dimensions. Then, the module eigengene was compared with the indicator variable using a Kruskal-Wallis test.

### Detection and characterization of modules

The gene expression profile of each module were decomposed via singular value decomposition and the value of the module eigengene, V1 (i.e., the first principal component), was plotted for each sample. We then compared the module eigengene to the indicator variable using a Kruskal-Wallis test.

### Detection of positive selection

Whole-genome alignments of 37 individual pigs and 11 wild boars were downloaded from the NCBI Sequence Read Archive, (ftp://ftp.sra.ebi.ac.uk/vol1/ERA164/ERA164657/bam/, Accession Number. ERP001813). SAMtools/BCFtools [94] was used to call SNPs for each individual animal. The results were merged, and SNPs with low frequency within all samples (<5%) where filtered out. These remaining SNPs were used to generate the consensus sequence for the module genes. PAML [95] was used to perform the ka/ks analysis.

### Availability of supporting data

All the supporting data are included as additional files.

## Additional file

Additional file 1: Table S1. Summary of all the gene coexpression network modules and their parameters. **Table S2.** Attributes of genes in all the gene coexpression network modules. **Table S3.** Calculation of the topological overlap for each possible pair of modules. Module eigengenes and their evolutionary rates (Ka/Ks) in the common module between LT and Lde. **Table S5.** GO annotation analysis of the module eigengene in the common module between LT and Lde. **Table S6.** Module eigengene and their evolutionary rates (Ka/Ks) in six prenatal highly Lde-specific modules. **Table S7.** Module eigengene and their evolutionary rates (Ka/Ks) in prenatal five highly LT-specific modules. **Table S8.** GO annotation analysis of the module eigengene in six prenatal highly Lde-specific modules. **Table S9.** GO annotation analysis of the module eigengene in five prenatal highly LT-specific modules. **Table S10.** Module eigengene and their evolutionary rates (Ka/Ks) in 15 postnatal highly Lde-specific modules. **Table S11.** Module eigengene and their evolutionary rates (Ka/Ks) in 13 postnatal highly LT-specific modules. **Table S12.** GO annotation analysis of the module eigengenes in 15 postnatal highly Lde-specific modules. **Table S13.** GO annotation analysis of the module eigengenes in 13 postnatal highly LT-specific modules.

## Abbreviations

Lde: Landrace
LT: Lantang
WGCNA: Weighted gene coexpression network analysis
DEG: Differentially expressed gene

## References

[1] Chen K, Baxter T, Muir WM, Groenen MA, Schook LB (2007). Genetic resources, genome mapping and evolutionary genomics of the pig (Sus scrofa). Int J Biol Sci.

[2] Groenen MA, Archibald AL, Uenishi H, Tuggle CK, Takeuchi Y, Rothschild MF (2012). Analyses of pig genomes provide insight into porcine demography and evolution. Nature.

[3] Larson G, Dobney K, Albarella U, Fang M, Matisoo-Smith E, Robins J (2005). Worldwide phylogeography of wild boar reveals multiple centers of pig domestication. Science.

[4] Giuffra E, Kijas JM, Amarger V, Carlborg O, Jeon JT, Andersson L (2000). The origin of the domestic pig: independent domestication and subsequent introgression. Genetics.

[5] Kijas JM, Andersson L (2001). A phylogenetic study of the origin of the domestic pig estimated from the near-complete mtDNA genome. J Mol Evol.

[6] Wu GS, Yao YG, Qu KX, Ding ZL, Li H, Palanichamy MG (2007). Population phylogenomic analysis of mitochondrial DNA in wild boars and domestic pigs revealed multiple domestication events in East Asia. Genome Biol.

[7] Rubin CJ, Megens HJ, Martinez Barrio A, Maqbool K, Sayyab S, Schwochow D (2012). Strong signatures of selection in the domestic pig genome. Proc Natl Acad Sci U S A.

[8] Megens HJ, Crooijmans RP, San Cristobal M, Hui X, Li N, Groenen MA (2008). Biodiversity of pig breeds from China and Europe estimated from pooled DNA samples: differences in microsatellite variation between two areas of domestication. Genet Sel Evol.

[9] Okumura N, Kurosawa Y, Kobayashi E, Watanobe T, Ishiguro N, Yasue H (2001). Genetic relationship amongst the major non-coding regions of mitochondrial DNAs in wild boars and several breeds of domesticated pigs. Anim Genet.

[10] Li X, Yang S, Tang Z, Li K, Rothschild MF, Liu B (2014). Genome-wide scans to detect positive selection in Large White and Tongcheng pigs. Anim Genet.

[11] Zhao X, Mo D, Li A, Gong W, Xiao S, Zhang Y (2011). Comparative analyses by sequencing of transcriptomes during skeletal muscle development between pig breeds differing in muscle growth rate and fatness. PLoS One.

[12] Newcom DW, Stalder KJ, Baas TJ, Goodwin RN, Parrish FC, Wiegand BR (2004). Breed differences and genetic parameters of myoglobin concentration in porcine longissimus muscle. J Anim Sci.

[13] Tang Z, Li Y, Wan P, Li X, Zhao S, Liu B (2007). LongSAGE analysis of skeletal muscle at three prenatal stages in Tongcheng and Landrace pigs. Genome Biol.

[14] Konopka G, Friedrich T, Davis-Turak J, Winden K, Oldham MC, Gao F (2012). Human-specific transcriptional networks in the brain. Neuron.

[15] Mason IL (1984). Evolution of domesticated animals.

[16] Oldham MC, Konopka G, Iwamoto K, Langfelder P, Kato T, Horvath S (2008). Functional organization of the transcriptome in human brain. Nat Neurosci.

[17] Johnson MB, Kawasawa YI, Mason CE, Krsnik Z, Coppola G, Bogdanovic D (2009). Functional and evolutionary insights into human brain development through global transcriptome analysis. Neuron.

[18] Miller JA, Horvath S, Geschwind DH (2010). Divergence of human and mouse brain transcriptome highlights Alzheimer disease pathways. Proc Natl Acad Sci U S A.

[19] Brawand D, Soumillon M, Necsulea A, Julien P, Csardi G, Harrigan P (2011). The evolution of gene expression levels in mammalian organs. Nature.

[20] Voineagu I, Wang X, Johnston P, Lowe JK, Tian Y, Horvath S (2011). Transcriptomic analysis of autistic brain reveals convergent molecular pathology. Nature.

[21] Oldham MC, Horvath S, Geschwind DH (2006). Conservation and evolution of gene coexpression networks in human and chimpanzee brains. Proc Natl Acad Sci U S A.

[22] Zhang B, Horvath S (2005). A general framework for weighted gene co-expression network analysis. Stat Appl Genet Mol Biol.

[23] Ravasz E, Somera AL, Mongru DA, Oltvai ZN, Barabasi AL (2002). Hierarchical organization of modularity in metabolic networks. Science.

[24] Fan L, Simard LR (2002). Survival motor neuron (SMN) protein: role in neurite outgrowth and neuromuscular maturation during neuronal differentiation and development. Hum Mol Genet.

[25] Kiely PA, Sant A, O’Connor R (2002). RACK1 is an insulin-like growth factor 1 (IGF-1) receptor-interacting protein that can regulate IGF-1-mediated Akt activation and protection from cell death. J Biol Chem.

[26] Ambekar C, Das B, Yeger H, Dror Y (2010). SBDS-deficiency results in deregulation of reactive oxygen species leading to increased cell death and decreased cell growth. Pediatr Blood Cancer.

[27] Prather D, Krogan NJ, Emili A, Greenblatt JF, Winston F (2005). Identification and characterization of Elf1, a conserved transcription elongation factor in Saccharomyces cerevisiae. Mol Cell Biol.

[28] Bostrom K, Tintut Y, Kao SC, Stanford WP, Demer LL (2000). HOXB7 overexpression promotes differentiation of C3H10T1/2 cells to smooth muscle cells. J Cell Biochem.

[29] Shirvani SM, Mookanamparambil L, Ramoni MF, Chin MT (2007). Transcription factor CHF1/Hey2 regulates the global transcriptional response to platelet-derived growth factor in vascular smooth muscle cells. Physiol Genomics.

[30] Maves L, Waskiewicz AJ, Paul B, Cao Y, Tyler A, Moens CB (2007). Pbx homeodomain proteins direct Myod activity to promote fast-muscle differentiation. Development.

[31] Liguori L, Andolfo I, de Antonellis P, Aglio V, di Dato V, Marino N (2012). The metallophosphodiesterase Mpped2 impairs tumorigenesis in neuroblastoma. Cell Cycle.

[32] Perrot R, Eyer J (2009). Neuronal intermediate filaments and neurodegenerative disorders. Brain Res Bull.

[33] Staus DP, Blaker AL, Medlin MD, Taylor JM, Mack CP (2011). Formin homology domain-containing protein 1 regulates smooth muscle cell phenotype. Arterioscler Thromb Vasc Biol.

[34] Sandmann T, Jensen LJ, Jakobsen JS, Karzynski MM, Eichenlaub MP, Bork P (2006). A temporal map of transcription factor activity: mef2 directly regulates target genes at all stages of muscle development. Dev Cell.

[35] Rathbone CR, Booth FW, Lees SJ (2009). Sirt1 increases skeletal muscle precursor cell proliferation. Eur J Cell Biol.

[36] Yu Y, Qi L, Wu J, Wang Y, Fang W, Zhang H (2013). Kindlin 2 regulates myogenic related factor myogenin via a canonical Wnt signaling in myogenic differentiation. PLoS One.

[37] Capetanaki Y, Milner DJ, Weitzer G (1997). Desmin in muscle formation and maintenance: knockouts and consequences. Cell Struct Funct.

[38] Cheng G, Merriam AP, Gong B, Leahy P, Khanna S, Porter JD (2004). Conserved and muscle-group-specific gene expression patterns shape postnatal development of the novel extraocular muscle phenotype. Physiol Genomics.

[39] Waddell JN, Zhang P, Wen Y, Gupta SK, Yevtodiyenko A, Schmidt JV (2010). Dlk1 is necessary for proper skeletal muscle development and regeneration. PLoS One.

[40] Mangan ME, Olmsted JB (1996). A muscle-specific variant of microtubule-associated protein 4 (MAP4) is required in myogenesis. Development.

[41] Kawai S, Abiko Y, Amano A (2010). Odd-skipped related 2 regulates genes related to proliferation and development. Biochem Biophys Res Commun.

[42] Cha Y, Heo SH, Ahn HJ, Yang SK, Song JH, Suh W (2013). Tcea3 regulates the vascular differentiation potential of mouse embryonic stem cells. Gene Expr.

[43] Tee JM, Peppelenbosch MP (2010). Anchoring skeletal muscle development and disease: the role of ankyrin repeat domain containing proteins in muscle physiology. Crit Rev Biochem Mol Biol.

[44] Dubinska-Magiera M, Jablonska J, Saczko J, Kulbacka J, Jagla T, Daczewska M (2014). Contribution of small heat shock proteins to muscle development and function. FEBS Lett.

[45] Watts R, Johnsen VL, Shearer J, Hittel DS (2013). Myostatin-induced inhibition of the long noncoding RNA Malat1 is associated with decreased myogenesis. Am J Physiol Cell Physiol.

[46] Cohn RD, Henry MD, Michele DE, Barresi R, Saito F, Moore SA (2002). Disruption of DAG1 in differentiated skeletal muscle reveals a role for dystroglycan in muscle regeneration. Cell.

[47] Feo S, Antona V, Barbieri G, Passantino R, Cali L, Giallongo A (1995). Transcription of the human beta enolase gene (ENO-3) is regulated by an intronic muscle-specific enhancer that binds myocyte-specific enhancer factor 2 proteins and ubiquitous G-rich-box binding factors. Mol Cell Biol.

[48] Burguiere AC, Nord H, von Hofsten J (2011). Alkali-like myosin light chain-1 (myl1) is an early marker for differentiating fast muscle cells in zebrafish. Dev Dyn.

[49] Hsiao CD, Tsai WY, Horng LS, Tsai HJ (2003). Molecular structure and developmental expression of three muscle-type troponin T genes in zebrafish. Dev Dyn.

[50] Quiat D, Voelker KA, Pei J, Grishin NV, Grange RW, Bassel-Duby R (2011). Concerted regulation of myofiber-specific gene expression and muscle performance by the transcriptional repressor Sox6. Proc Natl Acad Sci U S A.

[51] Bottinelli R, Reggiani C (2000). Human skeletal muscle fibres: molecular and functional diversity. Prog Biophys Mol Biol.

[52] Wang Y, Szczesna-Cordary D, Craig R, Diaz-Perez Z, Guzman G, Miller T (2007). Fast skeletal muscle regulatory light chain is required for fast and slow skeletal muscle development. FASEB J.

[53] Wang M, Yu H, Kim YS, Bidwell CA, Kuang S (2012). Myostatin facilitates slow and inhibits fast myosin heavy chain expression during myogenic differentiation. Biochem Biophys Res Commun.

[54] North KN, Yang N, Wattanasirichaigoon D, Mills M, Easteal S, Beggs AH (1999). A common nonsense mutation results in alpha-actinin-3 deficiency in the general population. Nat Genet.

[55] Wan L, Ma J, Wang N, Wang D, Xu G (2013). Molecular Cloning and Characterization of Different Expression of MYOZ2 and MYOZ3 in Tianfu Goat. PLoS One.

[56] Xu X, Qiu H, Du ZQ, Fan B, Rothschild MF, Yuan F (2010). Porcine CSRP3: polymorphism and association analyses with meat quality traits and comparative analyses with CSRP1 and CSRP2. Mol Biol Rep.

[57] Klover P, Chen W, Zhu BM, Hennighausen L (2009). Skeletal muscle growth and fiber composition in mice are regulated through the transcription factors STAT5a/b: linking growth hormone to the androgen receptor. FASEB J.

[58] Rosen GD, Sanes JR, LaChance R, Cunningham JM, Roman J, Dean DC (1992). Roles for the integrin VLA-4 and its counter receptor VCAM-1 in myogenesis. Cell.

[59] Odemis V, Boosmann K, Dieterlen MT, Engele J (2007). The chemokine SDF1 controls multiple steps of myogenesis through atypical PKCzeta. J Cell Sci.

[60] Eom GH, Kim KB, Kim JH, Kim JY, Kim JR, Kee HJ (2011). Histone methyltransferase SETD3 regulates muscle differentiation. J Biol Chem.

[61] Ochi H, Westerfield M (2007). Signaling networks that regulate muscle development: lessons from zebrafish. Dev Growth Differ.

[62] Frock RL, Kudlow BA, Evans AM, Jameson SA, Hauschka SD, Kennedy BK (2006). Lamin A/C and emerin are critical for skeletal muscle satellite cell differentiation. Genes Dev.

[63] Dacwag CS, Ohkawa Y, Pal S, Sif S, Imbalzano AN (2007). The protein arginine methyltransferase Prmt5 is required for myogenesis because it facilitates ATP-dependent chromatin remodeling. Mol Cell Biol.

[64] Olson EN, Klein WH (1994). bHLH factors in muscle development: dead lines and commitments, what to leave in and what to leave out. Genes Dev.

[65] Wang F, Wang H, Wu H, Qiu H, Zeng C, Sun L (2013). TEAD1 controls C2C12 cell proliferation and differentiation and regulates three novel target genes. Cell Signal.

[66] Ahn HJ, Cha Y, Moon SH, Jung JE, Park KS (2012). Ell3 enhances differentiation of mouse embryonic stem cells by regulating epithelial-mesenchymal transition and apoptosis. PLoS One.

[67] Petersson SJ, Jorgensen LH, Andersen DC, Norgaard RC, Jensen CH, Schroder HD (2013). SPARC is up-regulated during skeletal muscle regeneration and inhibits myoblast differentiation. Histol Histopathol.

[68] Xiao Q, Wang G, Yin X, Luo Z, Margariti A, Zeng L (2011). Chromobox protein homolog 3 is essential for stem cell differentiation to smooth muscles in vitro and in embryonic arteriogenesis. Arterioscler Thromb Vasc Biol.

[69] Jones FS, Meech R, Edelman DB, Oakey RJ, Jones PL (2001). Prx1 controls vascular smooth muscle cell proliferation and tenascin-C expression and is upregulated with Prx2 in pulmonary vascular disease. Circ Res.

[70] Camarata T, Krcmery J, Snyder D, Park S, Topczewski J, Simon HG (2010). Pdlim7 (LMP4) regulation of Tbx5 specifies zebrafish heart atrio-ventricular boundary and valve formation. Dev Biol.

[71] Zhu C, Hu DL, Liu YQ, Zhang QJ, Chen FK, Kong XQ (2011). Fabp3 inhibits proliferation and promotes apoptosis of embryonic myocardial cells. Cell Biochem Biophys.

[72] Hu G, Wang X, Saunders DN, Henderson M, Russell AJ, Herring BP (2010). Modulation of myocardin function by the ubiquitin E3 ligase UBR5. J Biol Chem.

[73] Chen Z, Zhao TJ, Li J, Gao YS, Meng FG, Yan YB (2011). Slow skeletal muscle myosin-binding protein-C (MyBPC1) mediates recruitment of muscle-type creatine kinase (CK) to myosin. Biochem J.

[74] Wohlgemuth SL, Crawford BD, Pilgrim DB (2007). The myosin co-chaperone UNC-45 is required for skeletal and cardiac muscle function in zebrafish. Dev Biol.

[75] Takada F, Vander Woude DL, Tong HQ, Thompson TG, Watkins SC, Kunkel LM (2001). Myozenin: an alpha-actinin- and gamma-filamin-binding protein of skeletal muscle Z lines. Proc Natl Acad Sci U S A.

[76] Ge Y, Yoon MS, Chen J (2011). Raptor and Rheb negatively regulate skeletal myogenesis through suppression of insulin receptor substrate 1 (IRS1). J Biol Chem.

[77] Parakati R, DiMario JX (2013). Repression of myoblast proliferation and fibroblast growth factor receptor 1 promoter activity by KLF10 protein. J Biol Chem.

[78] Sun L, Ma K, Wang H, Xiao F, Gao Y, Zhang W (2007). JAK1-STAT1-STAT3, a key pathway promoting proliferation and preventing premature differentiation of myoblasts. J Cell Biol.

[79] Liu C, Gersch RP, Hawke TJ, Hadjiargyrou M (2010). Silencing of Mustn1 inhibits myogenic fusion and differentiation. Am J Physiol Cell Physiol.

[80] Tsika RW, Schramm C, Simmer G, Fitzsimons DP, Moss RL, Ji J (2008). Overexpression of TEAD-1 in transgenic mouse striated muscles produces a slower skeletal muscle contractile phenotype. J Biol Chem.

[81] Heanue TA, Reshef R, Davis RJ, Mardon G, Oliver G, Tomarev S (1999). Synergistic regulation of vertebrate muscle development by Dach2, Eya2, and Six1, homologs of genes required for Drosophila eye formation. Genes Dev.

[82] Sambasivan R, Comai G, Le Roux I, Gomes D, Konge J, Dumas G (2013). Embryonic founders of adult muscle stem cells are primed by the determination gene Mrf4. Dev Biol.

[83] Simone C, Stiegler P, Bagella L, Pucci B, Bellan C, De Falco G (2002). Activation of MyoD-dependent transcription by cdk9/cyclin T2. Oncogene.

[84] Benhaddou A, Keime C, Ye T, Morlon A, Michel I, Jost B (2012). Transcription factor TEAD4 regulates expression of myogenin and the unfolded protein response genes during C2C12 cell differentiation. Cell Death Differ.

[85] Fan C, Fu Z, Su Q, Angelini DJ, Van Eyk J, Johns RA (2011). S100A11 mediates hypoxia-induced mitogenic factor (HIMF)-induced smooth muscle cell migration, vesicular exocytosis, and nuclear activation. Mol Cell Proteomics.

[86] Kemp TJ, Sadusky TJ, Simon M, Brown R, Eastwood M, Sassoon DA (2001). Identification of a novel stretch-responsive skeletal muscle gene (Smpx). Genomics.

[87] Ebert SM, Dyle MC, Kunkel SD, Bullard SA, Bongers KS, Fox DK (2012). Stress-induced skeletal muscle Gadd45a expression reprograms myonuclei and causes muscle atrophy. J Biol Chem.

[88] Cánovas E, Quintanilla R, Badaoui B, Porredón C, Gallardo D, Pena RN (2009). Pig HDL-binding protein (HDLBP) genotype is associated with intramuscular fat percentage. Livestock Science.

[89] Wang X, Xue C, Liu H, Xu Y, Zhao R, Jiang Z (2009). Differential display of expressed genes reveals a novel function of SFRS18 in regulation of intramuscular fat deposition. Int J Biol Sci.

[90] Tillander V, Arvidsson Nordstrom E, Reilly J, Strozyk M, Van Veldhoven PP, Hunt MC (2014). Acyl-CoA thioesterase 9 (ACOT9) in mouse may provide a novel link between fatty acid and amino acid metabolism in mitochondria. Cell Mol Life Sci.

[91] Wood JD, Enser M, Fisher AV, Nute GR, Sheard PR, Richardson RI (2008). Fat deposition, fatty acid composition and meat quality: A review. Meat Sci.

[92] Xu H, Xu Y, Liang X, Wang Y, Jin F, Liu D (2013). Porcine skeletal muscle differentially expressed gene ATP5B: molecular characterization, expression patterns, and association analysis with meat quality traits. Mamm Genome.

[93] Audic S, Claverie JM (1997). The significance of digital gene expression profiles. Genome Res.

[94] Li H, Handsaker B, Wysoker A, Fennell T, Ruan J, Homer N (2009). The Sequence Alignment/Map format and SAMtools. Bioinformatics.

[95] Yang Z (2007). PAML 4: phylogenetic analysis by maximum likelihood. Mol Biol Evol.

