# Supplementary material for: Gene coexpression networks reveal key drivers of phenotypic divergence in porcine muscle
Source: BMC Genomics. 2015 Feb 5;16(1):50. doi: 10.1186/s12864-015-1238-5 (PMC4328970; doi:10.1186/s12864-015-1238-5)
Supplement: Additional file 1: Table S1. — Summary of all the gene coexpression network modules and their parameters. Table S2. Attributes of genes in all the gene coexpression network modules. Table S3. Calculation of the topological overlap for each possible pair of modules. Module eigengenes and their evolutionary rates (Ka/Ks) in the common module between LT and Lde. Table S5. GO annotation analysis of the module eigengene in the common module between LT and Lde. Table S6. Module eigengene and their evolutionary rates (Ka/Ks) in six prenatal highly Lde-specific modules. Table S7. Module eigengene and their evolutionary rates (Ka/Ks) in prenatal five highly LT-specific modules. Table S8. GO annotation analysis of the module eigengene in six prenatal highly Lde-specific modules. Table S9. GO annotation analysis of the module eigengene in five prenatal highly LT-specific modules. Table S10. Module eigengene and their evolutionary rates (Ka/Ks) in 15 postnatal highly Lde-specific modules. Table S11. Module eigengene and their evolutionary rates (Ka/Ks) in 13 postnatal highly LT-specific modules. Table S12. GO annotation analysis of the module eigengenes in 15 postnatal highly Lde-specific modules. Table S13. GO annotation analysis of the module eigengenes in 13 postnatal highly LT-specific modules. [file 12864_2015_1238_MOESM1_ESM.pdf]

Table S1-1 Summary of all modules and their parameters of LDE prenatal.

Module	Freq	PC1_Var	Min_TO	Max_COR	Density	Tree_order
black	61	0.847755	0.4562256	0.999712	0.4198118	1
grey60	39	0.9503686	0.4740164	0.9998951	0.4774982	2
grey	49	0.5132573	0.4850699	0.9992925	0.1852281	3
salmon	45	0.8717378	0.4578818	0.9991979	0.4295504	4
pink	59	0.9258893	0.4082066	0.9997321	0.5184597	5
green	89	0.7934244	0.3785996	0.9998728	0.4183458	6
midnightblu	42	0.9615473	0.3779887	0.999761	0.5844929	7
red	64	0.9314472	0.3892089	0.9998089	0.5415761	8
navy	45	0.9029569	0.4503008	1	0.472216	9
greenyellow	50	0.5715346	0.4827451	0.9992722	0.1869076	10
royalblue	33	0.9292506	0.4880899	0.9991953	0.4565705	11
brown	177	0.8722138	0.4151683	0.9999713	0.4617138	12
lightcyan	42	0.7473861	0.5235713	0.9997069	0.3053057	13
lightyellow	36	0.8827629	0.4994704	0.9997471	0.4078755	14
magenta	54	0.9414638	0.4482136	0.9998062	0.4666865	15
yellow	138	0.809274	0.4862307	0.9996832	0.3540754	16
purple	54	0.8252491	0.4752626	0.9992828	0.3442903	17
darkturquoi	27	0.8538651	0.4893295	0.9973522	0.3531297	18
tan	50	0.930918	0.414812	0.9997358	0.5097721	19
darkgreen	31	0.8995778	0.4121452	0.999148	0.4749301	20
blue	194	0.9204767	0.3747012	0.9999377	0.5422207	21
turquoise	2183	0.8227848	0.1341299	1	0.6868792	22
lightgreen	37	0.6499104	0.5893286	0.9999727	0.1876737	23
darkred	32	0.6991279	0.6498156	0.9987959	0.16837	24

Table S1-2 Summary of all modules and their parameters of LDE postnatal.

Module	Freq	PC1_Var	Min_TO	Max_COR	Density	Tree_order
pink	158	0.9810339	0.2316582	1	0.7362309	14
red	137	0.9753795	0.2429791	1	0.7317501	15
darkgrey	62	0.9566825	0.2578196	0.9998767	0.704993	13
grey60	78	0.9582232	0.332907	0.9999389	0.6208285	26
midnightblu	87	0.9663096	0.3371629	0.9999419	0.6087564	19
salmon	90	0.9460452	0.3658081	1	0.5783936	29
yellow	172	0.9018443	0.3270557	0.9999424	0.572269	25
black	120	0.965186	0.3841767	0.9999919	0.5696409	21
green	139	0.9312421	0.357461	0.9999836	0.5609609	18
royalblue	73	0.9560256	0.4041474	0.9999691	0.5536606	5
skyblue	51	0.9542876	0.4225319	0.999996	0.5455539	11
blue	189	0.9138188	0.3478074	0.9999928	0.5451768	20
lightyellow	76	0.9398845	0.4121325	0.9998747	0.5283844	4
purple	101	0.9075914	0.3608833	0.9998024	0.5250488	32
magenta	110	0.9056494	0.4167575	0.9998518	0.510229	6
darkturquoi	64	0.8789288	0.3990456	1	0.4796635	24
grey	20	0.9072864	0.4431668	0.9999707	0.4744381	9
brown	186	0.8383873	0.3947422	0.9999837	0.4707019	3
lightcyan	85	0.8667667	0.4339632	0.999867	0.4582715	8
white	72	0.7914479	0.2313004	0.9999962	0.4173313	17
darkred	73	0.8043369	0.3919687	0.9997368	0.4050453	28
paleturquois	39	0.8872046	0.4958577	0.9993934	0.3977021	30
darkgreen	73	0.7774061	0.3917048	0.9999712	0.3911474	31
orange	60	0.803238	0.4295227	1	0.3863773	7
lightgreen	77	0.7606187	0.4373364	0.9999982	0.3671703	10
turquoise	541	0.6089392	0.2715343	0.9999945	0.3502898	12
violet	83	0.7796454	0.2402957	1	0.2949356	16
darkorange	59	0.7936951	0.5104428	0.9992878	0.2746057	27
tan	90	0.7559772	0.5507977	0.9993051	0.244862	1
greenyellow	92	0.5952734	0.4638785	0.9996641	0.2443304	23
navy	110	0.5965289	0.2532651	1	0.1928733	2
darkmagent	22	0.7512989	0.6638631	0.9997978	0.1369532	22

Table S1-3 Summary of all modules and their parameters of LT prenatal.

Module	Freq	PC1_Var	Min_TO	Max_COR	Density	Tree_order
blue	261	0.9005179	0.3483671	1	0.541041	8
turquoise	1417	0.8398105	0.174814	1	0.63974	35
darkturquoi	39	0.9202871	0.4052916	0.999923	0.4808496	4
purple	69	0.9584632	0.3922852	0.9999067	0.5654861	19
green	115	0.9254245	0.4436122	0.9998986	0.460445	34
brown	142	0.8929812	0.4296307	0.9998734	0.4364474	21
black	77	0.8786501	0.4725467	0.9998534	0.4027008	33
darkred	41	0.9607883	0.4024963	0.9998248	0.5378638	5
grey	34	0.5209626	0.5904118	0.9998011	0.1625959	12
skyblue	33	0.9182154	0.496891	0.9997632	0.4235548	26
sienna3	23	0.9536893	0.4899955	0.9997439	0.4535686	23
pink	105	0.5002351	0.5101938	0.9997377	0.107591	10
greenyellow	66	0.8517739	0.4055984	0.9997362	0.458934	17
yellowgreer	22	0.9375686	0.4224674	0.9997324	0.5275564	18
darkgreen	62	0.5144787	0.4624105	0.9997311	0.2033513	15
lightyellow	44	0.913123	0.4518834	0.9997225	0.4427861	6
darkmagent	24	0.8618092	0.6058346	0.9996784	0.2896591	29
navy	79	0.674292	0.458213	0.9996643	0.2363878	3
yellow	132	0.7415002	0.5299141	0.9996272	0.2744862	11
magenta	93	0.5185746	0.4725088	0.9996217	0.1962893	2
lightcyan	51	0.9168062	0.4796412	0.9996135	0.4432369	25
tan	60	0.908904	0.5060209	0.9996077	0.392378	24
royalblue	43	0.6668555	0.581284	0.9995997	0.185259	28
white	56	0.5683779	0.4038855	0.9995816	0.2769348	7
salmon	60	0.9100359	0.4975263	0.9994959	0.427801	32
skyblue3	22	0.9347778	0.5388459	0.9994379	0.3773428	27
midnightblu	51	0.8966745	0.5060103	0.9993965	0.3786119	9
darkorange	35	0.9618457	0.4362885	0.9993595	0.4995416	16
violet	57	0.6718741	0.47711	0.9991757	0.1429144	20
orange	37	0.5273219	0.5358947	0.9990643	0.1741324	22
darkgrey	39	0.5712759	0.5742534	0.9989653	0.170726	30
red	86	0.633063	0.5825643	0.9987991	0.2134371	14
grey60	50	0.5386871	0.5681801	0.9983891	0.1766869	31
lightgreen	47	0.816093	0.6128843	0.9980298	0.2505689	1
paleturquois	26	0.8459702	0.5883147	0.9970693	0.2782481	13

Table S1-4 Summary of all modules and their parameters of LT postnatal.

Module	Freq	PC1_Var	Min_TO	Max_COR	Density	Density_ index	Tree_order
lightyellow	66	0.9910363	0.2386506	0.9999958	0.7479792	5.4225585	33
sienna3	28	0.9871024	0.2421022	0.9999002	0.7428563	5.3854192	34
violet	79	0.987148	0.2414932	1	0.7402272	5.3663595	32
grey60	68	0.9809624	0.2414317	0.9999492	0.7316343	5.3040637	31
darkmagent	31	0.9704914	0.2534457	0.9999493	0.7211378	5.2279685	30
black	108	0.9704392	0.2492331	0.9999919	0.7191221	5.2133553	29
tan	78	0.9570796	0.2615006	0.999899	0.6972834	5.0550335	28
lightgreen	66	0.9780811	0.3545265	1	0.6121065	4.4375341	4
blue	348	0.886449	0.2697177	0.9999639	0.6007785	4.3554107	27
magenta	96	0.9424085	0.3602905	0.9999828	0.5775385	4.1869297	18
yellow	164	0.8685782	0.3559183	1	0.5080697	3.6833077	3
red	131	0.9359852	0.419599	0.9998798	0.4933262	3.5764227	24
darkred	60	0.8890597	0.4161513	0.9992738	0.4496018	3.2594384	6
darkgreen	57	0.8414891	0.4082556	0.9998435	0.4308618	3.1235807	5
orange	44	0.9263049	0.4757554	0.9985796	0.4244731	3.0772651	25
turquoise	543	0.6998872	0.2756069	0.999938	0.4224378	3.0625094	26
yellowgreer	24	0.9234479	0.5355965	0.998141	0.40552	2.9398624	12
greenyellow	78	0.8575255	0.5027646	0.9996288	0.3711036	2.6903565	11
skyblue	42	0.9104009	0.5331329	0.9999157	0.3692172	2.6766808	20
lightcyan	73	0.751225	0.4325327	0.9996656	0.3621938	2.6257642	17
darkgrey	50	0.8417537	0.5083142	0.9999587	0.3451191	2.5019789	7
darkturquoi	50	0.8608001	0.5036625	0.9988274	0.3442425	2.4956239	2
midnightblu	73	0.7920277	0.5057456	0.9993159	0.3075535	2.2296429	10
salmon	76	0.8620401	0.5691738	0.9993406	0.307082	2.2262249	21
paleturquois	37	0.8199537	0.525986	0.9979774	0.3044187	2.2069171	14
pink	138	0.6885435	0.3700147	1	0.299072	2.1681555	9
white	73	0.558945	0.2543592	0.9999786	0.2559738	1.8557102	8
royalblue	65	0.674809	0.4833357	0.9996327	0.2497974	1.8109342	23
darkorange	42	0.6908029	0.5465729	0.9992081	0.2412209	1.7487579	1
green	141	0.6341449	0.5452966	0.9995913	0.2164624	1.5692685	15
brown	176	0.5474794	0.4056553	0.99978	0.2120038	1.5369448	16
navy	109	0.6099034	0.5326865	1	0.1737145	1.2593622	19
grey	55	0.5792981	0.5236668	0.9980123	0.1379384	1	13
purple	80	0.6083508	0.628406	0.9994714	0.1148295	0.8324689	22

Table S2-1 Attributes of genes in all modules of LDE prenatal.

GeneSymbol	Module	kTotal	kWithin	eigencorr	eigenpval	meanExpr
gi|10304379|gb|AF288822.1|AF288822	turquoise	335.35	323.703	0.644397	0.240513	41.59
gi|106073315|gb|DQ508264.1|	green	127.07	18.0861	0.730541	0.160949	1.25
gi|108796051|ref|NM_213912.2|	darkturquoise	235.38	17.2808	0.982419	0.002791	274.918
gi|108796069|ref|NM_001042375.1|	green	151.98	39.6258	0.934073	0.020118	395.418
gi|109639160|ref|NM_001025222.2|	green	235.37	52.931	0.978592	0.003748	211.948
gi|109639161|ref|NM_001006593.2|	brown	244.98	118.202	0.975424	0.004608	30.324
gi|112181313|ref|NM_214438.2|	navy	242.34	31.7399	0.982611	0.002745	450.918
gi|113205497|ref|NM_001044527.1|	green	241.36	53.7763	0.989651	0.001262	428.998
gi|113205555|ref|NM_001044525.1|	turquoise	1274.5	1273.28	0.953966	0.011774	22.26
gi|113205565|ref|NM_001044529.1|	blue	280.5	135.467	0.964125	0.008113	20.234
gi|113205585|ref|NM_001044535.1|	darkturquoise	137.28	7.1262	0.749571	0.144655	37.066
gi|113205597|ref|NM_001044537.1|	yellow	108.18	42.0523	0.81642	0.091778	18.194
gi|113205623|ref|NM_001044545.1|	grey60	235.12	31.1664	0.98408	0.002406	236.636
gi|113205635|ref|NM_001044548.1|	blue	297.08	140.656	0.972952	0.005318	17.346
gi|113205649|ref|NM_001044550.1|	green	179.47	48.2775	0.980732	0.003201	41.604
gi|113205651|ref|NM_001044553.1|	turquoise	662.23	659.552	0.806528	0.099137	24.964
gi|113205653|ref|NM_001044551.1|	royalblue	267.11	19.3794	0.923517	0.025098	21.472
gi|113205675|ref|NM_001044558.1|	turquoise	1081.5	1080.46	0.909053	0.032472	18.144
gi|113205761|ref|NM_001044578.1|	turquoise	1149.8	1147.98	0.929864	0.022061	60.598
gi|113205811|ref|NM_001044593.1|	yellow	145.01	53.9093	0.866982	0.057061	320.802
gi|113205825|ref|NM_001044594.1|	green	221.3	47.2423	0.940534	0.017251	21.02
gi|113205849|ref|NM_001044600.1|	turquoise	1233.6	1232.35	0.947337	0.014392	11.814
gi|113205859|ref|NM_001044605.1|	pink	222.67	45.6059	0.988435	0.00149	41.894
gi|113205873|ref|NM_001044606.1|	magenta	333.13	43.7934	0.991024	0.001019	144.146
gi|113205905|ref|NM_001044614.1|	turquoise	793.04	790.676	0.832365	0.080288	47.386
gi|113205907|ref|NM_001044616.1|	pink	194.86	44.9686	0.985195	0.002158	21.004
gi|113205929|ref|NM_001044620.1|	brown	249.04	124.819	0.988938	0.001394	179.71
gi|113931658|ref|NM_001044598.2|	turquoise	1553.6	1553.18	0.996528	0.000245	2.692
gi|114326182|ref|NM_001048072.1|	turquoise	1568.8	1568.42	0.999113	3.17E-05	191.378
gi|114326213|ref|NM_001048069.1|	darkturquoise	166.69	17.7127	0.991355	0.000964	78.646
gi|114703735|ref|NM_001048187.1|	midnightblue	244.76	36.201	0.994421	0.0005	6.374
gi|115312275|ref|NM_001048232.1|	turquoise	1451.6	1451.14	0.979289	0.003567	2.016
gi|115545523|dbj|AK230638.1|	magenta	337.17	43.5469	0.989168	0.001351	23.196
gi|115545530|dbj|AK230645.1|	turquoise	1236.5	1235.71	0.938947	0.017942	11.15
gi|115545542|dbj|AK230657.1|	lightyellow	192.01	15.9105	0.851769	0.066964	168.954
gi|115545566|dbj|AK230681.1|	magenta	332.28	44.9916	0.996192	0.000282	161.62
gi|115545596|dbj|AK230711.1|	royalblue	139.59	19.4675	0.927376	0.023236	2.812
gi|115545597|dbj|AK230712.1|	turquoise	1463.8	1463.18	0.983906	0.002445	42.424
gi|115545598|dbj|AK230713.1|	turquoise	1548.3	1547.95	0.995305	0.000386	12.808
gi|115545600|dbj|AK230715.1|	red	265.89	45.0549	0.95967	0.009663	77.63
gi|115545604|dbj|AK230719.1|	turquoise	1439.8	1439.19	0.979871	0.003418	2.628
gi|115545622|dbj|AK230737.1|	turquoise	809.13	807.577	0.847385	0.069908	32.576
gi|115545643|dbj|AK230758.1|	turquoise	1489.1	1488.41	0.989022	0.001378	56.918
gi|115545669|dbj|AK230784.1|	brown	145.89	77.0327	0.861295	0.060705	69.388
gi|115545681|dbj|AK230796.1|	turquoise	137.83	58.5181	0.208284	0.736735	32.984
gi|115545734|dbj|AK234167.1|	turquoise	1050.5	1049.46	0.901285	0.036675	1.09

gi|115545741|dbj|AK234174.1|	turquoise	816.27	815.513	0.858606	0.062452	31.632
gi|115545742|dbj|AK234175.1|	greenyellow	201.7	22.9102	0.986741	0.001829	8.424
gi|115545749|dbj|AK234182.1|	black	281.21	42.8926	0.998702	5.61E-05	36.86
gi|115545756|dbj|AK234189.1|	turquoise	1504.4	1503.92	0.988132	0.001549	4.612
gi|115545757|dbj|AK234190.1|	turquoise	391.91	386.049	0.671127	0.214881	22.106
gi|115545765|dbj|AK234198.1|	yellow	303.25	62.457	0.892123	0.041838	84.382
gi|115545780|dbj|AK234213.1|	turquoise	1500.8	1500.26	0.990298	0.001146	7.072
gi|115545783|dbj|AK234216.1|	turquoise	1340.1	1339.44	0.958092	0.010233	3.852
gi|115545785|dbj|AK234218.1|	turquoise	1391.3	1390.76	0.972035	0.00559	17.362
gi|115545812|dbj|AK234245.1|	blue	267.94	129.718	0.953736	0.011862	48
gi|115545821|dbj|AK234254.1|	blue	308.67	151.192	0.989463	0.001296	54.512
gi|115545827|dbj|AK234260.1|	turquoise	1439.6	1438.82	0.981099	0.003111	32.232
gi|115545844|dbj|AK234277.1|	grey60	246.09	32.6347	0.994248	0.000523	205.5
gi|115545853|dbj|AK234286.1|	turquoise	505.92	501.468	0.749889	0.144387	24.61
gi|115545859|dbj|AK234292.1|	turquoise	552.02	543.257	0.749311	0.144874	80.23
gi|115545865|dbj|AK234298.1|	turquoise	1460.2	1459.49	0.982619	0.002744	3.392
gi|115545868|dbj|AK234301.1|	turquoise	1430.2	1429.4	0.979799	0.003436	44.586
gi|115545894|dbj|AK234327.1|	turquoise	814.68	811.682	0.848239	0.069332	38.908
gi|115545900|dbj|AK234333.1|	turquoise	1523.6	1523.04	0.993519	0.000626	61.454
gi|115545908|dbj|AK234341.1|	turquoise	326.87	306.329	0.600303	0.284449	110.692
gi|115545914|dbj|AK234347.1|	turquoise	975.91	973.158	0.890631	0.042699	12.63
gi|115545916|dbj|AK234349.1|	lightgreen	42.479	11.905	0.816704	0.091569	13.662
gi|115545921|dbj|AK237362.1|	turquoise	1214.9	1213.15	0.940477	0.017276	74.556
gi|115545930|dbj|AK237371.1|	turquoise	1279.9	1278.52	0.952478	0.012347	39.69
gi|115545949|dbj|AK237390.1|	turquoise	830.3	826.916	0.855621	0.06441	44.838
gi|115545973|dbj|AK237414.1|	midnightblue	229.84	36.5081	0.996251	0.000275	101.384
gi|115545984|dbj|AK237425.1|	red	248.18	50.9308	0.989623	0.001267	32.564
gi|115546047|dbj|AK237489.1|	royalblue	223.68	24.079	0.976332	0.004356	26.984
gi|115546071|dbj|AK237513.1|	turquoise	174.67	81.9318	0.224002	0.717195	55.918
gi|115546075|dbj|AK237517.1|	purple	201.11	31.5106	0.962323	0.008729	40.392
gi|115546083|dbj|AK237525.1|	turquoise	1368.6	1367.73	0.969607	0.006331	34.53
gi|115546137|dbj|AK240375.1|	turquoise	261.25	222.177	0.511441	0.378436	7.742
gi|115546138|dbj|AK240376.1|	turquoise	120.33	92.1125	0.333295	0.583629	220.41
gi|115546153|dbj|AK240391.1|	greenyellow	77.704	6.38123	-0.57003	0.315701	15.196
gi|115546179|dbj|AK240417.1|	brown	313.56	102.822	0.928412	0.022744	17.832
gi|115546190|dbj|AK240428.1|	turquoise	1359.1	1358.35	0.962583	0.008639	5.748
gi|115546201|dbj|AK240439.1|	yellow	163.5	50.0297	0.844995	0.07153	63.814
gi|115546222|dbj|AK240460.1|	grey	76.416	8.32889	-0.0098	0.987526	34.832
gi|115546238|dbj|AK240476.1|	turquoise	1473.2	1472.75	0.985808	0.002025	5.86
gi|115546241|dbj|AK240479.1|	turquoise	199.15	186.146	0.509009	0.381099	90.79
gi|115546260|dbj|AK240498.1|	turquoise	1121.1	1119.93	0.916154	0.028775	28.842
gi|115546268|dbj|AK240506.1|	turquoise	1505.4	1504.82	0.990893	0.001042	4.316
gi|115546292|dbj|AK240530.1|	turquoise	193.64	176.922	0.496044	0.39537	16.106
gi|115546313|dbj|AK240551.1|	turquoise	179.44	115.323	0.335119	0.581441	50.758
gi|115546315|dbj|AK240553.1|	turquoise	1319.7	1318.93	0.954827	0.011447	25.808
gi|115546324|dbj|AK230843.1|	turquoise	344.69	321.358	0.615661	0.268923	68.718
gi|115546327|dbj|AK230846.1|	turquoise	1497.1	1496.64	0.986084	0.001966	3.65
gi|115546331|dbj|AK230850.1|	salmon	236.82	26.3498	0.950147	0.013261	85.292

gi|115546371|dbj|AK230890.1|	blue	330.28	138.118	0.969883	0.006246	99.04
gi|115546413|dbj|AK230932.1|	turquoise	1557.8	1557.4	0.997983	0.000109	4.284
gi|115546432|dbj|AK230951.1|	turquoise	1553.6	1553.18	0.996528	0.000245	1.654
gi|115546438|dbj|AK230957.1|	turquoise	1539.4	1538.91	0.994368	0.000507	80.016
gi|115546439|dbj|AK230958.1|	turquoise	99.433	87.6827	0.373508	0.535736	7.58
gi|115546451|dbj|AK230970.1|	turquoise	1427.6	1427.12	0.974877	0.004762	22.304
gi|115546452|dbj|AK230971.1|	turquoise	676.84	672.773	0.805173	0.100159	23.234
gi|115546454|dbj|AK230973.1|	turquoise	1460.4	1459.69	0.983802	0.002469	67.054
gi|115546460|dbj|AK230979.1|	turquoise	1413.5	1412.97	0.976715	0.00425	16.668
gi|115546462|dbj|AK230981.1|	blue	308.08	145.717	0.980901	0.003159	21.856
gi|115546465|dbj|AK230984.1|	navy	227.21	23.8321	0.92011	0.026779	177.574
gi|115546507|dbj|AK231026.1|	brown	213.82	107.375	0.952091	0.012497	6.882
gi|115546529|dbj|AK234367.1|	turquoise	272.89	234.783	0.51532	0.374197	92.414
gi|115546548|dbj|AK234386.1|	brown	212.62	120.255	0.98139	0.003039	19.588
gi|115546576|dbj|AK234414.1|	grey	220.02	10.2254	0.786739	0.114366	107.17
gi|115546586|dbj|AK234424.1|	midnightblue	219.05	36.5754	0.996456	0.000253	63.068
gi|115546623|dbj|AK234461.1|	greenyellow	208.59	21.6044	0.966518	0.007318	319.186
gi|115546656|dbj|AK234494.1|	brown	109.47	46.722	0.731224	0.160356	18.586
gi|115546695|dbj|AK234533.1|	lightcyan	296.14	26.3889	0.992669	0.000753	124.574
gi|115546696|dbj|AK234534.1|	turquoise	1542.7	1542.27	0.995131	0.000408	54.66
gi|115546712|dbj|AK237558.1|	turquoise	842.04	840.086	0.848439	0.069197	107.328
gi|115546736|dbj|AK237582.1|	turquoise	1499.4	1498.83	0.989631	0.001265	12.326
gi|115546753|dbj|AK237599.1|	turquoise	353.04	333.894	0.62135	0.263232	32.424
gi|115546833|dbj|AK237679.1|	grey	201.9	11.2602	0.877889	0.050275	31.604
gi|115546838|dbj|AK237684.1|	red	197.19	50.5139	0.989689	0.001255	61.634
gi|115546849|dbj|AK237695.1|	tan	256.7	34.7129	0.960641	0.009318	37.326
gi|115546889|dbj|AK237735.1|	turquoise	1531.4	1530.89	0.994757	0.000455	4.652
gi|115546917|dbj|AK240563.1|	grey	278.95	16.1809	0.94193	0.016651	72.038
gi|115546929|dbj|AK240575.1|	blue	320.72	147.609	0.983826	0.002463	58.494
gi|115546934|dbj|AK240580.1|	blue	343.36	145.064	0.979382	0.003543	13.96
gi|115546953|dbj|AK240599.1|	turquoise	428.54	421.029	0.698866	0.189152	36.678
gi|115546964|dbj|AK240610.1|	turquoise	1427.1	1426.26	0.978814	0.00369	21.368
gi|115546978|dbj|AK231033.1|	blue	326.34	113.468	0.923579	0.025067	22.608
gi|115547025|dbj|AK231080.1|	turquoise	1564.7	1564.32	0.998487	7.06E-05	5.716
gi|115547032|dbj|AK231087.1|	turquoise	1430.1	1429.61	0.97773	0.003976	3.168
gi|115547045|dbj|AK231100.1|	turquoise	1571.2	1570.81	0.999644	8.06E-06	2.112
gi|115547065|dbj|AK231120.1|	navy	286.53	30.7539	0.968731	0.006606	19.978
gi|115547083|dbj|AK231138.1|	lightgreen	75.286	16.0447	0.950377	0.01317	11.748
gi|115547094|dbj|AK231149.1|	turquoise	1456.4	1455.68	0.982179	0.002848	26.552
gi|115547099|dbj|AK231154.1|	turquoise	129.71	102.629	0.36966	0.540285	25.346
gi|115547117|dbj|AK231172.1|	grey	285.2	16.3459	0.954057	0.011739	11.844
gi|115547122|dbj|AK231177.1|	turquoise	1417.8	1417.33	0.973129	0.005266	10.27
gi|115547124|dbj|AK231179.1|	lightgreen	105.49	11.7393	0.927234	0.023304	51.124
gi|115547142|dbj|AK231197.1|	grey	289.13	14.087	0.863074	0.059557	619.35
gi|115547162|dbj|AK231217.1|	turquoise	405.92	393.58	0.6726	0.213492	15.708
gi|115547167|dbj|AK231222.1|	lightyellow	193.44	15.8962	0.851889	0.066884	3.356
gi|115547172|dbj|AK231227.1|	turquoise	1557.6	1557.26	0.997654	0.000136	7.99
gi|115547186|dbj|AK234559.1|	turquoise	956.74	953.231	0.882774	0.047324	64.824

gi|115547198|dbj|AK234571.1|	lightcyan	257.01	24.7174	0.985468	0.002098	30.044
gi|115547214|dbj|AK234587.1|	brown	288.17	113.275	0.957794	0.010342	2.758
gi|115547217|dbj|AK234590.1|	turquoise	1548.2	1547.79	0.996807	0.000216	1.214
gi|115547230|dbj|AK234603.1|	darkred	40.398	8.07496	0.77574	0.123107	167.894
gi|115547231|dbj|AK234604.1|	black	244.87	37.8172	0.969036	0.00651	64.232
gi|115547260|dbj|AK234633.1|	lightyellow	290.17	24.19	0.981083	0.003114	52.786
gi|115547290|dbj|AK234663.1|	turquoise	1470.6	1470.08	0.981758	0.00295	3.88
gi|115547309|dbj|AK234682.1|	turquoise	1548.5	1548.03	0.996371	0.000262	9.126
gi|115547340|dbj|AK234713.1|	turquoise	1545.8	1545.34	0.995982	0.000306	93.028
gi|115547352|dbj|AK234725.1|	brown	213.46	94.7723	0.921653	0.026013	58.926
gi|115547361|dbj|AK234734.1|	turquoise	1555.9	1555.45	0.997205	0.000177	99.81
gi|115547364|dbj|AK234737.1|	greenyellow	53.587	1.15866	0.379649	0.528493	16.19
gi|115547369|dbj|AK234742.1|	magenta	291.11	39.395	0.968235	0.006764	26.83
gi|115547381|dbj|AK237762.1|	turquoise	975.06	973.107	0.894159	0.040672	55.238
gi|115547390|dbj|AK237771.1|	purple	209.13	36.348	0.999018	3.70E-05	16.592
gi|115547397|dbj|AK237778.1|	pink	172.51	41.7681	0.967926	0.006862	9.164
gi|115547414|dbj|AK237795.1|	turquoise	528.35	520.987	0.749326	0.144862	81.458
gi|115547436|dbj|AK237818.1|	turquoise	1343	1341.91	0.965917	0.007514	25.238
gi|115547445|dbj|AK237827.1|	turquoise	985.25	984.295	0.89881	0.038048	1002.29
gi|115547488|dbj|AK237870.1|	turquoise	1546.3	1545.83	0.997151	0.000182	81.3
gi|115547522|dbj|AK237904.1|	turquoise	1513.7	1513.13	0.992263	0.000816	68.794
gi|115547541|dbj|AK237923.1|	salmon	236.16	23.5956	0.909323	0.032328	389.436
gi|115547599|dbj|AK231257.1|	turquoise	1553.6	1553.18	0.996528	0.000245	1.748
gi|115547606|dbj|AK231264.1|	turquoise	1510.9	1510.45	0.989883	0.00122	4.126
gi|115547607|dbj|AK231265.1|	turquoise	131.73	104.754	0.378832	0.529455	16.278
gi|115547647|dbj|AK231305.1|	turquoise	1558	1557.62	0.997128	0.000185	7.094
gi|115547679|dbj|AK231337.1|	grey60	237.56	30.5592	0.980612	0.003231	42.232
gi|115547758|dbj|AK231416.1|	turquoise	1128.4	1127.67	0.924397	0.024669	18.944
gi|115547780|dbj|AK234756.1|	brown	253.82	129.058	0.996421	0.000257	9.152
gi|115547803|dbj|AK234779.1|	turquoise	1258.8	1257.47	0.950404	0.01316	108.706
gi|115547849|dbj|AK234825.1|	blue	343.68	152.123	0.990806	0.001057	38.476
gi|115547856|dbj|AK234832.1|	turquoise	1509	1508.44	0.99167	0.000911	77.15
gi|115547886|dbj|AK234862.1|	turquoise	486.26	480.621	0.721951	0.168468	16.41
gi|115547932|dbj|AK234909.1|	navy	208.01	29.8224	0.966997	0.007161	70.86
gi|115547946|dbj|AK234923.1|	turquoise	1181.4	1179.92	0.930461	0.021782	18.578
gi|115547950|dbj|AK234927.1|	turquoise	1513.8	1513.38	0.989032	0.001377	58.586
gi|115547955|dbj|AK234932.1|	lightyellow	207.38	16.6614	0.875721	0.051602	33.722
gi|115547984|dbj|AK237968.1|	turquoise	1433.6	1433.22	0.977908	0.003929	3.642
gi|115547985|dbj|AK237969.1|	darkgreen	257.93	22.8352	0.983882	0.002451	10.936
gi|115548000|dbj|AK237984.1|	yellow	301.99	70.0446	0.934897	0.019745	29.556
gi|115548002|dbj|AK237986.1|	black	255.59	35.6223	0.961695	0.008948	275.056
gi|115548023|dbj|AK238007.1|	turquoise	1434.5	1434.13	0.976646	0.004269	6.08
gi|115548072|dbj|AK238056.1|	midnightblue	240.16	37.1165	0.999506	1.32E-05	19.294
gi|115548093|dbj|AK238077.1|	turquoise	749.25	744.247	0.826	0.084818	61.466
gi|115548136|dbj|AK238120.1|	royalblue	164.25	23.9797	0.974254	0.00494	3.05
gi|115548140|dbj|AK238124.1|	turquoise	1501	1500.35	0.989918	0.001213	4.794
gi|115548164|dbj|AK238148.1|	purple	60.158	2.4644	0.28382	0.643541	0.864
gi|115548176|dbj|AK234948.1|	turquoise	374.89	369.663	0.684342	0.202508	249.268

gi|115548217|dbj|AK231527.1|	turquoise	1551.2	1550.75	0.99725	0.000173	4.996
gi|115548219|dbj|AK231529.1|	turquoise	879.1	876.893	0.856225	0.064013	48.836
gi|115548230|dbj|AK231444.1|	turquoise	1530.5	1530.15	0.993112	0.000685	12.142
gi|115548316|dbj|AK234973.1|	turquoise	1421.4	1420.74	0.979466	0.003521	28.906
gi|115548319|dbj|AK231543.1|	darkred	93.571	13.3749	0.973057	0.005287	63.934
gi|115548328|dbj|AK231701.1|	turquoise	156.49	150.73	0.517481	0.37184	57.002
gi|115548360|dbj|AK231733.1|	yellow	298.05	60.8998	0.889147	0.043561	53.734
gi|115548383|dbj|AK235000.1|	turquoise	1321.8	1320.95	0.963045	0.008481	39.186
gi|115548388|dbj|AK235005.1|	brown	102.57	40.9317	0.695004	0.192679	99.57
gi|115548389|dbj|AK235006.1|	turquoise	1351.8	1350.79	0.965632	0.007609	25.418
gi|115548394|dbj|AK235011.1|	turquoise	390.86	383.076	0.661312	0.2242	80.85
gi|115548409|dbj|AK235026.1|	turquoise	1391.5	1390.93	0.971322	0.005805	42.016
gi|115548424|dbj|AK235041.1|	turquoise	590.25	585.258	0.76651	0.130589	36.29
gi|115548435|dbj|AK235052.1|	turquoise	1559.8	1559.37	0.998313	8.32E-05	1.884
gi|115548441|dbj|AK235058.1|	black	279.13	41.4806	0.977615	0.004007	39.832
gi|115548447|dbj|AK235064.1|	green	126.87	36.323	0.888017	0.044221	5.914
gi|115548454|dbj|AK235071.1|	yellow	232.05	68.5335	0.938284	0.018233	22.858
gi|115548467|dbj|AK235084.1|	turquoise	1091.5	1089.29	0.916459	0.02862	218.056
gi|115548500|dbj|AK235117.1|	turquoise	1077.8	1075.9	0.911849	0.030999	86.268
gi|115548514|dbj|AK235131.1|	turquoise	787.73	783.811	0.842126	0.073491	73.72
gi|115548519|dbj|AK235136.1|	tan	301.23	37.2295	0.976173	0.004399	16.876
gi|115548520|dbj|AK235137.1|	lightgreen	105.57	6.20908	0.201321	0.745412	231.096
gi|115548526|dbj|AK235143.1|	turquoise	1336.4	1335.3	0.962114	0.008802	70.79
gi|115548554|dbj|AK235171.1|	blue	221.8	100.956	0.897877	0.03857	43.53
gi|115548564|dbj|AK231548.1|	turquoise	1257.6	1256.16	0.949101	0.013679	64.736
gi|115548572|dbj|AK231556.1|	brown	286.62	112.08	0.95414	0.011708	67.906
gi|115548574|dbj|AK231558.1|	green	111.64	24.5887	0.79905	0.104815	201.602
gi|115548575|dbj|AK231559.1|	turquoise	1516.1	1515.72	0.990619	0.001089	32.482
gi|115548591|dbj|AK231575.1|	turquoise	975.75	973.036	0.892933	0.041373	23.474
gi|115548658|dbj|AK231642.1|	turquoise	1285.2	1283.87	0.955089	0.011348	70.956
gi|115548691|dbj|AK231676.1|	turquoise	1095.3	1093.53	0.918465	0.027604	4.392
gi|115548698|dbj|AK231683.1|	lightyellow	208.53	17.0779	0.88193	0.04783	64.764
gi|115548717|dbj|AK231783.1|	midnightblue	209.63	32.2486	0.970883	0.005938	68.094
gi|115548727|dbj|AK231793.1|	turquoise	404.55	399.194	0.687262	0.199803	21.148
gi|115548797|dbj|AK231755.1|	yellow	128.23	59.5422	0.901097	0.036779	96.14
gi|115548801|dbj|AK231759.1|	darkred	77.295	11.776	0.905348	0.034456	70.752
gi|115548831|dbj|AK231946.1|	turquoise	1216.9	1215.38	0.943348	0.016049	148.494
gi|115548849|dbj|AK235252.1|	brown	313.54	93.8273	0.906068	0.034068	14.722
gi|115548869|dbj|AK235272.1|	brown	164.45	89.4717	0.902854	0.035813	8.468
gi|115548882|dbj|AK235285.1|	turquoise	205.42	194.628	0.545857	0.341242	175.238
gi|115548919|dbj|AK235322.1|	turquoise	1214.1	1212.79	0.936753	0.018912	54.846
gi|115548922|dbj|AK235325.1|	turquoise	1233.2	1231.94	0.944959	0.015373	502.7
gi|115548932|dbj|AK235335.1|	turquoise	770.35	767.935	0.831051	0.081216	24.508
gi|115548933|dbj|AK235336.1|	turquoise	1366.4	1365.55	0.967426	0.007023	22.282
gi|115548942|dbj|AK235345.1|	yellow	180.45	65.4399	0.924105	0.024811	51.586
gi|115548955|dbj|AK235358.1|	turquoise	1511.5	1511.12	0.989902	0.001216	87.488
gi|115548956|dbj|AK235359.1|	turquoise	1447.4	1446.7	0.981867	0.002923	101.738
gi|115548969|dbj|AK235372.1|	turquoise	1312.5	1311.67	0.961628	0.008971	9.836

gi|115548982|dbj|AK238171.1|	turquoise	1506.4	1505.86	0.989971	0.001204	21.126
gi|115549078|dbj|AK238267.1|	turquoise	975.15	971.952	0.888238	0.044092	25.586
gi|115549177|dbj|AK231853.1|	pink	124.83	30.5504	0.894972	0.04021	32.994
gi|115549179|dbj|AK231855.1|	turquoise	1279	1277.65	0.952565	0.012313	48.664
gi|115549210|dbj|AK231886.1|	grey	62.247	9.18708	0.14602	0.814745	12.056
gi|115549219|dbj|AK231895.1|	turquoise	235.26	211.673	0.519946	0.369157	30.526
gi|115549239|dbj|AK231915.1|	turquoise	1133.3	1131.79	0.919247	0.027211	60.006
gi|115549258|dbj|AK235178.1|	turquoise	1451.1	1450.44	0.98034	0.003299	65.436
gi|115549275|dbj|AK235196.1|	turquoise	1546.2	1545.68	0.997191	0.000179	64.394
gi|115549299|dbj|AK235220.1|	purple	191.6	34.9445	0.987906	0.001594	64.916
gi|115549306|dbj|AK235227.1|	turquoise	1159.4	1157.91	0.928739	0.02259	5.128
gi|115549313|dbj|AK235234.1|	green	136.33	35.457	0.9002	0.037275	92.668
gi|115549321|dbj|AK232099.1|	turquoise	1565.1	1564.75	0.998624	6.12E-05	25.3
gi|115549331|dbj|AK232109.1|	grey	293.13	15.0593	0.926737	0.023541	177.638
gi|115549338|dbj|AK232116.1|	green	88.192	22.2939	0.788688	0.112837	49.424
gi|115549349|dbj|AK232127.1|	midnightblue	223.54	32.3946	0.971468	0.005761	24.136
gi|115549359|dbj|AK235381.1|	turquoise	1430.7	1430.24	0.976231	0.004383	10.81
gi|115549368|dbj|AK235390.1|	grey60	288.77	24.6279	0.932347	0.020908	17.108
gi|115549388|dbj|AK235410.1|	turquoise	566.42	560.915	0.766138	0.130894	12.432
gi|115549396|dbj|AK235418.1|	turquoise	1246.4	1245.02	0.944938	0.015381	17.678
gi|115549402|dbj|AK235424.1|	turquoise	1512.8	1512.3	0.990924	0.001037	1.082
gi|115549412|dbj|AK235434.1|	turquoise	338.27	319.988	0.631961	0.252703	58.656
gi|115549416|dbj|AK235438.1|	turquoise	1564.8	1564.4	0.998907	4.34E-05	8.216
gi|115549428|dbj|AK235450.1|	turquoise	1435.3	1434.68	0.980227	0.003328	247.09
gi|115549440|dbj|AK235462.1|	brown	342.85	79.2676	0.857262	0.063332	167.73
gi|115549454|dbj|AK235476.1|	turquoise	125.67	99.9939	0.39005	0.51627	19.072
gi|115549455|dbj|AK235477.1|	turquoise	1268.2	1267.69	0.951723	0.012641	106.994
gi|115549486|dbj|AK235508.1|	turquoise	1381	1380.04	0.970957	0.005916	76.292
gi|115549499|dbj|AK235521.1|	turquoise	966.08	963.639	0.888539	0.043916	16.928
gi|115549511|dbj|AK235533.1|	grey	91.043	6.35105	0.135694	0.82776	41.04
gi|115549536|dbj|AK235558.1|	turquoise	1373.5	1372.67	0.968466	0.00669	8.904
gi|115549537|dbj|AK235559.1|	turquoise	1535.8	1535.33	0.99543	0.000371	56.876
gi|115549562|dbj|AK238370.1|	turquoise	1222.7	1221.66	0.935882	0.019301	6.464
gi|115549571|dbj|AK238379.1|	greenyellow	65.499	6.84371	-0.48379	0.408975	16.148
gi|115549608|dbj|AK238416.1|	lightgreen	50.741	15.5516	0.947305	0.014405	59.674
gi|115549613|dbj|AK238421.1|	darkturquoise	149.04	10.378	0.866698	0.057241	50.148
gi|115549624|dbj|AK238432.1|	turquoise	1556.2	1555.79	0.996503	0.000248	3.48
gi|115549680|dbj|AK231959.1|	yellow	169.13	71.7982	0.956349	0.010876	216.416
gi|115549689|dbj|AK231968.1|	pink	246.21	46.6176	0.991692	0.000908	44.57
gi|115549693|dbj|AK231972.1|	turquoise	332.57	323.583	0.623937	0.260654	39.092
gi|115549744|dbj|AK232023.1|	pink	264.14	41.8561	0.963144	0.008447	89.204
gi|115549787|dbj|AK232067.1|	red	244.37	51.5768	0.992656	0.000755	66.752
gi|115549792|dbj|AK232072.1|	turquoise	669.29	666.358	0.794279	0.108486	2.356
gi|115549831|dbj|AK238491.1|	blue	243.55	117.387	0.931952	0.02109	5.426
gi|115549856|dbj|AK238516.1|	lightcyan	269.8	22.7881	0.941017	0.017043	53.16
gi|115549900|dbj|AK232134.1|	yellow	224.74	83.7276	0.994005	0.000557	56.362
gi|115549902|dbj|AK232136.1|	grey	281.05	16.8655	0.964302	0.008053	19.806
gi|115549949|dbj|AK232184.1|	lightcyan	288.89	27.3479	0.994937	0.000432	129.124

gi|115549970|dbj|AK232205.1|	navy	209.54	27.8455	0.947334	0.014393	16.65
gi|115549976|dbj|AK232211.1|	turquoise	1504.4	1503.99	0.98794	0.001587	10.23
gi|115550002|dbj|AK232237.1|	grey	30.781	1.74702	0.225579	0.715239	65.304
gi|115550007|dbj|AK232242.1|	brown	186.39	99.4268	0.931274	0.021404	99.774
gi|115550039|dbj|AK232274.1|	turquoise	1018	1016.62	0.893483	0.041058	42.668
gi|115550042|dbj|AK232277.1|	yellow	255.02	74.6595	0.959014	0.009899	173.994
gi|115550054|dbj|AK232289.1|	greenyellow	218.83	21.4019	0.983842	0.00246	12.222
gi|115550060|dbj|AK232295.1|	brown	216	117.826	0.981231	0.003078	58.822
gi|115550075|dbj|AK232310.1|	turquoise	100.27	52.2389	0.199689	0.747449	0.284
gi|115550091|dbj|AK232326.1|	turquoise	1555.8	1555.43	0.99685	0.000212	42.678
gi|115550094|dbj|AK232329.1|	turquoise	1458.4	1457.83	0.98377	0.002476	30.666
gi|115550108|dbj|AK235587.1|	lightgreen	56.501	16.7457	0.988942	0.001394	1.326
gi|115550124|dbj|AK235603.1|	grey60	274.19	31.7816	0.988608	0.001457	158.174
gi|115550125|dbj|AK235604.1|	lightcyan	202.69	6.4366	0.261776	0.670543	33.82
gi|115550130|dbj|AK235609.1|	grey	254.09	14.2704	0.910822	0.031537	15.538
gi|115550133|dbj|AK235612.1|	turquoise	645.35	641.889	0.801933	0.102615	561.656
gi|115550154|dbj|AK235633.1|	turquoise	788.98	785.428	0.844114	0.072131	7.53
gi|115550155|dbj|AK235634.1|	turquoise	1322.5	1321.4	0.958241	0.010179	54.46
gi|115550159|dbj|AK235638.1|	green	187.89	35.443	0.85963	0.061785	7.542
gi|115550165|dbj|AK235644.1|	turquoise	309.08	297.282	0.628022	0.256598	99.152
gi|115550169|dbj|AK235648.1|	turquoise	1549.3	1548.88	0.995746	0.000333	35.246
gi|115550178|dbj|AK235657.1|	turquoise	1371.4	1370.8	0.964518	0.00798	25.382
gi|115550183|dbj|AK235662.1|	blue	319.81	146.685	0.981949	0.002903	18.624
gi|115550185|dbj|AK235664.1|	salmon	68.602	11.9245	0.773955	0.124544	22.234
gi|115550191|dbj|AK235670.1|	turquoise	1149.8	1148.65	0.923725	0.024996	28.038
gi|115550230|dbj|AK235709.1|	turquoise	1335.6	1334.63	0.962339	0.008724	35.576
gi|115550272|dbj|AK235751.1|	magenta	342.46	43.5327	0.988003	0.001575	67.452
gi|115550292|dbj|AK235771.1|	yellow	299.24	69.3137	0.933544	0.020359	101.19
gi|115550330|dbj|AK238595.1|	green	167.73	34.5702	0.893264	0.041183	11.114
gi|115550332|dbj|AK238597.1|	turquoise	378.19	367.248	0.667227	0.21857	155.276
gi|115550345|dbj|AK238610.1|	turquoise	1550.1	1549.64	0.997245	0.000174	5.454
gi|115550346|dbj|AK238611.1|	turquoise	1491	1490.63	0.985281	0.002139	11.066
gi|115550351|dbj|AK238616.1|	turquoise	1469.8	1469.13	0.986119	0.001959	27.102
gi|115550354|dbj|AK238619.1|	black	188.77	27.0131	0.885831	0.045507	101.478
gi|115550357|dbj|AK238622.1|	turquoise	1565.7	1565.23	0.999469	1.47E-05	50.96
gi|115550390|dbj|AK238655.1|	turquoise	453.1	448.412	0.726658	0.164336	34.614
gi|115550392|dbj|AK238657.1|	blue	321.14	151.126	0.989365	0.001314	28.948
gi|115550393|dbj|AK238658.1|	brown	193.57	110.418	0.959515	0.009719	3.688
gi|115550394|dbj|AK238659.1|	darkred	78.36	8.64465	0.823342	0.086732	39.142
gi|115550416|dbj|AK238681.1|	turquoise	527.78	523.659	0.749093	0.145058	42.74
gi|115550429|dbj|AK238694.1|	yellow	299.9	48.2501	0.814473	0.093212	28.96
gi|115550430|dbj|AK238695.1|	turquoise	1502.7	1502.14	0.989274	0.001331	7.046
gi|115550441|dbj|AK238706.1|	turquoise	794.6	792.221	0.834222	0.07898	61.432
gi|115550476|dbj|AK238741.1|	turquoise	1556.6	1556.22	0.996912	0.000206	13.592
gi|115550482|dbj|AK238747.1|	turquoise	1374.3	1373.81	0.964538	0.007974	4.23
gi|115550487|dbj|AK238752.1|	turquoise	1467.7	1467.02	0.986013	0.001981	35.662
gi|115550524|dbj|AK232354.1|	turquoise	1295.9	1294.74	0.954898	0.01142	7.282
gi|115550537|dbj|AK232367.1|	yellow	241.82	67.8817	0.920677	0.026497	27.038

gi|115550567|dbj|AK232397.1|	yellow	109.49	38.5244	0.784915	0.115802	14.39
gi|115550607|dbj|AK232437.1|	greenyellow	95.528	6.98401	-0.41826	0.483419	33.66
gi|115550629|dbj|AK232459.1|	tan	305.38	40.1307	0.993252	0.000665	61.57
gi|115550647|dbj|AK232477.1|	turquoise	1521.2	1520.82	0.991153	0.000998	0.982
gi|115550656|dbj|AK232486.1|	turquoise	173.91	155.521	0.454034	0.442432	16.412
gi|115550671|dbj|AK232501.1|	turquoise	174.97	140.026	0.406753	0.496764	36.928
gi|115550708|dbj|AK235782.1|	blue	349.71	140.85	0.973241	0.005234	85.134
gi|115550714|dbj|AK235788.1|	turquoise	321.44	317.551	0.646491	0.238476	3.758
gi|115550750|dbj|AK235824.1|	brown	257.56	84.7007	0.88855	0.043909	13.006
gi|115550755|dbj|AK235829.1|	turquoise	1228.7	1227.73	0.946843	0.014594	71.226
gi|115550758|dbj|AK235833.1|	turquoise	407.89	400.224	0.691132	0.196232	56.152
gi|115550785|dbj|AK235860.1|	turquoise	329.04	318.348	0.643764	0.241129	63.136
gi|115550795|dbj|AK235870.1|	darkred	86.091	10.3403	0.86365	0.059187	18.114
gi|115550804|dbj|AK235879.1|	turquoise	1457.4	1456.74	0.984591	0.002291	11.168
gi|115550806|dbj|AK235881.1|	turquoise	1115.1	1114.43	0.925059	0.024348	56.246
gi|115550817|dbj|AK235892.1|	turquoise	1107.6	1105.8	0.923259	0.025224	45.186
gi|115550848|dbj|AK235923.1|	midnightblue	244.29	36.8188	0.997754	0.000128	57.084
gi|115550874|dbj|AK235949.1|	turquoise	1359.3	1358.45	0.968024	0.006831	57.056
gi|115550882|dbj|AK235957.1|	turquoise	1443.4	1442.95	0.979262	0.003574	4.542
gi|115550889|dbj|AK235964.1|	turquoise	441.11	433.66	0.70615	0.182552	43.858
gi|115550896|dbj|AK235971.1|	turquoise	1526	1525.5	0.993469	0.000633	90.356
gi|115550899|dbj|AK238760.1|	turquoise	1000.7	997.495	0.892947	0.041365	32.854
gi|115550900|dbj|AK238761.1|	turquoise	1536.3	1535.9	0.99353	0.000624	23.184
gi|115550918|dbj|AK238779.1|	turquoise	261.3	222.642	0.500987	0.389914	60.228
gi|115550923|dbj|AK238784.1|	blue	236.64	103.407	0.903894	0.035245	12.992
gi|115550943|dbj|AK238804.1|	turquoise	1557	1556.56	0.998017	0.000106	39.08
gi|115550946|dbj|AK238807.1|	turquoise	292.95	258.335	0.546593	0.340458	95.998
gi|115550971|dbj|AK238832.1|	turquoise	131.14	108.535	0.394668	0.510861	41.966
gi|115550975|dbj|AK238836.1|	tan	329.71	35.4261	0.963759	0.008237	206.558
gi|115550976|dbj|AK238837.1|	turquoise	1450.6	1450.07	0.982153	0.002854	77.072
gi|115550982|dbj|AK238843.1|	darkturquoise	190.69	7.24497	0.775449	0.123342	59.4
gi|115550999|dbj|AK238860.1|	turquoise	119.37	44.7817	0.087672	0.888515	58.664
gi|115551001|dbj|AK238862.1|	turquoise	1473.4	1472.93	0.983105	0.002629	307.042
gi|115551027|dbj|AK238888.1|	turquoise	163.18	111.935	0.347036	0.567177	20.862
gi|115551063|dbj|AK238924.1|	turquoise	1190.3	1189.43	0.928401	0.022749	13.232
gi|115551074|dbj|AK238935.1|	black	141.49	19.8644	0.816048	0.092051	14.044
gi|115551088|dbj|AK238949.1|	lightgreen	110.38	8.61384	0.778171	0.121158	22.218
gi|115551105|dbj|AK232538.1|	turquoise	407.19	394.903	0.674764	0.211455	21.212
gi|115551122|dbj|AK232555.1|	lightcyan	294.29	25.852	0.993368	0.000648	24.636
gi|115551173|dbj|AK232606.1|	turquoise	1534.5	1534.03	0.99454	0.000484	22.312
gi|115551176|dbj|AK232609.1|	yellow	277.25	65.3768	0.904668	0.034824	55.63
gi|115551177|dbj|AK232610.1|	greenyellow	54.495	6.19997	-0.18713	0.763139	25.252
gi|115551181|dbj|AK232614.1|	yellow	186.97	57.5565	0.901396	0.036614	94.642
gi|115551189|dbj|AK232622.1|	turquoise	779.81	775.459	0.838161	0.07623	18.582
gi|115551213|dbj|AK232646.1|	yellow	116.3	52.3411	0.872425	0.05364	8.634
gi|115551216|dbj|AK232649.1|	turquoise	1105.3	1104.07	0.918982	0.027343	35.31
gi|115551270|dbj|AK232704.1|	grey60	230.8	30.6585	0.980687	0.003213	203.382
gi|115551308|dbj|AK235986.1|	yellow	119.06	47.404	0.846699	0.070373	37.834

gi|115551350|dbj|AK236028.1|	magenta	294.31	39.73	0.96878	0.006591	65.746
gi|115551358|dbj|AK236036.1|	turquoise	144.65	96.4195	0.348655	0.565244	30.32
gi|115551364|dbj|AK236042.1|	turquoise	1519.9	1519.5	0.990114	0.001178	8.302
gi|115551374|dbj|AK236052.1|	turquoise	1250	1249.26	0.950163	0.013255	75.36
gi|115551399|dbj|AK236077.1|	darkturquoise	155.07	17.0193	0.979997	0.003386	23.528
gi|115551408|dbj|AK236086.1|	grey	315.48	17.2484	0.967176	0.007103	79.31
gi|115551411|dbj|AK236089.1|	turquoise	1381.3	1380.65	0.968344	0.006729	3.492
gi|115551432|dbj|AK236110.1|	turquoise	1481.5	1480.88	0.987093	0.001757	40.794
gi|115551465|dbj|AK236143.1|	turquoise	1559.9	1559.49	0.99757	0.000144	13.58
gi|115551480|dbj|AK236158.1|	turquoise	1560.9	1560.41	0.998878	4.51E-05	9.568
gi|115551482|dbj|AK236160.1|	turquoise	1365.4	1364.54	0.969466	0.006375	51.184
gi|115551497|dbj|AK238961.1|	turquoise	1355	1353.91	0.967626	0.006958	198.012
gi|115551503|dbj|AK238967.1|	turquoise	1550.4	1550	0.996239	0.000277	7.508
gi|115551518|dbj|AK238982.1|	magenta	271.27	32.2286	0.924233	0.024749	21.03
gi|115551521|dbj|AK238985.1|	turquoise	968.6	968.043	0.892648	0.041537	56.978
gi|115551534|dbj|AK238998.1|	green	80.002	16.814	0.678328	0.208114	44.472
gi|115551591|dbj|AK239055.1|	turquoise	1470.3	1469.65	0.985299	0.002135	21.432
gi|115551607|dbj|AK239071.1|	turquoise	896.58	892.185	0.865679	0.05789	39.92
gi|115551630|dbj|AK239094.1|	turquoise	1446.4	1445.66	0.981041	0.003125	47.83
gi|115551631|dbj|AK239095.1|	turquoise	239.9	220.293	0.560989	0.325196	13.562
gi|115551642|dbj|AK239106.1|	darkgreen	237.59	22.2685	0.975125	0.004692	80.378
gi|115551644|dbj|AK239108.1|	turquoise	1547.4	1546.95	0.99694	0.000203	4.192
gi|115551673|dbj|AK239137.1|	turquoise	1204.9	1203.49	0.937777	0.018457	15.03
gi|115551776|dbj|AK236182.1|	navy	263.12	34.0915	0.996415	0.000258	98.218
gi|115551787|dbj|AK236193.1|	turquoise	375.35	356.98	0.637615	0.247141	23.482
gi|115551791|dbj|AK236197.1|	turquoise	1405.9	1405.35	0.970674	0.006002	22.576
gi|115551807|dbj|AK236213.1|	turquoise	1537.9	1537.55	0.99442	0.0005	2.924
gi|115551834|dbj|AK236241.1|	turquoise	220.43	172.317	0.447649	0.449689	46.19
gi|115551842|dbj|AK236249.1|	turquoise	1398.6	1397.65	0.974736	0.004802	75.79
gi|115551880|dbj|AK236287.1|	magenta	335.82	43.454	0.98862	0.001455	20.302
gi|115551885|dbj|AK236292.1|	turquoise	1557	1556.57	0.997451	0.000154	5.804
gi|115551894|dbj|AK236301.1|	brown	292.76	118.414	0.970182	0.006153	19.21
gi|115551900|dbj|AK236307.1|	grey	62.515	8.78442	-0.08947	0.886237	0.584
gi|115551903|dbj|AK236310.1|	yellow	129.6	54.4743	0.875796	0.051555	68.594
gi|115551917|dbj|AK236324.1|	royalblue	239.19	24.8476	0.982631	0.002741	74.214
gi|115551923|dbj|AK236330.1|	greenyellow	209.81	22.7331	0.980031	0.003377	5.752
gi|115551932|dbj|AK236339.1|	turquoise	973.85	970.671	0.888022	0.044218	91.254
gi|115551938|dbj|AK236345.1|	turquoise	472.8	466.879	0.706236	0.182474	45.638
gi|115551966|dbj|AK239159.1|	turquoise	1470.7	1470.13	0.985191	0.002159	14.296
gi|115551974|dbj|AK239167.1|	blue	338.09	150.795	0.988368	0.001503	7.646
gi|115551993|dbj|AK239186.1|	turquoise	606.12	595.964	0.767192	0.130032	113.838
gi|115552005|dbj|AK239198.1|	turquoise	1031.8	1028.83	0.900269	0.037237	14.132
gi|115552041|dbj|AK239234.1|	blue	224.77	106.853	0.910933	0.031479	14.562
gi|115552057|dbj|AK239250.1|	turquoise	651.54	645.86	0.799641	0.104362	33.224
gi|115552064|dbj|AK239257.1|	turquoise	1299.8	1299.11	0.950356	0.013179	64.83
gi|115552080|dbj|AK239273.1|	turquoise	1398	1397.51	0.972731	0.005384	37.442
gi|115552084|dbj|AK239277.1|	turquoise	1110.4	1108.77	0.918885	0.027392	9.066
gi|115552089|dbj|AK239282.1|	turquoise	1163.9	1162.92	0.922565	0.025564	49.208

gi|115552097|dbj|AK239290.1|	turquoise	1443.1	1442.32	0.981748	0.002952	126.934
gi|115552101|dbj|AK239294.1|	turquoise	1007.8	1005.53	0.900498	0.03711	52.868
gi|115552127|dbj|AK239320.1|	turquoise	1553.6	1553.18	0.996528	0.000245	2.032
gi|115552144|dbj|AK239337.1|	turquoise	1319.2	1318.16	0.959206	0.00983	10.818
gi|115552159|dbj|AK239352.1|	turquoise	1442.9	1442.21	0.981832	0.002932	24.794
gi|115552176|dbj|AK230485.1|	turquoise	1506.9	1506.34	0.990108	0.001179	5.622
gi|115552177|dbj|AK230486.1|	green	120.1	22.2265	0.756937	0.138487	1496.508
gi|115552187|dbj|AK230496.1|	turquoise	938.65	935.349	0.879464	0.049317	72.87
gi|115552213|dbj|AK230522.1|	turquoise	1527.9	1527.45	0.992624	0.00076	14.82
gi|115552215|dbj|AK230524.1|	turquoise	1267.5	1266.13	0.950689	0.013047	6.93
gi|115552216|dbj|AK230525.1|	turquoise	1561.2	1560.82	0.997815	0.000123	5.488
gi|115552220|dbj|AK230529.1|	turquoise	994.6	993.05	0.890442	0.042809	19.208
gi|115552242|dbj|AK230551.1|	turquoise	1417.8	1417.01	0.978593	0.003748	13.082
gi|115552246|dbj|AK230555.1|	turquoise	1256.6	1255.44	0.944473	0.015575	18.608
gi|115552250|dbj|AK230559.1|	blue	301.81	132.392	0.959404	0.009759	120.304
gi|115552260|dbj|AK230569.1|	purple	212.21	36.2755	0.997889	0.000116	93.67
gi|115552265|dbj|AK230574.1|	purple	208.91	26.2659	0.9191	0.027284	32.124
gi|115552269|dbj|AK230578.1|	salmon	107.57	25.4944	0.942316	0.016486	217.304
gi|115552281|dbj|AK230590.1|	turquoise	1539.3	1538.88	0.993449	0.000636	31.058
gi|115552282|dbj|AK230591.1|	greenyellow	77.515	7.0156	-0.11436	0.854715	170.878
gi|115552285|dbj|AK230594.1|	turquoise	1516.5	1516.06	0.989856	0.001224	3.29
gi|115552309|dbj|AK230619.1|	purple	160.96	27.6448	0.92814	0.022873	211.136
gi|115552311|dbj|AK230621.1|	turquoise	1405.5	1404.94	0.976987	0.004176	44.86
gi|115552314|dbj|AK230624.1|	turquoise	406.05	398.719	0.672208	0.213861	54.536
gi|115552353|dbj|AK232758.1|	turquoise	814.1	810.117	0.847625	0.069746	17.754
gi|115552369|dbj|AK236374.1|	turquoise	1143.9	1142.01	0.927744	0.023061	32.044
gi|115552393|dbj|AK236398.1|	brown	222.3	110.274	0.959119	0.009861	9.932
gi|115552399|dbj|AK236404.1|	grey	306.39	17.5046	0.972333	0.005501	74.198
gi|115552402|dbj|AK236407.1|	turquoise	1544.9	1544.49	0.996456	0.000253	7.48
gi|115552411|dbj|AK236416.1|	blue	254.93	122.316	0.941125	0.016997	57.48
gi|115552412|dbj|AK236417.1|	turquoise	1561.8	1561.36	0.998212	9.07E-05	4.318
gi|115552415|dbj|AK236420.1|	lightyellow	298.53	23.7437	0.977006	0.004171	11.102
gi|115552428|dbj|AK236433.1|	salmon	247.79	27.2813	0.948673	0.013851	116.852
gi|115552430|dbj|AK236435.1|	turquoise	645.03	638.701	0.793279	0.109261	34.206
gi|115552448|dbj|AK236453.1|	turquoise	1443.4	1442.95	0.976526	0.004302	14.526
gi|115552473|dbj|AK236478.1|	turquoise	1365.1	1364.07	0.967721	0.006928	90.036
gi|115552482|dbj|AK236487.1|	brown	114.22	49.4657	0.758528	0.137165	6.222
gi|115552494|dbj|AK236499.1|	turquoise	1527.8	1527.26	0.994483	0.000492	9.328
gi|115552519|dbj|AK236524.1|	green	185.05	50.9591	0.986556	0.001867	48.56
gi|115552523|dbj|AK236528.1|	brown	226.44	82.4642	0.896398	0.039402	90.916
gi|115552527|dbj|AK236532.1|	green	104.08	28.5043	0.835992	0.077741	39.638
gi|115552537|dbj|AK236542.1|	turquoise	779.92	777.257	0.843206	0.072751	16.982
gi|115552575|dbj|AK232780.1|	red	188.46	48.0601	0.978849	0.003681	9.784
gi|115552593|dbj|AK232798.1|	turquoise	1440.9	1440.44	0.976101	0.004419	7.156
gi|115552602|dbj|AK232807.1|	salmon	230.66	28.9222	0.971792	0.005663	69.362
gi|115552623|dbj|AK232828.1|	brown	314.65	103.35	0.929264	0.022342	11.16
gi|115552627|dbj|AK232832.1|	turquoise	168.89	132.063	0.429563	0.470383	2.124
gi|115552638|dbj|AK232843.1|	midnightblue	164.04	28.7866	0.9464	0.014776	3.086

gi|115552724|dbj|AK232929.1|	turquoise	37.617	26.5676	0.171953	0.782146	40.052
gi|115552725|dbj|AK232930.1|	turquoise	1483.1	1482.47	0.988202	0.001536	14.884
gi|115552766|dbj|AK239359.1|	turquoise	1355.1	1354.08	0.968415	0.006706	36.628
gi|115552791|dbj|AK239385.1|	brown	237.41	124.71	0.989653	0.001261	49.456
gi|115552800|dbj|AK239394.1|	turquoise	1462.3	1461.7	0.980905	0.003158	12.556
gi|115552819|dbj|AK239413.1|	green	187.92	49.439	0.984631	0.002282	19.388
gi|115552841|dbj|AK239435.1|	greenyellow	182.78	22.6311	0.986595	0.001859	4.008
gi|115552842|dbj|AK239436.1|	grey	269	15.2521	0.913354	0.030215	431.184
gi|115552865|dbj|AK239459.1|	grey	283.31	15.3666	0.910355	0.031783	34.524
gi|115552903|dbj|AK239497.1|	turquoise	536.07	523.397	0.732034	0.159653	232.17
gi|115552930|dbj|AK239524.1|	turquoise	346.68	323.121	0.616356	0.268227	9.336
gi|115552944|dbj|AK239538.1|	tan	340.83	35.4057	0.963382	0.008365	50.872
gi|115552956|dbj|AK239550.1|	yellow	241.95	45.0467	0.791027	0.11101	15.82
gi|115552967|dbj|AK239553.1|	turquoise	1052.5	1050.69	0.900343	0.037196	9.89
gi|115552977|dbj|AK239563.1|	purple	174.57	31.0413	0.963673	0.008266	90.462
gi|115552989|dbj|AK239575.1|	red	148.78	34.3256	0.906126	0.034036	19.912
gi|115553014|dbj|AK239600.1|	grey60	268.5	32.71	0.994818	0.000447	24.166
gi|115553025|dbj|AK239611.1|	blue	318.65	107.525	0.911465	0.0312	159.888
gi|115553034|dbj|AK239620.1|	turquoise	1139.8	1139.28	0.927807	0.023031	69.82
gi|115553038|dbj|AK239624.1|	brown	280.02	107.875	0.949507	0.013516	330.476
gi|115553060|dbj|AK239646.1|	navy	247.72	30.1321	0.96408	0.008128	83.924
gi|115553080|dbj|AK239666.1|	turquoise	1166.6	1164.97	0.933717	0.02028	22.234
gi|115553090|dbj|AK239676.1|	turquoise	643.29	642.553	0.8061	0.09946	38.74
gi|115553094|dbj|AK239680.1|	turquoise	1387	1386.06	0.97259	0.005425	17.062
gi|115553103|dbj|AK239689.1|	yellow	163.35	56.1824	0.896977	0.039076	78.516
gi|115553121|dbj|AK239707.1|	brown	325.24	94.8637	0.909527	0.03222	105.31
gi|115553137|dbj|AK239723.1|	turquoise	1568.9	1568.55	0.999366	1.92E-05	58.992
gi|115553166|dbj|AK232966.1|	darkturquoise	147.8	13.6578	0.926516	0.023647	17.114
gi|115553171|dbj|AK232969.1|	yellow	84.755	33.9497	0.750787	0.143631	103.796
gi|115553187|dbj|AK232985.1|	turquoise	1460.8	1460.1	0.983495	0.002539	6.554
gi|115553196|dbj|AK232994.1|	greenyellow	56.202	6.00697	-0.12003	0.847546	19.162
gi|115553202|dbj|AK233000.1|	grey	285.77	15.9799	0.940913	0.017088	71.268
gi|115553209|dbj|AK233007.1|	turquoise	1497.2	1496.81	0.987804	0.001614	75.494
gi|115553216|dbj|AK233014.1|	turquoise	1508.1	1507.72	0.988104	0.001555	5.93
gi|115553226|dbj|AK233024.1|	turquoise	1544.3	1543.82	0.996614	0.000236	55.87
gi|115553231|dbj|AK233029.1|	turquoise	1376.2	1375.4	0.970926	0.005925	118.474
gi|115553328|dbj|AK233126.1|	greenyellow	252.17	17.5586	0.935895	0.019295	66.178
gi|115553331|dbj|AK233129.1|	tan	245.78	32.3014	0.946204	0.014857	2.76
gi|115553344|dbj|AK233142.1|	turquoise	127.1	96.058	0.366367	0.544183	8.966
gi|115553351|dbj|AK233149.1|	black	217.67	32.9392	0.931257	0.021411	16.802
gi|115553370|dbj|AK236573.1|	turquoise	1359.2	1358.5	0.962638	0.008621	29.014
gi|115553373|dbj|AK236576.1|	turquoise	134.26	76.2413	0.247299	0.688369	191.344
gi|115553396|dbj|AK236599.1|	turquoise	1541.2	1540.73	0.996345	0.000265	8.912
gi|115553398|dbj|AK236601.1|	darkturquoise	210.91	15.2288	0.943553	0.015962	31.09
gi|115553400|dbj|AK236603.1|	turquoise	98.403	50.5415	0.199001	0.748306	2.668
gi|115553414|dbj|AK236617.1|	tan	320.63	38.4461	0.983132	0.002623	31.808
gi|115553434|dbj|AK236637.1|	lightyellow	301.82	25.5428	0.994942	0.000432	11.5
gi|115553451|dbj|AK236654.1|	turquoise	1466.4	1465.71	0.983049	0.002642	42.71

gi|115553452|dbj|AK236655.1|	turquoise	1101.1	1099.92	0.909083	0.032456	80.03
gi|115553453|dbj|AK236656.1|	turquoise	1390.5	1389.9	0.969763	0.006283	21.354
gi|115553457|dbj|AK236660.1|	turquoise	697.33	693.919	0.808429	0.09771	34.586
gi|115553468|dbj|AK236671.1|	brown	290.99	113.969	0.958188	0.010198	117.404
gi|115553471|dbj|AK236674.1|	red	167.69	43.5627	0.956975	0.010644	46.508
gi|115553473|dbj|AK236676.1|	blue	332.11	99.8909	0.895082	0.040147	21.6
gi|115553496|dbj|AK236699.1|	turquoise	1495.1	1494.65	0.985702	0.002048	33.684
gi|115553497|dbj|AK236700.1|	lightgreen	71.372	16.498	0.997081	0.000189	491.272
gi|115553524|dbj|AK236727.1|	lightgreen	49.288	12.8666	0.842336	0.073348	15.118
gi|115553527|dbj|AK236730.1|	brown	199.63	99.9662	0.942855	0.016257	19.694
gi|115553549|dbj|AK236752.1|	navy	277.8	32.3848	0.982516	0.002768	103.616
gi|115553569|dbj|AK239756.1|	blue	287.17	141.185	0.973239	0.005234	89.178
gi|115553571|dbj|AK239758.1|	turquoise	1498.6	1498.2	0.987869	0.001601	37.594
gi|115553590|dbj|AK239777.1|	turquoise	969.11	967.26	0.888004	0.044229	23.882
gi|115553601|dbj|AK239788.1|	brown	212.33	119.282	0.979189	0.003593	13.296
gi|115553671|dbj|AK239858.1|	yellow	221.79	60.2682	0.910706	0.031598	171.656
gi|115553700|dbj|AK239887.1|	magenta	310.77	43.0285	0.986825	0.001812	42.238
gi|115553719|dbj|AK239906.1|	salmon	148.97	28.8172	0.9792	0.00359	127.23
gi|115553726|dbj|AK239913.1|	turquoise	1553.4	1553.01	0.996891	0.000208	23.416
gi|115553748|dbj|AK239935.1|	blue	328.48	150.915	0.988896	0.001402	36.944
gi|115553764|dbj|AK236768.1|	turquoise	1497.1	1496.64	0.988696	0.00144	2.458
gi|115553811|dbj|AK236816.1|	turquoise	1366.4	1365.67	0.963794	0.008225	18.052
gi|115553814|dbj|AK236819.1|	brown	277.64	123.55	0.983357	0.002571	75.126
gi|115553862|dbj|AK236867.1|	turquoise	1483.7	1483.22	0.985621	0.002065	4.544
gi|115553967|dbj|AK233176.1|	turquoise	1373.9	1373.33	0.96628	0.007395	37.388
gi|115553998|dbj|AK233207.1|	turquoise	1530.5	1530.06	0.994503	0.000489	5.032
gi|115554002|dbj|AK233211.1|	turquoise	1504	1503.61	0.988342	0.001508	5.064
gi|115554007|dbj|AK233216.1|	turquoise	61.945	52.519	0.300167	0.623635	0.674
gi|115554022|dbj|AK233231.1|	red	256.59	45.3366	0.962973	0.008505	152.784
gi|115554027|dbj|AK233236.1|	turquoise	1368.6	1367.55	0.969873	0.006249	40.63
gi|115554046|dbj|AK233255.1|	turquoise	1553.6	1553.18	0.996528	0.000245	0.378
gi|115554063|dbj|AK233272.1|	turquoise	1569.1	1568.74	0.999649	7.88E-06	16.862
gi|115554075|dbj|AK233284.1|	turquoise	962.01	958.416	0.883168	0.047089	128.58
gi|115554089|dbj|AK233298.1|	yellow	124.73	43.5583	0.826509	0.084452	9.822
gi|115554124|dbj|AK233334.1|	black	293.33	40.2721	0.980318	0.003305	126.866
gi|115554129|dbj|AK233339.1|	turquoise	1552	1551.63	0.99728	0.00017	26.128
gi|115554130|dbj|AK233340.1|	yellow	132.63	49.9609	0.861882	0.060326	44.144
gi|115554132|dbj|AK233342.1|	midnightblue	203.25	33.493	0.977947	0.003918	6.814
gi|115554133|dbj|AK233343.1|	navy	222.65	28.4815	0.957307	0.010521	94.646
gi|115554167|dbj|AK239962.1|	turquoise	1558.9	1558.54	0.997928	0.000113	9.9
gi|115554174|dbj|AK239969.1|	turquoise	1122.5	1120.78	0.925224	0.024269	15.384
gi|115554233|dbj|AK240028.1|	turquoise	1571.4	1571.02	0.999774	4.07E-06	1.828
gi|115554282|dbj|AK240077.1|	turquoise	274.6	240.848	0.528383	0.36001	299.774
gi|115554284|dbj|AK240079.1|	turquoise	115.58	89.5669	0.329144	0.588616	31.662
gi|115554299|dbj|AK240094.1|	grey60	217.98	31.9371	0.9897	0.001253	175.436
gi|115554303|dbj|AK240098.1|	turquoise	1321.9	1320.84	0.962201	0.008772	28.906
gi|115554309|dbj|AK240104.1|	magenta	234.88	25.9444	0.882545	0.047461	11.014
gi|115554312|dbj|AK240107.1|	yellow	282.65	70.3144	0.93481	0.019784	217.844

gi|115554315|dbj|AK240110.1|	lightcyan	270.33	25.1504	0.972668	0.005402	30.296
gi|115554351|dbj|AK240146.1|	turquoise	1503.2	1502.76	0.989919	0.001213	40.826
gi|115554358|dbj|AK233368.1|	turquoise	215.83	167.205	0.43259	0.466906	78.666
gi|115554371|dbj|AK233381.1|	turquoise	1429.5	1428.84	0.979393	0.00354	73.068
gi|115554372|dbj|AK233382.1|	purple	237.32	33.1393	0.977241	0.004107	59.254
gi|115554373|dbj|AK233383.1|	salmon	206.64	29.0284	0.968779	0.006591	26.776
gi|115554376|dbj|AK233386.1|	turquoise	480.53	473.711	0.733066	0.158759	24.874
gi|115554409|dbj|AK233419.1|	midnightblue	251.57	35.6065	0.990835	0.001052	232.884
gi|115554410|dbj|AK233420.1|	darkred	114.41	11.7624	0.874915	0.052098	164.778
gi|115554415|dbj|AK233425.1|	turquoise	1486.6	1486	0.987024	0.001771	21.904
gi|115554417|dbj|AK233427.1|	blue	188.62	83.2891	0.856073	0.064112	24.136
gi|115554452|dbj|AK233462.1|	turquoise	1558.6	1558.15	0.997906	0.000115	7.258
gi|115554459|dbj|AK233469.1|	blue	296.17	146.445	0.982156	0.002854	19.05
gi|115554492|dbj|AK233502.1|	turquoise	1518.5	1517.96	0.992565	0.000769	25.046
gi|115554528|dbj|AK233538.1|	greenyellow	143.9	19.0739	0.950998	0.012925	19.244
gi|115554532|dbj|AK233542.1|	lightcyan	290.44	25.6572	0.981093	0.003112	124.416
gi|115554563|dbj|AK236973.1|	black	293.44	41.4012	0.984349	0.002345	22.35
gi|115554566|dbj|AK236976.1|	midnightblue	264.72	30.8505	0.959952	0.009563	16.28
gi|115554588|dbj|AK236998.1|	turquoise	1556.5	1556.1	0.996844	0.000213	40.588
gi|115554610|dbj|AK237020.1|	turquoise	1368.1	1367.17	0.968562	0.00666	30.334
gi|115554617|dbj|AK237028.1|	turquoise	162.21	140.733	0.452048	0.444686	5.362
gi|115554649|dbj|AK237060.1|	turquoise	58.808	22.0805	0.107251	0.863706	15.64
gi|115554680|dbj|AK237091.1|	turquoise	1456.7	1455.98	0.983785	0.002473	21.26
gi|115554713|dbj|AK237124.1|	green	96.787	26.0815	0.808159	0.097912	14.308
gi|115554725|dbj|AK237136.1|	turquoise	1290.8	1289.99	0.95248	0.012346	18.766
gi|115554738|dbj|AK237149.1|	blue	314.41	123.722	0.943296	0.016071	11.878
gi|115554762|dbj|AK233574.1|	blue	214.1	93.6038	0.880702	0.048569	98.264
gi|115554769|dbj|AK233581.1|	turquoise	1420.4	1419.98	0.973404	0.005186	17.066
gi|115554826|dbj|AK233638.1|	turquoise	675.8	667.707	0.794417	0.10838	110.256
gi|115554840|dbj|AK233652.1|	turquoise	505.86	491.869	0.716403	0.173376	31.388
gi|115554855|dbj|AK233667.1|	turquoise	1104.2	1103.33	0.922111	0.025787	22.766
gi|115554857|dbj|AK233669.1|	grey	314.47	16.4533	0.940244	0.017377	181.488
gi|115554899|dbj|AK233711.1|	turquoise	1170.4	1169.82	0.934907	0.01974	84.312
gi|115554919|dbj|AK233731.1|	turquoise	1120.4	1118.53	0.923164	0.02527	94.22
gi|115554929|dbj|AK233741.1|	green	250.97	40.4263	0.900733	0.03698	11.244
gi|115554973|dbj|AK240172.1|	yellow	282.09	72.4601	0.938554	0.018115	42.182
gi|115554977|dbj|AK240176.1|	turquoise	1133.6	1132.64	0.91678	0.028456	14.732
gi|115554982|dbj|AK240181.1|	darkturquoise	167.77	17.5162	0.985978	0.001989	90.776
gi|115554993|dbj|AK240192.1|	turquoise	1527.6	1527.2	0.991652	0.000914	3.97
gi|115555012|dbj|AK240211.1|	turquoise	1012.6	1010.42	0.902878	0.0358	208.94
gi|115555020|dbj|AK240219.1|	turquoise	961.76	959.13	0.88558	0.045655	24.182
gi|115555021|dbj|AK240220.1|	turquoise	1543.3	1542.93	0.994314	0.000514	30.804
gi|115555023|dbj|AK240222.1|	turquoise	1221	1219.7	0.937136	0.018741	55.294
gi|115555058|dbj|AK240257.1|	purple	127.35	13.0906	0.739673	0.153067	52.632
gi|115555063|dbj|AK240262.1|	green	153.3	31.7696	0.857884	0.062924	28.772
gi|115555097|dbj|AK240296.1|	turquoise	235	209.777	0.511209	0.378689	64.742
gi|115555129|dbj|AK240329.1|	brown	172.18	82.6702	0.896854	0.039145	152.368
gi|115555134|dbj|AK240334.1|	turquoise	1553.3	1552.9	0.996645	0.000233	8.87

gi|115555150|dbj|AK233765.1|	salmon	184.89	27.0445	0.950821	0.012995	98.898
gi|115555162|dbj|AK233777.1|	turquoise	159.31	121.118	0.417785	0.483966	58.212
gi|115555163|dbj|AK233778.1|	grey	77.274	7.90457	0.032561	0.95855	64.804
gi|115555188|dbj|AK233803.1|	blue	310.38	144.924	0.980055	0.003371	41.642
gi|115555249|dbj|AK233864.1|	turquoise	575.13	567.496	0.762126	0.134189	20.074
gi|115555306|dbj|AK233921.1|	turquoise	457.92	455.051	0.722953	0.167586	24.126
gi|115555322|dbj|AK233937.1|	turquoise	440.42	432.172	0.707277	0.181537	24.036
gi|115555353|dbj|AK233966.1|	lightcyan	292.25	24.6414	0.964724	0.007911	20.3
gi|115555354|dbj|AK233967.1|	brown	188.82	92.2685	0.912911	0.030446	29.302
gi|115555420|dbj|AK234033.1|	brown	180.13	108.7	0.957044	0.010618	20.712
gi|115555451|dbj|AK234064.1|	turquoise	1285.6	1284.44	0.955875	0.011053	38.33
gi|115555475|dbj|AK234088.1|	turquoise	198.27	182.491	0.490783	0.401196	18.794
gi|115555495|dbj|AK234108.1|	turquoise	1393.1	1392.43	0.970383	0.006091	38.71
gi|115555527|dbj|AK234140.1|	blue	331.21	105.483	0.907441	0.03333	24.796
gi|115555541|dbj|AK237173.1|	turquoise	133.77	115.389	0.423639	0.477205	51.576
gi|115555561|dbj|AK237193.1|	brown	229.02	105.081	0.947978	0.014132	4.17
gi|115555567|dbj|AK237199.1|	turquoise	145.96	45.9975	0.120142	0.847399	3.008
gi|115555619|dbj|AK237252.1|	turquoise	918.18	916.477	0.86607	0.05764	84.994
gi|115555639|dbj|AK237272.1|	green	229.92	53.2706	0.982111	0.002865	154.294
gi|115555653|dbj|AK237286.1|	turquoise	99.539	34.7291	0.1016	0.870862	83.954
gi|115555675|dbj|AK237308.1|	turquoise	858.49	854.217	0.857387	0.06325	17.792
gi|115555693|dbj|AK237326.1|	turquoise	1355.5	1354.45	0.968013	0.006834	57.284
gi|115555697|dbj|AK237330.1|	darkgreen	201.41	20.6525	0.952691	0.012264	2.984
gi|117661185|gb|DQ629176.1|	grey	224.44	14.404	0.887747	0.044379	281.95
gi|118403765|ref|NM_001078687.1|	salmon	238.69	28.185	0.959206	0.00983	209.95
gi|118403777|ref|NM_001078685.1|	turquoise	1409.8	1409.23	0.970601	0.006024	13.598
gi|118403821|ref|NM_001078679.1|	turquoise	1532	1531.59	0.994454	0.000495	31.586
gi|118403911|ref|NM_001078662.1|	green	100.82	22.6464	0.767356	0.129899	95.426
gi|124558111|gb|EF154832.1|	turquoise	1553	1552.51	0.997952	0.000111	6.294
gi|1245714|gb|S80644.1|	turquoise	1254.7	1254.13	0.946934	0.014557	21.028
gi|125630297|ref|NM_001005152.2|	pink	230.9	46.899	0.993368	0.000648	32.382
gi|125630305|ref|NM_214281.2|	turquoise	1484.7	1484.3	0.984849	0.002234	6.718
gi|138753472|emb|AM503091.1|	turquoise	1519	1518.51	0.992617	0.000761	29.524
gi|139530445|gb|EF486522.1|	salmon	212.29	29.668	0.981482	0.003016	527.782
gi|146198449|dbj|AB292846.1|	blue	344.42	114.713	0.92573	0.024025	28.852
gi|146741279|dbj|AB271920.1|	turquoise	1481.9	1481.54	0.986081	0.001967	8.696
gi|147899058|ref|NM_001097509.1|	pink	271.08	38.2974	0.941298	0.016922	37.162
gi|147900046|ref|NM_001097474.1|	lightcyan	299.88	25.4673	0.976217	0.004387	258.694
gi|147900501|ref|NM_001097506.1|	turquoise	1091.8	1090.65	0.906993	0.03357	9.732
gi|147902484|ref|NM_001097419.1|	turquoise	1436	1435.28	0.980487	0.003262	46.312
gi|147903738|ref|NM_001097413.1|	turquoise	1494.8	1494.41	0.986026	0.001979	7.002
gi|147905343|ref|NM_001097438.1|	turquoise	1209.6	1208.7	0.933332	0.020456	7.538
gi|147906088|ref|NM_213946.2|	grey	273.66	12.2692	0.816903	0.091423	50.504
gi|148222590|ref|NM_001097486.1|	lightcyan	281.29	25.6915	0.972783	0.005368	43.282
gi|148223598|ref|NM_001097476.1|	magenta	337.18	44.0052	0.991056	0.001014	87.812
gi|148224637|ref|NM_001097469.1|	salmon	74.34	16.4121	0.833091	0.079775	34.502
gi|148225749|ref|NM_001097504.1|	turquoise	1283.6	1282.91	0.955928	0.011033	18.95
gi|148228719|ref|NM_001097439.1|	turquoise	1133.9	1131.84	0.924502	0.024618	105.792

gi|148230690|ref|NM_001097489.1|	darkred	74.672	9.64525	0.817787	0.090774	1.78
gi|148231659|ref|NM_001097516.1|	turquoise	1519.8	1519.41	0.99088	0.001044	3.378
gi|148232727|ref|NM_001097447.1|	turquoise	1546.7	1546.2	0.997007	0.000197	22.466
gi|148233297|ref|NM_001097485.1|	turquoise	1535	1534.57	0.99444	0.000497	45.768
gi|148234109|ref|NM_001093735.1|	turquoise	164.05	147.916	0.459529	0.436209	29.658
gi|148235350|ref|NM_001097475.1|	navy	263.75	27.4338	0.946175	0.014869	83.108
gi|148237120|ref|NM_001097421.1|	turquoise	1429.2	1428.72	0.974256	0.004939	17.84
gi|148237281|ref|NM_001097478.1|	turquoise	744.91	740.844	0.821507	0.08806	526.736
gi|148747452|ref|NM_001098603.1|	turquoise	1527	1526.57	0.993521	0.000625	5.552
gi|149364039|gb|EF468461.1|	salmon	256.52	25.4571	0.92837	0.022764	62.83
gi|149944498|ref|NM_214343.2|	turquoise	937.2	934.11	0.879462	0.049318	81.652
gi|150246527|ref|NM_001044575.2|	turquoise	463.22	457.303	0.708447	0.180484	24.58
gi|153791354|ref|NM_001099940.1|	turquoise	1442	1441.56	0.977221	0.004113	3.862
gi|153791726|ref|NM_001099925.1|	turquoise	1553.6	1553.18	0.996528	0.000245	0.378
gi|153792026|ref|NM_001099932.1|	midnightblue	153.92	27.8061	0.938278	0.018236	324.414
gi|153792048|ref|NM_001099934.1|	turquoise	1200.7	1198.93	0.937656	0.018511	86.282
gi|154147629|ref|NM_001100190.1|	yellow	144.89	53.8581	0.882256	0.047634	109.278
gi|155369755|ref|NM_001101026.1|	turquoise	1174	1172.12	0.932838	0.020682	17.82
gi|155369765|ref|NM_001101031.1|	turquoise	1458.1	1457.53	0.984418	0.00233	13.62
gi|156120135|ref|NM_001101827.1|	turquoise	224.13	184.181	0.458661	0.437191	49.846
gi|156151354|ref|NM_001037146.2|	brown	267.45	127.346	0.990957	0.001031	82.46
gi|157427686|ref|NM_214374.2|	midnightblue	192.45	33.5073	0.97793	0.003923	102.71
gi|157427704|ref|NM_001105288.1|	yellow	164.46	67.9264	0.941979	0.01663	8.99
gi|157427710|ref|NM_001105292.1|	turquoise	1472.8	1472.18	0.983553	0.002526	9.4
gi|157427714|ref|NM_001105294.1|	darkgreen	257.04	23.5947	0.990936	0.001034	14.842
gi|157427721|ref|NM_001105299.1|	lightcyan	272.49	26.0511	0.995164	0.000403	100.532
gi|157427723|ref|NM_001105300.1|	greenyellow	100.24	4.4091	0.358846	0.553108	66.934
gi|157427727|ref|NM_001105302.1|	turquoise	1477.2	1476.64	0.986818	0.001813	24.644
gi|157427731|ref|NM_001105304.1|	turquoise	1372.7	1372.16	0.966683	0.007264	41.878
gi|15824733|gb|AF329358.1|AF329358	magenta	333.17	40.6207	0.973351	0.005201	20.066
gi|158262676|ref|NM_001109945.1|	turquoise	1502.9	1502.57	0.988217	0.001533	2.378
gi|158262678|ref|NM_001109946.1|	turquoise	1563.2	1562.84	0.997902	0.000115	2.164
gi|158262680|ref|NM_001109947.1|	green	88.225	18.9772	0.72815	0.163032	45.834
gi|158631261|ref|NM_001099936.1|	turquoise	165.81	151.631	0.498668	0.392472	83.53
gi|15864598|emb|AJ309014.1|	turquoise	109.81	87.0384	0.336249	0.580085	6.046
gi|160420270|ref|NM_001044582.1|	turquoise	1510	1509.59	0.989964	0.001205	125.334
gi|162139822|ref|NM_001111257.1|	turquoise	839.42	835.504	0.854533	0.065129	70.368
gi|16304807|emb|AJ416019.1|	blue	325.72	144.437	0.978425	0.003792	27.302
gi|163310772|ref|NM_001097521.2|	brown	175.72	106.694	0.956117	0.010962	50.094
gi|164394|gb|L10363.1|PIGBSP	turquoise	1553.6	1553.18	0.996528	0.000245	8.314
gi|164486|gb|M29072.1|PIGHEP1	turquoise	1557.8	1557.3	0.997993	0.000108	33.572
gi|164518957|ref|NM_001113287.1|	turquoise	363.8	343.232	0.624738	0.259858	592.974
gi|164664445|ref|NM_001113439.1|	tan	340.7	35.763	0.966943	0.007179	113.09
gi|164664451|ref|NM_001113442.1|	turquoise	173.22	155.494	0.455163	0.441152	23.3
gi|165973437|ref|NM_001113701.1|	turquoise	363.68	352.654	0.664381	0.221274	8.304
gi|166796042|ref|NM_001114275.1|	turquoise	1514.3	1513.78	0.992562	0.000769	18.392
gi|166796054|ref|NM_001114281.1|	turquoise	1402.4	1401.47	0.975169	0.00468	14.564
gi|167908788|ref|NM_001114675.1|	turquoise	1152.2	1150.51	0.92558	0.024097	66.502

gi|167908790|ref|NM_001114676.1|	turquoise	295.62	273.416	0.572332	0.313297	73.26
gi|167908792|ref|NM_001114670.1|	turquoise	1153.7	1152.42	0.921628	0.026026	78.072
gi|167908796|ref|NM_001114672.1|	turquoise	376.91	371.342	0.665921	0.21981	34.362
gi|171905894|gb|EU561660.1|	black	135.82	18.2969	0.779388	0.120187	265.254
gi|172072681|ref|NM_001122994.1|	turquoise	1570.2	1569.77	0.999543	1.17E-05	69.454
gi|172073149|ref|NM_001122990.1|	turquoise	251.45	229.488	0.536971	0.350756	36.442
gi|178056483|ref|NM_001123101.1|	black	250.32	36.4713	0.93181	0.021155	41.854
gi|178056487|ref|NM_001123204.1|	royalblue	162.72	19.3514	0.934473	0.019936	39.08
gi|178056489|ref|NM_001123144.1|	turquoise	1567.6	1567.15	0.99954	1.18E-05	15.676
gi|178056503|ref|NM_001123076.1|	pink	248.04	44.7127	0.980819	0.00318	170.728
gi|178056505|ref|NM_001123131.1|	turquoise	1473.2	1472.64	0.985108	0.002177	10.508
gi|178056515|ref|NM_001123121.1|	turquoise	1545.2	1544.8	0.995741	0.000333	39.804
gi|178056521|ref|NM_001123111.1|	turquoise	1024.2	1022.4	0.899715	0.037544	3.882
gi|178056525|ref|NM_001123147.1|	navy	192.04	25.3517	0.932299	0.02093	10.128
gi|178056541|ref|NM_001123177.1|	turquoise	1507.5	1507.03	0.989698	0.001253	1.04
gi|178056566|ref|NM_001123160.1|	turquoise	171.26	151.642	0.459812	0.435888	53.418
gi|178056609|ref|NM_001123174.1|	turquoise	1368	1367.39	0.969597	0.006335	24.954
gi|178056622|ref|NM_001123213.1|	turquoise	920.18	917.938	0.873528	0.052956	72.642
gi|178056656|ref|NM_001123157.1|	turquoise	1237.2	1236.1	0.947469	0.014338	82.412
gi|178056662|ref|NM_001123124.1|	turquoise	1549.1	1548.73	0.995732	0.000334	16.886
gi|178056668|ref|NM_001123099.1|	turquoise	201.11	184.906	0.493675	0.397991	251.118
gi|178056676|ref|NM_001123078.1|	turquoise	310.75	303.736	0.622852	0.261735	98.016
gi|178056709|ref|NM_001123194.1|	turquoise	1531.9	1531.49	0.993459	0.000634	14.114
gi|178056780|ref|NM_001123122.1|	blue	322.07	125.839	0.946743	0.014635	105.144
gi|178056859|ref|NM_001123091.1|	navy	226.48	30.4145	0.972299	0.005511	45.228
gi|178056875|ref|NM_001123200.1|	turquoise	1498.4	1497.81	0.989192	0.001347	17.032
gi|178056883|ref|NM_001123198.1|	turquoise	1460.8	1460.29	0.982058	0.002877	28.69
gi|178057066|ref|NM_001123096.1|	turquoise	1298.1	1297.27	0.957615	0.010408	33.866
gi|178057176|ref|NM_001123090.1|	turquoise	1486.3	1485.79	0.986853	0.001806	49.468
gi|178057228|ref|NM_001123071.1|	turquoise	1563.4	1563.06	0.998103	9.91E-05	31.864
gi|178057315|ref|NM_001123097.1|	turquoise	1208.5	1207.1	0.941869	0.016677	69.244
gi|1841944|emb|X95846.1|	midnightblue	215.05	36.2315	0.994412	0.000501	5.86
gi|186886351|gb|EU617320.1|	turquoise	1502	1501.61	0.988646	0.00145	3.816
gi|187672044|gb|EU650276.1|	red	246.02	43.6677	0.95506	0.011359	8.976
gi|190352252|gb|EU780792.1|	turquoise	1553.7	1553.31	0.996728	0.000225	8.348
gi|190360618|ref|NM_001128433.1|	red	181.42	47.8391	0.977446	0.004052	26.03
gi|190360622|ref|NM_001128462.1|	turquoise	1513.6	1513.2	0.990733	0.001069	16.89
gi|190360630|ref|NM_001128465.1|	turquoise	496.6	491.058	0.733613	0.158285	59.79
gi|190360646|ref|NM_001128455.1|	turquoise	239.03	198.843	0.471202	0.423053	4.874
gi|190360656|ref|NM_001128470.1|	salmon	266.79	23.7253	0.909577	0.032194	88.004
gi|190360658|ref|NM_001128471.1|	turquoise	1523.9	1523.35	0.993818	0.000583	29.848
gi|1912|emb|X07617.1|	salmon	179.15	31.8921	0.998845	4.71E-05	37.168
gi|1920|emb|X15073.1|	turquoise	1545	1544.63	0.995228	0.000395	16.296
gi|194018685|ref|NM_001129963.1|	navy	268.32	29.0049	0.954202	0.011684	475.18
gi|194018691|ref|NM_001129967.1|	turquoise	1056.1	1054.76	0.905821	0.034201	2.908
gi|194018701|ref|NM_001129947.1|	pink	282.18	39.1265	0.947223	0.014439	4239.712
gi|194018717|ref|NM_001129954.1|	lightyellow	283.73	24.3574	0.974197	0.004956	5802.936
gi|194018721|ref|NM_001129949.1|	green	255.72	50.3454	0.960858	0.009241	3313.478

gi|194033418|ref|XM_001924233.1|	lightgreen	78.951	14.7114	0.956332	0.010882	79.502
gi|194033446|ref|XM_001927265.1|	turquoise	1537.4	1536.98	0.995797	0.000327	66.866
gi|194033456|ref|XM_001924731.1|	turquoise	1522.3	1521.89	0.992821	0.000729	5.918
gi|194033493|ref|XM_001924291.1|	yellow	163.83	42.9696	0.801428	0.102999	52.294
gi|194033502|ref|XM_001926129.1|	darkturquoise	211.98	17.1556	0.977348	0.004079	30.822
gi|194033556|ref|XM_001927696.1|	turquoise	1483.4	1482.78	0.988547	0.001469	26.01
gi|194033590|ref|XM_001929195.1|	red	254.38	49.7468	0.983314	0.002581	201.388
gi|194033594|ref|XM_001927795.1|	turquoise	1561.8	1561.4	0.998176	9.35E-05	47.206
gi|194033596|ref|XM_001927813.1|	turquoise	1518.2	1517.8	0.992348	0.000803	7.738
gi|194033603|ref|XM_001925557.1|	turquoise	61.987	45.6704	0.274193	0.655312	3.528
gi|194033615|ref|XM_001924714.1|	turquoise	1514.6	1514.11	0.991982	0.000861	26.79
gi|194033624|ref|XM_001924823.1|	turquoise	1000.3	998.236	0.891333	0.042293	5.836
gi|194033632|ref|XM_001927932.1|	turquoise	1473.9	1473.29	0.983344	0.002574	7.962
gi|194033649|ref|XM_001925294.1|	turquoise	172.62	116.906	0.364076	0.546899	18.762
gi|194033718|ref|XM_001926719.1|	lightyellow	295.48	25.1233	0.981996	0.002892	31.812
gi|194033747|ref|XM_001927557.1|	turquoise	1185.1	1184.04	0.935369	0.019532	7.524
gi|194033755|ref|XM_001924851.1|	darkred	51.929	10.5237	0.898614	0.038158	52.224
gi|194033834|ref|XM_001924991.1|	turquoise	1415.3	1414.56	0.977511	0.004035	39.49
gi|194033859|ref|XM_001927866.1|	yellow	241.9	82.2804	0.989262	0.001334	197.16
gi|194033869|ref|XM_001924394.1|	turquoise	1420	1419.47	0.977545	0.004026	4.916
gi|194033897|ref|XM_001926189.1|	turquoise	1426.5	1425.88	0.977516	0.004034	11.414
gi|194033899|ref|XM_001928651.1|	turquoise	1553.6	1553.18	0.996528	0.000245	1.558
gi|194033910|ref|XM_001928928.1|	yellow	180.22	78.5059	0.980117	0.003356	63.556
gi|194033956|ref|XM_001926015.1|	turquoise	1550	1549.6	0.995851	0.000321	8.138
gi|194033964|ref|XM_001925719.1|	brown	253.16	128.302	0.996386	0.000261	5.85
gi|194034057|ref|XM_001924352.1|	turquoise	1516.7	1516.34	0.990922	0.001037	1.288
gi|194034095|ref|XM_001928221.1|	turquoise	939.66	938.234	0.889842	0.043157	1.686
gi|194034109|ref|XM_001927571.1|	turquoise	1094.8	1092.76	0.91551	0.029104	98.244
gi|194034148|ref|XM_001924679.1|	red	204.15	50.0207	0.987803	0.001614	26.202
gi|194034150|ref|XM_001927070.1|	turquoise	844.67	839.69	0.851037	0.067453	2.956
gi|194034162|ref|XM_001925693.1|	brown	290.24	104.96	0.93395	0.020174	59.212
gi|194034170|ref|XM_001924292.1|	turquoise	811.09	808.845	0.837946	0.076379	11.722
gi|194034216|ref|XM_001928595.1|	turquoise	227.47	213.548	0.528683	0.359684	77.6
gi|194034220|ref|XM_001928748.1|	turquoise	190.88	173.873	0.4887	0.403508	20.62
gi|194034226|ref|XM_001928937.1|	turquoise	527.91	521.661	0.74288	0.150326	194.496
gi|194034244|ref|XM_001925555.1|	turquoise	1561.8	1561.31	0.99843	7.47E-05	11.6
gi|194034272|ref|XM_001924582.1|	turquoise	1553.6	1553.18	0.996528	0.000245	2.786
gi|194034274|ref|XM_001924792.1|	turquoise	1545.9	1545.49	0.996557	0.000242	8.448
gi|194034359|ref|XM_001927037.1|	blue	301.63	146.492	0.982033	0.002883	65.002
gi|194034424|ref|XM_001926336.1|	darkred	66.416	7.34754	0.739659	0.153079	11.988
gi|194034434|ref|XM_001925498.1|	turquoise	1468.4	1467.71	0.985291	0.002137	2.852
gi|194034436|ref|XM_001927720.1|	turquoise	410.98	404.578	0.678637	0.207825	64.164
gi|194034443|ref|XM_001928249.1|	turquoise	1547.5	1547.14	0.995243	0.000394	16.81
gi|194034473|ref|XM_001924194.1|	turquoise	1267.9	1266.88	0.946537	0.01472	11.724
gi|194034521|ref|XM_001926126.1|	turquoise	1025.6	1022.72	0.899287	0.037783	48.586
gi|194034626|ref|XM_001929026.1|	grey	63.051	9.36151	0.061841	0.921312	44.412
gi|194034692|ref|XM_001928709.1|	turquoise	1198.2	1197.15	0.930685	0.021677	12.96
gi|194034703|ref|XM_001926714.1|	turquoise	293.94	283.178	0.604232	0.280455	6.914

gi|194034713|ref|XM_001925291.1|	turquoise	1521.6	1521.22	0.99058	0.001096	14.392
gi|194034719|ref|XM_001925911.1|	turquoise	1556.4	1556.07	0.996771	0.00022	3.202
gi|194034722|ref|XM_001924603.1|	turquoise	1443.6	1443.09	0.979606	0.003485	2.372
gi|194034729|ref|XM_001924943.1|	turquoise	1534.5	1534.14	0.993918	0.000569	52.876
gi|194034776|ref|XM_001928952.1|	turquoise	1466	1465.4	0.985205	0.002155	12.016
gi|194034818|ref|XM_001927452.1|	turquoise	451.38	441.175	0.703201	0.185216	7.334
gi|194034832|ref|XM_001928118.1|	turquoise	1508.1	1507.68	0.989036	0.001376	28.392
gi|194034834|ref|XM_001925793.1|	yellow	174.73	69.5232	0.94754	0.01431	106.26
gi|194034847|ref|XM_001925969.1|	turquoise	173.86	154.408	0.47488	0.418928	23.066
gi|194034864|ref|XM_001929220.1|	magenta	300.34	39.7605	0.969576	0.006341	25.36
gi|194034892|ref|XM_001929418.1|	turquoise	1430.5	1429.82	0.978621	0.00374	14.156
gi|194034896|ref|XM_001924578.1|	blue	330.68	145.599	0.981253	0.003073	57.344
gi|194034933|ref|XM_001927442.1|	turquoise	1459.3	1458.63	0.984023	0.002418	63.162
gi|194034946|ref|XM_001924579.1|	turquoise	595.7	589.17	0.77472	0.123928	38.052
gi|194034959|ref|XM_001927036.1|	turquoise	347.02	323.747	0.607179	0.277469	319.06
gi|194034969|ref|XM_001925674.1|	greenyellow	244.42	18.2783	0.909198	0.032394	682.554
gi|194034997|ref|XM_001928355.1|	turquoise	1532.4	1531.95	0.993205	0.000672	17.406
gi|194035041|ref|XM_001927251.1|	turquoise	1450.6	1450.03	0.978225	0.003844	59.61
gi|194035108|ref|XM_001926680.1|	red	132.71	32.9779	0.895543	0.039886	24.248
gi|194035136|ref|XM_001924656.1|	green	272.27	48.606	0.955363	0.011245	52.02
gi|194035178|ref|XM_001925137.1|	yellow	230.29	76.258	0.971771	0.005669	41.71
gi|194035184|ref|XM_001924626.1|	turquoise	591.85	584.942	0.774006	0.124503	45.06
gi|194035192|ref|XM_001924962.1|	turquoise	1410.1	1409.24	0.976777	0.004233	25.788
gi|194035197|ref|XM_001925318.1|	turquoise	1191.9	1190.22	0.936381	0.019078	112.7
gi|194035207|ref|XM_001925835.1|	turquoise	1564.3	1563.91	0.998191	9.23E-05	13.586
gi|194035216|ref|XM_001928520.1|	lightyellow	306.32	25.8033	0.991864	0.00088	57.412
gi|194035227|ref|XM_001928768.1|	turquoise	794.07	789.12	0.838536	0.075969	126.208
gi|194035243|ref|XM_001927441.1|	turquoise	1555.1	1554.65	0.99774	0.000129	48.55
gi|194035253|ref|XM_001925769.1|	turquoise	1297.9	1297.19	0.949852	0.013379	1.712
gi|194035288|ref|XM_001927095.1|	yellow	267.72	78.7735	0.970013	0.006205	84.276
gi|194035296|ref|XM_001927594.1|	turquoise	357.14	349.031	0.664204	0.221442	148.982
gi|194035333|ref|XM_001925774.1|	lightgreen	81.027	12.3635	0.90876	0.032627	32.802
gi|194035410|ref|XM_001926931.1|	turquoise	1516.8	1516.25	0.991927	0.00087	4.766
gi|194035412|ref|XM_001925476.1|	turquoise	1534.1	1533.7	0.993225	0.000669	22.126
gi|194035414|ref|XM_001925502.1|	turquoise	1392.7	1392.14	0.96833	0.006733	13.306
gi|194035424|ref|XM_001926628.1|	turquoise	674	669.607	0.800289	0.103867	30.536
gi|194035434|ref|XM_001926912.1|	turquoise	1282.3	1281.49	0.947001	0.01453	6.586
gi|194035451|ref|XM_001928072.1|	turquoise	1517	1516.49	0.992745	0.000741	17.79
gi|194035475|ref|XM_001924744.1|	turquoise	1484.9	1484.45	0.983794	0.00247	6.326
gi|194035479|ref|XM_001924978.1|	turquoise	1538.4	1538.01	0.993822	0.000582	9.712
gi|194035517|ref|XM_001925462.1|	turquoise	1160.7	1158.98	0.932045	0.021047	46
gi|194035561|ref|XM_001924535.1|	turquoise	1355.2	1354.14	0.966234	0.00741	32.684
gi|194035566|ref|XM_001925085.1|	turquoise	367.23	364.533	0.684552	0.202314	1.92
gi|194035578|ref|XM_001928185.1|	turquoise	123.35	93.8958	0.350117	0.563501	4.064
gi|194035588|ref|XM_001924955.1|	turquoise	1075.2	1073.07	0.91444	0.029654	35.426
gi|194035600|ref|XM_001927804.1|	pink	183.63	38.7932	0.95279	0.012226	136.806
gi|194035607|ref|XM_001928505.1|	turquoise	1476.3	1475.71	0.987546	0.001665	78.814
gi|194035627|ref|XM_001924280.1|	salmon	96.334	18.2177	0.862982	0.059616	26.326

gi|194035631|ref|XM_001924325.1|	turquoise	1546.9	1546.45	0.997262	0.000172	82.598
gi|194035643|ref|XM_001927050.1|	turquoise	504.21	499.344	0.75404	0.140903	100.666
gi|194035657|ref|XM_001928441.1|	turquoise	1091.9	1090.21	0.910194	0.031868	52.744
gi|194035679|ref|XM_001925389.1|	turquoise	821.68	818.688	0.841426	0.073973	441.622
gi|194035708|ref|XM_001927062.1|	turquoise	1532.3	1531.82	0.994928	0.000433	18.184
gi|194035716|ref|XM_001925987.1|	turquoise	1310.1	1309.06	0.955228	0.011295	18.55
gi|194035722|ref|XM_001926997.1|	turquoise	1360.7	1359.93	0.968888	0.006557	47.808
gi|194035737|ref|XM_001928295.1|	brown	261.96	117.791	0.971449	0.005766	104.47
gi|194035753|ref|XM_001927135.1|	turquoise	1567.1	1566.69	0.999496	1.36E-05	53.61
gi|194035832|ref|XM_001928786.1|	turquoise	249.81	238.458	0.575555	0.309936	42.056
gi|194035836|ref|XM_001928869.1|	turquoise	1343.5	1342.82	0.960077	0.009518	55.688
gi|194035846|ref|XM_001928960.1|	blue	323.34	151.525	0.989641	0.001264	106.852
gi|194035848|ref|XM_001928966.1|	red	178.14	47.2082	0.974381	0.004903	43.322
gi|194035852|ref|XM_001929031.1|	turquoise	403	395.549	0.671249	0.214765	3.046
gi|194035880|ref|XM_001929177.1|	turquoise	1490.6	1490.24	0.986431	0.001894	1.636
gi|194035984|ref|XM_001925466.1|	turquoise	298.34	277.527	0.578775	0.306588	105.11
gi|194036005|ref|XM_001926377.1|	turquoise	208.57	150.906	0.388279	0.518347	133.048
gi|194036030|ref|XM_001928495.1|	turquoise	146.32	96.9727	0.328297	0.589634	66.226
gi|194036038|ref|XM_001928648.1|	blue	326.95	142.834	0.977239	0.004108	26.67
gi|194036052|ref|XM_001926689.1|	turquoise	1556.9	1556.47	0.996831	0.000214	1.79
gi|194036068|ref|XM_001926844.1|	turquoise	135.22	112.638	0.416439	0.485525	49.156
gi|194036099|ref|XM_001929400.1|	turquoise	1356.3	1355.77	0.965717	0.007581	21.056
gi|194036101|ref|XM_001929371.1|	turquoise	165.21	115.557	0.363922	0.547081	32.786
gi|194036118|ref|XM_001927231.1|	brown	149.49	86.6583	0.90679	0.033679	24.636
gi|194036122|ref|XM_001929505.1|	turquoise	1529.5	1529.09	0.993155	0.000679	9.154
gi|194036140|ref|XM_001929572.1|	blue	340.87	135.456	0.965439	0.007673	17.206
gi|194036211|ref|XM_001927870.1|	turquoise	1357	1356.51	0.966673	0.007267	2.084
gi|194036224|ref|XM_001929641.1|	blue	308.42	150.293	0.988071	0.001561	75.73
gi|194036252|ref|XM_001929660.1|	green	190.46	51.7022	0.989103	0.001363	41.324
gi|194036256|ref|XM_001929662.1|	turquoise	1258.5	1257.16	0.949181	0.013647	3.964
gi|194036270|ref|XM_001929667.1|	turquoise	1055.2	1053.24	0.908126	0.032964	44.224
gi|194036282|ref|XM_001924669.1|	brown	214.49	118.564	0.981315	0.003057	14.186
gi|194036295|ref|XM_001926489.1|	turquoise	1386.2	1385.27	0.969746	0.006288	28.862
gi|194036309|ref|XM_001927637.1|	navy	226.5	25.1436	0.928774	0.022574	148.8
gi|194036317|ref|XM_001927969.1|	turquoise	1523.7	1523.3	0.993635	0.000609	14.874
gi|194036348|ref|XM_001928686.1|	turquoise	1428.4	1427.85	0.979359	0.003549	28.404
gi|194036350|ref|XM_001926399.1|	turquoise	1415.5	1414.76	0.97379	0.005074	5.294
gi|194036374|ref|XM_001929178.1|	turquoise	1550.4	1549.91	0.997641	0.000137	19.842
gi|194036390|ref|XM_001929343.1|	turquoise	154.35	94.3974	0.327866	0.590153	45.53
gi|194036392|ref|XM_001929357.1|	turquoise	1469.2	1468.57	0.985197	0.002157	3.68
gi|194036406|ref|XM_001927918.1|	midnightblue	202.83	34.7267	0.9856	0.00207	18.838
gi|194036414|ref|XM_001927979.1|	turquoise	128.02	103.814	0.390373	0.515891	20.658
gi|194036424|ref|XM_001925710.1|	turquoise	132.79	69.0372	0.269129	0.661517	39.102
gi|194036434|ref|XM_001926977.1|	turquoise	684.81	677.457	0.798743	0.10505	174.104
gi|194036442|ref|XM_001925127.1|	turquoise	1560.4	1560.02	0.998171	9.39E-05	41.262
gi|194036460|ref|XM_001926279.1|	magenta	342.24	41.3432	0.975646	0.004546	27.508
gi|194036467|ref|XM_001928805.1|	turquoise	191.66	114.846	0.317149	0.603069	42.36
gi|194036601|ref|XM_001929330.1|	turquoise	1272.4	1271.62	0.945726	0.015054	11.784

gi|194036603|ref|XM_001929372.1|	turquoise	912.38	908.645	0.872223	0.053766	459.4
gi|194036611|ref|XM_001926603.1|	turquoise	1561.5	1561.16	0.998016	0.000106	28.062
gi|194036619|ref|XM_001927496.1|	turquoise	1423.2	1422.58	0.976076	0.004426	17.404
gi|194036621|ref|XM_001927321.1|	turquoise	824.58	822.333	0.839214	0.0755	70.158
gi|194036623|ref|XM_001927665.1|	turquoise	524.09	518.759	0.733132	0.158701	22.086
gi|194036639|ref|XM_001924618.1|	brown	228	123.01	0.986531	0.001873	128.9
gi|194036658|ref|XM_001926004.1|	turquoise	1449.7	1448.99	0.982297	0.00282	24.204
gi|194036687|ref|XM_001926116.1|	midnightblue	258.65	33.5788	0.977934	0.003922	49.27
gi|194036698|ref|XM_001928463.1|	turquoise	420.08	411.348	0.696158	0.191623	13.646
gi|194036737|ref|XM_001927243.1|	royalblue	258.08	21.0613	0.943513	0.015979	61.28
gi|194036739|ref|XM_001927255.1|	turquoise	1506.7	1506.27	0.987934	0.001588	30.982
gi|194036762|ref|XM_001925928.1|	darkgreen	323.97	21.2657	0.978329	0.003817	22.746
gi|194036768|ref|XM_001927459.1|	turquoise	1537.3	1536.91	0.993063	0.000693	5.244
gi|194036772|ref|XM_001927760.1|	turquoise	1112	1110.35	0.919214	0.027227	21.606
gi|194036795|ref|XM_001926863.1|	yellow	310.35	63.6789	0.903876	0.035255	17.26
gi|194036805|ref|XM_001928924.1|	red	232.3	50.1842	0.987955	0.001584	22.378
gi|194036851|ref|XM_001928643.1|	turquoise	1404.2	1403.27	0.975042	0.004715	47.666
gi|194036879|ref|XM_001927666.1|	green	108.61	15.9611	0.673886	0.21228	57.728
gi|194036901|ref|XM_001927323.1|	black	145.76	18.7926	0.775465	0.123329	88.024
gi|194036903|ref|XM_001927877.1|	turquoise	333.74	329.423	0.674387	0.211809	2.126
gi|194036925|ref|XM_001925464.1|	turquoise	1533.5	1533.16	0.993369	0.000648	6.47
gi|194036941|ref|XM_001925986.1|	lightgreen	89.422	9.77882	0.845316	0.071311	15.712
gi|194036956|ref|XM_001924342.1|	turquoise	1476.9	1476.41	0.987183	0.001738	29.376
gi|194036972|ref|XM_001927228.1|	turquoise	1304.4	1303.17	0.956606	0.01078	210.808
gi|194036974|ref|XM_001927747.1|	turquoise	901.24	897.093	0.86782	0.05653	438.702
gi|194036992|ref|XM_001928798.1|	tan	272.34	37.3475	0.97725	0.004105	17.398
gi|194037004|ref|XM_001928953.1|	turquoise	1553.3	1552.86	0.997031	0.000194	15.404
gi|194037019|ref|XM_001926939.1|	lightcyan	152.07	6.9697	0.174481	0.778977	14.772
gi|194037021|ref|XM_001926975.1|	turquoise	1560.1	1559.69	0.998916	4.29E-05	2.618
gi|194037031|ref|XM_001925224.1|	turquoise	337.54	328.649	0.625619	0.258982	3.954
gi|194037039|ref|XM_001926976.1|	turquoise	1372.2	1371.44	0.966785	0.00723	18.666
gi|194037065|ref|XM_001927928.1|	brown	204.79	100.201	0.943371	0.016038	26.412
gi|194037139|ref|XM_001925875.1|	yellow	131.71	58.5827	0.897157	0.038974	55.446
gi|194037164|ref|XM_001928669.1|	turquoise	872.46	867.679	0.858715	0.062381	1.148
gi|194037172|ref|XM_001928663.1|	grey60	290.16	27.5748	0.95702	0.010627	68.298
gi|194037185|ref|XM_001929112.1|	purple	163.93	26.1381	0.915152	0.029288	23.798
gi|194037272|ref|XM_001928278.1|	turquoise	978.58	976.133	0.894194	0.040652	20.91
gi|194037329|ref|XM_001927218.1|	blue	209.51	73.9714	0.830171	0.081841	2.41
gi|194037339|ref|XM_001924386.1|	turquoise	1512.3	1511.93	0.98989	0.001218	4.304
gi|194037346|ref|XM_001924466.1|	lightcyan	281.13	26.1543	0.97996	0.003395	35.654
gi|194037354|ref|XM_001926198.1|	brown	280.46	102.4	0.930092	0.021954	42.51
gi|194037369|ref|XM_001925128.1|	turquoise	1374.1	1373.22	0.970491	0.006058	28.926
gi|194037372|ref|XM_001927549.1|	darkred	75.722	9.17114	0.804243	0.100862	63.18
gi|194037379|ref|XM_001925252.1|	turquoise	1431	1430.21	0.980043	0.003374	58.612
gi|194037499|ref|XM_001928879.1|	yellow	218.12	78.9129	0.979973	0.003392	47.322
gi|194037532|ref|XM_001929272.1|	red	210.71	49.7557	0.986543	0.00187	19.288
gi|194037543|ref|XM_001929293.1|	turquoise	340.67	331.757	0.650252	0.234831	66.758
gi|194037553|ref|XM_001929410.1|	navy	279.37	30.6783	0.970964	0.005913	518.492

gi|194037555|ref|XM_001929413.1|	blue	235.99	94.1658	0.882019	0.047776	63.236
gi|194037603|ref|XM_001927821.1|	turquoise	1453	1452.44	0.981624	0.002982	61.668
gi|194037611|ref|XM_001925424.1|	tan	285.18	37.8254	0.982123	0.002862	31.746
gi|194037617|ref|XM_001927127.1|	turquoise	1503.6	1503.21	0.987967	0.001582	35.976
gi|194037661|ref|XM_001928816.1|	turquoise	1453.8	1453.32	0.978816	0.003689	2.236
gi|194037682|ref|XM_001927000.1|	turquoise	1114.7	1112.4	0.919573	0.027048	79.74
gi|194037684|ref|XM_001927128.1|	darkturquoise	177.15	15.5682	0.964017	0.008149	83.722
gi|194037687|ref|XM_001927351.1|	turquoise	1552.3	1551.92	0.99639	0.00026	20.116
gi|194037705|ref|XM_001924362.1|	purple	171.03	27.9094	0.93617	0.019172	102.164
gi|194037707|ref|XM_001924251.1|	salmon	143.02	26.0806	0.948957	0.013737	11.932
gi|194037715|ref|XM_001927280.1|	turquoise	320.4	291.567	0.578977	0.306378	34.562
gi|194037750|ref|XM_001926886.1|	turquoise	1542.6	1542.18	0.994826	0.000446	2.518
gi|194037755|ref|XM_001925059.1|	brown	160.03	87.0876	0.904708	0.034803	13.32
gi|194037785|ref|XM_001924937.1|	turquoise	102.9	58.6365	0.228991	0.711008	3.172
gi|194037857|ref|XM_001927835.1|	turquoise	1560.7	1560.23	0.998822	4.85E-05	13.228
gi|194037865|ref|XM_001928013.1|	turquoise	1442.9	1442.13	0.982227	0.002837	88.838
gi|194037874|ref|XM_001925758.1|	turquoise	1453.7	1453.04	0.982626	0.002742	5.614
gi|194037879|ref|XM_001925784.1|	turquoise	1253.3	1252.28	0.948205	0.01404	22.676
gi|194037909|ref|XM_001924188.1|	turquoise	1447.1	1446.4	0.980947	0.003148	3.072
gi|194037914|ref|XM_001927045.1|	turquoise	1553.6	1553.18	0.996528	0.000245	0.756
gi|194037944|ref|XM_001924408.1|	turquoise	822.52	818.829	0.84809	0.069432	18.472
gi|194037952|ref|XM_001924515.1|	black	274.25	40.8838	0.992296	0.000811	136.058
gi|194038017|ref|XM_001928309.1|	tan	224.06	28.2182	0.915916	0.028897	17.818
gi|194038023|ref|XM_001928502.1|	turquoise	797.98	791.981	0.835975	0.077753	35.3
gi|194038031|ref|XM_001928655.1|	turquoise	1482.6	1482.1	0.987633	0.001648	8.358
gi|194038037|ref|XM_001929120.1|	green	279.64	42.8609	0.917222	0.028232	33.068
gi|194038066|ref|XM_001927909.1|	turquoise	1354.8	1353.98	0.966406	0.007354	266.846
gi|194038088|ref|XM_001925288.1|	turquoise	1454.9	1454.26	0.983455	0.002548	37.936
gi|194038212|ref|XM_001927641.1|	turquoise	1481	1480.63	0.983526	0.002532	11.734
gi|194038223|ref|XM_001926312.1|	turquoise	1239.1	1237.78	0.9448	0.015439	3.568
gi|194038225|ref|XM_001926546.1|	turquoise	161.08	134.98	0.424117	0.476653	55.254
gi|194038241|ref|XM_001927985.1|	turquoise	336.19	321.41	0.641716	0.243127	42.514
gi|194038245|ref|XM_001928187.1|	turquoise	1517.2	1516.82	0.990742	0.001068	90.006
gi|194038277|ref|XM_001926890.1|	turquoise	1390.1	1389.2	0.972101	0.005571	40.91
gi|194038313|ref|XM_001925341.1|	midnightblue	226.63	36.6195	0.996692	0.000228	12.226
gi|194038369|ref|XM_001929075.1|	turquoise	1408.8	1407.92	0.975025	0.00472	98.692
gi|194038424|ref|XM_001926793.1|	turquoise	465.88	457.532	0.721143	0.16918	46.082
gi|194038454|ref|XM_001928679.1|	turquoise	1413.7	1412.98	0.976989	0.004176	7.408
gi|194038456|ref|XM_001928767.1|	turquoise	672.54	666.136	0.79893	0.104906	26.262
gi|194038464|ref|XM_001924308.1|	pink	284.23	38.8364	0.944387	0.015611	788.818
gi|194038478|ref|XM_001926160.1|	darkturquoise	198.48	17.1627	0.974723	0.004806	26.24
gi|194038523|ref|XM_001928961.1|	turquoise	1069.9	1068.3	0.916463	0.028617	8.702
gi|194038579|ref|XM_001926122.1|	salmon	247.03	27.4062	0.952078	0.012502	70.04
gi|194038585|ref|XM_001926711.1|	turquoise	1474.5	1474.02	0.982406	0.002794	329.424
gi|194038687|ref|XM_001928174.1|	turquoise	1415.2	1414.42	0.977045	0.00416	42.76
gi|194038695|ref|XM_001928512.1|	turquoise	1452.9	1452.31	0.983122	0.002625	27.482
gi|194038708|ref|XM_001928665.1|	turquoise	1468.8	1468.45	0.983346	0.002573	162.876
gi|194038714|ref|XM_001926092.1|	turquoise	1376.3	1375.72	0.965735	0.007575	42.136

gi|194038719|ref|XM_001926310.1|	turquoise	1379.3	1378.39	0.969774	0.006279	17.04
gi|194038723|ref|XM_001929103.1|	yellow	162.61	58.3695	0.887051	0.044788	23.262
gi|194038735|ref|XM_001929188.1|	turquoise	394.78	375.907	0.656354	0.228949	41.952
gi|194038777|ref|XM_001928268.1|	turquoise	1252.1	1250.69	0.946942	0.014554	40.234
gi|194038781|ref|XM_001926473.1|	turquoise	1313.7	1312.53	0.957275	0.010533	103.76
gi|194038818|ref|XM_001924413.1|	turquoise	142.42	120.168	0.407708	0.495653	11.748
gi|194038838|ref|XM_001927354.1|	turquoise	1483.3	1482.67	0.988149	0.001546	22.394
gi|194038854|ref|XM_001928014.1|	salmon	136.78	23.7297	0.937297	0.01867	88.614
gi|194038889|ref|XM_001928918.1|	turquoise	1461.6	1460.85	0.9841	0.002401	18.144
gi|194038900|ref|XM_001925133.1|	blue	345.98	149.203	0.985794	0.002028	66.896
gi|194038904|ref|XM_001924445.1|	turquoise	150.36	123.342	0.400677	0.503841	181.856
gi|194038910|ref|XM_001926121.1|	blue	331.81	145.339	0.979939	0.003401	22.85
gi|194039226|ref|XM_001928899.1|	pink	265.18	45.3112	0.982642	0.002738	270.856
gi|194039262|ref|XM_001927034.1|	darkturquoise	181.96	7.73013	0.793601	0.109011	2.71
gi|194039264|ref|XM_001927148.1|	green	217.56	48.4686	0.949582	0.013487	17.42
gi|194039314|ref|XM_001926691.1|	turquoise	1465.8	1465.29	0.984622	0.002284	11.208
gi|194039316|ref|XM_001929265.1|	grey	62.985	9.48272	-0.0116	0.985229	76.966
gi|194039388|ref|XM_001929568.1|	turquoise	442.99	439.877	0.724945	0.165836	33.518
gi|194039390|ref|XM_001929570.1|	purple	185.48	32.5238	0.97398	0.005019	646.798
gi|194039486|ref|XM_001927002.1|	turquoise	281.8	245.238	0.53302	0.355005	58.212
gi|194039490|ref|XM_001927338.1|	red	223.67	48.452	0.980237	0.003325	65.578
gi|194039500|ref|XM_001925226.1|	turquoise	1156.5	1154.73	0.930717	0.021662	22.902
gi|194039546|ref|XM_001928863.1|	turquoise	1016.8	1015.21	0.906623	0.033769	74.624
gi|194039563|ref|XM_001929079.1|	turquoise	692.7	684.854	0.800188	0.103945	12.566
gi|194039565|ref|XM_001929124.1|	turquoise	1263.5	1262.32	0.949238	0.013624	32.994
gi|194039587|ref|XM_001929390.1|	lightyellow	308.74	26.2161	0.999961	2.94E-07	57.702
gi|194039625|ref|XM_001925622.1|	turquoise	1530.7	1530.27	0.994404	0.000502	9.53
gi|194039679|ref|XM_001928732.1|	turquoise	948.93	947.439	0.882218	0.047657	65.554
gi|194039687|ref|XM_001928955.1|	turquoise	1310.5	1309.38	0.960769	0.009273	107.684
gi|194039697|ref|XM_001929172.1|	turquoise	1072.8	1070.85	0.913374	0.030205	98.12
gi|194039709|ref|XM_001929236.1|	lightyellow	286.12	24.2829	0.970525	0.006048	63.436
gi|194039715|ref|XM_001925448.1|	turquoise	1249.2	1248.66	0.949775	0.01341	54.768
gi|194039719|ref|XM_001927905.1|	turquoise	1513.8	1513.27	0.989473	0.001295	1.086
gi|194039733|ref|XM_001928398.1|	turquoise	1549.3	1548.86	0.995759	0.000331	9.8
gi|194039735|ref|XM_001928389.1|	pink	243.83	44.1166	0.980234	0.003326	403.634
gi|194040165|ref|XM_001929121.1|	turquoise	1553.6	1553.18	0.996528	0.000245	0.566
gi|194040173|ref|XM_001926225.1|	darkred	53.323	11.4122	0.90153	0.03654	92.802
gi|194040238|ref|XM_001929612.1|	turquoise	248.85	230.737	0.55148	0.335257	20.88
gi|194040263|ref|XM_001927880.1|	yellow	153.69	64.9252	0.930365	0.021827	21.814
gi|194040280|ref|XM_001925339.1|	lightgreen	110.49	7.86981	0.49707	0.394237	317.058
gi|194040290|ref|XM_001927693.1|	turquoise	195.33	185.586	0.554276	0.33229	34.68
gi|194040321|ref|XM_001928400.1|	turquoise	501.15	494.909	0.743263	0.15	39.014
gi|194040341|ref|XM_001928820.1|	turquoise	1433.9	1433.15	0.978465	0.003781	25.034
gi|194040347|ref|XM_001926869.1|	turquoise	1553.6	1553.18	0.996528	0.000245	2.362
gi|194040411|ref|XM_001929558.1|	yellow	228.6	69.0828	0.943761	0.015874	253.72
gi|194040422|ref|XM_001929580.1|	turquoise	1276.3	1274.99	0.951792	0.012614	168.67
gi|194040485|ref|XM_001926913.1|	blue	345.11	146.688	0.98223	0.002836	81.41
gi|194040487|ref|XM_001926988.1|	blue	267.85	125.894	0.947974	0.014134	25.006

gi|194040489|ref|XM_001927223.1|	turquoise	124.83	99.0196	0.373015	0.536319	62.712
gi|194040496|ref|XM_001928226.1|	royalblue	254.17	21.7245	0.952157	0.012472	31.392
gi|194040558|ref|XM_001928742.1|	turquoise	155.51	137.738	0.463475	0.431751	1849.176
gi|194040597|ref|XM_001928150.1|	turquoise	180.6	156.44	0.455162	0.441153	29.566
gi|194040616|ref|XM_001929023.1|	red	131.7	34.5756	0.903406	0.035511	56.558
gi|194040672|ref|XM_001925213.1|	turquoise	1507.5	1507.11	0.989209	0.001343	1.554
gi|194040677|ref|XM_001927466.1|	turquoise	1546	1545.53	0.996281	0.000272	68.772
gi|194040689|ref|XM_001927163.1|	turquoise	1353.5	1352.99	0.962729	0.008589	6.104
gi|194040706|ref|XM_001927201.1|	magenta	346.29	42.0484	0.981411	0.003034	320.558
gi|194040741|ref|XM_001926132.1|	turquoise	1293.3	1292.03	0.956097	0.01097	22.546
gi|194040755|ref|XM_001927097.1|	navy	160.77	14.7628	0.818286	0.090409	157.49
gi|194040778|ref|XM_001927213.1|	turquoise	1294.5	1293.83	0.953462	0.011967	23.146
gi|194040784|ref|XM_001927731.1|	turquoise	1384.3	1383.63	0.970317	0.006112	17.252
gi|194040788|ref|XM_001925300.1|	turquoise	1499.8	1499.31	0.98737	0.001701	30.228
gi|194040884|ref|XM_001924115.1|	turquoise	1350.4	1349.86	0.960958	0.009206	2.704
gi|194040931|ref|XM_001926443.1|	turquoise	1552.9	1552.51	0.996426	0.000256	20.412
gi|194040939|ref|XM_001924716.1|	turquoise	1069.2	1068.01	0.903272	0.035584	43.148
gi|194040961|ref|XM_001924920.1|	turquoise	1244.4	1243.08	0.948534	0.013907	26.612
gi|194040969|ref|XM_001924529.1|	turquoise	1216.3	1215.34	0.933446	0.020404	39.874
gi|194040977|ref|XM_001927185.1|	brown	118.06	56.8007	0.797428	0.106059	22.626
gi|194040979|ref|XM_001928486.1|	black	286.16	42.7344	0.99713	0.000184	209.536
gi|194040991|ref|XM_001924397.1|	lightgreen	60.994	7.71751	0.82884	0.082787	7.568
gi|194041001|ref|XM_001924658.1|	brown	134.72	65.4409	0.845385	0.071265	6.894
gi|194041007|ref|XM_001926441.1|	yellow	266.88	63.3477	0.902757	0.035866	96.424
gi|194041032|ref|XM_001925741.1|	turquoise	364.4	347.437	0.643294	0.241588	59.64
gi|194041034|ref|XM_001928236.1|	turquoise	1210.9	1210.19	0.938947	0.017942	9.588
gi|194041036|ref|XM_001925819.1|	turquoise	1262.8	1262.22	0.945536	0.015133	59.44
gi|194041065|ref|XM_001928989.1|	turquoise	1384.3	1383.4	0.971374	0.005789	45.538
gi|194041087|ref|XM_001927225.1|	greenyellow	213.19	22.3512	0.991227	0.000985	36.108
gi|194041134|ref|XM_001928893.1|	turquoise	1493.8	1493.3	0.987318	0.001711	4.07
gi|194041160|ref|XM_001927391.1|	turquoise	1563.1	1562.75	0.998713	5.54E-05	6.584
gi|194041162|ref|XM_001927505.1|	turquoise	1506	1505.4	0.99139	0.000958	28.962
gi|194041182|ref|XM_001924270.1|	turquoise	1516.8	1516.41	0.990409	0.001126	7.634
gi|194041219|ref|XM_001928126.1|	turquoise	1071.9	1070.38	0.906052	0.034076	11.922
gi|194041222|ref|XM_001925236.1|	turquoise	1524.6	1524.04	0.994282	0.000519	5.032
gi|194041230|ref|XM_001925354.1|	turquoise	1311.8	1310.65	0.960031	0.009535	54.018
gi|194041234|ref|XM_001925415.1|	turquoise	1267.6	1266.95	0.953031	0.012133	21.048
gi|194041270|ref|XM_001926989.1|	turquoise	1491.8	1491.27	0.987335	0.001708	11.7
gi|194041303|ref|XM_001927800.1|	turquoise	449.51	433.081	0.683567	0.203228	39.422
gi|194041305|ref|XM_001927960.1|	pink	218.95	46.5206	0.992287	0.000812	38.65
gi|194041319|ref|XM_001928397.1|	turquoise	942.15	939.542	0.885414	0.045753	25.722
gi|194041329|ref|XM_001928735.1|	turquoise	1427.4	1426.84	0.974314	0.004923	3.3
gi|194041357|ref|XM_001926917.1|	midnightblue	193.89	33.7288	0.979207	0.003588	108.794
gi|194041366|ref|XM_001924481.1|	turquoise	1481.5	1480.87	0.987394	0.001696	40.798
gi|194041397|ref|XM_001928870.1|	turquoise	216.98	162.331	0.408774	0.494415	16.516
gi|194041401|ref|XM_001926294.1|	turquoise	1565	1564.6	0.998931	4.20E-05	3.062
gi|194041408|ref|XM_001929007.1|	turquoise	1530.6	1530.09	0.994774	0.000453	5.338
gi|194041423|ref|XM_001926755.1|	turquoise	1268	1266.79	0.947799	0.014204	48.486

gi|194041507|ref|XM_001924700.1|	turquoise	1560.1	1559.76	0.997921	0.000114	20.418
gi|194041520|ref|XM_001927592.1|	turquoise	1542	1541.53	0.996417	0.000257	84.21
gi|194041588|ref|XM_001927189.1|	turquoise	1514.3	1513.94	0.989873	0.001221	10.972
gi|194041609|ref|XM_001928028.1|	turquoise	1527.7	1527.21	0.994278	0.000519	56.202
gi|194041636|ref|XM_001929009.1|	turquoise	1186.2	1184.69	0.932136	0.021005	45.61
gi|194041638|ref|XM_001929068.1|	salmon	100.93	22.7425	0.917231	0.028227	49.698
gi|194041706|ref|XM_001926955.1|	turquoise	1553.6	1553.18	0.996528	0.000245	0.52
gi|194041741|ref|XM_001928824.1|	turquoise	606.17	600.04	0.784295	0.116292	6.236
gi|194041747|ref|XM_001926631.1|	green	154.63	33.3971	0.84534	0.071295	76.186
gi|194041758|ref|XM_001924615.1|	blue	278.43	115.089	0.927973	0.022952	24.776
gi|194041762|ref|XM_001927529.1|	turquoise	1366.7	1365.93	0.968856	0.006567	65.642
gi|194041780|ref|XM_001925192.1|	turquoise	1482.9	1482.34	0.986482	0.001883	101.728
gi|194041796|ref|XM_001928803.1|	turquoise	1333.8	1333.21	0.961053	0.009172	21.236
gi|194041867|ref|XM_001925382.1|	blue	322.59	130.106	0.9556	0.011156	23.184
gi|194041876|ref|XM_001926725.1|	turquoise	114.17	78.1122	0.293885	0.631272	10.51
gi|194041914|ref|XM_001928393.1|	tan	241.25	30.7119	0.933204	0.020514	72.36
gi|194041930|ref|XM_001925953.1|	turquoise	1559.3	1558.91	0.997321	0.000166	2.112
gi|194041932|ref|XM_001928812.1|	turquoise	912.01	909.036	0.873349	0.053066	53.46
gi|194041956|ref|XM_001929320.1|	turquoise	1553.6	1553.18	0.996528	0.000245	3.496
gi|194042013|ref|XM_001925277.1|	turquoise	907.95	906.236	0.866723	0.057225	20.594
gi|194042016|ref|XM_001926579.1|	turquoise	1557.6	1557.23	0.997234	0.000175	122.456
gi|194042125|ref|XM_001928342.1|	turquoise	1535.8	1535.4	0.993658	0.000606	258.016
gi|194042129|ref|XM_001927395.1|	greenyellow	181.74	20.4857	0.947455	0.014344	34.05
gi|194042141|ref|XM_001929000.1|	midnightblue	253.61	32.8067	0.973433	0.005177	51.128
gi|194042143|ref|XM_001927456.1|	turquoise	1028.8	1026.88	0.90203	0.036265	42.648
gi|194042161|ref|XM_001925705.1|	turquoise	1568.6	1568.26	0.999108	3.20E-05	18.06
gi|194042188|ref|XM_001924178.1|	turquoise	81.6	34.6889	0.129247	0.835897	47.832
gi|194042190|ref|XM_001924717.1|	turquoise	181.74	98.9852	0.25877	0.674239	86.078
gi|194042230|ref|XM_001927832.1|	red	141.74	36.3026	0.91508	0.029325	13.25
gi|194042287|ref|XM_001927955.1|	greenyellow	79.93	5.15803	-0.18772	0.762395	232.368
gi|194042295|ref|XM_001928522.1|	turquoise	1402.8	1402.01	0.974144	0.004972	65.996
gi|194042303|ref|XM_001928772.1|	blue	339.68	141.633	0.973858	0.005054	126.114
gi|194042305|ref|XM_001928810.1|	magenta	328.07	42.9695	0.984629	0.002282	103.902
gi|194042350|ref|XM_001924660.1|	blue	284.51	134.495	0.962519	0.008661	53.804
gi|194042373|ref|XM_001925458.1|	brown	145.01	71.9868	0.84294	0.072933	11.906
gi|194042396|ref|XM_001927868.1|	turquoise	1565.5	1565.12	0.998663	5.87E-05	17.252
gi|194042436|ref|XM_001925357.1|	turquoise	1533.5	1533.13	0.993958	0.000563	7.514
gi|194042438|ref|XM_001927977.1|	turquoise	95.118	52.8332	0.216934	0.725973	34.07
gi|194042440|ref|XM_001928136.1|	turquoise	1435.3	1434.63	0.979752	0.003448	49.604
gi|194042477|ref|XM_001926521.1|	turquoise	933.22	931.503	0.871276	0.054357	22.492
gi|194042479|ref|XM_001926556.1|	turquoise	1539.3	1538.85	0.995312	0.000385	9.582
gi|194042482|ref|XM_001925217.1|	turquoise	1507.2	1506.84	0.990153	0.001171	21.93
gi|194042486|ref|XM_001927572.1|	turquoise	1512.3	1511.72	0.99205	0.00085	12.586
gi|194042528|ref|XM_001927272.1|	pink	248.99	45.2601	0.984763	0.002253	652.286
gi|194042562|ref|XM_001925435.1|	brown	288.85	117.861	0.97008	0.006185	38.09
gi|194042587|ref|XM_001928493.1|	turquoise	1236.9	1235.76	0.946855	0.014589	26.936
gi|194042611|ref|XM_001927098.1|	turquoise	1502.6	1502.1	0.989108	0.001362	11.95
gi|194042690|ref|XM_001929322.1|	pink	200.03	43.5404	0.97872	0.003714	72.954

gi|194042725|ref|XM_001925659.1|	turquoise	1450	1449.37	0.982954	0.002665	13.504
gi|194042745|ref|XM_001927026.1|	purple	231.25	32.7622	0.9762	0.004392	8.374
gi|194042749|ref|XM_001925080.1|	turquoise	160.23	133.856	0.42118	0.480043	93.262
gi|194042787|ref|XM_001924736.1|	blue	283.92	140.777	0.972639	0.00541	81.024
gi|194042805|ref|XM_001927802.1|	purple	173.05	26.4419	0.911783	0.031033	238.778
gi|194042819|ref|XM_001928170.1|	darkred	102.79	10.8149	0.840121	0.074872	32.502
gi|194042829|ref|XM_001928949.1|	turquoise	1567	1566.56	0.999533	1.21E-05	24.544
gi|194042833|ref|XM_001929033.1|	yellow	193.76	52.2778	0.860533	0.061199	18.642
gi|194042865|ref|XM_001925507.1|	turquoise	1111.6	1110.44	0.91181	0.03102	21.548
gi|194042943|ref|XM_001929420.1|	turquoise	401.12	392.547	0.694442	0.193193	39.98
gi|194042969|ref|XM_001929585.1|	green	226.51	49.9531	0.973458	0.00517	40.122
gi|194043006|ref|XM_001928075.1|	turquoise	1297	1296.02	0.957995	0.010269	25.432
gi|194043042|ref|XM_001926896.1|	brown	319.16	92.6954	0.903478	0.035472	70.876
gi|194043063|ref|XM_001925272.1|	turquoise	1391.6	1390.8	0.974565	0.004851	25.352
gi|194043070|ref|XM_001928504.1|	tan	308.62	36.3423	0.970885	0.005937	13.16
gi|194043074|ref|XM_001928487.1|	turquoise	1446.6	1445.88	0.980737	0.0032	97.438
gi|194043076|ref|XM_001925356.1|	turquoise	1377.3	1376.79	0.971065	0.005883	35.736
gi|194043124|ref|XM_001926685.1|	turquoise	1488	1487.43	0.987477	0.001679	57.768
gi|194043177|ref|XM_001928608.1|	turquoise	1525.8	1525.44	0.991062	0.001013	4.906
gi|194043187|ref|XM_001925978.1|	blue	274.99	127.64	0.951321	0.012798	52.774
gi|194043189|ref|XM_001928908.1|	turquoise	877.25	876.09	0.862999	0.059606	16.408
gi|194043243|ref|XM_001924074.1|	blue	339.96	155.877	0.996047	0.000298	88.756
gi|194043267|ref|XM_001926534.1|	turquoise	204.79	191.749	0.516353	0.37307	3.398
gi|194043281|ref|XM_001927737.1|	turquoise	1521.9	1521.58	0.991819	0.000887	8.82
gi|194043291|ref|XM_001928076.1|	turquoise	830.24	827.546	0.850109	0.068074	101.718
gi|194043329|ref|XM_001929273.1|	black	270.48	39.3069	0.982611	0.002746	80.728
gi|194043337|ref|XM_001926296.1|	darkgreen	289.96	23.8223	0.997855	0.000119	18.516
gi|194043363|ref|XM_001929468.1|	green	262.54	50.4352	0.970091	0.006181	31.734
gi|194043388|ref|XM_001929537.1|	darkred	74.05	12.2399	0.942522	0.016399	36.634
gi|194043395|ref|XM_001929562.1|	turquoise	1550.9	1550.42	0.997344	0.000164	7.046
gi|194043399|ref|XM_001929569.1|	turquoise	1569	1568.64	0.999459	1.51E-05	9.186
gi|194043439|ref|XM_001927481.1|	turquoise	1505.1	1504.56	0.989318	0.001323	19.184
gi|194043488|ref|XM_001929645.1|	turquoise	1337	1336.45	0.963736	0.008245	8.978
gi|194043591|ref|XM_001927268.1|	pink	115.66	24.4241	0.850017	0.068136	75.99
gi|194043626|ref|XM_001926661.1|	turquoise	1548.6	1548.19	0.996179	0.000283	22.294
gi|194043630|ref|XM_001926758.1|	turquoise	1552.4	1551.98	0.996026	0.000301	1.784
gi|194043649|ref|XM_001925542.1|	turquoise	464.39	458.91	0.729398	0.161945	4.672
gi|194043669|ref|XM_001924741.1|	turquoise	108.8	71.4424	0.311851	0.609471	19.492
gi|194043731|ref|XM_001926974.1|	turquoise	122.05	107.077	0.407018	0.496456	3.596
gi|194043780|ref|XM_001928673.1|	turquoise	1541.1	1540.61	0.996444	0.000254	5.398
gi|194043830|ref|XM_001925246.1|	turquoise	921.01	919.142	0.86551	0.057997	6.942
gi|194043856|ref|XM_001928206.1|	turquoise	1428.1	1427.42	0.979257	0.003575	40.074
gi|194043858|ref|XM_001928335.1|	blue	310.23	131.438	0.956902	0.010671	32.064
gi|194043865|ref|XM_001928489.1|	turquoise	1553.6	1553.18	0.996528	0.000245	0.236
gi|194043908|ref|XM_001927890.1|	turquoise	1285	1283.63	0.95357	0.011926	63.282
gi|194043920|ref|XM_001925866.1|	navy	234.24	32.1134	0.982176	0.002849	9.594
gi|194043935|ref|XM_001924322.1|	turquoise	1340.3	1339.43	0.964176	0.008095	19.902
gi|194043947|ref|XM_001925017.1|	turquoise	749.53	745.683	0.826665	0.084341	24.918

gi|194043951|ref|XM_001927705.1|	turquoise	185.2	118.725	0.317258	0.602937	24.388
gi|194043957|ref|XM_001925803.1|	grey60	249.78	33.1018	0.997693	0.000133	1150.344
gi|194043959|ref|XM_001927448.1|	turquoise	1420.3	1419.53	0.977313	0.004088	44.33
gi|194043997|ref|XM_001926349.1|	turquoise	1153.1	1151.5	0.929966	0.022013	56.05
gi|194044032|ref|XM_001928774.1|	royalblue	205.09	19.956	0.936493	0.019028	37.174
gi|194044034|ref|XM_001928794.1|	turquoise	1547	1546.48	0.997222	0.000176	423.108
gi|194044044|ref|XM_001927332.1|	blue	270.56	122.709	0.941434	0.016864	11.314
gi|194044061|ref|XM_001927723.1|	turquoise	906.77	903.69	0.87542	0.051787	7.962
gi|194044069|ref|XM_001925245.1|	turquoise	132.66	103.803	0.362437	0.548843	15.082
gi|194044114|ref|XM_001924459.1|	turquoise	60.468	40.5908	0.216182	0.726908	95.016
gi|194044123|ref|XM_001926788.1|	lightyellow	304	24.7823	0.98724	0.001727	78.056
gi|194044132|ref|XM_001927798.1|	turquoise	1480.9	1480.37	0.987064	0.001763	75.62
gi|194044165|ref|XM_001924403.1|	turquoise	1530.3	1529.81	0.994908	0.000436	2.32
gi|194044169|ref|XM_001924460.1|	turquoise	1172.6	1171.03	0.930718	0.021662	74.538
gi|194044171|ref|XM_001924505.1|	brown	214.67	115.071	0.9696	0.006334	11.59
gi|194044267|ref|XM_001925000.1|	turquoise	1537.8	1537.43	0.993245	0.000666	3.968
gi|194044285|ref|XM_001926002.1|	turquoise	1103	1100.79	0.917143	0.028272	18.902
gi|194044289|ref|XM_001926487.1|	turquoise	1535.6	1535.14	0.995822	0.000324	72.626
gi|194044465|ref|XM_001928874.1|	midnightblue	236.14	34.8704	0.986448	0.00189	12.122
gi|194044483|ref|XM_001929150.1|	turquoise	894.59	891.21	0.872267	0.053739	104.984
gi|194044495|ref|XM_001929295.1|	turquoise	409.55	392.255	0.666646	0.219122	5.54
gi|194044506|ref|XM_001927374.1|	purple	201.8	34.4407	0.982363	0.002804	34.238
gi|194044550|ref|XM_001927682.1|	turquoise	1095	1092.53	0.914787	0.029475	59.878
gi|194044564|ref|XM_001925248.1|	turquoise	1548.3	1547.93	0.995962	0.000308	21.818
gi|194044568|ref|XM_001928462.1|	turquoise	1456.6	1455.85	0.984119	0.002397	32.936
gi|194044570|ref|XM_001928614.1|	turquoise	1528.9	1528.46	0.992554	0.00077	16.428
gi|194044572|ref|XM_001925488.1|	lightcyan	150.31	6.99391	0.287706	0.6388	25.408
gi|194044574|ref|XM_001928737.1|	turquoise	1553.6	1553.18	0.996528	0.000245	2.598
gi|194044593|ref|XM_001929063.1|	red	144.09	39.1359	0.93091	0.021573	35.88
gi|194044682|ref|XM_001928813.1|	turquoise	133.86	66.0464	0.249981	0.685061	24.616
gi|194044732|ref|XM_001927204.1|	turquoise	1434.9	1434.38	0.976706	0.004253	38.918
gi|194044782|ref|XM_001926654.1|	navy	249.62	32.9757	0.989022	0.001379	13.396
gi|194044800|ref|XM_001924191.1|	turquoise	64.889	51.5237	0.27825	0.650348	40.11
gi|194044879|ref|XM_001925452.1|	red	233	48.2948	0.978762	0.003704	8.698
gi|194044905|ref|XM_001926512.1|	turquoise	1532.8	1532.46	0.992514	0.000777	3.294
gi|194044921|ref|XM_001927440.1|	turquoise	1566.3	1565.91	0.999265	2.39E-05	24.44
gi|194044923|ref|XM_001927589.1|	turquoise	1511.3	1510.79	0.991692	0.000908	11.198
gi|194044925|ref|XM_001927734.1|	turquoise	702.98	696.932	0.809402	0.096982	85.18
gi|194044929|ref|XM_001925991.1|	turquoise	1413.7	1413.09	0.971551	0.005736	7.658
gi|194044943|ref|XM_001926010.1|	pink	210.96	39.05	0.951991	0.012536	62.096
gi|194044951|ref|XM_001925471.1|	turquoise	1055.8	1053.56	0.910465	0.031725	32.374
gi|194044985|ref|XM_001927811.1|	turquoise	1467.2	1466.72	0.983478	0.002543	5.184
gi|194044987|ref|XM_001927848.1|	turquoise	419.83	414.505	0.685855	0.201106	38.478
gi|194045046|ref|XM_001924519.1|	turquoise	291.55	269.848	0.571964	0.313681	20.188
gi|194045056|ref|XM_001924869.1|	turquoise	1553.6	1553.18	0.996528	0.000245	1.748
gi|194045058|ref|XM_001925649.1|	turquoise	1553.6	1553.18	0.996528	0.000245	1.04
gi|194045072|ref|XM_001927451.1|	blue	240.14	108.747	0.915185	0.029271	8.412
gi|194045084|ref|XM_001925136.1|	turquoise	103.23	92.603	0.388081	0.518579	4.694

gi|194045109|ref|XM_001927874.1|	turquoise	1289.4	1288.1	0.95507	0.011355	34.44
gi|194045111|ref|XM_001927898.1|	yellow	271.87	69.0897	0.92774	0.023063	57.822
gi|194271212|gb|EU714326.1|	turquoise	1397.4	1396.9	0.968205	0.006773	10.292
gi|194332486|ref|NM_001130213.1|	darkred	67.897	7.19021	0.700799	0.187394	75.436
gi|194474045|ref|NM_001130534.1|	pink	206.94	45.0401	0.985237	0.002148	24.728
gi|194474047|ref|NM_001130535.1|	turquoise	315.19	305.195	0.637727	0.247031	68.814
gi|1955|emb|X17058.1|	turquoise	1371.4	1370.5	0.969552	0.006349	11.838
gi|195539469|ref|NM_001130733.1|	red	181.99	43.8187	0.959447	0.009743	84.19
gi|196259975|ref|NM_001131045.1|	turquoise	1381.2	1380.37	0.972239	0.005529	87.648
gi|197251933|ref|NM_001134354.1|	turquoise	1555.8	1555.45	0.99718	0.00018	6.124
gi|197717772|gb|EF113595.2|	green	156.89	35.2203	0.879775	0.049129	232.98
gi|198282078|ref|NM_001134823.1|	brown	110.82	58.7603	0.821755	0.08788	950.9
gi|19919837|gb|AF490841.1|	turquoise	139.83	107.047	0.396038	0.509259	128.1
gi|201066355|ref|NM_001134967.1|	turquoise	1558.9	1558.48	0.997572	0.000144	3.982
gi|201066357|ref|NM_001134968.1|	pink	276.61	41.3342	0.961008	0.009188	300.808
gi|2035|emb|X16638.1|	turquoise	1468	1467.28	0.985867	0.002013	35.81
gi|208610189|ref|NM_001135680.1|	turquoise	1435.4	1434.61	0.979507	0.003511	30.11
gi|209863046|ref|NM_001135965.1|	greenyellow	178.42	22.3353	0.989378	0.001312	46.43
gi|209863048|ref|NM_001135966.1|	turquoise	634.66	625.151	0.776882	0.122191	7.194
gi|212549622|ref|NM_001137629.1|	turquoise	1497.6	1497.14	0.986301	0.001921	13.242
gi|212549654|ref|NM_001137635.1|	turquoise	1498.9	1498.42	0.987716	0.001631	5.438
gi|212549656|ref|NM_001137636.1|	turquoise	1515.4	1514.96	0.992052	0.00085	7.34
gi|217416473|ref|NM_001142668.1|	turquoise	264.14	249.955	0.585028	0.300113	17.816
gi|217416475|ref|NM_001142669.1|	red	153.5	37.939	0.92681	0.023506	7.152
gi|218082010|ref|NM_001142666.1|	lightgreen	129.87	7.2068	0.624107	0.260485	30.602
gi|2286009|emb|AJ000786.1|	brown	181.79	107.549	0.95288	0.012191	65.132
gi|29373949|emb|AJ539380.1|	yellow	188.76	52.9523	0.881351	0.048177	73.652
gi|2988384|gb|AF033855.1|AF033855	lightcyan	115.97	5.319	0.314437	0.606345	11.63
gi|34582610|gb|AY368623.1|	turquoise	1164.1	1162.55	0.933574	0.020346	476.262
gi|35384837|gb|AY374470.1|	royalblue	179.14	25.2421	0.986924	0.001791	19.72
gi|37050908|emb|AJ583708.1|	turquoise	1451.4	1450.86	0.978727	0.003713	2.468
gi|38455773|gb|AY349420.1|	turquoise	1472.8	1472.4	0.982636	0.00274	3.566
gi|4186150|emb|AJ236938.1|	greenyellow	114.85	14.066	0.892543	0.041597	40.006
gi|45268992|gb|AY550051.1|	purple	109.72	2.69096	0.351705	0.561607	27.182
gi|46095042|gb|AY574215.1|	turquoise	1458.5	1457.78	0.984235	0.00237	20.65
gi|47522617|ref|NM_213934.1|	turquoise	1329.1	1327.99	0.961939	0.008863	33.782
gi|47522629|ref|NM_213928.1|	turquoise	1493	1492.61	0.98608	0.001967	12.082
gi|47522635|ref|NM_213927.1|	turquoise	138.38	114.75	0.403448	0.500612	14.276
gi|47522641|ref|NM_213922.1|	lightgreen	70.962	15.3653	0.942953	0.016216	37.852
gi|47522647|ref|NM_213921.1|	turquoise	1428	1427.41	0.980483	0.003264	16.674
gi|47522661|ref|NM_213911.1|	turquoise	1566.9	1566.56	0.99889	4.44E-05	275.984
gi|47522679|ref|NM_213904.1|	turquoise	1503	1502.37	0.990028	0.001194	49.03
gi|47522681|ref|NM_213901.1|	brown	185.82	108.779	0.956579	0.01079	4.214
gi|47522685|ref|NM_213898.1|	greenyellow	157.84	20.5448	0.957798	0.010341	14.436
gi|47522691|ref|NM_213897.1|	lightyellow	209.66	16.9606	0.882202	0.047667	66.05
gi|47522703|ref|NM_213891.1|	turquoise	862.17	860.509	0.870552	0.05481	81.136
gi|47522707|ref|NM_213889.1|	turquoise	1482.3	1481.74	0.988421	0.001493	4.166
gi|47522709|ref|NM_213885.1|	turquoise	1543.6	1543.21	0.994159	0.000535	2.694

gi|47522727|ref|NM_213949.1|	turquoise	1336	1335.25	0.961278	0.009094	35.748
gi|47522737|ref|NM_213954.1|	navy	253.27	31.6949	0.977444	0.004053	41.276
gi|47522753|ref|NM_213962.1|	turquoise	1103	1101.93	0.917399	0.028142	51.56
gi|47522761|ref|NM_213967.1|	royalblue	252.66	20.8195	0.942409	0.016447	35.43
gi|47522763|ref|NM_213968.1|	turquoise	1408.9	1408.06	0.974863	0.004766	130.722
gi|47522769|ref|NM_213971.1|	turquoise	1057	1055.74	0.901246	0.036696	11.086
gi|47522773|ref|NM_213973.1|	turquoise	1541.6	1541.21	0.994695	0.000463	13.352
gi|47522779|ref|NM_213977.1|	turquoise	1563.8	1563.35	0.998886	4.46E-05	31.994
gi|47522785|ref|NM_213980.1|	red	155.8	39.9964	0.937392	0.018628	45.766
gi|47522797|ref|NM_213986.1|	red	190.84	48.687	0.982245	0.002832	1.516
gi|47522799|ref|NM_213987.1|	lightyellow	276.67	23.3942	0.959648	0.009671	16.74
gi|47522821|ref|NM_213998.1|	turquoise	1112.8	1111.66	0.911589	0.031135	8.254
gi|47522831|ref|NM_214003.1|	turquoise	591.58	581.415	0.762656	0.133752	142.956
gi|47522845|ref|NM_214010.1|	turquoise	203.82	188.989	0.505756	0.384668	35.24
gi|47522855|ref|NM_214017.1|	turquoise	681.33	678.332	0.807366	0.098507	4.182
gi|47522861|ref|NM_214020.1|	green	192.32	49.2558	0.960798	0.009263	21.024
gi|47522869|ref|NM_214024.1|	turquoise	1163.9	1162.07	0.93318	0.020525	78.79
gi|47522871|ref|NM_214025.1|	turquoise	1175.8	1174.89	0.935684	0.01939	1.788
gi|47522897|ref|NM_214039.1|	magenta	301.88	40.2166	0.971875	0.005638	19.062
gi|47522915|ref|NM_214050.1|	brown	257.5	113.599	0.960583	0.009338	6.366
gi|47522919|ref|NM_214052.1|	yellow	205.49	76.1337	0.970789	0.005967	11.878
gi|47522921|ref|NM_214053.1|	turquoise	1087.7	1085.24	0.913635	0.03007	13.756
gi|47522923|ref|NM_214054.1|	turquoise	1564.2	1563.76	0.998621	6.14E-05	11.564
gi|47522935|ref|NM_214060.1|	grey60	266.89	30.9955	0.983481	0.002542	101.822
gi|47522939|ref|NM_214062.1|	turquoise	1416.6	1416.05	0.972727	0.005384	35.502
gi|47522941|ref|NM_214063.1|	yellow	131.07	43.6762	0.796735	0.106591	93.43
gi|47522943|ref|NM_214064.1|	turquoise	1039.9	1038.57	0.898256	0.038358	2.84
gi|47522957|ref|NM_214072.1|	turquoise	1349.7	1348.89	0.960087	0.009515	45.354
gi|47522983|ref|NM_214086.1|	green	106.7	27.6882	0.838577	0.075941	62.31
gi|47523015|ref|NM_214103.1|	turquoise	339.25	321.036	0.631249	0.253406	200.302
gi|47523037|ref|NM_214115.1|	turquoise	325.02	313.156	0.636457	0.248278	185.094
gi|47523039|ref|NM_214116.1|	royalblue	119.31	16.5973	0.888189	0.044121	112.73
gi|47523067|ref|NM_214132.1|	green	282.48	43.5982	0.923453	0.025129	3.146
gi|47523085|ref|NM_214144.1|	blue	278.41	137.351	0.96689	0.007196	69.4
gi|47523093|ref|NM_213884.1|	turquoise	1563.6	1563.17	0.998671	5.81E-05	73
gi|47523095|ref|NM_213881.1|	lightgreen	107.98	10.7673	0.85285	0.066244	112.302
gi|47523129|ref|NM_213866.1|	turquoise	1553.6	1553.18	0.996528	0.000245	0.33
gi|47523187|ref|NM_213830.1|	pink	119.02	28.8497	0.882242	0.047642	5.528
gi|47523211|ref|NM_213815.1|	turquoise	1549.6	1549.22	0.995969	0.000307	17.088
gi|47523255|ref|NM_213792.1|	yellow	186.64	79.4322	0.982991	0.002656	15.674
gi|47523261|ref|NM_213791.1|	turquoise	94.481	80.9089	0.395366	0.510045	4.146
gi|47523281|ref|NM_213781.1|	turquoise	694.28	689.179	0.810905	0.09586	2.17
gi|47523317|ref|NM_213763.1|	turquoise	1555.4	1555.05	0.997139	0.000184	68.082
gi|47523343|ref|NM_213746.1|	turquoise	1532	1531.56	0.993057	0.000694	3.89
gi|47523353|ref|NM_213741.1|	turquoise	1014.8	1011.63	0.896279	0.039469	6.238
gi|47523403|ref|NM_214157.1|	turquoise	660.9	652.407	0.78887	0.112695	19.156
gi|47523443|ref|NM_214181.1|	brown	206.3	121.483	0.986038	0.001976	27.274
gi|47523445|ref|NM_214183.1|	magenta	293.08	39.7631	0.969694	0.006304	14.876

gi|47523461|ref|NM_214192.1|	lightgreen	77.999	12.7477	0.901706	0.036443	630.138
gi|47523463|ref|NM_214188.1|	yellow	191.3	72.8661	0.957153	0.010578	55.326
gi|47523473|ref|NM_214198.1|	brown	290.68	95.4194	0.910207	0.031861	6.118
gi|47523483|ref|NM_214199.1|	green	251.12	52.7142	0.978045	0.003892	66.146
gi|47523487|ref|NM_214201.1|	turquoise	216.77	181.805	0.470494	0.423849	56.41
gi|47523491|ref|NM_214204.1|	yellow	281.92	75.3799	0.959515	0.009719	572.888
gi|47523501|ref|NM_214213.1|	midnightblue	223.01	36.4466	0.995808	0.000326	9.668
gi|47523533|ref|NM_214229.1|	turquoise	1546.7	1546.32	0.995702	0.000338	3.396
gi|47523537|ref|NM_214231.1|	turquoise	1516.2	1515.71	0.992816	0.00073	1.954
gi|47523543|ref|NM_214230.1|	turquoise	1489.5	1488.9	0.988214	0.001533	92.214
gi|47523553|ref|NM_214240.1|	turquoise	1363.7	1362.8	0.965151	0.007768	37.614
gi|47523559|ref|NM_214239.1|	yellow	169.69	49.1582	0.845233	0.071368	38.99
gi|47523561|ref|NM_214245.1|	turquoise	1392.6	1391.98	0.97141	0.005778	15.99
gi|47523567|ref|NM_214244.1|	salmon	110.66	25.3583	0.940358	0.017328	19.754
gi|47523569|ref|NM_214249.1|	turquoise	734	727.876	0.818108	0.090539	49.86
gi|47523579|ref|NM_214250.1|	turquoise	1214.7	1213.22	0.943039	0.016179	35.598
gi|47523581|ref|NM_214257.1|	greenyellow	112.65	4.29465	0.36925	0.54077	5.72
gi|47523607|ref|NM_214267.1|	turquoise	1521.6	1521.07	0.993079	0.00069	19.348
gi|47523617|ref|NM_214276.1|	turquoise	1292	1291.21	0.952874	0.012193	23.024
gi|47523637|ref|NM_214287.1|	brown	165.88	59.6403	0.816464	0.091745	14.46
gi|47523641|ref|NM_214289.1|	grey	225.77	13.7947	0.855539	0.064464	31.388
gi|47523665|ref|NM_214304.1|	turquoise	236.89	228.584	0.561863	0.324275	32.21
gi|47523669|ref|NM_214306.1|	turquoise	1053.6	1052.29	0.912486	0.030666	41.044
gi|47523673|ref|NM_214308.1|	lightgreen	66.789	16.4358	0.966709	0.007255	97.33
gi|47523675|ref|NM_214305.1|	turquoise	1510.3	1509.87	0.988517	0.001475	3.39
gi|47523679|ref|NM_214307.1|	darkgreen	234.52	22.9587	0.980323	0.003304	134.25
gi|47523681|ref|NM_214312.1|	turquoise	1428.4	1427.97	0.975055	0.004712	17.096
gi|47523683|ref|NM_214309.1|	blue	307.64	151.309	0.989561	0.001278	33.842
gi|47523689|ref|NM_214316.1|	turquoise	188.85	174.837	0.522006	0.366918	17.414
gi|47523691|ref|NM_214313.1|	lightcyan	187.68	7.42701	0.328583	0.589291	19.236
gi|47523699|ref|NM_214317.1|	turquoise	1553.6	1553.18	0.996528	0.000245	7.086
gi|47523715|ref|NM_214327.1|	turquoise	1539.9	1539.43	0.99515	0.000405	12.928
gi|47523717|ref|NM_214328.1|	turquoise	158.56	136.753	0.434561	0.464644	2.266
gi|47523719|ref|NM_214330.1|	turquoise	1350.7	1349.97	0.959817	0.009611	82.924
gi|47523721|ref|NM_214331.1|	turquoise	248.36	233.347	0.57046	0.315253	75.684
gi|47523723|ref|NM_214332.1|	brown	305.03	113.798	0.956495	0.010822	505.572
gi|47523725|ref|NM_214333.1|	turquoise	321.9	316.935	0.657045	0.228286	84.526
gi|47523733|ref|NM_214337.1|	turquoise	1092.7	1091.48	0.915138	0.029295	3.96
gi|47523737|ref|NM_214339.1|	darkturquoise	227.1	7.62832	0.773561	0.124862	44.31
gi|47523743|ref|NM_214342.1|	turquoise	1503.9	1503.24	0.991101	0.001006	29.856
gi|47523769|ref|NM_214356.1|	red	235.39	52.5421	0.997495	0.00015	52.11
gi|47523779|ref|NM_214361.1|	turquoise	541.9	538.347	0.74945	0.144757	79.832
gi|47523785|ref|NM_214364.1|	turquoise	1433.6	1433.1	0.974996	0.004728	10.522
gi|47523789|ref|NM_214366.1|	turquoise	426.71	408.336	0.67008	0.215869	98.04
gi|47523799|ref|NM_214372.1|	turquoise	1549	1548.65	0.995452	0.000368	241.998
gi|47523805|ref|NM_214375.1|	green	145.14	18.2016	0.731072	0.160488	190.166
gi|47523819|ref|NM_214382.1|	turquoise	527.55	523.766	0.742342	0.150785	61.498
gi|47523843|ref|NM_214395.1|	turquoise	1553.6	1553.18	0.996528	0.000245	11.668

gi|47523845|ref|NM_214396.1|	salmon	200.17	31.3796	0.993985	0.000559	114.9
gi|47523847|ref|NM_214397.1|	turquoise	1560.4	1560.02	0.997514	0.000149	22.316
gi|47523867|ref|NM_214407.1|	turquoise	1129.1	1127.64	0.923327	0.025191	113.446
gi|47523869|ref|NM_214408.1|	turquoise	278.76	257.541	0.572768	0.312842	81.484
gi|47523887|ref|NM_214418.1|	yellow	203.21	52.6843	0.863729	0.059137	201.71
gi|47523913|ref|NM_214432.1|	turquoise	1326.7	1325.57	0.962276	0.008745	22.266
gi|47523931|ref|NM_214440.1|	turquoise	1486.5	1485.95	0.987145	0.001746	10.862
gi|48374062|ref|NM_001001535.1|	pink	264.72	45.0411	0.981667	0.002972	2674.704
gi|48374070|ref|NM_001001539.1|	blue	332.87	126.648	0.948172	0.014053	94.876
gi|48675942|ref|NM_001001640.1|	darkturquoise	217.35	16.9088	0.978215	0.003847	9.986
gi|48675952|ref|NM_001001643.1|	purple	184.02	25.1411	0.904275	0.035038	184.924
gi|48675954|ref|NM_001001644.1|	turquoise	1368.6	1367.54	0.968974	0.00653	15.194
gi|48675956|ref|NM_001001645.1|	lightcyan	174.2	7.3623	0.273665	0.655958	76.2
gi|48976064|ref|NM_001001649.2|	green	249.67	41.7936	0.926888	0.023469	255.366
gi|48976128|ref|NM_001001770.1|	brown	119.35	61.3325	0.808906	0.097353	132.582
gi|49274614|ref|NM_001001860.1|	purple	148.93	24.0855	0.886643	0.045028	622.422
gi|50657387|ref|NM_001002824.1|	turquoise	1377.8	1377.3	0.967551	0.006983	4.016
gi|50979292|ref|NM_213742.1|	turquoise	1263.8	1262.42	0.949166	0.013653	1.576
gi|51592101|ref|NM_001004028.1|	turquoise	1465.6	1465.22	0.981955	0.002902	14.478
gi|51592104|ref|NM_001004027.1|	turquoise	1348.6	1348.01	0.959668	0.009664	8.454
gi|51592110|ref|NM_001004031.1|	yellow	143.54	55.8645	0.87917	0.049496	77.256
gi|51592116|ref|NM_001004034.1|	turquoise	1508.4	1507.97	0.988163	0.001543	14.932
gi|51592138|ref|NM_001004045.1|	turquoise	1545.6	1545.1	0.996718	0.000226	72.206
gi|51592144|ref|NM_001004049.1|	brown	231.46	101.037	0.933681	0.020297	5.326
gi|51592146|ref|NM_001004050.1|	turquoise	1545	1544.57	0.995585	0.000352	8.826
gi|52350674|gb|AY609390.1|	turquoise	1561.1	1560.67	0.998402	7.67E-05	13.782
gi|52350683|gb|AY609399.1|	turquoise	1368.9	1368.05	0.967406	0.007029	70.514
gi|52350686|gb|AY609402.1|	midnightblue	282.17	28.2014	0.940592	0.017226	23.018
gi|52350690|gb|AY609406.1|	yellow	95.744	42.0309	0.810356	0.096269	36.172
gi|52350718|gb|AY609433.1|	purple	155.71	25.2205	0.90652	0.033824	18.226
gi|52350726|gb|AY609441.1|	turquoise	817.48	813.837	0.844886	0.071604	17.476
gi|52351064|gb|AY609469.1|	turquoise	599.04	596.695	0.782403	0.117789	7.698
gi|52351065|gb|AY609470.1|	brown	189.46	107.04	0.950835	0.012989	41.19
gi|52351069|gb|AY609474.1|	turquoise	1473.3	1472.67	0.98498	0.002205	15.262
gi|52351075|gb|AY609480.1|	turquoise	1353.7	1352.69	0.965928	0.007511	16.624
gi|52351085|gb|AY609490.1|	turquoise	1503.5	1503.14	0.988803	0.00142	7.414
gi|52351086|gb|AY609491.1|	royalblue	177.22	25.1261	0.985827	0.002021	5.692
gi|52351090|gb|AY609495.1|	turquoise	1401	1400.36	0.973983	0.005018	268.918
gi|52351103|gb|AY609508.1|	turquoise	1319.5	1318.84	0.955693	0.011121	9.956
gi|52351109|gb|AY609514.1|	turquoise	1478.6	1477.93	0.986758	0.001826	35.906
gi|52351111|gb|AY609516.1|	turquoise	1554.1	1553.72	0.996596	0.000238	45.294
gi|52351116|gb|AY609521.1|	turquoise	1540.2	1539.8	0.995571	0.000354	72.904
gi|52351120|gb|AY609525.1|	turquoise	159.69	126.264	0.405901	0.497755	120.326
gi|52351125|gb|AY609530.1|	turquoise	921.62	917.741	0.873049	0.053252	71.804
gi|52351129|gb|AY609534.1|	magenta	304.82	40.8384	0.975371	0.004623	9.2
gi|52351137|gb|AY609542.1|	turquoise	1560.2	1559.76	0.998739	5.37E-05	7.776
gi|52351148|gb|AY609553.1|	turquoise	1472.2	1471.77	0.982975	0.00266	59.81
gi|52351160|gb|AY609565.1|	brown	204.97	111.609	0.961883	0.008882	6.312

gi|52351167|gb|AY609572.1|	turquoise	105.86	42.0347	0.15914	0.798235	91.158
gi|52351174|gb|AY609579.1|	turquoise	1370.3	1369.35	0.969255	0.006441	15.78
gi|52351176|gb|AY609581.1|	green	116.82	33.895	0.880594	0.048634	16.648
gi|52351191|gb|AY609596.1|	grey60	210.55	27.3328	0.957349	0.010506	46.086
gi|52351201|gb|AY609606.1|	royalblue	197.81	25.269	0.988727	0.001434	28.244
gi|52351209|gb|AY609612.1|	turquoise	657.03	653.453	0.784997	0.115737	59.138
gi|52351212|gb|AY609615.1|	turquoise	1507	1506.36	0.990579	0.001096	13.14
gi|52351225|gb|AY609657.1|	blue	303.11	150.683	0.988457	0.001486	25.106
gi|52351234|gb|AY609666.1|	lightyellow	252.73	21.5418	0.934464	0.019941	68.178
gi|52351253|gb|AY609685.1|	turquoise	203.53	155.56	0.403277	0.50081	64.912
gi|52351260|gb|AY609692.1|	turquoise	1457.8	1457.23	0.981674	0.00297	49.872
gi|52351277|gb|AY609709.1|	salmon	107.98	25.1108	0.938204	0.018268	53.06
gi|52351285|gb|AY609717.1|	brown	228.74	120.754	0.98204	0.002881	4.718
gi|52351294|gb|AY609726.1|	blue	326.05	138.815	0.969577	0.006341	10.13
gi|52351299|gb|AY609731.1|	darkgreen	281.14	22.6945	0.986535	0.001872	3.066
gi|52351303|gb|AY609735.1|	turquoise	1391.7	1391.16	0.969075	0.006498	34.372
gi|52351306|gb|AY609738.1|	turquoise	1329.4	1328.37	0.958717	0.010007	57.196
gi|52351323|gb|AY609755.1|	turquoise	930.23	926.545	0.875623	0.051662	162.578
gi|52351324|gb|AY609756.1|	lightgreen	59.214	16.8703	0.99363	0.00061	2.168
gi|52351346|gb|AY609778.1|	yellow	184.3	79.6668	0.983685	0.002496	42.936
gi|52351354|gb|AY609784.1|	turquoise	102.92	77.2418	0.341133	0.574233	28.532
gi|52351363|gb|AY609793.1|	turquoise	1567.4	1566.99	0.999023	3.67E-05	5.722
gi|52351368|gb|AY609798.1|	yellow	304.84	55.7967	0.862171	0.060139	39.328
gi|52351374|gb|AY609804.1|	brown	242.12	120.91	0.981725	0.002958	3.918
gi|52351397|gb|AY609827.1|	blue	311.19	133.707	0.962486	0.008673	13.934
gi|52351402|gb|AY609832.1|	turquoise	1563.7	1563.31	0.999142	3.02E-05	5.944
gi|52351406|gb|AY609836.1|	grey60	249.49	32.7087	0.995222	0.000396	77.06
gi|52351419|gb|AY609849.1|	turquoise	485.4	470.591	0.707255	0.181556	98.418
gi|52351429|gb|AY609859.1|	turquoise	949.91	948.726	0.888285	0.044064	15.614
gi|52351430|gb|AY609860.1|	turquoise	1301.9	1300.68	0.958418	0.010115	151.146
gi|52351432|gb|AY609862.1|	turquoise	637.04	629.434	0.784052	0.116484	37.714
gi|52351441|gb|AY609871.1|	turquoise	1525.2	1524.69	0.993155	0.000679	20.07
gi|52351444|gb|AY609874.1|	turquoise	1462.9	1462.27	0.983773	0.002475	111.832
gi|52351454|gb|AY609884.1|	turquoise	216.93	185.288	0.476129	0.417528	85.632
gi|52351457|gb|AY609887.1|	yellow	290.31	67.2795	0.914145	0.029806	80.81
gi|52351462|gb|AY609892.1|	turquoise	302.4	287.733	0.605914	0.27875	68.854
gi|52351490|gb|AY609920.1|	turquoise	879.83	876.678	0.867217	0.056912	37.088
gi|52351540|gb|AY609970.1|	brown	317.08	88.6703	0.88538	0.045773	16.252
gi|52351547|gb|AY609977.1|	purple	201.76	33.8655	0.977436	0.004055	59.39
gi|52351582|gb|AY610012.1|	red	262.93	45.1388	0.960026	0.009536	32.22
gi|52351587|gb|AY610017.1|	turquoise	753.14	747.962	0.825974	0.084836	106.458
gi|52351591|gb|AY610021.1|	blue	281.3	131.399	0.957496	0.010452	54.354
gi|52351593|gb|AY610023.1|	turquoise	130.61	66.3076	0.259291	0.673598	147.776
gi|52351594|gb|AY610024.1|	blue	276.82	116.552	0.929675	0.02215	11.878
gi|52351601|gb|AY610031.1|	brown	310.26	96.085	0.908321	0.03286	9.224
gi|52351606|gb|AY610036.1|	turquoise	1423	1422.26	0.979337	0.003554	3.26
gi|52351609|gb|AY610039.1|	black	195.88	27.1286	0.870143	0.055066	69.476
gi|52351619|gb|AY610049.1|	turquoise	932.35	928.869	0.877782	0.05034	24.394

gi|52351620|gb|AY610050.1|	turquoise	271.07	236.797	0.520189	0.368893	42.84
gi|52351621|gb|AY610051.1|	turquoise	421.42	413.142	0.6862	0.200786	66.024
gi|52351639|gb|AY610069.1|	navy	270.36	33.7009	0.992121	0.000838	23.358
gi|52351640|gb|AY610070.1|	turquoise	131.17	57.266	0.14116	0.820868	2.504
gi|52351660|gb|AY610090.1|	brown	223.61	126.978	0.995938	0.000311	7.032
gi|52351676|gb|AY610106.1|	grey60	245.2	31.9829	0.990024	0.001194	18.782
gi|52351682|gb|AY610112.1|	turquoise	1180.1	1178.44	0.936817	0.018883	22.098
gi|52351696|gb|AY610126.1|	turquoise	1558.2	1557.77	0.99851	6.90E-05	8.656
gi|52351697|gb|AY610127.1|	magenta	311.23	42.5603	0.98488	0.002227	153.516
gi|52351701|gb|AY610131.1|	turquoise	855.54	851.942	0.858473	0.062539	25.752
gi|52351702|gb|AY610132.1|	turquoise	838.75	836.516	0.850921	0.067531	54.798
gi|52351717|gb|AY610147.1|	brown	253	127.568	0.994235	0.000525	19.642
gi|52351736|gb|AY610166.1|	black	251.94	38.4073	0.979127	0.003609	139.796
gi|52351755|gb|AY610185.1|	brown	255.27	122.12	0.980627	0.003227	4.382
gi|52351777|gb|AY610207.1|	magenta	296.06	39.7295	0.968461	0.006692	27.448
gi|52351803|gb|AY610221.1|	turquoise	561.85	556.522	0.769481	0.128167	5.638
gi|52351807|gb|AY610225.1|	midnightblue	229	34.9589	0.987409	0.001693	24.796
gi|52351848|gb|AY610266.1|	turquoise	1300	1299.3	0.95007	0.013292	23.414
gi|52351862|gb|AY610280.1|	turquoise	1480.4	1479.97	0.984021	0.002419	4.006
gi|52351873|gb|AY610291.1|	pink	281.19	39.9859	0.951254	0.012824	131.136
gi|52351877|gb|AY610295.1|	yellow	243.7	73.5987	0.946505	0.014733	146.912
gi|52351890|gb|AY610308.1|	turquoise	1033.4	1031.61	0.899321	0.037763	49.962
gi|52351898|gb|AY610316.1|	turquoise	1522	1521.47	0.992818	0.00073	4.306
gi|52351905|gb|AY610323.1|	lightyellow	310.33	25.2283	0.986571	0.001864	29.782
gi|52351956|gb|AY610374.1|	turquoise	1340.3	1339.49	0.966354	0.007371	43.476
gi|52351961|gb|AY610379.1|	turquoise	1329.1	1328.3	0.960468	0.009379	22.018
gi|52351978|gb|AY610394.1|	red	247.77	50.0307	0.985071	0.002185	133.056
gi|52352028|gb|AY610444.1|	turquoise	1533.8	1533.3	0.995118	0.000409	5.982
gi|52352037|gb|AY610453.1|	turquoise	1372.8	1371.83	0.970321	0.00611	8.722
gi|52352038|gb|AY610454.1|	turquoise	1134.5	1133.72	0.923809	0.024955	29.304
gi|52352065|gb|AY610481.1|	darkgreen	272.29	24.1384	0.997528	0.000148	6.062
gi|52352069|gb|AY610485.1|	turquoise	1418	1417.4	0.972688	0.005396	10.644
gi|52352072|gb|AY610488.1|	turquoise	1454.7	1453.93	0.983619	0.002511	16.418
gi|52352707|gb|AY609627.1|	turquoise	1402.3	1401.71	0.976495	0.004311	8.366
gi|52352717|gb|AY609637.1|	turquoise	624.87	615.458	0.773464	0.12494	19.198
gi|52352721|gb|AY609641.1|	turquoise	1532.9	1532.52	0.992931	0.000713	7.252
gi|52352730|gb|AY609650.1|	turquoise	1484.1	1483.6	0.986429	0.001894	27.258
gi|54020965|ref|NM_001005726.1|	turquoise	464.68	449.588	0.693803	0.193779	281.55
gi|54607194|ref|NM_001006592.1|	red	257.63	49.4895	0.982862	0.002686	4419.722
gi|55741485|ref|NM_213855.1|	grey	200.03	12.0369	0.805759	0.099717	623.904
gi|55741489|ref|NM_214136.1|	red	232.59	50.4905	0.989954	0.001207	18.784
gi|55741720|ref|NM_001001534.1|	black	291.56	40.9955	0.970331	0.006107	254.328
gi|55741808|ref|NM_213748.1|	blue	332.66	137.172	0.967766	0.006913	1382.118
gi|55741810|ref|NM_001001863.1|	pink	232.17	42.2908	0.971842	0.005648	1892.508
gi|55741850|ref|NM_001001264.1|	turquoise	1334	1332.89	0.962408	0.0087	0.69
gi|55742741|ref|NM_213920.1|	yellow	114.29	50.5924	0.845934	0.070892	1625.384
gi|56199611|gb|AY705447.1|	yellow	226.21	78.8899	0.982554	0.002759	14.028
gi|56606056|ref|NM_001008481.1|	turquoise	100.81	81.0352	0.348507	0.565421	23.296

gi|56711365|ref|NM_001008688.1|	black	197.96	28.6808	0.905181	0.034547	957.722
gi|56792885|gb|AY705918.1|	lightgreen	85.761	7.50931	0.395867	0.509459	2.688
gi|56792886|gb|AY705919.1|	turquoise	1449.3	1448.67	0.979012	0.003639	6.262
gi|57527981|ref|NM_001009576.1|	turquoise	1538.6	1538.16	0.99405	0.00055	18.642
gi|57528019|ref|NM_001009581.1|	turquoise	1462.2	1461.46	0.983629	0.002508	12.954
gi|57528034|ref|NM_001009582.1|	purple	215.49	32.8456	0.976893	0.004202	493.376
gi|58801554|ref|NM_001011727.1|	turquoise	118.34	62.9596	0.230466	0.70918	35.666
gi|59709497|ref|NM_001012299.1|	turquoise	1553.6	1553.18	0.996528	0.000245	0.426
gi|6007613|gb|AF120099.1|AF120099	turquoise	1440.2	1439.54	0.98229	0.002822	17.278
gi|60097952|ref|NM_001012406.1|	blue	314.89	141.836	0.974918	0.00475	14.59
gi|61097882|ref|NM_001012956.1|	turquoise	1496.1	1495.59	0.987823	0.00161	18.436
gi|61696631|gb|AY803094.1|	tan	299.26	39.5085	0.991574	0.000927	22.096
gi|61696637|gb|AY803100.1|	yellow	240.27	78.0741	0.97878	0.003699	24.432
gi|61696660|gb|AY803123.1|	turquoise	1553.6	1553.18	0.996528	0.000245	0.472
gi|62082661|gb|AY870324.1|	pink	152.78	36.4631	0.937513	0.018574	37.738
gi|6581130|gb|AF210153.1|AF210153	turquoise	1193.1	1192.19	0.9317	0.021206	6.624
gi|68534989|ref|NM_001025224.1|	red	260.47	49.17	0.980943	0.003149	43.262
gi|71834281|ref|NM_214197.2|	brown	167.05	89.227	0.901399	0.036612	226.084
gi|72535167|ref|NM_001031778.1|	turquoise	1254.5	1253.19	0.950357	0.013178	17.822
gi|72535177|ref|NM_001031781.1|	turquoise	164.92	144.464	0.448372	0.448866	266.014
gi|72535201|ref|NM_001031794.1|	turquoise	370.85	362.9	0.648592	0.236438	517.742
gi|73853881|ref|NM_001032355.1|	turquoise	1406.7	1405.84	0.975312	0.004639	21.748
gi|73853889|ref|NM_001032359.1|	green	218.7	52.432	0.979222	0.003584	72.186
gi|73853893|ref|NM_001032358.1|	turquoise	1470.4	1469.94	0.985809	0.002025	23.64
gi|74136760|ref|NM_001033014.1|	yellow	140.22	65.2353	0.924755	0.024496	78.028
gi|77628017|ref|NM_001008689.2|	turquoise	477.98	470.969	0.72522	0.165595	31.612
gi|77681320|dbj|AB236885.1|	turquoise	1555.2	1554.77	0.996648	0.000233	11.03
gi|77745211|gb|DQ222847.1|	turquoise	1552.3	1551.88	0.99765	0.000137	8.078
gi|80861404|ref|NM_001037145.1|	turquoise	914.91	913.724	0.872015	0.053896	1.992
gi|80971505|ref|NM_001037147.1|	turquoise	1426	1425.5	0.975466	0.004596	2.91
gi|82617535|ref|NM_001037322.1|	brown	128.25	69.4764	0.844818	0.071651	32.914
gi|83616153|gb|DQ241739.1|	turquoise	1474.3	1473.86	0.984751	0.002255	4.758
gi|83816988|ref|NM_001037965.1|	turquoise	1506.5	1506.11	0.989234	0.001339	14.57
gi|83921642|ref|NM_001038007.1|	turquoise	1510	1509.39	0.99149	0.000941	222.13
gi|84490428|ref|NM_001038636.1|	turquoise	1508.2	1507.78	0.990504	0.001109	10.5
gi|84490432|ref|NM_001038631.1|	turquoise	178.95	163.794	0.48142	0.411614	6.488
gi|84619523|ref|NM_001038694.1|	turquoise	176.3	156.93	0.470032	0.424368	6.922
gi|85838731|gb|DQ351713.1|	turquoise	1351.3	1350.24	0.965312	0.007715	2.51
gi|89886172|ref|NM_001039748.1|	brown	106.2	40.9163	0.693094	0.194429	60.502
gi|89886174|ref|NM_001039749.1|	turquoise	1483.9	1483.19	0.988343	0.001508	9.4
gi|90017493|ref|NM_214184.2|	turquoise	244.99	229.763	0.569916	0.315821	269.218
gi|91176934|gb|DQ450676.1|	turquoise	1483.9	1483.46	0.986784	0.00182	6.728
gi|92020093|dbj|AB237777.1|	magenta	339.83	44.9817	0.995503	0.000362	62.462
gi|9802381|gb|AF281156.1|AF281156	turquoise	1497.6	1497.15	0.987395	0.001696	5.012
gnl|UG|Ssc#S14766801	turquoise	1536.3	1535.92	0.994357	0.000508	1.772
gnl|UG|Ssc#S14767272	turquoise	1553.6	1553.18	0.996528	0.000245	1.086
gnl|UG|Ssc#S15981094	turquoise	1548	1547.52	0.99693	0.000204	6.672
gnl|UG|Ssc#S15981318	turquoise	1553.6	1553.18	0.996528	0.000245	1.228

gnl|UG|Ssc#S15981406	turquoise	1553.6	1553.18	0.996528	0.000245	0.85
gnl|UG|Ssc#S15981469	turquoise	1565.7	1565.33	0.998457	7.27E-05	3.34
gnl|UG|Ssc#S15981525	darkgreen	238.82	22.9018	0.981044	0.003124	7.806
gnl|UG|Ssc#S16350384	turquoise	1566.7	1566.31	0.999232	2.55E-05	2.764
gnl|UG|Ssc#S16350749	turquoise	1555.8	1555.44	0.997316	0.000167	3.55
gnl|UG|Ssc#S16444449	grey	51.508	8.01581	-0.0476	0.939411	17.834
gnl|UG|Ssc#S16511729	salmon	256.78	25.1143	0.923306	0.025201	152.936
gnl|UG|Ssc#S16512910	lightcyan	268.61	24.7687	0.983008	0.002652	9.376
gnl|UG|Ssc#S16513514	darkturquoise	246.75	16.2254	0.965116	0.00778	14.128
gnl|UG|Ssc#S16513635	turquoise	1538.6	1538.15	0.99461	0.000475	1.98
gnl|UG|Ssc#S16513921	black	263.05	40.5245	0.977175	0.004125	30.448
gnl|UG|Ssc#S16514147	turquoise	932.74	929.492	0.879113	0.04953	9.798
gnl|UG|Ssc#S16514640	green	265.86	49.7022	0.95818	0.010202	38.518
gnl|UG|Ssc#S16514775	blue	270.8	132.256	0.958908	0.009937	57.216
gnl|UG|Ssc#S16514926	turquoise	1465.2	1464.76	0.981095	0.003111	1.182
gnl|UG|Ssc#S16514958	black	233.8	33.0854	0.944117	0.015724	344.932
gnl|UG|Ssc#S16515033	turquoise	484.71	479.806	0.718234	0.171752	28.26
gnl|UG|Ssc#S16515706	grey60	250.36	30.6843	0.981359	0.003047	27.284
gnl|UG|Ssc#S16515995	turquoise	1505.4	1505.02	0.988146	0.001547	5.046
gnl|UG|Ssc#S16516579	turquoise	833.39	830.278	0.850143	0.068052	4.342
gnl|UG|Ssc#S16517655	turquoise	1563.2	1562.81	0.998198	9.18E-05	14.338
gnl|UG|Ssc#S16517726	pink	198.63	46.1586	0.990942	0.001034	23.09
gnl|UG|Ssc#S16518132	black	105.88	9.51914	0.565553	0.320395	6.856
gnl|UG|Ssc#S16761546	turquoise	845.93	843.773	0.849732	0.068327	0.282
gnl|UG|Ssc#S16761675	turquoise	1567	1566.57	0.999188	2.78E-05	4.368
gnl|UG|Ssc#S16763004	red	200.03	49.5241	0.986068	0.00197	6.648
gnl|UG|Ssc#S16763114	blue	325.48	132.504	0.959279	0.009804	6.828
gnl|UG|Ssc#S16763478	navy	241.26	31.7696	0.980483	0.003263	21.422
gnl|UG|Ssc#S16763868	darkred	119.03	11.3595	0.856912	0.063561	15.358
gnl|UG|Ssc#S16763939	turquoise	1114.2	1112.95	0.912733	0.030538	43.312
gnl|UG|Ssc#S16764113	turquoise	1432.7	1431.94	0.979741	0.003451	44.752
gnl|UG|Ssc#S16764287	turquoise	1546.2	1545.83	0.994852	0.000443	3.242
gnl|UG|Ssc#S16764576	turquoise	1442.2	1441.68	0.976253	0.004377	2.99
gnl|UG|Ssc#S16764603	turquoise	1519.4	1518.9	0.992941	0.000711	4.4
gnl|UG|Ssc#S16765575	turquoise	1553.6	1553.18	0.996528	0.000245	0.142
gnl|UG|Ssc#S16766234	brown	219.55	106.212	0.949717	0.013433	7.026
gnl|UG|Ssc#S16767076	turquoise	113.75	86.7592	0.322274	0.596886	50.542
gnl|UG|Ssc#S16767178	blue	286.52	141.46	0.974492	0.004872	8.364
gnl|UG|Ssc#S16769298	turquoise	1568.1	1567.71	0.999478	1.43E-05	2.816
gnl|UG|Ssc#S16769299	turquoise	1449.3	1448.55	0.98284	0.002691	23.216
gnl|UG|Ssc#S16769740	lightgreen	48.028	12.7203	0.846245	0.070681	13.53
gnl|UG|Ssc#S16769741	turquoise	192.48	167.455	0.467781	0.4269	29.752
gnl|UG|Ssc#S16769794	turquoise	1115.5	1113.86	0.920946	0.026364	1.156
gnl|UG|Ssc#S16769802	turquoise	1553.6	1553.18	0.996528	0.000245	1.464
gnl|UG|Ssc#S16770187	turquoise	1261.9	1260.76	0.952969	0.012157	52.768
gnl|UG|Ssc#S17499549	turquoise	1015.6	1014.31	0.890365	0.042853	5.582
gnl|UG|Ssc#S17499579	green	137.69	39.866	0.909731	0.032112	64.37
gnl|UG|Ssc#S17499681	turquoise	347.83	339.178	0.641929	0.242919	171.194

gnl|UG|Ssc#S17500019	turquoise	1529.7	1529.17	0.994359	0.000508	3.416
gnl|UG|Ssc#S17500067	turquoise	1502.4	1501.89	0.989096	0.001365	1.714
gnl|UG|Ssc#S17500137	turquoise	1551.1	1550.6	0.996856	0.000212	3.958
gnl|UG|Ssc#S17501425	brown	251.37	111.111	0.963054	0.008477	13.128
gnl|UG|Ssc#S17503526	turquoise	1531.2	1530.68	0.994663	0.000468	19.538
gnl|UG|Ssc#S17503531	turquoise	1543.4	1542.97	0.996545	0.000244	0.604
gnl|UG|Ssc#S17503771	turquoise	1541.1	1540.74	0.995062	0.000416	15.174
gnl|UG|Ssc#S17503882	turquoise	1316.6	1315.55	0.956355	0.010873	31.748
gnl|UG|Ssc#S17504469	turquoise	1421.5	1421.03	0.973638	0.005118	9.726
gnl|UG|Ssc#S17505417	turquoise	1420.8	1420.21	0.975235	0.004661	5.822
gnl|UG|Ssc#S17506191	purple	233.71	34.4261	0.986358	0.001909	49.954
gnl|UG|Ssc#S17509834	turquoise	1570.4	1570.02	0.999449	1.55E-05	1.596
gnl|UG|Ssc#S17509854	yellow	225.98	49.9927	0.824858	0.085638	36.682
gnl|UG|Ssc#S17509886	grey	287.83	16.6552	0.964174	0.008096	297.692
gnl|UG|Ssc#S17509925	turquoise	1531.1	1530.75	0.992671	0.000752	13.808
gnl|UG|Ssc#S17509934	yellow	288.81	59.1083	0.875966	0.051451	18.31
gnl|UG|Ssc#S17510046	turquoise	1514.7	1514.34	0.989358	0.001316	13.126
gnl|UG|Ssc#S17510077	greenyellow	235.07	20.2303	0.938163	0.018287	40.664
gnl|UG|Ssc#S17510145	turquoise	1519.5	1518.98	0.992033	0.000853	6.312
gnl|UG|Ssc#S17510233	yellow	211.78	59.6649	0.908696	0.032661	119.288
gnl|UG|Ssc#S17510300	turquoise	1571.1	1570.75	0.999627	8.66E-06	1.406
gnl|UG|Ssc#S17510311	turquoise	1377	1376.02	0.971785	0.005665	20.1
gnl|UG|Ssc#S17510396	turquoise	544.66	536.379	0.753983	0.14095	103.442
gnl|UG|Ssc#S17510398	salmon	168.8	29.8734	0.980698	0.00321	361.384
gnl|UG|Ssc#S17510408	turquoise	1547.3	1546.83	0.995358	0.000379	14.988
gnl|UG|Ssc#S17510469	turquoise	1285.9	1285.17	0.950588	0.013087	6.234
gnl|UG|Ssc#S17510489	turquoise	1333.7	1332.79	0.964003	0.008154	30.71
gnl|UG|Ssc#S17510525	turquoise	719.75	716.713	0.806934	0.098832	34.826
gnl|UG|Ssc#S17510588	turquoise	1455.6	1454.99	0.984471	0.002318	5.272
gnl|UG|Ssc#S17510601	turquoise	818.19	814.753	0.854751	0.064984	15.808
gnl|UG|Ssc#S17510736	turquoise	146.58	60.941	0.181272	0.770468	17.33
gnl|UG|Ssc#S17510761	turquoise	1566.9	1566.48	0.998729	5.44E-05	3.904
gnl|UG|Ssc#S17510766	turquoise	207.75	166.367	0.452167	0.444552	23.298
gnl|UG|Ssc#S17510781	lightgreen	54.774	15.4019	0.912191	0.03082	3.588
gnl|UG|Ssc#S17510796	brown	224.41	112.289	0.965611	0.007616	10.612
gnl|UG|Ssc#S17510986	midnightblue	251.82	36.1917	0.994091	0.000545	524.668
gnl|UG|Ssc#S17511034	turquoise	1500.7	1500.3	0.987992	0.001577	10.178
gnl|UG|Ssc#S17511213	turquoise	1359	1358.53	0.962343	0.008722	8.184
gnl|UG|Ssc#S17511222	brown	317.26	89.1831	0.887553	0.044493	7.762
gnl|UG|Ssc#S17511240	turquoise	253.94	214.403	0.504613	0.385924	86.772
gnl|UG|Ssc#S17511285	green	251.2	53.3132	0.987261	0.001723	314.614
gnl|UG|Ssc#S17511386	navy	253.95	31.8617	0.979461	0.003523	101.386
gnl|UG|Ssc#S17511415	turquoise	1553.6	1553.18	0.996528	0.000245	0.284
gnl|UG|Ssc#S17511419	pink	182.46	43.0254	0.975206	0.004669	24.436
gnl|UG|Ssc#S17511420	turquoise	433.45	416.994	0.680883	0.205727	19.134
gnl|UG|Ssc#S17511439	blue	301.21	150.263	0.987691	0.001636	51.878
gnl|UG|Ssc#S17511660	brown	279.56	117.322	0.970932	0.005923	6.566
gnl|UG|Ssc#S17511723	grey60	218.82	30.8415	0.982909	0.002675	39.716

gnl|UG|Ssc#S17511731	yellow	255.75	83.2955	0.990808	0.001056	14.242
gnl|UG|Ssc#S17511815	blue	340.92	140.001	0.971228	0.005833	4.58
gnl|UG|Ssc#S17511884	turquoise	866.73	864.944	0.856529	0.063813	8.978
gnl|UG|Ssc#S17511887	blue	253.18	118.477	0.93429	0.02002	32.114
gnl|UG|Ssc#S17511933	turquoise	1333.8	1332.99	0.961258	0.009101	10.034
gnl|UG|Ssc#S17512154	midnightblue	269.14	34.1567	0.981434	0.003028	54.326
gnl|UG|Ssc#S17512186	turquoise	930.59	927.806	0.879817	0.049103	28.676
gnl|UG|Ssc#S17512211	brown	253.55	110.629	0.95644	0.010842	3.59
gnl|UG|Ssc#S17512237	brown	267.73	126.237	0.988552	0.001468	20.406
gnl|UG|Ssc#S17512262	turquoise	576.04	564.611	0.750932	0.143509	14.928
gnl|UG|Ssc#S17512269	royalblue	209.67	26.5279	0.998832	4.79E-05	77.53
gnl|UG|Ssc#S17512292	purple	144.14	23.096	0.888325	0.044041	26.49
gnl|UG|Ssc#S17512305	tan	266.47	35.293	0.967599	0.006967	10.552
gnl|UG|Ssc#S17513098	pink	211.06	36.0105	0.932879	0.020663	43.826
gnl|UG|Ssc#S17513113	brown	275.3	126.425	0.988537	0.001471	49.36
gnl|UG|Ssc#S17513155	turquoise	1553.6	1553.18	0.996528	0.000245	0.094
gnl|UG|Ssc#S17513253	darkred	70.027	13.9113	0.986217	0.001938	62.414
gnl|UG|Ssc#S17513449	turquoise	1081	1079.26	0.918075	0.0278	351.302
gnl|UG|Ssc#S17513547	turquoise	1525.4	1524.91	0.992924	0.000714	2.39
gnl|UG|Ssc#S17513694	blue	343.94	122.175	0.940496	0.017268	21.796
gnl|UG|Ssc#S17513789	turquoise	1554.9	1554.5	0.99782	0.000122	8.042
gnl|UG|Ssc#S17513836	turquoise	273.84	268.957	0.611196	0.273413	38.17
gnl|UG|Ssc#S17513916	turquoise	1554.9	1554.5	0.996742	0.000223	30.222
gnl|UG|Ssc#S17513958	grey	249.36	15.4768	0.926707	0.023556	41.502
gnl|UG|Ssc#S17513979	navy	283.93	28.2912	0.945555	0.015125	30.55
gnl|UG|Ssc#S17514217	turquoise	1054.1	1052.06	0.903377	0.035527	11.44
gnl|UG|Ssc#S17514245	darkgreen	325.58	21.0836	0.976839	0.004217	34.842
gnl|UG|Ssc#S17514318	greenyellow	91.276	6.74249	-0.45223	0.444481	48.384
gnl|UG|Ssc#S17514325	yellow	84.336	30.975	0.727072	0.163974	35.792
gnl|UG|Ssc#S17514578	purple	97.521	9.50374	0.672393	0.213687	20.052
gnl|UG|Ssc#S17514582	turquoise	1553.6	1553.18	0.996528	0.000245	6.754
gnl|UG|Ssc#S17514632	turquoise	125.11	93.4322	0.337819	0.578202	11.43
gnl|UG|Ssc#S17514665	turquoise	1553.6	1553.18	0.996528	0.000245	0.142
gnl|UG|Ssc#S17514667	blue	307.34	145.739	0.981076	0.003116	23.448
gnl|UG|Ssc#S17514753	turquoise	1425.4	1424.6	0.977703	0.003983	19.196
gnl|UG|Ssc#S17515039	yellow	117.99	51.5705	0.846891	0.070242	111.258
gnl|UG|Ssc#S17515082	turquoise	1553.6	1553.18	0.996528	0.000245	1.558
gnl|UG|Ssc#S17515091	turquoise	1552.8	1552.41	0.995913	0.000313	3.3
gnl|UG|Ssc#S17515316	turquoise	1444.7	1444.04	0.980256	0.00332	54.976
gnl|UG|Ssc#S17515524	turquoise	188.29	104.154	0.281885	0.645904	3.854
gnl|UG|Ssc#S17515611	turquoise	1394.3	1393.59	0.974728	0.004805	119.022
gnl|UG|Ssc#S17515701	turquoise	1484.1	1483.69	0.985105	0.002177	14.356
gnl|UG|Ssc#S17515754	turquoise	189.16	102.829	0.262129	0.670109	23.876
gnl|UG|Ssc#S17515809	brown	156.14	84.0897	0.904197	0.03508	56.766
gnl|UG|Ssc#S17515842	turquoise	1180.8	1179.34	0.932338	0.020912	11.334
gnl|UG|Ssc#S17515868	turquoise	1392.3	1391.75	0.967689	0.006938	6.112
gnl|UG|Ssc#S17515869	turquoise	128	105.344	0.3878	0.518909	4.462
gnl|UG|Ssc#S17516031	turquoise	465.33	453.148	0.710498	0.178645	7.858

gnl|UG|Ssc#S17516064	turquoise	1446.4	1445.92	0.977729	0.003976	9.36
gnl|UG|Ssc#S17516095	magenta	332.22	41.4372	0.979026	0.003635	22.31
gnl|UG|Ssc#S17516151	turquoise	637.48	635.166	0.792719	0.109695	5.744
gnl|UG|Ssc#S17516197	turquoise	1553.6	1553.18	0.996528	0.000245	0.566
gnl|UG|Ssc#S17516311	pink	283.69	38.8898	0.944982	0.015363	218.222
gnl|UG|Ssc#S17516362	turquoise	273.54	236.938	0.525039	0.363629	17.584
gnl|UG|Ssc#S17516571	turquoise	1493	1492.47	0.987529	0.001669	3.124
gnl|UG|Ssc#S17516685	turquoise	1533.6	1533.16	0.994768	0.000454	5.452
gnl|UG|Ssc#S17516715	turquoise	893.24	890.457	0.869123	0.055707	1.112
gnl|UG|Ssc#S17516828	grey60	171.06	22.3838	0.911759	0.031046	145.872
gnl|UG|Ssc#S17516917	turquoise	1396.1	1395.15	0.973083	0.00528	45.374
gnl|UG|Ssc#S17516920	red	272.55	45.2479	0.960496	0.009369	19.284
gnl|UG|Ssc#S17516962	turquoise	1505.7	1505.29	0.987605	0.001654	2.596
gnl|UG|Ssc#S17517004	magenta	327.13	43.5433	0.988845	0.001412	25.662
gnl|UG|Ssc#S17517257	turquoise	1319.9	1319.23	0.956003	0.011005	3.708
gnl|UG|Ssc#S17517303	turquoise	1436.5	1435.97	0.975363	0.004625	2.184
gnl|UG|Ssc#S17517405	magenta	329	44.189	0.991979	0.000861	28.704
gnl|UG|Ssc#S17517598	blue	274.26	133.093	0.959626	0.009679	11.862
gnl|UG|Ssc#S17517636	turquoise	1553.6	1553.18	0.996528	0.000245	6.754
gnl|UG|Ssc#S17517701	turquoise	1478.9	1478.48	0.983762	0.002478	3.372
gnl|UG|Ssc#S17517714	turquoise	1494.1	1493.68	0.986388	0.001903	12.938
gnl|UG|Ssc#S17517759	turquoise	1502.9	1502.51	0.9893	0.001326	8.948
gnl|UG|Ssc#S17517764	salmon	97.123	23.3753	0.920031	0.026819	4.482
gnl|UG|Ssc#S17517969	green	78.956	22.3756	0.77379	0.124677	1.124
gnl|UG|Ssc#S17518103	turquoise	1452.4	1451.79	0.979601	0.003487	24.538
gnl|UG|Ssc#S17518104	turquoise	1453.9	1453.3	0.979147	0.003603	19.118
gnl|UG|Ssc#S17518115	pink	206.46	42.1312	0.971888	0.005634	95.858
gnl|UG|Ssc#S17518307	darkred	63.788	13.8455	0.988083	0.001559	37.442
gnl|UG|Ssc#S17518379	turquoise	1417.2	1416.46	0.973482	0.005163	4.422
gnl|UG|Ssc#S17518385	yellow	309.7	66.0547	0.915879	0.028915	5.456
gnl|UG|Ssc#S17518466	turquoise	1412.4	1411.69	0.974919	0.00475	2.66
gnl|UG|Ssc#S17518565	grey	316.31	16.6497	0.948986	0.013725	17.95
gnl|UG|Ssc#S17518685	turquoise	1570.4	1570.02	0.999449	1.55E-05	1.596
gnl|UG|Ssc#S17518691	turquoise	563.01	552.919	0.753879	0.141038	5.262
gnl|UG|Ssc#S17518692	turquoise	1570.6	1570.24	0.99978	3.92E-06	2.106
gnl|UG|Ssc#S17518758	turquoise	1100.7	1099.91	0.922963	0.025369	4.828
gnl|UG|Ssc#S17518793	turquoise	1535.3	1534.85	0.993238	0.000667	14.174
gnl|UG|Ssc#S17518795	navy	285.07	31.2539	0.973912	0.005038	57.868
gnl|UG|Ssc#S17518818	turquoise	1336.4	1335.57	0.961977	0.008849	1.496
gnl|UG|Ssc#S17518997	turquoise	862.91	861.595	0.862094	0.060188	12.252
gnl|UG|Ssc#S17519017	turquoise	1492.9	1492.34	0.988856	0.00141	75.96
gnl|UG|Ssc#S17524473	green	215.63	50.5834	0.969286	0.006432	22.06
gnl|UG|Ssc#S17524578	red	264.11	48.03	0.974638	0.00483	3.078
gnl|UG|Ssc#S17524628	turquoise	1571.4	1571	0.999719	5.67E-06	1.97
gnl|UG|Ssc#S17524702	turquoise	1057.4	1056.32	0.910737	0.031582	33.454
gnl|UG|Ssc#S17524908	turquoise	1516.5	1515.95	0.991927	0.00087	7.016
gnl|UG|Ssc#S17524953	turquoise	36.406	29.1853	0.211685	0.732501	88.892
gnl|UG|Ssc#S17524983	turquoise	157.26	123.277	0.427589	0.472654	17.956

gnl|UG|Ssc#S17524990	turquoise	1446.5	1446.03	0.980356	0.003295	14.064
gnl|UG|Ssc#S17525025	turquoise	1278.7	1277.45	0.952098	0.012495	5.486
gnl|UG|Ssc#S17525035	turquoise	130.53	62.5334	0.242127	0.694754	22.496
gnl|UG|Ssc#S17525079	turquoise	1550.9	1550.52	0.996804	0.000217	1.888
gnl|UG|Ssc#S17525080	turquoise	1499	1498.57	0.987858	0.001603	10.928
gnl|UG|Ssc#S17525104	turquoise	158.92	137.053	0.449577	0.447495	33.57
gnl|UG|Ssc#S17525130	blue	298.12	133.446	0.961802	0.00891	53.276
gnl|UG|Ssc#S17525225	turquoise	1455.3	1454.6	0.982426	0.002789	14.698
gnl|UG|Ssc#S17525302	turquoise	1158.8	1157.31	0.931134	0.021469	1.79
gnl|UG|Ssc#S17525376	brown	250.12	83.9752	0.897254	0.03892	85.17
gnl|UG|Ssc#S17525410	magenta	328.15	44.6688	0.994994	0.000425	29.984
gnl|UG|Ssc#S17525473	turquoise	1372.8	1371.95	0.968617	0.006642	10.158
gnl|UG|Ssc#S17525713	turquoise	1502.7	1502.22	0.989111	0.001362	6.732
gnl|UG|Ssc#S17525753	turquoise	1546.1	1545.67	0.996173	0.000284	25.154
gnl|UG|Ssc#S17525768	turquoise	1548.7	1548.28	0.996053	0.000297	6.912
gnl|UG|Ssc#S17525783	turquoise	1082.7	1081.95	0.918446	0.027613	1.196
gnl|UG|Ssc#S17525847	purple	196.49	28.879	0.940366	0.017324	3.54
gnl|UG|Ssc#S17525865	turquoise	1031.1	1029.41	0.897634	0.038707	30.614
gnl|UG|Ssc#S17525951	turquoise	1479.1	1478.63	0.984875	0.002228	19.5
gnl|UG|Ssc#S17526090	turquoise	1489.5	1488.93	0.987903	0.001594	1.398
gnl|UG|Ssc#S17526093	turquoise	893.07	890.166	0.868821	0.055898	24.872
gnl|UG|Ssc#S17526150	tan	341.68	36.0508	0.969841	0.006259	13.634
gnl|UG|Ssc#S17526185	blue	340.05	157.407	0.998373	7.88E-05	5.376
gnl|UG|Ssc#S17526193	turquoise	701.16	698.986	0.827803	0.083526	11.546
gnl|UG|Ssc#S17526265	turquoise	1451.3	1450.63	0.981909	0.002913	14.828
gnl|UG|Ssc#S17526422	black	294.03	41.8671	0.987274	0.00172	24.954
gnl|UG|Ssc#S17526634	black	293.58	41.8276	0.986495	0.00188	48.616
gnl|UG|Ssc#S17526742	darkturquoise	201.74	17.8388	0.995664	0.000343	19.82
gnl|UG|Ssc#S17526834	turquoise	132.08	110.768	0.395187	0.510254	112.632
gnl|UG|Ssc#S17526874	red	269.84	44.9408	0.95819	0.010198	86.93
gnl|UG|Ssc#S17526922	greenyellow	238.5	19.9197	0.971892	0.005633	15.478
gnl|UG|Ssc#S17526927	magenta	305.23	41.0935	0.976571	0.00429	10.1
gnl|UG|Ssc#S17526940	turquoise	1168.1	1167.21	0.928002	0.022939	10.754
gnl|UG|Ssc#S17526944	turquoise	1553.6	1553.18	0.996528	0.000245	3.402
gnl|UG|Ssc#S17526987	purple	192.15	32.7691	0.974367	0.004908	37.834
gnl|UG|Ssc#S17527052	turquoise	425.88	416.992	0.703703	0.184762	25.418
gnl|UG|Ssc#S17527330	navy	186.89	27.1289	0.944775	0.015449	25.896
gnl|UG|Ssc#S17527438	grey60	192.86	27.4843	0.957946	0.010287	64.762
gnl|UG|Ssc#S17527442	brown	215.82	120.23	0.983607	0.002513	8.224
gnl|UG|Ssc#S17527454	brown	216.23	106.562	0.95415	0.011704	15.692
gnl|UG|Ssc#S18260201	turquoise	1480.4	1479.94	0.987626	0.001649	6.836
gnl|UG|Ssc#S18260225	turquoise	1134.6	1132.96	0.927092	0.023371	18.34
gnl|UG|Ssc#S18260863	purple	78.255	9.97264	0.693788	0.193792	8.06
gnl|UG|Ssc#S18261767	turquoise	1277	1276.46	0.952367	0.01239	6.83
gnl|UG|Ssc#S18262171	blue	273.01	133.044	0.960075	0.009519	26.126
gnl|UG|Ssc#S18262357	turquoise	1513.7	1513.14	0.992807	0.000732	6.498
gnl|UG|Ssc#S18263529	green	166.2	48.2064	0.974131	0.004975	27.542
gnl|UG|Ssc#S18263925	brown	256.85	128.589	0.995313	0.000385	6.596

gnl|UG|Ssc#S18264158	turquoise	1229.4	1228.62	0.941041	0.017033	5.594
gnl|UG|Ssc#S18267174	turquoise	1537.1	1536.72	0.993282	0.00066	43.168
gnl|UG|Ssc#S18267480	turquoise	1538.4	1538.03	0.994226	0.000526	9.98
gnl|UG|Ssc#S18267647	turquoise	447.1	439.364	0.705346	0.183277	55.516
gnl|UG|Ssc#S18268289	turquoise	1329.4	1328.73	0.95767	0.010388	3.614
gnl|UG|Ssc#S18268314	turquoise	1566.9	1566.56	0.998779	5.12E-05	3.624
gnl|UG|Ssc#S18268722	turquoise	1463.3	1462.59	0.984879	0.002227	7.12
gnl|UG|Ssc#S18269927	turquoise	1560.2	1559.8	0.998631	6.08E-05	6.348
gnl|UG|Ssc#S18270450	turquoise	1456	1455.28	0.982079	0.002872	2.184
gnl|UG|Ssc#S18271608	turquoise	991.98	989.643	0.89302	0.041323	15.64
gnl|UG|Ssc#S18272408	turquoise	1570.9	1570.54	0.999793	3.56E-06	2.716
gnl|UG|Ssc#S18272610	turquoise	127.71	105.672	0.384304	0.523015	1.758
gnl|UG|Ssc#S18272798	turquoise	358.94	356.228	0.684177	0.202662	11.88
gnl|UG|Ssc#S18273087	turquoise	1401	1400.19	0.974903	0.004755	4.858
gnl|UG|Ssc#S18274097	turquoise	1492.3	1491.73	0.989108	0.001362	54.598
gnl|UG|Ssc#S18274130	turquoise	1349.2	1348.55	0.966833	0.007215	3.376
gnl|UG|Ssc#S18274330	turquoise	749.83	745.335	0.830392	0.081683	4.604
gnl|UG|Ssc#S18275000	turquoise	1507.3	1506.78	0.990395	0.001128	16.57
gnl|UG|Ssc#S18276175	turquoise	1248.2	1247.47	0.940055	0.017459	5.88
gnl|UG|Ssc#S18276682	tan	231.79	25.6934	0.898	0.038502	9.668
gnl|UG|Ssc#S18276715	turquoise	1566.3	1565.85	0.999156	2.94E-05	14.914
gnl|UG|Ssc#S18277539	blue	260.21	128.769	0.9526	0.0123	135.15
gnl|UG|Ssc#S18278819	turquoise	1504.5	1504.05	0.989926	0.001212	1.766
gnl|UG|Ssc#S18279395	turquoise	1565	1564.6	0.998386	7.78E-05	9.334
gnl|UG|Ssc#S18279557	turquoise	1542	1541.54	0.995848	0.000321	5.706
gnl|UG|Ssc#S18281053	turquoise	1523.8	1523.33	0.993089	0.000689	65.68
gnl|UG|Ssc#S18282381	salmon	227.82	29.6306	0.973622	0.005122	89.302
gnl|UG|Ssc#S18282485	turquoise	1530.3	1529.84	0.992875	0.000721	2.484
gnl|UG|Ssc#S18283093	turquoise	1409.1	1408.47	0.971086	0.005876	21.354
gnl|UG|Ssc#S18283235	green	74.389	15.7727	0.67104	0.214963	17.418
gnl|UG|Ssc#S18284050	turquoise	1362.6	1362.02	0.969886	0.006245	14.026
gnl|UG|Ssc#S18284292	turquoise	1567.6	1567.25	0.998921	4.26E-05	6.332
gnl|UG|Ssc#S18284609	tan	292.17	35.4095	0.966546	0.007308	10.772
gnl|UG|Ssc#S18284670	turquoise	1541.1	1540.69	0.993831	0.000581	6.106
gnl|UG|Ssc#S18284924	turquoise	1291.7	1291.08	0.950698	0.013043	4.036
gnl|UG|Ssc#S18289737	turquoise	1564.9	1564.47	0.999121	3.13E-05	9.096
gnl|UG|Ssc#S18290449	royalblue	188.93	24.9175	0.985969	0.001991	185.454
gnl|UG|Ssc#S18291190	blue	260.46	118.709	0.935962	0.019265	19.976
gnl|UG|Ssc#S18291250	turquoise	1563.3	1562.89	0.998669	5.83E-05	4.094
gnl|UG|Ssc#S18291761	grey	260.89	12.4328	0.834694	0.078649	16.596
gnl|UG|Ssc#S18291801	turquoise	1387.6	1387.14	0.970717	0.005989	8.518
gnl|UG|Ssc#S18292679	navy	196.88	25.7599	0.93221	0.020971	14.25
gnl|UG|Ssc#S18292832	turquoise	1525.8	1525.39	0.993349	0.00065	3.86
gnl|UG|Ssc#S18295298	turquoise	923.7	921.256	0.87667	0.051019	8.59
gnl|UG|Ssc#S18296629	turquoise	1568.2	1567.86	0.999018	3.69E-05	2.068
gnl|UG|Ssc#S18301666	turquoise	1499.4	1498.8	0.989877	0.001221	7.94
gnl|UG|Ssc#S18302191	turquoise	1558.5	1558.13	0.997081	0.000189	2.684
gnl|UG|Ssc#S18332789	turquoise	645.84	640.314	0.794887	0.108016	4.142

gnl|UG|Ssc#S18336060	turquoise	1325.5	1324.83	0.957252	0.010541	7.654
gnl|UG|Ssc#S18336569	blue	352.59	143.625	0.977308	0.004089	30.788
gnl|UG|Ssc#S18337624	turquoise	1539.9	1539.52	0.993976	0.000561	6.89
gnl|UG|Ssc#S18353806	turquoise	1511.7	1511.19	0.990616	0.00109	15.114
gnl|UG|Ssc#S18353913	turquoise	1454	1453.35	0.98217	0.00285	10.244
gnl|UG|Ssc#S18354111	turquoise	1537	1536.5	0.995673	0.000341	9.47
gnl|UG|Ssc#S18354218	blue	258.23	117.57	0.933664	0.020304	17.988
gnl|UG|Ssc#S18354245	brown	317.57	98.846	0.916805	0.028443	8.85
gnl|UG|Ssc#S18354389	greenyellow	85.04	8.29897	0.731064	0.160495	14.242
gnl|UG|Ssc#S18354391	red	205.45	50.4924	0.990207	0.001162	44.278
gnl|UG|Ssc#S18354485	darkturquoise	161.83	17.4891	0.985722	0.002044	49.012
gnl|UG|Ssc#S18354494	lightyellow	310.39	25.5578	0.989531	0.001284	81.862
gnl|UG|Ssc#S18354663	blue	287.68	143.17	0.97644	0.004326	22.768
gnl|UG|Ssc#S18354739	turquoise	844.95	842.734	0.845195	0.071394	3.186
gnl|UG|Ssc#S18354820	turquoise	1543.4	1542.97	0.996035	0.000299	8.192
gnl|UG|Ssc#S18355531	turquoise	655.83	649.496	0.793168	0.109346	14.738
gnl|UG|Ssc#S18355677	turquoise	1549.9	1549.44	0.997552	0.000145	16.78
gnl|UG|Ssc#S18355850	turquoise	1276	1274.72	0.954212	0.01168	23.548
gnl|UG|Ssc#S18355852	yellow	264	57.6811	0.864503	0.05864	28.404
gnl|UG|Ssc#S18355973	tan	235.72	33.3205	0.952589	0.012304	55.092
gnl|UG|Ssc#S18355989	turquoise	171.11	100.478	0.282227	0.645486	18.234
gnl|UG|Ssc#S18355994	turquoise	671.75	667.094	0.800466	0.103732	6.48
gnl|UG|Ssc#S18356013	turquoise	1032.2	1029.97	0.898855	0.038023	9.482
gnl|UG|Ssc#S18356019	turquoise	1502.8	1502.37	0.987252	0.001725	2.752
gnl|UG|Ssc#S18356022	turquoise	1567.2	1566.75	0.999438	1.60E-05	18.308
gnl|UG|Ssc#S18356106	turquoise	1143.1	1141.06	0.925806	0.023988	79.476
gnl|UG|Ssc#S18356219	blue	257.16	116.625	0.932339	0.020912	71.426
gnl|UG|Ssc#S18356252	turquoise	1308.1	1307.3	0.951971	0.012544	1.656
gnl|UG|Ssc#S18356685	turquoise	1545.4	1544.88	0.997069	0.00019	2.612
gnl|UG|Ssc#S18356838	lightyellow	270.25	21.8145	0.94485	0.015418	25.446
gnl|UG|Ssc#S18357219	grey	78.111	7.6924	-0.00144	0.998169	39.888
gnl|UG|Ssc#S18357288	turquoise	1105.3	1102.91	0.917119	0.028284	27.88
gnl|UG|Ssc#S18357309	turquoise	1537	1536.61	0.993503	0.000628	11.214
gnl|UG|Ssc#S18357319	turquoise	1252.1	1251.52	0.943499	0.015985	2.974
gnl|UG|Ssc#S18357321	blue	327.5	156.252	0.996778	0.000219	38.726
gnl|UG|Ssc#S18357333	turquoise	1348.8	1347.72	0.965933	0.007509	17.6
gnl|UG|Ssc#S18357377	turquoise	1219	1218.36	0.942128	0.016567	4.234
gnl|UG|Ssc#S18357398	turquoise	1208.2	1207.16	0.932105	0.021019	12.782
gnl|UG|Ssc#S18357401	turquoise	1308.5	1307.77	0.956414	0.010852	6.334
gnl|UG|Ssc#S18357511	turquoise	910.33	907.373	0.877702	0.050389	136.606
gnl|UG|Ssc#S18357529	turquoise	1571.4	1571.03	0.999739	5.05E-06	1.924
gnl|UG|Ssc#S18357539	turquoise	1322.2	1321.48	0.963775	0.008231	13.508
gnl|UG|Ssc#S18357580	turquoise	1340.9	1339.92	0.963482	0.008331	22.298
gnl|UG|Ssc#S18357620	magenta	304.87	40.8005	0.975372	0.004623	47.212
gnl|UG|Ssc#S18357732	turquoise	1439.3	1438.61	0.977957	0.003916	7.902
gnl|UG|Ssc#S18358120	turquoise	998.53	996.228	0.894237	0.040628	45.702
gnl|UG|Ssc#S18358217	turquoise	1555.7	1555.31	0.996683	0.000229	7.41
gnl|UG|Ssc#S18358279	red	154.99	37.7393	0.926715	0.023552	30.29

gnl|UG|Ssc#S18358324	midnightblue	226.42	36.7682	0.997578	0.000143	31.66
gnl|UG|Ssc#S18358603	turquoise	1521.1	1520.5	0.993767	0.00059	14.454
gnl|UG|Ssc#S18358611	turquoise	598.42	590.351	0.770773	0.127118	2.354
gnl|UG|Ssc#S18358663	turquoise	1264.6	1263.85	0.943057	0.016172	27.57
gnl|UG|Ssc#S18359073	turquoise	615.56	610.266	0.781068	0.118848	53.26
gnl|UG|Ssc#S18359075	royalblue	209.47	25.8539	0.993263	0.000663	5.85
gnl|UG|Ssc#S18359101	brown	129.06	58.3786	0.823346	0.086729	7.264
gnl|UG|Ssc#S18359197	magenta	316.51	36.5287	0.947581	0.014293	10.626
gnl|UG|Ssc#S18359204	black	171.69	24.488	0.859996	0.061547	149.918
gnl|UG|Ssc#S18359209	turquoise	1532.3	1531.95	0.992438	0.000788	17.614
gnl|UG|Ssc#S18359264	black	287.66	41.9333	0.992286	0.000812	49.778
gnl|UG|Ssc#S18359292	grey60	232.78	32.3673	0.992435	0.000789	25.896
gnl|UG|Ssc#S18359512	darkred	54.598	7.95953	0.726985	0.164049	9.814
gnl|UG|Ssc#S18359550	turquoise	1469.2	1468.61	0.985395	0.002114	38.736
gnl|UG|Ssc#S18359557	brown	103.2	46.2964	0.735417	0.156726	49.07
gnl|UG|Ssc#S18359685	turquoise	1481.7	1481.25	0.984816	0.002241	49.388
gnl|UG|Ssc#S18359692	turquoise	1531.5	1531.08	0.994395	0.000503	6.812
gnl|UG|Ssc#S18359711	turquoise	1563.5	1563.12	0.99899	3.85E-05	13.106
gnl|UG|Ssc#S18359743	turquoise	1304.7	1303.65	0.958431	0.01011	24.018
gnl|UG|Ssc#S18359803	turquoise	1517.7	1517.32	0.99217	0.000831	19.85
gnl|UG|Ssc#S18359879	turquoise	77.403	51.4	0.243146	0.693495	4.188
gnl|UG|Ssc#S18360059	turquoise	1531.1	1530.66	0.991998	0.000858	15.492
gnl|UG|Ssc#S18360061	turquoise	1550.5	1550.07	0.995652	0.000344	4.784
gnl|UG|Ssc#S18360447	turquoise	190.76	165.911	0.497607	0.393643	6.898
gnl|UG|Ssc#S18360885	turquoise	1568.1	1567.72	0.999055	3.49E-05	41.814
gnl|UG|Ssc#S18361249	turquoise	1521.1	1520.73	0.991115	0.001004	2.75
gnl|UG|Ssc#S18361423	yellow	257.9	73.953	0.955529	0.011182	217.404
gnl|UG|Ssc#S18361504	turquoise	1554.9	1554.48	0.997053	0.000192	5.212
gnl|UG|Ssc#S18377137	turquoise	1440	1439.49	0.976882	0.004205	34.416
gnl|UG|Ssc#S18377961	purple	232.22	33.1762	0.978294	0.003826	56.822
gnl|UG|Ssc#S18378075	turquoise	1331.1	1330.04	0.961569	0.008992	7.742
gnl|UG|Ssc#S18378125	turquoise	1333	1331.82	0.962473	0.008677	15.708
gnl|UG|Ssc#S18378241	turquoise	1562.1	1561.72	0.998238	8.88E-05	10.322
gnl|UG|Ssc#S18378422	yellow	179.55	54.2952	0.88603	0.045389	36.316
gnl|UG|Ssc#S18378493	turquoise	1478.4	1477.98	0.982826	0.002695	8.368
gnl|UG|Ssc#S18378555	turquoise	1516.4	1516.03	0.991602	0.000923	10.936
gnl|UG|Ssc#S18378622	turquoise	368.4	344.755	0.626622	0.257987	0.728
gnl|UG|Ssc#S18378635	turquoise	935.51	931.518	0.876091	0.051374	32.046
gnl|UG|Ssc#S18378680	pink	157.98	37.8487	0.947126	0.014479	89.55
gnl|UG|Ssc#S18378791	turquoise	1338.2	1337.4	0.959502	0.009724	42.39
gnl|UG|Ssc#S18378854	darkgreen	236.8	22.1421	0.97426	0.004938	26.316
gnl|UG|Ssc#S18378962	turquoise	1444.7	1443.96	0.981849	0.002928	14.756
gnl|UG|Ssc#S18378972	turquoise	601.94	595.643	0.77767	0.12156	71.884
gnl|UG|Ssc#S18379116	turquoise	1535.7	1535.27	0.993552	0.000621	71.356
gnl|UG|Ssc#S18379252	greenyellow	195.84	22.8506	0.994218	0.000527	37.534
gnl|UG|Ssc#S18379340	turquoise	1564.8	1564.36	0.999206	2.69E-05	1.634
gnl|UG|Ssc#S18379413	yellow	142.83	38.5722	0.76767	0.129642	6.348
gnl|UG|Ssc#S18379594	blue	329.77	154.076	0.993597	0.000614	27.862

gnl|UG|Ssc#S18379693	turquoise	1224.9	1223.42	0.945488	0.015153	30.318
gnl|UG|Ssc#S18379831	darkgreen	284.22	24.0666	0.998695	5.66E-05	6.934
gnl|UG|Ssc#S18380006	turquoise	1556.5	1556.02	0.998238	8.88E-05	47.024
gnl|UG|Ssc#S18380089	turquoise	1132.7	1131.14	0.921209	0.026233	1.226
gnl|UG|Ssc#S18380107	green	286.49	39.4794	0.895249	0.040052	47.406
gnl|UG|Ssc#S18380485	royalblue	138.96	19.717	0.929136	0.022403	28.552
gnl|UG|Ssc#S18380498	turquoise	1555.3	1554.89	0.998284	8.53E-05	2.186
gnl|UG|Ssc#S18380653	blue	293.62	125.445	0.94625	0.014838	22.318
gnl|UG|Ssc#S18380710	turquoise	1526.7	1526.19	0.994379	0.000505	42.374
gnl|UG|Ssc#S18381103	turquoise	1429.1	1428.27	0.979255	0.003576	17.824
gnl|UG|Ssc#S18381113	turquoise	1553.6	1553.18	0.996528	0.000245	0.566
gnl|UG|Ssc#S18381258	blue	335.52	145.555	0.980392	0.003286	54.082
gnl|UG|Ssc#S18381735	turquoise	1551.4	1551.03	0.995573	0.000353	4.43
gnl|UG|Ssc#S18381759	turquoise	1512	1511.63	0.988591	0.00146	3.642
gnl|UG|Ssc#S18381810	lightcyan	212.17	18.8035	0.912728	0.030541	37.342
gnl|UG|Ssc#S18381906	turquoise	779.66	776.224	0.84535	0.071289	3.7
gnl|UG|Ssc#S18382205	turquoise	1507.4	1506.94	0.990135	0.001174	1.504
gnl|UG|Ssc#S18382284	black	297.99	38.0547	0.957136	0.010584	45.738
gnl|UG|Ssc#S18382407	brown	250.26	117.988	0.974407	0.004896	23.992
gnl|UG|Ssc#S18382475	turquoise	1526.2	1525.77	0.994165	0.000535	19.176
gnl|UG|Ssc#S18382627	brown	213.66	106.156	0.950601	0.013082	13.542
gnl|UG|Ssc#S18382745	turquoise	1490.4	1490.03	0.987529	0.001669	23.462
gnl|UG|Ssc#S18382807	turquoise	1360.5	1359.67	0.967609	0.006964	101.01
gnl|UG|Ssc#S18383139	turquoise	1497.6	1497.16	0.988203	0.001535	5.042
gnl|UG|Ssc#S18383171	lightgreen	72.529	13.7299	0.919523	0.027073	9.698
gnl|UG|Ssc#S18383234	turquoise	1561.1	1560.63	0.999032	3.61E-05	15.106
gnl|UG|Ssc#S18383248	turquoise	603.14	594.538	0.768094	0.129296	109.836
gnl|UG|Ssc#S18383312	grey	309.48	15.883	0.921601	0.026039	21.01
gnl|UG|Ssc#S18383319	turquoise	1109.5	1107.05	0.917888	0.027895	17.566
gnl|UG|Ssc#S18383368	turquoise	1525.6	1525.1	0.993613	0.000612	1.132
gnl|UG|Ssc#S18383442	salmon	202.18	28.1715	0.960365	0.009416	49.204
gnl|UG|Ssc#S18383473	turquoise	848.85	846.5	0.846556	0.070469	29.062
gnl|UG|Ssc#S18383636	turquoise	88.348	49.2194	0.212083	0.732005	17.554
gnl|UG|Ssc#S18383647	turquoise	1548.5	1548.16	0.995075	0.000415	2.594
gnl|UG|Ssc#S18383725	greenyellow	88.31	5.18414	-0.34143	0.573874	78.988
gnl|UG|Ssc#S18383839	turquoise	1511.8	1511.44	0.989409	0.001306	28.664
gnl|UG|Ssc#S18383932	yellow	99.678	42.606	0.794768	0.108108	125.264
gnl|UG|Ssc#S18384142	turquoise	939.48	937.581	0.874586	0.052301	12.952
gnl|UG|Ssc#S18384560	lightgreen	31.106	6.80504	0.67742	0.208963	6.874
gnl|UG|Ssc#S18385097	turquoise	1413.2	1412.75	0.975095	0.0047	8.948
gnl|UG|Ssc#S18386392	turquoise	1145.8	1144.18	0.929431	0.022264	16.828
gnl|UG|Ssc#S18386541	turquoise	1542.1	1541.69	0.996279	0.000272	29.126
gnl|UG|Ssc#S18386573	turquoise	1426.9	1426.37	0.973854	0.005055	11.57
gnl|UG|Ssc#S18386751	lightyellow	224.23	16.6721	0.882676	0.047383	48.998
gnl|UG|Ssc#S18386777	turquoise	721.4	714.11	0.81067	0.096035	1.842
gnl|UG|Ssc#S18386841	turquoise	1351.3	1350.2	0.965657	0.0076	19.458
gnl|UG|Ssc#S18387078	lightcyan	227.3	21.2913	0.941476	0.016846	116.064
gnl|UG|Ssc#S18387120	turquoise	1339.3	1338.72	0.958905	0.009939	0.756

gnl|UG|Ssc#S18387146	turquoise	1546.9	1546.52	0.996331	0.000267	6.392
gnl|UG|Ssc#S18387207	magenta	337.18	42.8754	0.984815	0.002241	25.632
gnl|UG|Ssc#S18387223	turquoise	1568.6	1568.2	0.999755	4.60E-06	70.27
gnl|UG|Ssc#S18387292	magenta	331.05	41.8468	0.979953	0.003397	48.124
gnl|UG|Ssc#S18387444	turquoise	1559.6	1559.19	0.998505	6.94E-05	1.636
gnl|UG|Ssc#S18387501	turquoise	1498.8	1498.2	0.989537	0.001283	40.584
gnl|UG|Ssc#S18387558	turquoise	383.61	375.468	0.662876	0.222708	72.244
gnl|UG|Ssc#S18387783	royalblue	218.84	25.5206	0.989878	0.001221	65.11
gnl|UG|Ssc#S18387794	turquoise	1558.2	1557.74	0.998068	0.000102	26.578
gnl|UG|Ssc#S18388327	turquoise	1551.2	1550.83	0.996587	0.000239	4.264
gnl|UG|Ssc#S18545724	turquoise	1553.6	1553.18	0.996528	0.000245	1.89
gnl|UG|Ssc#S18545870	blue	293.87	133.908	0.962275	0.008746	14.796
gnl|UG|Ssc#S18546038	grey	70.108	9.82681	-0.03396	0.956768	42.052
gnl|UG|Ssc#S18546109	turquoise	1553.6	1553.18	0.996528	0.000245	0.804
gnl|UG|Ssc#S18546249	turquoise	161.86	144.913	0.470546	0.42379	14.344
gnl|UG|Ssc#S18546252	turquoise	1096.7	1095.52	0.913261	0.030264	17.8
gnl|UG|Ssc#S18546322	turquoise	1571.1	1570.75	0.999627	8.66E-06	1.406
gnl|UG|Ssc#S18546390	black	286.06	35.4307	0.927691	0.023086	133.922
gnl|UG|Ssc#S18546426	turquoise	1227.3	1226.18	0.93718	0.018722	73.002
gnl|UG|Ssc#S18546455	green	98.337	19.862	0.730773	0.160747	34.06
gnl|UG|Ssc#S18546458	brown	170.71	77.0048	0.881808	0.047903	3.55
gnl|UG|Ssc#S18546477	purple	257.7	31.9546	0.972227	0.005533	95.766
gnl|UG|Ssc#S18546487	turquoise	1551.1	1550.67	0.997807	0.000123	15.778
gnl|UG|Ssc#S18546502	turquoise	1408.5	1407.87	0.970992	0.005905	12.25
gnl|UG|Ssc#S18546548	turquoise	1511.3	1510.86	0.990989	0.001025	3.026
gnl|UG|Ssc#S18546597	turquoise	1560.2	1559.81	0.997361	0.000163	6.854
gnl|UG|Ssc#S18546732	turquoise	1558.7	1558.26	0.998628	6.10E-05	8.906
gnl|UG|Ssc#S18546780	turquoise	1555.8	1555.42	0.997808	0.000123	9.74
gnl|UG|Ssc#S18546943	turquoise	1016.9	1014.43	0.898528	0.038206	11.014
gnl|UG|Ssc#S18546946	blue	248.7	118.963	0.934047	0.02013	13.348
gnl|UG|Ssc#S18546970	turquoise	1553.6	1553.18	0.996528	0.000245	3.024
gnl|UG|Ssc#S18547043	turquoise	1343.4	1342.43	0.965284	0.007724	12.792
gnl|UG|Ssc#S18547054	turquoise	1453.8	1453.09	0.982316	0.002815	2.426
gnl|UG|Ssc#S18547326	turquoise	1550.6	1550.23	0.996654	0.000232	8.406
gnl|UG|Ssc#S18547346	turquoise	1493	1492.52	0.986595	0.001859	17.558
gnl|UG|Ssc#S18547360	blue	315.42	142.929	0.976567	0.004291	64.294
gnl|UG|Ssc#S18547573	pink	267.77	40.1755	0.955788	0.011085	283.934
gnl|UG|Ssc#S18547766	red	232.05	51.6131	0.994197	0.00053	18.122
gnl|UG|Ssc#S18548111	yellow	114.81	42.0217	0.809111	0.097199	33.19
gnl|UG|Ssc#S18548354	lightyellow	273.55	21.2051	0.95061	0.013078	11.93
gnl|UG|Ssc#S18548374	turquoise	1500.4	1499.96	0.987069	0.001762	2.126
gnl|UG|Ssc#S18548467	turquoise	1258.5	1257.34	0.952055	0.012511	42.718
gnl|UG|Ssc#S18548520	greenyellow	57.271	3.46993	-0.08695	0.889429	31.116
gnl|UG|Ssc#S18548531	tan	261	33.1635	0.954404	0.011607	26.948
gnl|UG|Ssc#S18548560	blue	311.93	148.698	0.985863	0.002014	46.514
gnl|UG|Ssc#S18548660	turquoise	1070.1	1067.38	0.909382	0.032297	126.948
gnl|UG|Ssc#S18548755	turquoise	1525.4	1524.92	0.993754	0.000592	14.692
gnl|UG|Ssc#S18548853	turquoise	1553.6	1553.18	0.996528	0.000245	0.708

gnl|UG|Ssc#S18548934	turquoise	475.07	468.074	0.731534	0.160087	33.224
gnl|UG|Ssc#S18549014	turquoise	513.91	510.634	0.73702	0.155345	0.588
gnl|UG|Ssc#S18549214	turquoise	1537.4	1537.04	0.993527	0.000625	6.584
gnl|UG|Ssc#S18549337	turquoise	397.81	381.269	0.655128	0.230127	63.252
gnl|UG|Ssc#S18549381	turquoise	1553.6	1553.18	0.996528	0.000245	0.142
gnl|UG|Ssc#S18549395	lightcyan	291.23	27.0834	0.986907	0.001795	52.166
gnl|UG|Ssc#S18549403	turquoise	1357	1356.39	0.969531	0.006355	31.784
gnl|UG|Ssc#S18549419	turquoise	1548.5	1548.06	0.995781	0.000329	3.798
gnl|UG|Ssc#S18549491	yellow	233.44	76.7857	0.970884	0.005938	19.764
gnl|UG|Ssc#S18549494	red	262.26	45.6448	0.96232	0.00873	85.238
gnl|UG|Ssc#S18549522	turquoise	593.5	582.51	0.759417	0.136427	17.148
gnl|UG|Ssc#S18549536	turquoise	157.38	83.3489	0.233171	0.70583	165.034
gnl|UG|Ssc#S18549545	turquoise	1447.4	1446.89	0.981063	0.003119	6.604
gnl|UG|Ssc#S18549601	turquoise	629.13	624.991	0.786334	0.114684	17.734
gnl|UG|Ssc#S18549761	grey	295.71	15.1972	0.937128	0.018745	102.028
gnl|UG|Ssc#S18549874	lightyellow	250.33	21.0247	0.926265	0.023767	4.778
gnl|UG|Ssc#S18549877	turquoise	1553.6	1553.18	0.996528	0.000245	0.756
gnl|UG|Ssc#S18549905	royalblue	146.07	21.0779	0.943994	0.015776	39.382
gnl|UG|Ssc#S18549962	turquoise	1322.5	1321.87	0.956135	0.010956	12.638
gnl|UG|Ssc#S18550002	grey	318.17	15.8257	0.927995	0.022942	56.344
gnl|UG|Ssc#S18550086	turquoise	1327.3	1326.7	0.955379	0.011239	19.348
gnl|UG|Ssc#S18550127	turquoise	1537.6	1537.25	0.994222	0.000527	47.838
gnl|UG|Ssc#S18550181	turquoise	316.98	304.61	0.626807	0.257802	1.19
gnl|UG|Ssc#S18550190	brown	118.12	40.8047	0.709103	0.179895	24.714
gnl|UG|Ssc#S18550295	turquoise	1284.1	1283.43	0.948871	0.013771	5.03
gnl|UG|Ssc#S18550348	turquoise	1473.8	1473.34	0.983909	0.002444	6.466
gnl|UG|Ssc#S18550420	blue	239.63	109.78	0.916401	0.028649	36.144
gnl|UG|Ssc#S18550520	turquoise	561.39	556.161	0.752838	0.141909	37.668
gnl|UG|Ssc#S18550560	blue	329.79	155.224	0.995244	0.000393	15.082
gnl|UG|Ssc#S18550631	purple	181.98	27.189	0.925294	0.024235	1.676
gnl|UG|Ssc#S18550647	turquoise	174.98	158.219	0.491825	0.40004	2.492
gnl|UG|Ssc#S18550712	turquoise	1348	1347.36	0.959231	0.009821	15.832
gnl|UG|Ssc#S18550902	black	292.5	41.6707	0.979801	0.003436	168.894
gnl|UG|Ssc#S18551025	turquoise	136.18	121.962	0.448073	0.449207	24.406
gnl|UG|Ssc#S18551055	brown	267.11	105.913	0.939903	0.017525	11.54
gnl|UG|Ssc#S18551170	turquoise	1345.6	1344.99	0.958632	0.010037	2.232
gnl|UG|Ssc#S18551209	grey60	258.53	29.5389	0.97326	0.005228	239.84
gnl|UG|Ssc#S18551494	brown	191.15	101.443	0.948605	0.013878	106.508
gnl|UG|Ssc#S18551519	darkturquoise	174.98	6.00281	0.739862	0.152905	32.158
gnl|UG|Ssc#S18551520	turquoise	456.72	448.883	0.709108	0.179891	36.162
gnl|UG|Ssc#S18551577	turquoise	1558.9	1558.55	0.996984	0.000199	22.924
gnl|UG|Ssc#S18551614	turquoise	1334.1	1333.54	0.959597	0.00969	2.288
gnl|UG|Ssc#S18551640	darkturquoise	175.42	8.89264	0.828862	0.082771	12.876
gnl|UG|Ssc#S18551688	turquoise	1484.3	1483.65	0.988461	0.001485	22.65
gnl|UG|Ssc#S18551801	turquoise	847.98	845.882	0.848576	0.069104	102.958
gnl|UG|Ssc#S18551834	navy	193.31	24.2211	0.917397	0.028143	29.176
gnl|UG|Ssc#S18551845	red	201.4	50.5198	0.990002	0.001198	2.12
gnl|UG|Ssc#S18552012	black	256.05	36.1018	0.933267	0.020486	48.172

gnl|UG|Ssc#S18552081	turquoise	1252.4	1251.38	0.950219	0.013233	14.848
gnl|UG|Ssc#S18552112	turquoise	1551.1	1550.71	0.996908	0.000206	12.394
gnl|UG|Ssc#S18552226	tan	318.28	38.0012	0.981494	0.003014	18.202
gnl|UG|Ssc#S18552237	blue	292.43	110.328	0.917095	0.028296	31.722
gnl|UG|Ssc#S18552312	black	112.89	13.7609	0.651949	0.233191	41.68
gnl|UG|Ssc#S18552383	pink	127.61	31.299	0.903428	0.035499	21.084
gnl|UG|Ssc#S18552418	turquoise	1518.5	1518.05	0.991619	0.00092	3.414
gnl|UG|Ssc#S18552425	brown	234.35	121.01	0.984931	0.002216	16
gnl|UG|Ssc#S18552433	turquoise	255.22	233.788	0.569453	0.316306	19.226
gnl|UG|Ssc#S18552515	lightgreen	72.312	4.93793	0.073729	0.906211	11.726
gnl|UG|Ssc#S18552627	turquoise	1137.4	1135.67	0.928667	0.022624	43.956
gnl|UG|Ssc#S18552822	purple	174.64	27.5104	0.931203	0.021436	2.656
gnl|UG|Ssc#S18553035	green	240.57	52.4682	0.988142	0.001547	82.94
gnl|UG|Ssc#S18553137	turquoise	1057.3	1056.02	0.900639	0.037032	72.572
gnl|UG|Ssc#S18553195	turquoise	1421.8	1420.94	0.977437	0.004055	130.464
gnl|UG|Ssc#S18553224	turquoise	1512	1511.5	0.991239	0.000983	6.042
gnl|UG|Ssc#S18553277	red	149.17	40.9268	0.940883	0.017101	2.756
gnl|UG|Ssc#S18553314	turquoise	1540	1539.49	0.995748	0.000333	2.608
gnl|UG|Ssc#S18553358	turquoise	1547.6	1547.27	0.995117	0.000409	12.292
gnl|UG|Ssc#S18553403	pink	254.78	45.5661	0.984618	0.002285	418.774
gnl|UG|Ssc#S18553548	turquoise	1554	1553.47	0.998018	0.000106	62.802
gnl|UG|Ssc#S18553581	turquoise	422.41	417.217	0.689231	0.197984	77.634
gnl|UG|Ssc#S18553622	turquoise	1548.7	1548.25	0.997045	0.000193	24.558
gnl|UG|Ssc#S18553702	navy	246.61	28.7809	0.960611	0.009329	45.94
gnl|UG|Ssc#S18553743	turquoise	433.54	426.129	0.708723	0.180237	17.15
gnl|UG|Ssc#S18553763	blue	325.24	153.053	0.992018	0.000855	11.774
gnl|UG|Ssc#S18553917	turquoise	1543.8	1543.43	0.995015	0.000422	2.706
gnl|UG|Ssc#S18553939	turquoise	1540.9	1540.55	0.993749	0.000593	18.862
gnl|UG|Ssc#S18554123	turquoise	900.34	898.353	0.860164	0.061438	11.556
gnl|UG|Ssc#S18554156	blue	301.22	134.348	0.963312	0.008389	10.58
gnl|UG|Ssc#S18554195	turquoise	891.25	889.9	0.867999	0.056417	159.298
gnl|UG|Ssc#S18554224	green	103.24	16.0405	0.664472	0.221188	4.874
gnl|UG|Ssc#S18554258	turquoise	1351.6	1350.97	0.963288	0.008397	12.75
gnl|UG|Ssc#S18554282	turquoise	155.49	136.564	0.427833	0.472373	25.106
gnl|UG|Ssc#S18554366	grey60	212.14	29.3692	0.973001	0.005304	71.63
gnl|UG|Ssc#S18554424	magenta	329.31	45.4303	0.998105	9.90E-05	23.498
gnl|UG|Ssc#S18554456	navy	151.42	16.5793	0.826436	0.084505	0.094
gnl|UG|Ssc#S18554486	pink	197.87	45.4031	0.987248	0.001725	42.48
gnl|UG|Ssc#S18554614	blue	318.14	145.055	0.979867	0.003419	8.062
gnl|UG|Ssc#S18554740	turquoise	1048.2	1045.79	0.908343	0.032849	19.804
gnl|UG|Ssc#S18554743	turquoise	1477.7	1477.16	0.984556	0.002299	4.048
gnl|UG|Ssc#S18554829	turquoise	1232.2	1231.54	0.937789	0.018452	3.628
gnl|UG|Ssc#S18554887	yellow	221.09	82.2368	0.989594	0.001272	49.448
gnl|UG|Ssc#S18554897	brown	287.8	118.614	0.968814	0.00658	109.696
gnl|UG|Ssc#S18555004	yellow	251.81	66.9042	0.938452	0.01816	90.844
gnl|UG|Ssc#S18555039	turquoise	1539.5	1539.04	0.995767	0.00033	32.738
gnl|UG|Ssc#S18555055	turquoise	1501.4	1500.79	0.991085	0.001009	22.152
gnl|UG|Ssc#S18555071	lightgreen	62.042	17.1752	0.994886	0.000439	11.7

gnl|UG|Ssc#S18555169	turquoise	1506.6	1506.17	0.988391	0.001499	1.312
gnl|UG|Ssc#S18555277	turquoise	154.35	96.725	0.323839	0.595	21.836
gnl|UG|Ssc#S18555329	green	104.93	24.3159	0.786999	0.114161	7.974
gnl|UG|Ssc#S18555410	turquoise	1555.1	1554.69	0.997216	0.000176	58.646
gnl|UG|Ssc#S18555414	red	206.22	51.0515	0.992381	0.000797	25.374
gnl|UG|Ssc#S18555438	darkgreen	137.58	11.6061	0.837802	0.076479	6.06
gnl|UG|Ssc#S18555480	blue	337.63	146.167	0.981294	0.003063	15.036
gnl|UG|Ssc#S18555551	brown	113.78	50.719	0.748988	0.145146	42.922
gnl|UG|Ssc#S18555613	turquoise	962.94	960.667	0.887572	0.044482	24.054
gnl|UG|Ssc#S18555750	green	98.242	13.1225	0.631358	0.253299	10.836
gnl|UG|Ssc#S18555802	tan	313.26	39.694	0.991002	0.001023	18.038
gnl|UG|Ssc#S18555925	blue	321.91	139.094	0.970384	0.006091	18.266
gnl|UG|Ssc#S18555986	magenta	308.31	35.1957	0.939434	0.017729	32.43
gnl|UG|Ssc#S18556194	tan	320.63	40.5313	0.995816	0.000325	37.976
gnl|UG|Ssc#S18556295	turquoise	699.38	695.426	0.818231	0.090449	91.196
gnl|UG|Ssc#S18556357	turquoise	1564.1	1563.7	0.999383	1.84E-05	24.986
gnl|UG|Ssc#S18556589	yellow	233.61	71.8281	0.952712	0.012256	232.77
gnl|UG|Ssc#S18556629	turquoise	1550.1	1549.69	0.995616	0.000348	2.168
gnl|UG|Ssc#S18556647	turquoise	1064.7	1062.99	0.914722	0.029509	1.382
gnl|UG|Ssc#S18556801	tan	305.15	36.6102	0.972843	0.00535	21.764
gnl|UG|Ssc#S18557007	turquoise	1207	1206.44	0.94101	0.017046	5.54
gnl|UG|Ssc#S18557168	darkturquoise	188.08	17.5162	0.981938	0.002906	12.918
gnl|UG|Ssc#S18557176	turquoise	1553.6	1553.18	0.996528	0.000245	0.992
gnl|UG|Ssc#S18557214	turquoise	1462	1461.58	0.980046	0.003373	13.58
gnl|UG|Ssc#S18557247	turquoise	1525.1	1524.63	0.993663	0.000605	41.234
gnl|UG|Ssc#S18557342	grey60	224.15	32.2482	0.99188	0.000877	54.958
gnl|UG|Ssc#S18557502	yellow	98.449	39.3811	0.80319	0.101659	31.762
gnl|UG|Ssc#S18557525	turquoise	946.94	945.319	0.872671	0.053487	20.288
gnl|UG|Ssc#S18557598	turquoise	1290.1	1289.22	0.948835	0.013786	28.182
gnl|UG|Ssc#S18557699	black	277.87	38.4102	0.949857	0.013377	52.308
gnl|UG|Ssc#S18557753	turquoise	1435.9	1435.33	0.979238	0.00358	9.854
gnl|UG|Ssc#S18557837	brown	326.72	95.814	0.908374	0.032832	595.22
gnl|UG|Ssc#S18557979	turquoise	1393.2	1392.63	0.970865	0.005944	5.364
gnl|UG|Ssc#S18558052	turquoise	1535.2	1534.71	0.994695	0.000463	2.62
gnl|UG|Ssc#S18558087	turquoise	1533.8	1533.44	0.993354	0.00065	6.49
gnl|UG|Ssc#S18558114	turquoise	1561.8	1561.4	0.997774	0.000126	5.304
gnl|UG|Ssc#S18558128	turquoise	1510.5	1510.04	0.989685	0.001256	5.992
gnl|UG|Ssc#S18558146	turquoise	1513.5	1512.91	0.992729	0.000743	6.608
gnl|UG|Ssc#S18558154	turquoise	1452.3	1451.52	0.983305	0.002583	3.548
gnl|UG|Ssc#S18558242	green	154.53	42.8	0.931367	0.021361	41.124
gnl|UG|Ssc#S18558244	turquoise	715.51	711.448	0.812509	0.094667	4.02
gnl|UG|Ssc#S18558287	turquoise	1532	1531.61	0.993229	0.000668	1.714
gnl|UG|Ssc#S18558291	turquoise	1155.2	1153.42	0.931927	0.021102	76.588
gnl|UG|Ssc#S18558299	lightcyan	167.69	6.486	0.155554	0.802744	100.696
gnl|UG|Ssc#S18558304	turquoise	1489.6	1489.05	0.987306	0.001714	13.872
gnl|UG|Ssc#S18558403	lightcyan	292.58	26.0627	0.984654	0.002277	31.51
gnl|UG|Ssc#S19537170	purple	108.3	16.0071	0.791607	0.110559	19.628
gnl|UG|Ssc#S19537215	turquoise	1418.7	1418.16	0.975655	0.004543	7.236

gnl|UG|Ssc#S19538625	turquoise	868.98	866.882	0.866875	0.057129	0.804
gnl|UG|Ssc#S19538787	turquoise	417.38	410.194	0.681351	0.205291	13.426
gnl|UG|Ssc#S19538970	turquoise	963.81	961.394	0.888455	0.043965	274.398
gnl|UG|Ssc#S19538990	greenyellow	96.594	6.13085	0.017206	0.978093	10.626
gnl|UG|Ssc#S19539102	blue	308.41	148.422	0.984875	0.002228	4.618
gnl|UG|Ssc#S19539303	turquoise	1564.6	1564.23	0.998575	6.45E-05	5.124
gnl|UG|Ssc#S19539315	salmon	190.87	30.4577	0.989046	0.001374	13.978
gnl|UG|Ssc#S19539468	turquoise	687.38	680.333	0.800439	0.103753	33.944
gnl|UG|Ssc#S19539620	green	111.05	31.0651	0.843827	0.072326	170.942
gnl|UG|Ssc#S19539677	turquoise	1564.9	1564.55	0.998925	4.23E-05	63.076
gnl|UG|Ssc#S19539691	turquoise	902.1	900.078	0.864284	0.058781	21.292
gnl|UG|Ssc#S19539710	turquoise	1119.3	1118.23	0.925859	0.023963	6.44
gnl|UG|Ssc#S19539925	turquoise	1472.5	1472.05	0.984514	0.002308	48.504
gnl|UG|Ssc#S19540021	turquoise	1549	1548.53	0.997119	0.000186	3.374
gnl|UG|Ssc#S19540060	navy	207.87	27.796	0.950561	0.013098	10.44
gnl|UG|Ssc#S19540127	turquoise	1476.6	1476.05	0.98337	0.002568	0.944
gnl|UG|Ssc#S19540194	turquoise	1109.1	1107.33	0.922437	0.025627	5.086
gnl|UG|Ssc#S19540267	lightcyan	160.72	7.38253	0.243906	0.692556	32.124
gnl|UG|Ssc#S19540289	red	258.96	47.2962	0.971405	0.005779	14.52
gnl|UG|Ssc#S19540314	turquoise	1354.3	1353.64	0.960273	0.009448	14.904
gnl|UG|Ssc#S19540415	turquoise	759.09	756.478	0.819666	0.0894	9.598
gnl|UG|Ssc#S19540428	blue	297.14	140.648	0.973088	0.005278	15.48
gnl|UG|Ssc#S19540474	grey	197.01	12.0931	0.774652	0.123982	17.526
gnl|UG|Ssc#S19540505	blue	331.76	157.696	0.998865	4.59E-05	53.67
gnl|UG|Ssc#S19540541	turquoise	1485.1	1484.57	0.984833	0.002237	2.614
gnl|UG|Ssc#S19540568	turquoise	1563.7	1563.32	0.99823	8.94E-05	3.542
gnl|UG|Ssc#S19540688	greenyellow	58.627	4.91198	-0.37729	0.531269	62.296
gnl|UG|Ssc#S19540774	blue	326.61	154.497	0.994041	0.000552	55.412
gnl|UG|Ssc#S19540874	turquoise	595.79	592.753	0.771692	0.126372	0.814
gnl|UG|Ssc#S19540920	turquoise	1553.6	1553.18	0.996528	0.000245	0.708
gnl|UG|Ssc#S19540934	pink	148.37	29.1164	0.895529	0.039893	41.954
gnl|UG|Ssc#S19541038	turquoise	1501.8	1501.39	0.989054	0.001372	31.836
gnl|UG|Ssc#S19541067	salmon	98.478	7.30517	0.686142	0.20084	53.544
gnl|UG|Ssc#S19541103	turquoise	1242.8	1242.06	0.939654	0.017634	62.64
gnl|UG|Ssc#S19541195	blue	341.72	133.911	0.961343	0.009071	12.83
gnl|UG|Ssc#S19541250	turquoise	1530.6	1530.05	0.994383	0.000505	2.118
gnl|UG|Ssc#S19541354	turquoise	1313.2	1312.61	0.953403	0.01199	3.936
gnl|UG|Ssc#S19541498	brown	322.5	101.419	0.923754	0.024982	20.452
gnl|UG|Ssc#S19541523	turquoise	761.13	759.029	0.827964	0.083411	26.918
gnl|UG|Ssc#S19541571	turquoise	1383.5	1383.04	0.966994	0.007163	8.498
gnl|UG|Ssc#S19541572	yellow	199.75	68.6394	0.937595	0.018538	29.966
gnl|UG|Ssc#S19541579	lightyellow	301.97	25.7587	0.992029	0.000853	30.722
gnl|UG|Ssc#S19541658	purple	226.36	34.4201	0.987935	0.001588	21.17
gnl|UG|Ssc#S19541682	turquoise	1553.6	1553.18	0.996528	0.000245	2.55
gnl|UG|Ssc#S19541687	darkgreen	224.44	20.7408	0.960103	0.009509	15.13
gnl|UG|Ssc#S19541728	turquoise	1214.4	1213.22	0.93549	0.019477	21.472
gnl|UG|Ssc#S19541809	turquoise	725.08	718.759	0.814235	0.093388	17.276
gnl|UG|Ssc#S19541855	yellow	181.51	62.5498	0.908083	0.032987	48.014

gnl|UG|Ssc#S19541858	turquoise	1507.4	1506.94	0.990135	0.001174	1.504
gnl|UG|Ssc#S19541978	turquoise	546.05	541.223	0.76144	0.134755	6.302
gnl|UG|Ssc#S19541992	turquoise	1488.8	1488.2	0.988526	0.001473	10.666
gnl|UG|Ssc#S19542004	turquoise	421.37	409.444	0.693823	0.19376	82.262
gnl|UG|Ssc#S19542017	turquoise	1506.5	1506.07	0.987613	0.001652	27.398
gnl|UG|Ssc#S19542021	turquoise	705.17	699.172	0.812092	0.094976	16.028
gnl|UG|Ssc#S19542031	darkturquoise	226.64	17.97	0.996268	0.000274	153.76
gnl|UG|Ssc#S19542061	blue	347.71	149.955	0.98747	0.001681	174.058
gnl|UG|Ssc#S19542087	salmon	166.23	28.0735	0.966869	0.007203	50.146
gnl|UG|Ssc#S19542228	purple	163.99	21.4509	0.867133	0.056965	17.324
gnl|UG|Ssc#S19542246	salmon	109.32	25.1732	0.938235	0.018255	21.844
gnl|UG|Ssc#S19542276	turquoise	1361.7	1360.7	0.968913	0.006549	8.996
gnl|UG|Ssc#S19542298	yellow	204.26	66.9201	0.93571	0.019379	47.216
gnl|UG|Ssc#S19542401	turquoise	1542.3	1541.91	0.994193	0.000531	4.824
gnl|UG|Ssc#S19542447	turquoise	1566.7	1566.27	0.998795	5.02E-05	14.392
gnl|UG|Ssc#S19542511	purple	208.28	35.8356	0.994118	0.000541	52.162
gnl|UG|Ssc#S19542525	turquoise	1491.3	1490.81	0.987092	0.001757	89.338
gnl|UG|Ssc#S19542616	turquoise	603.28	598.394	0.774389	0.124195	13.116
gnl|UG|Ssc#S19542622	yellow	260.76	65.4981	0.922844	0.025427	43.896
gnl|UG|Ssc#S19542648	turquoise	1406.5	1405.62	0.97493	0.004747	9.86
gnl|UG|Ssc#S19542649	blue	340.54	154.905	0.99489	0.000438	191.984
gnl|UG|Ssc#S19542661	blue	205.7	89.7791	0.872899	0.053346	2.486
gnl|UG|Ssc#S19542674	turquoise	1570.6	1570.25	0.99988	1.57E-06	10.214
gnl|UG|Ssc#S19542684	darkred	71.432	12.5939	0.944602	0.015521	135.568
gnl|UG|Ssc#S19542685	turquoise	1534.4	1533.87	0.995259	0.000392	8.124
gnl|UG|Ssc#S19542749	turquoise	336.66	327.624	0.630449	0.254197	59.058
gnl|UG|Ssc#S19542751	brown	256.38	95.0349	0.925008	0.024373	50.494
gnl|UG|Ssc#S19542771	turquoise	1438.1	1437.65	0.976776	0.004234	5.916
gnl|UG|Ssc#S19542862	turquoise	1355.7	1355.12	0.962015	0.008836	23.498
gnl|UG|Ssc#S19542887	turquoise	661.37	654.377	0.794631	0.108214	0.464
gnl|UG|Ssc#S19542900	turquoise	1038.6	1037.68	0.900618	0.037043	0.682
gnl|UG|Ssc#S19543384	turquoise	1453.2	1452.68	0.982517	0.002768	33.168
gnl|UG|Ssc#S19543409	navy	285.71	26.3161	0.931571	0.021266	6.646
gnl|UG|Ssc#S19543418	turquoise	1471.9	1471.51	0.981636	0.002979	47.638
gnl|UG|Ssc#S19544231	turquoise	1448.9	1448.41	0.97928	0.003569	13.148
gnl|UG|Ssc#S19544740	turquoise	1293.4	1292.18	0.957189	0.010565	15.67
gnl|UG|Ssc#S19544906	magenta	312.88	34.508	0.935655	0.019403	50.86
gnl|UG|Ssc#S19545017	turquoise	1454.1	1453.45	0.982014	0.002888	21.208
gnl|UG|Ssc#S19545319	turquoise	1485.6	1485.05	0.986851	0.001806	2.44
gnl|UG|Ssc#S19545362	pink	127.97	31.1111	0.901763	0.036411	64.14
gnl|UG|Ssc#S19545397	navy	176.94	20.5665	0.878773	0.049736	57.346
gnl|UG|Ssc#S19545449	turquoise	1556.3	1555.89	0.998302	8.40E-05	45.494
gnl|UG|Ssc#S19545458	turquoise	264.09	253.571	0.584958	0.300186	18.886
gnl|UG|Ssc#S19545494	turquoise	1534.1	1533.56	0.994954	0.00043	5.248
gnl|UG|Ssc#S19545503	turquoise	1397.2	1396.25	0.974795	0.004785	11.226
gnl|UG|Ssc#S19545587	turquoise	1509.8	1509.44	0.989178	0.001349	2.248
gnl|UG|Ssc#S19545599	turquoise	1549.9	1549.48	0.996014	0.000302	10.02
gnl|UG|Ssc#S19545642	greenyellow	49.097	4.57481	0.60775	0.276892	75.184

gnl|UG|Ssc#S19545689	turquoise	1448.9	1448.45	0.982617	0.002744	2.22
gnl|UG|Ssc#S19545704	turquoise	1500.4	1499.85	0.987497	0.001675	9.878
gnl|UG|Ssc#S19545759	turquoise	1492.5	1492.02	0.98521	0.002154	3.13
gnl|UG|Ssc#S19545784	turquoise	905.33	902.732	0.876207	0.051303	16.086
gnl|UG|Ssc#S19545891	darkturquoise	204.24	16.7869	0.972876	0.005341	47.276
gnl|UG|Ssc#S19545902	turquoise	1078.2	1076.06	0.915498	0.02911	60.632
gnl|UG|Ssc#S19545927	turquoise	1473.8	1473.41	0.98551	0.002089	5.636
gnl|UG|Ssc#S19545946	turquoise	1553.6	1553.18	0.996528	0.000245	2.126
gnl|UG|Ssc#S19545977	turquoise	60.709	40.9897	0.21497	0.728415	95.3
gnl|UG|Ssc#S19545994	turquoise	1552.3	1551.87	0.997608	0.00014	3.232
gnl|UG|Ssc#S19546082	turquoise	199.1	181.45	0.50073	0.390197	60.314
gnl|UG|Ssc#S19546091	midnightblue	224.59	35.2596	0.988818	0.001417	27.744
gnl|UG|Ssc#S19546106	turquoise	133.39	81.3854	0.321315	0.598042	225.138
gnl|UG|Ssc#S19546116	turquoise	1563.1	1562.67	0.999244	2.49E-05	6.59
gnl|UG|Ssc#S19546121	darkturquoise	151.88	14.5246	0.932239	0.020957	9.084
gnl|UG|Ssc#S19546165	turquoise	1398.8	1398.36	0.973716	0.005095	18.182
gnl|UG|Ssc#S19546179	lightcyan	285.42	26.4161	0.996026	0.000301	12.818
gnl|UG|Ssc#S19546199	turquoise	1553.6	1553.18	0.996528	0.000245	1.654
gnl|UG|Ssc#S19546257	yellow	239.61	71.6123	0.937784	0.018454	172.382
gnl|UG|Ssc#S19546269	turquoise	1052.6	1051.54	0.900105	0.037328	13.586
gnl|UG|Ssc#S19546309	turquoise	298.54	292.615	0.646943	0.238038	9.454
gnl|UG|Ssc#S19546334	turquoise	1525.3	1524.88	0.991554	0.000931	18.626
gnl|UG|Ssc#S19546570	turquoise	1518	1517.38	0.993179	0.000676	10.098
gnl|UG|Ssc#S19546988	green	187.59	28.1822	0.818855	0.089993	63.078
gnl|UG|Ssc#S19547504	turquoise	1364.3	1363.23	0.968007	0.006836	60.588
gnl|UG|Ssc#S19547688	turquoise	1124.8	1123.07	0.92206	0.025812	29.838
gnl|UG|Ssc#S19547691	navy	286.79	32.224	0.981104	0.003109	77.132
gnl|UG|Ssc#S19547820	turquoise	1498	1497.62	0.988237	0.001529	3.336
gnl|UG|Ssc#S19547860	turquoise	1380.3	1379.55	0.973116	0.00527	47.332
gnl|UG|Ssc#S19547872	turquoise	1436.7	1436.04	0.98146	0.003022	1.8
gnl|UG|Ssc#S19547874	turquoise	1553.6	1553.18	0.996528	0.000245	3.07
gnl|UG|Ssc#S19547916	yellow	203.28	62.7253	0.91742	0.028131	11.756
gnl|UG|Ssc#S19548011	lightyellow	319.67	24.7697	0.985391	0.002115	15.39
gnl|UG|Ssc#S19548031	turquoise	1417	1416.15	0.976499	0.004309	32.576
gnl|UG|Ssc#S19548276	grey60	268.84	30.4199	0.979441	0.003528	36.648
gnl|UG|Ssc#S19548366	turquoise	1499.1	1498.52	0.990546	0.001102	2.838
gnl|UG|Ssc#S19548981	turquoise	1513.8	1513.25	0.992257	0.000817	272.796
gnl|UG|Ssc#S19549069	turquoise	1553.6	1553.18	0.996528	0.000245	3.024
gnl|UG|Ssc#S19549096	turquoise	1415.5	1414.98	0.973933	0.005032	2.786
gnl|UG|Ssc#S19549165	salmon	118.57	21.6111	0.908126	0.032965	57.038
gnl|UG|Ssc#S19549209	brown	243.82	121.124	0.981174	0.003092	30.982
gnl|UG|Ssc#S19549226	turquoise	1147.8	1146.31	0.928244	0.022824	11.266
gnl|UG|Ssc#S19549265	turquoise	1028.9	1026.17	0.901255	0.036691	10.746
gnl|UG|Ssc#S19549267	turquoise	151.45	132.872	0.461562	0.433912	5.616
gnl|UG|Ssc#S19549286	darkgreen	258.16	6.69102	0.696467	0.191341	9.692
gnl|UG|Ssc#S19549310	turquoise	1556.4	1555.93	0.997335	0.000165	7.09
gnl|UG|Ssc#S19550112	turquoise	1516	1515.49	0.992402	0.000794	0.836
gnl|UG|Ssc#S19550176	tan	331.63	39.2391	0.988178	0.00154	4.236

gnl|UG|Ssc#S19550257	brown	289.22	119.173	0.972913	0.00533	5.812
gnl|UG|Ssc#S19550604	lightyellow	305.12	25.0005	0.981892	0.002917	16.902
gnl|UG|Ssc#S20942207	green	274.09	31.4307	0.842799	0.07303	32.126
gnl|UG|Ssc#S20945025	magenta	331.49	43.3711	0.986928	0.001791	106.732
gnl|UG|Ssc#S20945959	turquoise	1543.5	1543.03	0.996514	0.000247	7.616
gnl|UG|Ssc#S20946380	greenyellow	175.64	16.0376	0.893193	0.041224	28.994
gnl|UG|Ssc#S20947929	darkgreen	266.79	23.9303	0.995329	0.000383	10.62
gnl|UG|Ssc#S20958918	turquoise	1553.6	1553.18	0.996528	0.000245	0.566
gnl|UG|Ssc#S21554547	grey	321.29	16.3765	0.946039	0.014925	24.986
gnl|UG|Ssc#S21557354	turquoise	1547.5	1547.14	0.995807	0.000326	3.728
gnl|UG|Ssc#S21575274	turquoise	1517.7	1517.34	0.99021	0.001161	4.914
gnl|UG|Ssc#S21579411	blue	341.33	150.581	0.988245	0.001527	33.182
gnl|UG|Ssc#S21579717	green	75.626	19.6113	0.730602	0.160897	0.838
gnl|UG|Ssc#S22271795	turquoise	175.55	140.64	0.406152	0.497463	29.64
gnl|UG|Ssc#S22272079	turquoise	64.214	39.0173	0.195695	0.752433	5.118
gnl|UG|Ssc#S22272334	turquoise	1277.9	1276.8	0.954864	0.011433	18.748
gnl|UG|Ssc#S22273127	blue	300.47	140.024	0.972695	0.005394	2.868
gnl|UG|Ssc#S22273263	blue	314.63	117.33	0.930908	0.021573	8.656
gnl|UG|Ssc#S22274163	turquoise	1012.2	1009.92	0.90191	0.036331	12.592
gnl|UG|Ssc#S22274609	turquoise	1216.4	1215.26	0.941228	0.016952	13.816
gnl|UG|Ssc#S22275000	brown	201.46	108.258	0.95502	0.011374	38.33
gnl|UG|Ssc#S22275082	turquoise	1489.5	1488.88	0.987154	0.001744	18.65
gnl|UG|Ssc#S22275237	blue	329.76	135.117	0.963067	0.008473	11.808
gnl|UG|Ssc#S22275831	lightyellow	241.74	20.9003	0.927347	0.02325	7.346
gnl|UG|Ssc#S22276105	turquoise	1539.4	1538.95	0.996081	0.000294	3.342
gnl|UG|Ssc#S22276551	turquoise	1003.7	1000.98	0.894906	0.040247	10.992
gnl|UG|Ssc#S22277516	brown	248.89	112.371	0.964227	0.008078	68.002
gnl|UG|Ssc#S22278253	midnightblue	209.59	32.302	0.971182	0.005847	9.656
gnl|UG|Ssc#S22278936	turquoise	1551.1	1550.77	0.996177	0.000284	7.758
gnl|UG|Ssc#S22279005	turquoise	1488.1	1487.69	0.984956	0.00221	1.938
gnl|UG|Ssc#S22279857	blue	236.52	108.322	0.913704	0.030034	66.396
gnl|UG|Ssc#S22280355	turquoise	1551.8	1551.33	0.997472	0.000153	9.862
gnl|UG|Ssc#S22281441	turquoise	1317.4	1316.16	0.959191	0.009835	283.24
gnl|UG|Ssc#S22282278	salmon	82.104	18.7829	0.859708	0.061734	1.822
gnl|UG|Ssc#S22282766	turquoise	1302.5	1301.3	0.959425	0.009751	8.684
gnl|UG|Ssc#S22282893	lightgreen	71.354	12.4689	0.924388	0.024674	25.89
gnl|UG|Ssc#S22283333	navy	273.06	28.4683	0.954208	0.011682	43.128
gnl|UG|Ssc#S22283855	turquoise	1546.1	1545.58	0.996236	0.000277	1.458
gnl|UG|Ssc#S22284475	turquoise	626.18	616.671	0.774209	0.124339	31.142
gnl|UG|Ssc#S22284504	green	97.754	27.9117	0.830892	0.081329	8.032
gnl|UG|Ssc#S22286222	purple	197.77	34.0036	0.982453	0.002783	71.734
gnl|UG|Ssc#S22287254	lightyellow	276.68	19.4563	0.933151	0.020539	86.922
gnl|UG|Ssc#S22287785	turquoise	1387.1	1386.57	0.967489	0.007002	0.566
gnl|UG|Ssc#S22287912	turquoise	1469.3	1468.9	0.981477	0.003018	5.694
gnl|UG|Ssc#S22288091	turquoise	1558.4	1557.97	0.997029	0.000194	4.982
gnl|UG|Ssc#S22289618	turquoise	1350.8	1349.74	0.966925	0.007185	11.776
gnl|UG|Ssc#S22290406	turquoise	1422.8	1422.18	0.977178	0.004125	14.64
gnl|UG|Ssc#S22300839	turquoise	1567.3	1566.95	0.99891	4.32E-05	14.152

gnl|UG|Ssc#S22311415	turquoise	1488.1	1487.46	0.988756	0.001429	12.798
gnl|UG|Ssc#S22313154	turquoise	1445.4	1444.69	0.982652	0.002736	602.086
gnl|UG|Ssc#S22314469	turquoise	1458.8	1458.3	0.97919	0.003592	5.808
gnl|UG|Ssc#S22315401	yellow	110.13	43.1498	0.819994	0.089161	12.998
gnl|UG|Ssc#S22316206	turquoise	1229.4	1227.98	0.941537	0.016819	49.502
gnl|UG|Ssc#S22316499	turquoise	1414.5	1413.71	0.974584	0.004845	3.914
gnl|UG|Ssc#S22317157	yellow	121.59	46.5707	0.814922	0.092881	22.84
gnl|UG|Ssc#S23213478	turquoise	1520.7	1520.36	0.990617	0.00109	2.078
gnl|UG|Ssc#S23689580	turquoise	1412.1	1411.41	0.974936	0.004745	6.576
gnl|UG|Ssc#S23689635	turquoise	1483.7	1483.28	0.984547	0.002301	8.492
gnl|UG|Ssc#S23689648	turquoise	1521.9	1521.45	0.993312	0.000656	9.13
gnl|UG|Ssc#S23689683	blue	332.97	151.67	0.989912	0.001214	12.634
gnl|UG|Ssc#S23689697	turquoise	1209.7	1208.41	0.938812	0.018001	3.042
gnl|UG|Ssc#S23689699	turquoise	1544.9	1544.5	0.994428	0.000499	2.5
gnl|UG|Ssc#S23689759	brown	261.66	124.035	0.985908	0.002004	7.258
gnl|UG|Ssc#S23689765	turquoise	1540.6	1540.13	0.995933	0.000311	24.946
gnl|UG|Ssc#S23689779	turquoise	1454.3	1453.83	0.983905	0.002445	2.99
gnl|UG|Ssc#S23689821	greenyellow	202.28	21.8044	0.988073	0.001561	19.002
gnl|UG|Ssc#S23689827	turquoise	72.812	50.7477	0.243435	0.693139	0.746
gnl|UG|Ssc#S23689835	turquoise	1548	1547.58	0.996272	0.000273	14.954
gnl|UG|Ssc#S23689840	green	109.74	25.286	0.802507	0.102178	46.58
gnl|UG|Ssc#S23689853	yellow	187.49	78.2152	0.977898	0.003931	25.24
gnl|UG|Ssc#S23689871	turquoise	687.59	679.418	0.798318	0.105375	8.03
gnl|UG|Ssc#S23689938	turquoise	1517.3	1516.78	0.993302	0.000657	6.154
gnl|UG|Ssc#S23690002	midnightblue	235.45	34.8204	0.986586	0.001861	4.228
gnl|UG|Ssc#S23690339	turquoise	1482	1481.53	0.987176	0.00174	22.506
gnl|UG|Ssc#S23690342	royalblue	183.81	20.6675	0.948049	0.014103	38.92
gnl|UG|Ssc#S23690384	brown	262.12	123.847	0.984375	0.002339	3.594
gnl|UG|Ssc#S23690435	turquoise	1553.6	1553.18	0.996528	0.000245	1.558
gnl|UG|Ssc#S23690458	turquoise	1467.9	1467.31	0.984504	0.00231	7.168
gnl|UG|Ssc#S23690475	grey60	286.46	28.3535	0.964123	0.008113	124.482
gnl|UG|Ssc#S23690513	turquoise	952.23	951.304	0.889282	0.043482	9.674
gnl|UG|Ssc#S23690535	blue	345.6	150.953	0.988874	0.001406	46.724
gnl|UG|Ssc#S23690544	turquoise	1408.6	1408.13	0.972301	0.005511	17.958
gnl|UG|Ssc#S23690802	turquoise	296.29	283.549	0.616303	0.26828	63.54
gnl|UG|Ssc#S23690807	darkgreen	263.36	23.8431	0.993766	0.00059	5.534
gnl|UG|Ssc#S23691069	blue	290.77	133.05	0.96158	0.008988	100.79
gnl|UG|Ssc#S23691229	turquoise	1522.3	1521.85	0.992565	0.000769	39.258
gnl|UG|Ssc#S23691231	turquoise	1553.6	1553.18	0.996528	0.000245	0.85
gnl|UG|Ssc#S23691372	turquoise	1550.2	1549.79	0.996487	0.00025	13.828
gnl|UG|Ssc#S23691718	salmon	84.307	20.1737	0.883203	0.047068	86.056
gnl|UG|Ssc#S23691825	turquoise	1553.6	1553.18	0.996528	0.000245	1.748
gnl|UG|Ssc#S23691900	darkred	88.897	8.88421	0.79788	0.105711	59.768
gnl|UG|Ssc#S23692548	turquoise	1526	1525.62	0.991567	0.000928	10.994
gnl|UG|Ssc#S23692648	brown	208	99.5219	0.933477	0.02039	10.962
gnl|UG|Ssc#S23692872	turquoise	702.26	697.534	0.81413	0.093466	37.794
gnl|UG|Ssc#S23693351	turquoise	1040.7	1038.86	0.897735	0.03865	20.092
gnl|UG|Ssc#S23693357	lightyellow	304.67	25.4215	0.993466	0.000633	97.828

gnl|UG|Ssc#S23693410	yellow	237.33	85.0757	0.998184	9.29E-05	137.158
gnl|UG|Ssc#S23693714	turquoise	1289.2	1287.89	0.955145	0.011327	108.168
gnl|UG|Ssc#S23693871	red	274.46	44.179	0.954219	0.011678	7.358
gnl|UG|Ssc#S23694531	turquoise	609.84	605.752	0.766788	0.130362	25.342
gnl|UG|Ssc#S23694886	turquoise	1524.3	1523.84	0.993398	0.000643	17.2
gnl|UG|Ssc#S23694918	turquoise	1529.6	1529.26	0.992875	0.000721	1.62
gnl|UG|Ssc#S23694970	greenyellow	98.431	11.484	0.820911	0.088494	70.49
gnl|UG|Ssc#S23695047	green	207.37	50.9954	0.973961	0.005024	25.232
gnl|UG|Ssc#S23695102	brown	244.2	102.195	0.934647	0.019858	55.258
gnl|UG|Ssc#S23695110	turquoise	1554.2	1553.82	0.997114	0.000186	10.436
gnl|UG|Ssc#S23695118	turquoise	1529	1528.54	0.994901	0.000437	9.66
gnl|UG|Ssc#S23695121	turquoise	643.35	640.304	0.800202	0.103934	5.8
gnl|UG|Ssc#S23695151	turquoise	687.05	679.216	0.79981	0.104233	3.52
gnl|UG|Ssc#S23695164	turquoise	1111.5	1109.9	0.923838	0.024941	153.54
gnl|UG|Ssc#S23695171	turquoise	1546.8	1546.41	0.994769	0.000454	8.014
gnl|UG|Ssc#S23695220	turquoise	1259.7	1258.62	0.946012	0.014936	3.636
gnl|UG|Ssc#S23695269	blue	305.45	129.673	0.954984	0.011387	17.374
gnl|UG|Ssc#S23695295	blue	352.37	131.41	0.956747	0.010728	37.582
gnl|UG|Ssc#S23695348	turquoise	1140	1137.91	0.925385	0.024191	9.872
gnl|UG|Ssc#S23695397	turquoise	1380.8	1380.3	0.968429	0.006702	15.602
gnl|UG|Ssc#S23695399	salmon	154.36	28.8736	0.973844	0.005058	16.986
gnl|UG|Ssc#S23695759	grey	104.19	7.49351	0.248813	0.686501	27.258
gnl|UG|Ssc#S23695762	turquoise	172.56	167.05	0.531567	0.356571	15.504
gnl|UG|Ssc#S23695768	turquoise	1476.1	1475.63	0.983776	0.002475	3.246
gnl|UG|Ssc#S23695825	turquoise	1552	1551.52	0.997588	0.000142	27.456
gnl|UG|Ssc#S23695896	turquoise	1512.9	1512.31	0.992604	0.000763	58.644
gnl|UG|Ssc#S23695912	turquoise	1523.1	1522.71	0.99144	0.000949	2.528
gnl|UG|Ssc#S23696008	blue	300.64	142.856	0.976288	0.004368	15.606
gnl|UG|Ssc#S23696033	turquoise	1425.9	1425.1	0.97825	0.003838	13.172
gnl|UG|Ssc#S23696561	turquoise	1541.7	1541.24	0.996515	0.000247	5.696
gnl|UG|Ssc#S23696616	brown	111.83	58.7664	0.825819	0.084948	127.484
gnl|UG|Ssc#S23696659	turquoise	1541.1	1540.66	0.995505	0.000362	11.444
gnl|UG|Ssc#S23696666	turquoise	1554.2	1553.78	0.997635	0.000138	20.81
gnl|UG|Ssc#S23696689	turquoise	1371.6	1370.56	0.968993	0.006524	39.612
gnl|UG|Ssc#S23696693	yellow	241.54	76.7197	0.960918	0.00922	21.644
gnl|UG|Ssc#S23696740	turquoise	123.49	82.5031	0.310902	0.61062	21.048
gnl|UG|Ssc#S23696826	turquoise	1272.8	1271.56	0.951009	0.012921	40.99
gnl|UG|Ssc#S23696828	turquoise	1542.5	1542.06	0.996286	0.000272	16.078
gnl|UG|Ssc#S23696834	turquoise	1552.5	1552.09	0.996524	0.000246	24.928
gnl|UG|Ssc#S23697317	turquoise	1549.6	1549.16	0.996361	0.000263	23.786
gnl|UG|Ssc#S23697383	turquoise	536.58	531.791	0.759105	0.136686	46.946
gnl|UG|Ssc#S23697414	turquoise	602.23	598.221	0.769913	0.127816	6.894
gnl|UG|Ssc#S23697660	turquoise	403.47	398.881	0.7117	0.177569	13.504
gnl|UG|Ssc#S23697932	blue	341.79	141.727	0.974843	0.004772	82.268
gnl|UG|Ssc#S23697971	turquoise	1405.8	1404.97	0.976429	0.004329	37.94
gnl|UG|Ssc#S23697986	turquoise	1554.6	1554.2	0.998136	9.66E-05	32.59
gnl|UG|Ssc#S23698009	turquoise	1520	1519.42	0.992651	0.000756	6.44
gnl|UG|Ssc#S23698058	turquoise	1228.8	1227.32	0.944689	0.015485	18.248

gnl|UG|Ssc#S23698110	turquoise	1309.2	1308.49	0.956183	0.010938	24.696
gnl|UG|Ssc#S23698294	turquoise	1549.4	1548.96	0.995243	0.000394	6.454
gnl|UG|Ssc#S23698716	lightyellow	253.84	16.8735	0.894914	0.040242	41.832
gnl|UG|Ssc#S23698749	turquoise	761.09	758.621	0.824653	0.085786	1.374
gnl|UG|Ssc#S23698752	turquoise	1504.7	1504.15	0.990978	0.001027	54.242
gnl|UG|Ssc#S23698843	blue	300.08	144.308	0.978575	0.003752	8.882
gnl|UG|Ssc#S23699327	turquoise	1571.7	1571.32	0.999808	3.20E-06	5.302
gnl|UG|Ssc#S23699362	turquoise	1250.3	1248.94	0.947081	0.014497	6.494
gnl|UG|Ssc#S23699367	turquoise	1513.1	1512.66	0.991862	0.00088	1.904
gnl|UG|Ssc#S23699463	turquoise	1550.4	1549.9	0.997646	0.000137	12.738
gnl|UG|Ssc#S23699748	greenyellow	75.84	5.75037	-0.50437	0.386187	5.416
gnl|UG|Ssc#S23699771	greenyellow	73.317	6.89755	-0.55132	0.335431	20.426
gnl|UG|Ssc#S23699793	turquoise	92.523	44.4906	0.21269	0.731251	21.336
gnl|UG|Ssc#S23699804	turquoise	1529.2	1528.71	0.99465	0.000469	15.036
gnl|UG|Ssc#S23699836	pink	212.77	46.8988	0.994194	0.000531	25.204
gnl|UG|Ssc#S23699859	turquoise	1547.7	1547.23	0.996041	0.000299	77.888
gnl|UG|Ssc#S23699860	turquoise	1429.3	1428.73	0.977225	0.004112	4.678
gnl|UG|Ssc#S23700002	yellow	239.99	79.2516	0.974642	0.004829	73.072
gnl|UG|Ssc#S23700108	purple	213.05	28.5153	0.938712	0.018045	5.782
gnl|UG|Ssc#S23700159	black	284.81	40.6938	0.973422	0.00518	59.348
gnl|UG|Ssc#S23700414	turquoise	1483.8	1483.28	0.985694	0.00205	5.298
gnl|UG|Ssc#S23700455	turquoise	1546	1545.57	0.996562	0.000242	18.534
gnl|UG|Ssc#S23700560	turquoise	1558.8	1558.43	0.997223	0.000176	3.54
gnl|UG|Ssc#S23701101	turquoise	1522.1	1521.72	0.990673	0.00108	11.02
gnl|UG|Ssc#S23701109	turquoise	1466.2	1465.65	0.98102	0.00313	51.358
gnl|UG|Ssc#S23701124	pink	146.11	32.4915	0.91481	0.029464	4.1
gnl|UG|Ssc#S23701146	darkred	72.975	9.23973	0.771491	0.126536	5.23
gnl|UG|Ssc#S23701149	turquoise	902.81	900.759	0.880393	0.048755	11.388
gnl|UG|Ssc#S23701211	turquoise	1489.9	1489.28	0.988874	0.001406	26.722
gnl|UG|Ssc#S23701220	turquoise	1553.6	1553.18	0.996528	0.000245	1.558
gnl|UG|Ssc#S23701295	turquoise	1374.1	1373.11	0.970373	0.006094	295.582
gnl|UG|Ssc#S23701300	blue	315.85	131.194	0.956716	0.01074	25.292
gnl|UG|Ssc#S23755031	yellow	163.28	69.234	0.938183	0.018278	24.904
gnl|UG|Ssc#S23755238	turquoise	1411.4	1410.63	0.976377	0.004343	8.354
gnl|UG|Ssc#S23755367	turquoise	1546	1545.58	0.99707	0.00019	28.528
gnl|UG|Ssc#S23755372	turquoise	90.848	65.2555	0.292927	0.632439	3.322
gnl|UG|Ssc#S23755373	royalblue	188.72	24.5365	0.982595	0.002749	12.974
gnl|UG|Ssc#S23755435	turquoise	1548.9	1548.5	0.995486	0.000364	270.42
gnl|UG|Ssc#S23755540	turquoise	933.99	931.667	0.87459	0.052298	42.98
gnl|UG|Ssc#S23755550	turquoise	310.29	300.186	0.61301	0.271587	40.802
gnl|UG|Ssc#S23755551	yellow	173.86	67.2629	0.928528	0.022689	62.498
gnl|UG|Ssc#S23755686	turquoise	1284.7	1283.98	0.949992	0.013323	2.626
gnl|UG|Ssc#S23756069	turquoise	1387.7	1386.84	0.973127	0.005267	5.316
gnl|UG|Ssc#S23756098	green	187.08	48.6243	0.981442	0.003026	84.108
gnl|UG|Ssc#S23756108	turquoise	1512.3	1511.88	0.991468	0.000945	7.604
gnl|UG|Ssc#S23756754	turquoise	432.54	413.644	0.670882	0.215112	16.35
gnl|UG|Ssc#S23756816	turquoise	1366	1365.13	0.96989	0.006243	11.58
gnl|UG|Ssc#S23756850	pink	262.88	45.5012	0.983669	0.002499	90.372

gnl|UG|Ssc#S23756925	turquoise	1343.4	1342.34	0.962322	0.00873	19.36
gnl|UG|Ssc#S23756992	yellow	143.83	55.9624	0.87227	0.053737	48.908
gnl|UG|Ssc#S23757023	turquoise	1557.6	1557.18	0.997461	0.000154	5.958
gnl|UG|Ssc#S23757412	turquoise	1538	1537.52	0.996016	0.000302	7.944
gnl|UG|Ssc#S23757521	lightyellow	287.9	23.7353	0.976264	0.004374	6.838
gnl|UG|Ssc#S23757528	turquoise	1529.6	1529.16	0.993541	0.000623	10.62
gnl|UG|Ssc#S23757556	tan	312.21	40.7333	0.997194	0.000178	21.012
gnl|UG|Ssc#S23757653	magenta	324.75	44.6007	0.994245	0.000524	14.442
gnl|UG|Ssc#S23757695	turquoise	249.05	227.754	0.53983	0.347687	44.472
gnl|UG|Ssc#S23757708	turquoise	1553.6	1553.18	0.996528	0.000245	0.708
gnl|UG|Ssc#S23758068	turquoise	1460.5	1460.01	0.98001	0.003383	8.088
gnl|UG|Ssc#S23758125	turquoise	1294.6	1293.57	0.958839	0.009962	220.986
gnl|UG|Ssc#S23758306	turquoise	1553.6	1553.18	0.996528	0.000245	1.322
gnl|UG|Ssc#S23758340	lightcyan	180.17	7.54151	0.333928	0.582869	7.666
gnl|UG|Ssc#S23758349	turquoise	294.49	267.196	0.562177	0.323945	24.84
gnl|UG|Ssc#S23758422	turquoise	123.49	106.71	0.404389	0.499515	75.588
gnl|UG|Ssc#S23758429	tan	324.52	38.1727	0.981253	0.003073	12.306
gnl|UG|Ssc#S23758705	black	269.94	40.603	0.9913	0.000973	75.552
gnl|UG|Ssc#S23758834	turquoise	1562.8	1562.4	0.998281	8.55E-05	1.936
gnl|UG|Ssc#S23758881	turquoise	1518.2	1517.75	0.992573	0.000767	32.7
gnl|UG|Ssc#S23758924	turquoise	104.36	36.7239	0.061578	0.921646	24.874
gnl|UG|Ssc#S23758929	turquoise	1557.4	1557.02	0.997272	0.000171	18.958
gnl|UG|Ssc#S23758940	turquoise	1565.2	1564.79	0.99915	2.97E-05	6.568
gnl|UG|Ssc#S23758955	green	112.75	15.8567	0.660761	0.224726	66.424
gnl|UG|Ssc#S23759053	turquoise	1373.2	1372.43	0.965606	0.007617	17.656
gnl|UG|Ssc#S23759508	purple	183.09	33.391	0.974987	0.004731	760.134
gnl|UG|Ssc#S23759611	turquoise	1521	1520.51	0.993196	0.000673	5.824
gnl|UG|Ssc#S23759640	lightgreen	75.793	7.63105	0.596654	0.288171	139.402
gnl|UG|Ssc#S23759685	blue	287.42	133.735	0.96223	0.008762	10.756
gnl|UG|Ssc#S23760026	turquoise	1380.4	1379.85	0.972315	0.005507	2.73
gnl|UG|Ssc#S23760234	turquoise	1514.2	1513.7	0.991709	0.000905	15.22
gnl|UG|Ssc#S23760326	turquoise	1540.8	1540.42	0.995174	0.000402	6.37
gnl|UG|Ssc#S23760463	turquoise	281.07	263.025	0.575084	0.310427	153.976
gnl|UG|Ssc#S23760465	yellow	284.99	45.2903	0.792453	0.109901	26.09
gnl|UG|Ssc#S23761029	turquoise	1570	1569.58	0.999878	1.63E-06	4.124
gnl|UG|Ssc#S23761814	turquoise	119.41	87.9102	0.328048	0.589933	16.884
gnl|UG|Ssc#S23761842	turquoise	1488.1	1487.69	0.985586	0.002073	5.674
gnl|UG|Ssc#S23762266	turquoise	1066.9	1064.7	0.911117	0.031382	35.246
gnl|UG|Ssc#S23762445	turquoise	1528.1	1527.68	0.993774	0.000589	2.546
gnl|UG|Ssc#S23762471	blue	318.37	146.582	0.982169	0.002851	18.91
gnl|UG|Ssc#S23762560	turquoise	208.79	149.378	0.387311	0.519483	41.082
gnl|UG|Ssc#S23762736	pink	230.88	45.3634	0.986935	0.001789	23.27
gnl|UG|Ssc#S23762759	grey	284.78	13.9716	0.886995	0.044821	26.69
gnl|UG|Ssc#S23762936	turquoise	1551.4	1550.98	0.996835	0.000214	19.358
gnl|UG|Ssc#S23762974	black	267.16	39.1482	0.969798	0.006272	43.358
gnl|UG|Ssc#S23763002	turquoise	1150.3	1149.18	0.933327	0.020458	2.578
gnl|UG|Ssc#S23763174	turquoise	1397.3	1396.84	0.973174	0.005253	7.034
gnl|UG|Ssc#S23763192	red	164.36	42.7975	0.952899	0.012184	4.118

gnl|UG|Ssc#S23763197	turquoise	1503.3	1502.84	0.988426	0.001492	2.25
gnl|UG|Ssc#S23763649	black	245.51	37.4943	0.970263	0.006128	64.192
gnl|UG|Ssc#S23763725	turquoise	1417.7	1416.82	0.978238	0.003841	10.04
gnl|UG|Ssc#S23763835	turquoise	134.41	109.705	0.397212	0.507887	21.988
gnl|UG|Ssc#S23763873	turquoise	1517.5	1517.04	0.98963	0.001266	10.5
gnl|UG|Ssc#S23764031	turquoise	1553.6	1553.18	0.996528	0.000245	0.944
gnl|UG|Ssc#S23764110	blue	306.53	126.573	0.950483	0.013128	9.292
gnl|UG|Ssc#S23764143	turquoise	1428.9	1428.36	0.977765	0.003967	3.608
gnl|UG|Ssc#S23764348	turquoise	1532.6	1532.2	0.993566	0.000619	2.666
gnl|UG|Ssc#S23764997	turquoise	1534.9	1534.52	0.994066	0.000548	12.444
gnl|UG|Ssc#S23765215	turquoise	1458.8	1458.04	0.983778	0.002474	26.722
gnl|UG|Ssc#S23765368	turquoise	1523.2	1522.8	0.991891	0.000876	2.898
gnl|UG|Ssc#S23765833	turquoise	1553.6	1553.18	0.996528	0.000245	9.92
gnl|UG|Ssc#S23765932	lightcyan	280.04	26.916	0.995814	0.000325	74.18
gnl|UG|Ssc#S23765987	grey60	186	28.4214	0.965346	0.007704	102.008
gnl|UG|Ssc#S23766263	turquoise	1553.6	1553.18	0.996528	0.000245	0.33
gnl|UG|Ssc#S23766271	turquoise	1511.4	1510.96	0.991133	0.001001	2.416
gnl|UG|Ssc#S23766410	turquoise	1555.7	1555.24	0.997873	0.000118	3.596
gnl|UG|Ssc#S23766475	turquoise	1483.6	1483.2	0.984038	0.002415	20.086
gnl|UG|Ssc#S23766507	blue	300.14	137.476	0.967902	0.00687	37.052
gnl|UG|Ssc#S23766544	turquoise	1479.9	1479.52	0.983518	0.002534	8.756
gnl|UG|Ssc#S23766579	blue	241.2	110.587	0.918852	0.027409	44.594
gnl|UG|Ssc#S23766637	pink	226.77	47.8757	0.998756	5.27E-05	182.242
gnl|UG|Ssc#S23766692	turquoise	777.42	772.828	0.836339	0.077498	40.002
gnl|UG|Ssc#S23767117	yellow	250.96	61.3493	0.912337	0.030744	6.668
gnl|UG|Ssc#S23767489	turquoise	1507.2	1506.81	0.990873	0.001045	2.32
gnl|UG|Ssc#S23767637	turquoise	907.76	906.343	0.881482	0.048099	41.522
gnl|UG|Ssc#S23768000	magenta	254.2	25.6925	0.876729	0.050983	23.624
gnl|UG|Ssc#S23768028	turquoise	341.75	337.597	0.666755	0.219019	28.14
gnl|UG|Ssc#S23768102	brown	240.29	118.84	0.978979	0.003647	8.276
gnl|UG|Ssc#S23768212	blue	271.63	113.047	0.923614	0.02505	168.706
gnl|UG|Ssc#S23768340	turquoise	1530.1	1529.76	0.992247	0.000818	1.7
gnl|UG|Ssc#S23768398	turquoise	1481.9	1481.25	0.986736	0.00183	11.218
gnl|UG|Ssc#S23768479	turquoise	562.61	555.092	0.758249	0.137396	14.758
gnl|UG|Ssc#S23768511	turquoise	1527.6	1527.04	0.994349	0.000509	3.744
gnl|UG|Ssc#S23768582	blue	304.93	151.705	0.98991	0.001215	90.626
gnl|UG|Ssc#S23769110	black	262.71	40.1533	0.973332	0.005207	59.774
gnl|UG|Ssc#S23769348	turquoise	623.06	619.48	0.783406	0.116994	1.636
gnl|UG|Ssc#S23769376	turquoise	1093.8	1092.09	0.910507	0.031703	9.378
gnl|UG|Ssc#S23769437	yellow	213.63	62.4473	0.917258	0.028213	43.874
gnl|UG|Ssc#S23770014	turquoise	1433.4	1432.99	0.977394	0.004066	12.132
gnl|UG|Ssc#S23770448	lightyellow	186.09	15.241	0.850283	0.067958	12.094
gnl|UG|Ssc#S23770611	turquoise	1521.2	1520.83	0.990606	0.001091	20.206
gnl|UG|Ssc#S23770901	turquoise	1374.6	1373.68	0.9692	0.006459	8.078
gnl|UG|Ssc#S23770961	yellow	236.71	83.9327	0.993808	0.000584	55.904
gnl|UG|Ssc#S23771071	magenta	338.74	42.404	0.983997	0.002424	6.962
gnl|UG|Ssc#S23771394	turquoise	1505.2	1504.63	0.988876	0.001406	9.4
gnl|UG|Ssc#S23771589	darkred	116.07	6.45804	0.697123	0.190741	4.62

gnl|UG|Ssc#S23771863	brown	127.62	70.97	0.846523	0.070492	1.346
gnl|UG|Ssc#S23772025	turquoise	1437.2	1436.48	0.980915	0.003156	3.456
gnl|UG|Ssc#S23772152	turquoise	660.73	657.239	0.805395	0.099992	36.69
gnl|UG|Ssc#S23772206	turquoise	1554.1	1553.65	0.99798	0.000109	25.116
gnl|UG|Ssc#S23772402	grey60	232.13	31.265	0.985849	0.002017	142.368
gnl|UG|Ssc#S23772512	turquoise	1518.3	1517.85	0.991475	0.000944	4.574
gnl|UG|Ssc#S23775145	lightyellow	282.21	24.1293	0.968573	0.006656	47.506
gnl|UG|Ssc#S23775239	turquoise	1381.4	1380.84	0.966797	0.007226	7.514
gnl|UG|Ssc#S23775247	salmon	190	30.8751	0.993394	0.000644	5553.974
gnl|UG|Ssc#S23775351	darkgreen	313.21	22.8606	0.99247	0.000784	21.824
gnl|UG|Ssc#S23775370	turquoise	1341.2	1340.41	0.963137	0.008449	34.172
gnl|UG|Ssc#S23775512	purple	203.55	32.5772	0.96745	0.007015	3.366
gnl|UG|Ssc#S23775545	lightcyan	285.78	27.4339	0.997157	0.000182	10.436
gnl|UG|Ssc#S23775557	lightcyan	287.18	27.4116	0.993435	0.000638	98.962
gnl|UG|Ssc#S23775889	black	81.294	9.06583	0.493229	0.398485	12.058
gnl|UG|Ssc#S23775891	grey	64.953	1.08643	-0.18188	0.769706	37.4
gnl|UG|Ssc#S23776294	turquoise	1516.6	1516.18	0.990859	0.001048	11.824
gnl|UG|Ssc#S23776646	turquoise	850.88	848.775	0.847213	0.070025	14.986
gnl|UG|Ssc#S23776684	turquoise	1366.2	1365.67	0.965015	0.007814	2.618
gnl|UG|Ssc#S23776700	turquoise	906.25	903.988	0.875187	0.05193	9.786
gnl|UG|Ssc#S23776942	turquoise	1483.7	1483.33	0.984274	0.002362	1.796
gnl|UG|Ssc#S24609641	midnightblue	249.94	36.4315	0.995542	0.000357	5.794
gnl|UG|Ssc#S24610008	turquoise	1315.6	1314.61	0.958673	0.010023	19.336
gnl|UG|Ssc#S24610239	turquoise	1340.2	1339.56	0.960161	0.009488	2.07
gnl|UG|Ssc#S24610495	turquoise	1530.4	1529.99	0.992929	0.000713	16.296
gnl|UG|Ssc#S25135543	black	279.1	37.5007	0.940245	0.017377	8.69
gnl|UG|Ssc#S25135664	yellow	96.962	38.2788	0.77296	0.125347	81.882
gnl|UG|Ssc#S25135824	black	257.2	38.1567	0.978029	0.003896	26.824
gnl|UG|Ssc#S26389653	turquoise	1560.6	1560.18	0.998024	0.000105	2.386
gnl|UG|Ssc#S26391783	turquoise	1203.2	1202.11	0.932855	0.020674	6.464
gnl|UG|Ssc#S26391884	turquoise	1549	1548.55	0.997489	0.000151	2.928
gnl|UG|Ssc#S26392586	blue	266.58	114.366	0.92629	0.023755	2.774
gnl|UG|Ssc#S26392759	turquoise	1560	1559.58	0.997751	0.000128	9.43
gnl|UG|Ssc#S26394012	turquoise	1436.4	1435.93	0.981256	0.003072	4.584
gnl|UG|Ssc#S26395675	green	258.95	46.9326	0.949668	0.013452	49.14
gnl|UG|Ssc#S26396016	turquoise	1541.5	1541.07	0.995189	0.0004	1.874
gnl|UG|Ssc#S26396713	turquoise	1304	1302.7	0.957269	0.010535	55.31
gnl|UG|Ssc#S26399010	turquoise	1512	1511.54	0.988623	0.001454	15.856
gnl|UG|Ssc#S26399811	grey60	190.76	27.5347	0.959673	0.009662	66.138
gnl|UG|Ssc#S26400047	turquoise	1559.4	1558.96	0.998413	7.59E-05	6.732
gnl|UG|Ssc#S26400566	turquoise	1416	1415.53	0.973466	0.005168	4.572
gnl|UG|Ssc#S26401286	turquoise	1367.8	1367.26	0.965458	0.007667	5.056
gnl|UG|Ssc#S26401464	turquoise	329.29	305.546	0.597819	0.286981	12.884
gnl|UG|Ssc#S26403713	tan	350.53	35.1589	0.963208	0.008425	19.344
gnl|UG|Ssc#S26403765	turquoise	1556.1	1555.77	0.996859	0.000211	5.164
gnl|UG|Ssc#S26404602	turquoise	1540.9	1540.48	0.99617	0.000284	2.19
gnl|UG|Ssc#S26408370	turquoise	1491.1	1490.62	0.988181	0.00154	6.312
gnl|UG|Ssc#S26645293	turquoise	1477.3	1476.84	0.983044	0.002644	6.492

gnl|UG|Ssc#S26646909	turquoise	1547.4	1547.03	0.99538	0.000377	7.254
gnl|UG|Ssc#S26647414	turquoise	1547.4	1547.02	0.996736	0.000224	3.07
gnl|UG|Ssc#S26648597	turquoise	1436.7	1436.21	0.977347	0.004079	4.73
gnl|UG|Ssc#S26649787	turquoise	1404.7	1404.1	0.973714	0.005096	5.916
gnl|UG|Ssc#S26649927	turquoise	820.88	817.338	0.852411	0.066536	27.818
gnl|UG|Ssc#S26650236	blue	257.73	125.992	0.947595	0.014287	20.82
gnl|UG|Ssc#S26651687	turquoise	1491.1	1490.51	0.988701	0.001439	6.452
gnl|UG|Ssc#S26651783	greenyellow	201.68	22.6241	0.993357	0.000649	20.654
gnl|UG|Ssc#S26652967	yellow	106.82	39.6207	0.775546	0.123263	81.092
gnl|UG|Ssc#S26710759	darkred	78.442	13.4175	0.964323	0.008046	142.93
gnl|UG|Ssc#S26711201	turquoise	1439.9	1439.4	0.977856	0.003943	6.676
gnl|UG|Ssc#S26711451	turquoise	1555.7	1555.36	0.996875	0.00021	10.348
gnl|UG|Ssc#S26711650	red	131.09	35.1865	0.908564	0.032731	13.95
gnl|UG|Ssc#S26712023	purple	76.214	5.65291	0.532425	0.355647	1.612
gnl|UG|Ssc#S26712602	turquoise	1384.4	1383.74	0.969252	0.006442	6.27
gnl|UG|Ssc#S26713657	lightcyan	276.16	25.1731	0.971752	0.005675	76.518
gnl|UG|Ssc#S26713880	brown	250.37	120.985	0.978894	0.003669	0.274
gnl|UG|Ssc#S26713936	lightcyan	232.91	20.7423	0.941403	0.016877	38.906
gnl|UG|Ssc#S26714423	yellow	264.25	80.4693	0.978054	0.00389	110.182
gnl|UG|Ssc#S26714441	black	170.8	24.3766	0.853083	0.066089	150.346
gnl|UG|Ssc#S26714791	turquoise	332.46	311.624	0.603507	0.28119	57.954
gnl|UG|Ssc#S26714922	turquoise	1023.3	1021.21	0.900724	0.036985	29.472
gnl|UG|Ssc#S26715181	turquoise	1553.6	1553.18	0.996528	0.000245	1.322
gnl|UG|Ssc#S26715223	turquoise	1522.1	1521.66	0.990536	0.001104	28.558
gnl|UG|Ssc#S26715792	turquoise	1544.8	1544.38	0.995087	0.000413	18.172
gnl|UG|Ssc#S26716026	turquoise	1565.8	1565.36	0.999276	2.34E-05	66.78
gnl|UG|Ssc#S26716503	turquoise	1504.9	1504.44	0.988368	0.001503	13.682
gnl|UG|Ssc#S26717032	turquoise	1518.3	1517.91	0.991504	0.000939	36.452
gnl|UG|Ssc#S26717691	turquoise	1503.3	1502.67	0.990253	0.001154	17.356
gnl|UG|Ssc#S26718109	blue	306.5	143.444	0.977543	0.004026	20.786
gnl|UG|Ssc#S26718597	red	143.36	35.2945	0.911971	0.030935	115.48
gnl|UG|Ssc#S26719135	turquoise	1568.6	1568.17	0.999002	3.78E-05	17.196
gnl|UG|Ssc#S26719371	salmon	194.77	30.2682	0.989073	0.001369	130.224
gnl|UG|Ssc#S26719435	black	281.12	42.4184	0.985585	0.002073	168.174
gnl|UG|Ssc#S26719940	turquoise	1450.7	1450.25	0.980072	0.003367	2.71
gnl|UG|Ssc#S26720320	turquoise	1130.3	1128.16	0.922431	0.02563	10.528
gnl|UG|Ssc#S26720420	turquoise	905.25	903.348	0.868204	0.056287	74.562
gnl|UG|Ssc#S26720426	tan	292.64	31.2963	0.938335	0.018211	87.396
gnl|UG|Ssc#S26720448	turquoise	1301.6	1300.31	0.956257	0.01091	1.716
gnl|UG|Ssc#S26720534	turquoise	1530.6	1530.13	0.994395	0.000503	26.336
gnl|UG|Ssc#S26720557	brown	144.49	42.6439	0.761633	0.134596	62.746
gnl|UG|Ssc#S26720630	blue	155.16	68.9595	0.815834	0.092209	32.986
gnl|UG|Ssc#S26720632	turquoise	1165.9	1164.13	0.930809	0.02162	20.02
gnl|UG|Ssc#S26720841	red	279.26	41.8389	0.941503	0.016834	22.08
gnl|UG|Ssc#S26720873	turquoise	1433.8	1433.34	0.977533	0.004029	12.776
gnl|UG|Ssc#S26721025	turquoise	1057.2	1056.56	0.91308	0.030358	46.608
gnl|UG|Ssc#S26721293	turquoise	1546.8	1546.38	0.995835	0.000323	4.22
gnl|UG|Ssc#S26721534	turquoise	806.78	805.705	0.856615	0.063756	40.576

gnl|UG|Ssc#S26721796	turquoise	1047.7	1045.47	0.904219	0.035068	4.884
gnl|UG|Ssc#S26721858	turquoise	1478.8	1478.33	0.983922	0.002441	6.936
gnl|UG|Ssc#S26722221	blue	280.66	136.27	0.965882	0.007526	15.108
gnl|UG|Ssc#S26722332	turquoise	1448.2	1447.73	0.979677	0.003467	6.4
gnl|UG|Ssc#S26722436	turquoise	1571.6	1571.17	0.999811	3.12E-06	2.016
gnl|UG|Ssc#S26722459	turquoise	1098.5	1096.62	0.921095	0.026289	29.068
gnl|UG|Ssc#S26722558	brown	164.32	92.1783	0.910356	0.031782	54.132
gnl|UG|Ssc#S26722585	turquoise	1314.6	1313.89	0.955441	0.011215	2.158
gnl|UG|Ssc#S26722922	salmon	89.413	21.6147	0.898365	0.038297	23.772
gnl|UG|Ssc#S26723327	salmon	219.02	24.7123	0.922279	0.025705	30.278
gnl|UG|Ssc#S26723763	yellow	301.08	65.7917	0.909374	0.032301	52.528
gnl|UG|Ssc#S26723792	blue	308.6	142.471	0.976433	0.004327	2.25
gnl|UG|Ssc#S26723882	turquoise	888.97	884.364	0.863083	0.059552	10.45
gnl|UG|Ssc#S26724210	turquoise	1553.6	1553.18	0.996528	0.000245	0.708
gnl|UG|Ssc#S26724539	turquoise	878.02	876.812	0.873259	0.053122	5.896
gnl|UG|Ssc#S26724557	turquoise	1239.1	1238.31	0.939716	0.017606	6.12
gnl|UG|Ssc#S26724832	purple	191.47	25.3731	0.903497	0.035462	58.118
gnl|UG|Ssc#S26725357	turquoise	1197.7	1197.06	0.936797	0.018892	3.82
gnl|UG|Ssc#S26725722	red	205.86	51.7625	0.995205	0.000398	10.256
gnl|UG|Ssc#S26725945	red	243.29	51.7878	0.994299	0.000516	5.24
gnl|UG|Ssc#S26726115	turquoise	1260.6	1259.33	0.951486	0.012733	22.592
gnl|UG|Ssc#S26726120	magenta	316.27	43.7088	0.99066	0.001082	23.66
gnl|UG|Ssc#S26726211	turquoise	618.01	615.788	0.781091	0.11883	4.042
gnl|UG|Ssc#S26726574	turquoise	1490	1489.39	0.989391	0.00131	7.502
gnl|UG|Ssc#S26726837	turquoise	1197.6	1196.81	0.940188	0.017401	15.948
gnl|UG|Ssc#S26727063	turquoise	352.09	343.101	0.643484	0.241402	9.142
gnl|UG|Ssc#S26727199	royalblue	256.63	22.4139	0.957708	0.010374	17.986
gnl|UG|Ssc#S26727288	turquoise	1544.4	1543.92	0.996734	0.000224	9.754
gnl|UG|Ssc#S26727906	turquoise	1147.4	1146.4	0.920851	0.026411	7.39
gnl|UG|Ssc#S26728186	turquoise	1495.1	1494.67	0.986069	0.00197	9.43
gnl|UG|Ssc#S26728677	turquoise	1566.3	1565.9	0.999023	3.67E-05	3.754
gnl|UG|Ssc#S26728690	grey60	266.3	25.2942	0.938474	0.01815	44.726
gnl|UG|Ssc#S26728759	pink	263.47	43.6995	0.974253	0.00494	61.292
gnl|UG|Ssc#S26728785	turquoise	1545.2	1544.69	0.996431	0.000256	4.524
gnl|UG|Ssc#S26728925	turquoise	1513.1	1512.61	0.989679	0.001257	8.666
gnl|UG|Ssc#S26729898	turquoise	1486.3	1485.83	0.985033	0.002193	8.908
gnl|UG|Ssc#S26731491	brown	129.41	48.1705	0.795018	0.107915	49.368
gnl|UG|Ssc#S26732528	turquoise	1563.8	1563.38	0.999063	3.44E-05	3.274
gnl|UG|Ssc#S26732732	turquoise	1345.2	1344.63	0.95929	0.0098	6.01
gnl|UG|Ssc#S26732866	turquoise	1300.8	1300.1	0.952308	0.012413	5.46
gnl|UG|Ssc#S26733049	greenyellow	100.35	7.15476	-0.21345	0.730306	226.518
gnl|UG|Ssc#S26733269	turquoise	202.83	195.971	0.545865	0.341235	95.154
gnl|UG|Ssc#S26733295	turquoise	110.47	84.9428	0.322345	0.596801	7.47
gnl|UG|Ssc#S26733681	turquoise	1510.3	1509.8	0.989738	0.001246	9.66
gnl|UG|Ssc#S26733687	darkred	110.47	8.88245	0.733526	0.15836	50.884
gnl|UG|Ssc#S26733997	turquoise	1547.9	1547.43	0.996721	0.000225	16.284
gnl|UG|Ssc#S26734006	blue	311.49	143.946	0.977925	0.003924	7.596
gnl|UG|Ssc#S26734160	turquoise	1509.1	1508.69	0.989051	0.001373	4.032

gnl|UG|Ssc#S26734197	grey60	277.3	28.5433	0.964551	0.007969	13.898
gnl|UG|Ssc#S26734209	red	252.35	45.3417	0.961515	0.00901	1.052
gnl|UG|Ssc#S26734441	black	295.52	39.5416	0.962973	0.008505	66.006
gnl|UG|Ssc#S26734833	brown	226.19	119.093	0.978346	0.003813	20.596
gnl|UG|Ssc#S26735228	turquoise	1479.7	1479.1	0.98623	0.001936	11.24
gnl|UG|Ssc#S26735265	turquoise	339.99	312.361	0.59751	0.287297	32.958
gnl|UG|Ssc#S26735315	turquoise	945.97	944.34	0.875937	0.051469	2.312
gnl|UG|Ssc#S26735434	darkred	63.287	6.46721	0.697583	0.190322	30.526
gnl|UG|Ssc#S26735527	turquoise	1558.8	1558.46	0.997092	0.000188	15.098
gnl|UG|Ssc#S27598964	turquoise	1438.9	1438.49	0.978608	0.003744	5.868
gnl|UG|Ssc#S27599980	turquoise	1212.5	1211.35	0.934547	0.019903	82.618
gnl|UG|Ssc#S27600018	magenta	333.47	41.1639	0.975897	0.004476	125.624
gnl|UG|Ssc#S27600466	turquoise	225.75	217.191	0.555604	0.330884	36.17
gnl|UG|Ssc#S27600517	turquoise	925.36	922.612	0.882409	0.047542	74.128
gnl|UG|Ssc#S27600609	turquoise	1558.6	1558.21	0.997672	0.000135	3.232
gnl|UG|Ssc#S27600696	turquoise	1571	1570.59	0.999583	1.02E-05	1.454
gnl|UG|Ssc#S27600966	green	239.19	51.3701	0.983492	0.00254	18.336
gnl|UG|Ssc#S27601215	turquoise	1197.7	1196.52	0.931314	0.021385	31.94
gnl|UG|Ssc#S27601831	turquoise	1502.4	1501.87	0.989181	0.001349	8.102
gnl|UG|Ssc#S27602031	turquoise	550.24	549.283	0.772205	0.125958	7.366
gnl|UG|Ssc#S27602037	turquoise	161.37	139.041	0.464779	0.430281	7.414
gnl|UG|Ssc#S27602393	blue	271.51	130.113	0.955093	0.011347	18.716
gnl|UG|Ssc#S27602398	turquoise	1237.8	1236.88	0.938624	0.018084	12.972
gnl|UG|Ssc#S27602719	turquoise	1569.6	1569.24	0.999477	1.43E-05	8.17
gnl|UG|Ssc#S27603973	blue	326.73	137.206	0.966566	0.007302	7.908
gnl|UG|Ssc#S29456965	navy	276.76	31.1763	0.975514	0.004583	77.176
gnl|UG|Ssc#S29459150	brown	158.41	85.2331	0.903458	0.035483	157.06
gnl|UG|Ssc#S29460541	blue	307.18	137.02	0.967849	0.006887	10.594
gnl|UG|Ssc#S29462637	red	176.14	46.6156	0.972036	0.00559	5.2
gnl|UG|Ssc#S29463694	blue	286.49	131.776	0.958622	0.010041	2.074
gnl|UG|Ssc#S29465510	yellow	216.05	58.5517	0.894129	0.040689	1660.374
gnl|UG|Ssc#S29485097	turquoise	1411	1410.33	0.975059	0.004711	3.934
gnl|UG|Ssc#S29966064	blue	318.5	155.842	0.996066	0.000296	28.778
gnl|UG|Ssc#S29970127	turquoise	1422.5	1421.97	0.973191	0.005248	5.04
gnl|UG|Ssc#S29971166	turquoise	1406.8	1406.31	0.971696	0.005692	2.064
gnl|UG|Ssc#S29971491	turquoise	1562.4	1561.99	0.998975	3.94E-05	14.002
gnl|UG|Ssc#S29971660	turquoise	1289.1	1288.04	0.957757	0.010356	4.056
gnl|UG|Ssc#S29974861	black	286.12	43.1309	0.99202	0.000855	75.452
gnl|UG|Ssc#S29976380	turquoise	1451.3	1450.73	0.981593	0.002989	8.624
gnl|UG|Ssc#S29977062	turquoise	1549.4	1548.96	0.996862	0.000211	1.738
gnl|UG|Ssc#S29977171	turquoise	1378.4	1377.8	0.970046	0.006195	6.364
gnl|UG|Ssc#S29977753	turquoise	1360	1359.42	0.961659	0.00896	2.692
gnl|UG|Ssc#S29978365	green	213.41	50.0105	0.960232	0.009463	41.58
gnl|UG|Ssc#S29978506	turquoise	467.24	462.542	0.709568	0.179478	15.448
gnl|UG|Ssc#S29978907	turquoise	120.49	103.416	0.416429	0.485536	4.124
gnl|UG|Ssc#S29979056	green	108.59	23.2325	0.774336	0.124237	0.862
gnl|UG|Ssc#S29979252	turquoise	780.3	778.614	0.840801	0.074403	5.192
gnl|UG|Ssc#S29979418	blue	348.55	149.793	0.986762	0.001825	3.164

gnl|UG|Ssc#S29979787	brown	261.43	123.45	0.985471	0.002098	5.1
gnl|UG|Ssc#S29980298	darkgreen	277.63	23.8381	0.996483	0.00025	5.532
gnl|UG|Ssc#S29982058	turquoise	1321.4	1320.62	0.955099	0.011344	17.878
gnl|UG|Ssc#S29982561	purple	230.29	34.7508	0.988248	0.001527	12.564
gnl|UG|Ssc#S29983177	turquoise	1557	1556.58	0.997236	0.000174	5.682
gnl|UG|Ssc#S29983540	turquoise	1045.1	1043.21	0.904088	0.035139	7.08
gnl|UG|Ssc#S29984324	turquoise	1448.7	1448.25	0.97966	0.003472	126.372
gnl|UG|Ssc#S29987248	turquoise	1553.5	1553.1	0.996054	0.000297	2.728
gnl|UG|Ssc#S29987869	turquoise	1558.5	1558.13	0.997769	0.000126	9.03
gnl|UG|Ssc#S29988897	grey	101.85	1.35563	-0.42255	0.478459	7.332
gnl|UG|Ssc#S29989732	pink	243.23	47.6893	0.996454	0.000253	86.912
gnl|UG|Ssc#S29989765	turquoise	1168.1	1166.82	0.925046	0.024354	13.266
gnl|UG|Ssc#S29989999	royalblue	182.15	25.234	0.987317	0.001711	22.006
gnl|UG|Ssc#S29990179	green	261.76	49.1025	0.953117	0.0121	47.588
gnl|UG|Ssc#S29990650	turquoise	1369	1368.13	0.968892	0.006556	55.188
gnl|UG|Ssc#S31101544	midnightblue	228.85	35.0964	0.98779	0.001617	112.296
gnl|UG|Ssc#S31101691	darkgreen	229.78	21.2356	0.964636	0.007941	51.938
gnl|UG|Ssc#S31101791	blue	257.22	123.614	0.942835	0.016266	2.456
gnl|UG|Ssc#S31101896	navy	292.06	27.079	0.934934	0.019728	133.604
gnl|UG|Ssc#S31101927	turquoise	855.95	852.547	0.862287	0.060064	35.5
gnl|UG|Ssc#S31102082	turquoise	1553.6	1553.18	0.996528	0.000245	1.89
gnl|UG|Ssc#S31102306	grey	57.626	9.24731	0.038775	0.950643	22.976
gnl|UG|Ssc#S31102648	turquoise	51.3	37.7679	0.232483	0.706681	4.084
gnl|UG|Ssc#S31102731	black	71.95	7.54356	0.445963	0.451609	31.718
gnl|UG|Ssc#S31102746	magenta	291.38	38.8082	0.965285	0.007724	43.52
gnl|UG|Ssc#S31103357	turquoise	1553.6	1553.18	0.996528	0.000245	4.298
gnl|UG|Ssc#S31103552	turquoise	1458.7	1458.24	0.981383	0.003041	1.034
gnl|UG|Ssc#S31104316	turquoise	1554.8	1554.36	0.99761	0.00014	4.41
gnl|UG|Ssc#S31106641	blue	330.78	157.86	0.999069	3.41E-05	30.844
gnl|UG|Ssc#S31107148	magenta	315.8	35.7469	0.947077	0.014499	12.076
gnl|UG|Ssc#S31107257	turquoise	1446.7	1446.2	0.977903	0.00393	0.708
gnl|UG|Ssc#S31107474	turquoise	1191.8	1190.33	0.938634	0.01808	12.43
gnl|UG|Ssc#S31107757	black	222.47	31.4042	0.89825	0.038362	18.09
gnl|UG|Ssc#S31111249	turquoise	1294.3	1293.35	0.958637	0.010035	4.96
gnl|UG|Ssc#S31111456	turquoise	1553.5	1553.12	0.996253	0.000275	9.676
gnl|UG|Ssc#S31111777	greenyellow	210.12	21.531	0.971632	0.005711	13.826
gnl|UG|Ssc#S31112392	turquoise	1464.5	1463.84	0.984987	0.002203	6.066
gnl|UG|Ssc#S31113730	turquoise	996.92	994.996	0.899079	0.037899	2.552
gnl|UG|Ssc#S31113853	purple	215.67	34.18	0.983975	0.002429	30.224
gnl|UG|Ssc#S31114178	turquoise	1436.4	1435.79	0.975667	0.00454	37.472
gnl|UG|Ssc#S31114330	black	292.69	40.3012	0.97903	0.003634	10.918
gnl|UG|Ssc#S31114548	turquoise	1552.8	1552.42	0.9961	0.000292	1.832
gnl|UG|Ssc#S31114563	turquoise	1456.6	1456.1	0.979581	0.003492	5.36
gnl|UG|Ssc#S31114631	turquoise	1553.6	1553.18	0.996528	0.000245	1.322
gnl|UG|Ssc#S31114666	tan	335.09	37.3962	0.977222	0.004113	32.828
gnl|UG|Ssc#S31114805	turquoise	1456.9	1456.16	0.983296	0.002585	2.6
gnl|UG|Ssc#S31114839	turquoise	1531	1530.53	0.994717	0.000461	1.808
gnl|UG|Ssc#S31115237	turquoise	1536	1535.47	0.995608	0.000349	1.312

gnl|UG|Ssc#S31115449	turquoise	1411.5	1410.75	0.975625	0.004552	2.336
gnl|UG|Ssc#S31115737	turquoise	1553.6	1553.18	0.996528	0.000245	0.426
gnl|UG|Ssc#S31115826	turquoise	1554.4	1554	0.996988	0.000198	1.966
gnl|UG|Ssc#S31115857	turquoise	1563.4	1563	0.999043	3.56E-05	4.65
gnl|UG|Ssc#S31116211	tan	172.24	14.0125	0.778508	0.120889	3.74
gnl|UG|Ssc#S31116246	lightyellow	318.5	25.4608	0.992254	0.000817	7.57
gnl|UG|Ssc#S31116354	tan	284.55	38.6463	0.986684	0.001841	30.22
gnl|UG|Ssc#S31116516	turquoise	1553.6	1553.18	0.996528	0.000245	0.188
gnl|UG|Ssc#S31118355	turquoise	1508.8	1508.42	0.988013	0.001573	2.324
gnl|UG|Ssc#S31118680	turquoise	572.42	561.605	0.753536	0.141325	1.146
gnl|UG|Ssc#S31119321	turquoise	1545	1544.6	0.995362	0.000379	4.444
gnl|UG|Ssc#S31119453	salmon	115.57	20.2027	0.895253	0.04005	5.892
gnl|UG|Ssc#S31120434	royalblue	195.66	26.1881	0.995727	0.000335	5.534
gnl|UG|Ssc#S31120438	turquoise	1387.4	1386.53	0.969664	0.006314	37.018
gnl|UG|Ssc#S31120797	brown	322.16	93.927	0.906437	0.033869	17.502
gnl|UG|Ssc#S31121510	turquoise	1432.4	1431.87	0.979539	0.003502	11.278
gnl|UG|Ssc#S31121517	turquoise	346.98	339.207	0.662975	0.222613	11.182
gnl|UG|Ssc#S31122178	turquoise	1415.6	1414.82	0.975984	0.004451	1.58
gnl|UG|Ssc#S31122252	turquoise	903.02	900.401	0.876375	0.0512	25.532
gnl|UG|Ssc#S31122306	turquoise	1570	1569.6	0.999276	2.34E-05	57.288
gnl|UG|Ssc#S31122328	turquoise	1172.4	1171.65	0.937196	0.018715	14.25
gnl|UG|Ssc#S31122485	lightcyan	84.293	6.25235	0.704785	0.183784	2.714
gnl|UG|Ssc#S31123459	brown	191.45	93.392	0.915272	0.029227	16.55
gnl|UG|Ssc#S31123896	salmon	174.52	27.416	0.956771	0.010719	14.63
gnl|UG|Ssc#S31124721	blue	307.57	142.133	0.975214	0.004667	9.29
gnl|UG|Ssc#S31124728	turquoise	1570.5	1570.09	0.999367	1.91E-05	27.312
gnl|UG|Ssc#S31124818	turquoise	1465.5	1464.78	0.985746	0.002039	28.438
gnl|UG|Ssc#S31125151	turquoise	1357.4	1356.71	0.969632	0.006324	46.654
gnl|UG|Ssc#S31128135	turquoise	1555.5	1555.06	0.998049	0.000103	2.912
gnl|UG|Ssc#S31128151	turquoise	1519.5	1519.04	0.990304	0.001145	8.552
gnl|UG|Ssc#S31128707	turquoise	1244.3	1243.62	0.948741	0.013824	1.902
gnl|UG|Ssc#S31129032	turquoise	1553.6	1553.18	0.996528	0.000245	0.33
gnl|UG|Ssc#S31129394	pink	238.68	47.7449	0.997211	0.000177	247.776
gnl|UG|Ssc#S31130453	turquoise	1559.7	1559.23	0.998721	5.49E-05	4.676
gnl|UG|Ssc#S31130713	turquoise	1539.5	1539.09	0.993959	0.000563	3.392
gnl|UG|Ssc#S31131447	turquoise	1363.7	1362.75	0.966047	0.007472	29.224
gnl|UG|Ssc#S31131596	turquoise	1553.6	1553.18	0.996528	0.000245	1.558
gnl|UG|Ssc#S31133234	turquoise	1525.4	1524.94	0.993845	0.000579	6.15
gnl|UG|Ssc#S31133660	darkturquoise	146.45	15.2146	0.954126	0.011713	16.398
gnl|UG|Ssc#S31133665	turquoise	1174.2	1172.5	0.935859	0.019311	15.906
gnl|UG|Ssc#S31133718	turquoise	990.91	990.03	0.900874	0.036902	0.78
gnl|UG|Ssc#S31133723	red	257.44	49.5023	0.982845	0.00269	55.22
gnl|UG|Ssc#S31133725	brown	202.93	94.2784	0.919775	0.026946	14.712
gnl|UG|Ssc#S31133918	lightcyan	286.57	27.0755	0.998759	5.25E-05	21.308
gnl|UG|Ssc#S31133980	yellow	84.403	23.3217	0.627631	0.256986	12.722
gnl|UG|Ssc#S31134054	blue	332.95	150.934	0.988803	0.00142	9.516
gnl|UG|Ssc#S31134161	blue	340.27	143.978	0.977623	0.004005	7.656
gnl|UG|Ssc#S31134214	turquoise	1486.6	1486.17	0.985844	0.002018	3.318

gnl|UG|Ssc#S31134253	turquoise	1056.7	1054.51	0.909251	0.032366	26.372
gnl|UG|Ssc#S31986007	brown	179.45	94.6511	0.930632	0.021702	7.724
gnl|UG|Ssc#S31986306	turquoise	1359.9	1359.19	0.970049	0.006194	5.136
gnl|UG|Ssc#S31986337	lightyellow	183.26	14.1236	0.833534	0.079464	25.436
gnl|UG|Ssc#S32419644	red	184.13	40.4421	0.941663	0.016766	34.582
gnl|UG|Ssc#S32771805	brown	316.34	94.8938	0.904016	0.035178	13.616
gnl|UG|Ssc#S32800732	blue	352.52	128.945	0.952505	0.012336	83.21
gnl|UG|Ssc#S34495577	turquoise	1385.9	1385.32	0.968116	0.006802	8.156
gnl|UG|Ssc#S34497945	turquoise	1542.9	1542.49	0.995279	0.000389	7.266
gnl|UG|Ssc#S34500484	turquoise	1464.8	1464.37	0.981225	0.00308	9.552
gnl|UG|Ssc#S34500573	blue	216.44	95.5279	0.885708	0.04558	57.308
gnl|UG|Ssc#S34500909	turquoise	1336.8	1335.69	0.963285	0.008398	10.196
gnl|UG|Ssc#S34502088	yellow	166.61	68.804	0.945106	0.015311	28.832
gnl|UG|Ssc#S34504168	brown	275.35	120.825	0.979033	0.003633	103.036
gnl|UG|Ssc#S34508066	magenta	306.4	41.9202	0.98073	0.003202	18.604
gnl|UG|Ssc#S34509910	grey	271.74	13.5993	0.871834	0.054008	44.19
gnl|UG|Ssc#S34510284	turquoise	1547.6	1547.2	0.995279	0.000389	1.938
gnl|UG|Ssc#S34510310	turquoise	1553.6	1553.18	0.996528	0.000245	0.566
gnl|UG|Ssc#S34511356	turquoise	842.32	838.662	0.857506	0.063172	15.026
gnl|UG|Ssc#S34511666	turquoise	230.89	221.395	0.549832	0.337008	11.054
gnl|UG|Ssc#S34512754	turquoise	1303.9	1302.75	0.959076	0.009877	12.652
gnl|UG|Ssc#S34512833	turquoise	910.8	908.971	0.863372	0.059366	18.834
gnl|UG|Ssc#S34513996	pink	213.76	40.8577	0.965042	0.007805	31.14
gnl|UG|Ssc#S34514135	turquoise	1495.1	1494.65	0.988	0.001575	4.114
gnl|UG|Ssc#S34514406	turquoise	1535.6	1535.2	0.992855	0.000724	7.696
gnl|UG|Ssc#S34516266	turquoise	1487.7	1487.05	0.988404	0.001496	34.92
gnl|UG|Ssc#S34516415	blue	312.65	140.935	0.973474	0.005165	11.496
gnl|UG|Ssc#S34516476	turquoise	1568.5	1568.14	0.999071	3.40E-05	3.056
gnl|UG|Ssc#S34516523	turquoise	1538.9	1538.37	0.995914	0.000313	6.384
gnl|UG|Ssc#S34517367	turquoise	1213.9	1212.66	0.936035	0.019233	94.232
gnl|UG|Ssc#S34517545	turquoise	1178.4	1177.44	0.925699	0.02404	13.034
gnl|UG|Ssc#S34518006	turquoise	1405	1404.29	0.976544	0.004297	21.632
gnl|UG|Ssc#S34518323	turquoise	1559.5	1559.12	0.997244	0.000174	6.778
gnl|UG|Ssc#S34518426	turquoise	1310.3	1309.2	0.95791	0.0103	10.026
gnl|UG|Ssc#S34521528	turquoise	1465.9	1465.47	0.982671	0.002731	3.39
gnl|UG|Ssc#S34521601	turquoise	980.66	978.496	0.889733	0.04322	40.752
gnl|UG|Ssc#S34521876	lightyellow	211.63	14.5219	0.854021	0.065467	21.576
gnl|UG|Ssc#S34521911	turquoise	1489	1488.63	0.986402	0.0019	4.548
gnl|UG|Ssc#S34524196	turquoise	1552.7	1552.28	0.996367	0.000263	3.866
gnl|UG|Ssc#S34524449	turquoise	1479.6	1479.1	0.985208	0.002155	4.932
gnl|UG|Ssc#S34527038	turquoise	1389.6	1389.13	0.969532	0.006355	6.288
gnl|UG|Ssc#S34527542	turquoise	1215.4	1214.71	0.934673	0.019846	1.54
gnl|UG|Ssc#S34527927	turquoise	1409.9	1409.02	0.976933	0.004191	17.912
gnl|UG|Ssc#S34528527	turquoise	810.5	809.175	0.842916	0.07295	39.054
gnl|UG|Ssc#S34529144	turquoise	1438.2	1437.74	0.976808	0.004225	3.294
gnl|UG|Ssc#S34529284	greenyellow	196.96	22.8175	0.975676	0.004537	33.684
gnl|UG|Ssc#S34529317	turquoise	326.41	298.796	0.584053	0.30112	7.886
gnl|UG|Ssc#S34529539	turquoise	1321.1	1320.11	0.962343	0.008722	4.39

gnl|UG|Ssc#S34529604	turquoise	1365	1364.18	0.963923	0.008181	5.398
gnl|UG|Ssc#S34529627	turquoise	1503.3	1502.82	0.988655	0.001448	3.138
gnl|UG|Ssc#S34529832	turquoise	1329.9	1328.86	0.958951	0.009922	3.416
gnl|UG|Ssc#S34530291	turquoise	1339.1	1338.32	0.961657	0.008961	17.53
gnl|UG|Ssc#S34530397	turquoise	1545.8	1545.39	0.994674	0.000466	5.986
gnl|UG|Ssc#S34530843	turquoise	1517.8	1517.39	0.989593	0.001273	15.212
gnl|UG|Ssc#S34530890	midnightblue	249.31	36.5312	0.996135	0.000288	14.2
gnl|UG|Ssc#S34531282	turquoise	1440.9	1440.34	0.980081	0.003364	32.764
gnl|UG|Ssc#S34531381	turquoise	232.19	180.633	0.438152	0.46053	36.654
gnl|UG|Ssc#S34531531	yellow	84.374	30.7642	0.732692	0.159082	26.644
gnl|UG|Ssc#S34532625	turquoise	1439.7	1439.23	0.975791	0.004505	8.456
gnl|UG|Ssc#S34532931	turquoise	1529.9	1529.49	0.99335	0.00065	3.658
gnl|UG|Ssc#S34533088	turquoise	1112.6	1110.89	0.91965	0.027009	6.128
gnl|UG|Ssc#S34533546	turquoise	1514.1	1513.75	0.989736	0.001246	14.924
gnl|UG|Ssc#S34533729	turquoise	1569.8	1569.41	0.999252	2.46E-05	4.368
gnl|UG|Ssc#S35164631	turquoise	1542.8	1542.43	0.994211	0.000528	1.79
gnl|UG|Ssc#S35166313	brown	283.16	105.16	0.936839	0.018873	5.838
gnl|UG|Ssc#S35166842	turquoise	1570.7	1570.26	0.999655	7.68E-06	3.76
gnl|UG|Ssc#S35167355	blue	339.05	150.683	0.988386	0.0015	13.328
gnl|UG|Ssc#S35167435	royalblue	150.12	21.6787	0.952103	0.012493	2.418
gnl|UG|Ssc#S35167446	yellow	144.09	56.6195	0.898791	0.038059	19.236
gnl|UG|Ssc#S35169280	blue	317.11	150.05	0.987786	0.001617	19.674
gnl|UG|Ssc#S35169669	turquoise	429.67	424.21	0.715653	0.174042	2.912
gnl|UG|Ssc#S35171904	lightcyan	298.09	26.1322	0.978214	0.003847	291.396
gnl|UG|Ssc#S35171934	turquoise	1528.4	1527.95	0.992945	0.000711	20.23
gnl|UG|Ssc#S35173188	turquoise	1032.6	1031.6	0.897301	0.038893	466.114
gnl|UG|Ssc#S35173683	green	258.27	50.8166	0.964122	0.008114	17.85
gnl|UG|Ssc#S35322082	turquoise	119.74	85.6901	0.318672	0.60123	1.334
gnl|UG|Ssc#S35322342	turquoise	65.715	54.5413	0.33325	0.583682	2.756
gnl|UG|Ssc#S35322980	turquoise	557.2	553.623	0.757947	0.137647	2.992
gnl|UG|Ssc#S35323260	turquoise	1507.2	1506.66	0.990515	0.001107	7.498
gnl|UG|Ssc#S35323577	brown	297.27	110.548	0.953906	0.011797	4.782
gnl|UG|Ssc#S35323677	turquoise	353.3	344.894	0.637176	0.247572	13.366
gnl|UG|Ssc#S35323988	turquoise	1475.7	1475.28	0.982373	0.002802	14.786
gnl|UG|Ssc#S35324071	turquoise	1502.5	1502.08	0.988102	0.001555	1.828
gnl|UG|Ssc#S35324845	turquoise	1410.2	1409.66	0.975209	0.004668	3.772
gnl|UG|Ssc#S35325128	darkgreen	225.79	21.9478	0.970413	0.006082	4.584
gnl|UG|Ssc#S35325248	turquoise	1164.3	1163.46	0.929694	0.02214	19.02
gnl|UG|Ssc#S35325457	turquoise	1492.7	1492.22	0.987473	0.00168	3.618
gnl|UG|Ssc#S35325791	tan	322.58	38.4158	0.98284	0.002692	17.484
gnl|UG|Ssc#S35325947	brown	252.99	125.449	0.989683	0.001256	9.312
gnl|UG|Ssc#S35326006	turquoise	203.15	189.83	0.513931	0.375714	18.414
gnl|UG|Ssc#S35326767	yellow	179.07	68.5961	0.937865	0.018418	17.626
gnl|UG|Ssc#S35327002	turquoise	1536.3	1535.85	0.993277	0.000661	5.436
gnl|UG|Ssc#S35327311	turquoise	1068.5	1067.18	0.902542	0.035984	10.282
gnl|UG|Ssc#S35328709	brown	83.056	34.9023	0.72343	0.167167	9.406
gnl|UG|Ssc#S35329254	turquoise	956.7	955.409	0.877386	0.050582	18.498
gnl|UG|Ssc#S35329402	green	229.34	53.4715	0.9842	0.002378	122.382

gnl|UG|Ssc#S35329994	turquoise	1544.3	1543.86	0.994949	0.000431	4.678
gnl|UG|Ssc#S35331200	pink	234.04	40.7143	0.959759	0.009632	13.93
gnl|UG|Ssc#S35331378	turquoise	1562.6	1562.2	0.998444	7.37E-05	3.51
gnl|UG|Ssc#S35331631	turquoise	1242.9	1241.98	0.948373	0.013972	2.364
gnl|UG|Ssc#S35332920	turquoise	1201.7	1200.97	0.943117	0.016146	11.038
gnl|UG|Ssc#S35332961	turquoise	785.4	781.168	0.83838	0.076078	21.226
gnl|UG|Ssc#S35333016	red	202.52	48.4116	0.980266	0.003318	11.87
gnl|UG|Ssc#S35334259	pink	173.79	42.0217	0.96997	0.006219	22.234
gnl|UG|Ssc#S35336579	green	97.333	23.9941	0.805805	0.099682	8.538
gnl|UG|Ssc#S35336616	turquoise	1476.3	1475.75	0.985183	0.00216	6.432
gnl|UG|Ssc#S35723183	turquoise	1541.2	1540.82	0.994891	0.000438	28.954
gnl|UG|Ssc#S35723730	turquoise	1445.9	1445.19	0.979437	0.003529	37.032
gnl|UG|Ssc#S38479783	turquoise	1545.5	1545.03	0.997042	0.000193	50.432
gnl|UG|Ssc#S38479786	green	193.48	47.0806	0.959099	0.009868	128.982
gnl|UG|Ssc#S38480083	greenyellow	68.515	0.81236	0.089514	0.886179	42.256
gnl|UG|Ssc#S38480459	turquoise	1570.6	1570.22	0.999495	1.36E-05	1.548
gnl|UG|Ssc#S38481028	turquoise	1542.2	1541.75	0.995703	0.000338	19.83
gnl|UG|Ssc#S38481167	turquoise	1220.7	1219.23	0.944061	0.015748	3.242
gnl|UG|Ssc#S38481210	turquoise	1280.6	1279.89	0.948404	0.01396	2.254
gnl|UG|Ssc#S38481364	lightcyan	284.3	27.2531	0.997793	0.000124	30.318
gnl|UG|Ssc#S38481793	blue	249.65	104.082	0.904549	0.034889	7.082
gnl|UG|Ssc#S38481847	blue	269.99	130.778	0.956422	0.010849	22.482
gnl|UG|Ssc#S38481918	turquoise	1110.8	1109.83	0.920907	0.026383	23.518
gnl|UG|Ssc#S38482262	turquoise	1435.3	1434.8	0.975481	0.004592	23.64
gnl|UG|Ssc#S38482319	brown	293.57	109.303	0.945015	0.015349	86.904
gnl|UG|Ssc#S38482329	blue	207.29	86.1347	0.862981	0.059617	50.806
gnl|UG|Ssc#S38482352	turquoise	1448.6	1447.85	0.982674	0.002731	8.694
gnl|UG|Ssc#S38482401	turquoise	1506.7	1506.24	0.989631	0.001265	1.352
gnl|UG|Ssc#S38483118	turquoise	1532.5	1532.11	0.99356	0.00062	4.272
gnl|UG|Ssc#S39762052	brown	246.35	129.7	0.999157	2.94E-05	13.762
gnl|UG|Ssc#S39768843	brown	238.58	86.2226	0.895771	0.039756	6.718
gnl|UG|Ssc#S39772429	lightcyan	234.37	17.7338	0.887659	0.044431	55.712
gnl|UG|Ssc#S39773791	midnightblue	232.44	36.2271	0.994396	0.000503	87.732
gnl|UG|Ssc#S39776069	tan	278.72	34.8531	0.963149	0.008445	112.226
gnl|UG|Ssc#S39777926	yellow	222.98	64.2478	0.925951	0.023918	303.14
gnl|UG|Ssc#S39778104	yellow	311.18	57.2478	0.87106	0.054492	26.268
gnl|UG|Ssc#S39778806	darkred	110.28	11.3693	0.852955	0.066175	84.868
gnl|UG|Ssc#S39779823	turquoise	1496.3	1495.85	0.986237	0.001934	3.584
gnl|UG|Ssc#S39780074	turquoise	1517.2	1516.81	0.990624	0.001088	5.292
gnl|UG|Ssc#S39789448	turquoise	1557.4	1557	0.997823	0.000122	20.162
gnl|UG|Ssc#S39793738	turquoise	1524.8	1524.3	0.992868	0.000722	3.19
gnl|UG|Ssc#S39794612	darkgreen	302.04	6.995	0.714032	0.175486	21.388
gnl|UG|Ssc#S39794773	grey60	186.53	26.4019	0.94741	0.014362	279.002
gnl|UG|Ssc#S39797758	yellow	148.04	64.2378	0.929319	0.022316	14.092
gnl|UG|Ssc#S39798692	turquoise	1121.4	1120.36	0.916304	0.028699	9.78
gnl|UG|Ssc#S39803256	yellow	73.97	23.3712	0.652372	0.232783	9.408
gnl|UG|Ssc#S39804944	turquoise	1562.5	1562.03	0.999176	2.84E-05	3.082
gnl|UG|Ssc#S39808414	greenyellow	234.16	20.587	0.949075	0.013689	5.63

gnl|UG|Ssc#S39812694	turquoise	1189	1187.9	0.928829	0.022547	5.452
gnl|UG|Ssc#S39814752	turquoise	1422.6	1421.84	0.979046	0.00363	28.952
gnl|UG|Ssc#S39833392	magenta	341.19	44.4348	0.992771	0.000737	44.35
gnl|UG|Ssc#S39838237	yellow	203.93	79.9793	0.983337	0.002576	7.974
gnl|UG|Ssc#S39839709	pink	266.91	43.6798	0.97394	0.00503	43.444
gnl|UG|Ssc#S39839769	grey60	171.14	25.8981	0.944844	0.015421	86.49
gnl|UG|Ssc#S39839770	brown	292.81	101.841	0.935007	0.019695	17.954
gnl|UG|Ssc#S39839881	turquoise	1317.9	1317.07	0.956389	0.010861	3.058
gnl|UG|Ssc#S39841622	black	256.27	39.5445	0.964539	0.007973	32.394
gnl|UG|Ssc#S39842447	purple	138.21	22.3895	0.869037	0.055762	422.45
gnl|UG|Ssc#S39842527	greenyellow	182.51	22.5092	0.975981	0.004453	3.332
gnl|UG|Ssc#S39851973	tan	326.86	38.6443	0.983935	0.002438	29.692
gnl|UG|Ssc#S39853219	turquoise	1542.8	1542.42	0.99431	0.000515	4.846
gnl|UG|Ssc#S39855926	turquoise	1496.4	1495.95	0.986085	0.001966	2.03
gnl|UG|Ssc#S39858087	green	232.54	53.8573	0.987584	0.001658	50.428
gnl|UG|Ssc#S39861498	tan	332.48	40.5668	0.995528	0.000359	176.924
gnl|UG|Ssc#S39872348	turquoise	1293.9	1293.15	0.949526	0.013509	7.508
gnl|UG|Ssc#S39879611	turquoise	306.33	301.154	0.628701	0.255925	3.464
gnl|UG|Ssc#S39885581	yellow	197	68.342	0.936111	0.019198	22.862
gnl|UG|Ssc#S39888860	midnightblue	186.88	31.0119	0.962855	0.008546	35.706
gnl|UG|Ssc#S39889520	blue	343.73	150.463	0.988213	0.001533	170.682
gnl|UG|Ssc#S39891679	turquoise	928.22	925.238	0.880274	0.048827	196.964
gnl|UG|Ssc#S39900214	lightcyan	272.95	25.8636	0.987597	0.001655	18.09
gnl|UG|Ssc#S39912933	greenyellow	79.621	7.4164	-0.44164	0.456539	52.298
gnl|UG|Ssc#S39928606	brown	265.31	114.068	0.960848	0.009245	2.774
gnl|UG|Ssc#S39932918	turquoise	1214.6	1213.71	0.933384	0.020432	4.312
gnl|UG|Ssc#S39935005	turquoise	1568.9	1568.49	0.999487	1.40E-05	7.912
gnl|UG|Ssc#S39943227	turquoise	1480.6	1480.09	0.983743	0.002482	5.952
gnl|UG|Ssc#S39951768	turquoise	1110.2	1109.1	0.911668	0.031093	11.334
gnl|UG|Ssc#S39955157	turquoise	1559.4	1559.03	0.997365	0.000162	10.63
gnl|UG|Ssc#S39957439	turquoise	1481.3	1480.81	0.984573	0.002295	11.01
gnl|UG|Ssc#S39959286	turquoise	1508.5	1508.09	0.990138	0.001174	3.964
gnl|UG|Ssc#S39959416	turquoise	1426.6	1426.09	0.976349	0.004351	13.136
gnl|UG|Ssc#S39959676	turquoise	1536.1	1535.67	0.994826	0.000446	2.666
gnl|UG|Ssc#S39960115	brown	314.37	84.8365	0.873736	0.052826	17.608
gnl|UG|Ssc#S39964958	lightgreen	44.909	14.9562	0.910214	0.031857	6.552
gnl|UG|Ssc#S39967395	turquoise	1428.4	1427.56	0.979078	0.003621	9.972
gnl|UG|Ssc#S39967671	purple	177.65	31.8757	0.960938	0.009213	18.218
gnl|UG|Ssc#S39979082	turquoise	272.65	265.066	0.597543	0.287263	106.744
gnl|UG|Ssc#S39979775	blue	322.54	156.754	0.997498	0.00015	9.574
gnl|UG|Ssc#S39979866	darkred	90.698	12.8989	0.922066	0.025809	51.01
gnl|UG|Ssc#S39980022	pink	146.71	36.4575	0.936717	0.018928	18.472
gnl|UG|Ssc#S39980075	turquoise	1517	1516.58	0.989946	0.001208	3.666
gnl|UG|Ssc#S39980686	turquoise	1553.6	1553.18	0.996528	0.000245	0.142
gnl|UG|Ssc#S39982552	green	247	50.9936	0.974764	0.004794	151.878
gnl|UG|Ssc#S39982735	turquoise	1530.2	1529.76	0.992815	0.00073	3.184
gnl|UG|Ssc#S39983835	turquoise	1223.8	1222.57	0.946113	0.014894	3.614
gnl|UG|Ssc#S39984038	turquoise	182.84	134.823	0.372569	0.536845	108.874

gnl|UG|Ssc#S39984272	grey60	228.69	29.1753	0.969952	0.006224	27.068
gnl|UG|Ssc#S39984816	turquoise	1553.6	1553.18	0.996528	0.000245	0.378
gnl|UG|Ssc#S39985253	turquoise	654.01	648.001	0.79748	0.106018	3.788
gnl|UG|Ssc#S39985763	turquoise	1553.6	1553.18	0.996528	0.000245	0.898
gnl|UG|Ssc#S39987785	turquoise	1233.5	1231.94	0.94336	0.016043	2.084
gnl|UG|Ssc#S39988753	brown	206.13	91.8904	0.91358	0.030099	2.984
gnl|UG|Ssc#S39989284	tan	335.48	37.9845	0.980334	0.003301	14.036
gnl|UG|Ssc#S39990281	blue	339.64	154.535	0.994371	0.000507	10.904
gnl|UG|Ssc#S39990452	turquoise	1571	1570.59	0.999583	1.02E-05	1.454
gnl|UG|Ssc#S39991071	turquoise	550.69	543.158	0.753817	0.14109	5.778
gnl|UG|Ssc#S39991153	turquoise	1520.5	1520.04	0.992938	0.000712	4.262
gnl|UG|Ssc#S39992206	turquoise	1553.6	1553.18	0.996528	0.000245	3.07
gnl|UG|Ssc#S39992562	blue	315.86	153.288	0.992443	0.000788	7.464
gnl|UG|Ssc#S39993494	red	266.65	47.168	0.970283	0.006122	94.282
gnl|UG|Ssc#S39993541	turquoise	1553.6	1553.18	0.996528	0.000245	2.268
gnl|UG|Ssc#S39994120	tan	305.67	35.43	0.964808	0.007883	39.494
gnl|UG|Ssc#S39994161	turquoise	1458	1457.37	0.984247	0.002368	12.308
gnl|UG|Ssc#S39994288	turquoise	1553.6	1553.18	0.996528	0.000245	0.188
gnl|UG|Ssc#S39994657	yellow	246.29	57.0963	0.863555	0.059248	20.196
gnl|UG|Ssc#S39995011	turquoise	1355.6	1355.06	0.96186	0.00889	0.52
gnl|UG|Ssc#S39995335	turquoise	724.11	720.639	0.827605	0.083668	21.07
gnl|UG|Ssc#S39995545	turquoise	1553.6	1553.18	0.996528	0.000245	2.786
gnl|UG|Ssc#S39996844	turquoise	231.36	217.89	0.558337	0.327994	60.62
gnl|UG|Ssc#S39997671	purple	165.56	28.4	0.930065	0.021967	0.488
gnl|UG|Ssc#S39998520	turquoise	1553.6	1553.18	0.996528	0.000245	0.426
gnl|UG|Ssc#S39998964	brown	257.62	124.687	0.989018	0.001379	6.754
gnl|UG|Ssc#S39998988	grey60	269.16	29.9238	0.976526	0.004302	1470.916
gnl|UG|Ssc#S39999498	turquoise	1553.6	1553.18	0.996528	0.000245	0.566
gnl|UG|Ssc#S40000845	turquoise	1531.6	1531.18	0.99444	0.000497	1.794
gnl|UG|Ssc#S40003052	turquoise	1531.1	1530.68	0.993926	0.000568	92.334
gnl|UG|Ssc#S40003133	turquoise	1568.5	1568.12	0.999606	9.38E-06	2.758
gnl|UG|Ssc#S40004648	turquoise	136.15	111.449	0.401831	0.502496	78.814
gnl|UG|Ssc#S40004911	turquoise	1310.2	1309.63	0.955537	0.011179	1.16
gnl|UG|Ssc#S40006504	turquoise	1528.8	1528.46	0.992792	0.000734	3.422
gnl|UG|Ssc#S40007606	turquoise	1233.3	1231.86	0.942807	0.016277	11.742
gnl|UG|Ssc#S40007947	turquoise	1515.4	1515.01	0.989459	0.001297	22.536
gnl|UG|Ssc#S40008864	royalblue	194.99	24.4169	0.980837	0.003175	15.308
gnl|UG|Ssc#S40012596	turquoise	389.07	367.388	0.6433	0.241582	4.536
gnl|UG|Ssc#S40012957	turquoise	1553.6	1553.18	0.996528	0.000245	1.512
gnl|UG|Ssc#S40014860	turquoise	1393.4	1392.64	0.972465	0.005462	9.792
gnl|UG|Ssc#S40017556	royalblue	165.24	23.8283	0.973987	0.005017	4.07
gnl|UG|Ssc#S40020159	navy	270.13	30.1337	0.965076	0.007794	251.016
gnl|UG|Ssc#S40022499	tan	338.47	39.7213	0.990598	0.001093	8.198
gnl|UG|Ssc#S40024674	salmon	222.91	24.9717	0.923976	0.024874	24.616
gnl|UG|Ssc#S40034650	brown	286.59	99.3936	0.922768	0.025464	50.096
gnl|UG|Ssc#S40034722	tan	293.72	38.1854	0.983714	0.002489	254.708
gnl|UG|Ssc#S40035688	darkgreen	229.78	19.4497	0.949396	0.013561	3.628
gnl|UG|Ssc#S40037863	grey60	265.12	32.5653	0.993788	0.000587	78.852

gnl|UG|Ssc#S40038955	black	278.45	39.5795	0.983889	0.002449	344.28
gnl|UG|Ssc#S40039005	turquoise	631.28	626.717	0.780415	0.119369	10.99
gnl|UG|Ssc#S40039737	turquoise	1554.9	1554.56	0.996619	0.000236	11.828
gnl|UG|Ssc#S40042081	brown	246.5	127.288	0.994426	0.000499	19.844
gnl|UG|Ssc#S40042631	tan	238.38	32.4764	0.949446	0.013541	2607.526
gnl|UG|Ssc#S40042784	turquoise	287.09	272.721	0.597965	0.286832	24.444
gnl|UG|Ssc#S40043958	yellow	295.36	56.4998	0.858643	0.062428	97.33
gnl|UG|Ssc#S40044675	turquoise	1495.2	1494.66	0.987146	0.001746	11.35
gnl|UG|Ssc#S40045135	grey	97.69	1.20606	-0.30465	0.618191	19.016
gnl|UG|Ssc#S40045387	turquoise	932.08	930.277	0.868363	0.056187	11.942
gnl|UG|Ssc#S40046162	turquoise	1413.7	1412.88	0.975681	0.004536	5.136
gnl|UG|Ssc#S40047541	grey	63.06	7.97278	-0.08854	0.887414	3.086
gnl|UG|Ssc#S40047900	turquoise	873.33	871.533	0.860323	0.061335	9.81
gnl|UG|Ssc#S40048435	turquoise	243.65	228.014	0.55444	0.332117	39.73
gnl|UG|Ssc#S40048913	turquoise	1464.8	1464.2	0.98385	0.002458	28.18
gnl|UG|Ssc#S40048956	tan	223.16	30.1347	0.933309	0.020467	49.726
gnl|UG|Ssc#S40049940	turquoise	1541.8	1541.28	0.996154	0.000286	4.234
gnl|UG|Ssc#S40049981	yellow	238.93	82.9964	0.991442	0.000949	79.732
gnl|UG|Ssc#S40050544	magenta	341.15	40.0562	0.968451	0.006695	37.284
gnl|UG|Ssc#S40050849	turquoise	336.61	308.886	0.592829	0.292086	24.326
gnl|UG|Ssc#S40050895	turquoise	1546.4	1545.94	0.996564	0.000242	4.612
gnl|UG|Ssc#S40051540	tan	341.21	37.5312	0.976846	0.004215	44.178
gnl|UG|Ssc#S40052404	darkgreen	254.44	23.1669	0.987114	0.001753	61.444
gnl|UG|Ssc#S40053273	yellow	223.09	83.9961	0.994757	0.000455	335.016
gnl|UG|Ssc#S40053418	yellow	155.88	56.8861	0.89652	0.039333	148.696
gnl|UG|Ssc#S40054185	grey	85.948	10.057	0.302185	0.621184	91.598
gnl|UG|Ssc#S40054290	turquoise	276.31	244.351	0.533151	0.354864	31.532
gnl|UG|Ssc#S40055771	turquoise	1389.9	1389.35	0.969899	0.006241	3.624
gnl|UG|Ssc#S40055820	green	241.56	54.2497	0.991315	0.00097	7.49
gnl|UG|Ssc#S40055957	purple	200.16	31.0912	0.955499	0.011194	23.418
gnl|UG|Ssc#S40059478	blue	279.75	138.896	0.970131	0.006169	22.852
gnl|UG|Ssc#S40060284	tan	180.58	23.5288	0.874682	0.052242	4.768
gnl|UG|Ssc#S40060840	darkgreen	308.97	22.0418	0.984647	0.002278	75.742
gnl|UG|Ssc#S40061661	grey	38.201	0.12932	0.074195	0.905619	29.024
gnl|UG|Ssc#S40063653	yellow	127.4	39.0237	0.788392	0.113069	5.074
gnl|UG|Ssc#S40064881	brown	190.11	108.4	0.953969	0.011773	17.614
gnl|UG|Ssc#S40066476	turquoise	1261	1259.69	0.951466	0.012741	80.588
gnl|UG|Ssc#S40068231	turquoise	133.59	92.0379	0.309266	0.612601	15.052
gnl|UG|Ssc#S40069929	turquoise	1434.6	1434.16	0.978241	0.00384	9.78
gnl|UG|Ssc#S40070781	turquoise	1511.6	1511.21	0.988495	0.001479	10.914
gnl|UG|Ssc#S40072585	brown	180.49	98.8747	0.940703	0.017178	7.038
gnl|UG|Ssc#S40073180	red	218.14	46.9651	0.973534	0.005148	114.234
gnl|UG|Ssc#S40077180	blue	325.23	133.004	0.961633	0.008969	12.266
gnl|UG|Ssc#S40077859	turquoise	143.56	118.473	0.398076	0.506878	0.866
gnl|UG|Ssc#S40078819	turquoise	998.16	995.504	0.895088	0.040143	29.618
gnl|UG|Ssc#S40079585	turquoise	1009.9	1007.14	0.896682	0.039242	35.346
gnl|UG|Ssc#S40082625	blue	284.24	141.726	0.974441	0.004886	7.248
gnl|UG|Ssc#S40082993	turquoise	765.93	760.902	0.830469	0.081629	4.61

gnl|UG|Ssc#S40084501	turquoise	1553.6	1553.18	0.996528	0.000245	0.566
gnl|UG|Ssc#S40084519	turquoise	437.39	429.765	0.698017	0.189926	6.878
gnl|UG|Ssc#S40084984	turquoise	1066.5	1064.35	0.913335	0.030225	7.33
gnl|UG|Ssc#S40086007	turquoise	1553.6	1553.18	0.996528	0.000245	1.842
gnl|UG|Ssc#S40087890	turquoise	1203.1	1201.59	0.938108	0.018311	3.076
gnl|UG|Ssc#S40089767	purple	189.72	33.1839	0.97863	0.003738	119.576
gnl|UG|Ssc#S40091110	turquoise	458.17	455.866	0.736375	0.155901	26.372
gnl|UG|Ssc#S40091442	brown	300.79	106.932	0.939071	0.017888	5.046
gnl|UG|Ssc#S40091470	turquoise	1390.8	1390.32	0.970042	0.006196	42.53
gnl|UG|Ssc#S40092912	turquoise	1485.6	1485.13	0.984887	0.002225	1.924
gnl|UG|Ssc#S40094025	blue	311.15	151.787	0.990237	0.001156	19.254
gnl|UG|Ssc#S40094148	turquoise	1500	1499.53	0.989792	0.001236	4.616
gnl|UG|Ssc#S40094285	brown	142.65	66.9036	0.853929	0.065528	19.57
gnl|UG|Ssc#S40094835	red	162.54	42.5166	0.951359	0.012783	3.632
gnl|UG|Ssc#S40094925	turquoise	1303.7	1302.45	0.956844	0.010692	2.366
gnl|UG|Ssc#S40095003	turquoise	1486.1	1485.42	0.987871	0.001601	3.306
gnl|UG|Ssc#S40095231	turquoise	1553.6	1553.18	0.996528	0.000245	0.33
gnl|UG|Ssc#S40095449	turquoise	571.78	569.303	0.782033	0.118082	2.84
gnl|UG|Ssc#S40095498	red	261.98	44.8398	0.958168	0.010206	7.8
gnl|UG|Ssc#S40096829	turquoise	1183.3	1181.65	0.9347	0.019834	49.04
gnl|UG|Ssc#S40097075	brown	247.75	122.505	0.983429	0.002554	3.514
gnl|UG|Ssc#S40097724	turquoise	420.7	418.301	0.703981	0.184511	3.51
gnl|UG|Ssc#S40097726	brown	250.76	124.844	0.989277	0.001331	4.216
gnl|UG|Ssc#S40098899	turquoise	1553.6	1553.18	0.996528	0.000245	0.188
gnl|UG|Ssc#S40099212	turquoise	1553.6	1553.18	0.996528	0.000245	0.094
gnl|UG|Ssc#S40099717	blue	328.23	156.631	0.997348	0.000164	4.446
gnl|UG|Ssc#S40100108	turquoise	1446.1	1445.37	0.981422	0.003031	14.222
gnl|UG|Ssc#S40100454	royalblue	231.28	23.9492	0.975702	0.00453	24.29
gnl|UG|Ssc#S40101480	brown	336.56	78.2128	0.852668	0.066366	6.33
gnl|UG|Ssc#S40102872	turquoise	1062.7	1060.23	0.907871	0.0331	1.898
gnl|UG|Ssc#S40102974	black	172.98	22.8535	0.837635	0.076596	11.012
gnl|UG|Ssc#S40103595	turquoise	1553.6	1553.18	0.996528	0.000245	0.142
gnl|UG|Ssc#S40104089	turquoise	1198.1	1197.11	0.92976	0.02211	44.884
gnl|UG|Ssc#S40105175	turquoise	887.09	885.887	0.875	0.052046	0.572
gnl|UG|Ssc#S40106146	turquoise	1494.7	1494.16	0.98951	0.001288	31.522
gnl|UG|Ssc#S40106217	grey60	239.18	32.8524	0.995969	0.000307	26.042
gnl|UG|Ssc#S40106548	turquoise	1553.6	1553.18	0.996528	0.000245	0.756
gnl|UG|Ssc#S40106937	turquoise	1531.5	1531.17	0.992445	0.000787	2.514
gnl|UG|Ssc#S40106980	brown	260.04	128.699	0.995271	0.00039	8.12
gnl|UG|Ssc#S40107966	turquoise	195.38	139.561	0.396771	0.508403	4.52
gnl|UG|Ssc#S40108865	tan	266.68	36.9815	0.976568	0.004291	53.896
gnl|UG|Ssc#S40110548	darkturquoise	141.95	14.0927	0.933501	0.020379	12.302
gnl|UG|Ssc#S40110625	turquoise	634.69	629.049	0.79451	0.108307	15.658
gnl|UG|Ssc#S40110683	turquoise	153.12	81.6637	0.222371	0.71922	40.778
gnl|UG|Ssc#S40111130	brown	236.99	120.508	0.981167	0.003094	4.664
gnl|UG|Ssc#S40117649	blue	262.95	119.742	0.936975	0.018813	21.326
gnl|UG|Ssc#S40125669	magenta	304.91	34.5013	0.938645	0.018075	7.002
gnl|UG|Ssc#S40127760	grey60	257.77	33.2675	0.998595	6.32E-05	19.582

gnl|UG|Ssc#S40127774	magenta	263.2	26.4512	0.883117	0.047119	137.98
gnl|UG|Ssc#S40131124	turquoise	1503.2	1502.68	0.988769	0.001426	3.6
gnl|UG|Ssc#S40132479	turquoise	242.59	199.02	0.469179	0.425327	2.538
gnl|UG|Ssc#S40133187	darkgreen	258.81	22.0524	0.980197	0.003335	6.706
gnl|UG|Ssc#S40141315	purple	194.29	28.5028	0.936436	0.019053	24.236
gnl|UG|Ssc#S40141829	turquoise	842.54	840.434	0.866293	0.057499	104.494
gnl|UG|Ssc#S40143363	turquoise	641.93	636.162	0.792723	0.109692	34.224
gnl|UG|Ssc#S40143536	pink	224	44.9497	0.983566	0.002523	57.318
gnl|UG|Ssc#S40143544	blue	345.9	140.006	0.972425	0.005474	27.798
gnl|UG|Ssc#S40144447	yellow	163.93	39.9167	0.784171	0.116389	61.212
gnl|UG|Ssc#S40144457	yellow	108.9	48.4919	0.834842	0.078545	103.862
gnl|UG|Ssc#S40146297	lightgreen	87.877	6.38143	0.516511	0.372898	24.542
gnl|UG|Ssc#S40146561	green	145.43	36.9012	0.903744	0.035327	17.814
gnl|UG|Ssc#S40146833	yellow	230.58	77.3048	0.9725	0.005452	15.862
gnl|UG|Ssc#S40148087	turquoise	1536.6	1536.12	0.994865	0.000441	13.178
gnl|UG|Ssc#S40148092	grey60	284.8	28.8548	0.968384	0.006716	105.28
gnl|UG|Ssc#S40149693	magenta	278.79	34.329	0.937658	0.01851	55.084
gnl|UG|Ssc#S40149996	blue	318.4	155.358	0.995471	0.000366	15.31
gnl|UG|Ssc#S40150666	navy	243.62	33.0685	0.990737	0.001069	16.406
gnl|UG|Ssc#S40152348	black	281.35	38.205	0.94574	0.015048	25.23
gnl|UG|Ssc#S40153033	brown	228.31	124.072	0.988725	0.001435	9.866
gnl|UG|Ssc#S40153514	darkgreen	286.81	8.75994	0.765439	0.131466	100.71
gnl|UG|Ssc#S40154377	brown	253.38	129.421	0.997203	0.000177	3.47
gnl|UG|Ssc#S40154544	turquoise	1538.9	1538.43	0.996055	0.000297	151.586
gnl|UG|Ssc#S40154742	blue	299.5	142.609	0.976175	0.004399	7.128
gnl|UG|Ssc#S40154789	salmon	250.59	25.832	0.937018	0.018794	42.914
gnl|UG|Ssc#S40155274	green	120.46	33.05	0.871932	0.053947	1.354
gnl|UG|Ssc#S40156252	turquoise	1510	1509.59	0.988392	0.001499	7.294
gnl|UG|Ssc#S40156624	turquoise	1471.1	1470.71	0.984413	0.00233	2.272
gnl|UG|Ssc#S40156654	turquoise	862.06	857.677	0.857787	0.062988	3.532
gnl|UG|Ssc#S40157220	turquoise	1525.1	1524.73	0.992139	0.000836	74.016
gnl|UG|Ssc#S40158599	grey60	266.69	30.3464	0.978946	0.003656	23.818
gnl|UG|Ssc#S40159132	royalblue	234.13	23.8725	0.9748	0.004784	21.816
gnl|UG|Ssc#S40160168	turquoise	1565.6	1565.18	0.99872	5.50E-05	7.422
gnl|UG|Ssc#S40162503	lightgreen	86.407	7.35547	0.782137	0.118	125.582
gnl|UG|Ssc#S40163661	lightgreen	59.513	16.9296	0.993902	0.000571	11.904
gnl|UG|Ssc#S40170485	turquoise	318.94	308.528	0.642805	0.242064	32.714
gnl|UG|Ssc#S40171668	black	123.17	15.5178	0.698422	0.189557	62.2
gnl|UG|Ssc#S40172065	turquoise	1498.1	1497.56	0.988637	0.001452	9.894
gnl|UG|Ssc#S40173770	turquoise	1186.6	1184.97	0.937828	0.018434	195.028
gnl|UG|Ssc#S40174573	lightcyan	278.59	23.6371	0.957497	0.010452	9.68
gnl|UG|Ssc#S40174963	red	254.92	46.5423	0.968649	0.006632	136.212
gnl|UG|Ssc#S40175618	turquoise	1511.7	1511.28	0.988904	0.001401	2.028
gnl|UG|Ssc#S40177122	turquoise	1082.8	1080.95	0.913073	0.030361	34.184
gnl|UG|Ssc#S40180694	tan	310.85	36.7435	0.973909	0.005039	53.082
gnl|UG|Ssc#S40182904	turquoise	970.48	968.237	0.892993	0.041338	24.522
gnl|UG|Ssc#S40184013	lightgreen	72.008	9.47914	0.859072	0.062148	97.54
gnl|UG|Ssc#S40186233	blue	300.67	131.331	0.956686	0.01075	753.842

gnl|UG|Ssc#S40191766	brown	332.99	86.2968	0.878751	0.04975	5.004
gnl|UG|Ssc#S40193201	black	296.54	36.0234	0.941093	0.01701	41.136
gnl|UG|Ssc#S40195327	blue	337.23	139.161	0.971184	0.005846	161.268
gnl|UG|Ssc#S40196887	blue	314.1	145.041	0.979922	0.003405	29.41
gnl|UG|Ssc#S40204438	blue	290.05	144.83	0.979306	0.003563	22.922
gnl|UG|Ssc#S40204678	turquoise	1508.7	1508.2	0.990888	0.001043	95.958
gnl|UG|Ssc#S40205238	turquoise	1431.8	1431.25	0.974731	0.004804	8.128
gnl|UG|Ssc#S40206941	blue	337.61	124.709	0.947254	0.014426	81.838
gnl|UG|Ssc#S40207636	turquoise	1515.9	1515.47	0.991974	0.000862	1.79
gnl|UG|Ssc#S40210420	turquoise	177.92	88.5632	0.227912	0.712346	21.712
gnl|UG|Ssc#S40212608	turquoise	290.71	282.43	0.614836	0.269752	0.56
gnl|UG|Ssc#S40213312	pink	264.87	41.6466	0.964341	0.00804	100.3
gnl|UG|Ssc#S40214545	turquoise	1151.2	1150.24	0.922455	0.025618	3.15
gnl|UG|Ssc#S40214866	black	251.47	38.6268	0.977233	0.00411	11.566
gnl|UG|Ssc#S40215438	turquoise	1378.4	1377.93	0.969825	0.006264	2.18
gnl|UG|Ssc#S40215563	brown	154.56	90.1645	0.905508	0.03437	2.694
gnl|UG|Ssc#S40219335	green	231.18	54.24	0.990287	0.001148	4.696
gnl|UG|Ssc#S40224927	pink	193.88	45.1484	0.985922	0.002001	5.624
gnl|UG|Ssc#S40226524	navy	284.11	27.3479	0.940009	0.017479	33.8
gnl|UG|Ssc#S40227949	brown	172.27	91.062	0.907741	0.03317	10.148
gnl|UG|Ssc#S40232606	magenta	316.58	39.2236	0.966911	0.007189	17.432
gnl|UG|Ssc#S40234755	turquoise	1523.9	1523.46	0.993015	0.0007	4.004
gnl|UG|Ssc#S40235024	turquoise	98.603	90.8553	0.418834	0.482754	30.086
gnl|UG|Ssc#S40237707	turquoise	1515.7	1515.32	0.990095	0.001182	2.094
gnl|UG|Ssc#S40238117	turquoise	668.22	665.288	0.797849	0.105735	31.56
gnl|UG|Ssc#S40239504	turquoise	176.5	71.5085	0.187012	0.763285	12.454
gnl|UG|Ssc#S40239854	turquoise	1523.7	1523.3	0.991042	0.001016	2.086
gnl|UG|Ssc#S40242427	turquoise	457.92	445.032	0.697117	0.190747	1.586
gnl|UG|Ssc#S40247487	turquoise	1343.5	1342.69	0.964811	0.007882	22.562
gnl|UG|Ssc#S40258460	turquoise	658.27	652.432	0.797115	0.106299	29.244
gnl|UG|Ssc#S40262471	turquoise	439.59	434.273	0.725758	0.165123	0.178
gnl|UG|Ssc#S40262955	darkgreen	247.69	21.8704	0.974866	0.004765	4.81
gnl|UG|Ssc#S40265049	brown	243.06	129.098	0.997729	0.00013	6.334
gnl|UG|Ssc#S40267793	midnightblue	260.68	32.4203	0.970941	0.00592	93.876
gnl|UG|Ssc#S40268747	royalblue	164.51	24.0165	0.974676	0.004819	4.754
gnl|UG|Ssc#S40271107	green	267.48	46.6125	0.946158	0.014876	18.514
gnl|UG|Ssc#S40272618	midnightblue	267.43	29.9221	0.95326	0.012045	69.936
gnl|UG|Ssc#S40276387	magenta	322.78	44.3732	0.993164	0.000678	9.302
gnl|UG|Ssc#S40277991	turquoise	1464.6	1464.12	0.984639	0.00228	10.982
gnl|UG|Ssc#S40284317	brown	255.29	111.168	0.957403	0.010486	5.026
gnl|UG|Ssc#S40287461	purple	181.44	26.5706	0.92055	0.02656	29.874
gnl|UG|Ssc#S40288024	green	150.39	28.4008	0.827036	0.084075	10.21
gnl|UG|Ssc#S40288165	turquoise	1544.7	1544.35	0.995361	0.000379	2.15
gnl|UG|Ssc#S40288641	turquoise	1518.9	1518.49	0.992802	0.000732	5.722
gnl|UG|Ssc#S40288802	black	140.2	17.95	0.753888	0.14103	141.052
gnl|UG|Ssc#S40290915	turquoise	1441.9	1441.44	0.976391	0.004339	6.31
gnl|UG|Ssc#S40291311	blue	346.59	114.742	0.926242	0.023779	12.25
gnl|UG|Ssc#S40291735	black	242.08	36.3866	0.939469	0.017714	28.58

gnl|UG|Ssc#S40292473	turquoise	1051.9	1050.13	0.900905	0.036885	6.71
gnl|UG|Ssc#S40295633	turquoise	1524.8	1524.32	0.992609	0.000762	0.996
gnl|UG|Ssc#S40301371	turquoise	687.45	686.417	0.820038	0.089129	16.202
gnl|UG|Ssc#S40312675	blue	312.7	146.085	0.981612	0.002985	28.038
gnl|UG|Ssc#S40315766	blue	336.96	120.421	0.936764	0.018907	32.216
gnl|UG|Ssc#S40318462	darkred	32.198	1.7376	0.20024	0.746761	242.476
gnl|UG|Ssc#S40322346	black	277.57	42.7209	0.989554	0.00128	128.266
gnl|UG|Ssc#S40323161	brown	251.81	127.682	0.995382	0.000376	10.086
gnl|UG|Ssc#S40324300	turquoise	144.97	132.367	0.44542	0.452229	42.096
gnl|UG|Ssc#S40327466	turquoise	1554.6	1554.24	0.997079	0.000189	4.044
gnl|UG|Ssc#S40327511	turquoise	499.4	493.874	0.720676	0.169592	4.056
gnl|UG|Ssc#S40329149	turquoise	1147.9	1146.12	0.929734	0.022122	19.584
gnl|UG|Ssc#S40330262	turquoise	1554.1	1553.73	0.996738	0.000224	73.226
gnl|UG|Ssc#S40334339	turquoise	1460.9	1460.49	0.982577	0.002753	11.858
gnl|UG|Ssc#S40334954	blue	272.44	131.513	0.956888	0.010676	58.798
gnl|UG|Ssc#S40335524	navy	270.38	32.8698	0.984972	0.002206	26.046
gnl|UG|Ssc#S40335680	turquoise	1058.4	1057.34	0.913083	0.030356	3.91
gnl|UG|Ssc#S40335794	turquoise	1561.8	1561.39	0.997884	0.000117	4.382
gnl|UG|Ssc#S40336522	turquoise	1534.2	1533.86	0.99344	0.000637	4.15
gnl|UG|Ssc#S40340051	turquoise	1364.9	1363.86	0.967429	0.007022	23.636
gnl|UG|Ssc#S40340827	turquoise	198.94	182.465	0.504444	0.38611	52.56
gnl|UG|Ssc#S40349544	turquoise	1571.2	1570.83	0.999632	8.46E-06	116.172
gnl|UG|Ssc#S40356278	turquoise	1506.4	1506.03	0.989978	0.001203	16.836
gnl|UG|Ssc#S40358887	brown	272.91	126.619	0.988816	0.001417	4.398
gnl|UG|Ssc#S40367565	turquoise	1070.5	1069.29	0.903723	0.035338	5.012
gnl|UG|Ssc#S40370377	lightcyan	304.77	25.269	0.978239	0.003841	127.348
gnl|UG|Ssc#S40376380	lightcyan	226.28	18.4389	0.896457	0.039369	22.354
gnl|UG|Ssc#S40377740	midnightblue	264.75	34.0263	0.980838	0.003175	214.666
gnl|UG|Ssc#S40385100	turquoise	600.97	597.452	0.766707	0.130428	18.302
gnl|UG|Ssc#S40386137	black	294.94	39.4413	0.962282	0.008743	25.588
gnl|UG|Ssc#S40388648	navy	151.42	16.5793	0.826436	0.084505	0.664
gnl|UG|Ssc#S40389825	turquoise	1553.7	1553.24	0.998148	9.56E-05	11.556
gnl|UG|Ssc#S40390861	darkgreen	221.1	8.1352	0.756068	0.13921	143.678
gnl|UG|Ssc#S40396824	pink	227.28	47.4927	0.996522	0.000246	46.79
gnl|UG|Ssc#S40397505	turquoise	1537.8	1537.41	0.994007	0.000556	3.208
gnl|UG|Ssc#S40400100	purple	83.621	6.62668	0.589037	0.295981	0.854
gnl|UG|Ssc#S40400469	turquoise	1352.1	1351.08	0.966403	0.007355	2.116
gnl|UG|Ssc#S40401313	tan	282.84	37.8877	0.982277	0.002825	945.06
gnl|UG|Ssc#S40403544	turquoise	1553.6	1553.18	0.996528	0.000245	0.426
gnl|UG|Ssc#S40406739	blue	271.27	110.761	0.918297	0.027688	50.392
gnl|UG|Ssc#S40407699	turquoise	477.62	472.676	0.714253	0.175289	19.302
gnl|UG|Ssc#S40414470	salmon	125.5	27.0083	0.958515	0.01008	59.78
gnl|UG|Ssc#S40416565	turquoise	1539.8	1539.37	0.994867	0.000441	6.59
gnl|UG|Ssc#S40416576	blue	346.03	125.874	0.947023	0.014521	6.818
gnl|UG|Ssc#S40424734	brown	236.46	129.447	0.999539	1.19E-05	33.396
gnl|UG|Ssc#S40426021	darkred	62.86	11.8891	0.909178	0.032405	34.334
gnl|UG|Ssc#S40426329	turquoise	741.13	737.135	0.831483	0.08091	38.78
gnl|UG|Ssc#S40427363	brown	254.72	129.822	0.998196	9.20E-05	12.6

gnl|UG|Ssc#S40429450	turquoise	1498.2	1497.62	0.988803	0.00142	2.834
gnl|UG|Ssc#S40429630	turquoise	1555.5	1555.08	0.996829	0.000214	14.592
gnl|UG|Ssc#S40430442	blue	326.16	147.547	0.983784	0.002473	8.286
gnl|UG|Ssc#S40433705	blue	330.78	135.383	0.964025	0.008147	16.148
gnl|UG|Ssc#S40435051	turquoise	1488.4	1487.81	0.988991	0.001384	0.694
gnl|UG|Ssc#S40437142	brown	244.46	112.48	0.964481	0.007993	6.042
gnl|UG|Ssc#S40437350	black	294.18	35.5007	0.931795	0.021162	65.074
gnl|UG|Ssc#S40437415	turquoise	739.35	736.61	0.815475	0.092473	28.142
gnl|UG|Ssc#S40437556	darkgreen	309.13	22.2115	0.985897	0.002006	18.598
gnl|UG|Ssc#S40438133	turquoise	1504	1503.59	0.987792	0.001616	10.156
gnl|UG|Ssc#S40438408	yellow	213.43	73.8456	0.967481	0.007005	47.9
gnl|UG|Ssc#S40439319	turquoise	1506.6	1506.23	0.988665	0.001446	17.45
gnl|UG|Ssc#S40439733	turquoise	453.58	448.946	0.713077	0.176338	16.924
gnl|UG|Ssc#S40440480	darkred	78.144	11.4817	0.858681	0.062403	253.192
gnl|UG|Ssc#S40440719	turquoise	387.65	379.653	0.672841	0.213264	13.286
gnl|UG|Ssc#S40441928	turquoise	1231.9	1230.3	0.943096	0.016155	0.784
gnl|UG|Ssc#S40442316	green	159.1	30.8691	0.825108	0.085458	85.37
gnl|UG|Ssc#S40442649	turquoise	1553.6	1553.18	0.996528	0.000245	1.04
gnl|UG|Ssc#S40442878	navy	220.27	28.2332	0.955066	0.011357	107.69
gnl|UG|Ssc#S40443043	turquoise	1555.8	1555.34	0.998131	9.70E-05	6.88
gnl|UG|Ssc#S40443068	tan	326.64	37.5364	0.978635	0.003737	165.472
gnl|UG|Ssc#S40443177	turquoise	1545.1	1544.73	0.995811	0.000325	1.492
gnl|UG|Ssc#S40443438	turquoise	421.04	402.422	0.669096	0.2168	91.56
gnl|UG|Ssc#S40452557	turquoise	1516.5	1516.06	0.992847	0.000725	7.322
gnl|UG|Ssc#S40453605	brown	204.75	118.162	0.976965	0.004182	10.562
gnl|UG|Ssc#S40463925	turquoise	1437.7	1437.27	0.977646	0.003999	7.082
gnl|UG|Ssc#S40464521	turquoise	1507.1	1506.71	0.989505	0.001289	18.556
gnl|UG|Ssc#S40466766	turquoise	1492.4	1491.94	0.987708	0.001633	6.384
gnl|UG|Ssc#S40471332	royalblue	262.36	21.3006	0.945486	0.015153	6.64
gnl|UG|Ssc#S40471679	turquoise	296.64	272.11	0.581551	0.303709	12.918
gnl|UG|Ssc#S40471826	turquoise	1558.5	1558.07	0.997635	0.000138	22.148
gnl|UG|Ssc#S40472465	turquoise	34.304	24.4605	0.161854	0.794824	27.974
gnl|UG|Ssc#S40474141	blue	288.95	139.203	0.970517	0.00605	60.248
gnl|UG|Ssc#S40474257	turquoise	1496.1	1495.71	0.986645	0.001849	11.59
gnl|UG|Ssc#S40474761	blue	280.31	140.047	0.971726	0.005683	7.862
gnl|UG|Ssc#S40474839	turquoise	207.35	183.051	0.487454	0.404893	10.9
gnl|UG|Ssc#S40475158	turquoise	267.85	252.156	0.577142	0.308285	21.038
gnl|UG|Ssc#S40475244	midnightblue	192.07	33.8993	0.980254	0.003321	4.588
gnl|UG|Ssc#S40475596	pink	213.71	45.6019	0.98803	0.001569	12.708
gnl|UG|Ssc#S40475805	turquoise	604.12	598.501	0.778794	0.120661	149.58
gnl|UG|Ssc#S40476333	blue	201.07	88.2975	0.868289	0.056234	209.32
gnl|UG|Ssc#S40476889	brown	308.28	105.086	0.939177	0.017842	35.584
gnl|UG|Ssc#S40477070	turquoise	91.733	82.11	0.382452	0.525194	1.704
gnl|UG|Ssc#S40478259	brown	264.42	128.121	0.993552	0.000621	162.468
gnl|UG|Ssc#S40478547	yellow	248.22	73.6791	0.95727	0.010535	22.266
gnl|UG|Ssc#S40479246	turquoise	1529.9	1529.39	0.994093	0.000545	20.458
gnl|UG|Ssc#S40481010	brown	263.16	116.099	0.971693	0.005693	33.694
gnl|UG|Ssc#S40481152	turquoise	1499.4	1498.83	0.990512	0.001108	6.588

gnl|UG|Ssc#S40481511	yellow	245.81	72.3009	0.954022	0.011753	120.762
gnl|UG|Ssc#S40482232	yellow	124.71	58.6745	0.900406	0.037161	129.286
gnl|UG|Ssc#S40482312	turquoise	476.24	461.922	0.707001	0.181785	56.846
gnl|UG|Ssc#S40482366	turquoise	1447.9	1447.5	0.981431	0.003029	1.002
gnl|UG|Ssc#S40482424	turquoise	346	334.529	0.651879	0.233258	41.514
gnl|UG|Ssc#S40482470	tan	329.43	36.6797	0.971843	0.005648	21.732
gnl|UG|Ssc#S40482579	turquoise	300.45	289.774	0.630319	0.254325	79.13
gnl|UG|Ssc#S40482663	turquoise	1508.5	1508.12	0.991046	0.001016	4.972
gnl|UG|Ssc#S40482883	turquoise	1107.5	1105.98	0.922676	0.025509	47.644
gnl|UG|Ssc#S40483013	turquoise	1559.4	1558.99	0.998855	4.65E-05	8.982
gnl|UG|Ssc#S40483020	brown	243.65	125.516	0.990196	0.001164	9.46
gnl|UG|Ssc#S40483452	turquoise	1269.7	1268.56	0.950496	0.013123	1.212
gnl|UG|Ssc#S40483502	turquoise	1454.1	1453.43	0.982286	0.002823	9.59
gnl|UG|Ssc#S40483512	turquoise	1490.3	1489.78	0.988382	0.001501	3.476
gnl|UG|Ssc#S40483811	turquoise	1463.6	1463.05	0.982207	0.002841	3.978
gnl|UG|Ssc#S40485684	lightyellow	200.97	12.0732	0.802369	0.102283	13.692
gnl|UG|Ssc#S40486793	blue	302.55	118.963	0.934219	0.020052	3.638
gnl|UG|Ssc#S40488267	turquoise	281.16	277.584	0.6313	0.253356	5.492
gnl|UG|Ssc#S40490801	turquoise	1541.4	1540.97	0.994682	0.000465	3.464
gnl|UG|Ssc#S40490912	turquoise	1437.3	1436.86	0.977166	0.004128	3.634
gnl|UG|Ssc#S40491599	turquoise	1379.2	1378.39	0.967358	0.007045	3.916
gnl|UG|Ssc#S40494040	brown	258.44	129.13	0.996088	0.000294	11.696
gnl|UG|Ssc#S40494112	blue	325.05	148.78	0.985823	0.002022	29.972
gnl|UG|Ssc#S40494421	magenta	321.66	44.4227	0.993539	0.000623	43.39
gnl|UG|Ssc#S40495537	midnightblue	206.93	34.0433	0.981972	0.002898	132.254
gnl|UG|Ssc#S40496270	brown	219.21	124.691	0.99142	0.000953	46.564
gnl|UG|Ssc#S40496968	brown	108.66	50.9032	0.763423	0.133121	8.456
gnl|UG|Ssc#S40499571	turquoise	150.25	138.995	0.479099	0.414206	0.998
gnl|UG|Ssc#S40500401	midnightblue	245.1	36.625	0.996777	0.00022	29.784
gnl|UG|Ssc#S40500672	yellow	107.79	33.0219	0.731287	0.160301	466.84
gnl|UG|Ssc#S40500952	black	288.92	39.9427	0.961517	0.00901	107.52
gnl|UG|Ssc#S40505966	turquoise	1507	1506.37	0.991554	0.000931	21.592
gnl|UG|Ssc#S40509095	blue	318.98	139.794	0.971557	0.005734	7.144
gnl|UG|Ssc#S40512983	grey60	241.08	31.9559	0.989706	0.001252	13.77
gnl|UG|Ssc#S40520406	turquoise	1546.8	1546.37	0.995937	0.000311	18.762
gnl|UG|Ssc#S40522263	turquoise	1393	1392.11	0.974331	0.004918	108.814
gnl|UG|Ssc#S40524341	turquoise	208.7	149.665	0.385832	0.52122	18.882
gnl|UG|Ssc#S40524913	green	94.204	15.7309	0.657926	0.22744	100.444
gnl|UG|Ssc#S40527077	brown	245.52	129.8	0.99925	2.46E-05	10.638
gnl|UG|Ssc#S40527391	midnightblue	149.73	27.0386	0.932377	0.020894	28.236
gnl|UG|Ssc#S40527867	green	184.24	51.1362	0.985766	0.002034	6.222
gnl|UG|Ssc#S40529248	green	202.84	51.2767	0.976232	0.004383	182.3
gnl|UG|Ssc#S40543017	turquoise	1510.7	1510.32	0.989576	0.001276	3.364
gnl|UG|Ssc#S40546869	red	192.96	47.3713	0.976259	0.004376	35.772
gnl|UG|Ssc#S40546896	turquoise	580.85	576.58	0.76235	0.134005	0.236
gnl|UG|Ssc#S40550337	turquoise	1348.2	1347.35	0.964742	0.007905	67.512
gnl|UG|Ssc#S40551411	turquoise	1212.9	1211.69	0.938933	0.017948	24.136
gnl|UG|Ssc#S40553929	darkturquoise	159.01	12.1635	0.898969	0.03796	73.27

gnl|UG|Ssc#S40554875	yellow	312.42	58.4113	0.874773	0.052185	43.798
gnl|UG|Ssc#S40558823	turquoise	1536	1535.44	0.995651	0.000344	5.24
gnl|UG|Ssc#S40559315	yellow	157.77	35.0979	0.735686	0.156495	8.228
gnl|UG|Ssc#S40564541	turquoise	669.74	664.513	0.804585	0.100603	18.048
gnl|UG|Ssc#S40570043	turquoise	1012.6	1010.58	0.891992	0.041913	29.098
gnl|UG|Ssc#S40571777	turquoise	1208.9	1207.75	0.934121	0.020096	9.814
gnl|UG|Ssc#S40573713	turquoise	1439.3	1438.56	0.979949	0.003398	12.98
gnl|UG|Ssc#S40576057	turquoise	1552.9	1552.49	0.996255	0.000275	5.112
gnl|UG|Ssc#S40577963	blue	260.44	125.781	0.947117	0.014482	16.19
gnl|UG|Ssc#S40583885	royalblue	237.9	23.0748	0.966999	0.007161	21.662
gnl|UG|Ssc#S40584531	grey	284.1	15.4295	0.923809	0.024955	25.876
gnl|UG|Ssc#S40588179	turquoise	1400.9	1400.48	0.973133	0.005265	36.762
gnl|UG|Ssc#S40588301	blue	244.13	112.516	0.922596	0.025549	665.198
gnl|UG|Ssc#S40588989	green	196.82	50.2578	0.966981	0.007167	17.716
gnl|UG|Ssc#S40591094	turquoise	1340.6	1339.57	0.965328	0.00771	37.134
gnl|UG|Ssc#S41445227	turquoise	1553.6	1553.18	0.996528	0.000245	0.52
gnl|UG|Ssc#S41446009	turquoise	597.95	591.68	0.777527	0.121674	32.426
gnl|UG|Ssc#S41446582	turquoise	1507.8	1507.19	0.991657	0.000914	49.088
gnl|UG|Ssc#S41575095	turquoise	720.93	717.938	0.815631	0.092358	17.872
gnl|UG|Ssc#S41575462	purple	167.31	28.044	0.92989	0.022049	77.28
gnl|UG|Ssc#S41577006	turquoise	1500.8	1500.39	0.989246	0.001337	4.664
gnl|UG|Ssc#S41577334	turquoise	347.04	323.462	0.616868	0.267713	9.438
gnl|UG|Ssc#S41578369	turquoise	1504.7	1504.15	0.99033	0.00114	2.006
gnl|UG|Ssc#S43495814	turquoise	1556.6	1556.26	0.997009	0.000196	4.152
gnl|UG|Ssc#S46917266	red	181.97	46.5952	0.972316	0.005506	2.452
gnl|UG|Ssc#S5983468	blue	202.01	75.5611	0.833929	0.079186	12.67
gnl|UG|Ssc#S5984485	darkred	68.291	7.02903	0.683914	0.202906	5.038
gnl|UG|Ssc#S5985980	turquoise	1539.2	1538.78	0.994424	0.000499	3.456
gnl|UG|Ssc#S5991729	turquoise	1322.5	1321.42	0.957995	0.010269	4.252
gnl|UG|Ssc#S5992815	yellow	256.15	80.416	0.977127	0.004138	28.488
gnl|UG|Ssc#S5993568	blue	286.86	123.354	0.942694	0.016326	6.196
gnl|UG|Ssc#S5995726	turquoise	1553.7	1553.28	0.997423	0.000157	9.342
gnl|UG|Ssc#S5997273	magenta	288.48	37.2472	0.955419	0.011224	6.304
gnl|UG|Ssc#S5999567	blue	274.99	113.618	0.924969	0.024392	14.356
gnl|UG|Ssc#S5999689	brown	282.99	106.894	0.94016	0.017413	4.786
gnl|UG|Ssc#S5999899	blue	305.02	148.666	0.985436	0.002105	27.488
gnl|UG|Ssc#S6001862	turquoise	1439.9	1439.25	0.97972	0.003456	0.938
gnl|UG|Ssc#S6002926	turquoise	317.84	293.435	0.596122	0.288714	2.41
gnl|UG|Ssc#S6002981	tan	245.38	34.4558	0.961268	0.009097	61.35
gnl|UG|Ssc#S6003000	turquoise	1518.8	1518.35	0.992219	0.000823	4.022
gnl|UG|Ssc#S6006110	turquoise	1389.8	1389.17	0.974617	0.004836	5.122
gnl|UG|Ssc#S6007055	yellow	297.59	72.4965	0.944403	0.015605	31.402
gnl|UG|Ssc#S6008489	lightgreen	99.193	7.88784	0.430735	0.469036	12.308
gnl|UG|Ssc#S6009554	turquoise	1488.4	1487.97	0.986346	0.001911	4.656
gnl|UG|Ssc#S6010002	turquoise	1539	1538.54	0.995751	0.000332	2.802
gnl|UG|Ssc#S6010568	turquoise	1537.5	1537.06	0.994344	0.00051	3.24
gnl|UG|Ssc#S6011299	darkred	90.36	11.431	0.90578	0.034223	66.704
gnl|UG|Ssc#S6013604	brown	276.13	122.716	0.981836	0.002931	10.628

gnl|UG|Ssc#S6014656	black	278.17	42.9004	0.993392	0.000644	42.28
gnl|UG|Ssc#S6015201	turquoise	1479.9	1479.26	0.986803	0.001816	10.62
gnl|UG|Ssc#S6016211	turquoise	1556.5	1556.1	0.9978	0.000124	5.442
gnl|UG|Ssc#S6024301	turquoise	1486.7	1486.14	0.988724	0.001435	5.51
gnl|UG|Ssc#S6027323	turquoise	1553.6	1553.18	0.996528	0.000245	0.378
gnl|UG|Ssc#S6029367	turquoise	1452.2	1451.6	0.982985	0.002658	18.978
gnl|UG|Ssc#S6029610	lightgreen	108.55	6.68126	0.417058	0.484808	53.362
gnl|UG|Ssc#S6030301	brown	246.52	129.003	0.997403	0.000159	3.246
gnl|UG|Ssc#S6032057	red	228.2	50.6379	0.989303	0.001326	3.036
gnl|UG|Ssc#S6040545	turquoise	1494.8	1494.27	0.989551	0.00128	6.682
gnl|UG|Ssc#S6043533	turquoise	1553.6	1553.18	0.996528	0.000245	0.472
gnl|UG|Ssc#S6047303	green	253.24	50.0809	0.957932	0.010292	74.176
gnl|UG|Ssc#S6048180	turquoise	1553.6	1553.18	0.996528	0.000245	0.52
gnl|UG|Ssc#S6048273	turquoise	1553.6	1553.18	0.996528	0.000245	0.614
gnl|UG|Ssc#S6048306	turquoise	1423.2	1422.65	0.978755	0.003705	4.038
gnl|UG|Ssc#S6048475	turquoise	324.2	302.819	0.607456	0.27719	15.898
gnl|UG|Ssc#S6052686	turquoise	1363.8	1363.19	0.966194	0.007423	4.446
gnl|UG|Ssc#S6053423	turquoise	1553.6	1553.18	0.996528	0.000245	0.284
gnl|UG|Ssc#S6054625	turquoise	1473.9	1473.32	0.985085	0.002182	7.396
gnl|UG|Ssc#S6055465	brown	198.44	105.316	0.946354	0.014795	6.576
gnl|UG|Ssc#S6055548	turquoise	1330.5	1329.58	0.963728	0.008247	11.1
gnl|UG|Ssc#S6056024	turquoise	1539.2	1538.66	0.996114	0.000291	13.406
gnl|UG|Ssc#S6057322	greenyellow	177.45	22.4513	0.986104	0.001962	8.732
gnl|UG|Ssc#S6057912	turquoise	1531.3	1530.95	0.992497	0.000779	4.01
gnl|UG|Ssc#S6058133	blue	344.9	135.106	0.964614	0.007948	11.388
gnl|UG|Ssc#S6058262	magenta	317.05	43.8924	0.991297	0.000973	10.516
gnl|UG|Ssc#S6058884	turquoise	306.53	284.803	0.586904	0.298177	23.678
gnl|UG|Ssc#S6059254	green	278.21	46.1874	0.939623	0.017647	57.516
gnl|UG|Ssc#S6060797	turquoise	1514.7	1514.33	0.991395	0.000957	6.146
gnl|UG|Ssc#S6062152	turquoise	1500.2	1499.52	0.990504	0.001109	2.01
gnl|UG|Ssc#S6063101	brown	249.34	126.946	0.992908	0.000716	10.162
gnl|UG|Ssc#S6063275	turquoise	1387.5	1386.62	0.970267	0.006127	10.096
gnl|UG|Ssc#S6063458	yellow	270.02	72.0451	0.938044	0.018339	71.956
gnl|UG|Ssc#S6064423	turquoise	1407.1	1406.54	0.972886	0.005338	16.772
gnl|UG|Ssc#S6065016	greenyellow	185.41	22.5096	0.971464	0.005762	10.262
gnl|UG|Ssc#S6065178	turquoise	1146	1145.09	0.923391	0.02516	6.458
gnl|UG|Ssc#S6072016	brown	223.43	124.436	0.989763	0.001241	9.148
gnl|UG|Ssc#S6072575	turquoise	1203	1201.58	0.941799	0.016707	17.584
gnl|UG|Ssc#S6072690	turquoise	1383.8	1383.3	0.96653	0.007314	4.948
gnl|UG|Ssc#S6072825	turquoise	1312.9	1311.77	0.960443	0.009388	60.81
gnl|UG|Ssc#S6072888	turquoise	958.41	956.025	0.890832	0.042583	8.142
gnl|UG|Ssc#S6072982	turquoise	1283.1	1282.26	0.9471	0.014489	10.258
gnl|UG|Ssc#S6072986	midnightblue	235.4	35.415	0.98968	0.001257	30.484
gnl|UG|Ssc#S6073003	lightgreen	82.115	7.65972	0.504154	0.386429	23.632
gnl|UG|Ssc#S6073264	turquoise	1555.4	1555	0.997222	0.000176	16.82
gnl|UG|Ssc#S6073842	turquoise	1483.1	1482.61	0.986061	0.001971	1.956
gnl|UG|Ssc#S6073975	purple	188.11	33.0987	0.976554	0.004294	58.842
gnl|UG|Ssc#S6074397	turquoise	1489.5	1488.93	0.987027	0.00177	5.206

gnl|UG|Ssc#S6074613	turquoise	951.05	949.36	0.87393	0.052706	8.984
gnl|UG|Ssc#S6075066	turquoise	160.14	113.253	0.335049	0.581524	77.348
gnl|UG|Ssc#S6075552	red	244.47	42.084	0.945668	0.015078	5.082
gnl|UG|Ssc#S6075829	navy	180.93	25.4645	0.931869	0.021128	61.414
gnl|UG|Ssc#S6076069	turquoise	1421	1420.37	0.97725	0.004105	1.906
gnl|UG|Ssc#S6076809	turquoise	1152.5	1151.25	0.929854	0.022066	35.704
gnl|UG|Ssc#S6076838	turquoise	1439.8	1439.07	0.978493	0.003774	17.7
gnl|UG|Ssc#S6076883	turquoise	1511	1510.57	0.990415	0.001125	2.748
gnl|UG|Ssc#S6076898	turquoise	619.27	613.935	0.789326	0.112338	64.932
gnl|UG|Ssc#S6076952	turquoise	1312	1310.7	0.958594	0.010051	10.206
gnl|UG|Ssc#S6077016	turquoise	1522.1	1521.67	0.993125	0.000684	3.018
gnl|UG|Ssc#S6077174	turquoise	1231.1	1230.25	0.938173	0.018282	4.846
gnl|UG|Ssc#S6077334	greenyellow	77.805	5.21376	-0.60897	0.275655	12.686
gnl|UG|Ssc#S6077926	turquoise	707.35	699.611	0.805513	0.099902	0.548
gnl|UG|Ssc#S6081209	turquoise	1551.9	1551.48	0.996164	0.000285	26.942
gnl|UG|Ssc#S6081340	greenyellow	146.15	19.2432	0.938039	0.018341	5.694
gnl|UG|Ssc#S6081526	blue	278.17	136.298	0.965421	0.007679	41.128
gnl|UG|Ssc#S6082353	turquoise	1256	1254.65	0.950449	0.013142	6.266
gnl|UG|Ssc#S6082550	turquoise	1524.3	1523.7	0.993039	0.000696	15.826
gnl|UG|Ssc#S6083832	turquoise	1499.1	1498.69	0.988443	0.001489	4.768
gnl|UG|Ssc#S6085378	pink	281.47	38.1859	0.942468	0.016421	3.952
gnl|UG|Ssc#S6085466	pink	125.02	31.0666	0.898153	0.038416	9.926
gnl|UG|Ssc#S6085531	pink	221.68	46.5978	0.993165	0.000678	12.692
gnl|UG|Ssc#S6085536	yellow	262.71	74.4364	0.948503	0.013919	59.642
gnl|UG|Ssc#S6085659	brown	259.14	129.071	0.996112	0.000291	12.012
gnl|UG|Ssc#S6085710	yellow	179.48	76.1332	0.969299	0.006428	27.354
gnl|UG|Ssc#S6085740	green	254	50.4641	0.962187	0.008776	25.528
gnl|UG|Ssc#S6089318	turquoise	1515.5	1515.16	0.989695	0.001254	3.792
gnl|UG|Ssc#S6665509	navy	252.38	27.816	0.951182	0.012853	20.24
gnl|UG|Ssc#S6666043	turquoise	1386.1	1385.19	0.972653	0.005406	8.006
gnl|UG|Ssc#S6666060	magenta	280.44	33.4625	0.933413	0.020419	10.688
gnl|UG|Ssc#S6666342	navy	257.9	28.6725	0.958322	0.01015	22.29
gnl|UG|Ssc#S6666554	lightcyan	271.53	22.5224	0.954638	0.011518	19.592

Table S2-2 Attributes of genes in all modules of LDE postnatal.

GeneSymbol	Module	kTotal	kWithin	eigencorr	eigenpval	meanExpr
gi|10304379|gb|AF288822.1|AF28882:	black	781.52979	107.52506	0.9971711	0.00018054	50.062
gi|106073315|gb|DQ508264.1|	pink	824.19918	143.39123	0.9911071	0.00100535	6.784
gi|108796051|ref|NM_213912.2|	salmon	220.05951	71.241571	0.9798696	0.0034182	550.324
gi|108796069|ref|NM_001042375.1|	black	808.60078	96.252924	0.9733935	0.00518889	259.792
gi|109639160|ref|NM_001025222.2|	violet	43.14702	5.9621342	-0.763034	0.13344124	1759.048
gi|109639161|ref|NM_001006593.2|	turquoise	373.18633	59.217211	0.0062775	0.99200725	4.792
gi|112181313|ref|NM_214438.2|	grey	538.23284	15.322396	0.9901666	0.00116882	294.976
gi|11276047|gb|AF319661.1|	red	913.65807	125.10588	0.9950036	0.00042363	2.626
gi|113205497|ref|NM_001044527.1|	yellow	150.35371	69.68024	0.8490825	0.0687637	14058.178
gi|113205581|ref|NM_001044532.1|	turquoise	184.81457	42.542882	-0.442367	0.45571192	18.386
gi|113205585|ref|NM_001044535.1|	yellow	272.64186	123.21247	0.9791383	0.00360575	56.038
gi|113205611|ref|NM_001044541.1|	pink	567.90864	103.34024	0.9253224	0.02422108	25.746
gi|113205623|ref|NM_001044545.1|	white	1016.314	38.452198	0.9860314	0.00197766	12.58
gi|113205635|ref|NM_001044548.1|	tan	152.72499	47.837038	0.9969418	0.00020292	6.404
gi|113205649|ref|NM_001044550.1|	greenyellow	57.480542	12.982026	0.3396728	0.57598186	951.642
gi|113205651|ref|NM_001044553.1|	lightyellow	478.67518	64.075082	0.9988779	4.51E-05	30.714
gi|113205653|ref|NM_001044551.1|	navy	334.22517	20.315511	0.8065937	0.09908803	545.134
gi|113205665|ref|NM_001044554.1|	turquoise	176.89003	51.678805	0.5695071	0.31624921	98.064
gi|113205675|ref|NM_001044558.1|	lightyellow	662.91553	54.615389	0.9604463	0.00938688	8.938
gi|113205689|ref|NM_001044559.1|	orange	510.07289	14.60127	0.7071829	0.18162158	17.994
gi|113205765|ref|NM_001044576.1|	blue	711.86186	140.11187	0.9805355	0.00325033	22.344
gi|113205779|ref|NM_001044584.1|	magenta	398.53794	78.779532	0.9733092	0.00521351	33.084
gi|113205811|ref|NM_001044593.1|	white	832.1032	41.122124	0.9361511	0.01918053	325.586
gi|113205825|ref|NM_001044594.1|	magenta	167.36443	60.987984	0.9211055	0.02628431	173.672
gi|113205835|ref|NM_001044599.1|	midnightblue	965.459	77.102599	0.9946174	0.00047366	18.506
gi|113205859|ref|NM_001044605.1|	darkmagenta	82.162091	8.5007178	0.9165536	0.02857154	84.228
gi|113205873|ref|NM_001044606.1|	darkgreen	219.50399	27.761186	0.8745254	0.05233837	422.486
gi|113205877|ref|NM_001044607.1|	tan	252.99227	37.356893	0.9011845	0.03673054	27.002
gi|113205897|ref|NM_001044613.1|	violet	964.24174	45.088618	0.9713101	0.00580829	15.934
gi|113205905|ref|NM_001044614.1|	turquoise	1017.9805	216.70743	0.9449713	0.01536741	172.024
gi|113205907|ref|NM_001044616.1|	green	846.40143	116.13899	0.9979441	0.00011187	80.066
gi|113205909|ref|NM_001044615.1|	royalblue	555.23752	61.481467	0.9901395	0.00117366	6.016
gi|113205921|ref|NM_001044618.1|	navy	55.915099	4.8016707	-0.494954	0.39657543	43.464
gi|113205929|ref|NM_001044620.1|	royalblue	851.19013	51.426216	0.9473713	0.01437837	106.52
gi|114326182|ref|NM_001048072.1|	midnightblue	971.10269	77.173527	0.9947663	0.00045416	35.736
gi|114326213|ref|NM_001048069.1|	darkred	43.152655	1.3347993	-0.03924	0.95005135	20.44
gi|115545542|dbj|AK230657.1|	turquoise	990.65344	217.38321	0.9501349	0.01326641	64.296
gi|115545552|dbj|AK230667.1|	darkgrey	863.30913	50.969493	0.9854376	0.00210491	19.572
gi|115545568|dbj|AK230683.1|	grey60	296.40061	64.17835	0.9829243	0.00267169	19.478
gi|115545600|dbj|AK230715.1|	salmon	186.00121	57.274913	0.9365921	0.01898344	56.482
gi|115545603|dbj|AK230718.1|	yellow	299.72946	126.34284	0.9830424	0.00264407	2.622
gi|115545609|dbj|AK230724.1|	turquoise	939.01878	214.49577	0.9512281	0.0128347	18.306
gi|115545622|dbj|AK230737.1|	royalblue	757.60605	59.225752	0.979929	0.0034031	8.89
gi|115545632|dbj|AK230747.1|	turquoise	155.67202	49.558684	0.4099851	0.49300746	16.238
gi|115545643|dbj|AK230758.1|	green	927.6774	96.905381	0.9556177	0.01114899	22.462
gi|115545669|dbj|AK230784.1|	black	928.8554	96.246948	0.97207	0.00557971	90.554

gi|115545681|dbj|AK230796.1|	tan	142.07647	40.676478	0.9525994	0.01229979	22.922
gi|115545725|dbj|AK234158.1|	darkturquoise	237.74895	45.81015	0.9914675	0.00094492	6.214
gi|115545741|dbj|AK234174.1|	blue	728.1961	115.90482	0.9356782	0.0193926	14.574
gi|115545742|dbj|AK234175.1|	paleturquoise	182.11813	24.577239	0.9607481	0.00928006	29.414
gi|115545749|dbj|AK234182.1|	pink	982.1235	132.54613	0.9742714	0.00493485	10.164
gi|115545756|dbj|AK234189.1|	tan	71.698938	17.700543	0.6298095	0.25482872	7.954
gi|115545757|dbj|AK234190.1|	green	891.05192	114.93836	0.9958962	0.00031539	14.654
gi|115545762|dbj|AK234195.1|	blue	947.68607	140.76706	0.9795875	0.00349015	4.464
gi|115545765|dbj|AK234198.1|	navy	70.554522	9.414729	-0.7455	0.14809816	38.49
gi|115545770|dbj|AK234203.1|	turquoise	184.66545	61.428111	0.5671875	0.31867927	8.586
gi|115545783|dbj|AK234216.1|	red	901.00883	126.87972	0.9980789	0.00010105	4.036
gi|115545816|dbj|AK234249.1|	brown	634.87948	131.2239	0.9931965	0.00067296	19.198
gi|115545821|dbj|AK234254.1|	lightcyan	194.65389	34.376393	0.846848	0.07027169	46.856
gi|115545827|dbj|AK234260.1|	midnightblue	730.25861	69.528167	0.9733939	0.00518877	8.862
gi|115545844|dbj|AK234277.1|	grey60	303.1603	67.689503	0.994859	0.00044215	1790.29
gi|115545853|dbj|AK234286.1|	blue	362.98553	87.239467	0.8741225	0.05258731	41.852
gi|115545868|dbj|AK234301.1|	darkgrey	950.32283	51.950497	0.9887536	0.00142928	9.692
gi|115545894|dbj|AK234327.1|	tan	136.30157	39.068058	0.9438861	0.0158216	38.03
gi|115545904|dbj|AK234337.1|	blue	582.49305	112.97569	0.9330477	0.02058601	13.85
gi|115545921|dbj|AK237362.1|	darkorange	118.44861	30.829499	0.9546205	0.0115251	12.902
gi|115545926|dbj|AK237367.1|	brown	637.97898	130.78303	0.9899955	0.00119942	12.678
gi|115545943|dbj|AK237384.1|	salmon	165.41784	63.75326	0.9577794	0.01034785	2.266
gi|115545949|dbj|AK237390.1|	darkgreen	110.60386	19.544333	0.7771117	0.12200675	54.098
gi|115545955|dbj|AK237396.1|	violet	984.77305	43.774279	0.9596332	0.0096766	7.578
gi|115545973|dbj|AK237414.1|	lightcyan	507.00319	58.869211	0.9857805	0.0020311	206.346
gi|115545984|dbj|AK237425.1|	yellow	239.18885	113.081	0.9602501	0.00945651	23.528
gi|115545987|dbj|AK237428.1|	lightgreen	318.36969	33.757977	0.8930095	0.04132912	11.812
gi|115545992|dbj|AK237433.1|	yellow	310.26963	127.88976	0.9879732	0.00158042	46.362
gi|115546038|dbj|AK237480.1|	brown	646.80119	130.7867	0.9894064	0.00130679	4.69
gi|115546047|dbj|AK237489.1|	navy	100.6716	11.893648	0.6454028	0.23953447	10.17
gi|115546054|dbj|AK237496.1|	purple	212.44848	46.287676	0.8759766	0.05144466	10.888
gi|115546075|dbj|AK237517.1|	purple	233.95098	44.741012	0.8696715	0.05536245	24.31
gi|115546087|dbj|AK237529.1|	turquoise	477.73416	120.96869	0.8201227	0.08906745	37.538
gi|115546117|dbj|AK240359.1|	brown	461.59614	94.693909	0.9163468	0.02867689	14.792
gi|115546125|dbj|AK230834.1|	blue	676.32084	136.62487	0.9761323	0.0044105	21.91
gi|115546138|dbj|AK240376.1|	red	994.74738	115.01611	0.9767856	0.00423109	61.678
gi|115546153|dbj|AK240391.1|	turquoise	907.61434	217.15521	0.9636361	0.0082786	8.424
gi|115546160|dbj|AK240398.1|	red	957.63475	123.36556	0.9917767	0.00089407	9.194
gi|115546163|dbj|AK240401.1|	salmon	223.96547	77.423277	0.9982883	8.50E-05	6.526
gi|115546179|dbj|AK240417.1|	salmon	190.52608	66.465093	0.9665957	0.00729203	22.01
gi|115546190|dbj|AK240428.1|	skyblue	467.24044	44.12717	0.9979418	0.00011206	4.698
gi|115546193|dbj|AK240431.1|	lightcyan	184.99736	35.389858	0.8528371	0.06625283	62.38
gi|115546201|dbj|AK240439.1|	lightyellow	368.61351	60.388182	0.9850428	0.00219096	19.886
gi|115546215|dbj|AK240453.1|	darkgreen	261.53851	40.493139	0.9783952	0.00379968	25.708
gi|115546231|dbj|AK240469.1|	black	724.66219	101.25788	0.9848088	0.00224249	3.702
gi|115546234|dbj|AK240472.1|	turquoise	449.43915	147.73792	0.8994058	0.03771629	9.184
gi|115546241|dbj|AK240479.1|	grey60	296.12371	62.877015	0.9784712	0.00377968	383.234
gi|115546292|dbj|AK240530.1|	turquoise	270.4829	98.050924	0.687239	0.19982413	11.092

gi|115546304|dbj|AK240542.1|	darkgrey	686.16623	39.161297	0.9324194	0.02087446	25.588
gi|115546313|dbj|AK240551.1|	darkred	208.16291	44.67118	0.9811746	0.00309185	7.124
gi|115546324|dbj|AK230843.1|	turquoise	958.1072	212.37857	0.9481362	0.01406769	67.886
gi|115546331|dbj|AK230850.1|	royalblue	592.40451	62.462036	0.9935709	0.00061822	48.964
gi|115546334|dbj|AK230853.1|	darkorange	141.0497	34.923504	0.9938199	0.00058267	53.898
gi|115546337|dbj|AK230856.1|	brown	860.86239	120.47563	0.9649436	0.00783769	9.246
gi|115546371|dbj|AK230890.1|	yellow	217.75383	81.86102	0.8835561	0.04685696	43.832
gi|115546374|dbj|AK230893.1|	blue	695.44537	142.83068	0.9865565	0.00186735	9.226
gi|115546403|dbj|AK230922.1|	red	691.43369	104.57572	0.9586906	0.01001608	4.176
gi|115546406|dbj|AK230925.1|	greenyellow	168.2292	36.688939	0.9367437	0.01891582	15.88
gi|115546428|dbj|AK230947.1|	darkred	137.27511	32.218164	0.894242	0.04062481	6
gi|115546438|dbj|AK230957.1|	brown	373.5783	65.582105	0.8467106	0.07036478	71.408
gi|115546446|dbj|AK230965.1|	turquoise	233.82097	90.902246	0.7367965	0.15553738	48.586
gi|115546452|dbj|AK230971.1|	black	659.18269	98.804635	0.9792114	0.00358684	11.158
gi|115546465|dbj|AK230984.1|	magenta	365.16186	83.277461	0.9880901	0.00155746	66.902
gi|115546477|dbj|AK230996.1|	turquoise	185.0763	72.569949	0.6082714	0.27636468	25.204
gi|115546487|dbj|AK231006.1|	salmon	227.89498	76.896805	0.9964652	0.00025215	13.18
gi|115546495|dbj|AK231014.1|	midnightblue	892.09164	75.347843	0.9901546	0.00117096	3.58
gi|115546548|dbj|AK234386.1|	blue	391.58717	90.253028	0.883185	0.04707839	16.108
gi|115546569|dbj|AK234407.1|	white	274.49058	16.603383	-0.557773	0.32858983	9.722
gi|115546576|dbj|AK234414.1|	lightyellow	531.9984	61.216026	0.9875728	0.00165991	48.17
gi|115546586|dbj|AK234424.1|	navy	83.428461	7.6488472	-0.928895	0.02251612	175.52
gi|115546610|dbj|AK234448.1|	yellow	220.21979	73.666654	0.8548278	0.06493345	22.672
gi|115546623|dbj|AK234461.1|	brown	695.44263	136.32816	0.9984617	7.24E-05	84.236
gi|115546625|dbj|AK234463.1|	darkgrey	881.36576	48.237538	0.9741307	0.00497528	2.424
gi|115546628|dbj|AK234466.1|	white	809.14679	44.358674	0.9502351	0.01322665	3.76
gi|115546638|dbj|AK234476.1|	turquoise	311.17383	114.98564	0.7412011	0.15175913	9.192
gi|115546656|dbj|AK234494.1|	turquoise	210.70889	79.060669	0.5841978	0.30097062	25.784
gi|115546680|dbj|AK234518.1|	turquoise	1010.6358	198.24435	0.909776	0.03208856	70.592
gi|115546695|dbj|AK234533.1|	skyblue	569.3791	39.61232	0.9731974	0.0052462	64.332
gi|115546696|dbj|AK234534.1|	darkgrey	826.44417	51.242291	0.986597	0.00185893	25.928
gi|115546712|dbj|AK237558.1|	orange	106.29193	23.629062	0.8507658	0.06763438	172.356
gi|115546753|dbj|AK237599.1|	turquoise	352.82481	61.615869	-0.0328	0.95824501	28.9
gi|115546780|dbj|AK237626.1|	navy	748.81425	17.021828	-0.148673	0.81140389	9.702
gi|115546792|dbj|AK237638.1|	turquoise	341.76033	110.37463	0.7783749	0.12099576	11.918
gi|115546833|dbj|AK237679.1|	lightcyan	546.36838	53.451399	0.9558732	0.0110533	14.886
gi|115546838|dbj|AK237684.1|	blue	919.08547	149.86229	0.9941156	0.00054138	11.368
gi|115546849|dbj|AK237695.1|	turquoise	240.48682	94.380353	0.683648	0.20315324	19.862
gi|115546853|dbj|AK237699.1|	blue	312.49155	67.262666	0.8191547	0.08977349	4.73
gi|115546855|dbj|AK237701.1|	black	882.70032	104.50818	0.9904208	0.00112384	5.512
gi|115546868|dbj|AK237714.1|	green	949.46286	110.1111	0.9833484	0.00257294	16.85
gi|115546917|dbj|AK240563.1|	tan	330.94304	33.279754	0.874477	0.05236822	23.84
gi|115546929|dbj|AK240575.1|	turquoise	174.21588	53.304572	0.4304057	0.46941457	25.672
gi|115546934|dbj|AK240580.1|	darkred	184.7832	42.953638	0.9736801	0.0051055	8.318
gi|115546953|dbj|AK240599.1|	red	917.55432	124.98127	0.9948141	0.00044795	11.08
gi|115546978|dbj|AK231033.1|	tan	153.8875	47.460475	0.9926424	0.00075676	28.572
gi|115547033|dbj|AK231088.1|	turquoise	861.34298	202.25917	0.9327838	0.02070697	24.702
gi|115547065|dbj|AK231120.1|	turquoise	169.86096	40.731797	-0.360898	0.55067036	99.518

gi|115547094|dbj|AK231149.1|	turquoise	965.00757	209.96432	0.9366422	0.01896107	11.596
gi|115547117|dbj|AK231172.1|	salmon	241.58119	73.367635	0.9858696	0.00201207	49.874
gi|115547124|dbj|AK231179.1|	black	694.80488	100.28541	0.9828777	0.00268262	12.568
gi|115547136|dbj|AK231191.1|	brown	687.89797	129.69049	0.9911976	0.00099007	8.186
gi|115547142|dbj|AK231197.1|	purple	231.63217	49.806984	0.8902526	0.04291839	426.966
gi|115547167|dbj|AK231222.1|	white	733.66157	41.953524	0.9175074	0.0280872	4.47
gi|115547176|dbj|AK234549.1|	turquoise	240.05238	53.170674	-0.25909	0.67384577	10.75
gi|115547198|dbj|AK234571.1|	darkred	164.72547	38.962617	0.9457875	0.01502865	141.406
gi|115547204|dbj|AK234577.1|	turquoise	732.72945	155.5075	0.8419725	0.07359709	25.618
gi|115547230|dbj|AK234603.1|	brown	423.3036	89.705124	0.9139831	0.02988995	29.552
gi|115547231|dbj|AK234604.1|	brown	712.15792	136.05725	0.9985565	6.58E-05	12.458
gi|115547242|dbj|AK234615.1|	salmon	196.34101	72.028898	0.9836707	0.00249873	6.428
gi|115547251|dbj|AK234624.1|	yellow	224.47031	76.858221	0.8769221	0.05086505	18.496
gi|115547260|dbj|AK234633.1|	darkturquoise	98.46622	17.481082	0.7816602	0.11837824	78.402
gi|115547282|dbj|AK234655.1|	darkorange	208.76194	24.982238	0.9209128	0.0263799	242.45
gi|115547298|dbj|AK234671.1|	pink	941.81911	148.27333	0.9976724	0.00013476	17.092
gi|115547309|dbj|AK234682.1|	red	956.98073	121.00537	0.9876757	0.00163936	0.972
gi|115547320|dbj|AK234693.1|	pink	1006.2733	128.13705	0.9670355	0.00714898	74.428
gi|115547329|dbj|AK234702.1|	pink	885.46441	148.79423	0.9985561	6.58E-05	4.336
gi|115547340|dbj|AK234713.1|	midnightblue	956.81187	73.869123	0.9854316	0.00210621	25.004
gi|115547352|dbj|AK234725.1|	turquoise	429.60205	112.73144	0.7341641	0.15780828	10.472
gi|115547361|dbj|AK234734.1|	green	675.92367	98.500157	0.9633288	0.00838336	60.448
gi|115547381|dbj|AK237762.1|	blue	809.65312	151.67253	0.9987193	5.50E-05	22.28
gi|115547397|dbj|AK237778.1|	pink	812.08636	144.97044	0.993314	0.00065561	7.588
gi|115547403|dbj|AK237784.1|	turquoise	509.47829	159.45749	0.9016381	0.03648049	8.746
gi|115547414|dbj|AK237795.1|	turquoise	138.60537	51.070694	0.5277542	0.360689	61.786
gi|115547436|dbj|AK237818.1|	violet	115.74814	5.3486216	-0.576197	0.30926744	18.896
gi|115547445|dbj|AK237827.1|	royalblue	694.85224	56.743232	0.9727886	0.00536635	67.862
gi|115547450|dbj|AK237832.1|	salmon	210.42726	57.332929	0.9316416	0.02123333	3.486
gi|115547470|dbj|AK237852.1|	white	901.55566	47.113774	0.9760846	0.00442372	4.858
gi|115547485|dbj|AK237867.1|	purple	182.65811	42.505826	0.8628417	0.0597068	52.864
gi|115547488|dbj|AK237870.1|	turquoise	779.21787	192.62506	0.9367797	0.0188998	30.194
gi|115547522|dbj|AK237904.1|	violet	53.9654	8.6722365	-0.846109	0.07077269	82.688
gi|115547541|dbj|AK237923.1|	turquoise	392.52736	131.64488	0.830513	0.08159755	1245.13
gi|115547575|dbj|AK231233.1|	skyblue	566.6924	43.084276	0.9912936	0.00097392	28.652
gi|115547631|dbj|AK231289.1|	grey60	314.19318	68.764166	0.9978174	0.00012236	6.634
gi|115547636|dbj|AK231294.1|	green	500.92988	62.329656	0.8654556	0.05803199	27.126
gi|115547667|dbj|AK231325.1|	blue	954.1155	135.49776	0.9700779	0.00618533	5.398
gi|115547679|dbj|AK231337.1|	darkred	181.08424	37.432567	0.9247751	0.0244858	33.212
gi|115547688|dbj|AK231346.1|	turquoise	315.99337	116.29007	0.8177364	0.09081109	2.528
gi|115547715|dbj|AK231373.1|	red	864.47075	125.78058	0.9963496	0.00026461	4.38
gi|115547725|dbj|AK231383.1|	lightgreen	200.53444	24.622741	0.7405594	0.15230795	1.796
gi|115547754|dbj|AK231412.1|	salmon	162.10628	61.946525	0.9520704	0.01250523	21.722
gi|115547780|dbj|AK234756.1|	darkred	206.78642	40.685702	0.9464369	0.01476086	4.552
gi|115547803|dbj|AK234779.1|	turquoise	934.63163	201.93232	0.9204463	0.02661172	39.108
gi|115547837|dbj|AK234813.1|	pink	850.7406	147.59653	0.9969478	0.00020233	3.17
gi|115547849|dbj|AK234825.1|	turquoise	204.07521	83.049354	0.671306	0.2147115	20.252
gi|115547856|dbj|AK234832.1|	yellow	292.23252	124.2921	0.9790574	0.0036267	14.936

gi|115547861|dbj|AK234837.1|	blue	675.40199	84.039885	0.8661519	0.05758835	22.73
gi|115547869|dbj|AK234845.1|	darkturquoise	220.2516	46.571994	0.9958156	0.00032472	31.752
gi|115547882|dbj|AK234858.1|	tan	235.47206	39.489027	0.9152179	0.0292542	27.976
gi|115547885|dbj|AK234861.1|	brown	167.91349	21.498589	0.603741	0.2809531	19.26
gi|115547886|dbj|AK234862.1|	turquoise	319.52666	100.48316	0.7436805	0.14964432	11.036
gi|115547910|dbj|AK234886.1|	navy	985.23867	18.014461	-0.023754	0.96975892	14.214
gi|115547932|dbj|AK234909.1|	magenta	327.395	79.231913	0.9790986	0.00361601	38.876
gi|115547946|dbj|AK234923.1|	brown	464.63215	92.139214	0.9189576	0.02735592	23.65
gi|115547985|dbj|AK237969.1|	turquoise	734.5487	204.24652	0.963837	0.00821031	24.518
gi|115548000|dbj|AK237984.1|	darkgreen	244.63539	43.616012	0.9972929	0.00016901	38.892
gi|115548002|dbj|AK237986.1|	brown	585.33056	121.94148	0.9785324	0.00376361	171.864
gi|115548018|dbj|AK238002.1|	blue	368.83237	73.520506	0.8400815	0.07489962	11.468
gi|115548040|dbj|AK238024.1|	violet	860.91887	46.4743	0.9948606	0.00044195	9.104
gi|115548067|dbj|AK238051.1|	salmon	212.11877	69.175372	0.9739991	0.00501321	3.396
gi|115548072|dbj|AK238056.1|	turquoise	324.67404	61.071423	-0.078577	0.90005549	31.344
gi|115548078|dbj|AK238062.1|	darkorange	218.81994	11.847756	0.7233809	0.16720947	4.382
gi|115548084|dbj|AK238068.1|	red	478.32021	79.204586	0.9029781	0.035745	7.648
gi|115548129|dbj|AK238113.1|	red	931.76331	126.71449	0.9976172	0.00013957	3.212
gi|115548136|dbj|AK238120.1|	paleturquoise	175.92358	24.136773	0.9444888	0.01556881	6.362
gi|115548176|dbj|AK234948.1|	lightyellow	574.40311	59.963591	0.9824641	0.00278023	143.722
gi|115548199|dbj|AK231509.1|	yellow	255.58259	95.957489	0.9164941	0.02860182	2.134
gi|115548219|dbj|AK231529.1|	darkturquoise	133.47867	38.891689	0.9546875	0.0114997	34.56
gi|115548236|dbj|AK231450.1|	skyblue	479.33463	44.382447	0.999099	3.25E-05	7.714
gi|115548239|dbj|AK231453.1|	purple	293.43219	77.8571	0.9924903	0.00078033	7.62
gi|115548250|dbj|AK231464.1|	darkorange	160.1326	33.722424	0.9899511	0.00120741	2.28
gi|115548260|dbj|AK231474.1|	darkred	212.93198	41.355547	0.9555232	0.01118447	6.53
gi|115548298|dbj|AK234955.1|	yellow	313.31823	129.04798	0.9889041	0.00140072	18.378
gi|115548312|dbj|AK234969.1|	red	893.18817	124.73169	0.9945263	0.00048574	5.554
gi|115548314|dbj|AK234971.1|	turquoise	319.23425	60.757023	-0.105162	0.86635088	15.382
gi|115548316|dbj|AK234973.1|	brown	135.64657	20.953627	0.5641679	0.32184968	27.768
gi|115548319|dbj|AK231543.1|	darkmagenta	124.56805	9.6777558	0.9225106	0.0255908	37.02
gi|115548323|dbj|AK231696.1|	darkred	182.71535	38.243914	0.9303018	0.02185612	5.276
gi|115548328|dbj|AK231701.1|	green	964.35845	106.41499	0.9751526	0.00468414	5.422
gi|115548334|dbj|AK231707.1|	turquoise	704.58848	174.08603	0.9072533	0.03343071	30.756
gi|115548360|dbj|AK231733.1|	blue	917.99076	130.13706	0.9599909	0.00954879	47.492
gi|115548373|dbj|AK234990.1|	red	758.83614	116.28908	0.9804194	0.00327939	1.954
gi|115548383|dbj|AK235000.1|	turquoise	870.88718	203.42942	0.9342765	0.02002564	14.194
gi|115548388|dbj|AK235005.1|	lightcyan	619.88284	46.549259	0.9124757	0.03067196	31.48
gi|115548389|dbj|AK235006.1|	brown	772.68315	132.44867	0.9877241	0.00162972	13.966
gi|115548394|dbj|AK235011.1|	blue	794.15315	132.0859	0.9653698	0.0076957	18.008
gi|115548406|dbj|AK235023.1|	red	945.23832	124.87238	0.9943867	0.00050442	1.358
gi|115548409|dbj|AK235026.1|	brown	150.45337	19.8866	0.527977	0.36044812	15.792
gi|115548413|dbj|AK235030.1|	darkmagenta	275.68302	5.161233	-0.786895	0.1142433	12.144
gi|115548441|dbj|AK235058.1|	orange	324.55326	36.181135	0.9897463	0.00124448	43.698
gi|115548454|dbj|AK235071.1|	lightgreen	402.28732	39.521986	0.9416187	0.01678435	5.144
gi|115548459|dbj|AK235076.1|	red	860.27555	124.4075	0.9940912	0.00054475	3.702
gi|115548467|dbj|AK235084.1|	turquoise	343.26411	108.57626	0.7416417	0.15138271	105.174
gi|115548500|dbj|AK235117.1|	blue	568.94354	102.33673	0.9085462	0.03274066	67.994

gi|115548514|dbj|AK235131.1|	black	912.45622	103.98515	0.989402	0.00130762	40.874
gi|115548519|dbj|AK235136.1|	grey60	314.80173	69.237177	0.9995098	1.30E-05	30.196
gi|115548520|dbj|AK235137.1|	turquoise	286.64289	103.61897	0.694042	0.19355991	36.294
gi|115548526|dbj|AK235143.1|	turquoise	558.19245	173.65392	0.9247881	0.02447948	20.974
gi|115548564|dbj|AK231548.1|	yellow	173.39436	80.015319	0.8817118	0.04796078	145.702
gi|115548572|dbj|AK231556.1|	midnightblue	804.74192	74.363759	0.9874442	0.0016857	34.466
gi|115548574|dbj|AK231558.1|	turquoise	197.13269	70.385347	0.6112601	0.27334863	654.922
gi|115548620|dbj|AK231604.1|	navy	124.60951	35.297913	0.9588067	0.00997407	6.292
gi|115548658|dbj|AK231642.1|	brown	800.97951	124.89943	0.9735354	0.00514754	79.756
gi|115548698|dbj|AK231683.1|	white	213.95273	15.526975	-0.724678	0.16607036	114.27
gi|115548706|dbj|AK231691.1|	brown	650.94478	131.56682	0.9900594	0.00118797	48.782
gi|115548717|dbj|AK231783.1|	royalblue	441.60992	55.055825	0.9647237	0.00791129	352.914
gi|115548727|dbj|AK231793.1|	royalblue	360.4268	46.209979	0.9247981	0.02447465	8.67
gi|115548749|dbj|AK231815.1|	pink	850.58289	148.09877	0.9976633	0.00013555	3.666
gi|115548797|dbj|AK231755.1|	turquoise	375.38579	92.234271	0.7044158	0.18411745	31.79
gi|115548800|dbj|AK231758.1|	greenyellow	85.08938	14.371134	0.4723904	0.42171959	29.016
gi|115548801|dbj|AK231759.1|	yellow	198.26869	90.308823	0.907552	0.03327089	57.926
gi|115548802|dbj|AK231760.1|	lightgreen	537.20814	33.332087	0.8626623	0.05982231	6.364
gi|115548807|dbj|AK231765.1|	yellow	149.44932	62.801265	0.8230331	0.08695486	33.966
gi|115548828|dbj|AK231943.1|	yellow	278.98069	130.60874	0.9927755	0.00073634	22.928
gi|115548831|dbj|AK231946.1|	blue	620.01121	88.998436	0.8843537	0.04638215	14.46
gi|115548849|dbj|AK235252.1|	blue	509.96054	91.505064	0.884307	0.04640987	14.144
gi|115548852|dbj|AK235255.1|	darkturquoise	178.69853	41.238152	0.9664571	0.00733733	62.236
gi|115548861|dbj|AK235264.1|	blue	817.77129	151.6116	0.9981763	9.35E-05	9.648
gi|115548869|dbj|AK235272.1|	paleturquoise	177.97931	23.427078	0.9392895	0.01779241	3.844
gi|115548882|dbj|AK235285.1|	brown	299.5347	41.163192	0.7366916	0.15562772	71.254
gi|115548922|dbj|AK235325.1|	midnightblue	893.48596	76.404608	0.9930992	0.00068743	144.162
gi|115548932|dbj|AK235335.1|	magenta	309.86951	86.33648	0.9989808	3.91E-05	22.164
gi|115548933|dbj|AK235336.1|	blue	885.07598	142.40215	0.9825488	0.00276013	10.612
gi|115548942|dbj|AK235345.1|	yellow	253.40841	114.99263	0.9639104	0.00818542	8.196
gi|115548954|dbj|AK235357.1|	turquoise	391.6864	68.296256	0.5981388	0.28665444	7.896
gi|115548955|dbj|AK235358.1|	darkmagenta	71.667896	6.4582736	0.9118747	0.03098552	56.24
gi|115548956|dbj|AK235359.1|	turquoise	1015.2382	215.19746	0.9409617	0.01706676	8.212
gi|115548992|dbj|AK238181.1|	navy	175.91141	40.603423	0.9786528	0.00373207	15.188
gi|115549006|dbj|AK238195.1|	pink	894.62975	149.62591	0.9996875	6.63E-06	1.668
gi|115549015|dbj|AK238204.1|	turquoise	275.40566	103.01962	0.7326402	0.15912726	3.116
gi|115549057|dbj|AK238246.1|	midnightblue	843.90306	76.158915	0.9921465	0.00083449	14.806
gi|115549078|dbj|AK238267.1|	darkmagenta	72.795114	8.8541451	0.945585	0.01511246	41.586
gi|115549145|dbj|AK238334.1|	midnightblue	916.32396	74.621169	0.988112	0.00155317	5.798
gi|115549177|dbj|AK231853.1|	brown	188.87035	30.521123	0.6483216	0.23670037	16.826
gi|115549179|dbj|AK231855.1|	red	893.15251	126.53179	0.9975624	0.00014442	20.452
gi|115549199|dbj|AK231875.1|	navy	79.081388	36.442145	0.9348258	0.0197768	1.798
gi|115549229|dbj|AK231905.1|	yellow	279.90546	123.3362	0.9780571	0.00388903	49.066
gi|115549239|dbj|AK231915.1|	midnightblue	922.9414	76.303015	0.9925998	0.00076334	42.672
gi|115549243|dbj|AK231919.1|	orange	228.40005	14.902841	0.7379663	0.15453128	25.536
gi|115549251|dbj|AK231927.1|	red	917.20999	127.28631	0.9985982	6.30E-05	3.082
gi|115549258|dbj|AK235178.1|	turquoise	933.8651	204.77151	0.9309569	0.02155088	10.506
gi|115549275|dbj|AK235196.1|	turquoise	881.7846	214.26461	0.965135	0.00777382	21.364

gi|115549311|dbj|AK235232.1|	salmon	191.42077	67.680023	0.9705801	0.00603074	26.928
gi|115549313|dbj|AK235234.1|	midnightblue	624.13906	63.701406	0.9547705	0.01146827	112.428
gi|115549331|dbj|AK232109.1|	darkgreen	102.58573	12.038272	0.645985	0.23896839	376.264
gi|115549349|dbj|AK232127.1|	lightcyan	406.53928	55.005431	0.9769361	0.0041901	52.9
gi|115549356|dbj|AK235378.1|	skyblue	574.15899	42.37714	0.9874395	0.00168665	14.834
gi|115549358|dbj|AK235380.1|	yellow	303.87893	121.15166	0.9738551	0.00505481	15.048
gi|115549368|dbj|AK235390.1|	green	852.74819	116.68908	0.9991979	2.73E-05	5.768
gi|115549412|dbj|AK235434.1|	turquoise	264.22926	90.892143	0.6548244	0.23041955	37.634
gi|115549416|dbj|AK235438.1|	skyblue	627.8844	39.929977	0.973089	0.00527799	2.896
gi|115549428|dbj|AK235450.1|	brown	732.44012	134.8806	0.9969592	0.0002012	72.316
gi|115549440|dbj|AK235462.1|	darkred	126.6215	30.451326	0.8845775	0.04624918	220.136
gi|115549451|dbj|AK235473.1|	turquoise	245.26756	93.538693	0.7388327	0.15378741	11.078
gi|115549474|dbj|AK235496.1|	pink	832.44031	146.16187	0.9950042	0.00042356	13.632
gi|115549475|dbj|AK235497.1|	magenta	264.97688	73.685935	0.9650048	0.00781723	68.216
gi|115549482|dbj|AK235504.1|	greenyellow	197.93696	40.489993	0.9751654	0.00468054	7.56
gi|115549486|dbj|AK235508.1|	red	825.84961	121.2253	0.9889756	0.00138722	5.18
gi|115549499|dbj|AK235521.1|	green	766.58229	113.53533	0.9941312	0.00053923	8.798
gi|115549531|dbj|AK235553.1|	lightcyan	369.34861	57.249648	0.9891367	0.00135695	76.29
gi|115549536|dbj|AK235558.1|	orange	273.82404	36.634663	0.9883787	0.00150127	6.71
gi|115549537|dbj|AK235559.1|	turquoise	961.34628	222.76604	0.9661831	0.00742709	12.178
gi|115549542|dbj|AK235564.1|	tan	164.53376	29.95908	0.8511828	0.06735556	20.284
gi|115549562|dbj|AK238370.1|	white	925.46103	47.282408	0.9784127	0.00379508	5.27
gi|115549583|dbj|AK238391.1|	darkturquoise	241.81328	38.45244	0.9460857	0.0149055	0.818
gi|115549608|dbj|AK238416.1|	lightgreen	468.71915	36.161609	0.896741	0.03920867	20.55
gi|115549613|dbj|AK238421.1|	grey60	282.38046	60.552824	0.9705005	0.00605514	27.794
gi|115549633|dbj|AK238441.1|	magenta	384.31766	76.342445	0.9672064	0.00709366	45.356
gi|115549680|dbj|AK231959.1|	blue	544.92876	111.06323	0.9290949	0.0224221	26.11
gi|115549689|dbj|AK231968.1|	lightgreen	98.144822	12.649494	0.5626059	0.32349277	83.096
gi|115549696|dbj|AK231975.1|	turquoise	166.78553	53.846851	0.4874545	0.40489286	27.91
gi|115549709|dbj|AK231988.1|	orange	235.92661	37.534119	0.9961094	0.00029115	10.068
gi|115549710|dbj|AK231989.1|	turquoise	354.22449	55.984903	-0.07636	0.90287005	18.918
gi|115549744|dbj|AK232023.1|	lightyellow	425.55352	63.275377	0.9956888	0.00033958	135.396
gi|115549779|dbj|AK232059.1|	white	804.51865	43.372335	0.9513661	0.01278051	15.1
gi|115549787|dbj|AK232067.1|	royalblue	580.5174	62.531814	0.9935504	0.00062117	58.134
gi|115549809|dbj|AK232089.1|	turquoise	856.97884	219.76096	0.9786344	0.00373687	22.914
gi|115549835|dbj|AK238495.1|	yellow	250.24382	113.62085	0.9617572	0.00892589	14.206
gi|115549846|dbj|AK238506.1|	greenyellow	45.17311	9.1217382	0.3298287	0.58779263	45.322
gi|115549856|dbj|AK238516.1|	darkorange	69.817336	18.674546	0.7881639	0.11324747	19.05
gi|115549861|dbj|AK238521.1|	blue	548.86835	122.46312	0.950745	0.01302492	3.912
gi|115549895|dbj|AK238555.1|	magenta	127.51338	38.436048	0.8220382	0.08767524	8.488
gi|115549900|dbj|AK232134.1|	darkgreen	216.79004	37.305846	0.9572655	0.01053654	48.654
gi|115549949|dbj|AK232184.1|	brown	805.57416	128.2756	0.9781483	0.00386486	60.502
gi|115549956|dbj|AK232191.1|	violet	949.76045	46.106476	0.9787375	0.00370992	9.14
gi|115549963|dbj|AK232198.1|	turquoise	481.29645	115.89844	0.747602	0.14631725	44.358
gi|115549970|dbj|AK232205.1|	pink	607.88586	116.06899	0.9478517	0.014183	110.26
gi|115549997|dbj|AK232232.1|	turquoise	459.11871	152.11183	0.8807852	0.04851841	2.4
gi|115550002|dbj|AK232237.1|	skyblue	403.37247	42.044621	0.9871741	0.00174031	16.962
gi|115550007|dbj|AK232242.1|	blue	584.02087	109.74198	0.925868	0.02395815	130.588

gi|115550036|dbj|AK232271.1|	blue	742.84607	136.12464	0.9739202	0.00503598	74.618
gi|115550039|dbj|AK232274.1|	lightgreen	366.49073	31.217289	0.9105748	0.03166731	48.472
gi|115550042|dbj|AK232277.1|	yellow	277.63291	110.43175	0.9499479	0.01334074	81.342
gi|115550054|dbj|AK232289.1|	tan	278.99299	26.984922	0.7917388	0.1104565	13.192
gi|115550058|dbj|AK232293.1|	tan	210.8276	39.920621	0.9202752	0.02669692	11.596
gi|115550060|dbj|AK232295.1|	darkgreen	117.7451	15.620672	0.7180664	0.17190025	69.238
gi|115550075|dbj|AK232310.1|	lightyellow	394.23194	57.313863	0.9738829	0.00504676	1.22
gi|115550091|dbj|AK232326.1|	yellow	190.99152	78.152187	0.8746578	0.05225662	3.706
gi|115550101|dbj|AK235580.1|	darkred	202.9249	45.75911	0.983919	0.00244205	27.276
gi|115550102|dbj|AK235581.1|	brown	830.38359	125.61592	0.9733503	0.00520151	7.51
gi|115550120|dbj|AK235599.1|	turquoise	672.55286	169.22028	0.903132	0.03566079	18.678
gi|115550124|dbj|AK235603.1|	darkgreen	100.75848	14.71661	0.6938188	0.19376453	362.496
gi|115550125|dbj|AK235604.1|	tan	169.89846	30.185785	0.8530695	0.06609835	4.514
gi|115550130|dbj|AK235609.1|	salmon	227.49214	77.499973	0.9984969	6.99E-05	11.912
gi|115550133|dbj|AK235612.1|	darkorange	160.96357	29.484245	0.9476757	0.01425444	107.012
gi|115550140|dbj|AK235619.1|	tan	167.5943	44.957379	0.9775538	0.00402328	38.764
gi|115550155|dbj|AK235634.1|	turquoise	413.49795	141.33074	0.8575143	0.06316606	27.342
gi|115550159|dbj|AK235638.1|	yellow	279.88144	130.29897	0.9919463	0.00086656	160.858
gi|115550165|dbj|AK235644.1|	yellow	298.38515	132.59223	0.9960409	0.00029886	53.794
gi|115550185|dbj|AK235664.1|	brown	875.75562	111.85396	0.9460071	0.01493792	8.382
gi|115550186|dbj|AK235665.1|	turquoise	915.15766	219.25791	0.9691271	0.00648155	19.204
gi|115550191|dbj|AK235670.1|	turquoise	796.88328	207.13381	0.9560885	0.01097285	9.544
gi|115550210|dbj|AK235689.1|	greenyellow	98.925732	17.486783	0.7539717	0.14096024	10.884
gi|115550254|dbj|AK235733.1|	tan	80.135582	20.150412	0.693576	0.19398717	28.126
gi|115550257|dbj|AK235736.1|	green	653.21647	103.36732	0.9735785	0.00513502	14.25
gi|115550270|dbj|AK235749.1|	salmon	228.0379	74.954037	0.9907844	0.00106052	8.802
gi|115550272|dbj|AK235751.1|	lightgreen	420.63899	28.600189	0.9045014	0.03491468	64.822
gi|115550273|dbj|AK235752.1|	blue	803.54679	147.94015	0.9926648	0.00075331	21.066
gi|115550292|dbj|AK235771.1|	skyblue	615.18576	40.65116	0.9774938	0.00403938	23.278
gi|115550321|dbj|AK238586.1|	turquoise	228.1312	86.545512	0.6934254	0.19412536	27.97
gi|115550332|dbj|AK238597.1|	pink	665.21487	117.05377	0.9504943	0.01312397	56.998
gi|115550354|dbj|AK238619.1|	tan	86.573439	22.608928	0.7538025	0.14110175	79.598
gi|115550357|dbj|AK238622.1|	green	953.06863	105.22681	0.9720316	0.0055912	31.668
gi|115550392|dbj|AK238657.1|	lightcyan	317.16828	49.616275	0.9550761	0.01135275	16.84
gi|115550394|dbj|AK238659.1|	green	569.23454	92.473347	0.9490696	0.01369158	7.134
gi|115550410|dbj|AK238675.1|	royalblue	673.33093	63.955302	0.9981044	9.90E-05	1.61
gi|115550429|dbj|AK238694.1|	blue	469.00405	73.382986	0.837373	0.07677754	30.546
gi|115550430|dbj|AK238695.1|	brown	732.88351	133.63169	0.9949134	0.00043516	16.026
gi|115550441|dbj|AK238706.1|	navy	93.032488	37.379393	0.9719322	0.00562096	8.602
gi|115550443|dbj|AK238708.1|	turquoise	823.77348	195.30902	0.9364508	0.01904649	14.924
gi|115550448|dbj|AK238713.1|	grey60	277.52959	57.344214	0.9592131	0.00982744	62.19
gi|115550486|dbj|AK238751.1|	red	799.45382	116.31837	0.9806101	0.00323169	2.174
gi|115550510|dbj|AK232340.1|	darkgreen	161.52574	20.927646	0.8035898	0.10135662	9.522
gi|115550524|dbj|AK232354.1|	darkturquoise	154.98372	39.464241	0.9551005	0.01134357	138.668
gi|115550537|dbj|AK232367.1|	yellow	295.07532	129.94319	0.9913982	0.00095643	20.528
gi|115550567|dbj|AK232397.1|	lightyellow	625.03028	58.16862	0.9754209	0.00460867	11.17
gi|115550593|dbj|AK232423.1|	yellow	120.95408	52.772095	0.7879894	0.11338423	157.048
gi|115550595|dbj|AK232425.1|	green	847.50829	113.76254	0.9942156	0.00052765	5.004

gi|115550607|dbj|AK232437.1|	magenta	357.7385	79.119142	0.9767689	0.00423564	25.548
gi|115550650|dbj|AK232480.1|	pink	897.03211	145.90294	0.9945807	0.00047852	1.726
gi|115550656|dbj|AK232486.1|	turquoise	939.56758	217.09557	0.9507537	0.01302145	35.886
gi|115550661|dbj|AK232491.1|	yellow	271.13721	117.26104	0.9664834	0.00732872	21.062
gi|115550668|dbj|AK232498.1|	grey	303.77948	12.231518	0.9453339	0.01521661	3.102
gi|115550708|dbj|AK235782.1|	grey60	307.77368	66.771941	0.9916083	0.00092164	93.34
gi|115550716|dbj|AK235790.1|	orange	191.86713	35.666807	0.9729914	0.00530665	0.206
gi|115550732|dbj|AK235806.1|	lightcyan	268.68699	48.281488	0.9438213	0.01584885	17.412
gi|115550746|dbj|AK235820.1|	paleturquoise	169.19396	21.822245	0.9147418	0.0294988	7.41
gi|115550755|dbj|AK235829.1|	brown	312.27608	58.053511	0.8131073	0.09422292	18.316
gi|115550758|dbj|AK235833.1|	turquoise	350.39271	88.804283	0.6663879	0.21936666	25.958
gi|115550768|dbj|AK235843.1|	turquoise	998.76278	199.9013	0.9147382	0.02950069	21.108
gi|115550785|dbj|AK235860.1|	navy	69.120478	5.8900866	-0.980346	0.00329789	39.074
gi|115550806|dbj|AK235881.1|	green	974.66294	101.40293	0.9635264	0.00831594	20.878
gi|115550812|dbj|AK235887.1|	turquoise	580.2985	151.2698	0.8769572	0.05084356	5.554
gi|115550819|dbj|AK235894.1|	blue	942.68666	139.75541	0.9778837	0.0039351	13.824
gi|115550826|dbj|AK235901.1|	turquoise	531.98155	169.42332	0.9289448	0.0224928	20.074
gi|115550838|dbj|AK235913.1|	purple	279.84486	76.294955	0.9881557	0.00154464	135.132
gi|115550848|dbj|AK235923.1|	darkmagenta	174.61948	3.336137	-0.69683	0.19100872	241.606
gi|115550868|dbj|AK235943.1|	greenyellow	195.50858	40.434969	0.9753845	0.00461889	11.982
gi|115550874|dbj|AK235949.1|	greenyellow	54.188232	11.768751	0.3389664	0.57682797	21.918
gi|115550888|dbj|AK235963.1|	skyblue	505.94778	43.192405	0.9932317	0.00066774	3.788
gi|115550889|dbj|AK235964.1|	tan	150.72261	22.033715	0.7662116	0.13083365	72.794
gi|115550900|dbj|AK238761.1|	turquoise	927.79272	196.81542	0.9146623	0.02953973	19.732
gi|115550915|dbj|AK238776.1|	yellow	272.61364	85.272865	0.8896631	0.04326071	5.222
gi|115550943|dbj|AK238804.1|	black	935.14272	100.55415	0.9819369	0.00290631	12.454
gi|115550946|dbj|AK238807.1|	turquoise	846.50813	208.19947	0.9424616	0.01642427	16.784
gi|115550960|dbj|AK238821.1|	lightyellow	414.97692	63.161152	0.9953056	0.00038583	6.312
gi|115550961|dbj|AK238822.1|	darkgrey	918.82154	50.856267	0.9848677	0.00222949	48.594
gi|115550973|dbj|AK238834.1|	skyblue	290.19128	31.813978	0.9240941	0.02481643	2.6
gi|115550975|dbj|AK238836.1|	blue	522.03415	115.04593	0.9376101	0.01853097	118.318
gi|115550976|dbj|AK238837.1|	red	987.66865	117.18214	0.9808948	0.00316092	35.526
gi|115550989|dbj|AK238850.1|	salmon	217.7294	71.561122	0.9813393	0.00305145	31.154
gi|115550999|dbj|AK238860.1|	darkred	211.56584	45.054652	0.9798357	0.00342681	25.47
gi|115551001|dbj|AK238862.1|	brown	838.30688	113.71395	0.9458798	0.01499048	169.798
gi|115551006|dbj|AK238867.1|	turquoise	905.60228	223.38991	0.9727157	0.00538787	12.41
gi|115551027|dbj|AK238888.1|	brown	582.98627	119.41889	0.974862	0.00476635	8.436
gi|115551063|dbj|AK238924.1|	grey	351.86657	13.394531	0.9656872	0.00759049	23.222
gi|115551074|dbj|AK238935.1|	magenta	268.71584	79.804355	0.9823774	0.00280082	5.076
gi|115551086|dbj|AK238947.1|	pink	880.72335	149.24487	0.9991952	2.74E-05	3.196
gi|115551105|dbj|AK232538.1|	black	728.20574	90.493092	0.9618532	0.00889244	19.392
gi|115551122|dbj|AK232555.1|	orange	450.18809	17.08399	0.7553959	0.1397705	66.85
gi|115551124|dbj|AK232557.1|	purple	303.71604	75.568958	0.9845687	0.00229578	20.778
gi|115551148|dbj|AK232581.1|	magenta	388.79125	78.923121	0.9743697	0.00490667	18.536
gi|115551177|dbj|AK232610.1|	navy	158.74197	33.700394	0.9322764	0.02094025	26.7
gi|115551181|dbj|AK232614.1|	yellow	281.53348	122.80747	0.9769932	0.0041746	49.676
gi|115551189|dbj|AK232622.1|	turquoise	957.5477	208.9591	0.9420683	0.01659195	9.086
gi|115551213|dbj|AK232646.1|	darkgrey	953.25642	52.546867	0.9911276	0.00100189	5.452

gi|115551216|dbj|AK232649.1|	brown	719.65113	134.63897	0.9949185	0.0004345	21.268
gi|115551255|dbj|AK232688.1|	black	777.01254	103.91734	0.9900243	0.00119425	11.382
gi|115551270|dbj|AK232704.1|	navy	432.38052	10.762438	0.0001063	0.99986466	556.554
gi|115551283|dbj|AK232717.1|	brown	526.29194	88.064595	0.9040467	0.03516186	49.422
gi|115551304|dbj|AK235982.1|	midnightblue	811.9423	72.559368	0.9825404	0.00276213	9.6
gi|115551308|dbj|AK235986.1|	tan	122.6802	33.112917	0.8863062	0.04522625	12.688
gi|115551350|dbj|AK236028.1|	turquoise	979.41017	219.66538	0.9522512	0.01243491	6.78
gi|115551358|dbj|AK236036.1|	turquoise	680.193	189.70133	0.9313778	0.02135548	17.47
gi|115551374|dbj|AK236052.1|	midnightblue	892.34143	77.285272	0.9954336	0.00037016	7.484
gi|115551375|dbj|AK236053.1|	blue	782.8505	150.73723	0.9974987	0.00015011	7.59
gi|115551387|dbj|AK236065.1|	brown	731.26947	133.36744	0.9943828	0.00050495	14.732
gi|115551391|dbj|AK236069.1|	green	477.73189	62.650193	0.8661708	0.05757637	10.458
gi|115551397|dbj|AK236075.1|	brown	433.23408	76.501981	0.8744567	0.05238077	48.296
gi|115551408|dbj|AK236086.1|	tan	119.43601	36.60337	0.9051562	0.0345597	88.862
gi|115551416|dbj|AK236094.1|	paleturquoise	178.92902	25.868023	0.9698994	0.0062406	33.626
gi|115551438|dbj|AK236116.1|	lightgreen	204.9924	24.194609	0.7630856	0.13339858	29.284
gi|115551444|dbj|AK236122.1|	turquoise	1013.2528	201.32295	0.915925	0.02889214	2.592
gi|115551449|dbj|AK236127.1|	yellow	212.60967	100.82079	0.9334858	0.02038562	175.8
gi|115551452|dbj|AK236130.1|	turquoise	449.46141	146.39754	0.8801628	0.04889411	16.232
gi|115551471|dbj|AK236149.1|	lightgreen	145.88473	15.974304	0.7797144	0.11992646	20.074
gi|115551473|dbj|AK236151.1|	purple	257.41519	64.629748	0.9498628	0.0133746	6.67
gi|115551475|dbj|AK236153.1|	turquoise	470.73882	154.70962	0.9073784	0.03336373	113.442
gi|115551476|dbj|AK236154.1|	midnightblue	897.17163	77.562319	0.9960916	0.00029315	12.04
gi|115551480|dbj|AK236158.1|	turquoise	989.30838	220.44231	0.9569799	0.0106419	5.924
gi|115551482|dbj|AK236160.1|	magenta	419.76548	77.220013	0.967384	0.00703631	11.576
gi|115551497|dbj|AK238961.1|	blue	618.66925	128.33535	0.9629011	0.00852991	75.646
gi|115551518|dbj|AK238982.1|	tan	112.84552	34.28066	0.9051918	0.03454046	53.66
gi|115551521|dbj|AK238985.1|	white	923.4202	47.295466	0.9812665	0.0030693	247.302
gi|115551534|dbj|AK238998.1|	skyblue	357.00234	38.807828	0.9691612	0.00647085	56.894
gi|115551587|dbj|AK239051.1|	brown	184.73748	20.768368	0.5400358	0.34746692	8.564
gi|115551605|dbj|AK239069.1|	darkturquoise	142.2862	39.228165	0.9579477	0.01028631	17.186
gi|115551606|dbj|AK239070.1|	violet	166.69904	8.4344384	0.3713695	0.53826306	10.646
gi|115551623|dbj|AK239087.1|	black	797.73804	105.03541	0.992362	0.0008004	4.398
gi|115551627|dbj|AK239091.1|	darkgreen	254.38832	41.572303	0.9848011	0.00224419	23.578
gi|115551631|dbj|AK239095.1|	lightcyan	272.70212	39.187098	0.9032201	0.03561264	3.85
gi|115551640|dbj|AK239104.1|	blue	654.7377	77.87746	0.8464491	0.07054199	27.258
gi|115551642|dbj|AK239106.1|	salmon	219.43226	73.244098	0.9859714	0.00199039	88.07
gi|115551652|dbj|AK239116.1|	green	954.9557	100.27686	0.963179	0.00843458	142.774
gi|115551691|dbj|AK230600.1|	magenta	117.11158	34.555046	0.8032351	0.10162556	11.092
gi|115551695|dbj|AK232156.1|	turquoise	313.79379	103.94719	0.7173216	0.17256064	20.118
gi|115551776|dbj|AK236182.1|	blue	738.80866	109.95035	0.9243519	0.0246911	116.754
gi|115551783|dbj|AK236189.1|	midnightblue	979.7029	75.351192	0.9895969	0.00127175	10.506
gi|115551790|dbj|AK236196.1|	tan	197.29013	30.975271	0.8444326	0.0719132	19.056
gi|115551791|dbj|AK236197.1|	royalblue	603.02796	63.09816	0.9956978	0.00033852	9.426
gi|115551828|dbj|AK236235.1|	blue	684.61108	125.64404	0.9563208	0.01088627	6.838
gi|115551842|dbj|AK236249.1|	turquoise	463.92355	135.18121	0.8462752	0.07065995	10.588
gi|115551862|dbj|AK236269.1|	paleturquoise	175.50553	26.822862	0.9825342	0.0027636	9.038
gi|115551864|dbj|AK236271.1|	turquoise	189.66965	73.840828	0.6051815	0.279492	6.088

gi|115551880|dbj|AK236287.1|	green	945.4074	112.55803	0.9904779	0.00111382	4.846
gi|115551881|dbj|AK236288.1|	red	888.14051	124.5843	0.9942386	0.00052451	5.596
gi|115551894|dbj|AK236301.1|	yellow	260.60282	104.94621	0.9395119	0.01769532	20.162
gi|115551903|dbj|AK236310.1|	brown	113.77527	14.576623	0.428485	0.47162302	9.402
gi|115551909|dbj|AK236316.1|	pink	847.6046	146.29429	0.9951659	0.00040317	2.544
gi|115551914|dbj|AK236321.1|	black	931.44433	100.76068	0.9822754	0.00282514	4.848
gi|115551917|dbj|AK236324.1|	darkorange	111.73861	31.073275	0.9507001	0.01304262	155.186
gi|115551923|dbj|AK236330.1|	darkred	194.03771	44.117634	0.9739578	0.00502511	2.864
gi|115551932|dbj|AK236339.1|	blue	860.84673	130.99362	0.9632777	0.00840083	82.006
gi|115551933|dbj|AK236340.1|	orange	440.3458	29.707564	0.939547	0.01768002	20.538
gi|115551938|dbj|AK236345.1|	brown	768.93948	134.06881	0.9928371	0.00072694	15.604
gi|115551942|dbj|AK236349.1|	red	852.28699	125.86886	0.9965588	0.0002422	5.452
gi|115551948|dbj|AK236355.1|	lightcyan	414.90852	59.116574	0.9937341	0.00059483	7.978
gi|115551969|dbj|AK239162.1|	brown	439.23241	88.562783	0.9078825	0.03309431	6.136
gi|115551993|dbj|AK239186.1|	midnightblue	867.07591	72.304427	0.9811158	0.00310633	10.344
gi|115551997|dbj|AK239190.1|	lightgreen	375.88145	39.095533	0.9462616	0.014833	3.708
gi|115552025|dbj|AK239218.1|	turquoise	964.91442	226.677	0.9734737	0.0051655	4.346
gi|115552029|dbj|AK239222.1|	darkred	130.47449	21.056992	0.7978277	0.10575157	24.43
gi|115552039|dbj|AK239232.1|	turquoise	1014.6113	220.03199	0.9516439	0.01267173	3.422
gi|115552041|dbj|AK239234.1|	tan	153.66251	45.636855	0.9860862	0.00196604	9.322
gi|115552061|dbj|AK239254.1|	turquoise	280.57539	107.6196	0.7844283	0.11618612	7.492
gi|115552064|dbj|AK239257.1|	black	843.74409	104.21008	0.9900179	0.00119541	11.37
gi|115552079|dbj|AK239272.1|	grey	301.76919	12.735248	0.9531444	0.01208926	6.006
gi|115552089|dbj|AK239282.1|	blue	846.72	140.75264	0.9807578	0.00319491	41.632
gi|115552097|dbj|AK239290.1|	pink	695.7687	131.85706	0.9738125	0.00506713	30.618
gi|115552101|dbj|AK239294.1|	darkorange	99.587904	11.171426	0.6394644	0.24532873	20.376
gi|115552119|dbj|AK239312.1|	turquoise	922.09665	223.58969	0.9741227	0.00497759	5.712
gi|115552139|dbj|AK239332.1|	turquoise	198.99721	80.062448	0.6734209	0.21271829	12.642
gi|115552153|dbj|AK239346.1|	navy	900.46682	18.663099	-0.088942	0.88690537	9.422
gi|115552177|dbj|AK230486.1|	skyblue	547.34411	43.574733	0.9941126	0.0005418	1821.55
gi|115552179|dbj|AK230488.1|	greenyellow	204.98117	34.404129	0.9684045	0.0067097	35.09
gi|115552191|dbj|AK230500.1|	turquoise	216.84245	78.822458	0.5962433	0.28859025	22.298
gi|115552195|dbj|AK230504.1|	brown	323.30857	58.050926	0.8136236	0.09384047	24.962
gi|115552216|dbj|AK230525.1|	royalblue	719.27547	63.136587	0.9950459	0.00041827	3.64
gi|115552220|dbj|AK230529.1|	black	897.11051	103.7031	0.9885574	0.00146681	6.568
gi|115552234|dbj|AK230543.1|	red	894.62975	127.58416	0.9992509	2.46E-05	2.438
gi|115552246|dbj|AK230555.1|	turquoise	630.50937	164.71268	0.9071055	0.03350991	4.144
gi|115552250|dbj|AK230559.1|	lightcyan	426.46525	42.532068	0.9171782	0.02825406	49.056
gi|115552260|dbj|AK230569.1|	tan	227.9715	36.243036	0.9278066	0.02303138	30.424
gi|115552269|dbj|AK230578.1|	white	1002.7339	43.076693	0.9885676	0.00146485	12.818
gi|115552272|dbj|AK230581.1|	brown	643.32612	130.22667	0.9927593	0.00073881	12.298
gi|115552281|dbj|AK230590.1|	green	801.04309	114.6422	0.9964464	0.00025415	19.204
gi|115552282|dbj|AK230591.1|	midnightblue	626.84529	65.19363	0.9596721	0.00966268	45.446
gi|115552295|dbj|AK230605.1|	midnightblue	901.19487	70.849683	0.9768104	0.00422434	13.254
gi|115552309|dbj|AK230619.1|	turquoise	321.01327	85.365439	0.6340248	0.25066909	114.394
gi|115552311|dbj|AK230621.1|	skyblue	343.63553	36.677224	0.9577045	0.0103753	26.226
gi|115552314|dbj|AK230624.1|	turquoise	620.83461	167.11183	0.8746933	0.05223472	30.668
gi|115552353|dbj|AK232758.1|	turquoise	841.22783	204.37263	0.9480046	0.01412097	11.118

gi|115552359|dbj|AK232764.1|	grey60	284.32478	54.258069	0.9479596	0.0141392	76.344
gi|115552369|dbj|AK236374.1|	green	872.36687	115.52444	0.9962839	0.00027178	2.934
gi|115552393|dbj|AK236398.1|	salmon	228.72767	72.907579	0.9841866	0.00238145	20.292
gi|115552399|dbj|AK236404.1|	yellow	272.42883	92.417761	0.9079084	0.0330805	29.664
gi|115552409|dbj|AK236414.1|	turquoise	440.67937	145.30532	0.8617729	0.06039592	3.124
gi|115552411|dbj|AK236416.1|	navy	251.37636	32.067198	0.9293218	0.02231533	2.172
gi|115552413|dbj|AK236418.1|	black	684.95477	96.14953	0.9738662	0.00505158	12.39
gi|115552415|dbj|AK236420.1|	darkred	133.45244	28.923009	0.8608583	0.06098757	24.242
gi|115552428|dbj|AK236433.1|	brown	72.121576	6.0097184	0.1077948	0.86301765	1815.946
gi|115552430|dbj|AK236435.1|	turquoise	765.39292	195.29717	0.9448087	0.01543517	28.692
gi|115552454|dbj|AK236459.1|	blue	567.58703	106.0047	0.9166033	0.0285462	11.562
gi|115552473|dbj|AK236478.1|	red	824.65027	122.56788	0.9910971	0.00100705	67.94
gi|115552477|dbj|AK236482.1|	red	760.06663	115.64747	0.9792034	0.00358892	5.59
gi|115552482|dbj|AK236487.1|	pink	885.32223	148.1656	0.9976938	0.0001329	16.784
gi|115552488|dbj|AK236493.1|	turquoise	752.72679	187.44442	0.9191965	0.02723604	13.894
gi|115552491|dbj|AK236496.1|	magenta	281.92377	85.747864	0.9980763	0.00010126	8.988
gi|115552492|dbj|AK236497.1|	darkorange	119.43258	30.827153	0.9538894	0.01180341	16.906
gi|115552519|dbj|AK236524.1|	white	601.46919	34.194558	0.8584151	0.06257689	10.022
gi|115552523|dbj|AK236528.1|	darkorange	139.23407	33.721227	0.9836271	0.00250872	73.542
gi|115552527|dbj|AK236532.1|	pink	854.28658	148.29408	0.9979212	0.00011374	47.78
gi|115552544|dbj|AK236549.1|	darkorange	99.540024	30.386587	0.9384803	0.01814708	17.472
gi|115552549|dbj|AK236554.1|	green	656.99483	88.134951	0.9385805	0.01810305	14.94
gi|115552559|dbj|AK236564.1|	yellow	265.88512	120.13031	0.9732843	0.00522079	15.784
gi|115552575|dbj|AK232780.1|	green	971.33043	104.05905	0.970984	0.00590734	32.604
gi|115552583|dbj|AK232788.1|	blue	567.51986	114.81994	0.937741	0.01847306	26.182
gi|115552627|dbj|AK232832.1|	blue	935.1136	144.99434	0.9862166	0.0019385	2.656
gi|115552638|dbj|AK232843.1|	magenta	256.40333	84.545421	0.9949247	0.00043371	15.054
gi|115552645|dbj|AK232850.1|	darkturquoise	119.16024	33.414014	0.9189823	0.02734353	55.89
gi|115552656|dbj|AK232861.1|	pink	993.39705	131.40809	0.9724256	0.0054738	15.138
gi|115552676|dbj|AK232881.1|	skyblue	654.65054	37.149394	0.9564647	0.01083277	26.306
gi|115552724|dbj|AK232929.1|	purple	303.94901	75.318161	0.9844578	0.00232053	44.658
gi|115552731|dbj|AK232936.1|	violet	49.918088	8.6045821	-0.957021	0.01062687	17.42
gi|115552766|dbj|AK239359.1|	darkorange	173.21945	20.870884	0.8501555	0.06804315	19.158
gi|115552791|dbj|AK239385.1|	black	722.17012	100.2467	0.9824864	0.00277493	17.748
gi|115552842|dbj|AK239436.1|	yellow	198.08664	89.366067	0.9033985	0.03551523	654.514
gi|115552862|dbj|AK239456.1|	navy	44.447228	20.747329	0.8519466	0.06684571	99.656
gi|115552865|dbj|AK239459.1|	yellow	223.34069	79.136542	0.8810003	0.04838878	302.608
gi|115552884|dbj|AK239478.1|	brown	351.76135	49.974873	0.7824708	0.11773506	43.284
gi|115552903|dbj|AK239497.1|	turquoise	348.45623	127.3445	0.8410021	0.07426466	93.74
gi|115552915|dbj|AK239509.1|	turquoise	322.88311	117.03605	0.8141244	0.09346998	23.386
gi|115552930|dbj|AK239524.1|	lightgreen	521.8447	35.970717	0.8909167	0.0425338	46.592
gi|115552932|dbj|AK239526.1|	yellow	274.33858	120.20836	0.9727702	0.00537178	16.896
gi|115552944|dbj|AK239538.1|	lightcyan	481.79086	58.871597	0.9890729	0.00136892	22.112
gi|115552956|dbj|AK239550.1|	salmon	209.56553	73.257644	0.9870203	0.00177167	14.654
gi|115552967|dbj|AK239553.1|	grey	493.80682	15.401154	0.9917099	0.00090496	3.938
gi|115552977|dbj|AK239563.1|	midnightblue	950.73351	74.535818	0.987659	0.00164268	70.788
gi|115552989|dbj|AK239575.1|	turquoise	901.04282	207.91034	0.9443834	0.01561293	43.926
gi|115553011|dbj|AK239597.1|	yellow	295.47776	128.42362	0.9892914	0.00132811	15.094

gi|115553014|dbj|AK239600.1|	turquoise	659.0049	178.55483	0.9034407	0.0354922	48.788
gi|115553015|dbj|AK239601.1|	blue	874.37973	133.50238	0.9665168	0.00731782	31.152
gi|115553025|dbj|AK239611.1|	turquoise	383.65084	59.835807	0.0713757	0.90919887	59.34
gi|115553031|dbj|AK239617.1|	green	778.7619	105.49305	0.9760188	0.00444192	11.966
gi|115553034|dbj|AK239620.1|	brown	822.96567	108.51197	0.939453	0.01772101	10.694
gi|115553038|dbj|AK239624.1|	paleturquoise	176.30164	25.79111	0.9698647	0.00625135	216.782
gi|115553041|dbj|AK239627.1|	turquoise	296.392	109.51421	0.7618258	0.13443649	14.328
gi|115553045|dbj|AK239631.1|	brown	701.08599	132.37873	0.9883925	0.0014986	8.942
gi|115553060|dbj|AK239646.1|	turquoise	944.70426	221.53261	0.9688538	0.00656754	165.182
gi|115553074|dbj|AK239660.1|	brown	87.821007	8.7745791	0.4001959	0.50440304	27.808
gi|115553082|dbj|AK239668.1|	greenyellow	161.4821	18.965333	0.6851165	0.20178992	79.716
gi|115553090|dbj|AK239676.1|	red	767.45465	114.08828	0.9763724	0.00434428	23.138
gi|115553091|dbj|AK239677.1|	blue	979.03334	119.96301	0.9403144	0.01734646	20.93
gi|115553103|dbj|AK239689.1|	darkorange	167.55309	28.969838	0.9517376	0.01263508	50.106
gi|115553107|dbj|AK239693.1|	darkgreen	232.2132	42.852189	0.991037	0.00101726	45.844
gi|115553121|dbj|AK239707.1|	black	891.08782	103.38138	0.9887203	0.00143563	19.85
gi|115553124|dbj|AK239710.1|	black	648.00698	95.848508	0.9734834	0.00516268	3.632
gi|115553140|dbj|AK239726.1|	green	827.6487	109.35838	0.9834697	0.00254493	22.718
gi|115553142|dbj|AK239728.1|	purple	285.6935	77.62309	0.9921339	0.00083649	73.714
gi|115553143|dbj|AK239729.1|	yellow	195.34843	75.377804	0.8656799	0.05788897	11.824
gi|115553145|dbj|AK239731.1|	red	872.67918	126.51077	0.9976081	0.00014038	8.55
gi|115553157|dbj|AK239743.1|	red	932.04923	127.06527	0.9982108	9.08E-05	7.638
gi|115553159|dbj|AK239745.1|	skyblue	271.66104	29.989971	0.9107311	0.03158508	17.766
gi|115553169|dbj|AK232967.1|	lightgreen	275.80664	25.373288	0.8795516	0.04926392	67.084
gi|115553171|dbj|AK232969.1|	salmon	248.71657	67.402936	0.9676563	0.00694864	39.42
gi|115553181|dbj|AK232979.1|	blue	962.96339	137.216	0.9723874	0.00548515	19.366
gi|115553189|dbj|AK232987.1|	royalblue	619.42411	64.207283	0.9992246	2.59E-05	13.542
gi|115553196|dbj|AK232994.1|	darkturquoise	138.67177	36.988387	0.9440789	0.01574058	312.066
gi|115553202|dbj|AK233000.1|	blue	593.02688	119.86648	0.9455746	0.01511677	56.898
gi|115553207|dbj|AK233005.1|	blue	799.88868	138.1317	0.9755803	0.004564	4.982
gi|115553209|dbj|AK233007.1|	red	921.04355	127.71555	0.9993342	2.06E-05	200.636
gi|115553226|dbj|AK233024.1|	turquoise	267.31666	93.883844	0.7377767	0.15469427	36.972
gi|115553231|dbj|AK233029.1|	turquoise	657.1307	180.53941	0.9099023	0.03202186	53.246
gi|115553249|dbj|AK233047.1|	blue	723.97217	137.35475	0.9777824	0.00396211	314.876
gi|115553264|dbj|AK233062.1|	pink	847.6046	146.29429	0.9951659	0.00040317	2.544
gi|115553284|dbj|AK233082.1|	paleturquoise	141.21707	19.391028	0.9114881	0.03118781	7.634
gi|115553328|dbj|AK233126.1|	paleturquoise	174.11173	24.675817	0.968834	0.00657379	40.426
gi|115553344|dbj|AK233142.1|	lightcyan	305.42222	53.752337	0.9736354	0.00511848	8.056
gi|115553351|dbj|AK233149.1|	magenta	423.27004	75.52335	0.9617724	0.00892058	14.668
gi|115553356|dbj|AK233154.1|	pink	833.19125	145.01647	0.9933919	0.00064419	4.222
gi|115553369|dbj|AK236572.1|	green	772.32441	113.30712	0.9939354	0.00056642	13.926
gi|115553370|dbj|AK236573.1|	black	810.81331	108.84444	0.9996706	7.18E-06	11.844
gi|115553373|dbj|AK236576.1|	turquoise	311.61126	114.85035	0.7405236	0.15233855	54.728
gi|115553381|dbj|AK236584.1|	midnightblue	880.07614	64.867663	0.9581743	0.01020363	19.168
gi|115553384|dbj|AK236587.1|	purple	276.4421	74.047871	0.9811087	0.00310809	235.772
gi|115553397|dbj|AK236600.1|	grey60	300.71578	65.706569	0.9879349	0.00158798	11.96
gi|115553406|dbj|AK236609.1|	yellow	281.27961	123.86282	0.9788894	0.00367034	12.642
gi|115553415|dbj|AK236618.1|	turquoise	245.72288	92.857388	0.7223416	0.16812384	20.314

gi|115553434|dbj|AK236637.1|	greenyellow	236.9933	37.708473	0.9719778	0.00560728	21.624
gi|115553440|dbj|AK236643.1|	turquoise	121.56954	37.189966	0.4422718	0.45582042	16.386
gi|115553452|dbj|AK236655.1|	midnightblue	938.93563	78.656214	0.9989192	4.26E-05	27.908
gi|115553453|dbj|AK236656.1|	brown	586.40068	119.34025	0.9727624	0.00537408	22.72
gi|115553457|dbj|AK236660.1|	black	817.62291	92.669796	0.9656174	0.00761358	17.412
gi|115553468|dbj|AK236671.1|	orange	343.67911	34.373785	0.977881	0.00393582	32.878
gi|115553471|dbj|AK236674.1|	midnightblue	923.87959	77.421979	0.9955601	0.0003549	25.398
gi|115553478|dbj|AK236681.1|	magenta	310.64332	81.860431	0.9868532	0.00180595	11.708
gi|115553483|dbj|AK236686.1|	purple	223.09216	50.549532	0.8896324	0.04327857	18.196
gi|115553496|dbj|AK236699.1|	blue	840.50487	150.04394	0.9953576	0.00037944	0.79
gi|115553497|dbj|AK236700.1|	green	905.44062	113.87848	0.9918353	0.00088452	41.898
gi|115553505|dbj|AK236708.1|	darkred	82.632982	16.361978	0.7238068	0.1668352	12.09
gi|115553521|dbj|AK236724.1|	turquoise	555.46681	149.7976	0.8750897	0.05199025	11.084
gi|115553524|dbj|AK236727.1|	salmon	158.71194	55.962773	0.9306049	0.0217147	11.184
gi|115553527|dbj|AK236730.1|	turquoise	474.02749	157.97822	0.8881561	0.04413963	69.25
gi|115553540|dbj|AK236743.1|	red	902.07225	125.17703	0.9952766	0.00038941	13.696
gi|115553549|dbj|AK236752.1|	darkorange	178.52709	19.347486	0.8340768	0.07908228	157.598
gi|115553555|dbj|AK236758.1|	pink	903.31158	149.81116	0.9999235	8.03E-07	21.228
gi|115553566|dbj|AK239753.1|	paleturquoise	173.94898	25.787304	0.9765033	0.00430832	18.292
gi|115553569|dbj|AK239756.1|	orange	285.67102	19.462045	0.7910929	0.11095925	23.726
gi|115553571|dbj|AK239758.1|	black	882.68088	106.68126	0.9949471	0.00043084	19.42
gi|115553590|dbj|AK239777.1|	darkmagenta	73.369305	5.3581262	0.7634474	0.13310101	26.69
gi|115553648|dbj|AK239835.1|	greenyellow	181.67072	38.196985	0.9318572	0.02113362	7.45
gi|115553654|dbj|AK239841.1|	salmon	226.6611	71.006347	0.9789695	0.00364951	4.924
gi|115553671|dbj|AK239858.1|	black	842.01685	108.27889	0.9983756	7.86E-05	38.728
gi|115553680|dbj|AK239867.1|	lightcyan	323.44286	41.057401	0.9134114	0.03018577	3.828
gi|115553700|dbj|AK239887.1|	brown	741.37908	135.77867	0.9957641	0.00033073	12.45
gi|115553703|dbj|AK239890.1|	black	744.41755	100.32474	0.9829544	0.00266464	27.228
gi|115553719|dbj|AK239906.1|	midnightblue	694.69022	64.920246	0.9591652	0.00984469	117.038
gi|115553747|dbj|AK239934.1|	grey60	302.3134	66.971189	0.9922626	0.00081606	30.098
gi|115553748|dbj|AK239935.1|	salmon	205.36603	69.331145	0.9750223	0.00472095	40.856
gi|115553757|dbj|AK239944.1|	skyblue	485.42571	43.912914	0.996912	0.0002059	17.25
gi|115553777|dbj|AK236782.1|	pink	894.62975	149.62591	0.9996875	6.63E-06	1.412
gi|115553782|dbj|AK236787.1|	white	243.8461	17.047208	-0.654458	0.23077229	11.376
gi|115553811|dbj|AK236816.1|	white	990.0018	40.411791	0.982728	0.0027178	2.84
gi|115553814|dbj|AK236819.1|	tan	82.26292	22.540669	0.7344439	0.15756648	36.212
gi|115553831|dbj|AK236836.1|	violet	757.38592	43.043359	0.9926597	0.0007541	19.978
gi|115553846|dbj|AK236851.1|	greenyellow	213.86994	37.679012	0.9406481	0.01720211	21.74
gi|115553858|dbj|AK236863.1|	salmon	98.885969	39.80335	0.85851	0.06251493	17.924
gi|115553862|dbj|AK236867.1|	magenta	226.35098	80.785733	0.9840591	0.00241026	6.466
gi|115553894|dbj|AK236899.1|	midnightblue	805.43922	74.779461	0.988385	0.00150004	8.348
gi|115553962|dbj|AK233171.1|	lightgreen	123.17666	15.8526	0.5940869	0.29079658	39.418
gi|115553967|dbj|AK233176.1|	tan	176.41965	34.552612	0.8777203	0.05037731	39.294
gi|115554012|dbj|AK233221.1|	violet	876.55176	46.724055	0.9952875	0.00038807	27.042
gi|115554020|dbj|AK233229.1|	yellow	242.01968	102.47793	0.9346872	0.01983948	4.58
gi|115554022|dbj|AK233231.1|	blue	394.42822	77.082095	0.8480237	0.06947699	320.59
gi|115554075|dbj|AK233284.1|	darkred	162.05446	38.443283	0.9413317	0.01690751	53.848
gi|115554113|dbj|AK233322.1|	orange	206.44821	36.210217	0.9785385	0.00376202	6.954

gi|115554124|dbj|AK233334.1|	darkgreen	166.25229	13.664977	0.6637125	0.22191054	224.934
gi|115554126|dbj|AK233336.1|	darkgrey	899.38893	52.368046	0.9912853	0.00097531	5.984
gi|115554130|dbj|AK233340.1|	salmon	236.77167	75.769327	0.9929563	0.00070888	31.4
gi|115554133|dbj|AK233343.1|	black	818.80107	103.58363	0.9892787	0.00133048	39.262
gi|115554161|dbj|AK239956.1|	yellow	161.04083	61.732211	0.8276705	0.08362107	31.54
gi|115554180|dbj|AK239975.1|	orange	454.82969	28.623033	0.9300087	0.02199315	14.426
gi|115554213|dbj|AK240008.1|	turquoise	913.76428	202.02055	0.9283212	0.02278738	19.874
gi|115554214|dbj|AK240009.1|	blue	972.23544	132.52648	0.9647489	0.00790285	5.6
gi|115554223|dbj|AK240018.1|	turquoise	385.5069	132.73867	0.8374585	0.07671805	4.216
gi|115554227|dbj|AK240022.1|	violet	986.38197	44.231485	0.9619895	0.008845	17.474
gi|115554228|dbj|AK240023.1|	yellow	293.71116	133.86998	0.9977322	0.00012959	8.846
gi|115554247|dbj|AK240042.1|	yellow	291.13572	130.99668	0.9931899	0.00067394	6.692
gi|115554271|dbj|AK240066.1|	white	233.15953	14.951233	-0.756706	0.13867885	31.24
gi|115554273|dbj|AK240068.1|	darkorange	86.820207	13.13041	0.744038	0.1493401	14.578
gi|115554282|dbj|AK240077.1|	lightyellow	344.08922	51.819239	0.9546668	0.01150757	86.374
gi|115554284|dbj|AK240079.1|	midnightblue	909.62175	69.720767	0.9734216	0.00518068	7.382
gi|115554299|dbj|AK240094.1|	lightcyan	600.66347	42.48091	0.9003712	0.03718026	11.778
gi|115554309|dbj|AK240104.1|	darkred	175.11612	41.572279	0.970574	0.00603261	8.686
gi|115554312|dbj|AK240107.1|	grey60	303.45338	65.080445	0.9858067	0.0020255	125.76
gi|115554314|dbj|AK240109.1|	turquoise	972.87954	221.69907	0.9627126	0.00859474	53.156
gi|115554351|dbj|AK240146.1|	lightyellow	430.56469	59.348535	0.9818465	0.00292811	9.592
gi|115554358|dbj|AK233368.1|	skyblue	602.53537	39.028518	0.9692956	0.00642871	21.496
gi|115554363|dbj|AK233373.1|	midnightblue	903.29994	77.628114	0.99628	0.00027221	9.318
gi|115554371|dbj|AK233381.1|	darkmagenta	60.913006	2.7729453	0.727486	0.16361197	15.94
gi|115554373|dbj|AK233383.1|	yellow	303.2636	133.43758	0.9966065	0.00023718	9.506
gi|115554409|dbj|AK233419.1|	blue	842.17944	141.91557	0.9825297	0.00276467	1366.408
gi|115554410|dbj|AK233420.1|	yellow	292.86106	120.84279	0.9714359	0.00577025	73.478
gi|115554413|dbj|AK233423.1|	salmon	219.56089	76.874474	0.9969879	0.00019835	5.18
gi|115554415|dbj|AK233425.1|	turquoise	379.41723	107.95749	0.7581209	0.13750241	10.44
gi|115554455|dbj|AK233465.1|	yellow	306.85487	131.02737	0.9916412	0.00091622	8.352
gi|115554459|dbj|AK233469.1|	lightcyan	401.98105	58.548906	0.9936291	0.00060985	14.114
gi|115554528|dbj|AK233538.1|	grey60	298.90766	66.183078	0.990116	0.00117784	140.376
gi|115554532|dbj|AK233542.1|	violet	101.54477	6.0762665	-0.594351	0.29052633	250.42
gi|115554540|dbj|AK233550.1|	white	941.00732	41.167011	0.976999	0.00417302	14.934
gi|115554557|dbj|AK236967.1|	turquoise	427.96754	142.73559	0.8477714	0.06964727	3.906
gi|115554560|dbj|AK236970.1|	brown	258.87954	40.255424	0.7276863	0.16343699	17.232
gi|115554563|dbj|AK236973.1|	purple	197.6118	43.729645	0.8645361	0.05861946	40.214
gi|115554566|dbj|AK236976.1|	green	712.35769	107.12704	0.9814321	0.00302877	46.522
gi|115554588|dbj|AK236998.1|	darkgrey	767.80251	48.254399	0.973971	0.00502131	2.726
gi|115554610|dbj|AK237020.1|	turquoise	511.3545	152.62961	0.8610071	0.06089116	7.622
gi|115554627|dbj|AK237038.1|	navy	174.76908	37.394996	0.9578355	0.01032732	6.38
gi|115554633|dbj|AK237044.1|	tan	247.49902	41.122082	0.9376081	0.0185319	8.176
gi|115554649|dbj|AK237060.1|	tan	168.56296	31.610337	0.8594789	0.06188328	13.824
gi|115554695|dbj|AK237106.1|	green	772.71074	78.832789	0.9114309	0.03121774	24.91
gi|115554713|dbj|AK237124.1|	greenyellow	77.055637	9.9452314	0.4918273	0.40003831	50.328
gi|115554725|dbj|AK237136.1|	tan	168.64141	27.909668	0.8278082	0.08352271	9.648
gi|115554740|dbj|AK237151.1|	brown	615.94428	112.25808	0.9595816	0.00969509	16.372
gi|115554764|dbj|AK233576.1|	paleturquoise	166.49272	20.092821	0.9057237	0.034253	2.658

gi|115554769|dbj|AK233581.1|	brown	789.83949	129.42383	0.980579	0.00323945	7.876
gi|115554826|dbj|AK233638.1|	navy	175.92025	37.182688	0.941622	0.01678294	42.316
gi|115554840|dbj|AK233652.1|	black	617.76979	94.264228	0.9695948	0.00633526	29.622
gi|115554857|dbj|AK233669.1|	lightgreen	565.08101	37.86175	0.9375784	0.01854504	352.272
gi|115554861|dbj|AK233673.1|	grey60	265.5928	54.729952	0.9499561	0.01333746	28.8
gi|115554866|dbj|AK233678.1|	turquoise	238.60203	62.59885	0.6241185	0.26047377	13.16
gi|115554868|dbj|AK233680.1|	blue	360.55056	81.622197	0.8611881	0.06077403	6.348
gi|115554875|dbj|AK233687.1|	violet	79.485894	7.520462	-0.827101	0.08402847	13.248
gi|115554899|dbj|AK233711.1|	turquoise	588.64963	178.27076	0.9267509	0.02353463	24.932
gi|115554918|dbj|AK233730.1|	navy	114.50055	40.779505	0.9673587	0.00704446	11.512
gi|115554929|dbj|AK233741.1|	greenyellow	121.41954	21.314593	0.824432	0.08594501	11.672
gi|115554960|dbj|AK240159.1|	navy	165.496	36.148847	0.9690351	0.00651044	6.312
gi|115554973|dbj|AK240172.1|	lightcyan	253.34906	35.421202	0.8811035	0.04832663	37.31
gi|115554982|dbj|AK240181.1|	darkred	153.52041	37.353805	0.9333926	0.0204282	159.984
gi|115554984|dbj|AK240183.1|	brown	809.99765	120.46326	0.9612728	0.00909535	14.342
gi|115555002|dbj|AK240201.1|	turquoise	302.30159	115.92259	0.7575341	0.13798989	16.394
gi|115555012|dbj|AK240211.1|	greenyellow	155.57795	21.337355	0.7767329	0.12231038	30.844
gi|115555020|dbj|AK240219.1|	turquoise	928.83216	216.92851	0.9589029	0.0099393	6.28
gi|115555021|dbj|AK240220.1|	brown	84.321826	11.707869	0.4551757	0.44113773	49.464
gi|115555035|dbj|AK240234.1|	turquoise	275.11055	56.859084	-0.146456	0.81419562	12.592
gi|115555041|dbj|AK240240.1|	green	699.67619	96.597578	0.9577816	0.01034706	29.136
gi|115555043|dbj|AK240242.1|	darkturquoise	237.61824	44.160373	0.9829301	0.00267032	8.414
gi|115555058|dbj|AK240257.1|	darkred	170.89338	41.37103	0.972788	0.00536653	23.716
gi|115555063|dbj|AK240262.1|	blue	645.63776	136.8715	0.9759588	0.00445857	27.836
gi|115555072|dbj|AK240271.1|	turquoise	190.81992	71.652736	0.5515383	0.33519538	15.072
gi|115555076|dbj|AK240275.1|	darkgrey	861.75597	49.451176	0.9794102	0.00353562	9.754
gi|115555097|dbj|AK240296.1|	brown	805.07013	128.3204	0.9785702	0.00375369	66.95
gi|115555127|dbj|AK240327.1|	navy	166.84219	35.908055	0.9678123	0.0068986	3.436
gi|115555129|dbj|AK240329.1|	turquoise	573.32568	149.04053	0.8713678	0.05429956	89.024
gi|115555138|dbj|AK240338.1|	yellow	256.71228	115.6948	0.9639561	0.00816996	8.99
gi|115555145|dbj|AK240345.1|	black	703.18887	94.217468	0.9701379	0.00616679	97.352
gi|115555150|dbj|AK233765.1|	lightyellow	489.40774	63.989485	0.9985239	6.81E-05	46.042
gi|115555155|dbj|AK233770.1|	turquoise	956.91527	219.16917	0.9587889	0.00998051	21.938
gi|115555162|dbj|AK233777.1|	violet	189.26677	5.1725826	-0.024497	0.96881271	117.69
gi|115555163|dbj|AK233778.1|	yellow	275.5889	109.27816	0.9489619	0.01373482	36.06
gi|115555187|dbj|AK233802.1|	greenyellow	226.69553	38.089234	0.9675575	0.00698041	9.236
gi|115555188|dbj|AK233803.1|	violet	72.193817	5.2621784	-0.551535	0.33519884	11.53
gi|115555205|dbj|AK233820.1|	pink	813.24552	138.02737	0.9834617	0.00254676	5.25
gi|115555219|dbj|AK233834.1|	turquoise	338.70983	121.77198	0.7993513	0.10458398	5.984
gi|115555288|dbj|AK233903.1|	turquoise	577.25633	140.29942	0.8335941	0.07942153	50.144
gi|115555322|dbj|AK233937.1|	darkorange	145.62895	30.710214	0.9615153	0.00901038	10.84
gi|115555347|dbj|AK233960.1|	salmon	220.07039	76.758292	0.996812	0.00021598	1.548
gi|115555353|dbj|AK233966.1|	darkgreen	118.36683	11.874259	0.5955899	0.28925834	18.308
gi|115555420|dbj|AK234033.1|	turquoise	198.82433	72.938105	0.5470393	0.33998209	24.84
gi|115555451|dbj|AK234064.1|	greenyellow	176.27534	27.791886	0.8797047	0.04917122	24.51
gi|115555475|dbj|AK234088.1|	royalblue	618.55907	64.313537	0.9995581	1.11E-05	16.364
gi|115555495|dbj|AK234108.1|	violet	862.12141	46.630804	0.9976891	0.00013331	13.934
gi|115555513|dbj|AK234126.1|	pink	583.45176	113.0345	0.9423523	0.01647084	45.134

gi|115555517|dbj|AK234130.1|	violet	603.24183	36.35663	0.9761113	0.00441632	1.904
gi|115555541|dbj|AK237173.1|	turquoise	276.79396	102.63346	0.7198442	0.17032691	80.362
gi|115555553|dbj|AK237185.1|	turquoise	822.56274	191.36607	0.9157437	0.02898483	46.472
gi|115555561|dbj|AK237193.1|	darkred	187.02161	39.397546	0.938868	0.01797684	6.44
gi|115555588|dbj|AK237220.1|	turquoise	343.26973	59.274364	-0.08755	0.88867026	8.828
gi|115555616|dbj|AK237249.1|	lightcyan	152.64978	26.941206	0.7760997	0.12281846	2.488
gi|115555619|dbj|AK237252.1|	darkturquoise	159.09586	38.648327	0.9489711	0.01373112	194.276
gi|115555627|dbj|AK237260.1|	navy	909.36003	18.795538	-0.07922	0.89923914	2.624
gi|115555639|dbj|AK237272.1|	red	837.64075	123.35965	0.9925321	0.00077383	91.996
gi|115555653|dbj|AK237286.1|	tan	206.14276	30.80858	0.8618771	0.06032863	20.842
gi|115555661|dbj|AK237294.1|	greenyellow	171.02273	29.212303	0.8264058	0.08452633	14.918
gi|115555675|dbj|AK237308.1|	green	923.03101	108.52348	0.9798479	0.00342372	18.198
gi|115555681|dbj|AK237314.1|	darkmagenta	98.466885	10.189783	0.951665	0.01266348	21.736
gi|115555693|dbj|AK237326.1|	darkgrey	895.48788	50.004643	0.9816584	0.00297367	11.514
gi|115555721|dbj|AK234149.1|	pink	970.52074	143.65031	0.99092	0.00103721	7.08
gi|116175276|ref|NM_001077227.1|	skyblue	508.97655	44.024736	0.9972008	0.00017771	8.3
gi|117661185|gb|DQ629176.1|	brown	724.85245	119.6258	0.9607831	0.0092677	391.506
gi|118403765|ref|NM_001078687.1|	darkgrey	666.90451	38.890262	0.9299623	0.02201486	153.78
gi|118403821|ref|NM_001078679.1|	blue	977.7803	130.91986	0.9616685	0.00895682	5.358
gi|118403857|ref|NM_001078670.1|	orange	145.56174	27.978185	0.9192314	0.02721852	19.718
gi|118403869|ref|NM_001078669.1|	lightyellow	572.70759	53.184508	0.9593894	0.00976405	15.738
gi|118403911|ref|NM_001078662.1|	navy	70.202723	32.960442	0.9476067	0.01428252	43.528
gi|119310177|ref|NM_001032376.2|	turquoise	798.03513	182.53246	0.8930182	0.04132412	16.226
gi|1245714|gb|S80644.1|	brown	680.79368	133.4887	0.9968989	0.00020721	6.476
gi|125490322|ref|NM_213824.2|	red	688.9285	108.10166	0.9654498	0.00766913	6.396
gi|125630297|ref|NM_001005152.2|	red	976.68448	119.4312	0.9849539	0.00221049	9.006
gi|125630333|ref|NM_214068.2|	green	937.51192	113.42087	0.9923058	0.00080925	9.876
gi|139530445|gb|EF486522.1|	turquoise	783.14382	186.59437	0.9236352	0.02504005	130.098
gi|145279656|ref|NM_214014.2|	lightcyan	335.38371	55.661648	0.983059	0.0026402	6.29
gi|146198449|dbj|AB292846.1|	purple	302.04992	78.542565	0.9939988	0.00055757	107.41
gi|146741279|dbj|AB271920.1|	red	923.25583	123.81538	0.9928517	0.00072472	4.388
gi|147899058|ref|NM_001097509.1|	magenta	200.85631	76.275814	0.9696809	0.00630845	178.64
gi|147900046|ref|NM_001097474.1|	purple	241.59099	49.791387	0.8912395	0.04234728	553.29
gi|147900501|ref|NM_001097506.1|	green	774.96664	108.25445	0.9826289	0.00274119	23.364
gi|147902484|ref|NM_001097419.1|	yellow	259.13811	102.76028	0.9342677	0.02002964	46.69
gi|147906088|ref|NM_213946.2|	blue	908.64443	140.25975	0.9790387	0.00363154	144.588
gi|148222423|ref|NM_001097426.1|	turquoise	818.87143	191.90046	0.9211051	0.02628451	12.77
gi|148222590|ref|NM_001097486.1|	yellow	311.61992	125.94518	0.9824534	0.00278276	103.824
gi|148223598|ref|NM_001097476.1|	greenyellow	192.19843	38.290298	0.9513181	0.01279937	60.608
gi|148224637|ref|NM_001097469.1|	darkturquoise	117.90953	35.810322	0.9322479	0.02095337	33.922
gi|148225749|ref|NM_001097504.1|	royalblue	392.92729	50.814292	0.9457363	0.01504983	9.998
gi|148228021|ref|NM_001097420.1|	blue	815.6891	151.55949	0.9981466	9.58E-05	5.734
gi|148229131|ref|NM_001097428.1|	turquoise	257.52999	52.472426	-0.277928	0.6507409	9.67
gi|148233142|ref|NM_001097435.1|	royalblue	368.08566	47.961604	0.9328076	0.02069606	24.026
gi|148233297|ref|NM_001097485.1|	green	964.24821	109.55811	0.9830601	0.00263994	26.374
gi|148233557|ref|NM_001097460.1|	green	406.10766	67.699234	0.8778746	0.05028319	24.042
gi|148234109|ref|NM_001093735.1|	darkorange	135.04008	34.629699	0.9907435	0.00106759	19.944
gi|148235350|ref|NM_001097475.1|	green	815.00071	112.10447	0.9904587	0.00111718	105.43

gi|148237281|ref|NM_001097478.1|	turquoise	342.17719	123.61717	0.8230467	0.08694502	211.854
gi|149364039|gb|EF468461.1|	red	740.53342	113.5805	0.975552	0.00457194	3.644
gi|149944498|ref|NM_214343.2|	green	970.94358	101.94656	0.9657349	0.00757472	19.014
gi|149944608|ref|NM_001001546.2|	lightgreen	393.33861	35.402017	0.8787854	0.04972879	2.846
gi|150246527|ref|NM_001044575.2|	black	903.39617	101.74859	0.9852702	0.00214125	74.618
gi|153792275|ref|NM_001097505.1|	darkturquoise	121.85393	34.545422	0.9210927	0.02629066	34.57
gi|153792324|ref|NM_001097517.1|	brown	790.80158	127.3891	0.9759484	0.00446145	7.696
gi|153792599|ref|NM_001099930.1|	turquoise	880.89342	192.54857	0.9129206	0.03044049	2.626
gi|154147629|ref|NM_001100190.1|	darkgreen	161.27607	16.449341	0.6840388	0.20279019	38.082
gi|154147643|ref|NM_001100193.1|	tan	241.51649	40.223415	0.9269631	0.02343316	23.356
gi|156120135|ref|NM_001101827.1|	red	965.55813	121.30966	0.9882907	0.00151834	31.556
gi|156120149|ref|NM_001101823.1|	orange	177.23384	33.104924	0.948152	0.01406131	2.634
gi|156120151|ref|NM_001101824.1|	turquoise	742.494	205.0379	0.9653796	0.00769244	2.194
gi|156123600|gb|EU030283.2|	brown	678.04408	134.75677	0.9974461	0.00015487	13.994
gi|156151354|ref|NM_001037146.2|	paleturquoise	178.56291	24.771519	0.954446	0.01159133	38.642
gi|157427686|ref|NM_214374.2|	magenta	333.83057	82.886421	0.9888691	0.00140736	899.148
gi|157427714|ref|NM_001105294.1|	pink	897.1293	148.6751	0.9983702	7.90E-05	3.52
gi|157427721|ref|NM_001105299.1|	brown	682.07762	129.9874	0.9841301	0.00239418	37.688
gi|157427723|ref|NM_001105300.1|	navy	40.012119	9.1837642	0.5741041	0.3114474	72.924
gi|157427727|ref|NM_001105302.1|	turquoise	1012.9004	216.01669	0.9427656	0.01629506	4.48
gi|157427731|ref|NM_001105304.1|	royalblue	569.14135	63.229737	0.9957352	0.00033412	36.054
gi|157427747|ref|NM_001105296.1|	blue	666.43909	131.0639	0.9661508	0.00743771	9.986
gi|158262680|ref|NM_001109947.1|	brown	642.1091	115.89411	0.9574509	0.01046833	9.788
gi|158631261|ref|NM_001099936.1|	blue	674.2153	120.7246	0.9482039	0.0140403	32.74
gi|160420270|ref|NM_001044582.1|	black	836.336	104.57196	0.9913405	0.00096608	39.268
gi|162139822|ref|NM_001111257.1|	red	932.29467	125.79665	0.996146	0.00028705	27.128
gi|162287024|ref|NM_001111258.1|	grey	486.19051	15.720181	0.9961612	0.00028535	10.246
gi|162951810|ref|NM_001112690.1|	purple	288.94918	79.982121	0.9993969	1.78E-05	11.448
gi|16304807|emb|AJ416019.1|	turquoise	867.85056	216.41871	0.967387	0.00703533	64.818
gi|163115794|gb|EU009401.2|	blue	908.90838	150.44045	0.9955576	0.00035519	13.862
gi|163310772|ref|NM_001097521.2|	tan	168.50913	37.164951	0.9047647	0.03477182	36.278
gi|163915140|ref|NM_001113047.1|	navy	73.409063	8.2794306	-0.683235	0.20353683	200.664
gi|163915152|ref|NM_001113053.1|	royalblue	634.58257	62.571431	0.9940016	0.00055718	3.93
gi|164518957|ref|NM_001113287.1|	navy	760.14656	16.356755	-0.153399	0.80545537	308.13
gi|164664445|ref|NM_001113439.1|	lightcyan	264.17425	45.129975	0.9326086	0.02078743	47.692
gi|164664451|ref|NM_001113442.1|	midnightblue	1000.0149	70.778308	0.9762357	0.00438194	20.97
gi|164664453|ref|NM_001113443.1|	turquoise	572.65302	177.90833	0.9367252	0.01892405	25.266
gi|164664455|ref|NM_001113444.1|	darkturquoise	188.60458	41.987838	0.9711636	0.00585272	83.23
gi|165973425|ref|NM_001113706.1|	lightgreen	128.70362	17.503804	0.6921972	0.19525303	54.846
gi|165973431|ref|NM_001113698.1|	lightcyan	218.90161	37.942368	0.891758	0.04204822	32.388
gi|166796042|ref|NM_001114275.1|	black	504.44382	78.004435	0.9286429	0.02263525	4.098
gi|166796054|ref|NM_001114281.1|	lightcyan	403.29224	58.073673	0.9898731	0.00122149	6.068
gi|167908788|ref|NM_001114675.1|	turquoise	925.57694	215.87892	0.9566851	0.01075096	18.742
gi|167908792|ref|NM_001114670.1|	turquoise	286.50646	105.26256	0.7363592	0.15591392	23.504
gi|167908794|ref|NM_001114671.1|	turquoise	351.19606	61.621815	-0.031295	0.9601605	44.126
gi|171905894|gb|EU561660.1|	turquoise	620.51703	162.43046	0.8623086	0.06005021	367.422
gi|172072681|ref|NM_001122994.1|	royalblue	547.4494	62.311426	0.9923832	0.00079706	72.584
gi|172073180|ref|NM_001099931.1|	pink	957.01097	146.6234	0.9952485	0.00039289	17.638

gi|178056465|ref|NM_001123083.1|	brown	593.91266	119.91442	0.9713445	0.0057979	9.876
gi|178056481|ref|NM_001123135.1|	darkgrey	959.24191	51.311628	0.985663	0.00205628	6.244
gi|178056483|ref|NM_001123101.1|	black	698.09098	103.32771	0.9887403	0.00143183	34.612
gi|178056487|ref|NM_001123204.1|	turquoise	354.14228	112.66902	0.7390376	0.15361162	28.176
gi|178056503|ref|NM_001123076.1|	greenyellow	82.280878	12.846718	0.4249105	0.47573912	829.288
gi|178056523|ref|NM_001123127.1|	grey60	315.04584	69.206442	0.9992449	2.49E-05	5.114
gi|178056529|ref|NM_001123203.1|	yellow	309.87416	121.34568	0.9732352	0.00523513	10.174
gi|178056549|ref|NM_001123162.1|	pink	897.81561	146.25528	0.9950693	0.00041532	1.79
gi|178056555|ref|NM_001123148.1|	darkturquoise	231.07641	36.328035	0.9318867	0.02112	29.802
gi|178056566|ref|NM_001123160.1|	darkorange	109.89605	18.307581	0.8149394	0.09286795	51.46
gi|178056595|ref|NM_001123136.1|	grey60	299.00251	63.540118	0.9805779	0.00323974	39.052
gi|178056622|ref|NM_001123213.1|	blue	907.59156	150.43115	0.9955717	0.00035351	47.842
gi|178056662|ref|NM_001123124.1|	royalblue	474.48524	56.885254	0.9720159	0.00559588	4.5
gi|178056668|ref|NM_001123099.1|	brown	683.51166	134.03451	0.9951781	0.00040165	87.994
gi|178056676|ref|NM_001123078.1|	magenta	264.7038	84.560275	0.9949961	0.00042459	279.664
gi|178056709|ref|NM_001123194.1|	skyblue	700.15653	34.426755	0.9387365	0.01803453	6.224
gi|178056815|ref|NM_001123159.1|	violet	43.268567	9.319737	-0.928548	0.02268018	16.202
gi|178056859|ref|NM_001123091.1|	lightcyan	622.77357	50.551302	0.9398233	0.01755971	33.896
gi|178056883|ref|NM_001123198.1|	blue	830.01594	148.85522	0.994369	0.00050681	8.504
gi|178057054|ref|NM_001123079.1|	darkturquoise	240.92448	40.022155	0.9561786	0.01093925	25.056
gi|178057066|ref|NM_001123096.1|	darkorange	138.02542	28.961233	0.949599	0.01347975	43.83
gi|178057124|ref|NM_001123104.1|	blue	371.19186	77.10009	0.8510787	0.06742514	22.016
gi|178057176|ref|NM_001123090.1|	turquoise	983.51425	225.39909	0.9692148	0.00645402	18.672
gi|178057317|ref|NM_001123205.1|	darkgreen	233.69738	42.022009	0.9862167	0.00193848	57.096
gi|187672044|gb|EU650276.1|	magenta	444.26856	73.377688	0.953907	0.0117967	18.88
gi|190360618|ref|NM_001128433.1|	lightcyan	568.46889	53.639889	0.959022	0.0098963	97.63
gi|190360620|ref|NM_001128486.1|	lightyellow	279.14128	50.353247	0.9434866	0.01598987	6.392
gi|190360638|ref|NM_001128469.1|	lightcyan	272.51952	40.708078	0.9060486	0.03407781	24.934
gi|190360656|ref|NM_001128470.1|	lightcyan	215.46066	37.710494	0.8750073	0.05204107	112.022
gi|1912|emb|X07617.1|	lightyellow	530.77171	53.982422	0.9630172	0.00849004	9.726
gi|1920|emb|X15073.1|	midnightblue	903.45757	76.965821	0.9945546	0.00048198	13.336
gi|194018685|ref|NM_001129963.1|	turquoise	278.91866	87.678458	0.6627361	0.22284102	425.022
gi|194018717|ref|NM_001129954.1|	black	637.37659	90.172364	0.9609343	0.00921437	1460.098
gi|194018721|ref|NM_001129949.1|	violet	46.062555	9.0899648	-0.981551	0.00299976	33475.862
gi|194033408|ref|XM_001926440.1|	brown	680.29691	132.47491	0.9935292	0.00062424	102.552
gi|194033418|ref|XM_001924233.1|	orange	333.05997	34.596077	0.9820826	0.00287129	10.99
gi|194033446|ref|XM_001927265.1|	turquoise	996.71291	209.44988	0.9336394	0.02031554	5.184
gi|194033502|ref|XM_001926129.1|	yellow	310.75	130.66756	0.9920962	0.00084251	5.458
gi|194033586|ref|XM_001929183.1|	greenyellow	121.53395	20.983117	0.8327576	0.08001049	18.302
gi|194033590|ref|XM_001929195.1|	lightyellow	589.93126	58.63094	0.9771243	0.00413903	114.69
gi|194033594|ref|XM_001927795.1|	turquoise	975.0972	217.96703	0.9528098	0.01221836	8.902
gi|194033596|ref|XM_001927813.1|	brown	143.72511	14.430694	0.416073	0.48594818	5.77
gi|194033615|ref|XM_001924714.1|	darkorange	158.13059	31.5298	0.9692818	0.00643304	4.156
gi|194033638|ref|XM_001927936.1|	turquoise	579.50064	178.22831	0.941561	0.01680908	35.348
gi|194033649|ref|XM_001925294.1|	darkred	188.75445	32.828823	0.8843067	0.04641006	8.56
gi|194033677|ref|XM_001928828.1|	turquoise	211.1081	80.681851	0.6710244	0.21497731	16.744
gi|194033685|ref|XM_001926077.1|	darkturquoise	152.23752	40.714516	0.9614174	0.00904466	37.176
gi|194033718|ref|XM_001926719.1|	turquoise	430.95405	59.722974	0.084581	0.89243663	15.306

gi|194033751|ref|XM_001924525.1|	pink	976.83549	142.41426	0.9890989	0.00136404	4.278
gi|194033753|ref|XM_001924715.1|	purple	258.58814	73.740306	0.983075	0.00263646	23.472
gi|194033755|ref|XM_001924851.1|	navy	84.718662	28.437762	0.9035437	0.03543596	30.258
gi|194033859|ref|XM_001927866.1|	darkgreen	266.9809	41.291621	0.985373	0.00211891	185.242
gi|194033888|ref|XM_001925347.1|	turquoise	918.14224	210.70592	0.9522522	0.01243451	100.61
gi|194033910|ref|XM_001928928.1|	grey60	313.35903	68.773426	0.9980927	1.00E-04	47.064
gi|194033964|ref|XM_001925719.1|	paleturquoise	175.11129	23.025982	0.9344004	0.01996943	3.716
gi|194034011|ref|XM_001927812.1|	brown	577.79684	113.66547	0.9589533	0.0099211	25.542
gi|194034018|ref|XM_001925500.1|	turquoise	882.37402	218.13269	0.9702442	0.00613399	18.238
gi|194034042|ref|XM_001925859.1|	darkturquoise	145.81699	39.212074	0.9513418	0.01279006	15.046
gi|194034126|ref|XM_001926626.1|	blue	964.97775	144.80087	0.9853072	0.00213319	10.3
gi|194034144|ref|XM_001926437.1|	turquoise	334.01254	99.070598	0.7401442	0.15266332	8.272
gi|194034148|ref|XM_001924679.1|	turquoise	854.60376	213.90902	0.9554852	0.01119875	40.152
gi|194034158|ref|XM_001927669.1|	green	864.51866	108.42914	0.981207	0.0030839	22.924
gi|194034162|ref|XM_001925693.1|	magenta	438.3793	75.156805	0.9594965	0.00972562	61.882
gi|194034164|ref|XM_001925794.1|	lightyellow	312.44864	55.922494	0.9674694	0.00700876	5.208
gi|194034166|ref|XM_001925915.1|	yellow	244.95466	117.22557	0.9679641	0.00685002	54.804
gi|194034216|ref|XM_001928595.1|	turquoise	387.31766	57.253786	-0.011919	0.98482432	90.926
gi|194034220|ref|XM_001928748.1|	turquoise	839.65085	206.61364	0.9560604	0.01098336	20.238
gi|194034226|ref|XM_001928937.1|	darkorange	99.778214	9.732801	0.5816004	0.30365773	90.926
gi|194034238|ref|XM_001924848.1|	lightcyan	501.04635	58.627218	0.9847018	0.00226619	10.068
gi|194034244|ref|XM_001925555.1|	green	832.59569	112.25756	0.9902585	0.0011525	7.5
gi|194034359|ref|XM_001927037.1|	darkred	127.59167	29.8741	0.8955704	0.03987006	41.094
gi|194034393|ref|XM_001926625.1|	royalblue	741.99906	59.184223	0.9807911	0.00318663	12.708
gi|194034443|ref|XM_001928249.1|	turquoise	956.92097	201.37437	0.9211159	0.02627914	4.306
gi|194034463|ref|XM_001926549.1|	black	814.43549	105.16383	0.9922699	0.00081491	58.406
gi|194034501|ref|XM_001925096.1|	magenta	299.98318	80.367673	0.9841637	0.0023866	4.634
gi|194034516|ref|XM_001925886.1|	brown	399.7604	70.337795	0.8430492	0.07285864	1.578
gi|194034537|ref|XM_001925533.1|	turquoise	515.92981	58.16378	0.2368848	0.70123393	15.614
gi|194034539|ref|XM_001927513.1|	black	804.03664	107.10313	0.9962689	0.00027343	8.916
gi|194034592|ref|XM_001925373.1|	green	816.39103	87.504424	0.9341629	0.02007722	19.994
gi|194034626|ref|XM_001929026.1|	turquoise	826.04697	185.46356	0.9062982	0.0339434	30.252
gi|194034657|ref|XM_001924580.1|	pink	731.32188	134.6219	0.9782504	0.00383785	27.486
gi|194034678|ref|XM_001928189.1|	lightcyan	461.26629	52.894848	0.9653408	0.00770533	15.56
gi|194034680|ref|XM_001925553.1|	turquoise	182.89198	49.360273	0.4121536	0.49049061	11.028
gi|194034692|ref|XM_001928709.1|	royalblue	769.08148	59.796527	0.9822401	0.00283358	9.4
gi|194034703|ref|XM_001926714.1|	turquoise	947.42117	213.67815	0.9485321	0.01390775	11.468
gi|194034713|ref|XM_001925291.1|	royalblue	429.33442	54.491357	0.9616044	0.00897923	8.566
gi|194034832|ref|XM_001928118.1|	turquoise	602.39265	176.54681	0.907274	0.03341963	21.026
gi|194034834|ref|XM_001925793.1|	darkred	67.078464	8.234767	0.5279611	0.36046533	79.876
gi|194034838|ref|XM_001928143.1|	purple	290.58943	79.537145	0.9978235	0.00012185	6.112
gi|194034874|ref|XM_001929334.1|	violet	898.4166	46.676847	0.9893048	0.00132563	20.74
gi|194034890|ref|XM_001929399.1|	yellow	256.94442	122.09373	0.9767024	0.0042538	22.824
gi|194034900|ref|XM_001924788.1|	lightgreen	145.13798	19.933271	0.7201238	0.17007989	15.232
gi|194034933|ref|XM_001927442.1|	turquoise	1016.9373	209.6591	0.9318075	0.02115661	6.372
gi|194034969|ref|XM_001925674.1|	midnightblue	997.67708	71.916711	0.9797241	0.00345525	1093.45
gi|194035059|ref|XM_001924311.1|	orange	167.34585	33.286098	0.9489323	0.0137467	3.27
gi|194035100|ref|XM_001925477.1|	darkgrey	743.65113	46.413991	0.9659629	0.0074995	11.006

gi|194035108|ref|XM_001926680.1|	turquoise	134.60638	33.124669	-0.415869	0.48618395	37.754
gi|194035136|ref|XM_001924656.1|	navy	48.374028	6.4429902	0.3899782	0.51635403	54.408
gi|194035172|ref|XM_001924332.1|	purple	148.37316	39.676682	0.8421874	0.07344955	9.742
gi|194035184|ref|XM_001924626.1|	grey	572.89253	13.986957	0.9735968	0.00512969	27.956
gi|194035216|ref|XM_001928520.1|	darkturquoise	120.4354	32.975342	0.9133191	0.03023361	90.988
gi|194035235|ref|XM_001924419.1|	navy	179.12099	39.85774	0.9831294	0.00262377	15.902
gi|194035245|ref|XM_001924870.1|	pink	973.23687	142.71182	0.9895442	0.0012814	8.786
gi|194035288|ref|XM_001927095.1|	midnightblue	947.61519	67.750378	0.9671481	0.00711251	6.846
gi|194035296|ref|XM_001927594.1|	turquoise	124.04873	46.239186	0.5547969	0.33173902	56.57
gi|194035304|ref|XM_001928396.1|	lightyellow	327.53589	55.044945	0.9643873	0.00802432	5.426
gi|194035333|ref|XM_001925774.1|	darkgreen	77.715385	10.545285	0.6184176	0.26616159	19.224
gi|194035378|ref|XM_001924314.1|	magenta	216.46879	77.227023	0.973585	0.00513312	2
gi|194035408|ref|XM_001926476.1|	pink	718.36997	131.02379	0.9727929	0.00536508	17.852
gi|194035493|ref|XM_001926740.1|	darkgreen	219.16473	18.63318	0.7419418	0.15112648	9.826
gi|194035503|ref|XM_001927458.1|	darkgreen	247.2838	43.03569	0.9947366	0.00045803	60.18
gi|194035509|ref|XM_001927620.1|	yellow	227.72761	84.071035	0.8924751	0.04163568	26.878
gi|194035517|ref|XM_001925462.1|	blue	393.20815	91.435914	0.8862099	0.04528304	21.496
gi|194035561|ref|XM_001924535.1|	blue	478.7791	109.23333	0.9251523	0.02430326	7.206
gi|194035592|ref|XM_001927531.1|	darkmagenta	149.67037	8.4342875	0.8609837	0.06090632	18.92
gi|194035600|ref|XM_001927804.1|	turquoise	667.65782	191.5725	0.9384711	0.01815111	186.116
gi|194035607|ref|XM_001928505.1|	midnightblue	704.24996	70.620754	0.9764802	0.00431466	10.196
gi|194035627|ref|XM_001924280.1|	navy	122.81927	38.759157	0.9456828	0.01507194	22.276
gi|194035657|ref|XM_001928441.1|	turquoise	946.62865	208.5186	0.9381602	0.01828799	8.218
gi|194035679|ref|XM_001925389.1|	turquoise	492.77413	59.610729	0.2227029	0.71880752	175.628
gi|194035693|ref|XM_001925021.1|	brown	662.20358	134.44006	0.9981424	9.61E-05	5.09
gi|194035716|ref|XM_001925987.1|	lightcyan	317.35099	55.02576	0.9796846	0.00346534	26.31
gi|194035722|ref|XM_001926997.1|	turquoise	206.42355	73.225564	0.6317769	0.25288507	24.978
gi|194035733|ref|XM_001925711.1|	navy	77.752288	9.1519027	-0.801528	0.10292284	11.378
gi|194035737|ref|XM_001928295.1|	lightgreen	431.02627	38.349988	0.9332744	0.02048224	97.734
gi|194035770|ref|XM_001927929.1|	magenta	136.39399	55.864934	0.8925295	0.04160448	11.758
gi|194035776|ref|XM_001929384.1|	yellow	258.41001	121.33863	0.9762051	0.0043904	87.38
gi|194035796|ref|XM_001927740.1|	turquoise	683.40734	137.80486	0.8042466	0.10085926	34.222
gi|194035836|ref|XM_001928869.1|	royalblue	712.47663	61.935105	0.9911142	0.00100414	44.664
gi|194035846|ref|XM_001928960.1|	green	904.67282	104.1095	0.9700274	0.00620095	36.472
gi|194035848|ref|XM_001928966.1|	lightyellow	213.18922	41.188637	0.8991603	0.03785304	351.124
gi|194035852|ref|XM_001929031.1|	blue	937.87279	141.38407	0.9809437	0.00314882	32.292
gi|194035954|ref|XM_001927715.1|	darkred	130.00598	24.746176	0.8397224	0.07514779	12.962
gi|194035974|ref|XM_001924643.1|	lightgreen	388.57729	35.890982	0.9332623	0.02048777	14
gi|194035999|ref|XM_001924070.1|	brown	763.49415	133.90238	0.9936937	0.0006006	127.11
gi|194036005|ref|XM_001926377.1|	turquoise	339.8448	116.76059	0.7780483	0.12125689	95.21
gi|194036011|ref|XM_001926398.1|	midnightblue	944.47799	71.164226	0.9777202	0.00397872	5.498
gi|194036046|ref|XM_001928851.1|	midnightblue	764.86998	59.03149	0.9396985	0.01761401	18.688
gi|194036068|ref|XM_001926844.1|	turquoise	1005.5271	220.23036	0.9549814	0.01138852	3.528
gi|194036118|ref|XM_001927231.1|	turquoise	452.53964	116.78174	0.7564901	0.13885848	3.404
gi|194036127|ref|XM_001929512.1|	brown	514.0712	109.30153	0.9538718	0.01181014	66.488
gi|194036134|ref|XM_001929587.1|	lightgreen	261.13373	29.994988	0.8678908	0.05648519	12.146
gi|194036224|ref|XM_001929641.1|	darkred	190.68163	42.515183	0.9634822	0.00833102	84.986
gi|194036226|ref|XM_001929643.1|	darkgreen	178.85859	21.50819	0.8154021	0.09252676	26.75

gi|194036240|ref|XM_001929654.1|	darkgreen	181.11787	19.121929	0.7356002	0.15656819	12.734
gi|194036252|ref|XM_001929660.1|	turquoise	205.13796	71.128959	0.5887614	0.29626428	41.512
gi|194036256|ref|XM_001929662.1|	turquoise	162.80289	64.000629	0.5647325	0.32125624	1.246
gi|194036268|ref|XM_001929668.1|	pink	828.63074	146.09322	0.9948978	0.00043715	7.874
gi|194036284|ref|XM_001924881.1|	salmon	194.3165	62.961301	0.9546076	0.01153001	50.076
gi|194036295|ref|XM_001926489.1|	midnightblue	989.17182	69.037178	0.9710698	0.00588121	7.184
gi|194036309|ref|XM_001927637.1|	brown	59.613161	5.8855109	0.3272828	0.5908542	328.15
gi|194036315|ref|XM_001927860.1|	darkgrey	921.85502	53.000004	0.9934335	0.00063812	69.232
gi|194036317|ref|XM_001927969.1|	darkgrey	790.58339	48.453589	0.9749133	0.0047518	8.112
gi|194036348|ref|XM_001928686.1|	midnightblue	812.75501	74.576281	0.9881236	0.00155091	10.568
gi|194036390|ref|XM_001929343.1|	turquoise	200.46633	81.479781	0.6389569	0.24582568	46.902
gi|194036406|ref|XM_001927918.1|	pink	930.29135	149.17918	0.998958	4.04E-05	8.584
gi|194036424|ref|XM_001925710.1|	turquoise	348.40326	80.59803	0.6243406	0.26025278	45.752
gi|194036434|ref|XM_001926977.1|	darkorange	87.926517	22.995621	0.8604814	0.06123191	17.246
gi|194036458|ref|XM_001928544.1|	turquoise	109.01719	31.303426	0.4420117	0.4561175	5.21
gi|194036460|ref|XM_001926279.1|	purple	285.98113	79.263706	0.9974431	0.00015514	8.68
gi|194036462|ref|XM_001928649.1|	green	863.15396	106.30625	0.9757919	0.00450498	67.268
gi|194036467|ref|XM_001928805.1|	turquoise	633.14526	146.53796	0.8408236	0.07438765	25.378
gi|194036542|ref|XM_001925670.1|	turquoise	792.3469	204.75622	0.9648848	0.00785733	109.568
gi|194036571|ref|XM_001927806.1|	blue	892.68524	131.08626	0.9626509	0.00861599	26.716
gi|194036621|ref|XM_001927321.1|	turquoise	305.20083	50.125462	-0.056688	0.92786085	120.608
gi|194036623|ref|XM_001927665.1|	midnightblue	901.24937	76.945641	0.994654	0.00046885	11.282
gi|194036639|ref|XM_001924618.1|	black	735.10541	104.88465	0.9919919	0.00085923	28.946
gi|194036656|ref|XM_001928010.1|	darkgrey	1000.6718	50.622131	0.9825573	0.00275814	18.394
gi|194036668|ref|XM_001926328.1|	grey	595.30599	14.300965	0.9749921	0.00472948	14.752
gi|194036700|ref|XM_001927375.1|	red	784.19887	114.93586	0.9781278	0.00387027	5.724
gi|194036737|ref|XM_001927243.1|	green	943.35913	105.25109	0.9720095	0.00559779	38.256
gi|194036739|ref|XM_001927255.1|	blue	988.23535	135.14179	0.9685912	0.0066505	7.076
gi|194036748|ref|XM_001928875.1|	turquoise	240.27808	54.329227	-0.256082	0.67754564	3.502
gi|194036762|ref|XM_001925928.1|	blue	631.40716	134.6723	0.9724204	0.00547532	87.986
gi|194036768|ref|XM_001927459.1|	darkgrey	932.22167	54.31363	0.9985021	6.96E-05	2.318
gi|194036770|ref|XM_001925929.1|	navy	124.75324	39.30141	0.9531829	0.01207445	2.306
gi|194036795|ref|XM_001926863.1|	grey60	311.90302	69.086939	0.9989783	3.92E-05	174.856
gi|194036805|ref|XM_001928924.1|	turquoise	362.60614	60.190832	0.0249556	0.9682289	53.816
gi|194036851|ref|XM_001928643.1|	lightgreen	384.5819	26.276843	0.8801981	0.04887278	28.772
gi|194036879|ref|XM_001927666.1|	black	943.16914	96.651095	0.9733251	0.00520886	155.17
gi|194036893|ref|XM_001927714.1|	navy	349.23392	20.383485	0.8076291	0.09830969	15.028
gi|194036901|ref|XM_001927323.1|	turquoise	265.26545	96.52481	0.7655752	0.13135457	52.318
gi|194036936|ref|XM_001925783.1|	green	868.57166	116.12826	0.9974498	0.00015454	8.698
gi|194036950|ref|XM_001928451.1|	navy	63.196496	26.314096	0.8778812	0.05027916	14.1
gi|194036956|ref|XM_001924342.1|	royalblue	665.15001	63.121938	0.995471	0.00036563	11.508
gi|194036972|ref|XM_001927228.1|	turquoise	392.10885	136.69505	0.8590123	0.06218721	30.534
gi|194036974|ref|XM_001927747.1|	brown	830.95867	124.12301	0.969714	0.00629818	145.628
gi|194037015|ref|XM_001929116.1|	turquoise	224.55357	91.463556	0.6888315	0.19835264	73.85
gi|194037021|ref|XM_001926975.1|	royalblue	676.23593	61.433109	0.9895109	0.00128752	5.482
gi|194037094|ref|XM_001927574.1|	greenyellow	48.493293	9.7655141	0.2005613	0.74635987	117.348
gi|194037139|ref|XM_001925875.1|	purple	225.68266	64.119413	0.9527203	0.01225297	19.742
gi|194037172|ref|XM_001928663.1|	turquoise	190.13882	76.671424	0.6279963	0.25662366	70.81

gi|194037193|ref|XM_001929169.1|	turquoise	123.57975	38.258388	0.4430532	0.45492827	8.274
gi|194037198|ref|XM_001929213.1|	orange	125.79154	27.770677	0.8883187	0.04404455	50.594
gi|194037354|ref|XM_001926198.1|	salmon	217.29811	70.353432	0.9770286	0.00416497	52.78
gi|194037372|ref|XM_001927549.1|	green	704.95893	97.562549	0.9611605	0.00913476	15.442
gi|194037379|ref|XM_001925252.1|	blue	918.406	142.65174	0.9823954	0.00279654	5.07
gi|194037499|ref|XM_001928879.1|	royalblue	634.15363	59.036777	0.9817705	0.00294649	16.17
gi|194037532|ref|XM_001929272.1|	darkorange	173.97792	30.587878	0.9645927	0.00795523	350.794
gi|194037543|ref|XM_001929293.1|	turquoise	221.63645	82.892722	0.6779867	0.20843287	33.104
gi|194037553|ref|XM_001929410.1|	greenyellow	168.25649	22.992314	0.7458615	0.14779123	1429.172
gi|194037555|ref|XM_001929413.1|	darkred	38.848783	5.1974738	0.4763732	0.41725537	46.446
gi|194037571|ref|XM_001929457.1|	turquoise	911.55409	218.51706	0.9665716	0.00729992	16.694
gi|194037611|ref|XM_001925424.1|	paleturquoise	171.12706	22.464989	0.9392911	0.0177917	20.352
gi|194037617|ref|XM_001927127.1|	blue	890.59092	152.07947	0.9981529	9.53E-05	48.608
gi|194037680|ref|XM_001926355.1|	brown	87.346177	7.0111875	0.3703011	0.53952648	8.016
gi|194037682|ref|XM_001927000.1|	black	914.04871	102.7422	0.986535	0.00187183	15.938
gi|194037707|ref|XM_001924251.1|	navy	105.46184	38.730186	0.9739235	0.00503503	19.654
gi|194037755|ref|XM_001925059.1|	green	889.29654	110.02046	0.9834851	0.00254138	10.48
gi|194037797|ref|XM_001925445.1|	blue	917.73318	150.12069	0.994833	0.00044551	11.596
gi|194037819|ref|XM_001928548.1|	yellow	274.18358	129.13612	0.9901892	0.00116479	8.506
gi|194037863|ref|XM_001925621.1|	yellow	205.15822	90.930507	0.9087516	0.03263144	86.31
gi|194037865|ref|XM_001928013.1|	darkturquoise	149.86738	38.776833	0.9517782	0.01261921	41.448
gi|194037879|ref|XM_001925784.1|	red	901.22843	126.72527	0.9977726	0.00012615	11.164
gi|194037903|ref|XM_001927180.1|	purple	256.82755	57.864502	0.9224889	0.02560146	24.806
gi|194037930|ref|XM_001928852.1|	turquoise	959.30022	210.05534	0.9432536	0.0160883	13.558
gi|194037950|ref|XM_001926744.1|	tan	107.90595	27.969298	0.8070065	0.09877751	6.158
gi|194037952|ref|XM_001924515.1|	darkorange	151.66353	33.642775	0.9898812	0.00122002	64.628
gi|194037997|ref|XM_001926200.1|	violet	990.19088	43.677214	0.9569133	0.0106665	17.92
gi|194038023|ref|XM_001928502.1|	tan	136.7527	39.886979	0.9447594	0.01545575	31.88
gi|194038027|ref|XM_001928638.1|	darkorange	118.92851	28.80874	0.932387	0.02088935	2.146
gi|194038031|ref|XM_001928655.1|	salmon	99.299672	39.063937	0.8543025	0.06528081	7.126
gi|194038037|ref|XM_001929120.1|	skyblue	512.17325	42.76928	0.9912071	0.00098846	63.398
gi|194038045|ref|XM_001925447.1|	brown	756.91638	132.62837	0.9918337	0.00088479	6.526
gi|194038066|ref|XM_001927909.1|	greenyellow	71.210466	10.715546	0.537814	0.34984985	157.11
gi|194038088|ref|XM_001925288.1|	lightyellow	495.64954	63.230066	0.9951397	0.00040645	23.108
gi|194038104|ref|XM_001925531.1|	green	871.69895	111.70218	0.9884765	0.00148238	14.398
gi|194038194|ref|XM_001927473.1|	green	807.48723	109.86867	0.9860817	0.001967	75.722
gi|194038223|ref|XM_001926312.1|	lightyellow	575.50939	60.235378	0.9833263	0.00257805	1.482
gi|194038225|ref|XM_001926546.1|	lightcyan	493.14583	37.831753	0.8834714	0.04690748	12.58
gi|194038241|ref|XM_001927985.1|	turquoise	209.3505	81.443987	0.5966638	0.28816052	43.488
gi|194038245|ref|XM_001928187.1|	violet	90.647567	7.5552643	-0.782826	0.11745392	207.306
gi|194038277|ref|XM_001926890.1|	turquoise	458.63572	138.91566	0.8196264	0.08942925	38.65
gi|194038297|ref|XM_001926748.1|	yellow	292.70558	130.63544	0.9924175	0.0007917	7.518
gi|194038313|ref|XM_001925341.1|	violet	43.197196	8.3182122	-0.937201	0.01871237	75.882
gi|194038369|ref|XM_001929075.1|	orange	430.77861	14.206532	0.6952522	0.19245148	45.766
gi|194038432|ref|XM_001925495.1|	salmon	106.54847	41.928149	0.8691501	0.05569046	13.894
gi|194038454|ref|XM_001928679.1|	turquoise	452.72378	112.66777	0.7781772	0.12115382	41.792
gi|194038464|ref|XM_001924308.1|	skyblue	495.23077	42.948798	0.992296	0.00081079	131.502
gi|194038478|ref|XM_001926160.1|	yellow	310.66149	127.27382	0.9854801	0.00209571	22.55

gi|194038505|ref|XM_001928042.1|	turquoise	159.17634	56.434235	0.5619392	0.32419482	17.636
gi|194038533|ref|XM_001929074.1|	blue	841.25435	149.02461	0.9939996	0.00055746	49.842
gi|194038541|ref|XM_001929173.1|	turquoise	158.41571	38.747328	-0.491624	0.40026398	49.476
gi|194038545|ref|XM_001929151.1|	navy	146.22358	40.765433	0.9654255	0.00767719	4.882
gi|194038579|ref|XM_001926122.1|	green	775.32196	111.05888	0.9891565	0.00135326	10.864
gi|194038585|ref|XM_001926711.1|	turquoise	535.26505	168.22255	0.9086855	0.03266661	120.414
gi|194038610|ref|XM_001926123.1|	turquoise	192.33954	55.518829	0.5180305	0.37124213	11.318
gi|194038708|ref|XM_001928665.1|	lightyellow	419.31931	58.969615	0.9810412	0.00312472	76.942
gi|194038714|ref|XM_001926092.1|	darkorange	112.30916	32.237131	0.9609309	0.00921557	11.7
gi|194038723|ref|XM_001929103.1|	darkgreen	153.17624	30.608605	0.8873801	0.0445944	27.892
gi|194038729|ref|XM_001929090.1|	magenta	176.85945	66.62668	0.9377471	0.01847039	283.6
gi|194038781|ref|XM_001926473.1|	turquoise	116.38506	32.154199	0.4168253	0.4850774	76.47
gi|194038808|ref|XM_001924784.1|	skyblue	638.25661	37.797957	0.9608634	0.00923939	70.276
gi|194038816|ref|XM_001925028.1|	pink	911.2619	149.78175	0.9998705	1.77E-06	4.632
gi|194038854|ref|XM_001928014.1|	red	917.25604	125.37381	0.9954366	0.0003698	29.94
gi|194038856|ref|XM_001925833.1|	blue	996.45624	132.95072	0.9646183	0.00794665	9.872
gi|194038862|ref|XM_001928271.1|	green	567.11813	87.059643	0.9361023	0.01920237	47.53
gi|194038869|ref|XM_001928468.1|	pink	892.52327	144.08521	0.9920432	0.00085099	5.94
gi|194038879|ref|XM_001928723.1|	turquoise	456.50776	60.36423	0.1503247	0.80932396	8.966
gi|194038896|ref|XM_001924415.1|	navy	79.968222	32.311154	0.8865319	0.04509322	20.822
gi|194038900|ref|XM_001925133.1|	lightcyan	591.25612	54.662393	0.9597371	0.00963942	14.402
gi|194038904|ref|XM_001924445.1|	yellow	291.29386	132.18868	0.9946441	0.00047015	178.25
gi|194038910|ref|XM_001926121.1|	salmon	227.01325	75.851932	0.9941607	0.00053517	30.168
gi|194038924|ref|XM_001927577.1|	violet	991.8746	43.159959	0.9531155	0.01210039	19.658
gi|194038958|ref|XM_001929118.1|	black	854.38294	103.57153	0.9891751	0.00134978	10.732
gi|194039226|ref|XM_001928899.1|	greenyellow	181.79011	37.790486	0.9633626	0.0083718	260.556
gi|194039238|ref|XM_001924410.1|	violet	946.93427	45.941053	0.9797175	0.00345694	1.938
gi|194039264|ref|XM_001927148.1|	turquoise	300.41248	48.838835	-0.041591	0.94705995	27.506
gi|194039300|ref|XM_001928656.1|	salmon	225.57235	77.699046	0.9990269	3.64E-05	5.78
gi|194039310|ref|XM_001929246.1|	turquoise	491.32489	158.25726	0.8869608	0.04484078	23.792
gi|194039316|ref|XM_001929265.1|	brown	160.49364	11.015594	0.4861385	0.40635645	69.186
gi|194039362|ref|XM_001929482.1|	darkgreen	61.291201	12.950032	0.6577463	0.22761252	46.32
gi|194039364|ref|XM_001929510.1|	red	953.13388	120.71263	0.9872388	0.00172718	10.718
gi|194039388|ref|XM_001929568.1|	blue	764.70725	149.34827	0.9959011	0.00031482	18.646
gi|194039390|ref|XM_001929570.1|	blue	542.12714	88.742881	0.8757144	0.0516058	260.05
gi|194039392|ref|XM_001929581.1|	darkorange	83.394908	19.063883	0.8138286	0.09368878	13.614
gi|194039480|ref|XM_001926227.1|	turquoise	842.47168	213.86949	0.9691435	0.00647639	59.532
gi|194039486|ref|XM_001927002.1|	orange	215.03808	14.443812	0.6906202	0.19670365	31.934
gi|194039490|ref|XM_001927338.1|	blue	960.37069	138.66968	0.9752871	0.00464626	185.96
gi|194039546|ref|XM_001928863.1|	turquoise	991.76879	206.09726	0.9273852	0.02323183	17.856
gi|194039573|ref|XM_001927296.1|	turquoise	439.41836	147.15439	0.8557462	0.06432754	52.552
gi|194039577|ref|XM_001927339.1|	magenta	374.46309	82.205185	0.9844894	0.00231347	19.804
gi|194039585|ref|XM_001929378.1|	brown	840.10341	123.10828	0.9677357	0.00692317	9.676
gi|194039587|ref|XM_001929390.1|	green	872.98958	111.47956	0.9875146	0.00167156	15.144
gi|194039595|ref|XM_001929425.1|	green	644.19265	75.228295	0.9058543	0.03418256	12.44
gi|194039625|ref|XM_001925622.1|	pink	891.69021	149.36923	0.9993371	2.05E-05	5.476
gi|194039636|ref|XM_001927064.1|	darkred	162.22688	37.342035	0.9461579	0.01487572	28.226
gi|194039679|ref|XM_001928732.1|	darkgrey	860.45532	51.027276	0.985645	0.00206017	154.422

gi|194039687|ref|XM_001928955.1|	turquoise	949.05428	197.94538	0.9129025	0.03044988	24.594
gi|194039697|ref|XM_001929172.1|	yellow	238.67089	73.049304	0.8540116	0.06547338	36.354
gi|194039707|ref|XM_001929228.1|	lightyellow	347.81219	58.026459	0.9762992	0.00436444	44.868
gi|194039709|ref|XM_001929236.1|	magenta	341.68374	72.887703	0.9596619	0.00966634	36.068
gi|194039733|ref|XM_001928398.1|	royalblue	545.71747	61.494483	0.9896168	0.00126811	8.662
gi|194039735|ref|XM_001928389.1|	royalblue	744.16896	62.099494	0.9912767	0.00097676	152.064
gi|194039851|ref|XM_001928253.1|	turquoise	507.27833	146.85592	0.8442312	0.07205061	33.43
gi|194039979|ref|XM_001927951.1|	greenyellow	206.75274	29.292393	0.8545504	0.06511683	0.996
gi|194040110|ref|XM_001929541.1|	purple	304.20968	74.442276	0.9816107	0.00298525	31.47
gi|194040173|ref|XM_001926225.1|	purple	270.30626	68.114475	0.9590465	0.00988747	70.666
gi|194040232|ref|XM_001929613.1|	black	770.99855	105.18244	0.9926015	0.00076307	10.72
gi|194040254|ref|XM_001929631.1|	brown	191.86749	28.622188	0.6340473	0.25064691	19.664
gi|194040263|ref|XM_001927880.1|	greenyellow	157.03418	29.364645	0.9257713	0.02400468	32.916
gi|194040280|ref|XM_001925339.1|	turquoise	904.26804	206.80468	0.9327763	0.02071045	8.634
gi|194040288|ref|XM_001927655.1|	yellow	310.0952	124.66822	0.9795862	0.00349047	5.028
gi|194040321|ref|XM_001928400.1|	turquoise	298.92891	104.05228	0.7184176	0.17158908	14.554
gi|194040325|ref|XM_001928445.1|	greenyellow	201.59211	39.068335	0.9811768	0.00309132	3.888
gi|194040339|ref|XM_001928739.1|	brown	341.12461	58.830878	0.8040599	0.10100056	25.18
gi|194040341|ref|XM_001928820.1|	royalblue	825.01031	53.722381	0.9571111	0.01059347	18.634
gi|194040377|ref|XM_001929415.1|	blue	647.47516	136.0277	0.9760471	0.0044341	7.018
gi|194040411|ref|XM_001929558.1|	turquoise	947.47478	221.84339	0.967485	0.00700374	117.254
gi|194040420|ref|XM_001929571.1|	black	872.65026	107.19231	0.9959998	0.00030352	6.92
gi|194040422|ref|XM_001929580.1|	greenyellow	153.48934	33.636352	0.9165916	0.02855215	92.386
gi|194040449|ref|XM_001927957.1|	greenyellow	55.062325	11.603393	0.2687539	0.66197701	167.77
gi|194040485|ref|XM_001926913.1|	turquoise	228.49111	50.054097	-0.291696	0.63393772	48.018
gi|194040487|ref|XM_001926988.1|	salmon	233.5142	73.530234	0.9869681	0.00178235	13.678
gi|194040489|ref|XM_001927223.1|	darkorange	108.37949	15.704486	0.7964714	0.10679421	18.074
gi|194040496|ref|XM_001928226.1|	turquoise	1010.8707	218.18078	0.9499813	0.01332746	9.51
gi|194040529|ref|XM_001924631.1|	lightyellow	666.83117	47.438608	0.9330989	0.02056257	8.596
gi|194040558|ref|XM_001928742.1|	blue	902.53415	150.39472	0.9951152	0.00040953	19.812
gi|194040603|ref|XM_001928485.1|	brown	805.70415	108.14974	0.9399603	0.01750011	21.884
gi|194040641|ref|XM_001925120.1|	turquoise	137.34615	54.003123	0.5282591	0.36014309	12.528
gi|194040677|ref|XM_001927466.1|	midnightblue	948.82822	78.084679	0.9973408	0.00016455	5.952
gi|194040706|ref|XM_001927201.1|	turquoise	391.53663	59.404772	0.0048255	0.99385598	89.624
gi|194040743|ref|XM_001927771.1|	turquoise	517.6192	156.85639	0.8622519	0.06008677	17.506
gi|194040755|ref|XM_001927097.1|	green	731.88332	107.13403	0.9823135	0.00281605	24.58
gi|194040778|ref|XM_001927213.1|	green	527.38389	82.770397	0.9245008	0.02461879	15.226
gi|194040784|ref|XM_001927731.1|	turquoise	1017.2913	207.92635	0.9277684	0.02304956	25.61
gi|194040788|ref|XM_001925300.1|	red	867.37383	125.04673	0.9951095	0.00041025	31.006
gi|194040816|ref|XM_001925581.1|	brown	708.28374	136.3472	0.9990906	3.29E-05	18.456
gi|194040900|ref|XM_001928469.1|	orange	308.57417	19.269242	0.7906061	0.11133856	68.16
gi|194040929|ref|XM_001925268.1|	royalblue	576.84583	63.138602	0.9956333	0.00034617	11.228
gi|194040939|ref|XM_001924716.1|	green	663.66932	104.54427	0.9761749	0.00439872	14.552
gi|194040961|ref|XM_001924920.1|	white	971.34336	46.988572	0.9923499	0.0008023	3.282
gi|194040979|ref|XM_001928486.1|	yellow	147.50203	66.374925	0.8407036	0.07447033	331.414
gi|194040981|ref|XM_001928597.1|	midnightblue	582.99457	56.126423	0.9292877	0.02233136	45.276
gi|194040983|ref|XM_001927342.1|	turquoise	464.11173	59.352439	0.1816113	0.77004282	74.196
gi|194040993|ref|XM_001924500.1|	lightgreen	442.78563	34.608344	0.9330087	0.02060387	7.162

gi|194041007|ref|XM_001926441.1|	brown	873.09281	115.30214	0.9511806	0.01285336	24.082
gi|194041034|ref|XM_001928236.1|	lightyellow	302.60667	52.240609	0.9537682	0.01184978	5.104
gi|194041080|ref|XM_001924608.1|	turquoise	255.09752	56.00468	-0.256857	0.67659175	5.4
gi|194041087|ref|XM_001927225.1|	salmon	231.405	76.485662	0.9950323	0.00041999	86.848
gi|194041154|ref|XM_001925630.1|	tan	148.84204	45.121009	0.9783141	0.00382105	13.19
gi|194041236|ref|XM_001928454.1|	pink	892.54668	144.93218	0.9931937	0.00067337	44.208
gi|194041241|ref|XM_001928861.1|	darkturquoise	256.85194	38.577502	0.9491067	0.01367671	1335.15
gi|194041305|ref|XM_001927960.1|	turquoise	369.41721	61.731647	-0.010909	0.98611077	19.19
gi|194041319|ref|XM_001928397.1|	darkorange	147.45495	11.991565	0.6595206	0.22591269	12.438
gi|194041329|ref|XM_001928735.1|	turquoise	970.51502	197.33858	0.9165416	0.02857764	2.478
gi|194041338|ref|XM_001928892.1|	grey60	308.5097	67.552455	0.9943832	0.0005049	13.948
gi|194041353|ref|XM_001926876.1|	tan	135.13226	29.797894	0.8543763	0.06523198	5.148
gi|194041357|ref|XM_001926917.1|	green	564.16344	68.536363	0.8864343	0.04515075	21.758
gi|194041366|ref|XM_001924481.1|	black	859.27262	107.21585	0.9960678	0.00029583	11.298
gi|194041368|ref|XM_001924555.1|	turquoise	999.69878	215.3362	0.9448957	0.01539888	26.018
gi|194041393|ref|XM_001928536.1|	black	615.87284	93.580041	0.9672811	0.0070695	32.348
gi|194041450|ref|XM_001928163.1|	lightgreen	322.98207	32.817268	0.9137446	0.03001325	34.026
gi|194041518|ref|XM_001927414.1|	turquoise	466.04304	50.095564	0.1437316	0.81762734	30.728
gi|194041588|ref|XM_001927189.1|	turquoise	456.19688	143.61577	0.8717504	0.05406071	7.296
gi|194041596|ref|XM_001927803.1|	navy	65.439929	31.269625	0.9036187	0.03539505	7.354
gi|194041636|ref|XM_001929009.1|	purple	145.27559	36.231925	0.8155124	0.0924455	39.698
gi|194041732|ref|XM_001927648.1|	blue	515.11223	72.855684	0.8411918	0.07413401	14.07
gi|194041758|ref|XM_001924615.1|	darkorange	144.8559	13.160146	0.685203	0.20170971	14.898
gi|194041760|ref|XM_001924661.1|	turquoise	622.67303	187.37635	0.9514585	0.01274431	1.484
gi|194041762|ref|XM_001927529.1|	darkgrey	998.94579	51.634212	0.9867422	0.00182883	6.774
gi|194041780|ref|XM_001925192.1|	turquoise	1001.3097	211.52785	0.9349154	0.01973628	37.77
gi|194041796|ref|XM_001928803.1|	red	667.02878	99.74286	0.9500391	0.01330448	9.73
gi|194041820|ref|XM_001929110.1|	blue	757.6057	147.4655	0.9928305	0.00072795	7.522
gi|194041839|ref|XM_001929344.1|	salmon	240.57708	74.110803	0.9880961	0.0015563	16.526
gi|194041857|ref|XM_001924202.1|	midnightblue	823.87545	75.834758	0.9914183	0.00095308	6.23
gi|194041859|ref|XM_001924277.1|	turquoise	250.33588	49.155099	-0.282008	0.64575436	4.346
gi|194041878|ref|XM_001924973.1|	yellow	241.47921	105.47716	0.9431859	0.01611692	66.414
gi|194041914|ref|XM_001928393.1|	turquoise	176.60253	52.801066	0.4507188	0.44619699	51.124
gi|194041916|ref|XM_001925662.1|	pink	990.27986	139.84058	0.9852218	0.00215179	2.844
gi|194041932|ref|XM_001928812.1|	lightgreen	206.79798	15.705744	0.7436621	0.14965998	32.948
gi|194041960|ref|XM_001929335.1|	grey60	290.68606	58.259911	0.9621353	0.00879435	46.704
gi|194041962|ref|XM_001926774.1|	tan	152.0101	24.688449	0.7857985	0.11510565	8.94
gi|194042016|ref|XM_001926579.1|	green	782.64405	114.01357	0.9948954	0.00043746	26.504
gi|194042046|ref|XM_001928978.1|	darkorange	83.000931	24.353908	0.8711312	0.05444743	16.914
gi|194042125|ref|XM_001928342.1|	skyblue	651.75909	38.349	0.9636883	0.00826083	126.964
gi|194042129|ref|XM_001927395.1|	blue	933.78672	143.40689	0.9837429	0.0024822	19.998
gi|194042141|ref|XM_001929000.1|	green	678.84117	104.70397	0.9766749	0.00426131	56.38
gi|194042170|ref|XM_001929360.1|	violet	217.02645	7.430984	0.1079829	0.86277961	22.518
gi|194042188|ref|XM_001924178.1|	violet	207.97686	7.0043162	0.0769593	0.90210919	48.256
gi|194042190|ref|XM_001924717.1|	blue	847.47004	140.21867	0.9799859	0.00338868	16.03
gi|194042230|ref|XM_001927832.1|	midnightblue	955.63161	77.371138	0.995344	0.0003811	14.928
gi|194042273|ref|XM_001927415.1|	green	814.28092	113.61561	0.9942085	0.00052862	14.278
gi|194042287|ref|XM_001927955.1|	midnightblue	839.04337	76.096461	0.9920042	0.00085724	64.744

gi|194042295|ref|XM_001928522.1|	turquoise	615.07178	166.62067	0.8778839	0.0502775	28.306
gi|194042303|ref|XM_001928772.1|	brown	768.15456	124.47032	0.9697871	0.00627543	57.818
gi|194042305|ref|XM_001928810.1|	magenta	443.70615	71.005266	0.9460184	0.01493323	28.378
gi|194042311|ref|XM_001925999.1|	blue	311.45859	60.671208	0.7940679	0.10864948	17.798
gi|194042329|ref|XM_001929115.1|	turquoise	355.23674	111.73704	0.8106368	0.0960594	0.976
gi|194042367|ref|XM_001926555.1|	turquoise	980.64099	222.10411	0.9615686	0.00899174	39.524
gi|194042373|ref|XM_001925458.1|	lightcyan	454.97497	52.163546	0.9624932	0.00867043	7.266
gi|194042394|ref|XM_001927941.1|	orange	167.6193	28.877551	0.9109056	0.03149334	1.374
gi|194042396|ref|XM_001927868.1|	green	530.89218	87.67806	0.9365261	0.01901286	4.08
gi|194042438|ref|XM_001927977.1|	turquoise	1013.4814	201.89665	0.915951	0.02887889	33.294
gi|194042457|ref|XM_001928703.1|	tan	78.898382	19.707179	0.6869812	0.20006257	14.77
gi|194042459|ref|XM_001928724.1|	turquoise	210.65513	47.377569	-0.253248	0.68103554	14.24
gi|194042469|ref|XM_001928931.1|	orange	191.86713	35.666807	0.9729914	0.00530665	0.618
gi|194042472|ref|XM_001924771.1|	midnightblue	936.32495	78.463245	0.998373	7.88E-05	18.616
gi|194042477|ref|XM_001926521.1|	lightyellow	321.66194	49.335179	0.9444038	0.01560436	39.796
gi|194042479|ref|XM_001926556.1|	turquoise	700.00607	173.84312	0.8849007	0.04605738	4.766
gi|194042490|ref|XM_001925381.1|	salmon	165.34166	54.51745	0.9252102	0.02427528	67.726
gi|194042528|ref|XM_001927272.1|	pink	961.7948	133.69074	0.9761579	0.00440344	645.53
gi|194042562|ref|XM_001925435.1|	black	637.42952	95.783054	0.9724987	0.00545209	6.358
gi|194042583|ref|XM_001928329.1|	yellow	272.20058	127.4598	0.9865489	0.00186894	46.458
gi|194042598|ref|XM_001924401.1|	magenta	572.40403	52.74897	0.8677041	0.05660331	23.704
gi|194042624|ref|XM_001927934.1|	turquoise	920.56387	197.96278	0.919789	0.02693948	5.788
gi|194042632|ref|XM_001928164.1|	blue	867.66056	151.26711	0.9972417	0.00017382	4.606
gi|194042649|ref|XM_001928781.1|	brown	660.34572	128.5946	0.9832316	0.00260002	13.024
gi|194042680|ref|XM_001929224.1|	white	760.9147	42.647932	0.9212484	0.02621349	6.994
gi|194042688|ref|XM_001929282.1|	green	555.88465	65.082929	0.87411	0.05259504	6.946
gi|194042690|ref|XM_001929322.1|	black	687.46484	100.4648	0.9828935	0.0026789	39.034
gi|194042702|ref|XM_001925079.1|	turquoise	896.73438	203.74986	0.9293353	0.02230898	3.126
gi|194042725|ref|XM_001925659.1|	blue	760.31966	125.60734	0.9534563	0.01196932	3.734
gi|194042749|ref|XM_001925080.1|	turquoise	367.36857	119.16723	0.8325653	0.08014611	126.154
gi|194042783|ref|XM_001924319.1|	lightgreen	406.51049	35.944733	0.8815855	0.04803666	3.362
gi|194042787|ref|XM_001924736.1|	navy	106.85981	5.839109	-0.745705	0.14792384	56.614
gi|194042789|ref|XM_001925275.1|	tan	168.62869	36.592253	0.9030709	0.0356942	58.51
gi|194042801|ref|XM_001927517.1|	turquoise	634.86428	173.26646	0.9028624	0.03580832	31.544
gi|194042805|ref|XM_001927802.1|	yellow	227.06857	105.97742	0.9426674	0.01633675	184.598
gi|194042813|ref|XM_001928193.1|	black	898.77052	101.19092	0.9831472	0.00261964	10.908
gi|194042821|ref|XM_001928374.1|	black	891.22625	104.10319	0.9894266	0.00130306	15.546
gi|194042829|ref|XM_001928949.1|	navy	86.799857	24.692129	0.8439497	0.07224278	5.852
gi|194042833|ref|XM_001929033.1|	navy	64.727504	19.618124	0.7927038	0.10970674	38.874
gi|194042835|ref|XM_001929055.1|	grey60	255.91538	52.198207	0.9396938	0.01761605	19.124
gi|194042841|ref|XM_001929155.1|	red	609.36606	95.763642	0.9409012	0.01709281	8.17
gi|194042857|ref|XM_001927712.1|	black	723.77673	100.42982	0.9831904	0.00260958	24.2
gi|194042859|ref|XM_001927602.1|	turquoise	764.77135	183.9304	0.9187286	0.02747098	20.21
gi|194042865|ref|XM_001925507.1|	turquoise	483.52769	150.99041	0.8544456	0.06518608	9.664
gi|194042903|ref|XM_001928981.1|	violet	45.119173	7.9466588	-0.864785	0.05846039	32.764
gi|194042939|ref|XM_001929407.1|	midnightblue	969.78496	73.815943	0.9852176	0.00215271	7.302
gi|194042943|ref|XM_001929420.1|	turquoise	464.9057	139.74007	0.8185902	0.09018605	20.542
gi|194042960|ref|XM_001929554.1|	turquoise	830.99489	211.19376	0.966658	0.00727171	9.804

gi|194042969|ref|XM_001929585.1|	navy	98.403407	29.734596	0.9255721	0.02410064	193.812
gi|194043008|ref|XM_001928156.1|	darkgrey	930.24481	52.375127	0.9904933	0.00111112	5.016
gi|194043036|ref|XM_001924558.1|	turquoise	335.79982	63.002716	0.5487884	0.33811915	4.598
gi|194043042|ref|XM_001926896.1|	blue	933.6651	136.46874	0.9718427	0.00564777	15.704
gi|194043052|ref|XM_001927455.1|	turquoise	148.90707	57.341908	0.5735408	0.31203479	18.556
gi|194043063|ref|XM_001925272.1|	brown	665.2763	117.82633	0.9612946	0.00908769	18.848
gi|194043066|ref|XM_001928232.1|	violet	962.41645	45.661629	0.9740455	0.00499984	43.462
gi|194043070|ref|XM_001928504.1|	grey60	315.42234	68.360951	0.9965234	0.00024595	12.764
gi|194043072|ref|XM_001925326.1|	green	777.98162	113.00633	0.9934924	0.00062956	4.972
gi|194043074|ref|XM_001928487.1|	green	951.60027	103.56812	0.9680557	0.00682075	16.196
gi|194043076|ref|XM_001925356.1|	midnightblue	900.65753	69.810649	0.9737021	0.00509911	26.668
gi|194043112|ref|XM_001929270.1|	turquoise	610.86532	180.04847	0.9336943	0.02029052	52.218
gi|194043122|ref|XM_001929345.1|	blue	983.28912	132.62897	0.9643577	0.00803428	8.904
gi|194043175|ref|XM_001928565.1|	turquoise	282.19005	103.4893	0.7651475	0.13170507	7.024
gi|194043187|ref|XM_001925978.1|	blue	778.38699	144.58624	0.9877422	0.00162612	19.756
gi|194043225|ref|XM_001929262.1|	turquoise	821.0155	208.48957	0.9544693	0.01158248	7.074
gi|194043233|ref|XM_001929306.1|	turquoise	765.61443	181.91791	0.9023138	0.03610899	11.532
gi|194043243|ref|XM_001924074.1|	salmon	240.03255	72.202984	0.9818908	0.00291742	68.692
gi|194043277|ref|XM_001927490.1|	turquoise	745.36549	189.68249	0.915638	0.02903892	7.862
gi|194043291|ref|XM_001928076.1|	white	776.02337	39.023284	0.9488966	0.01376103	43.526
gi|194043329|ref|XM_001929273.1|	turquoise	110.67801	36.836705	0.4647255	0.43034122	39.418
gi|194043337|ref|XM_001926296.1|	red	873.43045	126.71235	0.9979419	0.00011205	14.72
gi|194043361|ref|XM_001929445.1|	pink	974.37121	142.46464	0.989176	0.00134961	4.718
gi|194043363|ref|XM_001929468.1|	lightcyan	461.42552	58.094293	0.9880057	0.00157403	162.84
gi|194043378|ref|XM_001929539.1|	blue	839.48609	142.23075	0.9830169	0.00265003	5.072
gi|194043405|ref|XM_001929590.1|	grey60	303.10148	65.530454	0.9875904	0.00165638	16.642
gi|194043439|ref|XM_001927481.1|	darkgrey	1015.3843	49.910834	0.9792576	0.00357492	8.568
gi|194043453|ref|XM_001927547.1|	brown	672.56331	128.81169	0.9823542	0.00280635	3.338
gi|194043490|ref|XM_001927785.1|	white	901.36265	46.021506	0.9710691	0.00588143	12.622
gi|194043531|ref|XM_001926134.1|	midnightblue	775.52123	71.594942	0.9791403	0.00360523	4.45
gi|194043583|ref|XM_001928616.1|	yellow	282.90392	113.47361	0.9564608	0.01083422	5.662
gi|194043591|ref|XM_001927268.1|	brown	664.80346	127.96415	0.9849968	0.00220105	17.698
gi|194043669|ref|XM_001924741.1|	brown	763.00413	130.12626	0.98212	0.00286231	23.884
gi|194043695|ref|XM_001925613.1|	blue	721.59421	145.14838	0.9890988	0.00136407	1.824
gi|194043731|ref|XM_001926974.1|	greenyellow	59.378975	12.868511	0.3536842	0.55924851	12.594
gi|194043848|ref|XM_001928131.1|	red	819.82323	120.92182	0.9884509	0.00148731	10.556
gi|194043858|ref|XM_001928335.1|	turquoise	304.25965	110.38724	0.7955778	0.10748285	76.054
gi|194043900|ref|XM_001924998.1|	turquoise	1015.4742	211.12644	0.9349593	0.01971645	39.902
gi|194043908|ref|XM_001927890.1|	turquoise	974.0854	225.79752	0.9714591	0.00576326	9.248
gi|194043935|ref|XM_001924322.1|	green	446.66972	66.922288	0.8791116	0.0495307	1.484
gi|194043951|ref|XM_001927705.1|	darkgreen	181.40545	32.678214	0.9170676	0.02831019	13.102
gi|194043957|ref|XM_001925803.1|	skyblue	360.95422	37.847906	0.9652311	0.0077418	3064.368
gi|194043959|ref|XM_001927448.1|	white	697.08564	39.73389	0.9019631	0.03630163	6.996
gi|194043976|ref|XM_001928406.1|	brown	793.08424	120.81544	0.9617727	0.0089205	4.504
gi|194044012|ref|XM_001927891.1|	turquoise	609.80416	176.86295	0.904452	0.03494152	17.09
gi|194044032|ref|XM_001928774.1|	brown	788.83163	118.89265	0.965297	0.0077199	20.81
gi|194044034|ref|XM_001928794.1|	brown	766.13486	119.1398	0.9585327	0.01007333	118.444
gi|194044044|ref|XM_001927332.1|	tan	145.22068	44.04279	0.9697189	0.00629664	4.438

gi|194044110|ref|XM_001924775.1|	darkred	184.67848	44.289216	0.9829432	0.00266726	24.15
gi|194044123|ref|XM_001926788.1|	black	684.2301	91.981529	0.9652811	0.00772517	20.748
gi|194044132|ref|XM_001927798.1|	greenyellow	100.37967	20.225648	0.8021294	0.10246513	15.31
gi|194044163|ref|XM_001924534.1|	turquoise	142.77867	44.66972	0.5124511	0.37733052	32.086
gi|194044169|ref|XM_001924460.1|	midnightblue	802.86189	63.303291	0.9535368	0.01193842	37.84
gi|194044196|ref|XM_001928527.1|	turquoise	169.38473	51.077948	0.43645	0.46247911	16.842
gi|194044237|ref|XM_001927396.1|	darkgrey	753.72895	47.573637	0.9709691	0.00591187	15.104
gi|194044271|ref|XM_001926068.1|	pink	688.92996	121.39168	0.9577433	0.0103611	6.476
gi|194044273|ref|XM_001926112.1|	violet	960.90105	45.259512	0.9724035	0.00548036	8.988
gi|194044289|ref|XM_001926487.1|	darkmagenta	174.98044	8.0638952	0.8543071	0.06527778	56.314
gi|194044331|ref|XM_001925019.1|	purple	255.03097	69.921469	0.9692718	0.00643616	16.212
gi|194044358|ref|XM_001928282.1|	turquoise	103.17512	27.165352	0.3977236	0.5072895	9.698
gi|194044362|ref|XM_001926993.1|	lightcyan	700.04135	44.061534	0.8938497	0.04084857	21.558
gi|194044424|ref|XM_001927947.1|	pink	894.62975	149.62591	0.9996875	6.63E-06	0.45
gi|194044465|ref|XM_001928874.1|	magenta	422.0434	77.656906	0.9682882	0.00674666	39.924
gi|194044483|ref|XM_001929150.1|	darkorange	102.44799	10.888755	0.6201265	0.26445313	160.252
gi|194044504|ref|XM_001927363.1|	greenyellow	52.740469	11.922616	0.3900562	0.51626261	53.778
gi|194044550|ref|XM_001927682.1|	brown	139.07506	16.504478	0.5320572	0.35604277	108.86
gi|194044572|ref|XM_001925488.1|	turquoise	307.72986	111.20831	0.7266049	0.16438225	7.666
gi|194044576|ref|XM_001925640.1|	tan	249.1473	41.045529	0.9419039	0.01666221	16.362
gi|194044593|ref|XM_001929063.1|	white	903.95455	42.664716	0.9733234	0.00520937	35.416
gi|194044597|ref|XM_001926862.1|	brown	637.58798	114.86296	0.9546814	0.01150203	11.608
gi|194044682|ref|XM_001928813.1|	turquoise	191.79321	76.025513	0.5896417	0.29535864	10.456
gi|194044686|ref|XM_001928889.1|	pink	972.64037	143.59552	0.9908295	0.00105275	5.378
gi|194044696|ref|XM_001926221.1|	paleturquoise	178.21086	26.39031	0.9757171	0.0045258	16.596
gi|194044738|ref|XM_001929453.1|	pink	894.62975	149.62591	0.9996875	6.63E-06	0.128
gi|194044782|ref|XM_001926654.1|	darkred	163.90271	40.136447	0.9603293	0.00942838	43.064
gi|194044788|ref|XM_001928382.1|	brown	562.20956	119.36572	0.9748401	0.00477257	2.88
gi|194044819|ref|XM_001926406.1|	blue	944.16717	139.72657	0.9767757	0.0042338	21.8
gi|194044859|ref|XM_001927209.1|	purple	233.11406	45.067747	0.8684131	0.05615514	21.206
gi|194044861|ref|XM_001927107.1|	white	960.14731	45.37614	0.9901273	0.00117584	4.446
gi|194044921|ref|XM_001927440.1|	navy	1014.667	17.465744	0.0793997	0.89901151	10.232
gi|194044929|ref|XM_001925991.1|	blue	913.58066	150.60397	0.9956277	0.00034683	2.094
gi|194044943|ref|XM_001926010.1|	violet	894.62975	47.034779	0.9933959	0.00064361	1.668
gi|194044951|ref|XM_001925471.1|	turquoise	293.92977	108.7085	0.7698354	0.12787875	3.992
gi|194045002|ref|XM_001926288.1|	darkgreen	158.06636	29.787986	0.8832479	0.04704085	206.036
gi|194045026|ref|XM_001925965.1|	darkorange	217.25696	23.843359	0.8994709	0.03768007	20.438
gi|194045094|ref|XM_001925372.1|	lightgreen	215.32052	25.998059	0.8359156	0.077794	9.874
gi|194045111|ref|XM_001927898.1|	turquoise	869.61445	198.64123	0.9304446	0.02178944	19.252
gi|194271212|gb|EU714326.1|	black	719.61622	104.55526	0.9911628	0.00099594	3.06
gi|194332486|ref|NM_001130213.1|	navy	100.39154	36.498469	0.923664	0.02502603	20.552
gi|194474047|ref|NM_001130535.1|	orange	283.47359	37.28858	0.9967652	0.00022075	51.062
gi|194474049|ref|NM_001130531.1|	blue	866.26294	148.06159	0.99194	0.00086759	1.246
gi|195539469|ref|NM_001130733.1|	skyblue	563.3461	43.154678	0.9917727	0.00089472	74.936
gi|195539471|ref|NM_001130734.1|	lightgreen	405.54705	38.152809	0.914042	0.02985951	0.668
gi|196259975|ref|NM_001131045.1|	turquoise	559.85873	174.39653	0.9340572	0.02012529	17.784
gi|197717772|gb|EF113595.2|	green	962.34348	103.13851	0.9674171	0.00702561	28.898
gi|198282078|ref|NM_001134823.1|	brown	784.77117	131.75287	0.9861704	0.00194825	224.672

gi|19919837|gb|AF490841.1|	black	840.33362	105.60694	0.9929465	0.00071036	19.942
gi|201066357|ref|NM_001134968.1|	violet	43.447027	9.9570167	-0.988069	0.00156169	2362.302
gi|209863050|ref|NM_001135960.1|	yellow	236.80635	110.01988	0.9515798	0.01269679	213.644
gi|209954779|ref|NM_214223.2|	blue	582.23761	111.06239	0.9297789	0.02210075	5.566
gi|211578395|ref|NM_001136510.1|	black	858.19332	91.600703	0.9623939	0.00870474	28.856
gi|212549664|ref|NM_001137639.1|	tan	80.84378	13.861913	0.6371204	0.24762618	51.934
gi|213021240|ref|NM_001139472.1|	lightcyan	616.81354	52.53356	0.9464867	0.0147404	5.098
gi|217416473|ref|NM_001142668.1|	blue	848.52359	148.00783	0.9930129	0.00070036	7.682
gi|217416475|ref|NM_001142669.1|	skyblue	497.60461	44.332118	0.9986881	5.70E-05	6.856
gi|218082010|ref|NM_001142666.1|	blue	532.34919	99.90484	0.9057887	0.03421796	19.924
gi|2286009|emb|AJ000786.1|	turquoise	558.2772	139.4046	0.8143567	0.09329824	23.566
gi|2286014|emb|AJ000791.1|	white	173.57387	13.306732	-0.720632	0.16963135	108.042
gi|29150628|gb|AY216477.1|	tan	198.31645	45.939559	0.9746428	0.00482866	37.546
gi|33772282|gb|AY346132.1|AY3461	violet	979.84316	43.916322	0.9611494	0.00913867	9.35
gi|34582610|gb|AY368623.1|	green	969.31982	107.11034	0.977184	0.00412288	24.29
gi|35384837|gb|AY374470.1|	turquoise	265.51305	89.49111	0.6366006	0.24813643	45.822
gi|37700454|gb|AY421755.1|	blue	542.93625	124.14541	0.9543817	0.01161579	3.106
gi|4186150|emb|AJ236938.1|	turquoise	175.64975	67.920966	0.6063106	0.27834819	33.394
gi|45268992|gb|AY550051.1|	lightgreen	211.13381	23.050251	0.7985414	0.10520416	24.488
gi|45758483|gb|AY553927.1|	turquoise	610.01067	184.01669	0.9447672	0.01545248	6.948
gi|47522609|ref|NM_213938.1|	magenta	215.53149	79.052233	0.978532	0.00376373	24.836
gi|47522617|ref|NM_213934.1|	brown	741.96288	133.74502	0.990238	0.00115613	13.422
gi|47522629|ref|NM_213928.1|	black	901.26972	103.22887	0.9880667	0.00156205	49.376
gi|47522635|ref|NM_213927.1|	black	756.90437	101.19015	0.9846125	0.00228603	65.686
gi|47522641|ref|NM_213922.1|	turquoise	259.09193	79.60848	0.6927601	0.19473593	278.802
gi|47522643|ref|NM_213923.1|	turquoise	190.56214	65.87216	0.5016763	0.38915482	15.472
gi|47522659|ref|NM_213914.1|	violet	942.14695	46.386162	0.9821696	0.00285044	8.396
gi|47522661|ref|NM_213911.1|	turquoise	1018.6875	210.82358	0.9330846	0.0205691	30.758
gi|47522667|ref|NM_213910.1|	darkturquoise	184.31375	45.86773	0.9916758	0.00091056	0.116
gi|47522679|ref|NM_213904.1|	blue	921.1179	142.10568	0.9811886	0.00308842	15.414
gi|47522681|ref|NM_213901.1|	black	722.27179	104.07004	0.9902073	0.00116158	3.39
gi|47522685|ref|NM_213898.1|	turquoise	193.42182	72.366114	0.5521858	0.33450788	88.894
gi|47522689|ref|NM_213896.1|	turquoise	741.94346	183.27298	0.9177485	0.02796515	24.55
gi|47522691|ref|NM_213897.1|	turquoise	895.87726	196.98502	0.9227548	0.02547087	85.094
gi|47522703|ref|NM_213891.1|	lightyellow	458.31771	63.71928	0.9974506	0.00015446	14.29
gi|47522711|ref|NM_213943.1|	lightgreen	556.71278	36.954472	0.9179213	0.02787784	8.152
gi|47522717|ref|NM_213944.1|	green	834.61249	115.49361	0.9967846	0.00021877	20.498
gi|47522719|ref|NM_213945.1|	lightyellow	324.74072	56.907993	0.9722083	0.00553846	6.286
gi|47522737|ref|NM_213954.1|	yellow	287.71007	117.34384	0.9661605	0.00743454	372.544
gi|47522751|ref|NM_213961.1|	magenta	224.53463	78.552066	0.9778707	0.00393856	5.392
gi|47522753|ref|NM_213962.1|	blue	959.05885	146.03792	0.9874243	0.0016897	121.492
gi|47522763|ref|NM_213968.1|	brown	894.62337	110.3462	0.9419068	0.01666098	30.566
gi|47522769|ref|NM_213971.1|	brown	363.67162	73.810497	0.8633191	0.05939979	16.834
gi|47522773|ref|NM_213973.1|	royalblue	515.91429	59.230316	0.9814522	0.00302384	7.17
gi|47522777|ref|NM_213976.1|	black	729.95092	100.994	0.9843458	0.00234563	6.192
gi|47522779|ref|NM_213977.1|	navy	130.1696	41.787747	0.9740144	0.00500878	40.73
gi|47522785|ref|NM_213980.1|	black	769.21146	102.22761	0.986685	0.00184066	63.336
gi|47522789|ref|NM_213982.1|	grey	419.02228	15.443909	0.9935362	0.00062323	11.314

gi|47522791|ref|NM_213983.1|	yellow	251.19828	91.480864	0.90838	0.03282911	234.298
gi|47522821|ref|NM_213998.1|	lightyellow	457.23571	55.435183	0.9688321	0.00657437	7.882
gi|47522837|ref|NM_214006.1|	magenta	168.18365	62.920855	0.9286517	0.02263111	16.626
gi|47522843|ref|NM_214009.1|	tan	70.523875	16.125917	0.6127319	0.27186661	7.546
gi|47522861|ref|NM_214020.1|	darkturquoise	209.6074	40.124234	0.9619381	0.00886285	109.848
gi|47522869|ref|NM_214024.1|	green	913.31397	112.10442	0.9878261	0.00160948	29.146
gi|47522881|ref|NM_214031.1|	blue	905.84967	151.10604	0.9962677	0.00027356	9.49
gi|47522897|ref|NM_214039.1|	salmon	226.63455	64.970163	0.9585084	0.01008214	85.154
gi|47522913|ref|NM_214049.1|	lightgreen	375.69483	39.283857	0.9487295	0.01382825	15.214
gi|47522915|ref|NM_214050.1|	paleturquoise	178.05471	23.707384	0.939339	0.01777077	9.304
gi|47522935|ref|NM_214060.1|	darkturquoise	101.05968	20.101597	0.8103603	0.09626556	89.144
gi|47522937|ref|NM_214061.1|	lightcyan	167.17754	28.277902	0.7974982	0.10600461	12.758
gi|47522939|ref|NM_214062.1|	blue	958.25273	136.63086	0.9723545	0.00549492	70.504
gi|47522941|ref|NM_214063.1|	turquoise	986.19462	216.60879	0.9460579	0.01491696	5.756
gi|47522943|ref|NM_214064.1|	lightcyan	419.42998	50.145848	0.9499778	0.01332883	18.434
gi|47522957|ref|NM_214072.1|	brown	609.49756	122.92278	0.9819528	0.00290248	19.31
gi|47522967|ref|NM_214077.1|	magenta	289.53998	79.836513	0.9822043	0.00284212	4.57
gi|47523015|ref|NM_214103.1|	turquoise	696.61287	192.73204	0.9366428	0.01896082	48.372
gi|47523035|ref|NM_214114.1|	greenyellow	82.10059	15.494472	0.714547	0.17502701	69.22
gi|47523037|ref|NM_214115.1|	red	962.08587	123.05783	0.9911939	0.00099068	45.688
gi|47523039|ref|NM_214116.1|	turquoise	850.5423	202.40047	0.9391922	0.01783493	192.26
gi|47523059|ref|NM_214128.1|	lightgreen	234.94378	29.153764	0.8350791	0.07837921	2.152
gi|47523085|ref|NM_214144.1|	skyblue	333.50822	35.804342	0.9515675	0.01270162	22.21
gi|47523093|ref|NM_213884.1|	royalblue	398.42723	50.419142	0.9446654	0.01549497	0.988
gi|47523105|ref|NM_213878.1|	turquoise	157.07427	46.757231	0.5019812	0.38881908	44.136
gi|47523187|ref|NM_213830.1|	brown	754.35549	135.45958	0.9947564	0.00045544	2.438
gi|47523195|ref|NM_213826.1|	darkred	115.56868	23.887299	0.8039176	0.10110829	10.45
gi|47523213|ref|NM_213817.1|	navy	66.912458	7.818377	-0.562513	0.32359073	13.896
gi|47523231|ref|NM_213804.1|	turquoise	208.14973	48.505099	-0.294761	0.63020674	6.728
gi|47523255|ref|NM_213792.1|	violet	891.49673	47.021398	0.9945255	0.00048583	7.614
gi|47523261|ref|NM_213791.1|	pink	990.08316	141.39646	0.98755	0.00166446	4.99
gi|47523273|ref|NM_213785.1|	lightyellow	574.95719	52.077404	0.9549903	0.01138514	1.184
gi|47523281|ref|NM_213781.1|	purple	299.32478	79.079859	0.9958604	0.00031952	11.118
gi|47523317|ref|NM_213763.1|	royalblue	764.31286	59.569305	0.9815983	0.00298827	5.3
gi|47523403|ref|NM_214157.1|	yellow	268.01585	125.66417	0.983462	0.0025467	10.046
gi|47523419|ref|NM_214166.1|	turquoise	313.04042	99.778657	0.7157629	0.17394496	18.3
gi|47523427|ref|NM_214171.1|	lightcyan	251.47554	42.89359	0.9134352	0.03017343	10.738
gi|47523443|ref|NM_214181.1|	turquoise	282.19523	56.561508	-0.133892	0.83003371	8.858
gi|47523445|ref|NM_214183.1|	midnightblue	658.23467	67.49596	0.9670587	0.00714147	9.8
gi|47523457|ref|NM_214189.1|	pink	900.21735	147.50177	0.9967891	0.0002183	2.112
gi|47523461|ref|NM_214192.1|	greenyellow	200.27602	40.602595	0.9691874	0.00646263	55.736
gi|47523463|ref|NM_214188.1|	green	521.69567	71.371939	0.8961479	0.03954332	10.1
gi|47523471|ref|NM_214193.1|	magenta	264.80187	72.762606	0.962448	0.00868605	19.79
gi|47523477|ref|NM_214200.1|	blue	946.6493	147.2993	0.9896167	0.00126812	5.728
gi|47523483|ref|NM_214199.1|	turquoise	352.23658	50.525865	-0.088069	0.88801202	96.192
gi|47523487|ref|NM_214201.1|	navy	32.863867	12.013891	0.7320264	0.15965949	49.772
gi|47523491|ref|NM_214204.1|	grey60	258.17084	53.43162	0.9435446	0.0159654	385.558
gi|47523501|ref|NM_214213.1|	midnightblue	862.39933	71.341988	0.9782888	0.00382773	23.266

gi|47523543|ref|NM_214230.1|	blue	570.44841	119.12538	0.9430462	0.01617605	1.164
gi|47523545|ref|NM_214236.1|	lightcyan	276.73912	45.690694	0.9324747	0.02084899	136.106
gi|47523553|ref|NM_214240.1|	midnightblue	712.60207	66.899009	0.9655219	0.00764521	25.354
gi|47523561|ref|NM_214245.1|	green	686.56505	94.752599	0.9547065	0.01149252	12.126
gi|47523569|ref|NM_214249.1|	yellow	176.64747	82.234335	0.8844176	0.04634413	18.002
gi|47523631|ref|NM_214279.1|	greenyellow	84.987984	4.3001427	0.2516709	0.68297826	29.732
gi|47523665|ref|NM_214304.1|	greenyellow	95.757856	16.398276	0.6032304	0.28147152	20.192
gi|47523669|ref|NM_214306.1|	turquoise	976.57451	214.57486	0.9426307	0.01635233	36.024
gi|47523671|ref|NM_214303.1|	navy	41.00995	18.200364	0.7473944	0.14649289	24.248
gi|47523673|ref|NM_214308.1|	greenyellow	203.94133	38.823923	0.9508422	0.01298657	27.916
gi|47523679|ref|NM_214307.1|	turquoise	643.06501	163.96823	0.8886624	0.04384374	168.29
gi|47523681|ref|NM_214312.1|	orange	378.40452	33.486818	0.9706085	0.00602203	9.86
gi|47523691|ref|NM_214313.1|	turquoise	423.16787	145.16713	0.8693902	0.05553935	11.838
gi|47523695|ref|NM_214315.1|	lightgreen	477.33399	37.851269	0.9118829	0.03098124	5.042
gi|47523697|ref|NM_214320.1|	turquoise	319.56785	118.60193	0.8191238	0.08979607	5.196
gi|47523711|ref|NM_214323.1|	blue	639.73962	125.73229	0.9549687	0.01139331	4.032
gi|47523719|ref|NM_214330.1|	violet	39.213186	5.364492	-0.769818	0.12789284	644.734
gi|47523721|ref|NM_214331.1|	royalblue	834.76267	51.057133	0.9457944	0.01502578	65.558
gi|47523723|ref|NM_214332.1|	salmon	226.17127	71.677982	0.9808628	0.00316884	355.768
gi|47523725|ref|NM_214333.1|	black	831.63531	108.68902	0.9992147	2.64E-05	33.152
gi|47523737|ref|NM_214339.1|	pink	865.35452	141.99287	0.9891634	0.00135197	31.594
gi|47523743|ref|NM_214342.1|	yellow	310.61843	130.03801	0.9906292	0.0010874	26.838
gi|47523769|ref|NM_214356.1|	darkorange	85.78232	26.056055	0.8925698	0.04158135	27.37
gi|47523779|ref|NM_214361.1|	green	790.90509	111.91421	0.9905577	0.00109985	105.712
gi|47523785|ref|NM_214364.1|	black	768.02918	107.94899	0.997978	0.00010911	28.87
gi|47523789|ref|NM_214366.1|	turquoise	405.75022	122.40089	0.7730515	0.12527318	75.766
gi|47523799|ref|NM_214372.1|	black	761.01555	102.10365	0.9864363	0.00189241	34.022
gi|47523805|ref|NM_214375.1|	blue	938.48582	144.81837	0.9862952	0.00192197	527.014
gi|47523817|ref|NM_214381.1|	yellow	139.40698	56.56788	0.800619	0.10361546	52.706
gi|47523835|ref|NM_214391.1|	purple	240.29538	63.899513	0.9479217	0.01415458	24.604
gi|47523845|ref|NM_214396.1|	green	253.50947	40.923908	0.775529	0.123277	701.55
gi|47523847|ref|NM_214397.1|	blue	947.16108	140.07448	0.9784772	0.00377811	40.92
gi|47523867|ref|NM_214407.1|	darkgrey	673.18735	42.210115	0.9464513	0.01475496	85.436
gi|47523869|ref|NM_214408.1|	navy	109.02879	35.371553	0.9534456	0.01197342	74.202
gi|47523875|ref|NM_214409.1|	lightcyan	312.93312	38.970663	0.8984567	0.0382457	364.676
gi|47523887|ref|NM_214418.1|	blue	600.04587	130.62949	0.9661204	0.0074477	54.658
gi|47523931|ref|NM_214440.1|	lightgreen	410.82435	38.051768	0.9117831	0.03103344	3.146
gi|48374062|ref|NM_001001535.1|	tan	118.09508	24.474901	0.7975838	0.10593884	4157.498
gi|48374066|ref|NM_001001537.1|	lightyellow	517.60664	55.466353	0.9687547	0.0065988	4.024
gi|48374070|ref|NM_001001539.1|	turquoise	452.05397	60.216545	0.1580275	0.7996337	35.688
gi|48675942|ref|NM_001001640.1|	turquoise	146.2046	48.289231	0.467989	0.42666548	7.22
gi|48675944|ref|NM_001001637.1|	royalblue	695.86987	63.71155	0.9972637	0.00017175	27.854
gi|48675952|ref|NM_001001643.1|	brown	748.01322	122.57676	0.9657219	0.00757904	89.962
gi|48675956|ref|NM_001001645.1|	darkred	195.64174	41.217292	0.9524548	0.01235582	70.016
gi|48976064|ref|NM_001001649.2|	turquoise	158.89505	37.587784	-0.287386	0.63919	65.286
gi|48976128|ref|NM_001001770.1|	royalblue	633.03006	58.605744	0.9801812	0.0033393	56.194
gi|49274614|ref|NM_001001860.1|	red	887.98496	126.39506	0.9972735	0.00017083	38.73
gi|49274638|ref|NM_001001861.1|	darkturquoise	186.3246	46.670554	0.9960825	0.00029417	5.282

gi|50979302|ref|NM_214301.1|	lightcyan	397.996	57.365728	0.9890699	0.00136948	93.422
gi|51491905|ref|NM_001003924.1|	turquoise	257.54402	55.27605	-0.182574	0.76883799	10.4
gi|51592110|ref|NM_001004031.1|	turquoise	761.23755	194.8211	0.9364176	0.01906135	14.142
gi|51592138|ref|NM_001004045.1|	green	893.78475	115.8979	0.9977417	0.00012878	5.894
gi|52350674|gb|AY609390.1|	orange	301.62196	36.302888	0.9914796	0.0009429	11.004
gi|52350683|gb|AY609399.1|	turquoise	901.18544	212.91756	0.9568902	0.01067503	18.658
gi|52350685|gb|AY609401.1|	turquoise	264.70895	97.510539	0.668595	0.21727415	2.028
gi|52350686|gb|AY609402.1|	orange	76.528363	13.854706	0.7124091	0.17693434	20.884
gi|52350700|gb|AY609415.1|	brown	148.31214	15.032859	0.4328214	0.46664007	12.49
gi|52350704|gb|AY609419.1|	turquoise	402.18263	61.586662	0.0584013	0.92568349	14.132
gi|52350710|gb|AY609425.1|	blue	472.45846	60.58929	0.8089304	0.09733415	32.048
gi|52350723|gb|AY609438.1|	darkgreen	140.69343	19.816713	0.7995237	0.10445208	52.646
gi|52350726|gb|AY609441.1|	greenyellow	199.84395	39.024437	0.9886919	0.00144106	30.97
gi|52350728|gb|AY609443.1|	greenyellow	183.69752	37.834424	0.9785528	0.00375826	29.572
gi|52350742|gb|AY609457.1|	navy	112.63095	33.79654	0.9059051	0.03415514	138.978
gi|52351065|gb|AY609470.1|	turquoise	792.32141	197.23587	0.9483861	0.01396666	17.778
gi|52351066|gb|AY609471.1|	lightcyan	496.65111	57.16011	0.9786281	0.00373854	4.74
gi|52351067|gb|AY609472.1|	midnightblue	900.22358	67.491171	0.9664649	0.00733477	13.428
gi|52351069|gb|AY609474.1|	midnightblue	802.72916	69.43348	0.9734821	0.00516308	8.818
gi|52351072|gb|AY609477.1|	royalblue	653.65144	58.14231	0.9785032	0.00377128	48.514
gi|52351075|gb|AY609480.1|	darkred	143.71837	34.799889	0.915637	0.02903945	7.784
gi|52351083|gb|AY609488.1|	violet	179.3741	8.2619225	0.2976799	0.62665674	37.894
gi|52351086|gb|AY609491.1|	violet	206.76414	7.5344979	0.1592572	0.79808783	45.258
gi|52351090|gb|AY609495.1|	orange	169.24546	29.107852	0.9402984	0.01735342	157.554
gi|52351095|gb|AY609500.1|	midnightblue	865.17774	77.762007	0.9967265	0.00022472	18.722
gi|52351120|gb|AY609525.1|	blue	997.06725	123.7863	0.9476976	0.01424554	20.698
gi|52351125|gb|AY609530.1|	black	654.01013	93.953272	0.9686266	0.0066393	8.09
gi|52351148|gb|AY609553.1|	tan	81.259891	15.789798	0.6831513	0.20361496	68.642
gi|52351153|gb|AY609558.1|	turquoise	553.09126	165.7487	0.889918	0.04311255	10.114
gi|52351155|gb|AY609560.1|	grey60	300.86325	65.885178	0.9893507	0.00131711	45.722
gi|52351156|gb|AY609561.1|	red	878.09122	126.14479	0.9969996	0.0001972	14.376
gi|52351160|gb|AY609565.1|	blue	760.39243	148.25967	0.9936679	0.00060428	7.6
gi|52351167|gb|AY609572.1|	white	910.69065	47.541908	0.9786859	0.0037234	125.772
gi|52351176|gb|AY609581.1|	pink	978.17043	135.54147	0.9789688	0.00364968	3.664
gi|52351185|gb|AY609590.1|	salmon	231.51412	70.078468	0.9754341	0.00460495	11.61
gi|52351201|gb|AY609606.1|	turquoise	333.03163	57.017786	-0.075925	0.90342186	54.084
gi|52351209|gb|AY609612.1|	darkgrey	877.35865	52.802857	0.9927564	0.00073925	56.918
gi|52351212|gb|AY609615.1|	greenyellow	224.14363	40.291403	0.9797657	0.00344464	22.03
gi|52351257|gb|AY609689.1|	yellow	248.67806	99.341334	0.9278739	0.02299945	120.21
gi|52351277|gb|AY609709.1|	red	962.98044	120.37439	0.986786	0.00181979	39.988
gi|52351285|gb|AY609717.1|	salmon	224.50195	70.474277	0.9774503	0.00405107	3.866
gi|52351294|gb|AY609726.1|	purple	272.90593	77.032578	0.9922066	0.00082493	15.618
gi|52351296|gb|AY609728.1|	green	794.99946	87.277104	0.931363	0.02136234	36.134
gi|52351300|gb|AY609732.1|	purple	250.59322	66.786327	0.9567946	0.01071041	46.658
gi|52351303|gb|AY609735.1|	blue	905.50147	128.64369	0.9575722	0.01042381	4.898
gi|52351323|gb|AY609755.1|	royalblue	817.73131	57.041197	0.9713427	0.00579844	28.266
gi|52351333|gb|AY609765.1|	lightcyan	554.83445	55.087489	0.9659689	0.00749753	16.408
gi|52351341|gb|AY609773.1|	greenyellow	147.54086	17.720435	0.6558641	0.22941964	4.498

gi|52351346|gb|AY609778.1|	orange	128.86354	23.916021	0.8822292	0.04765028	44.014
gi|52351351|gb|AY609781.1|	greenyellow	142.52231	24.724382	0.766359	0.13071301	21.456
gi|52351368|gb|AY609798.1|	brown	530.84451	107.70945	0.9515693	0.01270091	10.22
gi|52351373|gb|AY609803.1|	blue	899.76878	146.72124	0.989194	0.00134625	53.04
gi|52351374|gb|AY609804.1|	darkred	186.12321	40.114718	0.9451362	0.01529877	10.224
gi|52351378|gb|AY609808.1|	pink	899.24766	146.39644	0.9952084	0.00039787	5.546
gi|52351391|gb|AY609821.1|	turquoise	868.80554	196.7122	0.9269497	0.0234396	6.978
gi|52351393|gb|AY609823.1|	navy	276.09236	23.491315	0.862543	0.05989918	9.27
gi|52351397|gb|AY609827.1|	turquoise	207.84859	77.390247	0.6935072	0.19405029	8.394
gi|52351406|gb|AY609836.1|	darkgreen	254.06684	43.487639	0.9973733	0.00016154	126.928
gi|52351416|gb|AY609846.1|	turquoise	863.54055	190.26343	0.9097038	0.03212676	25.244
gi|52351419|gb|AY609849.1|	greenyellow	45.592831	7.6382168	0.3065394	0.61590364	48.642
gi|52351430|gb|AY609860.1|	blue	882.44182	103.18282	0.9064951	0.0338375	14.472
gi|52351432|gb|AY609862.1|	navy	74.52256	9.3439201	-0.810659	0.09604269	41.984
gi|52351435|gb|AY609865.1|	turquoise	360.19923	119.44271	0.8073859	0.0984923	5.622
gi|52351436|gb|AY609866.1|	darkorange	116.79586	33.912466	0.9766286	0.00427397	39.636
gi|52351444|gb|AY609874.1|	brown	909.18237	107.83027	0.9372112	0.01870786	53.938
gi|52351447|gb|AY609877.1|	magenta	384.01805	76.213304	0.967253	0.00707859	35.67
gi|52351453|gb|AY609883.1|	salmon	253.18866	65.511068	0.9604605	0.00938183	15.078
gi|52351454|gb|AY609884.1|	lightgreen	641.74309	31.219329	0.8934789	0.04106043	28.722
gi|52351457|gb|AY609887.1|	blue	625.38733	79.03973	0.8534657	0.06583532	59.952
gi|52351462|gb|AY609892.1|	magenta	197.81725	64.958599	0.9361852	0.01916527	10.786
gi|52351474|gb|AY609904.1|	orange	319.50582	11.31583	0.7118336	0.17744877	99.664
gi|52351476|gb|AY609906.1|	greenyellow	217.83596	39.030101	0.9691805	0.0064648	31.694
gi|52351490|gb|AY609920.1|	turquoise	259.13542	86.072	0.7233838	0.16720691	18.352
gi|52351500|gb|AY609930.1|	darkturquoise	159.63181	39.675429	0.9541415	0.01170721	18.7
gi|52351521|gb|AY609951.1|	grey60	274.20339	60.097827	0.9699135	0.00623622	156.176
gi|52351524|gb|AY609954.1|	darkgreen	233.62172	42.213788	0.9886507	0.00144892	14.01
gi|52351527|gb|AY609957.1|	turquoise	260.12905	87.002372	0.6574643	0.2278831	27.04
gi|52351536|gb|AY609966.1|	darkgrey	930.67878	50.713732	0.9840149	0.00242027	23.156
gi|52351538|gb|AY609968.1|	pink	796.98709	143.27085	0.9909026	0.0010402	40.888
gi|52351540|gb|AY609970.1|	black	850.28788	108.1573	0.9981625	9.45E-05	4.302
gi|52351544|gb|AY609974.1|	turquoise	952.22528	201.29561	0.9231155	0.0252941	20.48
gi|52351547|gb|AY609977.1|	turquoise	536.20585	157.92578	0.8791103	0.04953148	28.904
gi|52351567|gb|AY609997.1|	grey60	233.9363	49.469639	0.9279725	0.02295266	22.476
gi|52351573|gb|AY610003.1|	turquoise	451.75321	150.22894	0.8723038	0.05371585	8.152
gi|52351582|gb|AY610012.1|	green	862.05545	117.02501	0.9998866	1.45E-06	9.526
gi|52351587|gb|AY610017.1|	pink	747.94482	138.02522	0.983187	0.00261039	108.904
gi|52351591|gb|AY610021.1|	violet	929.76947	45.942237	0.9789143	0.00366385	9.898
gi|52351593|gb|AY610023.1|	turquoise	977.55984	223.6631	0.966925	0.00718484	16.17
gi|52351594|gb|AY610024.1|	tan	107.06844	31.910103	0.8681171	0.05634209	86.354
gi|52351609|gb|AY610039.1|	tan	125.22725	30.189932	0.8575911	0.06311576	60.022
gi|52351611|gb|AY610041.1|	darkgrey	901.88333	52.77344	0.9925275	0.00077454	14.584
gi|52351614|gb|AY610044.1|	turquoise	831.2514	219.03897	0.9851611	0.00216504	12.016
gi|52351621|gb|AY610051.1|	royalblue	680.25157	62.903111	0.9944625	0.00049425	26.502
gi|52351639|gb|AY610069.1|	turquoise	555.73167	167.69548	0.9174269	0.02812794	69.738
gi|52351674|gb|AY610104.1|	blue	824.34869	142.08476	0.9827018	0.002724	5.286
gi|52351676|gb|AY610106.1|	pink	927.44147	148.08803	0.9974679	0.0001529	27.404

gi|52351677|gb|AY610107.1|	blue	985.84737	125.11504	0.9501895	0.01324475	39.346
gi|52351697|gb|AY610127.1|	purple	275.88109	67.212543	0.9581524	0.01021164	2090.66
gi|52351702|gb|AY610132.1|	midnightblue	897.80969	78.450167	0.9984586	7.26E-05	26.78
gi|52351709|gb|AY610139.1|	lightcyan	301.80879	42.823472	0.9232116	0.02524703	13.964
gi|52351717|gb|AY610147.1|	turquoise	414.1347	136.94585	0.8091785	0.09714843	10.008
gi|52351733|gb|AY610163.1|	turquoise	159.63162	63.494117	0.5652731	0.32068833	14.27
gi|52351736|gb|AY610166.1|	magenta	351.96051	83.820724	0.9904281	0.00112256	142.87
gi|52351738|gb|AY610168.1|	magenta	95.204829	40.175595	0.8106254	0.09606784	39.4
gi|52351742|gb|AY610172.1|	yellow	228.86608	103.84552	0.9387711	0.01801933	38.57
gi|52351745|gb|AY610175.1|	turquoise	371.51768	132.30159	0.8541723	0.06536695	5.952
gi|52351750|gb|AY610180.1|	midnightblue	911.68377	77.767379	0.9966514	0.00023249	24.578
gi|52351755|gb|AY610185.1|	turquoise	461.03914	151.60102	0.8979814	0.03851174	2.432
gi|52351761|gb|AY610191.1|	turquoise	980.27821	206.58412	0.9328735	0.02066584	26.742
gi|52351763|gb|AY610193.1|	turquoise	686.69929	192.55713	0.9467457	0.01463412	48.222
gi|52351796|gb|AY610214.1|	grey60	285.69103	63.414831	0.9810221	0.00312943	77.432
gi|52351803|gb|AY610221.1|	green	790.47579	114.12563	0.9948826	0.00043912	12.622
gi|52351805|gb|AY610223.1|	grey60	283.56672	56.985449	0.9572605	0.01053838	6.87
gi|52351807|gb|AY610225.1|	greenyellow	135.83754	24.182241	0.8763466	0.0512176	77.176
gi|52351871|gb|AY610289.1|	darkturquoise	199.60858	40.940447	0.961861	0.00888972	6.712
gi|52351873|gb|AY610291.1|	lightgreen	164.75646	21.595899	0.7836353	0.11681285	243.45
gi|52351877|gb|AY610295.1|	darkorange	124.19321	27.57462	0.9277766	0.02304566	155.248
gi|52351890|gb|AY610308.1|	red	577.04404	87.947555	0.9242922	0.0247201	26.934
gi|52351923|gb|AY610341.1|	grey60	313.8181	68.501609	0.997317	0.00016676	9.334
gi|52351935|gb|AY610353.1|	green	955.73873	111.51247	0.9878584	0.00160309	7.652
gi|52351947|gb|AY610365.1|	greenyellow	61.043672	9.9081258	0.4764041	0.41722087	25.522
gi|52351956|gb|AY610374.1|	purple	307.32382	72.751613	0.976143	0.00440754	130.65
gi|52351961|gb|AY610379.1|	brown	470.72814	97.547007	0.9303045	0.02185485	9.46
gi|52351978|gb|AY610394.1|	turquoise	357.19374	87.93174	0.6698378	0.21609828	140.36
gi|52351995|gb|AY610411.1|	lightgreen	287.98852	32.013466	0.887481	0.04453521	26.334
gi|52352038|gb|AY610454.1|	skyblue	403.47329	42.095053	0.9873889	0.00169683	20.708
gi|52352062|gb|AY610478.1|	tan	227.34346	26.795987	0.7937373	0.10890547	11.162
gi|52352069|gb|AY610485.1|	royalblue	468.0856	57.052405	0.9723791	0.0054876	5.11
gi|52352072|gb|AY610488.1|	violet	986.28209	43.932222	0.9603345	0.00942654	7.376
gi|52352090|gb|AY610506.1|	darkorange	114.87423	23.038501	0.8845162	0.04628559	17.466
gi|52352705|gb|AY609625.1|	greenyellow	93.312231	16.273484	0.7210988	0.1692192	61.622
gi|52352724|gb|AY609644.1|	black	859.04095	102.9351	0.9874641	0.00168169	17.168
gi|52352730|gb|AY609650.1|	red	697.84066	107.55704	0.9646535	0.00793484	64.99
gi|54020965|ref|NM_001005726.1|	black	835.65424	100.97853	0.9833182	0.00257992	56.298
gi|54607194|ref|NM_001006592.1|	lightyellow	391.27318	59.49712	0.9833101	0.00258181	8968.848
gi|55741470|ref|NM_214042.1|	yellow	262.98005	118.65053	0.9702777	0.00612366	58.81
gi|55741485|ref|NM_213855.1|	blue	468.36956	91.24538	0.88625	0.04525936	1927.712
gi|55741489|ref|NM_214136.1|	white	969.50178	46.961922	0.9880525	0.00156483	81.572
gi|55741720|ref|NM_001001534.1|	darkgreen	238.29685	43.084487	0.9926464	0.00075614	2271.864
gi|55741808|ref|NM_213748.1|	tan	252.9335	40.478335	0.9370655	0.0187726	2553.502
gi|55741810|ref|NM_001001863.1|	navy	68.769687	11.322116	0.5641569	0.32186129	5340.018
gi|55741820|ref|NM_214202.1|	darkred	208.96604	45.229401	0.981838	0.00293017	2.466
gi|55742741|ref|NM_213920.1|	turquoise	156.14195	52.751464	0.5432034	0.34407664	261.64
gi|55742771|ref|NM_001007191.1|	navy	978.93302	17.970736	-0.044554	0.94329071	17.44

gi|55742846|ref|NM_214329.1|	turquoise	145.99234	25.318574	0.3363387	0.5799772	64.448
gi|56606056|ref|NM_001008481.1|	lightcyan	723.00309	44.013872	0.8949848	0.04020219	9.63
gi|56711365|ref|NM_001008688.1|	yellow	165.30758	72.341802	0.8549065	0.06488144	5923.556
gi|56711367|ref|NM_001008691.1|	magenta	85.946894	34.70276	0.7749639	0.12373144	18.066
gi|56792875|gb|AY682217.1|	white	278.9731	16.638308	-0.594517	0.29035665	5.992
gi|57527981|ref|NM_001009576.1|	royalblue	498.15827	59.536679	0.9819571	0.00290144	10.516
gi|57528034|ref|NM_001009582.1|	magenta	152.85813	63.902663	0.9246518	0.02454557	18.254
gi|58332861|ref|NM_001011507.1|	greenyellow	89.506839	18.180061	0.764732	0.13204581	34.452
gi|58801554|ref|NM_001011727.1|	magenta	482.36163	66.716174	0.9285309	0.02268818	50.18
gi|60302865|ref|NM_001012613.1|	pink	926.77249	148.32899	0.9977798	0.00012554	3.478
gi|60419073|gb|AY856514.2|	paleturquoise	149.41749	19.886778	0.9083373	0.03285185	19.602
gi|61098755|gb|AY820766.1|	navy	62.56553	9.2964369	-0.71395	0.17555867	11.396
gi|61696637|gb|AY803100.1|	purple	294.15746	77.977648	0.9920263	0.0008537	3.562
gi|62082661|gb|AY870324.1|	lightyellow	318.20854	56.482186	0.9699053	0.00623877	49.048
gi|6650729|gb|AF116346.1|AF116346	lightyellow	638.8355	53.853553	0.9569825	0.01064093	7.51
gi|68534989|ref|NM_001025224.1|	turquoise	241.70027	54.344529	-0.26242	0.66975174	172.544
gi|70778850|ref|NM_214094.2|	navy	54.349047	25.503033	0.8345888	0.07872286	5.592
gi|71834281|ref|NM_214197.2|	darkred	219.89639	40.920369	0.9543191	0.01163959	121.884
gi|72535201|ref|NM_001031794.1|	turquoise	963.65016	206.27326	0.9304054	0.02180775	96.148
gi|73853889|ref|NM_001032359.1|	navy	294.24817	28.072728	0.8734441	0.05300741	555.606
gi|73853893|ref|NM_001032358.1|	green	860.8863	116.35098	0.99809	0.00010017	8.862
gi|74024908|ref|NM_001032379.1|	turquoise	884.6622	204.63641	0.9343252	0.02000352	34.302
gi|74136750|ref|NM_001033009.1|	pink	710.73398	130.90131	0.9726394	0.00541043	10.314
gi|74136760|ref|NM_001033014.1|	turquoise	454.39615	121.42464	0.8297282	0.08215472	149.766
gi|77628017|ref|NM_001008689.2|	tan	155.01799	44.446407	0.9703792	0.00609242	81.746
gi|78364925|gb|DQ225117.1|	lightgreen	322.38501	36.301111	0.9389946	0.01792141	5.278
gi|80971509|ref|NM_001037151.1|	yellow	239.57174	112.63181	0.9579238	0.01029507	12.162
gi|80971515|ref|NM_001037152.1|	lightcyan	247.9365	40.271097	0.9080163	0.0330229	3.79
gi|82617535|ref|NM_001037322.1|	pink	934.87902	143.14888	0.9904012	0.00112729	3.692
gi|83921642|ref|NM_001038007.1|	green	990.30849	98.794167	0.9583373	0.01014429	63.646
gi|84579912|ref|NM_001038644.1|	black	665.55666	97.007027	0.9753385	0.00463181	17.21
gi|84619523|ref|NM_001038694.1|	darkmagenta	248.25485	4.7570421	-0.844937	0.07156953	9.77
gi|84874691|ref|NM_001038632.1|	darkmagenta	78.055922	8.4214047	0.9685468	0.00666457	17.908
gi|89574200|gb|DQ402993.1|	lightcyan	429.05922	45.578124	0.9319322	0.02109901	3.21
gi|89886172|ref|NM_001039748.1|	turquoise	992.69528	206.8584	0.9296183	0.02217608	40.826
gi|89886176|ref|NM_001039750.1|	red	936.02577	123.19101	0.9916628	0.00091268	4.418
gi|90017493|ref|NM_214184.2|	brown	824.88394	116.56028	0.9596336	0.00967647	245.14
gi|92020093|dbj|AB237777.1|	turquoise	124.08682	45.422563	0.5223089	0.36658972	14.468
gnl|UG|Ssc#S14768592	salmon	216.40111	72.788768	0.9849959	0.00220126	5.7
gnl|UG|Ssc#S14889148	red	785.58494	119.60485	0.9861691	0.00194853	2.59
gnl|UG|Ssc#S14891120	turquoise	279.33282	58.82413	0.6030049	0.28170053	30.63
gnl|UG|Ssc#S14891216	yellow	292.4885	122.00244	0.9744985	0.00486985	2.872
gnl|UG|Ssc#S14894901	orange	191.98059	35.123806	0.9674735	0.00700745	13.114
gnl|UG|Ssc#S14900031	white	264.38956	16.795092	-0.551096	0.33566514	55.172
gnl|UG|Ssc#S14900094	darkturquoise	237.85665	44.723346	0.98898	0.0013864	5.288
gnl|UG|Ssc#S15981045	darkturquoise	227.22413	45.271762	0.9891201	0.00136007	3.858
gnl|UG|Ssc#S16511729	purple	207.05113	58.434912	0.9308284	0.02161061	138.328
gnl|UG|Ssc#S16513699	pink	894.62975	149.62591	0.9996875	6.63E-06	1.476

gnl|UG|Ssc#S16513921	lightgreen	228.38194	22.170291	0.7326965	0.15907853	5.418
gnl|UG|Ssc#S16514435	darkgrey	851.25917	49.48276	0.9794193	0.00353329	4.53
gnl|UG|Ssc#S16514740	turquoise	433.64206	146.88185	0.8843079	0.04640938	6.758
gnl|UG|Ssc#S16514775	lightgreen	477.20929	38.860131	0.9327694	0.02071359	4.206
gnl|UG|Ssc#S16514958	pink	872.08932	148.90816	0.9987571	5.26E-05	77.926
gnl|UG|Ssc#S16515033	navy	163.06111	39.477583	0.9542109	0.01168077	12.246
gnl|UG|Ssc#S16515706	grey60	314.90295	68.420208	0.9967709	0.00022016	34.856
gnl|UG|Ssc#S16515765	yellow	250.06895	116.19273	0.9645336	0.00797508	21.078
gnl|UG|Ssc#S16515841	pink	894.97665	145.04011	0.9933798	0.00064597	1.598
gnl|UG|Ssc#S16516581	pink	942.00351	144.7948	0.9928165	0.00073008	7.256
gnl|UG|Ssc#S16517530	darkgrey	1000.0671	50.346312	0.981127	0.00310358	5.122
gnl|UG|Ssc#S16517601	pink	946.52929	145.63748	0.9938987	0.00057157	4.67
gnl|UG|Ssc#S16517614	darkgrey	992.54319	51.851955	0.9876534	0.00164381	9.042
gnl|UG|Ssc#S16517726	royalblue	393.90701	50.265673	0.9437654	0.01587235	228.74
gnl|UG|Ssc#S16518321	turquoise	393.4555	61.724555	0.0467265	0.94052767	8.052
gnl|UG|Ssc#S16761746	darkmagenta	84.675523	10.015471	0.9503156	0.01319473	62.274
gnl|UG|Ssc#S16761785	navy	87.102131	32.928668	0.8951851	0.04008848	24.838
gnl|UG|Ssc#S16761818	orange	276.47693	37.642946	0.9995206	1.26E-05	5.536
gnl|UG|Ssc#S16763004	lightyellow	340.04317	48.53029	0.9402568	0.01737145	5.044
gnl|UG|Ssc#S16763253	pink	931.16854	148.2198	0.9976311	0.00013836	5.694
gnl|UG|Ssc#S16763399	navy	894.62975	19.364124	0.0047011	0.99401438	1.668
gnl|UG|Ssc#S16763478	lightgreen	356.54688	37.285999	0.9216761	0.02600194	11.652
gnl|UG|Ssc#S16763868	greenyellow	63.192719	13.581205	0.498656	0.39248485	24.158
gnl|UG|Ssc#S16764486	pink	913.35599	149.63395	0.9996656	7.34E-06	5.216
gnl|UG|Ssc#S16764576	midnightblue	851.18008	74.311297	0.9871779	0.00173955	5.306
gnl|UG|Ssc#S16767076	magenta	339.27799	84.399372	0.9925487	0.00077126	43.86
gnl|UG|Ssc#S16767694	red	908.49305	127.64415	0.999264	2.40E-05	5.658
gnl|UG|Ssc#S16768026	yellow	306.84562	127.85507	0.9880101	0.00157317	28.05
gnl|UG|Ssc#S16768494	pink	949.21872	147.95618	0.9971915	0.00017859	3.622
gnl|UG|Ssc#S16769025	turquoise	382.9655	123.32494	0.8237652	0.08642595	17.066
gnl|UG|Ssc#S16769591	red	953.52131	122.63981	0.9905252	0.00110553	4.854
gnl|UG|Ssc#S16769624	blue	975.3702	116.24941	0.9329416	0.02063461	14.214
gnl|UG|Ssc#S16770187	yellow	278.44124	125.27983	0.9822155	0.00283944	15.526
gnl|UG|Ssc#S16771004	pink	918.87517	149.58726	0.9995805	1.03E-05	3.456
gnl|UG|Ssc#S17499579	turquoise	767.73066	203.10637	0.9450912	0.01531747	13.918
gnl|UG|Ssc#S17499681	darkorange	171.08489	21.132704	0.8512588	0.06730474	323.88
gnl|UG|Ssc#S17500017	skyblue	651.11938	38.392302	0.9639446	0.00817384	8.016
gnl|UG|Ssc#S17501425	orange	248.11881	37.253713	0.9957905	0.00032764	7.624
gnl|UG|Ssc#S17503882	turquoise	974.33729	200.43367	0.915764	0.02897449	7.504
gnl|UG|Ssc#S17504229	purple	148.07333	39.869576	0.8400548	0.07491808	25.174
gnl|UG|Ssc#S17505316	pink	694.80583	122.04845	0.9587225	0.01000453	3.196
gnl|UG|Ssc#S17505417	salmon	239.36964	71.813953	0.9814692	0.00301969	21.244
gnl|UG|Ssc#S17505677	pink	804.472	141.39393	0.9882174	0.00153258	2.892
gnl|UG|Ssc#S17505852	pink	952.90607	145.89389	0.9942396	0.00052437	3.4
gnl|UG|Ssc#S17508314	pink	860.77958	147.68324	0.9970775	0.00018957	1.592
gnl|UG|Ssc#S17509886	darkgreen	141.86205	29.666695	0.8803576	0.04877641	628.702
gnl|UG|Ssc#S17510046	green	924.84566	109.63231	0.9821177	0.00286286	6.056
gnl|UG|Ssc#S17510077	violet	117.21627	3.6151283	-0.377178	0.53140486	68.53

gnl|UG|Ssc#S17510233	navy	106.28728	32.043607	0.8940798	0.04071726	64.248
gnl|UG|Ssc#S17510311	brown	643.44408	123.59987	0.9737203	0.00509383	6.412
gnl|UG|Ssc#S17510336	turquoise	837.48465	186.30334	0.9048093	0.03474763	11.64
gnl|UG|Ssc#S17510396	blue	352.89215	84.191328	0.8653877	0.05807534	66.802
gnl|UG|Ssc#S17510398	green	932.74203	109.52602	0.9817993	0.00293953	135.022
gnl|UG|Ssc#S17510452	lightgreen	194.03721	21.881026	0.7031342	0.1852767	15.46
gnl|UG|Ssc#S17510525	skyblue	491.96362	44.562338	0.9998439	2.34E-06	44.822
gnl|UG|Ssc#S17510796	salmon	223.62514	72.436418	0.9835648	0.00252304	11.56
gnl|UG|Ssc#S17510917	turquoise	827.25219	205.79066	0.9399386	0.01750956	2.068
gnl|UG|Ssc#S17510986	blue	897.36593	151.76845	0.9975891	0.00014205	150.16
gnl|UG|Ssc#S17511033	white	247.52563	14.979054	-0.444701	0.45304842	27.494
gnl|UG|Ssc#S17511146	black	943.41123	96.506952	0.9727163	0.00538769	7.24
gnl|UG|Ssc#S17511222	darkred	131.28429	20.412418	0.7784746	0.12091604	10.318
gnl|UG|Ssc#S17511240	violet	204.21758	7.8763108	0.1664142	0.78909692	166.85
gnl|UG|Ssc#S17511285	white	234.34557	16.799497	-0.653234	0.23195108	537.194
gnl|UG|Ssc#S17511386	darkgrey	825.18073	49.994574	0.9813186	0.00305651	567.994
gnl|UG|Ssc#S17511419	turquoise	222.52803	75.132932	0.6241423	0.26045009	28.844
gnl|UG|Ssc#S17511439	darkgreen	240.33719	39.359297	0.9693617	0.00640804	32.688
gnl|UG|Ssc#S17511723	lightgreen	346.64308	34.381444	0.9044227	0.03495746	13.298
gnl|UG|Ssc#S17511887	salmon	195.70657	72.724291	0.9859203	0.00200127	5.518
gnl|UG|Ssc#S17512005	blue	903.32273	142.68249	0.9831182	0.0026264	26.74
gnl|UG|Ssc#S17512032	pink	851.25251	146.16805	0.9950022	0.00042382	3.474
gnl|UG|Ssc#S17512154	yellow	256.75701	119.96542	0.9735468	0.00514423	349.08
gnl|UG|Ssc#S17512186	greenyellow	239.04369	33.98481	0.9541504	0.0117038	29.262
gnl|UG|Ssc#S17512237	tan	150.34164	35.362081	0.9184241	0.02762421	4.28
gnl|UG|Ssc#S17512269	grey	562.34631	14.588076	0.9790584	0.00362644	28.13
gnl|UG|Ssc#S17512288	yellow	262.00834	79.055999	0.8812521	0.04823714	5.872
gnl|UG|Ssc#S17513253	pink	618.36318	118.28061	0.9518896	0.01257573	14.35
gnl|UG|Ssc#S17513449	darkorange	164.97643	32.178893	0.9786163	0.00374162	25.22
gnl|UG|Ssc#S17513694	paleturquoise	150.2437	15.826283	0.8390516	0.07561201	7.906
gnl|UG|Ssc#S17513715	turquoise	132.41377	35.639205	0.4744735	0.4193833	22.848
gnl|UG|Ssc#S17513789	turquoise	903.27116	220.91397	0.9710784	0.00587861	6.13
gnl|UG|Ssc#S17513958	brown	689.98024	135.10572	0.9983801	7.82E-05	10.416
gnl|UG|Ssc#S17513979	magenta	400.58016	76.773326	0.9672627	0.00707544	109.992
gnl|UG|Ssc#S17514069	turquoise	831.84255	207.17384	0.9561614	0.01094566	8.132
gnl|UG|Ssc#S17514190	blue	423.88343	97.599921	0.8991596	0.0378534	1.036
gnl|UG|Ssc#S17514318	lightyellow	514.01149	54.464277	0.9649548	0.00783395	11.37
gnl|UG|Ssc#S17514409	magenta	243.64261	82.927256	0.9904278	0.0011226	126.616
gnl|UG|Ssc#S17514565	lightcyan	604.3646	52.610328	0.9478482	0.01418439	10.894
gnl|UG|Ssc#S17514632	magenta	409.85032	77.872581	0.9697822	0.00627696	399.728
gnl|UG|Ssc#S17514667	magenta	434.31013	72.14281	0.9503223	0.01319209	3.254
gnl|UG|Ssc#S17514838	darkorange	128.19322	29.689685	0.9506208	0.01307394	14.204
gnl|UG|Ssc#S17515039	yellow	299.26062	127.01277	0.9844446	0.00232349	8.298
gnl|UG|Ssc#S17515108	navy	278.9891	27.923176	0.8672704	0.05687803	10.774
gnl|UG|Ssc#S17515316	black	814.88095	108.48438	0.9989504	4.08E-05	29.644
gnl|UG|Ssc#S17515611	brown	753.7086	124.8141	0.9761094	0.00441685	27.462
gnl|UG|Ssc#S17515701	turquoise	968.2933	212.13383	0.9420248	0.01661055	22.88
gnl|UG|Ssc#S17515754	red	913.6448	124.91804	0.9947153	0.00046081	7.496

gnl|UG|Ssc#S17515773	green	972.93841	104.27827	0.9702377	0.00613601	25.942
gnl|UG|Ssc#S17515809	darkmagenta	270.76359	5.1307161	-0.795233	0.10774913	19.2
gnl|UG|Ssc#S17515842	green	763.1865	112.37684	0.9922882	0.00081201	10.278
gnl|UG|Ssc#S17516048	grey60	255.26032	53.494203	0.9446099	0.01551818	54.216
gnl|UG|Ssc#S17516064	magenta	316.37154	85.303607	0.9958908	0.00031601	4.746
gnl|UG|Ssc#S17516079	tan	136.45774	43.148507	0.9731838	0.00525019	16.908
gnl|UG|Ssc#S17516095	tan	129.69556	37.725223	0.9279069	0.02298378	17.85
gnl|UG|Ssc#S17516148	pink	984.43784	141.92093	0.988367	0.00150352	23.446
gnl|UG|Ssc#S17516311	yellow	238.41156	109.74134	0.9508722	0.01297473	416.9
gnl|UG|Ssc#S17516549	greenyellow	159.28707	24.425968	0.8458557	0.07094463	28.444
gnl|UG|Ssc#S17516828	grey60	307.56397	68.163945	0.9963503	0.00026454	124.356
gnl|UG|Ssc#S17516920	lightcyan	394.39923	59.019481	0.9947366	0.00045802	20.44
gnl|UG|Ssc#S17516962	navy	70.9106	35.471009	0.9447424	0.01546283	3.986
gnl|UG|Ssc#S17517004	turquoise	837.78046	208.4526	0.9612522	0.00910258	15.058
gnl|UG|Ssc#S17517072	yellow	300.42333	130.07152	0.9906018	0.00109217	4.114
gnl|UG|Ssc#S17517096	navy	69.903871	8.7068118	-0.729809	0.16158626	26.954
gnl|UG|Ssc#S17517121	greenyellow	178.69443	35.549762	0.9648795	0.00785911	3.07
gnl|UG|Ssc#S17517147	lightcyan	609.28237	52.799293	0.9483332	0.01398801	2.102
gnl|UG|Ssc#S17517405	brown	602.23056	125.61291	0.9848229	0.00223936	4.738
gnl|UG|Ssc#S17517774	salmon	166.84305	65.00622	0.9621601	0.00878575	8.344
gnl|UG|Ssc#S17517964	turquoise	1018.4303	211.6276	0.9353788	0.01952728	21.412
gnl|UG|Ssc#S17518104	lightyellow	287.48379	51.324374	0.9479426	0.01414612	2.9
gnl|UG|Ssc#S17518111	red	952.16383	123.12044	0.9913589	0.00096299	3.364
gnl|UG|Ssc#S17518115	green	701.32953	91.8697	0.9482576	0.01401858	11.064
gnl|UG|Ssc#S17518180	lightyellow	393.4967	62.558295	0.9933669	0.00064786	12.206
gnl|UG|Ssc#S17518305	brown	623.1071	125.516	0.9856305	0.00206328	10.732
gnl|UG|Ssc#S17518307	yellow	269.09853	117.99104	0.9685933	0.00664984	18.75
gnl|UG|Ssc#S17518363	greenyellow	159.26933	34.267184	0.9421189	0.01657038	29.408
gnl|UG|Ssc#S17518385	yellow	315.5642	122.47497	0.9776034	0.00400997	163.926
gnl|UG|Ssc#S17518565	yellow	299.96269	134.89187	0.9994749	1.44E-05	36.39
gnl|UG|Ssc#S17518664	turquoise	387.27241	136.4377	0.8663887	0.05743778	16.424
gnl|UG|Ssc#S17518706	magenta	183.44408	68.527466	0.9451848	0.01527855	15.52
gnl|UG|Ssc#S17518750	brown	559.18813	119.92456	0.9760648	0.00442918	10.732
gnl|UG|Ssc#S17518758	darkgreen	105.09753	18.763428	0.7821195	0.11801368	22.104
gnl|UG|Ssc#S17518763	darkturquoise	185.31379	42.744314	0.9787791	0.00369908	17.348
gnl|UG|Ssc#S17518772	darkmagenta	78.239046	9.3598668	0.9741839	0.00496	20.602
gnl|UG|Ssc#S17518773	yellow	301.66711	131.51531	0.9942907	0.00051741	40.37
gnl|UG|Ssc#S17518795	brown	699.47681	119.42663	0.9617498	0.00892847	18.71
gnl|UG|Ssc#S17518997	lightcyan	337.09796	55.658603	0.9831013	0.00263033	14.306
gnl|UG|Ssc#S17519017	midnightblue	901.71713	73.419853	0.9843964	0.00233429	9.424
gnl|UG|Ssc#S17524473	brown	765.09338	134.79592	0.9940097	0.00055606	27.03
gnl|UG|Ssc#S17524578	lightgreen	438.9502	36.530454	0.9524069	0.01237442	10.518
gnl|UG|Ssc#S17524627	turquoise	430.27043	122.39446	0.7665706	0.13054001	26.698
gnl|UG|Ssc#S17524633	lightgreen	259.58591	30.80129	0.8834241	0.0469357	112.362
gnl|UG|Ssc#S17524752	turquoise	963.6287	206.17013	0.9286625	0.022626	22.624
gnl|UG|Ssc#S17524900	brown	729.12689	135.67757	0.9981947	9.21E-05	10.296
gnl|UG|Ssc#S17524908	pink	864.40729	144.67838	0.9929614	0.00070812	1.65
gnl|UG|Ssc#S17524953	brown	630.32044	124.40071	0.9842037	0.00237759	17.242

gnl|UG|Ssc#S17524981	lightyellow	551.75474	62.490462	0.9922672	0.00081534	14.526
gnl|UG|Ssc#S17524990	black	645.61665	98.395739	0.978056	0.00388932	11.888
gnl|UG|Ssc#S17525104	darkturquoise	176.15284	44.564043	0.9866508	0.00184777	23.934
gnl|UG|Ssc#S17525203	magenta	128.23091	44.43934	0.8456977	0.07105196	3.938
gnl|UG|Ssc#S17525225	red	969.36198	120.14576	0.9862318	0.00193531	4.434
gnl|UG|Ssc#S17525376	navy	288.66787	24.740921	0.8330758	0.07978628	15.928
gnl|UG|Ssc#S17525378	black	849.52004	108.29892	0.9984326	7.45E-05	10.93
gnl|UG|Ssc#S17525409	turquoise	212.50142	71.117316	0.5477448	0.33923041	8.764
gnl|UG|Ssc#S17525410	grey	535.09449	14.889707	0.9837403	0.00248279	7.036
gnl|UG|Ssc#S17525682	red	921.20767	125.90422	0.9963512	0.00026444	72.126
gnl|UG|Ssc#S17525753	grey	603.33466	14.193849	0.9738832	0.00504669	5.38
gnl|UG|Ssc#S17525826	brown	91.72254	8.2190244	0.1909114	0.75840875	3.778
gnl|UG|Ssc#S17525865	black	915.01122	102.27127	0.9861922	0.00194365	13.94
gnl|UG|Ssc#S17526139	darkgreen	162.52337	15.519248	0.6644781	0.22118174	18.044
gnl|UG|Ssc#S17526150	darkgreen	225.9427	40.492436	0.974733	0.004803	18.524
gnl|UG|Ssc#S17526193	turquoise	971.97277	210.74563	0.9382369	0.01825421	51.252
gnl|UG|Ssc#S17526232	brown	601.9492	127.52189	0.9869242	0.00179136	9.218
gnl|UG|Ssc#S17526234	pink	894.62975	149.62591	0.9996875	6.63E-06	1.54
gnl|UG|Ssc#S17526265	black	848.79176	107.59268	0.9971708	0.00018057	3.01
gnl|UG|Ssc#S17526422	darkgreen	244.01673	43.457028	0.9974788	0.0001519	60.114
gnl|UG|Ssc#S17526634	grey60	272.20097	59.705216	0.9676592	0.0069477	30.83
gnl|UG|Ssc#S17526834	purple	199.80047	54.578317	0.9145634	0.02959066	31.928
gnl|UG|Ssc#S17526835	orange	213.02218	11.46129	0.708653	0.18029958	5.088
gnl|UG|Ssc#S17526874	blue	974.20899	136.40143	0.9710006	0.00590227	201.596
gnl|UG|Ssc#S17526922	black	908.67034	102.12119	0.9858024	0.00202642	10.146
gnl|UG|Ssc#S17526927	yellow	312.91879	127.91133	0.9876647	0.00164154	19.942
gnl|UG|Ssc#S17526940	blue	598.63254	111.87693	0.9286539	0.02263006	11.84
gnl|UG|Ssc#S17526987	tan	272.56661	26.674342	0.809198	0.0971339	13.45
gnl|UG|Ssc#S17527330	orange	205.73748	35.329202	0.9742316	0.00494628	42.81
gnl|UG|Ssc#S17527420	purple	268.23392	65.866361	0.9546356	0.0115194	75.458
gnl|UG|Ssc#S17527438	brown	769.70219	134.25551	0.9928331	0.00072755	14.792
gnl|UG|Ssc#S17527454	darkred	199.93685	40.324594	0.9420032	0.01661977	14.714
gnl|UG|Ssc#S17527505	blue	811.2297	147.24768	0.9915092	0.00093799	8.488
gnl|UG|Ssc#S18260225	turquoise	911.45954	208.5542	0.9401943	0.01739854	24.804
gnl|UG|Ssc#S18261002	turquoise	183.25753	74.439066	0.6553735	0.2298913	41.746
gnl|UG|Ssc#S18262172	brown	871.62396	117.15825	0.9579881	0.01027156	9.406
gnl|UG|Ssc#S18262357	magenta	479.37906	68.69662	0.9360449	0.01922807	5.214
gnl|UG|Ssc#S18263440	turquoise	844.5999	193.77597	0.9261179	0.02383801	11.838
gnl|UG|Ssc#S18263529	lightyellow	525.81652	60.20509	0.9838123	0.00246635	159.506
gnl|UG|Ssc#S18263775	brown	821.19693	127.12457	0.9764166	0.00433214	17.588
gnl|UG|Ssc#S18263925	paleturquoise	190.89669	22.859323	0.9408052	0.01713425	6.302
gnl|UG|Ssc#S18266615	green	960.61185	109.7576	0.983154	0.00261806	8.034
gnl|UG|Ssc#S18267174	lightyellow	673.88141	51.875716	0.9481864	0.01404736	12.344
gnl|UG|Ssc#S18267480	blue	488.97652	95.58924	0.899954	0.03741161	2.732
gnl|UG|Ssc#S18267647	brown	753.6177	131.86451	0.9871792	0.00173927	9.088
gnl|UG|Ssc#S18268726	brown	616.59312	129.92872	0.9914428	0.00094902	2.338
gnl|UG|Ssc#S18268814	turquoise	438.01365	59.145613	0.0952858	0.87886219	10.476
gnl|UG|Ssc#S18269890	red	750.81837	115.24529	0.9785771	0.0037519	2.204

gnl|UG|Ssc#S18270450	violet	907.68042	47.007861	0.9907782	0.00106159	9.116
gnl|UG|Ssc#S18270499	navy	894.62975	19.364124	0.0047011	0.99401438	1.348
gnl|UG|Ssc#S18272639	purple	283.80615	78.037152	0.9941726	0.00053354	13.2
gnl|UG|Ssc#S18272798	pink	1003.0901	132.19343	0.9735664	0.00513854	4.676
gnl|UG|Ssc#S18272948	turquoise	333.0758	124.75013	0.7968934	0.10646945	9.41
gnl|UG|Ssc#S18274718	turquoise	357.54514	110.48152	0.8226096	0.08726132	7.324
gnl|UG|Ssc#S18276256	pink	842.37751	140.95241	0.9877068	0.00163316	15.742
gnl|UG|Ssc#S18277539	orange	172.45247	16.273991	0.7698809	0.12784179	56.136
gnl|UG|Ssc#S18277825	violet	855.60095	46.315063	0.9968602	0.0002111	2.472
gnl|UG|Ssc#S18281860	lightcyan	599.49472	54.211337	0.9566395	0.01076786	11.994
gnl|UG|Ssc#S18282381	navy	153.77298	34.948221	0.910334	0.03179411	67.876
gnl|UG|Ssc#S18283093	darkturquoise	158.7219	38.584021	0.9511339	0.01287169	20.704
gnl|UG|Ssc#S18283235	turquoise	1014.03	207.29657	0.9268655	0.02347982	8.802
gnl|UG|Ssc#S18284222	black	795.55243	108.04192	0.9981713	9.38E-05	2.46
gnl|UG|Ssc#S18284670	pink	923.87745	149.0656	0.9988184	4.88E-05	14.528
gnl|UG|Ssc#S18289792	lightcyan	561.15593	56.397027	0.9699634	0.00622075	7.256
gnl|UG|Ssc#S18290449	turquoise	864.69617	211.0511	0.9616961	0.00894721	462.178
gnl|UG|Ssc#S18291801	lightgreen	413.82847	37.493574	0.9023628	0.03608211	8.244
gnl|UG|Ssc#S18292679	darkgrey	823.2405	50.998339	0.9855554	0.00207944	6.348
gnl|UG|Ssc#S18292759	darkgrey	945.6037	53.941585	0.9968428	0.00021286	11.44
gnl|UG|Ssc#S18295197	violet	790.10937	44.008215	0.9912673	0.00097834	3.034
gnl|UG|Ssc#S18301441	darkgrey	614.73716	39.184639	0.9302786	0.02186696	8.536
gnl|UG|Ssc#S18301820	tan	237.70699	39.010513	0.9176637	0.02800809	5.886
gnl|UG|Ssc#S18304172	turquoise	240.03843	90.243158	0.6994401	0.18862971	4.412
gnl|UG|Ssc#S18333765	turquoise	540.99333	167.797	0.8989252	0.03798411	2.426
gnl|UG|Ssc#S18336060	navy	974.40316	18.567806	0.1324765	0.83182041	2.7
gnl|UG|Ssc#S18336254	violet	907.56758	46.927368	0.9913781	0.00095979	3.396
gnl|UG|Ssc#S18336569	navy	81.223613	6.8985806	-0.976383	0.00434142	17.206
gnl|UG|Ssc#S18354030	magenta	207.35533	76.668817	0.9714203	0.00577498	2.48
gnl|UG|Ssc#S18354391	turquoise	201.7854	47.960308	-0.292289	0.63321519	83.09
gnl|UG|Ssc#S18354494	navy	133.05634	36.325289	0.955789	0.0110848	48.238
gnl|UG|Ssc#S18354763	turquoise	946.50786	218.67289	0.9543308	0.01163512	9.514
gnl|UG|Ssc#S18354874	darkred	201.21475	43.166029	0.977828	0.00394995	8.736
gnl|UG|Ssc#S18355787	greenyellow	44.933767	7.2127302	0.36433	0.54659764	27.126
gnl|UG|Ssc#S18355850	tan	104.56254	25.121006	0.8002147	0.10392407	6.294
gnl|UG|Ssc#S18355951	green	845.45924	112.88182	0.9927799	0.00073567	15.354
gnl|UG|Ssc#S18355973	white	235.22528	16.613629	-0.622693	0.26189266	39.922
gnl|UG|Ssc#S18355999	yellow	243.80045	108.51805	0.9492408	0.01362297	8.734
gnl|UG|Ssc#S18356219	lightcyan	200.12975	34.780674	0.8680452	0.05638758	18.608
gnl|UG|Ssc#S18356676	darkgreen	104.39495	22.766181	0.8010598	0.10327935	27.48
gnl|UG|Ssc#S18357153	darkturquoise	239.62089	42.11151	0.9687228	0.00660887	2.1
gnl|UG|Ssc#S18357219	turquoise	371.9052	102.82345	0.7706554	0.1272129	43.116
gnl|UG|Ssc#S18357288	royalblue	710.60401	55.991851	0.9696043	0.00633232	8.048
gnl|UG|Ssc#S18357398	brown	753.22109	134.14107	0.9949434	0.00043132	14.216
gnl|UG|Ssc#S18357511	turquoise	338.3924	127.42138	0.8117529	0.09522837	24.218
gnl|UG|Ssc#S18357580	blue	972.5377	140.60494	0.9783869	0.00380186	16.7
gnl|UG|Ssc#S18357620	darkgreen	233.85152	43.015688	0.9927268	0.00074379	1430.466
gnl|UG|Ssc#S18357751	black	826.53627	106.50944	0.9951946	0.00039959	15.7

gnl|UG|Ssc#S18357775	orange	325.37526	35.595462	0.9872958	0.00171564	10.384
gnl|UG|Ssc#S18358012	darkgrey	941.03572	52.910669	0.992701	0.00074774	3.416
gnl|UG|Ssc#S18358064	yellow	181.05897	84.910132	0.8951572	0.04010431	44.296
gnl|UG|Ssc#S18358279	turquoise	436.70925	110.53558	0.7880174	0.1133623	43.6
gnl|UG|Ssc#S18358567	blue	897.48588	150.81705	0.9964061	0.0002585	2.03
gnl|UG|Ssc#S18358587	magenta	494.97441	64.348669	0.9201694	0.02674963	8.456
gnl|UG|Ssc#S18358692	darkturquoise	159.17725	39.828687	0.9588797	0.00994767	5.21
gnl|UG|Ssc#S18358900	lightcyan	666.4084	48.911754	0.9248064	0.02447062	6.51
gnl|UG|Ssc#S18358928	navy	507.51552	11.882465	-0.041488	0.94719077	20.32
gnl|UG|Ssc#S18359073	brown	806.18787	127.93387	0.9813366	0.00305212	10.764
gnl|UG|Ssc#S18359101	darkgrey	932.79021	53.725162	0.9960663	0.00029599	9.812
gnl|UG|Ssc#S18359197	yellow	231.13137	106.65682	0.9464514	0.01475491	7.118
gnl|UG|Ssc#S18359209	brown	572.80608	122.32115	0.9784423	0.0037873	6.556
gnl|UG|Ssc#S18359264	magenta	106.46834	42.52302	0.8340223	0.07912054	65.608
gnl|UG|Ssc#S18359557	violet	148.89601	8.0499274	0.520161	0.36892365	11.118
gnl|UG|Ssc#S18359743	turquoise	973.36303	222.20316	0.960339	0.00942493	11.128
gnl|UG|Ssc#S18359767	brown	728.74067	118.60619	0.9585744	0.0100582	8.904
gnl|UG|Ssc#S18359803	lightyellow	628.72735	56.131069	0.9666009	0.00729033	9.382
gnl|UG|Ssc#S18360059	midnightblue	988.47457	75.531328	0.9901684	0.00116851	9.066
gnl|UG|Ssc#S18360480	pink	895.07316	145.0791	0.9934342	0.00063803	2.396
gnl|UG|Ssc#S18360885	green	974.66766	107.03135	0.9770471	0.00415995	18.52
gnl|UG|Ssc#S18361423	turquoise	939.47053	222.42963	0.9714855	0.00575529	4.276
gnl|UG|Ssc#S18361485	grey60	307.27475	65.280138	0.9867163	0.00183418	6.886
gnl|UG|Ssc#S18377137	turquoise	246.729	95.659838	0.6627011	0.22287432	39.42
gnl|UG|Ssc#S18377744	pink	629.31228	118.44973	0.9520427	0.01251604	4.978
gnl|UG|Ssc#S18377876	turquoise	490.07141	160.16658	0.9106089	0.03164933	6.424
gnl|UG|Ssc#S18378149	blue	985.28606	132.35807	0.9637099	0.00825348	12.23
gnl|UG|Ssc#S18378291	blue	869.28344	147.74244	0.9914035	0.00095555	5.446
gnl|UG|Ssc#S18378422	magenta	422.618	74.633141	0.9594272	0.00975049	11.808
gnl|UG|Ssc#S18378552	yellow	300.29502	134.60691	0.9988473	4.70E-05	44.074
gnl|UG|Ssc#S18378606	turquoise	152.57019	48.528771	0.5289955	0.35934725	53.4
gnl|UG|Ssc#S18378680	darkred	67.917192	13.370553	0.6874553	0.19962408	42.68
gnl|UG|Ssc#S18378791	lightyellow	465.27737	61.945766	0.9907012	0.00107491	29.936
gnl|UG|Ssc#S18378792	red	519.14329	81.979651	0.9101755	0.03187764	10.664
gnl|UG|Ssc#S18378854	lightgreen	250.12565	30.665243	0.8550907	0.06475981	19.208
gnl|UG|Ssc#S18378957	lightyellow	568.19889	59.102097	0.9789302	0.00365972	4.966
gnl|UG|Ssc#S18378982	violet	913.07835	46.678649	0.9863894	0.00190221	3.862
gnl|UG|Ssc#S18378997	black	927.6836	99.99883	0.9805162	0.00325514	9.106
gnl|UG|Ssc#S18379057	magenta	171.57946	66.52572	0.937796	0.01844877	22.252
gnl|UG|Ssc#S18379095	red	950.81714	123.54037	0.9920861	0.00084412	1.746
gnl|UG|Ssc#S18379108	pink	926.41031	149.32193	0.999168	2.88E-05	35.868
gnl|UG|Ssc#S18379116	lightyellow	375.35936	60.012218	0.983719	0.00248766	19.9
gnl|UG|Ssc#S18379252	black	803.29181	108.67588	0.999357	1.96E-05	10.864
gnl|UG|Ssc#S18379332	turquoise	225.88539	67.276679	0.570968	0.31472125	25.488
gnl|UG|Ssc#S18379352	magenta	335.14695	77.230096	0.9727493	0.00537796	3.558
gnl|UG|Ssc#S18379413	darkgrey	645.86793	41.270081	0.9410982	0.01700796	14.896
gnl|UG|Ssc#S18379594	orange	282.41121	37.308561	0.9966778	0.00022975	3.622
gnl|UG|Ssc#S18379751	darkgreen	254.77043	42.178708	0.9889291	0.001396	35.814

gnl|UG|Ssc#S18379761	yellow	304.59825	130.64985	0.9910994	0.00100666	6.404
gnl|UG|Ssc#S18380107	brown	326.95832	62.568698	0.8275381	0.08371573	56.844
gnl|UG|Ssc#S18380485	pink	936.53503	145.34819	0.9935717	0.0006181	18.072
gnl|UG|Ssc#S18380508	pink	894.62975	149.62591	0.9996875	6.63E-06	0.128
gnl|UG|Ssc#S18380537	greenyellow	92.85046	13.156553	0.4795079	0.41374952	32.482
gnl|UG|Ssc#S18380653	darkgreen	210.42919	40.739821	0.9754114	0.00461132	35.38
gnl|UG|Ssc#S18380710	darkgrey	990.90988	50.755975	0.983353	0.00257189	16.706
gnl|UG|Ssc#S18380942	pink	897.85258	148.09906	0.9976258	0.00013882	17.29
gnl|UG|Ssc#S18381258	tan	133.84209	38.293093	0.9323137	0.02092308	15.392
gnl|UG|Ssc#S18381264	yellow	261.93765	124.64444	0.9820805	0.00287178	3.42
gnl|UG|Ssc#S18381418	navy	166.86774	40.75471	0.9711673	0.0058516	2.078
gnl|UG|Ssc#S18381432	turquoise	516.32956	164.15659	0.898965	0.03796189	28.418
gnl|UG|Ssc#S18381627	pink	894.62975	149.62591	0.9996875	6.63E-06	1.54
gnl|UG|Ssc#S18382551	navy	99.908692	7.5068219	-0.831227	0.08109147	18.696
gnl|UG|Ssc#S18382627	pink	742.31316	135.66717	0.9797098	0.00345889	16.96
gnl|UG|Ssc#S18382640	navy	62.931566	4.6456546	-0.821304	0.0882084	18.272
gnl|UG|Ssc#S18383248	turquoise	241.22569	52.801973	0.5164111	0.37300683	88.486
gnl|UG|Ssc#S18383312	yellow	293.20146	133.2237	0.9967039	0.00022705	19.078
gnl|UG|Ssc#S18383420	brown	573.92661	83.849797	0.8916719	0.04209781	29.312
gnl|UG|Ssc#S18383442	magenta	398.11957	78.096319	0.9713285	0.00580273	13.556
gnl|UG|Ssc#S18383451	royalblue	610.6862	63.950929	0.9983678	7.91E-05	11.612
gnl|UG|Ssc#S18383491	brown	714.30819	131.837	0.9930462	0.00069537	22.916
gnl|UG|Ssc#S18383636	red	938.19307	125.98013	0.9962989	0.00027014	2.452
gnl|UG|Ssc#S18383637	turquoise	423.13704	53.028318	0.1998348	0.74726616	15.534
gnl|UG|Ssc#S18383725	greenyellow	51.238247	12.369248	0.239197	0.69837459	56.816
gnl|UG|Ssc#S18383839	green	915.58534	114.66862	0.9937464	0.00059309	11.232
gnl|UG|Ssc#S18383932	darkgreen	182.3456	28.953112	0.8894818	0.04336613	72.27
gnl|UG|Ssc#S18384163	lightyellow	517.68344	63.172332	0.9955141	0.00036042	3.552
gnl|UG|Ssc#S18384305	black	852.38635	106.16603	0.9943902	0.00050395	8.462
gnl|UG|Ssc#S18384469	midnightblue	817.12655	67.540129	0.9670581	0.00714165	8.328
gnl|UG|Ssc#S18384885	lightgreen	222.60766	23.183162	0.711881	0.17740636	12.712
gnl|UG|Ssc#S18385915	pink	894.62975	149.62591	0.9996875	6.63E-06	2.182
gnl|UG|Ssc#S18386342	white	810.24748	45.108066	0.9462312	0.0148455	1.824
gnl|UG|Ssc#S18386541	magenta	566.21498	50.931025	0.8599095	0.06160328	21.344
gnl|UG|Ssc#S18386569	green	821.73615	116.20181	0.9986684	5.83E-05	0.934
gnl|UG|Ssc#S18387078	darkorange	121.22062	25.383685	0.8956086	0.03984842	24.2
gnl|UG|Ssc#S18387120	yellow	208.75574	86.259766	0.9001293	0.03731435	12.398
gnl|UG|Ssc#S18387223	green	976.30679	106.54348	0.9760676	0.00442841	15.392
gnl|UG|Ssc#S18387292	brown	821.88267	124.69916	0.9708352	0.0059527	12.534
gnl|UG|Ssc#S18387383	black	829.63122	105.2165	0.9926681	0.0007528	12.454
gnl|UG|Ssc#S18387501	turquoise	115.70718	38.863929	0.5322476	0.35583747	21.05
gnl|UG|Ssc#S18387558	turquoise	689.36601	180.89166	0.8974153	0.03882931	16.364
gnl|UG|Ssc#S18387565	pink	894.62975	149.62591	0.9996875	6.63E-06	1.09
gnl|UG|Ssc#S18387589	turquoise	770.18589	182.45056	0.9146173	0.02956287	20.492
gnl|UG|Ssc#S18387708	yellow	283.44289	128.15746	0.9880101	0.00157316	98.382
gnl|UG|Ssc#S18387783	brown	692.7367	133.87206	0.9930163	0.00069985	26.86
gnl|UG|Ssc#S18546005	blue	728.68664	98.546098	0.9006721	0.03701368	35.446
gnl|UG|Ssc#S18546038	royalblue	860.4598	50.698129	0.9440342	0.01575934	9.102

gnl|UG|Ssc#S18546074	turquoise	932.52369	201.32217	0.9243897	0.02467273	11.62
gnl|UG|Ssc#S18546081	green	804.72801	112.88057	0.9920088	0.00085651	13.14
gnl|UG|Ssc#S18546149	grey60	269.16847	56.570052	0.9557162	0.01111207	78.546
gnl|UG|Ssc#S18546387	lightgreen	561.78267	33.595103	0.9428111	0.01627573	8.258
gnl|UG|Ssc#S18546390	magenta	128.10629	44.329655	0.8499468	0.06818315	101.666
gnl|UG|Ssc#S18546426	brown	580.38244	119.76145	0.976407	0.00433476	16.67
gnl|UG|Ssc#S18546477	orange	229.3237	14.164301	0.7145794	0.17499818	37.866
gnl|UG|Ssc#S18546502	green	815.17523	111.2253	0.989305	0.00132559	4.57
gnl|UG|Ssc#S18546597	red	900.72637	125.36582	0.9955339	0.00035804	2.238
gnl|UG|Ssc#S18546698	paleturquoise	174.02344	22.63022	0.9467761	0.01462163	3.868
gnl|UG|Ssc#S18546855	grey60	253.41983	55.423058	0.9530671	0.01211904	23.912
gnl|UG|Ssc#S18546948	grey60	312.28029	68.867653	0.9982919	8.47E-05	14.914
gnl|UG|Ssc#S18546984	black	592.88902	88.619323	0.955902	0.01104254	1.26
gnl|UG|Ssc#S18547064	turquoise	563.80814	59.594233	0.3333602	0.58355054	3.64
gnl|UG|Ssc#S18547312	purple	276.49384	75.709252	0.9863213	0.0019165	21.44
gnl|UG|Ssc#S18547360	turquoise	971.86328	206.50099	0.9346199	0.01986995	12.056
gnl|UG|Ssc#S18547454	turquoise	779.37401	198.53929	0.9292267	0.02236006	4.58
gnl|UG|Ssc#S18547573	lightgreen	342.54419	36.501092	0.9428254	0.01626968	164.39
gnl|UG|Ssc#S18547696	yellow	313.54492	122.60112	0.9783581	0.00380946	10.952
gnl|UG|Ssc#S18547766	lightcyan	594.62757	51.087185	0.9414777	0.01684481	33.25
gnl|UG|Ssc#S18547863	greenyellow	228.35904	39.194958	0.997581	0.00014277	10.164
gnl|UG|Ssc#S18548324	darkturquoise	216.77667	46.015941	0.994623	0.00047293	19.602
gnl|UG|Ssc#S18548354	magenta	413.29733	76.068498	0.9641788	0.00809461	6.548
gnl|UG|Ssc#S18548531	skyblue	551.99013	42.742191	0.9899004	0.00121655	12.306
gnl|UG|Ssc#S18548560	tan	176.92113	40.319216	0.9454638	0.0151627	8.408
gnl|UG|Ssc#S18548647	pink	895.07316	145.0791	0.9934342	0.00063803	2.396
gnl|UG|Ssc#S18548755	lightgreen	383.03482	38.748368	0.9449185	0.01538938	8.286
gnl|UG|Ssc#S18548799	turquoise	136.96006	45.270903	0.491163	0.4007749	9.17
gnl|UG|Ssc#S18548805	grey60	308.67107	68.256564	0.9965128	0.00024707	18.76
gnl|UG|Ssc#S18548934	turquoise	364.88403	92.883321	0.7285332	0.16269791	14.168
gnl|UG|Ssc#S18548948	black	601.90262	89.064925	0.9571401	0.01058275	23.852
gnl|UG|Ssc#S18549036	black	746.60657	103.99098	0.9902862	0.00114758	59.016
gnl|UG|Ssc#S18549168	lightgreen	516.5169	38.746966	0.9473242	0.01439756	21.086
gnl|UG|Ssc#S18549184	brown	491.13306	98.956997	0.9346963	0.01983535	8.658
gnl|UG|Ssc#S18549395	lightcyan	487.50345	56.543575	0.9761512	0.00440527	15.288
gnl|UG|Ssc#S18549494	black	871.24092	106.84381	0.995268	0.00039048	72.476
gnl|UG|Ssc#S18549536	turquoise	196.45487	69.467694	0.6096494	0.27497303	75.332
gnl|UG|Ssc#S18549559	paleturquoise	161.08981	21.639457	0.9321271	0.02100908	4.442
gnl|UG|Ssc#S18549601	skyblue	616.6673	40.130908	0.9746268	0.00483324	6.006
gnl|UG|Ssc#S18549808	brown	639.50009	129.26561	0.9901561	0.00117069	7.538
gnl|UG|Ssc#S18549874	purple	254.31544	64.673931	0.9504923	0.01312474	9.776
gnl|UG|Ssc#S18549905	turquoise	825.07545	195.94947	0.933197	0.02051763	99.034
gnl|UG|Ssc#S18550002	turquoise	418.34635	60.887779	0.0908258	0.88451614	7.484
gnl|UG|Ssc#S18550089	purple	305.68219	74.857705	0.9825275	0.00276519	7.004
gnl|UG|Ssc#S18550098	turquoise	1013.7219	201.62882	0.9164113	0.02864397	5.804
gnl|UG|Ssc#S18550130	turquoise	186.1498	44.42408	-0.300726	0.62295569	10.416
gnl|UG|Ssc#S18550180	turquoise	171.75441	64.136092	0.651906	0.23323262	33.696
gnl|UG|Ssc#S18550687	black	722.13071	104.91417	0.9920549	0.00084912	4.928

gnl|UG|Ssc#S18550712	blue	871.04339	150.76186	0.9969749	0.00019964	3.11
gnl|UG|Ssc#S18550902	lightyellow	337.43289	58.046998	0.9767178	0.00424958	87.922
gnl|UG|Ssc#S18551086	greenyellow	61.909127	14.618535	0.4253285	0.47525747	38.49
gnl|UG|Ssc#S18551209	darkorange	143.46672	29.442892	0.9486163	0.01387382	144.588
gnl|UG|Ssc#S18551494	blue	657.22926	118.57607	0.943926	0.01580481	25.27
gnl|UG|Ssc#S18551520	darkmagenta	110.30881	8.9516192	0.9263673	0.02371832	7.846
gnl|UG|Ssc#S18551559	turquoise	392.92149	61.652636	0.0482776	0.9385549	8.012
gnl|UG|Ssc#S18551571	blue	938.68723	148.75848	0.9921076	0.00084068	22.328
gnl|UG|Ssc#S18551577	brown	735.13785	118.07266	0.9569122	0.01066692	14.4
gnl|UG|Ssc#S18551614	red	938.82636	125.89874	0.9961582	0.00028568	1.614
gnl|UG|Ssc#S18551801	blue	836.20434	121.6062	0.94492	0.01538877	36.224
gnl|UG|Ssc#S18551834	paleturquoise	164.63467	19.568092	0.8976211	0.03871377	69.614
gnl|UG|Ssc#S18551845	royalblue	824.4534	54.689456	0.961234	0.00910896	49.928
gnl|UG|Ssc#S18552012	turquoise	255.96665	102.20631	0.7239938	0.16667092	111.7
gnl|UG|Ssc#S18552237	turquoise	461.09061	153.09261	0.9007119	0.03699166	8.602
gnl|UG|Ssc#S18552312	turquoise	612.15948	153.85244	0.8392143	0.07549932	25.242
gnl|UG|Ssc#S18552383	navy	71.981367	13.4231	0.6477347	0.2372695	63.146
gnl|UG|Ssc#S18552404	yellow	294.55485	134.6428	0.9993096	2.18E-05	24.054
gnl|UG|Ssc#S18552570	salmon	230.64752	73.026861	0.9859439	0.00199625	130.354
gnl|UG|Ssc#S18552999	salmon	195.26922	70.027207	0.9777621	0.00396752	18.358
gnl|UG|Ssc#S18553035	darkred	65.12127	2.1092339	0.1199045	0.84769945	202.584
gnl|UG|Ssc#S18553137	green	863.66192	112.64851	0.9917116	0.00090469	20.644
gnl|UG|Ssc#S18553158	yellow	285.71999	121.44848	0.9772958	0.00409268	64.642
gnl|UG|Ssc#S18553245	turquoise	404.96599	142.24225	0.8711973	0.05440607	23.212
gnl|UG|Ssc#S18553368	turquoise	268.02305	106.17267	0.7621113	0.13420108	13.178
gnl|UG|Ssc#S18553403	magenta	190.33557	73.344725	0.9599694	0.00955646	507.954
gnl|UG|Ssc#S18553581	green	942.85682	107.37288	0.9768993	0.00420012	34.422
gnl|UG|Ssc#S18553619	magenta	229.63245	79.381548	0.9802642	0.00331839	3.682
gnl|UG|Ssc#S18553622	magenta	343.34959	84.550121	0.9928356	0.00072717	10.568
gnl|UG|Ssc#S18553623	green	750.82546	110.74984	0.9886875	0.00144189	6.888
gnl|UG|Ssc#S18553702	yellow	135.31005	61.325004	0.8218586	0.0878055	17.572
gnl|UG|Ssc#S18553815	greenyellow	166.51187	35.670441	0.9276021	0.0231286	2.984
gnl|UG|Ssc#S18553939	magenta	234.29256	82.096957	0.9879344	0.00158806	7.384
gnl|UG|Ssc#S18554195	turquoise	181.7556	45.132045	0.5167165	0.37267392	238.488
gnl|UG|Ssc#S18554238	turquoise	606.11546	181.83388	0.9365027	0.01902331	8.91
gnl|UG|Ssc#S18554366	green	967.37018	106.95924	0.9782188	0.00384621	10.212
gnl|UG|Ssc#S18554454	magenta	230.52417	81.327642	0.9855992	0.00207002	2.95
gnl|UG|Ssc#S18554465	green	814.95291	114.2318	0.9945007	0.00048914	6.364
gnl|UG|Ssc#S18554486	turquoise	449.0966	149.90894	0.8768058	0.05093622	14.062
gnl|UG|Ssc#S18554583	violet	900.45231	46.910847	0.9916158	0.00092041	3.438
gnl|UG|Ssc#S18554602	orange	313.62869	34.953952	0.9822965	0.0028201	9.272
gnl|UG|Ssc#S18554787	brown	665.17874	125.14562	0.9760763	0.00442601	30.396
gnl|UG|Ssc#S18554865	darkgreen	91.917875	18.865024	0.76695	0.13022991	14.386
gnl|UG|Ssc#S18554887	darkturquoise	135.73978	36.306205	0.9356868	0.01938876	50.798
gnl|UG|Ssc#S18554897	lightgreen	471.86569	36.322253	0.8890347	0.04362652	12.022
gnl|UG|Ssc#S18555004	darkred	156.39119	36.88601	0.9394878	0.01770586	39.044
gnl|UG|Ssc#S18555055	brown	756.77774	132.64467	0.9901146	0.00117809	13.662
gnl|UG|Ssc#S18555062	lightcyan	683.8715	47.187344	0.9164535	0.02862248	10.322

gnl|UG|Ssc#S18555355	blue	986.27344	139.99998	0.9769734	0.00417997	10.672
gnl|UG|Ssc#S18555410	turquoise	899.7946	211.60716	0.9488661	0.01377328	16.432
gnl|UG|Ssc#S18555414	navy	74.435066	32.79661	0.9572869	0.01052868	929.572
gnl|UG|Ssc#S18555551	turquoise	217.09275	57.131915	0.5795863	0.30574565	16.882
gnl|UG|Ssc#S18555613	brown	721.68427	125.24947	0.9729349	0.00532325	7.086
gnl|UG|Ssc#S18555750	blue	706.64679	141.57181	0.9849316	0.0022154	7.936
gnl|UG|Ssc#S18555897	brown	729.93962	123.94498	0.9749724	0.00473505	28.164
gnl|UG|Ssc#S18555925	darkred	101.26784	19.968616	0.752254	0.14239906	18.576
gnl|UG|Ssc#S18556194	lightgreen	341.46435	33.445058	0.8518037	0.06694102	16.118
gnl|UG|Ssc#S18556295	darkturquoise	236.78909	44.296051	0.9832582	0.00259384	41.372
gnl|UG|Ssc#S18556436	darkred	184.89778	42.56075	0.9727755	0.00537021	3.792
gnl|UG|Ssc#S18556442	purple	229.39194	51.420842	0.8938159	0.04086786	10.42
gnl|UG|Ssc#S18556589	navy	79.039962	35.73731	0.9322924	0.02093289	28.894
gnl|UG|Ssc#S18556801	darkgreen	245.35905	42.236294	0.9882616	0.00152398	42.882
gnl|UG|Ssc#S18556879	blue	651.68068	136.77341	0.9772185	0.00411357	2.252
gnl|UG|Ssc#S18556951	green	593.65175	96.24633	0.9575735	0.01042333	24.568
gnl|UG|Ssc#S18557192	lightyellow	634.05991	57.447298	0.972491	0.0054544	4.412
gnl|UG|Ssc#S18557214	midnightblue	742.89038	71.932719	0.9805086	0.00325705	3.39
gnl|UG|Ssc#S18557422	darkturquoise	213.16832	44.695176	0.9834289	0.00255435	19.44
gnl|UG|Ssc#S18557470	grey60	282.55867	62.798087	0.9792174	0.00358529	37.094
gnl|UG|Ssc#S18557502	brown	604.52141	127.64085	0.9889312	0.0013956	6.794
gnl|UG|Ssc#S18557542	yellow	251.0617	101.6859	0.9326362	0.02077478	29.664
gnl|UG|Ssc#S18557598	green	796.38823	108.71341	0.9840068	0.0024221	7.946
gnl|UG|Ssc#S18557699	brown	831.02224	116.31605	0.9519268	0.01256122	10.696
gnl|UG|Ssc#S18557771	yellow	289.77829	132.61363	0.9958964	0.00031537	2.772
gnl|UG|Ssc#S18557837	paleturquoise	173.07571	24.827722	0.9648067	0.00788347	165.334
gnl|UG|Ssc#S18557900	brown	572.49617	105.97875	0.9410202	0.01704154	14.184
gnl|UG|Ssc#S18557979	royalblue	456.85851	56.775262	0.9711188	0.00586633	7.39
gnl|UG|Ssc#S18557981	pink	894.62975	149.62591	0.9996875	6.63E-06	1.476
gnl|UG|Ssc#S18558242	red	901.54092	126.39498	0.9972807	0.00017015	4.706
gnl|UG|Ssc#S18558291	midnightblue	979.83523	73.955348	0.9856665	0.00205555	15.092
gnl|UG|Ssc#S18558299	blue	799.4294	149.50193	0.9948975	0.0004372	30.594
gnl|UG|Ssc#S19537170	white	966.12684	46.886121	0.9936876	0.00060148	57.954
gnl|UG|Ssc#S19537267	yellow	300.60459	124.52119	0.9800091	0.00338281	7.506
gnl|UG|Ssc#S19537684	turquoise	196.29441	79.227571	0.6453199	0.23961509	0.732
gnl|UG|Ssc#S19537979	yellow	181.28798	57.532164	0.8137062	0.09377936	12.772
gnl|UG|Ssc#S19538858	grey60	289.39021	60.483563	0.9700619	0.00619028	8.67
gnl|UG|Ssc#S19538970	white	948.48679	47.170635	0.9881552	0.00154473	21.278
gnl|UG|Ssc#S19538990	greenyellow	181.467	38.515569	0.9672076	0.00709327	8.366
gnl|UG|Ssc#S19539315	tan	124.92017	37.195872	0.9236859	0.02501532	15.7
gnl|UG|Ssc#S19539468	midnightblue	932.00442	77.228965	0.9950185	0.00042174	20.276
gnl|UG|Ssc#S19539494	magenta	174.58419	62.789634	0.9277259	0.02306976	1.884
gnl|UG|Ssc#S19539620	green	486.40711	80.003705	0.9155537	0.02908211	53.214
gnl|UG|Ssc#S19539691	greenyellow	219.65777	40.140952	0.9780558	0.00388936	13.414
gnl|UG|Ssc#S19539740	grey60	286.2825	56.975502	0.9574497	0.01046879	32.392
gnl|UG|Ssc#S19540060	black	691.15515	100.58018	0.9829305	0.00267024	2.88
gnl|UG|Ssc#S19540140	darkturquoise	251.02665	31.258873	0.8980681	0.03846318	3.142
gnl|UG|Ssc#S19540267	turquoise	308.73293	99.709601	0.6832115	0.20355896	8.148

gnl|UG|Ssc#S19540289	red	1001.434	113.87192	0.9746772	0.00481886	8.632
gnl|UG|Ssc#S19540314	black	928.45573	99.494503	0.9793927	0.00354012	8.488
gnl|UG|Ssc#S19540375	black	781.51735	92.668876	0.9657436	0.00757187	23.758
gnl|UG|Ssc#S19540378	purple	262.82203	66.349784	0.9548215	0.01144895	21.944
gnl|UG|Ssc#S19540384	lightcyan	561.71457	48.179781	0.9263413	0.02373081	10.87
gnl|UG|Ssc#S19540409	midnightblue	925.0819	78.547122	0.9986335	6.06E-05	7.874
gnl|UG|Ssc#S19540410	turquoise	211.03863	79.772608	0.6269816	0.25762963	32.462
gnl|UG|Ssc#S19540415	royalblue	766.02314	60.10422	0.9832233	0.00260195	4.262
gnl|UG|Ssc#S19540417	black	615.45627	90.214006	0.9598817	0.00958776	0.862
gnl|UG|Ssc#S19540505	tan	154.04505	32.385724	0.8790742	0.04955342	28.902
gnl|UG|Ssc#S19540539	lightgreen	367.5988	32.986891	0.8898574	0.04314778	21.28
gnl|UG|Ssc#S19540632	orange	257.8113	36.178226	0.9846659	0.00227415	0.678
gnl|UG|Ssc#S19540688	navy	278.33422	28.436039	0.9032384	0.03560264	22.686
gnl|UG|Ssc#S19540774	purple	274.10921	71.423256	0.9718078	0.00565824	35.916
gnl|UG|Ssc#S19540934	midnightblue	761.26154	71.968042	0.9805699	0.00324173	15.258
gnl|UG|Ssc#S19541050	royalblue	521.89486	60.949358	0.9874331	0.00168793	9.712
gnl|UG|Ssc#S19541067	brown	506.51278	91.471042	0.9112589	0.03130791	23.582
gnl|UG|Ssc#S19541084	darkturquoise	181.64553	45.045644	0.987633	0.00164788	137.074
gnl|UG|Ssc#S19541103	green	447.56592	74.6244	0.9002075	0.03727095	13.406
gnl|UG|Ssc#S19541153	magenta	339.30796	82.025251	0.986116	0.00195975	6.46
gnl|UG|Ssc#S19541498	orange	237.21983	30.979572	0.9576545	0.01039364	14.43
gnl|UG|Ssc#S19541523	blue	991.08599	134.01075	0.9664418	0.00734231	64.982
gnl|UG|Ssc#S19541571	midnightblue	687.1908	69.076333	0.971657	0.00570357	8.938
gnl|UG|Ssc#S19541572	grey60	250.78689	50.880589	0.9337379	0.02027065	23.174
gnl|UG|Ssc#S19541579	turquoise	679.49756	165.45359	0.8679128	0.0564713	60.24
gnl|UG|Ssc#S19541658	orange	185.82742	14.047596	0.6990068	0.18902411	15.638
gnl|UG|Ssc#S19541728	black	893.87634	103.04506	0.9872693	0.00172101	20.872
gnl|UG|Ssc#S19541992	green	704.64825	88.063787	0.9350543	0.01967354	11.024
gnl|UG|Ssc#S19542004	greenyellow	178.39339	29.678306	0.8368607	0.0771344	100.56
gnl|UG|Ssc#S19542017	brown	748.5538	126.96591	0.9755255	0.00457936	27.308
gnl|UG|Ssc#S19542031	royalblue	429.38256	54.236794	0.9607552	0.00927756	84.09
gnl|UG|Ssc#S19542061	blue	783.25431	150.18688	0.9968304	0.0002141	26.676
gnl|UG|Ssc#S19542087	lightyellow	506.8248	63.223442	0.995125	0.0004083	11.318
gnl|UG|Ssc#S19542100	orange	238.31487	18.541036	0.8026238	0.10208947	8.898
gnl|UG|Ssc#S19542226	darkturquoise	179.83386	41.136767	0.9668515	0.00720874	32.202
gnl|UG|Ssc#S19542246	turquoise	309.69292	101.98517	0.7158961	0.1738265	3.184
gnl|UG|Ssc#S19542298	turquoise	704.72	173.94921	0.9050209	0.03463298	10.67
gnl|UG|Ssc#S19542511	orange	123.42128	23.448643	0.8720777	0.05385664	3.74
gnl|UG|Ssc#S19542514	turquoise	1000.3422	217.8916	0.9483682	0.0139739	5.834
gnl|UG|Ssc#S19542525	greenyellow	135.00098	21.950977	0.8308482	0.08135996	114.05
gnl|UG|Ssc#S19542622	turquoise	177.4843	43.632789	-0.332549	0.58452475	20.984
gnl|UG|Ssc#S19542649	lightgreen	490.03113	35.558192	0.9501344	0.0132666	7.6
gnl|UG|Ssc#S19542654	yellow	285.7925	112.11372	0.9530605	0.01212159	5.86
gnl|UG|Ssc#S19542684	black	836.85064	102.30746	0.9861417	0.00195431	44.948
gnl|UG|Ssc#S19542699	turquoise	424.43832	115.84822	0.8013558	0.10305382	11.566
gnl|UG|Ssc#S19542749	brown	800.56342	126.01776	0.9728403	0.00535111	106.928
gnl|UG|Ssc#S19542862	midnightblue	731.55586	72.13208	0.980988	0.00313786	9.528
gnl|UG|Ssc#S19543319	blue	774.83778	148.03031	0.9936267	0.00061019	11.206

gnl|UG|Ssc#S19543409	turquoise	420.94434	129.81931	0.8159708	0.09210787	4.582
gnl|UG|Ssc#S19543418	red	973.24303	117.77398	0.9822102	0.00284071	21.38
gnl|UG|Ssc#S19543452	violet	932.52361	46.397601	0.9813407	0.00305111	6.388
gnl|UG|Ssc#S19543565	grey60	306.00179	67.239283	0.9930873	0.00068921	13.094
gnl|UG|Ssc#S19543756	black	714.97128	94.470419	0.9706863	0.0059982	3.824
gnl|UG|Ssc#S19543764	magenta	349.26084	75.960895	0.9689334	0.00654245	21.192
gnl|UG|Ssc#S19544231	white	265.5444	14.643953	-0.622222	0.26236201	6.228
gnl|UG|Ssc#S19545362	black	804.71183	102.09031	0.9860169	0.00198074	64.432
gnl|UG|Ssc#S19545397	skyblue	430.19033	43.376659	0.9940893	0.00054501	235.046
gnl|UG|Ssc#S19545412	salmon	217.67475	67.448971	0.9682889	0.00674643	6.842
gnl|UG|Ssc#S19545451	navy	190.12464	36.894045	0.943416	0.01601968	14.724
gnl|UG|Ssc#S19545467	black	942.98953	98.817715	0.9781297	0.00386977	24.364
gnl|UG|Ssc#S19545641	lightyellow	344.64774	57.80163	0.9759563	0.00445925	4.888
gnl|UG|Ssc#S19545786	royalblue	836.37002	54.767795	0.9617061	0.00894372	9.156
gnl|UG|Ssc#S19545977	midnightblue	923.57985	73.892436	0.9857725	0.00203282	81.978
gnl|UG|Ssc#S19546005	lightgreen	506.3479	37.785645	0.9162234	0.02873982	11.256
gnl|UG|Ssc#S19546024	skyblue	442.59988	42.756601	0.9914457	0.00094853	13.186
gnl|UG|Ssc#S19546046	darkgreen	210.81755	23.776209	0.8157285	0.09228627	14.4
gnl|UG|Ssc#S19546062	tan	213.68687	44.244233	0.9599443	0.0095654	13.104
gnl|UG|Ssc#S19546082	turquoise	507.02135	164.41469	0.9105931	0.03165766	18.264
gnl|UG|Ssc#S19546091	tan	145.95141	45.074937	0.9807295	0.00320195	23.972
gnl|UG|Ssc#S19546104	lightyellow	399.90332	59.370028	0.9818052	0.00293809	6.838
gnl|UG|Ssc#S19546106	turquoise	198.72091	78.525681	0.5954358	0.28941594	48.568
gnl|UG|Ssc#S19546114	darkred	126.65317	29.445663	0.8757604	0.0515775	12.412
gnl|UG|Ssc#S19546165	blue	823.7533	149.10814	0.9941621	0.00053499	4.58
gnl|UG|Ssc#S19546218	darkorange	133.36712	33.768012	0.9835394	0.00252889	14.712
gnl|UG|Ssc#S19546257	royalblue	491.39946	59.13044	0.9805611	0.00324392	24.882
gnl|UG|Ssc#S19546269	brown	774.54116	133.94795	0.9915829	0.00092582	8.596
gnl|UG|Ssc#S19546291	greenyellow	174.14445	30.821504	0.9119415	0.03095064	7.35
gnl|UG|Ssc#S19546334	turquoise	1020.5283	213.98067	0.9390351	0.01790368	12.28
gnl|UG|Ssc#S19547691	lightgreen	275.3976	32.141657	0.8698782	0.05523261	115.694
gnl|UG|Ssc#S19547811	turquoise	473.66316	143.99171	0.874718	0.05221947	8.71
gnl|UG|Ssc#S19547860	midnightblue	788.08559	70.591786	0.9765223	0.00430312	26.28
gnl|UG|Ssc#S19547879	lightgreen	232.30823	28.616384	0.8532049	0.06600844	3.088
gnl|UG|Ssc#S19548031	darkorange	63.045681	14.754205	0.7280466	0.16312241	11.858
gnl|UG|Ssc#S19548740	white	243.40119	17.127126	-0.683241	0.20353118	64.64
gnl|UG|Ssc#S19548981	darkgrey	1002.2189	50.728738	0.9828888	0.00268001	10.322
gnl|UG|Ssc#S19549058	brown	712.2742	132.69572	0.9903533	0.00113573	11.094
gnl|UG|Ssc#S19549165	turquoise	997.15564	220.25303	0.9567662	0.01072093	12.104
gnl|UG|Ssc#S19549174	turquoise	775.41143	208.29948	0.9676527	0.0069498	7.16
gnl|UG|Ssc#S19549267	turquoise	699.23362	184.83107	0.909406	0.0322843	6.55
gnl|UG|Ssc#S19549653	navy	50.525259	25.401521	0.8378716	0.07643079	26.434
gnl|UG|Ssc#S19550340	grey60	237.41524	48.871073	0.9271314	0.02335283	2.46
gnl|UG|Ssc#S19550965	pink	940.66374	148.63009	0.998156	9.50E-05	42.192
gnl|UG|Ssc#S20938341	darkgreen	190.53194	20.408017	0.7586626	0.13705287	5.236
gnl|UG|Ssc#S20944779	salmon	205.58847	69.7549	0.9762175	0.00438697	6.48
gnl|UG|Ssc#S20945025	grey60	309.64421	67.757728	0.9946223	0.00047301	113.82
gnl|UG|Ssc#S20945368	tan	133.1585	24.987378	0.7853349	0.11547089	3.142

gnl|UG|Ssc#S21107071	turquoise	990.21466	208.25089	0.9344487	0.0199475	29.25
gnl|UG|Ssc#S21107119	green	701.12214	77.816146	0.9056742	0.03427973	3.922
gnl|UG|Ssc#S21550606	navy	936.7011	18.806275	-0.057201	0.92720857	2.962
gnl|UG|Ssc#S21554547	yellow	263.81736	115.83071	0.9641506	0.00810415	7.47
gnl|UG|Ssc#S21558492	white	919.03342	47.355391	0.9758989	0.0044752	11.398
gnl|UG|Ssc#S21573643	turquoise	889.58333	217.03392	0.966843	0.00721149	4.236
gnl|UG|Ssc#S21575132	turquoise	229.37674	60.732598	0.5493962	0.33747234	2.232
gnl|UG|Ssc#S21575274	turquoise	405.23079	139.33432	0.8590899	0.06213664	25.24
gnl|UG|Ssc#S22271675	salmon	162.628	59.408029	0.9435438	0.01596576	10.676
gnl|UG|Ssc#S22272334	lightyellow	637.90087	52.033785	0.9496982	0.01344016	8.884
gnl|UG|Ssc#S22272834	pink	970.00414	144.99987	0.9929003	0.00071736	5.034
gnl|UG|Ssc#S22273263	salmon	175.82583	53.341518	0.9193262	0.02717104	5.83
gnl|UG|Ssc#S22273337	black	527.83353	81.510259	0.9377103	0.01848666	5.854
gnl|UG|Ssc#S22274193	turquoise	252.0118	94.056872	0.6994027	0.18866377	10.904
gnl|UG|Ssc#S22274398	skyblue	494.15398	44.19388	0.9981557	9.50E-05	10.974
gnl|UG|Ssc#S22274609	red	853.99243	123.21115	0.9920603	0.00084825	6.866
gnl|UG|Ssc#S22274737	orange	184.71879	33.917377	0.958588	0.01005327	5.194
gnl|UG|Ssc#S22275000	turquoise	655.39257	184.10046	0.9250528	0.02435135	33.356
gnl|UG|Ssc#S22275082	royalblue	688.98074	63.454652	0.9964441	0.00025441	8.51
gnl|UG|Ssc#S22275237	salmon	235.4404	75.691167	0.992909	0.00071603	16.19
gnl|UG|Ssc#S22275831	grey60	308.43917	67.616999	0.9943268	0.00051252	10.022
gnl|UG|Ssc#S22275888	lightgreen	491.47103	34.595142	0.9404485	0.01728842	7.424
gnl|UG|Ssc#S22275958	turquoise	311.19631	115.84796	0.8072725	0.09857753	26.214
gnl|UG|Ssc#S22276100	white	923.95895	47.595563	0.977856	0.00394248	2.576
gnl|UG|Ssc#S22276642	red	775.65153	117.78301	0.9830593	0.00264011	3.644
gnl|UG|Ssc#S22277516	turquoise	362.90439	55.149165	0.0313991	0.96002797	51.278
gnl|UG|Ssc#S22277550	pink	838.63131	145.43667	0.9939706	0.0005615	10.006
gnl|UG|Ssc#S22278253	green	706.87572	106.20538	0.9798017	0.00343546	9.55
gnl|UG|Ssc#S22278643	darkturquoise	121.42028	34.267743	0.9269214	0.02345309	25.504
gnl|UG|Ssc#S22279857	turquoise	976.70878	201.62906	0.9238814	0.02492003	30.344
gnl|UG|Ssc#S22279946	green	582.69636	94.364721	0.953374	0.01200093	5.244
gnl|UG|Ssc#S22280355	lightyellow	310.3706	45.712586	0.926914	0.02345666	4.122
gnl|UG|Ssc#S22281680	magenta	404.46629	75.446037	0.9633126	0.00838888	10.132
gnl|UG|Ssc#S22281703	brown	282.92076	21.423961	0.6230728	0.26151456	11.04
gnl|UG|Ssc#S22282893	turquoise	211.64921	86.198657	0.6621438	0.22340594	12.884
gnl|UG|Ssc#S22283333	turquoise	956.76316	202.08608	0.9229504	0.02537495	32.524
gnl|UG|Ssc#S22284067	tan	135.50663	32.276321	0.8780479	0.05017754	8.456
gnl|UG|Ssc#S22284475	turquoise	826.24472	197.17514	0.9403379	0.01733631	18.532
gnl|UG|Ssc#S22284677	turquoise	342.72032	120.13708	0.8177318	0.09081444	4.618
gnl|UG|Ssc#S22285435	grey60	298.25948	62.355732	0.9766711	0.00426235	5.238
gnl|UG|Ssc#S22286222	orange	92.43738	21.357685	0.8162646	0.09189168	9.398
gnl|UG|Ssc#S22286260	magenta	282.56259	81.954226	0.9880963	0.00155624	17.074
gnl|UG|Ssc#S22286650	black	646.89029	96.546055	0.9744994	0.00486957	9.934
gnl|UG|Ssc#S22287254	lightyellow	396.38173	52.406178	0.9568066	0.01070597	26.444
gnl|UG|Ssc#S22287363	midnightblue	920.24846	78.346336	0.9980913	0.00010008	7.9
gnl|UG|Ssc#S22304929	lightcyan	330.87264	54.658322	0.9790682	0.0036239	4.598
gnl|UG|Ssc#S22312937	blue	927.52472	148.49316	0.9917335	0.00090111	7.208
gnl|UG|Ssc#S22313154	darkturquoise	214.21316	44.226831	0.9807243	0.00320325	176.978

gnl|UG|Ssc#S22313956	pink	863.60806	148.75168	0.9985468	6.65E-05	11.062
gnl|UG|Ssc#S22316206	skyblue	520.75554	44.099292	0.9973272	0.00016581	13.52
gnl|UG|Ssc#S23689583	turquoise	874.34406	201.92272	0.9312713	0.02140487	10.656
gnl|UG|Ssc#S23689697	red	958.36023	120.30653	0.9865149	0.001876	2.482
gnl|UG|Ssc#S23689787	yellow	181.30718	83.855239	0.8881884	0.04412073	35.788
gnl|UG|Ssc#S23689840	yellow	294.25073	125.81589	0.9817167	0.00295951	34.43
gnl|UG|Ssc#S23689938	pink	922.41256	148.65775	0.9982624	8.69E-05	8.516
gnl|UG|Ssc#S23690002	purple	218.59412	61.966309	0.9413316	0.01690756	31.89
gnl|UG|Ssc#S23690003	navy	138.09466	40.229868	0.9715908	0.00572351	16.012
gnl|UG|Ssc#S23690384	salmon	211.85509	68.405541	0.9710479	0.00588787	6.712
gnl|UG|Ssc#S23690475	brown	876.81658	115.81838	0.9542843	0.01165284	17.852
gnl|UG|Ssc#S23690683	black	713.5623	102.05622	0.9861496	0.00195264	17.61
gnl|UG|Ssc#S23690802	darkgrey	1011.6454	49.436287	0.9771188	0.00414052	22.296
gnl|UG|Ssc#S23690807	brown	439.42	89.178482	0.9021346	0.0362074	9.384
gnl|UG|Ssc#S23690967	green	770.81759	109.64655	0.9863499	0.0019105	20.15
gnl|UG|Ssc#S23691069	navy	857.93329	18.528427	-0.116761	0.85167352	27.26
gnl|UG|Ssc#S23691079	midnightblue	929.07851	77.829573	0.9968149	0.00021568	40.656
gnl|UG|Ssc#S23691136	darkturquoise	228.40167	41.792724	0.9707408	0.00598153	27.896
gnl|UG|Ssc#S23691157	turquoise	541.37748	59.396405	0.3025798	0.6207056	8.138
gnl|UG|Ssc#S23691372	grey60	296.97318	61.30422	0.9730397	0.00529246	26.014
gnl|UG|Ssc#S23691718	darkturquoise	174.62184	37.318787	0.9403159	0.01734581	46.236
gnl|UG|Ssc#S23691805	darkred	145.09072	35.846972	0.9361259	0.01919182	9.482
gnl|UG|Ssc#S23691900	pink	966.98102	143.89986	0.9913522	0.00096412	7.316
gnl|UG|Ssc#S23691927	magenta	412.40467	79.504277	0.9743809	0.00490348	5.262
gnl|UG|Ssc#S23692548	magenta	380.175	79.607168	0.9771871	0.00412205	6.262
gnl|UG|Ssc#S23692872	blue	879.26216	148.09012	0.992467	0.00078397	19.126
gnl|UG|Ssc#S23692986	yellow	309.55553	119.84716	0.9712165	0.00583668	11.616
gnl|UG|Ssc#S23693254	darkorange	81.827663	16.532595	0.7682174	0.12919562	21.344
gnl|UG|Ssc#S23693266	yellow	310.31918	125.3929	0.9818273	0.00293275	19.37
gnl|UG|Ssc#S23693320	lightgreen	525.24035	38.408138	0.9424997	0.01640807	9.728
gnl|UG|Ssc#S23693357	purple	264.29484	62.940731	0.942102	0.01657758	205.098
gnl|UG|Ssc#S23693410	blue	740.48678	143.47793	0.9864061	0.00189873	10.042
gnl|UG|Ssc#S23693441	turquoise	491.49538	59.636596	0.2372011	0.70084263	7.302
gnl|UG|Ssc#S23694531	green	813.71022	109.84252	0.9849597	0.00220922	17.318
gnl|UG|Ssc#S23694535	turquoise	347.77713	60.14568	-0.070051	0.91088078	5.57
gnl|UG|Ssc#S23694970	black	930.88715	95.079295	0.9694047	0.00639458	16.624
gnl|UG|Ssc#S23695084	brown	715.6268	127.86162	0.9789904	0.00364407	13.296
gnl|UG|Ssc#S23695102	tan	110.20738	32.824229	0.8734588	0.05299826	26.1
gnl|UG|Ssc#S23695121	grey	393.83816	14.366637	0.9796359	0.00347777	4.238
gnl|UG|Ssc#S23695134	red	901.00883	126.87972	0.9980789	0.00010105	4.036
gnl|UG|Ssc#S23695164	blue	745.42089	143.73628	0.9861063	0.0019618	3.688
gnl|UG|Ssc#S23695204	blue	882.02141	140.74084	0.9803352	0.00330051	35.044
gnl|UG|Ssc#S23695295	skyblue	472.0266	41.130205	0.9835555	0.00252517	10.712
gnl|UG|Ssc#S23695328	turquoise	982.89672	217.30842	0.9487322	0.01382716	9.52
gnl|UG|Ssc#S23695397	royalblue	627.26765	63.17668	0.9959515	0.00030904	6.256
gnl|UG|Ssc#S23695759	navy	241.09091	34.101055	0.9220084	0.02583796	22.42
gnl|UG|Ssc#S23695896	pink	983.21053	139.70417	0.9850687	0.00218526	55.714
gnl|UG|Ssc#S23696502	skyblue	570.61191	42.835213	0.9898788	0.00122045	22.116

gnl|UG|Ssc#S23696553	darkorange	98.985227	26.749754	0.9160114	0.02884803	10.78
gnl|UG|Ssc#S23696616	skyblue	544.19557	43.3845	0.9932916	0.00065891	55.488
gnl|UG|Ssc#S23696619	midnightblue	898.49935	78.586627	0.9988134	4.91E-05	10.564
gnl|UG|Ssc#S23696689	green	976.54818	106.18092	0.9750615	0.00470986	4.112
gnl|UG|Ssc#S23696700	brown	708.80682	135.51753	0.9957493	0.00033247	2.29
gnl|UG|Ssc#S23696826	skyblue	378.94275	39.067438	0.9717616	0.00567211	8.832
gnl|UG|Ssc#S23697216	darkgreen	207.56493	22.790329	0.7965939	0.1066999	64.26
gnl|UG|Ssc#S23697230	brown	189.52432	31.256023	0.6680706	0.21777081	23.756
gnl|UG|Ssc#S23697813	yellow	290.67833	133.33569	0.9969047	0.00020662	6.828
gnl|UG|Ssc#S23697932	orange	354.8827	34.534078	0.9787852	0.00369748	13.008
gnl|UG|Ssc#S23698058	brown	551.80114	116.19046	0.969078	0.00649698	6.43
gnl|UG|Ssc#S23698110	brown	533.6517	114.29002	0.965438	0.00767304	11.33
gnl|UG|Ssc#S23698716	navy	121.68511	38.503148	0.9406344	0.01720801	70.35
gnl|UG|Ssc#S23698735	darkorange	164.52508	31.915473	0.9773311	0.00408317	4.852
gnl|UG|Ssc#S23698752	lightgreen	473.41365	35.527024	0.946284	0.01482377	20.642
gnl|UG|Ssc#S23698821	salmon	242.03103	68.597977	0.9705469	0.0060409	9.064
gnl|UG|Ssc#S23699498	violet	851.02099	45.968008	0.9945124	0.00048758	1.586
gnl|UG|Ssc#S23699782	darkturquoise	184.31375	45.86773	0.9916758	0.00091056	0.116
gnl|UG|Ssc#S23699859	red	1010.9087	112.47799	0.9721454	0.00555721	7.148
gnl|UG|Ssc#S23699941	pink	842.34213	141.85028	0.9890012	0.0013824	3.172
gnl|UG|Ssc#S23700002	turquoise	187.69878	67.470532	0.6116617	0.27294409	133.324
gnl|UG|Ssc#S23700016	magenta	321.14915	75.431346	0.96798	0.00684494	5.844
gnl|UG|Ssc#S23700159	yellow	305.48539	124.18054	0.9815683	0.00299555	191.884
gnl|UG|Ssc#S23700422	purple	288.92792	79.053235	0.9962918	0.00027092	495.626
gnl|UG|Ssc#S23701109	green	956.62208	106.78775	0.9756966	0.00453152	24.404
gnl|UG|Ssc#S23701196	brown	721.43515	131.35033	0.9855117	0.00208889	18.978
gnl|UG|Ssc#S23701257	turquoise	1014.0038	202.19604	0.9162708	0.02871565	14.448
gnl|UG|Ssc#S23701295	darkgrey	991.98158	49.372098	0.9774052	0.0040632	3.606
gnl|UG|Ssc#S23701405	lightcyan	516.31651	56.946995	0.9764227	0.00433046	2.604
gnl|UG|Ssc#S23701516	yellow	308.03509	123.32676	0.976765	0.00423671	17.738
gnl|UG|Ssc#S23755332	pink	894.62975	149.62591	0.9996875	6.63E-06	3.53
gnl|UG|Ssc#S23755373	darkgreen	71.5464	12.413019	0.6289589	0.25567036	27.916
gnl|UG|Ssc#S23755435	navy	1009.868	17.623853	0.1093281	0.86107693	133.828
gnl|UG|Ssc#S23755492	red	894.62975	127.58416	0.9992509	2.46E-05	0.256
NA	royalblue	766.27394	58.879186	0.9785157	0.003768	14.334
NA	lightgreen	139.10809	16.529862	0.7098179	0.17925395	26.134
NA	skyblue	631.77222	39.698691	0.9718057	0.00565889	224.852
NA	blue	749.97243	148.74463	0.9948448	0.00044398	5.734
NA	salmon	199.15313	71.581842	0.9828712	0.00268415	10.964
NA	grey60	309.71183	67.443483	0.9939263	0.00056769	8.554
NA	grey60	308.86853	67.471052	0.993676	0.00060313	3.444
NA	turquoise	162.44962	60.104393	0.5696701	0.31607866	4.694
NA	green	406.6307	59.336477	0.8512153	0.06733385	80.644
NA	lightcyan	170.64364	29.682828	0.8067858	0.09894344	18.262
NA	pink	894.62975	149.62591	0.9996875	6.63E-06	1.862
NA	salmon	222.63524	76.720826	0.9965414	0.00024404	45.472
NA	grey60	304.62512	65.69234	0.9879067	0.00159353	16.582
NA	lightcyan	236.42875	45.284416	0.9263477	0.02372771	8.29

NA	blue	979.89826	139.84471	0.9766688	0.00426298	30.196
NA	darkturquoise	217.0021	40.082471	0.9566151	0.01077693	18.052
NA	brown	754.57407	134.25598	0.9912827	0.00097574	28.024
NA	blue	565.21515	114.49649	0.9340396	0.0201333	13.576
NA	blue	859.97336	144.8371	0.9863064	0.00191963	12.094
NA	turquoise	205.53205	82.40131	0.6172036	0.26737703	5.418
NA	blue	786.93816	126.37702	0.9543611	0.01162359	21.328
NA	royalblue	723.25415	58.947298	0.9795312	0.00350457	86.13
NA	brown	557.38066	115.74766	0.9684456	0.00669664	28.696
NA	purple	113.36514	21.153026	0.7245126	0.16621548	25.598
NA	turquoise	637.83992	164.92745	0.8813683	0.04816724	69.196
NA	green	820.0557	114.83188	0.9956411	0.00034523	11.214
NA	blue	430.14336	103.60212	0.9131944	0.03029829	9.676
NA	yellow	232.50395	104.70656	0.9419965	0.01662266	5.142
NA	violet	894.62975	47.034779	0.9933959	0.00064361	2.31
NA	yellow	225.95661	67.68163	0.8347634	0.07860047	84.39
NA	turquoise	344.52646	58.962477	-0.012563	0.98400426	21.354
NA	black	933.46708	100.4185	0.98153	0.00300488	22.586
NA	grey	389.51823	13.628545	0.9682422	0.00676131	17.298
NA	darkmagenta	104.0479	8.8136909	0.9181332	0.02777084	19.64
NA	purple	212.98938	55.451398	0.9178378	0.02792003	19.614
NA	black	767.90508	103.52557	0.9893181	0.00132315	14.188
NA	royalblue	592.53957	59.884322	0.9850879	0.00218106	8.178
NA	purple	289.73993	77.355764	0.9911819	0.0009927	24.35
NA	blue	991.14676	136.27093	0.9705466	0.006041	3.284
NA	red	887.26571	126.6702	0.9978162	0.00012247	2.934
NA	black	693.31689	99.866943	0.9818334	0.00293128	16.842
NA	grey60	300.34256	66.320773	0.9906455	0.00108457	9.758
NA	green	901.62364	112.61254	0.9891523	0.00135403	6.558
NA	turquoise	389.4946	134.32665	0.8232308	0.08681192	7.816
NA	yellow	165.28551	58.51015	0.8125876	0.09460832	37.442
NA	salmon	212.61751	76.026062	0.9949071	0.00043596	15.48
NA	paleturquoise	117.69939	15.88157	0.8777022	0.05038832	11.464
NA	darkgreen	245.05768	40.263937	0.9759429	0.00446299	106.794
NA	darkred	115.92128	18.883382	0.7545864	0.14044637	13.1
NA	blue	845.1703	131.47026	0.9644131	0.0080156	7.656
NA	darkmagenta	276.44679	5.101085	-0.790765	0.11121504	8.144
NA	purple	286.48421	79.792649	0.999068	3.42E-05	54.07
NA	pink	815.60818	135.95137	0.9803181	0.00330482	10.864
NA	grey60	267.82806	57.704483	0.9609242	0.00921792	55.208
NA	blue	384.5387	74.787178	0.8412025	0.07412667	16.25
NA	lightgreen	240.1735	27.217804	0.7836587	0.11679435	6.154
NA	black	835.81908	108.18616	0.9981739	9.37E-05	3.398
NA	blue	593.76879	94.290489	0.8890317	0.04362825	122.164
NA	greenyellow	183.53656	27.059908	0.8083516	0.09776766	93.678
NA	brown	702.10031	135.28045	0.9986694	5.83E-05	9.786
NA	tan	126.03538	41.130341	0.9495914	0.01348279	9.262
NA	lightgreen	481.12727	35.420494	0.8759268	0.05147526	1.6

NA	violet	954.57338	45.879023	0.9747481	0.00479871	2.91
NA	yellow	226.61981	69.970509	0.8425312	0.07321365	59.888
NA	red	632.07488	95.599819	0.9411733	0.01697564	7.014
NA	red	918.06453	127.13013	0.9983331	8.17E-05	5.78
NA	blue	925.31655	149.36801	0.9935228	0.00062517	2.884
NA	darkred	193.11641	45.083858	0.9822869	0.0028224	13.038
NA	blue	567.52854	118.81708	0.9445278	0.0155525	13.188
NA	darkorange	106.71612	20.839483	0.8439942	0.07221242	38.668
NA	paleturquoise	176.71667	25.233141	0.9659448	0.00750546	4.058
NA	midnightblue	841.49345	77.672885	0.9964414	0.0002547	13.906
NA	grey60	309.13087	64.670074	0.9848243	0.00223907	60.632
NA	grey60	294.20114	65.753252	0.9888441	0.0014121	54.24
NA	brown	81.36692	7.2756325	0.1224794	0.84444517	10.648
NA	greenyellow	176.27386	36.180399	0.9436521	0.01592009	15.172
NA	greenyellow	203.44374	37.64239	0.9325846	0.02079849	46.686
NA	violet	856.61238	46.538813	0.9965079	0.00024759	1.464
NA	salmon	223.08638	68.972158	0.9734953	0.00515921	62.25
NA	red	824.62724	120.82346	0.9883262	0.00151144	4.478
NA	pink	935.54945	148.30349	0.9977181	0.0001308	52.302
NA	darkgreen	188.14979	27.615092	0.8791806	0.04948886	28.388
NA	turquoise	994.41634	196.36589	0.906837	0.03365387	17.528
NA	red	894.62975	127.58416	0.9992509	2.46E-05	1.668
NA	darkgreen	268.48355	40.868459	0.9832073	0.00260567	128.134
NA	blue	549.70488	105.55331	0.9169082	0.02839113	4063.742
NA	green	903.27406	115.79258	0.996767	0.00022056	5.64
NA	paleturquoise	179.21049	24.748644	0.9654419	0.00767176	7.312
NA	turquoise	376.09511	59.178834	0.0320218	0.9592356	14.654
NA	tan	162.67189	38.205593	0.9352657	0.01957823	49.43
NA	brown	608.29229	128.20656	0.9896658	0.00125914	55.336
NA	magenta	317.48428	86.056201	0.9978989	0.00011558	15.24
NA	brown	509.0349	107.76199	0.9512695	0.01281842	10.63
NA	paleturquoise	179.50662	28.051103	0.9963797	0.00026135	27.522
NA	green	940.37491	111.26837	0.9861349	0.00195574	16.374
NA	darkturquoise	266.04167	33.207441	0.9135767	0.03010012	8.038
NA	purple	215.01103	52.99851	0.9037055	0.03534772	29.486
NA	darkred	202.46599	45.690679	0.9875371	0.00166705	24.554
NA	brown	449.25343	91.838873	0.909159	0.03241518	25.55
NA	purple	230.20985	64.795917	0.9513265	0.01279606	1781.64
NA	brown	511.55678	107.17688	0.9459394	0.01496587	26.208
NA	midnightblue	714.60482	70.350009	0.9757419	0.00451889	1.266
NA	royalblue	774.84676	60.560218	0.9853131	0.00213191	5.708
NA	yellow	285.13219	132.16491	0.995312	0.00038505	15.582
NA	green	870.15893	115.22654	0.996977	0.00019944	17.374
NA	white	999.38734	39.470029	0.9821979	0.00284366	11.544
NA	greenyellow	120.13188	20.985183	0.8204012	0.08886461	62.89
NA	blue	587.27493	85.903768	0.870695	0.05472032	30.95
NA	white	965.3902	47.070545	0.9898017	0.00123441	5.528
NA	red	926.13641	126.19481	0.9968524	0.00021189	4.082

NA	magenta	273.39982	83.912165	0.9934017	0.00064277	15.848
NA	magenta	390.42949	76.057141	0.9657993	0.00755345	7.274
NA	darkgreen	122.11823	17.402649	0.7452958	0.14827123	13.99
NA	lightgreen	343.44456	37.65756	0.9297578	0.02211064	51.804
NA	brown	784.08839	123.96262	0.9686495	0.00663206	7.338
NA	turquoise	848.98349	202.52133	0.9333751	0.02043622	3.138
NA	turquoise	547.08882	58.31449	0.3060211	0.61653184	4.276
NA	yellow	244.97512	100.11021	0.9281143	0.02288541	16.132
NA	royalblue	433.54544	54.599329	0.9623398	0.00872346	6.778
NA	turquoise	218.89256	82.130809	0.6742209	0.21196567	5.032
NA	turquoise	357.27677	53.427905	0.0430038	0.9452627	2.21
NA	red	830.50899	122.70696	0.9913727	0.0009607	12
NA	lightyellow	344.76281	54.206922	0.9627354	0.00858689	2.944
NA	turquoise	608.36236	152.58861	0.8443571	0.07196467	2.962
NA	lightgreen	227.84292	27.471195	0.7876874	0.11362104	4.882
NA	pink	920.92757	148.82378	0.9984916	7.03E-05	2.898
NA	white	248.16822	17.051746	-0.629188	0.25544392	114.796
NA	red	854.43061	125.46074	0.9958826	0.00031695	4.39
NA	pink	952.93167	147.17569	0.9960802	0.00029442	11.766
NA	pink	931.50851	148.88256	0.9985359	6.72E-05	17.44
NA	blue	493.83597	106.855	0.9227642	0.02546628	6.634
NA	darkgrey	919.80096	48.564704	0.9750154	0.00472289	143.694
NA	pink	847.89892	147.69553	0.9971073	0.00018668	5.272
NA	violet	923.64711	46.82666	0.9861197	0.00195895	2.878
NA	white	272.60523	17.112746	-0.631549	0.25311013	20.752
NA	navy	900.1079	19.180737	0.0995576	0.8734491	4.156
NA	red	910.76223	124.33722	0.9936804	0.0006025	4.284
NA	violet	920.27497	47.035738	0.9897094	0.00125119	10.262
NA	darkturquoise	100.41519	8.7870684	0.6281714	0.25645023	22.236
NA	brown	751.78263	134.54855	0.9953054	0.00038586	1.296
NA	turquoise	551.51748	171.88429	0.9271978	0.02332115	31.774
NA	navy	56.553169	17.905553	0.7681575	0.12924442	158.896
NA	violet	55.98216	6.9224373	-0.913231	0.03027949	11.116
NA	red	694.03523	108.23619	0.9658153	0.00754818	3.604
NA	violet	820.15906	44.919288	0.9900272	0.00119374	9.192
NA	lightyellow	449.30568	62.905512	0.9944669	0.00049365	36.128
NA	red	887.63265	126.7277	0.9979082	0.00011481	2.998
NA	pink	894.62975	149.62591	0.9996875	6.63E-06	1.604
NA	pink	893.13252	149.63556	0.9997119	5.87E-06	22.29
NA	brown	403.63363	71.584904	0.8591616	0.06208991	78.162
NA	red	907.4193	126.70647	0.9977028	0.00013213	5.376
NA	red	898.47308	123.82367	0.9930188	0.00069948	1.854
NA	white	930.25147	47.427691	0.9814208	0.00303153	17.78
NA	pink	936.72945	149.0119	0.9987016	5.62E-05	88.628
NA	violet	767.06956	43.242158	0.9928072	0.0007315	2.508
NA	darkorange	194.04048	23.294011	0.8880723	0.04418867	10.708
NA	darkgreen	142.81333	17.146294	0.7329825	0.15883074	32.234
NA	salmon	220.14941	75.086978	0.9920884	0.00084375	9.996

NA	green	708.04687	99.88509	0.9660351	0.00747573	33.678
NA	yellow	309.57859	129.09306	0.9881533	0.0015451	18.932
NA	yellow	218.65691	99.707947	0.9324243	0.0208722	14.254
NA	navy	925.35654	19.201661	-0.024925	0.96826826	14.18
NA	orange	93.748733	21.589274	0.8138004	0.09370962	945.186
NA	lightyellow	428.99042	59.425556	0.982312	0.00281641	169.246
NA	red	883.66829	126.09424	0.996891	0.000208	2.484
NA	red	896.47829	126.92136	0.9980977	9.96E-05	9.07
NA	darkgrey	972.97383	52.050247	0.9887638	0.00142734	6.38
NA	white	820.04062	44.260468	0.9594205	0.00975286	7.292
NA	salmon	220.07039	76.758292	0.996812	0.00021598	1.438
NA	skyblue	421.3587	42.616749	0.9903973	0.00112798	6.698
NA	blue	957.93826	138.20645	0.9747424	0.00480032	22.134
NA	greenyellow	100.57591	15.001332	0.704001	0.1844924	17.216
NA	magenta	174.655	61.038145	0.9205137	0.02657821	1.286
NA	turquoise	481.53771	157.42327	0.9043072	0.03502019	10.34
NA	green	659.13795	102.34997	0.9713885	0.0057846	2.29
NA	pink	901.63362	149.45743	0.9994345	1.61E-05	5.644
NA	red	864.12154	126.37022	0.9973916	0.00015986	91.158
NA	pink	894.62975	149.62591	0.9996875	6.63E-06	1.604
NA	violet	761.49259	43.583285	0.9972912	0.00016917	9.682
NA	darkgrey	1019.3023	48.797429	0.9742262	0.00494783	9.17
NA	salmon	227.30199	70.78213	0.9792279	0.0035826	20.71
NA	white	946.87234	47.332975	0.9863398	0.00191262	109.294
NA	navy	885.08609	19.077057	-0.051533	0.93441577	2.55
NA	turquoise	893.81652	221.94815	0.9786876	0.00372296	4.464
NA	grey60	312.4841	67.593958	0.9940828	0.00054591	16.74
NA	red	821.89563	122.19143	0.9906044	0.00109172	2.718
NA	greenyellow	215.45433	35.529882	0.9785879	0.00374906	44.114
NA	darkorange	118.97893	26.941915	0.9195838	0.02704209	8.864
NA	white	968.91131	41.946883	0.9758996	0.00447501	12.08
NA	darkgrey	896.30265	50.415795	0.9831256	0.00262466	7.804
NA	tan	77.398885	20.314467	0.7009717	0.18723747	51.822
NA	turquoise	245.58554	85.710669	0.6736369	0.21251503	23.188
NA	orange	339.13224	21.116941	0.8232797	0.08677656	31.862
NA	darkgrey	851.82461	49.421129	0.9793862	0.0035418	4.822
NA	pink	925.31556	148.58008	0.9981493	9.55E-05	3.646
NA	white	909.34792	47.251474	0.9762923	0.00436633	14.064
NA	white	978.54476	46.593548	0.990087	0.00118303	2.904
NA	tan	157.05228	38.659186	0.9325513	0.02081377	10.368
NA	midnightblue	840.81798	72.238333	0.9809136	0.00315625	11.038
NA	greenyellow	117.0297	7.226886	0.3759454	0.53285894	26.566
NA	turquoise	249.67242	90.464272	0.7091018	0.17989651	4.736
NA	pink	924.99809	148.49586	0.9980195	0.00010577	4.96
NA	red	904.56782	124.8292	0.994564	0.00048073	21.452
NA	darkred	129.08954	16.666057	0.6981958	0.18976298	41.21
NA	salmon	231.01987	74.542994	0.989915	0.00121392	4.072
NA	salmon	217.23128	72.359451	0.983438	0.00255225	36.902

NA	red	891.86133	127.11867	0.9985299	6.76E-05	13.744
NA	white	881.45287	46.965672	0.9701858	0.00615201	12.388
NA	red	893.75392	127.04235	0.9983214	8.25E-05	2.43
NA	purple	307.92752	74.726227	0.9823047	0.00281814	7.252
NA	darkgreen	255.53793	40.381424	0.9764787	0.00431506	21.732
NA	turquoise	1019.1965	219.45189	0.9500487	0.01330066	1.424
NA	purple	248.36007	59.424596	0.9302404	0.02188479	6.106
NA	red	900.90829	125.25432	0.9953975	0.00037457	2.304
NA	pink	938.82636	146.68924	0.9954264	0.00037104	1.614
NA	darkred	196.3803	44.600889	0.9768424	0.00421562	7.95
NA	pink	932.70744	149.20199	0.9989862	3.87E-05	5.738
NA	pink	887.34141	140.85007	0.9873252	0.00170968	7.674
NA	darkorange	173.65034	29.40698	0.9530288	0.01213382	13.732
NA	turquoise	992.49952	222.40725	0.9617557	0.0089264	7.082
NA	red	872.48026	126.10953	0.9969642	0.0002007	6.71
NA	pink	601.56736	116.48209	0.9487617	0.0138153	9.964
NA	skyblue	329.39724	34.818877	0.9478862	0.01416896	5.014
NA	lightyellow	440.42938	62.242565	0.9919541	0.0008653	8.918
NA	pink	947.07705	147.75757	0.996896	0.00020749	23.78
NA	greenyellow	120.61072	26.170053	0.8416564	0.07381434	62.456
NA	turquoise	476.68466	141.90548	0.8616804	0.06045566	1.942
NA	lightyellow	654.20575	54.278459	0.9587597	0.00999105	1.906
NA	green	963.79466	105.47241	0.9727746	0.00537049	4.116
NA	lightyellow	294.43903	52.611093	0.9535858	0.01191964	54.518
NA	lightyellow	492.98782	61.853944	0.9901393	0.00117368	8.852
NA	black	865.154	96.864412	0.974206	0.00495363	23.946
NA	pink	858.81047	147.49	0.9968129	0.00021589	1.528
NA	turquoise	804.77155	196.53365	0.9321083	0.02101776	24.844
NA	magenta	200.29539	65.838336	0.9382754	0.01823722	45.068
NA	skyblue	644.28079	38.782611	0.9664536	0.00733845	4.712
NA	darkmagenta	75.659263	8.3814492	0.9745445	0.0048567	36.676
NA	turquoise	232.93739	93.608564	0.6823332	0.20437604	6.75
NA	paleturquoise	159.10879	21.444261	0.9239282	0.02489719	7.934
NA	green	462.73704	77.168971	0.9075528	0.03327046	4.102
NA	tan	103.28683	30.350976	0.8312876	0.08104882	6.102
NA	salmon	192.34758	69.544475	0.9764235	0.00433022	2.558
NA	red	948.99039	124.03892	0.992948	0.00071013	2.458
NA	green	927.76401	109.60654	0.9820389	0.00288177	19.498
NA	skyblue	468.12598	43.575738	0.995409	0.00037316	10.512
NA	navy	65.435955	30.909452	0.8910255	0.04247093	2.426
NA	yellow	243.04428	105.4319	0.9416066	0.01678954	71.006
NA	greenyellow	191.85181	39.34881	0.9746716	0.00482047	54.36
NA	turquoise	1006.5838	196.86198	0.9084407	0.03279677	11.294
NA	turquoise	724.73348	178.64122	0.9033541	0.03553944	48.366
NA	royalblue	649.01002	62.204503	0.9927793	0.00073575	13.446
NA	blue	849.89253	135.95645	0.9728485	0.00534868	2.154
NA	turquoise	878.80999	210.28151	0.9541499	0.011704	4.31
NA	lightyellow	733.55852	46.174223	0.9217625	0.02595931	16.854

NA	grey60	315.13567	68.030583	0.9957497	0.00033242	23.316
NA	turquoise	126.90573	42.939322	0.517943	0.37133746	9.376
NA	violet	48.032032	6.5827922	-0.848577	0.06910383	135.298
NA	turquoise	748.96387	202.64131	0.9546733	0.01150509	20.256
NA	blue	803.25747	150.52713	0.9966618	0.00023141	248.594
NA	white	777.11628	43.457853	0.9385549	0.01811429	55.664
NA	purple	300.61647	76.231509	0.9864842	0.00188241	5564.096
NA	red	962.68352	120.57294	0.987066	0.00176233	7.658
NA	red	928.58718	126.90616	0.9979097	0.00011469	2.192
NA	darkgrey	1010.4448	50.131992	0.9801024	0.00335919	7.292
NA	green	555.5389	90.998581	0.9446799	0.01548891	15.186
NA	midnightblue	906.91414	78.518261	0.9985761	6.45E-05	8.258
NA	blue	774.25186	150.28862	0.9967363	0.00022371	1.054
NA	black	723.13359	104.77168	0.9917342	0.000901	1.314
NA	turquoise	187.66109	68.655504	0.5296572	0.35863241	6.866
NA	salmon	238.76728	74.824833	0.9904648	0.0011161	15.874
NA	green	420.5468	61.370134	0.859458	0.06189688	34.642
NA	turquoise	288.72614	59.292776	-0.153998	0.80470126	6.834
NA	midnightblue	930.31718	78.16033	0.997555	0.00014508	84.47
NA	turquoise	841.60799	217.99947	0.9735283	0.00514962	50.454
NA	magenta	242.48969	66.210196	0.9418521	0.01668437	5.22
NA	blue	710.03683	124.75583	0.954724	0.0114859	77.784
NA	green	891.04481	111.2913	0.9864632	0.00188678	9.85
NA	magenta	288.42251	86.078526	0.9988542	4.65E-05	6.77
NA	midnightblue	854.33552	62.704055	0.951212	0.01284103	10.446
NA	darkturquoise	110.02695	12.236331	0.6936649	0.19390564	93.812
NA	lightgreen	482.24329	35.363906	0.8752885	0.05186779	2.466
NA	lightyellow	562.46942	59.000694	0.9785901	0.00374848	20.372
NA	yellow	244.6839	114.89404	0.9633704	0.00836916	15.048
NA	orange	191.86713	35.666807	0.9729914	0.00530665	0.138
NA	pink	906.47851	149.81623	0.9999213	8.38E-07	8.908
NA	black	771.43826	98.933758	0.9799159	0.00340644	5.19
NA	pink	894.97665	145.04011	0.9933798	0.00064597	1.598
NA	white	874.46912	46.853802	0.967791	0.00690544	2.362
NA	red	945.70932	124.77927	0.9942261	0.00052622	2.002
NA	darkgreen	79.656867	15.374273	0.6868584	0.20017623	22.504
NA	green	881.57829	109.37489	0.9822442	0.00283259	6.004
NA	brown	365.00108	69.091621	0.85218	0.06669019	16.084
NA	turquoise	671.49832	159.55824	0.8801355	0.0489106	12.488
NA	magenta	137.93907	49.908063	0.8799184	0.04904187	20.26
NA	royalblue	789.63978	56.206146	0.9678763	0.00687811	8.28
NA	navy	81.79352	21.84905	0.8650212	0.0583093	5.312
NA	darkgrey	807.1015	49.288218	0.9784257	0.00379165	3.276
NA	darkturquoise	177.76087	44.924643	0.9863085	0.00191918	77.44
NA	turquoise	290.03727	107.64913	0.7677678	0.12956219	5.808
NA	pink	919.26378	149.69822	0.9997261	5.44E-06	14.578
NA	darkgrey	896.01391	52.854806	0.9927943	0.00073347	3.194
NA	green	975.58626	101.44134	0.9637978	0.00822364	38.302

NA	magenta	168.36889	67.374907	0.939643	0.01763818	59.888
NA	midnightblue	881.44427	77.525242	0.9959326	0.00031121	10.198
NA	orange	201.43589	34.616505	0.9763096	0.00436158	16.91
NA	navy	163.76366	28.452984	0.926249	0.0237751	6.61
NA	navy	64.033081	22.29852	0.7728977	0.12539747	2.41
NA	violet	42.048406	5.2373587	-0.737568	0.15487374	207.372
NA	turquoise	162.49753	37.077627	-0.497181	0.39411343	7.61
NA	lightcyan	279.4729	42.009512	0.9202677	0.02670063	14.164
NA	violet	907.85743	46.743534	0.9902659	0.00115117	2.882
NA	darkgreen	167.13868	32.160752	0.9083526	0.03284367	4.812
NA	salmon	239.78498	68.198552	0.9702396	0.00613541	30.376
NA	white	904.46156	47.043623	0.9773629	0.00407459	11.89
NA	yellow	213.99621	97.493064	0.9230685	0.02531708	19.1
NA	red	488.76196	79.139854	0.9038821	0.03525151	23.314
NA	tan	82.952049	14.956011	0.6609774	0.22451961	1.566
NA	tan	145.68842	39.683952	0.9353413	0.01954414	8.148
NA	darkgrey	924.54371	51.698078	0.9882559	0.00152509	3.406
NA	magenta	184.04395	62.8977	0.9274855	0.02318408	317.736
NA	lightcyan	597.73808	51.876661	0.9451505	0.0152928	13.382
NA	red	927.30232	126.22576	0.9968445	0.00021268	3.946
NA	skyblue	314.09912	34.854221	0.9444603	0.01558072	11.978
NA	lightyellow	414.17632	62.944837	0.9950088	0.00042298	9.028
NA	turquoise	479.85704	141.21399	0.8284414	0.08307079	8.22
NA	lightgreen	566.90684	37.445957	0.9317195	0.0211973	6.166
NA	lightcyan	474.86805	58.929251	0.9880669	0.00156202	58.654
NA	orange	367.85662	32.187872	0.9598279	0.00960697	6.814
NA	red	763.09945	116.25359	0.9802857	0.00331296	2.64
NA	magenta	197.86228	56.522227	0.9112902	0.03129152	6.606
NA	brown	914.16449	105.12901	0.9304686	0.02177824	18.444
NA	white	956.49765	47.42007	0.9871417	0.00174691	6.274
NA	grey60	295.1945	60.961512	0.971883	0.00563569	40.804
NA	pink	894.62975	149.62591	0.9996875	6.63E-06	1.668
NA	skyblue	407.3083	40.527168	0.9802818	0.00331394	8.862
NA	skyblue	262.9467	28.91035	0.9026613	0.03591842	82.858
NA	violet	872.9933	46.914952	0.996199	0.00028114	2.234
NA	purple	252.29834	59.044346	0.9297527	0.02211304	17.926
NA	darkgreen	184.68507	34.565544	0.9290758	0.02243107	143.366
NA	red	879.02141	125.73441	0.9963087	0.00026907	4.202
NA	yellow	312.04684	124.15569	0.9813429	0.00305057	11.194
NA	magenta	256.15843	77.341852	0.9754933	0.00458838	128.41
NA	salmon	214.31108	75.780852	0.9941264	0.0005399	21.34
NA	purple	239.32818	68.706759	0.9663074	0.00738631	27.248
NA	blue	635.33834	103.68785	0.9120355	0.03090154	116.238
NA	pink	760.51495	137.27875	0.9821554	0.00285383	3.87
NA	grey60	312.84082	67.380217	0.9933802	0.00064591	12.136
NA	purple	251.45793	66.176154	0.9584887	0.01008929	13.638
NA	pink	894.62975	149.62591	0.9996875	6.63E-06	1.476
NA	violet	875.6729	46.878497	0.9965541	0.0002427	6.074

NA	red	923.51728	126.94426	0.9979985	0.00010746	5.948
NA	red	823.65106	120.00775	0.9868408	0.00180851	3.838
NA	pink	940.92135	148.78547	0.9983712	7.89E-05	13.004
NA	purple	292.52214	74.456029	0.9822044	0.0028421	188.322
NA	purple	297.62453	78.23107	0.992864	0.00072285	8.46
NA	grey	370.21007	14.786319	0.9843391	0.00234712	15.734
NA	tan	297.77796	34.488274	0.872576	0.05354644	40.194
NA	red	882.95563	125.97811	0.9967038	0.00022706	2.42
NA	red	901.29773	125.6264	0.9960129	0.00030204	2.496
NA	orange	97.224104	14.207022	0.7480443	0.1459434	15.022
NA	salmon	220.07039	76.758292	0.996812	0.00021598	1.492
NA	purple	297.25222	78.978198	0.9954541	0.00036767	25.656
NA	darkgreen	242.81174	43.382657	0.9969414	0.00020296	13.35
NA	violet	43.091144	9.5191844	-0.989812	0.00123261	56.93
NA	green	837.56195	115.76443	0.9982643	8.68E-05	12.534
NA	brown	456.70929	93.655182	0.9210623	0.02630572	22.168
NA	blue	965.43968	144.49948	0.984873	0.00222831	3.258
NA	green	906.65673	112.33859	0.9884088	0.00149545	13.234
NA	tan	134.40146	39.582384	0.9418338	0.01669221	26.542
NA	white	262.10493	15.523331	-0.686759	0.20026816	20.112
NA	royalblue	565.4824	61.336115	0.9897138	0.00125039	9.338
NA	turquoise	812.4552	207.93588	0.9633117	0.00838919	1.444
NA	black	610.21532	92.022854	0.964506	0.00798436	9.19
NA	yellow	227.39911	105.46134	0.9448779	0.0154063	11.536
NA	red	1003.8475	115.06592	0.976826	0.00422007	6.858
NA	grey60	310.98971	68.437112	0.9969637	0.00020075	20.81
NA	turquoise	306.40314	59.943548	-0.104433	0.86727425	5.276
NA	green	905.02013	110.17556	0.9835991	0.00251515	28.712
NA	brown	489.98468	102.7989	0.9433302	0.01605591	36.346
NA	royalblue	564.93333	57.557311	0.9770776	0.00415169	9.456
NA	lightcyan	468.3286	58.935336	0.9890094	0.00138085	4.336
NA	magenta	356.96053	74.974901	0.9648551	0.00786726	2.308
NA	darkgrey	1001.9119	50.68619	0.9825679	0.00275563	21.4
NA	turquoise	909.78666	221.29207	0.9660895	0.00745784	10.32
NA	lightyellow	624.2223	58.289818	0.9760518	0.00443278	3.32
NA	red	924.30271	126.63432	0.9975533	0.00014523	9.862
NA	blue	941.39595	148.46593	0.991591	0.00092449	5.584
NA	grey60	307.41653	66.889187	0.9919531	0.00086547	84.538
NA	navy	842.75173	17.953253	0.0385861	0.95088278	6.934
NA	lightyellow	703.37516	49.989801	0.9402702	0.01736562	34.484
NA	violet	833.80033	45.832289	0.9944487	0.0004961	4.418
NA	tan	161.90575	32.691321	0.8675619	0.05669334	49.954
NA	red	609.83584	97.438444	0.9442675	0.01566147	7.042
NA	darkgrey	860.83227	50.149917	0.9818819	0.00291956	4.182
NA	brown	759.91707	124.02037	0.9689506	0.00653702	7.408
NA	purple	228.80246	64.062878	0.9481013	0.01408182	87.934
NA	midnightblue	989.4866	75.196783	0.9891963	0.00134582	10.908
NA	navy	899.0255	19.122883	0.1090879	0.86138094	1.918

NA	red	941.82462	122.96712	0.9911668	0.00099526	3.16
NA	turquoise	1011.7613	219.83711	0.9529517	0.01216357	17.132
NA	tan	134.5823	34.406945	0.8985034	0.03821962	13.002
NA	black	533.74306	82.775534	0.940928	0.01708129	9.834
NA	yellow	278.54702	116.67485	0.9672089	0.00709284	8.408
NA	magenta	262.0199	81.333868	0.9865889	0.00186061	3.772
NA	darkred	151.7718	37.329724	0.9361297	0.01919009	28.864
NA	green	383.9182	49.842836	0.8161615	0.09196757	48.102
NA	turquoise	966.03333	198.53392	0.9140418	0.02985963	2.156
NA	navy	356.80566	20.65141	0.8174086	0.09105143	170.824
NA	magenta	467.35346	67.637735	0.9327673	0.02071457	28.814
NA	turquoise	278.88877	56.653792	-0.141017	0.82104857	41.872
NA	midnightblue	800.44246	75.876815	0.9915024	0.00093913	52.236
NA	turquoise	561.97538	58.51523	0.3131442	0.607908	1.868
NA	salmon	200.34522	66.919184	0.9675592	0.00697987	46.294
NA	turquoise	912.94718	226.89616	0.9841947	0.00237961	3.782
NA	green	459.49285	76.660633	0.9061509	0.03402271	3.592
NA	pink	926.89683	148.31671	0.9977622	0.00012704	2.32
NA	blue	838.21376	142.01455	0.9827017	0.00272401	5.902
NA	lightcyan	691.98182	44.932331	0.8993904	0.03772486	9.178
NA	blue	977.83946	140.14455	0.9774617	0.00404802	11.66
NA	black	773.99751	107.30861	0.9967557	0.00022172	12.236
NA	black	934.89608	100.3213	0.9814087	0.00303447	10.494
NA	turquoise	434.83349	134.94477	0.8315927	0.08083293	3.854
NA	turquoise	361.96894	61.121267	-0.034087	0.9566074	13.438
NA	turquoise	915.21294	199.20673	0.9297292	0.02212404	0.72
NA	turquoise	261.21463	57.084552	-0.223621	0.71766761	8.336
NA	lightyellow	390.62857	62.48525	0.9928995	0.00071747	137.426
NA	magenta	213.74331	78.136426	0.9759917	0.00444944	2.582
NA	lightgreen	301.09245	33.567949	0.8908093	0.04259593	7.59
NA	magenta	228.3112	77.400658	0.9746646	0.00482246	21.122
NA	grey	497.22474	15.399421	0.9932069	0.00067142	7.612
NA	magenta	227.6126	81.192243	0.985094	0.00217974	12.136
NA	black	611.94874	89.994516	0.9593338	0.00978401	3.58
NA	midnightblue	934.17969	77.270923	0.9953208	0.00038396	1.66
NA	royalblue	481.13334	57.168493	0.9733049	0.00521475	6.848
NA	orange	327.84163	35.246024	0.9819531	0.00290241	4.516
NA	royalblue	749.52685	60.496346	0.9848342	0.00223688	4.198
NA	lightcyan	556.45092	53.602082	0.9571839	0.0105666	18.222
NA	lightyellow	354.1048	55.985362	0.9685427	0.00666585	6.17
NA	darkgreen	105.99915	21.479367	0.7857401	0.11515159	109.16
NA	darkgrey	528.01864	33.149631	0.8962905	0.03946278	2.696
NA	royalblue	650.79945	63.694772	0.9974315	0.00015621	12.11
NA	white	273.13458	16.681582	-0.557377	0.32900911	3.188
NA	yellow	303.03486	117.19265	0.9662687	0.00739901	9.016
NA	turquoise	735.63073	189.07344	0.9409682	0.01706394	38.34
NA	turquoise	581.27795	58.097894	0.3353454	0.58116844	13.8
NA	turquoise	973.47697	197.20097	0.9088871	0.03255946	13.356

NA	navy	64.648586	9.012268	0.496525	0.39483835	7.82
NA	brown	863.68919	119.31825	0.9634047	0.00835744	16.304
NA	darkred	175.3161	40.778783	0.9584551	0.01010151	11.142
NA	pink	668.55064	125.48744	0.9637702	0.00823302	2.966
NA	turquoise	1015.1454	218.07594	0.9474656	0.01433995	13.076
NA	pink	924.96131	149.26805	0.9991038	3.22E-05	27.704
NA	navy	85.081516	35.239683	0.9146489	0.02954663	91.238
NA	yellow	216.36047	100.72939	0.9317023	0.02120523	79.704
NA	yellow	298.21749	122.62143	0.9783122	0.00382156	1144.25
NA	orange	167.14358	24.913612	0.9105337	0.0316889	36.722
NA	turquoise	901.70303	211.67236	0.9513776	0.01277602	16.744
NA	turquoise	993.28854	218.67793	0.9523679	0.01238955	23.764
NA	tan	152.90422	47.842223	0.9972242	0.00017548	14.86
NA	red	843.63929	123.67426	0.9930373	0.0006967	1.952
NA	navy	71.027515	18.308756	0.7203204	0.16990622	1.976
NA	red	927.51955	126.28991	0.9968763	0.00020948	3.088
NA	yellow	284.97166	127.32404	0.9867332	0.0018307	21.582
NA	yellow	307.26268	122.71742	0.9760711	0.00442745	289.064
NA	purple	262.95051	66.437202	0.9559245	0.01103412	16.632
NA	yellow	259.89818	121.75938	0.9768124	0.00422377	10.302
NA	paleturquoise	163.1452	25.070426	0.9742356	0.00494514	6.334
NA	midnightblue	724.74121	71.670187	0.9797636	0.00344518	6.724
NA	tan	191.88662	44.157078	0.9728248	0.00535569	18.056
NA	darkred	180.16217	38.144974	0.9424317	0.01643699	9.95
NA	pink	911.58594	147.51768	0.9967785	0.00021939	4.748
NA	yellow	228.95343	84.334366	0.8903253	0.04287627	9.948
NA	purple	202.03866	46.60251	0.880033	0.04897254	32.198
NA	darkgreen	241.03536	42.697063	0.990837	0.00105146	10.076
NA	pink	894.62975	149.62591	0.9996875	6.63E-06	1.796
NA	midnightblue	844.80172	76.676535	0.9938349	0.00058055	43.566
NA	darkturquoise	135.73169	11.151749	0.663426	0.22218342	63.024
NA	orange	111.05115	23.698068	0.8605972	0.06115682	41.178
NA	navy	79.028479	36.935502	0.9659193	0.00751388	9.8
NA	purple	296.48306	79.541143	0.9970894	0.00018841	29.262
NA	darkgrey	847.14549	51.00155	0.9855911	0.00207176	5.746
NA	skyblue	411.94146	41.319168	0.9838836	0.00245009	5.552
NA	pink	894.62975	149.62591	0.9996875	6.63E-06	0.514
NA	purple	291.85782	80.220632	0.9997342	5.20E-06	36.304
NA	brown	472.39421	98.183992	0.9321848	0.02098246	94.19
NA	darkred	204.17818	46.471892	0.9898081	0.00123326	14.124
NA	darkturquoise	143.93032	34.945606	0.9287831	0.02256908	12.932
NA	turquoise	919.69324	215.70237	0.9562505	0.01091247	5.722
NA	red	990.78034	116.62991	0.97972	0.00345629	32.846
NA	turquoise	880.25415	203.29902	0.942816	0.01627363	167.232
NA	purple	299.63542	74.554212	0.9822803	0.00282399	11.506
NA	tan	90.694908	16.313075	0.6564606	0.22884656	4.19
NA	white	772.74025	43.546354	0.9350585	0.01967164	3.24
NA	green	545.30461	88.528485	0.9384668	0.01815301	22.844

NA	white	844.15548	45.714347	0.9563423	0.01087829	5.532
NA	midnightblue	965.481	76.649021	0.9932815	0.0006604	10.702
NA	purple	279.35616	74.860664	0.9827121	0.00272155	94.166
NA	yellow	301.19829	132.49539	0.9948198	0.00044721	24.334
NA	purple	168.61011	44.360767	0.8705281	0.05482492	5.518
NA	turquoise	190.70089	70.880218	0.6750276	0.21120747	9.686
NA	pink	908.35001	149.01511	0.9988025	4.97E-05	15.058
NA	white	844.72532	46.265014	0.9573497	0.01050556	12.968
NA	red	912.24855	125.77878	0.9962073	0.00028023	6.652
NA	pink	936.8088	147.98584	0.9972694	0.00017122	4.94
NA	violet	901.61103	46.463434	0.986457	0.00188808	9.364
NA	blue	973.39319	137.07606	0.9725209	0.00544552	78.76
NA	red	941.77689	125.88929	0.9961979	0.00028127	4.64
NA	navy	822.14715	18.304996	0.0888475	0.88702481	1.7
NA	white	958.87065	47.175026	0.9863265	0.00191541	5.444
NA	turquoise	998.42758	199.10187	0.9112019	0.03133781	6.674
NA	lightyellow	242.77677	45.625878	0.9221722	0.02575727	3.474
NA	green	775.74328	112.8981	0.9927739	0.00073658	7.086
NA	midnightblue	742.75904	71.26362	0.9786708	0.00372735	5.922
NA	turquoise	1007.6634	214.44862	0.9399333	0.01751188	5.19
NA	magenta	467.69889	66.446495	0.9297934	0.02209395	15.272
NA	black	685.88474	101.54341	0.9849587	0.00220942	5.222
NA	grey60	300.61057	67.192842	0.9932305	0.00066793	96.436
NA	green	685.77857	105.34623	0.9781862	0.00385482	8.334
NA	turquoise	844.47957	192.45749	0.920393	0.02663823	29.536
NA	yellow	165.52104	63.783094	0.8307772	0.08141029	64.968
NA	darkturquoise	237.56578	37.925781	0.9451498	0.0152931	2.37
NA	lightgreen	228.85746	28.955755	0.8265339	0.08443447	2090.904
NA	darkred	186.94655	39.919374	0.9442857	0.01565385	48.878
NA	purple	281.02007	77.971828	0.9944328	0.00049823	20.676
NA	yellow	315.45449	123.63144	0.979667	0.00346982	3.702
NA	yellow	206.61984	91.887041	0.9141338	0.02981214	4.198
NA	green	657.05859	90.83888	0.9461191	0.0148917	12.444
NA	brown	681.11055	121.24511	0.9657773	0.00756073	19.704
NA	purple	310.56938	72.551456	0.9757491	0.00451689	15.91
NA	turquoise	917.20001	202.09565	0.9305008	0.02176322	19.398
NA	lightcyan	393.37358	48.547693	0.951611	0.01268458	26.696
NA	yellow	267.6956	116.73898	0.9678708	0.00687986	13.466
NA	purple	281.38169	69.617902	0.9658834	0.00752571	5.994
NA	darkorange	144.94786	26.86066	0.9193357	0.0271663	1027.686
NA	pink	1015.5611	127.98761	0.9667308	0.007248	19.756
NA	greenyellow	131.95206	18.522522	0.6830771	0.20368388	22.962
NA	violet	948.12465	45.809579	0.9749051	0.00475414	7.738
NA	purple	299.26087	77.700331	0.9912329	0.00098412	8.294
NA	salmon	236.00964	72.673057	0.9835513	0.00252614	6.776
NA	blue	635.037	132.63071	0.9687475	0.00660108	3.27
NA	turquoise	652.4906	176.07863	0.9147702	0.02948419	467.066
NA	violet	46.941427	7.8264859	-0.91697	0.02835951	6.314

NA	salmon	230.36304	74.947263	0.9914256	0.00095188	7.74
NA	violet	939.64972	46.42534	0.9812027	0.00308495	5.986
NA	turquoise	863.03351	218.2866	0.9688688	0.0065628	27.338
NA	darkturquoise	156.36007	36.04521	0.9309408	0.02155834	52.652
NA	darkgreen	196.32309	28.685876	0.8886653	0.04384201	409.45
NA	turquoise	881.18851	223.96742	0.9831972	0.002608	8.472
NA	violet	875.90554	46.727464	0.9941062	0.00054267	16.628
NA	blue	639.42955	135.56246	0.9736292	0.00512026	4.182
NA	white	1000.47	44.818353	0.9920321	0.00085277	7.442
NA	brown	627.71092	130.7905	0.9936839	0.000602	9.09
NA	brown	748.26674	134.66069	0.9956843	0.00034012	0.648
NA	white	267.26137	17.108256	-0.581448	0.30381608	5.754
NA	white	880.44698	46.993085	0.9673284	0.00705424	10.376
NA	white	894.62975	47.124713	0.9699838	0.00621445	0.962
NA	blue	926.66304	148.64883	0.9920507	0.00084979	3.336
NA	purple	138.96461	32.818502	0.7931687	0.10934605	45.506
NA	black	596.44285	92.083471	0.9637478	0.00824063	6.484
NA	turquoise	170.62468	48.209099	0.3851015	0.52207807	4.502
NA	darkmagenta	231.24477	4.8669715	-0.784432	0.11618333	5.374
NA	purple	204.59881	52.507175	0.9061437	0.03402661	36.374
NA	darkgreen	242.5376	38.932149	0.9661166	0.00744894	37.44
NA	turquoise	290.41976	107.98523	0.7641007	0.13256405	42.778
NA	turquoise	322.69699	116.62647	0.7467059	0.14707562	11.9
NA	turquoise	547.23245	171.71038	0.9120047	0.03091764	11.754
NA	lightgreen	442.97592	36.730974	0.9558958	0.01104483	42.158
NA	purple	294.00012	75.880543	0.9866011	0.00185807	7.782
NA	salmon	246.45116	68.503031	0.9708586	0.00594554	12.616
NA	darkred	183.32937	41.465594	0.9624136	0.00869793	2.83
NA	grey60	292.67954	62.615489	0.977715	0.00398012	12.666
NA	purple	284.14912	78.599009	0.9954423	0.0003691	8.656
NA	midnightblue	720.51371	66.14822	0.9631064	0.00845945	6.134
NA	violet	161.40976	8.3858237	0.4044594	0.4994333	11.934
NA	darkturquoise	107.69828	21.184813	0.8175088	0.09097794	5.662
NA	yellow	269.30275	118.32406	0.9685539	0.0066623	4.82
NA	darkgreen	183.83494	28.003022	0.8813873	0.0481558	6.078
NA	turquoise	892.8224	224.55517	0.9825962	0.00274893	9.25
NA	yellow	296.21078	134.20895	0.9982238	8.98E-05	8.524
NA	turquoise	927.09392	215.74935	0.9564462	0.01083967	7.34
NA	blue	775.87155	135.89636	0.9733047	0.00521482	6.278
NA	red	681.6595	106.31915	0.9620094	0.00883809	12.108
NA	lightgreen	390.19914	35.394556	0.9262007	0.02379828	8.742
NA	paleturquoise	181.17117	23.714116	0.9416376	0.01677626	6.462
NA	darkred	202.16238	46.13568	0.9871956	0.00173596	2.668
NA	turquoise	621.94538	169.70992	0.908792	0.03260998	14.53
NA	paleturquoise	159.0558	19.2313	0.8911736	0.04238538	4.512
NA	salmon	205.09757	71.971838	0.9832103	0.00260495	3.638
NA	paleturquoise	154.45698	22.947028	0.9510547	0.01290285	77.926
NA	salmon	194.21831	58.747898	0.9388755	0.01797357	4.47

NA	tan	121.16107	35.333668	0.9073649	0.03337095	52.268
NA	darkturquoise	251.91821	36.736207	0.9364241	0.01905842	35.444
NA	tan	159.00031	30.619462	0.8629635	0.05962846	2.078
NA	grey60	309.14265	66.756298	0.9913213	0.00096928	67.304
NA	darkgreen	231.36004	41.866263	0.9849305	0.00221564	101.02
NA	darkred	219.06196	44.112899	0.9773811	0.00406971	3.976
NA	darkred	169.17798	39.745484	0.9510906	0.01288873	5.266
NA	yellow	307.90569	124.55989	0.9822976	0.00281986	61.324
NA	darkgreen	202.56724	36.518393	0.9513443	0.01278908	9.338
NA	paleturquoise	146.5521	17.682213	0.8683328	0.05620586	3.796
NA	paleturquoise	184.85475	27.615043	0.9890723	0.00136903	3.93
NA	salmon	199.44845	65.978672	0.9642715	0.00806333	12.008
NA	navy	79.041458	20.518187	0.7715225	0.1265099	14.076
NA	pink	894.62975	149.62591	0.9996875	6.63E-06	1.732
NA	lightcyan	252.07114	39.279101	0.8940846	0.04071455	14.966
NA	turquoise	223.3055	61.871016	0.5219201	0.36701195	26.222
NA	grey60	315.11161	68.819562	0.9982074	9.11E-05	6.112
NA	darkgreen	251.18438	42.165903	0.9906617	0.00108174	12.184
NA	green	928.64516	105.8042	0.9733236	0.0052093	134.056
NA	yellow	243.29551	112.50803	0.9591166	0.00986219	10.412
NA	yellow	230.17999	103.00988	0.9373776	0.01863399	71.61
NA	darkred	83.003643	17.632686	0.7668759	0.13029041	33.338
NA	skyblue	439.06296	43.250376	0.9937529	0.00059217	9.484
NA	turquoise	802.32092	198.54959	0.9270307	0.02340093	31.238
NA	pink	976.43173	137.42934	0.9818285	0.00293247	1.286
NA	yellow	309.97622	128.53897	0.9890708	0.00136931	12.748
NA	purple	261.26	65.573624	0.9503966	0.01316263	13.952
NA	royalblue	400.72514	51.595774	0.9491467	0.01366066	89.386
NA	salmon	105.16864	39.343862	0.8574415	0.06321375	49.154
NA	blue	577.85654	112.71965	0.9349024	0.01974214	25.89
NA	yellow	179.21481	75.281589	0.8647758	0.0584661	72.332
NA	grey	45.499302	0.0093525	-0.111052	0.85889574	43.324
NA	darkgrey	758.76937	46.279454	0.9654204	0.00767887	5.616
NA	salmon	227.62261	77.805535	0.9992686	2.37E-05	26.916
NA	greenyellow	71.870173	6.5696112	0.4644437	0.43065894	135.68
NA	orange	247.32689	37.629157	0.9977373	0.00012916	46.772
NA	salmon	252.29425	66.50596	0.9637863	0.00822756	11.49
NA	darkred	201.91667	46.576479	0.9907466	0.00106705	3.928
NA	purple	220.8174	56.648632	0.9211611	0.02625677	112.576
NA	greenyellow	239.03027	37.051451	0.9562794	0.0109017	0.586
NA	orange	74.521816	8.5040896	0.6237544	0.26083602	12.014
NA	greenyellow	99.373925	14.771209	0.7075525	0.18128895	27.926
NA	grey60	314.70048	68.724742	0.9978371	0.00012071	13.052
NA	yellow	299.71689	127.32422	0.9852628	0.00214287	16.392
NA	yellow	294.15012	115.99386	0.9641214	0.00811401	246.866
NA	darkgrey	1011.4944	50.411262	0.9813338	0.0030528	7.332
NA	darkgrey	894.23404	50.645579	0.9842104	0.00237606	4.524
NA	darkorange	168.41127	30.24248	0.9599865	0.00955037	53.108

NA	blue	941.88891	148.08233	0.9909386	0.00103403	2.482
NA	darkgreen	252.5695	40.8083	0.9818545	0.00292619	84.786
NA	purple	295.27161	78.722063	0.9950535	0.00041732	13.864
NA	pink	621.66421	120.33435	0.9552219	0.0112978	5.156
NA	yellow	240.41446	107.31448	0.9480987	0.01408285	19.336
NA	brown	813.50523	126.92448	0.9801145	0.00335613	15.624
NA	salmon	227.55178	72.520433	0.9837025	0.00249145	2.536
NA	blue	910.94741	144.36783	0.9855395	0.00208289	13.024
NA	turquoise	165.48708	64.100533	0.595419	0.28943314	61.652
NA	black	915.55288	99.184525	0.978686	0.00372338	3.772
NA	purple	305.10441	76.128279	0.9864386	0.00189194	10.708
NA	darkorange	141.31044	32.475733	0.9750265	0.00471977	15.164
NA	darkturquoise	228.69683	42.316545	0.9696167	0.00632844	34.224
NA	yellow	306.58823	128.75906	0.9890841	0.00136683	14.922
NA	navy	60.171926	29.042642	0.9074357	0.03333308	469.454
NA	darkred	192.23186	35.367255	0.9045998	0.03486129	14.7
NA	lightcyan	142.4372	24.44029	0.7502073	0.14411909	15.144
NA	paleturquoise	165.54382	20.568854	0.9163513	0.0286746	28.908
NA	yellow	313.95951	128.72432	0.9884068	0.00149583	5.882
NA	greenyellow	201.34106	39.507436	0.9911758	0.00099374	7.252
NA	yellow	252.6786	117.35974	0.9675591	0.00697989	19.888
NA	pink	925.50841	143.8911	0.991575	0.00092712	3.346
NA	tan	173.2182	28.927751	0.8296627	0.0822013	60.614
NA	lightcyan	338.97651	56.407489	0.9861337	0.00195601	6.638
NA	pink	915.63554	148.31361	0.9978044	0.00012346	3.402
NA	turquoise	944.05344	206.31503	0.9345784	0.01988875	8.274
NA	lightcyan	217.19279	38.087699	0.8852645	0.04584177	52.9
NA	turquoise	487.63165	143.95204	0.8574751	0.06319176	20.958
NA	greenyellow	197.61157	39.970286	0.9566594	0.0107605	7.7
NA	lightcyan	138.79638	23.163532	0.7327779	0.15900794	5.636
NA	grey60	314.72079	69.104301	0.9989219	4.25E-05	38.772
NA	tan	113.34178	28.757148	0.8290096	0.08266591	6.456
NA	purple	240.66031	54.14388	0.9133215	0.03023237	26.768
NA	blue	681.14798	90.277192	0.878093	0.0501501	7.53
NA	darkgrey	997.17342	50.774107	0.9829967	0.00265475	6.094
NA	black	669.31465	98.61728	0.9792456	0.00357801	39.996
NA	purple	294.41104	76.822276	0.9891644	0.00135178	5.302
NA	pink	894.62975	149.62591	0.9996875	6.63E-06	1.348
NA	red	832.05018	120.256	0.9873116	0.00171244	6.848
NA	magenta	427.01671	74.178091	0.9574965	0.01045158	34.934
NA	pink	974.65766	144.8498	0.9926407	0.00075702	15.73
NA	magenta	157.97238	63.354299	0.9239307	0.02489599	7.214
NA	brown	849.37351	122.44447	0.9675889	0.0069703	31.352
NA	darkred	41.52989	5.5291669	0.4801412	0.41304214	292.206
NA	greenyellow	60.958694	13.144684	0.4853154	0.4072725	12.97
NA	darkgrey	960.29165	51.906972	0.9883755	0.00150189	4.58
NA	magenta	513.973	63.043151	0.9129236	0.0304389	5.706
NA	darkgreen	182.99196	34.59866	0.9288835	0.02252173	70.158

NA	lightcyan	527.43753	57.532756	0.9775378	0.00402758	5.446
NA	magenta	247.67521	82.984051	0.9908034	0.00105724	4.976
NA	turquoise	488.68292	59.700482	0.215885	0.72727695	13.406
NA	purple	273.27863	76.992063	0.9912009	0.00098951	38.5
NA	darkturquoise	200.55248	38.101543	0.9465848	0.01470012	156.006
NA	lightcyan	196.83248	34.616311	0.8505365	0.06778789	5.87
NA	turquoise	290.74884	55.435608	-0.168186	0.78687296	8.31
NA	turquoise	247.21094	93.82426	0.6893955	0.19783226	10.782
NA	pink	1004.1733	130.92498	0.9714636	0.00576188	45.272
NA	magenta	207.69633	76.468183	0.9707127	0.00599011	4.288
NA	magenta	289.36206	71.5651	0.9577543	0.01035707	9.486
NA	salmon	180.15517	67.375341	0.9703213	0.00611023	1.88
NA	turquoise	328.19568	60.115206	-0.105985	0.86530828	9.844
NA	red	894.62975	127.58416	0.9992509	2.46E-05	1.156
NA	darkorange	157.264	28.279075	0.9442125	0.01568454	85.036
NA	green	897.48587	109.52735	0.9821994	0.0028433	7.432
NA	brown	604.02532	123.7293	0.9827273	0.00271798	6.626
NA	darkred	199.6352	46.097809	0.9874209	0.00169039	29.3
NA	salmon	172.45418	66.046479	0.9652032	0.0077511	6.01
NA	white	954.08481	43.044445	0.9796737	0.0034681	20.7
NA	yellow	242.26084	111.21038	0.956681	0.01075249	203.338
NA	grey60	263.33586	57.086221	0.9579788	0.01027496	18.414
NA	grey60	314.63871	67.319081	0.9933166	0.00065523	9.144
NA	greenyellow	242.10766	37.052212	0.9754897	0.00458937	561.086
NA	lightyellow	323.45608	57.330849	0.9730528	0.00528861	7.702
NA	red	1010.0172	113.2408	0.9735128	0.00515412	24.956
NA	purple	271.45699	76.191404	0.9887185	0.00143597	5.09
NA	lightgreen	391.17452	34.647751	0.8602908	0.0613556	14.738
NA	darkred	215.63893	44.786419	0.9792777	0.00356973	3.646
NA	yellow	289.51444	133.07184	0.9965631	0.00024175	2.122
NA	darkred	173.40607	37.956454	0.9441882	0.01569471	21.29
NA	turquoise	242.55846	91.067994	0.7021349	0.18618207	57.572
NA	grey60	312.98443	68.470364	0.9969876	0.00019838	4.3
NA	greenyellow	55.079723	12.87191	0.2991409	0.62488122	61.326
NA	pink	836.62305	146.02289	0.9948128	0.00044812	17.188
NA	white	902.27407	47.442812	0.9738624	0.00505269	10.072
NA	greenyellow	51.348056	8.117986	0.3817067	0.5260701	21.53
NA	turquoise	166.82848	61.742284	0.5824742	0.30275302	89.56
NA	black	905.38436	100.85145	0.9825289	0.00276487	23.73
NA	royalblue	737.97899	57.844443	0.9750542	0.00471194	12.292
NA	greenyellow	72.149925	12.870412	0.4694753	0.42499386	26.624
NA	yellow	307.49843	130.73921	0.9921974	0.00082639	15.368
NA	white	274.17295	16.880345	-0.589009	0.29600987	159.446
NA	grey	567.7703	14.699835	0.9811609	0.00309522	11.224
NA	red	884.33261	125.72147	0.996291	0.00027101	4.904
NA	purple	304.40115	76.228256	0.9870853	0.00175839	6.024
NA	violet	863.86377	46.361648	0.9947378	0.00045787	2.1
NA	brown	660.6513	131.1069	0.993875	0.00057491	2.848

NA	pink	894.62975	149.62591	0.9996875	6.63E-06	1.412
NA	royalblue	837.59903	54.89234	0.9622907	0.00874048	24.256
NA	grey60	220.7718	46.45399	0.9148638	0.02943606	17.628
NA	turquoise	200.67928	74.24649	0.6425645	0.24229918	4.49
NA	purple	237.76304	67.476016	0.9626831	0.00860491	731.748
NA	purple	267.03793	74.790262	0.9847009	0.00226639	19.74
NA	green	641.44722	102.10606	0.9709221	0.00592619	4.542
NA	grey60	292.23832	58.278328	0.9622948	0.00873906	8.486
NA	darkred	195.11276	42.782746	0.9651083	0.00778272	5.568
NA	navy	48.781083	17.476148	0.7927182	0.10969556	94.166
NA	paleturquoise	187.83814	23.766256	0.9522165	0.01244838	260.766
NA	blue	640.43986	137.83571	0.9782008	0.00385097	14.458
NA	turquoise	471.54463	152.44995	0.862719	0.05978581	2.09
NA	orange	369.32951	33.191302	0.9697198	0.00629635	9.344
NA	white	258.28426	16.20861	-0.709688	0.17937009	64.866
NA	greenyellow	100.07996	11.018116	0.6209028	0.263678	20.624
NA	turquoise	544.47802	171.45027	0.9321779	0.02098564	14.076
NA	darkred	201.88744	45.327974	0.9835879	0.00251772	25.772
NA	darkgreen	162.64061	33.800839	0.9149752	0.02937884	15.356
NA	darkred	211.41245	41.539467	0.9543267	0.01163669	7.03
NA	yellow	273.39915	105.5872	0.9390797	0.01788414	2.674
NA	grey	429.8974	14.949534	0.9875083	0.00167282	18.584
NA	black	874.59073	102.80632	0.9868123	0.00181438	17.466
NA	white	916.64185	47.242739	0.9785238	0.00376587	18.35
NA	purple	306.41566	76.325581	0.9873417	0.00170637	25.312
NA	yellow	269.88865	103.87074	0.9361291	0.01919038	78.944
NA	grey60	286.42172	62.682974	0.9784069	0.0037966	11.334
NA	brown	197.08296	21.386381	0.5878274	0.29722585	12.78
NA	turquoise	237.04924	70.097853	0.5993968	0.28537162	4.532
NA	lightyellow	494.3734	62.179667	0.9913023	0.00097247	14.21
NA	green	916.81027	115.01827	0.9956006	0.00035005	2.548
NA	brown	694.2636	136.39268	0.9987987	5.00E-05	6.856
NA	royalblue	743.90472	61.694418	0.9893933	0.00130923	6.454
NA	greenyellow	91.512608	10.335338	0.57275	0.31285995	237.388
NA	brown	115.58719	11.305632	0.3131012	0.6079599	12.118
NA	darkmagenta	358.73367	3.7412243	-0.700096	0.18803277	30.906
NA	navy	899.8914	19.168376	0.1016906	0.87074701	2.048
NA	blue	553.25703	86.800406	0.8722343	0.05375912	8.246
NA	brown	529.62183	113.45339	0.9643788	0.00802719	15.994
NA	violet	44.397548	8.2588774	-0.860663	0.06111383	2.472
NA	darkgreen	264.20477	42.723932	0.9965148	0.00024686	17.072
NA	tan	347.01544	30.677121	0.8381396	0.07624458	6.444
NA	red	1007.1251	115.05766	0.9769037	0.00419893	20.888
NA	red	899.16037	126.02209	0.9966895	0.00022854	5.712
NA	yellow	284.45911	130.0728	0.9914998	0.00093956	120.698
NA	lightyellow	632.47109	54.802597	0.9614715	0.00902569	6.904
NA	white	179.49519	13.736927	-0.759154	0.13664548	8.124
NA	yellow	268.66329	123.18643	0.9794117	0.00353523	5.49

NA	darkgrey	774.73602	44.033752	0.9560623	0.01098262	13.386
NA	midnightblue	989.43292	71.491602	0.9783593	0.00380912	7.018
NA	blue	662.02119	121.61895	0.9487745	0.01381011	6.838
NA	turquoise	220.3768	89.3886	0.6899021	0.19736519	0.238
NA	greenyellow	48.021434	9.331978	0.4053005	0.49845402	20.468
NA	turquoise	749.08581	175.98227	0.9079486	0.03305902	4.008
NA	grey60	314.51513	69.150858	0.999078	3.36E-05	5.65
NA	paleturquoise	181.9877	27.560503	0.9873906	0.00169649	90.52
NA	turquoise	236.56797	89.126707	0.6666224	0.21914405	16.608
NA	yellow	276.26722	128.09806	0.9879604	0.00158295	17.43
NA	violet	727.19041	40.534453	0.9741263	0.00497655	6.276
NA	lightyellow	240.9861	38.086037	0.8875109	0.04451767	25.964
NA	brown	281.2506	36.465726	0.7088711	0.18010363	11.378
NA	blue	465.1693	108.59315	0.9238189	0.02495045	29.92
NA	darkred	132.64104	23.293257	0.8226787	0.08721128	17.468
NA	darkgrey	955.59338	49.074727	0.976743	0.00424272	7.664
NA	tan	274.96631	31.202297	0.8492515	0.06865003	15.51
NA	white	930.35576	46.987478	0.9796623	0.00347102	3.34
NA	turquoise	957.88064	213.4217	0.9461473	0.01488008	2.408
NA	turquoise	210.17767	48.495203	-0.302452	0.62086034	6.088
NA	purple	282.74983	78.625257	0.9961251	0.00028939	5.746
NA	violet	667.38419	38.326372	0.9721508	0.00555558	2.036
NA	turquoise	942.2606	227.21177	0.9800466	0.00337331	6.332
NA	salmon	188.78093	69.540493	0.9759885	0.00445033	8.478
NA	magenta	181.51975	72.100737	0.9552086	0.01130281	24.706
NA	grey60	228.84634	47.383559	0.9205105	0.02657977	11.632
NA	brown	634.45836	127.31216	0.9878785	0.00159911	7.164
NA	blue	867.98346	131.32309	0.9643639	0.00803219	22.042
NA	turquoise	218.04508	81.19194	0.6671919	0.21860369	340.864
NA	yellow	313.08756	129.53886	0.9902171	0.00115983	102.718
NA	lightcyan	187.1842	28.303672	0.8383709	0.07608404	51.636
NA	green	468.4736	76.416464	0.9050177	0.03463472	21.488
NA	lightgreen	367.12433	36.180113	0.9401361	0.01742378	9.486
NA	turquoise	709.15424	199.15887	0.9649791	0.00782582	3.842
NA	tan	285.92036	36.097287	0.8916303	0.0421218	3.558
NA	lightgreen	369.92511	31.666685	0.9270259	0.02340318	46.222
NA	pink	762.23226	132.36516	0.9749978	0.00472786	155.31
NA	purple	191.69469	46.493034	0.8731938	0.05316264	1008.516
NA	violet	53.122186	10.246999	-0.928236	0.02282768	11.132
NA	red	930.45374	126.765	0.9976623	0.00013564	2.064
NA	red	918.23482	124.50586	0.9939961	0.00055795	3.572
NA	darkgreen	236.49934	41.724464	0.9874515	0.00168423	20.696
NA	turquoise	842.51965	184.26752	0.8897908	0.04318648	5.16
NA	purple	289.67604	78.548656	0.9942644	0.00052099	33.242
NA	black	913.13432	103.32829	0.987796	0.00161544	199.596
NA	royalblue	537.66542	58.826115	0.9811163	0.00310622	28.368
NA	turquoise	572.86623	171.00012	0.9185249	0.02757344	7.718
NA	tan	118.9328	30.753034	0.8630135	0.05959626	10.248

NA	lightyellow	518.03976	60.414752	0.9853734	0.00211882	18.13
NA	magenta	199.03181	76.024117	0.9688956	0.00655435	30.312
NA	black	600.55005	92.956302	0.9658266	0.00754446	12.102
NA	skyblue	406.56595	41.64748	0.9855115	0.00208893	163.098
NA	darkorange	128.78824	31.1911	0.9636009	0.00829056	3.974
NA	purple	203.06944	49.00002	0.8924211	0.04166674	14.254
NA	magenta	437.70475	76.408011	0.9633109	0.00838947	8.662
NA	green	722.02333	106.14491	0.9803031	0.00330858	8.828
NA	black	907.4721	102.24334	0.9862674	0.00192782	28.73
NA	red	934.90685	125.98264	0.9963182	0.00026803	5.218
NA	turquoise	872.89478	202.7012	0.9270377	0.02339754	27.2
NA	darkturquoise	225.23757	40.861383	0.9605976	0.00933328	10.49
NA	lightcyan	466.85906	53.611478	0.9640121	0.008151	19.224
NA	yellow	154.06111	60.856415	0.8211611	0.0883119	20.542
NA	brown	703.11399	136.2249	0.997552	0.00014534	11.212
NA	magenta	100.26961	42.00027	0.8198795	0.08924466	23.23
NA	royalblue	723.5996	61.528268	0.9894672	0.00129558	29.382
NA	navy	82.063074	7.3271889	-0.567639	0.31820614	6.6
NA	tan	89.924163	22.550178	0.7286933	0.16255824	23.228
NA	turquoise	809.57927	213.47268	0.9733632	0.00519772	12.518
NA	turquoise	901.47354	220.56193	0.9700791	0.00618497	8.486
NA	blue	853.5275	143.40111	0.9840214	0.0024188	9.212
NA	yellow	235.99709	84.447839	0.886904	0.04487418	15.364
NA	turquoise	399.81854	138.37645	0.8587135	0.06238209	4.5
NA	lightyellow	298.50063	48.130895	0.9365928	0.01898311	123.366
NA	lightcyan	273.7527	46.771602	0.9414236	0.01686806	15.382
NA	pink	766.79369	136.9849	0.981767	0.00294734	6.14
NA	darkgrey	841.19766	51.54784	0.9877963	0.00161539	6.848
NA	black	941.84313	98.830004	0.978192	0.0038533	6.618
NA	black	819.76705	107.89626	0.9976909	0.00013316	1.112
NA	red	944.03277	123.59893	0.9924168	0.00079181	8.292
NA	lightyellow	622.15134	56.928769	0.9699872	0.0062134	9.078
NA	turquoise	917.00012	222.64383	0.9750843	0.00470341	3.838
NA	salmon	222.58417	74.508506	0.9906701	0.0010803	138.208
NA	turquoise	195.66813	46.395687	-0.386784	0.52010199	35.68
NA	turquoise	939.65933	212.32749	0.9479899	0.01412695	3.832
NA	purple	295.24065	79.910807	0.9985997	6.29E-05	15.684
NA	blue	954.82947	144.23996	0.9845784	0.00229362	11.744
NA	red	929.51148	126.83827	0.9977905	0.00012463	4.254
NA	turquoise	363.16186	129.92002	0.8515466	0.06711256	6.604
NA	red	861.14398	126.08984	0.9969153	0.00020557	6.406
NA	darkgreen	267.24754	41.612003	0.9886561	0.00144789	17.258
NA	lightgreen	297.8019	30.138564	0.8309143	0.08131315	3.29
NA	grey	301.06685	12.174571	0.9442843	0.01565442	4.116
NA	darkturquoise	216.84366	43.503024	0.9799072	0.00340863	71.968
NA	green	557.3467	80.872851	0.9208253	0.0264233	7.234
NA	darkred	111.80595	25.437864	0.8261485	0.08471084	11.4
NA	brown	417.3262	86.032572	0.905376	0.03444081	5.694

NA	navy	141.72693	40.651485	0.9803926	0.0032861	9.636
NA	magenta	352.30248	66.726728	0.9394587	0.01771852	36.04
NA	salmon	221.6329	72.790082	0.9846671	0.00227389	2.974
NA	darkorange	198.29562	11.171578	0.7277661	0.1633673	5.71
NA	pink	844.92618	147.27961	0.9965444	0.00024372	3.412
NA	yellow	275.57484	125.6439	0.9833469	0.00257328	3.448
NA	lightcyan	383.78833	57.889381	0.9907951	0.00105868	3.626
NA	greenyellow	160.63435	28.76712	0.9221695	0.0257586	49.002
NA	magenta	365.49299	81.37314	0.982584	0.00275181	12.478
NA	tan	106.90268	29.497746	0.8271553	0.0839895	25.732
NA	red	871.61577	125.80543	0.9964709	0.00025154	2.542
NA	salmon	219.14187	75.53241	0.9929973	0.00070271	1.786
NA	pink	894.97665	145.04011	0.9933798	0.00064597	1.598
NA	salmon	243.06126	71.852357	0.9809633	0.00314396	4.4
NA	red	869.17738	126.73725	0.9979353	0.00011259	3.178
NA	yellow	306.08965	128.53376	0.9883906	0.00149896	31.642
NA	salmon	215.84626	76.295692	0.9955058	0.00036142	28.204
NA	red	962.96654	122.24248	0.9899629	0.00120529	7.74
NA	darkgreen	151.4862	15.246882	0.6929133	0.19459533	3.998
NA	blue	592.35664	128.18375	0.9613562	0.00906609	5.538
NA	darkturquoise	147.69566	36.889349	0.9360152	0.01924137	71.476
NA	royalblue	434.26891	53.058148	0.9578289	0.01032974	14.328
NA	darkgreen	204.94062	37.607643	0.9544543	0.01158818	25.95
NA	navy	192.48254	33.510739	0.9109952	0.03144626	37.466
NA	lightcyan	154.98391	28.921106	0.7954046	0.10761649	0.522
NA	pink	980.96181	142.572	0.9892976	0.00132696	4.81
NA	paleturquoise	180.53407	26.713806	0.9790043	0.00364047	24.868
NA	paleturquoise	171.8646	21.381171	0.9119092	0.03096749	18.622
NA	purple	306.74077	74.582537	0.9821017	0.0028667	60.938
NA	white	875.76124	46.883784	0.9679744	0.00684671	2.49
NA	red	891.7913	127.4722	0.9990665	3.42E-05	10.792
NA	black	741.9391	105.50406	0.993237	0.00066697	9.492
NA	red	932.31159	125.90103	0.9961963	0.00028145	3.604
NA	pink	894.62975	149.62591	0.9996875	6.63E-06	1.796
NA	darkorange	159.86962	27.421918	0.9366625	0.01895204	12.94
NA	salmon	239.59776	71.398444	0.9791008	0.00361545	22.114
NA	greenyellow	184.92012	36.620845	0.9621334	0.00879499	15.412
NA	turquoise	289.19603	96.139714	0.6981039	0.18984668	6.594
NA	lightcyan	629.37945	51.328081	0.9402918	0.01735628	35.848
NA	purple	241.85184	52.886714	0.9071963	0.03346124	24.142
NA	tan	376.82751	25.871309	0.7943882	0.10840174	16.828
NA	midnightblue	987.15869	74.398311	0.9868691	0.00180268	14.92
NA	pink	958.53379	145.39051	0.9935003	0.00062842	6.636
NA	lightcyan	563.12054	53.607229	0.9569909	0.01063783	6.014
NA	magenta	466.45873	70.096844	0.9415526	0.01681269	3.834
NA	grey60	285.98775	61.385662	0.9743038	0.00492556	13.388
NA	purple	285.005	78.842497	0.9964543	0.00025331	8.93
NA	brown	714.38308	135.80107	0.9960226	0.00030093	20.036

NA	black	896.24548	104.30595	0.9898535	0.00122503	2.084
NA	violet	883.19289	46.68497	0.991519	0.00093637	3.698
NA	turquoise	954.12282	202.26967	0.9225462	0.02557333	6.952
NA	turquoise	903.89131	210.18828	0.9503436	0.01318364	1.076
NA	pink	915.45441	146.30619	0.995	0.0004241	4.044
NA	pink	927.31643	148.27467	0.9977018	0.00013221	3.414
NA	pink	908.09749	149.57467	0.9995979	9.68E-06	9.938
NA	pink	928.4587	148.15552	0.9975308	0.00014723	3.286
NA	turquoise	880.75695	214.97744	0.9551806	0.01131334	2.758
NA	red	894.62975	127.58416	0.9992509	2.46E-05	1.926
NA	violet	151.51666	8.381195	0.500131	0.39085777	12.766
NA	turquoise	917.73546	223.95079	0.9778254	0.00395064	1.482
NA	white	931.45784	42.829634	0.9797776	0.0034416	2.266
NA	red	937.17013	126.31037	0.9969155	0.00020554	5.588
NA	grey60	299.74079	65.44283	0.9879308	0.00158877	85.844
NA	magenta	459.99118	72.897982	0.9508734	0.01297427	52.122
NA	darkturquoise	98.319966	17.604933	0.7666357	0.13048676	69.328
NA	yellow	210.30197	97.10964	0.9220485	0.0258182	93.966
NA	magenta	515.47542	59.519772	0.8995221	0.03765162	12.226
NA	purple	281.40472	71.973988	0.9740666	0.00499375	4.432
NA	darkred	213.94143	45.585517	0.9860086	0.00198249	4.026
NA	greenyellow	72.689673	6.39547	0.3658272	0.54482284	42.484
NA	pink	895.15131	149.32515	0.9992845	2.30E-05	9.284
NA	tan	109.1342	18.229684	0.7107542	0.17841475	3.104
NA	violet	871.29275	46.883929	0.996282	0.000272	2.106
NA	violet	86.528585	2.9296746	0.3166824	0.60363216	6.654
NA	purple	281.46817	77.301682	0.9912534	0.00098066	38.982
NA	turquoise	161.57762	61.79635	0.6480912	0.23692373	1.138
NA	turquoise	260.83581	55.835059	-0.182677	0.76870859	17.074
NA	turquoise	329.43043	61.220324	-0.09055	0.88486597	10.114
NA	darkgreen	265.56117	41.962099	0.9915228	0.00093575	111.738

Table S2-3 Attributes of genes in all modules of LT prenatal.

GeneSymbol	Module	kTotal	kWithin	eigencorr	eigenpval	meanExpr
gi|194033493|ref|XM_001924291.1|	turquoise	1054.6642	1007.8911	0.998818	4.88E-05	51.704
gnl|UG|Ssc#S23765833	turquoise	1054.441	1012.4636	0.999748	4.80E-06	0.998
gnl|UG|Ssc#S17516362	turquoise	1054.1549	1011.1127	0.999521	1.26E-05	16.744
gnl|UG|Ssc#S40046162	turquoise	1053.7264	1013.4352	0.9999381	5.84E-07	5.98
gnl|UG|Ssc#S40335794	turquoise	1053.7098	1013.1976	0.9998576	2.04E-06	2.51
gnl|UG|Ssc#S40429630	turquoise	1053.4764	1013.5268	0.9999022	1.16E-06	6.172
gnl|UG|Ssc#S40003133	turquoise	1053.4395	1013.316	0.9998675	1.83E-06	1.82
gnl|UG|Ssc#S40207636	turquoise	1053.3004	1008.1965	0.998927	4.22E-05	8.352
gnl|UG|Ssc#S18554195	turquoise	1052.9716	1011.8657	0.9995056	1.32E-05	226.234
gnl|UG|Ssc#S31113730	turquoise	1052.7768	1012.268	0.9996412	8.16E-06	6.196
gi|115551932|dbj|AK236339.1|	turquoise	1052.6329	1011.1703	0.9993167	2.14E-05	83.81
gi|194044495|ref|XM_001929295.1|	turquoise	1052.5896	1006.1068	0.9984421	7.38E-05	6.63
gi|115550899|dbj|AK238760.1|	turquoise	1052.4197	1003.0932	0.9976686	0.00013509	34.748
gi|115555134|dbj|AK240334.1|	turquoise	1051.8734	1012.6715	0.9997064	6.04E-06	7.064
gnl|UG|Ssc#S40091110	turquoise	1051.5297	1009.5922	0.9989343	4.18E-05	25.186
gi|115554410|dbj|AK233420.1|	turquoise	1051.5215	1006.4645	0.998367	7.92E-05	121.846
gnl|UG|Ssc#S18361249	turquoise	1051.4461	1012.6394	0.999701	6.21E-06	6.734
gnl|UG|Ssc#S35328709	turquoise	1051.4384	1006.2958	0.9983694	7.90E-05	10.282
gi|115548982|dbj|AK238171.1|	turquoise	1051.3525	1011.929	0.999516	1.28E-05	29.864
gi|115550091|dbj|AK232326.1|	turquoise	1051.2964	1012.4102	0.9995577	1.12E-05	16.728
gi|194037715|ref|XM_001927280.1|	turquoise	1051.1837	1006.769	0.9983712	7.89E-05	40.156
gi|115548514|dbj|AK235131.1|	turquoise	1051.0373	1000.9548	0.9971477	0.00018278	82.9
gnl|UG|Ssc#S34516266	turquoise	1050.7433	999.61157	0.9969206	0.00020503	50.498
gi|194038523|ref|XM_001928961.1|	turquoise	1050.6596	1011.6022	0.9993118	2.17E-05	14.154
gnl|UG|Ssc#S26401464	turquoise	1050.6548	1009.6883	0.9989135	4.30E-05	12.976
gnl|UG|Ssc#S19547504	turquoise	1050.3592	1007.2876	0.9984115	7.60E-05	48.652
gi|194036256|ref|XM_001929662.1|	turquoise	1050.2019	1010.3364	0.9990896	3.30E-05	10.146
gnl|UG|Ssc#S40330262	turquoise	1050.099	1013.0316	0.9996175	8.98E-06	36.796
gnl|UG|Ssc#S27600517	turquoise	1049.877	999.96308	0.9971429	0.00018325	76.636
gi|115552369|dbj|AK236374.1|	turquoise	1049.7518	1003.3503	0.9979219	0.00011368	38.648
gi|115549428|dbj|AK235450.1|	turquoise	1049.0197	1000.099	0.9968576	0.00021136	252.93
gi|115545523|dbj|AK230638.1|	turquoise	1049.0183	1001.3638	0.9971532	0.00018226	10.756
gnl|UG|Ssc#S27602031	turquoise	1048.9169	1011.3671	0.9992398	2.52E-05	9.856
gnl|UG|Ssc#S39797758	turquoise	1048.8885	996.17809	0.9960901	0.00029331	12.398
gnl|UG|Ssc#S40097724	turquoise	1048.711	1008.1287	0.9985528	6.61E-05	5.368
gnl|UG|Ssc#S19539677	turquoise	1048.593	1005.1164	0.997871	0.00011789	57.64
gi|194036903|ref|XM_001927877.1|	turquoise	1048.5737	1010.8482	0.9991494	2.98E-05	3.626
gnl|UG|Ssc#S40471679	turquoise	1048.2008	1008.5187	0.9986393	6.02E-05	7.528
gnl|UG|Ssc#S23695825	turquoise	1047.9085	999.24506	0.9968337	0.00021377	36.45
gnl|UG|Ssc#S40476333	turquoise	1047.9025	996.53714	0.9960561	0.00029714	128.712
gi|178056566|ref|NM_001123160.1|	turquoise	1047.87	998.04402	0.9966684	0.00023073	45.34
gnl|UG|Ssc#S39842447	turquoise	1047.87	1002.1019	0.9972445	0.00017357	399.278
gnl|UG|Ssc#S23768479	turquoise	1047.6043	999.95687	0.9968363	0.00021351	11.356
gnl|UG|Ssc#S17511420	turquoise	1047.5625	993.38915	0.9954697	0.00036578	14.598
gnl|UG|Ssc#S40087890	turquoise	1047.5427	1011.9636	0.9992968	2.24E-05	2.496
gnl|UG|Ssc#S31122178	turquoise	1047.375	1010.1845	0.9988975	4.39E-05	5.284

gnl|UG|Ssc#S6077926	turquoise	1047.2688	1011.8309	0.9992564	2.43E-05	6.088
gnl|UG|Ssc#S38482401	turquoise	1046.9732	1010.3666	0.9990092	3.74E-05	3.344
gi|194040422|ref|XM_001929580.1|	turquoise	1046.8919	1001.6103	0.9972324	0.0001747	198.746
gnl|UG|Ssc#S31115449	turquoise	1046.7379	1007.6381	0.9983386	8.13E-05	3.542
gnl|UG|Ssc#S23699804	turquoise	1046.7157	1007.4285	0.9982633	8.69E-05	18.056
gi|115553457|dbj|AK236660.1|	turquoise	1046.5598	1005.5445	0.9982061	9.12E-05	37.81
gnl|UG|Ssc#S26733295	turquoise	1046.4114	998.67228	0.9965247	0.00024581	7.662
gnl|UG|Ssc#S17514753	turquoise	1046.3823	1007.4831	0.9983255	8.22E-05	40.788
gi|115547162|dbj|AK231217.1|	turquoise	1046.3777	994.88866	0.9960211	0.00030111	11.298
gnl|UG|Ssc#S31102648	turquoise	1046.1154	997.02954	0.9960843	0.00029397	5.23
gi|115552819|dbj|AK239413.1|	turquoise	1046.1049	1008.305	0.9984513	7.31E-05	15.252
gi|115550524|dbj|AK232354.1|	turquoise	1045.6186	996.05847	0.9959024	0.00031467	18.096
gnl|UG|Ssc#S40443177	turquoise	1045.5362	1009.4609	0.9986609	5.88E-05	5.244
gnl|UG|Ssc#S19541809	turquoise	1045.0865	1009.5276	0.9987019	5.61E-05	14.794
gi|115554919|dbj|AK233731.1|	turquoise	1045.029	1001.3089	0.9969653	0.00020059	96.498
gnl|UG|Ssc#S18557502	turquoise	1044.9576	1008.2748	0.9984324	7.45E-05	32.266
gi|194039262|ref|XM_001927034.1|	turquoise	1044.9272	992.28068	0.9950259	0.0004208	7.186
gi|115554167|dbj|AK239962.1|	turquoise	1044.9219	1006.6946	0.9981719	9.38E-05	5.008
gi|47523819|ref|NM_214382.1|	turquoise	1044.7347	992.95986	0.9952497	0.00039274	38.16
gnl|UG|Ssc#S18356022	turquoise	1044.4747	1008.0548	0.998321	8.26E-05	19.166
gnl|UG|Ssc#S40483512	turquoise	1044.0601	1010.1035	0.9987808	5.11E-05	6.774
gnl|UG|Ssc#S18357288	turquoise	1043.8846	992.65117	0.9951884	0.00040036	32.63
gnl|UG|Ssc#S31133718	turquoise	1043.8708	1009.4244	0.9987173	5.51E-05	2.506
gnl|UG|Ssc#S26728785	turquoise	1043.4889	994.38603	0.9954532	0.00036779	10.446
gnl|UG|Ssc#S23760234	turquoise	1043.4407	992.6863	0.9950321	0.00042002	9.23
gnl|UG|Ssc#S17526090	turquoise	1043.3022	1009.6315	0.9986144	6.19E-05	3.038
gnl|UG|Ssc#S18386392	turquoise	1043.2916	994.67235	0.9954824	0.00036425	16.412
gnl|UG|Ssc#S18557753	turquoise	1043.1762	1000.3943	0.9967202	0.00022536	17.164
gnl|UG|Ssc#S17515524	turquoise	1043.1605	1008.6364	0.9984168	7.56E-05	4.052
gnl|UG|Ssc#S18553622	turquoise	1043.1451	988.88632	0.9943162	0.00051395	42.66
gnl|UG|Ssc#S19545946	turquoise	1043.1219	991.76905	0.9953233	0.00038366	1.638
gi|115554282|dbj|AK240077.1|	turquoise	1042.8367	991.53397	0.9948147	0.00044788	201.248
gi|115550817|dbj|AK235892.1|	turquoise	1042.6641	1008.4871	0.9984843	7.08E-05	43.424
gnl|UG|Ssc#S18357333	turquoise	1042.3943	1005.371	0.9978943	0.00011596	15.246
gi|47522661|ref|NM_213911.1|	turquoise	1042.3529	991.12455	0.9950022	0.00042381	308.788
gi|115552213|dbj|AK230522.1|	turquoise	1042.2183	992.21108	0.9949966	0.00042452	14.592
gi|115551630|dbj|AK239094.1|	turquoise	1042.2023	1008.2161	0.9982801	8.56E-05	38.442
gnl|UG|Ssc#S17510766	turquoise	1042.0976	990.88303	0.9946168	0.00047375	18.444
gnl|UG|Ssc#S18275000	turquoise	1041.9986	1006.8213	0.9980075	0.00010674	20.81
gnl|UG|Ssc#S18554424	turquoise	1041.66	1007.4572	0.9982606	8.71E-05	8.7
gnl|UG|Ssc#S17516685	turquoise	1041.3725	988.53144	0.9941268	0.00053984	15.596
gnl|UG|Ssc#S39995335	turquoise	1041.3278	987.77974	0.9940466	0.00055093	25.332
gi|115549536|dbj|AK235558.1|	turquoise	1041.1911	1001.5888	0.9969227	0.00020483	8.27
gnl|UG|Ssc#S22272334	turquoise	1041.1451	1003.8308	0.9976291	0.00013853	31.76
gnl|UG|Ssc#S21557354	turquoise	1040.9611	1006.7776	0.9979646	0.0001102	2.228
gnl|UG|Ssc#S39995545	turquoise	1040.6067	1007.9272	0.9981356	9.66E-05	2.08
gnl|UG|Ssc#S18556647	turquoise	1040.4762	1007.8573	0.9981511	9.54E-05	5.912
gnl|UG|Ssc#S26733687	turquoise	1040.4576	1000.4411	0.99719	0.00017873	55.334

gnl|UG|Ssc#S18382745	turquoise	1040.2677	1006.6829	0.9978953	0.00011587	27.63
gnl|UG|Ssc#S19549265	turquoise	1040.1888	1007.7719	0.9981063	9.89E-05	11.768
gnl|UG|Ssc#S40301371	turquoise	1040.1568	998.53831	0.9963813	0.00026117	16.106
gnl|UG|Ssc#S31134253	turquoise	1040.0686	1003.618	0.9973679	0.00016204	33.014
gi|50979292|ref|NM_213742.1|	turquoise	1039.9973	1004.6852	0.9977592	0.00012729	3.702
gnl|UG|Ssc#S6040545	turquoise	1039.8737	981.30481	0.9924973	0.00077923	16.344
gnl|UG|Ssc#S6048475	turquoise	1039.7948	999.16475	0.9964974	0.00024871	14.848
gi|115550332|dbj|AK238597.1|	turquoise	1039.5775	990.24474	0.9943558	0.00050859	160.174
gnl|UG|Ssc#S6073842	turquoise	1039.5085	981.64683	0.9926006	0.00076321	5.114
gi|115553187|dbj|AK232985.1|	turquoise	1039.3394	993.39788	0.9951362	0.00040689	12.92
gnl|UG|Ssc#S26724557	turquoise	1039.3306	1002.1288	0.997413	0.00015789	14.22
gnl|UG|Ssc#S40482424	turquoise	1039.2698	1004.3842	0.9973158	0.00016687	43.88
gi|47523353|ref|NM_213741.1|	turquoise	1039.2468	994.23295	0.99549	0.00036333	8.126
gnl|UG|Ssc#S40068231	turquoise	1038.9434	1006.8841	0.9978678	0.00011815	16.324
gnl|UG|Ssc#S23696666	turquoise	1038.8517	991.4022	0.9945691	0.00048006	50.696
gi|115550795|dbj|AK235870.1|	turquoise	1038.6271	1005.7963	0.997619	0.00013942	14.938
gi|194037164|ref|XM_001928669.1|	turquoise	1038.1715	1006.2884	0.9976827	0.00013386	9.404
gnl|UG|Ssc#S39804944	turquoise	1038.0107	1001.5627	0.9966576	0.00023185	6.562
gi|194040165|ref|XM_001929121.1|	turquoise	1037.8778	1002.8356	0.9972042	0.00017739	3.008
gnl|UG|Ssc#S23697383	turquoise	1037.6854	1006.006	0.9976379	0.00013776	41.374
gnl|UG|Ssc#S18386777	turquoise	1037.4852	1002.3606	0.9968293	0.00021422	7.386
gnl|UG|Ssc#S27601215	turquoise	1037.3875	977.33897	0.9915953	0.00092378	39.3
gnl|UG|Ssc#S19539303	turquoise	1037.1417	1005.0537	0.9973528	0.00016343	8.092
gnl|UG|Ssc#S18555039	turquoise	1037.1277	999.14164	0.99675	0.0002223	38.046
gnl|UG|Ssc#S18557168	turquoise	1036.8709	990.22665	0.9942707	0.00052013	10.284
gnl|UG|Ssc#S40336522	turquoise	1036.6726	1005.2117	0.9973628	0.00016251	4.652
gnl|UG|Ssc#S18550631	turquoise	1036.465	1005.0956	0.9973562	0.00016312	1.702
gi|194035753|ref|XM_001927135.1|	turquoise	1035.5809	997.51498	0.9957194	0.00033598	68.604
gnl|UG|Ssc#S41446009	turquoise	1035.5387	992.82383	0.995116	0.00040943	32.84
gnl|UG|Ssc#S6003000	turquoise	1035.3178	1004.2762	0.997133	0.0001842	3.676
gi|115549078|dbj|AK238267.1|	turquoise	1035.3171	998.62318	0.9964904	0.00024945	25.792
gnl|UG|Ssc#S40564541	turquoise	1035.2625	996.73697	0.9956553	0.00034355	25.454
gnl|UG|Ssc#S39984038	turquoise	1035.1268	1001.5266	0.9965502	0.00024311	102.004
gnl|UG|Ssc#S40154544	turquoise	1034.9553	985.68694	0.9940923	0.0005446	156.742
gnl|UG|Ssc#S23691825	turquoise	1034.9521	1003.8144	0.9969985	0.00019731	3.154
gi|194041507|ref|XM_001924700.1|	turquoise	1034.8869	1003.7239	0.9969838	0.00019876	61.802
gnl|UG|Ssc#S26718597	turquoise	1034.8823	1002.1597	0.9966352	0.00023418	65.77
gnl|UG|Ssc#S23695118	turquoise	1034.8133	1003.7841	0.9970411	0.00019312	23.12
gnl|UG|Ssc#S23756754	turquoise	1034.7835	993.35615	0.995611	0.00034882	8.624
gnl|UG|Ssc#S22281441	turquoise	1034.778	983.42192	0.9928463	0.00072554	132.862
gi|115545900|dbj|AK234333.1|	turquoise	1034.7399	973.33951	0.990694	0.00107614	55.392
gnl|UG|Ssc#S40156654	turquoise	1034.6616	1000.6071	0.9965334	0.00024488	4.978
gi|55741850|ref|NM_001001264.1|	turquoise	1034.4724	1003.6642	0.9969667	0.00020045	1.906
gnl|UG|Ssc#S40105175	turquoise	1034.4724	1003.6642	0.9969667	0.00020045	1.906
gnl|UG|Ssc#S40441928	turquoise	1034.4222	1003.6276	0.9969568	0.00020143	2.892
gi|115547290|dbj|AK234663.1|	turquoise	1034.2827	1003.6608	0.996949	0.00020221	13.654
gnl|UG|Ssc#S40466766	turquoise	1034.2606	1002.1841	0.996661	0.00023149	8.946
gnl|UG|Ssc#S23775512	turquoise	1034.0485	1003.0172	0.9968072	0.00021646	3.132

gnl|UG|Ssc#S18557525	turquoise	1034.0341	1001.1111	0.9964268	0.00025627	37.862
gnl|UG|Ssc#S19540920	turquoise	1033.9025	1003.2479	0.9968539	0.00021173	1.972
gnl|UG|Ssc#S23699362	turquoise	1033.7281	1001.4832	0.9964477	0.00025402	6.514
gnl|UG|Ssc#S26720557	turquoise	1033.5266	994.16235	0.995177	0.00040179	45.982
gnl|UG|Ssc#S35331378	turquoise	1033.4796	995.76404	0.9956238	0.00034729	4.694
gi|194034150|ref|XM_001927070.1|	turquoise	1033.3378	973.22522	0.9905238	0.00110578	4.42
gi|115547124|dbj|AK231179.1|	turquoise	1033.3044	989.24903	0.9941937	0.00053065	41.33
gnl|UG|Ssc#S17525302	turquoise	1032.899	989.88479	0.9940264	0.00055374	4.242
gnl|UG|Ssc#S40094925	turquoise	1032.844	996.39683	0.9958321	0.0003228	3.336
gnl|UG|Ssc#S18358611	turquoise	1032.7838	1000.9431	0.9962882	0.00027131	4.412
gnl|UG|Ssc#S19546309	turquoise	1032.7447	1001.2697	0.9966274	0.000235	12.138
gnl|UG|Ssc#S23689871	turquoise	1032.7211	982.76645	0.9928786	0.00072064	6.17
gnl|UG|Ssc#S40570043	turquoise	1032.6054	995.14494	0.9954142	0.00037253	26.232
gi|194043626|ref|XM_001926661.1|	turquoise	1032.4377	978.61556	0.9916015	0.00092277	17.014
gi|115553166|dbj|AK232966.1|	turquoise	1032.3069	980.35025	0.9922239	0.00082219	16.99
gnl|UG|Ssc#S34516476	turquoise	1032.26	970.65311	0.9901332	0.00117477	7.04
gi|172073149|ref|NM_001122990.1|	turquoise	1032.2513	998.38128	0.9957373	0.00033387	38.594
gnl|UG|Ssc#S40482883	turquoise	1031.929	995.51944	0.9954167	0.00037222	37.47
gi|194039715|ref|XM_001925448.1|	turquoise	1031.9114	997.35607	0.9958074	0.00032568	71.254
gnl|UG|Ssc#S19550112	turquoise	1031.7493	999.23767	0.995978	0.00030601	2.98
gnl|UG|Ssc#S18555986	turquoise	1031.712	999.25586	0.9958996	0.00031499	15.754
gi|115553137|dbj|AK239723.1|	turquoise	1031.4721	998.93812	0.9958177	0.00032448	91.154
gi|52351441|gb|AY609871.1|	turquoise	1031.2509	1001.1835	0.996291	0.000271	65.096
gi|194041930|ref|XM_001925953.1|	turquoise	1031.1968	1001.2355	0.9963105	0.00026887	9.45
gnl|UG|Ssc#S17526967	turquoise	1031.1728	999.54899	0.9961687	0.00028452	4.8
gi|115554046|dbj|AK233255.1|	turquoise	1031.0101	1001.0945	0.9962725	0.00027303	2.378
gi|47523129|ref|NM_213866.1|	turquoise	1031.0101	1001.0945	0.9962725	0.00027303	2.378
gi|194037909|ref|XM_001924188.1|	turquoise	1030.7153	1000.1058	0.9960333	0.00029973	11.378
gnl|UG|Ssc#S40471826	turquoise	1030.701	974.56946	0.9912836	0.0009756	26.016
gi|209863048|ref|NM_001135966.1|	turquoise	1030.4662	995.73802	0.9953811	0.00037657	9.786
gi|47523317|ref|NM_213763.1|	turquoise	1030.2587	1000.5689	0.9961237	0.00028954	152.592
gnl|UG|Ssc#S39994161	turquoise	1030.2263	978.70708	0.9918516	0.00088189	18.004
gnl|UG|Ssc#S39991071	turquoise	1029.9443	999.75142	0.9960829	0.00029412	5.43
gnl|UG|Ssc#S23762974	turquoise	1029.7035	1000.1523	0.9960485	0.000298	22.174
gnl|UG|Ssc#S23776942	turquoise	1029.591	997.64412	0.9958234	0.00032381	2.27
gi|52351462|gb|AY609892.1|	turquoise	1029.3231	993.23296	0.9946851	0.00046476	60.926
gi|194038456|ref|XM_001928767.1|	turquoise	1029.2684	968.8189	0.9893759	0.00131245	45.028
gi|115545921|dbj|AK237362.1|	turquoise	1029.2641	970.84908	0.9898799	0.00122025	71.51
gi|115548519|dbj|AK235136.1|	turquoise	1029.123	998.04235	0.9957486	0.00033255	5.502
gi|115550982|dbj|AK238843.1|	turquoise	1028.9194	975.45271	0.9916069	0.00092188	41.808
gnl|UG|Ssc#S18382407	turquoise	1028.8379	999.53834	0.9958419	0.00032167	28.016
gnl|UG|Ssc#S40479246	turquoise	1028.5451	981.25719	0.9923316	0.00080518	24.002
gi|194038818|ref|XM_001924413.1|	turquoise	1028.5372	998.24502	0.9955336	0.00035808	27.472
gnl|UG|Ssc#S18353913	turquoise	1028.284	993.2345	0.9948395	0.00044467	18.186
gnl|UG|Ssc#S23696834	turquoise	1027.936	997.78642	0.9954366	0.00036981	27.472
gi|194043187|ref|XM_001925978.1|	turquoise	1027.4563	967.06461	0.9890593	0.00137148	43.618
gi|115553331|dbj|AK233129.1|	turquoise	1027.4029	997.49432	0.9953423	0.00038131	4.174
gnl|UG|Ssc#S18556629	turquoise	1027.1451	997.5647	0.9953572	0.0003795	6.568

gi|115546268|dbj|AK240506.1|	turquoise	1026.9669	997.8583	0.9954013	0.0003741	20.668
gnl|UG|Ssc#S40156252	turquoise	1026.8334	990.289	0.9944282	0.00049884	7.922
gnl|UG|Ssc#S17513916	turquoise	1026.5003	997.62191	0.9953409	0.00038149	15.512
gi|194034095|ref|XM_001928221.1|	turquoise	1026.3605	997.16876	0.9953549	0.00037977	2.048
gnl|UG|Ssc#S23764348	turquoise	1026.3198	997.48045	0.9953031	0.00038615	5.26
gi|115548409|dbj|AK235026.1|	turquoise	1026.2641	979.54218	0.9921598	0.00083237	50.956
gnl|UG|Ssc#S18549522	turquoise	1026.0318	973.84372	0.9905859	0.00109494	12.954
gi|115555541|dbj|AK237173.1|	turquoise	1025.5851	977.9242	0.9923291	0.00080557	56.726
gi|115547984|dbj|AK237968.1|	turquoise	1025.5542	996.97966	0.9951566	0.00040434	11.098
gi|115552285|dbj|AK230594.1|	turquoise	1025.2226	990.34461	0.9940561	0.00054961	3.838
gi|115552399|dbj|AK236404.1|	turquoise	1025.163	965.44177	0.9886263	0.00145359	41.906
gnl|UG|Ssc#S40110625	turquoise	1024.9446	993.72575	0.9949055	0.00043618	24.14
gnl|UG|Ssc#S17511933	turquoise	1024.8598	994.91091	0.9946809	0.00046532	12.394
gi|115553811|dbj|AK236816.1|	turquoise	1024.497	982.65347	0.9925527	0.00077064	16.782
gnl|UG|Ssc#S31114563	turquoise	1024.3034	985.62899	0.99353	0.00062412	10.15
gnl|UG|Ssc#S15981318	turquoise	1024.2116	995.81528	0.9948582	0.00044226	4.342
gnl|UG|Ssc#S39985763	turquoise	1024.2116	995.81528	0.9948582	0.00044226	4.342
gnl|UG|Ssc#S40106146	turquoise	1024.2067	991.90546	0.9942466	0.00052342	43.906
gi|52351111|gb|AY609516.1|	turquoise	1023.9811	995.54487	0.9947703	0.00045364	27.104
gnl|UG|Ssc#S17524953	turquoise	1023.9475	994.37466	0.9945688	0.00048009	76.426
gnl|UG|Ssc#S17518818	turquoise	1023.7604	992.67748	0.9941693	0.00053399	4.038
gnl|UG|Ssc#S18274330	turquoise	1023.2561	993.6562	0.994349	0.00050951	6.422
gi|165973437|ref|NM_001113701.1|	turquoise	1023.1638	993.47916	0.9945116	0.00048768	9.342
gi|194044921|ref|XM_001927440.1|	turquoise	1022.9989	959.83669	0.9874482	0.0016849	32.732
gi|115550165|dbj|AK235644.1|	turquoise	1022.9092	991.45755	0.9941631	0.00053484	86.814
gnl|UG|Ssc#S6666043	turquoise	1022.9027	978.95931	0.9913228	0.00096904	15.196
gnl|UG|Ssc#S40158599	turquoise	1022.8611	993.09293	0.9945074	0.00048825	20.512
gi|194043388|ref|XM_001929537.1|	turquoise	1022.7044	980.40933	0.991994	0.00085889	27.896
gi|115553090|dbj|AK239676.1|	turquoise	1022.5823	972.91192	0.9904545	0.00111792	31.068
gi|194034719|ref|XM_001925911.1|	turquoise	1022.0444	993.96877	0.99444	0.00049726	7.748
gi|194044800|ref|XM_001924191.1|	turquoise	1021.7498	971.64151	0.9900222	0.00119463	33.86
gnl|UG|Ssc#S19549069	turquoise	1021.6244	993.7427	0.9943055	0.0005154	18.71
gnl|UG|Ssc#S18382205	turquoise	1021.3124	992.24336	0.9940312	0.00055306	7.754
gi|115547856|dbj|AK234832.1|	turquoise	1021.1391	959.51591	0.9874148	0.00169163	81.33
gnl|UG|Ssc#S6027323	turquoise	1021.0915	993.27409	0.9941673	0.00053427	4.494
gnl|UG|Ssc#S23699793	turquoise	1021.0786	987.05432	0.9932511	0.00066488	25.238
gnl|UG|Ssc#S18378622	turquoise	1020.885	993.04936	0.9941423	0.00053771	2.27
gnl|UG|Ssc#S18383234	turquoise	1020.8841	975.71559	0.9909538	0.00103144	9.706
gnl|UG|Ssc#S40102872	turquoise	1020.8806	993.14143	0.9941454	0.00053728	14.234
gnl|UG|Ssc#S26735527	turquoise	1020.8786	988.65961	0.9934944	0.00062927	18.658
gi|194040321|ref|XM_001928400.1|	turquoise	1020.7194	960.25684	0.9873424	0.00170623	32.332
gnl|UG|Ssc#S17510761	turquoise	1020.6196	991.08732	0.9940142	0.00055543	3.844
gnl|UG|Ssc#S18378125	turquoise	1020.5539	959.24192	0.9868046	0.00181596	11.296
gi|115546238|dbj|AK240476.1|	turquoise	1020.5465	992.40067	0.9941309	0.00053927	7.646
gnl|UG|Ssc#S34533546	turquoise	1020.4096	988.00663	0.9937137	0.00059775	14.986
gi|194035880|ref|XM_001929177.1|	turquoise	1020.3462	992.76429	0.9940676	0.00054802	7.132
gnl|UG|Ssc#S19548366	turquoise	1020.3079	991.66842	0.9937878	0.00058722	4.018
gnl|UG|Ssc#S23695220	turquoise	1020.1021	968.28832	0.9896512	0.00126181	7.1

gi|194018691|ref|NM_001129967.1|	turquoise	1019.7917	984.41637	0.9927553	0.00073942	3.504
gi|115546222|dbj|AK240460.1|	turquoise	1019.7733	990.93018	0.9937225	0.00059649	18.434
gi|115552282|dbj|AK230591.1|	turquoise	1019.7543	984.31166	0.9931136	0.00068529	144.308
gi|194041408|ref|XM_001929007.1|	turquoise	1019.7299	974.88114	0.9909338	0.00103486	1.626
gi|115548230|dbj|AK231444.1|	turquoise	1019.6801	992.16006	0.9938692	0.00057572	22.828
gnl|UG|Ssc#S18555480	turquoise	1019.6169	956.357	0.9861075	0.00196155	3.498
gnl|UG|Ssc#S39987785	turquoise	1019.605	992.10494	0.9938695	0.00057568	9.146
gi|194042943|ref|XM_001929420.1|	turquoise	1019.5814	973.73952	0.9906638	0.00108139	36.58
gnl|UG|Ssc#S18359512	turquoise	1019.4816	990.82433	0.993897	0.00057182	7.834
gnl|UG|Ssc#S31128707	turquoise	1019.4548	991.76031	0.9937797	0.00058837	3.574
gnl|UG|Ssc#S40095449	turquoise	1019.1938	990.56331	0.9935104	0.00062696	7.732
gi|52351898|gb|AY610316.1|	turquoise	1019.0944	991.56379	0.9937292	0.00059553	13.874
gi|194044165|ref|XM_001924403.1|	turquoise	1018.8723	990.52899	0.9935485	0.00062145	4.262
gnl|UG|Ssc#S40483452	turquoise	1018.7788	991.4407	0.9936789	0.00060271	11.952
gnl|UG|Ssc#S17516197	turquoise	1018.7068	991.37132	0.9936743	0.00060337	11.582
gnl|UG|Ssc#S18551640	turquoise	1018.6025	967.05071	0.9886549	0.00144813	12.45
gnl|UG|Ssc#S17516715	turquoise	1018.5348	984.67984	0.9923004	0.00081009	3.69
gnl|UG|Ssc#S18558244	turquoise	1018.5004	984.27424	0.9924808	0.0007818	4.382
gnl|UG|Ssc#S38482329	turquoise	1018.4975	983.29776	0.9925352	0.00077335	43.534
gi|194037369|ref|XM_001925128.1|	turquoise	1018.4453	975.91199	0.9907682	0.00106332	25.518
gi|52352730|gb|AY609650.1|	turquoise	1018.4232	954.26628	0.9860137	0.00198142	39.142
gnl|UG|Ssc#S16514926	turquoise	1018.3039	990.53807	0.9935022	0.00062814	6.36
gnl|UG|Ssc#S6076069	turquoise	1018.0755	987.09185	0.9930257	0.00069843	3.306
gnl|UG|Ssc#S17514582	turquoise	1018.0124	990.80209	0.9935229	0.00062514	14.56
gi|61696631|gb|AY803094.1|	turquoise	1017.9713	985.78475	0.9927438	0.00074119	12.204
gnl|UG|Ssc#S19541978	turquoise	1017.962	988.69281	0.9931235	0.00068381	8.3
gnl|UG|Ssc#S31114805	turquoise	1017.713	986.10621	0.9926075	0.00076215	7.138
gnl|UG|Ssc#S40235024	turquoise	1017.7102	951.81494	0.9851659	0.002164	34.016
gnl|UG|Ssc#S40234755	turquoise	1017.6976	961.93228	0.9877829	0.00161803	4.824
gnl|UG|Ssc#S40476889	turquoise	1017.4228	973.54687	0.9906209	0.00108884	24.82
gi|194038889|ref|XM_001928918.1|	turquoise	1017.3845	977.04033	0.9911576	0.0009968	19.628
gnl|UG|Ssc#S23699327	turquoise	1017.3006	979.72664	0.9923672	0.00079959	16.342
gnl|UG|Ssc#S6010568	turquoise	1017.088	989.89882	0.9933715	0.00064718	4.626
gnl|UG|Ssc#S18553917	turquoise	1017.0867	989.96694	0.9933698	0.00064743	5.37
gi|115554452|dbj|AK233462.1|	turquoise	1016.9955	989.79088	0.9933479	0.00065064	24.05
gnl|UG|Ssc#S18546780	turquoise	1016.6586	982.99872	0.9918482	0.00088244	28.666
gi|48976128|ref|NM_001001770.1|	turquoise	1016.2809	964.59643	0.9878476	0.00160522	119.276
gnl|UG|Ssc#S16769794	turquoise	1016.1949	989.21459	0.9931356	0.000682	4.562
gnl|UG|Ssc#S40084984	turquoise	1015.8414	978.27249	0.9915371	0.00093338	6.046
gi|115550169|dbj|AK235648.1|	turquoise	1015.8347	988.98932	0.9930476	0.00069515	73.526
gi|51592144|ref|NM_001004049.1|	turquoise	1015.7883	988.61172	0.9929632	0.00070785	13.504
gi|115547599|dbj|AK231257.1|	turquoise	1015.275	988.54136	0.9929221	0.00071406	5.008
gi|153791726|ref|NM_001099925.1|	turquoise	1015.275	988.54136	0.9929221	0.00071406	1.828
gi|155369755|ref|NM_001101026.1|	turquoise	1015.275	988.54136	0.9929221	0.00071406	1.15
gi|164394|gb|L10363.1|PIGBSP	turquoise	1015.275	988.54136	0.9929221	0.00071406	1.082
gi|178056541|ref|NM_001123177.1|	turquoise	1015.275	988.54136	0.9929221	0.00071406	2.03
gi|194033899|ref|XM_001928651.1|	turquoise	1015.275	988.54136	0.9929221	0.00071406	0.406
gi|194034057|ref|XM_001924352.1|	turquoise	1015.275	988.54136	0.9929221	0.00071406	3.654

gi|194034272|ref|XM_001924582.1|	turquoise	1015.275	988.54136	0.9929221	0.00071406	10.962
gi|194037687|ref|XM_001927351.1|	turquoise	1015.275	988.54136	0.9929221	0.00071406	2.64
gi|194037914|ref|XM_001927045.1|	turquoise	1015.275	988.54136	0.9929221	0.00071406	4.33
gi|194040347|ref|XM_001926869.1|	turquoise	1015.275	988.54136	0.9929221	0.00071406	0.204
gi|194041706|ref|XM_001926955.1|	turquoise	1015.275	988.54136	0.9929221	0.00071406	3.79
gi|194041956|ref|XM_001929320.1|	turquoise	1015.275	988.54136	0.9929221	0.00071406	10.826
gi|194043865|ref|XM_001928489.1|	turquoise	1015.275	988.54136	0.9929221	0.00071406	2.91
gi|194044574|ref|XM_001928737.1|	turquoise	1015.275	988.54136	0.9929221	0.00071406	5.482
gi|194045058|ref|XM_001925649.1|	turquoise	1015.275	988.54136	0.9929221	0.00071406	3.924
gi|47523699|ref|NM_214317.1|	turquoise	1015.275	988.54136	0.9929221	0.00071406	0.474
gi|47523911|ref|NM_214427.1|	turquoise	1015.275	988.54136	0.9929221	0.00071406	5.076
gi|52351619|gb|AY610049.1|	turquoise	1015.275	988.54136	0.9929221	0.00071406	2.098
gi|56711333|ref|NM_213755.2|	turquoise	1015.275	988.54136	0.9929221	0.00071406	7.308
gi|59709497|ref|NM_001012299.1|	turquoise	1015.275	988.54136	0.9929221	0.00071406	7.512
gi|61696660|gb|AY803123.1|	turquoise	1015.275	988.54136	0.9929221	0.00071406	6.158
gnl|UG|Ssc#S14767272	turquoise	1015.275	988.54136	0.9929221	0.00071406	5.616
gnl|UG|Ssc#S16761546	turquoise	1015.275	988.54136	0.9929221	0.00071406	1.76
gnl|UG|Ssc#S16765575	turquoise	1015.275	988.54136	0.9929221	0.00071406	1.692
gnl|UG|Ssc#S16769802	turquoise	1015.275	988.54136	0.9929221	0.00071406	2.368
gnl|UG|Ssc#S16864702	turquoise	1015.275	988.54136	0.9929221	0.00071406	2.098
gnl|UG|Ssc#S17513155	turquoise	1015.275	988.54136	0.9929221	0.00071406	2.03
gnl|UG|Ssc#S17514665	turquoise	1015.275	988.54136	0.9929221	0.00071406	2.3
gnl|UG|Ssc#S17526184	turquoise	1015.275	988.54136	0.9929221	0.00071406	18.474
gnl|UG|Ssc#S17526944	turquoise	1015.275	988.54136	0.9929221	0.00071406	14.548
gnl|UG|Ssc#S18381113	turquoise	1015.275	988.54136	0.9929221	0.00071406	3.586
gnl|UG|Ssc#S18387120	turquoise	1015.275	988.54136	0.9929221	0.00071406	3.384
gnl|UG|Ssc#S18546109	turquoise	1015.275	988.54136	0.9929221	0.00071406	3.518
gnl|UG|Ssc#S18549381	turquoise	1015.275	988.54136	0.9929221	0.00071406	1.556
gnl|UG|Ssc#S18549877	turquoise	1015.275	988.54136	0.9929221	0.00071406	9.068
gnl|UG|Ssc#S18550181	turquoise	1015.275	988.54136	0.9929221	0.00071406	2.774
gnl|UG|Ssc#S18554224	turquoise	1015.275	988.54136	0.9929221	0.00071406	2.572
gnl|UG|Ssc#S18557364	turquoise	1015.275	988.54136	0.9929221	0.00071406	2.03
gnl|UG|Ssc#S19538625	turquoise	1015.275	988.54136	0.9929221	0.00071406	1.692
gnl|UG|Ssc#S19541682	turquoise	1015.275	988.54136	0.9929221	0.00071406	7.038
gnl|UG|Ssc#S23691231	turquoise	1015.275	988.54136	0.9929221	0.00071406	2.234
gnl|UG|Ssc#S23694918	turquoise	1015.275	988.54136	0.9929221	0.00071406	3.79
gnl|UG|Ssc#S23757708	turquoise	1015.275	988.54136	0.9929221	0.00071406	2.842
gnl|UG|Ssc#S23758306	turquoise	1015.275	988.54136	0.9929221	0.00071406	9.542
gnl|UG|Ssc#S23766263	turquoise	1015.275	988.54136	0.9929221	0.00071406	1.76
gnl|UG|Ssc#S26652967	turquoise	1015.275	988.54136	0.9929221	0.00071406	1.286
gnl|UG|Ssc#S26720448	turquoise	1015.275	988.54136	0.9929221	0.00071406	2.098
gnl|UG|Ssc#S26724210	turquoise	1015.275	988.54136	0.9929221	0.00071406	1.962
gnl|UG|Ssc#S31103357	turquoise	1015.275	988.54136	0.9929221	0.00071406	28.556
gnl|UG|Ssc#S31114631	turquoise	1015.275	988.54136	0.9929221	0.00071406	7.578
gnl|UG|Ssc#S31116516	turquoise	1015.275	988.54136	0.9929221	0.00071406	1.894
gnl|UG|Ssc#S31129032	turquoise	1015.275	988.54136	0.9929221	0.00071406	2.842
gnl|UG|Ssc#S34510310	turquoise	1015.275	988.54136	0.9929221	0.00071406	4.128
gnl|UG|Ssc#S34531531	turquoise	1015.275	988.54136	0.9929221	0.00071406	0.338

gnl|UG|Ssc#S39980686	turquoise	1015.275	988.54136	0.9929221	0.00071406	1.894
gnl|UG|Ssc#S39984816	turquoise	1015.275	988.54136	0.9929221	0.00071406	1.894
gnl|UG|Ssc#S39992206	turquoise	1015.275	988.54136	0.9929221	0.00071406	16.376
gnl|UG|Ssc#S39993541	turquoise	1015.275	988.54136	0.9929221	0.00071406	2.978
gnl|UG|Ssc#S39994288	turquoise	1015.275	988.54136	0.9929221	0.00071406	1.556
gnl|UG|Ssc#S39995011	turquoise	1015.275	988.54136	0.9929221	0.00071406	4.128
gnl|UG|Ssc#S39998520	turquoise	1015.275	988.54136	0.9929221	0.00071406	2.91
gnl|UG|Ssc#S40074903	turquoise	1015.275	988.54136	0.9929221	0.00071406	1.556
gnl|UG|Ssc#S40084501	turquoise	1015.275	988.54136	0.9929221	0.00071406	4.872
gnl|UG|Ssc#S40086007	turquoise	1015.275	988.54136	0.9929221	0.00071406	9.27
gnl|UG|Ssc#S40095231	turquoise	1015.275	988.54136	0.9929221	0.00071406	2.64
gnl|UG|Ssc#S40098899	turquoise	1015.275	988.54136	0.9929221	0.00071406	2.774
gnl|UG|Ssc#S40099212	turquoise	1015.275	988.54136	0.9929221	0.00071406	2.3
gnl|UG|Ssc#S40103595	turquoise	1015.275	988.54136	0.9929221	0.00071406	1.76
gnl|UG|Ssc#S40106548	turquoise	1015.275	988.54136	0.9929221	0.00071406	3.248
gnl|UG|Ssc#S40295633	turquoise	1015.275	988.54136	0.9929221	0.00071406	1.556
gnl|UG|Ssc#S40403544	turquoise	1015.275	988.54136	0.9929221	0.00071406	2.368
gnl|UG|Ssc#S40546896	turquoise	1015.275	988.54136	0.9929221	0.00071406	1.828
gnl|UG|Ssc#S41445227	turquoise	1015.275	988.54136	0.9929221	0.00071406	1.894
gnl|UG|Ssc#S6001862	turquoise	1015.275	988.54136	0.9929221	0.00071406	1.76
gnl|UG|Ssc#S6048273	turquoise	1015.275	988.54136	0.9929221	0.00071406	4.602
gnl|UG|Ssc#S6053423	turquoise	1015.275	988.54136	0.9929221	0.00071406	3.858
gnl|UG|Ssc#S26399010	turquoise	1015.2402	950.1229	0.9845278	0.0023049	22.07
gi|115548328|dbj|AK231701.1|	turquoise	1014.9358	979.2921	0.9916846	0.00090912	46.906
gnl|UG|Ssc#S39998988	turquoise	1014.7372	952.93659	0.9860678	0.00196995	886.026
gi|194042143|ref|XM_001927456.1|	turquoise	1014.2742	980.58683	0.9918761	0.0008779	57.218
gnl|UG|Ssc#S23689835	turquoise	1014.2474	945.67051	0.9835552	0.00252523	15.234
gnl|UG|Ssc#S18554123	turquoise	1014.1711	985.20073	0.9923845	0.00079687	19.42
gnl|UG|Ssc#S34531381	turquoise	1014.1633	986.13471	0.9925596	0.00076955	27.066
gi|194033556|ref|XM_001927696.1|	turquoise	1014.1421	949.7876	0.9851482	0.00216786	31.658
gnl|UG|Ssc#S40156624	turquoise	1013.8825	987.1189	0.9925436	0.00077205	4.85
gnl|UG|Ssc#S19542021	turquoise	1013.824	987.16707	0.9925542	0.0007704	16.682
gnl|UG|Ssc#S40277991	turquoise	1013.7483	967.88807	0.9893073	0.00132515	7.348
gi|178056521|ref|NM_001123111.1|	turquoise	1013.6138	981.55792	0.9921251	0.00083789	6.146
gi|47523553|ref|NM_214240.1|	turquoise	1013.4093	970.31552	0.9894723	0.00129463	42.236
gnl|UG|Ssc#S40079585	turquoise	1013.234	954.31908	0.9866006	0.00185817	29.394
gi|56607105|gb|AY825267.1|	turquoise	1012.7904	984.90968	0.9923366	0.00080439	2.122
gnl|UG|Ssc#S23776700	turquoise	1012.6903	979.1309	0.9910322	0.00101807	16.304
gnl|UG|Ssc#S23690513	turquoise	1012.6304	949.85751	0.9846586	0.00227577	15.752
gi|52351085|gb|AY609490.1|	turquoise	1012.4741	985.96766	0.9922843	0.00081262	7.33
gnl|UG|Ssc#S40496968	turquoise	1012.284	984.13852	0.9923318	0.00080514	6.658
gnl|UG|Ssc#S38480459	turquoise	1011.9945	982.72101	0.9919181	0.00087111	5.358
gnl|UG|Ssc#S19540568	turquoise	1011.7073	985.57899	0.9921675	0.00083115	4.136
gnl|UG|Ssc#S23695110	turquoise	1011.6034	942.88866	0.9832998	0.00258419	13.436
gnl|UG|Ssc#S31115237	turquoise	1011.4911	985.05155	0.9919962	0.00085853	3.97
gnl|UG|Ssc#S39814752	turquoise	1011.3841	958.29817	0.9876465	0.00164518	29.652
gnl|UG|Ssc#S18555277	turquoise	1011.3827	974.46925	0.9903654	0.00113359	23.184
gnl|UG|Ssc#S18380089	turquoise	1011.2517	982.91694	0.991849	0.0008823	4.844

gi|115549219|dbj|AK231895.1|	turquoise	1011.1645	961.77334	0.9872916	0.00171648	29.794
gi|115550647|dbj|AK232477.1|	turquoise	1010.7942	984.75414	0.9919484	0.00086622	5.846
gi|115549454|dbj|AK235476.1|	turquoise	1010.6523	983.441	0.9916434	0.00091586	26.274
gi|194034722|ref|XM_001924603.1|	turquoise	1010.5885	983.49923	0.9917141	0.00090429	6.584
gnl|UG|Ssc#S31118355	turquoise	1010.5082	983.85612	0.99182	0.00088702	6.49
gnl|UG|Ssc#S18557176	turquoise	1010.3225	983.69251	0.9918977	0.00087442	3.058
gnl|UG|Ssc#S40435051	turquoise	1010.3204	984.29283	0.9917963	0.00089088	3.334
gnl|UG|Ssc#S39985253	turquoise	1010.2656	979.92476	0.991044	0.00101606	7.112
gnl|UG|Ssc#S17515868	turquoise	1010.2296	984.19182	0.9918494	0.00088225	12.802
gnl|UG|Ssc#S17511415	turquoise	1010.2212	984.28887	0.9918412	0.00088357	3.122
gnl|UG|Ssc#S40482366	turquoise	1010.063	984.25329	0.9917771	0.00089399	5.238
gi|115552101|dbj|AK239294.1|	turquoise	1009.8323	977.20471	0.991137	0.0010003	49.97
gnl|UG|Ssc#S18558052	turquoise	1009.7947	942.22607	0.9825265	0.00276543	2.434
gi|115550918|dbj|AK238779.1|	turquoise	1009.7169	982.82053	0.9915124	0.00093747	43.584
gnl|UG|Ssc#S19541858	turquoise	1009.6657	983.80123	0.9917181	0.00090363	4.24
gnl|UG|Ssc#S31107257	turquoise	1009.5969	983.74059	0.9917028	0.00090613	2.85
gnl|UG|Ssc#S23768340	turquoise	1009.4796	982.63992	0.9914626	0.00094572	5.518
gnl|UG|Ssc#S31115737	turquoise	1009.4318	983.50024	0.9915942	0.00092397	4.392
gnl|UG|Ssc#S31107757	turquoise	1009.2847	939.36929	0.982027	0.00288464	20.948
gnl|UG|Ssc#S19545319	turquoise	1009.1519	982.91841	0.9914494	0.00094793	10.642
gi|47523799|ref|NM_214372.1|	turquoise	1009.1363	978.30559	0.9907429	0.00106769	427.722
gnl|UG|Ssc#S35169669	turquoise	1009.0091	980.51526	0.9911545	0.00099734	5.868
gi|115545908|dbj|AK234341.1|	turquoise	1008.6368	940.69099	0.9824192	0.0027909	150.458
gi|47522923|ref|NM_214054.1|	turquoise	1008.5258	981.53458	0.9913832	0.00095894	20.262
gnl|UG|Ssc#S18549536	turquoise	1008.3484	939.735	0.9819762	0.00289685	144.09
gnl|UG|Ssc#S19540194	turquoise	1008.1645	980.7226	0.9912093	0.00098808	8.224
gnl|UG|Ssc#S40485684	turquoise	1008.1	981.27091	0.9914059	0.00095516	12.51
gnl|UG|Ssc#S18548853	turquoise	1008.0586	982.3771	0.9913589	0.000963	5.872
gnl|UG|Ssc#S18547054	turquoise	1007.906	982.27758	0.9913196	0.00096956	5.18
gnl|UG|Ssc#S40061661	turquoise	1007.0257	970.0367	0.9902944	0.00114612	15.748
gnl|UG|Ssc#S40475158	turquoise	1006.925	971.48638	0.9892918	0.00132804	14.466
gnl|UG|Ssc#S18554743	turquoise	1006.8641	980.59081	0.9909575	0.0010308	8.01
gnl|UG|Ssc#S23757412	turquoise	1006.6208	953.38294	0.9853678	0.00212003	12.938
gi|194040597|ref|XM_001928150.1|	turquoise	1006.5712	951.21897	0.9851982	0.00215695	27.632
gnl|UG|Ssc#S39997671	turquoise	1006.4286	978.14685	0.9904732	0.00111464	2.124
gi|115548691|dbj|AK231676.1|	turquoise	1006.2326	967.80835	0.9889208	0.00139757	6.458
gnl|UG|Ssc#S40262471	turquoise	1005.9128	980.31875	0.9907929	0.00105905	2.116
gnl|UG|Ssc#S6048180	turquoise	1005.9128	980.31875	0.9907929	0.00105905	2.116
gnl|UG|Ssc#S19549096	turquoise	1005.8975	980.43693	0.9908699	0.0010458	2.886
gnl|UG|Ssc#S16514147	turquoise	1005.7839	949.83705	0.9848387	0.00223588	12.612
gi|113931658|ref|NM_001044598.2|	turquoise	1005.6709	979.15831	0.9905181	0.00110678	16.292
gnl|UG|Ssc#S17526940	turquoise	1005.5968	980.12159	0.9907692	0.00106314	13.998
gi|85838731|gb|DQ351713.1|	turquoise	1005.5938	977.73288	0.9903828	0.00113053	3.138
gnl|UG|Ssc#S19546179	turquoise	1005.5916	974.94444	0.9902709	0.0011503	5.658
gnl|UG|Ssc#S18554456	turquoise	1005.5223	980.09761	0.9907845	0.00106051	1.904
gnl|UG|Ssc#S20958918	turquoise	1005.5223	980.09761	0.9907845	0.00106051	1.904
gnl|UG|Ssc#S40400469	turquoise	1005.4917	979.95704	0.9907187	0.00107187	5.604
gnl|UG|Ssc#S18545724	turquoise	1005.4105	980.17596	0.9907131	0.00107284	4.366

gnl|UG|Ssc#S18359879	turquoise	1005.1479	952.53246	0.9847682	0.00225146	8.16
gi|194034818|ref|XM_001927452.1|	turquoise	1004.7555	974.67652	0.9896374	0.00126432	10.48
gi|115554417|dbj|AK233427.1|	turquoise	1004.4942	976.6231	0.9902954	0.00114595	26.67
gi|47523637|ref|NM_214287.1|	turquoise	1004.4485	979.4456	0.9905177	0.00110685	20.956
gi|52350718|gb|AY609433.1|	turquoise	1004.3314	976.2668	0.9901814	0.00116619	20.37
gnl|UG|Ssc#S19540874	turquoise	1004.3115	976.89739	0.990236	0.00115648	4.2
gnl|UG|Ssc#S15981406	turquoise	1004.245	979.2761	0.9904644	0.00111618	3.336
gi|115551917|dbj|AK236324.1|	turquoise	1004.2057	937.61346	0.9816426	0.0029775	53.486
gi|115549537|dbj|AK235559.1|	turquoise	1004.1557	951.03138	0.9845169	0.00230732	100.382
gnl|UG|Ssc#S17513098	turquoise	1003.7281	967.7204	0.9889418	0.00139361	37.07
gi|48675942|ref|NM_001001640.1|	turquoise	1003.5288	964.19182	0.9877442	0.00162571	16.69
gnl|UG|Ssc#S18354820	turquoise	1003.2555	939.95776	0.9820327	0.00288327	18.226
gi|194041638|ref|XM_001929068.1|	turquoise	1003.2203	977.05132	0.9901878	0.00116505	38.422
gnl|UG|Ssc#S35332961	turquoise	1002.9338	974.88287	0.9895436	0.00128152	25.16
gi|83816988|ref|NM_001037965.1|	turquoise	1002.7111	956.46369	0.9856581	0.00205735	20.262
gi|115549455|dbj|AK235477.1|	turquoise	1002.229	968.93394	0.9885947	0.00145966	97.93
gi|52351432|gb|AY609862.1|	turquoise	1002.1269	961.02819	0.9872424	0.00172644	37.97
gi|194045056|ref|XM_001924869.1|	turquoise	1002.1255	976.78079	0.9898318	0.00122895	6.338
gnl|UG|Ssc#S31103552	turquoise	1002.06	977.188	0.9899453	0.00120847	3.97
gi|115553373|dbj|AK236576.1|	turquoise	1001.4907	964.22493	0.9883167	0.00151328	121.488
gnl|UG|Ssc#S34511666	turquoise	1001.4295	951.22079	0.9848324	0.00223728	15.384
gnl|UG|Ssc#S18361504	turquoise	1001.4042	976.42436	0.9897691	0.00124033	12.076
gnl|UG|Ssc#S39979866	turquoise	1001.2136	970.97323	0.9888908	0.00140325	43.944
gi|194036772|ref|XM_001927760.1|	turquoise	1001.1328	970.24466	0.9889845	0.00138554	36.332
gnl|UG|Ssc#S19542648	turquoise	1000.8024	953.8786	0.9850616	0.00218682	11.532
gi|47523725|ref|NM_214333.1|	turquoise	1000.7048	961.79628	0.9873294	0.00170884	84.924
gnl|UG|Ssc#S17512262	turquoise	1000.5035	975.76545	0.9896239	0.0012668	24.222
gnl|UG|Ssc#S18260225	turquoise	1000.3263	968.16887	0.9885147	0.00147502	23.024
gnl|UG|Ssc#S18354494	turquoise	1000.2417	971.00994	0.988554	0.00146746	48.29
gnl|UG|Ssc#S34517545	turquoise	1000.2395	945.37583	0.9834856	0.00254126	18.954
gnl|UG|Ssc#S18276715	turquoise	1000.1725	926.30956	0.9785357	0.00376276	23.708
gnl|UG|Ssc#S17503531	turquoise	1000.1723	975.20029	0.9895506	0.00128023	2.074
gnl|UG|Ssc#S6043533	turquoise	1000.1723	975.20029	0.9895506	0.00128023	2.074
gi|47523281|ref|NM_213781.1|	turquoise	999.96759	974.98296	0.9894327	0.00130194	4.018
gi|115548164|dbj|AK238148.1|	turquoise	999.92715	972.20729	0.9892834	0.0013296	2.682
gi|115554027|dbj|AK233236.1|	turquoise	999.86727	927.59254	0.9794512	0.0035251	34.198
gnl|UG|Ssc#S40224927	turquoise	999.85874	972.3596	0.9892913	0.00132813	9.006
gnl|UG|Ssc#S39999498	turquoise	999.60729	974.67778	0.9894189	0.00130449	4.084
gi|194038245|ref|XM_001928187.1|	turquoise	999.60438	934.58118	0.9805566	0.00324506	125.396
gnl|UG|Ssc#S18354389	turquoise	999.30501	973.73704	0.989339	0.00131927	9.614
gi|194044069|ref|XM_001925245.1|	turquoise	999.00359	963.53326	0.9877518	0.00162421	13.08
gnl|UG|Ssc#S40000845	turquoise	998.9032	974.02548	0.9892544	0.001335	3.3
gnl|UG|Ssc#S40400100	turquoise	998.71122	973.88794	0.98906	0.00137134	2.846
gnl|UG|Ssc#S18551025	turquoise	998.24326	967.23299	0.988562	0.00146593	31.16
gnl|UG|Ssc#S40082993	turquoise	997.96615	972.32946	0.9886978	0.00143992	7.556
gnl|UG|Ssc#S18555169	turquoise	997.43507	973.32072	0.9889052	0.00140052	3.35
gnl|UG|Ssc#S23699771	turquoise	997.40235	972.42754	0.9887951	0.0014214	19.036
gnl|UG|Ssc#S18357377	turquoise	997.12644	963.92026	0.9879252	0.00158989	10.684

gi|115547488|dbj|AK237870.1|	turquoise	997.00612	920.90076	0.9772748	0.00409835	81.514
gnl|UG|Ssc#S40551411	turquoise	996.87721	948.9409	0.9837075	0.00249029	20.972
gnl|UG|Ssc#S23755372	turquoise	996.83557	967.14581	0.9879563	0.00158376	4.44
gnl|UG|Ssc#S18302191	turquoise	996.81836	960.68471	0.9869137	0.00179352	8.928
gi|194040961|ref|XM_001924920.1|	turquoise	996.76364	937.6273	0.9810016	0.0031345	50.476
gi|52351454|gb|AY609884.1|	turquoise	996.49123	971.22564	0.9887058	0.00143839	79.11
gnl|UG|Ssc#S23692548	turquoise	996.28578	952.4191	0.984908	0.0022206	20.666
gnl|UG|Ssc#S16350384	turquoise	996.27883	959.23841	0.9869814	0.00177963	3.226
gi|58801554|ref|NM_001011727.1|	turquoise	996.10093	937.5117	0.9816107	0.00298524	28.39
gi|52351174|gb|AY609579.1|	turquoise	995.82006	949.52624	0.9841518	0.00238929	14.438
gnl|UG|Ssc#S26722221	turquoise	995.42094	961.41706	0.9874415	0.00168624	6.336
gnl|UG|Ssc#S40242427	turquoise	995.18312	970.9473	0.9883856	0.00149993	2.36
gi|115552265|dbj|AK230574.1|	turquoise	995.16916	962.92145	0.9879683	0.00158139	23.786
gi|115554738|dbj|AK237149.1|	turquoise	995.01034	926.02721	0.9783302	0.00381679	6.994
gnl|UG|Ssc#S19540127	turquoise	994.2368	970.24006	0.9881179	0.00155202	2.672
gi|115548219|dbj|AK231529.1|	turquoise	993.75668	966.72031	0.9875325	0.00166798	73.828
gi|157427727|ref|NM_001105302.1|	turquoise	993.61193	926.33774	0.9786686	0.00372794	30.746
gnl|UG|Ssc#S18552081	turquoise	993.35523	938.1756	0.9819702	0.0028983	13.548
gnl|UG|Ssc#S40483811	turquoise	993.30547	940.65447	0.9820009	0.00289091	5.06
gi|194035253|ref|XM_001925769.1|	turquoise	992.81051	968.20819	0.9877362	0.00162731	3.296
gi|115545916|dbj|AK234349.1|	turquoise	992.31928	919.69974	0.9767243	0.00424783	34.42
gnl|UG|Ssc#S40107966	turquoise	992.30238	951.62168	0.9844589	0.00232028	6.104
gnl|UG|Ssc#S6010002	turquoise	991.9409	930.15021	0.9795514	0.0034994	2.93
gnl|UG|Ssc#S26722459	turquoise	991.83793	935.96263	0.9804995	0.00325934	33.092
gi|115553452|dbj|AK236655.1|	turquoise	991.73411	963.74373	0.9868588	0.00180479	88.468
gi|194033603|ref|XM_001925557.1|	turquoise	991.73161	967.98076	0.9874961	0.00167527	4.882
gnl|UG|Ssc#S17512186	turquoise	991.65536	967.62253	0.9874736	0.00167979	23.638
gi|115550714|dbj|AK235788.1|	turquoise	990.28328	965.54853	0.9870059	0.00177461	5.198
gnl|UG|Ssc#S21579411	turquoise	990.16149	927.77457	0.9791646	0.00359894	13.426
gnl|UG|Ssc#S40440719	turquoise	990.06414	959.08319	0.9861717	0.00194798	12.546
gi|115551189|dbj|AK232622.1|	turquoise	989.78525	966.60166	0.9870947	0.00175648	17.62
gnl|UG|Ssc#S22274163	turquoise	989.73881	964.68091	0.9869998	0.00177587	11.53
gnl|UG|Ssc#S23691229	turquoise	989.33575	915.3615	0.9762133	0.00438813	25.998
gnl|UG|Ssc#S18552433	turquoise	989.29428	946.58273	0.9831466	0.00261977	46.104
gi|194041065|ref|XM_001928989.1|	turquoise	989.13297	918.60255	0.9771823	0.00412333	40.422
gi|194038478|ref|XM_001926160.1|	turquoise	988.36675	938.7453	0.9810149	0.00313122	26.018
gi|194038579|ref|XM_001926122.1|	turquoise	988.29637	945.32194	0.9838939	0.00244776	44.972
gnl|UG|Ssc#S40155274	turquoise	988.28136	964.87984	0.9866998	0.00183762	3.338
gi|47523085|ref|NM_214144.1|	turquoise	987.97908	952.90171	0.9842127	0.00237555	32.086
gnl|UG|Ssc#S31116211	turquoise	987.63408	960.73372	0.9867017	0.00183721	3.682
gnl|UG|Ssc#S18272610	turquoise	987.52854	964.26549	0.9865505	0.0018686	5.016
gi|157427723|ref|NM_001105300.1|	turquoise	987.42229	954.18023	0.985368	0.00211999	49.312
gi|47522647|ref|NM_213921.1|	turquoise	987.14501	939.23023	0.9823131	0.00281616	18.928
gnl|UG|Ssc#S26719371	turquoise	986.70804	959.44581	0.9854639	0.00209922	69.496
gi|115551497|dbj|AK238961.1|	turquoise	986.6553	932.45863	0.9801195	0.00335487	189.538
gi|194033897|ref|XM_001926189.1|	turquoise	986.64782	910.89225	0.9747889	0.00478711	17.652
gnl|UG|Ssc#S6073975	turquoise	986.51657	923.94172	0.9774625	0.00404781	35.148
gnl|UG|Ssc#S18355850	turquoise	986.31537	935.19521	0.9807557	0.00319544	20.83

gnl|UG|Ssc#S18552515	turquoise	986.27273	960.42574	0.9862603	0.00192932	21.318
gi|194041876|ref|XM_001926725.1|	turquoise	986.02269	954.02238	0.9851124	0.00217569	9.634
gi|194035410|ref|XM_001926931.1|	turquoise	985.98499	951.76638	0.9854927	0.00209298	7.038
gi|47523869|ref|NM_214408.1|	turquoise	985.97694	944.52663	0.9823023	0.00281874	55.982
gi|47522769|ref|NM_213971.1|	turquoise	985.96493	961.21548	0.9860428	0.00197524	44.516
gi|155369765|ref|NM_001101031.1|	turquoise	985.93512	954.12188	0.9861006	0.00196299	17.662
gnl|UG|Ssc#S21554547	turquoise	985.91315	908.03434	0.9742397	0.00494396	8.088
gnl|UG|Ssc#S26721293	turquoise	985.39484	913.05896	0.9752718	0.00465056	2.606
gi|47523733|ref|NM_214337.1|	turquoise	985.33846	960.16069	0.9857277	0.00204242	6.382
gi|52351191|gb|AY609596.1|	turquoise	984.89162	905.65811	0.9730065	0.0053022	17.868
gnl|UG|Ssc#S6076952	turquoise	984.54867	915.02968	0.9754419	0.00460276	19.472
gi|115547230|dbj|AK234603.1|	turquoise	983.74473	959.48113	0.9855444	0.00208182	158.714
gnl|UG|Ssc#S6011299	turquoise	983.66861	950.37259	0.98366	0.00250119	39.954
gnl|UG|Ssc#S18381906	turquoise	982.57748	950.19722	0.9846629	0.00227482	3.62
gnl|UG|Ssc#S19542887	turquoise	982.44692	958.51992	0.985326	0.00212911	1.706
gnl|UG|Ssc#S19545994	turquoise	982.01523	949.30101	0.9843732	0.00233947	5.2
gnl|UG|Ssc#S26722332	turquoise	981.95293	953.38271	0.9842154	0.00237494	12.402
gnl|UG|Ssc#S16515995	turquoise	981.6427	956.67613	0.9847975	0.00224499	7.114
gnl|UG|Ssc#S22271795	turquoise	981.28257	938.78086	0.9807581	0.00319484	39.95
gi|194044267|ref|XM_001925000.1|	turquoise	981.05913	946.01566	0.9828658	0.00268541	4.336
gi|194043189|ref|XM_001928908.1|	turquoise	980.96105	955.02164	0.9844188	0.00232927	16.446
gnl|UG|Ssc#S40588179	turquoise	980.85329	941.11752	0.9819756	0.00289699	32.176
gi|115551787|dbj|AK236193.1|	turquoise	980.79859	954.87692	0.9843812	0.00233768	15.072
gnl|UG|Ssc#S18556357	turquoise	980.76099	900.7644	0.9716526	0.00570492	39.96
gnl|UG|Ssc#S31133980	turquoise	980.3261	956.41672	0.9845251	0.0023055	14.608
gi|194033596|ref|XM_001927813.1|	turquoise	979.70311	943.88994	0.9820609	0.00287648	19.946
gi|194043291|ref|XM_001928076.1|	turquoise	979.53839	941.31352	0.9812613	0.00307057	127.152
gi|115545756|dbj|AK234189.1|	turquoise	979.32948	904.48142	0.9729634	0.00531487	8.492
gnl|UG|Ssc#S18548111	turquoise	979.32239	920.16782	0.9770036	0.00417177	24.356
gi|113205585|ref|NM_001044535.1|	turquoise	978.83533	945.89675	0.982592	0.00274992	25.992
gnl|UG|Ssc#S19541038	turquoise	978.78487	946.43449	0.9829373	0.00266864	38.886
gi|194042829|ref|XM_001928949.1|	turquoise	978.38693	915.41922	0.9767841	0.0042315	28.548
gnl|UG|Ssc#S23690544	turquoise	978.1065	933.26135	0.9820773	0.00287256	23.3
gnl|UG|Ssc#S18378972	turquoise	978.01983	921.9896	0.9777907	0.00395991	67.7
gnl|UG|Ssc#S18546322	turquoise	977.99286	918.48783	0.9769811	0.00417788	1.396
gi|194040931|ref|XM_001926443.1|	turquoise	977.97046	899.49449	0.9714472	0.00576684	15.964
gi|194041032|ref|XM_001925741.1|	turquoise	977.86911	928.52054	0.9783845	0.00380251	42.558
gnl|UG|Ssc#S17518307	turquoise	977.86101	925.38854	0.9786728	0.00372684	32.544
gnl|UG|Ssc#S19543418	turquoise	977.72331	903.60418	0.9726281	0.00541378	51.418
gi|113205675|ref|NM_001044558.1|	turquoise	977.38801	922.92025	0.9782425	0.00383994	32.088
gnl|UG|Ssc#S31130453	turquoise	976.82711	895.82629	0.970339	0.00610478	8.168
gi|115549299|dbj|AK235220.1|	turquoise	976.75623	920.23027	0.9772939	0.0040932	40.684
gnl|UG|Ssc#S22275831	turquoise	976.48054	943.32963	0.9830973	0.00263127	1.642
gi|115552144|dbj|AK239337.1|	turquoise	976.20682	926.60113	0.9783702	0.00380626	9.232
gnl|UG|Ssc#S17518691	turquoise	976.14704	950.42933	0.9833541	0.00257162	4.056
gnl|UG|Ssc#S23761842	turquoise	976.13925	939.02918	0.9817848	0.00294304	9.24
gnl|UG|Ssc#S19545458	turquoise	976.12481	953.91418	0.9838873	0.00244925	22.266
gi|115550390|dbj|AK238655.1|	turquoise	975.81221	949.49313	0.983251	0.00259551	28.56

gnl|UG|Ssc#S17526742	turquoise	975.73564	953.74503	0.9837049	0.00249089	19.066
gi|194043951|ref|XM_001927705.1|	turquoise	975.36625	914.28188	0.9750075	0.00472513	28.438
gnl|UG|Ssc#S40442649	turquoise	975.14634	946.67796	0.9825281	0.00276505	3.18
gi|115552977|dbj|AK239563.1|	turquoise	975.12629	897.41653	0.9708026	0.00596265	95.47
gnl|UG|Ssc#S18551519	turquoise	974.29843	944.47019	0.9835705	0.00252171	19.76
gnl|UG|Ssc#S40324300	turquoise	973.86377	937.88279	0.9816232	0.0029822	37.14
gnl|UG|Ssc#S31131596	turquoise	973.52536	889.32252	0.9689079	0.00655047	0.738
gnl|UG|Ssc#S17517636	turquoise	973.51683	945.80846	0.9827056	0.00272309	2.944
gnl|UG|Ssc#S22313154	turquoise	973.15885	901.10404	0.9722407	0.00552878	381.264
gi|115555306|dbj|AK233921.1|	turquoise	972.98919	929.04197	0.9794995	0.00351269	29.856
gnl|UG|Ssc#S26713936	turquoise	972.87106	949.10829	0.9829709	0.00266078	37.948
gnl|UG|Ssc#S18551801	turquoise	972.49451	908.01862	0.9742284	0.00494719	143.614
gi|52351444|gb|AY609874.1|	turquoise	972.2367	889.25636	0.9685321	0.00666923	147.482
gnl|UG|Ssc#S40559315	turquoise	971.44413	931.72888	0.9790057	0.00364011	6.916
gi|115549306|dbj|AK235227.1|	turquoise	971.38271	942.26309	0.9818285	0.00293246	5.506
gnl|UG|Ssc#S18553743	turquoise	970.97425	945.88302	0.9823505	0.00280725	10.072
gnl|UG|Ssc#S23771863	turquoise	969.96943	947.56864	0.9824076	0.00279365	1.804
gi|194038838|ref|XM_001927354.1|	turquoise	969.62323	920.84089	0.9775283	0.00403013	20.368
gi|190360622|ref|NM_001128462.1|	turquoise	969.62321	922.65769	0.9777094	0.00398162	21.844
gnl|UG|Ssc#S40091470	turquoise	969.4205	935.66051	0.980597	0.00323496	34.294
gnl|UG|Ssc#S19549267	turquoise	969.20815	944.38081	0.9815642	0.00299654	6.446
gnl|UG|Ssc#S18384560	turquoise	968.8272	946.97926	0.9821059	0.00286569	9.06
gnl|UG|Ssc#S40206941	turquoise	968.78967	886.89824	0.9678563	0.00688451	53.662
gnl|UG|Ssc#S17510489	turquoise	968.08558	889.35541	0.9686512	0.00663152	34.544
gi|115546736|dbj|AK237582.1|	turquoise	967.68062	918.84001	0.9779268	0.00392363	18.184
gnl|UG|Ssc#S18274097	turquoise	967.47672	909.16023	0.9752929	0.00464463	60.676
gnl|UG|Ssc#S40084519	turquoise	967.26579	943.62892	0.9818334	0.00293129	11.74
gnl|UG|Ssc#S23700108	turquoise	966.56566	941.45418	0.9815935	0.00298942	8.45
gnl|UG|Ssc#S18546390	turquoise	966.54306	905.28576	0.9730237	0.00529715	81.876
gi|115550708|dbj|AK235782.1|	turquoise	966.44808	886.69045	0.9676545	0.00694921	47.76
gnl|UG|Ssc#S40477070	turquoise	966.39883	944.57291	0.9814917	0.00301421	2.688
gnl|UG|Ssc#S19545891	turquoise	965.94277	919.97049	0.9763316	0.00435551	33.762
gnl|UG|Ssc#S18553277	turquoise	965.52835	879.91192	0.9664436	0.00734174	5.802
gnl|UG|Ssc#S18379340	turquoise	965.09878	936.4277	0.9809871	0.00313807	5.36
gnl|UG|Ssc#S40050544	turquoise	964.89956	914.78276	0.9747825	0.00478891	22.014
gi|115551842|dbj|AK236249.1|	turquoise	964.7607	901.6713	0.9725256	0.00544412	85.824
gnl|UG|Ssc#S40483502	turquoise	964.65197	917.06995	0.9752052	0.00466931	14.822
gnl|UG|Ssc#S23756925	turquoise	964.52531	939.43215	0.9808335	0.00317611	26.34
gnl|UG|Ssc#S40077859	turquoise	964.38044	943.10335	0.9808194	0.00317961	2.048
gi|194034896|ref|XM_001924578.1|	turquoise	964.31274	916.17721	0.9767409	0.0042433	38.24
gi|194039565|ref|XM_001929124.1|	turquoise	963.86225	931.57998	0.9797669	0.00344434	29.388
gnl|UG|Ssc#S17525783	turquoise	963.5163	940.98484	0.9807165	0.00320519	5.376
gnl|UG|Ssc#S17517257	turquoise	963.13631	941.56132	0.9807639	0.00319339	5.258
gnl|UG|Ssc#S17511240	turquoise	963.00594	898.31101	0.9714388	0.00576939	105.314
gnl|UG|Ssc#S17514578	turquoise	962.56414	921.90989	0.9787229	0.00371375	14.476
gnl|UG|Ssc#S23771589	turquoise	962.54411	940.19326	0.9805524	0.0032461	4.488
gi|115545542|dbj|AK230657.1|	turquoise	962.46729	935.74457	0.9805801	0.00323917	125.724
gnl|UG|Ssc#S34521876	turquoise	962.32861	885.25705	0.9677172	0.00692909	10.49

gnl|UG|Ssc#S18354218	turquoise	962.31624	889.09571	0.9699765	0.0062167	10.932
gnl|UG|Ssc#S40102974	turquoise	962.21459	936.2527	0.9793187	0.00355917	10.07
gnl|UG|Ssc#S40094835	turquoise	962.21064	938.39132	0.9805622	0.00324365	5.972
gnl|UG|Ssc#S18558087	turquoise	962.15649	879.59443	0.965731	0.00757603	11.54
gi|148228719|ref|NM_001097439.1|	turquoise	962.08645	901.17202	0.9711807	0.00584753	124.864
gi|156120135|ref|NM_001101827.1|	turquoise	961.87615	885.47051	0.9675485	0.00698329	55.384
gi|115550806|dbj|AK235881.1|	turquoise	961.74885	881.4552	0.9665865	0.00729504	107.178
gnl|UG|Ssc#S40048913	turquoise	961.53502	933.49208	0.9796392	0.00347692	20.554
gnl|UG|Ssc#S17524702	turquoise	961.1495	873.59655	0.9642402	0.0080739	36.516
gnl|UG|Ssc#S40291311	turquoise	960.65716	902.71529	0.9743656	0.00490785	3.322
gi|194044564|ref|XM_001925248.1|	turquoise	960.04182	907.49536	0.9737118	0.00509631	26.896
gi|115548319|dbj|AK231543.1|	turquoise	959.96519	920.1576	0.9771089	0.00414321	63.826
gnl|UG|Ssc#S23695348	turquoise	959.84911	925.30299	0.9777316	0.00397568	10.366
gi|115545949|dbj|AK237390.1|	turquoise	959.77753	925.31983	0.9768476	0.00421419	49.372
gnl|UG|Ssc#S18267647	turquoise	959.36055	936.3853	0.9796484	0.00347458	63.162
gnl|UG|Ssc#S19546257	turquoise	958.90929	879.52845	0.9658639	0.00753215	83.344
gnl|UG|Ssc#S19548284	turquoise	958.8691	933.66743	0.9798359	0.00342675	11.788
gnl|UG|Ssc#S39979082	turquoise	958.81076	879.97172	0.9664589	0.00733673	99.526
gnl|UG|Ssc#S40475596	turquoise	958.65362	932.20663	0.9799677	0.00339329	8.742
gi|194037750|ref|XM_001926886.1|	turquoise	957.38947	931.71897	0.979491	0.00351487	4.076
gnl|UG|Ssc#S40108865	turquoise	957.37676	931.97015	0.9797574	0.00344676	36.356
gnl|UG|Ssc#S17518997	turquoise	957.09012	935.87758	0.9790444	0.00363006	14.046
gi|115551534|dbj|AK238998.1|	turquoise	956.93983	909.49813	0.9741974	0.00495612	50.304
gi|115554129|dbj|AK233339.1|	turquoise	956.80346	900.34095	0.9717726	0.00566883	34.04
gi|115548956|dbj|AK235359.1|	turquoise	956.65857	867.00309	0.9624796	0.00867514	125.804
gi|115551903|dbj|AK236310.1|	turquoise	954.9754	922.52188	0.9769924	0.0041748	59.972
gnl|UG|Ssc#S23689779	turquoise	954.58865	933.11023	0.9787055	0.00371828	7.016
gi|115553103|dbj|AK239689.1|	turquoise	954.37777	879.66841	0.9662107	0.00741804	55.734
gi|115546201|dbj|AK240439.1|	turquoise	953.98151	918.0539	0.9762105	0.0043889	48.394
gnl|UG|Ssc#S17525847	turquoise	953.48249	931.39688	0.977871	0.00393849	2.154
gi|194042295|ref|XM_001928522.1|	turquoise	953.46099	912.29831	0.974454	0.00488255	90.556
gi|194043076|ref|XM_001925356.1|	turquoise	953.14224	886.49092	0.9679782	0.0068455	46.92
gnl|UG|Ssc#S6024301	turquoise	953.09708	887.76634	0.9682913	0.00674568	5.282
gi|194036698|ref|XM_001928463.1|	turquoise	951.90671	908.57538	0.9727738	0.00537073	11.616
gi|201066355|ref|NM_001134967.1|	turquoise	951.77097	912.09284	0.9743203	0.00492085	4.648
gi|194034434|ref|XM_001925498.1|	turquoise	951.16728	926.73074	0.9776598	0.00399489	3.85
gnl|UG|Ssc#S40478547	turquoise	951.00056	931.13529	0.9776256	0.00400402	6.018
gi|115550946|dbj|AK238807.1|	turquoise	950.84465	859.99678	0.9605845	0.00933791	83.75
gi|115550154|dbj|AK235633.1|	turquoise	950.43078	917.99505	0.9752177	0.00466581	10.114
gi|115548572|dbj|AK231556.1|	turquoise	950.2353	869.42075	0.9628146	0.00855964	63.114
gnl|UG|Ssc#S23760498	turquoise	949.97711	922.26892	0.9775311	0.00402936	36.974
gnl|UG|Ssc#S40494421	turquoise	949.75078	916.50798	0.9756229	0.00455212	21.23
gi|115548424|dbj|AK235041.1|	turquoise	948.72648	907.49133	0.9740266	0.00500528	51.542
gnl|UG|Ssc#S23763835	turquoise	948.71859	924.33967	0.9766256	0.00427479	25.2
gnl|UG|Ssc#S18357401	turquoise	948.59857	922.25859	0.9761083	0.00441715	6.064
gnl|UG|Ssc#S23701220	turquoise	948.41396	927.81243	0.9771766	0.00412488	3.768
gnl|UG|Ssc#S40334954	turquoise	947.91462	858.19935	0.9598162	0.00961116	36.704
gnl|UG|Ssc#S40340827	turquoise	946.91902	875.04208	0.9645008	0.00798613	58.39

gi|115554680|dbj|AK237091.1|	turquoise	946.68146	870.73286	0.9632368	0.0084148	23.008
gnl|UG|Ssc#S16769740	turquoise	946.22783	923.80581	0.9766577	0.00426602	15.08
gnl|UG|Ssc#S22289618	turquoise	946.07734	857.25324	0.9596785	0.00966039	12.69
gnl|UG|Ssc#S26734197	turquoise	945.86744	924.58209	0.9760079	0.00444496	6.016
gi|115552353|dbj|AK232758.1|	turquoise	945.52849	887.2982	0.9699522	0.00622424	20.234
gi|47522871|ref|NM_214025.1|	turquoise	945.4097	920.3824	0.9750406	0.00471577	5.054
gi|47523343|ref|NM_213746.1|	turquoise	945.32244	915.70117	0.9740501	0.0049985	3.53
gnl|UG|Ssc#S23697986	turquoise	944.73766	920.46243	0.9754199	0.00460895	44.174
gnl|UG|Ssc#S6076809	turquoise	943.69969	906.17107	0.9729105	0.00533044	23.526
gi|52351807|gb|AY610225.1|	turquoise	942.67399	882.96236	0.9670367	0.00714861	19.46
gnl|UG|Ssc#S18356106	turquoise	942.40786	849.63186	0.9576846	0.01038259	75.596
gnl|UG|Ssc#S23701146	turquoise	942.14352	921.18777	0.9750949	0.00470041	5.324
gi|194035578|ref|XM_001928185.1|	turquoise	942.06292	921.53967	0.9751658	0.00468041	6.774
gnl|UG|Ssc#S18260201	turquoise	941.86205	862.22228	0.9617232	0.00893776	7.898
gi|47523931|ref|NM_214440.1|	turquoise	941.43645	920.2795	0.9754519	0.00459998	17.064
gnl|UG|Ssc#S29971660	turquoise	940.2228	911.82879	0.9732515	0.00523037	6.14
gi|115554713|dbj|AK237124.1|	turquoise	940.1801	918.96285	0.9753098	0.00463988	17.886
gi|115554532|dbj|AK233542.1|	turquoise	940.07026	915.8584	0.9749321	0.00474647	121.028
gnl|UG|Ssc#S19544740	turquoise	939.6962	912.80353	0.9756388	0.00454766	11.026
gnl|UG|Ssc#S40524341	turquoise	939.2855	857.94241	0.960704	0.00929566	12.452
gnl|UG|Ssc#S18359073	turquoise	938.46978	914.48639	0.9740481	0.00499909	43.462
gnl|UG|Ssc#S29990650	turquoise	938.13859	861.30314	0.9617333	0.00893423	59.062
gnl|UG|Ssc#S18555410	turquoise	938.04079	849.17068	0.9572839	0.01052976	56.584
gi|115553451|dbj|AK236654.1|	turquoise	937.85289	894.06117	0.9694252	0.00638818	44.298
gnl|UG|Ssc#S18357529	turquoise	937.83744	858.92929	0.960262	0.00945229	3.152
gi|115545734|dbj|AK234167.1|	turquoise	937.78621	918.32458	0.974139	0.00497289	4.532
gnl|UG|Ssc#S17517701	turquoise	937.47394	899.46533	0.9705728	0.00603298	6.036
gnl|UG|Ssc#S26717691	turquoise	937.42683	917.58865	0.9741277	0.00497614	12.136
gi|115554303|dbj|AK240098.1|	turquoise	937.38077	894.06727	0.9695596	0.00634624	27.968
gi|52352721|gb|AY609641.1|	turquoise	937.34945	916.65514	0.9742539	0.00493989	7.246
gi|115547803|dbj|AK234779.1|	turquoise	937.33308	885.01949	0.9668265	0.00721684	117.212
gi|194037670|ref|XM_001924938.1|	turquoise	936.80229	903.41205	0.972807	0.00536093	22.158
gi|115548801|dbj|AK231759.1|	turquoise	935.9682	912.0473	0.9733522	0.00520096	54.002
gnl|UG|Ssc#S6015201	turquoise	935.70873	900.3394	0.9730666	0.00528454	8.2
gnl|UG|Ssc#S40078819	turquoise	935.39564	897.67853	0.9702362	0.00613647	26.97
gi|194036099|ref|XM_001929400.1|	turquoise	935.28119	877.78364	0.9662659	0.00739994	16.186
gi|115549831|dbj|AK238491.1|	turquoise	934.42259	914.12004	0.973253	0.00522995	4.458
gi|47523211|ref|NM_213815.1|	turquoise	933.92389	891.57246	0.9702777	0.00612367	2.884
gnl|UG|Ssc#S23763197	turquoise	933.90399	859.87062	0.9606369	0.00931937	7.548
gi|115552005|dbj|AK239198.1|	turquoise	933.66421	852.75723	0.9579483	0.01028608	19.874
gnl|UG|Ssc#S40100108	turquoise	931.98051	848.43981	0.9577998	0.0103404	10.376
gnl|UG|Ssc#S17524908	turquoise	931.58999	848.87936	0.957871	0.01031436	10.904
gi|194033624|ref|XM_001924823.1|	turquoise	931.33486	900.00188	0.971405	0.0057796	8.554
gi|115547445|dbj|AK237827.1|	turquoise	930.15835	888.45812	0.9677235	0.00692708	1112.53
gnl|UG|Ssc#S17512292	turquoise	929.53916	880.88793	0.9652072	0.00774975	45.93
gnl|UG|Ssc#S6081526	turquoise	928.68659	840.42611	0.9547408	0.0114795	22.098
gnl|UG|Ssc#S35167446	turquoise	928.25823	910.03478	0.9717728	0.00566877	13.728
gnl|UG|Ssc#S22286222	turquoise	928.05559	885.78017	0.9670417	0.00714698	45.22

gnl|UG|Ssc#S38482262	turquoise	927.99461	887.77877	0.9694245	0.00638841	31.966
gi|52351303|gb|AY609735.1|	turquoise	925.26053	901.09911	0.9700576	0.0061916	31.114
gnl|UG|Ssc#S24610495	turquoise	924.89498	889.12315	0.96936	0.00640858	23.462
gi|115550155|dbj|AK235634.1|	turquoise	924.66812	851.83206	0.958659	0.01002753	57.382
gnl|UG|Ssc#S19541195	turquoise	923.76155	901.48889	0.9713143	0.00580705	4.594
gi|115550178|dbj|AK235657.1|	turquoise	922.46488	845.46399	0.9573505	0.01050527	28.56
gnl|UG|Ssc#S26714922	turquoise	922.21904	873.59719	0.9642116	0.00808355	32.302
gi|194044985|ref|XM_001927811.1|	turquoise	921.655	856.43925	0.9606341	0.00932036	3.766
gnl|UG|Ssc#S40182904	turquoise	921.61897	885.10315	0.9680042	0.00683721	17.326
gnl|UG|Ssc#S18377961	turquoise	921.5411	871.41278	0.9629854	0.00850094	40.91
gi|194037857|ref|XM_001927835.1|	turquoise	921.29872	824.6001	0.9502334	0.01322732	18.744
gi|115550758|dbj|AK235833.1|	turquoise	921.2634	824.14969	0.9502412	0.01322422	63.248
gnl|UG|Ssc#S40482579	turquoise	920.23122	869.12812	0.9646324	0.0079419	73.338
gnl|UG|Ssc#S18550647	turquoise	918.3725	898.83914	0.9692911	0.00643013	2.952
gnl|UG|Ssc#S23763192	turquoise	918.26331	899.30444	0.9690231	0.00651423	4.638
gnl|UG|Ssc#S18381810	turquoise	917.95628	892.8639	0.9703001	0.00611678	22.246
gi|194033859|ref|XM_001927866.1|	turquoise	917.80042	899.39268	0.9691264	0.00648177	91.778
gi|47523717|ref|NM_214328.1|	turquoise	917.23088	899.20926	0.9687918	0.0065871	8.388
gi|194043439|ref|XM_001927481.1|	turquoise	916.73475	830.75587	0.9536804	0.01188341	19.768
gi|115552159|dbj|AK239352.1|	turquoise	915.86402	821.91618	0.9495287	0.01350781	25.198
gnl|UG|Ssc#S18548520	turquoise	914.50089	874.10119	0.963882	0.00819507	19.714
gnl|UG|Ssc#S26728690	turquoise	914.40748	895.4106	0.9680501	0.00682254	24.066
gi|194042745|ref|XM_001927026.1|	turquoise	913.9453	893.2038	0.9673898	0.00703441	4.16
gnl|UG|Ssc#S6002981	turquoise	911.67334	880.04019	0.9643196	0.0080471	41.502
gnl|UG|Ssc#S34510284	turquoise	910.62646	863.01308	0.9614602	0.00902967	5.196
gi|194042440|ref|XM_001928136.1|	turquoise	910.26584	859.52195	0.9600797	0.00951713	64.116
gnl|UG|Ssc#S23764031	turquoise	910.12461	889.8094	0.9671628	0.00710777	3.39
gi|194035243|ref|XM_001927441.1|	turquoise	909.51437	841.96884	0.9547948	0.01145907	55.388
gnl|UG|Ssc#S40287461	turquoise	909.46095	868.87773	0.9629339	0.00851862	19.038
gnl|UG|Ssc#S18558299	turquoise	909.07077	836.36039	0.954506	0.01156855	78.936
gnl|UG|Ssc#S17516031	turquoise	908.91581	819.77081	0.9485354	0.01390643	2.646
gi|194034892|ref|XM_001929418.1|	turquoise	907.73808	824.88363	0.950512	0.01311695	13.922
gi|115545785|dbj|AK234218.1|	turquoise	907.73713	859.83801	0.9634239	0.00835091	27.838
gnl|UG|Ssc#S23697660	turquoise	907.52489	817.50073	0.9477441	0.01422665	20.612
gnl|UG|Ssc#S29979056	turquoise	906.66242	885.49112	0.9657556	0.00756789	4.896
gi|115545566|dbj|AK230681.1|	turquoise	906.27893	871.15119	0.9619899	0.00884487	59.328
gnl|UG|Ssc#S40095003	turquoise	906.08056	884.04115	0.9658782	0.00752742	6.056
gnl|UG|Ssc#S18383171	turquoise	905.94787	872.57309	0.9628358	0.00855235	7.318
gi|115546696|dbj|AK234534.1|	turquoise	905.77571	851.34412	0.9595967	0.00968968	51.03
gi|52351397|gb|AY609827.1|	turquoise	905.08282	882.71422	0.9665731	0.00729941	10.778
gnl|UG|Ssc#S18360447	turquoise	904.85445	879.33437	0.9646123	0.00794865	7.82
gnl|UG|Ssc#S26734209	turquoise	904.19724	882.99946	0.9646396	0.00793948	3.45
gi|115548955|dbj|AK235358.1|	turquoise	904.06126	807.05016	0.9454098	0.01518508	103.698
gi|115550874|dbj|AK235949.1|	turquoise	903.96231	808.29149	0.9454254	0.01517863	59.524
gi|115547217|dbj|AK234590.1|	turquoise	903.15584	883.77565	0.965699	0.00758658	2.006
gi|194034997|ref|XM_001928355.1|	turquoise	903.04142	866.71157	0.9643834	0.00802561	29.998
gnl|UG|Ssc#S40104089	turquoise	902.82404	855.68375	0.9621825	0.00877799	35.496
gnl|UG|Ssc#S19540774	turquoise	902.79811	845.95634	0.9558676	0.0110554	41.732

gi|47523673|ref|NM_214308.1|	turquoise	902.73002	871.05672	0.9645473	0.00797049	64.438
gi|115550357|dbj|AK238622.1|	turquoise	902.6405	808.23272	0.9467037	0.01465131	73.38
gi|115549359|dbj|AK235381.1|	turquoise	902.26604	872.4691	0.962165	0.00878405	22.288
gnl|UG|Ssc#S27598964	turquoise	901.97795	876.77367	0.9644922	0.00798901	8.192
gnl|UG|Ssc#S40039005	turquoise	901.77699	879.59459	0.9656225	0.0076119	13.762
gnl|UG|Ssc#S31133665	turquoise	901.0909	879.16414	0.964877	0.00785997	12.476
gnl|UG|Ssc#S26723882	turquoise	900.6967	855.10817	0.9584816	0.01009187	6.714
gnl|UG|Ssc#S40573713	turquoise	900.25959	795.73396	0.9420732	0.01658987	7.302
gi|115552766|dbj|AK239359.1|	turquoise	899.9761	795.21022	0.9418801	0.01667241	40.078
gnl|UG|Ssc#S40437415	turquoise	899.416	809.97146	0.9460308	0.01492815	29.024
gnl|UG|Ssc#S23696033	turquoise	898.86635	854.77189	0.9600265	0.00953609	14.614
gi|194044570|ref|XM_001928614.1|	turquoise	898.53329	821.49869	0.9495532	0.01349802	23.13
gi|47523543|ref|NM_214230.1|	turquoise	897.61833	804.04522	0.9440691	0.01574467	47.94
gnl|UG|Ssc#S26720630	turquoise	897.43114	860.03869	0.9591974	0.00983307	19.7
gi|194038454|ref|XM_001928679.1|	turquoise	896.69977	874.60763	0.9624634	0.00868073	11.58
gi|52351429|gb|AY609859.1|	turquoise	896.58809	874.78947	0.9631118	0.0084576	18.31
gi|178056780|ref|NM_001123122.1|	turquoise	895.27719	840.99829	0.9546481	0.01151463	43.326
gnl|UG|Ssc#S40014860	turquoise	892.56861	860.26714	0.9589172	0.00993413	3.264
gnl|UG|Ssc#S23690475	turquoise	892.51243	874.75075	0.9623013	0.0087368	55.31
gnl|UG|Ssc#S18296629	turquoise	890.33118	806.98055	0.9452713	0.01524258	2.402
gnl|UG|Ssc#S18355973	turquoise	890.07418	838.3411	0.9564789	0.0108275	30.81
gi|19919837|gb|AF490841.1|	turquoise	889.91707	816.97949	0.9481868	0.01404722	124.256
gi|190352252|gb|EU780792.1|	turquoise	889.83384	870.37016	0.9610214	0.0091837	11.232
gi|115551364|dbj|AK236042.1|	turquoise	889.79903	787.75475	0.9392333	0.01781696	9.17
gnl|UG|Ssc#S40474141	turquoise	887.66125	806.96386	0.9454166	0.01518226	30.304
gi|115546576|dbj|AK234414.1|	turquoise	887.35531	869.51759	0.9616084	0.00897781	90.122
gnl|UG|Ssc#S23697414	turquoise	886.25377	864.64436	0.9594834	0.00973031	15.512
gi|115549179|dbj|AK231855.1|	turquoise	884.15196	776.76897	0.936352	0.01909067	50.232
gi|194036974|ref|XM_001927747.1|	turquoise	883.25088	848.074	0.9577804	0.01034751	562.674
gi|115546315|dbj|AK240553.1|	turquoise	882.73301	866.37579	0.9594767	0.00973271	31.344
gi|194033446|ref|XM_001927265.1|	turquoise	882.44687	800.86972	0.9433006	0.01606842	130.97
gnl|UG|Ssc#S23701101	turquoise	882.0186	792.5352	0.9415665	0.01680671	10.272
gi|194036442|ref|XM_001925127.1|	turquoise	880.89255	791.78053	0.9408456	0.01711682	64.414
gnl|UG|Ssc#S23765215	turquoise	880.16349	862.16704	0.9589136	0.00993542	26.618
gnl|UG|Ssc#S23695121	turquoise	879.29068	856.38031	0.9597112	0.00964871	11.316
gi|194034473|ref|XM_001924194.1|	turquoise	876.68999	784.22847	0.939892	0.01752981	14.144
gi|194034969|ref|XM_001925674.1|	turquoise	876.06879	847.80008	0.95552	0.01118566	574.562
gi|115551216|dbj|AK232649.1|	turquoise	875.967	852.12516	0.9560567	0.01098475	97.338
gnl|UG|Ssc#S40385100	turquoise	875.09201	855.66964	0.9572631	0.01053743	16.448
gi|194041007|ref|XM_001926441.1|	turquoise	874.96253	821.26854	0.951679	0.01265799	73.866
gi|115547099|dbj|AK231154.1|	turquoise	874.65179	852.83698	0.9573262	0.0105142	20.31
gnl|UG|Ssc#S39794773	turquoise	873.97307	855.17951	0.9564682	0.01083148	168.094
gi|115549486|dbj|AK235508.1|	turquoise	873.65901	760.30529	0.9304201	0.0218009	69.356
gnl|UG|Ssc#S18386751	turquoise	872.63372	832.28419	0.9517313	0.01263754	19.61
gi|115554769|dbj|AK233581.1|	turquoise	872.29707	813.65077	0.9513554	0.01278472	13.222
gnl|UG|Ssc#S18284924	turquoise	872.00599	833.37696	0.9524036	0.01237569	4.504
gnl|UG|Ssc#S23700455	turquoise	871.93447	796.58949	0.9413317	0.01690751	42.974
gi|194035561|ref|XM_001924535.1|	turquoise	871.83861	801.55685	0.9437213	0.01589094	27.314

gnl|UG|Ssc#S17510311	turquoise	870.58223	839.02938	0.9532101	0.01206398	20.602
gnl|UG|Ssc#S23690435	turquoise	870.32527	757.63511	0.9301485	0.02192774	4.016
gnl|UG|Ssc#S23763725	turquoise	869.91133	793.01278	0.9419276	0.0166521	9.46
gi|194040991|ref|XM_001924397.1|	turquoise	869.52052	835.5736	0.9534009	0.01199062	12.84
gnl|UG|Ssc#S16515033	turquoise	869.18182	845.85872	0.9542493	0.01166615	22.68
gi|194036348|ref|XM_001928686.1|	turquoise	868.79637	753.88533	0.9285032	0.02270127	44.686
gi|115554617|dbj|AK237028.1|	turquoise	868.27058	852.69424	0.9554707	0.01120417	3.338
gi|194035588|ref|XM_001924955.1|	turquoise	867.87536	810.62745	0.9465137	0.01472931	40.308
gi|194037004|ref|XM_001928953.1|	turquoise	867.5444	807.55041	0.9440419	0.0157561	24.838
gnl|UG|Ssc#S17511884	turquoise	866.71322	849.69982	0.9549882	0.01138596	8.982
gi|115551088|dbj|AK238949.1|	turquoise	866.70057	832.19394	0.9542427	0.01166864	17.134
gnl|UG|Ssc#S40558823	turquoise	866.2058	785.53614	0.9396337	0.01764222	3.164
gnl|UG|Ssc#S18551170	turquoise	865.97097	802.22381	0.9455562	0.01512438	4.474
gi|47523669|ref|NM_214306.1|	turquoise	865.92911	832.12361	0.9512331	0.01283275	49.454
gnl|UG|Ssc#S26735434	turquoise	865.78354	822.37743	0.9489125	0.01375467	16.356
gnl|UG|Ssc#S40591094	turquoise	865.7161	770.96313	0.9361662	0.01917375	33.258
gnl|UG|Ssc#S18387078	turquoise	865.21695	764.26852	0.9319309	0.02109961	75.852
gi|115554855|dbj|AK233667.1|	turquoise	864.94644	831.93266	0.951076	0.01289446	18.58
gi|194034933|ref|XM_001927442.1|	turquoise	864.15264	814.39976	0.9467927	0.01461484	73.574
gnl|UG|Ssc#S18558287	turquoise	863.25687	777.35899	0.9374541	0.01860008	4.724
gi|194035333|ref|XM_001925774.1|	turquoise	863.04745	847.65676	0.9539467	0.01178152	31.622
gnl|UG|Ssc#S31102746	turquoise	862.25043	746.53306	0.9259015	0.02394205	19.78
gnl|UG|Ssc#S18359550	turquoise	862.24507	755.868	0.9301224	0.02193997	47.804
gnl|UG|Ssc#S23701211	turquoise	862.23544	756.61759	0.9291571	0.02239282	22.4
gnl|UG|Ssc#S24610008	turquoise	862.18438	805.65296	0.9457863	0.01502914	24.58
gnl|UG|Ssc#S23772206	turquoise	861.20266	800.45014	0.9468004	0.01461166	28.896
gi|115552084|dbj|AK239277.1|	turquoise	860.95528	797.1368	0.9434161	0.01601963	10.954
gnl|UG|Ssc#S40553929	turquoise	859.58796	819.91644	0.9488115	0.01379526	50.728
gnl|UG|Ssc#S23693351	turquoise	858.54615	831.10262	0.9509489	0.01294451	13.342
gnl|UG|Ssc#S39980022	turquoise	858.53975	841.67284	0.9539015	0.01179881	14.726
gnl|UG|Ssc#S23755367	turquoise	858.33813	759.98986	0.9308804	0.02158642	25.18
gi|194034847|ref|XM_001925969.1|	turquoise	858.27056	841.91093	0.952762	0.01223686	19.946
gnl|UG|Ssc#S17518104	turquoise	857.91133	795.29688	0.9417122	0.01674429	52.556
gi|115555097|dbj|AK240296.1|	turquoise	857.8205	822.38411	0.9501943	0.01324283	79.196
gnl|UG|Ssc#S40050895	turquoise	855.96656	743.5496	0.9258964	0.0239445	6.504
gi|115548447|dbj|AK235064.1|	turquoise	855.64796	840.29832	0.952112	0.01248904	5.974
gnl|UG|Ssc#S6002926	turquoise	855.13622	831.75767	0.9508283	0.01299205	3.896
gnl|UG|Ssc#S17525104	turquoise	853.74085	797.8123	0.9420499	0.01659982	28.972
gi|194041520|ref|XM_001927592.1|	turquoise	853.08532	735.99941	0.9224282	0.0256313	114.108
gnl|UG|Ssc#S38482352	turquoise	851.97603	739.08262	0.9235113	0.02510055	18.796
gi|115552903|dbj|AK239497.1|	turquoise	851.32213	770.26476	0.9337878	0.02024789	177.418
gnl|UG|Ssc#S40475244	turquoise	849.82801	833.9895	0.9505579	0.01309881	4.904
gnl|UG|Ssc#S34500573	turquoise	849.77772	771.61852	0.9345047	0.01992215	55.272
gnl|UG|Ssc#S31122328	turquoise	849.6517	802.32956	0.946778	0.01462087	16.632
gnl|UG|Ssc#S19546121	turquoise	847.42474	827.08839	0.9486012	0.01387991	7.94
gnl|UG|Ssc#S18336569	turquoise	846.82243	816.54556	0.9465876	0.01469896	20.666
gnl|UG|Ssc#S22290406	turquoise	845.44124	779.13592	0.9353893	0.01952253	22.156
gi|194039500|ref|XM_001925226.1|	turquoise	844.79165	817.79998	0.9516137	0.01268355	16.532

gnl|UG|Ssc#S18378962	turquoise	844.66105	744.06967	0.9255128	0.02412924	9.788
gi|194044732|ref|XM_001927204.1|	turquoise	844.63247	823.52963	0.9474238	0.01435697	27.36
gi|194040741|ref|XM_001926132.1|	turquoise	844.20109	803.419	0.9489867	0.01372487	23.604
gi|115545894|dbj|AK234327.1|	turquoise	844.14089	798.1162	0.9411075	0.01700396	53.08
gi|194043780|ref|XM_001928673.1|	turquoise	843.50195	744.3607	0.9255926	0.02409076	11.984
gnl|UG|Ssc#S23696826	turquoise	843.2606	787.20423	0.9391372	0.01785899	35.792
gnl|UG|Ssc#S29983540	turquoise	841.89668	811.09974	0.9503846	0.01316741	10.94
gnl|UG|Ssc#S39889520	turquoise	841.8079	820.11158	0.9470582	0.01450619	91.26
gnl|UG|Ssc#S18385097	turquoise	841.55701	775.05509	0.9389622	0.01793558	9.54
gnl|UG|Ssc#S18547346	turquoise	841.44649	776.58211	0.9356732	0.01939486	19.434
gi|115548023|dbj|AK238007.1|	turquoise	841.13655	805.35102	0.9474823	0.01433314	8.876
gnl|UG|Ssc#S40204678	turquoise	840.42553	735.56066	0.9240897	0.02481856	67.774
gi|115545812|dbj|AK234245.1|	turquoise	840.12382	771.57623	0.9335927	0.02033686	35.162
gi|115552187|dbj|AK230496.1|	turquoise	839.29428	808.57975	0.9447682	0.01545206	60.462
gi|84490428|ref|NM_001038636.1|	turquoise	837.53769	751.72177	0.927897	0.02298848	16.008
gnl|UG|Ssc#S17516828	turquoise	837.23231	820.23698	0.9464062	0.01477351	73.706
gnl|UG|Ssc#S6052686	turquoise	835.07848	799.48095	0.9415849	0.01679884	7.428
gi|47522869|ref|NM_214024.1|	turquoise	834.92624	791.07571	0.939511	0.01769573	100.862
gi|115546083|dbj|AK237525.1|	turquoise	834.40257	765.45533	0.931019	0.021522	27.012
gnl|UG|Ssc#S22282278	turquoise	833.59027	808.89287	0.9436863	0.01590568	5.04
gi|194043591|ref|XM_001927268.1|	turquoise	832.02847	809.54631	0.9434752	0.0159947	54.02
gi|194041303|ref|XM_001927800.1|	turquoise	831.39638	814.56539	0.944451	0.0155846	45.904
gnl|UG|Ssc#S40550337	turquoise	830.99031	717.02498	0.9159879	0.02886	80.422
gnl|UG|Ssc#S19545977	turquoise	829.40371	774.97186	0.9352368	0.01959124	116.874
gnl|UG|Ssc#S17503771	turquoise	829.19713	707.38167	0.9126179	0.03059792	28.228
gi|115547932|dbj|AK234909.1|	turquoise	828.64692	804.12934	0.9419538	0.01664087	51.716
gi|194035041|ref|XM_001927251.1|	turquoise	824.77447	784.59737	0.9372017	0.01871207	57.34
gi|148223598|ref|NM_001097476.1|	turquoise	823.19693	701.01853	0.9104839	0.03171511	49.102
gi|115551432|dbj|AK236110.1|	turquoise	822.9348	719.54442	0.9199834	0.02684243	45.538
gi|148224637|ref|NM_001097469.1|	turquoise	822.85324	740.83482	0.9233789	0.02516524	36.376
gi|115548564|dbj|AK231548.1|	turquoise	820.495	720.03238	0.9166274	0.02853393	102.65
gnl|UG|Ssc#S34518323	turquoise	820.40585	782.3448	0.9367508	0.01891264	9.744
gnl|UG|Ssc#S17515754	turquoise	820.24236	692.322	0.9078103	0.03313287	24.796
gnl|UG|Ssc#S26731491	turquoise	820.04597	751.11137	0.9297856	0.02209762	45.058
gi|1955|emb|X17058.1|	turquoise	819.00233	699.08858	0.9099229	0.03201096	13.55
gnl|UG|Ssc#S23775889	turquoise	817.91323	798.39209	0.9400144	0.01747664	8.23
gnl|UG|Ssc#S6073003	turquoise	816.73971	800.34172	0.9416579	0.01676755	21.45
gi|194033615|ref|XM_001924714.1|	turquoise	816.59081	783.6697	0.9364892	0.01902936	19.612
gi|194035434|ref|XM_001926912.1|	turquoise	815.8579	801.33874	0.941385	0.01688463	9.708
gnl|UG|Ssc#S26649927	turquoise	815.23394	740.44695	0.9266158	0.02359927	30.178
gi|115550133|dbj|AK235612.1|	turquoise	815.04596	698.13512	0.9098374	0.03205611	411.43
gnl|UG|Ssc#S39990452	turquoise	812.98025	798.26635	0.9397703	0.01758274	5.39
gnl|UG|Ssc#S19542004	turquoise	812.95207	751.03226	0.927483	0.02318528	65.706
gnl|UG|Ssc#S26649787	turquoise	812.90557	779.55147	0.9358962	0.01929475	18.036
gi|194037952|ref|XM_001924515.1|	turquoise	812.1277	700.31614	0.9108956	0.0314986	92.666
gnl|UG|Ssc#S40092912	turquoise	809.40947	794.48765	0.9384223	0.01817257	2.278
gi|115551074|dbj|AK238935.1|	turquoise	807.51751	793.29208	0.9376452	0.01851545	11.086
gnl|UG|Ssc#S23695912	turquoise	806.96807	769.25914	0.9345915	0.01988282	3.104

gnl|UG|Ssc#S6077334	turquoise	806.31979	789.01946	0.9381021	0.01831359	14.886
gnl|UG|Ssc#S17517969	turquoise	805.65682	791.13519	0.9368509	0.01886806	2.5
gnl|UG|Ssc#S27600696	turquoise	804.49316	774.42745	0.9389884	0.0179241	2.198
gi|194038031|ref|XM_001928655.1|	turquoise	804.06656	746.39659	0.92642	0.02369306	14.36
gnl|UG|Ssc#S19550604	turquoise	803.89437	721.9363	0.9202851	0.02669199	6.13
gi|115545914|dbj|AK234347.1|	turquoise	803.43961	783.00283	0.9361852	0.01916524	8.996
gi|115550975|dbj|AK238836.1|	turquoise	802.81812	677.96578	0.9038355	0.03527689	144.41
gi|194038464|ref|XM_001924308.1|	turquoise	801.78815	770.77941	0.937964	0.01837453	539.154
gnl|UG|Ssc#S40160168	turquoise	801.72181	708.74395	0.9148743	0.0294307	18.112
gnl|UG|Ssc#S19545642	turquoise	801.51218	778.12874	0.9344586	0.01994301	75.658
gi|115552448|dbj|AK236453.1|	turquoise	800.89221	707.29464	0.9132581	0.03026528	20.676
gnl|UG|Ssc#S40095498	turquoise	800.64598	786.41355	0.9370497	0.01877961	5.326
gnl|UG|Ssc#S6082353	turquoise	798.34009	765.38885	0.9303584	0.02182967	5.692
gi|115551213|dbj|AK232646.1|	turquoise	798.23212	784.6417	0.9349577	0.01971717	8.564
gnl|UG|Ssc#S31114839	turquoise	798.22047	665.4885	0.898266	0.03835235	2.102
gnl|UG|Ssc#S40481010	turquoise	797.93129	740.56311	0.9238335	0.02494336	21.54
gi|194034832|ref|XM_001928118.1|	turquoise	795.45046	685.27748	0.9081329	0.03296076	35.614
gnl|UG|Ssc#S18549014	turquoise	795.15046	778.15481	0.9347568	0.01980799	3.282
gnl|UG|Ssc#S18355531	turquoise	795.08994	777.45479	0.9366053	0.01897755	12.064
gi|115545596|dbj|AK230711.1|	turquoise	794.92729	780.9165	0.9338823	0.02020485	8.812
gi|194039719|ref|XM_001927905.1|	turquoise	793.22501	769.8138	0.9310866	0.02149058	5.314
gi|115550785|dbj|AK235860.1|	turquoise	792.85632	753.88561	0.927944	0.02296618	67.562
gnl|UG|Ssc#S40162503	turquoise	790.60264	673.15403	0.9010169	0.03682308	112.388
gnl|UG|Ssc#S23769376	turquoise	790.57399	770.34123	0.9359079	0.01928948	10.14
gi|115553471|dbj|AK236674.1|	turquoise	789.78564	718.32292	0.91678	0.0284563	39.314
gnl|UG|Ssc#S18554282	turquoise	789.51755	765.09298	0.9299878	0.02200293	16.608
gnl|UG|Ssc#S26713657	turquoise	789.19885	754.11455	0.9313992	0.02134556	42.462
gi|178056656|ref|NM_001123157.1|	turquoise	789.10315	775.1741	0.9328693	0.02066774	79.262
gnl|UG|Ssc#S26651687	turquoise	788.55126	673.79984	0.9030011	0.03573241	7.512
gnl|UG|Ssc#S18378635	turquoise	787.71395	659.63515	0.896853	0.03914558	29.738
gi|115548316|dbj|AK234973.1|	turquoise	785.97349	697.67368	0.9093935	0.03229096	42.862
gi|178056875|ref|NM_001123200.1|	turquoise	785.73373	717.86707	0.9156895	0.02901257	27.538
gi|194041932|ref|XM_001928812.1|	turquoise	785.32038	769.38886	0.9305466	0.02174187	61.484
gnl|UG|Ssc#S19542674	turquoise	785.28612	738.74207	0.9263456	0.02372875	15.746
gnl|UG|Ssc#S31123896	turquoise	784.69423	700.3441	0.9122288	0.03080066	12.178
gnl|UG|Ssc#S23766410	turquoise	784.02332	655.85379	0.8945578	0.04044499	3.19
gi|157427721|ref|NM_001105299.1|	turquoise	783.39101	747.97358	0.9248486	0.02445018	59.42
gi|194040290|ref|XM_001927693.1|	turquoise	783.27554	768.44027	0.9309113	0.02157208	38.11
gnl|UG|Ssc#S22276551	turquoise	778.98963	749.35559	0.9247963	0.02447551	21.358
gnl|UG|Ssc#S23776294	turquoise	778.48701	758.10484	0.9269565	0.02343636	7.624
gnl|UG|Ssc#S31121517	turquoise	776.28972	744.2482	0.9264616	0.02367314	10.906
gnl|UG|Ssc#S18555438	turquoise	775.03512	730.4954	0.9239901	0.02486705	3.686
gnl|UG|Ssc#S18548531	turquoise	770.90282	756.39656	0.9263946	0.02370523	22.396
gi|115547381|dbj|AK237762.1|	turquoise	770.90173	729.7477	0.9260926	0.02385017	48.612
gnl|UG|Ssc#S27600018	turquoise	769.70616	656.17445	0.8949338	0.04023116	88.868
gi|2035|emb|X16638.1|	turquoise	769.67319	629.21756	0.8844593	0.04631937	33.326
gi|115554373|dbj|AK233383.1|	turquoise	769.4882	741.41197	0.922335	0.02567713	20.03
gnl|UG|Ssc#S17518103	turquoise	768.9073	706.31879	0.9152808	0.02922194	27.486

gnl|UG|Ssc#S40003052	turquoise	767.72679	643.54355	0.8915675	0.042158	96.438
gi|47523913|ref|NM_214432.1|	turquoise	767.36618	722.36015	0.9180111	0.02783249	35.1
gi|194035216|ref|XM_001928520.1|	turquoise	767.27216	686.64772	0.9085158	0.03275684	34.492
gnl|UG|Ssc#S18554740	turquoise	764.00921	709.51839	0.9133061	0.03024034	27.978
gi|52351877|gb|AY610295.1|	turquoise	763.49815	629.39504	0.8847148	0.04616763	142.428
gnl|UG|Ssc#S23755540	turquoise	763.15926	672.58702	0.9032407	0.03560141	37.108
gnl|UG|Ssc#S23699748	turquoise	761.98216	743.99706	0.9222035	0.02574186	4.528
gi|80861404|ref|NM_001037145.1|	turquoise	758.4203	741.01616	0.921063	0.02630537	5.152
gi|194034170|ref|XM_001924292.1|	turquoise	758.31438	733.7807	0.9252726	0.02424517	9.586
gi|115555495|dbj|AK234108.1|	turquoise	754.10879	694.9831	0.9090018	0.03249858	43.138
gnl|UG|Ssc#S19539925	turquoise	754.0458	649.55555	0.8935215	0.0410361	45.198
gnl|UG|Ssc#S17516962	turquoise	753.88409	703.15987	0.911741	0.03105546	12.096
gnl|UG|Ssc#S18272798	turquoise	753.03095	717.11321	0.9174659	0.02810821	23.154
gi|194035984|ref|XM_001925466.1|	turquoise	752.45911	674.74834	0.9020129	0.03627429	60.794
gi|148232727|ref|NM_001097447.1|	turquoise	750.96093	671.77725	0.9027492	0.0358703	21.956
gnl|UG|Ssc#S23768511	turquoise	750.57867	652.97055	0.8957774	0.03975283	3.606
gi|167908796|ref|NM_001114672.1|	turquoise	749.88241	730.35399	0.9177846	0.02794691	48.93
gnl|UG|Ssc#S23758422	turquoise	749.69662	726.4084	0.9171355	0.02827573	61.518
gnl|UG|Ssc#S23689683	turquoise	748.9888	653.48115	0.8962149	0.03950546	6.368
gi|89886174|ref|NM_001039749.1|	turquoise	747.72057	604.58442	0.8746903	0.05223659	13.654
gi|194038695|ref|XM_001928512.1|	turquoise	747.52465	622.769	0.8828523	0.04727722	24.29
gnl|UG|Ssc#S18378422	turquoise	746.21401	730.37625	0.9208822	0.02639506	36.74
gnl|UG|Ssc#S26726115	turquoise	743.28273	632.00711	0.8856711	0.04560118	18.786
gnl|UG|Ssc#S26728186	turquoise	743.2493	595.49513	0.871428	0.05426193	12.958
gi|115550094|dbj|AK232329.1|	turquoise	743.09943	605.06551	0.8750617	0.05200752	37.664
gi|115554492|dbj|AK233502.1|	turquoise	742.87905	600.93617	0.8743181	0.05246644	28.232
gi|194037865|ref|XM_001928013.1|	turquoise	742.30547	598.32331	0.8727932	0.05341144	104.92
gi|194036005|ref|XM_001926377.1|	turquoise	741.8855	635.23485	0.8906883	0.04266598	103.6
gi|115546833|dbj|AK237679.1|	turquoise	741.09276	709.80692	0.9210507	0.0263115	14.43
gi|52351419|gb|AY609849.1|	turquoise	740.08819	698.5412	0.9083056	0.03286873	106.416
gi|194035475|ref|XM_001924744.1|	turquoise	739.50729	672.64343	0.905031	0.03462749	10.964
gi|194041747|ref|XM_001926631.1|	turquoise	737.89557	713.51335	0.9140032	0.02987956	60.034
gi|115550351|dbj|AK238616.1|	turquoise	737.71105	648.92135	0.8936497	0.0409628	33.42
gnl|UG|Ssc#S18550086	turquoise	737.45933	652.45136	0.8937059	0.04093071	27.558
gnl|UG|Ssc#S34518006	turquoise	737.00702	708.71426	0.9150624	0.02933406	17.064
gnl|UG|Ssc#S23701109	turquoise	736.49712	665.67719	0.90046	0.03713105	101.444
gnl|UG|Ssc#S39983835	turquoise	736.08588	706.73409	0.9108962	0.03149826	7.704
gnl|UG|Ssc#S17515869	turquoise	735.81601	718.24022	0.913859	0.02995407	4.222
gi|115547186|dbj|AK234559.1|	turquoise	734.29595	613.24411	0.8786462	0.04981343	67.114
gnl|UG|Ssc#S19542298	turquoise	733.77618	719.17684	0.9138205	0.02997401	21.21
gnl|UG|Ssc#S40177122	turquoise	733.0739	676.85626	0.9053588	0.03445012	35.708
gnl|UG|Ssc#S17515611	turquoise	730.1493	606.6368	0.8763964	0.05118704	169.282
gnl|UG|Ssc#S31111777	turquoise	725.83962	635.11689	0.8901941	0.04295233	5.902
gnl|UG|Ssc#S31124818	turquoise	725.18409	707.52492	0.9109739	0.03145747	32.214
gnl|UG|Ssc#S18552237	turquoise	724.63404	663.27953	0.9001548	0.03730022	9.658
gi|115550896|dbj|AK235971.1|	turquoise	724.26762	578.96536	0.8640621	0.058923	87.472
gnl|UG|Ssc#S40370377	turquoise	723.65012	706.69295	0.9119909	0.03092484	62.814
gnl|UG|Ssc#S22284475	turquoise	723.55439	674.22951	0.9001544	0.03730044	43.52

gi|194044923|ref|XM_001927589.1|	turquoise	723.3038	669.4676	0.9067721	0.03368871	16.482
gi|115553034|dbj|AK239620.1|	turquoise	722.47275	705.09916	0.9093231	0.03232823	55.328
gi|194040677|ref|XM_001927466.1|	turquoise	721.87563	574.12663	0.8627104	0.05979135	92.484
gi|115552430|dbj|AK236435.1|	turquoise	720.34784	676.20993	0.9038541	0.03526674	44.142
gnl|UG|Ssc#S29974861	turquoise	720.04564	699.48376	0.9156106	0.02905295	31.332
gi|194035197|ref|XM_001925318.1|	turquoise	719.83264	630.83506	0.8850263	0.04598288	146.236
gnl|UG|Ssc#S6064423	turquoise	718.40204	642.49937	0.8969204	0.03910764	23.728
gi|194039563|ref|XM_001929079.1|	turquoise	715.94897	584.96526	0.8676011	0.05666853	13.998
gnl|UG|Ssc#S40318462	green	714.84941	80.280731	0.9613514	0.00906779	193.368
gnl|UG|Ssc#S19546570	turquoise	713.58537	633.9742	0.8944452	0.04050907	7.522
gi|47523255|ref|NM_213792.1|	turquoise	712.43115	598.94979	0.8733423	0.05307053	15.028
gi|47522943|ref|NM_214064.1|	turquoise	712.32493	689.89033	0.9054402	0.03440611	6.09
gi|115552215|dbj|AK230524.1|	turquoise	711.32919	588.32527	0.8694186	0.05552148	12.24
gnl|UG|Ssc#S19542685	turquoise	711.27641	695.8538	0.9064439	0.03386503	18.344
gi|194043856|ref|XM_001928206.1|	turquoise	711.0726	591.98301	0.8708875	0.05459987	40.812
gnl|UG|Ssc#S20945025	turquoise	709.82823	565.70692	0.8588663	0.0622824	56.038
gnl|UG|Ssc#S23770448	turquoise	709.31885	695.14604	0.908323	0.03285947	8.616
gi|115554351|dbj|AK240146.1|	turquoise	707.54239	689.50097	0.9054959	0.03437605	75.204
gnl|UG|Ssc#S40356278	turquoise	705.5311	618.77871	0.8846795	0.0461886	20.192
gi|115555353|dbj|AK233966.1|	turquoise	705.36579	691.95112	0.905188	0.0345425	10.674
gi|115552494|dbj|AK236499.1|	turquoise	704.43305	586.63116	0.8692225	0.0556449	21.882
gnl|UG|Ssc#S40004648	turquoise	704.23261	644.65507	0.8923372	0.04171494	50.79
gi|115546964|dbj|AK240610.1|	turquoise	704.15035	594.2538	0.8721817	0.05379186	20.56
gnl|UG|Ssc#S23759611	green	702.1644	78.159304	0.9549155	0.01141342	6.85
gnl|UG|Ssc#S18554366	turquoise	701.07756	677.6235	0.899696	0.0375549	43.246
gi|194038277|ref|XM_001926890.1|	turquoise	699.46085	624.12994	0.889166	0.04355002	51.154
gi|57528034|ref|NM_001009582.1|	turquoise	697.43382	611.49502	0.8769007	0.05087815	403.054
gnl|UG|Ssc#S26720534	green	694.15935	83.378408	0.9696109	0.00633027	31.948
gnl|UG|Ssc#S26391884	turquoise	692.41747	643.16015	0.8922502	0.04176496	7.066
gnl|UG|Ssc#S18292832	turquoise	692.20553	636.25773	0.8936125	0.04098406	8.908
gnl|UG|Ssc#S17525951	turquoise	691.84262	635.98726	0.8858646	0.04548684	17.272
gi|115548389|dbj|AK235006.1|	turquoise	690.68027	569.00332	0.8594083	0.06192928	48.126
gnl|UG|Ssc#S19547860	turquoise	690.6012	663.01046	0.8949332	0.04023153	54.226
gi|52351234|gb|AY609666.1|	turquoise	690.1829	594.25513	0.874132	0.05258147	44.012
gnl|UG|Ssc#S17525376	turquoise	688.09346	599.7894	0.8793384	0.04939318	29.532
gnl|UG|Ssc#S40576057	turquoise	686.63562	648.43744	0.9022604	0.03613829	9.49
gnl|UG|Ssc#S31102731	turquoise	686.39445	650.89639	0.8928064	0.04144556	7.35
gnl|UG|Ssc#S19542246	turquoise	686.376	662.81776	0.8966984	0.03923268	27.462
gi|194040238|ref|XM_001929612.1|	turquoise	684.36311	648.06151	0.8900371	0.04304344	14.616
gnl|UG|Ssc#S18549962	turquoise	682.51255	599.90002	0.8725967	0.05353357	30.818
gi|115547522|dbj|AK237904.1|	green	682.44556	85.119567	0.9744709	0.00487771	85.474
gnl|UG|Ssc#S18357580	turquoise	681.85447	582.81622	0.8670909	0.05699181	33.072
gnl|UG|Ssc#S23757023	turquoise	680.75373	548.16428	0.8557909	0.06429807	10.612
gi|115555693|dbj|AK237326.1|	turquoise	680.62062	569.39265	0.8606776	0.06110469	53.188
gnl|UG|Ssc#S23756069	turquoise	677.09487	628.18472	0.8947852	0.04031565	2.108
gi|115546371|dbj|AK230890.1|	turquoise	676.81445	540.14554	0.8490733	0.06876989	54.664
gi|47522941|ref|NM_214063.1|	turquoise	676.41051	571.39244	0.863431	0.05932795	56.744
gnl|UG|Ssc#S18548934	turquoise	674.68137	520.28951	0.8404378	0.07465371	34.478

gi|115547340|dbj|AK234713.1|	green	673.14798	80.412548	0.9623609	0.00871615	108.582
gnl|UG|Ssc#S23766637	turquoise	673.02795	639.00397	0.890435	0.04281266	101.48
gi|115552311|dbj|AK230621.1|	turquoise	672.95145	528.0942	0.8424845	0.07324566	51.622
gi|115555023|dbj|AK240222.1|	turquoise	672.07112	555.40783	0.8606504	0.06112232	65.86
gi|115549412|dbj|AK235434.1|	turquoise	670.21444	637.11291	0.8854213	0.04574898	58.6
gnl|UG|Ssc#S23760465	turquoise	669.13276	656.40208	0.8956605	0.03981903	15.334
gi|115545821|dbj|AK234254.1|	turquoise	668.83543	644.9045	0.8903832	0.04284268	37.038
gnl|UG|Ssc#S6072825	green	668.74249	81.246684	0.9635627	0.00830359	69.118
gnl|UG|Ssc#S17518758	turquoise	668.45305	647.18337	0.8903038	0.04288869	6.204
gnl|UG|Ssc#S40141315	turquoise	668.24092	523.28074	0.8396906	0.07516977	13.658
gi|115553434|dbj|AK236637.1|	turquoise	665.88814	636.31636	0.8849139	0.04604955	8.544
gi|178057176|ref|NM_001123090.1|	turquoise	665.77364	583.84309	0.8744999	0.05235411	47.4
gi|194043281|ref|XM_001927737.1|	green	665.06578	84.960131	0.9741503	0.00496966	13.532
gnl|UG|Ssc#S26716503	turquoise	664.54567	594.98634	0.8745272	0.05233724	17.204
gnl|UG|Ssc#S18268722	turquoise	664.47827	617.20058	0.8793678	0.04937534	11.382
gnl|UG|Ssc#S18552112	green	661.95868	83.267267	0.9696409	0.00632091	19.566
gnl|UG|Ssc#S17509854	turquoise	661.52928	626.70715	0.8844521	0.04632363	19.818
gi|196259975|ref|NM_001131045.1|	turquoise	661.25048	595.63695	0.8744664	0.05237482	87.352
gnl|UG|Ssc#S18276682	turquoise	660.67018	615.44203	0.8870079	0.0448131	6.194
gi|115550971|dbj|AK238832.1|	turquoise	660.54832	635.17938	0.885056	0.04596531	36.522
gi|115554977|dbj|AK240176.1|	turquoise	660.25641	630.37554	0.8826221	0.04741492	13.942
gnl|UG|Ssc#S18381759	turquoise	657.79127	635.20786	0.8846065	0.04623196	5.884
gi|115547032|dbj|AK231087.1|	turquoise	657.61626	620.21311	0.8795463	0.04926716	10.296
gnl|UG|Ssc#S17511439	turquoise	657.27422	641.7877	0.8931143	0.0412691	46.84
gi|194035846|ref|XM_001928960.1|	turquoise	656.94524	644.07876	0.8866137	0.04504505	124.57
gnl|UG|Ssc#S26722436	turquoise	653.31567	634.16009	0.8937118	0.0409273	3.526
gi|194035192|ref|XM_001924962.1|	turquoise	651.77294	524.75276	0.8455633	0.07114331	25.03
gi|194038708|ref|XM_001928665.1|	green	651.36153	83.454897	0.9706559	0.00600751	179.728
gi|194038723|ref|XM_001929103.1|	turquoise	651.15998	638.81236	0.8851662	0.0459	23.168
gi|115550002|dbj|AK232237.1|	turquoise	650.20908	631.83262	0.8932259	0.04120519	555.12
gi|115550416|dbj|AK238681.1|	black	649.49444	42.887619	0.9261645	0.02381563	37.676
gnl|UG|Ssc#S18546249	turquoise	649.21173	634.78817	0.8830835	0.04713904	17.346
gnl|UG|Ssc#S5984485	turquoise	648.78668	634.66912	0.8848511	0.04608679	9.358
gnl|UG|Ssc#S19545784	turquoise	647.72479	507.37526	0.8320152	0.08053437	29.53
gi|194037372|ref|XM_001927549.1|	turquoise	647.25501	610.51361	0.8789629	0.04962099	53
gnl|UG|Ssc#S6016211	turquoise	641.32963	620.3525	0.879567	0.04925461	8.36
gnl|UG|Ssc#S23701149	turquoise	641.14578	583.99385	0.8653154	0.05812147	10.956
gnl|UG|Ssc#S39879611	turquoise	640.48654	628.27695	0.8813149	0.0481994	4.926
gnl|UG|Ssc#S18291190	turquoise	639.97247	489.89693	0.8239719	0.08627678	6.584
gnl|UG|Ssc#S39991153	green	638.80381	87.211355	0.9797183	0.00345674	3.322
gnl|UG|Ssc#S26724832	green	638.19597	73.74907	0.9420911	0.01658221	22.94
gi|194045109|ref|XM_001927874.1|	green	637.22153	87.15756	0.9795835	0.00349116	41.4
gi|115553396|dbj|AK236599.1|	turquoise	637.07859	547.08798	0.8566701	0.06371978	15.84
gnl|UG|Ssc#S18556295	green	636.41635	77.704835	0.9538256	0.01182783	69.278
gi|115547260|dbj|AK234633.1|	turquoise	636.30092	612.87166	0.8763913	0.0511902	36.706
gnl|UG|Ssc#S26718109	turquoise	635.73247	604.49216	0.8729182	0.05333378	13.344
gnl|UG|Ssc#S18387501	turquoise	635.65634	583.48336	0.8657364	0.05785299	37.77
gi|115545757|dbj|AK234190.1|	green	633.70953	82.810456	0.9693314	0.0064175	27.168

gnl|UG|Ssc#S5983468	turquoise	633.61741	619.28915	0.8831938	0.04707318	3.918
gnl|UG|Ssc#S19545449	turquoise	631.60473	480.28405	0.8222776	0.08750178	45.312
gi|47523867|ref|NM_214407.1|	green	631.51777	86.082606	0.9774699	0.00404582	124.838
gnl|UG|Ssc#S23775891	turquoise	627.97979	604.44042	0.8784662	0.04992289	81.464
gnl|UG|Ssc#S23761029	black	626.87535	43.585086	0.9291482	0.022397	2.39
gi|125630305|ref|NM_214281.2|	turquoise	622.58445	578.12506	0.8758545	0.05151966	19.628
gi|115547094|dbj|AK231149.1|	green	622.54638	80.970913	0.9631259	0.00845276	26.518
gnl|UG|Ssc#S17517759	green	622.3644	87.835627	0.9813109	0.00305842	8.682
gnl|UG|Ssc#S26710759	turquoise	619.23262	575.49943	0.8721557	0.05380803	92.524
gnl|UG|Ssc#S40490912	turquoise	619.21444	595.38246	0.8731116	0.05321366	6.122
gi|115550075|dbj|AK232310.1|	turquoise	618.48691	596.83042	0.8685423	0.05607358	4.66
gnl|UG|Ssc#S18355677	turquoise	618.129	489.36597	0.8262257	0.08465545	8.996
gi|194043070|ref|XM_001928504.1|	turquoise	617.61979	552.40448	0.8582149	0.06270766	4.674
gi|178056505|ref|NM_001123131.1|	turquoise	617.5338	464.86703	0.8162835	0.09187779	14.126
gi|115550755|dbj|AK235829.1|	turquoise	617.23917	560.68496	0.8608854	0.06097	101.35
gnl|UG|Ssc#S18383312	turquoise	617.09736	593.50311	0.8691588	0.05568496	9.51
gnl|UG|Ssc#S18555055	turquoise	616.25864	479.78533	0.8201508	0.08904698	40.998
gnl|UG|Ssc#S23762560	turquoise	616.2279	593.81097	0.8709179	0.05458079	31.488
gnl|UG|Ssc#S40389825	turquoise	616.12417	600.07705	0.8751977	0.05192371	11.888
gnl|UG|Ssc#S40006504	turquoise	615.36484	511.87838	0.8387032	0.07585346	5.49
gi|115548394|dbj|AK235011.1|	black	615.27187	44.846364	0.937808	0.01844343	98.28
gnl|UG|Ssc#S16769298	turquoise	614.81936	507.74711	0.8401736	0.07483604	5.26
gi|115548467|dbj|AK235084.1|	turquoise	613.67325	503.22555	0.8301772	0.08183579	236.442
gnl|UG|Ssc#S40481152	turquoise	613.21929	580.11363	0.8689797	0.05579777	3.112
gnl|UG|Ssc#S6085710	turquoise	611.01465	596.46156	0.871048	0.05449946	22.068
gnl|UG|Ssc#S40258460	green	608.96782	85.850075	0.9769846	0.00417694	30.75
gi|194041397|ref|XM_001928870.1|	green	608.44323	84.424006	0.9733229	0.00520951	15.932
gnl|UG|Ssc#S19545902	green	607.62979	88.983095	0.9844067	0.00233198	56.014
gnl|UG|Ssc#S18281053	turquoise	604.68698	501.4715	0.8315923	0.08083321	80.48
gi|47522935|ref|NM_214060.1|	turquoise	599.79916	586.01465	0.8653187	0.05811936	62.776
gnl|UG|Ssc#S40500672	turquoise	599.53568	508.78054	0.833729	0.07932668	206.356
gnl|UG|Ssc#S41577006	black	597.90045	41.407561	0.9177119	0.0279837	8.834
gi|115549787|dbj|AK232067.1|	turquoise	596.37206	503.78246	0.8310516	0.08121586	49.868
gi|115553590|dbj|AK239777.1|	turquoise	596.24738	447.91917	0.8039438	0.10108848	33.034
gi|52351587|gb|AY610017.1|	turquoise	594.90799	501.34667	0.8361819	0.07760798	127.482
gi|47522779|ref|NM_213977.1|	turquoise	594.36745	516.22861	0.8358008	0.0778742	49.154
gnl|UG|Ssc#S17510601	turquoise	593.86703	497.47167	0.830038	0.08193462	17.906
gnl|UG|Ssc#S40054290	green	593.01747	87.357317	0.980841	0.00317424	33.462
gi|52350726|gb|AY609441.1|	turquoise	592.64146	537.01611	0.8484289	0.06920371	17.36
gnl|UG|Ssc#S40327466	turquoise	592.05065	537.39153	0.8468454	0.07027345	1.256
gi|115549388|dbj|AK235410.1|	turquoise	591.3242	573.90414	0.8648802	0.05839939	22.202
gi|167908792|ref|NM_001114670.1|	turquoise	591.30582	544.03704	0.8609427	0.06093287	74.032
gnl|UG|Ssc#S27599980	turquoise	590.40105	489.14492	0.8251214	0.08544865	91.36
gi|52351086|gb|AY609491.1|	turquoise	590.28203	573.94132	0.8607333	0.0610686	2.764
gnl|UG|Ssc#S23694886	green	589.63036	87.029214	0.979585	0.00349079	19.67
gi|49274614|ref|NM_001001860.1|	turquoise	588.33823	477.59185	0.8167519	0.09153346	575.974
gnl|UG|Ssc#S17526874	turquoise	588.20062	554.64255	0.851724	0.06699417	74.572
gi|194043267|ref|XM_001926534.1|	turquoise	588.18152	563.07746	0.854599	0.06508466	6.948

gnl|UG|Ssc#S40044675	turquoise	586.76588	496.78133	0.833476	0.07950461	10.572
gi|194033456|ref|XM_001924731.1|	turquoise	581.86771	421.9879	0.7921776	0.11011537	10.83
gnl|UG|Ssc#S40554875	turquoise	581.0729	561.59896	0.853933	0.06552547	25.058
gi|194036619|ref|XM_001927496.1|	green	580.4676	86.759723	0.9796519	0.00347368	27.176
gi|194038714|ref|XM_001926092.1|	turquoise	580.44021	558.90866	0.8551065	0.06474938	47.672
gnl|UG|Ssc#S29977062	turquoise	580.24134	440.69862	0.7995166	0.10445754	2.496
gnl|UG|Ssc#S39959676	turquoise	579.92465	441.1847	0.7991257	0.10475659	4.112
gi|52351696|gb|AY610126.1|	turquoise	579.77249	435.50821	0.7963961	0.10685218	21.452
gnl|UG|Ssc#S39994657	turquoise	579.2925	519.61598	0.8387023	0.07585415	10.744
gnl|UG|Ssc#S19545587	turquoise	578.65465	429.73409	0.7954313	0.10759591	4.378
gnl|UG|Ssc#S31122252	turquoise	578.0919	494.91069	0.8287015	0.08288537	31.436
gi|115550346|dbj|AK238611.1|	turquoise	576.75481	560.26373	0.8546241	0.06506807	19.624
gi|47522831|ref|NM_214003.1|	turquoise	575.91926	493.14981	0.8255057	0.08517239	117.826
gi|115551181|dbj|AK232614.1|	turquoise	575.86286	529.66798	0.8426892	0.07310532	66.062
gnl|UG|Ssc#S22272079	turquoise	575.36809	551.06243	0.8492514	0.06865011	4.69
gi|115552725|dbj|AK232930.1|	turquoise	574.22283	452.19311	0.8144765	0.09320978	30.826
gi|194043074|ref|XM_001928487.1|	turquoise	573.21934	452.30346	0.8042953	0.1008224	133.566
gnl|UG|Ssc#S18551494	turquoise	573.08093	510.21721	0.8324189	0.08024938	89.386
gi|115554063|dbj|AK233272.1|	green	572.90157	91.79593	0.9915597	0.00092966	31.314
gnl|UG|Ssc#S40043958	black	572.76654	47.806818	0.9538391	0.01182267	57.086
gi|194033834|ref|XM_001924991.1|	turquoise	570.07589	423.66821	0.7895125	0.11219213	39.032
gnl|UG|Ssc#S22276105	turquoise	569.8054	479.60158	0.8267719	0.08426392	5.23
gi|52351905|gb|AY610323.1|	turquoise	569.74861	536.76289	0.844321	0.0719893	28.63
gnl|UG|Ssc#S19541728	turquoise	569.21168	542.3373	0.8547218	0.06500348	27.176
gi|61097882|ref|NM_001012956.1|	black	568.40049	46.356397	0.9485413	0.01390406	31.642
gnl|UG|Ssc#S18553702	turquoise	568.3508	542.24248	0.8512158	0.06733353	18.85
gi|115548919|dbj|AK235322.1|	turquoise	566.93938	516.78782	0.8429721	0.07291143	54.528
gnl|UG|Ssc#S29980298	green	566.01718	89.375206	0.9866588	0.00184611	3.398
gnl|UG|Ssc#S38481793	turquoise	565.39936	548.41283	0.8625579	0.05988958	2.23
gnl|UG|Ssc#S18359557	turquoise	564.74362	526.16631	0.8395015	0.07530057	22.764
gnl|UG|Ssc#S34504168	turquoise	564.04072	488.16201	0.8266009	0.08438648	55.642
gi|109639161|ref|NM_001006593.2|	turquoise	563.66003	549.01056	0.8561628	0.06405329	14.12
gi|115546623|dbj|AK234461.1|	turquoise	563.64337	532.3892	0.8423202	0.07335836	262.534
gnl|UG|Ssc#S31118680	green	563.41019	88.599865	0.9847674	0.00225164	2.788
gnl|UG|Ssc#S18557214	turquoise	562.14657	428.58552	0.7948754	0.10802508	27.444
gnl|UG|Ssc#S26733997	green	561.32592	89.685537	0.9867447	0.00182831	13.834
gi|194036434|ref|XM_001926977.1|	green	560.82776	86.894146	0.9788359	0.00368426	174.676
gi|115545530|dbj|AK230645.1|	turquoise	559.36489	538.07727	0.8446218	0.07178421	12.822
gnl|UG|Ssc#S40089767	turquoise	558.62421	498.67308	0.8264898	0.0844661	49.184
gi|115553171|dbj|AK232969.1|	turquoise	558.23048	538.33492	0.8475143	0.06982095	83.892
gnl|UG|Ssc#S18358603	green	557.8373	91.57512	0.991041	0.00101658	20.002
gnl|UG|Ssc#S26721796	black	557.51208	48.880539	0.9621972	0.00877289	6.08
gi|115546075|dbj|AK237517.1|	turquoise	557.4162	506.74001	0.8334077	0.07955264	26.84
gi|47523607|ref|NM_214267.1|	turquoise	557.15932	459.45293	0.8098774	0.09662605	19.358
gnl|UG|Ssc#S18553548	turquoise	556.3649	427.35385	0.8010447	0.10329091	43.902
gnl|UG|Ssc#S18388327	turquoise	556.27187	407.56547	0.7812596	0.1186965	13.258
gnl|UG|Ssc#S40069929	turquoise	555.82238	449.14625	0.8094908	0.09691488	6.616
gnl|UG|Ssc#S26732732	black	554.51749	48.454541	0.9580254	0.01025794	15.878

gi|52351368|gb|AY609798.1|	turquoise	554.07758	530.57288	0.8561124	0.06408643	21.392
gnl|UG|Ssc#S31111249	turquoise	551.93763	446.81783	0.8078522	0.0981422	5.724
gi|52351609|gb|AY610039.1|	turquoise	551.3062	491.17267	0.831746	0.08072459	11.104
gnl|UG|Ssc#S17509834	green	550.06767	85.824462	0.9767492	0.00424103	0.846
gi|194038854|ref|XM_001928014.1|	turquoise	549.65248	535.59351	0.8447079	0.07172555	31.042
gi|194040173|ref|XM_001926225.1|	turquoise	549.37172	515.48985	0.8346678	0.07866751	69.34
gi|166796042|ref|NM_001114275.1|	turquoise	549.08295	502.38573	0.8424146	0.07329363	33.164
gi|115551894|dbj|AK236301.1|	turquoise	548.86751	502.41165	0.833739	0.07931962	9.904
gi|194041780|ref|XM_001925192.1|	green	548.85508	82.993438	0.9688967	0.006554	129.576
gnl|UG|Ssc#S18360061	turquoise	547.50033	532.49043	0.848513	0.06914705	4.258
gnl|UG|Ssc#S40145713	turquoise	547.12472	521.45975	0.8360037	0.07773244	10.674
gnl|UG|Ssc#S18548660	turquoise	545.81597	392.58711	0.7724181	0.12578506	133.716
gi|115555451|dbj|AK234064.1|	green	545.75538	85.915208	0.9782354	0.00384181	40.964
gi|194041230|ref|XM_001925354.1|	turquoise	544.2758	516.02791	0.84895	0.06885281	41.228
gnl|UG|Ssc#S35171934	black	543.56285	46.701382	0.9519327	0.01255893	20.804
gi|115553328|dbj|AK233126.1|	turquoise	543.41527	473.11289	0.8190473	0.08985196	35.24
gnl|UG|Ssc#S19538787	turquoise	540.38853	512.71525	0.832676	0.08006805	22.648
gnl|UG|Ssc#S18557699	turquoise	540.02691	515.76828	0.8366262	0.07729791	43.262
gnl|UG|Ssc#S40483013	turquoise	539.17141	466.34027	0.8117376	0.09523972	17.44
gnl|UG|Ssc#S16516579	turquoise	538.88637	476.94159	0.8334548	0.0795195	5.944
gi|194035852|ref|XM_001929031.1|	turquoise	538.53198	508.19223	0.8310547	0.0812137	11.414
gnl|UG|Ssc#S40154742	turquoise	538.2426	473.34716	0.8243953	0.08597147	2.526
gi|167908790|ref|NM_001114676.1|	green	538.1076	88.994701	0.9844182	0.0023294	76.084
gnl|UG|Ssc#S17510145	turquoise	537.90569	508.75119	0.8317847	0.08069724	9.566
gi|52351323|gb|AY609755.1|	green	536.11619	76.885792	0.951863	0.01258611	163.28
gnl|UG|Ssc#S26733269	turquoise	535.54353	496.75255	0.8404449	0.07464881	115.204
gnl|UG|Ssc#S40050849	green	534.56896	91.512422	0.9914496	0.00094788	25.012
gi|115547231|dbj|AK234604.1|	turquoise	534.17003	521.34538	0.8370123	0.07702878	35.262
gi|115553571|dbj|AK239758.1|	turquoise	532.37555	395.77416	0.7740073	0.12450195	67.028
gnl|UG|Ssc#S18386841	green	531.70981	81.944678	0.966501	0.00732298	14.11
gnl|UG|Ssc#S17511034	green	530.41793	89.260548	0.9868052	0.00181583	15.34
gi|149944498|ref|NM_214343.2|	green	530.22133	91.708535	0.9926616	0.0007538	115.828
gnl|UG|Ssc#S6029367	turquoise	529.18654	512.24273	0.8483011	0.0692899	10.64
gi|115555163|dbj|AK233778.1|	turquoise	528.00402	510.6163	0.8367831	0.07718845	46.172
gnl|UG|Ssc#S17519017	turquoise	527.40498	389.70078	0.7738595	0.12462114	181.912
gi|115550537|dbj|AK232367.1|	green	527.15795	93.866283	0.9976165	0.00013964	9.264
gnl|UG|Ssc#S18283093	turquoise	526.98058	513.06429	0.8376358	0.07659473	20.816
gi|194044289|ref|XM_001926487.1|	green	526.06546	91.882716	0.9918115	0.0008884	99.66
gnl|UG|Ssc#S20945959	turquoise	525.32674	441.92618	0.8147539	0.09300492	8.294
gi|194036956|ref|XM_001924342.1|	green	524.80343	89.17811	0.9864837	0.00188251	50.44
gi|73853893|ref|NM_001032358.1|	turquoise	523.45512	381.71111	0.7678075	0.12952987	27.05
gnl|UG|Ssc#S23758068	turquoise	523.35004	510.51373	0.8442925	0.0720088	20.892
gnl|UG|Ssc#S6062152	turquoise	523.02078	368.4781	0.7633156	0.13320934	2.298
gnl|UG|Ssc#S17526834	turquoise	522.21934	491.57999	0.823978	0.08627232	92.492
gi|115553862|dbj|AK236867.1|	green	522.1495	83.831035	0.971273	0.00581955	15.636
gi|115548933|dbj|AK235336.1|	turquoise	519.74464	450.59882	0.8046377	0.10056343	28.988
gi|115551027|dbj|AK238888.1|	turquoise	518.90884	503.89918	0.8339547	0.07916805	38.736
gi|194044169|ref|XM_001924460.1|	turquoise	517.98944	470.92921	0.8327745	0.07999857	76.66

gnl|UG|Ssc#S39980075	green	517.91608	89.042816	0.9856041	0.00206896	4.976
gnl|UG|Ssc#S23758940	green	517.77953	80.50633	0.9619003	0.00887604	9.488
gi|115550882|dbj|AK235957.1|	black	515.0102	51.474698	0.9729082	0.0053311	11.122
gnl|UG|Ssc#S40173770	turquoise	512.51563	403.1935	0.7839143	0.11659227	112.91
gnl|UG|Ssc#S23695171	turquoise	511.57881	353.28523	0.7539913	0.14094378	16.42
gi|115548093|dbj|AK238077.1|	black	511.38407	51.577682	0.9739916	0.00501538	54.798
gi|52351137|gb|AY609542.1|	green	511.17454	91.061622	0.9912645	0.0009788	13.724
gi|52351621|gb|AY610051.1|	turquoise	510.33683	441.75892	0.7995081	0.10446403	66.064
gnl|UG|Ssc#S18378241	turquoise	509.89969	383.47157	0.7701555	0.12761867	14.57
gnl|UG|Ssc#S34527038	turquoise	509.12647	357.91418	0.7500809	0.14422551	11.942
gnl|UG|Ssc#S23759053	green	508.71527	84.197105	0.9733589	0.00519898	21.458
gi|115549900|dbj|AK232134.1|	turquoise	508.43872	485.22618	0.82326	0.08679085	39.078
gi|47523569|ref|NM_214249.1|	black	508.27517	46.886298	0.9530691	0.01211829	73.918
gnl|UG|Ssc#S17515701	turquoise	506.90731	472.99784	0.8333572	0.07958817	24.598
gnl|UG|Ssc#S16763868	turquoise	506.83036	479.16536	0.8158353	0.09220765	20.018
gnl|UG|Ssc#S18269927	black	505.88194	51.941865	0.975472	0.00459434	10.57
gnl|UG|Ssc#S23766692	green	505.44821	79.035342	0.9577652	0.01035306	25.488
gnl|UG|Ssc#S19547688	turquoise	503.19877	489.00098	0.8207504	0.08861051	26.968
gi|115553726|dbj|AK239913.1|	turquoise	502.24216	471.35876	0.8137261	0.0937646	33.19
gi|194041034|ref|XM_001928236.1|	turquoise	501.87074	440.88538	0.7991823	0.10471325	29.39
gnl|UG|Ssc#S26721858	turquoise	499.8983	448.60183	0.8021979	0.10241308	6.156
gnl|UG|Ssc#S19542511	turquoise	499.05341	452.41925	0.8034981	0.10142608	23.006
gi|194042436|ref|XM_001925357.1|	green	498.48758	75.858156	0.9484435	0.01394349	7.58
gi|115553351|dbj|AK233149.1|	turquoise	495.64688	478.86725	0.8174515	0.09101996	10.498
gnl|UG|Ssc#S18552425	turquoise	495.27601	450.27279	0.8048544	0.1003997	8.276
gnl|UG|Ssc#S40096829	turquoise	493.67626	452.47599	0.8036325	0.10132426	107.208
gnl|UG|Ssc#S34529604	green	493.19632	86.072277	0.9778968	0.00393161	8.728
gnl|UG|Ssc#S19548031	green	493.04283	79.528321	0.9587802	0.00998366	52.144
gi|56792886|gb|AY705919.1|	turquoise	492.8437	424.02015	0.7901376	0.11170398	16.088
gi|115546071|dbj|AK237513.1|	turquoise	492.3392	371.65148	0.7585681	0.13713129	37.77
gnl|UG|Ssc#S17510396	turquoise	489.77954	401.89919	0.7748127	0.1238531	108.826
gi|115548554|dbj|AK235171.1|	turquoise	489.73684	465.04384	0.8117768	0.0952106	26.582
gnl|UG|Ssc#S17527052	black	486.5851	50.582163	0.9667426	0.00724417	21.918
gi|194041036|ref|XM_001925819.1|	turquoise	483.37224	405.99	0.7910627	0.11098273	58.11
gnl|UG|Ssc#S34502088	turquoise	482.19024	461.11075	0.8062506	0.09934635	16.936
gi|194037272|ref|XM_001928278.1|	black	481.12032	46.204501	0.9417294	0.01673689	24.828
gi|51592138|ref|NM_001004045.1|	green	479.59553	94.205959	0.998643	6.00E-05	122.258
gi|115554312|dbj|AK240107.1|	turquoise	477.55371	461.83461	0.8105723	0.09610745	104.128
gnl|UG|Ssc#S39959416	turquoise	477.18232	399.67643	0.7821138	0.11801818	18.594
gi|52351116|gb|AY609521.1|	green	476.93582	86.560731	0.9799499	0.00339779	88.722
gnl|UG|Ssc#S15981469	green	476.8335	87.563096	0.9832572	0.00259407	8.676
gi|194038066|ref|XM_001927909.1|	turquoise	473.50524	385.0559	0.7644632	0.1322664	285.724
gnl|UG|Ssc#S17518565	turquoise	473.03189	323.10706	0.7350477	0.15704493	11.466
gnl|UG|Ssc#S26392759	black	472.65002	53.176438	0.9815813	0.00299241	13.856
gi|194043124|ref|XM_001926685.1|	turquoise	472.29564	325.82171	0.727219	0.16384528	53.926
gi|164486|gb|M29072.1|PIGHEP1	black	472.2936	49.612757	0.9608986	0.00922698	37.332
gi|194043063|ref|XM_001925272.1|	turquoise	469.95189	431.64687	0.8073727	0.09850225	27.33
gi|115545827|dbj|AK234260.1|	green	464.92695	86.225513	0.9783017	0.00382431	46.712

gi|194036611|ref|XM_001926603.1|	green	461.82911	92.179282	0.9928299	0.00072803	26.274
gnl|UG|Ssc#S38479783	green	460.63285	90.977778	0.9907762	0.00106194	58.876
gnl|UG|Ssc#S19546199	turquoise	460.50373	406.16523	0.7780741	0.12123625	2.322
gi|194035412|ref|XM_001925476.1|	turquoise	459.19308	423.66053	0.7899719	0.11183335	17.888
gnl|UG|Ssc#S18548467	turquoise	457.52342	354.39421	0.7565221	0.13883183	40.236
gnl|UG|Ssc#S40020159	turquoise	457.03926	392.64219	0.7807213	0.11912457	200.674
gnl|UG|Ssc#S18277539	turquoise	456.54984	439.37077	0.8029455	0.10184523	71.714
gi|115546529|dbj|AK234367.1|	turquoise	456.48162	419.78145	0.7948687	0.10803025	54.692
gi|115551880|dbj|AK236287.1|	turquoise	456.17193	418.08417	0.7956433	0.10743233	12.594
gnl|UG|Ssc#S26726574	turquoise	455.8253	352.0664	0.7461256	0.14756731	10.958
gi|115553231|dbj|AK233029.1|	green	454.45965	71.421656	0.9365571	0.01899905	131.296
gnl|UG|Ssc#S23758429	turquoise	453.74463	412.73934	0.7874618	0.11379805	4.632
gnl|UG|Ssc#S17516917	green	452.38466	87.541025	0.9809399	0.00314974	34.134
gnl|UG|Ssc#S31112392	turquoise	451.8478	365.9875	0.7609276	0.13517789	5.462
gnl|UG|Ssc#S24610239	turquoise	451.64451	333.17025	0.7398835	0.15288652	5.71
gi|115545643|dbj|AK230758.1|	green	451.61879	83.598924	0.9712861	0.00581558	111.578
gnl|UG|Ssc#S6054625	turquoise	448.6196	308.49137	0.7129721	0.17643146	9.24
gi|194040489|ref|XM_001927223.1|	turquoise	448.28484	415.09554	0.7830076	0.11730971	61.818
gnl|UG|Ssc#S18383319	green	447.66736	91.295207	0.990537	0.00110346	22.468
gnl|UG|Ssc#S23695295	turquoise	446.3511	418.23846	0.7873595	0.11387835	18.42
gi|115551449|dbj|AK236127.1|	black	446.08442	49.992543	0.9712872	0.00581523	58.58
gi|194042125|ref|XM_001928342.1|	green	443.10401	93.440304	0.9965664	0.0002414	248.056
gnl|UG|Ssc#S27602393	turquoise	442.14333	414.48994	0.797086	0.10632138	9.466
gnl|UG|Ssc#S26399811	turquoise	441.85637	385.01268	0.7706494	0.12721773	44.348
gnl|UG|Ssc#S17515039	turquoise	441.13549	401.66032	0.7757315	0.12311424	65.286
gnl|UG|Ssc#S19540688	turquoise	440.31493	322.37064	0.7248543	0.16591571	47.45
gnl|UG|Ssc#S29977171	green	440.19584	89.834445	0.9870499	0.00176563	9.652
gnl|UG|Ssc#S23768398	turquoise	439.72298	336.88136	0.7514878	0.14304227	10.506
gnl|UG|Ssc#S18354485	green	439.33445	87.856081	0.983626	0.00250899	38.448
gnl|UG|Ssc#S40443438	turquoise	438.67275	393.79525	0.7732734	0.12509401	82.044
gi|138753472|emb|AM503091.1|	salmon	438.19162	31.7702	0.9119067	0.03096882	39
gnl|UG|Ssc#S19542087	turquoise	437.85896	422.37581	0.78764	0.11365823	58.436
gnl|UG|Ssc#S39855926	turquoise	435.82014	297.34735	0.7075921	0.18125336	3.072
gnl|UG|Ssc#S23699860	black	435.04341	42.861088	0.9223799	0.02565505	3.872
gi|178057066|ref|NM_001123096.1|	turquoise	433.83624	390.13322	0.76833	0.1291038	35.644
gnl|UG|Ssc#S31130713	turquoise	433.50269	358.02328	0.763529	0.1330339	5.236
gnl|UG|Ssc#S27600609	black	432.73107	54.806445	0.9896481	0.00126238	3.208
gi|194038781|ref|XM_001926473.1|	green	431.8559	57.699815	0.8918982	0.04196745	123.242
gnl|UG|Ssc#S18547360	turquoise	431.80028	381.78107	0.7818214	0.11825023	35.708
gi|115552523|dbj|AK236528.1|	green	430.23586	88.783528	0.9842708	0.00236248	62.132
gi|194036317|ref|XM_001927969.1|	black	429.45655	53.097564	0.981324	0.00305521	19.514
gnl|UG|Ssc#S18357511	turquoise	428.61246	331.01493	0.7300868	0.16134451	94.798
gi|115554826|dbj|AK233638.1|	black	428.46534	54.695535	0.9904074	0.0011262	129.538
gnl|UG|Ssc#S22275082	turquoise	427.6352	323.69283	0.7365938	0.15571191	30.434
gnl|UG|Ssc#S6076898	black	427.62412	50.611698	0.9675864	0.00697112	54.588
gnl|UG|Ssc#S18549545	green	426.74184	88.742906	0.9848712	0.0022287	9.8
gi|115551607|dbj|AK239071.1|	green	426.15415	76.861548	0.9525447	0.01232099	35.644
gnl|UG|Ssc#S16769299	green	425.51603	89.611428	0.9877933	0.00161597	24.592

gi|52351069|gb|AY609474.1|	green	423.42736	90.504518	0.9890265	0.00137764	27.14
gi|194040672|ref|XM_001925213.1|	black	421.29119	53.420626	0.9845824	0.00229273	1.416
gnl|UG|Ssc#S18546487	green	421.26773	91.164801	0.9910667	0.0010122	29.372
gi|115549313|dbj|AK235234.1|	turquoise	420.5498	386.94457	0.7675082	0.12977407	112.252
gnl|UG|Ssc#S18382475	green	418.888	81.685228	0.9658119	0.0075493	21.686
gi|194038904|ref|XM_001924445.1|	green	418.1117	87.395387	0.9816713	0.00297053	159.486
gnl|UG|Ssc#S31101896	turquoise	416.6774	386.33149	0.7646737	0.13209368	62.074
gnl|UG|Ssc#S18380653	turquoise	416.64781	347.30413	0.7511764	0.14330385	10.602
gi|72535167|ref|NM_001031778.1|	darkgrey	416.56767	13.932673	0.9315346	0.02128285	20.544
gnl|UG|Ssc#S18386541	green	413.8092	89.101438	0.9875828	0.0016579	39.036
gnl|UG|Ssc#S18356685	green	413.3819	90.087723	0.9892121	0.00134287	2.298
gnl|UG|Ssc#S18359197	turquoise	412.8189	389.20818	0.7920456	0.11021795	4.754
gnl|UG|Ssc#S18270450	turquoise	412.14676	331.19436	0.7403061	0.15252472	0.9
gnl|UG|Ssc#S23758924	green	411.25381	89.993507	0.987981	0.00157889	32.182
gnl|UG|Ssc#S17506191	green	407.51509	80.23648	0.9635635	0.00830329	37.914
gi|194043947|ref|XM_001925017.1|	turquoise	405.96581	390.41793	0.7890772	0.11253249	20.802
gi|115548922|dbj|AK235325.1|	green	405.14346	58.866318	0.8964812	0.03935514	374.856
gi|115552473|dbj|AK236478.1|	black	404.82613	55.315244	0.9958688	0.00031855	88.174
gnl|UG|Ssc#S18387783	turquoise	404.69319	351.58007	0.7434968	0.14980075	42.406
gi|194043042|ref|XM_001926896.1|	turquoise	403.29287	303.1167	0.7087926	0.1801742	54.042
gi|115551807|dbj|AK236213.1|	turquoise	402.38797	296.4766	0.7085529	0.18038949	4.306
gnl|UG|Ssc#S17503526	green	399.61054	82.843766	0.9719365	0.00561965	28.852
gnl|UG|Ssc#S34508066	turquoise	399.54804	373.56484	0.7625171	0.13386664	11.008
gnl|UG|Ssc#S40157220	green	398.54533	68.411481	0.9296026	0.02218344	99.586
gi|194041366|ref|XM_001924481.1|	turquoise	396.89356	311.26856	0.7162287	0.17353092	54.382
gnl|UG|Ssc#S39891679	green	396.33834	79.14417	0.9583383	0.01014395	140.328
gi|194043006|ref|XM_001928075.1|	green	396.32863	85.107702	0.978001	0.0039039	26.19
gi|194044285|ref|XM_001926002.1|	green	392.9474	89.437235	0.9873866	0.0016973	27.228
gnl|UG|Ssc#S19542622	turquoise	392.19267	318.62498	0.7216564	0.1687275	19.428
gi|115552250|dbj|AK230559.1|	turquoise	390.80755	339.07313	0.7374634	0.15496355	134.72
gnl|UG|Ssc#S40520406	green	389.75831	77.532289	0.9529144	0.01217795	30.54
gnl|UG|Ssc#S18261767	black	389.19287	49.35509	0.9601292	0.00949951	10.594
gi|73853881|ref|NM_001032355.1|	turquoise	389.16046	300.41015	0.7104738	0.17866595	25.116
gnl|UG|Ssc#S18387558	black	387.12555	56.332848	0.9990694	3.41E-05	75.398
gi|148747452|ref|NM_001098603.1|	green	386.71334	66.201641	0.9198029	0.02693254	17.67
gi|52351777|gb|AY610207.1|	turquoise	386.67426	345.36557	0.7400654	0.15273077	19.096
gnl|UG|Ssc#S23770611	turquoise	385.178	283.39502	0.7039819	0.18450968	25.742
gnl|UG|Ssc#S23695269	turquoise	383.31171	333.38657	0.7310735	0.16048667	9.818
gi|115546465|dbj|AK230984.1|	turquoise	382.52526	340.52343	0.734814	0.15724677	148.926
gnl|UG|Ssc#S41446582	green	382.40538	87.103776	0.9805825	0.00323859	50.526
gnl|UG|Ssc#S29987869	black	381.13175	46.675864	0.9452707	0.01524283	15.098
gi|115554133|dbj|AK233343.1|	turquoise	379.23237	352.65222	0.7436091	0.14970512	67.742
gnl|UG|Ssc#S17500137	green	378.25495	87.33498	0.9826131	0.00274492	7.274
gnl|UG|Ssc#S40159132	turquoise	377.99276	348.45785	0.7456942	0.14793312	5.432
gnl|UG|Ssc#S39994120	turquoise	375.29054	343.41077	0.7394068	0.15329504	22.748
gi|115545622|dbj|AK230737.1|	turquoise	374.31108	277.81452	0.7015727	0.18669192	44.822
gi|77745211|gb|DQ222847.1|	black	372.64487	51.114583	0.9751684	0.00467969	13.308
gi|115552411|dbj|AK236416.1|	green	371.94819	77.414337	0.9541842	0.01169095	56.268

gi|114326213|ref|NM_001048069.1|	green	371.66243	75.441404	0.9470287	0.01451824	44.224
gnl|UG|Ssc#S40268747	blue	370.51845	170.11949	0.9529174	0.0121768	1.954
gi|52351606|gb|AY610036.1|	black	369.77221	55.697762	0.9954675	0.00036605	3.846
gi|115547390|dbj|AK237771.1|	turquoise	369.76824	337.87151	0.7367814	0.15555034	17.004
gnl|UG|Ssc#S6075066	blue	369.19274	163.55741	0.9433177	0.01606117	86.122
gi|190360646|ref|NM_001128455.1|	blue	369.09785	170.60201	0.9536843	0.01188188	11.346
gi|115550441|dbj|AK238706.1|	turquoise	369.00426	330.93397	0.7281506	0.16303167	71.08
gnl|UG|Ssc#S17526922	blue	368.56822	158.46518	0.9364259	0.01905765	6.986
gnl|UG|Ssc#S26392586	blue	368.24377	156.59863	0.9336474	0.02031191	9.234
gnl|UG|Ssc#S6666554	darkred	368.01834	34.078868	0.9874356	0.00168744	13.39
gi|52351995|gb|AY610411.1|	turquoise	367.8995	243.08253	0.6683227	0.217532	11.238
gi|52351490|gb|AY609920.1|	magenta	367.51661	11.823045	-0.365511	0.54519745	32.972
gi|115552930|dbj|AK239524.1|	green	367.42797	84.009723	0.974826	0.00477657	15.518
gnl|UG|Ssc#S40215563	blue	367.11998	189.56468	0.9794634	0.00352196	7.45
gnl|UG|Ssc#S26720841	blue	366.88453	181.52834	0.9690951	0.0064916	27.55
gnl|UG|Ssc#S6075552	darkred	366.60342	34.057715	0.9870264	0.00177044	8.896
gnl|UG|Ssc#S32771805	blue	366.38647	189.62381	0.9796327	0.00347859	6.94
gi|194037603|ref|XM_001927821.1|	green	366.35555	80.216315	0.9609628	0.00920432	103.528
gnl|UG|Ssc#S17524990	blue	366.24678	164.08066	0.9441426	0.01571383	45.052
gnl|UG|Ssc#S18549905	white	366.05938	24.980907	0.9731473	0.00526089	27.608
gnl|UG|Ssc#S40091442	grey60	365.93225	14.198292	0.7426603	0.15051345	1.428
gi|163310772|ref|NM_001097521.2|	blue	365.70342	182.60285	0.9706173	0.00601933	57.542
gnl|UG|Ssc#S18381103	green	365.52295	85.32737	0.9770471	0.00415998	17.75
gi|194037682|ref|XM_001927000.1|	blue	365.00824	176.72383	0.9627972	0.00856562	122.908
gnl|UG|Ssc#S18356013	white	364.99449	22.860558	0.9554206	0.01122304	16.624
gnl|UG|Ssc#S26726120	blue	364.51061	157.43158	0.9352906	0.01956697	17.24
gnl|UG|Ssc#S40210420	black	364.18592	54.358922	0.9931345	0.00068218	25.954
gnl|UG|Ssc#S18557837	blue	364.15447	183.97052	0.9726268	0.00541415	198.92
gnl|UG|Ssc#S40191766	turquoise	363.88943	317.15148	0.7203454	0.16988412	2.222
gnl|UG|Ssc#S23697932	blue	363.22223	170.90681	0.9547994	0.01145734	47.392
gi|115548217|dbj|AK231527.1|	darkturquoi	363.17033	26.141834	0.9621267	0.00879734	17.182
gnl|UG|Ssc#S40064881	blue	363.15918	193.43458	0.9844466	0.00232303	8.05
gnl|UG|Ssc#S40453605	blue	362.86239	184.08897	0.9728601	0.00534527	4.894
gnl|UG|Ssc#S39851973	darkturquoi	362.61111	27.426725	0.9718957	0.00563187	20.708
gnl|UG|Ssc#S19539102	blue	362.11314	170.75715	0.9547262	0.01148506	4.474
gnl|UG|Ssc#S40239504	blue	362.03938	187.84754	0.9771759	0.00412506	14.546
gnl|UG|Ssc#S19542751	turquoise	362.00491	291.97817	0.6994744	0.18859852	25.32
gi|52351660|gb|AY610090.1|	blue	361.90177	176.2346	0.9623202	0.00873026	3.19
gi|115554376|dbj|AK233386.1|	blue	361.7914	170.65271	0.9536108	0.01191005	36.966
gi|115552260|dbj|AK230569.1|	blue	360.81639	178.54318	0.9647033	0.00791814	114.53
gi|4186150|emb|AJ236938.1|	blue	360.70407	194.56329	0.9860287	0.00197822	25.462
gi|115553748|dbj|AK239935.1|	blue	360.60196	181.02863	0.9688399	0.00657192	31.144
gi|194040884|ref|XM_001924115.1|	blue	360.3349	172.21689	0.9558367	0.01106694	15.926
gi|194037354|ref|XM_001926198.1|	blue	360.25007	195.32971	0.9872037	0.0017343	37.11
gi|115552309|dbj|AK230619.1|	darkred	360.14689	35.59045	0.9964815	0.0002504	200.272
gnl|UG|Ssc#S34529284	blue	359.93106	167.81708	0.9497343	0.01342577	18.74
gnl|UG|Ssc#S40017556	blue	359.53828	190.47435	0.9811819	0.00309008	2.036
gnl|UG|Ssc#S18379116	green	359.4928	82.846217	0.9691999	0.00645871	111.832

gi|52352707|gb|AY609627.1|	turquoise	359.27873	267.82787	0.6867005	0.20032233	13.064
gnl|UG|Ssc#S17526185	darkred	359.22729	35.62801	0.9967925	0.00021796	3.292
gnl|UG|Ssc#S39996844	blue	359.04391	176.1709	0.9623542	0.00871847	64.698
gi|194036224|ref|XM_001929641.1|	blue	359.00756	166.5636	0.9490599	0.01369545	78.062
gi|194041087|ref|XM_001927225.1|	blue	358.97404	194.77694	0.9867202	0.00183339	15.936
gnl|UG|Ssc#S23758955	turquoise	358.5403	309.95645	0.7159164	0.17380849	43.942
gi|194036639|ref|XM_001924618.1|	blue	358.51117	183.64635	0.9723155	0.00550652	74.728
gnl|UG|Ssc#S26713880	blue	358.25767	175.65592	0.962018	0.00883508	12.588
gnl|UG|Ssc#S35166313	blue	358.16557	191.16745	0.981712	0.00296066	1.328
gi|108796051|ref|NM_213912.2|	darkturquoi	358.11439	27.856939	0.9748068	0.00478201	349.102
gnl|UG|Ssc#S18383248	black	357.58978	51.742086	0.9753319	0.00463365	120.134
gnl|UG|Ssc#S18291250	darkgrey	357.46995	15.421653	0.9552717	0.01127902	7.026
gnl|UG|Ssc#S18557247	turquoise	357.27817	290.69361	0.6978904	0.19004138	67.11
gi|115552482|dbj|AK236487.1|	blue	357.21822	175.46282	0.9618389	0.0088974	3.304
gi|115552097|dbj|AK239290.1|	salmon	357.05681	40.522842	0.9641799	0.00809424	119.648
gnl|UG|Ssc#S40094148	lightyellow	356.95023	33.570878	0.993507	0.00062745	8.678
gi|115548869|dbj|AK235272.1|	blue	356.66925	191.82012	0.9831431	0.0026206	13.528
gnl|UG|Ssc#S29976380	turquoise	356.50118	275.55109	0.7072816	0.18153274	8.376
gnl|UG|Ssc#S23775351	blue	356.48324	183.2327	0.9720833	0.00557574	14.512
gnl|UG|Ssc#S31107148	blue	356.43589	168.0722	0.9514337	0.01275403	4.75
gnl|UG|Ssc#S16766234	blue	356.13256	178.26466	0.9642966	0.00805488	1.6
gnl|UG|Ssc#S35167435	blue	356.10511	189.93353	0.9807625	0.00319374	6.172
gnl|UG|Ssc#S17510588	green	355.86246	81.364516	0.9682756	0.00675065	14.564
gi|115552415|dbj|AK236420.1|	darkred	355.73768	34.26091	0.9881217	0.00155128	7.968
gnl|UG|Ssc#S6007055	darkred	355.56109	34.060697	0.9865076	0.00187752	8.298
gnl|UG|Ssc#S5999689	blue	354.63224	198.70651	0.992074	0.00084605	1.134
gnl|UG|Ssc#S18383932	darkred	354.43921	35.22077	0.9937818	0.00058806	112.768
gi|194042373|ref|XM_001925458.1|	darkred	354.41469	32.666803	0.9772896	0.00409436	25.596
gnl|UG|Ssc#S18354663	blue	354.28572	173.36955	0.958518	0.01007865	8.992
gnl|UG|Ssc#S40149693	blue	354.25342	167.84804	0.9512917	0.0128097	94.116
gnl|UG|Ssc#S40039737	black	354.02484	53.064805	0.9885906	0.00146044	14.846
gnl|UG|Ssc#S23775370	green	353.81498	67.40555	0.922077	0.02580416	31.43
gi|115549499|dbj|AK235521.1|	turquoise	353.63098	285.48607	0.6947987	0.19286658	17.57
gnl|UG|Ssc#S18357539	turquoise	353.61516	228.08875	0.6557709	0.22950928	20.812
gnl|UG|Ssc#S18545870	blue	353.59406	174.9412	0.9613397	0.00907189	11.806
gnl|UG|Ssc#S18354111	salmon	353.48386	40.020625	0.960506	0.00936573	8.064
gi|115551923|dbj|AK236330.1|	blue	352.73449	202.00455	0.9960643	0.00029622	3.62
gnl|UG|Ssc#S17510077	darkred	352.34364	33.016424	0.9794871	0.00351588	27.414
gnl|UG|Ssc#S23766507	blue	352.33201	174.84919	0.9609424	0.00921153	19.38
gi|115555420|dbj|AK234033.1|	blue	352.28767	201.47767	0.9954708	0.00036565	21.762
gnl|UG|Ssc#S23695102	blue	352.20447	201.03787	0.9948844	0.00043888	47.888
gi|115550054|dbj|AK232289.1|	darkred	352.14574	30.298978	0.9610362	0.0091785	7.03
gi|194038023|ref|XM_001928502.1|	white	351.80326	25.704115	0.9850063	0.00219896	71.2
gi|194040485|ref|XM_001926913.1|	darkred	351.75298	35.753687	0.9975705	0.0001437	16.556
gnl|UG|Ssc#S18548560	blue	351.67851	186.20552	0.9761904	0.00439444	13.604
gnl|UG|Ssc#S18554258	lightyellow	351.49674	33.98025	0.9957863	0.00032814	38.596
gnl|UG|Ssc#S6072016	blue	351.25062	197.13342	0.9901977	0.00116329	3.782
gnl|UG|Ssc#S40153033	blue	351.05799	199.31247	0.9930589	0.00069347	4.226

gnl|UG|Ssc#S17510796	blue	350.89959	201.03476	0.9946893	0.00046421	9.65
gnl|UG|Ssc#S6027765	darkred	350.47406	31.936431	0.9721985	0.00554137	2.646
gi|115552865|dbj|AK239459.1|	darkturquoi	350.46742	29.173797	0.9855365	0.00208353	21.222
gnl|UG|Ssc#S40051540	blue	350.3403	188.38479	0.9791255	0.00360905	13.832
gi|115549440|dbj|AK235462.1|	blue	350.08333	166.18942	0.9487292	0.01382835	142.348
gnl|UG|Ssc#S40472465	darkred	349.88884	33.651239	0.9837784	0.00247409	28.786
gi|194040977|ref|XM_001927185.1|	lightyellow	349.84029	33.017731	0.9890383	0.00137542	19.958
gi|194037065|ref|XM_001927928.1|	turquoise	349.6438	332.3609	0.7314298	0.1601772	24.896
gi|47523443|ref|NM_214181.1|	darkturquoi	349.57129	27.804615	0.9734996	0.00515796	16.758
gnl|UG|Ssc#S40133187	turquoise	349.47085	240.33364	0.6535251	0.23167077	2.742
gnl|UG|Ssc#S18554897	blue	349.30825	199.52887	0.9928058	0.00073171	38.686
gnl|UG|Ssc#S40042631	blue	349.19965	171.06425	0.9561578	0.01094701	1125.238
gnl|UG|Ssc#S18553939	turquoise	349.15344	276.16159	0.692138	0.19530739	16.442
gnl|UG|Ssc#S40060840	blue	348.88145	198.15519	0.9918802	0.00087726	55.604
gnl|UG|Ssc#S18555613	turquoise	348.8721	278.66398	0.6911434	0.19622199	26.39
gnl|UG|Ssc#S6057322	blue	348.86978	189.2427	0.9799117	0.00340749	4.088
gnl|UG|Ssc#S29979787	blue	348.80961	192.18876	0.9840168	0.00241982	1.562
gnl|UG|Ssc#S39928606	blue	348.69803	198.04966	0.9915146	0.00093711	0.468
gnl|UG|Ssc#S40583885	blue	348.61799	196.34335	0.9893448	0.00131821	12.152
gi|194034521|ref|XM_001926126.1|	green	348.61735	80.755	0.963403	0.00835804	66.172
gi|52351701|gb|AY610131.1|	darkturquoi	348.54379	25.959601	0.9573626	0.01050082	33.638
gi|115545859|dbj|AK234292.1|	green	348.31473	74.915159	0.9495486	0.01349988	82.328
gnl|UG|Ssc#S32800732	darkred	348.17975	34.822419	0.991844	0.00088311	71.696
gnl|UG|Ssc#S17527454	blue	347.77775	199.34414	0.9927329	0.00074285	14.174
gi|194042562|ref|XM_001925435.1|	blue	347.7404	201.05509	0.9952434	0.00039352	6.956
gnl|UG|Ssc#S19545494	darkred	347.63534	35.997754	0.998758	5.25E-05	12.532
gi|115552627|dbj|AK232832.1|	blue	347.44425	174.15772	0.9604326	0.00939173	9.73
gnl|UG|Ssc#S18359075	darkred	347.41321	32.304206	0.9749922	0.00472946	11.152
gnl|UG|Ssc#S35169280	blue	347.26331	170.30454	0.9547854	0.01146262	10.86
gi|113205635|ref|NM_001044548.1|	darkred	347.14061	36.030359	0.999205	2.69E-05	14.738
gnl|UG|Ssc#S40154377	blue	347.13722	200.74601	0.9949167	0.00043474	0.544
gnl|UG|Ssc#S17511660	darkred	347.12674	34.839273	0.9922195	0.00082289	2.984
gnl|UG|Ssc#S40172065	magenta	346.86075	29.685341	0.91956	0.02705397	27.23
gi|115553025|dbj|AK239611.1|	blue	346.54625	162.98618	0.9448619	0.015413	136.016
gi|194042013|ref|XM_001925277.1|	turquoise	346.51584	325.22064	0.7311085	0.16045624	26.854
gnl|UG|Ssc#S40483020	blue	346.49342	182.70727	0.9725891	0.00542532	6.848
gi|115547780|dbj|AK234756.1|	blue	346.45972	203.28297	0.9978386	0.00012058	1.824
gi|115550393|dbj|AK238658.1|	blue	346.41352	204.28177	0.9992263	2.58E-05	3.912
gnl|UG|Ssc#S31133660	darkred	346.28472	31.949137	0.974206	0.00495365	22.726
gi|178057315|ref|NM_001123097.1|	salmon	346.16171	41.317343	0.9698469	0.00625688	75.254
gnl|UG|Ssc#S26712023	darkred	346.00016	35.430085	0.9956498	0.0003442	2.818
gnl|UG|Ssc#S23756108	salmon	345.90441	41.74733	0.9712923	0.0058137	15.668
gi|115550804|dbj|AK235879.1|	turquoise	345.71997	316.00594	0.7430308	0.1501977	12.506
gi|60097952|ref|NM_001012406.1|	blue	345.66428	196.82025	0.9901087	0.00117915	16.962
gnl|UG|Ssc#S18263925	blue	345.59976	198.47212	0.9917608	0.00089665	1.24
gi|115553495|dbj|AK236698.1|	white	345.5913	25.135352	0.9805022	0.00325865	57.332
gnl|UG|Ssc#S31120434	blue	345.56375	191.21546	0.98305	0.0026423	1.78
gnl|UG|Ssc#S40110683	blue	345.26497	198.40048	0.9924595	0.00078513	38.494

gi|115546292|dbj|AK240530.1|	lightyellow	345.08171	34.561116	0.9993134	2.16E-05	39.326
gi|115553080|dbj|AK239666.1|	turquoise	344.72904	273.39763	0.6909015	0.1964447	14.65
gnl|UG|Ssc#S40478259	blue	344.2656	201.34546	0.9957454	0.00033292	42.382
gnl|UG|Ssc#S23758349	magenta	343.54938	31.651582	0.9389761	0.01792951	38.09
gnl|UG|Ssc#S40312675	blue	342.32044	199.52243	0.9939828	0.00055981	20.688
gnl|UG|Ssc#S18379252	blue	342.30815	204.28213	0.9995186	1.27E-05	19.29
gi|47523723|ref|NM_214332.1|	blue	342.23371	203.62895	0.9985219	6.82E-05	152.87
gnl|UG|Ssc#S17526987	blue	342.2061	179.41722	0.9685948	0.00664937	43.722
gnl|UG|Ssc#S23755373	white	342.03562	27.38695	0.9915675	0.00092836	4.622
gi|115555058|dbj|AK240257.1|	magenta	341.7857	30.829015	0.9411806	0.0169725	47.928
gnl|UG|Ssc#S40214545	white	341.78024	26.151249	0.980565	0.00324294	20.572
gnl|UG|Ssc#S18357398	magenta	341.30302	32.580129	0.9471461	0.01447027	24.958
gi|56606056|ref|NM_001008481.1|	lightyellow	341.28284	34.344034	0.9977143	0.00013113	46.332
gnl|UG|Ssc#S17526265	turquoise	341.26003	266.35878	0.6767253	0.20961438	18.534
gnl|UG|Ssc#S6085659	blue	340.92452	204.16958	0.9994154	1.70E-05	6.87
gnl|UG|Ssc#S40034650	black	340.88227	50.651046	0.9744853	0.0048736	23.262
gnl|UG|Ssc#S19546165	blue	340.81412	154.6308	0.9304991	0.02176404	37.032
gnl|UG|Ssc#S34521528	magenta	340.69916	31.100653	0.9371219	0.01874751	11.652
gnl|UG|Ssc#S23698843	blue	340.49188	192.51539	0.9849514	0.00221102	2.62
gnl|UG|Ssc#S23769348	blue	340.05483	158.10873	0.937279	0.01867775	5.844
gnl|UG|Ssc#S23693714	green	340.03061	70.28444	0.933691	0.02029201	81.758
gnl|UG|Ssc#S39772429	white	339.96546	27.54092	0.9958973	0.00031526	43.988
gnl|UG|Ssc#S35325947	blue	339.93318	202.91292	0.9981245	9.75E-05	3.736
gi|115553527|dbj|AK236730.1|	blue	339.81777	197.32465	0.9906312	0.00108705	13.482
gi|115547541|dbj|AK237923.1|	magenta	339.79211	29.747349	0.9106949	0.03160412	361.506
gnl|UG|Ssc#S17510046	darkgrey	339.59768	15.094287	0.9406716	0.01719193	33.752
gi|208610189|ref|NM_001135680.1|	turquoise	339.51997	322.75084	0.7493724	0.14482244	51.706
gnl|UG|Ssc#S17512211	blue	339.37176	198.30862	0.9918436	0.00088318	0.892
gi|209863046|ref|NM_001135965.1|	darkturquoi	339.28713	28.440228	0.9790001	0.00364157	32.16
gnl|UG|Ssc#S18553195	turquoise	339.26019	221.88441	0.6342823	0.25041555	127.936
gnl|UG|Ssc#S23690384	blue	339.17238	188.26884	0.9790924	0.00361762	0.662
gnl|UG|Ssc#S6063101	blue	339.16256	201.31206	0.9963885	0.00026039	7.798
gnl|UG|Ssc#S17514667	lightyellow	339.15955	30.109966	0.9679765	0.00684604	21.104
gnl|UG|Ssc#S41575095	turquoise	339.06307	278.33569	0.6905135	0.19680188	24.898
gnl|UG|Ssc#S40106980	blue	339.01802	195.91949	0.9886655	0.0014461	3.834
gnl|UG|Ssc#S17518466	blue	337.89917	184.85167	0.974034	0.00500313	14.352
gnl|UG|Ssc#S40288165	lightyellow	337.89752	33.680487	0.9930954	0.00068801	5.066
gi|115550999|dbj|AK238860.1|	darkred	337.75233	34.409654	0.9892948	0.00132748	45.242
gnl|UG|Ssc#S19538990	black	337.72195	52.432071	0.9789034	0.0036667	6.618
gi|194041588|ref|XM_001927189.1|	blue	337.67335	182.06997	0.9703247	0.00610919	28.9
gnl|UG|Ssc#S19547691	magenta	337.66578	31.740237	0.9490325	0.01370648	104.022
gnl|UG|Ssc#S17527442	turquoise	337.49568	251.49995	0.6727894	0.21331292	3.408
gnl|UG|Ssc#S17513694	lightyellow	337.43674	30.295523	0.9688067	0.00658239	7.858
gnl|UG|Ssc#S26651783	blue	337.26162	201.14987	0.9958946	0.00031558	11.066
gnl|UG|Ssc#S22317157	blue	337.01678	176.67246	0.9644493	0.00800343	18.314
gnl|UG|Ssc#S19545704	darkgrey	336.98095	15.264936	0.9475793	0.01429366	16.692
gnl|UG|Ssc#S19546082	lightyellow	336.90062	34.393915	0.998163	9.45E-05	78.67
gnl|UG|Ssc#S40042081	blue	336.85624	198.18045	0.9923335	0.00080488	7.116

gnl|UG|Ssc#S18268314	lightyellow	336.51489	33.17474	0.9898359	0.00122821	11.634
gnl|UG|Ssc#S40132479	darkturquoi	336.50256	27.725262	0.9713643	0.0057919	6.64
gi|52352072|gb|AY610488.1|	turquoise	336.50168	236.03945	0.6492887	0.23576333	27.326
gnl|UG|Ssc#S18336060	darkturquoi	336.399	25.178439	0.9485559	0.01389814	6.366
gnl|UG|Ssc#S40527077	blue	336.35238	199.91564	0.9945654	0.00048054	5.416
gnl|UG|Ssc#S39778104	darkred	336.27506	34.815522	0.9918815	0.00087704	23.47
gnl|UG|Ssc#S6065016	blue	335.94451	201.61728	0.9965731	0.0002407	4.456
gnl|UG|Ssc#S23690342	magenta	335.72761	29.839189	0.9114708	0.03119688	47.978
gi|194037611|ref|XM_001925424.1|	magenta	335.65631	29.352	0.9113953	0.03123639	41.358
gnl|UG|Ssc#S40329149	green	335.63692	76.713394	0.9517151	0.01264386	19.972
gnl|UG|Ssc#S40144457	lightyellow	335.00828	34.325836	0.9977021	0.00013219	83.77
gi|2286009|emb|AJ000786.1|	blue	334.93942	199.43127	0.9936027	0.00061363	50.004
gnl|UG|Ssc#S18547326	white	334.87458	26.075591	0.9823485	0.00280771	20.548
gi|115550230|dbj|AK235709.1|	turquoise	334.7384	251.31592	0.670739	0.21524685	28.362
gnl|UG|Ssc#S23762471	darkred	334.72513	34.969252	0.9928999	0.00071741	17.476
gnl|UG|Ssc#S16767178	blue	334.62403	166.71891	0.9505021	0.01312087	8.248
gnl|UG|Ssc#S39762052	blue	334.55256	197.12425	0.9906021	0.00109211	4.958
gi|178056609|ref|NM_001123174.1|	green	334.2942	78.74204	0.9586348	0.01003628	26.558
gnl|UG|Ssc#S40443043	black	334.03961	40.86464	0.9278366	0.02301717	11.476
gnl|UG|Ssc#S18556589	white	333.91841	25.744738	0.9709082	0.00593043	144.144
gi|115552841|dbj|AK239435.1|	blue	333.89118	200.42204	0.9950743	0.00041468	4.494
gnl|UG|Ssc#S23690807	white	333.60366	27.276892	0.9874324	0.00168808	12.108
gi|156151354|ref|NM_001037146.2|	blue	333.35395	202.314	0.997413	0.0001579	11.95
gnl|UG|Ssc#S40227949	blue	333.34114	187.61308	0.9790346	0.00363259	11.502
gnl|UG|Ssc#S40499571	blue	333.28979	182.41655	0.9726172	0.005417	2.732
gi|82617535|ref|NM_001037322.1|	lightyellow	333.10998	33.402435	0.9902824	0.00114825	54.896
gnl|UG|Ssc#S39957439	black	332.82752	51.127268	0.9725431	0.00543893	30.492
gi|194035451|ref|XM_001928072.1|	salmon	332.43682	44.435973	0.9848057	0.00224316	30.77
gnl|UG|Ssc#S40111130	blue	332.42935	203.17932	0.9987191	5.50E-05	3.77
gnl|UG|Ssc#S23768102	blue	332.35668	202.92878	0.9984083	7.62E-05	3.72
gnl|UG|Ssc#S17518793	turquoise	332.13446	256.64723	0.6717036	0.21433643	21.148
gi|52351374|gb|AY609804.1|	blue	331.65639	200.56604	0.9953109	0.00038518	4.796
gnl|UG|Ssc#S18387207	blue	330.74076	195.62366	0.9895117	0.00128738	14.478
gnl|UG|Ssc#S18378854	lightyellow	330.63771	30.961356	0.9743634	0.00490849	27.306
gi|194040979|ref|XM_001928486.1|	lightyellow	330.56341	31.152334	0.9749221	0.00474931	232.03
gi|194033964|ref|XM_001925719.1|	blue	330.48681	179.21146	0.9667957	0.00722687	1.432
gnl|UG|Ssc#S23758340	blue	330.36484	192.63314	0.9849239	0.00221709	5.25
gnl|UG|Ssc#S17516095	blue	330.36215	181.52645	0.9711239	0.00586478	13.166
gnl|UG|Ssc#S40494040	blue	330.33573	198.28826	0.9927697	0.00073722	4.418
gi|52351125|gb|AY609530.1|	green	330.13052	77.504682	0.9554525	0.01121103	68.966
gi|115548591|dbj|AK231575.1|	turquoise	330.08607	238.20449	0.6528719	0.23230053	18.768
gnl|UG|Ssc#S17510233	lightyellow	329.9665	34.105438	0.9958292	0.00032314	78.914
gnl|UG|Ssc#S18552822	darkred	329.76885	30.88016	0.9661569	0.00743571	7.288
gnl|UG|Ssc#S23759508	turquoise	329.47615	238.83572	0.6562382	0.22906022	644.394
gnl|UG|Ssc#S27603973	lightyellow	329.45145	30.159171	0.9660709	0.00746395	4.388
gnl|UG|Ssc#S17499579	turquoise	329.31328	265.53382	0.6817519	0.20491729	2.364
gnl|UG|Ssc#S18553137	black	329.29383	52.324139	0.9858962	0.0020064	108.018
gnl|UG|Ssc#S18555551	turquoise	329.26506	266.54662	0.6778703	0.20854183	46.6

gnl|UG|Ssc#S6076883	blue	328.85702	136.54212	0.9016576	0.03646976	9.844
gnl|UG|Ssc#S40522263	salmon	328.83491	38.003496	0.9515443	0.0127107	76.646
gnl|UG|Ssc#S22282893	lightyellow	328.81118	33.464099	0.9907218	0.00107133	41.916
gnl|UG|Ssc#S18360885	salmon	328.47113	40.854295	0.9642265	0.00807854	78.794
gi|115555561|dbj|AK237193.1|	blue	328.4439	202.95764	0.9987993	4.99E-05	1.92
gnl|UG|Ssc#S40335524	darkred	328.20891	33.776954	0.9856415	0.00206092	3.788
gi|115552246|dbj|AK230555.1|	turquoise	328.05029	291.34033	0.7214926	0.1688719	25.69
gi|56792885|gb|AY705918.1|	blue	328.0369	200.43107	0.9956267	0.00034695	2.144
gnl|UG|Ssc#S40474761	magenta	328.00588	33.096199	0.947462	0.01434142	3.782
gnl|UG|Ssc#S40427363	blue	327.76977	188.47411	0.980739	0.00319959	5.176
gnl|UG|Ssc#S18382284	magenta	327.50313	32.069556	0.95026	0.01321676	22.996
gi|115551631|dbj|AK239095.1|	blue	327.46668	169.12866	0.9551523	0.01132402	16.574
gi|115554007|dbj|AK233216.1|	turquoise	327.27814	285.10549	0.7062884	0.18242737	5.9
gi|115549396|dbj|AK235418.1|	black	327.22523	41.072998	0.9236626	0.02502668	23.176
gnl|UG|Ssc#S19541572	green	327.17785	75.359006	0.9492015	0.01363872	11.112
gi|115545742|dbj|AK234175.1|	blue	326.69147	198.72491	0.9939649	0.0005623	3.612
gi|52351717|gb|AY610147.1|	turquoise	326.5085	250.25169	0.673201	0.2129253	9.866
gnl|UG|Ssc#S19541067	magenta	326.38769	34.684102	0.9782687	0.00383301	29.118
gnl|UG|Ssc#S40097075	blue	326.21091	193.53427	0.987211	0.00173282	3.838
gi|115547352|dbj|AK234725.1|	blue	325.87541	180.58437	0.9700957	0.00617983	51
gi|115553468|dbj|AK236671.1|	blue	325.68329	179.41509	0.9688409	0.00657159	85.648
gi|194043731|ref|XM_001926974.1|	lightyellow	325.2829	30.566262	0.9717069	0.00568858	22.458
gi|115546137|dbj|AK240375.1|	black	324.98525	51.572545	0.9790185	0.00363678	9.032
gnl|UG|Ssc#S17512237	blue	324.8194	200.59347	0.9959486	0.00030937	3.754
gi|194044572|ref|XM_001925488.1|	blue	324.58957	196.1388	0.99085	0.00104923	22.736
gnl|UG|Ssc#S23701300	blue	324.55615	162.21872	0.9425655	0.01638008	24.396
gi|115554358|dbj|AK233368.1|	turquoise	324.3132	249.44921	0.6621786	0.22337267	66.846
gnl|UG|Ssc#S17505417	white	324.26898	28.317728	0.9983427	8.10E-05	11.842
gnl|UG|Ssc#S40072585	blue	324.17916	195.90899	0.9898953	0.00121747	7.222
gi|47523473|ref|NM_214198.1|	blue	324.11302	171.60862	0.9584226	0.01011328	1.156
gi|115551974|dbj|AK239167.1|	blue	324.09655	174.02392	0.9610066	0.00918891	3.454
gi|194035631|ref|XM_001924325.1|	green	323.94794	69.806432	0.9343774	0.01997985	133.328
gnl|UG|Ssc#S40358887	blue	323.81668	201.81868	0.9976742	0.0001346	1.726
gnl|UG|Ssc#S18356219	darkred	323.76705	32.190614	0.9754507	0.00460031	53.108
gi|194037139|ref|XM_001925875.1|	darkred	323.68632	33.357324	0.9828178	0.00269667	52.936
gi|115555021|dbj|AK240220.1|	blue	323.67272	185.72135	0.9772274	0.00411116	35.408
gnl|UG|Ssc#S18546038	white	323.47528	26.233891	0.9837843	0.00247273	60.09
gnl|UG|Ssc#S23767489	white	323.447	27.967717	0.9991053	3.21E-05	4.794
gi|52351129|gb|AY609534.1|	blue	323.3105	184.8877	0.9761188	0.00441425	14.4
gnl|UG|Ssc#S5997273	blue	322.99532	199.29795	0.9943428	0.00051035	3.904
gi|194038424|ref|XM_001926793.1|	turquoise	322.32546	282.04713	0.6914668	0.19592455	28.57
gi|16304807|emb|AJ416019.1|	darkturquoi	322.23077	29.072162	0.9846073	0.00228719	14.718
gi|115546934|dbj|AK240580.1|	blue	321.94533	177.42973	0.9661042	0.007453	6.408
gi|194044550|ref|XM_001927682.1|	salmon	321.7828	44.664294	0.9855027	0.00209082	104.03
gnl|UG|Ssc#S18556194	turquoise	321.58562	262.62088	0.673695	0.21246038	22.28
gnl|UG|Ssc#S31107474	green	321.58229	74.894312	0.946305	0.01481513	14.56
gnl|UG|Ssc#S34516415	darkred	321.56502	33.696907	0.9849407	0.00221339	2.11
gi|115549275|dbj|AK235196.1|	turquoise	321.42604	224.45274	0.6358267	0.24889666	100.08

gi|115550125|dbj|AK235604.1|	darkred	321.38929	33.19001	0.9817246	0.00295759	20.59
gnl|UG|Ssc#S31986007	blue	321.29596	183.72657	0.9743308	0.00491784	6.382
gnl|UG|Ssc#S40186233	darkred	321.25006	32.368698	0.9767203	0.0042489	515.072
gi|56711365|ref|NM_001008688.1|	magenta	321.02613	29.290843	0.8933755	0.0411196	801.904
gnl|UG|Ssc#S5991729	turquoise	320.81054	216.27438	0.6350904	0.24962044	5.548
gnl|UG|Ssc#S26732528	blue	320.65359	170.32994	0.954493	0.0115735	6.594
gnl|UG|Ssc#S40424734	blue	320.17916	198.95146	0.9946619	0.00046781	12.716
gi|115553601|dbj|AK239788.1|	blue	319.85519	195.61266	0.9897577	0.00124241	5.924
gi|113205873|ref|NM_001044606.1|	darkred	319.69414	32.525825	0.9778187	0.00395242	118.01
gi|186886351|gb|EU617320.1|	lightyellow	319.36755	29.238293	0.9575265	0.01044058	19.052
gnl|UG|Ssc#S15981525	blue	319.28996	162.55951	0.9451584	0.01528951	5.748
gi|47523743|ref|NM_214342.1|	blue	319.28276	135.26174	0.8991968	0.03783267	32.77
gnl|UG|Ssc#S19542031	white	319.07531	25.797965	0.9762338	0.00438247	290.744
gnl|UG|Ssc#S40496270	lightyellow	318.65923	25.630063	0.9287343	0.0225921	39.698
gi|115547214|dbj|AK234587.1|	blue	318.44639	192.53846	0.9868094	0.00181498	1.81
gi|113205929|ref|NM_001044620.1|	blue	318.33388	195.62475	0.9907477	0.00106685	121.186
gi|194038241|ref|XM_001927985.1|	blue	318.31121	141.97238	0.91084	0.0315278	58.374
gi|115553414|dbj|AK236617.1|	darkred	318.24513	32.710125	0.9791717	0.00359711	35.208
gi|194035643|ref|XM_001927050.1|	salmon	318.21824	43.035195	0.9781767	0.00385734	152.628
gi|115553473|dbj|AK236676.1|	blue	318.07678	185.96979	0.9770449	0.00416055	15.36
gnl|UG|Ssc#S18558403	lightyellow	317.6561	29.751568	0.9611915	0.00912389	12.75
gi|52351961|gb|AY610379.1|	white	317.36765	26.413305	0.9785712	0.00375344	54.334
gi|52351299|gb|AY609731.1|	blue	317.36674	195.61925	0.9902662	0.00115112	0.382
gnl|UG|Ssc#S35166842	blue	317.36674	195.61925	0.9902662	0.00115112	0.152
gnl|UG|Ssc#S40035688	blue	317.36674	195.61925	0.9902662	0.00115112	0.306
gi|194043997|ref|XM_001926349.1|	green	316.76899	71.268537	0.9372129	0.01870711	72.19
gi|194044782|ref|XM_001926654.1|	yellow	316.31695	50.919514	0.8945404	0.04045491	10.798
gnl|UG|Ssc#S19548011	darkturquoi	316.13537	27.410353	0.9675728	0.00697547	11.056
gi|115554459|dbj|AK233469.1|	blue	316.12076	157.49425	0.937714	0.01848502	20.202
gi|194035227|ref|XM_001928768.1|	salmon	316.00483	42.484881	0.9752342	0.00466115	123.438
gnl|UG|Ssc#S19549226	blue	315.92726	193.31381	0.9869256	0.00179108	14.95
gnl|UG|Ssc#S18355994	brown	315.85032	80.118112	0.9317438	0.02118606	13.596
gi|194038719|ref|XM_001926310.1|	darkgrey	315.81589	15.322183	0.9482303	0.01402962	17.496
gi|115551642|dbj|AK239106.1|	darkturquoi	315.7751	26.147127	0.9575626	0.01042734	70.624
gnl|UG|Ssc#S23765932	magenta	315.5941	33.565214	0.9873955	0.0016955	60.388
gnl|UG|Ssc#S19537215	lightyellow	315.55599	30.880481	0.9702668	0.00612701	8.31
gnl|UG|Ssc#S40290915	lightyellow	315.31185	33.300697	0.9911164	0.00100378	13.724
gi|115546454|dbj|AK230973.1|	turquoise	315.01829	264.57457	0.6934088	0.19414054	81.49
gi|27357069|gb|AY166682.1|	turquoise	314.87326	287.01996	0.7022879	0.18604336	6.14
gi|52351160|gb|AY609565.1|	blue	314.8444	163.2657	0.9451664	0.01528619	3.096
gnl|UG|Ssc#S31133234	salmon	314.19587	45.572691	0.9907311	0.00106973	9.824
gi|115547065|dbj|AK231120.1|	brown	314.12052	94.646403	0.9671712	0.00710505	13.93
gnl|UG|Ssc#S18357732	brown	313.95202	92.845017	0.9629221	0.00852268	21.926
gnl|UG|Ssc#S18361423	lightyellow	313.91697	28.304914	0.9501399	0.01326445	91.978
gi|52351120|gb|AY609525.1|	turquoise	313.85053	218.87444	0.6305386	0.25410798	93.798
gnl|UG|Ssc#S19542447	salmon	313.5972	43.52735	0.9789347	0.00365853	22.01
gnl|UG|Ssc#S16444449	magenta	313.2348	33.240152	0.9768621	0.00421024	17.754
gi|115549902|dbj|AK232136.1|	brown	313.13469	96.204853	0.971384	0.00578594	48.75

gnl|UG|Ssc#S26734833	blue	312.60874	171.81521	0.9586802	0.01001983	8.724
gnl|UG|Ssc#S6085378	yellow	312.5962	58.346567	0.9303225	0.02184647	11.222
gnl|UG|Ssc#S18551834	brown	312.49602	86.977876	0.9459377	0.01496655	32.902
gnl|UG|Ssc#S40153514	turquoise	312.27725	241.67415	0.6546998	0.23053949	73.922
gi|194044123|ref|XM_001926788.1|	lightyellow	312.0828	28.406033	0.9519259	0.01256156	82.416
gnl|UG|Ssc#S6030301	darkturquoi	311.98654	27.840538	0.9770345	0.00416337	0.314
gi|54607194|ref|NM_001006592.1|	brown	311.68312	95.949689	0.9717591	0.00567286	5876.728
gnl|UG|Ssc#S26722558	blue	311.65918	175.41826	0.9634223	0.00835145	35.902
gnl|UG|Ssc#S31128151	green	311.43176	56.174255	0.8876505	0.04443582	16.16
gi|194036603|ref|XM_001929372.1|	green	311.38231	70.574897	0.9361416	0.01918477	329.73
gi|158631261|ref|NM_001099936.1|	turquoise	311.37388	210.14724	0.621995	0.2625885	102.806
gi|47523679|ref|NM_214307.1|	turquoise	311.30632	254.49059	0.6800088	0.20654277	44.098
gnl|UG|Ssc#S39777926	yellow	311.2896	62.108953	0.9557349	0.01110507	325.156
gnl|UG|Ssc#S40117649	blue	311.16905	180.73546	0.9709742	0.00591031	12.36
gnl|UG|Ssc#S18555750	lightyellow	311.09867	30.010307	0.963856	0.00820388	6.158
gnl|UG|Ssc#S29979418	magenta	311.09231	32.39811	0.9454225	0.01517984	0.828
gi|194035607|ref|XM_001928505.1|	turquoise	311.07646	209.01146	0.6246541	0.25994104	86.834
gnl|UG|Ssc#S19542649	blue	310.80793	165.10065	0.9513683	0.01277965	100.612
gnl|UG|Ssc#S29465510	lightyellow	310.77948	28.883024	0.9553281	0.01125779	1260.64
gnl|UG|Ssc#S18263529	brown	310.63664	103.0007	0.986833	0.00181011	132.176
gi|52352028|gb|AY610444.1|	turquoise	310.62614	221.29547	0.6385601	0.24621435	7.566
gnl|UG|Ssc#S6081340	blue	310.45258	191.85722	0.9860256	0.00197889	3.89
gnl|UG|Ssc#S17500067	white	310.19502	26.425748	0.9915282	0.00093485	3.352
gnl|UG|Ssc#S26395675	brown	310.13352	93.14599	0.9636792	0.00826393	61.018
gi|194042230|ref|XM_001927832.1|	yellow	309.93299	47.073251	0.8629129	0.05966098	37.096
gi|194043920|ref|XM_001925866.1|	brown	309.48649	102.62762	0.9858983	0.00200595	19.466
gi|194042787|ref|XM_001924736.1|	white	309.34494	25.151187	0.9691339	0.00647941	94.36
gnl|UG|Ssc#S22273263	blue	309.30368	187.34213	0.980667	0.00321751	5.394
gi|194035184|ref|XM_001924626.1|	midnightblu	309.29265	33.319705	0.9609485	0.00920939	78.394
gnl|UG|Ssc#S19541658	blue	308.94556	177.40156	0.9667234	0.00725042	22.226
gi|115545844|dbj|AK234277.1|	brown	308.83327	95.213596	0.9700337	0.00619899	68.872
gnl|UG|Ssc#S34514135	darkorange	308.3701	25.378456	0.9595093	0.009721	7.412
gnl|UG|Ssc#S18553403	brown	308.36914	102.78161	0.9868592	0.00180471	253.552
gnl|UG|Ssc#S40401313	brown	308.29448	88.295302	0.9521153	0.01248777	391.684
gnl|UG|Ssc#S35167355	darkred	308.28994	34.070436	0.9878242	0.00160985	4.306
gnl|UG|Ssc#S35322980	darkred	308.23828	32.091454	0.9755014	0.00458611	14.178
gi|194036467|ref|XM_001928805.1|	black	308.18986	48.055048	0.9626745	0.00860786	38.788
gi|52351285|gb|AY609717.1|	blue	308.17322	184.54481	0.9754999	0.00458653	1
gi|178056668|ref|NM_001123099.1|	brown	308.0895	94.786304	0.9671354	0.00711661	378.642
gi|52351676|gb|AY610106.1|	brown	308.08085	99.183893	0.9774568	0.00404933	18.534
gi|194044568|ref|XM_001928462.1|	darkturquoi	307.96758	29.893915	0.9907751	0.00106212	61.536
gnl|UG|Ssc#S19547872	green	307.86977	54.417171	0.8809105	0.04844287	6.698
gnl|UG|Ssc#S17527330	magenta	307.73259	32.300262	0.9313523	0.02136731	30.17
gi|47523581|ref|NM_214257.1|	brown	307.67201	105.56002	0.9934621	0.00063397	18.068
gnl|UG|Ssc#S18332789	blue	307.65388	147.68717	0.9225598	0.02556663	13.554
gnl|UG|Ssc#S19545397	brown	307.61882	106.00713	0.9946028	0.0004756	85.74
gnl|UG|Ssc#S40482470	magenta	307.42936	31.882702	0.9718348	0.00565014	10.166
gnl|UG|Ssc#S31122485	brown	307.36506	94.106308	0.9682008	0.00677448	15.522

gi|194018685|ref|NM_001129963.1|	brown	307.36222	104.41891	0.9911396	0.00099986	535.856
gi|115549744|dbj|AK232023.1|	brown	307.33951	100.5741	0.9811315	0.00310246	173.062
gi|194033649|ref|XM_001925294.1|	blue	307.27893	162.54133	0.9436303	0.01592926	17.394
gi|52351406|gb|AY609836.1|	violet	307.14302	14.660541	0.8663455	0.05746525	55.84
gi|115552967|dbj|AK239553.1|	magenta	307.08012	32.654581	0.9356645	0.01939875	34.482
gi|47523845|ref|NM_214396.1|	brown	307.05181	104.64653	0.9915342	0.00093387	202.534
gnl|UG|Ssc#S23690002	brown	307.04956	101.72295	0.9840389	0.00241482	13.712
gnl|UG|Ssc#S25135664	darkturquoi	306.96896	28.606369	0.9790117	0.00363853	41.048
gi|178056503|ref|NM_001123076.1|	brown	306.8789	93.156466	0.9629594	0.00850988	201.504
gnl|UG|Ssc#S18356838	magenta	306.8441	33.208466	0.9803151	0.00330557	10.564
gnl|UG|Ssc#S40012957	turquoise	306.77275	254.00938	0.6706162	0.21536281	1.348
gi|115551993|dbj|AK239186.1|	green	306.7581	68.094567	0.9243481	0.02469295	112.6
gnl|UG|Ssc#S26734006	blue	306.62963	172.70382	0.9601245	0.00950121	4.824
gi|47522685|ref|NM_213898.1|	lightyellow	306.61296	31.668131	0.9788806	0.00367262	32.222
gi|194035136|ref|XM_001924656.1|	darkgreen	306.43994	18.621348	0.88743	0.04456513	23.176
gi|47523445|ref|NM_214183.1|	yellow	306.34129	60.437036	0.9399583	0.01750101	55.874
gnl|UG|Ssc#S40414470	brown	306.16852	80.367357	0.9320734	0.02103385	50.678
gi|201066357|ref|NM_001134968.1|	brown	306.1313	102.04944	0.9856522	0.00205861	208.308
gnl|UG|Ssc#S38481847	blue	306.06774	186.21786	0.9790115	0.0036386	13.152
gnl|UG|Ssc#S23700159	greenyellow	306.02878	40.92867	0.9700585	0.00619133	31.888
gi|115546978|dbj|AK231033.1|	darkred	305.85216	30.856167	0.967486	0.00700341	16.628
gnl|UG|Ssc#S40174963	brown	305.63879	107.06243	0.9969823	0.00019891	134.524
gnl|UG|Ssc#S34531282	green	305.52528	66.48399	0.9191304	0.02726922	66.508
gnl|UG|Ssc#S40226524	brown	305.38393	101.43963	0.9851847	0.0021599	20.17
gnl|UG|Ssc#S6055465	blue	305.37349	193.81357	0.988878	0.00140566	4.19
gnl|UG|Ssc#S17512154	greenyellow	305.01639	40.085822	0.961985	0.00884657	32.618
gi|115555675|dbj|AK237308.1|	turquoise	304.81044	259.14175	0.6765508	0.20977794	28.796
gi|194039226|ref|XM_001928899.1|	brown	304.78109	96.731401	0.9723301	0.00550217	260.806
gnl|UG|Ssc#S29460541	brown	304.65127	79.319834	0.9286148	0.02264856	6.732
gnl|UG|Ssc#S18553035	brown	304.59279	101.64891	0.984573	0.00229482	98.324
gnl|UG|Ssc#S26727199	lightyellow	304.53881	28.95932	0.9548294	0.01144597	11.168
gi|115553121|dbj|AK239707.1|	lightyellow	304.49818	28.564856	0.9514063	0.01276476	60.23
gnl|UG|Ssc#S34527927	magenta	304.387	32.744326	0.9418854	0.01667016	50.096
gi|157427686|ref|NM_214374.2|	brown	304.36181	97.689052	0.9769144	0.00419601	604.248
gi|194034424|ref|XM_001926336.1|	lightyellow	304.34178	27.660944	0.9440718	0.01574357	19.786
gi|115548383|dbj|AK235000.1|	turquoise	304.2879	218.0053	0.6375396	0.24721485	59.764
gnl|UG|Ssc#S32419644	brown	303.96451	107.27614	0.9977002	0.00013235	120.84
gi|115545669|dbj|AK230784.1|	brown	303.93625	99.606216	0.9803629	0.00329356	78.984
gi|194034436|ref|XM_001927720.1|	darkorange	303.6347	28.089263	0.980594	0.00323572	76.488
gi|115549680|dbj|AK231959.1|	white	303.3682	28.214665	0.9934034	0.00064251	202.738
gi|115550848|dbj|AK235923.1|	brown	303.33755	96.035468	0.9704289	0.00607713	107.562
gi|194018701|ref|NM_001129947.1|	brown	303.03826	108.01503	0.9995491	1.15E-05	1476.36
gi|115549608|dbj|AK238416.1|	brown	302.83197	104.4682	0.9912508	0.0009811	80.514
gi|55741810|ref|NM_001001863.1|	brown	302.71506	101.06436	0.9847452	0.00225657	1158.53
gi|194041001|ref|XM_001924658.1|	blue	302.6184	189.48374	0.9835409	0.00252854	4.006
gnl|UG|Ssc#S39979775	blue	302.45712	185.0672	0.9778558	0.00394254	5.41
	greenyellow					

gi|194271212|gb|EU714326.1|	greenyellow	301.84959	36.524442	0.9422239	0.01652556	47.584
gnl|UG|Ssc#S40099717	darkturquoi	301.79447	28.390787	0.9819806	0.0028958	5.672
gnl|UG|Ssc#S18383473	greenyellow	301.74423	41.793262	0.9735615	0.00513996	49.072
gnl|UG|Ssc#S31114548	brown	301.66327	96.204951	0.9710735	0.00588008	8.402
gi|118403765|ref|NM_001078687.1|	brown	301.44609	94.69122	0.970128	0.00616985	107.734
gi|47523641|ref|NM_214289.1|	blue	301.35831	186.49424	0.9795169	0.00350824	21.048
gnl|UG|Ssc#S19540021	black	301.29272	29.289435	0.8533683	0.06589998	7.5
gnl|UG|Ssc#S17510398	brown	301.2144	86.673404	0.9437293	0.01588756	556.998
gnl|UG|Ssc#S40527867	brown	300.89018	85.471268	0.9403672	0.01732362	32.928
gnl|UG|Ssc#S19550257	midnightblu	300.68746	35.415118	0.9760752	0.00442632	6.276
gnl|UG|Ssc#S23756816	black	300.68708	49.327142	0.9680777	0.00681373	15.292
gnl|UG|Ssc#S29982561	salmon	300.59891	44.802244	0.9866739	0.00184296	12.802
gnl|UG|Ssc#S39988753	blue	300.56771	175.81907	0.9658007	0.00755298	0.612
gnl|UG|Ssc#S17509886	magenta	300.44972	31.848467	0.9351589	0.01962635	398.152
gi|115551270|dbj|AK232704.1|	brown	300.2558	87.278038	0.9462902	0.01482124	124.826
gnl|UG|Ssc#S23770961	brown	300.23777	81.686571	0.9351818	0.01961602	51.16
gi|47523567|ref|NM_214244.1|	brown	300.08116	86.667074	0.9439025	0.0158147	27.844
gnl|UG|Ssc#S17524983	blue	300.02448	133.15797	0.8978679	0.0385753	20.698
gi|47522691|ref|NM_213897.1|	brown	299.71064	95.305313	0.9676922	0.00693712	56.34
gnl|UG|Ssc#S39839770	white	299.46958	26.486026	0.9762195	0.00438643	7.41
gnl|UG|Ssc#S18558146	turquoise	299.31969	225.11508	0.6396702	0.24512733	9.42
gnl|UG|Ssc#S6058262	white	299.084	25.552064	0.9654662	0.00766368	5.812
gnl|UG|Ssc#S6075829	yellow	299.02416	55.582956	0.934709	0.01982961	76.24
gnl|UG|Ssc#S39992562	blue	298.97599	164.72895	0.9505883	0.01308679	1.946
gnl|UG|Ssc#S35329402	white	298.96285	19.896015	0.1901631	0.75934418	131.592
gnl|UG|Ssc#S31114666	darkturquoi	298.86459	25.834594	0.9593569	0.00977573	33.172
gnl|UG|Ssc#S40397505	black	298.83324	36.712002	0.9016876	0.03645323	5.742
gnl|UG|Ssc#S39998964	blue	298.69015	191.07408	0.9859235	0.00200059	3.92
gnl|UG|Ssc#S40212608	brown	298.3455	97.724935	0.9741063	0.00498231	12.312
gi|10304379|gb|AF288822.1|AF28882	blue	298.2811	170.26651	0.9582259	0.01018483	85.772
gnl|UG|Ssc#S40097726	blue	298.20977	186.63719	0.9801861	0.00333805	1.216
gi|115549689|dbj|AK231968.1|	white	298.18333	18.052569	0.2918713	0.63372388	29.914
gi|115550607|dbj|AK232437.1|	darkorange	298.138	28.761487	0.9852798	0.00213917	64.382
gnl|UG|Ssc#S29978907	darkred	298.05558	31.375649	0.969818	0.00626585	10.674
gi|115552393|dbj|AK236398.1|	blue	298.02826	186.9441	0.9806223	0.00322866	3.968
gi|109639160|ref|NM_001025222.2|	brown	297.99736	97.816419	0.9775712	0.00401861	253.49
gnl|UG|Ssc#S23763002	yellow	297.91148	63.786412	0.9569239	0.01066258	10.674
gnl|UG|Ssc#S40143363	turquoise	297.83642	174.10048	0.5855299	0.29959484	39.99
gnl|UG|Ssc#S40529248	brown	297.78666	94.184868	0.9649539	0.00783426	464.38
gnl|UG|Ssc#S23756098	purple	297.72008	48.884974	0.9519573	0.01254932	215.67
gi|194036309|ref|XM_001927637.1|	darkorange	297.62965	29.598201	0.9914341	0.00095047	139.95
gnl|UG|Ssc#S40146561	purple	297.59137	51.386125	0.9623951	0.00870435	67.434
gnl|UG|Ssc#S29459150	white	297.54082	28.151245	0.996675	0.00023004	258.17
gnl|UG|Ssc#S6008489	brown	297.50048	103.40982	0.9892898	0.0013284	7.688
gnl|UG|Ssc#S17511815	blue	297.43306	190.753	0.9854182	0.0021091	1.904
gi|45268992|gb|AY550051.1|	white	297.25784	24.658895	0.9688473	0.00656957	38.49
gi|52351736|gb|AY610166.1|	brown	297.13704	86.891416	0.9478005	0.01420377	136.72
gi|47523789|ref|NM_214366.1|	turquoise	296.95932	261.31079	0.6879421	0.19917411	118.878

gi|194036972|ref|XM_001927228.1|	grey60	296.88189	15.809824	0.802947	0.10184413	240.14
gi|194042611|ref|XM_001927098.1|	salmon	296.78511	46.458815	0.9938149	0.00058338	17.662
gnl|UG|Ssc#S40101480	blue	296.70106	188.00418	0.9822236	0.00283751	2.056
gi|47523721|ref|NM_214331.1|	yellow	296.69553	65.80664	0.9713065	0.00580939	134.522
gnl|UG|Ssc#S19541250	salmon	296.62675	44.914598	0.9866687	0.00184405	2.314
gnl|UG|Ssc#S23691718	magenta	296.61202	31.968289	0.9518529	0.01259004	117.428
gnl|UG|Ssc#S23698752	midnightblu	296.56618	32.895431	0.960273	0.00944838	82.914
gnl|UG|Ssc#S34527542	yellow	296.41014	62.976617	0.9610489	0.00917404	17.392
gi|112181313|ref|NM_214438.2|	white	296.35694	20.091333	0.194513	0.75390908	554.688
gnl|UG|Ssc#S34529627	black	296.25666	52.010725	0.9805319	0.00325124	1.722
gnl|UG|Ssc#S23757521	violet	296.19823	15.6526	0.8799413	0.04902803	3.258
gnl|UG|Ssc#S40426021	darkred	296.15034	29.793441	0.9594844	0.00972994	16.598
gnl|UG|Ssc#S40377740	brown	296.07824	86.119427	0.9447925	0.01544191	267.046
gnl|UG|Ssc#S18387146	salmon	295.97001	46.334148	0.9940833	0.00054584	12.33
gnl|UG|Ssc#S34512754	turquoise	295.9535	247.41979	0.6958774	0.19187958	12.358
gi|115554089|dbj|AK233298.1|	darkturquoi	295.93554	25.556511	0.9564096	0.01085325	7.61
gi|150246527|ref|NM_001044575.2|	greenyellow	295.85308	37.746217	0.950339	0.01318547	74.688
gi|194040411|ref|XM_001929558.1|	brown	295.84915	83.416495	0.9350788	0.01966251	185.484
gnl|UG|Ssc#S39932918	white	295.84477	18.969618	0.1221204	0.84489885	9.882
gi|89886172|ref|NM_001039748.1|	brown	295.66045	90.108278	0.9564468	0.01083942	116.398
gnl|UG|Ssc#S23692648	blue	295.53387	186.35291	0.9794779	0.00351823	8.178
gnl|UG|Ssc#S23755550	darkorange	295.37638	29.148558	0.9881798	0.00153992	62.286
gnl|UG|Ssc#S40045135	white	295.36835	19.624624	0.1645738	0.79140795	61.486
gnl|UG|Ssc#S40053273	white	295.19597	27.933521	0.9941561	0.0005358	243.498
gi|47523805|ref|NM_214375.1|	yellow	294.83918	57.613045	0.9223073	0.02569079	585.196
gnl|UG|Ssc#S25135824	brown	294.82649	98.189527	0.9764551	0.00432155	24.832
gnl|UG|Ssc#S6085740	brown	294.82039	95.905304	0.9730412	0.00529201	19.592
gi|115549338|dbj|AK232116.1|	darkorange	294.73518	28.968098	0.9867551	0.00182618	67.136
gnl|UG|Ssc#S39982552	greenyellow	294.55722	42.677465	0.9782406	0.00384045	221.694
gnl|UG|Ssc#S40396824	greenyellow	294.46042	43.797396	0.9868393	0.00180881	41.73
gi|115552527|dbj|AK236532.1|	purple	294.35405	54.49273	0.9748572	0.00476772	81.296
gi|80971505|ref|NM_001037147.1|	turquoise	294.3509	235.30806	0.6537674	0.23143733	3.316
gnl|UG|Ssc#S23697317	green	294.3295	66.797428	0.9210464	0.02631362	35.276
gnl|UG|Ssc#S40094025	blue	294.32131	183.83807	0.9767971	0.00422795	5.776
gnl|UG|Ssc#S23762266	black	294.27724	33.220156	0.8779087	0.05026242	37.548
gnl|UG|Ssc#S18383647	lightyellow	294.27538	31.568839	0.9798513	0.00342286	6.724
gi|52351620|gb|AY610050.1|	blue	294.03783	173.40571	0.9636995	0.00825702	43.464
gi|194036687|ref|XM_001926116.1|	white	293.84054	19.822801	0.2006707	0.74622348	40.982
gnl|UG|Ssc#S19549165	greenyellow	293.78031	37.047645	0.9409949	0.01705245	49.13
gi|194034216|ref|XM_001928595.1|	brown	293.66221	104.7812	0.9924561	0.00078566	119.698
gi|194018721|ref|NM_001129949.1|	brown	293.63692	100.91738	0.9837836	0.0024729	2856.814
gi|47523491|ref|NM_214204.1|	midnightblu	293.58003	30.098361	0.9363064	0.01911102	676.036
gnl|UG|Ssc#S40482312	blue	293.55432	166.54066	0.954567	0.0115454	50.898
gnl|UG|Ssc#S35331200	brown	293.51521	106.9734	0.9974567	0.00015391	54.426
gi|114703735|ref|NM_001048187.1|	brown	293.4182	81.170025	0.92839	0.02275483	20.308
gnl|UG|Ssc#S22277516	lightyellow	292.96934	26.434527	0.9397088	0.01760951	46.472
	midnightblu					

gi|115554973|dbj|AK240172.1|	yellow	292.70224	46.908372	0.8573136	0.06329759	28.844
gi|115552602|dbj|AK232807.1|	brown	292.55585	78.214427	0.9192954	0.02718646	81.268
gi|48374062|ref|NM_001001535.1|	darkgreen	292.36207	20.973404	0.9082401	0.03290361	2885.426
gnl|UG|Ssc#S18549214	darkturquoi	292.28856	29.70696	0.9886972	0.00144004	15.71
gnl|UG|Ssc#S38481364	midnightblu	292.25848	35.812242	0.9779798	0.00390956	19.174
gnl|UG|Ssc#S40232606	brown	292.09719	98.93083	0.9799695	0.00339283	6.968
gi|194037172|ref|XM_001928663.1|	brown	291.93741	85.677149	0.9433185	0.01606087	72.914
gi|115550354|dbj|AK238619.1|	brown	291.8038	99.77102	0.981417	0.00303245	85.364
gi|147899058|ref|NM_001097509.1|	white	291.29736	20.06898	0.1946324	0.75375995	30.696
gi|139530445|gb|EF486522.1|	brown	291.08662	99.539858	0.9801159	0.00335578	452.242
gi|115552944|dbj|AK239538.1|	brown	291.00222	104.2658	0.9910112	0.00102165	42.444
gnl|UG|Ssc#S40500401	darkgreen	290.95895	18.989369	0.830909	0.08131686	34.694
gi|149364039|gb|EF468461.1|	white	290.89797	18.967298	0.1091966	0.86124332	43.588
gi|157427731|ref|NM_001105304.1|	darkgrey	290.8001	16.028141	0.9688903	0.00655601	51.208
gi|194034834|ref|XM_001925793.1|	brown	290.63422	102.52792	0.9880113	0.00157294	81.732
gi|178056859|ref|NM_001123091.1|	greenyellow	290.61839	41.410237	0.9729773	0.00531079	65.478
gi|52351702|gb|AY610132.1|	grey60	290.57154	12.129514	0.5202516	0.36882511	65.828
gnl|UG|Ssc#S40045387	greenyellow	290.16898	39.611499	0.9663885	0.00735978	23.912
gnl|UG|Ssc#S6014656	yellow	289.9825	67.901194	0.9771841	0.00412287	21.684
gnl|UG|Ssc#S18379594	white	289.53492	25.364417	0.9778105	0.00395463	23.822
gnl|UG|Ssc#S31120438	turquoise	289.37792	245.74184	0.678806	0.20766642	26.262
gi|113205497|ref|NM_001044527.1|	purple	289.12431	54.153928	0.9738118	0.00506734	425.972
gnl|UG|Ssc#S40315766	darkturquoi	289.06186	29.152419	0.9872007	0.00173492	25.588
gi|115552519|dbj|AK236524.1|	greenyellow	288.89952	43.588284	0.9844721	0.00231733	66.258
gnl|UG|Ssc#S40077180	darkturquoi	288.88828	28.067917	0.9795876	0.00349013	4.854
gi|194042969|ref|XM_001929585.1|	yellowgreen	288.6675	16.024332	0.9748449	0.00477119	86.81
gnl|UG|Ssc#S23762736	brown	288.64218	93.941871	0.968379	0.00671779	9.36
gi|115549349|dbj|AK232127.1|	greenyellow	288.55313	42.412895	0.9774505	0.00405101	31.774
gnl|UG|Ssc#S31133918	purple	288.36206	56.242789	0.9816878	0.00296653	8.342
gi|52351582|gb|AY610012.1|	magenta	288.29141	32.936188	0.9729562	0.005317	19.596
gi|115547955|dbj|AK234932.1|	brown	288.25703	95.173069	0.9713128	0.0058075	21.492
gi|147902484|ref|NM_001097419.1|	turquoise	288.04805	243.48534	0.6702494	0.21570928	51.11
gnl|UG|Ssc#S23690458	grey60	287.97547	16.056354	0.7373456	0.15506487	15.74
gnl|UG|Ssc#S26716026	black	287.86203	44.730526	0.9480119	0.01411804	92.704
gnl|UG|Ssc#S38479786	turquoise	287.55698	227.58848	0.6406129	0.24420516	94.686
gi|194036805|ref|XM_001928924.1|	darkgreen	287.51482	21.878818	0.9037484	0.03532435	43.702
gnl|UG|Ssc#S18557598	darkgreen	287.48453	21.430239	0.8974511	0.03880918	51.518
gi|115552724|dbj|AK232929.1|	violet	287.42378	16.19736	0.9257626	0.0240089	54.712
gnl|UG|Ssc#S17504469	darkorange	287.15032	29.760218	0.9925719	0.00076766	41.096
gnl|UG|Ssc#S26714423	turquoise	287.14924	257.1805	0.6745057	0.21169795	78.254
gnl|UG|Ssc#S23765987	greenyellow	286.97047	43.133666	0.9817668	0.00294739	71.432
gi|190360656|ref|NM_001128470.1|	brown	286.94262	73.304196	0.9056767	0.03427837	91.048
gnl|UG|Ssc#S18284050	yellow	286.89956	36.639361	0.7785758	0.12083521	17.108
gnl|UG|Ssc#S40148092	darkorange	286.76034	29.21246	0.9890708	0.00136931	37.702
gnl|UG|Ssc#S18267174	darkgrey	286.45943	16.600979	0.97169	0.00569366	64.186
gnl|UG|Ssc#S17510469	lightyellow	286.3926	27.650938	0.9463834	0.01478288	12.82
gnl|UG|Ssc#S39839769	yellow	286.38605	59.152575	0.952692	0.01226392	13.046
gi|115551399|dbj|AK236077.1|	darkturquoi	286.27928	29.159191	0.9866311	0.00185185	33.152

gi|115551308|dbj|AK235986.1|	greenyellow	286.25585	43.122248	0.9809262	0.00315315	21.586
gnl|UG|Ssc#S23757695	midnightblu	286.16399	36.219642	0.980857	0.00317027	59.428
gi|194037039|ref|XM_001926976.1|	white	286.15316	20.591963	0.0825876	0.8949659	41.456
gi|194036118|ref|XM_001927231.1|	pink	286.13134	19.543693	-0.819283	0.08967952	18.536
gi|115548698|dbj|AK231683.1|	white	286.08794	26.730945	0.98366	0.00250119	55.818
gnl|UG|Ssc#S17516920	brown	286.04688	99.673347	0.9814745	0.00301842	13.454
gi|115548072|dbj|AK238056.1|	purple	286.03888	56.28203	0.9820232	0.00288554	32.27
gi|194042350|ref|XM_001924660.1|	turquoise	286.01048	206.43689	0.6206905	0.26388996	35.52
gi|194036621|ref|XM_001927321.1|	yellow	285.99186	54.061605	0.8996869	0.03755996	90.392
gnl|UG|Ssc#S18558242	purple	285.9067	55.618544	0.9793474	0.00355178	205.736
gnl|UG|Ssc#S22278253	yellowgreen	285.76421	16.338015	0.9790318	0.00363333	31.874
gnl|UG|Ssc#S6666060	magenta	285.72869	14.25601	-0.228166	0.7120309	3.466
gi|194044943|ref|XM_001926010.1|	darkgreen	285.65619	21.696922	0.8835994	0.04683116	91.834
gnl|UG|Ssc#S29966064	darkred	285.48311	31.695193	0.9730263	0.00529638	20.364
gi|71834281|ref|NM_214197.2|	yellow	285.4655	57.93549	0.9236201	0.02504743	154.696
gnl|UG|Ssc#S31101544	purple	285.45163	56.051704	0.9811162	0.00310624	55.494
gnl|UG|Ssc#S35323577	blue	285.3207	172.97088	0.9621868	0.00877648	8.93
gi|194041762|ref|XM_001927529.1|	turquoise	285.23109	237.14832	0.6729051	0.21320396	56.294
gi|47522785|ref|NM_213980.1|	magenta	285.1704	31.00602	0.9167659	0.02846346	77.412
gnl|UG|Ssc#S40482232	salmon	285.12682	46.44198	0.9939636	0.00056247	73.132
gnl|UG|Ssc#S17516311	darkorange	285.03763	29.686626	0.9919354	0.00086832	132.112
gnl|UG|Ssc#S23775247	darkorange	284.98927	28.469977	0.9839083	0.00244448	4889.31
gi|2988384|gb|AF033855.1|AF033855	yellow	284.9026	55.350494	0.8993555	0.03774433	20.648
gi|115551350|dbj|AK236028.1|	darkgrey	284.85723	14.850823	0.9411224	0.01699755	18.354
gnl|UG|Ssc#S6072986	white	284.77746	19.913508	0.1930477	0.75573944	25.71
gnl|UG|Ssc#S39858087	greenyellow	284.75779	44.408831	0.9917981	0.00089057	61.986
gi|55741485|ref|NM_213855.1|	purple	284.48149	55.829374	0.9802832	0.0033136	469.446
gi|115555012|dbj|AK240211.1|	turquoise	284.31836	223.19972	0.6619745	0.22356743	186.958
gnl|UG|Ssc#S17503882	grey60	284.28458	14.099355	0.7482334	0.14578366	31.1
gnl|UG|Ssc#S22279857	blue	284.15577	157.19571	0.9370524	0.01877843	0.308
gnl|UG|Ssc#S18558128	grey60	283.98694	16.021883	0.9366109	0.01897503	8.784
gi|194036406|ref|XM_001927918.1|	darkorange	283.69289	30.748971	0.9994981	1.35E-05	145.16
gnl|UG|Ssc#S35325791	brown	283.66873	90.762509	0.9588895	0.00994414	25.82
gnl|UG|Ssc#S34528527	brown	283.60352	99.471985	0.9813043	0.00306002	66.832
gi|115553524|dbj|AK236727.1|	brown	283.46924	99.46165	0.9803356	0.00330044	42.8
gnl|UG|Ssc#S18282381	darkgreen	283.46564	21.655228	0.8865272	0.04509598	55.08
gi|194043243|ref|XM_001924074.1|	blue	283.41615	162.59563	0.9464934	0.01473764	52.614
gnl|UG|Ssc#S16763939	black	283.4159	49.317163	0.9680389	0.00682611	29.286
gnl|UG|Ssc#S19542276	brown	283.21596	80.343507	0.9283976	0.02275121	13.868
gi|1841944|emb|X95846.1|	white	283.11927	17.813357	0.1965429	0.75137436	16.594
gnl|UG|Ssc#S23757556	turquoise	282.97484	215.62257	0.6364815	0.24825337	11.588
gi|47523403|ref|NM_214157.1|	salmon	282.86312	46.120025	0.9926726	0.00075211	23.444
gi|52351873|gb|AY610291.1|	darkorange	282.78057	29.786377	0.992829	0.00072818	123.95
gnl|UG|Ssc#S19540428	brown	282.77784	87.733232	0.9506959	0.01304428	8.722
gnl|UG|Ssc#S23758125	turquoise	282.72483	189.70224	0.6068105	0.27784215	244.998
gi|194041867|ref|XM_001925382.1|	darkred	282.57845	27.171055	0.9410358	0.01703481	14.39
gi|115548002|dbj|AK237986.1|	yellow	282.49849	66.355504	0.9666657	0.00726922	227.186
gi|47522939|ref|NM_214062.1|	yellow	282.27908	66.872393	0.9652477	0.00773629	118.008

gi|115554857|dbj|AK233669.1|	greenyellow	282.18839	41.55539	0.9746513	0.00482624	111.094
gi|115555162|dbj|AK233777.1|	blue	281.96414	142.50256	0.9130711	0.0303623	66.58
gnl|UG|Ssc#S29989732	white	281.95949	20.517974	0.0869017	0.8894928	90.918
gnl|UG|Ssc#S19541992	blue	281.95062	181.176	0.9736542	0.00511302	21.496
gi|147905343|ref|NM_001097438.1|	darkgreen	281.91354	22.172674	0.8960653	0.03959001	21.484
gnl|UG|Ssc#S23757528	blue	281.78793	160.19861	0.9443969	0.01560727	13.49
gnl|UG|Ssc#S18555004	violet	281.70437	15.897344	0.913844	0.02996183	65.97
gi|115549949|dbj|AK232184.1|	violet	281.59166	16.798678	0.9247263	0.02450945	122.752
gi|118403777|ref|NM_001078685.1|	greenyellow	281.44114	41.149203	0.9731902	0.00524833	20.44
gi|115546452|dbj|AK230971.1|	white	281.42033	24.984575	0.967096	0.00712939	60.298
gi|115547797|dbj|AK234773.1|	darkturquoi	281.34218	28.604009	0.980702	0.00320879	25.942
gnl|UG|Ssc#S26714441	yellowgreen	281.33247	16.572068	0.9821474	0.00285574	98.454
gnl|UG|Ssc#S40272618	greenyellow	281.32717	38.279376	0.9582132	0.01018947	66.098
gi|55741489|ref|NM_214136.1|	purple	281.24854	54.304712	0.9744833	0.00487417	55.276
gnl|UG|Ssc#S40214866	magenta	280.93084	32.02135	0.9407014	0.01717904	18.74
gi|115554409|dbj|AK233419.1|	purple	280.88626	59.405906	0.9935257	0.00062475	283.672
gnl|UG|Ssc#S18357620	greenyellow	280.71275	43.053809	0.9805152	0.00325539	28.918
gnl|UG|Ssc#S35327002	purple	280.68624	55.715411	0.9800678	0.00336795	17.584
gi|194042528|ref|XM_001927272.1|	purple	280.67965	58.703597	0.9910544	0.0010143	694.498
gnl|UG|Ssc#S40584531	yellow	280.67594	49.92202	0.8707359	0.05469475	11.396
gnl|UG|Ssc#S18547766	yellowgreen	280.66991	16.82482	0.9850175	0.0021965	27.066
gnl|UG|Ssc#S18272408	purple	280.53445	55.584846	0.9792573	0.00357499	6.554
gi|115547142|dbj|AK231197.1|	blue	280.42454	161.39847	0.9443897	0.01561026	274.442
gnl|UG|Ssc#S23772512	lightyellow	280.38984	24.415251	0.9144885	0.02962925	13.078
gi|47523483|ref|NM_214199.1|	brown	280.29865	96.950146	0.9753052	0.00464116	60.092
gi|217416475|ref|NM_001142669.1|	brown	280.20075	74.414518	0.9083303	0.03285555	14.822
gnl|UG|Ssc#S35336579	darkorange	280.19227	30.580565	0.9984303	7.46E-05	25.892
gi|194038910|ref|XM_001926121.1|	darkturquoi	280.13134	21.337268	0.915477	0.0291214	6.552
gi|153791354|ref|NM_001099940.1|	black	279.97777	51.283989	0.9777576	0.00396873	32.462
gi|115545765|dbj|AK234198.1|	blue	279.88853	165.28183	0.9505921	0.01308528	46.678
gi|194036140|ref|XM_001929572.1|	yellow	279.86693	55.031271	0.895997	0.0396286	8.046
gi|194042587|ref|XM_001928493.1|	greenyellow	279.60908	38.887529	0.955418	0.01122398	30.744
gnl|UG|Ssc#S23689840	turquoise	279.4419	259.08867	0.6760854	0.21021444	43.38
gi|178056487|ref|NM_001123204.1|	darkorange	279.19173	29.555378	0.9910127	0.00102139	46.534
gi|47523769|ref|NM_214356.1|	white	279.09878	20.275753	0.0032238	0.99589531	51.616
gi|115546190|dbj|AK240428.1|	darkturquoi	279.03962	23.642612	0.9319041	0.02111197	9.72
gnl|UG|Ssc#S26719940	black	279.02177	48.480734	0.964076	0.00812938	4.962
gi|115552220|dbj|AK230529.1|	blue	279.00307	155.60033	0.9359748	0.01925948	51.286
gi|73853889|ref|NM_001032359.1|	yellowgreen	278.95819	16.68118	0.9829415	0.00266765	304.17
gi|47523689|ref|NM_214316.1|	darkturquoi	278.82066	28.614482	0.9806872	0.00321247	31.446
gi|47523681|ref|NM_214312.1|	brown	278.79905	70.0294	0.8929613	0.04135672	51.952
gnl|UG|Ssc#S17509934	brown	278.64209	86.014899	0.9446903	0.0154846	7.636
gnl|UG|Ssc#S35173683	magenta	278.58833	32.276067	0.9575202	0.01044291	21.694
gnl|UG|Ssc#S34530890	purple	278.5153	59.114684	0.9925296	0.00077422	15.69
gnl|UG|Ssc#S17501425	darkturquoi	278.51039	29.261195	0.9880577	0.00156382	11.992
gnl|UG|Ssc#S18354245	blue	278.25467	173.30465	0.9634992	0.0083252	5.816
gnl|UG|Ssc#S17525865	brown	277.99147	81.023389	0.9276213	0.02311949	73.23
gi|115545741|dbj|AK234174.1|	turquoise	277.9259	178.59613	0.592857	0.29205697	26.714

gnl|UG|Ssc#S6085466	darkorange	277.87623	27.329625	0.9747639	0.0047942	21.918
gi|194474045|ref|NM_001130534.1|	brown	277.82527	100.88569	0.9833337	0.00257634	24.342
gnl|UG|Ssc#S23698009	salmon	277.82331	45.918107	0.9906578	0.00108243	19.712
gi|115549856|dbj|AK238516.1|	purple	277.50802	53.377527	0.971128	0.00586354	39.77
gi|194044114|ref|XM_001924459.1|	brown	277.41401	100.87129	0.9829819	0.00265822	118.184
gnl|UG|Ssc#S6085531	brown	277.16956	88.040226	0.9535976	0.0119151	35.35
gnl|UG|Ssc#S35723730	grey60	277.1662	16.563383	0.9306675	0.02168555	55.59
gnl|UG|Ssc#S26735265	turquoise	277.02217	245.02307	0.6671998	0.21859627	21.628
gnl|UG|Ssc#S18558304	turquoise	277.00853	225.3237	0.6521551	0.23299217	10.502
gi|178056489|ref|NM_001123144.1|	pink	276.86464	20.098705	-0.818963	0.08991382	61.576
gi|194038369|ref|XM_001929075.1|	midnightblu	276.72102	33.92311	0.9636409	0.00827697	197.158
gnl|UG|Ssc#S18546548	salmon	276.70848	46.785934	0.9959718	0.00030672	7.452
gi|115547364|dbj|AK234737.1|	magenta	276.69465	32.183366	0.9697311	0.00629286	28.02
gnl|UG|Ssc#S40494112	yellow	276.47913	64.377252	0.9518974	0.0125727	14.654
gnl|UG|Ssc#S18301666	yellow	276.41084	53.849611	0.9190825	0.02729323	29.286
gnl|UG|Ssc#S40376380	blue	276.26305	122.78729	0.8779199	0.05025559	6.084
gi|115552989|dbj|AK239575.1|	turquoise	276.08034	239.45202	0.6546984	0.23054077	27.914
gnl|UG|Ssc#S31104316	black	275.93683	48.927346	0.9669051	0.0071913	6.656
gi|194035566|ref|XM_001925085.1|	yellow	275.86646	54.225961	0.8943222	0.04057915	14.904
gnl|UG|Ssc#S17511285	greenyellow	275.84535	43.808046	0.9913637	0.00096219	316.952
gi|115546313|dbj|AK240551.1|	midnightblu	275.81037	35.89878	0.9800172	0.00338076	43.152
gi|115553094|dbj|AK239680.1|	yellow	275.71976	68.24815	0.9761529	0.00440481	36.784
gnl|UG|Ssc#S18358324	turquoise	275.66026	224.8228	0.6363882	0.24834494	14.06
gi|113205859|ref|NM_001044605.1|	white	275.37082	20.085984	-0.017271	0.97801037	30.194
gnl|UG|Ssc#S18556801	darkturquoi	275.32154	20.343131	0.9046491	0.03483453	8.522
gnl|UG|Ssc#S19540415	purple	275.32153	56.17408	0.9819432	0.00290479	12.702
gnl|UG|Ssc#S23758705	yellow	275.29946	58.941013	0.931014	0.02152433	163.23
gi|52351354|gb|AY609784.1|	turquoise	275.21648	190.59513	0.5979169	0.28688092	23.84
gnl|UG|Ssc#S40174573	midnightblu	275.10934	37.322642	0.9890982	0.00136418	3.15
gnl|UG|Ssc#S18359209	magenta	274.83828	15.305705	-0.124435	0.84197452	30.386
gnl|UG|Ssc#S40204438	lightyellow	274.61284	23.041037	0.898569	0.03818297	13.65
gi|194034244|ref|XM_001925555.1|	yellow	274.589	61.465916	0.9521231	0.01248474	42.694
gnl|UG|Ssc#S23699836	darkorange	274.52384	30.06409	0.9947405	0.00045751	28.398
gi|115548500|dbj|AK235117.1|	midnightblu	274.42433	34.869913	0.9755364	0.00457629	130.348
gi|115552638|dbj|AK232843.1|	purple	274.42042	57.652738	0.987343	0.0017061	27.812
gnl|UG|Ssc#S19541498	darkred	274.27427	27.199722	0.9394488	0.01772284	23.344
gi|115552127|dbj|AK239320.1|	lightyellow	274.20477	26.944437	0.9427553	0.01629943	3.2
gnl|UG|Ssc#S18381258	magenta	274.03114	33.385044	0.9839324	0.002439	62.466
gnl|UG|Ssc#S18546477	blue	273.93286	144.75246	0.9218822	0.02590021	87.928
gnl|UG|Ssc#S40094285	blue	273.59755	156.03512	0.9394795	0.01770946	20.838
gi|115545930|dbj|AK237371.1|	salmon	273.49592	46.482156	0.9936629	0.000605	54.336
gi|194036414|ref|XM_001927979.1|	white	273.09451	27.054019	0.9815833	0.00299191	31.42
gnl|UG|Ssc#S17524473	purple	273.02407	55.747813	0.9800526	0.00337178	39.648
gnl|UG|Ssc#S17524578	yellow	273.02284	51.588288	0.9177072	0.02798606	13.67
gnl|UG|Ssc#S18553763	blue	272.88602	167.52116	0.9547942	0.01145928	3.462
gi|115553226|dbj|AK233024.1|	green	272.73801	54.484319	0.8811953	0.04827138	66.804
gnl|UG|Ssc#S17511723	brown	272.69144	101.42976	0.9856165	0.00206629	13.692
gi|115551465|dbj|AK236143.1|	grey60	272.69118	15.6446	0.6703356	0.21562786	38.082

gnl|UG|Ssc#S40213312	darkorange	272.57007	28.034826	0.980161	0.00334438	101.602
gi|147900501|ref|NM_001097506.1|	darkorange	272.54127	26.698089	0.9698146	0.00626689	19.088
gi|194042141|ref|XM_001929000.1|	white	272.53769	20.493918	0.0619638	0.92115574	25.904
gi|194035178|ref|XM_001925137.1|	yellow	272.50238	65.222652	0.9630445	0.00848067	20.332
gnl|UG|Ssc#S22315401	magenta	272.04018	30.978657	0.9339822	0.02015937	21.902
gnl|UG|Ssc#S38481210	yellow	272.00198	67.45397	0.9872022	0.00173461	6.49
gi|115550039|dbj|AK232274.1|	pink	271.63444	19.798221	-0.843897	0.07227862	62.976
gi|38455773|gb|AY349420.1|	violet	271.60611	11.618418	0.8057376	0.09973295	11.304
gnl|UG|Ssc#S18284292	black	271.52048	43.618037	0.9403567	0.01732817	11.488
gi|194039390|ref|XM_001929570.1|	blue	271.38829	162.38026	0.9487752	0.01380984	651.3
gi|125630297|ref|NM_001005152.2|	greenyellow	271.26061	37.05295	0.9436436	0.01592368	78.956
gi|62082661|gb|AY870324.1|	white	271.20331	20.097203	-0.025678	0.96730874	53.694
gi|194039687|ref|XM_001928955.1|	blue	271.10757	160.32047	0.944717	0.01547343	116.384
gi|194044682|ref|XM_001928813.1|	blue	271.06938	147.42232	0.9224272	0.02563181	22.802
gi|146198449|dbj|AB292846.1|	turquoise	270.89045	194.343	0.6195194	0.26505968	6.186
gnl|UG|Ssc#S34532931	salmon	270.84359	43.483746	0.9777394	0.00397359	5.554
gnl|UG|Ssc#S27601831	turquoise	270.54739	237.28953	0.6865559	0.20045616	17.892
gnl|UG|Ssc#S40588989	purple	270.52905	53.801455	0.9728546	0.00534689	23.394
gnl|UG|Ssc#S19546269	purple	270.20472	54.672414	0.9763965	0.00433766	20.54
gnl|UG|Ssc#S29456965	darkorange	270.12866	29.199129	0.9892687	0.00133234	12.698
gi|148230690|ref|NM_001097489.1|	lightyellow	270.04816	21.239545	0.8789918	0.04960344	7.344
gi|194043957|ref|XM_001925803.1|	brown	269.91795	80.185538	0.9260034	0.02389306	1230.364
gi|115550007|dbj|AK232242.1|	brown	269.62718	72.757439	0.9051609	0.03455717	115.78
gi|194034713|ref|XM_001925291.1|	blue	269.53929	123.32581	0.8809748	0.04840417	41.894
gi|115552041|dbj|AK239234.1|	blue	269.50948	163.42455	0.9492104	0.01363512	12.232
gnl|UG|Ssc#S31134214	turquoise	269.26206	240.65264	0.6848624	0.20202566	7.482
gnl|UG|Ssc#S18554829	brown	269.19839	76.767024	0.9177041	0.02798762	15.724
gnl|UG|Ssc#S18551055	blue	269.18131	170.3647	0.9589265	0.00993076	5.776
gnl|UG|Ssc#S16517726	brown	269.03374	81.117867	0.9324003	0.02088321	40.014
gi|47523039|ref|NM_214116.1|	brown	268.86759	90.157182	0.9576347	0.01040088	90.334
gnl|UG|Ssc#S26647414	yellowgreer	268.82416	17.353082	0.9919825	0.00086073	6.134
gnl|UG|Ssc#S40433705	midnightblu	268.74165	37.782536	0.9916364	0.00091701	3.814
gi|115551408|dbj|AK236086.1|	darkred	268.66948	28.638062	0.9512533	0.01282479	36.934
gi|194038735|ref|XM_001929188.1|	midnightblu	268.43412	35.574276	0.9780344	0.00389506	37.396
gi|194035296|ref|XM_001927594.1|	midnightblu	268.4127	37.717387	0.9907659	0.00106372	111.998
gi|194034443|ref|XM_001928249.1|	midnightblu	268.32939	33.525884	0.9597192	0.00964581	36.58
gnl|UG|Ssc#S26394012	turquoise	268.21285	181.39916	0.5992298	0.28554175	6.868
gnl|UG|Ssc#S19540505	violet	268.17035	15.877291	0.9117808	0.03103463	44.552
gnl|UG|Ssc#S18551577	salmon	268.14119	19.208728	0.7998623	0.10419327	45.57
gi|115312275|ref|NM_001048232.1|	brown	268.1307	95.000322	0.9680786	0.00681344	10.906
gi|115551885|dbj|AK236292.1|	darkgreen	268.11886	22.87156	0.9136193	0.03007811	20.99
gnl|UG|Ssc#S35326006	darkgreen	267.99013	19.579529	0.7922505	0.11005876	114.164
gi|194036762|ref|XM_001925928.1|	green	267.95203	47.526277	0.8521148	0.06673362	25.078
gi|115552402|dbj|AK236407.1|	turquoise	267.7981	225.40412	0.6762366	0.21007263	8.818
gnl|UG|Ssc#S16514640	brown	267.74779	99.566837	0.9812732	0.00306764	32.42
gnl|UG|Ssc#S17513113	blue	267.50024	173.04134	0.9636801	0.00826361	33.854
	yellowgreer					

gnl|UG|Ssc#S17525035	pink	267.07496	19.732357	-0.831897	0.08061784	43.14
gnl|UG|Ssc#S31119453	yellow	266.98891	61.728515	0.9666939	0.00726003	19.176
gnl|UG|Ssc#S18547573	brown	266.82231	80.103207	0.9263831	0.02371077	196.18
gnl|UG|Ssc#S26391783	greenyellow	266.66841	36.778702	0.9482122	0.01403692	19.562
gnl|UG|Ssc#S17511386	yellowgreen	266.63731	17.476911	0.994607	0.00047503	98.316
gi|47522983|ref|NM_214086.1|	white	266.58615	19.597233	-0.054986	0.93002534	66.432
gi|194044465|ref|XM_001928874.1|	purple	266.51752	58.073422	0.9890083	0.00138107	15.546
gi|83616153|gb|DQ241739.1|	grey60	266.47804	15.246893	0.7181475	0.17182836	9.014
gi|115555129|dbj|AK240329.1|	blue	266.47513	166.12735	0.9542184	0.0116779	132.538
gi|194037329|ref|XM_001927218.1|	salmon	266.43039	46.89398	0.9959727	0.00030661	6.148
gi|115546753|dbj|AK237599.1|	midnightblu	266.38946	35.335547	0.9770015	0.00417235	60.28
gi|194035848|ref|XM_001928966.1|	purple	266.14841	59.091774	0.9925525	0.00077066	86.446
gi|148234109|ref|NM_001093735.1|	darkgrey	265.9824	15.876491	0.9782782	0.00383052	25.394
gnl|UG|Ssc#S39900214	brown	265.56521	82.558797	0.9356623	0.01939976	9.608
gnl|UG|Ssc#S18552012	greenyellow	265.49365	39.034005	0.9607477	0.00928023	38.242
gnl|UG|Ssc#S39872348	brown	265.44274	74.253216	0.9086385	0.03269157	29.118
gi|194044132|ref|XM_001927798.1|	skyblue	265.44024	21.07904	0.9521752	0.01246444	98.936
gi|113205907|ref|NM_001044616.1|	purple	265.27491	60.46131	0.9974441	0.00015506	34.992
gnl|UG|Ssc#S19540060	purple	265.24245	61.044118	0.9994816	1.42E-05	11.35
gnl|UG|Ssc#S22284504	brown	265.09926	99.543404	0.9813933	0.00303824	13.908
gnl|UG|Ssc#S31986306	salmon	264.99291	42.054618	0.9707873	0.00596732	9.052
gi|52351697|gb|AY610127.1|	brown	264.87282	76.145453	0.9155783	0.02906951	96.17
gi|178056883|ref|NM_001123198.1|	grey60	264.84055	16.516072	0.9472364	0.01443337	32.538
gnl|UG|Ssc#S6063275	yellow	264.82255	64.958401	0.949333	0.01358606	34.994
gi|115554132|dbj|AK233342.1|	purple	264.77325	56.497492	0.9832186	0.00260305	6.766
gnl|UG|Ssc#S27600966	purple	264.66895	60.639325	0.9980885	0.00010029	18.716
gi|47523533|ref|NM_214229.1|	salmon	264.61466	44.000277	0.9814405	0.00302671	5.162
gi|115552791|dbj|AK239385.1|	blue	264.48854	149.11736	0.9308249	0.02161226	23.766
gi|47523067|ref|NM_214132.1|	darkorange	264.48695	28.85163	0.9866174	0.00185468	10.434
gnl|UG|Ssc#S18290449	darkturquoi	264.03112	27.470295	0.9745561	0.00485338	195.59
gi|115545973|dbj|AK237414.1|	purple	263.67319	61.091068	0.9996566	7.64E-06	66.21
gnl|UG|Ssc#S40219335	brown	263.48101	93.217749	0.9658028	0.0075523	14.194
gnl|UG|Ssc#S23771394	yellow	263.45009	61.93984	0.9321679	0.02099024	9.77
gnl|UG|Ssc#S40271107	purple	263.35143	58.721463	0.9912396	0.00098299	11.784
gnl|UG|Ssc#S17513979	darkgreen	263.08928	22.096099	0.8530205	0.06613097	42.468
gi|115552281|dbj|AK230590.1|	brown	262.90603	80.32298	0.9271032	0.02336629	100.872
gnl|UG|Ssc#S18278819	black	262.78175	49.26374	0.9686396	0.00663519	4.698
gnl|UG|Ssc#S18355852	turquoise	262.65183	232.81185	0.6467166	0.23825758	18.82
gi|194039697|ref|XM_001929172.1|	black	262.51198	37.420571	0.8985072	0.03821748	88.968
gi|115548969|dbj|AK235372.1|	blue	262.49067	153.99621	0.9341353	0.02008977	11.888
gnl|UG|Ssc#S34509960	darkorange	262.44573	28.068614	0.980544	0.00324821	24.158
gnl|UG|Ssc#S40110548	violet	262.44094	16.55146	0.904603	0.03485956	11.05
gnl|UG|Ssc#S23696740	blue	262.33835	163.51154	0.9504733	0.01313226	30.234
gnl|UG|Ssc#S6077016	darkorange	262.22796	28.127555	0.981575	0.00299394	13.16
gnl|UG|Ssc#S26720320	grey60	262.21938	16.02304	0.787978	0.11339315	5.996
gi|146741279|dbj|AB271920.1|	darkgreen	262.2035	21.67972	0.8499872	0.06815603	34.416
gnl|UG|Ssc#S6073264	salmon	261.95464	42.993045	0.976715	0.00425036	27.264
gnl|UG|Ssc#S18382627	turquoise	261.82343	174.51427	0.5850884	0.30005066	10.15

gi|194041357|ref|XM_001926917.1|	purple	261.81917	57.559077	0.9872302	0.00172892	106.948
gnl|UG|Ssc#S35326767	yellow	261.70408	69.385115	0.9869667	0.00178264	27.338
gnl|UG|Ssc#S39768843	midnightblu	261.66521	38.469292	0.9968677	0.00021035	4.918
gi|115548575|dbj|AK231559.1|	green	261.61413	40.359471	0.8176759	0.09085544	12.028
gnl|UG|Ssc#S6057912	salmon	261.47938	44.3853	0.9845743	0.00229452	8.69
gi|115550272|dbj|AK235751.1|	darkorange	261.47003	29.847643	0.9934794	0.00063145	47.9
gi|194036390|ref|XM_001929343.1|	greenyellow	261.37652	38.892863	0.9625375	0.00865514	57.822
gnl|UG|Ssc#S17500019	yellow	261.37141	33.39488	0.7436388	0.14967986	10.06
gi|115545598|dbj|AK230713.1|	yellow	261.21471	54.659015	0.9031734	0.03563818	26.69
gi|115546507|dbj|AK231026.1|	blue	261.19106	167.7916	0.9565774	0.01079093	2.072
gi|115550060|dbj|AK232295.1|	magenta	261.08786	13.389656	-0.292118	0.63342294	53.466
gi|194043337|ref|XM_001926296.1|	midnightblu	260.99447	36.17683	0.9839341	0.00243862	6.068
gi|115546331|dbj|AK230850.1|	brown	260.86155	90.636492	0.9588211	0.00996886	83.454
gnl|UG|Ssc#S19539315	brown	260.68979	84.281741	0.9429772	0.0162053	14.826
gnl|UG|Ssc#S46917266	brown	260.51914	82.437683	0.9380432	0.01833958	20.642
gi|115554725|dbj|AK237136.1|	lightyellow	260.19794	26.064932	0.9366354	0.01896412	23.748
gi|115552177|dbj|AK230486.1|	greenyellow	259.8026	37.985125	0.9475009	0.01432556	993.234
gi|115554566|dbj|AK236976.1|	purple	259.76948	60.83209	0.9987582	5.25E-05	22.906
gi|147900046|ref|NM_001097474.1|	brown	259.70066	87.350598	0.9515242	0.01271855	150.368
gnl|UG|Ssc#S18549395	greenyellow	259.44111	40.75458	0.9694555	0.00637871	18.834
gnl|UG|Ssc#S6082550	salmon	259.31213	41.063036	0.9658651	0.00753175	20.934
gnl|UG|Ssc#S40106937	salmon	259.09389	45.273035	0.9881252	0.0015506	6.98
gi|194038223|ref|XM_001926312.1|	darkgreen	259.09035	17.598721	0.7552606	0.13988339	22.836
gnl|UG|Ssc#S23775145	yellowgreer	258.88003	16.537124	0.9808968	0.00316043	29.508
gnl|UG|Ssc#S18358663	brown	258.86582	66.945507	0.8860327	0.04538761	56.29
gi|47522897|ref|NM_214039.1|	lightyellow	258.86529	20.812611	0.8737786	0.05280011	14.42
gnl|UG|Ssc#S40007606	yellow	258.69045	35.228692	0.7561447	0.13914617	13.312
gnl|UG|Ssc#S16513635	black	258.6506	48.373195	0.9642157	0.00808216	4.682
gnl|UG|Ssc#S19546106	salmon	258.4944	37.841466	0.9496805	0.01344723	210.746
gi|194044987|ref|XM_001927848.1|	yellowgreer	258.44053	16.883742	0.9867975	0.00181742	39.472
gnl|UG|Ssc#S17525713	salmon	258.42042	45.914682	0.9912452	0.00098206	11.668
gi|115553014|dbj|AK239600.1|	purple	258.38101	56.771176	0.98426	0.00236491	19.254
gnl|UG|Ssc#S40215438	white	258.31276	24.285459	0.9697593	0.00628407	8.29
gnl|UG|Ssc#S29978365	brown	258.29809	75.555779	0.9184719	0.02760014	28.712
gnl|UG|Ssc#S18358217	salmon	258.26546	45.461666	0.9886864	0.0014421	11.538
gnl|UG|Ssc#S40500952	darkorange	257.98043	25.901908	0.9632694	0.00840367	84.418
gnl|UG|Ssc#S40323161	blue	257.63076	162.09611	0.9486658	0.01385386	2.65
gi|115546586|dbj|AK234424.1|	greenyellow	257.58675	42.404615	0.9830836	0.00263445	52.43
gi|194037379|ref|XM_001925252.1|	black	257.36407	48.768204	0.9674276	0.00702224	56.718
gnl|UG|Ssc#S39967671	lightcyan	257.19501	31.359143	0.9406913	0.01718342	18.052
gi|115547849|dbj|AK234825.1|	blue	257.03735	143.56387	0.9182924	0.02769057	42.812
gi|52351201|gb|AY609606.1|	brown	256.99576	72.039257	0.9051381	0.03456951	14.52
gi|218082010|ref|NM_001142666.1|	yellow	256.83355	52.818186	0.9229089	0.02539531	28.792
gnl|UG|Ssc#S40265049	blue	256.80043	142.8111	0.9203724	0.0266485	1.95
gnl|UG|Ssc#S31120797	blue	256.76481	158.16411	0.9420713	0.01659068	8.336
gi|115547758|dbj|AK231416.1|	turquoise	256.5298	196.99024	0.6197904	0.26478895	21.152
gnl|UG|Ssc#S26719435	brown	256.47954	94.011894	0.9671994	0.00709592	149.556
gnl|UG|Ssc#S41575462	grey60	256.07995	10.986621	0.5080793	0.38211767	64.71

gi|194035627|ref|XM_001924280.1|	yellowgreer	256.04896	17.576909	0.9952946	0.00038719	43.336
gi|47522629|ref|NM_213928.1|	purple	255.89962	54.177065	0.9749526	0.00474067	34.844
gnl|UG|Ssc#S18551209	greenyellow	255.83873	35.972381	0.9412072	0.01696103	118.936
gnl|UG|Ssc#S40037863	purple	255.49954	54.860029	0.9769808	0.00417797	25.862
gi|194038037|ref|XM_001929120.1|	greenyellow	255.49732	36.410026	0.9440937	0.01573437	53.234
gnl|UG|Ssc#S18548354	magenta	255.34145	32.499648	0.983204	0.00260643	12.002
gi|115547886|dbj|AK234862.1|	white	255.31473	19.012657	-0.054025	0.93124665	19.274
gnl|UG|Ssc#S6058133	brown	255.31109	84.254664	0.9387785	0.01801609	6.122
gi|194039264|ref|XM_001927148.1|	white	255.26135	19.096486	-0.068135	0.91331527	17.518
gnl|UG|Ssc#S18267480	black	254.99816	32.926614	0.8709971	0.05453129	10.082
gi|194038313|ref|XM_001925341.1|	white	254.77158	19.529487	-0.042991	0.94527885	22.672
gnl|UG|Ssc#S18551845	purple	254.7617	60.647069	0.9981554	9.51E-05	18.634
gnl|UG|Ssc#S23696659	salmon	254.59811	45.318997	0.988159	0.00154398	27.298
gi|194035722|ref|XM_001926997.1|	blue	254.48902	142.43184	0.9161316	0.02878663	68.682
gi|194033594|ref|XM_001927795.1|	salmon	254.40835	42.216276	0.9708216	0.00595686	95.586
gi|178056622|ref|NM_001123213.1|	darkgrey	254.39471	15.241757	0.9941603	0.00053523	71.506
gi|52351176|gb|AY609581.1|	yellow	254.3139	65.216028	0.970971	0.00591129	28.81
gi|115555639|dbj|AK237272.1|	purple	254.16156	52.849035	0.9690471	0.00650668	95.868
gi|115553719|dbj|AK239906.1|	white	254.02733	19.617716	0.0402393	0.9487796	65.184
gnl|UG|Ssc#S23755435	yellow	253.93179	64.662528	0.9659466	0.00750488	391.902
gi|57527981|ref|NM_001009576.1|	yellow	253.34849	64.271928	0.9751713	0.00467887	51.892
gi|115553060|dbj|AK239646.1|	purple	253.24867	54.881603	0.9774182	0.00405972	64.674
gnl|UG|Ssc#S26732866	black	253.1739	40.388268	0.9199247	0.02687173	13.8
gi|113205825|ref|NM_001044594.1|	purple	253.12759	58.325082	0.9898258	0.00123004	33.38
gi|115548176|dbj|AK234948.1|	pink	253.03255	21.131573	-0.838402	0.07606253	305.126
gi|194037755|ref|XM_001925059.1|	magenta	253.01114	29.733743	0.9271011	0.02336731	14.016
gnl|UG|Ssc#S16764576	darkorange	252.77204	26.762499	0.9702613	0.00612871	6.636
gnl|UG|Ssc#S40146833	greenyellow	252.72015	39.356647	0.9553737	0.01124065	27.942
gi|194036252|ref|XM_001929660.1|	purple	252.64062	55.105194	0.9781149	0.00387369	46.382
gi|77681320|dbj|AB236885.1|	brown	252.63376	80.689234	0.931935	0.02109771	20.618
gnl|UG|Ssc#S17515082	darkorange	252.25414	27.67178	0.9773575	0.00407606	1.808
gnl|UG|Ssc#S6055548	black	252.18505	46.610199	0.9557252	0.01110872	25.564
gi|115549416|dbj|AK235438.1|	midnightblu	252.11601	34.724349	0.97268	0.00539844	26.508
gi|47523715|ref|NM_214327.1|	grey60	251.96233	16.63106	0.9610407	0.00917691	11.418
gnl|UG|Ssc#S25135543	brown	251.90426	86.817329	0.9491636	0.0136539	4.204
gnl|UG|Ssc#S40577963	blue	251.90241	158.62255	0.9434205	0.01601775	12.264
gnl|UG|Ssc#S19539468	black	251.79439	27.034824	0.8253089	0.08531379	45.754
gnl|UG|Ssc#S18546455	yellow	251.7528	62.82189	0.9566223	0.01077425	79.162
gnl|UG|Ssc#S29979252	yellow	251.67653	72.095919	0.9901419	0.00117323	11.038
gi|115546849|dbj|AK237695.1|	brown	251.23314	85.80157	0.9479832	0.01412966	22.796
gi|194044879|ref|XM_001925452.1|	brown	251.18813	93.780591	0.9669236	0.00718531	8.118
gnl|UG|Ssc#S19538970	grey60	251.15197	15.007174	0.6363948	0.2483385	212.876
gnl|UG|Ssc#S23770014	midnightblu	250.72916	38.406207	0.9959011	0.00031482	28.822
gnl|UG|Ssc#S19541354	purple	250.6966	56.764014	0.9843379	0.0023474	10.364
gi|164518957|ref|NM_001113287.1|	skyblue	250.65295	24.382618	0.9820122	0.0028882	726.32
gi|114326182|ref|NM_001048072.1|	salmon	250.62737	42.977575	0.9765453	0.00429681	330.448
gi|115550183|dbj|AK235662.1|	yellow	250.57515	61.311507	0.9615154	0.00901035	10.726
gi|115555063|dbj|AK240262.1|	purple	250.53408	59.076933	0.9926086	0.00076197	42.774

gnl|UG|Ssc#S34516523	darkgrey	250.41538	17.037549	0.9919954	0.00085866	9.286
gnl|UG|Ssc#S18546458	blue	250.07516	148.19598	0.9278709	0.02300085	2.386
gnl|UG|Ssc#S18359204	blue	249.87557	117.22238	0.8744718	0.05237142	99.156
gnl|UG|Ssc#S41577334	midnightblu	249.73377	35.934349	0.9831944	0.00260865	19.446
gi|115551503|dbj|AK238967.1|	skyblue	249.62062	24.117044	0.9794013	0.00353792	13.502
gnl|UG|Ssc#S17515809	blue	249.52129	140.62676	0.9149819	0.02937538	53.774
gi|115546917|dbj|AK240563.1|	yellowgreer	249.40383	17.005674	0.9893695	0.00131362	73.322
gi|194041423|ref|XM_001926755.1|	turquoise	248.83155	192.44318	0.6076033	0.27704011	52.394
gnl|UG|Ssc#S18359685	turquoise	248.68645	196.34553	0.6189095	0.26566953	71.286
gnl|UG|Ssc#S39888860	black	248.36816	35.085548	0.8779147	0.05025876	29.396
gnl|UG|Ssc#S23690802	yellow	248.33277	58.401722	0.935997	0.01924954	108.47
gnl|UG|Ssc#S23757653	darkturquoi	248.28843	21.458938	0.9155373	0.02909052	9.47
gi|50657387|ref|NM_001002824.1|	darkorange	248.23391	27.278293	0.9744257	0.00489064	11.6
gnl|UG|Ssc#S23693871	lightyellow	248.13967	17.799633	0.8423752	0.07332065	16.074
gi|194033418|ref|XM_001924233.1|	darkturquoi	248.13925	23.241501	0.9388242	0.01799607	68.726
gnl|UG|Ssc#S18546946	lightcyan	248.03612	34.556257	0.961961	0.00885489	3.562
gnl|UG|Ssc#S40471332	darkorange	247.95339	27.866212	0.9789909	0.00364393	12.44
gnl|UG|Ssc#S40442878	yellow	247.84649	63.710506	0.9757073	0.00452855	87.674
gnl|UG|Ssc#S6666342	darkturquoi	247.67549	21.601326	0.9163634	0.02866841	16.39
gnl|UG|Ssc#S18283235	turquoise	247.52903	190.8886	0.5989908	0.28578539	25.704
gi|115550976|dbj|AK238837.1|	salmon	247.49222	39.881058	0.9576142	0.0104084	71.458
gi|52351956|gb|AY610374.1|	lightcyan	246.991	34.853571	0.9645471	0.00797054	64.008
gnl|UG|Ssc#S40170485	midnightblu	246.95111	35.696887	0.9750214	0.00472118	56.262
gnl|UG|Ssc#S17526634	brown	246.9353	89.164562	0.9528802	0.01219117	22.218
gnl|UG|Ssc#S6032057	purple	246.74258	58.633738	0.9911002	0.00100652	7.03
gi|194044929|ref|XM_001925991.1|	yellow	246.63541	54.536549	0.8871327	0.04473972	45.65
gi|47522861|ref|NM_214020.1|	white	246.31207	18.935647	0.0711732	0.90945608	25.604
gi|115546548|dbj|AK234386.1|	blue	246.23988	150.77977	0.9321761	0.02098648	9.412
gi|115553370|dbj|AK236573.1|	brown	246.14207	92.015184	0.9614593	0.00902999	75.048
gnl|UG|Ssc#S18547043	yellow	246.09049	48.763214	0.9040595	0.03515492	26.804
gnl|UG|Ssc#S17509925	blue	246.09036	141.56883	0.916866	0.02841257	33.558
gnl|UG|Ssc#S18354739	violet	246.00355	3.4010785	0.167894	0.78723932	9.184
gi|194036424|ref|XM_001925710.1|	blue	245.92683	135.04554	0.9075298	0.03328276	52.748
gi|115550392|dbj|AK238657.1|	white	245.89247	20.734392	0.9358874	0.01929868	10.742
gnl|UG|Ssc#S23689821	blue	245.8585	144.33429	0.9209418	0.02636551	9.222
gi|52352065|gb|AY610481.1|	lightgreen	245.29264	24.008188	0.9476678	0.01425768	1.724
gi|194044044|ref|XM_001927332.1|	blue	245.20321	131.33973	0.8971678	0.03896841	11.434
gi|194043649|ref|XM_001925542.1|	midnightblu	245.17575	30.954993	0.947425	0.01435649	14.376
gnl|UG|Ssc#S39773791	greenyellow	244.9057	34.147358	0.9212973	0.02618931	33.7
gnl|UG|Ssc#S35329994	purple	244.90525	57.935341	0.9886829	0.00144277	10.078
gnl|UG|Ssc#S29485097	green	244.4698	47.390464	0.8512606	0.06730355	2.71
gnl|UG|Ssc#S6074613	yellowgreer	244.23111	16.874569	0.9862852	0.00192408	26.724
gi|113205849|ref|NM_001044600.1|	lightcyan	244.19616	34.08351	0.9577619	0.01035429	24.618
gi|115547309|dbj|AK234682.1|	black	243.95479	36.647408	0.8921484	0.04182346	23.882
gnl|UG|Ssc#S18382807	pink	243.94057	20.402821	-0.880378	0.04876382	125.968
gnl|UG|Ssc#S26725722	purple	243.36115	58.349209	0.9899348	0.00121034	9.574
gnl|UG|Ssc#S18557342	turquoise	243.35762	179.81496	0.5905648	0.29440971	27.922
gnl|UG|Ssc#S23698058	turquoise	243.34394	159.68659	0.5650165	0.32095787	20.664

gi|113205905|ref|NM_001044614.1|	darkorange	243.17602	26.82828	0.9717595	0.00567275	199.308
gi|115548717|dbj|AK231783.1|	purple	243.03405	58.52469	0.9906033	0.0010919	67.262
gi|194034359|ref|XM_001927037.1|	turquoise	242.96303	193.52867	0.6119489	0.27265482	54.722
gi|194036392|ref|XM_001929357.1|	black	242.82882	42.727955	0.9374862	0.01858588	6.396
gnl|UG|Ssc#S16350749	midnightblu	242.71459	36.621133	0.983415	0.00255754	13.404
gi|194040487|ref|XM_001926988.1|	blue	242.65951	140.91527	0.9158093	0.02895132	13.69
gnl|UG|Ssc#S17516151	yellow	242.62767	64.99374	0.9625018	0.00866746	25.99
gi|115553998|dbj|AK233207.1|	midnightblu	242.58079	37.274468	0.9904458	0.00111945	14.384
gi|190360618|ref|NM_001128433.1|	purple	242.57567	58.954058	0.9921328	0.00083667	17.088
gi|115548441|dbj|AK235058.1|	brown	242.40398	86.869536	0.948812	0.01379504	25.096
gnl|UG|Ssc#S35164631	lightcyan	242.40308	34.029854	0.9589318	0.00992885	1.456
gnl|UG|Ssc#S17511033	darkturquoi	242.39353	26.435139	0.9656242	0.00761134	22.538
gnl|UG|Ssc#S16511729	brown	242.20777	78.865778	0.9237618	0.02497829	83.156
gi|47523501|ref|NM_214213.1|	purple	242.04855	58.213483	0.9893884	0.00131013	11.924
gnl|UG|Ssc#S23696008	blue	241.87905	117.83286	0.8796151	0.04922548	8.198
gnl|UG|Ssc#S39853219	salmon	241.68953	38.912327	0.9516443	0.01267155	13.852
gnl|UG|Ssc#S6047303	purple	241.66872	57.013531	0.9852216	0.00215185	35.854
gi|54020965|ref|NM_001005726.1|	magenta	241.61313	15.990352	0.0110412	0.98594218	360.584
gnl|UG|Ssc#S40127774	magenta	241.57577	29.388987	0.9235551	0.02507916	41.808
gnl|UG|Ssc#S34530291	skyblue	241.5678	23.924951	0.9784184	0.00379358	24.694
gi|52351755|gb|AY610185.1|	blue	241.51233	148.69038	0.927329	0.02325862	1.02
gnl|UG|Ssc#S17518115	greenyellow	241.14295	33.764946	0.9202984	0.02668534	37.006
gi|52351253|gb|AY609685.1|	skyblue	241.07799	22.711322	0.9652688	0.00772925	54.752
gi|115550487|dbj|AK238752.1|	skyblue	241.07651	24.135886	0.9801255	0.00335334	63.776
gnl|UG|Ssc#S17517714	skyblue	240.55153	24.187594	0.9813228	0.00305549	21.538
gnl|UG|Ssc#S23699367	darkgreen	240.54823	17.13504	0.7275747	0.16353449	5.42
gnl|UG|Ssc#S17513449	salmon	240.43208	40.781831	0.9646199	0.00794611	329.714
gnl|UG|Ssc#S18387223	salmon	240.40044	42.660824	0.9746137	0.00483694	115.344
gnl|UG|Ssc#S16769741	yellow	240.04516	67.09343	0.9816377	0.0029787	27.324
gi|115551518|dbj|AK238982.1|	darkturquoi	240.02256	23.37255	0.9309594	0.02154968	9.798
gnl|UG|Ssc#S23760326	salmon	239.93366	43.188761	0.9784162	0.00379414	10.962
gnl|UG|Ssc#S18387292	turquoise	239.6648	209.05126	0.6408152	0.24400735	37.09
gnl|UG|Ssc#S31133723	violet	239.62349	17.554648	0.9244895	0.02462429	18.198
gnl|UG|Ssc#S19546091	purple	239.60969	57.519263	0.9869047	0.00179535	18.116
gi|115553671|dbj|AK239858.1|	purple	239.54981	49.884826	0.9570107	0.01063052	153.754
gi|115552064|dbj|AK239257.1|	grey	239.40101	10.847503	0.7541618	0.14080126	77.112
gi|194035600|ref|XM_001927804.1|	purple	239.33114	56.185327	0.9823168	0.00281528	124.75
gi|115549210|dbj|AK231886.1|	white	239.30942	17.700061	-0.123412	0.84326655	5.972
gnl|UG|Ssc#S16763478	greenyellow	239.26101	36.852987	0.9512096	0.01284195	17.756
gi|194034864|ref|XM_001929220.1|	brown	239.24344	85.571451	0.9433438	0.01605017	28.294
gnl|UG|Ssc#S19544231	blue	238.91778	128.66637	0.898189	0.03839546	35.074
gi|160420270|ref|NM_001044582.1|	grey60	238.64623	16.207228	0.9634981	0.00832558	162.178
gnl|UG|Ssc#S39964958	pink	237.93901	19.536503	-0.88297	0.04720706	6.454
gnl|UG|Ssc#S18380485	greenyellow	237.92885	36.433015	0.9452533	0.01525007	30.016
gi|194034162|ref|XM_001925693.1|	brown	237.87694	63.890199	0.8720115	0.05389791	63.868
gnl|UG|Ssc#S6072982	yellowgreer	237.87314	16.591153	0.9842793	0.00236056	10.95
	yellowgreer					

gnl|UG|Ssc#S23695047	turquoise	237.70394	212.71657	0.6297607	0.25487703	25.66
gnl|UG|Ssc#S17525130	white	237.65502	23.263778	0.9428077	0.01627718	16.678
gi|115552269|dbj|AK230578.1|	yellow	237.58919	53.292275	0.9233046	0.02520154	248.638
gnl|UG|Ssc#S23762445	red	237.28935	26.393901	0.8937053	0.04093103	8.198
gi|115550159|dbj|AK235638.1|	turquoise	237.27879	203.90697	0.6356133	0.24910639	4.7
gi|194036460|ref|XM_001926279.1|	grey	237.22243	10.612099	0.6607983	0.22469072	7.002
gi|194039388|ref|XM_001929568.1|	darkorange	237.12868	27.980998	0.98031	0.00330686	48.22
gi|194033869|ref|XM_001924394.1|	turquoise	237.1005	152.59532	0.5656496	0.32029298	11.928
gnl|UG|Ssc#S23764997	midnightblu	236.81574	35.90023	0.9785613	0.00375603	26.532
gnl|UG|Ssc#S29988897	yellow	236.71285	59.716193	0.9558285	0.01107001	28.94
gnl|UG|Ssc#S17511213	brown	236.26793	82.849963	0.9383047	0.01822432	17.522
gnl|UG|Ssc#S40048956	turquoise	236.25628	160.46055	0.5614175	0.32474438	34.636
gnl|UG|Ssc#S40474839	salmon	236.24832	41.978265	0.9711219	0.00586539	8.138
gi|52351593|gb|AY610023.1|	pink	236.21309	21.33607	-0.864967	0.0583439	223.9
gi|194034692|ref|XM_001928709.1|	violet	235.89916	3.9990079	0.2247907	0.71621658	29.182
gnl|UG|Ssc#S31115857	navy	235.8032	28.67047	0.9292762	0.02233678	9.13
gi|195539469|ref|NM_001130733.1|	brown	235.76901	82.761559	0.9375254	0.01856849	60.17
gi|162139822|ref|NM_001111257.1|	salmon	235.74884	37.622755	0.9439951	0.01577575	71.254
gi|34582610|gb|AY368623.1|	salmon	235.68116	42.007396	0.9705966	0.00602567	656.618
gnl|UG|Ssc#S18384142	yellow	235.63778	44.396117	0.8619025	0.06031223	29.05
gi|194045084|ref|XM_001925136.1|	brown	235.50506	64.923849	0.8790068	0.04959436	10.282
gnl|UG|Ssc#S18546597	salmon	235.44656	39.621386	0.9568196	0.01070114	15.206
gi|115551358|dbj|AK236036.1|	blue	235.29001	146.3628	0.9254809	0.02414462	38.558
gnl|UG|Ssc#S23694531	darkorange	235.23376	27.244215	0.9747966	0.00478491	58.594
gi|115546327|dbj|AK230846.1|	grey60	235.03728	11.216184	0.5377857	0.34988022	7.466
gi|47523683|ref|NM_214309.1|	lightcyan	235.01418	35.870492	0.9712437	0.00582842	22.75
gnl|UG|Ssc#S6072690	darkorange	234.9686	26.55766	0.9689712	0.00653055	21.454
gi|115545600|dbj|AK230715.1|	greenyellow	234.66422	36.496813	0.9389721	0.01793126	40.89
gi|115551591|dbj|AK239055.1|	turquoise	234.64817	167.85063	0.5807485	0.30454048	24.988
gi|194474047|ref|NM_001130535.1|	turquoise	234.62303	211.30004	0.6305118	0.25413443	107.038
gnl|UG|Ssc#S40481511	darkturquoi	234.58856	18.982255	0.8866754	0.04500872	89.402
gnl|UG|Ssc#S39946921	darkorange	234.36864	27.265859	0.9746193	0.00483537	144.236
gi|47522737|ref|NM_213954.1|	purple	234.2631	57.084858	0.9852957	0.0021357	53.226
gi|115554563|dbj|AK236973.1|	darkgreen	234.12428	20.29658	0.8996552	0.03757761	12.226
gnl|UG|Ssc#S17510300	salmon	234.11438	37.052478	0.9415468	0.01681519	2.75
gnl|UG|Ssc#S18383725	purple	234.09044	52.446175	0.9675885	0.00697043	80.128
gnl|UG|Ssc#S23755551	white	233.64069	24.749553	0.9650752	0.00779375	34.496
gi|115550430|dbj|AK238695.1|	darkorange	233.5067	27.077191	0.9734719	0.00516605	23.65
gi|178057228|ref|NM_001123071.1|	midnightblu	233.49044	37.344421	0.99153	0.00093456	89.876
gnl|UG|Ssc#S18357309	black	233.35214	44.021735	0.9443549	0.01562483	12.222
gi|194035836|ref|XM_001928869.1|	turquoise	233.12667	153.7112	0.5627361	0.32335577	83.506
gnl|UG|Ssc#S17514245	blue	232.85548	125.66984	0.8936176	0.04098115	29.406
gnl|UG|Ssc#S18555414	purple	232.73112	57.253071	0.9858307	0.00202038	31.062
gnl|UG|Ssc#S40055957	skyblue	232.47562	26.103426	0.9961433	0.00028734	15.548
gi|47523487|ref|NM_214201.1|	black	232.41277	37.994345	0.900313	0.03721247	72.562
gi|190360658|ref|NM_001128471.1|	lightcyan	232.39298	38.322495	0.9861232	0.00195821	63.19
gnl|UG|Ssc#S35334259	darkgreen	232.24055	21.879869	0.8856972	0.04558577	14.852
gi|115550330|dbj|AK238595.1|	purple	232.2261	53.147749	0.9702931	0.00611892	7.858

gi|194042805|ref|XM_001927802.1|	lightcyan	231.76876	34.699843	0.9637525	0.00823903	238.802
gi|115548435|dbj|AK235052.1|	black	231.52665	37.923694	0.9034858	0.03546754	5.566
gi|194033747|ref|XM_001927557.1|	greenyellow	231.49674	27.540779	0.8874351	0.04456213	12.302
gi|47523095|ref|NM_213881.1|	yellow	231.48039	55.649467	0.8937798	0.04088849	116.116
gnl|UG|Ssc#S29989765	yellowgreen	231.38495	16.230123	0.9787443	0.00370814	29.02
gnl|UG|Ssc#S40488267	lightgreen	231.16241	25.086966	0.9503781	0.01316997	13.744
gi|115554002|dbj|AK233211.1|	midnightblu	230.89295	36.026894	0.9798428	0.00342502	20.43
gi|115546438|dbj|AK230957.1|	midnightblu	230.76835	34.468814	0.9698548	0.00625441	126.71
gi|52351363|gb|AY609793.1|	black	230.75336	44.313505	0.9462191	0.01485051	9.576
gi|194043488|ref|XM_001929645.1|	yellowgreen	230.70055	16.17475	0.9787867	0.0036971	13.56
gnl|UG|Ssc#S16764603	lightcyan	230.52528	38.438214	0.9854343	0.00210562	3.942
gnl|UG|Ssc#S17511222	lightgreen	230.37233	23.005757	0.9114125	0.03122741	2.944
gi|55742741|ref|NM_213920.1|	red	230.19805	22.765006	0.8514663	0.06716615	1411.28
gnl|UG|Ssc#S23701295	darkgreen	229.94375	14.331288	-0.708743	0.18021843	367.018
gi|47523037|ref|NM_214115.1|	skyblue	229.8506	25.509929	0.9911852	0.00099215	178.788
gnl|UG|Ssc#S39842527	yellow	229.69441	52.472705	0.9142968	0.02972802	0.478
gi|47523579|ref|NM_214250.1|	lightyellow	229.42193	15.020734	0.8032837	0.1015887	61.3
gi|113205649|ref|NM_001044550.1|	greenyellow	229.25863	36.864607	0.9404739	0.01727742	74.694
gnl|UG|Ssc#S18295298	yellow	229.20156	69.495577	0.9806051	0.00323295	11.786
gi|194042303|ref|XM_001928772.1|	midnightblu	229.12843	32.957713	0.9611318	0.00914488	148.574
gnl|UG|Ssc#S40047900	purple	229.10104	53.693233	0.9727761	0.00537005	15.518
gi|194044593|ref|XM_001929063.1|	purple	229.08227	53.913518	0.9733697	0.00519583	41.988
gi|115552216|dbj|AK230525.1|	darkgreen	228.92069	20.31872	0.7846484	0.11601236	24.368
gnl|UG|Ssc#S23695151	lightcyan	228.66852	39.493747	0.9928874	0.00071931	8.83
gi|194038900|ref|XM_001925133.1|	yellowgreen	228.57279	13.800443	0.9451445	0.01529531	37.584
gnl|UG|Ssc#S19540267	magenta	228.40646	14.364346	-0.099681	0.87329209	29.118
gi|115550429|dbj|AK238694.1|	darkturquoi	228.39698	25.494044	0.9547726	0.01146749	16.29
gi|115547607|dbj|AK231265.1|	darkgreen	228.17424	18.491962	0.7762196	0.1227222	14.864
gi|115550750|dbj|AK235824.1|	salmon	227.97537	37.534515	0.9444055	0.01560366	9.588
gnl|UG|Ssc#S23769110	brown	227.87265	72.741524	0.908419	0.03280832	34.34
gnl|UG|Ssc#S26650236	turquoise	227.75958	161.3504	0.565477	0.3204742	13.908
gnl|UG|Ssc#S18553314	lightcyan	227.74319	37.417058	0.9806933	0.00321094	5.26
gnl|UG|Ssc#S40053418	pink	227.67516	9.3118445	0.5036981	0.38692957	110.146
gnl|UG|Ssc#S23772025	blue	227.67276	123.64605	0.889757	0.04320612	6.118
gnl|UG|Ssc#S19541855	brown	227.67165	74.43966	0.9097349	0.0321103	32.296
gi|52351640|gb|AY610070.1|	skyblue	227.64763	24.493153	0.9850703	0.00218492	9.946
gi|115554762|dbj|AK233574.1|	pink	227.44943	21.789935	-0.837511	0.07668168	88.734
gnl|UG|Ssc#S26396016	paleturquois	227.39388	8.2739621	0.8025195	0.10216868	6.796
gnl|UG|Ssc#S34500909	grey60	227.20312	15.232286	0.6256575	0.25894387	21.992
gnl|UG|Ssc#S18379831	white	226.74431	24.22697	0.9613631	0.00906368	4.638
gnl|UG|Ssc#S40042784	skyblue	226.73567	26.105703	0.9953665	0.00037835	29.964
gnl|UG|Ssc#S18546970	lightcyan	226.72903	38.80461	0.9875156	0.00167136	4.66
gnl|UG|Ssc#S40475805	skyblue	226.66041	26.175207	0.9962822	0.00027197	132.38
gi|194041160|ref|XM_001927391.1|	lightcyan	226.55148	39.501877	0.9926583	0.00075431	12.806
gnl|UG|Ssc#S17510736	blue	226.54313	127.58393	0.894208	0.04064419	20.676
gi|194036282|ref|XM_001924669.1|	blue	226.51036	131.96097	0.9012249	0.03670823	10.624
gi|115551063|dbj|AK238924.1|	brown	226.45316	58.071153	0.8488947	0.06889007	27.128
gnl|UG|Ssc#S40024674	brown	226.3351	78.215642	0.9252686	0.02424707	16.618

gi|47523015|ref|NM_214103.1|	pink	226.28842	17.760975	-0.857552	0.06314146	189.864
gnl|UG|Ssc#S6029610	brown	226.26897	59.173125	0.853514	0.06580327	27.846
gi|194040969|ref|XM_001924529.1|	yellow	226.25413	70.256954	0.9916173	0.00092016	85.216
gnl|UG|Ssc#S16763114	midnightblu	226.19841	34.081494	0.9713288	0.00580264	4.076
gnl|UG|Ssc#S39839709	darkgreen	226.18058	21.044507	0.9220386	0.0258231	9.414
gnl|UG|Ssc#S19545362	brown	226.15834	77.396244	0.9244335	0.02465149	35.916
gnl|UG|Ssc#S19545017	magenta	226.01132	15.492028	-0.134027	0.82986331	26.996
gi|153792048|ref|NM_001099934.1|	lightcyan	225.81439	38.001446	0.9848166	0.00224077	144.416
gi|194039486|ref|XM_001927002.1|	pink	225.66185	22.750263	-0.888217	0.04410397	98.452
gi|164664451|ref|NM_001113442.1|	darkgreen	225.61321	10.572344	0.7113491	0.17788219	40.522
gi|115550482|dbj|AK238747.1|	grey60	225.55284	14.800622	0.8453167	0.07131099	5.7
gi|115548388|dbj|AK235005.1|	turquoise	225.50689	139.08379	0.5304346	0.35779313	126.084
gi|194042190|ref|XM_001924717.1|	skyblue	225.18353	25.09574	0.9906843	0.00107783	124.124
gi|194042305|ref|XM_001928810.1|	blue	225.12566	115.52803	0.8680072	0.05641155	78.604
gnl|UG|Ssc#S19542017	grey60	225.07239	11.001504	0.4815467	0.41147307	50.816
gnl|UG|Ssc#S40340051	skyblue	224.9483	26.295099	0.9988809	4.49E-05	27.736
gnl|UG|Ssc#S27602037	purple	224.93373	55.008099	0.9774393	0.00405404	18.256
gi|47523779|ref|NM_214361.1|	yellow	224.89747	38.404168	0.7764818	0.12251183	98.056
gi|194038777|ref|XM_001928268.1|	magenta	224.56574	15.322783	-0.149792	0.80999409	62.916
gi|52351547|gb|AY609977.1|	skyblue	224.56399	26.036468	0.9953523	0.0003801	50.076
gi|194045072|ref|XM_001927451.1|	skyblue	224.29669	25.88705	0.9957563	0.00033165	12.064
gnl|UG|Ssc#S40588301	skyblue	224.26501	25.541446	0.9933949	0.00064376	242.774
gi|194043830|ref|XM_001925246.1|	royalblue	224.13933	19.441364	0.9477526	0.01422322	8.896
gnl|UG|Ssc#S18387444	blue	224.05379	129.33402	0.8945101	0.04047214	2.204
gi|115551001|dbj|AK238862.1|	salmon	224.04064	41.031813	0.9651412	0.00777173	524.732
gnl|UG|Ssc#S40388648	blue	223.8883	102.54621	0.8427415	0.07306941	24.338
gi|115555150|dbj|AK233765.1|	grey60	223.86364	12.388829	0.6142776	0.27031247	75.198
gnl|UG|Ssc#S18357319	yellow	223.79637	64.581639	0.9609709	0.00920146	22.716
gi|47522641|ref|NM_213922.1|	black	223.54268	31.268795	0.8509098	0.06753808	29.578
gi|194038687|ref|XM_001928174.1|	lightcyan	223.32501	37.948637	0.9831997	0.00260743	52.494
gi|194042161|ref|XM_001925705.1|	midnightblu	223.19412	34.640465	0.9719405	0.00561846	29.772
gi|158262676|ref|NM_001109945.1|	purple	223.18718	52.03848	0.9658701	0.00753009	10.514
gi|115550185|dbj|AK235664.1|	greenyellow	223.17647	36.058899	0.9368423	0.0188719	15.482
gnl|UG|Ssc#S34533088	midnightblu	223.13007	35.698637	0.9797959	0.00343694	18.182
gnl|UG|Ssc#S40546869	purple	223.02802	54.183036	0.9741961	0.00495648	14.49
gnl|UG|Ssc#S40474257	salmon	222.97942	37.729968	0.9452638	0.01524569	26.662
gnl|UG|Ssc#S19541687	blue	222.7302	124.20787	0.8880213	0.04421852	4.536
gi|115551776|dbj|AK236182.1|	greenyellow	222.64511	35.579972	0.9308855	0.02158408	49.798
gi|148225749|ref|NM_001097504.1|	pink	222.59495	20.083503	-0.894483	0.04048768	39.36
gi|72535177|ref|NM_001031781.1|	lightcyan	222.56291	40.141247	0.9966693	0.00023063	295.422
gi|194044032|ref|XM_001928774.1|	blue	222.54359	111.55582	0.8594334	0.06191295	41.974
gnl|UG|Ssc#S35324845	black	222.51372	40.518745	0.9213292	0.0261735	4.24
gnl|UG|Ssc#S31116354	black	222.46999	24.780278	0.8055519	0.09987306	8.24
gnl|UG|Ssc#S23762759	blue	222.45081	121.84161	0.8827429	0.04734269	10.584
gnl|UG|Ssc#S40437350	brown	222.36777	74.061018	0.9145291	0.02960829	37.078
gi|194044171|ref|XM_001924505.1|	blue	222.10971	123.24096	0.8863236	0.04521597	5.26
gnl|UG|Ssc#S20947929	blue	222.0802	134.23321	0.9054769	0.0343863	12.38
gi|115547083|dbj|AK231138.1|	darkgreen	222.0331	21.17857	0.8236255	0.08652678	9.454

gi|124558111|gb|EF154832.1|	darkgreen	222.01314	17.05444	-0.571675	0.31398196	19.596
gi|194036941|ref|XM_001925986.1|	black	221.86989	25.217015	0.8213099	0.08820379	20.184
gi|47522761|ref|NM_213967.1|	turquoise	221.63115	156.77325	0.5528045	0.33385124	56.18
gi|194042690|ref|XM_001929322.1|	purple	221.60052	50.704255	0.9610715	0.00916608	90.348
gnl|UG|Ssc#S40439319	magenta	221.5825	17.158865	0.016975	0.97838779	40.462
gi|194036601|ref|XM_001929330.1|	blue	221.56051	95.090176	0.8223959	0.08741608	23.556
gnl|UG|Ssc#S26645293	brown	221.54622	66.529496	0.8835596	0.04685487	15.018
gi|52352037|gb|AY610453.1|	pink	221.25246	22.125412	-0.903763	0.03531655	11.056
gi|115554075|dbj|AK233284.1|	magenta	221.01384	16.863307	-0.002834	0.99639226	163.734
gi|115553764|dbj|AK236768.1|	midnightblu	221.0063	31.012537	0.9409738	0.01706155	10.992
gnl|UG|Ssc#S38482319	darkgreen	220.68619	17.390531	-0.569116	0.31665892	84.834
gnl|UG|Ssc#S19542228	darkgreen	220.55558	17.660442	-0.532754	0.35529212	17.818
gnl|UG|Ssc#S40267793	purple	220.52183	53.298655	0.9706597	0.00600635	23.262
gi|115547172|dbj|AK231227.1|	skyblue	220.4677	24.31972	0.9834466	0.00255024	6.976
gnl|UG|Ssc#S23698716	yellow	220.36027	61.79062	0.9370287	0.01878895	60.822
gi|47523675|ref|NM_214305.1|	skyblue3	220.05333	13.896208	0.9442778	0.01565715	2.524
gnl|UG|Ssc#S23775557	turquoise	219.91947	172.88967	0.5914843	0.29346534	37.858
gi|194040778|ref|XM_001927213.1|	purple	219.89157	50.40656	0.9593422	0.009781	31.056
gnl|UG|Ssc#S18553358	lightcyan	219.65252	38.080718	0.984273	0.00236198	31.804
gi|115545780|dbj|AK234213.1|	blue	219.64993	115.94653	0.8742029	0.05253761	25.396
gi|194043363|ref|XM_001929468.1|	yellowgreen	219.61747	12.346419	0.9233945	0.02515764	26.3
gnl|UG|Ssc#S40512983	brown	219.18994	79.831368	0.9280471	0.02291726	5.672
gnl|UG|Ssc#S19542616	royalblue	219.17056	18.276683	0.9163928	0.02865343	11.226
gi|115548831|dbj|AK231946.1|	salmon	219.10327	36.311308	0.9381162	0.01830741	116.178
gnl|UG|Ssc#S6006110	midnightblu	219.07128	34.357368	0.9698136	0.00626722	9.552
gi|52350686|gb|AY609402.1|	brown	218.88498	66.573911	0.888829	0.04374651	8.816
gi|194042129|ref|XM_001927395.1|	midnightblu	218.85286	27.942688	0.9169555	0.02836712	25.698
gi|115550394|dbj|AK238659.1|	yellow	218.80918	66.28948	0.9564089	0.01085352	66.9
gnl|UG|Ssc#S35333016	purple	218.55074	49.185244	0.9542753	0.01165625	14.618
gi|115547436|dbj|AK237818.1|	lightgreen	218.51186	24.788573	0.9469364	0.01455598	41.67
gnl|UG|Ssc#S40048435	darkgreen	218.47481	17.259606	-0.51414	0.37548473	82.974
gnl|UG|Ssc#S16767076	yellow	218.40693	65.199096	0.9621638	0.00878445	121.18
gnl|UG|Ssc#S18353806	green	218.23242	37.338313	0.8024918	0.10218972	28.66
gi|194035708|ref|XM_001927062.1|	blue	218.17162	115.09374	0.868023	0.05640161	27.838
gnl|UG|Ssc#S38481167	tan	218.12844	35.531746	0.9361239	0.01919271	6.422
gi|115552842|dbj|AK239436.1|	yellow	218.03565	57.067655	0.9094995	0.03223484	296.484
gnl|UG|Ssc#S31134161	brown	217.8926	78.956329	0.9246652	0.02453907	2.96
gi|194041609|ref|XM_001928028.1|	grey60	217.89225	14.75997	0.625296	0.25930303	72.568
gi|115547025|dbj|AK231080.1|	grey60	217.83924	13.620692	0.7895086	0.11219521	12.88
gi|212549622|ref|NM_001137629.1|	grey60	217.65473	12.7242	0.7106151	0.17853941	20.636
gnl|UG|Ssc#S19542661	lightyellow	217.51633	19.687218	0.8748916	0.05211241	2.414
gnl|UG|Ssc#S26711201	yellow	217.1941	62.879997	0.9692547	0.00644152	11.752
gnl|UG|Ssc#S31102082	lightcyan	216.96205	38.818198	0.9902266	0.00115814	3.294
gi|47523093|ref|NM_213884.1|	skyblue3	216.83039	16.625782	0.9853377	0.00212657	136.68
gi|194034148|ref|XM_001924679.1|	turquoise	216.82007	191.80806	0.6105358	0.27407877	31.71
gnl|UG|Ssc#S18378493	lightcyan	216.55522	40.439717	0.9989379	4.15E-05	15.348
gnl|UG|Ssc#S6060797	skyblue	216.44116	23.513338	0.9766736	0.00426168	8.534
gnl|UG|Ssc#S29983177	salmon	216.39846	35.007681	0.9292233	0.02236166	10.594

gnl|UG|Ssc#S35322082	purple	216.34901	52.509224	0.9674928	0.00700122	6.478
gi|194035716|ref|XM_001925987.1|	yellow	216.05819	33.410085	0.7313125	0.16027903	30.74
gi|91176934|gb|DQ450676.1|	grey60	215.7268	15.234234	0.7808815	0.11899707	8.406
gi|115548797|dbj|AK231755.1|	midnightblu	215.64212	30.17722	0.9402057	0.01739361	69.638
gnl|UG|Ssc#S20451601	sienna3	215.51627	17.190338	0.9725013	0.00545133	2.878
gi|194041329|ref|XM_001928735.1|	darkorange	215.4305	22.188296	0.9327027	0.02074423	9.702
gnl|UG|Ssc#S18378075	skyblue	215.40514	25.901807	0.9929627	0.00070792	8.196
gnl|UG|Ssc#S23700711	yellowgreer	215.32949	12.268839	0.9209126	0.02638	5.498
gnl|UG|Ssc#S40131124	black	215.24829	37.245398	0.8974046	0.03883533	4.616
gi|52351209|gb|AY609612.1|	darkmagent	215.08052	9.6638255	0.8747363	0.05220817	63.672
gnl|UG|Ssc#S17517405	turquoise	215.03368	178.02878	0.5930638	0.29184497	15.13
gnl|UG|Ssc#S39967395	skyblue	214.88552	24.080009	0.9760316	0.00443838	8.616
gnl|UG|Ssc#S39789448	orange	214.83855	11.941308	0.9071413	0.03349068	38.92
gnl|UG|Ssc#S35329254	greenyellow	214.83402	27.055921	0.8757674	0.05157323	28.768
gi|194041219|ref|XM_001928126.1|	greenyellow	214.7395	24.325049	0.8562835	0.06397388	19.292
gnl|UG|Ssc#S40284317	lightcyan	214.73294	40.289546	0.997605	0.00014065	0.118
gnl|UG|Ssc#S18546426	grey	214.4075	12.207038	0.7552114	0.13992443	88.608
gi|115548526|dbj|AK235143.1|	yellow	214.25152	59.129019	0.9267759	0.02352265	107.132
gi|194033755|ref|XM_001924851.1|	red	213.97459	32.743298	0.9753179	0.00463759	35.328
gi|194044925|ref|XM_001927734.1|	midnightblu	213.96777	32.40235	0.9605149	0.00936255	142.472
gnl|UG|Ssc#S38481028	grey60	213.86124	16.616266	0.7798992	0.11977915	27.386
gnl|UG|Ssc#S40038955	yellow	213.70836	66.199463	0.9559667	0.01101836	398.304
gnl|UG|Ssc#S23770901	skyblue3	213.69098	15.881986	0.9747937	0.00478573	10.488
gnl|UG|Ssc#S40335680	violet	213.51599	12.559664	0.8797903	0.04911939	5.754
gnl|UG|Ssc#S23696828	tan	213.47619	42.202562	0.9761185	0.00441432	64.982
gi|115548520|dbj|AK235137.1|	darkturquoi	213.38398	22.566994	0.9301	0.02195044	250.98
gnl|UG|Ssc#S17526093	midnightblu	213.35421	29.844539	0.9418791	0.01667285	33.602
gi|52350683|gb|AY609399.1|	orange	213.32319	12.360884	0.9265268	0.02364188	127.218
gi|47522727|ref|NM_213949.1|	pink	213.15771	20.785219	0.8040558	0.1010037	26.792
gi|52351260|gb|AY609692.1|	sienna3	213.12767	17.136565	0.9722608	0.00552281	50.184
gnl|UG|Ssc#S18546943	darkgreen	213.07128	21.168535	0.8537485	0.06564775	19.69
gi|194041401|ref|XM_001926294.1|	orange	212.85638	11.909594	0.9025397	0.03598506	4.182
gi|47523561|ref|NM_214245.1|	lightgreen	212.6852	26.58032	0.9673403	0.0070504	40.534
gnl|UG|Ssc#S40143544	turquoise	212.58371	162.51958	0.5732321	0.31235686	16.048
gnl|UG|Ssc#S35327311	navy	212.56704	32.496128	0.9604041	0.00940182	18.12
gnl|UG|Ssc#S17512305	blue	212.48255	106.88438	0.8528278	0.066259	5.46
gnl|UG|Ssc#S23760463	grey60	212.47033	15.369399	0.7681662	0.1292373	89.456
gnl|UG|Ssc#S6058884	tan	212.40716	39.307511	0.9587424	0.00999732	32.558
gnl|UG|Ssc#S17517004	turquoise	212.36436	149.62794	0.5520988	0.33460015	9.38
gnl|UG|Ssc#S29463694	orange	212.35239	12.691157	0.9426173	0.01635805	8.028
gnl|UG|Ssc#S23768000	darkturquoi	212.33748	21.181261	0.9197376	0.02696516	7.388
gnl|UG|Ssc#S18279395	skyblue3	212.30336	16.139224	0.9807764	0.00319028	24.734
gnl|UG|Ssc#S17526150	lightgreen	212.23991	22.395802	0.9054484	0.03440168	6.372
gnl|UG|Ssc#S39780074	tan	212.19651	37.051687	0.9452696	0.0152433	9.368
gnl|UG|Ssc#S17525753	yellow	211.94943	43.893743	0.8157223	0.09229085	77.304
gi|115550476|dbj|AK238741.1|	tan	211.72924	43.068956	0.9796806	0.00346635	27.95
gnl|UG|Ssc#S31122306	darkgreen	211.604	18.206692	-0.494607	0.39695998	201.19
gnl|UG|Ssc#S16769072	darkturquoi	211.56688	23.966427	0.9433679	0.01604	9

gi|194042016|ref|XM_001926579.1|	midnightblu	211.50782	30.679303	0.9480047	0.01412092	229.296
gi|147906088|ref|NM_213946.2|	purple	211.02178	48.903585	0.9528452	0.01220468	41.384
gnl|UG|Ssc#S31124728	darkgreen	210.96159	17.865819	-0.436617	0.4622884	45.374
gnl|UG|Ssc#S18550190	lightgreen	210.9141	27.060307	0.9673285	0.0070542	21.236
gnl|UG|Ssc#S6081209	darkgreen	210.88564	18.105287	-0.433132	0.46628313	38.842
gi|194040263|ref|XM_001927880.1|	darkgreen	210.85657	18.108037	-0.446917	0.45052246	26.906
gnl|UG|Ssc#S31133725	pink	210.76765	19.923857	-0.978124	0.00387139	7.626
gnl|UG|Ssc#S18292679	turquoise	210.53778	121.50356	0.5017523	0.38907111	3.694
gnl|UG|Ssc#S40543017	darkgrey	210.49903	14.73084	0.9325402	0.02081888	6.794
gnl|UG|Ssc#S40505966	tan	210.49838	42.383066	0.9762962	0.00436526	63.578
gnl|UG|Ssc#S40193201	turquoise	210.42804	164.48145	0.594459	0.29041557	17.674
gnl|UG|Ssc#S40047541	yellow	210.35614	63.795613	0.9488269	0.01378904	10.68
gnl|UG|Ssc#S29977753	yellow	210.34917	60.455075	0.9588044	0.0099749	7.738
gi|194037705|ref|XM_001924362.1|	sienna3	210.17805	18.246097	0.985764	0.00203463	63.574
gi|115550923|dbj|AK238784.1|	turquoise	210.07479	167.87936	0.5787176	0.30664738	7.36
gi|194035657|ref|XM_001928441.1|	tan	210.00827	43.371528	0.982379	0.00280045	118.834
gnl|UG|Ssc#S39794612	grey60	209.9675	14.630638	0.6934212	0.19412919	15.626
gi|194041319|ref|XM_001928397.1|	midnightblu	209.94169	29.783316	0.9385381	0.01812168	28.882
gi|52351090|gb|AY609495.1|	turquoise	209.86342	149.94436	0.5508475	0.33592919	398.6
gnl|UG|Ssc#S18549601	navy	209.8469	31.901366	0.964178	0.00809489	34.396
gi|194035679|ref|XM_001925389.1|	darkgreen	209.82891	17.390396	-0.545292	0.34184543	493.61
gnl|UG|Ssc#S16513514	sienna3	209.82038	16.307755	0.9622427	0.00875711	11.962
gnl|UG|Ssc#S18383636	tan	209.81466	41.588192	0.9702659	0.0061273	27.528
gnl|UG|Ssc#S26715223	darkgreen	209.46668	18.473886	-0.440244	0.45813689	46.936
gi|115546889|dbj|AK237735.1|	skyblue	209.45584	24.844737	0.9847017	0.0022662	13.428
gi|194034226|ref|XM_001928937.1|	yellow	209.22273	64.805423	0.9550553	0.01136061	157.192
gnl|UG|Ssc#S39993494	purple	209.10182	48.867337	0.9530586	0.01212233	46.732
gnl|UG|Ssc#S41578369	skyblue3	209.05859	15.669483	0.9708445	0.00594985	7.756
gi|117661185|gb|DQ629176.1|	yellow	209.03344	52.721908	0.9057008	0.03426538	160.946
gi|115554610|dbj|AK237020.1|	tan	209.0136	45.448705	0.9915959	0.00092369	54.518
gi|115554415|dbj|AK233425.1|	orange	208.98299	12.968052	0.9543776	0.01161734	41.58
gi|194041234|ref|XM_001925415.1|	greenyellow	208.9052	31.257261	0.9034927	0.03546378	32.552
gi|194034703|ref|XM_001926714.1|	red	208.90452	25.568677	0.8481419	0.06939726	15.14
gnl|UG|Ssc#S39955157	violet	208.90359	11.444759	-0.989495	0.00129047	18.36
gnl|UG|Ssc#S5995726	skyblue3	208.90047	16.985203	0.9894156	0.00130509	14.254
gnl|UG|Ssc#S23695164	salmon	208.63455	33.019057	0.9170195	0.02833458	211.696
gnl|UG|Ssc#S40049940	orange	208.63172	13.112802	0.9302632	0.02187416	18.276
gi|115550671|dbj|AK232501.1|	tan	208.48966	40.752778	0.9651002	0.00778541	47.74
gi|194034274|ref|XM_001924792.1|	lightcyan	208.23787	37.47358	0.9814126	0.00303353	16.146
gnl|UG|Ssc#S40292473	pink	208.19919	22.469369	0.9402138	0.0173901	12.12
gi|115551791|dbj|AK236197.1|	skyblue3	208.17978	14.172236	0.9518147	0.01260497	42.354
gnl|UG|Ssc#S26711650	purple	207.91076	49.507759	0.9552037	0.01130464	59.554
gnl|UG|Ssc#S5999899	magenta	207.84474	28.740598	0.9509063	0.01296127	18.14
gnl|UG|Ssc#S40440480	darkgreen	207.79721	17.099502	-0.548348	0.33858836	290.866
gnl|UG|Ssc#S19542525	darkgrey	207.69376	15.872916	0.9802263	0.00332793	80.286
gi|61696637|gb|AY803100.1|	brown	207.5662	68.544424	0.8928915	0.04139673	6.514
gnl|UG|Ssc#S17525225	salmon	207.43738	35.164795	0.9347629	0.0198052	14.372
gi|52352717|gb|AY609637.1|	skyblue3	207.42689	17.299355	0.993814	0.00058351	30.796

gnl|UG|Ssc#S34524196	orange	207.36738	12.389156	0.9405707	0.01723552	13.162
gnl|UG|Ssc#S23755238	skyblue	207.25626	23.888928	0.9765111	0.00430617	9.062
gi|115548882|dbj|AK235285.1|	grey60	207.20396	15.968834	0.7367815	0.15555029	81.272
gi|115546656|dbj|AK234494.1|	magenta	207.12454	12.197508	0.6524616	0.23269635	36.934
gi|48976064|ref|NM_001001649.2|	greenyellow	207.0383	31.455977	0.9026612	0.03591847	221.056
gnl|UG|Ssc#S26724539	turquoise	207.01468	117.10631	0.4989375	0.39217423	15.076
gnl|UG|Ssc#S39778806	greenyellow	207.01046	29.063212	0.9001174	0.03732095	64.268
gnl|UG|Ssc#S18356252	yellow	206.75322	61.089012	0.9265001	0.02365467	10.054
gi|47523261|ref|NM_213791.1|	sienna3	206.66434	19.078786	0.994954	0.00042996	12.962
gnl|UG|Ssc#S20946380	violet	206.57687	13.280049	0.905675	0.03427932	13.862
gnl|UG|Ssc#S40426329	tan	206.52632	45.928323	0.9948218	0.00044696	34.966
gnl|UG|Ssc#S34513996	navy	206.42552	37.230495	0.9329747	0.02061946	22.128
gi|52351167|gb|AY609572.1|	blue	206.39945	103.08517	0.841988	0.07358644	99.686
gnl|UG|Ssc#S6076838	grey60	206.26575	16.336653	0.7805467	0.11926344	19.376
gi|194041162|ref|XM_001927505.1|	skyblue	206.21159	23.705734	0.9757137	0.00452677	39.758
gi|194040341|ref|XM_001928820.1|	blue	206.12496	94.207236	0.8264621	0.08448594	53.054
gnl|UG|Ssc#S18379693	skyblue3	206.09889	17.113944	0.9915449	0.00093209	18.808
gi|194038585|ref|XM_001926711.1|	skyblue3	206.07496	17.521989	0.997176	0.00018007	494.478
gnl|UG|Ssc#S17515842	darkgreen	205.82199	8.5537272	0.7143911	0.17516597	23.418
gnl|UG|Ssc#S39793738	lightcyan	205.77127	38.906103	0.9908095	0.0010562	6.218
gi|115553038|dbj|AK239624.1|	blue	205.72091	106.98222	0.8530351	0.06612125	245.668
gi|115554899|dbj|AK233711.1|	skyblue3	205.59007	15.962353	0.9751186	0.00469375	87.406
gnl|UG|Ssc#S18387794	black	205.57056	20.544142	0.7733049	0.12506859	55.656
gnl|UG|Ssc#S6059254	darkgreen	205.43414	18.702867	0.9430281	0.01618372	29.956
gi|115555249|dbj|AK233864.1|	blue	205.38323	109.13056	0.8558838	0.0642369	26.752
gi|194039316|ref|XM_001929265.1|	pink	205.2339	20.75015	0.8293954	0.08239135	73.968
gnl|UG|Ssc#S31115826	lightcyan	205.2275	33.661142	0.9517664	0.01262382	4.264
gi|194044951|ref|XM_001925471.1|	tan	205.19331	39.755939	0.9585347	0.01007258	47.902
gnl|UG|Ssc#S17517303	greenyellow	205.14307	22.59117	0.8358115	0.07786676	2.396
gi|194042482|ref|XM_001925217.1|	grey	205.12718	13.245318	0.8212237	0.08826645	36.57
gnl|UG|Ssc#S40437556	lightgreen	205.01773	27.350474	0.9682975	0.00674368	9.518
gnl|UG|Ssc#S19543409	red	204.88955	30.183262	0.9123243	0.03075084	15.066
gnl|UG|Ssc#S31101927	darkgreen	204.82415	17.84546	-0.387564	0.51918635	94.564
gi|51592116|ref|NM_001004034.1|	orange	204.77386	13.255041	0.9632907	0.00839637	35.126
gi|52351890|gb|AY610308.1|	tan	204.76884	43.034508	0.980321	0.00330409	56.984
gi|115550130|dbj|AK235609.1|	greenyellow	204.7275	32.350722	0.9132287	0.0302805	6.964
gnl|UG|Ssc#S23689759	blue	204.66902	104.06491	0.8477895	0.06963507	2.65
gnl|UG|Ssc#S40237707	blue	204.60681	85.542426	0.8043763	0.10076113	4.126
gnl|UG|Ssc#S19542900	greenyellow	204.53742	26.426849	0.8791548	0.04950453	5.062
gnl|UG|Ssc#S6072575	lightcyan	204.49515	39.742683	0.9948402	0.00044458	24.318
gnl|UG|Ssc#S26403765	tan	204.2258	37.194343	0.9477089	0.01424097	17.504
gnl|UG|Ssc#S18555355	yellow	204.05107	30.373562	0.7076355	0.18121426	38.982
gi|108796069|ref|NM_001042375.1|	royalblue	204.00643	19.305042	0.9376313	0.01852161	425.82
gi|178056515|ref|NM_001123121.1|	salmon	203.92005	34.66023	0.9259214	0.02393249	57.226
gnl|UG|Ssc#S17510781	lightcyan	203.90975	39.636677	0.9948455	0.00044389	9.012
gnl|UG|Ssc#S40473833	violet	203.84186	16.172695	0.930296	0.02185884	5.38
	greenyellow					

gi|115547414|dbj|AK237795.1|	paleturquois	203.74027	11.334904	0.9189116	0.02737902	83.48
gnl|UG|Ssc#S40141829	sienna3	203.69713	18.598024	0.9901175	0.00117758	360.002
gnl|UG|Ssc#S18546732	navy	203.66032	36.122391	0.9784418	0.00378742	17.412
gi|115547117|dbj|AK231172.1|	violet	203.60013	15.805248	0.9403219	0.01734322	8.534
gnl|UG|Ssc#S40438133	salmon	203.43007	30.522086	0.9034769	0.03547244	17.114
gnl|UG|Ssc#S17525473	skyblue3	203.41685	17.425982	0.9953107	0.00038521	16.206
gi|194034946|ref|XM_001924579.1|	magenta	203.32408	15.795167	-0.009618	0.98775396	34.706
gnl|UG|Ssc#S23689580	sienna3	203.29341	18.341056	0.987378	0.00169904	16.92
gi|115555354|dbj|AK233967.1|	magenta	203.26466	16.049145	0.1437851	0.81755987	22.894
gnl|UG|Ssc#S29971166	midnightblu	203.19643	27.180414	0.9219463	0.0258686	7.958
gnl|UG|Ssc#S18550560	lightgreen	203.19465	24.328384	0.92827	0.02281163	8.804
gnl|UG|Ssc#S26728759	violet	203.00476	18.5915	0.9409438	0.01707446	10.946
gi|115545604|dbj|AK230719.1|	orange	202.92231	13.039753	0.9660194	0.00748091	10.292
gi|115555322|dbj|AK233937.1|	turquoise	202.8641	152.90534	0.5624395	0.323668	28.564
gi|194040689|ref|XM_001927163.1|	black	202.84568	33.541487	0.876063	0.05139158	11.754
gnl|UG|Ssc#S34509910	brown	202.78674	75.105528	0.9140345	0.02986338	12.896
gnl|UG|Ssc#S29970127	greenyellow	202.78276	25.13805	0.8562759	0.06397887	6.59
gi|194039733|ref|XM_001928398.1|	tan	202.74055	44.469604	0.9846329	0.00228149	18.998
gi|171905894|gb|EU561660.1|	yellow	202.69268	65.680527	0.962721	0.00859186	347.164
gnl|UG|Ssc#S40442316	red	202.5214	32.051456	0.9482077	0.01403875	75.164
gi|115549624|dbj|AK238432.1|	tan	202.39306	45.239793	0.990784	0.0010606	8.876
gnl|UG|Ssc#S23764143	tan	202.28776	38.729593	0.9560003	0.01100577	13.762
gnl|UG|Ssc#S17526193	lightcyan	202.03768	39.129125	0.9921959	0.00082663	52.596
gi|47522709|ref|NM_213885.1|	sienna3	201.92049	17.618699	0.9785746	0.00375256	10.516
gi|115546138|dbj|AK240376.1|	red	201.87814	33.542743	0.9829012	0.00267711	246.25
gnl|UG|Ssc#S22273127	tan	201.86542	45.098961	0.988158	0.00154419	0.796
gnl|UG|Ssc#S16517655	tan	201.85794	43.610468	0.9831339	0.00262274	30.486
gnl|UG|Ssc#S17511887	blue	201.7957	104.53763	0.8456994	0.07105085	14.268
gnl|UG|Ssc#S18549337	midnightblu	201.61786	27.076265	0.920842	0.02641504	203.516
gnl|UG|Ssc#S17510525	yellow	201.57085	57.391048	0.9247669	0.02448978	65.004
gnl|UG|Ssc#S17514325	pink	201.5435	22.466465	-0.9131	0.03034738	34.51
gnl|UG|Ssc#S19540314	grey	201.52064	12.927563	0.851826	0.06692617	46.296
gnl|UG|Ssc#S26725945	yellow	201.50963	51.811106	0.8827584	0.04733341	16.034
gi|167908788|ref|NM_001114675.1|	navy	201.46894	39.452617	0.9771462	0.0041331	63.268
gi|55741808|ref|NM_213748.1|	sienna3	201.42825	17.914691	0.9821206	0.00286218	771.028
gnl|UG|Ssc#S40004911	yellow	201.40308	53.531267	0.8856946	0.04558733	12.948
gi|47522773|ref|NM_213973.1|	black	201.39185	32.195232	0.8633737	0.05936473	38.376
gnl|UG|Ssc#S26720426	tan	201.29357	44.978756	0.9876033	0.00165381	84.368
gnl|UG|Ssc#S40149996	blue	201.27242	114.21846	0.8714156	0.05426972	9.89
gnl|UG|Ssc#S40034722	pink	201.19432	20.332382	-0.905394	0.03443095	170.012
gi|52351430|gb|AY609860.1|	tan	201.12799	45.032496	0.9910565	0.00101395	183.848
gnl|UG|Ssc#S40082625	turquoise	201.07932	165.57015	0.591585	0.29336204	3.414
gi|194035737|ref|XM_001928295.1|	lightgreen	201.03126	23.739603	0.9245348	0.02460228	51.598
gi|194036623|ref|XM_001927665.1|	tan	200.94866	43.308234	0.9778881	0.00393395	40.546
gi|194044506|ref|XM_001927374.1|	sienna3	200.90905	19.019109	0.9949048	0.00043626	40.372
gi|52351402|gb|AY609832.1|	lightcyan	200.81505	35.390871	0.966871	0.00720238	8.936
gnl|UG|Ssc#S18271608	blue	200.80705	104.26159	0.8465586	0.07046775	16.276
gi|52351862|gb|AY610280.1|	sienna3	200.72049	16.809403	0.9684525	0.00669446	8.34

gnl|UG|Ssc#S39838237	grey	200.41397	12.774548	0.8546886	0.06502543	9.218
gnl|UG|Ssc#S40438408	lightgreen	200.41325	28.638987	0.9905673	0.00109818	25.108
gnl|UG|Ssc#S18284609	brown	200.40393	64.227011	0.879612	0.04922734	5.858
gi|194041636|ref|XM_001929009.1|	navy	200.35825	39.328926	0.9905168	0.001107	67.848
gnl|UG|Ssc#S23689697	sienna3	200.32865	17.537557	0.9773896	0.0040674	11.668
gnl|UG|Ssc#S22275000	lightgreen	200.2981	28.560923	0.9918422	0.0008834	23.974
gi|115553700|dbj|AK239887.1|	lightgreen	200.26725	27.497905	0.9853567	0.00212245	27.32
gi|194039314|ref|XM_001926691.1|	lightcyan	200.21907	36.268844	0.9741723	0.00496333	15.644
gnl|UG|Ssc#S35723183	tan	200.18841	41.023733	0.9689588	0.00653446	73.826
gi|47522821|ref|NM_213998.1|	navy	200.01922	37.012928	0.9768514	0.00421314	8.436
gi|178056662|ref|NM_001123124.1|	tan	199.90175	42.371198	0.9724393	0.00546972	52.698
gnl|UG|Ssc#S40052404	midnightblu	199.79568	26.704787	0.9165334	0.02858182	29.116
gi|194042396|ref|XM_001927868.1|	skyblue	199.57999	21.043247	0.9493829	0.01356607	38.41
gi|113205597|ref|NM_001044537.1|	yellow	199.53673	58.325157	0.9208665	0.02640287	16.066
gi|47522797|ref|NM_213986.1|	greenyellow	199.42758	30.263401	0.8980836	0.03845447	5.342
gnl|UG|Ssc#S40509095	blue	199.34818	98.953549	0.832764	0.08000602	4.254
gi|115554588|dbj|AK236998.1|	darkgreen	199.27653	17.753474	-0.375221	0.53371322	116.708
gi|68534989|ref|NM_001025224.1|	navy	199.22741	37.221235	0.9287576	0.02258114	40.634
gnl|UG|Ssc#S40463925	sienna3	199.2135	19.173667	0.99639	0.00026023	14.326
gnl|UG|Ssc#S18558114	yellow	199.12411	54.329141	0.8877725	0.04436426	14.742
gnl|UG|Ssc#S18276175	navy	199.06078	36.905477	0.9773121	0.00408829	11.772
gi|47523785|ref|NM_214364.1|	red	198.84508	21.147475	0.7206486	0.16961645	50.642
gi|115554840|dbj|AK233652.1|	violet	198.7736	11.43873	-0.979102	0.00361504	37.32
gi|115545783|dbj|AK234216.1|	tan	198.64702	37.575693	0.9492577	0.01361621	6.172
gi|52351109|gb|AY609514.1|	magenta	198.52925	15.996616	0.0461922	0.94120717	44.634
gnl|UG|Ssc#S35322342	sienna3	198.25567	19.126949	0.9962062	0.00028035	16.984
gnl|UG|Ssc#S18262357	skyblue3	198.1767	15.833205	0.9737453	0.0050866	16.472
gnl|UG|Ssc#S22278936	skyblue3	198.16687	15.16829	0.9637045	0.00825534	29.478
gi|194042749|ref|XM_001925080.1|	skyblue3	198.12803	16.589602	0.9851632	0.00216459	112.698
gi|194018717|ref|NM_001129954.1|	darkmagent	198.00923	14.585752	0.9728041	0.00536177	2508.122
gi|115547045|dbj|AK231100.1|	turquoise	197.91196	137.05052	0.5656078	0.3203369	10.746
gi|106073315|gb|DQ508264.1|	yellowgreer	197.68013	11.070627	0.9006546	0.03702332	7.02
gi|115545681|dbj|AK230796.1|	lightgreen	197.67592	24.117777	0.9199879	0.02684015	37.17
gi|48675952|ref|NM_001001643.1|	darkgrey	197.60709	6.463167	0.5792117	0.30613439	132.738
gi|52351594|gb|AY610024.1|	tan	197.54452	36.725036	0.9386778	0.01806031	6.554
gi|52351103|gb|AY609508.1|	orange	197.51992	13.264332	0.94189	0.01666818	27.312
gnl|UG|Ssc#S35323677	darkgrey	197.39504	15.527911	0.9878541	0.00160394	18.978
gnl|UG|Ssc#S23693410	darkgreen	197.26545	18.013347	-0.351036	0.56240434	109.948
gi|194041758|ref|XM_001924615.1|	grey	197.25486	13.212418	0.8265311	0.08443647	25.616
gnl|UG|Ssc#S27602398	grey	197.20256	12.986997	0.8660376	0.05766114	19.366
gnl|UG|Ssc#S40416565	pink	197.19521	20.076524	-0.952104	0.01249235	13.78
gnl|UG|Ssc#S6056024	grey60	197.01087	15.844735	0.9521673	0.01246751	24.724
gi|157427704|ref|NM_001105288.1|	red	196.82284	27.548194	0.8579291	0.06289453	8.182
gnl|UG|Ssc#S34518426	yellow	196.77328	54.813771	0.8969648	0.03908261	32.45
gnl|UG|Ssc#S18355989	tan	196.67135	42.963002	0.9761938	0.0043935	22.842
gi|115552428|dbj|AK236433.1|	brown	196.54973	61.2375	0.8660165	0.05767456	80.75
	greenyellow					

gnl|UG|Ssc#S40022499	lightcyan	196.38272	35.673016	0.9664777	0.00733059	2.064
gi|115554174|dbj|AK239969.1|	turquoise	196.29682	151.34757	0.5601645	0.32606526	22.32
gnl|UG|Ssc#S17510408	tan	196.06141	36.050404	0.9347548	0.01980891	35.72
gnl|UG|Ssc#S31101791	lightcyan	196.02963	38.14977	0.9847239	0.00226129	1.252
gi|47522921|ref|NM_214053.1|	tan	195.96754	46.187819	0.9941926	0.00053079	18.694
gi|83921642|ref|NM_001038007.1|	grey60	195.93005	17.452562	0.8472915	0.06997161	256.912
gi|115551938|dbj|AK236345.1|	darkgrey	195.83045	13.678342	0.8988149	0.03804564	51.868
gnl|UG|Ssc#S40430442	lightgreen	195.57516	22.735211	0.899787	0.03750437	3.748
gnl|UG|Ssc#S23696693	magenta	195.56079	20.425523	0.8738359	0.05276463	11.636
gi|212549654|ref|NM_001137635.1|	tan	195.54245	46.312385	0.9946736	0.00046627	12.948
gnl|UG|Ssc#S19549209	lightgreen	195.48984	19.14769	0.8976372	0.0387047	14.346
gi|15864598|emb|AJ309014.1|	tan	195.46345	40.238952	0.9650385	0.007806	14.43
gnl|UG|Ssc#S18378680	yellow	195.36725	29.01684	0.7177252	0.17220267	97.314
gnl|UG|Ssc#S18377137	skyblue3	195.15374	15.223758	0.9664402	0.00734286	73.502
gi|115553569|dbj|AK239756.1|	lightgreen	195.13403	20.7102	0.8744619	0.05237758	59.98
gnl|UG|Ssc#S23700002	greenyellow	195.01584	28.909959	0.9019124	0.03632953	57.814
gi|194037785|ref|XM_001924937.1|	navy	194.94149	41.802539	0.9686767	0.00662345	5.108
gnl|UG|Ssc#S23695896	salmon	194.8734	19.510941	0.8036397	0.10131878	92.594
gnl|UG|Ssc#S40150666	navy	194.68293	35.97905	0.9850249	0.00219488	8.148
gi|77628017|ref|NM_001008689.2|	paleturquois	194.6556	12.094983	0.9197177	0.02697511	67.544
gnl|UG|Ssc#S31106641	pink	194.61232	19.490555	-0.964077	0.00812906	16.302
gi|113205653|ref|NM_001044551.1|	greenyellow	194.56665	23.376264	0.8351881	0.07830286	12.034
gi|115549368|dbj|AK235390.1|	turquoise	194.41986	143.39533	0.5496121	0.33724267	7.176
gi|115553216|dbj|AK233014.1|	skyblue3	194.40318	16.604772	0.9837707	0.00247584	38.164
gnl|UG|Ssc#S18383839	skyblue3	194.2972	16.302799	0.9821345	0.00285884	35.478
gi|52351075|gb|AY609480.1|	sienna3	194.16198	18.987882	0.9947441	0.00045705	22.592
gnl|UG|Ssc#S17513836	turquoise	194.03985	156.09338	0.5671019	0.31876899	22.414
gi|194037532|ref|XM_001929272.1|	navy	193.97399	40.572236	0.9646385	0.00793985	28.722
gi|115551177|dbj|AK232610.1|	tan	193.95266	38.505152	0.9551875	0.01131074	44.448
gnl|UG|Ssc#S18357321	blue	193.71779	102.20694	0.8440414	0.07218014	15.414
gnl|UG|Ssc#S23766579	violet	193.554	18.346598	0.9733226	0.00520958	30.314
gi|113205623|ref|NM_001044545.1|	yellow	193.35802	62.992901	0.9585537	0.0100657	199.004
gnl|UG|Ssc#S6089318	red	193.34971	24.159304	0.8227702	0.08714508	21.458
gi|48675954|ref|NM_001001644.1|	lightgreen	193.23088	27.517361	0.9899947	0.00119958	26.284
gnl|UG|Ssc#S18379413	tan	193.13212	37.410104	0.948363	0.01397601	3.788
gi|194041134|ref|XM_001928893.1|	darkgrey	192.98281	12.490023	0.9526251	0.01228985	2.488
gi|118403821|ref|NM_001078679.1|	lightcyan	192.96583	37.442312	0.9822335	0.00283515	70.448
gnl|UG|Ssc#S16514775	lightgreen	192.91895	20.855152	0.8943022	0.04059054	31.176
gnl|UG|Ssc#S18557007	red	192.85987	23.414298	0.8011819	0.10318629	10.626
gi|115548658|dbj|AK231642.1|	grey	192.76578	12.84975	0.8245211	0.08588081	100.078
gnl|UG|Ssc#S19548981	tan	192.72982	35.062981	0.9355396	0.01945491	570.218
gnl|UG|Ssc#S26389653	skyblue	192.5179	20.138386	0.938739	0.01803345	6.924
gi|115546953|dbj|AK240599.1|	pink	192.5048	23.986434	0.9197711	0.02694844	24.184
gnl|UG|Ssc#S26648597	turquoise	192.46923	156.29711	0.5643318	0.32167743	20.184
gi|115546324|dbj|AK230843.1|	tan	192.46816	43.462583	0.9824419	0.00278549	94.05
gi|148231659|ref|NM_001097516.1|	purple	192.42249	44.985977	0.9350293	0.01968482	1.39
gi|194040280|ref|XM_001925339.1|	yellow	192.37329	60.255757	0.9370576	0.0187761	247.244
gnl|UG|Ssc#S31102306	violet	192.34422	12.692841	-0.980629	0.00322695	18.7

gnl|UG|Ssc#S19540934	darkmagent	191.97623	15.575106	0.9889996	0.00138271	13.832
gi|115546439|dbj|AK230958.1|	yellow	191.91566	52.473761	0.9017992	0.03639179	21.478
gi|113205811|ref|NM_001044593.1|	paleturquois	191.34198	10.585045	0.8733946	0.05303807	255.88
gi|194036925|ref|XM_001925464.1|	grey	191.24005	13.123772	0.8716516	0.05412236	26.46
gnl|UG|Ssc#S40452557	red	191.14531	24.805783	0.77447	0.12412907	27.274
gnl|UG|Ssc#S40524913	red	191.14157	31.545712	0.9250841	0.02433621	224.486
gi|51592146|ref|NM_001004050.1|	orange	191.11538	13.084015	0.9340368	0.02013456	19.992
gi|115552623|dbj|AK232828.1|	skyblue3	191.03792	13.817214	0.9415474	0.01681492	4.258
gi|115550042|dbj|AK232277.1|	yellow	190.95441	47.033077	0.8429291	0.07294086	103.692
gnl|UG|Ssc#S43495814	pink	190.74008	19.137742	-0.957971	0.01027775	5.454
gi|194034776|ref|XM_001928952.1|	tan	190.65099	43.859901	0.9822085	0.00284113	65.866
gi|90017493|ref|NM_214184.2|	blue	190.58635	117.89523	0.8773807	0.05058462	341.618
gnl|UG|Ssc#S17518385	pink	190.57461	21.973619	-0.907945	0.03306102	1.658
gnl|UG|Ssc#S39935005	darkgreen	190.56158	15.645559	-0.319771	0.59990359	17.396
gnl|UG|Ssc#S27602719	tan	190.53024	45.468499	0.9913834	0.0009589	15.562
gnl|UG|Ssc#S40012596	darkgreen	190.37221	17.503112	-0.383983	0.52339281	8.502
gnl|UG|Ssc#S18383139	lightgreen	190.35903	27.302621	0.9901451	0.00117266	6.446
gi|115547985|dbj|AK237969.1|	yellow	190.17434	39.430192	0.8077792	0.09819699	5.624
gi|148235350|ref|NM_001097475.1|	turquoise	190.1677	153.43787	0.5521209	0.33457674	37.136
gi|52351148|gb|AY609553.1|	skyblue	190.08333	22.420748	0.9593714	0.00977052	104.5
gnl|UG|Ssc#S40163661	greenyellow	190.05376	23.798757	0.8540939	0.06541891	108.05
gnl|UG|Ssc#S16770187	darkgreen	189.88241	17.038538	-0.437524	0.46124901	82.2
gnl|UG|Ssc#S23756992	magenta	189.71399	16.357941	0.2089308	0.73592935	34.946
gnl|UG|Ssc#S18550420	tan	189.65183	42.406533	0.9759118	0.00447163	49.058
gi|194041796|ref|XM_001928803.1|	turquoise	189.48701	154.46797	0.5961956	0.28863896	27.29
gi|47522681|ref|NM_213901.1|	blue	189.41512	108.1267	0.857876	0.06292932	2.198
gnl|UG|Ssc#S17517598	violet	189.11658	18.560683	0.9770835	0.0041501	8.158
gnl|UG|Ssc#S39779823	yellow	188.97191	56.177885	0.9405319	0.01725233	14.14
gnl|UG|Ssc#S18359292	yellow	188.95357	28.25454	0.7127382	0.17664039	7.16
gnl|UG|Ssc#S40439733	turquoise	188.7673	136.5304	0.5584914	0.32783111	40.994
gi|52350690|gb|AY609406.1|	darkgreen	188.75063	16.938603	-0.305901	0.61667782	5.308
gi|194043669|ref|XM_001924741.1|	blue	188.68431	81.395634	0.7953558	0.10765415	36.884
gnl|UG|Ssc#S31114178	tan	188.5766	40.002182	0.9585737	0.01005845	55.516
gi|115548932|dbj|AK235335.1|	tan	188.56113	44.940765	0.9904673	0.00111568	37.506
gnl|UG|Ssc#S24609641	navy	188.45486	42.468422	0.9715481	0.0057364	10.178
gi|194042477|ref|XM_001926521.1|	navy	188.37557	36.858657	0.9844235	0.0023282	57.16
gnl|UG|Ssc#S26720420	midnightblu	188.3452	23.603758	0.8786466	0.04981315	86.952
gnl|UG|Ssc#S23213478	lightgreen	188.29701	15.84861	0.814378	0.09328251	5.364
gnl|UG|Ssc#S40049981	sienna3	188.21642	18.351336	0.9871526	0.00174469	34.418
gnl|UG|Ssc#S23696616	blue	188.16807	91.013886	0.8151787	0.09269142	213.292
gnl|UG|Ssc#S40247487	sienna3	188.13933	18.908012	0.9936063	0.00061313	29.016
gnl|UG|Ssc#S18359264	turquoise	188.01353	152.82261	0.5581779	0.32816231	32.5
gi|194042833|ref|XM_001929033.1|	tan	188.00008	30.802308	0.9023048	0.03611392	13.58
gi|115549402|dbj|AK235424.1|	tan	187.9817	31.916264	0.9098335	0.0320582	11.832
gi|115549511|dbj|AK235533.1|	pink	187.89491	20.878726	-0.918683	0.02749409	44.316
gi|115554022|dbj|AK233231.1|	navy	187.84313	38.465815	0.9250721	0.02434204	123.292
gnl|UG|Ssc#S23690535	blue	187.74501	97.96503	0.8374435	0.07672851	16.27
gnl|UG|Ssc#S40066476	turquoise	187.67062	122.9232	0.5180681	0.37120127	94.09

gnl|UG|Ssc#S31123459	navy	187.64231	13.925991	-0.594347	0.29052979	17.488
gi|194035108|ref|XM_001926680.1|	navy	187.52776	42.008385	0.9687934	0.00658657	29.818
gnl|UG|Ssc#S23697971	magenta	187.47599	14.017773	-0.094773	0.87951191	38.654
gi|194037021|ref|XM_001926975.1|	grey	187.34226	12.763136	0.8957609	0.03976217	6.44
gi|147903738|ref|NM_001097413.1|	royalblue	187.18039	19.61021	0.9714178	0.00577573	8.064
gnl|UG|Ssc#S19541523	greenyellow	187.05108	22.587438	0.8261527	0.08470778	47.06
gi|115550567|dbj|AK232397.1|	navy	187.00235	42.764261	0.9814961	0.00301314	9.754
gi|115545984|dbj|AK237425.1|	greenyellow	187.00178	28.420096	0.8951417	0.04011315	24.934
gi|194036030|ref|XM_001928495.1|	magenta	186.96129	16.087931	0.183661	0.76747698	62.056
gnl|UG|Ssc#S23700560	lightcyan	186.83912	36.063595	0.9714337	0.00577093	8.686
gi|115554284|dbj|AK240079.1|	turquoise	186.80765	122.81699	0.5011542	0.38973005	28.994
gnl|UG|Ssc#S6072888	navy	186.60296	36.334281	0.9899241	0.00121228	11.72
gnl|UG|Ssc#S34495577	lightgreen	186.30643	17.329136	0.8364936	0.07739038	17.436
gi|48675956|ref|NM_001001645.1|	lightgreen	186.24619	24.5494	0.9586845	0.0100183	73.758
gnl|UG|Ssc#S40407699	red	186.08129	23.473172	0.7811114	0.11881431	31.142
gnl|UG|Ssc#S40464521	lightcyan	185.88635	33.217408	0.954324	0.01163771	21.412
gi|194043908|ref|XM_001927890.1|	tan	185.8111	36.152059	0.9400359	0.01746728	89.858
gnl|UG|Ssc#S6048306	pink	185.75769	21.797792	0.8020726	0.10250831	5.922
gi|194036211|ref|XM_001927870.1|	blue	185.64287	111.33326	0.8644517	0.05867349	1.58
gnl|UG|Ssc#S23696689	lightgreen	185.59114	19.733438	0.8825796	0.0474404	77.724
gnl|UG|Ssc#S19546334	magenta	185.46794	14.776005	0.0221404	0.97181223	32.06
gi|115550345|dbj|AK238610.1|	paleturquois	185.45245	11.784183	0.9062455	0.03397179	20.006
gnl|UG|Ssc#S18380498	magenta	185.21728	15.330826	0.0773402	0.90162567	5.842
gnl|UG|Ssc#S17515316	lightgreen	185.20596	25.116297	0.9651024	0.00778467	121.986
gi|47522855|ref|NM_214017.1|	navy	185.13407	38.531088	0.9724338	0.00547136	15.032
gi|115550943|dbj|AK238804.1|	pink	185.04959	21.167013	-0.94696	0.0145464	93.744
gnl|UG|Ssc#S34497945	yellow	184.91656	56.508983	0.9107235	0.03158908	14.224
gi|178056525|ref|NM_001123147.1|	navy	184.85138	42.129895	0.9939521	0.00056409	11.552
gnl|UG|Ssc#S40055771	lightgreen	184.83014	23.489021	0.9323326	0.02091437	13.998
gi|194034626|ref|XM_001929026.1|	red	184.63	32.43235	0.9437085	0.01589634	56.798
gnl|UG|Ssc#S31101691	magenta	184.46114	14.845288	-0.001464	0.99813629	22.432
gnl|UG|Ssc#S40276387	brown	184.39722	62.128998	0.8718856	0.05397636	5.502
gnl|UG|Ssc#S18550712	pink	184.35727	20.392646	0.7724198	0.1257837	21.084
gnl|UG|Ssc#S18291761	brown	184.27407	51.295286	0.8294738	0.0823356	6.868
gi|194035517|ref|XM_001925462.1|	grey60	184.22985	17.644662	0.8902831	0.04290073	39.19
gnl|UG|Ssc#S18553224	lightgreen	184.04736	21.447406	0.8914953	0.04219968	8.226
gi|115549321|dbj|AK232099.1|	tan	184.00218	28.382846	0.8819055	0.04784446	57.04
gi|47523887|ref|NM_214418.1|	greenyellow	183.53934	23.163933	0.8489064	0.06888214	149.84
gnl|UG|Ssc#S26723327	brown	183.43005	63.6877	0.8735797	0.05292337	16.458
gnl|UG|Ssc#S18381735	grey	183.37487	13.142151	0.8340209	0.07912155	10.508
gnl|UG|Ssc#S18359101	pink	183.29228	16.567604	-0.975138	0.00468839	5.536
gnl|UG|Ssc#S19540289	royalblue	183.20038	20.376649	0.9438004	0.01585762	6.934
gi|115545597|dbj|AK230712.1|	lightgreen	183.19765	20.194974	0.897215	0.03894189	65.402
gi|194041305|ref|XM_001927960.1|	turquoise	183.17969	149.17585	0.5487148	0.33819743	23.102
gnl|UG|Ssc#S26726837	tan	183.15516	31.351568	0.9123116	0.03075747	20.428
gnl|UG|Ssc#S22287785	red	183.1086	31.598848	0.9119357	0.03095368	4.596
gnl|UG|Ssc#S19545927	yellow	182.98148	43.06111	0.8399077	0.07501969	14.018
gi|1912|emb|X07617.1|	darkgreen	182.93644	18.187658	0.9104563	0.03172968	34.448

gnl|UG|Ssc#S23756850	darkmagent	182.73244	14.805943	0.9797652	0.00344477	55.374
gi|194036739|ref|XM_001927255.1|	yellow	182.22983	46.950494	0.842003	0.07357613	77.578
gnl|UG|Ssc#S23775239	yellow	182.01691	56.035417	0.9052065	0.03453249	17.382
gi|52351591|gb|AY610021.1|	magenta	181.98273	23.128477	0.9002465	0.03724937	27.786
gi|115553400|dbj|AK236603.1|	grey	181.94265	12.848681	0.8843983	0.04635563	6.536
gi|115551834|dbj|AK236241.1|	lightgreen	181.65799	17.895877	0.8587979	0.06232701	43.006
gnl|UG|Ssc#S18359711	lightgreen	181.58867	16.841254	0.8272165	0.08394569	17.928
gi|212549656|ref|NM_001137636.1|	tan	181.52075	35.179879	0.9347054	0.01983126	23.322
gnl|UG|Ssc#S19547916	skyblue3	181.51264	13.702288	0.9388743	0.0179741	8.552
gi|115551673|dbj|AK239137.1|	orange	181.48404	12.612211	0.920755	0.02645825	16.15
gi|194042479|ref|XM_001926556.1|	skyblue	180.93403	14.576056	0.8704582	0.0548687	23.838
gnl|UG|Ssc#S23691900	white	180.85453	19.378268	0.9090951	0.0324491	50.754
gi|194039546|ref|XM_001928863.1|	lightcyan	180.75414	34.614842	0.9665973	0.00729154	57.158
gnl|UG|Ssc#S18551688	lightgreen	180.64802	15.536448	0.7995738	0.10441382	30.686
gnl|UG|Ssc#S18383442	yellow	180.51916	52.463862	0.9142477	0.02975333	58.036
gnl|UG|Ssc#S31134054	turquoise	180.44927	161.1939	0.6018119	0.28291296	5.442
gi|52352069|gb|AY610485.1|	yellow	180.42378	53.154792	0.8975901	0.03873116	32.996
gnl|UG|Ssc#S34529539	tan	180.33774	41.820244	0.9750268	0.00471968	18.414
gnl|UG|Ssc#S6665509	navy	180.31605	43.136862	0.9866669	0.00184441	6.676
gnl|UG|Ssc#S29462637	yellowgreen	179.96878	10.030152	0.8809992	0.04838946	2.532
gi|115548942|dbj|AK235345.1|	lightgreen	179.87998	21.006034	0.9064797	0.03384581	28.504
gi|148237120|ref|NM_001097421.1|	pink	179.76811	15.978084	0.7056213	0.18302889	34.622
gnl|UG|Ssc#S18554887	midnightblu	179.69837	23.731424	0.8871135	0.044751	22.488
gi|115548727|dbj|AK231793.1|	pink	179.39793	22.1671	0.9536833	0.01188229	32.84
gi|47522957|ref|NM_214072.1|	yellow	179.2461	45.414377	0.8319528	0.08057845	95.514
gi|115552575|dbj|AK232780.1|	red	179.18806	32.85326	0.9678195	0.00689629	16.154
gi|115549239|dbj|AK231915.1|	yellow	179.13781	31.399953	0.715285	0.17437005	111.924
gi|47523463|ref|NM_214188.1|	brown	179.10423	55.519246	0.8450697	0.07147911	31.082
gi|52351601|gb|AY610031.1|	lightgreen	178.97473	21.674193	0.9134301	0.0301761	4.336
gnl|UG|Ssc#S22282766	lightcyan	178.81511	31.461867	0.9462815	0.01482482	13.72
gnl|UG|Ssc#S15981094	orange	178.56647	11.912924	0.9524905	0.012342	15.678
gi|115546047|dbj|AK237489.1|	darkmagent	178.47958	14.796102	0.974846	0.0047709	10.278
gnl|UG|Ssc#S18554614	navy	178.42335	14.492904	-0.514731	0.37483985	2.938
gnl|UG|Ssc#S19538640	lightcyan	178.393	31.015494	0.9330886	0.02056724	6.988
gnl|UG|Ssc#S26727906	grey60	178.38306	17.062463	0.9207254	0.02647296	16.36
gi|47522763|ref|NM_213968.1|	orange	178.33142	12.672238	0.9434068	0.01602354	234.386
gi|115553814|dbj|AK236819.1|	lightgreen	178.29157	23.177407	0.9259874	0.02390075	45.65
gnl|UG|Ssc#S34533729	royalblue	178.08762	20.398262	0.960982	0.00919756	6.198
gi|194035288|ref|XM_001927095.1|	yellow	178.00472	50.833035	0.8907943	0.04260462	81.224
gnl|UG|Ssc#S18360059	lightcyan	177.90343	33.44037	0.9550698	0.01135513	22.976
gi|115549613|dbj|AK238421.1|	yellow	177.8387	28.437932	0.6684007	0.21745812	31.34
gi|52352038|gb|AY610454.1|	sienna3	177.82496	16.438503	0.9643029	0.00805275	36.16
gi|115550889|dbj|AK235964.1|	tan	177.78183	40.849083	0.9686713	0.00662514	72.51
gnl|UG|Ssc#S23701124	red	177.37212	31.518411	0.9554335	0.01121816	12.068
gi|115547679|dbj|AK231337.1|	navy	177.3645	37.146057	0.9104572	0.03172921	25.824
gnl|UG|Ssc#S40059478	violet	177.08582	15.769127	0.9928355	0.00072718	14.114
	paleturquois					

gnl|UG|Ssc#S19540541	violet	176.53602	13.259197	-0.968096	0.00680779	2.988
gi|115547647|dbj|AK231305.1|	pink	176.52277	23.953452	0.8511621	0.06736941	21.352
gnl|UG|Ssc#S18357219	blue	176.44962	108.65476	0.8606016	0.06115393	19.648
gnl|UG|Ssc#S34521911	yellow	176.35716	26.590274	0.6603735	0.22509677	15.014
gnl|UG|Ssc#S21579717	greenyellow	176.32517	22.636749	0.8367615	0.07720354	17.34
gi|47523843|ref|NM_214395.1|	darkgrey	176.28322	6.3332434	0.5803306	0.30497367	0.894
gi|115554993|dbj|AK240192.1|	paleturquois	176.25755	15.312233	0.9704691	0.0060648	13.056
gnl|UG|Ssc#S40490801	red	175.96742	30.996011	0.9332001	0.02051623	6.584
gi|47523719|ref|NM_214330.1|	darkmagent	175.80915	15.599485	0.988616	0.00145557	125.312
gi|115554528|dbj|AK233538.1|	lightgreen	175.77774	22.246661	0.9237547	0.02498175	12.568
gnl|UG|Ssc#S19542061	darkgreen	175.75778	16.230774	-0.32977	0.58786375	158.654
gnl|UG|Ssc#S17512269	lightgreen	175.75006	23.29288	0.9431101	0.01614898	49.668
gnl|UG|Ssc#S26734441	violet	175.6397	10.267183	-0.922469	0.02561139	39.184
gi|194038088|ref|XM_001925288.1|	lightcyan	175.36023	33.072965	0.9508345	0.01298958	64.2
gnl|UG|Ssc#S18558291	black	175.20012	23.77261	0.7792505	0.12029649	79.164
gi|178056709|ref|NM_001123194.1|	royalblue	174.95726	21.155347	0.9501177	0.01327324	51.162
gnl|UG|Ssc#S31119321	red	174.95351	31.013969	0.9059653	0.03412272	8.61
gi|164664445|ref|NM_001113439.1|	magenta	174.94683	16.295743	0.7709721	0.12695603	76.338
gi|115555475|dbj|AK234088.1|	pink	174.83196	24.269493	0.9388649	0.01797823	29.174
gnl|UG|Ssc#S23689765	paleturquois	174.69132	14.128944	0.9655847	0.00762441	35.96
gnl|UG|Ssc#S18552312	navy	174.4436	42.796415	0.9728057	0.00536132	24.178
gnl|UG|Ssc#S18548755	blue	174.41818	84.997219	0.8081878	0.09789048	26.628
gnl|UG|Ssc#S40195327	turquoise	174.41374	154.61111	0.5922848	0.2926438	56.738
gi|194040558|ref|XM_001928742.1|	lightcyan	174.38141	25.239777	0.8893712	0.04343049	2412.036
gnl|UG|Ssc#S40437142	lightgreen	174.30542	23.321649	0.936702	0.0189344	2.62
gi|194332486|ref|NM_001130213.1|	yellow	174.29626	37.704588	0.8295088	0.08231066	62.604
gi|154147629|ref|NM_001100190.1|	salmon	174.18285	19.199774	0.8066501	0.09904557	58.338
gnl|UG|Ssc#S34512833	royalblue	174.15523	22.112986	0.9788599	0.00367802	29.922
gnl|UG|Ssc#S17525080	pink	173.99675	7.050326	-0.500763	0.39016078	22.318
gnl|UG|Ssc#S23692872	greenyellow	173.8004	25.096915	0.8628612	0.05969428	33.016
gnl|UG|Ssc#S6085536	paleturquois	173.60291	13.768095	0.933521	0.02036958	29.996
gi|194041222|ref|XM_001925236.1|	red	173.55189	23.049929	0.7086207	0.18032853	12.928
gnl|UG|Ssc#S22274609	red	173.54772	30.878599	0.8972202	0.03893897	11.798
gnl|UG|Ssc#S18552418	tan	173.4788	32.14887	0.915268	0.02922854	8.242
gnl|UG|Ssc#S17516571	magenta	173.36024	9.7004839	0.5792327	0.30611266	9.348
gnl|UG|Ssc#S34529144	tan	173.1673	43.163586	0.9776342	0.00400174	9.882
gnl|UG|Ssc#S26720632	darkmagent	172.97894	9.9327657	0.8804087	0.04874556	26.246
gnl|UG|Ssc#S23768582	lightgreen	172.85158	17.657213	0.8657926	0.05781714	34.74
gi|194038017|ref|XM_001928309.1|	blue	172.73771	94.739554	0.8308578	0.08135318	14.29
gi|194037543|ref|XM_001929293.1|	darkmagent	172.5665	14.112615	0.962706	0.00859703	45.09
gnl|UG|Ssc#S17525025	navy	172.43893	13.952654	-0.691555	0.19584336	10.782
gnl|UG|Ssc#S26408370	greenyellow	172.42417	17.408348	0.7609989	0.13511904	11.406
gi|157427710|ref|NM_001105292.1|	turquoise	172.42247	96.042495	0.4666074	0.42822066	23.182
gi|194037684|ref|XM_001927128.1|	darkgreen	172.41744	15.984271	-0.280498	0.64759949	60.958
gi|115545868|dbj|AK234301.1|	navy	172.35938	42.658099	0.9808615	0.00316916	24.518
gi|166796054|ref|NM_001114281.1|	darkgreen	172.28343	6.9652589	0.6276486	0.25696831	19.712
gnl|UG|Ssc#S40127760	pink	172.1779	10.158765	0.4886427	0.40357241	8.884
gnl|UG|Ssc#S23763873	pink	171.88816	21.903403	0.9214544	0.02611156	14.282

gnl|UG|Ssc#S17524628	royalblue	171.69492	19.655141	0.9647197	0.00791264	4.236
gnl|UG|Ssc#S23695762	yellow	171.52383	45.526489	0.8375726	0.07663872	20.406
gi|1245714|gb|S80644.1|	magenta	171.34914	19.809603	0.8571833	0.06338297	30.716
gi|47523691|ref|NM_214313.1|	skyblue3	171.26498	13.143773	0.9319975	0.02106886	26.474
gnl|UG|Ssc#S23693357	darkgrey	171.06364	6.1986247	0.4994198	0.39164214	50.878
gnl|UG|Ssc#S40326987	turquoise	170.91826	110.87639	0.4824437	0.41047234	38.706
gi|194035207|ref|XM_001925835.1|	yellow	170.84341	38.446939	0.7813714	0.1186076	37.614
gnl|UG|Ssc#S31125151	skyblue	170.80748	18.804276	0.9178274	0.0279253	57.942
gnl|UG|Ssc#S35325457	red	170.61448	30.406505	0.8733813	0.05304635	6.118
gi|47522753|ref|NM_213962.1|	greenyellow	170.61043	20.905666	0.8078774	0.09812334	95.5
gnl|UG|Ssc#S23766475	paleturquois	170.59382	15.467605	0.9672321	0.00708535	60.514
gnl|UG|Ssc#S40495537	navy	170.42845	34.982871	0.990572	0.00109735	122.606
gi|115545865|dbj|AK234298.1|	darkgreen	170.16344	15.985807	0.8118094	0.09518638	11.812
gnl|UG|Ssc#S17518692	skyblue	170.09378	19.49924	0.9279202	0.02297749	8.342
gnl|UG|Ssc#S23766544	pink	170.05899	23.330422	0.8725587	0.0535572	23.09
gnl|UG|Ssc#S39808414	pink	169.57702	8.7811815	0.359106	0.55279862	1.484
gnl|UG|Ssc#S22283855	tan	169.25354	23.04662	0.8335682	0.0794397	11.632
gnl|UG|Ssc#S40063653	pink	169.24436	7.5420904	-0.535184	0.35267566	1.42
gnl|UG|Ssc#S22275237	turquoise	169.22777	118.61614	0.5071057	0.3831857	5.678
gi|194039490|ref|XM_001927338.1|	navy	169.17085	36.61099	0.9574273	0.01047702	81.926
gnl|UG|Ssc#S18550348	greenyellow	168.96988	20.66655	0.8001479	0.1039751	8.486
gi|115551411|dbj|AK236089.1|	black	168.71714	25.195003	0.7995765	0.10441172	7.524
gi|6007613|gb|AF120099.1|AF120099	red	168.68194	34.071336	0.9865154	0.0018759	21.608
gi|194042486|ref|XM_001927572.1|	salmon	168.24574	10.894962	0.679436	0.20707769	27.318
gnl|UG|Ssc#S23772402	red	168.22574	35.019154	0.9970139	0.0001958	87.652
gnl|UG|Ssc#S6009554	tan	168.16149	41.470164	0.9706342	0.00601415	10.58
gi|194034220|ref|XM_001928748.1|	navy	167.17854	34.037486	0.8798863	0.04906128	33.068
gi|194040939|ref|XM_001924716.1|	darkmagent	167.09799	8.9770393	0.8564873	0.06383994	62.394
gi|194033502|ref|XM_001926129.1|	pink	166.78875	18.48912	-0.801603	0.10286593	29.226
gi|194034959|ref|XM_001927036.1|	tan	166.78641	35.35966	0.9274138	0.02321823	413.974
gnl|UG|Ssc#S18262171	navy	166.73569	16.307454	-0.561155	0.32502132	11.008
gnl|UG|Ssc#S26733681	sienna3	166.66688	15.865067	0.9567953	0.01071014	23.25
gnl|UG|Ssc#S17514217	yellow	166.62817	45.934535	0.8512915	0.06728291	19.2
gnl|UG|Ssc#S23767637	yellow	166.49763	33.377472	0.7400582	0.15273688	51.076
gi|194037874|ref|XM_001925758.1|	lightcyan	166.47397	29.601505	0.926443	0.02368202	9.462
gnl|UG|Ssc#S40406739	darkgreen	166.20857	6.1713855	0.8752819	0.05187186	39.55
gi|35384837|gb|AY374470.1|	darkmagent	166.15761	13.137367	0.9447363	0.01546536	21.354
gnl|UG|Ssc#S40443068	pink	165.84372	17.545706	-0.816779	0.09151382	166.136
gnl|UG|Ssc#S18550127	pink	165.82193	16.827211	-0.892016	0.04189948	68.988
gi|51592110|ref|NM_001004031.1|	lightgreen	165.54219	18.21239	0.865478	0.05801769	59.232
gi|115555188|dbj|AK233803.1|	turquoise	165.45351	140.42814	0.5631927	0.32287528	13.502
gi|47522915|ref|NM_214050.1|	sienna3	165.35654	16.187313	0.9623663	0.0087143	10.77
gi|115546241|dbj|AK240479.1|	darkgrey	165.33315	6.2424651	0.5172832	0.3720563	94.782
gnl|UG|Ssc#S18274130	tan	165.24651	33.919894	0.9259404	0.02392333	13.17
gi|115549970|dbj|AK232205.1|	greenyellow	165.2133	23.598242	0.8423785	0.07331838	13.668
gnl|UG|Ssc#S22314469	red	165.04384	28.294258	0.8586201	0.06244307	11.05
gi|194040784|ref|XM_001927731.1|	grey	165.02823	10.939476	0.8036603	0.10130316	34.342
gi|115552057|dbj|AK239250.1|	lightcyan	164.71218	31.055155	0.9398947	0.01752864	22.17

gnl|UG|Ssc#S16513921	magenta	164.62703	22.572171	0.8995841	0.03761711	11.83
gi|187672044|gb|EU650276.1|	red	164.48272	31.350801	0.8909579	0.04251	9.384
gnl|UG|Ssc#S40008864	darkgreen	164.42626	16.475289	0.8893102	0.04346601	7.188
gnl|UG|Ssc#S17518795	turquoise	164.37745	129.5381	0.5227932	0.36606402	42.836
gi|157427714|ref|NM_001105294.1|	sienna3	164.3751	16.13127	0.9607609	0.00927555	29.106
gi|51592104|ref|NM_001004027.1|	yellow	164.34807	39.947467	0.8135911	0.09386456	18.744
gnl|UG|Ssc#S19545599	yellow	164.33432	33.75355	0.7760057	0.12289394	26.442
gnl|UG|Ssc#S22288091	lightcyan	163.78207	27.728914	0.9089351	0.03253399	9.364
gi|115546712|dbj|AK237558.1|	red	163.58204	28.008705	0.7953924	0.1076259	173.392
gnl|UG|Ssc#S29990179	navy	163.37231	40.754664	0.9571472	0.01058013	30.374
gnl|UG|Ssc#S26715792	midnightblu	163.35577	21.842421	0.85917	0.06208448	33.734
gnl|UG|Ssc#S19544906	pink	163.34342	17.754097	-0.819754	0.08933614	37.514
gi|115552412|dbj|AK236417.1|	lightcyan	163.33875	27.491698	0.9034531	0.0354854	11.552
gnl|UG|Ssc#S26404602	yellow	163.28461	38.654998	0.8216432	0.08796184	7.006
gnl|UG|Ssc#S18552383	turquoise	163.2763	105.28781	0.4630118	0.43227411	26.184
gnl|UG|Ssc#S40054185	lightgreen	163.03339	20.822129	0.8999639	0.03740612	61.028
gnl|UG|Ssc#S40367565	yellow	163.01133	50.219287	0.8837848	0.04672064	15.834
gnl|UG|Ssc#S35324071	darkmagent	163.00656	11.944226	0.9231135	0.02529508	3.978
gnl|UG|Ssc#S17517764	yellow	162.94235	18.205685	0.4906009	0.40139836	3.204
gnl|UG|Ssc#S26728677	turquoise	162.46873	110.64541	0.518596	0.37062647	10.032
gnl|UG|Ssc#S34530397	red	162.13184	25.934485	0.8624108	0.05998432	23.71
gi|52351277|gb|AY609709.1|	grey60	161.87237	13.450919	0.6048372	0.27984103	45.64
gnl|UG|Ssc#S19540474	brown	161.83406	53.503946	0.8326834	0.08006285	8.83
gnl|UG|Ssc#S23763649	red	161.54378	34.005077	0.9333039	0.02046875	21.3
gnl|UG|Ssc#S19543384	lightgreen	161.44954	21.39004	0.9180511	0.02781231	35.286
gnl|UG|Ssc#S40327511	red	161.01092	26.146907	0.7864072	0.11462658	48.91
gnl|UG|Ssc#S18549419	lightcyan	160.90818	28.929151	0.920952	0.02636043	11.13
gi|115548360|dbj|AK231733.1|	navy	160.79983	35.621145	0.9538221	0.01182915	24.714
gi|47523847|ref|NM_214397.1|	royalblue	160.76985	21.11567	0.9515232	0.01271895	43.442
gi|115554309|dbj|AK240104.1|	violet	160.6887	17.504514	0.9503044	0.01319918	10.496
gnl|UG|Ssc#S34529832	turquoise	160.5508	95.703126	0.4694848	0.42498312	3.966
gi|52351225|gb|AY609657.1|	navy	160.25903	15.499222	-0.461851	0.43358492	23.742
gnl|UG|Ssc#S40205238	pink	160.02302	23.194591	0.9581888	0.01019837	12.948
gi|194036101|ref|XM_001929371.1|	magenta	159.97459	13.741746	0.2091895	0.73560724	34.922
gnl|UG|Ssc#S18550295	paleturquoi:	159.92643	13.195782	0.9078663	0.03310294	10.792
gi|194038225|ref|XM_001926546.1|	lightgreen	159.87236	20.028859	0.8997972	0.03749865	56.846
gi|115548454|dbj|AK235071.1|	yellow	159.47414	22.033794	0.5625017	0.32360246	28.572
gnl|UG|Ssc#S5999567	yellow	159.45995	42.741221	0.8398713	0.07504491	7.672
gnl|UG|Ssc#S40390861	yellow	159.44692	45.610156	0.87212	0.05383028	128.578
gi|52351639|gb|AY610069.1|	brown	158.98105	48.182517	0.8110041	0.09578564	13.468
gnl|UG|Ssc#S40146297	greenyellow	158.97616	21.303304	0.8163243	0.09184782	12.748
gnl|UG|Ssc#S26722585	pink	158.94867	22.441823	0.890552	0.04274489	5.702
gi|115551122|dbj|AK232555.1|	yellow	158.92258	27.110188	0.6699207	0.21601996	27.402
gnl|UG|Ssc#S17525768	darkgrey	158.71363	13.76033	0.9382967	0.01822784	11.212
gnl|UG|Ssc#S40055820	darkgreen	158.68717	15.592619	0.8965654	0.03930765	2.532
gnl|UG|Ssc#S18383368	tan	158.53552	38.866689	0.9542965	0.01164818	3.882
gnl|UG|Ssc#S19542684	navy	158.31805	36.731228	0.906408	0.03388433	93.094
gnl|UG|Ssc#S29987248	skyblue	157.97248	16.703641	0.8920645	0.04187175	7.928

gi|178056483|ref|NM_001123101.1|	darkmagent	157.62258	15.528443	0.9898281	0.00122962	26.608
gnl|UG|Ssc#S23759685	navy	157.52541	15.331862	-0.440928	0.45735595	2.94
gnl|UG|Ssc#S19545759	pink	157.24458	18.867416	0.6827149	0.20402076	9.438
gnl|UG|Ssc#S40349544	lightcyan	157.23158	25.462361	0.8951104	0.04013091	192.828
gnl|UG|Ssc#S18291801	navy	157.21735	39.352058	0.9674453	0.00701654	11.278
gi|115554233|dbj|AK240028.1|	violet	157.14043	12.675955	-0.917166	0.02826009	2.738
gnl|UG|Ssc#S18356019	red	157.01483	32.594312	0.9671606	0.00710847	10.112
gnl|UG|Ssc#S40180694	magenta	156.84563	16.995382	0.7981237	0.10552442	15.072
gnl|UG|Ssc#S35325248	navy	156.70664	36.880778	0.9631936	0.00842961	22.296
gnl|UG|Ssc#S23763174	sienna3	156.63855	15.939094	0.9588947	0.00994225	13.87
gnl|UG|Ssc#S17516064	magenta	156.62403	10.711862	0.589484	0.29552081	30.766
gi|194036068|ref|XM_001926844.1|	lightcyan	156.56164	25.364494	0.8897704	0.04319832	63.29
gi|115547198|dbj|AK234571.1|	blue	156.49053	87.694106	0.8143389	0.09331142	11.354
gnl|UG|Ssc#S40291735	navy	156.32851	40.235233	0.9673121	0.00705951	11.19
gnl|UG|Ssc#S29982058	navy	156.30629	35.829449	0.9896729	0.00125785	18.65
gi|115549177|dbj|AK231853.1|	royalblue	156.27985	15.770119	0.9134758	0.03015238	11.384
gnl|UG|Ssc#S17514632	navy	156.10074	35.103254	0.8909352	0.04252313	36.276
gnl|UG|Ssc#S23767117	magenta	155.98087	7.7971781	0.4269243	0.47341931	4.784
gnl|UG|Ssc#S40100454	brown	155.97481	40.16546	0.7763448	0.12262173	12.68
gi|194041741|ref|XM_001928824.1|	red	155.66924	28.313833	0.7864574	0.11458712	14.572
gnl|UG|Ssc#S40106217	red	155.63987	35.070613	0.9952035	0.00039848	11.25
gi|194036992|ref|XM_001928798.1|	violet	155.6348	16.883753	0.9311886	0.02144324	9.572
gnl|UG|Ssc#S6013604	blue	155.52547	78.662988	0.7899148	0.11187793	4.126
gi|194042865|ref|XM_001925507.1|	royalblue	155.5033	18.62592	0.9168711	0.02841	24.508
gi|115555020|dbj|AK240219.1|	magenta	155.42184	4.904854	0.4140633	0.48827618	45.158
gi|148222590|ref|NM_001097486.1|	royalblue	155.28353	20.190171	0.9354435	0.01949813	17.568
gi|115551176|dbj|AK232609.1|	violet	155.11453	9.6275023	0.5515273	0.33520702	20.536
gi|115549258|dbj|AK235178.1|	navy	155.06616	30.309001	0.8784248	0.04994803	94.478
gi|194035424|ref|XM_001926628.1|	navy	155.03134	16.983305	-0.556428	0.33001239	45.6
gnl|UG|Ssc#S26735315	red	154.98299	28.601939	0.8272711	0.08390666	6.114
gi|52351978|gb|AY610394.1|	royalblue	154.98156	20.054774	0.9312617	0.0214093	96.522
gnl|UG|Ssc#S31129394	yellow	154.8066	49.759249	0.8939391	0.04079753	198.172
gnl|UG|Ssc#S23695759	yellow	154.35643	39.822957	0.8040534	0.10100549	33.946
gnl|UG|Ssc#S39951768	red	154.24701	31.012818	0.8940568	0.0407304	41.04
gi|198282078|ref|NM_001134823.1|	tan	154.24248	37.099108	0.942734	0.01630847	1589.518
gi|194036350|ref|XM_001926399.1|	yellow	154.07859	43.040252	0.8377794	0.07649485	12.4
gnl|UG|Ssc#S18380107	navy	153.85067	37.929187	0.9197016	0.02698317	21.132
gnl|UG|Ssc#S26723763	darkmagent	153.5934	16.074577	0.9952434	0.00039352	22.38
gnl|UG|Ssc#S40482663	magenta	153.57992	11.842283	0.0433571	0.94481329	9.268
gi|158262678|ref|NM_001109946.1|	pink	153.28398	22.343944	0.9754676	0.00459557	7.52
gnl|UG|Ssc#S40175618	royalblue	153.21772	22.443651	0.9728794	0.0053396	3.508
gi|194036295|ref|XM_001926489.1|	darkgrey	152.84199	13.13099	0.899569	0.03762549	34.484
gi|194036270|ref|XM_001929667.1|	navy	152.83561	35.966152	0.8974145	0.03882975	51.712
gnl|UG|Ssc#S23694970	lightgreen	152.71114	14.193302	0.7795241	0.1200782	56.806
gnl|UG|Ssc#S19542749	royalblue	152.66073	22.496297	0.9911971	0.00099015	69.196
gi|115547369|dbj|AK234742.1|	darkgreen	152.55689	6.0157183	0.8073249	0.09853813	20.426
gi|115551374|dbj|AK236052.1|	yellow	152.28013	18.15065	0.4769844	0.41657127	89.27
gi|115553967|dbj|AK233176.1|	navy	152.24621	34.012352	0.9693441	0.00641353	50.592

gnl|UG|Ssc#S17499681	royalblue	152.06684	21.168986	0.9540377	0.01174682	150.068
gi|115550656|dbj|AK232486.1|	navy	151.86453	27.145518	0.8764231	0.05117068	23.734
gi|115548136|dbj|AK238120.1|	navy	151.74007	16.08703	-0.400477	0.50407455	0.974
gi|52351682|gb|AY610112.1|	grey	151.43946	11.46641	0.8669562	0.05707729	21.064
gi|194036737|ref|XM_001927243.1|	magenta	151.27254	13.513391	0.6684221	0.21743788	50.968
gi|194042725|ref|XM_001925659.1|	violet	151.17237	13.416626	-0.915256	0.02923453	27.22
gi|194042819|ref|XM_001928170.1|	navy	150.95629	40.088253	0.9728912	0.00533612	26.894
gi|84619523|ref|NM_001038694.1|	brown	150.90219	49.657787	0.816719	0.09155763	13.34
gi|178056676|ref|NM_001123078.1|	pink	150.79968	23.65828	0.8220275	0.08768306	191.35
gnl|UG|Ssc#S17525410	violet	150.75093	14.890521	0.9862519	0.00193109	22.47
gi|194035479|ref|XM_001924978.1|	paleturquois	150.63435	16.819365	0.9963214	0.00026768	18.376
gi|115553202|dbj|AK233000.1|	navy	150.62704	39.379565	0.9591569	0.00984765	73.798
gnl|UG|Ssc#S18550520	darkmagent	150.51586	13.190022	0.9527123	0.01225608	40.048
gnl|UG|Ssc#S18380006	lightcyan	150.2819	24.588345	0.8820029	0.04778602	57.176
gnl|UG|Ssc#S35323260	red	150.13926	29.535486	0.8606662	0.06111208	13.976
gnl|UG|Ssc#S40288024	pink	149.98575	13.730172	0.9553845	0.01123658	12.448
gi|115548140|dbj|AK238124.1|	lightcyan	149.92296	26.510858	0.9090535	0.03247115	10.012
gi|194037707|ref|XM_001924251.1|	darkgreen	149.67678	6.4100703	0.830551	0.08157062	7.638
gi|194043959|ref|XM_001927448.1|	lightcyan	149.62876	23.729225	0.8728975	0.0533466	44.288
gnl|UG|Ssc#S18546252	lightgreen	149.511	13.584533	0.7667954	0.13035622	26.042
gnl|UG|Ssc#S19539691	royalblue	149.51034	14.969642	0.913138	0.03032756	23.814
gi|115550124|dbj|AK235603.1|	violet	149.42698	16.685508	0.9205402	0.02656502	144.472
gi|194036658|ref|XM_001926004.1|	orange	149.17445	6.2049955	0.6828993	0.20384925	30.742
gnl|UG|Ssc#S26728925	pink	148.99045	8.0427655	0.089726	0.88591085	10.51
gnl|UG|Ssc#S19542771	tan	148.7332	35.597434	0.9313955	0.02134726	27.482
gi|52351064|gb|AY609469.1|	navy	148.68222	36.610803	0.9271378	0.0233498	10.076
gnl|UG|Ssc#S23689699	paleturquois	148.55095	16.399822	0.9805839	0.00323824	7.204
gnl|UG|Ssc#S38481918	grey60	148.51774	12.392541	0.5821086	0.30313155	39.374
gnl|UG|Ssc#S40171668	paleturquois	148.51446	14.450718	0.9308349	0.0216076	37.444
gnl|UG|Ssc#S26403713	navy	148.28622	17.056558	-0.558428	0.32789824	16.016
gnl|UG|Ssc#S40184013	lightcyan	147.83866	24.714043	0.8837977	0.04671295	99.746
gi|115546260|dbj|AK240498.1|	yellow	147.64248	31.78501	0.7041727	0.18433716	30.354
gnl|UG|Ssc#S23758881	royalblue	147.63075	19.575636	0.9451963	0.01527375	47.948
gnl|UG|Ssc#S16764113	skyblue	147.19392	9.7214406	0.7807436	0.11910679	63.736
gnl|UG|Ssc#S35336616	violet	147.18226	12.655607	-0.895454	0.03993585	5.748
gi|72535201|ref|NM_001031794.1|	red	147.18094	35.333068	0.9542351	0.01167155	1398.636
gi|194033632|ref|XM_001927932.1|	pink	147.08001	21.838779	0.8544234	0.06520083	9.964
gnl|UG|Ssc#S17514318	royalblue	147.06798	20.467027	0.9823131	0.00281615	34.012
gi|52351540|gb|AY609970.1|	navy	147.02022	16.84669	-0.593469	0.29143003	7.206
gnl|UG|Ssc#S18284670	red	146.68727	33.340285	0.9824626	0.00278057	8.538
gnl|UG|Ssc#S23761814	red	146.68225	34.485638	0.9883113	0.00151432	25.108
gnl|UG|Ssc#S39833392	magenta	146.42276	20.380866	0.8259371	0.0848625	15.678
gi|92020093|dbj|AB237777.1|	darkgreen	146.38725	2.9338055	0.4936136	0.39805911	18.948
gi|115546695|dbj|AK234533.1|	navy	145.85136	16.15178	-0.345204	0.56936464	76.82
gi|153792026|ref|NM_001099932.1|	royalblue	145.85109	20.681336	0.9473208	0.01439896	338.326
gi|115555653|dbj|AK237286.1|	turquoise	145.81539	120.39307	0.5179051	0.37137871	85.946
gi|194037346|ref|XM_001924466.1|	navy	145.56987	33.547985	0.8977857	0.03862142	21.152
gnl|UG|Ssc#S18555925	magenta	145.25367	15.235133	0.6607065	0.2247785	10.822

gnl|UG|Ssc#S40262955	sienna3	145.104	12.99711	0.9194024	0.02713289	3.106
gi|115546838|dbj|AK237684.1|	royalblue	145.04434	21.134986	0.9719705	0.00560949	74.116
gi|115546460|dbj|AK230979.1|	grey60	144.93656	6.948366	0.2990224	0.6250253	24.852
gi|115548849|dbj|AK235252.1|	lightgreen	144.84898	16.764356	0.8504915	0.067818	14.562
gnl|UG|Ssc#S35331631	navy	144.59453	35.523911	0.980609	0.00323198	6.37
gnl|UG|Ssc#S40386137	violet	144.56924	16.367837	0.9828407	0.00269129	7.156
gi|115554124|dbj|AK233334.1|	darkmagent	143.76638	14.988227	0.9746571	0.0048246	73.648
gi|115550108|dbj|AK235587.1|	grey60	143.67525	6.0866822	0.1667357	0.78869327	12.828
gi|194033910|ref|XM_001928928.1|	turquoise	143.36413	88.847313	0.4431455	0.45482299	40.406
gi|194037185|ref|XM_001929112.1|	darkmagent	143.24999	14.865916	0.9720444	0.00558736	15.082
gnl|UG|Ssc#S23768212	salmon	143.08814	20.285021	0.8045426	0.10063537	171.268
gnl|UG|Ssc#S18282034	midnightblu	142.99589	16.5788	0.7988796	0.104945	24.404
gi|37050908|emb|AJ583708.1|	yellow	142.72432	41.66239	0.8431569	0.07278488	10.196
gi|194035832|ref|XM_001928786.1|	royalblue	142.6728	17.532269	0.895542	0.0398861	38.628
gnl|UG|Ssc#S31121510	tan	142.18794	13.144338	0.7067967	0.18196931	32.112
gnl|UG|Ssc#S18273087	violet	141.73324	14.408573	-0.910035	0.03195164	5.276
gi|115551966|dbj|AK239159.1|	paleturquois	141.48919	12.92147	0.895635	0.03983347	31.702
gnl|UG|Ssc#S18557979	paleturquois	141.1487	14.871827	0.9440745	0.01574241	23.066
gi|194043177|ref|XM_001928608.1|	pink	140.93917	20.267977	0.708399	0.18052781	5.912
gnl|UG|Ssc#S18358120	lightgreen	140.86197	10.228847	0.7382065	0.15432491	47.204
gnl|UG|Ssc#S18378555	navy	140.65537	14.699696	-0.320521	0.59899907	21.13
gi|115554372|dbj|AK233382.1|	magenta	140.30118	18.962016	0.7918573	0.11036435	48.008
gnl|UG|Ssc#S34517367	lightgreen	140.20923	10.398274	0.6918064	0.19561218	120.184
gi|115553549|dbj|AK236752.1|	navy	140.07453	35.491906	0.9219489	0.02586731	47.958
gi|115552242|dbj|AK230551.1|	midnightblu	139.97482	17.979942	0.8143203	0.09332517	16.868
gnl|UG|Ssc#S18378791	darkgreen	139.46361	5.1814243	0.777147	0.12197847	59.824
gi|84490432|ref|NM_001038631.1|	magenta	139.37174	20.594344	0.8530905	0.06608442	4.18
gi|15824733|gb|AF329358.1|AF32935	pink	139.14927	21.160865	0.7376789	0.15477829	14.51
gi|118403911|ref|NM_001078662.1|	pink	139.00273	22.33268	0.9469359	0.0145562	76.34
gnl|UG|Ssc#S17513253	darkgreen	138.91302	12.202411	0.878933	0.04963914	40.784
gnl|UG|Ssc#S40288641	grey60	138.88312	13.328981	0.6506206	0.23447452	6.23
gi|115553453|dbj|AK236656.1|	red	138.7495	33.277341	0.8815743	0.04804337	30.098
gnl|UG|Ssc#S17527438	violet	138.72137	16.832675	0.940525	0.0172553	32.398
gi|52350674|gb|AY609390.1|	skyblue3	138.69793	8.2805525	0.840135	0.07486268	20.502
gnl|UG|Ssc#S40073180	darkmagent	138.50856	12.031913	0.9208418	0.02641514	24.756
gi|74136760|ref|NM_001033014.1|	pink	138.36945	9.7551119	0.2583138	0.67479975	35.072
gnl|UG|Ssc#S22311415	midnightblu	137.94359	14.794755	0.7716283	0.12642421	21.952
gi|115551900|dbj|AK236307.1|	darkgrey	137.86485	13.83439	0.9531961	0.01206936	4.75
gnl|UG|Ssc#S26722922	navy	137.70671	22.250419	0.782106	0.11802437	13.494
gi|47523737|ref|NM_214339.1|	red	137.70626	26.630645	0.7632533	0.13326062	52.366
gi|194037555|ref|XM_001929413.1|	darkmagent	137.56659	14.608523	0.9681761	0.00678234	119.636
gnl|UG|Ssc#S26721025	orange	137.56603	7.3732789	0.5912471	0.29370891	38.632
gnl|UG|Ssc#S18549874	pink	137.46853	11.235974	0.499362	0.39170588	1.414
gnl|UG|Ssc#S26646909	grey60	137.36592	14.429878	0.6482478	0.23677189	12.52
gnl|UG|Ssc#S39984272	darkgrey	137.32957	5.6581276	0.3372869	0.57884039	11.818
gi|115551173|dbj|AK232606.1|	paleturquois	137.31916	16.35927	0.9850715	0.00218466	33.35
gi|115551482|dbj|AK236160.1|	turquoise	137.2365	103.19362	0.5176487	0.371658	60.764
gnl|UG|Ssc#S26711451	violet	137.17116	14.316216	-0.907461	0.03331976	18.72

gnl|UG|Ssc#S18550002	magenta	137.09799	10.868288	0.3773465	0.5312063	27.496
gi|47522919|ref|NM_214052.1|	darkgreen	137.05236	15.156782	0.8033302	0.1015534	4.7
gi|115548574|dbj|AK231558.1|	pink	136.99735	19.540795	0.7549239	0.14016449	202.522
gnl|UG|Ssc#S18546502	pink	136.94342	23.03909	0.8648781	0.05840076	13.984
gnl|UG|Ssc#S19545689	orange	136.89655	7.2436014	0.7689398	0.12860718	6.87
gi|194040788|ref|XM_001925300.1|	red	136.76983	16.514658	0.4957568	0.3956876	36.458
gnl|UG|Ssc#S23689938	pink	136.28012	17.885067	0.9825129	0.00276865	11.132
gnl|UG|Ssc#S23771071	magenta	136.23205	11.4403	0.4913081	0.400614	3.416
gi|115551644|dbj|AK239108.1|	paleturquois	136.08339	15.725512	0.9725015	0.00545128	8.524
gnl|UG|Ssc#S26715181	red	136.07568	12.450627	0.3729595	0.53638404	4.282
gnl|UG|Ssc#S16763004	royalblue	136.01521	15.530682	0.9143681	0.02969127	11.156
gi|194041914|ref|XM_001928393.1|	navy	135.89997	29.885687	0.8445305	0.07184644	43.99
gnl|UG|Ssc#S18555802	darkgrey	135.82353	5.8457336	0.4280296	0.47214699	11.07
gnl|UG|Ssc#S19547820	red	135.62765	25.861501	0.7709558	0.12696922	11.03
gnl|UG|Ssc#S18358279	darkgreen	135.62273	5.0790818	0.7497391	0.14451342	39.158
gi|194039587|ref|XM_001929390.1|	magenta	135.00275	17.329587	0.7752919	0.12346763	36.698
gi|194033956|ref|XM_001926015.1|	royalblue	134.94293	21.473066	0.9831219	0.00262552	20.346
gi|115554315|dbj|AK240110.1|	turquoise	134.66635	86.971257	0.4376954	0.46105289	28.588
gnl|UG|Ssc#S19548276	darkmagent	134.44201	11.404686	0.9080394	0.03301059	7.01
gi|194036122|ref|XM_001929505.1|	orange	134.41085	6.2227982	0.6864352	0.20056795	16.364
gi|194036768|ref|XM_001927459.1|	royalblue	134.23942	18.299796	0.9475657	0.01429918	7.442
gi|194041270|ref|XM_001926989.1|	grey60	134.2055	10.055254	0.6993891	0.18867609	25.574
gnl|UG|Ssc#S35171904	tan	134.16767	30.621044	0.8964631	0.03936537	177.212
gnl|UG|Ssc#S23701117	navy	133.92465	17.856953	-0.489408	0.40272273	121.558
gnl|UG|Ssc#S40288802	red	133.80842	34.861959	0.9418013	0.01670615	183.3
gi|47523665|ref|NM_214304.1|	violet	133.76098	11.330348	-0.846117	0.07076757	38.494
gnl|UG|Ssc#S5993568	magenta	133.5199	12.724837	0.6396908	0.24510714	1.324
gnl|UG|Ssc#S23695768	paleturquois	133.33652	12.656969	0.9348866	0.01974929	7.898
gi|113205555|ref|NM_001044525.1|	darkgreen	133.04633	4.1655955	0.6274672	0.25714815	12.534
gi|115552176|dbj|AK230485.1|	tan	133.02038	29.133429	0.8931474	0.04125013	17.972
gi|115549693|dbj|AK231972.1|	magenta	132.74368	15.571722	0.6779194	0.20849583	48.542
gi|194036879|ref|XM_001927666.1|	darkmagent	132.52954	11.34978	0.9052091	0.03453108	93.168
gnl|UG|Ssc#S39885581	paleturquois	132.44463	14.438806	0.943632	0.01592854	2.882
gi|194044905|ref|XM_001926512.1|	red	132.33689	19.222516	0.6053953	0.27927538	10.03
gnl|UG|Ssc#S6077174	skyblue	131.95096	6.1693038	0.6907414	0.19659203	10.088
gnl|UG|Ssc#S26400047	pink	131.63783	16.928189	0.8124034	0.09474502	13.59
gi|115547946|dbj|AK234923.1|	paleturquois	131.55365	13.180702	0.9091633	0.0324129	33.1
gi|47522635|ref|NM_213927.1|	navy	131.40538	29.399676	0.8761803	0.05131964	36.626
gnl|UG|Ssc#S31114330	pink	131.17927	15.580256	0.9631285	0.00845188	3.942
gi|113205761|ref|NM_001044578.1|	pink	131.08261	17.41235	0.9028143	0.03583461	47.838
gi|115550292|dbj|AK235771.1|	yellow	131.05353	20.529827	0.5105642	0.37939493	57.986
gnl|UG|Ssc#S22287912	grey60	130.87871	10.352679	0.7863953	0.11463593	16.016
gnl|UG|Ssc#S23755031	magenta	130.53505	15.53912	0.7352271	0.15689014	13.556
gnl|UG|Ssc#S17513789	royalblue	130.48028	19.747204	0.972818	0.00535769	32.228
gnl|UG|Ssc#S18551520	grey60	130.24079	10.344998	0.5259114	0.36268331	36.588
gnl|UG|Ssc#S26734160	royalblue	129.87226	0.8746316	-0.36875	0.54136206	8.76

paleturquois

gnl|UG|Ssc#S40196887	yellow	129.68515	21.410271	0.5361453	0.35164223	18.024
gi|194037499|ref|XM_001928879.1|	magenta	129.64473	14.355961	0.7356039	0.15656502	41.482
gnl|UG|Ssc#S19549310	yellow	129.62571	27.705173	0.7129469	0.17645397	16.926
gnl|UG|Ssc#S6065178	navy	129.12708	33.259427	0.9548137	0.01145191	10.422
gnl|UG|Ssc#S14766801	orange	128.89212	6.1762526	0.7325233	0.15922859	2.43
gi|194039709|ref|XM_001929236.1|	turquoise	128.67533	83.269325	0.4388273	0.45975759	40.422
gnl|UG|Ssc#S18549491	darkgreen	128.65739	10.321254	0.8292901	0.08246627	23.224
gnl|UG|Ssc#S18552627	turquoise	128.10673	77.344095	0.4384696	0.46016678	57.126
gnl|UG|Ssc#S19549286	magenta	127.90898	15.644485	0.7575338	0.13799021	4.908
gnl|UG|Ssc#S35332920	darkmagent	127.56487	9.9507428	0.8902569	0.04291588	12.1
gnl|UG|Ssc#S23698749	pink	127.52768	14.848877	0.7609421	0.13516589	9.914
gi|194037944|ref|XM_001924408.1|	red	127.41055	27.719424	0.9137402	0.03001554	25.534
gnl|UG|Ssc#S18554156	orange	127.14469	7.8022267	0.6637772	0.22184897	1.824
gi|194037031|ref|XM_001925224.1|	pink	127.12096	10.749862	0.4800848	0.41310505	16.688
gnl|UG|Ssc#S16515706	darkgrey	126.87033	10.467395	0.8893022	0.0434707	13.296
gi|194038212|ref|XM_001927641.1|	turquoise	126.83975	78.615212	0.4089367	0.49422532	14.182
gnl|UG|Ssc#S39990281	magenta	126.55809	12.195982	0.6149517	0.26963545	4.458
gi|158262680|ref|NM_001109947.1|	red	126.4389	33.70403	0.9161177	0.02879376	84.778
gnl|UG|Ssc#S17510986	red	126.28131	33.41287	0.8817248	0.04795299	323.622
gnl|UG|Ssc#S40143536	pink	126.21826	10.59152	0.4202951	0.48106508	29.604
gi|115546929|dbj|AK240575.1|	navy	125.60876	16.067573	-0.52206	0.36685983	29.992
gnl|UG|Ssc#S40125669	pink	125.28755	14.670319	-0.795023	0.10791072	2.192
gnl|UG|Ssc#S23689648	paleturquois	125.26659	6.9638758	0.7455371	0.14806648	31.398
gi|194044034|ref|XM_001928794.1|	grey60	125.08818	14.115277	0.9073007	0.03340533	662.266
gi|194042287|ref|XM_001927955.1|	royalblue	124.28437	16.491577	0.9396638	0.01762913	177.722
gi|194040496|ref|XM_001928226.1|	navy	124.17668	32.500936	0.9282545	0.02281898	13.302
gnl|UG|Ssc#S18555071	grey60	124.14932	15.631203	0.7798298	0.11983449	3.524
gnl|UG|Ssc#S31116246	magenta	123.5808	13.078853	0.6714222	0.21460184	1.976
gi|194043399|ref|XM_001929569.1|	grey	123.10063	9.762258	0.6965306	0.19128257	15.776
gnl|UG|Ssc#S17518379	pink	122.65854	12.914813	0.6126903	0.2719085	11.178
gnl|UG|Ssc#S40486793	red	122.56387	26.371472	0.7434282	0.14985912	0.928
gi|52351457|gb|AY609887.1|	magenta	122.23867	11.880437	0.603152	0.28155113	68.726
gi|52351294|gb|AY609726.1|	navy	121.99241	15.606902	-0.240726	0.6964843	4.036
gnl|UG|Ssc#S18279557	red	121.83231	17.772581	0.4835521	0.40923657	12.576
gnl|UG|Ssc#S17513958	pink	121.62676	9.9843872	0.1205734	0.84685394	34.548
gi|47522707|ref|NM_213889.1|	pink	121.58313	20.867754	0.7754547	0.1233367	14.306
gi|47522703|ref|NM_213891.1|	pink	121.37558	19.033013	0.7285721	0.16266393	85.104
gnl|UG|Ssc#S40527391	navy	121.33168	11.838578	0.6487086	0.23632522	33.608
gnl|UG|Ssc#S23689635	red	121.30213	28.844274	0.8025479	0.10214708	22.508
gi|113205565|ref|NM_001044529.1|	magenta	121.27358	7.3966781	0.3825631	0.52506249	6.684
gi|194043395|ref|XM_001929562.1|	orange	121.18017	7.211339	0.6113446	0.27326356	7.254
gi|194043630|ref|XM_001926758.1|	violet	121.09958	10.778203	-0.710241	0.17887428	5.93
gnl|UG|Ssc#S39812694	pink	120.84379	10.926738	0.1546422	0.80389099	10.656
gi|148233297|ref|NM_001097485.1|	paleturquois	120.43351	7.7125185	0.7726588	0.12559052	88.214
gnl|UG|Ssc#S39982735	violet	119.92415	12.297383	-0.738269	0.15427153	4.372
gnl|UG|Ssc#S34532625	grey	119.47193	9.4129846	0.8259833	0.08482936	9.202
gi|46095042|gb|AY574215.1|	orange	119.27147	6.5178397	0.6440243	0.24087611	31.33
gi|194042188|ref|XM_001924178.1|	grey	119.14994	9.7665651	0.8479018	0.06955923	57.462

gi|194040706|ref|XM_001927201.1|	navy	119.11598	13.315901	-0.168385	0.78662354	235.332
gnl|UG|Ssc#S39943227	red	119.10867	30.462587	0.8764513	0.05115337	11.646
gi|194037879|ref|XM_001925784.1|	royalblue	118.34151	18.650739	0.9709093	0.00593009	23.448
gnl|UG|Ssc#S31131447	violet	118.30624	14.784767	-0.827549	0.08370798	32.956
gi|47522679|ref|NM_213904.1|	grey	118.00224	10.706543	0.6304284	0.25421689	81.344
gnl|UG|Ssc#S18264158	grey	117.9387	10.511845	0.6658808	0.21984818	11.356
gnl|UG|Ssc#S34514406	grey	117.7159	9.7260672	0.7499658	0.14432238	14.558
gi|115552314|dbj|AK230624.1|	darkmagent	117.62412	4.8562493	0.7060462	0.18264566	59.42
gnl|UG|Ssc#S19545503	darkgrey	117.58372	12.195911	0.8937987	0.04087769	15.188
gnl|UG|Ssc#S23699859	violet	117.375	14.240994	-0.802569	0.10213124	199.186
gi|115552089|dbj|AK239282.1|	red	117.36265	21.45317	0.5770097	0.30842209	76.214
gnl|UG|Ssc#S22316206	darkmagent	117.3228	11.529461	0.9125057	0.03065631	41.008
gnl|UG|Ssc#S18359803	orange	117.15336	8.0623662	0.7798123	0.11984843	35.318
gnl|UG|Ssc#S40571777	red	116.85663	17.920345	0.4684881	0.42610398	7.832
gi|115551105|dbj|AK232538.1|	navy	116.7694	31.861339	0.925095	0.02433098	25.508
gi|115555527|dbj|AK234140.1|	violet	116.74737	12.438056	-0.846759	0.07033167	5.09
gnl|UG|Ssc#S40322346	pink	116.4783	15.745913	0.9052412	0.03451371	35.662
gi|115545853|dbj|AK234286.1|	grey60	116.3044	13.447853	0.7131436	0.17627844	18.256
gi|194044483|ref|XM_001929150.1|	grey	116.01495	9.984927	0.6412282	0.24360376	105.112
gi|115549571|dbj|AK238379.1|	grey	116.01326	10.483229	0.7176604	0.17226016	22.488
gnl|UG|Ssc#S18354391	violet	115.8186	8.4024316	0.4419313	0.4562093	36.016
gi|115546462|dbj|AK230981.1|	yellow	115.69287	26.430101	0.7164164	0.17336414	10.66
gi|115550900|dbj|AK238761.1|	violet	115.54716	9.7424221	-0.5748	0.31072213	61.948
gi|115553196|dbj|AK232994.1|	magenta	115.52087	10.330671	0.5375533	0.35012979	16.652
gi|29373949|emb|AJ539380.1|	grey	115.09092	10.113071	0.635521	0.24919706	54.628
gnl|UG|Ssc#S40154789	pink	115.00221	9.1759683	0.3211634	0.59822473	9.412
gi|115546114|dbj|AK240356.1|	pink	114.69327	10.569884	0.4477512	0.44957275	10.312
gi|115555619|dbj|AK237252.1|	grey	114.43237	8.7647584	0.6759082	0.21038069	122.902
gnl|UG|Ssc#S39912933	red	114.14775	31.488084	0.8305059	0.08160259	49.118
gi|115553344|dbj|AK233142.1|	red	113.84271	20.347156	0.5285412	0.35983816	18.898
gnl|UG|Ssc#S18359692	red	113.70335	21.078648	0.5412559	0.34616016	10.514
gnl|UG|Ssc#S19542862	orange	113.50241	4.6986728	0.6071564	0.27749218	47.97
gi|194037617|ref|XM_001927127.1|	magenta	113.28479	9.9854336	0.52242	0.36646913	79.634
gnl|UG|Ssc#S18380710	orange	112.70103	4.8985135	0.5910332	0.29392858	45.82
gi|47523617|ref|NM_214276.1|	red	112.48946	12.22289	0.3886429	0.51792014	59.31
gnl|UG|Ssc#S26729898	pink	111.80449	9.9914337	-0.178671	0.77372491	22.646
gnl|UG|Ssc#S18548374	violet	111.76483	11.247886	-0.638058	0.24670654	4.11
gi|194035414|ref|XM_001925502.1|	grey	110.7237	7.6027105	0.7585545	0.13714253	31.68
gnl|UG|Ssc#S18552226	navy	110.71807	16.270463	-0.381865	0.52588326	15.088
gi|115554371|dbj|AK233381.1|	royalblue	110.55741	6.5486917	0.2037769	0.74235019	114.748
gi|115549331|dbj|AK232109.1|	navy	110.54718	16.524761	-0.339006	0.57677992	105.248
gnl|UG|Ssc#S18555329	violet	109.91112	4.6410589	0.4649498	0.43008829	8.842
gi|197251933|ref|NM_001134354.1|	grey60	109.16731	12.379919	0.8828772	0.04726235	15.972
gi|115550191|dbj|AK235670.1|	navy	109.14793	14.407261	-0.371306	0.53833772	31.712
gi|194043858|ref|XM_001928335.1|	red	109.07052	19.838075	0.5010677	0.38982532	25.46
gnl|UG|Ssc#S26733049	red	109.05642	29.109049	0.7749717	0.12372517	115.15
gi|194042438|ref|XM_001927977.1|	turquoise	108.97295	80.549427	0.449232	0.44788756	42.646
gnl|UG|Ssc#S23764110	red	108.90578	19.862654	0.845067	0.07148094	6.362

gi|194044061|ref|XM_001927723.1|	navy	108.28883	14.960426	-0.532335	0.35574287	8.106
gi|47523187|ref|NM_213830.1|	red	108.16378	28.170597	0.848529	0.0691363	11.342
gi|194037661|ref|XM_001928816.1|	royalblue	108.15996	0.8755376	-0.352175	0.56104597	6.454
gnl|UG|Ssc#S18550902	pink	107.98454	6.6161693	0.0485938	0.93815286	90.926
gi|115547606|dbj|AK231264.1|	paleturquois	107.75107	8.7729123	0.8281791	0.08325791	8.896
gi|194039735|ref|XM_001928389.1|	violet	107.32499	10.466662	0.6765019	0.20982382	341.496
gnl|UG|Ssc#S31986337	violet	107.30499	8.9944318	0.614432	0.27015741	8.4
gnl|UG|Ssc#S23689853	red	106.92117	25.439199	0.852701	0.06634335	6.396
gnl|UG|Ssc#S20942207	pink	106.81647	10.33722	0.2955744	0.62921689	18.718
gi|190360630|ref|NM_001128465.1|	pink	106.70986	13.436478	0.908955	0.03252344	76.1
gnl|UG|Ssc#S22280355	orange	106.61465	6.6658957	0.5183051	0.37094318	24.592
gi|194033590|ref|XM_001929195.1|	magenta	106.51462	16.305653	0.7755014	0.12329918	157.532
gnl|UG|Ssc#S18549403	pink	106.48705	12.496971	0.3314944	0.58579109	30.124
gi|194043329|ref|XM_001929273.1|	red	106.04574	24.620461	0.8909986	0.04248647	68.258
gi|115549792|dbj|AK232072.1|	darkgrey	105.68928	10.051244	0.8606424	0.06112748	4.812
gnl|UG|Ssc#S40416576	yellow	105.55157	17.000587	0.4048804	0.49894312	4.256
gnl|UG|Ssc#S40070781	orange	105.54167	5.3755279	0.4767619	0.41682033	14.246
gi|115549976|dbj|AK232211.1|	paleturquois	105.32083	13.033888	0.9420989	0.01657892	17.84
gnl|UG|Ssc#S18268289	royalblue	105.12107	6.4016213	0.5347948	0.35309446	2.802
gi|194036374|ref|XM_001929178.1|	grey	104.75579	9.2757314	0.4611418	0.43438574	33.7
gnl|UG|Ssc#S26400566	orange	104.66219	7.9128564	0.53125	0.35691321	5.75
gi|115553496|dbj|AK236699.1|	violet	104.18946	12.28232	-0.666252	0.21949586	59.24
gnl|UG|Ssc#S18260863	midnightblu	104.02044	9.4648312	0.6678358	0.21799331	11.05
gnl|UG|Ssc#S29989999	navy	103.95318	15.465922	-0.2623	0.66989891	30.478
gnl|UG|Ssc#S23755686	violet	103.21289	14.327057	-0.78568	0.1151988	8.012
gi|115550629|dbj|AK232459.1|	red	103.15283	11.461802	0.3557653	0.55677113	33.212
gi|194039625|ref|XM_001925622.1|	pink	101.8036	10.177356	-0.188413	0.76153164	21.546
gnl|UG|Ssc#S19546116	red	101.65826	23.957393	0.6914313	0.19595716	11.206
gnl|UG|Ssc#S17515091	pink	101.4859	11.67355	0.2599647	0.6727696	2.442
gnl|UG|Ssc#S23696561	red	101.46108	17.149993	0.424263	0.47648558	11.94
gnl|UG|Ssc#S26735228	red	101.12542	29.096034	0.917767	0.02795582	29.14
gnl|UG|Ssc#S23760026	red	100.91786	26.685617	0.9121807	0.03082573	7.278
gnl|UG|Ssc#S26725357	red	100.83628	25.003277	0.7217347	0.16865845	6.542
gi|115547167|dbj|AK231222.1|	yellow	100.78719	29.236746	0.7214831	0.16888025	5.64
gnl|UG|Ssc#S31124721	magenta	100.42397	13.500656	0.5807873	0.30450022	6.18
gnl|UG|Ssc#S23690339	grey60	99.646263	11.577285	0.8734704	0.05299107	25.292
gi|115552800|dbj|AK239394.1|	grey60	98.813633	12.997608	0.8192393	0.08971173	21.764
gi|52351306|gb|AY609738.1|	violet	98.366504	10.698827	-0.764263	0.13243049	62.42
gi|194036851|ref|XM_001928643.1|	magenta	98.270088	16.666194	0.7641365	0.13253465	59.576
gi|115554982|dbj|AK240181.1|	turquoise	98.246405	60.105082	0.367073	0.543347	68.348
gi|52351346|gb|AY609778.1|	pink	98.041486	9.6707165	0.2868436	0.63985145	29.344
gnl|UG|Ssc#S39841622	pink	98.030425	11.097493	0.8711946	0.05440775	8.192
gnl|UG|Ssc#S23689827	darkgreen	98.008485	10.358365	0.7430997	0.15013894	4.87
gnl|UG|Ssc#S40334339	pink	97.815333	10.327179	-0.137532	0.82544184	37.374
gnl|UG|Ssc#S17513547	grey	97.788416	9.0691969	0.5983786	0.28640978	5.88
gi|47522617|ref|NM_213934.1|	darkgrey	97.740905	3.6566471	0.114311	0.85477234	56.36
gnl|UG|Ssc#S39989284	navy	97.714322	12.748612	-0.406044	0.4975884	4.676
gi|115553398|dbj|AK236601.1|	orange	97.606937	7.6433543	0.4951095	0.3964035	22.688

gi|115545749|dbj|AK234182.1|	pink	97.51669	12.135112	0.9023477	0.03609039	14.22
gnl|UG|Ssc#S40238117	pink	97.506771	12.35134	0.4218765	0.4792388	73.132
gi|47523461|ref|NM_214192.1|	orange	97.381847	7.0971903	0.4813825	0.41165626	457.41
gnl|UG|Ssc#S29971491	violet	97.173426	10.423643	-0.629573	0.25506281	18.72
gi|47523537|ref|NM_214231.1|	grey	97.067523	6.7016722	0.7961276	0.10705898	2.962
gnl|UG|Ssc#S29984324	darkgrey	96.395424	1.3535761	0.3136744	0.60726687	239.02
gi|115546413|dbj|AK230932.1|	red	94.611961	14.583933	0.3482028	0.56578378	10.476
gnl|UG|Ssc#S19541103	grey	94.528128	7.1281806	0.3545932	0.55816618	49.9
gnl|UG|Ssc#S23695399	red	94.366192	29.212216	0.8862727	0.04524601	24.224
gi|115548000|dbj|AK237984.1|	navy	94.273914	12.380101	-0.213275	0.73052214	14.764
gi|115554299|dbj|AK240094.1|	violet	94.075362	10.212084	0.7509986	0.14345332	114.108
gnl|UG|Ssc#S18551614	red	93.977683	19.475822	0.8321733	0.08042268	5.472
gnl|UG|Ssc#S19537170	yellow	93.587612	25.195941	0.6230357	0.26155156	28.962
gi|115547950|dbj|AK234927.1|	pink	93.375979	10.419943	0.5013471	0.38951754	81.274
gnl|UG|Ssc#S18554486	royalblue	93.332665	6.9219296	0.2414619	0.69557534	19.714
gnl|UG|Ssc#S26721534	red	93.094317	26.672651	0.7188232	0.17123002	46.772
gnl|UG|Ssc#S23769437	turquoise	93.061732	61.509379	0.3827956	0.52478896	22.63
gnl|UG|Ssc#S38483118	magenta	92.263068	7.7491876	0.3713247	0.53831603	17.042
gi|47522799|ref|NM_213987.1|	violet	91.290077	9.6886699	0.5810967	0.30417957	5.712
gi|115554130|dbj|AK233340.1|	turquoise	90.213106	57.376349	0.3638282	0.54719271	33.99
gnl|UG|Ssc#S23698110	royalblue	89.998554	7.2395111	0.2879789	0.63846697	45.434
gi|115549562|dbj|AK238370.1|	red	89.678015	19.462053	0.6040632	0.28062613	10.27
gnl|UG|Ssc#S23700414	pink	89.345414	8.7289765	0.3572189	0.55504198	12.53
gi|194036038|ref|XM_001928648.1|	pink	89.121566	9.5184211	0.2203448	0.72173533	19.34
gnl|UG|Ssc#S40144447	magenta	89.066828	6.5650659	0.3371812	0.57896712	61.374
gi|194040755|ref|XM_001927097.1|	orange	89.056153	6.8038187	0.405989	0.49765277	144.69
gi|115547122|dbj|AK231177.1|	turquoise	88.691625	62.786501	0.3960835	0.50920611	20.248
gnl|UG|Ssc#S23775545	royalblue	88.579906	10.915622	0.8580391	0.06282258	2.442
gnl|UG|Ssc#S23776646	pink	88.366285	7.9072606	0.3405323	0.57495281	26.086
gi|194037553|ref|XM_001929410.1|	navy	87.853387	18.456389	0.7359487	0.15626773	382.886
gi|115553209|dbj|AK233007.1|	pink	87.680174	9.9555708	-0.144039	0.81723968	107.014
gnl|UG|Ssc#S39803256	orange	87.61531	4.6743488	0.3488577	0.56500219	3.516
gnl|UG|Ssc#S31111456	pink	87.518871	12.349539	0.4061707	0.49744131	17.978
gnl|UG|Ssc#S40239854	royalblue	87.457952	7.1687308	0.403219	0.50087804	3.568
gnl|UG|Ssc#S39861498	red	87.256429	9.1621267	0.2590434	0.67390245	87.258
gnl|UG|Ssc#S39776069	grey60	86.018948	0.9704145	-0.420405	0.48093771	39.532
gnl|UG|Ssc#S19550176	yellow	85.599073	14.603628	0.3215127	0.59780362	2.162
gi|48374070|ref|NM_001001539.1|	yellow	85.426456	20.533738	0.5189764	0.37021236	34.254
gnl|UG|Ssc#S40152348	pink	85.423033	8.134854	0.2346228	0.70403285	12.992
gnl|UG|Ssc#S18558154	grey	84.803865	7.3487135	0.4894892	0.40263223	5.336
gi|9802381|gb|AF281156.1|AF281156	red	84.546083	25.634047	0.7230731	0.16748016	4.244
gi|56199611|gb|AY705447.1|	pink	83.827084	10.255233	0.2690681	0.66159171	6.032
gi|217416473|ref|NM_001142668.1|	grey	83.750958	6.0246088	0.3256086	0.59286906	25.094
gi|57528019|ref|NM_001009581.1|	darkmagent	82.875335	6.4420021	0.7747707	0.12388694	20.534
gnl|UG|Ssc#S6063458	violet	82.820762	8.6280867	0.5243076	0.36442122	28.864
gi|194033718|ref|XM_001926719.1|	darkgrey	82.730813	2.9699777	-0.108134	0.8625884	18.746
gnl|UG|Ssc#S23772152	pink	82.465172	11.155767	0.4950474	0.39647225	29.474
gnl|UG|Ssc#S19541579	pink	82.219453	10.229805	0.1309321	0.83376967	24.712

gnl|UG|Ssc#S23759640	pink	81.787564	9.8164569	0.2506876	0.68419	137.088
gnl|UG|Ssc#S35325128	darkgrey	81.629567	2.3821064	0.4409805	0.45729554	3.274
gnl|UG|Ssc#S17526422	darkgrey	81.132766	6.2411006	0.7031261	0.18528406	11.742
gnl|UG|Ssc#S34524449	pink	80.783227	10.666473	0.0157182	0.97998777	6.002
gnl|UG|Ssc#S18386573	orange	80.604022	4.0755121	0.1956622	0.752474	11.59
gnl|UG|Ssc#S19546988	turquoise	79.753378	49.862901	0.3662908	0.54427362	41.832
gnl|UG|Ssc#S26727063	violet	79.698039	7.3082235	0.4576109	0.43837899	8.094
gnl|UG|Ssc#S26714791	magenta	79.557859	12.558132	0.6045456	0.28013681	69.504
gnl|UG|Ssc#S34500484	royalblue	79.282236	7.1254927	0.212394	0.73161873	11.31
gi|115547397|dbj|AK237778.1|	red	79.190422	26.663199	0.8101462	0.09642534	7.934
gnl|UG|Ssc#S16761675	orange	78.815087	5.1990797	0.3426699	0.5723948	9.874
gi|194045111|ref|XM_001927898.1|	darkgrey	78.184197	3.9914617	0.2498192	0.68526056	41.854
gi|197717772|gb|EF113595.2|	violet	77.747873	5.9607208	0.2954235	0.62940045	214.092
gi|47523559|ref|NM_214239.1|	royalblue	76.024873	6.5245673	0.2624808	0.66967728	38.972
gi|115551480|dbj|AK236158.1|	grey	75.894558	5.914674	0.3156142	0.60492252	13.6
gnl|UG|Ssc#S17511731	magenta	75.867032	9.4822787	0.3633976	0.54770345	7.086
gnl|UG|Ssc#S16512910	pink	75.644033	9.366807	0.1189463	0.84891077	2.898
gi|115546179|dbj|AK240417.1|	pink	74.601181	7.7288409	0.1021572	0.87015594	9.336
gnl|UG|Ssc#S40491599	grey60	74.543503	1.9102551	-0.112987	0.85644759	5.814
gnl|UG|Ssc#S26719135	yellow	73.435157	16.511278	0.3954972	0.50989174	19.546
gi|115552956|dbj|AK239550.1|	darkgrey	72.862434	3.355201	-0.031279	0.96018066	4.798
gi|47522845|ref|NM_214010.1|	darkgrey	72.092266	3.7931817	0.1087504	0.86180815	55.652
gnl|UG|Ssc#S23765368	turquoise	71.245058	48.723916	0.3714506	0.53816727	2.366
gnl|UG|Ssc#S23691069	orange	70.610501	6.4071597	0.5646007	0.32139477	18.452
gnl|UG|Ssc#S31113853	darkgrey	69.514044	4.2739433	0.5158779	0.37358826	15.766
gnl|UG|Ssc#S23698294	red	67.787834	22.178342	0.6492326	0.23581769	11.946
gnl|UG|Ssc#S18549761	pink	66.676067	3.1494361	0.0871221	0.88921317	31.408
gnl|UG|Ssc#S23695397	pink	66.448307	10.035936	0.3524948	0.56066543	27.182
gi|194034729|ref|XM_001924943.1|	orange	66.43938	4.5440012	0.3948138	0.51069099	61.65
gnl|UG|Ssc#S23691372	pink	66.203477	7.2049603	-0.381882	0.52586395	19.412
gnl|UG|Ssc#S18549494	red	63.498291	18.822105	0.6380639	0.24670067	3.71
gnl|UG|Ssc#S18553581	darkgrey	62.588104	0.8247482	-0.07855	0.90008997	111.57
gnl|UG|Ssc#S26712602	orange	61.525405	5.0738592	0.2402667	0.69705225	12.89
gi|51592101|ref|NM_001004028.1|	navy	61.246305	6.1871724	-0.313774	0.60714603	11.71
gnl|UG|Ssc#S22287254	red	61.063532	18.720247	0.5551713	0.3313424	35.606
gi|115552537|dbj|AK236542.1|	red	59.132082	18.491569	0.6551695	0.2300875	10.148
gi|115551521|dbj|AK238985.1|	royalblue	58.166931	6.5916897	0.1561563	0.80198661	67.36
gnl|UG|Ssc#S35173188	red	57.633664	13.254297	0.4132659	0.48920061	8.596
gnl|UG|Ssc#S17526927	royalblue	52.965438	5.8856474	0.638346	0.24642412	3.408
gnl|UG|Ssc#S26726211	pink	50.11439	6.3569312	-0.03596	0.9542245	3.29
gnl|UG|Ssc#S16764287	yellow	49.874269	9.1898915	0.3252977	0.59324335	7.518
gi|194036795|ref|XM_001926863.1|	yellow	49.24125	8.9444093	0.1997142	0.74741665	10.528
gi|115553497|dbj|AK236700.1|	darkgrey	47.490745	2.2177019	0.1528746	0.80611486	314.974
gnl|UG|Ssc#S34511356	red	47.250071	11.394138	0.3657097	0.54496213	7.672
gi|113205651|ref|NM_001044553.1|	royalblue	47.091514	4.8134119	0.5311257	0.35704734	45.238
gnl|UG|Ssc#S23762936	royalblue	45.120711	5.7103483	0.2861237	0.64072966	23.108
gnl|UG|Ssc#S26723792	grey	43.027301	0.3756514	0.1690408	0.78580006	0.942
gnl|UG|Ssc#S18289737	turquoise	41.917366	12.586387	0.126011	0.8399834	17.876

Table S2-4 Attributes of genes in all modules of LT postnatal.

GeneSymbol	Module	kTotal	kWithin	eigencorr	eigenpval	meanExpr
gi|10304379|gb|AF288822.1|AF28882	darkgreen	227.9604	19.785	0.814487	0.093202	40.564
gi|106073315|gb|DQ508264.1|	midnightblue	115.1175	31.2781	0.918765	0.027453	94.888
gi|108796051|ref|NM_213912.2|	brown	114.2136	36.2588	0.713788	0.175704	390.69
gi|108796069|ref|NM_001042375.1|	turquoise	767.4647	238.488	0.937962	0.018375	307.39
gi|109639160|ref|NM_001025222.2|	green	142.9373	47.2479	0.923689	0.025014	1645.34
gi|109639161|ref|NM_001006593.2|	darkgrey	168.7855	28.3404	0.94345	0.016006	9.976
gi|112181313|ref|NM_214438.2|	white	192.6601	7.84721	0.161158	0.795699	486.494
gi|11276047|gb|AF319661.1|	yellow	227.7817	78.7817	0.897465	0.038802	2.074
gi|113205497|ref|NM_001044527.1|	green	75.26229	36.7015	0.793125	0.10938	6225.968
gi|113205581|ref|NM_001044532.1|	pink	196.4695	59.9456	0.953881	0.011807	23.858
gi|113205585|ref|NM_001044535.1|	turquoise	530.5862	165.242	0.829869	0.082055	36.464
gi|113205611|ref|NM_001044541.1|	darkmagenta	989.5859	26.5177	0.990016	0.001196	27.728
gi|113205623|ref|NM_001044545.1|	darkmagenta	973.8972	26.857	0.99297	0.000707	21.52
gi|113205635|ref|NM_001044548.1|	pink	270.3017	75.3635	0.997006	0.000197	7.026
gi|113205649|ref|NM_001044550.1|	green	91.251	40.3223	0.833347	0.079596	703.308
gi|113205651|ref|NM_001044553.1|	purple	68.54283	12.9862	0.636384	0.248349	44.358
gi|113205653|ref|NM_001044551.1|	turquoise	617.8179	203.639	0.905952	0.03413	627.042
gi|113205665|ref|NM_001044554.1|	lightcyan	275.9698	40.8369	0.971355	0.005795	91.424
gi|113205675|ref|NM_001044558.1|	white	163.2146	18.3262	0.731986	0.159694	17.26
gi|113205689|ref|NM_001044559.1|	blue	860.6251	233.497	0.969252	0.006442	17.694
gi|113205765|ref|NM_001044576.1|	lightyellow	852.4371	59.9727	0.988309	0.001515	15.828
gi|113205779|ref|NM_001044584.1|	green	136.3366	39.8727	0.851089	0.067418	50.568
gi|113205811|ref|NM_001044593.1|	black	980.7541	96.8313	0.993834	0.000581	518.232
gi|113205825|ref|NM_001044594.1|	darkorange	135.8717	18.2274	0.899606	0.037605	263.652
gi|113205835|ref|NM_001044599.1|	violet	986.2911	74.5655	0.997437	0.000156	101.432
gi|113205859|ref|NM_001044605.1|	blue	390.28	77.4084	0.729755	0.161633	96.68
gi|113205873|ref|NM_001044606.1|	magenta	258.9874	71.0626	0.966955	0.007175	315.914
gi|113205877|ref|NM_001044607.1|	royalblue	336.9907	32.7652	0.992102	0.000842	42.512
gi|113205897|ref|NM_001044613.1|	blue	769.0289	187.803	0.918958	0.027356	13.04
gi|113205905|ref|NM_001044614.1|	black	956.9403	96.1754	0.992875	0.000721	144.45
gi|113205907|ref|NM_001044616.1|	blue	700.7005	197.234	0.934613	0.019873	95.69
gi|113205909|ref|NM_001044615.1|	blue	776.2276	232.166	0.967627	0.006958	7.73
gi|113205921|ref|NM_001044618.1|	lightcyan	199.3814	28.1033	0.895502	0.039909	15.548
gi|113205929|ref|NM_001044620.1|	darkorange	159.7073	9.13064	0.579872	0.305449	206.106
gi|114326182|ref|NM_001048072.1|	navy	153.5	38.66	0.952197	0.012456	88.23
gi|114326213|ref|NM_001048069.1|	turquoise	640.3681	163.781	0.847542	0.069802	14.414
gi|115545542|dbj|AK230657.1|	blue	755.464	212.487	0.945365	0.015204	80.202
gi|115545552|dbj|AK230667.1|	turquoise	444.1552	116.277	0.739201	0.153472	22.25
gi|115545568|dbj|AK230683.1|	brown	131.6826	42.8046	0.819489	0.08953	21.626
gi|115545600|dbj|AK230715.1|	lightcyan	231.703	35.4866	0.92468	0.024532	58.736
gi|115545603|dbj|AK230718.1|	turquoise	363.7236	114.872	0.721685	0.168702	0.622
gi|115545609|dbj|AK230724.1|	sienna3	885.0631	25.0755	0.99184	0.000884	23.766
gi|115545622|dbj|AK230737.1|	turquoise	360.7645	109.21	0.710276	0.178843	13.412
gi|115545632|dbj|AK230747.1|	turquoise	656.52	206.513	0.922039	0.025823	15.87
gi|115545643|dbj|AK230758.1|	white	923.0399	29.5982	0.823772	0.086421	29.456
gi|115545669|dbj|AK230784.1|	lightcyan	313.9209	39.4494	0.95519	0.01131	143.292

gi|115545681|dbj|AK230796.1|	magenta	205.0892	64.9814	0.948723	0.013831	20.168
gi|115545725|dbj|AK234158.1|	turquoise	426.7444	124.092	0.739937	0.152841	1.962
gi|115545741|dbj|AK234174.1|	violet	906.6453	73.3303	0.994055	0.00055	18.3
gi|115545742|dbj|AK234175.1|	lightcyan	180.5357	11.8501	0.46254	0.432807	11.834
gi|115545749|dbj|AK234182.1|	red	563.7784	98.8987	0.975267	0.004652	7.304
gi|115545756|dbj|AK234189.1|	greenyellow	168.4556	51.0685	0.979375	0.003545	5.542
gi|115545757|dbj|AK234190.1|	tan	859.7974	66.2259	0.99055	0.001101	13.238
gi|115545762|dbj|AK234195.1|	darkred	242.5098	44.7733	0.991452	0.000947	5.842
gi|115545765|dbj|AK234198.1|	turquoise	574.9854	190.104	0.870568	0.0548	28.588
gi|115545770|dbj|AK234203.1|	turquoise	687.4944	176.234	0.874767	0.052189	8.85
gi|115545783|dbj|AK234216.1|	yellow	301.7624	110.602	0.978133	0.003869	2.53
gi|115545816|dbj|AK234249.1|	turquoise	200.7615	43.4901	0.437637	0.46112	27.126
gi|115545827|dbj|AK234260.1|	blue	751.4005	223.612	0.961985	0.008847	9.862
gi|115545844|dbj|AK234277.1|	brown	118.3431	42.3551	0.877719	0.050378	555.19
gi|115545853|dbj|AK234286.1|	black	958.4034	98.8656	0.99868	5.76E-05	30.558
gi|115545868|dbj|AK234301.1|	tan	948.2139	59.4798	0.966342	0.007375	8.424
gi|115545894|dbj|AK234327.1|	magenta	265.1835	81.917	0.998545	6.66E-05	26.018
gi|115545904|dbj|AK234337.1|	darkmagenta	794.3376	23.2632	0.964217	0.008082	18.468
gi|115545921|dbj|AK237362.1|	turquoise	843.2997	261.818	0.967687	0.006939	13.204
gi|115545926|dbj|AK237367.1|	green	102.2979	45.8212	0.907385	0.03336	15.292
gi|115545943|dbj|AK237384.1|	pink	119.5624	12.5781	0.088841	0.887033	1.898
gi|115545949|dbj|AK237390.1|	green	119.4141	58.5855	0.980488	0.003262	43.65
gi|115545955|dbj|AK237396.1|	turquoise	697.2448	210.728	0.927373	0.023238	4.996
gi|115545973|dbj|AK237414.1|	purple	113.1843	14.3897	0.757091	0.138358	160.61
gi|115545984|dbj|AK237425.1|	red	329.5938	73.6241	0.91096	0.031465	22.494
gi|115545987|dbj|AK237428.1|	greenyellow	115.3271	31.2738	0.861699	0.060444	18.036
gi|115545992|dbj|AK237433.1|	turquoise	708.9512	207.562	0.908835	0.032587	14.12
gi|115546038|dbj|AK237480.1|	navy	164.8134	43.3976	0.98777	0.001621	16.524
gi|115546047|dbj|AK237489.1|	turquoise	450.8548	138.552	0.787603	0.113687	29.384
gi|115546075|dbj|AK237517.1|	lightcyan	297.3675	43.5788	0.990237	0.001156	16.596
gi|115546087|dbj|AK237529.1|	salmon	165.6599	43.7241	0.955186	0.011311	48.612
gi|115546117|dbj|AK240359.1|	navy	156.8371	42.6814	0.981899	0.002915	27.052
gi|115546125|dbj|AK230834.1|	black	836.9002	90.3762	0.980232	0.003327	27.676
gi|115546138|dbj|AK240376.1|	black	937.6764	97.1449	0.994768	0.000454	92.706
gi|115546160|dbj|AK240398.1|	blue	745.7006	214.816	0.948799	0.0138	8.536
gi|115546163|dbj|AK240401.1|	lightcyan	169.1851	12.9137	0.522691	0.366175	4.77
gi|115546179|dbj|AK240417.1|	pink	272.0237	70.2201	0.965145	0.007771	10.546
gi|115546190|dbj|AK240428.1|	yellow	270.332	102.878	0.963222	0.00842	3.876
gi|115546193|dbj|AK240431.1|	lightcyan	204.428	30.631	0.895909	0.039679	52.396
gi|115546201|dbj|AK240439.1|	salmon	170.8633	45.4914	0.962997	0.008497	28.244
gi|115546215|dbj|AK240453.1|	green	93.31074	40.2935	0.850249	0.067981	30.938
gi|115546231|dbj|AK240469.1|	white	193.1636	24.6828	0.879959	0.049017	7.384
gi|115546234|dbj|AK240472.1|	turquoise	903.1725	267.944	0.978633	0.003737	13.624
gi|115546241|dbj|AK240479.1|	orange	286.3087	30.853	0.968767	0.006595	213.696
gi|115546292|dbj|AK240530.1|	darkorange	176.4019	21.9788	0.985945	0.001996	10.656
gi|115546304|dbj|AK240542.1|	blue	880.2415	248.676	0.985244	0.002147	26.04
gi|115546313|dbj|AK240551.1|	magenta	267.8723	75.4288	0.979804	0.003435	6.328
gi|115546324|dbj|AK230843.1|	darkgrey	154.8386	23.5217	0.913564	0.030107	87.472

gi|115546331|dbj|AK230850.1|	darkturquoise	211.3913	32.4981	0.978103	0.003877	67.248
gi|115546334|dbj|AK230853.1|	violet	937.0112	73.5181	0.994666	0.000467	54.728
gi|115546337|dbj|AK230856.1|	turquoise	401.1657	117.908	0.725006	0.165782	24.774
gi|115546371|dbj|AK230890.1|	red	616.9937	93.5819	0.963067	0.008473	43.102
gi|115546374|dbj|AK230893.1|	darkmagenta	972.5468	26.1962	0.987383	0.001698	14.17
gi|115546403|dbj|AK230922.1|	blue	818.7351	233.796	0.971603	0.00572	6.674
gi|115546406|dbj|AK230925.1|	blue	936.8187	211.632	0.949464	0.013534	14.562
gi|115546428|dbj|AK230947.1|	pink	260.2889	71.0475	0.982947	0.002666	7.94
gi|115546438|dbj|AK230957.1|	royalblue	195.0516	0.90031	-0.48291	0.409951	76.236
gi|115546446|dbj|AK230965.1|	turquoise	836.3576	245.863	0.94829	0.014005	63.508
gi|115546452|dbj|AK230971.1|	darkred	252.568	37.214	0.951419	0.01276	18.836
gi|115546465|dbj|AK230984.1|	skyblue	158.1999	21.4161	0.908692	0.032663	122.188
gi|115546477|dbj|AK230996.1|	skyblue	200.6065	29.772	0.977567	0.00402	33.306
gi|115546487|dbj|AK231006.1|	brown	213.6194	62.5746	0.933032	0.020593	4.462
gi|115546495|dbj|AK231014.1|	blue	868.779	238.632	0.976526	0.004302	7.13
gi|115546548|dbj|AK234386.1|	grey	159.5457	8.1857	0.871466	0.054238	8.404
gi|115546569|dbj|AK234407.1|	turquoise	826.8496	255.42	0.972686	0.005397	4.046
gi|115546576|dbj|AK234414.1|	green	166.0341	18.3423	0.030734	0.960874	85.82
gi|115546586|dbj|AK234424.1|	turquoise	320.7377	86.1084	0.653107	0.232073	209.138
gi|115546610|dbj|AK234448.1|	tan	956.613	63.5046	0.979963	0.003394	17.49
gi|115546623|dbj|AK234461.1|	red	465.5263	82.7269	0.935321	0.019553	133.084
gi|115546625|dbj|AK234463.1|	yellow	230.7213	76.4264	0.895147	0.04011	1.34
gi|115546628|dbj|AK234466.1|	violet	960.0195	73.621	0.994903	0.000436	9.502
gi|115546638|dbj|AK234476.1|	blue	880.9878	197.494	0.935032	0.019684	12.038
gi|115546656|dbj|AK234494.1|	yellow	144.9791	38.5926	0.720575	0.169682	22.612
gi|115546680|dbj|AK234518.1|	grey	196.9277	11.8213	0.169489	0.785238	104.382
gi|115546695|dbj|AK234533.1|	skyblue	133.0463	21.1203	0.903914	0.035234	107.052
gi|115546696|dbj|AK234534.1|	black	943.7573	98.1844	0.997325	0.000166	21.136
gi|115546753|dbj|AK237599.1|	darkred	241.7379	44.6765	0.99244	0.000788	47.154
gi|115546780|dbj|AK237626.1|	black	964.3285	94.4151	0.988777	0.001425	9.05
gi|115546792|dbj|AK237638.1|	black	950.2154	86.2763	0.971115	0.005868	13.312
gi|115546833|dbj|AK237679.1|	turquoise	411.7345	136.748	0.794284	0.108482	13.712
gi|115546838|dbj|AK237684.1|	blue	847.77	220.193	0.954784	0.011463	19.618
gi|115546849|dbj|AK237695.1|	turquoise	800.4459	248.556	0.963971	0.008165	19.636
gi|115546853|dbj|AK237699.1|	turquoise	437.6772	116.89	0.713627	0.175847	9.37
gi|115546855|dbj|AK237701.1|	blue	828.4999	229.545	0.964528	0.007977	7.67
gi|115546868|dbj|AK237714.1|	salmon	342.2607	39.7479	0.924595	0.024573	17.118
gi|115546917|dbj|AK240563.1|	turquoise	894.1529	249.434	0.96418	0.008094	95.612
gi|115546929|dbj|AK240575.1|	red	606.5861	105.49	0.989104	0.001363	34.718
gi|115546934|dbj|AK240580.1|	pink	244.8137	69.942	0.985007	0.002199	6.408
gi|115546953|dbj|AK240599.1|	blue	870.4838	210.646	0.944108	0.015728	10.15
gi|115546978|dbj|AK231033.1|	brown	240.5538	56.8374	0.889341	0.043448	18.204
gi|115547033|dbj|AK231088.1|	grey60	923.0691	62.4707	0.995597	0.00035	40.222
gi|115547065|dbj|AK231120.1|	green	76.38204	37.6942	0.818845	0.089999	56.944
gi|115547094|dbj|AK231149.1|	black	846.543	91.1403	0.981918	0.002911	15.298
gi|115547117|dbj|AK231172.1|	lightcyan	298.302	43.6231	0.986711	0.001835	27.044
gi|115547124|dbj|AK231179.1|	lightyellow	836.7745	59.1407	0.985512	0.002089	15.838
gi|115547136|dbj|AK231191.1|	salmon	206.1129	35.0655	0.898482	0.038232	9.39

gi|115547142|dbj|AK231197.1|	red	543.0336	109.199	0.996743	0.000223	290.914
gi|115547167|dbj|AK231222.1|	pink	264.1331	50.3171	0.869425	0.055518	4.764
gi|115547176|dbj|AK234549.1|	pink	84.70495	16.5444	0.293475	0.631771	12.538
gi|115547198|dbj|AK234571.1|	magenta	262.2151	81.9494	0.999016	3.71E-05	93.782
gi|115547204|dbj|AK234577.1|	red	679.7887	101.592	0.980033	0.003377	38.958
gi|115547230|dbj|AK234603.1|	green	104.2045	14.0299	0.06414	0.91839	52.162
gi|115547231|dbj|AK234604.1|	turquoise	355.79	121.707	0.771766	0.126313	29.948
gi|115547242|dbj|AK234615.1|	lightcyan	171.4165	11.362	0.455693	0.440552	4.45
gi|115547251|dbj|AK234624.1|	brown	152.5488	35.2737	0.686546	0.200466	8.65
gi|115547260|dbj|AK234633.1|	green	144.088	43.2869	0.886915	0.044868	66.394
gi|115547282|dbj|AK234655.1|	turquoise	571.6228	188.718	0.875824	0.051538	162.252
gi|115547298|dbj|AK234671.1|	yellow	304.1703	112.644	0.982854	0.002688	10.378
gi|115547309|dbj|AK234682.1|	grey60	971.7271	62.8244	0.996742	0.000223	4.054
gi|115547320|dbj|AK234693.1|	turquoise	846.3127	258.377	0.964948	0.007836	37.778
gi|115547329|dbj|AK234702.1|	purple	234.5094	20.0036	-0.82846	0.083057	3.874
gi|115547340|dbj|AK234713.1|	turquoise	792.6436	238.284	0.936407	0.019066	34.074
gi|115547352|dbj|AK234725.1|	darkgrey	197.7737	34.431	0.994419	0.0005	16.86
gi|115547361|dbj|AK234734.1|	blue	807.1884	240.501	0.977654	0.003996	73.678
gi|115547381|dbj|AK237762.1|	turquoise	832.6571	255.197	0.962572	0.008643	28.276
gi|115547397|dbj|AK237778.1|	lightgreen	280.3563	54.1712	0.974246	0.004942	4.154
gi|115547403|dbj|AK237784.1|	black	988.9575	94.2872	0.98825	0.001526	10.368
gi|115547414|dbj|AK237795.1|	skyblue	134.3369	24.2771	0.934058	0.020125	63.48
gi|115547436|dbj|AK237818.1|	salmon	119.5827	30.9916	0.869734	0.055323	23.016
gi|115547445|dbj|AK237827.1|	yellow	249.8617	90.652	0.928341	0.022778	113.398
gi|115547450|dbj|AK237832.1|	pink	268.8571	74.9737	0.996899	0.000207	3.81
gi|115547470|dbj|AK237852.1|	lightgreen	296.5663	60.5769	0.996981	0.000199	5.428
gi|115547485|dbj|AK237867.1|	green	128.4295	48.8709	0.923958	0.024883	63.624
gi|115547488|dbj|AK237870.1|	blue	950.5163	228.86	0.966633	0.00728	27.684
gi|115547522|dbj|AK237904.1|	salmon	180.4767	46.6103	0.970895	0.005934	68.606
gi|115547541|dbj|AK237923.1|	white	940.8706	29.5292	0.806336	0.099282	1362.92
gi|115547575|dbj|AK231233.1|	salmon	122.2765	30.4122	0.862935	0.059647	26.406
gi|115547631|dbj|AK231289.1|	turquoise	314.0808	97.7356	0.684465	0.202395	3.944
gi|115547636|dbj|AK231294.1|	brown	67.21735	18.074	0.313933	0.606954	27.666
gi|115547667|dbj|AK231325.1|	blue	916.6297	254.466	0.992134	0.000836	10.728
gi|115547679|dbj|AK231337.1|	turquoise	893.1742	268.723	0.984111	0.002399	33.386
gi|115547688|dbj|AK231346.1|	brown	173.3179	28.782	0.642805	0.242065	3.834
gi|115547715|dbj|AK231373.1|	yellow	298.3635	115.675	0.991538	0.000933	1.958
gi|115547725|dbj|AK231383.1|	brown	165.2087	46.1502	0.782109	0.118022	3.388
gi|115547754|dbj|AK231412.1|	brown	124.6842	45.0739	0.90012	0.037319	15.974
gi|115547780|dbj|AK234756.1|	magenta	245.8961	75.9633	0.981486	0.003016	2.278
gi|115547797|dbj|AK234773.1|	pink	158.5564	35.5263	0.829706	0.08217	70.322
gi|115547803|dbj|AK234779.1|	tan	878.8946	58.16	0.963613	0.008287	64.818
gi|115547837|dbj|AK234813.1|	darkred	210.9647	38.5036	0.95003	0.013308	2.19
gi|115547849|dbj|AK234825.1|	blue	786.3205	214.686	0.948625	0.01387	18.802
gi|115547856|dbj|AK234832.1|	turquoise	636.548	206.191	0.8836	0.046831	9.67
gi|115547861|dbj|AK234837.1|	turquoise	465.1628	155.377	0.81035	0.096273	25.77
gi|115547869|dbj|AK234845.1|	turquoise	785.908	249.821	0.938797	0.018008	5.402
gi|115547882|dbj|AK234858.1|	pink	260.9985	49.8052	0.885208	0.045875	31.138

gi|115547885|dbj|AK234861.1|	blue	837.5793	246.203	0.983832	0.002462	24.06
gi|115547886|dbj|AK234862.1|	salmon	134.5117	42.387	0.947235	0.014434	13.128
gi|115547910|dbj|AK234886.1|	blue	897.7562	245.525	0.981796	0.00294	13.75
gi|115547932|dbj|AK234909.1|	skyblue	193.5653	28.3261	0.96578	0.00756	58.084
gi|115547985|dbj|AK237969.1|	lightgreen	292.2377	60.4715	0.996599	0.000238	21.056
gi|115548000|dbj|AK237984.1|	pink	157.5735	44.6556	0.88067	0.048588	21.44
gi|115548002|dbj|AK237986.1|	turquoise	458.9062	152.636	0.79921	0.104692	240.994
gi|115548018|dbj|AK238002.1|	blue	710.4827	136.22	0.850235	0.06799	12.28
gi|115548040|dbj|AK238024.1|	yellow	304.1454	114.138	0.985969	0.001991	6.882
gi|115548067|dbj|AK238051.1|	magenta	242.79	75.5173	0.979924	0.003404	0.838
gi|115548072|dbj|AK238056.1|	yellowgreen	151.1501	13.4218	0.915506	0.029106	28.904
gi|115548078|dbj|AK238062.1|	turquoise	711.5019	232.748	0.914911	0.029412	6.252
gi|115548084|dbj|AK238068.1|	darkred	231.1805	43.0626	0.980214	0.003331	1.974
gi|115548129|dbj|AK238113.1|	lightgreen	284.6887	55.2378	0.978232	0.003843	1.348
gi|115548136|dbj|AK238120.1|	magenta	261.5381	82.2245	0.999435	1.61E-05	4.538
gi|115548176|dbj|AK234948.1|	darkred	223.5542	42.1033	0.972725	0.005385	288.41
gi|115548199|dbj|AK231509.1|	lightyellow	976.7888	63.0267	0.998349	8.05E-05	3.03
gi|115548219|dbj|AK231529.1|	salmon	279.3737	43.6617	0.948602	0.01388	34.414
gi|115548236|dbj|AK231450.1|	salmon	168.4087	39.4309	0.928426	0.022738	10.44
gi|115548239|dbj|AK231453.1|	turquoise	278.4469	74.775	0.635921	0.248804	3.7
gi|115548250|dbj|AK231464.1|	turquoise	250.1933	56.272	0.519921	0.369185	1.102
gi|115548260|dbj|AK231474.1|	pink	268.12	74.5955	0.992336	0.000805	2.906
gi|115548298|dbj|AK234955.1|	turquoise	618.3075	169.88	0.853635	0.065723	12.406
gi|115548312|dbj|AK234969.1|	darkred	246.3942	40.1709	0.971182	0.005847	2.794
gi|115548314|dbj|AK234971.1|	turquoise	472.649	140.008	0.784258	0.11632	29.092
gi|115548316|dbj|AK234973.1|	salmon	213.5464	47.154	0.972704	0.005391	31.104
gi|115548319|dbj|AK231543.1|	purple	168.4467	14.8038	0.749176	0.144988	35.766
gi|115548323|dbj|AK231696.1|	royalblue	244.0151	19.6236	0.863031	0.059585	3.174
gi|115548328|dbj|AK231701.1|	tan	968.0973	62.1502	0.97545	0.0046	16.754
gi|115548334|dbj|AK231707.1|	darkmagenta	907.4808	25.3476	0.981546	0.003001	32.212
gi|115548360|dbj|AK231733.1|	turquoise	945.3265	265.344	0.985177	0.002161	83.992
gi|115548373|dbj|AK234990.1|	yellow	236.7548	87.2751	0.920286	0.026692	1.904
gi|115548383|dbj|AK235000.1|	navy	315.6853	28.7249	0.842823	0.073014	18.1
gi|115548388|dbj|AK235005.1|	darkturquoise	199.9848	29.4568	0.953033	0.012132	52.566
gi|115548389|dbj|AK235006.1|	greenyellow	177.5431	51.3602	0.980201	0.003334	12.686
gi|115548394|dbj|AK235011.1|	turquoise	843.3251	229.613	0.943713	0.015895	29.02
gi|115548406|dbj|AK235023.1|	lightgreen	290.8829	60.4517	0.996508	0.000248	1.884
gi|115548409|dbj|AK235026.1|	blue	841.546	227.462	0.964851	0.007869	25.496
gi|115548413|dbj|AK235030.1|	pink	251.1565	51.4356	0.887242	0.044676	4.938
gi|115548441|dbj|AK235058.1|	turquoise	594.0615	189.104	0.888743	0.043797	81.064
gi|115548454|dbj|AK235071.1|	pink	261.0986	55.2476	0.903136	0.035658	10.506
gi|115548459|dbj|AK235076.1|	yellow	277.4683	104.491	0.965394	0.007687	4.614
gi|115548467|dbj|AK235084.1|	blue	812.3443	232.714	0.967234	0.007085	153.802
gi|115548500|dbj|AK235117.1|	turquoise	493.9496	105.279	0.736119	0.156121	68.666
gi|115548514|dbj|AK235131.1|	grey60	954.5899	62.9605	0.99719	0.000179	81.826
gi|115548519|dbj|AK235136.1|	brown	86.75561	25.6935	0.479767	0.41346	10.68
gi|115548520|dbj|AK235137.1|	navy	158.5952	11.6243	-0.49351	0.398175	38.198
gi|115548526|dbj|AK235143.1|	turquoise	906.1215	267.172	0.980388	0.003287	20.914

gi|115548564|dbj|AK231548.1|	greenyellow	151.6976	45.648	0.955925	0.011034	108.752
gi|115548572|dbj|AK231556.1|	black	857.8768	82.1847	0.961473	0.009025	52.83
gi|115548574|dbj|AK231558.1|	red	631.6646	105.145	0.987978	0.001579	659.306
gi|115548620|dbj|AK231604.1|	red	623.1132	93.4894	0.961142	0.009141	59.15
gi|115548658|dbj|AK231642.1|	white	954.4238	29.6026	0.812959	0.094333	117.946
gi|115548698|dbj|AK231683.1|	pink	160.3433	48.6355	0.902857	0.035811	75.034
gi|115548706|dbj|AK231691.1|	white	966.8351	29.591	0.801982	0.102577	79.55
gi|115548717|dbj|AK231783.1|	blue	751.9026	224.225	0.957692	0.01038	624.288
gi|115548727|dbj|AK231793.1|	paleturquoise	152.8818	15.0875	0.882949	0.04722	16.66
gi|115548749|dbj|AK231815.1|	yellow	308.9098	116.761	0.992666	0.000753	1.524
gi|115548797|dbj|AK231755.1|	blue	397.2048	108.018	0.799763	0.104269	42.66
gi|115548800|dbj|AK231758.1|	grey	145.2098	7.437	-0.81405	0.093524	43.676
gi|115548801|dbj|AK231759.1|	green	132.7411	55.9277	0.97414	0.004973	39.982
gi|115548802|dbj|AK231760.1|	royalblue	346.4589	32.1526	0.99111	0.001005	4.25
gi|115548807|dbj|AK231765.1|	green	134.9466	53.362	0.962254	0.008753	23.578
gi|115548828|dbj|AK231943.1|	darkgrey	92.20749	11.8229	0.736261	0.155998	10.808
gi|115548831|dbj|AK231946.1|	blue	753.5197	144.778	0.865282	0.058143	21.198
gi|115548849|dbj|AK235252.1|	red	449.3912	83.2272	0.936758	0.018909	19.668
gi|115548852|dbj|AK235255.1|	black	862.3985	90.056	0.979475	0.003519	45.962
gi|115548861|dbj|AK235264.1|	turquoise	930.1558	272.171	0.99066	0.001082	12.358
gi|115548869|dbj|AK235272.1|	magenta	231.1298	65.1831	0.94982	0.013392	1.936
gi|115548882|dbj|AK235285.1|	sienna3	996.3204	25.4324	0.994685	0.000465	116.038
gi|115548922|dbj|AK235325.1|	lightyellow	998.988	59.7048	0.987314	0.001712	249.932
gi|115548932|dbj|AK235335.1|	greenyellow	158.3638	43.6342	0.939069	0.017889	36.414
gi|115548933|dbj|AK235336.1|	blue	604.4051	159.475	0.888773	0.043779	15.342
gi|115548942|dbj|AK235345.1|	grey60	855.0403	58.3252	0.981567	0.002996	12.862
gi|115548954|dbj|AK235357.1|	violet	994.8504	70.7411	0.986701	0.001837	12.426
gi|115548955|dbj|AK235358.1|	blue	966.8243	228.748	0.969454	0.006379	69.436
gi|115548956|dbj|AK235359.1|	salmon	111.7009	24.7117	0.809755	0.096717	11.132
gi|115548992|dbj|AK238181.1|	navy	234.7612	24.698	0.686987	0.200057	1.166
gi|115549006|dbj|AK238195.1|	darkturquoise	186.2899	34.294	0.990652	0.001083	1.142
gi|115549015|dbj|AK238204.1|	blue	773.951	195.958	0.930729	0.021657	2.036
gi|115549057|dbj|AK238246.1|	blue	674.9137	200.39	0.935135	0.019637	20.384
gi|115549078|dbj|AK238267.1|	blue	870.9231	240.944	0.976039	0.004436	40.996
gi|115549145|dbj|AK238334.1|	violet	892.8452	72.0224	0.990452	0.001118	6.634
gi|115549177|dbj|AK231853.1|	navy	260.9165	33.6693	0.883428	0.046934	25.522
gi|115549179|dbj|AK231855.1|	turquoise	691.1547	226.283	0.901855	0.036361	19.92
gi|115549199|dbj|AK231875.1|	midnightblue	94.91706	36.36	0.94485	0.015418	3.252
gi|115549229|dbj|AK231905.1|	blue	945.0469	208.274	0.948127	0.014071	34.112
gi|115549239|dbj|AK231915.1|	blue	759.0744	221.676	0.959548	0.009707	64.518
gi|115549243|dbj|AK231919.1|	yellow	225.0531	71.1579	0.88306	0.047153	38.602
gi|115549251|dbj|AK231927.1|	lightgreen	261.8019	55.2131	0.978086	0.003881	1.202
gi|115549258|dbj|AK235178.1|	blue	501.8225	124.261	0.82831	0.083164	18.052
gi|115549275|dbj|AK235196.1|	purple	191.6127	18.2567	-0.90128	0.036677	20.604
gi|115549299|dbj|AK235220.1|	darkgreen	203.657	31.3202	0.935871	0.019306	58.818
gi|115549311|dbj|AK235232.1|	brown	161.23	54.3468	0.910591	0.031659	17.828
gi|115549313|dbj|AK235234.1|	turquoise	345.5065	112.105	0.722802	0.167718	133.966
gi|115549331|dbj|AK232109.1|	green	96.98978	52.838	0.94875	0.01382	259.438

gi|115549349|dbj|AK232127.1|	magenta	242.5931	74.1946	0.976704	0.004253	72.732
gi|115549356|dbj|AK235378.1|	green	79.69863	39.1105	0.837336	0.076803	15.294
gi|115549358|dbj|AK235380.1|	purple	92.72455	14.7511	0.924156	0.024786	20.488
gi|115549368|dbj|AK235390.1|	blue	811.1277	240.5	0.976251	0.004378	11.026
gi|115549412|dbj|AK235434.1|	red	381.5619	91.2418	0.957197	0.010562	37.732
gi|115549416|dbj|AK235438.1|	darkred	261.3548	43.4147	0.987103	0.001755	5.608
gi|115549428|dbj|AK235450.1|	navy	202.2337	28.4345	0.779016	0.120484	114.948
gi|115549440|dbj|AK235462.1|	pink	114.7023	15.9667	0.520745	0.368288	185.522
gi|115549451|dbj|AK235473.1|	blue	711.9039	144.325	0.861307	0.060697	8.32
gi|115549474|dbj|AK235496.1|	tan	929.1246	67.1243	0.992529	0.000774	17.24
gi|115549475|dbj|AK235497.1|	purple	134.0766	18.6288	0.88781	0.044342	86.376
gi|115549482|dbj|AK235504.1|	darkturquoise	202.1029	34.8602	0.996331	0.000267	3.16
gi|115549486|dbj|AK235508.1|	turquoise	602.2265	196.354	0.886757	0.044961	10.304
gi|115549499|dbj|AK235521.1|	blue	622.8935	187.931	0.919029	0.02732	14.9
gi|115549531|dbj|AK235553.1|	blue	452.9185	92.012	0.767906	0.129449	71.346
gi|115549536|dbj|AK235558.1|	lightyellow	989.9905	62.6525	0.99711	0.000186	9.398
gi|115549537|dbj|AK235559.1|	turquoise	270.0394	90.7031	0.661759	0.223773	13.35
gi|115549542|dbj|AK235564.1|	red	444.1723	100.375	0.97825	0.003838	14.096
gi|115549562|dbj|AK238370.1|	yellow	261.3789	97.8563	0.949622	0.01347	4.296
gi|115549583|dbj|AK238391.1|	magenta	242.0406	72.5592	0.971415	0.005777	4.038
gi|115549608|dbj|AK238416.1|	turquoise	924.8418	266.387	0.981034	0.003126	73.748
gi|115549613|dbj|AK238421.1|	brown	184.3989	25.5085	0.581893	0.303355	20.89
gi|115549680|dbj|AK231959.1|	red	478.4255	105.566	0.989193	0.001347	34.546
gi|115549689|dbj|AK231968.1|	green	106.8809	53.2789	0.953204	0.012066	71.21
gi|115549696|dbj|AK231975.1|	blue	924.1589	238.496	0.97457	0.004849	24.328
gi|115549709|dbj|AK231988.1|	blue	725.8977	194.568	0.925786	0.023998	14.938
gi|115549710|dbj|AK231989.1|	yellow	178.0537	49.2457	0.778286	0.121067	15.528
gi|115549744|dbj|AK232023.1|	grey	157.8776	14.0708	-0.91419	0.029782	353.304
gi|115549779|dbj|AK232059.1|	black	931.783	95.8576	0.991989	0.00086	16.086
gi|115549787|dbj|AK232067.1|	skyblue	285.1381	30.7526	0.981894	0.002917	102.95
gi|115549809|dbj|AK232089.1|	blue	941.1333	219.263	0.956314	0.010889	27.454
gi|115549835|dbj|AK238495.1|	lightcyan	130.0041	13.4058	0.740986	0.151943	8.206
gi|115549846|dbj|AK238506.1|	blue	679.7605	166.469	0.889174	0.043545	50.642
gi|115549856|dbj|AK238516.1|	turquoise	509.2793	171.646	0.806416	0.099222	23.284
gi|115549861|dbj|AK238521.1|	violet	943.4591	74.2854	0.996718	0.000226	5.836
gi|115549895|dbj|AK238555.1|	blue	889.1014	203.115	0.938955	0.017939	11.572
gi|115549900|dbj|AK232134.1|	blue	916.1949	216.176	0.95257	0.012311	52.966
gi|115549949|dbj|AK232184.1|	turquoise	500.8335	130.432	0.768594	0.128889	147.104
gi|115549956|dbj|AK232191.1|	yellow	307.2455	119.093	0.99834	8.11E-05	4.36
gi|115549963|dbj|AK232198.1|	turquoise	912.3992	269.988	0.990629	0.001088	46.164
gi|115549970|dbj|AK232205.1|	tan	982.083	66.0234	0.989119	0.00136	120.272
gi|115549997|dbj|AK232232.1|	navy	159.9458	34.1656	0.935271	0.019576	1.826
gi|115550002|dbj|AK232237.1|	yellow	286.0176	108.05	0.974162	0.004966	93.578
gi|115550007|dbj|AK232242.1|	navy	198.2592	10.0732	-0.43974	0.45871	165.38
gi|115550036|dbj|AK232271.1|	grey60	974.7402	61.1683	0.991363	0.000962	100.97
gi|115550039|dbj|AK232274.1|	greenyellow	239.4053	39.9682	0.910244	0.031842	58.802
gi|115550042|dbj|AK232277.1|	turquoise	903.8186	264.204	0.977898	0.003931	65.692
gi|115550054|dbj|AK232289.1|	brown	205.4739	61.8998	0.885803	0.045523	8.854

gi|115550058|dbj|AK232293.1|	yellow	270.3195	101.497	0.956105	0.010967	11.884
gi|115550060|dbj|AK232295.1|	brown	58.41663	20.1914	0.44089	0.457399	45.516
gi|115550075|dbj|AK232310.1|	yellow	301.2688	111.843	0.980687	0.003212	3.592
gi|115550091|dbj|AK232326.1|	turquoise	801.173	245.614	0.964541	0.007973	2.308
gi|115550101|dbj|AK235580.1|	darkorange	171.9391	18.892	0.924166	0.024782	22.946
gi|115550102|dbj|AK235581.1|	pink	226.2885	48.5423	0.870174	0.055047	6.71
gi|115550120|dbj|AK235599.1|	lightyellow	993.3209	62.4218	0.996378	0.000261	28.626
gi|115550124|dbj|AK235603.1|	lightcyan	296.4573	43.6132	0.982133	0.002859	398.826
gi|115550125|dbj|AK235604.1|	magenta	212.3018	61.5493	0.93631	0.019109	3.838
gi|115550130|dbj|AK235609.1|	pink	260.7483	63.2365	0.942504	0.016406	5.992
gi|115550133|dbj|AK235612.1|	turquoise	888.6248	265.155	0.979319	0.003559	93.032
gi|115550140|dbj|AK235619.1|	brown	170.839	54.7579	0.857802	0.062978	44.328
gi|115550155|dbj|AK235634.1|	blue	860.7678	242.939	0.979336	0.003555	28.85
gi|115550159|dbj|AK235638.1|	turquoise	639.7739	183.474	0.886901	0.044876	41.044
gi|115550165|dbj|AK235644.1|	turquoise	712.8653	223.043	0.933972	0.020164	47.054
gi|115550185|dbj|AK235664.1|	blue	859.4541	223.899	0.963245	0.008412	14.786
gi|115550186|dbj|AK235665.1|	blue	823.2751	232.544	0.973	0.005304	26.35
gi|115550191|dbj|AK235670.1|	violet	894.314	73.2338	0.993754	0.000592	13.352
gi|115550210|dbj|AK235689.1|	navy	332.4593	28.8949	0.796384	0.106862	19.652
gi|115550254|dbj|AK235733.1|	tan	628.3503	43.8202	0.904887	0.034706	29.846
gi|115550257|dbj|AK235736.1|	navy	186.2613	26.2964	0.745424	0.148162	31.158
gi|115550270|dbj|AK235749.1|	brown	151.5569	41.1752	0.76726	0.129977	5.11
gi|115550272|dbj|AK235751.1|	blue	827.501	241.26	0.978839	0.003683	71.904
gi|115550273|dbj|AK235752.1|	turquoise	666.4311	213.115	0.89529	0.040029	20.882
gi|115550292|dbj|AK235771.1|	royalblue	167.2402	5.95102	0.322061	0.597143	41.182
gi|115550321|dbj|AK238586.1|	tan	971.165	58.4439	0.962695	0.008601	29.312
gi|115550332|dbj|AK238597.1|	white	970.6104	29.2709	0.801486	0.102954	87.356
gi|115550354|dbj|AK238619.1|	orange	348.3342	22.1081	0.896843	0.039151	92.03
gi|115550357|dbj|AK238622.1|	violet	940.9664	74.2577	0.996635	0.000234	47.656
gi|115550392|dbj|AK238657.1|	darkgrey	194.5203	31.9121	0.973001	0.005304	16.404
gi|115550394|dbj|AK238659.1|	yellow	242.1995	81.6177	0.913878	0.029944	12.71
gi|115550410|dbj|AK238675.1|	greenyellow	246.3131	38.4086	0.907564	0.033264	4.112
gi|115550429|dbj|AK238694.1|	blue	812.7665	214.733	0.946906	0.014568	32.736
gi|115550430|dbj|AK238695.1|	greenyellow	172.3416	51.0166	0.980568	0.003242	20.708
gi|115550441|dbj|AK238706.1|	darkgrey	180.073	33.294	0.98272	0.00272	12.546
gi|115550443|dbj|AK238708.1|	turquoise	937.6827	269.198	0.986133	0.001956	14.814
gi|115550448|dbj|AK238713.1|	green	87.64738	47.5935	0.89285	0.04142	54.756
gi|115550486|dbj|AK238751.1|	royalblue	77.28413	4.39888	0.354252	0.558572	2.502
gi|115550510|dbj|AK232340.1|	pink	266.302	74.8206	0.992469	0.000784	5.184
gi|115550524|dbj|AK232354.1|	midnightblue	140.3301	44.0642	0.979447	0.003526	51.776
gi|115550537|dbj|AK232367.1|	turquoise	743.5039	214.464	0.930248	0.021881	15.042
gi|115550567|dbj|AK232397.1|	green	92.41745	38.8658	0.847769	0.069649	13.95
gi|115550593|dbj|AK232423.1|	green	94.29774	42.7241	0.853552	0.065778	112.958
gi|115550595|dbj|AK232425.1|	blue	838.7341	238.401	0.977556	0.004023	7.578
gi|115550607|dbj|AK232437.1|	green	131.1045	43.1649	0.888398	0.043998	51.112
gi|115550650|dbj|AK232480.1|	yellow	268.5106	92.1955	0.943694	0.015902	0.858
gi|115550656|dbj|AK232486.1|	blue	879.4224	251.236	0.986924	0.001791	50.32
gi|115550661|dbj|AK232491.1|	greenyellow	85.32788	3.37045	0.379788	0.528329	9.178

gi|115550668|dbj|AK232498.1|	lightyellow	993.6362	61.5109	0.993372	0.000647	6.636
gi|115550708|dbj|AK235782.1|	red	382.2551	90.5215	0.956063	0.010982	61.082
gi|115550716|dbj|AK235790.1|	blue	473.5696	137.549	0.852966	0.066167	7.454
gi|115550732|dbj|AK235806.1|	magenta	277.1026	76.521	0.983326	0.002578	19.134
gi|115550746|dbj|AK235820.1|	brown	146.6268	49.5569	0.875053	0.052013	6.298
gi|115550755|dbj|AK235829.1|	blue	745.3742	207.903	0.94505	0.015335	30.008
gi|115550758|dbj|AK235833.1|	turquoise	716.1467	196.067	0.905068	0.034608	33.16
gi|115550768|dbj|AK235843.1|	blue	585.7865	141.108	0.858639	0.062431	25.462
gi|115550785|dbj|AK235860.1|	skyblue	385.2774	24.3818	0.923699	0.025009	32.598
gi|115550806|dbj|AK235881.1|	blue	792.2042	236.329	0.972502	0.005451	24.862
gi|115550812|dbj|AK235887.1|	black	917.6768	76.4815	0.946994	0.014532	7.918
gi|115550819|dbj|AK235894.1|	grey60	981.1711	59.9666	0.987209	0.001733	16.17
gi|115550826|dbj|AK235901.1|	purple	154.6908	14.4257	-0.58352	0.301675	10.726
gi|115550838|dbj|AK235913.1|	orange	506.2526	34.0528	0.989849	0.001226	96.458
gi|115550848|dbj|AK235923.1|	magenta	257.2871	77.1137	0.98658	0.001862	226.978
gi|115550868|dbj|AK235943.1|	turquoise	866.0266	252.042	0.974367	0.004908	10.982
gi|115550874|dbj|AK235949.1|	turquoise	542.5831	137.656	0.780638	0.119191	28.276
gi|115550888|dbj|AK235963.1|	green	149.5765	20.5744	0.202997	0.743322	6.348
gi|115550889|dbj|AK235964.1|	yellowgreen	173.1299	18.5715	0.996117	0.00029	99.902
gi|115550900|dbj|AK238761.1|	salmon	137.109	40.0767	0.931376	0.021356	23.988
gi|115550915|dbj|AK238776.1|	turquoise	809.9299	256.968	0.951393	0.01277	5
gi|115550943|dbj|AK238804.1|	white	181.3868	23.9347	0.785164	0.115606	20.094
gi|115550946|dbj|AK238807.1|	black	925.8152	85.3792	0.968754	0.006599	29.456
gi|115550960|dbj|AK238821.1|	salmon	174.5931	34.5304	0.899028	0.037927	11.638
gi|115550961|dbj|AK238822.1|	turquoise	728.9321	237.557	0.926679	0.023569	40.53
gi|115550973|dbj|AK238834.1|	yellow	237.8177	80.4806	0.903986	0.035195	4.23
gi|115550975|dbj|AK238836.1|	blue	959.7864	230.688	0.969811	0.006268	135.068
gi|115550976|dbj|AK238837.1|	turquoise	915.2142	262.846	0.977016	0.004168	32.328
gi|115550999|dbj|AK238860.1|	royalblue	371.8338	33.1956	0.998834	4.78E-05	33.63
gi|115551001|dbj|AK238862.1|	turquoise	269.2456	63.6128	0.514075	0.375557	255.718
gi|115551006|dbj|AK238867.1|	tan	971.5925	66.8404	0.991471	0.000944	13.51
gi|115551027|dbj|AK238888.1|	darkred	224.4293	32.3476	0.919933	0.026867	14.12
gi|115551063|dbj|AK238924.1|	navy	94.89498	7.75132	0.328591	0.58928	32.512
gi|115551074|dbj|AK238935.1|	grey	163.8936	13.7543	-0.91695	0.02837	12.158
gi|115551086|dbj|AK238947.1|	lightgreen	289.7937	58.9529	0.991397	0.000957	1.67
gi|115551105|dbj|AK232538.1|	turquoise	843.7329	252.679	0.96962	0.006328	26.33
gi|115551122|dbj|AK232555.1|	pink	87.01946	24.8745	0.639572	0.245224	64.684
gi|115551124|dbj|AK232557.1|	red	392.1143	78.9708	0.926014	0.023888	10.42
gi|115551148|dbj|AK232581.1|	paleturquoise	136.4001	16.5299	0.904667	0.034825	39.002
gi|115551177|dbj|AK232610.1|	yellowgreen	146.6702	13.0903	0.917571	0.028055	35.448
gi|115551181|dbj|AK232614.1|	turquoise	623.366	204.538	0.892884	0.041401	53.052
gi|115551189|dbj|AK232622.1|	black	948.1935	96.899	0.994113	0.000542	16.154
gi|115551213|dbj|AK232646.1|	violet	981.0275	74.1176	0.996227	0.000278	8.91
gi|115551216|dbj|AK232649.1|	turquoise	430.5099	109.16	0.721794	0.168607	27.212
gi|115551255|dbj|AK232688.1|	grey60	839.5711	57.3831	0.978333	0.003816	15.008
gi|115551270|dbj|AK232704.1|	lightyellow	972.6258	63.2578	0.999097	3.26E-05	1533.93
gi|115551283|dbj|AK232717.1|	pink	185.9861	2.62084	-0.70383	0.184647	79.16
gi|115551304|dbj|AK235982.1|	lightyellow	979.2843	63.1712	0.9988	4.99E-05	12.236

gi|115551308|dbj|AK235986.1|	salmon	193.2072	31.9975	0.871123	0.054453	15.138
gi|115551350|dbj|AK236028.1|	blue	662.8035	154.667	0.881087	0.048337	10.688
gi|115551358|dbj|AK236036.1|	grey	287.5464	12.7862	0.965976	0.007495	12.868
gi|115551374|dbj|AK236052.1|	turquoise	485.0761	128.07	0.752134	0.1425	24.182
gi|115551375|dbj|AK236053.1|	turquoise	425.9409	137.533	0.801169	0.103196	6.346
gi|115551387|dbj|AK236065.1|	red	550.6787	87.8012	0.948398	0.013962	29.51
gi|115551391|dbj|AK236069.1|	tan	887.6143	66.6943	0.991716	0.000904	16.896
gi|115551397|dbj|AK236075.1|	blue	972.6207	232.192	0.971427	0.005773	68.722
gi|115551408|dbj|AK236086.1|	darkorange	208.7962	19.5731	0.986333	0.001914	151.916
gi|115551416|dbj|AK236094.1|	turquoise	736.9507	240.262	0.922726	0.025485	30.264
gi|115551438|dbj|AK236116.1|	blue	796.5224	236.875	0.974717	0.004807	29.028
gi|115551444|dbj|AK236122.1|	navy	355.0456	26.5507	0.796479	0.106789	6.238
gi|115551449|dbj|AK236127.1|	blue	794.7314	197.086	0.929984	0.022005	135.572
gi|115551452|dbj|AK236130.1|	white	916.477	29.5902	0.833188	0.079707	13.808
gi|115551471|dbj|AK236149.1|	blue	822.0582	236.288	0.973519	0.005152	21.19
gi|115551473|dbj|AK236151.1|	darkorange	156.8336	9.23945	0.502598	0.38814	7.526
gi|115551475|dbj|AK236153.1|	sienna3	972.7382	25.8454	0.997887	0.000117	138.358
gi|115551476|dbj|AK236154.1|	white	911.1978	29.2219	0.813754	0.093744	20.49
gi|115551480|dbj|AK236158.1|	white	201.0878	21.1434	0.833852	0.079241	5.588
gi|115551481|dbj|AK236159.1|	black	980.7694	96.0311	0.992104	0.000841	16.602
gi|115551482|dbj|AK236160.1|	paleturquoise	171.21	20.9865	0.98299	0.002656	29.776
gi|115551497|dbj|AK238961.1|	orange	329.9817	32.4664	0.977855	0.003943	100.144
gi|115551518|dbj|AK238982.1|	pink	211.8217	65.1324	0.974081	0.00499	43.332
gi|115551521|dbj|AK238985.1|	yellow	305.4764	112.325	0.98216	0.002853	161.28
gi|115551534|dbj|AK238998.1|	purple	117.991	20.8816	0.817759	0.090795	72.794
gi|115551587|dbj|AK239051.1|	midnightblue	115.4485	39.4177	0.956426	0.010847	22.622
gi|115551605|dbj|AK239069.1|	turquoise	375.5809	99.5425	0.666818	0.218958	13.942
gi|115551606|dbj|AK239070.1|	orange	463.0931	35.1127	0.996433	0.000256	11.86
gi|115551623|dbj|AK239087.1|	yellow	234.0266	79.4041	0.908494	0.032768	5.708
gi|115551627|dbj|AK239091.1|	turquoise	309.8219	83.6485	0.607872	0.276768	0.628
gi|115551631|dbj|AK239095.1|	navy	165.925	10.7372	-0.68887	0.198313	4.168
gi|115551640|dbj|AK239104.1|	tan	868.7177	60.4809	0.972055	0.005584	36.388
gi|115551642|dbj|AK239106.1|	red	449.2596	102.112	0.982038	0.002882	60.56
gi|115551652|dbj|AK239116.1|	turquoise	369.971	99.4844	0.662606	0.222965	178.732
gi|115551691|dbj|AK230600.1|	darkturquoise	210.3272	24.7877	0.913578	0.030099	18.848
gi|115551695|dbj|AK232156.1|	blue	834.4064	239.414	0.975038	0.004717	27.348
gi|115551776|dbj|AK236182.1|	brown	188.1621	29.7717	0.677614	0.208781	204.35
gi|115551783|dbj|AK236189.1|	lightyellow	865.0456	60.4947	0.990087	0.001183	13.904
gi|115551790|dbj|AK236196.1|	darkmagenta	967.2083	24.9794	0.978588	0.003749	23.924
gi|115551828|dbj|AK236235.1|	tan	946.0678	61.1264	0.971961	0.005612	9.444
gi|115551842|dbj|AK236249.1|	black	925.5482	95.2864	0.991101	0.001006	14.344
gi|115551862|dbj|AK236269.1|	lightcyan	267.8451	40.305	0.971181	0.005847	3.204
gi|115551864|dbj|AK236271.1|	grey60	835.9394	58.3089	0.98157	0.002995	5.462
gi|115551880|dbj|AK236287.1|	blue	953.7936	225.068	0.963659	0.008271	12.832
gi|115551881|dbj|AK236288.1|	yellow	231.8423	76.401	0.901269	0.036684	3.248
gi|115551894|dbj|AK236301.1|	turquoise	757.4298	244.629	0.930804	0.021622	11.856
gi|115551903|dbj|AK236310.1|	turquoise	722.0981	223.248	0.943108	0.01615	30.138
gi|115551909|dbj|AK236316.1|	darkred	265.896	39.3953	0.968398	0.006712	1.26

gi|115551914|dbj|AK236321.1|	turquoise	278.7328	68.0264	0.526198	0.362373	14.896
gi|115551917|dbj|AK236324.1|	turquoise	669.155	215.295	0.905314	0.034474	134.988
gi|115551923|dbj|AK236330.1|	magenta	244.2477	76.2672	0.982128	0.00286	3.43
gi|115551932|dbj|AK236339.1|	tan	954.0436	68.7753	0.998495	7.00E-05	96.72
gi|115551933|dbj|AK236340.1|	darkorange	186.8235	9.07866	0.566118	0.319801	31.002
gi|115551938|dbj|AK236345.1|	turquoise	469.5843	109.47	0.712632	0.176735	33.034
gi|115551942|dbj|AK236349.1|	yellow	302.7034	116.151	0.991252	0.000981	3.526
gi|115551948|dbj|AK236355.1|	darkgrey	214.8812	30.8363	0.970408	0.006084	11.422
gi|115551969|dbj|AK239162.1|	blue	799.6334	239.065	0.975517	0.004582	6.372
gi|115551993|dbj|AK239186.1|	navy	87.36436	6.05931	0.069273	0.91187	21.418
gi|115551997|dbj|AK239190.1|	blue	793.351	177.117	0.912603	0.030605	3.266
gi|115552025|dbj|AK239218.1|	lightgreen	300.6733	60.0005	0.995	0.000424	7.096
gi|115552029|dbj|AK239222.1|	turquoise	783.2297	251.356	0.945372	0.015201	25.136
gi|115552039|dbj|AK239232.1|	turquoise	317.1675	75.3947	0.578632	0.306736	8.03
gi|115552041|dbj|AK239234.1|	pink	315.6388	47.9872	0.849695	0.068352	7.672
gi|115552061|dbj|AK239254.1|	grey60	940.2921	62.8131	0.996661	0.000232	7.26
gi|115552064|dbj|AK239257.1|	turquoise	600.0853	149.057	0.832505	0.080188	30.608
gi|115552079|dbj|AK239272.1|	magenta	209.1768	67.569	0.955731	0.011107	5.856
gi|115552089|dbj|AK239282.1|	blue	787.8074	225.366	0.959206	0.00983	49.65
gi|115552097|dbj|AK239290.1|	blue	822.9204	191.795	0.924534	0.024603	36.76
gi|115552101|dbj|AK239294.1|	blue	712.0932	168.428	0.895372	0.039983	23.936
gi|115552119|dbj|AK239312.1|	royalblue	256.8168	14.6426	0.729947	0.161466	6.958
gi|115552139|dbj|AK239332.1|	turquoise	619.9254	191.203	0.885837	0.045503	12.496
gi|115552153|dbj|AK239346.1|	black	972.5342	98.8896	0.99867	5.82E-05	10.284
gi|115552177|dbj|AK230486.1|	midnightblue	80.13405	5.46155	-0.1822	0.769305	1883.564
gi|115552179|dbj|AK230488.1|	turquoise	311.3958	100.748	0.647111	0.237875	13.1
gi|115552191|dbj|AK230500.1|	orange	594.0747	31.175	0.968266	0.006754	24.222
gi|115552195|dbj|AK230504.1|	turquoise	614.2287	177.77	0.870967	0.05455	33.424
gi|115552216|dbj|AK230525.1|	paleturquoise	160.0631	20.0838	0.971807	0.005659	10.184
gi|115552220|dbj|AK230529.1|	purple	130.2814	8.98439	-0.84073	0.074455	16.2
gi|115552234|dbj|AK230543.1|	darkred	200.4863	34.3549	0.925642	0.024067	1.972
gi|115552246|dbj|AK230555.1|	blue	601.8463	138.526	0.850182	0.068025	6.198
gi|115552250|dbj|AK230559.1|	red	346.8277	77.0967	0.919953	0.026858	56.238
gi|115552260|dbj|AK230569.1|	navy	198.4393	10.6189	-0.62701	0.2576	43.896
gi|115552269|dbj|AK230578.1|	blue	495.1088	103.229	0.793374	0.109187	20.638
gi|115552272|dbj|AK230581.1|	lightcyan	143.1928	18.8872	0.794931	0.107982	23.684
gi|115552281|dbj|AK230590.1|	navy	144.4289	40.3466	0.94713	0.014477	22.134
gi|115552282|dbj|AK230591.1|	blue	887.2355	244.386	0.980904	0.003159	93.892
gi|115552295|dbj|AK230605.1|	grey60	946.7071	60.674	0.989546	0.001281	17.05
gi|115552309|dbj|AK230619.1|	turquoise	435.1014	144.748	0.757608	0.137928	164.238
gi|115552311|dbj|AK230621.1|	turquoise	195.2144	53.9389	0.544467	0.342726	28.264
gi|115552314|dbj|AK230624.1|	blue	776.4265	225.746	0.960792	0.009265	50.232
gi|115552353|dbj|AK232758.1|	blue	643.2168	190.74	0.924738	0.024504	9.188
gi|115552359|dbj|AK232764.1|	turquoise	866.5594	266.042	0.969557	0.006347	29.106
gi|115552369|dbj|AK236374.1|	blue	729.5315	222.355	0.957153	0.010578	9.59
gi|115552393|dbj|AK236398.1|	magenta	266.6278	79.5914	0.991853	0.000882	7.458
gi|115552399|dbj|AK236404.1|	sienna3	965.8294	25.9492	0.998704	5.60E-05	26.816
gi|115552409|dbj|AK236414.1|	turquoise	390.3032	117.627	0.733465	0.158413	0.77

gi|115552411|dbj|AK236416.1|	brown	94.65906	26.5331	0.741089	0.151855	4.896
gi|115552413|dbj|AK236418.1|	turquoise	741.0695	238.503	0.92091	0.026381	15.924
gi|115552415|dbj|AK236420.1|	brown	83.00468	21.9034	0.571935	0.313711	13.738
gi|115552428|dbj|AK236433.1|	midnightblue	140.5144	41.7442	0.967328	0.007054	1841.316
gi|115552430|dbj|AK236435.1|	turquoise	836.1104	251.265	0.951495	0.01273	33.88
gi|115552454|dbj|AK236459.1|	violet	857.6446	71.217	0.98809	0.001558	18.736
gi|115552473|dbj|AK236478.1|	violet	993.6555	72.8925	0.992809	0.000731	76.252
gi|115552477|dbj|AK236482.1|	turquoise	371.3805	122.276	0.740515	0.152346	3.292
gi|115552482|dbj|AK236487.1|	lightgreen	270.4299	56.2493	0.981785	0.002943	8.334
gi|115552488|dbj|AK236493.1|	blue	884.3519	253.916	0.992265	0.000816	14.694
gi|115552491|dbj|AK236496.1|	greenyellow	247.2889	42.3185	0.925435	0.024167	12.1
gi|115552492|dbj|AK236497.1|	black	980.9845	99.0855	0.999155	2.95E-05	15.732
gi|115552519|dbj|AK236524.1|	turquoise	689.1916	211.878	0.919051	0.027309	14.856
gi|115552523|dbj|AK236528.1|	darkmagenta	962.4803	26.9158	0.993293	0.000659	100.946
gi|115552527|dbj|AK236532.1|	grey60	924.6031	62.2913	0.994953	0.00043	70.518
gi|115552544|dbj|AK236549.1|	blue	667.7365	168.515	0.894995	0.040197	15.052
gi|115552549|dbj|AK236554.1|	turquoise	383.1438	96.8891	0.680749	0.205852	46.856
gi|115552559|dbj|AK236564.1|	tan	816.9161	62.7948	0.978958	0.003652	14.748
gi|115552575|dbj|AK232780.1|	turquoise	389.8388	84.4714	0.653721	0.231482	53.548
gi|115552583|dbj|AK232788.1|	white	992.8698	27.2011	0.727228	0.163837	30.672
gi|115552602|dbj|AK232807.1|	darkgreen	276.9922	36.4001	0.981705	0.002962	71.42
gi|115552627|dbj|AK232832.1|	darkgrey	191.4631	34.7681	0.99623	0.000278	9.2
gi|115552638|dbj|AK232843.1|	midnightblue	181.3766	42.5868	0.973838	0.00506	190.518
gi|115552645|dbj|AK232850.1|	purple	81.92244	14.265	0.749355	0.144837	45.07
gi|115552656|dbj|AK232861.1|	brown	299.9064	16.7575	0.425708	0.47482	12.676
gi|115552676|dbj|AK232881.1|	navy	110.7939	34.8486	0.866279	0.057508	22.97
gi|115552724|dbj|AK232929.1|	pink	125.5473	12.1919	0.224248	0.71689	32.016
gi|115552731|dbj|AK232936.1|	midnightblue	180.1905	43.0221	0.971201	0.005841	10.272
gi|115552766|dbj|AK239359.1|	blue	768.0927	223.369	0.956236	0.010918	19.1
gi|115552791|dbj|AK239385.1|	darkorange	139.011	8.68417	0.428964	0.471072	20.292
gi|115552842|dbj|AK239436.1|	brown	88.13491	38.7328	0.659504	0.225928	455.404
gi|115552862|dbj|AK239456.1|	yellow	268.722	88.5328	0.925847	0.023968	127.192
gi|115552865|dbj|AK239459.1|	brown	165.9537	54.1034	0.940431	0.017296	139.112
gi|115552884|dbj|AK239478.1|	turquoise	610.9085	174.559	0.864061	0.058924	62.134
gi|115552903|dbj|AK239497.1|	turquoise	939.0428	268.192	0.985717	0.002045	80.656
gi|115552915|dbj|AK239509.1|	red	325.5714	78.5902	0.925755	0.024013	23.264
gi|115552930|dbj|AK239524.1|	sienna3	962.4952	25.9292	0.998554	6.60E-05	55.616
gi|115552932|dbj|AK239526.1|	turquoise	786.6019	221.866	0.931581	0.021261	12.438
gi|115552944|dbj|AK239538.1|	pink	236.5404	59.7867	0.928594	0.022659	46.722
gi|115552956|dbj|AK239550.1|	purple	75.84729	13.3231	0.97172	0.005685	9.844
gi|115552967|dbj|AK239553.1|	paleturquoise	206.832	19.5163	0.951963	0.012547	10.566
gi|115552977|dbj|AK239563.1|	grey60	974.8206	63.1409	0.997718	0.000131	81.628
gi|115552989|dbj|AK239575.1|	turquoise	568.5952	159.052	0.828777	0.082831	52.36
gi|115553011|dbj|AK239597.1|	navy	117.7068	30.7501	0.847998	0.069495	8.752
gi|115553014|dbj|AK239600.1|	pink	240.1264	52.7269	0.905357	0.034451	53.692
gi|115553015|dbj|AK239601.1|	black	956.0028	97.4055	0.995245	0.000393	21.456
gi|115553025|dbj|AK239611.1|	white	170.3212	7.45695	0.26601	0.665344	81.498
gi|115553031|dbj|AK239617.1|	navy	153.0598	43.4112	0.985744	0.002039	19.548

gi|115553034|dbj|AK239620.1|	lightyellow	1000.717	60.7952	0.990966	0.001029	22.308
gi|115553038|dbj|AK239624.1|	grey	243.2707	11.8099	0.968949	0.006538	237.304
gi|115553041|dbj|AK239627.1|	blue	623.6915	179.637	0.913916	0.029925	9.798
gi|115553045|dbj|AK239631.1|	turquoise	462.7157	129.008	0.750355	0.143995	13.95
gi|115553060|dbj|AK239646.1|	red	482.8555	109.288	0.996928	0.000204	236.196
gi|115553074|dbj|AK239660.1|	blue	696.6624	211.144	0.947807	0.014201	47.204
gi|115553082|dbj|AK239668.1|	turquoise	965.1347	267.313	0.99196	0.000864	59.426
gi|115553090|dbj|AK239676.1|	white	976.0718	28.8078	0.756353	0.138972	22.278
gi|115553091|dbj|AK239677.1|	sienna3	989.7187	25.9517	0.998669	5.83E-05	28.858
gi|115553103|dbj|AK239689.1|	grey60	976.5827	59.9775	0.987239	0.001727	72.864
gi|115553107|dbj|AK239693.1|	pink	226.5859	68.844	0.985774	0.002032	25.484
gi|115553121|dbj|AK239707.1|	turquoise	484.1329	163.862	0.827229	0.083937	28.55
gi|115553124|dbj|AK239710.1|	black	730.4744	75.7641	0.944801	0.015439	5.234
gi|115553140|dbj|AK239726.1|	brown	85.45106	29.5788	0.562152	0.32397	24.936
gi|115553142|dbj|AK239728.1|	red	624.3603	95.2383	0.965279	0.007726	43.712
gi|115553143|dbj|AK239729.1|	orange	476.6342	30.6837	0.964257	0.008068	10.526
gi|115553145|dbj|AK239731.1|	darkred	239.8782	45.34	0.994582	0.000478	6.044
gi|115553157|dbj|AK239743.1|	darkgreen	284.7095	30.7145	0.94549	0.015152	5.046
gi|115553159|dbj|AK239745.1|	navy	127.9999	37.4173	0.912341	0.030742	25.354
gi|115553169|dbj|AK232967.1|	yellow	144.1465	40.7907	0.745608	0.148007	57.514
gi|115553171|dbj|AK232969.1|	orange	385.8249	35.4342	0.997503	0.00015	40.374
gi|115553181|dbj|AK232979.1|	tan	870.0004	63.2854	0.980977	0.00314	18.478
gi|115553189|dbj|AK232987.1|	paleturquoise	217.2958	17.2851	0.92409	0.024818	23.706
gi|115553196|dbj|AK232994.1|	pink	66.61514	7.3255	0.395229	0.510205	48.77
gi|115553202|dbj|AK233000.1|	blue	661.5182	203.703	0.936975	0.018813	52.326
gi|115553207|dbj|AK233005.1|	tan	897.4055	66.8972	0.992596	0.000764	11.506
gi|115553209|dbj|AK233007.1|	yellow	304.2173	111.602	0.980377	0.00329	129.784
gi|115553226|dbj|AK233024.1|	blue	798.8542	164.954	0.893166	0.041239	23.822
gi|115553231|dbj|AK233029.1|	blue	854.0271	242.32	0.978301	0.003824	69.024
gi|115553249|dbj|AK233047.1|	turquoise	497.1302	161.333	0.828622	0.082942	299.342
gi|115553264|dbj|AK233062.1|	paleturquoise	200.8147	18.1882	0.930544	0.021743	1.67
gi|115553284|dbj|AK233082.1|	skyblue	138.4744	25.6878	0.946564	0.014709	30.648
gi|115553328|dbj|AK233126.1|	grey	133.1267	8.84961	0.250985	0.683823	45.114
gi|115553344|dbj|AK233142.1|	turquoise	758.7661	225.813	0.939657	0.017632	8.86
gi|115553351|dbj|AK233149.1|	skyblue	196.2457	30.2441	0.980971	0.003142	29.952
gi|115553356|dbj|AK233154.1|	darkturquoise	157.6545	31.6828	0.972866	0.005344	3.458
gi|115553369|dbj|AK236572.1|	turquoise	610.8158	171.222	0.868159	0.056316	31.13
gi|115553370|dbj|AK236573.1|	darkgreen	264.7068	20.9752	0.832182	0.080416	14.57
gi|115553373|dbj|AK236576.1|	turquoise	883.3786	259.05	0.976158	0.004404	58.488
gi|115553381|dbj|AK236584.1|	turquoise	462.9796	157.211	0.797367	0.106105	23.052
gi|115553384|dbj|AK236587.1|	green	101.9058	30.7359	0.759033	0.136746	150.688
gi|115553397|dbj|AK236600.1|	turquoise	203.5287	69.161	0.573054	0.312543	9.438
gi|115553406|dbj|AK236609.1|	turquoise	717.8482	221.328	0.906231	0.03398	7.73
gi|115553415|dbj|AK236618.1|	blue	813.6949	200.048	0.931179	0.021448	20.968
gi|115553434|dbj|AK236637.1|	blue	899.088	209.026	0.948166	0.014056	12.974
gi|115553440|dbj|AK236643.1|	blue	825.8582	231.096	0.967632	0.006957	17.516
gi|115553452|dbj|AK236655.1|	turquoise	542.9118	152.802	0.819179	0.089756	44.64
gi|115553453|dbj|AK236656.1|	paleturquoise	162.3307	17.4861	0.929248	0.02235	33.414

gi|115553457|dbj|AK236660.1|	tan	859.0391	65.4552	0.987944	0.001586	14.702
gi|115553468|dbj|AK236671.1|	brown	180.0898	43.0239	0.650479	0.234611	45.206
gi|115553471|dbj|AK236674.1|	blue	766.8865	227.283	0.964012	0.008151	48.232
gi|115553478|dbj|AK236681.1|	greenyellow	119.2082	20.1537	0.746902	0.146909	28
gi|115553483|dbj|AK236686.1|	turquoise	436.9869	131.235	0.755996	0.13927	12.076
gi|115553496|dbj|AK236699.1|	lightgreen	291.1819	58.9128	0.991348	0.000965	3.794
gi|115553497|dbj|AK236700.1|	green	104.7653	14.5397	-0.04044	0.948524	66.124
gi|115553505|dbj|AK236708.1|	brown	86.96721	21.7105	0.416042	0.485985	7.498
gi|115553521|dbj|AK236724.1|	sienna3	983.0559	25.8549	0.99793	0.000113	14.646
gi|115553524|dbj|AK236727.1|	brown	82.6825	31.2406	0.533836	0.354127	51.99
gi|115553527|dbj|AK236730.1|	lightcyan	296.8174	42.4607	0.986519	0.001875	64.674
gi|115553540|dbj|AK236743.1|	darkred	198.5683	39.5945	0.957401	0.010487	9.672
gi|115553549|dbj|AK236752.1|	turquoise	731.3034	238.119	0.920402	0.026634	158.358
gi|115553555|dbj|AK236758.1|	lightgreen	302.9534	60.8267	0.997793	0.000124	7.636
gi|115553566|dbj|AK239753.1|	brown	191.0119	58.46	0.947007	0.014527	15.71
gi|115553571|dbj|AK239758.1|	blue	649.1723	189.864	0.924817	0.024465	31.552
gi|115553590|dbj|AK239777.1|	salmon	354.3692	39.8987	0.928254	0.022819	27.298
gi|115553648|dbj|AK239835.1|	blue	840.5099	221.469	0.960086	0.009515	7.724
gi|115553654|dbj|AK239841.1|	brown	95.68649	26.5583	0.678133	0.208296	1.534
gi|115553671|dbj|AK239858.1|	turquoise	621.1569	199.088	0.910719	0.031591	65.798
gi|115553680|dbj|AK239867.1|	violet	961.1018	72.9736	0.993145	0.000681	4.688
gi|115553700|dbj|AK239887.1|	turquoise	309.8378	93.1026	0.650618	0.234477	26.368
gi|115553703|dbj|AK239890.1|	turquoise	636.2472	152.901	0.837527	0.076671	39.566
gi|115553719|dbj|AK239906.1|	blue	838.5704	239.13	0.97573	0.004522	269.14
gi|115553747|dbj|AK239934.1|	grey	80.7206	6.7914	0.17232	0.781686	27.794
gi|115553748|dbj|AK239935.1|	brown	227.396	61.6337	0.92211	0.025788	26.496
gi|115553757|dbj|AK239944.1|	green	164.8039	19.4672	0.152344	0.806782	40.182
gi|115553782|dbj|AK236787.1|	grey	176.6163	12.3907	0.213662	0.730042	18.114
gi|115553811|dbj|AK236816.1|	blue	727.2225	215.399	0.952803	0.012221	5.446
gi|115553814|dbj|AK236819.1|	lightcyan	290.0467	41.1593	0.979569	0.003495	35.044
gi|115553831|dbj|AK236836.1|	darkorange	197.8691	9.03793	0.57202	0.313622	12.05
gi|115553846|dbj|AK236851.1|	turquoise	479.7347	102.288	0.734845	0.15722	12.128
gi|115553858|dbj|AK236863.1|	brown	90.31413	32.4001	0.752657	0.142061	21.986
gi|115553862|dbj|AK236867.1|	greenyellow	234.6028	47.8797	0.960413	0.009399	10.896
gi|115553894|dbj|AK236899.1|	turquoise	587.5343	150.151	0.820215	0.089	8.04
gi|115553962|dbj|AK233171.1|	brown	91.56896	22.2217	0.588908	0.296113	40.316
gi|115553967|dbj|AK233176.1|	green	163.3788	22.6654	0.298881	0.625197	50.312
gi|115554007|dbj|AK233216.1|	turquoise	314.9063	78.386	0.607194	0.277455	11.152
gi|115554012|dbj|AK233221.1|	tan	887.3551	67.3853	0.994395	0.000503	27.338
gi|115554020|dbj|AK233229.1|	turquoise	415.0701	113.797	0.724893	0.165881	2.296
gi|115554022|dbj|AK233231.1|	red	370.8781	85.7884	0.944304	0.015646	311.794
gi|115554075|dbj|AK233284.1|	navy	161.0863	11.6989	-0.59994	0.284816	48.25
gi|115554113|dbj|AK233322.1|	violet	958.7592	74.068	0.996135	0.000288	6.874
gi|115554124|dbj|AK233334.1|	turquoise	764.8283	245.276	0.933896	0.020198	235.574
gi|115554126|dbj|AK233336.1|	darkred	189.3444	37.2871	0.945882	0.01499	2.804
gi|115554130|dbj|AK233340.1|	blue	699.5199	193.162	0.929269	0.02234	27.6
gi|115554133|dbj|AK233343.1|	darkmagenta	805.7961	23.3139	0.96434	0.00804	53.59
gi|115554161|dbj|AK239956.1|	turquoise	489.6849	121.909	0.759155	0.136645	33.97

gi|115554180|dbj|AK239975.1|	turquoise	425.0257	141.58	0.77361	0.124823	19.686
gi|115554203|dbj|AK239998.1|	violet	929.4696	74.6499	0.997668	0.000135	1.766
gi|115554213|dbj|AK240008.1|	blue	895.8826	256.51	0.993478	0.000632	24.162
gi|115554214|dbj|AK240009.1|	lightyellow	866.327	60.545	0.990256	0.001153	8.184
gi|115554223|dbj|AK240018.1|	darkmagenta	949.2346	26.9086	0.993421	0.00064	4.338
gi|115554227|dbj|AK240022.1|	grey60	964.7992	62.1699	0.994503	0.000489	32.852
gi|115554228|dbj|AK240023.1|	turquoise	453.7853	144.239	0.781869	0.118213	2.47
gi|115554247|dbj|AK240042.1|	navy	120.3932	5.395	0.080124	0.898093	1.924
gi|115554271|dbj|AK240066.1|	lightcyan	197.7069	30.3396	0.900068	0.037348	35.76
gi|115554273|dbj|AK240068.1|	turquoise	359.3335	119.325	0.7562	0.1391	6.974
gi|115554282|dbj|AK240077.1|	black	947.2256	87.205	0.972941	0.005321	104.51
gi|115554284|dbj|AK240079.1|	black	889.9196	87.2515	0.973433	0.005177	9.056
gi|115554299|dbj|AK240094.1|	orange	555.3852	33.8698	0.988008	0.001574	25.912
gi|115554309|dbj|AK240104.1|	purple	149.7324	10.9433	-0.88843	0.043979	10.72
gi|115554312|dbj|AK240107.1|	red	641.8988	104.038	0.985272	0.002141	133.064
gi|115554314|dbj|AK240109.1|	lightyellow	987.1236	62.9945	0.998239	8.87E-05	64.456
gi|115554351|dbj|AK240146.1|	grey	141.3466	12.4058	-0.898	0.038502	16.006
gi|115554358|dbj|AK233368.1|	white	180.5065	25.4831	0.793961	0.108732	34.096
gi|115554363|dbj|AK233373.1|	blue	921.0186	200.653	0.940022	0.017473	11.254
gi|115554371|dbj|AK233381.1|	darkred	228.7375	41.1847	0.975453	0.0046	18.58
gi|115554373|dbj|AK233383.1|	turquoise	425.0313	142.207	0.778412	0.120966	5.18
gi|115554409|dbj|AK233419.1|	turquoise	785.8626	250.391	0.939841	0.017552	1350.794
gi|115554410|dbj|AK233420.1|	turquoise	967.6746	270.943	0.998702	5.62E-05	91.85
gi|115554413|dbj|AK233423.1|	pink	264.5653	75.5496	0.998359	7.98E-05	0.586
gi|115554415|dbj|AK233425.1|	white	184.7935	22.4411	0.901629	0.036486	12.866
gi|115554455|dbj|AK233465.1|	blue	802.5743	208.309	0.942551	0.016386	7.03
gi|115554459|dbj|AK233469.1|	orange	432.7407	16.9039	0.835546	0.078053	15.358
gi|115554528|dbj|AK233538.1|	brown	44.86015	9.04283	0.333763	0.583067	56.402
gi|115554532|dbj|AK233542.1|	yellowgreen	164.6008	16.7425	0.974261	0.004938	332.47
gi|115554540|dbj|AK233550.1|	turquoise	486.637	132.908	0.758333	0.137326	26.134
gi|115554557|dbj|AK236967.1|	navy	159.6298	45.2143	0.997556	0.000145	3.16
gi|115554560|dbj|AK236970.1|	turquoise	907.9899	267.837	0.986982	0.00178	29.122
gi|115554563|dbj|AK236973.1|	lightcyan	194.0766	27.5523	0.865247	0.058165	21.378
gi|115554566|dbj|AK236976.1|	navy	81.70945	6.23799	0.347243	0.566929	58.744
gi|115554588|dbj|AK236998.1|	tan	697.2829	49.8637	0.933007	0.020605	4.57
gi|115554610|dbj|AK237020.1|	darkgreen	241.5463	18.4304	0.794181	0.108562	9.118
gi|115554627|dbj|AK237038.1|	greenyellow	198.66	54.7941	0.994387	0.000504	16.33
gi|115554633|dbj|AK237044.1|	red	559.964	100.913	0.979278	0.00357	10.674
gi|115554695|dbj|AK237106.1|	red	424.6654	102.223	0.982534	0.002764	32.406
gi|115554713|dbj|AK237124.1|	green	105.6402	40.4307	0.850803	0.06761	40.422
gi|115554725|dbj|AK237136.1|	brown	153.3358	39.2959	0.774738	0.123913	8.248
gi|115554740|dbj|AK237151.1|	blue	740.1081	221.779	0.957132	0.010586	33.814
gi|115554764|dbj|AK233576.1|	brown	210.8215	62.4476	0.905916	0.034149	2.36
gi|115554769|dbj|AK233581.1|	turquoise	228.1852	51.7417	0.54032	0.347163	4.486
gi|115554826|dbj|AK233638.1|	brown	172.7535	31.8363	0.667068	0.218721	71.078
gi|115554840|dbj|AK233652.1|	turquoise	917.3302	261.232	0.976203	0.004391	20.872
gi|115554857|dbj|AK233669.1|	magenta	287.2688	55.8027	0.913268	0.03026	544.134
gi|115554866|dbj|AK233678.1|	blue	830.084	243.968	0.97961	0.003484	17.526

gi|115554868|dbj|AK233680.1|	grey60	950.1232	59.8423	0.986839	0.001809	7.5
gi|115554875|dbj|AK233687.1|	brown	148.0566	44.7553	0.896403	0.039399	8.054
gi|115554899|dbj|AK233711.1|	turquoise	895.7897	265.724	0.97432	0.004921	32.948
gi|115554918|dbj|AK233730.1|	darkgreen	221.9525	30.0774	0.950549	0.013102	20.534
gi|115554929|dbj|AK233741.1|	yellow	276.3271	104.28	0.965436	0.007674	16.35
gi|115554960|dbj|AK240159.1|	orange	440.1106	35.171	0.99603	0.0003	20.988
gi|115554973|dbj|AK240172.1|	pink	282.8855	71.3289	0.971754	0.005674	46.194
gi|115554982|dbj|AK240181.1|	red	513.5982	104.742	0.987693	0.001636	183.398
gi|115554984|dbj|AK240183.1|	turquoise	670.0858	213.637	0.911854	0.030996	3.684
gi|115555002|dbj|AK240201.1|	black	962.1241	97.5893	0.995986	0.000305	18.326
gi|115555012|dbj|AK240211.1|	grey	343.1577	12.0481	0.958793	0.009979	21.534
gi|115555020|dbj|AK240219.1|	darkgrey	126.6	18.1241	0.827363	0.083841	14.996
gi|115555021|dbj|AK240220.1|	paleturquoise	138.5165	10.2119	0.770126	0.127643	31.586
gi|115555035|dbj|AK240234.1|	turquoise	249.1189	74.4651	0.592759	0.292158	17.068
gi|115555041|dbj|AK240240.1|	turquoise	815.2331	255.411	0.955087	0.011349	35.902
gi|115555043|dbj|AK240242.1|	blue	630.895	190.904	0.923668	0.025024	4.822
gi|115555058|dbj|AK240257.1|	pink	255.1633	74.393	0.996024	0.000301	20.536
gi|115555063|dbj|AK240262.1|	lightcyan	313.626	40.3824	0.960264	0.009451	16.83
gi|115555072|dbj|AK240271.1|	turquoise	424.0945	140.965	0.802686	0.102042	10.174
gi|115555076|dbj|AK240275.1|	yellow	273.3508	101.117	0.961218	0.009115	5.598
gi|115555097|dbj|AK240296.1|	blue	706.0274	215.351	0.948745	0.013822	56.152
gi|115555127|dbj|AK240327.1|	turquoise	636.4489	158.099	0.839307	0.075435	28.298
gi|115555129|dbj|AK240329.1|	turquoise	621.4293	186.265	0.897353	0.038864	135.398
gi|115555138|dbj|AK240338.1|	turquoise	685.3878	217.23	0.899685	0.037561	1.78
gi|115555145|dbj|AK240345.1|	turquoise	708.0584	224.834	0.91156	0.03115	75.01
gi|115555150|dbj|AK233765.1|	turquoise	245.6534	58.6435	0.482565	0.410337	66.786
gi|115555155|dbj|AK233770.1|	tan	868.8902	62.7325	0.979469	0.00352	24.682
gi|115555162|dbj|AK233777.1|	magenta	219.7666	69.8692	0.962318	0.008731	81.068
gi|115555163|dbj|AK233778.1|	turquoise	935.4007	269.471	0.985959	0.001993	37.934
gi|115555187|dbj|AK233802.1|	blue	870.5563	225.561	0.960818	0.009255	9.866
gi|115555188|dbj|AK233803.1|	lightcyan	198.0175	30.362	0.895903	0.039682	21.934
gi|115555205|dbj|AK233820.1|	navy	228.96	26.5508	0.760564	0.135479	2.1
gi|115555219|dbj|AK233834.1|	turquoise	410.909	105.55	0.708312	0.180606	2.866
gi|115555288|dbj|AK233903.1|	turquoise	779.1761	218.744	0.920961	0.026356	42.264
gi|115555322|dbj|AK233937.1|	salmon	340.3768	36.6665	0.902966	0.035752	9.782
gi|115555347|dbj|AK233960.1|	pink	264.5653	75.5496	0.998359	7.98E-05	0.312
gi|115555353|dbj|AK233966.1|	turquoise	652.8266	166.198	0.85382	0.0656	28.312
gi|115555420|dbj|AK234033.1|	brown	212.4111	61.344	0.868554	0.056066	24.452
gi|115555451|dbj|AK234064.1|	blue	952.7233	212.636	0.950502	0.013121	17.152
gi|115555475|dbj|AK234088.1|	turquoise	460.9297	120.577	0.762101	0.134209	22.99
gi|115555495|dbj|AK234108.1|	blue	929.7319	224.409	0.961809	0.008908	12.858
gi|115555513|dbj|AK234126.1|	turquoise	942.0007	271.674	0.991194	0.000991	64.044
gi|115555517|dbj|AK234130.1|	lightyellow	874.6708	60.659	0.990653	0.001083	3.18
gi|115555541|dbj|AK237173.1|	tan	929.9465	68.8562	0.998667	5.84E-05	75.204
gi|115555553|dbj|AK237185.1|	darkturquoise	135.9456	24.6572	0.903264	0.035589	52.724
gi|115555561|dbj|AK237193.1|	magenta	244.6775	77.673	0.986233	0.001935	3.798
gi|115555588|dbj|AK237220.1|	darkgrey	122.9593	14.8135	0.782651	0.117592	12.022
gi|115555602|dbj|AK237234.1|	turquoise	705.2963	189.564	0.902242	0.036148	15.752

gi|115555616|dbj|AK237249.1|	pink	264.5653	75.5496	0.998359	7.98E-05	0.118
gi|115555619|dbj|AK237252.1|	blue	568.8471	143.377	0.8649	0.058387	157.412
gi|115555627|dbj|AK237260.1|	darkorange	144.016	18.1669	0.946959	0.014547	0.732
gi|115555639|dbj|AK237272.1|	turquoise	655.7731	197.248	0.904197	0.03508	217.68
gi|115555653|dbj|AK237286.1|	white	157.5014	5.86117	0.092319	0.882623	41.312
gi|115555661|dbj|AK237294.1|	blue	889.7256	249.634	0.985498	0.002092	13.65
gi|115555675|dbj|AK237308.1|	orange	392.1325	33.206	0.983461	0.002547	18.28
gi|115555681|dbj|AK237314.1|	grey60	929.9044	62.9415	0.997131	0.000184	17.914
gi|115555693|dbj|AK237326.1|	tan	934.7313	68.7016	0.998013	0.000106	26.106
gi|115555696|dbj|AK237329.1|	midnightblue	131.6438	33.7469	0.912325	0.03075	10.09
gi|115555721|dbj|AK234149.1|	blue	657.5844	135.479	0.849317	0.068606	5.774
gi|116175276|ref|NM_001077227.1|	purple	183.134	19.7675	-0.8227	0.087193	10.064
gi|117661185|gb|DQ629176.1|	navy	118.1405	30.1073	0.853707	0.065675	744.14
gi|118403765|ref|NM_001078687.1|	orange	346.9792	34.3658	0.989057	0.001372	173.502
gi|118403821|ref|NM_001078679.1|	grey60	924.2583	61.7059	0.993019	0.000699	10.154
gi|118403857|ref|NM_001078670.1|	lightcyan	214.1864	33.1263	0.921605	0.026037	11.624
gi|118403869|ref|NM_001078669.1|	blue	840.2835	238.389	0.978475	0.003779	36.222
gi|118403911|ref|NM_001078662.1|	green	131.4016	47.2006	0.90985	0.03205	52.434
gi|119310177|ref|NM_001032376.2|	lightyellow	970.3371	63.303	0.999238	2.53E-05	20.716
gi|1245714|gb|S80644.1|	pink	105.9402	14.0889	0.250424	0.684515	13.352
gi|125490322|ref|NM_213824.2|	blue	807.711	228.294	0.965423	0.007678	8.89
gi|125630297|ref|NM_001005152.2|	white	170.6361	23.2909	0.857614	0.063101	15.8
gi|125630333|ref|NM_214068.2|	turquoise	112.4889	33.3033	0.378822	0.529467	17.248
gi|139530445|gb|EF486522.1|	navy	406.2348	25.6708	0.779074	0.120437	138.754
gi|145279656|ref|NM_214014.2|	black	747.9881	77.7243	0.949935	0.013346	3.906
gi|146198449|dbj|AB292846.1|	red	425.3623	98.1751	0.973615	0.005124	12.65
gi|146741279|dbj|AB271920.1|	turquoise	337.5853	81.9456	0.631863	0.2528	4.028
gi|147899058|ref|NM_001097509.1|	grey	159.367	6.38873	-0.82032	0.088921	270.96
gi|147900046|ref|NM_001097474.1|	pink	279.5	73.4262	0.979311	0.003561	563.452
gi|147900501|ref|NM_001097506.1|	white	959.2085	29.624	0.801014	0.103314	32.956
gi|147906088|ref|NM_213946.2|	blue	884.2411	218.646	0.953401	0.011991	201.254
gi|148222423|ref|NM_001097426.1|	red	587.2744	108.998	0.996158	0.000286	12.292
gi|148222590|ref|NM_001097486.1|	red	565.8476	108.747	0.995612	0.000349	67.136
gi|148223598|ref|NM_001097476.1|	violet	994.3591	73.1253	0.993492	0.00063	71.374
gi|148224637|ref|NM_001097469.1|	red	311.9712	75.8018	0.918038	0.027819	66.77
gi|148225749|ref|NM_001097504.1|	navy	285.2369	32.9491	0.858954	0.062225	27.322
gi|148228021|ref|NM_001097420.1|	darkturquoise	201.1397	32.0166	0.974521	0.004863	11.108
gi|148229131|ref|NM_001097428.1|	pink	182.1795	49.8362	0.912489	0.030665	11.816
gi|148233142|ref|NM_001097435.1|	red	480.0165	93.4772	0.962145	0.008791	26.816
gi|148233297|ref|NM_001097485.1|	turquoise	627.0569	177.771	0.868294	0.05623	36.478
gi|148233557|ref|NM_001097460.1|	royalblue	160.0982	1.24643	-0.46675	0.428057	31.524
gi|148234109|ref|NM_001093735.1|	blue	881.5269	252.635	0.989268	0.001332	26.872
gi|148235350|ref|NM_001097475.1|	royalblue	296.9054	12.3182	0.74268	0.150497	182.244
gi|148237281|ref|NM_001097478.1|	blue	956.082	212.631	0.951886	0.012577	265.254
gi|149364039|gb|EF468461.1|	violet	942.6164	74.7919	0.998074	0.000101	5.98
gi|149944498|ref|NM_214343.2|	black	980.2012	98.1902	0.996872	0.00021	34.384
gi|149944608|ref|NM_001001546.2|	sienna3	779.9874	22.7133	0.972174	0.005549	5.35
gi|150246527|ref|NM_001044575.2|	black	943.635	97.2476	0.994926	0.000434	102.608

gi|153792026|ref|NM_001099932.1|	midnightblue	98.51824	31.7752	0.911797	0.031026	1510.836
gi|153792275|ref|NM_001097505.1|	blue	678.7958	172.54	0.899441	0.037697	4.35
gi|153792324|ref|NM_001097517.1|	turquoise	528.6212	162.555	0.840971	0.074286	15.094
gi|153792599|ref|NM_001099930.1|	white	217.2388	21.8936	0.722055	0.168376	10.642
gi|154147629|ref|NM_001100190.1|	turquoise	816.1832	250.927	0.948708	0.013837	53.18
gi|154147643|ref|NM_001100193.1|	turquoise	932.1115	263.53	0.984785	0.002248	27.852
gi|156120135|ref|NM_001101827.1|	white	970.3567	29.5668	0.796059	0.107112	43.466
gi|156120149|ref|NM_001101823.1|	white	194.6718	7.49975	0.156619	0.801405	3.094
gi|156120151|ref|NM_001101824.1|	lightyellow	973.3891	63.2518	0.99907	3.41E-05	16.432
gi|156123600|gb|EU030283.2|	purple	182.3646	14.4121	-0.86421	0.058829	32.31
gi|156151354|ref|NM_001037146.2|	pink	268.9252	61.0688	0.935263	0.01958	21.106
gi|157427686|ref|NM_214374.2|	darkgreen	254.1464	37.5227	0.98378	0.002474	2336.788
gi|157427714|ref|NM_001105294.1|	magenta	259.3951	79.6035	0.991829	0.000886	7.264
gi|157427721|ref|NM_001105299.1|	blue	927.8648	199.286	0.936534	0.019009	68.426
gi|157427723|ref|NM_001105300.1|	turquoise	482.7318	161.157	0.816699	0.091572	78.168
gi|157427727|ref|NM_001105302.1|	turquoise	357.4655	102.26	0.675528	0.210738	10.65
gi|157427731|ref|NM_001105304.1|	salmon	175.669	40.9784	0.939888	0.017532	54.912
gi|157427747|ref|NM_001105296.1|	tan	923.6903	67.5491	0.994148	0.000537	13.62
gi|158262680|ref|NM_001109947.1|	darkgreen	232.8018	16.6926	0.74125	0.151717	13.96
gi|158631261|ref|NM_001099936.1|	turquoise	706.0286	229.606	0.924063	0.024831	42.248
gi|160420270|ref|NM_001044582.1|	violet	984.071	71.9628	0.990341	0.001138	50.222
gi|162139822|ref|NM_001111257.1|	violet	958.23	74.4621	0.997202	0.000178	39.34
gi|162287024|ref|NM_001111258.1|	navy	101.6564	23.7685	0.763784	0.132824	17.122
gi|162951810|ref|NM_001112690.1|	navy	164.4712	43.3349	0.989189	0.001347	3.258
gi|16304807|emb|AJ416019.1|	lightcyan	299.9584	43.6402	0.986811	0.001815	101.348
gi|163115794|gb|EU009401.2|	blue	798.5354	238.687	0.974565	0.004851	21.352
gi|163310772|ref|NM_001097521.2|	darkgrey	195.0994	32.7353	0.980691	0.003212	44.562
gi|163915140|ref|NM_001113047.1|	lightcyan	119.9205	13.8236	0.725941	0.164963	158.29
gi|163915152|ref|NM_001113053.1|	blue	656.5304	180.703	0.913161	0.030315	7.212
gi|164518957|ref|NM_001113287.1|	red	699.3187	94.7022	0.96406	0.008135	221.662
gi|164664445|ref|NM_001113439.1|	magenta	275.2898	78.413	0.989019	0.001379	48.158
gi|164664451|ref|NM_001113442.1|	turquoise	458.4117	116.93	0.715035	0.174592	25.786
gi|164664455|ref|NM_001113444.1|	turquoise	367.5934	107.056	0.723099	0.167458	61.34
gi|165973425|ref|NM_001113706.1|	pink	82.97506	18.4232	0.383287	0.524211	88.536
gi|165973431|ref|NM_001113698.1|	orange	241.9893	28.6602	0.947785	0.01421	52.258
gi|166796042|ref|NM_001114275.1|	blue	851.1399	247.937	0.984656	0.002276	5.01
gi|166796054|ref|NM_001114281.1|	turquoise	317.2491	95.2891	0.666835	0.218942	7.12
gi|167908788|ref|NM_001114675.1|	tan	745.7562	48.354	0.928064	0.022909	24.292
gi|167908792|ref|NM_001114670.1|	turquoise	738.0704	211.345	0.929929	0.02203	20.664
gi|167908794|ref|NM_001114671.1|	salmon	152.3851	32.6489	0.880205	0.048869	60.426
gi|171905894|gb|EU561660.1|	grey60	876.1986	61.074	0.990985	0.001026	476.374
gi|172072681|ref|NM_001122994.1|	greenyellow	103.4245	20.6785	0.749009	0.145129	88.476
gi|172073180|ref|NM_001099931.1|	black	967.2434	98.8961	0.998542	6.68E-05	20.48
gi|178056465|ref|NM_001123083.1|	salmon	156.4511	41.5099	0.94518	0.01528	11.976
gi|178056481|ref|NM_001123135.1|	black	930.9575	96.7979	0.994578	0.000479	3.848
gi|178056483|ref|NM_001123101.1|	violet	983.5313	72.2794	0.99111	0.001005	67.724
gi|178056487|ref|NM_001123204.1|	turquoise	573.2257	191.628	0.842348	0.073339	23.908
gi|178056503|ref|NM_001123076.1|	midnightblue	138.9928	42.0729	0.96549	0.007656	622.692

gi|178056523|ref|NM_001123127.1|	violet	957.2575	75.0893	0.998884	4.47E-05	11.052
gi|178056529|ref|NM_001123203.1|	grey60	951.0809	61.9382	0.993764	0.000591	12.782
gi|178056549|ref|NM_001123162.1|	purple	48.36989	9.20899	0.438474	0.460161	0.772
gi|178056555|ref|NM_001123148.1|	turquoise	494.4596	157.409	0.817664	0.090864	18.842
gi|178056566|ref|NM_001123160.1|	tan	669.3997	46.6932	0.918141	0.027767	40.954
gi|178056595|ref|NM_001123136.1|	blue	949.8348	214.458	0.952832	0.01221	35.22
gi|178056622|ref|NM_001123213.1|	black	972.6048	97.644	0.996056	0.000297	58.106
gi|178056662|ref|NM_001123124.1|	darkred	201.1026	39.7999	0.958201	0.010194	7.8
gi|178056668|ref|NM_001123099.1|	white	179.0223	21.5369	0.689094	0.19811	181.132
gi|178056676|ref|NM_001123078.1|	green	133.2682	40.8634	0.869706	0.055341	658.606
gi|178056709|ref|NM_001123194.1|	navy	165.3578	39.5602	0.98155	0.003	10.532
gi|178056815|ref|NM_001123159.1|	greenyellow	206.6141	46.9681	0.956188	0.010936	23.718
gi|178056859|ref|NM_001123091.1|	brown	207.8385	52.2631	0.771775	0.126306	40.868
gi|178056883|ref|NM_001123198.1|	blue	680.0855	186.752	0.923497	0.025108	14.042
gi|178057054|ref|NM_001123079.1|	blue	812.493	233.296	0.968075	0.006814	15.398
gi|178057066|ref|NM_001123096.1|	tan	857.7605	65.8309	0.98905	0.001373	36.464
gi|178057124|ref|NM_001123104.1|	turquoise	474.0088	156.283	0.829432	0.082365	20.538
gi|178057176|ref|NM_001123090.1|	darkmagenta	956.7564	26.5184	0.990059	0.001188	22.168
gi|178057317|ref|NM_001123205.1|	brown	181.7331	58.5644	0.934788	0.019794	21.078
gi|187672044|gb|EU650276.1|	brown	66.94381	10.4781	0.327448	0.590655	18.554
gi|190360618|ref|NM_001128433.1|	purple	134.9639	22.1902	0.871522	0.054203	159.776
gi|190360620|ref|NM_001128486.1|	darkgreen	189.1172	25.0379	0.908482	0.032775	4.906
gi|190360638|ref|NM_001128469.1|	pink	232.5229	69.6185	0.984952	0.002211	17.546
gi|190360656|ref|NM_001128470.1|	brown	64.5935	24.8378	0.497805	0.393424	108.134
gi|1912|emb|X07617.1|	turquoise	188.9361	52.2085	0.4682	0.426428	10.738
gi|194018685|ref|NM_001129963.1|	pink	227.5396	56.723	0.918519	0.027576	523.638
gi|194018701|ref|NM_001129947.1|	brown	157.9308	26.3926	0.463079	0.432198	6032.722
gi|194018717|ref|NM_001129954.1|	blue	921.0166	246.825	0.98352	0.002533	2239.262
gi|194018721|ref|NM_001129949.1|	greenyellow	179.912	42.4807	0.92932	0.022316	35947.28
gi|194033408|ref|XM_001926440.1|	navy	160.0868	44.6338	0.99533	0.000383	151.096
gi|194033418|ref|XM_001924233.1|	purple	214.6251	20.9336	-0.79833	0.105365	11.806
gi|194033446|ref|XM_001927265.1|	darkred	251.5746	43.1011	0.983624	0.002509	9.212
gi|194033502|ref|XM_001926129.1|	turquoise	387.8784	132.045	0.797355	0.106115	9.904
gi|194033586|ref|XM_001929183.1|	tan	952.0397	63.8208	0.98164	0.002978	16.118
gi|194033590|ref|XM_001929195.1|	navy	222.4307	26.2088	0.719899	0.170279	212.95
gi|194033594|ref|XM_001927795.1|	turquoise	609.2366	141.928	0.812571	0.094621	16.172
gi|194033596|ref|XM_001927813.1|	midnightblue	149.123	43.9488	0.984274	0.002362	9.594
gi|194033615|ref|XM_001924714.1|	white	975.2162	27.3158	0.76907	0.128501	6.612
gi|194033638|ref|XM_001927936.1|	turquoise	872.52	249.219	0.958869	0.009951	28.282
gi|194033649|ref|XM_001925294.1|	pink	242.8828	71.8268	0.995377	0.000377	4.69
gi|194033677|ref|XM_001928828.1|	violet	996.2317	69.2339	0.982324	0.002814	17.056
gi|194033685|ref|XM_001926077.1|	royalblue	187.6305	9.87897	0.70114	0.187085	39.186
gi|194033718|ref|XM_001926719.1|	turquoise	283.0096	89.5339	0.696456	0.191351	19.76
gi|194033751|ref|XM_001924525.1|	navy	328.0041	28.8128	0.81625	0.091903	3.38
gi|194033753|ref|XM_001924715.1|	darkorange	204.5019	16.1213	0.903703	0.035349	14.704
gi|194033755|ref|XM_001924851.1|	royalblue	274.51	29.3192	0.951787	0.012616	22.558
gi|194033859|ref|XM_001927866.1|	orange	476.563	33.5592	0.987032	0.001769	81.928
gi|194033888|ref|XM_001925347.1|	turquoise	841.4294	261.429	0.960156	0.00949	173.606

gi|194033964|ref|XM_001925719.1|	pink	266.4503	75.4213	0.998653	5.93E-05	3.07
gi|194034011|ref|XM_001927812.1|	darkturquoise	141.0996	26.5632	0.924255	0.024738	51.272
gi|194034018|ref|XM_001925500.1|	blue	916.8302	214.58	0.95115	0.012865	14.26
gi|194034042|ref|XM_001925859.1|	blue	863.4749	245.5	0.98138	0.003042	20.87
gi|194034126|ref|XM_001926626.1|	red	598.4533	105.59	0.989447	0.001299	9.484
gi|194034144|ref|XM_001926437.1|	blue	666.2451	188.907	0.921806	0.025938	16.962
gi|194034148|ref|XM_001924679.1|	turquoise	481.903	136.436	0.776797	0.122259	54.886
gi|194034158|ref|XM_001927669.1|	navy	282.0872	32.5847	0.856632	0.063745	50.492
gi|194034162|ref|XM_001925693.1|	darkorange	151.8765	14.5789	0.861842	0.060351	135.57
gi|194034164|ref|XM_001925794.1|	yellowgreen	188.2427	18.2057	0.992248	0.000818	17.268
gi|194034166|ref|XM_001925915.1|	pink	95.65332	6.93954	-0.06497	0.917336	40.52
gi|194034220|ref|XM_001928748.1|	blue	845.8631	198.025	0.930614	0.021711	32.588
gi|194034226|ref|XM_001928937.1|	grey60	998.2986	59.5412	0.985723	0.002043	126.722
gi|194034238|ref|XM_001924848.1|	yellow	252.6582	89.7001	0.934221	0.020051	18.258
gi|194034244|ref|XM_001925555.1|	paleturquoise	230.8075	16.5003	0.909737	0.032109	23.098
gi|194034359|ref|XM_001927037.1|	pink	183.1012	52.0458	0.918037	0.02782	44.598
gi|194034393|ref|XM_001926625.1|	blue	749.9425	226.56	0.963073	0.008471	21.39
gi|194034443|ref|XM_001928249.1|	blue	639.9597	139.618	0.853613	0.065738	4.93
gi|194034463|ref|XM_001926549.1|	red	446.8914	103.026	0.984366	0.002341	95.66
gi|194034501|ref|XM_001925096.1|	darkgreen	272.229	37.7773	0.985395	0.002114	9.198
gi|194034516|ref|XM_001925886.1|	paleturquoise	183.3617	20.5008	0.965517	0.007647	5.476
gi|194034537|ref|XM_001925533.1|	green	146.2075	46.917	0.91506	0.029335	30.196
gi|194034539|ref|XM_001927513.1|	grey60	961.7969	60.8154	0.989948	0.001208	9.534
gi|194034592|ref|XM_001925373.1|	green	176.691	18.3124	0.140065	0.822248	28.932
gi|194034626|ref|XM_001929026.1|	darkgrey	168.826	30.1169	0.964961	0.007832	52.34
gi|194034657|ref|XM_001924580.1|	turquoise	641.6887	212.536	0.875256	0.051888	38.634
gi|194034678|ref|XM_001928189.1|	darkgrey	174.4439	30.2679	0.9664	0.007356	19.506
gi|194034680|ref|XM_001925553.1|	brown	43.72305	14.3205	0.442735	0.455291	11.6
gi|194034692|ref|XM_001928709.1|	salmon	154.463	39.7775	0.93034	0.021838	14.758
gi|194034703|ref|XM_001926714.1|	turquoise	455.6053	151.525	0.774891	0.12379	9.778
gi|194034713|ref|XM_001925291.1|	yellow	208.2537	68.6188	0.861295	0.060705	13.564
gi|194034832|ref|XM_001928118.1|	blue	940.4062	203.946	0.941748	0.016729	16.266
gi|194034834|ref|XM_001925793.1|	turquoise	403.1444	136.405	0.775645	0.123184	66.112
gi|194034838|ref|XM_001928143.1|	brown	85.51917	32.9036	0.586621	0.298469	1.468
gi|194034874|ref|XM_001929334.1|	lightyellow	905.2698	62.1801	0.995625	0.000347	5.54
gi|194034890|ref|XM_001929399.1|	brown	91.79244	23.7168	0.556803	0.329615	11.286
gi|194034900|ref|XM_001924788.1|	midnightblue	205.9459	34.5559	0.916677	0.028509	27.452
gi|194034933|ref|XM_001927442.1|	violet	950.857	75.0263	0.998713	5.54E-05	24.2
gi|194034969|ref|XM_001925674.1|	turquoise	710.7502	233.314	0.91232	0.030753	1460.734
gi|194035059|ref|XM_001924311.1|	brown	98.96395	25.6601	0.707504	0.181332	1.54
gi|194035100|ref|XM_001925477.1|	darkmagenta	961.581	26.9156	0.993435	0.000638	15.556
gi|194035108|ref|XM_001926680.1|	purple	86.88527	21.7128	0.901603	0.0365	32.97
gi|194035136|ref|XM_001924656.1|	paleturquoise	137.4029	15.1656	0.891067	0.042447	83.466
gi|194035172|ref|XM_001924332.1|	paleturquoise	134.0747	14.5313	0.881154	0.048296	31.85
gi|194035184|ref|XM_001924626.1|	navy	153.0856	11.5346	-0.57134	0.314331	26.958
gi|194035216|ref|XM_001928520.1|	salmon	301.2788	40.4538	0.932253	0.020951	96.986
gi|194035235|ref|XM_001924419.1|	grey	147.5461	13.9917	-0.89277	0.041469	47.896
gi|194035245|ref|XM_001924870.1|	turquoise	402.5867	104.318	0.704571	0.183977	10.494

gi|194035288|ref|XM_001927095.1|	turquoise	284.3998	89.5992	0.667834	0.217995	15.592
gi|194035296|ref|XM_001927594.1|	turquoise	898.6121	261.192	0.97454	0.004858	65.772
gi|194035304|ref|XM_001928396.1|	grey	161.6478	14.9909	-0.93437	0.019984	6.212
gi|194035333|ref|XM_001925774.1|	brown	186.6806	26.9637	0.577294	0.308127	22.956
gi|194035378|ref|XM_001924314.1|	greenyellow	220.0961	42.7806	0.932626	0.020779	3.918
gi|194035408|ref|XM_001926476.1|	blue	865.3024	227.452	0.962341	0.008723	42.506
gi|194035493|ref|XM_001926740.1|	orange	568.2207	33.0168	0.982011	0.002888	4.98
gi|194035503|ref|XM_001927458.1|	brown	136.4275	31.6557	0.555127	0.33139	40.074
gi|194035509|ref|XM_001927620.1|	pink	220.2194	41.2816	0.829454	0.082349	11.47
gi|194035517|ref|XM_001925462.1|	tan	878.2748	66.6415	0.991596	0.000924	22.512
gi|194035561|ref|XM_001924535.1|	blue	965.6501	222.417	0.96186	0.00889	8.746
gi|194035592|ref|XM_001927531.1|	navy	156.3972	6.26677	0.358933	0.553005	29.55
gi|194035600|ref|XM_001927804.1|	turquoise	805.3322	238.885	0.948993	0.013722	260.366
gi|194035607|ref|XM_001928505.1|	black	982.3185	98.6876	0.998003	0.000107	16.798
gi|194035627|ref|XM_001924280.1|	green	74.21428	34.6343	0.780334	0.119433	25.72
gi|194035657|ref|XM_001928441.1|	turquoise	251.0297	54.8413	0.524011	0.364743	18.726
gi|194035679|ref|XM_001925389.1|	turquoise	345.9063	105.15	0.714741	0.174854	312.33
gi|194035693|ref|XM_001925021.1|	turquoise	913.6352	269.594	0.981549	0.003	7.324
gi|194035716|ref|XM_001925987.1|	turquoise	333.9509	88.8334	0.620507	0.264073	29.974
gi|194035722|ref|XM_001926997.1|	royalblue	208.252	6.46875	0.502731	0.387994	19.588
gi|194035733|ref|XM_001925711.1|	midnightblue	110.2628	18.7787	0.760729	0.135342	11.688
gi|194035737|ref|XM_001928295.1|	royalblue	157.6933	9.36749	0.557467	0.328914	160.806
gi|194035770|ref|XM_001927929.1|	brown	85.16901	33.0374	0.564902	0.321078	2.14
gi|194035776|ref|XM_001929384.1|	purple	86.64428	13.3998	0.927795	0.023037	47.246
gi|194035796|ref|XM_001927740.1|	turquoise	896.0149	250.741	0.968781	0.00659	34.93
gi|194035836|ref|XM_001928869.1|	grey60	956.4633	62.9743	0.997261	0.000172	55.492
gi|194035846|ref|XM_001928960.1|	blue	810.7006	218.865	0.956442	0.010841	90.038
gi|194035848|ref|XM_001928966.1|	green	121.9564	38.5888	0.838497	0.075997	494.762
gi|194035852|ref|XM_001929031.1|	darkturquoise	183.5372	30.7862	0.958794	0.009979	40.736
gi|194035954|ref|XM_001927715.1|	royalblue	187.1107	20.6478	0.83603	0.077714	9.004
gi|194035974|ref|XM_001924643.1|	green	119.0966	33.9734	0.801855	0.102674	20.486
gi|194035999|ref|XM_001924070.1|	turquoise	557.7437	187.665	0.845993	0.070851	225.328
gi|194036005|ref|XM_001926377.1|	lightyellow	997.1174	61.8506	0.994478	0.000492	96.44
gi|194036011|ref|XM_001926398.1|	yellow	217.4493	70.1705	0.869333	0.055575	7.286
gi|194036046|ref|XM_001928851.1|	blue	807.1938	191.194	0.923828	0.024946	21.238
gi|194036068|ref|XM_001926844.1|	tan	845.1779	56.9761	0.960098	0.009511	6.268
gi|194036118|ref|XM_001927231.1|	magenta	253.7862	75.1479	0.979839	0.003426	4.618
gi|194036127|ref|XM_001929512.1|	yellowgreen	156.4013	14.9862	0.947902	0.014162	87.66
gi|194036134|ref|XM_001929587.1|	pink	258.3144	53.6278	0.901905	0.036333	15.912
gi|194036224|ref|XM_001929641.1|	grey	300.3437	12.8667	0.966334	0.007378	82.056
gi|194036226|ref|XM_001929643.1|	lightcyan	244.1518	37.6067	0.942398	0.016451	33.656
gi|194036240|ref|XM_001929654.1|	lightyellow	963.3138	63.3242	0.999312	2.17E-05	19.302
gi|194036252|ref|XM_001929660.1|	purple	91.85103	9.51437	-0.53222	0.355871	45.406
gi|194036256|ref|XM_001929662.1|	violet	931.0103	72.9269	0.993016	0.0007	2.782
gi|194036268|ref|XM_001929668.1|	turquoise	548.9216	163.825	0.836972	0.077057	8.93
gi|194036284|ref|XM_001924881.1|	pink	159.7745	46.705	0.898653	0.038136	33.212
gi|194036295|ref|XM_001926489.1|	tan	929.2363	68.6359	0.997811	0.000123	12.766
gi|194036309|ref|XM_001927637.1|	brown	75.59887	26.4013	0.569449	0.31631	388.142

gi|194036315|ref|XM_001927860.1|	violet	967.3498	74.7119	0.997864	0.000118	77.012
gi|194036317|ref|XM_001927969.1|	tan	773.1072	59.585	0.969063	0.006502	8.484
gi|194036348|ref|XM_001928686.1|	salmon	178.4925	47.211	0.973131	0.005266	22.654
gi|194036390|ref|XM_001929343.1|	pink	83.90439	15.3541	0.30723	0.615067	48.934
gi|194036406|ref|XM_001927918.1|	lightyellow	995.0191	62.667	0.997149	0.000183	21.938
gi|194036424|ref|XM_001925710.1|	darkgrey	155.3508	25.0728	0.917495	0.028094	39.886
gi|194036434|ref|XM_001926977.1|	navy	92.07783	28.5996	0.814105	0.093485	15.264
gi|194036458|ref|XM_001928544.1|	turquoise	935.6276	269.367	0.986139	0.001955	3.656
gi|194036460|ref|XM_001926279.1|	darkorange	155.9159	20.8064	0.943879	0.015825	6.888
gi|194036462|ref|XM_001928649.1|	turquoise	845.8552	260.825	0.969802	0.006271	70.654
gi|194036467|ref|XM_001928805.1|	blue	780.6029	216.106	0.948057	0.0141	37.092
gi|194036542|ref|XM_001925670.1|	turquoise	305.0244	70.201	0.60927	0.275356	100.412
gi|194036571|ref|XM_001927806.1|	turquoise	934.8652	258.145	0.984206	0.002377	37.834
gi|194036621|ref|XM_001927321.1|	brown	91.06317	37.3743	0.61407	0.270521	118.942
gi|194036623|ref|XM_001927665.1|	blue	541.8101	152.131	0.873397	0.053037	14.874
gi|194036639|ref|XM_001924618.1|	darkgrey	162.0982	27.9207	0.938299	0.018227	48.152
gi|194036656|ref|XM_001928010.1|	tan	921.77	65.04	0.985749	0.002038	28.264
gi|194036668|ref|XM_001926328.1|	blue	849.0143	246.134	0.984209	0.002376	22.214
gi|194036687|ref|XM_001926116.1|	green	111.9378	61.7205	0.993084	0.00069	194.234
gi|194036700|ref|XM_001927375.1|	black	956.2138	94.5446	0.989358	0.001316	9.32
gi|194036737|ref|XM_001927243.1|	turquoise	945.2664	267.012	0.990063	0.001187	62.046
gi|194036739|ref|XM_001927255.1|	turquoise	532.7841	135.137	0.797551	0.105964	15.358
gi|194036748|ref|XM_001928875.1|	greenyellow	202.2133	54.714	0.994627	0.000472	7.872
gi|194036762|ref|XM_001925928.1|	turquoise	950.3888	271.442	0.995154	0.000405	126.688
gi|194036768|ref|XM_001927459.1|	turquoise	529.3778	176.015	0.864499	0.058643	2.704
gi|194036770|ref|XM_001925929.1|	midnightblue	141.3761	36.0412	0.926731	0.023544	7.774
gi|194036795|ref|XM_001926863.1|	brown	110.8326	28.0072	0.741129	0.151821	41.74
gi|194036805|ref|XM_001928924.1|	midnightblue	164.4441	40.8077	0.962774	0.008574	34.222
gi|194036851|ref|XM_001928643.1|	turquoise	340.2573	111.37	0.726689	0.164308	45.586
gi|194036879|ref|XM_001927666.1|	blue	795.9015	196.662	0.931964	0.021084	229.56
gi|194036893|ref|XM_001927714.1|	blue	704.4409	209.634	0.944388	0.015611	16.064
gi|194036901|ref|XM_001927323.1|	tan	926.0184	66.9487	0.992401	0.000794	53.808
gi|194036936|ref|XM_001925783.1|	turquoise	268.7037	80.1374	0.618594	0.265985	13.186
gi|194036950|ref|XM_001928451.1|	sienna3	971.2171	23.2389	0.976713	0.004251	15.93
gi|194036956|ref|XM_001924342.1|	salmon	262.6368	45.6961	0.962815	0.00856	19.776
gi|194036972|ref|XM_001927228.1|	blue	751.763	166.354	0.893363	0.041127	22.542
gi|194036974|ref|XM_001927747.1|	navy	274.8684	27.3027	0.767853	0.129492	261.176
gi|194037015|ref|XM_001929116.1|	turquoise	483.2103	97.8245	0.722893	0.167639	98.864
gi|194037094|ref|XM_001927574.1|	midnightblue	137.8873	35.5807	0.92299	0.025355	138.698
gi|194037139|ref|XM_001925875.1|	pink	116.0606	21.6344	0.636471	0.248264	10.512
gi|194037172|ref|XM_001928663.1|	pink	288.7351	69.1228	0.958254	0.010175	58.214
gi|194037193|ref|XM_001929169.1|	darkmagenta	886.1265	25.2637	0.980496	0.00326	9.87
gi|194037198|ref|XM_001929213.1|	turquoise	168.8525	47.8366	0.444833	0.452898	46.564
gi|194037354|ref|XM_001926198.1|	magenta	271.3623	77.1143	0.98477	0.002251	27.72
gi|194037372|ref|XM_001927549.1|	blue	775.9735	225.104	0.958719	0.010006	28.362
gi|194037379|ref|XM_001925252.1|	lightyellow	961.6051	63.1842	0.998873	4.54E-05	15.102
gi|194037499|ref|XM_001928879.1|	skyblue	182.4084	30.6301	0.987173	0.001741	22.122
gi|194037532|ref|XM_001929272.1|	pink	282.4987	48.3518	0.854618	0.065072	345.362

gi|194037543|ref|XM_001929293.1|	turquoise	855.9705	250.253	0.968856	0.006567	27.42
gi|194037553|ref|XM_001929410.1|	red	484.8697	109.634	0.997698	0.000133	867.136
gi|194037555|ref|XM_001929413.1|	navy	135.2249	40.8175	0.936987	0.018808	203.322
gi|194037571|ref|XM_001929457.1|	darkmagenta	893.6666	25.0977	0.979691	0.003464	12.368
gi|194037611|ref|XM_001925424.1|	pink	183.6751	50.0974	0.912432	0.030694	20.76
gi|194037617|ref|XM_001927127.1|	turquoise	554.5856	148.127	0.82088	0.088516	34.656
gi|194037680|ref|XM_001926355.1|	tan	649.7879	48.6092	0.925086	0.024335	11.066
gi|194037682|ref|XM_001927000.1|	darkgrey	216.5537	32.2873	0.978951	0.003654	27.436
gi|194037707|ref|XM_001924251.1|	skyblue	261.1148	30.2876	0.977752	0.00397	14.84
gi|194037755|ref|XM_001925059.1|	yellow	255.8213	91.8695	0.929971	0.022011	16.118
gi|194037797|ref|XM_001925445.1|	red	539.7694	107.228	0.992871	0.000722	21.686
gi|194037819|ref|XM_001928548.1|	turquoise	717.8124	235.512	0.91865	0.02751	4.614
gi|194037863|ref|XM_001925621.1|	royalblue	147.1638	13.9479	0.711684	0.177583	62.554
gi|194037865|ref|XM_001928013.1|	tan	680.1668	50.1113	0.93178	0.021169	42.366
gi|194037879|ref|XM_001925784.1|	tan	945.0851	68.5723	0.997451	0.000154	17.48
gi|194037903|ref|XM_001927180.1|	red	442.1542	103.4	0.984878	0.002227	18.88
gi|194037930|ref|XM_001928852.1|	black	953.795	98.524	0.998003	0.000107	30.15
gi|194037950|ref|XM_001926744.1|	green	96.03611	16.215	0.491902	0.399956	8.518
gi|194037952|ref|XM_001924515.1|	blue	814.3735	210.85	0.942673	0.016334	58.406
gi|194037997|ref|XM_001926200.1|	blue	590.3223	134.702	0.845425	0.071237	23.21
gi|194038023|ref|XM_001928502.1|	darkorange	149.6911	19.0211	0.953306	0.012027	38.722
gi|194038027|ref|XM_001928638.1|	lightyellow	944.4728	63.1271	0.998695	5.66E-05	4.084
gi|194038031|ref|XM_001928655.1|	salmon	143.8825	33.9743	0.889126	0.043573	7.16
gi|194038037|ref|XM_001929120.1|	greenyellow	198.8508	50.3984	0.974644	0.004828	91.168
gi|194038045|ref|XM_001925447.1|	blue	771.5207	223.508	0.963441	0.008345	10.212
gi|194038066|ref|XM_001927909.1|	blue	608.5952	174.819	0.9055	0.034374	177.242
gi|194038088|ref|XM_001925288.1|	turquoise	493.1399	109.161	0.726547	0.164433	40.872
gi|194038104|ref|XM_001925531.1|	blue	561.083	118.298	0.819698	0.089377	23.016
gi|194038194|ref|XM_001927473.1|	turquoise	182.0386	42.1231	0.458064	0.437866	80.078
gi|194038223|ref|XM_001926312.1|	darkgreen	246.2013	33.5443	0.938246	0.01825	4.916
gi|194038225|ref|XM_001926546.1|	pink	224.9883	38.5148	0.816173	0.091959	13.742
gi|194038241|ref|XM_001927985.1|	turquoise	676.223	223.675	0.900047	0.03736	35.632
gi|194038245|ref|XM_001928187.1|	greenyellow	134.2576	33.6988	0.872738	0.053445	172.428
gi|194038277|ref|XM_001926890.1|	grey60	941.931	62.29	0.995057	0.000417	35.566
gi|194038297|ref|XM_001926748.1|	turquoise	868.5568	241.736	0.955156	0.011323	4.66
gi|194038313|ref|XM_001925341.1|	greenyellow	184.1793	51.2446	0.978277	0.003831	115.04
gi|194038369|ref|XM_001929075.1|	yellow	201.4081	59.8966	0.841889	0.073655	69.342
gi|194038432|ref|XM_001925495.1|	sienna3	919.3437	25.633	0.996229	0.000278	18.714
gi|194038464|ref|XM_001924308.1|	navy	150.1102	42.3165	0.965043	0.007804	332.15
gi|194038478|ref|XM_001926160.1|	turquoise	911.626	265.449	0.978131	0.003869	13.48
gi|194038505|ref|XM_001928042.1|	red	632.5955	99.4023	0.975238	0.00466	19.136
gi|194038533|ref|XM_001929074.1|	turquoise	681.6312	225.014	0.898841	0.038031	49.75
gi|194038541|ref|XM_001929173.1|	green	134.1047	42.1273	0.881808	0.047903	87.12
gi|194038545|ref|XM_001929151.1|	darkgreen	241.3591	34.9315	0.951314	0.012801	11.66
gi|194038579|ref|XM_001926122.1|	blue	978.4778	233.264	0.972156	0.005554	24.56
gi|194038585|ref|XM_001926711.1|	navy	193.521	14.2361	0.489098	0.403066	201.464
gi|194038610|ref|XM_001926123.1|	red	323.1933	74.4386	0.91451	0.029618	9.818
gi|194038708|ref|XM_001928665.1|	violet	978.5996	74.4686	0.99721	0.000177	121.05

gi|194038714|ref|XM_001926092.1|	turquoise	450.1252	126.896	0.77038	0.127436	8.744
gi|194038723|ref|XM_001929103.1|	pink	95.22659	15.0558	0.153682	0.805099	22.28
gi|194038729|ref|XM_001929090.1|	midnightblue	163.1643	42.7289	0.979433	0.00353	435.296
gi|194038781|ref|XM_001926473.1|	blue	879.8301	225.34	0.96329	0.008397	95.484
gi|194038808|ref|XM_001924784.1|	turquoise	139.3534	43.3377	0.443236	0.454719	90.608
gi|194038816|ref|XM_001925028.1|	turquoise	743.4067	228.898	0.917707	0.027986	7.486
gi|194038854|ref|XM_001928014.1|	orange	349.477	31.6226	0.970099	0.006179	15.852
gi|194038856|ref|XM_001925833.1|	white	881.545	28.9541	0.832233	0.080381	15.706
gi|194038862|ref|XM_001928271.1|	purple	119.3315	18.0196	0.747035	0.146797	59.938
gi|194038869|ref|XM_001928468.1|	lightyellow	803.2478	57.3822	0.979412	0.003535	2.34
gi|194038879|ref|XM_001928723.1|	turquoise	407.8391	130.382	0.761271	0.134894	8.2
gi|194038896|ref|XM_001924415.1|	navy	64.35461	5.53704	-0.07149	0.909052	37.826
gi|194038900|ref|XM_001925133.1|	turquoise	446.2598	151.363	0.771936	0.126175	20.734
gi|194038904|ref|XM_001924445.1|	darkmagenta	962.0912	26.7377	0.99177	0.000895	191.306
gi|194038910|ref|XM_001926121.1|	magenta	250.5831	77.1269	0.986672	0.001843	14.434
gi|194038924|ref|XM_001927577.1|	lightyellow	969.0572	63.3827	0.999497	1.36E-05	31.654
gi|194038958|ref|XM_001929118.1|	tan	903.0567	67.7928	0.99554	0.000357	18.33
gi|194039226|ref|XM_001928899.1|	grey	175.3473	11.729	0.387028	0.519815	222.37
gi|194039238|ref|XM_001924410.1|	violet	923.9278	72.6127	0.992143	0.000835	3.28
gi|194039264|ref|XM_001927148.1|	white	175.2323	21.095	0.709521	0.179521	25.722
gi|194039300|ref|XM_001928656.1|	brown	92.5233	22.3856	0.691663	0.195744	4.25
gi|194039310|ref|XM_001929246.1|	red	682.0386	101.288	0.979307	0.003562	23.744
gi|194039316|ref|XM_001929265.1|	purple	95	13.7912	0.736493	0.155799	72.066
gi|194039362|ref|XM_001929482.1|	green	157.0758	41.6463	0.891538	0.042175	38.924
gi|194039364|ref|XM_001929510.1|	grey60	804.4638	54.6482	0.968347	0.006728	9.754
gi|194039388|ref|XM_001929568.1|	blue	678.8386	205.636	0.939781	0.017578	20.524
gi|194039390|ref|XM_001929570.1|	turquoise	667.0287	183.216	0.884705	0.046174	375.328
gi|194039392|ref|XM_001929581.1|	salmon	258.5169	43.7403	0.950149	0.013261	10.976
gi|194039480|ref|XM_001926227.1|	sienna3	995.9706	25.364	0.994064	0.000549	82.714
gi|194039486|ref|XM_001927002.1|	yellow	247.5099	83.4507	0.915426	0.029148	43.004
gi|194039490|ref|XM_001927338.1|	red	647.6362	100.761	0.9789	0.003668	221.372
gi|194039546|ref|XM_001928863.1|	turquoise	735.8243	239.751	0.923612	0.025051	13.1
gi|194039573|ref|XM_001927296.1|	blue	830.1221	199.443	0.932392	0.020887	51.52
gi|194039577|ref|XM_001927339.1|	green	127.6047	53.2108	0.950138	0.013265	32.332
gi|194039585|ref|XM_001929378.1|	midnightblue	51.68645	12.7552	0.720842	0.169446	12.966
gi|194039587|ref|XM_001929390.1|	turquoise	526.138	166.609	0.842939	0.072934	26.588
gi|194039595|ref|XM_001929425.1|	tan	912.749	53.7084	0.946749	0.014633	14.456
gi|194039625|ref|XM_001925622.1|	blue	531.7364	125.004	0.830053	0.081924	3.39
gi|194039636|ref|XM_001927064.1|	pink	133.9226	11.1933	0.014556	0.981468	20.36
gi|194039679|ref|XM_001928732.1|	turquoise	847.9781	259.268	0.970972	0.005911	140.482
gi|194039687|ref|XM_001928955.1|	navy	179.3504	26.0351	0.745608	0.148006	23.954
gi|194039697|ref|XM_001929172.1|	blue	911.7039	199.628	0.936776	0.018901	33.478
gi|194039707|ref|XM_001929228.1|	skyblue	208.1497	28.2469	0.96505	0.007802	174.49
gi|194039709|ref|XM_001929236.1|	navy	130.8422	36.8908	0.903108	0.035674	75.234
gi|194039733|ref|XM_001928398.1|	paleturquoise	162.5733	15.1354	0.897208	0.038946	8.956
gi|194039735|ref|XM_001928389.1|	salmon	315.0477	35.5896	0.895596	0.039855	300.868
gi|194039851|ref|XM_001928253.1|	blue	938.2549	233.92	0.972183	0.005546	50.872
gi|194039979|ref|XM_001927951.1|	grey60	975.5428	61.86	0.993509	0.000627	3.166

gi|194040110|ref|XM_001929541.1|	blue	842.9525	231.205	0.966519	0.007317	17.352
gi|194040173|ref|XM_001926225.1|	blue	790.9397	225.976	0.959145	0.009852	50.048
gi|194040232|ref|XM_001929613.1|	violet	959.6363	75.2566	0.999329	2.08E-05	15.852
gi|194040254|ref|XM_001929631.1|	greenyellow	204.2699	52.933	0.98921	0.001343	34.666
gi|194040263|ref|XM_001927880.1|	blue	855.2298	234.946	0.969915	0.006236	23.59
gi|194040280|ref|XM_001925339.1|	red	408.0794	86.1942	0.944252	0.015668	8.666
gi|194040288|ref|XM_001927655.1|	purple	35.86712	6.90554	0.3593	0.552568	0.96
gi|194040321|ref|XM_001928400.1|	tan	921.2054	67.9716	0.9959	0.000315	21.998
gi|194040325|ref|XM_001928445.1|	grey60	961.9348	60.3554	0.988385	0.0015	1.838
gi|194040339|ref|XM_001928739.1|	darkred	217.3701	37.9297	0.958156	0.01021	36.146
gi|194040341|ref|XM_001928820.1|	navy	288.2681	28.0711	0.796614	0.106685	26.778
gi|194040377|ref|XM_001929415.1|	violet	903.1629	73.4465	0.994365	0.000507	10.266
gi|194040411|ref|XM_001929558.1|	turquoise	555.4994	155.706	0.819394	0.089599	151.278
gi|194040420|ref|XM_001929571.1|	darkred	182.3532	35.151	0.930634	0.021701	25.016
gi|194040422|ref|XM_001929580.1|	violet	977.5664	74.4579	0.997176	0.00018	100.576
gi|194040449|ref|XM_001927957.1|	midnightblue	131.4202	45.3452	0.989981	0.001202	157.2
gi|194040485|ref|XM_001926913.1|	lightcyan	135.9143	10.2715	0.467262	0.427484	27.184
gi|194040487|ref|XM_001926988.1|	pink	80.69845	7.24198	0.204915	0.740931	8.206
gi|194040489|ref|XM_001927223.1|	turquoise	809.3883	253.648	0.965695	0.007588	20.908
gi|194040496|ref|XM_001928226.1|	turquoise	859.5822	259.432	0.961822	0.008903	2.08
gi|194040529|ref|XM_001924631.1|	blue	805.1238	209.119	0.945442	0.015172	11.47
gi|194040558|ref|XM_001928742.1|	turquoise	354.5369	116.087	0.689602	0.197642	20.194
gi|194040603|ref|XM_001928485.1|	blue	705.4799	161.859	0.886775	0.04495	19.028
gi|194040616|ref|XM_001929023.1|	brown	142.567	19.0246	0.472971	0.421068	15.71
gi|194040641|ref|XM_001925120.1|	violet	970.7774	72.7159	0.992409	0.000793	14.086
gi|194040677|ref|XM_001927466.1|	darkorange	195.5229	17.2581	0.957209	0.010557	7.91
gi|194040706|ref|XM_001927201.1|	lightcyan	301.5058	41.852	0.977979	0.00391	148.794
gi|194040743|ref|XM_001927771.1|	black	935.7865	96.6736	0.993857	0.000577	25.316
gi|194040755|ref|XM_001927097.1|	green	153.2654	20.6241	0.108539	0.862076	53.676
gi|194040784|ref|XM_001927731.1|	blue	767.8136	219.036	0.957509	0.010447	29.708
gi|194040788|ref|XM_001925300.1|	black	960.2119	96.5042	0.993141	0.000681	33.384
gi|194040816|ref|XM_001925581.1|	green	133.4246	43.1403	0.887424	0.044569	34.078
gi|194040900|ref|XM_001928469.1|	turquoise	599.4706	165.469	0.856602	0.063764	84.828
gi|194040929|ref|XM_001925268.1|	darkred	243.0272	30.3571	0.911295	0.031289	14.726
gi|194040939|ref|XM_001924716.1|	blue	822.8504	229.685	0.970291	0.00612	36.628
gi|194040961|ref|XM_001924920.1|	blue	718.4498	178.227	0.910508	0.031703	7.584
gi|194040979|ref|XM_001928486.1|	brown	92.90766	37.4412	0.822309	0.087479	293.604
gi|194040981|ref|XM_001928597.1|	blue	827.2707	208.926	0.941001	0.01705	48.94
gi|194040983|ref|XM_001927342.1|	darkturquoise	184.3363	29.9492	0.953656	0.011893	106.51
gi|194040993|ref|XM_001924500.1|	brown	63.34219	25.2329	0.646344	0.238619	14.48
gi|194041007|ref|XM_001926441.1|	blue	800.8697	199.834	0.936827	0.018879	52.68
gi|194041034|ref|XM_001928236.1|	turquoise	677.5878	173.198	0.866919	0.057101	16.592
gi|194041080|ref|XM_001924608.1|	lightcyan	176.252	12.8935	0.505488	0.384962	6.702
gi|194041087|ref|XM_001927225.1|	magenta	270.1832	79.5407	0.992624	0.00076	61.502
gi|194041154|ref|XM_001925630.1|	turquoise	429.4907	126.146	0.762698	0.133718	11.542
gi|194041236|ref|XM_001928454.1|	violet	984.8514	74.9802	0.998573	6.47E-05	69.484
gi|194041241|ref|XM_001928861.1|	grey60	958.5502	61.9787	0.99397	0.000562	565.862
gi|194041305|ref|XM_001927960.1|	salmon	158.7493	37.8016	0.91942	0.027124	38.372

gi|194041319|ref|XM_001928397.1|	turquoise	416.9031	139.165	0.79087	0.111133	7.71
gi|194041329|ref|XM_001928735.1|	blue	651.3239	197.569	0.932597	0.020793	6.192
gi|194041338|ref|XM_001928892.1|	turquoise	396.8931	125.754	0.75629	0.139025	5.562
gi|194041353|ref|XM_001926876.1|	red	489.4108	107.95	0.994444	0.000497	4.638
gi|194041357|ref|XM_001926917.1|	brown	183.7713	45.3087	0.68129	0.205348	22.454
gi|194041366|ref|XM_001924481.1|	turquoise	623.4241	153.998	0.833011	0.079832	23.126
gi|194041368|ref|XM_001924555.1|	tan	920.5469	67.8438	0.995228	0.000395	30.428
gi|194041393|ref|XM_001928536.1|	turquoise	926.899	257.341	0.976226	0.004385	37.074
gi|194041450|ref|XM_001928163.1|	salmon	140.8313	32.9762	0.886578	0.045066	45.974
gi|194041518|ref|XM_001927414.1|	darkorange	184.5846	17.688	0.923977	0.024873	45.9
gi|194041588|ref|XM_001927189.1|	white	190.169	26.1202	0.809165	0.097159	8.658
gi|194041596|ref|XM_001927803.1|	blue	838.0416	193.992	0.926873	0.023476	7.5
gi|194041636|ref|XM_001929009.1|	blue	699.2441	210.185	0.945865	0.014997	58.392
gi|194041732|ref|XM_001927648.1|	blue	841.0428	236.39	0.974503	0.004868	13.49
gi|194041758|ref|XM_001924615.1|	orange	352.623	33.2251	0.980918	0.003155	23.942
gi|194041760|ref|XM_001924661.1|	skyblue	259.7392	30.3594	0.982862	0.002686	3.762
gi|194041762|ref|XM_001927529.1|	turquoise	345.5294	101.321	0.691839	0.195583	7.79
gi|194041780|ref|XM_001925192.1|	turquoise	415.3426	100.903	0.691268	0.196108	66.71
gi|194041796|ref|XM_001928803.1|	turquoise	904.6587	256.216	0.971795	0.005662	11.568
gi|194041820|ref|XM_001929110.1|	grey60	968.3563	61.1952	0.991464	0.000946	11.394
gi|194041839|ref|XM_001929344.1|	royalblue	269.3096	18.0018	0.814107	0.093483	13.434
gi|194041859|ref|XM_001924277.1|	greenyellow	223.7478	44.9288	0.942613	0.01636	10.59
gi|194041878|ref|XM_001924973.1|	green	122.4704	60.4092	0.990826	0.001053	52.008
gi|194041914|ref|XM_001928393.1|	turquoise	524.5656	173.662	0.85884	0.062299	48.172
gi|194041916|ref|XM_001925662.1|	violet	951.5732	74.0329	0.996043	0.000299	7.552
gi|194041932|ref|XM_001928812.1|	purple	173.1183	16.9088	0.734907	0.157166	40.218
gi|194041960|ref|XM_001929335.1|	red	593.2961	101.317	0.979378	0.003544	15.932
gi|194041962|ref|XM_001926774.1|	tan	922.3998	64.0569	0.982979	0.002659	8.576
gi|194042016|ref|XM_001926579.1|	white	183.9357	25.7207	0.848011	0.069485	45.216
gi|194042046|ref|XM_001928978.1|	red	479.7531	96.167	0.96854	0.006667	12.256
gi|194042125|ref|XM_001928342.1|	white	892.3154	29.4021	0.853653	0.065711	197.586
gi|194042129|ref|XM_001927395.1|	darkmagenta	909.6708	25.835	0.985003	0.0022	14.356
gi|194042141|ref|XM_001929000.1|	turquoise	429.7612	127.836	0.75037	0.143982	151.022
gi|194042170|ref|XM_001929360.1|	blue	873.9237	199.919	0.939795	0.017572	8.34
gi|194042188|ref|XM_001924178.1|	turquoise	714.2627	225.462	0.93865	0.018072	41.554
gi|194042190|ref|XM_001924717.1|	turquoise	825.1207	254.732	0.951343	0.01279	20.384
gi|194042230|ref|XM_001927832.1|	turquoise	296.3964	61.8577	0.570179	0.315546	24.682
gi|194042273|ref|XM_001927415.1|	darkturquoise	167.4626	30.5483	0.964758	0.0079	23.87
gi|194042287|ref|XM_001927955.1|	blue	896.3825	234.526	0.970052	0.006193	93.886
gi|194042295|ref|XM_001928522.1|	turquoise	343.609	83.9484	0.624236	0.260356	36.238
gi|194042303|ref|XM_001928772.1|	blue	722.8391	189.505	0.923843	0.024939	112.468
gi|194042305|ref|XM_001928810.1|	yellow	190.1313	63.5969	0.844018	0.072196	62.02
gi|194042311|ref|XM_001925999.1|	turquoise	527.4287	126.354	0.7799	0.119778	21.496
gi|194042329|ref|XM_001929115.1|	black	977.2427	99.2511	0.999349	1.99E-05	6.576
gi|194042367|ref|XM_001926555.1|	black	970.4379	98.5502	0.997732	0.00013	46.264
gi|194042373|ref|XM_001925458.1|	yellow	220.4879	78.1894	0.896281	0.039468	11.252
gi|194042394|ref|XM_001927941.1|	blue	873.8608	241.322	0.9778	0.003957	6.212
gi|194042396|ref|XM_001927868.1|	blue	755.6306	178.742	0.91117	0.031355	4.888

gi|194042438|ref|XM_001927977.1|	turquoise	813.1318	254.339	0.954169	0.011697	33.974
gi|194042457|ref|XM_001928703.1|	brown	118.5334	44.5793	0.832409	0.080256	6.372
gi|194042459|ref|XM_001928724.1|	magenta	241.0392	76.3599	0.984178	0.002383	21.684
gi|194042469|ref|XM_001928931.1|	darkgreen	243.4491	18.9813	0.786131	0.114844	2.26
gi|194042472|ref|XM_001924771.1|	turquoise	403.393	116.469	0.726971	0.164062	26.762
gi|194042477|ref|XM_001926521.1|	salmon	222.9539	38.8809	0.922442	0.025625	69.588
gi|194042479|ref|XM_001926556.1|	paleturquoise	155.0515	14.3636	0.869321	0.055583	5.792
gi|194042490|ref|XM_001925381.1|	yellow	256.158	85.2467	0.923328	0.02519	69.2
gi|194042528|ref|XM_001927272.1|	darkmagenta	987.5245	26.6954	0.991376	0.00096	978.536
gi|194042562|ref|XM_001925435.1|	brown	197.5553	47.1374	0.7164	0.173379	10.732
gi|194042583|ref|XM_001928329.1|	pink	127.5384	10.1107	-0.1658	0.789863	21.624
gi|194042598|ref|XM_001924401.1|	yellow	159.4008	40.3849	0.732339	0.159388	38.666
gi|194042624|ref|XM_001927934.1|	blue	842.7471	230.544	0.968302	0.006742	10.538
gi|194042632|ref|XM_001928164.1|	grey60	950.4739	63.05	0.997423	0.000157	8.012
gi|194042649|ref|XM_001928781.1|	turquoise	507.6462	149.455	0.805015	0.100278	19.852
gi|194042680|ref|XM_001929224.1|	lightcyan	256.445	31.8821	0.909736	0.03211	9.758
gi|194042688|ref|XM_001929282.1|	violet	851.5443	70.4304	0.985856	0.002015	14.528
gi|194042690|ref|XM_001929322.1|	royalblue	279.2881	26.1155	0.941189	0.016969	61.568
gi|194042702|ref|XM_001925079.1|	grey60	947.8703	63.2624	0.998158	9.49E-05	12.518
gi|194042725|ref|XM_001925659.1|	yellow	232.3145	74.7166	0.8915	0.042197	7.464
gi|194042749|ref|XM_001925080.1|	turquoise	388.1671	106.85	0.704715	0.183847	143.644
gi|194042783|ref|XM_001924319.1|	paleturquoise	201.3674	19.4315	0.957584	0.010419	5.042
gi|194042787|ref|XM_001924736.1|	purple	96.47347	22.2132	0.919684	0.026992	49.33
gi|194042789|ref|XM_001925275.1|	red	450.1993	101.938	0.981368	0.003044	54.314
gi|194042801|ref|XM_001927517.1|	blue	831.2691	240.192	0.979459	0.003523	36.676
gi|194042805|ref|XM_001927802.1|	skyblue	252.9134	31.9141	0.992973	0.000706	191.054
gi|194042811|ref|XM_001925327.1|	greenyellow	157.1301	37.3605	0.903762	0.035317	223.288
gi|194042813|ref|XM_001928193.1|	turquoise	508.6148	119.295	0.749394	0.144805	20.924
gi|194042821|ref|XM_001928374.1|	darkmagenta	985.7587	25.4987	0.982161	0.002853	27.514
gi|194042829|ref|XM_001928949.1|	blue	822.941	243.681	0.981371	0.003044	10.464
gi|194042833|ref|XM_001929033.1|	purple	50.52592	13.357	0.67646	0.209863	31.898
gi|194042835|ref|XM_001929055.1|	turquoise	577.3997	148.794	0.812747	0.09449	10.296
gi|194042841|ref|XM_001929155.1|	black	988.2076	97.1497	0.994595	0.000477	9.058
gi|194042857|ref|XM_001927712.1|	turquoise	354.5181	87.1032	0.639415	0.245377	41.764
gi|194042859|ref|XM_001927602.1|	lightyellow	956.4324	63.0856	0.998575	6.46E-05	22.242
gi|194042865|ref|XM_001925507.1|	blue	713.8232	217.516	0.954101	0.011723	13.848
gi|194042903|ref|XM_001928981.1|	greenyellow	161.2911	42.2002	0.931141	0.021465	46.526
gi|194042939|ref|XM_001929407.1|	skyblue	191.6508	28.9689	0.971433	0.005771	7.474
gi|194042943|ref|XM_001929420.1|	turquoise	783.7875	241.402	0.960376	0.009412	14.136
gi|194042960|ref|XM_001929554.1|	turquoise	802.0518	247.912	0.947847	0.014185	13.284
gi|194042969|ref|XM_001929585.1|	salmon	289.1923	39.544	0.924773	0.024487	338.674
gi|194043008|ref|XM_001928156.1|	lightyellow	996.7851	61.6339	0.993773	0.000589	6.44
gi|194043036|ref|XM_001924558.1|	grey60	920.2537	61.4789	0.992329	0.000806	8.584
gi|194043042|ref|XM_001926896.1|	lightcyan	259.616	36.294	0.935772	0.01935	18.256
gi|194043052|ref|XM_001927455.1|	tan	827.6358	64.471	0.985001	0.0022	21.452
gi|194043063|ref|XM_001925272.1|	tan	907.0298	66.0685	0.989515	0.001287	17.712
gi|194043066|ref|XM_001928232.1|	turquoise	886.0328	265.453	0.976618	0.004277	60.734
gi|194043070|ref|XM_001928504.1|	red	637.6222	93.9335	0.963267	0.008405	6.454

gi|194043072|ref|XM_001925326.1|	blue	726.6863	212.673	0.949064	0.013694	6.462
gi|194043074|ref|XM_001928487.1|	turquoise	382.3837	106.733	0.70304	0.185362	31.638
gi|194043076|ref|XM_001925356.1|	grey60	960.6564	60.4508	0.988865	0.001408	44.912
gi|194043112|ref|XM_001929270.1|	black	989.9807	98.7966	0.998299	8.42E-05	57.244
gi|194043122|ref|XM_001929345.1|	blue	832.1692	210.799	0.948716	0.013834	18.068
gi|194043175|ref|XM_001928565.1|	violet	944.9502	72.9071	0.993022	0.000699	3.78
gi|194043187|ref|XM_001925978.1|	turquoise	890.0419	263.556	0.971659	0.005703	26.572
gi|194043225|ref|XM_001929262.1|	royalblue	346.1074	32.7974	0.993786	0.000587	8.604
gi|194043233|ref|XM_001929306.1|	tan	925.8686	65.864	0.988472	0.001483	21.116
gi|194043243|ref|XM_001924074.1|	lightcyan	287.4385	40.248	0.977869	0.003939	60.576
gi|194043277|ref|XM_001927490.1|	turquoise	645.1845	162.377	0.847142	0.070073	10.028
gi|194043291|ref|XM_001928076.1|	black	991.7848	97.8503	0.99624	0.000277	44.674
gi|194043329|ref|XM_001929273.1|	red	669.417	100.972	0.979248	0.003577	31.996
gi|194043337|ref|XM_001926296.1|	black	919.1308	95.6936	0.992212	0.000824	6.556
gi|194043361|ref|XM_001929445.1|	turquoise	824.8591	243.332	0.94412	0.015723	3.158
gi|194043363|ref|XM_001929468.1|	purple	123.5499	19.3605	0.90817	0.032941	139.532
gi|194043378|ref|XM_001929539.1|	black	958.6912	93.6756	0.987054	0.001765	6.796
gi|194043405|ref|XM_001929590.1|	red	371.7348	70.8624	0.904606	0.034858	7.736
gi|194043439|ref|XM_001927481.1|	pink	136.6307	2.8397	-0.97092	0.005926	10.342
gi|194043453|ref|XM_001927547.1|	darkgrey	156.9591	26.6009	0.926079	0.023857	12.668
gi|194043490|ref|XM_001927785.1|	tan	942.3017	67.8939	0.995096	0.000412	15.608
gi|194043531|ref|XM_001926134.1|	blue	534.6487	142.314	0.859012	0.062187	9.82
gi|194043583|ref|XM_001928616.1|	turquoise	778.9763	250.001	0.943613	0.015936	2.476
gi|194043591|ref|XM_001927268.1|	turquoise	538.9652	157.207	0.822561	0.087296	31.408
gi|194043669|ref|XM_001924741.1|	turquoise	267.6497	63.06	0.529852	0.358422	53.128
gi|194043695|ref|XM_001925613.1|	white	190.493	20.8344	0.750724	0.143684	3.296
gi|194043731|ref|XM_001926974.1|	darkgreen	274.6459	34.1932	0.969588	0.006337	23.07
gi|194043848|ref|XM_001928131.1|	blue	853.0621	246.804	0.98338	0.002566	13.4
gi|194043858|ref|XM_001928335.1|	blue	772.2354	219.861	0.953255	0.012047	56.898
gi|194043900|ref|XM_001924998.1|	black	982.4577	98.2856	0.99723	0.000175	53.948
gi|194043908|ref|XM_001927890.1|	turquoise	857.4694	261.72	0.975835	0.004493	11.026
gi|194043935|ref|XM_001924322.1|	navy	132.9441	39.397	0.930416	0.021803	4.448
gi|194043951|ref|XM_001927705.1|	green	100.9303	20.7898	0.64656	0.23841	8.284
gi|194043957|ref|XM_001925803.1|	green	81.14182	41.5064	0.875689	0.051621	3212.444
gi|194043959|ref|XM_001927448.1|	navy	167.8973	9.72082	-0.40059	0.503945	8.722
gi|194043976|ref|XM_001928406.1|	darkred	245.5342	34.8353	0.941779	0.016716	10.126
gi|194044012|ref|XM_001927891.1|	tan	952.0768	60.6785	0.97	0.006209	14.838
gi|194044032|ref|XM_001928774.1|	purple	194.8504	16.2933	-0.87084	0.054629	29.68
gi|194044034|ref|XM_001928794.1|	blue	760.2529	200.109	0.937584	0.018543	166.268
gi|194044044|ref|XM_001927332.1|	magenta	207.8089	64.0088	0.943352	0.016047	3.6
gi|194044110|ref|XM_001924775.1|	lightyellow	1002.379	61.1783	0.992245	0.000819	33.352
gi|194044123|ref|XM_001926788.1|	blue	641.3066	136.159	0.846948	0.070204	33.102
gi|194044132|ref|XM_001927798.1|	turquoise	633.5899	179.746	0.867342	0.056833	15.506
gi|194044163|ref|XM_001924534.1|	blue	891.9267	230.584	0.966801	0.007225	39.624
gi|194044169|ref|XM_001924460.1|	grey60	961.2813	60.7951	0.990023	0.001195	50.348
gi|194044196|ref|XM_001928527.1|	royalblue	380.4747	33.3921	0.999152	2.96E-05	11.908
gi|194044237|ref|XM_001927396.1|	blue	842.2083	237.582	0.977122	0.00414	16.326
gi|194044271|ref|XM_001926068.1|	blue	842.8416	226.276	0.96041	0.0094	8.634

gi|194044273|ref|XM_001926112.1|	sienna3	999.721	24.4937	0.987146	0.001746	8.882
gi|194044289|ref|XM_001926487.1|	turquoise	404.7086	82.6403	0.68264	0.20409	48.786
gi|194044331|ref|XM_001925019.1|	skyblue	95.05129	15.9067	0.835781	0.077888	1.84
gi|194044358|ref|XM_001928282.1|	turquoise	287.5246	95.7714	0.680425	0.206154	9.332
gi|194044362|ref|XM_001926993.1|	green	163.7569	21.9742	0.398776	0.50606	35.826
gi|194044424|ref|XM_001927947.1|	blue	836.299	237.355	0.974008	0.005011	2.436
gi|194044465|ref|XM_001928874.1|	midnightblue	149.7997	43.7693	0.974871	0.004764	64.556
gi|194044483|ref|XM_001929150.1|	blue	728.9637	215.742	0.952201	0.012455	190.524
gi|194044504|ref|XM_001927363.1|	salmon	250.4465	48.9753	0.981041	0.003125	55.2
gi|194044550|ref|XM_001927682.1|	purple	78.79043	15.4323	0.676809	0.209536	114.436
gi|194044572|ref|XM_001925488.1|	brown	189.2836	41.1195	0.693676	0.193895	7.9
gi|194044576|ref|XM_001925640.1|	blue	908.2581	192.514	0.931672	0.021219	33.982
gi|194044593|ref|XM_001929063.1|	turquoise	780.3005	243.742	0.933052	0.020584	50.344
gi|194044597|ref|XM_001926862.1|	yellow	260.5975	88.8514	0.924336	0.024699	16.372
gi|194044682|ref|XM_001928813.1|	grey	213.625	11.4598	0.969228	0.00645	8.582
gi|194044686|ref|XM_001928889.1|	lightyellow	997.0074	62.4372	0.996396	0.00026	9.51
gi|194044738|ref|XM_001929453.1|	grey60	872.2639	60.1701	0.987938	0.001587	1.918
gi|194044782|ref|XM_001926654.1|	darkorange	152.1306	15.7878	0.894004	0.04076	42.056
gi|194044788|ref|XM_001928382.1|	darkred	256.3642	38.2442	0.964722	0.007912	7.778
gi|194044819|ref|XM_001926406.1|	grey60	977.7696	61.0754	0.990856	0.001048	34.47
gi|194044859|ref|XM_001927209.1|	lightcyan	245.0994	37.8053	0.941225	0.016953	17.282
gi|194044861|ref|XM_001927107.1|	lightyellow	999.8494	61.7927	0.994304	0.000516	5.538
gi|194044921|ref|XM_001927440.1|	black	993.3183	96.2039	0.992598	0.000764	15.008
gi|194044929|ref|XM_001925991.1|	darkred	231.1659	42.7034	0.982428	0.002789	6.364
gi|194044943|ref|XM_001926010.1|	black	964.6182	97.3443	0.994998	0.000424	2.638
gi|194044951|ref|XM_001925471.1|	darkmagenta	947.4995	26.6688	0.991577	0.000927	5.252
gi|194045002|ref|XM_001926288.1|	darkorange	152.3919	22.0577	0.972187	0.005545	166.37
gi|194045026|ref|XM_001925965.1|	red	517.9008	110.213	0.998648	5.97E-05	11.792
gi|194045094|ref|XM_001925372.1|	darkgrey	222.752	29.1674	0.955847	0.011063	7.96
gi|194045111|ref|XM_001927898.1|	navy	192.3158	29.9102	0.913466	0.030157	43.372
gi|194271212|gb|EU714326.1|	yellow	288.5661	109.398	0.977099	0.004146	4.572
gi|194332486|ref|NM_001130213.1|	darkgreen	251.4127	35.2711	0.976221	0.004386	41.528
gi|194474047|ref|NM_001130535.1|	salmon	214.2672	35.5444	0.903035	0.035714	68.006
gi|194474049|ref|NM_001130531.1|	lightgreen	303.4882	56.2589	0.981862	0.002924	3.26
gi|195539469|ref|NM_001130733.1|	salmon	155.9444	37.5662	0.914135	0.029812	143.066
gi|195539471|ref|NM_001130734.1|	blue	790.0937	226.795	0.965915	0.007515	3.432
gi|196259975|ref|NM_001131045.1|	blue	863.1986	232.554	0.968224	0.006767	18.968
gi|197717772|gb|EF113595.2|	royalblue	239.4021	5.32912	0.314685	0.606046	67.372
gi|198282078|ref|NM_001134823.1|	purple	178.0886	17.8461	-0.91114	0.03137	425.244
gi|19919837|gb|AF490841.1|	turquoise	298.1854	103.795	0.735561	0.156602	39.896
gi|201066357|ref|NM_001134968.1|	midnightblue	105.1378	35.8919	0.944056	0.01575	2388.228
gi|209863050|ref|NM_001135960.1|	turquoise	838.2506	251.647	0.967126	0.00712	46.866
gi|209954779|ref|NM_214223.2|	grey60	916.1437	62.5069	0.995726	0.000335	4.636
gi|211578395|ref|NM_001136510.1|	pink	172.83	51.0933	0.9179	0.027889	38.566
gi|212549664|ref|NM_001137639.1|	turquoise	345.7278	114.549	0.690921	0.196426	42.598
gi|213021240|ref|NM_001139472.1|	greenyellow	226.1964	46.8029	0.95569	0.011122	8.698
gi|217416473|ref|NM_001142668.1|	violet	971.6987	73.481	0.994498	0.000489	11.774
gi|217416475|ref|NM_001142669.1|	yellowgreen	181.0771	15.2756	0.950778	0.013012	14.778

gi|218082010|ref|NM_001142666.1|	skyblue	168.8669	28.9843	0.973826	0.005063	19.042
gi|2286009|emb|AJ000786.1|	magenta	279.5705	73.6392	0.97462	0.004835	29.632
gi|2286014|emb|AJ000791.1|	darkgreen	230.6165	29.5264	0.948709	0.013837	76.328
gi|27357069|gb|AY166682.1|	skyblue	132.629	21.6289	0.908613	0.032705	6.524
gi|29150628|gb|AY216477.1|	lightyellow	976.5372	62.4749	0.996626	0.000235	42.086
gi|33772282|gb|AY346132.1|AY34613	turquoise	718.4821	235.477	0.916244	0.028729	10.934
gi|34582610|gb|AY368623.1|	turquoise	415.3862	116.749	0.729241	0.162081	28.25
gi|35384837|gb|AY374470.1|	red	429.6961	104.559	0.98737	0.001701	49.288
gi|37700454|gb|AY421755.1|	grey60	918.7203	61.0011	0.990722	0.001071	3.3
gi|4186150|emb|AJ236938.1|	red	322.9202	73.65	0.909887	0.03203	32.97
gi|45268992|gb|AY550051.1|	yellowgreen	174.2023	18.4178	0.993376	0.000647	29.3
gi|45758483|gb|AY553927.1|	violet	985.2259	72.4136	0.99149	0.000941	10.132
gi|47522609|ref|NM_213938.1|	greenyellow	157.48	37.3421	0.900845	0.036918	106.94
gi|47522617|ref|NM_213934.1|	salmon	155.6562	45.4034	0.963403	0.008358	19.824
gi|47522629|ref|NM_213928.1|	darkred	241.5233	37.5321	0.958523	0.010077	62.138
gi|47522635|ref|NM_213927.1|	pink	307.0865	49.1221	0.862275	0.060072	113.03
gi|47522641|ref|NM_213922.1|	blue	858.5807	243.62	0.981229	0.003079	502.412
gi|47522643|ref|NM_213923.1|	red	656.2582	93.0536	0.96008	0.009517	14.528
gi|47522659|ref|NM_213914.1|	lightyellow	973.7402	63.426	0.999628	8.61E-06	6.474
gi|47522661|ref|NM_213911.1|	grey60	979.0561	58.6743	0.982617	0.002744	64.316
gi|47522667|ref|NM_213910.1|	lightyellow	969.2793	63.4091	0.999574	1.06E-05	3.268
gi|47522679|ref|NM_213904.1|	black	691.8101	67.4442	0.921995	0.025844	13.828
gi|47522681|ref|NM_213901.1|	brown	198.1965	61.4316	0.949989	0.013324	4.71
gi|47522685|ref|NM_213898.1|	magenta	259.2915	77.1994	0.985861	0.002014	92.02
gi|47522689|ref|NM_213896.1|	red	460.8479	98.1623	0.97281	0.00536	27.86
gi|47522691|ref|NM_213897.1|	lightcyan	272.5725	40.8258	0.959605	0.009687	109.84
gi|47522703|ref|NM_213891.1|	blue	889.6683	207.619	0.944161	0.015706	24.33
gi|47522711|ref|NM_213943.1|	violet	950.2477	74.3936	0.997007	0.000196	9.858
gi|47522717|ref|NM_213944.1|	blue	868.978	251.877	0.988109	0.001554	46.866
gi|47522719|ref|NM_213945.1|	skyblue	158.216	28.8139	0.973236	0.005235	16.65
gi|47522737|ref|NM_213954.1|	royalblue	296.0838	22.9165	0.890485	0.042784	207.622
gi|47522751|ref|NM_213961.1|	yellow	295.475	104.059	0.96513	0.007775	7.14
gi|47522753|ref|NM_213962.1|	turquoise	912.6313	271.821	0.988046	0.001566	152.43
gi|47522763|ref|NM_213968.1|	turquoise	290.0197	88.8882	0.63031	0.254334	48.716
gi|47522769|ref|NM_213971.1|	navy	142.632	44.8594	0.975718	0.004526	103.77
gi|47522773|ref|NM_213973.1|	salmon	246.2019	39.3864	0.92651	0.02365	10.336
gi|47522777|ref|NM_213976.1|	blue	804.8902	226.904	0.963267	0.008405	6.586
gi|47522779|ref|NM_213977.1|	midnightblue	152.3716	47.1006	0.998613	6.20E-05	38.68
gi|47522785|ref|NM_213980.1|	turquoise	860.6042	239.546	0.951411	0.012763	102.502
gi|47522789|ref|NM_213982.1|	yellow	230.7524	71.4897	0.885157	0.045905	17.114
gi|47522791|ref|NM_213983.1|	blue	695.7425	202.67	0.933743	0.020268	149.802
gi|47522821|ref|NM_213998.1|	royalblue	69.91508	2.26778	0.157088	0.800815	10.888
gi|47522837|ref|NM_214006.1|	navy	130.8214	37.2169	0.902655	0.035922	44.026
gi|47522843|ref|NM_214009.1|	navy	119.5856	39.1236	0.912544	0.030636	7.56
gi|47522861|ref|NM_214020.1|	midnightblue	137.3326	45.7827	0.991113	0.001004	59.824
gi|47522869|ref|NM_214024.1|	tan	888.355	67.5202	0.994681	0.000465	48.738
gi|47522881|ref|NM_214031.1|	white	150.6487	7.25178	0.296108	0.628568	10.226
gi|47522897|ref|NM_214039.1|	pink	265.0102	75.1512	0.995949	0.000309	105.058

gi|47522913|ref|NM_214049.1|	red	441.0161	103.799	0.985737	0.00204	15.944
gi|47522915|ref|NM_214050.1|	grey	318.1352	12.5089	0.957086	0.010603	10.21
gi|47522935|ref|NM_214060.1|	lightcyan	229.1807	33.3894	0.906745	0.033703	52.474
gi|47522937|ref|NM_214061.1|	brown	100.704	42.9441	0.796477	0.10679	6.444
gi|47522939|ref|NM_214062.1|	yellow	256.8122	89.8558	0.936045	0.019228	107.352
gi|47522941|ref|NM_214063.1|	darkmagenta	982.0127	26.5739	0.990787	0.00106	9.066
gi|47522943|ref|NM_214064.1|	tan	918.3418	68.0149	0.99585	0.000321	21.516
gi|47522957|ref|NM_214072.1|	paleturquoise	179.3318	19.7411	0.965027	0.00781	41.01
gi|47522967|ref|NM_214077.1|	greenyellow	212.127	51.2678	0.977285	0.004095	5.586
gi|47523015|ref|NM_214103.1|	grey	340.197	12.132	0.977487	0.004041	65.36
gi|47523035|ref|NM_214114.1|	sienna3	967.0406	26.0435	0.999415	1.70E-05	87.804
gi|47523037|ref|NM_214115.1|	tan	962.2402	68.8345	0.998501	6.97E-05	63.158
gi|47523039|ref|NM_214116.1|	magenta	260.2851	78.5532	0.988854	0.00141	197.894
gi|47523059|ref|NM_214128.1|	greenyellow	205.5721	49.5207	0.974196	0.004956	8.506
gi|47523085|ref|NM_214144.1|	brown	95.40206	36.4439	0.737924	0.154568	27.1
gi|47523093|ref|NM_213884.1|	brown	191.0893	56.4493	0.812908	0.09437	5.174
gi|47523105|ref|NM_213878.1|	darkturquoise	164.3339	31.7846	0.973947	0.005028	27.654
gi|47523121|ref|NM_213870.1|	pink	148.4186	45.2809	0.882002	0.047786	21.642
gi|47523187|ref|NM_213830.1|	skyblue	178.7451	30.1256	0.981908	0.002913	6.136
gi|47523195|ref|NM_213826.1|	brown	110.7788	42.6991	0.800581	0.103644	8.272
gi|47523213|ref|NM_213817.1|	pink	176.6726	40.3133	0.875397	0.051801	7.724
gi|47523231|ref|NM_213804.1|	brown	100.6947	41.2939	0.825678	0.085048	7.038
gi|47523255|ref|NM_213792.1|	black	920.2016	96.6982	0.994184	0.000532	12.212
gi|47523261|ref|NM_213791.1|	skyblue	323.2202	29.3378	0.970831	0.005954	4.27
gi|47523273|ref|NM_213785.1|	tan	908.8411	63.5084	0.980692	0.003211	4.328
gi|47523281|ref|NM_213781.1|	turquoise	388.0275	107.469	0.688448	0.198707	1.06
gi|47523317|ref|NM_213763.1|	turquoise	548.3794	140.116	0.785284	0.115511	18.966
gi|47523403|ref|NM_214157.1|	darkgreen	292.8041	36.3365	0.984348	0.002345	17.954
gi|47523419|ref|NM_214166.1|	royalblue	238.5871	3.38062	0.465864	0.429058	15.268
gi|47523427|ref|NM_214171.1|	yellowgreen	169.8363	16.6719	0.970661	0.006006	13.712
gi|47523443|ref|NM_214181.1|	brown	201.0806	61.9564	0.922623	0.025536	12.108
gi|47523445|ref|NM_214183.1|	yellow	247.5019	89.1614	0.929416	0.022271	20.27
gi|47523457|ref|NM_214189.1|	magenta	291.1335	54.8267	0.909367	0.032305	1.248
gi|47523461|ref|NM_214192.1|	turquoise	773.6177	230.328	0.927806	0.023032	86.254
gi|47523463|ref|NM_214188.1|	orange	207.1513	25.7231	0.921092	0.026291	12.904
gi|47523471|ref|NM_214193.1|	yellowgreen	165.9563	17.5488	0.9841	0.002401	26.932
gi|47523477|ref|NM_214200.1|	blue	879.5197	239.364	0.976893	0.004202	6.15
gi|47523483|ref|NM_214199.1|	midnightblue	147.8918	26.5829	0.84267	0.073118	108.73
gi|47523487|ref|NM_214201.1|	skyblue	382.6002	27.7014	0.957763	0.010354	100.962
gi|47523491|ref|NM_214204.1|	red	436.0511	105.77	0.989858	0.001224	313.884
gi|47523501|ref|NM_214213.1|	brown	165.8968	24.0989	0.441139	0.457114	41.286
gi|47523511|ref|NM_214214.1|	navy	135.9803	44.0243	0.96295	0.008513	25.448
gi|47523543|ref|NM_214230.1|	blue	825.0236	235.184	0.972378	0.005488	6.486
gi|47523545|ref|NM_214236.1|	midnightblue	79.58401	5.1242	-0.09557	0.878505	182.828
gi|47523553|ref|NM_214240.1|	blue	722.2735	214.592	0.947771	0.014216	27.092
gi|47523561|ref|NM_214245.1|	darkred	287.9114	40.0185	0.974154	0.004969	17.326
gi|47523569|ref|NM_214249.1|	salmon	242.278	46.9752	0.971384	0.005786	12.332
gi|47523597|ref|NM_214266.1|	midnightblue	95.96035	37.1173	0.948966	0.013733	14.768

gi|47523665|ref|NM_214304.1|	turquoise	324.5158	107.597	0.701867	0.186425	21.716
gi|47523669|ref|NM_214306.1|	violet	994.2246	70.5367	0.986109	0.001961	41.282
gi|47523671|ref|NM_214303.1|	brown	86.30895	37.3453	0.637807	0.246953	12.152
gi|47523673|ref|NM_214308.1|	turquoise	633.4107	194.567	0.906186	0.034004	11.414
gi|47523679|ref|NM_214307.1|	navy	192.0561	24.7913	0.706913	0.181864	24.846
gi|47523681|ref|NM_214312.1|	darkgreen	181.7563	27.989	0.892729	0.04149	13.28
gi|47523691|ref|NM_214313.1|	turquoise	892.0626	269.317	0.984846	0.002234	15.852
gi|47523695|ref|NM_214315.1|	blue	909.9456	206.286	0.944352	0.015626	2.16
gi|47523697|ref|NM_214320.1|	lightyellow	922.4254	62.6339	0.997104	0.000187	8.312
gi|47523711|ref|NM_214323.1|	royalblue	179.8411	7.34443	0.399789	0.504877	4.866
gi|47523719|ref|NM_214330.1|	midnightblue	144.1248	44.0428	0.977444	0.004053	553.788
gi|47523721|ref|NM_214331.1|	pink	284.5083	69.4515	0.958397	0.010122	100.006
gi|47523723|ref|NM_214332.1|	lightcyan	287.5807	40.4153	0.981249	0.003074	228.042
gi|47523725|ref|NM_214333.1|	skyblue	465.348	22.9546	0.913831	0.029968	51.702
gi|47523737|ref|NM_214339.1|	violet	976.935	75.113	0.99894	4.14E-05	74.434
gi|47523743|ref|NM_214342.1|	orange	402.558	32.0815	0.97294	0.005322	13.848
gi|47523769|ref|NM_214356.1|	darkgrey	183.5637	34.9147	0.997355	0.000163	44.036
gi|47523779|ref|NM_214361.1|	lightyellow	1002.148	60.8655	0.991233	0.000984	117.71
gi|47523785|ref|NM_214364.1|	greenyellow	150.4091	44.9094	0.948375	0.013971	27.422
gi|47523789|ref|NM_214366.1|	turquoise	747.6399	231.048	0.92425	0.024741	68.288
gi|47523799|ref|NM_214372.1|	darkturquoise	184.6254	26.2453	0.923166	0.02527	60.614
gi|47523805|ref|NM_214375.1|	turquoise	448.8228	137.546	0.782901	0.117394	1293.842
gi|47523817|ref|NM_214381.1|	navy	95.75348	8.31924	0.281198	0.646743	61.996
gi|47523835|ref|NM_214391.1|	midnightblue	79.89097	5.46915	-0.41129	0.491489	11.664
gi|47523845|ref|NM_214396.1|	pink	77.95613	12.3926	0.434348	0.464889	858.34
gi|47523847|ref|NM_214397.1|	violet	928.9604	73.7223	0.99519	0.0004	39.714
gi|47523867|ref|NM_214407.1|	grey	341.1913	9.86436	0.355569	0.557004	80.326
gi|47523869|ref|NM_214408.1|	grey	152.1965	14.1479	-0.94649	0.01474	101.306
gi|47523875|ref|NM_214409.1|	purple	129.4139	20.7586	0.892519	0.041611	301.56
gi|47523887|ref|NM_214418.1|	sienna3	959.9743	26.018	0.999208	2.67E-05	36.814
gi|47523931|ref|NM_214440.1|	darkturquoise	103.8717	20.4706	0.868483	0.056111	2.952
gi|48374062|ref|NM_001001535.1|	turquoise	305.2454	98.5389	0.670555	0.215421	5919.602
gi|48374066|ref|NM_001001537.1|	darkmagenta	831.1143	23.5826	0.967062	0.00714	5.638
gi|48374070|ref|NM_001001539.1|	magenta	209.5977	67.8855	0.957265	0.010537	63.718
gi|48675942|ref|NM_001001640.1|	tan	944.921	62.5345	0.976691	0.004257	9.332
gi|48675944|ref|NM_001001637.1|	grey	157.7645	13.8768	-0.96656	0.007303	37.604
gi|48675952|ref|NM_001001643.1|	turquoise	440.7761	116.094	0.725275	0.165547	144.262
gi|48675956|ref|NM_001001645.1|	red	378.6933	95.1264	0.96655	0.007307	51.248
gi|48976064|ref|NM_001001649.2|	brown	159.9608	30.1572	0.611359	0.273249	107.638
gi|48976128|ref|NM_001001770.1|	navy	183.2969	31.6897	0.820908	0.088496	85.692
gi|49274614|ref|NM_001001860.1|	black	985.9291	94.9019	0.989799	0.001235	55.85
gi|49274638|ref|NM_001001861.1|	turquoise	685.929	226.136	0.898625	0.038152	0.81
gi|50979302|ref|NM_214301.1|	pink	208.8229	63.8846	0.970838	0.005952	111.372
gi|51491905|ref|NM_001003924.1|	navy	119.2558	39.5781	0.911479	0.031193	8.292
gi|51592110|ref|NM_001004031.1|	turquoise	894.5132	259.775	0.970251	0.006132	18.472
gi|51592138|ref|NM_001004045.1|	skyblue	232.1569	32.1944	0.995984	0.000305	11.934
gi|52350674|gb|AY609390.1|	green	81.03332	27.4774	0.700314	0.187835	15.188
gi|52350683|gb|AY609399.1|	turquoise	261.0683	76.144	0.603127	0.281577	27.512

gi|52350685|gb|AY609401.1|	pink	289.5833	55.9544	0.897461	0.038804	2.66
gi|52350686|gb|AY609402.1|	lightcyan	257.1036	38.6467	0.944599	0.015523	26.87
gi|52350700|gb|AY609415.1|	green	99.42512	57.8515	0.961978	0.008849	19.732
gi|52350704|gb|AY609419.1|	green	112.9031	33.9984	0.797776	0.105791	22.37
gi|52350710|gb|AY609425.1|	purple	213.4124	20.5281	-0.77384	0.12464	28.856
gi|52350723|gb|AY609438.1|	green	85.30429	35.2153	0.825687	0.085042	30.004
gi|52350726|gb|AY609441.1|	royalblue	323.8802	19.8599	0.852075	0.06676	23.206
gi|52350728|gb|AY609443.1|	tan	812.6684	59.6284	0.969801	0.006271	35.324
gi|52350742|gb|AY609457.1|	green	122.0051	36.8487	0.82165	0.087957	149.976
gi|52351065|gb|AY609470.1|	navy	198.077	28.7715	0.813323	0.094063	27.186
gi|52351066|gb|AY609471.1|	grey	170.469	8.28387	0.842363	0.073329	6.79
gi|52351067|gb|AY609472.1|	white	976.938	28.2722	0.763057	0.133422	19.818
gi|52351069|gb|AY609474.1|	blue	804.3281	232.745	0.969754	0.006286	11.98
gi|52351072|gb|AY609477.1|	red	521.4685	110.738	0.999778	3.97E-06	71.29
gi|52351075|gb|AY609480.1|	pink	115.0692	13.2352	0.166238	0.789318	7.35
gi|52351083|gb|AY609488.1|	turquoise	641.8348	212.031	0.874586	0.052301	35.044
gi|52351086|gb|AY609491.1|	lightcyan	301.4618	41.847	0.974498	0.00487	21.094
gi|52351090|gb|AY609495.1|	brown	114.5043	40.3069	0.853062	0.066104	181.732
gi|52351095|gb|AY609500.1|	blue	701.1368	181.818	0.91732	0.028182	29.096
gi|52351120|gb|AY609525.1|	turquoise	764.3968	247.251	0.936168	0.019173	34.18
gi|52351125|gb|AY609530.1|	turquoise	439.5552	149.161	0.768622	0.128866	7.676
gi|52351148|gb|AY609553.1|	blue	839.4977	234.485	0.974976	0.004734	60.854
gi|52351153|gb|AY609558.1|	blue	914.8499	226.167	0.96429	0.008057	15.972
gi|52351155|gb|AY609560.1|	midnightblue	153.1638	42.3842	0.978262	0.003835	35.028
gi|52351156|gb|AY609561.1|	violet	997.652	70.3596	0.985592	0.002072	23.57
gi|52351160|gb|AY609565.1|	lightcyan	295.1319	40.6736	0.975399	0.004615	6.174
gi|52351167|gb|AY609572.1|	red	560.588	105.907	0.990061	0.001188	121.252
gi|52351176|gb|AY609581.1|	blue	898.6532	244.574	0.983223	0.002602	6.368
gi|52351185|gb|AY609590.1|	turquoise	517.9156	136.693	0.780606	0.119216	9.38
gi|52351201|gb|AY609606.1|	darkorange	118.4629	16.6494	0.870609	0.054775	49.552
gi|52351209|gb|AY609612.1|	black	980.6067	97.019	0.994251	0.000523	66.864
gi|52351212|gb|AY609615.1|	turquoise	522.5709	124.943	0.753428	0.141415	18.864
gi|52351257|gb|AY609689.1|	orange	561.0542	29.1651	0.955172	0.011317	95.278
gi|52351277|gb|AY609709.1|	lightyellow	974.1837	62.8421	0.997795	0.000124	51.864
gi|52351285|gb|AY609717.1|	brown	229.7148	55.3596	0.841065	0.074221	1.54
gi|52351294|gb|AY609726.1|	red	530.315	100.536	0.978045	0.003892	4.632
gi|52351296|gb|AY609728.1|	royalblue	403.551	32.5075	0.995872	0.000318	41.658
gi|52351300|gb|AY609732.1|	royalblue	314.1197	32.3819	0.981144	0.003099	32.45
gi|52351303|gb|AY609735.1|	yellow	158.35	49.1714	0.785672	0.115205	10.288
gi|52351323|gb|AY609755.1|	blue	757.6878	211.964	0.944956	0.015374	60.616
gi|52351333|gb|AY609765.1|	red	427.6887	103.805	0.985589	0.002072	26.416
gi|52351341|gb|AY609773.1|	grey60	964.1523	62.0393	0.994114	0.000542	5.726
gi|52351346|gb|AY609778.1|	royalblue	281.2193	28.8346	0.939559	0.017675	45.312
gi|52351351|gb|AY609781.1|	turquoise	454.5411	151.711	0.822734	0.087171	15.122
gi|52351368|gb|AY609798.1|	navy	187.1434	31.8201	0.885342	0.045796	19.674
gi|52351373|gb|AY609803.1|	turquoise	619.5164	195.387	0.88507	0.045957	46.446
gi|52351374|gb|AY609804.1|	magenta	252.7255	77.3728	0.985499	0.002092	4.536
gi|52351378|gb|AY609808.1|	blue	947.3261	235.924	0.974001	0.005013	5.136

gi|52351391|gb|AY609821.1|	grey60	980.5193	62.8623	0.996857	0.000211	12.87
gi|52351393|gb|AY609823.1|	white	940.0734	29.7143	0.833737	0.079321	22.298
gi|52351397|gb|AY609827.1|	violet	991.7686	70.8928	0.987177	0.00174	10.968
gi|52351406|gb|AY609836.1|	brown	126.8407	41.9708	0.808185	0.097892	74.332
gi|52351416|gb|AY609846.1|	tan	938.2989	66.601	0.991284	0.000975	46.648
gi|52351419|gb|AY609849.1|	white	173.7589	21.6823	0.72586	0.165034	55.808
gi|52351430|gb|AY609860.1|	turquoise	382.7239	123.635	0.726726	0.164276	13.002
gi|52351432|gb|AY609862.1|	blue	794.2927	216.989	0.953114	0.012101	31.094
gi|52351435|gb|AY609865.1|	sienna3	934.0576	25.8051	0.997593	0.000142	6.686
gi|52351436|gb|AY609866.1|	turquoise	338.9508	84.3669	0.615755	0.268829	41.306
gi|52351444|gb|AY609874.1|	black	968.4899	99.2316	0.999362	1.94E-05	86.276
gi|52351447|gb|AY609877.1|	salmon	210.6232	39.7764	0.928578	0.022666	59.436
gi|52351453|gb|AY609883.1|	orange	301.8186	30.3861	0.966355	0.007371	3.552
gi|52351454|gb|AY609884.1|	turquoise	647.4077	210.37	0.879523	0.049281	33.576
gi|52351462|gb|AY609892.1|	darkorange	184.2006	19.0581	0.937888	0.018408	19.598
gi|52351474|gb|AY609904.1|	turquoise	564.8286	154.291	0.832144	0.080443	100.202
gi|52351476|gb|AY609906.1|	violet	967.6915	73.211	0.993804	0.000585	53.002
gi|52351490|gb|AY609920.1|	blue	949.7571	217.403	0.955	0.011382	22.088
gi|52351500|gb|AY609930.1|	salmon	141.7772	41.6094	0.945517	0.015141	15.566
gi|52351521|gb|AY609951.1|	green	132.2751	57.7431	0.980328	0.003302	82.722
gi|52351524|gb|AY609954.1|	pink	194.5623	56.8253	0.941329	0.016909	9.82
gi|52351536|gb|AY609966.1|	turquoise	678.7508	221.245	0.894723	0.040351	21.33
gi|52351538|gb|AY609968.1|	turquoise	917.9467	269.052	0.991162	0.000996	56.406
gi|52351540|gb|AY609970.1|	white	163.6132	8.03935	0.266344	0.664934	5.324
gi|52351544|gb|AY609974.1|	turquoise	332.3249	93.231	0.642966	0.241908	29.728
gi|52351547|gb|AY609977.1|	blue	791.0371	218.918	0.953318	0.012023	71.53
gi|52351567|gb|AY609997.1|	green	109.0297	53.6208	0.953497	0.011954	10.246
gi|52351573|gb|AY610003.1|	darkmagenta	973.0673	26.2455	0.988248	0.001527	11.498
gi|52351582|gb|AY610012.1|	darkgrey	116.9327	16.5322	0.807031	0.098759	21.16
gi|52351587|gb|AY610017.1|	blue	908.3072	241.937	0.978098	0.003878	114.726
gi|52351591|gb|AY610021.1|	black	982.2582	99.2161	0.99924	2.52E-05	16.258
gi|52351593|gb|AY610023.1|	darkgreen	229.7062	17.9894	0.768654	0.12884	14.972
gi|52351594|gb|AY610024.1|	brown	178.6161	57.2775	0.958787	0.009981	61.638
gi|52351609|gb|AY610039.1|	green	130.6324	20.1529	0.32079	0.598675	4.992
gi|52351611|gb|AY610041.1|	turquoise	629.1899	197.614	0.877843	0.050302	9.218
gi|52351614|gb|AY610044.1|	turquoise	873.3606	264.425	0.977083	0.00415	11.634
gi|52351621|gb|AY610051.1|	blue	743.4083	224.058	0.958823	0.009968	39.19
gi|52351639|gb|AY610069.1|	red	401.958	82.3605	0.93502	0.019689	28.168
gi|52351674|gb|AY610104.1|	sienna3	984.3348	26.0055	0.99911	3.19E-05	11.082
gi|52351676|gb|AY610106.1|	red	531.2851	105.368	0.9884	0.001497	31.924
gi|52351677|gb|AY610107.1|	black	948.953	87.0856	0.972772	0.005371	43.348
gi|52351697|gb|AY610127.1|	brown	109.0704	21.3505	0.597236	0.287576	785.492
gi|52351702|gb|AY610132.1|	turquoise	447.2925	138.869	0.794039	0.108672	38.752
gi|52351709|gb|AY610139.1|	darkorange	144.8549	7.53943	0.329738	0.587902	20.414
gi|52351717|gb|AY610147.1|	magenta	270.7519	78.8671	0.990779	0.001061	6.68
gi|52351733|gb|AY610163.1|	red	470.5565	77.574	0.922135	0.025775	9.598
gi|52351736|gb|AY610166.1|	lightcyan	179.4378	13.7719	0.563021	0.323056	313.622
gi|52351738|gb|AY610168.1|	navy	130.2316	7.24906	0.374911	0.53408	41.954

gi|52351742|gb|AY610172.1|	midnightblue	132.2426	43.4128	0.987055	0.001765	31.446
gi|52351745|gb|AY610175.1|	black	981.4256	98.1841	0.996997	0.000197	10.502
gi|52351750|gb|AY610180.1|	white	129.0673	12.7707	0.626035	0.258569	34.702
gi|52351755|gb|AY610185.1|	magenta	293.5023	52.487	0.899653	0.037579	0.968
gi|52351761|gb|AY610191.1|	black	963.5199	96.4195	0.99292	0.000714	27.32
gi|52351763|gb|AY610193.1|	orange	625.5681	24.4672	0.915192	0.029268	49.146
gi|52351796|gb|AY610214.1|	green	119.4571	42.5255	0.875517	0.051727	49.48
gi|52351805|gb|AY610223.1|	blue	708.9835	200.753	0.93589	0.019298	2.736
gi|52351807|gb|AY610225.1|	purple	50.74911	10.949	0.591164	0.293795	34.436
gi|52351871|gb|AY610289.1|	green	92.03027	55.7749	0.950528	0.013111	2.816
gi|52351873|gb|AY610291.1|	blue	862.5275	198.176	0.935493	0.019476	181.004
gi|52351877|gb|AY610295.1|	turquoise	503.0464	125.331	0.783839	0.116652	178.258
gi|52351890|gb|AY610308.1|	turquoise	648.3239	170.084	0.859276	0.062016	24.644
gi|52351923|gb|AY610341.1|	turquoise	647.1909	214.366	0.880569	0.048649	8.418
gi|52351935|gb|AY610353.1|	blue	820.8646	242.34	0.980084	0.003364	9.788
gi|52351947|gb|AY610365.1|	green	133.9092	34.9706	0.80534	0.100033	23.942
gi|52351956|gb|AY610374.1|	blue	861.6441	248.663	0.987533	0.001668	27.424
gi|52351961|gb|AY610379.1|	yellow	298.0811	104.712	0.964543	0.007972	17.838
gi|52351978|gb|AY610394.1|	turquoise	406.8118	137.591	0.786651	0.114435	153.256
gi|52351995|gb|AY610411.1|	royalblue	130.2185	9.01956	0.582128	0.303111	21.332
gi|52352038|gb|AY610454.1|	turquoise	158.0124	50.2191	0.494164	0.39745	30.528
gi|52352062|gb|AY610478.1|	grey	274.7195	12.542	0.992707	0.000747	14.088
gi|52352069|gb|AY610485.1|	grey	156.1662	14.53	-0.95273	0.012249	8.546
gi|52352072|gb|AY610488.1|	blue	669.7226	169.734	0.902492	0.036011	6.886
gi|52352090|gb|AY610506.1|	blue	756.0995	209.668	0.940565	0.017238	13.66
gi|52352705|gb|AY609625.1|	turquoise	381.011	122.633	0.739005	0.15364	56.284
gi|52352724|gb|AY609644.1|	blue	925.9085	242.424	0.979164	0.003599	24.546
gi|52352730|gb|AY609650.1|	violet	917.8991	73.7301	0.995181	0.000401	95.92
gi|54020965|ref|NM_001005726.1|	turquoise	471.1881	139.311	0.790908	0.111103	82.362
gi|54607194|ref|NM_001006592.1|	yellow	238.2823	72.0512	0.884101	0.046532	12599.26
gi|55741470|ref|NM_214042.1|	turquoise	861.2913	264.188	0.968801	0.006584	48.38
gi|55741485|ref|NM_213855.1|	midnightblue	88.17778	4.73638	-0.24754	0.688071	2031.626
gi|55741489|ref|NM_214136.1|	salmon	165.8115	36.9749	0.911996	0.030922	58.668
gi|55741720|ref|NM_001001534.1|	brown	90.92589	34.2818	0.745626	0.147991	770.902
gi|55741808|ref|NM_213748.1|	midnightblue	83.22974	5.70522	-0.22376	0.717493	3662.24
gi|55741810|ref|NM_001001863.1|	green	101.501	42.7516	0.853433	0.065857	5274.71
gi|55741820|ref|NM_214202.1|	brown	203.9259	59.6096	0.834823	0.078558	1.428
gi|55742741|ref|NM_213920.1|	grey	253.1393	11.6066	0.901979	0.036293	275.752
gi|55742771|ref|NM_001007191.1|	turquoise	636.6807	211.587	0.874687	0.052238	9.7
gi|55742846|ref|NM_214329.1|	blue	735.1089	211.628	0.951472	0.012739	82.828
gi|56606056|ref|NM_001008481.1|	yellow	272.2299	101.482	0.962721	0.008592	12.144
gi|56711365|ref|NM_001008688.1|	green	126.9009	35.7913	0.826323	0.084586	4821.764
gi|56711367|ref|NM_001008691.1|	brown	94.80425	37.6146	0.802724	0.102013	4.894
gi|56792875|gb|AY682217.1|	brown	75.63334	18.2997	0.627233	0.25738	0.648
gi|57527981|ref|NM_001009576.1|	darkgreen	202.648	29.0953	0.910964	0.031463	14.558
gi|57528034|ref|NM_001009582.1|	magenta	225.9154	68.1217	0.957723	0.010369	19.554
gi|58332861|ref|NM_001011507.1|	blue	603.605	116.205	0.817819	0.090751	29.492
gi|58801554|ref|NM_001011727.1|	salmon	188.7925	48.7723	0.980954	0.003146	63.732

gi|60302865|ref|NM_001012613.1|	grey60	866.8105	60.6001	0.989403	0.001307	2.966
gi|60419073|gb|AY856514.2|	lightcyan	261.6074	40.4872	0.974949	0.004742	22.112
gi|61098755|gb|AY820766.1|	pink	89.69367	6.89136	0.24391	0.692552	10.02
gi|61696637|gb|AY803100.1|	brown	238.2935	22.6882	0.629377	0.255256	2.708
gi|6650729|gb|AF116346.1|AF116346	turquoise	251.5603	71.8343	0.612817	0.271781	8.882
gi|68534989|ref|NM_001025224.1|	brown	72.33837	26.4543	0.474296	0.419582	198.302
gi|70778850|ref|NM_214094.2|	midnightblue	78.81873	29.987	0.906742	0.033705	1.866
gi|71834281|ref|NM_214197.2|	pink	287.4646	69.8608	0.962643	0.008619	103.242
gi|72535177|ref|NM_001031781.1|	midnightblue	158.2597	28.9589	0.870809	0.054649	878.962
gi|72535201|ref|NM_001031794.1|	white	203.5278	25.0264	0.746951	0.146868	142.75
gi|73853889|ref|NM_001032359.1|	greenyellow	147.2312	41.7788	0.929109	0.022415	699.608
gi|73853893|ref|NM_001032358.1|	turquoise	199.9127	43.6434	0.450455	0.446497	10.134
gi|74024908|ref|NM_001032379.1|	turquoise	896.8457	255.621	0.970243	0.006134	49.376
gi|74136750|ref|NM_001033009.1|	midnightblue	174.2116	40.6774	0.966828	0.007216	5.282
gi|74136760|ref|NM_001033014.1|	violet	877.2253	71.0546	0.98772	0.001631	121.53
gi|77628017|ref|NM_001008689.2|	magenta	198.3406	62.2726	0.937477	0.01859	116.54
gi|78364925|gb|DQ225117.1|	royalblue	259.8118	19.7144	0.875299	0.051861	6.666
gi|80971509|ref|NM_001037151.1|	greenyellow	173.6786	50.7966	0.98155	0.003	6.418
gi|80971515|ref|NM_001037152.1|	white	173.9272	21.6802	0.862825	0.059717	4.078
gi|82617535|ref|NM_001037322.1|	white	150.8865	6.80926	0.197085	0.750698	8.584
gi|83921642|ref|NM_001038007.1|	grey60	990.9169	61.828	0.993426	0.000639	77.798
gi|84579912|ref|NM_001038644.1|	turquoise	812.2483	258.373	0.957208	0.010558	18.478
gi|84619523|ref|NM_001038694.1|	darkorange	137.6109	8.23363	0.355875	0.55664	9.704
gi|84874691|ref|NM_001038632.1|	pink	86.11592	9.03056	0.204033	0.74203	14.638
gi|89574200|gb|DQ402993.1|	darkturquoise	99.14844	13.2797	0.773918	0.124574	1.772
gi|89886172|ref|NM_001039748.1|	navy	123.5747	9.23937	-0.1409	0.8212	66.054
gi|89886176|ref|NM_001039750.1|	turquoise	844.7661	248.785	0.953204	0.012066	4.342
gi|90017493|ref|NM_214184.2|	blue	919.3536	215.969	0.951896	0.012573	400.832
gi|92020093|dbj|AB237777.1|	blue	775.7696	161.369	0.888913	0.043698	13.04
gnl|UG|Ssc#S14768592	brown	214.351	61.0552	0.866342	0.057467	1.8
gnl|UG|Ssc#S14889148	lightgreen	291.8695	60.0714	0.995203	0.000398	2.244
gnl|UG|Ssc#S14891120	darkgreen	258.4531	36.9605	0.982405	0.002794	32.252
gnl|UG|Ssc#S14891216	lightcyan	244.6337	34.1659	0.927005	0.023413	1.102
gnl|UG|Ssc#S14894901	green	70.96983	39.4257	0.818472	0.090272	16.488
gnl|UG|Ssc#S14900031	turquoise	392.6413	110.501	0.695867	0.191889	9.504
gnl|UG|Ssc#S14900094	darkturquoise	108.6304	18.8112	0.851017	0.067466	4.102
gnl|UG|Ssc#S15981045	white	965.3194	28.9882	0.798266	0.105415	1.142
gnl|UG|Ssc#S16511729	blue	706.7269	195.333	0.927265	0.023289	148.854
gnl|UG|Ssc#S16513699	lightgreen	304.8673	58.6608	0.99036	0.001135	0.794
gnl|UG|Ssc#S16513921	brown	108.9817	32.7383	0.582276	0.302958	7.502
gnl|UG|Ssc#S16514435	darkgrey	220.0109	28.8843	0.951863	0.012586	6.792
gnl|UG|Ssc#S16514740	black	905.9652	92.551	0.984843	0.002235	6.756
gnl|UG|Ssc#S16514775	pink	254.53	72.4299	0.989005	0.001382	6.992
gnl|UG|Ssc#S16515033	salmon	182.0088	45.2416	0.961103	0.009155	21.842
gnl|UG|Ssc#S16515706	turquoise	480.1598	154.036	0.81546	0.092484	20.516
gnl|UG|Ssc#S16515765	sienna3	985.0201	26.0117	0.999135	3.05E-05	15.44
gnl|UG|Ssc#S16515841	turquoise	567.2304	187.804	0.886666	0.045014	3.242
gnl|UG|Ssc#S16516581	yellow	248.1625	87.2663	0.923508	0.025102	6.374

gnl|UG|Ssc#S16517530	violet	945.0526	74.9882	0.998607	6.24E-05	11.172
gnl|UG|Ssc#S16517601	darkred	253.0364	40.9917	0.976083	0.004424	3.376
gnl|UG|Ssc#S16517614	violet	989.0092	72.2569	0.991041	0.001016	6.154
gnl|UG|Ssc#S16517726	paleturquoise	150.751	18.0733	0.94053	0.017253	470.708
gnl|UG|Ssc#S16518321	navy	163.5991	43.8705	0.991204	0.000989	15.906
gnl|UG|Ssc#S16761746	salmon	152.4442	48.4179	0.980126	0.003353	54.88
gnl|UG|Ssc#S16761785	yellowgreen	165.9927	15.3662	0.955772	0.011091	38.11
gnl|UG|Ssc#S16761818	royalblue	343.2137	22.3096	0.880689	0.048576	6.876
gnl|UG|Ssc#S16763004	pink	76.6578	21.8282	0.555095	0.331423	15.158
gnl|UG|Ssc#S16763253	yellow	303.6539	115.543	0.989416	0.001305	2.17
gnl|UG|Ssc#S16763399	purple	239.6388	20.5362	-0.75683	0.138574	2.758
gnl|UG|Ssc#S16763478	royalblue	395.2996	32.5846	0.995911	0.000314	17.246
gnl|UG|Ssc#S16763868	salmon	157.5059	48.6472	0.980301	0.003309	29.784
gnl|UG|Ssc#S16764486	darkturquoise	232.5725	32.4608	0.980693	0.003211	4.272
gnl|UG|Ssc#S16767076	greenyellow	120.5923	22.2739	0.771672	0.126389	98.16
gnl|UG|Ssc#S16767694	yellow	285.4079	104.009	0.962358	0.008717	1.674
gnl|UG|Ssc#S16768026	yellowgreen	165.7777	16.0837	0.965961	0.0075	14.168
gnl|UG|Ssc#S16768494	darkred	193.3378	37.8924	0.944932	0.015384	1.908
gnl|UG|Ssc#S16769025	lightcyan	253.3487	38.4381	0.96561	0.007616	10.782
gnl|UG|Ssc#S16769591	turquoise	891.7551	260.479	0.969639	0.006322	4.428
gnl|UG|Ssc#S16769624	turquoise	871.3649	246.377	0.958356	0.010138	13.292
gnl|UG|Ssc#S16770187	purple	159.3725	18.151	-0.79271	0.109701	7.354
gnl|UG|Ssc#S16771004	darkgreen	263.9833	38.0084	0.98331	0.002582	2.972
gnl|UG|Ssc#S17499579	turquoise	488.3164	142.811	0.814999	0.092824	0.542
gnl|UG|Ssc#S17499681	red	485.9356	109.702	0.997756	0.000128	315.204
gnl|UG|Ssc#S17500017	turquoise	873.7224	262.415	0.979045	0.00363	16.782
gnl|UG|Ssc#S17501425	brown	228.1769	60.9154	0.901652	0.036473	9.668
gnl|UG|Ssc#S17503882	tan	958.5272	66.3499	0.989708	0.001251	12.804
gnl|UG|Ssc#S17504229	blue	819.2003	233.373	0.97325	0.005231	15.202
gnl|UG|Ssc#S17505316	darkturquoise	136.1423	22.5702	0.878048	0.050177	1.652
gnl|UG|Ssc#S17505417	white	143.6732	13.1723	0.688499	0.198659	12.038
gnl|UG|Ssc#S17505677	purple	136.053	10.8798	-0.5669	0.318985	1.45
gnl|UG|Ssc#S17505852	yellow	282.0685	100.353	0.953334	0.012016	1.608
gnl|UG|Ssc#S17508314	yellow	209.0617	73.3576	0.880739	0.048546	0.566
gnl|UG|Ssc#S17509886	brown	179.079	56.6003	0.975402	0.004614	517.992
gnl|UG|Ssc#S17510046	turquoise	430.4638	94.6007	0.680092	0.206465	6.76
gnl|UG|Ssc#S17510077	darkorange	200.6176	18.4416	0.971047	0.005888	99.302
gnl|UG|Ssc#S17510233	green	94.51446	50.6329	0.924465	0.024636	87.974
gnl|UG|Ssc#S17510311	turquoise	271.2002	62.4344	0.567573	0.318275	7.972
gnl|UG|Ssc#S17510336	tan	908.6874	67.9846	0.995788	0.000328	17.336
gnl|UG|Ssc#S17510396	turquoise	418.3314	135.043	0.750217	0.144111	59.464
gnl|UG|Ssc#S17510398	grey	159.1383	13.23	0.319614	0.600094	256.824
gnl|UG|Ssc#S17510452	blue	967.4455	236.138	0.974986	0.004731	21.216
gnl|UG|Ssc#S17510525	green	165.5002	22.5211	0.414987	0.487206	75.978
gnl|UG|Ssc#S17510796	magenta	248.9736	77.1984	0.985123	0.002173	5.668
gnl|UG|Ssc#S17510917	lightcyan	229.3552	36.0917	0.937046	0.018781	4.184
gnl|UG|Ssc#S17510986	turquoise	885.4196	255.875	0.965514	0.007648	200.132
gnl|UG|Ssc#S17511033	orange	562.3572	32.5652	0.97801	0.003902	27.218

gnl|UG|Ssc#S17511146	tan	962.3049	59.6627	0.966572	0.0073	24.24
gnl|UG|Ssc#S17511222	pink	176.099	28.6982	0.759107	0.136684	2.49
gnl|UG|Ssc#S17511240	black	961.7817	90.1936	0.980198	0.003335	97.84
gnl|UG|Ssc#S17511285	brown	98.31572	36.6734	0.669445	0.21647	449.332
gnl|UG|Ssc#S17511386	turquoise	901.1112	271.136	0.985745	0.002039	532.772
gnl|UG|Ssc#S17511419	blue	858.4414	232.638	0.970282	0.006122	35.118
gnl|UG|Ssc#S17511723	pink	114.3998	11.446	-0.08419	0.892937	20.754
gnl|UG|Ssc#S17511887	magenta	277.6177	54.5217	0.909762	0.032096	7.496
gnl|UG|Ssc#S17512005	turquoise	792.5054	251.593	0.954805	0.011455	28.704
gnl|UG|Ssc#S17512032	turquoise	168.0091	38.2944	0.440361	0.458003	1.128
gnl|UG|Ssc#S17512154	green	83.22289	47.219	0.894004	0.040761	148.07
gnl|UG|Ssc#S17512186	turquoise	560.3517	181.911	0.867271	0.056878	21.872
gnl|UG|Ssc#S17512237	magenta	257.8846	75.7657	0.981177	0.003091	2.776
gnl|UG|Ssc#S17512269	yellow	139.9459	42.1924	0.748076	0.145917	54.436
gnl|UG|Ssc#S17512288	brown	67.19938	24.9255	0.673917	0.212252	3.21
gnl|UG|Ssc#S17513253	black	976.0026	88.6989	0.97641	0.004334	22.202
gnl|UG|Ssc#S17513449	blue	828.6787	170.589	0.903751	0.035323	23.518
gnl|UG|Ssc#S17513694	red	479.4392	109.246	0.996839	0.000213	9.21
gnl|UG|Ssc#S17513715	blue	843.0476	234.773	0.973596	0.00513	35.384
gnl|UG|Ssc#S17513789	turquoise	219.1361	55.4605	0.506428	0.38393	8.346
gnl|UG|Ssc#S17513958	turquoise	755.7763	234.611	0.92167	0.026005	17.208
gnl|UG|Ssc#S17513979	green	112.3749	55.9435	0.959789	0.009621	165.39
gnl|UG|Ssc#S17514069	red	397.9348	99.0413	0.975632	0.00455	9.722
gnl|UG|Ssc#S17514190	lightyellow	949.85	63.239	0.999049	3.52E-05	2.284
gnl|UG|Ssc#S17514318	salmon	186.4834	37.3202	0.910956	0.031467	27.132
gnl|UG|Ssc#S17514565	green	145.975	50.1288	0.947805	0.014202	22.052
gnl|UG|Ssc#S17514632	darkgreen	197.5766	29.904	0.905784	0.03422	634.34
gnl|UG|Ssc#S17514667	lightcyan	295.352	42.1253	0.985118	0.002174	14.014
gnl|UG|Ssc#S17514838	blue	924.7744	253.403	0.989526	0.001285	21.96
gnl|UG|Ssc#S17515039	brown	126.942	31.1074	0.651553	0.233573	4.496
gnl|UG|Ssc#S17515108	turquoise	730.1049	206.624	0.926452	0.023678	22.876
gnl|UG|Ssc#S17515316	yellow	213.9927	75.8848	0.88798	0.044243	24.224
gnl|UG|Ssc#S17515611	purple	182.4553	16.5371	-0.91882	0.027427	34.506
gnl|UG|Ssc#S17515701	black	892.264	94.0158	0.988519	0.001474	27.612
gnl|UG|Ssc#S17515754	violet	945.399	74.7223	0.997888	0.000116	17.406
gnl|UG|Ssc#S17515773	black	977.7564	91.4766	0.982089	0.00287	50.314
gnl|UG|Ssc#S17515809	brown	220.5013	42.0687	0.732394	0.159341	20.362
gnl|UG|Ssc#S17515842	yellow	213.6047	70.5003	0.869604	0.055405	14.254
gnl|UG|Ssc#S17516048	blue	759.0311	198.811	0.930107	0.021947	38.456
gnl|UG|Ssc#S17516064	darkred	258.6464	43.2381	0.988407	0.001496	10.132
gnl|UG|Ssc#S17516079	royalblue	309.6366	30.0984	0.973852	0.005056	11.566
gnl|UG|Ssc#S17516095	magenta	223.923	71.2545	0.968919	0.006547	17.684
gnl|UG|Ssc#S17516148	blue	847.778	213.169	0.950724	0.013033	26.29
gnl|UG|Ssc#S17516311	turquoise	700.1707	226.636	0.931639	0.021234	500.688
gnl|UG|Ssc#S17516549	blue	963.434	216.396	0.956192	0.010934	27.584
gnl|UG|Ssc#S17516828	midnightblue	83.21511	5.51948	-0.44531	0.452351	50.244
gnl|UG|Ssc#S17516920	lightcyan	274.1938	40.1626	0.964714	0.007915	31.482
gnl|UG|Ssc#S17516962	midnightblue	88.40938	31.6559	0.907886	0.033093	7.998

gnl|UG|Ssc#S17517004	turquoise	895.2715	268.518	0.981749	0.002952	13.14
gnl|UG|Ssc#S17517072	salmon	161.1993	42.9906	0.949597	0.013481	2.93
gnl|UG|Ssc#S17517096	purple	144.0793	22.3529	0.862648	0.059832	28.144
gnl|UG|Ssc#S17517121	orange	504.5468	34.1176	0.990385	0.00113	3.518
gnl|UG|Ssc#S17517147	darkgreen	261.1594	37.8341	0.990309	0.001143	4.394
gnl|UG|Ssc#S17517405	skyblue	154.9525	24.3148	0.937373	0.018636	17.88
gnl|UG|Ssc#S17517774	green	89.07704	42.7698	0.860684	0.0611	8.962
gnl|UG|Ssc#S17517964	turquoise	824.9533	239.124	0.946015	0.014935	23.752
gnl|UG|Ssc#S17518104	midnightblue	215.1056	33.8213	0.919402	0.027133	10.914
gnl|UG|Ssc#S17518111	turquoise	634.1944	210.214	0.872009	0.053899	2.286
gnl|UG|Ssc#S17518115	pink	198.8618	57.9148	0.944786	0.015445	28.994
gnl|UG|Ssc#S17518180	paleturquoise	172.0545	19.5278	0.949487	0.013524	29.24
gnl|UG|Ssc#S17518305	yellow	242.6386	85.0114	0.914722	0.029509	16.35
gnl|UG|Ssc#S17518307	turquoise	774.405	241.781	0.937881	0.018411	21.13
gnl|UG|Ssc#S17518363	turquoise	863.5923	258.424	0.965166	0.007763	28.978
gnl|UG|Ssc#S17518385	purple	113.1459	22.9092	0.902886	0.035795	5.498
gnl|UG|Ssc#S17518565	turquoise	738.7591	226.186	0.924236	0.024747	30.936
gnl|UG|Ssc#S17518664	sienna3	976.2211	25.8601	0.998007	0.000107	19.346
gnl|UG|Ssc#S17518706	green	116.3701	49.4294	0.928969	0.022481	15.24
gnl|UG|Ssc#S17518758	green	135.8439	48.7697	0.928039	0.022921	14.008
gnl|UG|Ssc#S17518763	turquoise	190.0961	63.0225	0.573952	0.311606	6.282
gnl|UG|Ssc#S17518772	blue	878.1758	249.626	0.986573	0.001864	28.812
gnl|UG|Ssc#S17518773	paleturquoise	122.0311	13.507	0.860243	0.061386	10.936
gnl|UG|Ssc#S17518795	grey	165.2936	13.381	-0.90492	0.034689	31.744
gnl|UG|Ssc#S17518997	black	977.3934	98.9456	0.998613	6.20E-05	29.604
gnl|UG|Ssc#S17519017	white	198.975	22.5095	0.834268	0.078948	10.946
gnl|UG|Ssc#S17524473	blue	778.0336	222.284	0.955564	0.011169	55.668
gnl|UG|Ssc#S17524578	greenyellow	244.3259	40.1697	0.911089	0.031397	26.42
gnl|UG|Ssc#S17524627	blue	929.2595	239.206	0.978829	0.003686	28.868
gnl|UG|Ssc#S17524633	yellowgreen	184.2265	18.2838	0.990731	0.00107	124.658
gnl|UG|Ssc#S17524752	navy	188.6895	26.883	0.752973	0.141796	33.116
gnl|UG|Ssc#S17524900	paleturquoise	159.0919	15.0504	0.885546	0.045675	23.48
gnl|UG|Ssc#S17524908	blue	622.5608	188.431	0.92213	0.025778	3.35
gnl|UG|Ssc#S17524953	navy	149.5577	44.5098	0.979898	0.003411	39.116
gnl|UG|Ssc#S17524981	turquoise	466.0582	123.647	0.732913	0.158891	27.14
gnl|UG|Ssc#S17524990	turquoise	324.3387	76.7705	0.620903	0.263678	14.026
gnl|UG|Ssc#S17525104	grey	101.4048	7.08812	0.140863	0.821242	16.532
gnl|UG|Ssc#S17525203	darkgreen	279.3347	34.9576	0.976662	0.004265	8.568
gnl|UG|Ssc#S17525225	violet	870.5102	71.7755	0.989679	0.001257	4.08
gnl|UG|Ssc#S17525376	navy	111.3646	33.5119	0.854273	0.0653	26.292
gnl|UG|Ssc#S17525378	purple	149.1272	13.7305	-0.56072	0.32548	11.668
gnl|UG|Ssc#S17525409	grey	273.7596	12.9673	0.969501	0.006364	7.146
gnl|UG|Ssc#S17525410	darkgrey	120.3033	17.6709	0.821991	0.08771	11.63
gnl|UG|Ssc#S17525682	lightyellow	972.4211	62.8442	0.997791	0.000125	213.61
gnl|UG|Ssc#S17525753	yellow	286.695	106.488	0.970109	0.006176	7.23
gnl|UG|Ssc#S17525826	greenyellow	175.6979	47.5829	0.961316	0.00908	10.284
gnl|UG|Ssc#S17525865	greenyellow	177.8976	48.3594	0.967344	0.007049	15.478
gnl|UG|Ssc#S17526139	turquoise	682.8407	191.463	0.895287	0.040031	23.1

gnl|UG|Ssc#S17526150	grey	279.0844	12.7283	0.949085	0.013685	9.556
gnl|UG|Ssc#S17526193	salmon	307.0942	41.8703	0.937624	0.018525	63.318
gnl|UG|Ssc#S17526232	turquoise	219.3327	59.061	0.55515	0.331365	10.164
gnl|UG|Ssc#S17526234	blue	906.7952	229.406	0.9664	0.007356	22.37
gnl|UG|Ssc#S17526265	yellow	223.8139	67.4677	0.873622	0.052897	3.878
gnl|UG|Ssc#S17526422	brown	151.837	36.4261	0.70759	0.181256	18.494
gnl|UG|Ssc#S17526634	red	447.5266	98.066	0.973202	0.005245	18.298
gnl|UG|Ssc#S17526834	skyblue	107.5979	17.629	0.862718	0.059787	26.508
gnl|UG|Ssc#S17526835	grey60	986.8885	62.7034	0.996352	0.000264	5.414
gnl|UG|Ssc#S17526874	blue	830.5002	201.55	0.939854	0.017547	193.284
gnl|UG|Ssc#S17526922	magenta	264.2728	79.3695	0.991338	0.000967	13.762
gnl|UG|Ssc#S17526927	blue	945.6076	209.049	0.94733	0.014395	8.724
gnl|UG|Ssc#S17526940	red	486.2478	97.5362	0.971671	0.005699	15.994
gnl|UG|Ssc#S17526987	magenta	245.2676	77.1516	0.985147	0.002168	23.61
gnl|UG|Ssc#S17527330	turquoise	449.8628	144.716	0.802003	0.102561	104.698
gnl|UG|Ssc#S17527420	red	554.6491	91.8124	0.957564	0.010427	68.984
gnl|UG|Ssc#S17527438	turquoise	799.0809	247.791	0.963291	0.008396	36.56
gnl|UG|Ssc#S17527454	magenta	255.9152	78.5702	0.988935	0.001395	12.678
gnl|UG|Ssc#S17527505	darkmagenta	933.8661	26.4886	0.990017	0.001196	10.248
gnl|UG|Ssc#S18260225	blue	705.7677	211.754	0.949567	0.013492	26.278
gnl|UG|Ssc#S18261002	turquoise	911.8005	269.398	0.981831	0.002932	41.806
gnl|UG|Ssc#S18262172	grey60	932.4881	62.1602	0.99457	0.00048	16.424
gnl|UG|Ssc#S18262357	darkgreen	210.5403	32.2704	0.940868	0.017107	8.432
gnl|UG|Ssc#S18263440	black	885.9208	92.6667	0.985132	0.002171	16.164
gnl|UG|Ssc#S18263529	greenyellow	207.2076	54.5935	0.994006	0.000557	266.718
gnl|UG|Ssc#S18263775	skyblue	297.4738	27.9231	0.956479	0.010827	29.658
gnl|UG|Ssc#S18263925	brown	216.1531	61.6376	0.924471	0.024633	2.372
gnl|UG|Ssc#S18266615	black	936.5918	92.1788	0.98386	0.002455	9.164
gnl|UG|Ssc#S18267174	navy	185.7059	8.51173	0.535611	0.352217	19.428
gnl|UG|Ssc#S18267480	blue	802.1522	179.383	0.914983	0.029375	1.384
gnl|UG|Ssc#S18267647	darkmagenta	983.1549	26.085	0.986553	0.001868	20.47
gnl|UG|Ssc#S18268726	grey	168.4623	13.4791	-0.95805	0.010248	9.148
gnl|UG|Ssc#S18268814	green	148.9794	20.117	0.203809	0.74231	18.398
gnl|UG|Ssc#S18269890	darkturquoise	203.1586	32.2792	0.974238	0.004944	0.866
gnl|UG|Ssc#S18270450	darkred	275.0257	43.1431	0.989947	0.001208	4.9
gnl|UG|Ssc#S18270499	violet	929.4696	74.6499	0.997668	0.000135	0.272
gnl|UG|Ssc#S18272639	darkorange	117.7784	17.3645	0.912277	0.030776	8.71
gnl|UG|Ssc#S18272798	navy	262.5663	26.9346	0.813194	0.094159	8.298
gnl|UG|Ssc#S18272948	white	972.5178	29.1546	0.778184	0.121148	11.62
gnl|UG|Ssc#S18274718	darkmagenta	939.3157	26.955	0.993726	0.000596	8.774
gnl|UG|Ssc#S18276256	violet	784.3139	66.2885	0.973546	0.005144	19.36
gnl|UG|Ssc#S18277539	pink	90.5189	15.0714	0.17445	0.779015	68.136
gnl|UG|Ssc#S18277825	white	166.5006	20.1833	0.748839	0.145272	3.54
gnl|UG|Ssc#S18281860	brown	51.52596	14.0055	0.362183	0.549145	24.806
gnl|UG|Ssc#S18282381	brown	134.1926	41.8511	0.847289	0.069973	56.394
gnl|UG|Ssc#S18283093	tan	937.3036	68.1633	0.996151	0.000286	22.744
gnl|UG|Ssc#S18283235	blue	934.2018	208.831	0.947544	0.014308	14.814
gnl|UG|Ssc#S18284222	white	806.8139	27.1452	0.847271	0.069986	4.28

gnl|UG|Ssc#S18284670	yellow	296.3186	109.752	0.976039	0.004436	8.428
gnl|UG|Ssc#S18289792	turquoise	324.9281	75.3573	0.59992	0.284838	11.72
gnl|UG|Ssc#S18290449	red	409.0446	101.359	0.980497	0.00326	427.76
gnl|UG|Ssc#S18291801	lightyellow	922.0185	62.6383	0.997122	0.000185	10.42
gnl|UG|Ssc#S18292679	pink	277.9826	66.8383	0.957372	0.010497	6.562
gnl|UG|Ssc#S18292759	red	487.2135	98.5013	0.973584	0.005133	16.992
gnl|UG|Ssc#S18295197	yellow	303.5973	117.116	0.993894	0.000572	1.504
gnl|UG|Ssc#S18301441	yellow	291.2468	110.281	0.977482	0.004043	4.34
gnl|UG|Ssc#S18301820	grey	282.9569	12.8693	0.992555	0.00077	5.328
gnl|UG|Ssc#S18304172	blue	871.7699	251.877	0.988024	0.00157	8.812
gnl|UG|Ssc#S18333765	purple	260.4747	20.2436	-0.74686	0.146943	1.138
gnl|UG|Ssc#S18336060	darkgrey	68.1998	7.8918	0.635024	0.249686	0.67
gnl|UG|Ssc#S18336254	yellow	298.8297	115.885	0.992554	0.00077	1.42
gnl|UG|Ssc#S18336569	turquoise	736.866	231.879	0.943183	0.016118	11.206
gnl|UG|Ssc#S18354030	darkred	187.821	9.59585	0.616823	0.267758	4.18
gnl|UG|Ssc#S18354494	navy	128.1274	8.25411	0.27518	0.654104	56.628
gnl|UG|Ssc#S18354763	turquoise	896.1343	266.965	0.976668	0.004263	21.746
gnl|UG|Ssc#S18354874	brown	197.9743	50.5028	0.835617	0.078002	5.712
gnl|UG|Ssc#S18355787	purple	84.744	14.5713	0.657287	0.228053	23.736
gnl|UG|Ssc#S18355850	turquoise	669.6793	210.884	0.916027	0.02884	6.502
gnl|UG|Ssc#S18355951	blue	827.9331	223.094	0.956291	0.010897	24.92
gnl|UG|Ssc#S18355973	turquoise	583.1352	132.649	0.788175	0.113239	31.996
gnl|UG|Ssc#S18355999	turquoise	893.9754	262.763	0.982153	0.002854	4.446
gnl|UG|Ssc#S18356219	turquoise	351.0624	116.446	0.697459	0.190435	23.11
gnl|UG|Ssc#S18356676	greenyellow	169.762	45.7002	0.952389	0.012382	28.772
gnl|UG|Ssc#S18357153	violet	929.4696	74.6499	0.997668	0.000135	0.498
gnl|UG|Ssc#S18357219	grey60	886.565	60.0572	0.98754	0.001666	45.718
gnl|UG|Ssc#S18357288	turquoise	383.4067	103.256	0.696676	0.19115	12.592
gnl|UG|Ssc#S18357398	turquoise	369.8538	84.1206	0.62653	0.258077	23.834
gnl|UG|Ssc#S18357511	red	564.9343	103.625	0.985555	0.00208	34.46
gnl|UG|Ssc#S18357580	blue	722.027	213.924	0.950665	0.013057	21.964
gnl|UG|Ssc#S18357620	green	86.05447	38.3378	0.821151	0.088319	630.332
gnl|UG|Ssc#S18357751	turquoise	764.4816	244.047	0.931063	0.021502	14.624
gnl|UG|Ssc#S18357775	darkgrey	224.7232	27.7927	0.944331	0.015635	7.234
gnl|UG|Ssc#S18358012	yellow	303.1603	110.371	0.97775	0.003971	4.34
gnl|UG|Ssc#S18358064	turquoise	417.7681	139.662	0.780699	0.119142	35.672
gnl|UG|Ssc#S18358279	red	401.8535	93.7076	0.962638	0.00862	62.898
gnl|UG|Ssc#S18358567	skyblue	220.3673	31.5166	0.989849	0.001226	12.35
gnl|UG|Ssc#S18358587	lightgreen	302.3543	57.7872	0.987286	0.001718	13.642
gnl|UG|Ssc#S18358692	turquoise	518.8062	172.6	0.817811	0.090756	9.176
gnl|UG|Ssc#S18358900	violet	980.1531	74.6742	0.997748	0.000128	6.744
gnl|UG|Ssc#S18358928	black	991.6817	95.3336	0.990593	0.001094	15.158
gnl|UG|Ssc#S18359073	blue	963.1138	240.3	0.979298	0.003564	16.804
gnl|UG|Ssc#S18359101	darkturquoise	199.308	25.3616	0.917153	0.028267	8.362
gnl|UG|Ssc#S18359197	red	563.8283	99.4294	0.975781	0.004508	6.744
gnl|UG|Ssc#S18359204	lightyellow	950.6844	63.2086	0.998954	4.06E-05	3.224
gnl|UG|Ssc#S18359209	paleturquoise	155.291	11.1314	0.841414	0.073981	10.538
gnl|UG|Ssc#S18359264	brown	91.88646	35.2121	0.793165	0.109349	98.918

gnl|UG|Ssc#S18359557	lightcyan	311.7514	41.4111	0.966128	0.007445	15.474
gnl|UG|Ssc#S18359743	lightyellow	946.9477	62.8661	0.997881	0.000117	30.572
gnl|UG|Ssc#S18359767	darkgrey	193.3329	32.3187	0.978401	0.003798	15.848
gnl|UG|Ssc#S18359803	salmon	135.9792	40.5518	0.938443	0.018164	12.93
gnl|UG|Ssc#S18360059	turquoise	396.6564	122.202	0.731784	0.15987	12.222
gnl|UG|Ssc#S18360480	darkgreen	280.1374	38.1764	0.990444	0.00112	1.124
gnl|UG|Ssc#S18360885	turquoise	260.4145	60.6222	0.496878	0.394448	24.512
gnl|UG|Ssc#S18361423	turquoise	880.2748	266.279	0.974211	0.004952	4.634
gnl|UG|Ssc#S18361485	turquoise	297.441	99.0613	0.673389	0.212749	3.126
gnl|UG|Ssc#S18377744	darkred	275.6615	41.1021	0.976908	0.004198	2.502
gnl|UG|Ssc#S18377876	red	459.8331	106.64	0.991624	0.000919	8.6
gnl|UG|Ssc#S18378149	white	893.2035	28.6296	0.806632	0.09906	7.992
gnl|UG|Ssc#S18378291	black	807.5662	87.6834	0.974061	0.004995	5.108
gnl|UG|Ssc#S18378422	navy	163.7423	37.6232	0.976959	0.004184	20.408
gnl|UG|Ssc#S18378552	turquoise	754.6259	222.147	0.947347	0.014388	39.192
gnl|UG|Ssc#S18378606	blue	732.271	215.832	0.947918	0.014156	49.004
gnl|UG|Ssc#S18378680	green	107.994	47.6875	0.910667	0.031619	40.67
gnl|UG|Ssc#S18378791	greenyellow	142.6443	42.5614	0.935458	0.019492	42.8
gnl|UG|Ssc#S18378792	blue	893.6351	214.278	0.951964	0.012547	15.496
gnl|UG|Ssc#S18378854	pink	221.6615	59.2509	0.931333	0.021376	23.87
gnl|UG|Ssc#S18378957	grey	162.8082	12.8034	-0.92922	0.022363	10.206
gnl|UG|Ssc#S18378982	navy	194.0409	33.3066	0.850321	0.067932	3.902
gnl|UG|Ssc#S18378997	grey60	799.3383	57.0125	0.976964	0.004183	12.886
gnl|UG|Ssc#S18379057	yellowgreen	137.3702	11.7671	0.890422	0.04282	36.324
gnl|UG|Ssc#S18379095	lightyellow	984.9663	62.4852	0.996581	0.00024	7.77
gnl|UG|Ssc#S18379108	yellow	309.3732	118.735	0.997001	0.000197	24.764
gnl|UG|Ssc#S18379116	grey	162.6458	14.3056	-0.98248	0.002777	50.834
gnl|UG|Ssc#S18379252	darkgrey	183.2305	34.8645	0.997113	0.000186	18.426
gnl|UG|Ssc#S18379332	navy	154.8084	38.3006	0.968705	0.006615	27.31
gnl|UG|Ssc#S18379352	blue	763.4744	229.029	0.966319	0.007383	0.666
gnl|UG|Ssc#S18379413	purple	142.1595	8.95021	0.631173	0.253481	11.874
gnl|UG|Ssc#S18379594	brown	197.3827	57.0179	0.80107	0.103271	5.296
gnl|UG|Ssc#S18379751	blue	438.9467	124.776	0.827283	0.083898	7.968
gnl|UG|Ssc#S18379761	turquoise	452.4503	130.735	0.774293	0.124271	3.1
gnl|UG|Ssc#S18380107	orange	572.589	32.9568	0.98225	0.002831	91.478
gnl|UG|Ssc#S18380485	red	657.9769	102.813	0.983227	0.002601	23.76
gnl|UG|Ssc#S18380508	grey60	950.6862	63.4941	0.998899	4.39E-05	2.378
gnl|UG|Ssc#S18380537	turquoise	559.3768	167.475	0.846234	0.070688	34.314
gnl|UG|Ssc#S18380653	pink	320.3631	43.6335	0.825981	0.084831	28.76
gnl|UG|Ssc#S18380710	tan	937.0602	67.9092	0.995936	0.000311	26.54
gnl|UG|Ssc#S18380942	darkgreen	286.7867	35.7481	0.975384	0.004619	8.632
gnl|UG|Ssc#S18381258	darkgrey	191.7128	34.3454	0.992336	0.000804	18.348
gnl|UG|Ssc#S18381264	salmon	275.384	34.7837	0.894185	0.040658	0.654
gnl|UG|Ssc#S18381418	darkgreen	242.8586	18.7037	0.780961	0.118934	2.068
gnl|UG|Ssc#S18381432	turquoise	141.1409	36.6245	0.462705	0.432621	21.414
gnl|UG|Ssc#S18381627	lightgreen	283.8522	60.4054	0.99641	0.000258	0.102
gnl|UG|Ssc#S18382551	red	426.653	77.6273	0.924527	0.024606	14.42
gnl|UG|Ssc#S18382627	turquoise	829.793	251.693	0.952816	0.012216	13.994

gnl|UG|Ssc#S18382640	sienna3	958.9575	25.975	0.998888	4.45E-05	18.52
gnl|UG|Ssc#S18383248	turquoise	461.2249	125.909	0.750051	0.14425	108.706
gnl|UG|Ssc#S18383312	turquoise	512.1469	153.493	0.802685	0.102043	11.928
gnl|UG|Ssc#S18383420	violet	988.3523	74.7533	0.997947	0.000112	37.492
gnl|UG|Ssc#S18383442	midnightblue	182.2926	43.3666	0.980213	0.003331	23.56
gnl|UG|Ssc#S18383451	pink	94.31265	13.8325	0.13351	0.830516	24.168
gnl|UG|Ssc#S18383491	black	843.1529	88.5942	0.976576	0.004288	31.506
gnl|UG|Ssc#S18383636	violet	905.9396	73.5922	0.994767	0.000454	2.684
gnl|UG|Ssc#S18383637	red	605.8954	103.143	0.983653	0.002503	29.272
gnl|UG|Ssc#S18383839	darkgreen	198.4645	30.0007	0.917753	0.027963	12.324
gnl|UG|Ssc#S18383932	turquoise	822.1569	260.295	0.960858	0.009241	103.854
gnl|UG|Ssc#S18384163	lightgreen	291.4019	57.6429	0.986901	0.001796	7.628
gnl|UG|Ssc#S18384305	lightcyan	288.2271	39.7599	0.977534	0.004029	14.738
gnl|UG|Ssc#S18384469	blue	650.8482	195.988	0.931557	0.021273	8.024
gnl|UG|Ssc#S18384885	midnightblue	117.911	37.4009	0.940452	0.017287	18.288
gnl|UG|Ssc#S18385915	darkred	265.4921	43.0857	0.987415	0.001692	0.558
gnl|UG|Ssc#S18386342	darkmagenta	987.1657	27.1977	0.995337	0.000382	1.27
gnl|UG|Ssc#S18386541	purple	65.01541	10.7553	0.569637	0.316114	24.638
gnl|UG|Ssc#S18386569	lightyellow	867.1263	60.4077	0.989797	0.001235	1.81
gnl|UG|Ssc#S18387078	red	601.1383	107.319	0.992532	0.000774	31.366
gnl|UG|Ssc#S18387120	green	135.9789	54.5121	0.96551	0.007649	14.236
gnl|UG|Ssc#S18387223	blue	864.6217	242.641	0.980152	0.003347	24.646
gnl|UG|Ssc#S18387292	turquoise	833.421	232.136	0.941727	0.016738	23.152
gnl|UG|Ssc#S18387383	royalblue	364.8971	33.1158	0.996384	0.000261	13.636
gnl|UG|Ssc#S18387501	royalblue	289.8343	19.0302	0.838545	0.075963	21.244
gnl|UG|Ssc#S18387558	black	993.3782	94.7125	0.989281	0.00133	24.012
gnl|UG|Ssc#S18387565	lightyellow	898.9128	61.1934	0.99246	0.000785	3.808
gnl|UG|Ssc#S18387589	darkturquoise	177.0703	24.505	0.90903	0.032484	21.444
gnl|UG|Ssc#S18387708	green	98.59889	44.6785	0.880107	0.048928	69.906
gnl|UG|Ssc#S18387783	yellow	152.9945	40.3464	0.756223	0.139081	36.756
gnl|UG|Ssc#S18546005	turquoise	770.4959	238.006	0.942292	0.016496	46.706
gnl|UG|Ssc#S18546038	darkgrey	94.07013	10.7004	0.729197	0.162119	11.32
gnl|UG|Ssc#S18546074	red	750.6959	79.3313	0.925022	0.024366	18.394
gnl|UG|Ssc#S18546081	paleturquoise	134.0411	13.5711	0.863767	0.059112	16.196
gnl|UG|Ssc#S18546149	turquoise	469.2935	155.45	0.790903	0.111107	64.536
gnl|UG|Ssc#S18546387	lightgreen	287.6626	55.1334	0.977934	0.003922	13.354
gnl|UG|Ssc#S18546390	skyblue	293.8636	30.5254	0.980927	0.003153	150.048
gnl|UG|Ssc#S18546426	yellow	238.9237	87.3779	0.920423	0.026623	26.734
gnl|UG|Ssc#S18546477	navy	114.6704	8.94061	-0.12111	0.846181	53.422
gnl|UG|Ssc#S18546502	turquoise	328.4631	83.1585	0.597318	0.287492	7.594
gnl|UG|Ssc#S18546597	yellow	288.3365	110.262	0.977639	0.004	1.68
gnl|UG|Ssc#S18546698	magenta	243.345	78.8523	0.990428	0.001123	2.286
gnl|UG|Ssc#S18546855	green	116.9576	61.5978	0.994295	0.000517	19.572
gnl|UG|Ssc#S18546948	turquoise	598.8568	181.26	0.871057	0.054494	5.096
gnl|UG|Ssc#S18546984	black	944.0516	97.9442	0.996992	0.000198	3.692
gnl|UG|Ssc#S18547064	sienna3	819.1681	23.5621	0.979497	0.003513	6.578
gnl|UG|Ssc#S18547312	purple	197.4655	10.9576	0.626092	0.258512	15.336
gnl|UG|Ssc#S18547360	red	517.9412	100.679	0.978627	0.003739	15.772

gnl|UG|Ssc#S18547454	violet	949.7404	74.7797	0.998046	0.000104	7.356
gnl|UG|Ssc#S18547573	pink	107.0283	11.8027	0.141095	0.820951	332.742
gnl|UG|Ssc#S18547696	turquoise	808.7314	255.12	0.963338	0.00838	6.44
gnl|UG|Ssc#S18547766	royalblue	396.2378	19.9733	0.848266	0.069313	41.924
gnl|UG|Ssc#S18547863	turquoise	559.5251	177.763	0.873487	0.052981	9.156
gnl|UG|Ssc#S18548324	brown	92.20627	31.7019	0.733907	0.15803	9.354
gnl|UG|Ssc#S18548354	darkgreen	294.6737	36.9226	0.988315	0.001514	9.722
gnl|UG|Ssc#S18548531	red	244.7847	50.3855	0.834315	0.078915	15.136
gnl|UG|Ssc#S18548560	lightcyan	292.5301	40.6791	0.976926	0.004193	8.022
gnl|UG|Ssc#S18548647	sienna3	927.5429	23.1195	0.976321	0.004358	2.134
gnl|UG|Ssc#S18548799	blue	773.6066	226.716	0.966402	0.007355	8.118
gnl|UG|Ssc#S18548805	brown	68.37851	28.1158	0.675186	0.211059	1.8
gnl|UG|Ssc#S18548934	violet	985.702	73.0841	0.993358	0.000649	20.852
gnl|UG|Ssc#S18548948	black	976.4059	97.086	0.994589	0.000477	22.194
gnl|UG|Ssc#S18549036	salmon	134.0516	30.1286	0.86282	0.059721	76.57
gnl|UG|Ssc#S18549168	red	394.0179	93.6763	0.962616	0.008628	21.986
gnl|UG|Ssc#S18549184	yellow	296.6987	103.649	0.963945	0.008174	16.618
gnl|UG|Ssc#S18549395	grey60	983.4183	59.2431	0.984682	0.002271	24.074
gnl|UG|Ssc#S18549494	grey60	814.7957	56.7435	0.976013	0.004444	9.968
gnl|UG|Ssc#S18549536	blue	878.346	213.929	0.948186	0.014047	56.118
gnl|UG|Ssc#S18549559	darkgrey	184.0671	32.7263	0.978475	0.003779	3.018
gnl|UG|Ssc#S18549601	darkred	193.811	38.5589	0.948921	0.013751	12.412
gnl|UG|Ssc#S18549808	greenyellow	205.3859	43.3462	0.938606	0.018092	19.266
gnl|UG|Ssc#S18549874	brown	95.49676	27.6769	0.486787	0.405635	2.6
gnl|UG|Ssc#S18549905	royalblue	339.7433	32.79	0.993851	0.000578	104.604
gnl|UG|Ssc#S18550002	navy	140.6198	10.9061	-0.33746	0.578628	18.318
gnl|UG|Ssc#S18550089	navy	169.671	37.9982	0.982832	0.002693	1.926
gnl|UG|Ssc#S18550098	violet	961.2466	74.4906	0.997268	0.000171	9.134
gnl|UG|Ssc#S18550130	pink	234.2335	68.6194	0.978113	0.003874	12.304
gnl|UG|Ssc#S18550180	blue	826.549	236.164	0.971496	0.005752	25.85
gnl|UG|Ssc#S18550687	grey60	846.7707	59.2221	0.984674	0.002272	8.148
gnl|UG|Ssc#S18550712	yellow	234.5795	82.1042	0.906126	0.034036	2.96
gnl|UG|Ssc#S18550902	royalblue	245.24	13.9779	0.731595	0.160034	181.758
gnl|UG|Ssc#S18551086	midnightblue	172.7196	39.5112	0.955931	0.011032	33.116
gnl|UG|Ssc#S18551209	purple	101.8594	17.6117	0.922644	0.025525	80.582
gnl|UG|Ssc#S18551494	turquoise	327.6987	111.06	0.700429	0.187731	33.23
gnl|UG|Ssc#S18551520	turquoise	599.5595	163.458	0.841551	0.073887	16.364
gnl|UG|Ssc#S18551559	darkorange	141.3796	18.7403	0.940974	0.017061	15.732
gnl|UG|Ssc#S18551571	white	963.935	29.2783	0.783024	0.117296	32.174
gnl|UG|Ssc#S18551577	turquoise	414.7022	99.4407	0.680723	0.205876	21.7
gnl|UG|Ssc#S18551614	blue	669.5185	200.435	0.934373	0.019982	3.012
gnl|UG|Ssc#S18551801	blue	721.9669	151.624	0.8739	0.052725	38.31
gnl|UG|Ssc#S18551834	lightcyan	169.9639	11.7819	0.412337	0.490278	72.948
gnl|UG|Ssc#S18551845	darkgreen	234.3204	34.4155	0.944819	0.015431	80.856
gnl|UG|Ssc#S18552012	turquoise	825.0018	257.286	0.959166	0.009844	132.252
gnl|UG|Ssc#S18552237	tan	951.4667	58.3595	0.962376	0.008711	12.518
gnl|UG|Ssc#S18552312	turquoise	586.164	188.948	0.859988	0.061552	21.55
gnl|UG|Ssc#S18552383	green	99.85963	42.8939	0.880721	0.048557	86.874

gnl|UG|Ssc#S18552404	royalblue	79.01556	5.79651	0.50725	0.383027	3.704
gnl|UG|Ssc#S18552570	magenta	209.63	65.3142	0.95019	0.013245	51.11
gnl|UG|Ssc#S18552999	royalblue	322.0176	32.0198	0.976265	0.004374	11.504
gnl|UG|Ssc#S18553035	grey	118.9086	12.0254	0.215452	0.727815	218.518
gnl|UG|Ssc#S18553137	black	965.4168	96.0627	0.992317	0.000807	43.786
gnl|UG|Ssc#S18553158	green	102.3188	60.1571	0.981013	0.003132	23.916
gnl|UG|Ssc#S18553245	red	488.5785	103.706	0.985829	0.002021	29.382
gnl|UG|Ssc#S18553368	grey60	976.3111	61.6157	0.99264	0.000757	16.28
gnl|UG|Ssc#S18553403	brown	90.90621	29.3768	0.671206	0.214806	618.788
gnl|UG|Ssc#S18553581	paleturquoise	132.3322	11.588	0.849444	0.06852	63.384
gnl|UG|Ssc#S18553619	darkgreen	226.6815	25.8276	0.888135	0.044152	8.942
gnl|UG|Ssc#S18553622	green	105.0512	49.0274	0.920111	0.026779	14.246
gnl|UG|Ssc#S18553623	salmon	353.781	39.9293	0.928323	0.022787	14.902
gnl|UG|Ssc#S18553702	grey	129.1486	12.6525	0.177797	0.77482	22.054
gnl|UG|Ssc#S18553815	white	977.0609	29.1928	0.78465	0.116011	4.736
gnl|UG|Ssc#S18553939	green	115.1755	55.6586	0.961507	0.009013	12.45
gnl|UG|Ssc#S18554195	greenyellow	185.2435	54.2349	0.994223	0.000527	308.552
gnl|UG|Ssc#S18554238	white	166.2236	6.73817	0.156244	0.801876	5.568
gnl|UG|Ssc#S18554366	darkmagenta	955.2059	25.0476	0.979201	0.003589	34.182
gnl|UG|Ssc#S18554454	green	130.6305	46.5966	0.903945	0.035217	4.18
gnl|UG|Ssc#S18554465	brown	82.45	10.5786	0.296623	0.627942	5.686
gnl|UG|Ssc#S18554486	black	926.7492	84.5146	0.966971	0.00717	12.986
gnl|UG|Ssc#S18554583	lightyellow	975.8028	63.1985	0.998896	4.40E-05	2.946
gnl|UG|Ssc#S18554602	magenta	239.8905	78.0934	0.988286	0.001519	7.222
gnl|UG|Ssc#S18554787	blue	568.1623	149.041	0.86385	0.059059	48.414
gnl|UG|Ssc#S18554865	red	637.3923	105.657	0.988864	0.001408	14.896
gnl|UG|Ssc#S18554887	black	955.2536	91.934	0.983564	0.002523	45.992
gnl|UG|Ssc#S18554897	magenta	280.662	74.6697	0.977598	0.004012	13.41
gnl|UG|Ssc#S18555004	magenta	292.8363	56.8223	0.91683	0.028431	42.864
gnl|UG|Ssc#S18555055	blue	735.1158	217.975	0.95272	0.012253	18.742
gnl|UG|Ssc#S18555062	turquoise	583.0594	168.407	0.855265	0.064645	19.518
gnl|UG|Ssc#S18555355	navy	95.08432	20.1863	0.71078	0.178392	10.636
gnl|UG|Ssc#S18555410	violet	963.5711	74.1334	0.996298	0.00027	14.88
gnl|UG|Ssc#S18555414	green	71.39997	36.7045	0.821001	0.088429	912.106
gnl|UG|Ssc#S18555551	lightyellow	911.8356	61.7647	0.994333	0.000512	24.158
gnl|UG|Ssc#S18555613	pink	179.8811	3.25217	-0.90768	0.033202	11.806
gnl|UG|Ssc#S18555897	green	89.552	37.991	0.853844	0.065584	32.752
gnl|UG|Ssc#S18555925	darkorange	152.5312	21.3739	0.952245	0.012437	11.604
gnl|UG|Ssc#S18556194	purple	191.0077	13.7399	0.750801	0.143619	21.332
gnl|UG|Ssc#S18556295	blue	810.3295	193.318	0.929342	0.022306	28.616
gnl|UG|Ssc#S18556436	darkgrey	70.28362	10.2449	0.696678	0.191148	0.766
gnl|UG|Ssc#S18556442	blue	894.6769	254.512	0.990759	0.001065	8.26
gnl|UG|Ssc#S18556589	pink	77.37907	21.1891	0.502786	0.387934	41.098
gnl|UG|Ssc#S18556801	lightcyan	243.9604	34.1925	0.94569	0.015069	11.898
gnl|UG|Ssc#S18556879	violet	849.7476	69.0774	0.982028	0.002884	4.122
gnl|UG|Ssc#S18556951	navy	173.5157	38.6885	0.923099	0.025302	57.762
gnl|UG|Ssc#S18557192	green	88.4385	46.6218	0.899574	0.037622	6.188
gnl|UG|Ssc#S18557422	turquoise	844.9153	253.833	0.971535	0.00574	14.998

gnl|UG|Ssc#S18557470	orange	278.9282	29.6274	0.960506	0.009366	25.64
gnl|UG|Ssc#S18557502	salmon	153.0231	36.0496	0.904931	0.034682	10.8
gnl|UG|Ssc#S18557542	turquoise	413.0407	134.626	0.774534	0.124077	20.438
gnl|UG|Ssc#S18557598	darkred	229.7074	35.8196	0.938513	0.018133	14.456
gnl|UG|Ssc#S18557699	red	360.5164	85.557	0.944172	0.015702	21.374
gnl|UG|Ssc#S18557771	yellow	91.20733	22.6554	0.609335	0.27529	0.898
gnl|UG|Ssc#S18557837	pink	275.8293	50.0852	0.865536	0.057981	31.122
gnl|UG|Ssc#S18557900	blue	799.884	206.56	0.937942	0.018384	15.432
gnl|UG|Ssc#S18557979	greenyellow	181.6502	44.7307	0.941904	0.016662	9.804
gnl|UG|Ssc#S18557981	turquoise	209.9876	48.1607	0.432855	0.466602	6.652
gnl|UG|Ssc#S18558242	tan	898.5837	65.9559	0.989906	0.001215	3.58
gnl|UG|Ssc#S18558291	black	993.7929	95.5444	0.991096	0.001007	23.4
gnl|UG|Ssc#S18558299	turquoise	834.806	247.448	0.948688	0.013845	30.302
gnl|UG|Ssc#S19537170	lightgreen	306.2589	58.9964	0.991574	0.000927	58.408
gnl|UG|Ssc#S19537267	green	131.8557	42.6148	0.872991	0.053289	2.058
gnl|UG|Ssc#S19537684	lightgreen	274.1379	58.7991	0.99097	0.001029	1.528
gnl|UG|Ssc#S19537979	darkturquoise	144.817	24.9167	0.911116	0.031383	9.648
gnl|UG|Ssc#S19538858	red	434.8519	87.085	0.948091	0.014086	2.716
gnl|UG|Ssc#S19538970	darkmagenta	928.7831	25.633	0.983422	0.002556	18.712
gnl|UG|Ssc#S19538990	violet	964.4406	72.6947	0.992451	0.000786	6.77
gnl|UG|Ssc#S19539315	pink	160.228	42.02	0.880226	0.048856	14.282
gnl|UG|Ssc#S19539468	skyblue	292.2413	27.5572	0.960036	0.009533	23.276
gnl|UG|Ssc#S19539494	yellow	296.36	112.565	0.982408	0.002794	4.806
gnl|UG|Ssc#S19539620	white	163.4524	22.8151	0.803207	0.101647	48.264
gnl|UG|Ssc#S19539691	tan	963.8645	63.1134	0.978608	0.003744	16.204
gnl|UG|Ssc#S19539740	black	978.2566	96.8874	0.993946	0.000565	19.508
gnl|UG|Ssc#S19540060	white	144.3717	6.35999	0.18986	0.759722	6.394
gnl|UG|Ssc#S19540140	lightyellow	998.8763	60.8206	0.991054	0.001014	1.902
gnl|UG|Ssc#S19540267	orange	544.8084	31.5764	0.971464	0.005762	7.918
gnl|UG|Ssc#S19540289	turquoise	831.5058	241.139	0.944847	0.015419	1.656
gnl|UG|Ssc#S19540314	navy	217.3687	28.0626	0.779818	0.119844	27.37
gnl|UG|Ssc#S19540375	turquoise	615.0864	196.225	0.873827	0.05277	32.394
gnl|UG|Ssc#S19540378	orange	303.919	32.4725	0.978354	0.00381	11.562
gnl|UG|Ssc#S19540384	darkturquoise	176.6825	31.8853	0.974108	0.004982	9.94
gnl|UG|Ssc#S19540409	violet	894.5835	72.3226	0.99127	0.000978	9.166
gnl|UG|Ssc#S19540410	tan	698.027	54.1327	0.948441	0.013944	48.094
gnl|UG|Ssc#S19540415	navy	303.8978	29.5891	0.806515	0.099147	8.334
gnl|UG|Ssc#S19540417	lightyellow	895.9311	61.7731	0.994297	0.000517	2.808
gnl|UG|Ssc#S19540505	lightcyan	299.9883	37.2399	0.949562	0.013495	35.886
gnl|UG|Ssc#S19540539	green	116.4731	56.0632	0.967929	0.006861	33.284
gnl|UG|Ssc#S19540632	lightyellow	946.9244	63.2833	0.999186	2.79E-05	3.548
gnl|UG|Ssc#S19540688	yellowgreen	171.0778	16.1078	0.962474	0.008677	32.604
gnl|UG|Ssc#S19540774	brown	213.7858	54.3365	0.836321	0.077511	23.824
gnl|UG|Ssc#S19540934	turquoise	733.3321	217.023	0.932763	0.020716	25.394
gnl|UG|Ssc#S19541050	grey	206.1836	11.7832	0.178598	0.773816	19.186
gnl|UG|Ssc#S19541067	pink	82.52837	17.414	0.278204	0.650404	34.598
gnl|UG|Ssc#S19541084	turquoise	256.926	71.5255	0.550599	0.336194	90.124
gnl|UG|Ssc#S19541103	turquoise	334.6318	111.511	0.746501	0.147249	22.354

gnl|UG|Ssc#S19541153	darkgreen	258.1029	37.573	0.977873	0.003938	10.138
gnl|UG|Ssc#S19541498	darkgrey	174.0963	29.5798	0.9596	0.009689	24.26
gnl|UG|Ssc#S19541523	blue	708.0574	197.8	0.92731	0.023267	53.744
gnl|UG|Ssc#S19541571	yellow	154.1891	48.8143	0.777971	0.121319	11.452
gnl|UG|Ssc#S19541572	lightcyan	230.7107	33.1105	0.904518	0.034906	13.154
gnl|UG|Ssc#S19541579	royalblue	221.5106	25.2071	0.89396	0.040785	50.544
gnl|UG|Ssc#S19541658	brown	158.6032	54.0683	0.945966	0.014955	14.732
gnl|UG|Ssc#S19541728	turquoise	231.8953	56.428	0.481096	0.411976	25.098
gnl|UG|Ssc#S19541992	greenyellow	166.3536	45.5277	0.9506	0.013082	14.336
gnl|UG|Ssc#S19542004	turquoise	821.7081	232.64	0.938628	0.018082	67.742
gnl|UG|Ssc#S19542017	turquoise	247.7	54.5636	0.488551	0.403674	41.2
gnl|UG|Ssc#S19542031	darkorange	137.6279	20.5945	0.95732	0.010517	145.856
gnl|UG|Ssc#S19542061	turquoise	844.6342	258.137	0.9578	0.01034	36.216
gnl|UG|Ssc#S19542087	navy	142.6213	42.3919	0.963443	0.008345	23.736
gnl|UG|Ssc#S19542100	pink	168.0812	47.9628	0.901575	0.036515	7.59
gnl|UG|Ssc#S19542226	red	370.854	92.244	0.960202	0.009474	30.29
gnl|UG|Ssc#S19542246	royalblue	324.0889	32.1721	0.983762	0.002478	4.182
gnl|UG|Ssc#S19542298	turquoise	882.9399	266.553	0.978866	0.003677	19.516
gnl|UG|Ssc#S19542511	pink	279.8728	74.1779	0.983874	0.002452	5.162
gnl|UG|Ssc#S19542514	tan	957.0508	68.6506	0.998039	0.000104	8.738
gnl|UG|Ssc#S19542525	green	59.87254	24.7635	0.674687	0.211527	150.736
gnl|UG|Ssc#S19542622	turquoise	635.8978	163.99	0.853326	0.065928	36.402
gnl|UG|Ssc#S19542649	turquoise	625.0719	172.18	0.875295	0.051864	16.04
gnl|UG|Ssc#S19542654	turquoise	645.0077	203.114	0.917832	0.027923	10.512
gnl|UG|Ssc#S19542684	turquoise	466.6621	146.365	0.797164	0.106262	78.514
gnl|UG|Ssc#S19542699	black	978.5669	98.5275	0.997714	0.000131	17.786
gnl|UG|Ssc#S19542749	navy	145.2266	44.628	0.978407	0.003797	109.508
gnl|UG|Ssc#S19542862	turquoise	340.2332	94.9911	0.642522	0.242341	16.686
gnl|UG|Ssc#S19543319	turquoise	845.0414	257.776	0.974161	0.004967	14.688
gnl|UG|Ssc#S19543409	lightgreen	253.7809	53.726	0.972661	0.005404	8.634
gnl|UG|Ssc#S19543418	blue	613.8957	151.651	0.872117	0.053832	27.47
gnl|UG|Ssc#S19543452	yellow	310.2227	118.836	0.997589	0.000142	3.77
gnl|UG|Ssc#S19543565	black	981.349	94.5542	0.988761	0.001428	10.178
gnl|UG|Ssc#S19544231	yellow	272.2626	100.139	0.959735	0.00964	8.608
gnl|UG|Ssc#S19545362	blue	741.1663	224.753	0.96024	0.00946	65.956
gnl|UG|Ssc#S19545397	yellow	274.1111	89.5968	0.930153	0.021926	546.526
gnl|UG|Ssc#S19545412	lightcyan	302.7954	42.9313	0.982006	0.00289	6.862
gnl|UG|Ssc#S19545451	green	81.6267	38.91	0.828169	0.083265	30.252
gnl|UG|Ssc#S19545467	blue	900.8557	230.881	0.966874	0.007202	35.938
gnl|UG|Ssc#S19545641	yellowgreen	177.3021	17.293	0.975274	0.00465	6.862
gnl|UG|Ssc#S19545786	turquoise	686.0152	224.375	0.899487	0.037671	13.874
gnl|UG|Ssc#S19545977	blue	594.6735	179.708	0.905769	0.034229	77.626
gnl|UG|Ssc#S19546005	lightyellow	989.5253	62.4002	0.996328	0.000267	9.964
gnl|UG|Ssc#S19546024	tan	960.8541	68.5004	0.99724	0.000174	42.928
gnl|UG|Ssc#S19546046	red	302.9114	73.2572	0.909929	0.032008	6.092
gnl|UG|Ssc#S19546062	magenta	223.8857	70.3826	0.963941	0.008175	3.984
gnl|UG|Ssc#S19546082	turquoise	650.6908	207.347	0.920493	0.026589	15.312
gnl|UG|Ssc#S19546091	magenta	289.2523	51.5657	0.896386	0.039409	22.016

gnl|UG|Ssc#S19546104	black	867.2252	79.967	0.956156	0.010948	10.074
gnl|UG|Ssc#S19546106	red	402.9668	76.2983	0.917639	0.02802	45.622
gnl|UG|Ssc#S19546114	midnightblue	171.4873	37.9489	0.939502	0.0177	9.07
gnl|UG|Ssc#S19546165	turquoise	438.4343	105.74	0.718087	0.171882	5.06
gnl|UG|Ssc#S19546218	darkorange	47.18578	2.74966	0.464608	0.430474	13.322
gnl|UG|Ssc#S19546257	turquoise	655.2155	175.648	0.86772	0.056593	53.572
gnl|UG|Ssc#S19546269	salmon	207.4634	48.7666	0.981054	0.003122	8.82
gnl|UG|Ssc#S19546291	blue	851.0584	244.887	0.980131	0.003352	4.322
gnl|UG|Ssc#S19546334	blue	579.7303	121.689	0.824282	0.086053	10.036
gnl|UG|Ssc#S19547691	midnightblue	198.469	30.823	0.888145	0.044146	158.274
gnl|UG|Ssc#S19547811	black	948.7043	96.2737	0.992889	0.000719	9.656
gnl|UG|Ssc#S19547860	grey60	906.5755	62.2336	0.994818	0.000447	31.818
gnl|UG|Ssc#S19547879	black	906.0291	94.3581	0.988774	0.001425	2.944
gnl|UG|Ssc#S19548031	yellow	164.5691	49.9609	0.786772	0.11434	24.926
gnl|UG|Ssc#S19548740	greenyellow	149.9233	41.4243	0.928676	0.02262	61.484
gnl|UG|Ssc#S19548981	yellow	232.6932	74.7323	0.893479	0.04106	10.834
gnl|UG|Ssc#S19549058	paleturquoise	169.6827	18.5718	0.948616	0.013874	24.338
gnl|UG|Ssc#S19549165	red	537.8796	105.493	0.989306	0.001325	16.796
gnl|UG|Ssc#S19549174	grey	247.9511	12.2371	0.98534	0.002126	8.574
gnl|UG|Ssc#S19549267	sienna3	960.1794	26.0823	0.999709	5.96E-06	9.342
gnl|UG|Ssc#S19549653	darkred	257.8744	19.198	0.796141	0.107049	26.688
gnl|UG|Ssc#S19550340	darkgreen	273.6065	34.3957	0.958463	0.010099	4.69
gnl|UG|Ssc#S19550965	yellow	310.3477	118.207	0.996378	0.000261	33.996
gnl|UG|Ssc#S20938341	sienna3	997.558	25.2344	0.993028	0.000698	10.914
gnl|UG|Ssc#S20944779	magenta	207.7938	64.1263	0.947032	0.014517	3.676
gnl|UG|Ssc#S20945025	red	573.4275	93.1993	0.962492	0.008671	69.23
gnl|UG|Ssc#S20945368	magenta	196.4009	60.1925	0.932334	0.020914	1.58
gnl|UG|Ssc#S21107071	violet	989.6679	72.0963	0.990648	0.001084	37.062
gnl|UG|Ssc#S21107119	blue	915.441	244.53	0.98208	0.002872	7.912
gnl|UG|Ssc#S21550606	white	210.0457	23.4179	0.936124	0.019193	1.87
gnl|UG|Ssc#S21554547	turquoise	871.1097	258.789	0.964261	0.008067	4.786
gnl|UG|Ssc#S21558492	lightgreen	308.0946	59.314	0.992625	0.000759	8.71
gnl|UG|Ssc#S21575132	white	931.4712	28.7854	0.779551	0.120056	4.052
gnl|UG|Ssc#S21575274	greenyellow	192.2271	54.9422	0.996212	0.00028	19.41
gnl|UG|Ssc#S22271675	brown	101.1839	33.8573	0.552338	0.334346	22.116
gnl|UG|Ssc#S22272334	purple	224.5463	19.7235	-0.85961	0.061796	14.436
gnl|UG|Ssc#S22272834	yellow	286.6086	106.455	0.969552	0.006349	4
gnl|UG|Ssc#S22273263	brown	128.9006	45.0417	0.864867	0.058408	2.202
gnl|UG|Ssc#S22273337	turquoise	440.6442	141.717	0.764726	0.132051	2.758
gnl|UG|Ssc#S22274193	black	960.7358	98.581	0.997886	0.000117	9.566
gnl|UG|Ssc#S22274398	midnightblue	123.422	30.5543	0.886956	0.044844	16.348
gnl|UG|Ssc#S22274609	purple	206.6163	15.1936	-0.82119	0.088292	5.422
gnl|UG|Ssc#S22274737	magenta	183.8336	55.7147	0.918072	0.027802	2.878
gnl|UG|Ssc#S22275000	red	395.8802	95.8181	0.967765	0.006914	28.666
gnl|UG|Ssc#S22275082	salmon	131.419	39.4938	0.927959	0.022959	10.846
gnl|UG|Ssc#S22275237	royalblue	288.7784	30.1709	0.959509	0.009721	5.836
gnl|UG|Ssc#S22275831	grey	121.7201	12.0788	0.193492	0.755184	2.36
gnl|UG|Ssc#S22275888	midnightblue	66.5293	3.63271	-0.30408	0.618891	11.72

gnl|UG|Ssc#S22275958	black	953.8163	98.5132	0.997979	0.000109	29.456
gnl|UG|Ssc#S22276100	darkturquoise	199.1864	31.4881	0.968685	0.006621	1.628
gnl|UG|Ssc#S22276642	darkred	240.9949	17.55	0.77144	0.126577	1.79
gnl|UG|Ssc#S22277516	turquoise	307.246	102.507	0.658928	0.22648	43.496
gnl|UG|Ssc#S22277550	darkturquoise	194.1711	35.1289	0.997656	0.000136	8.008
gnl|UG|Ssc#S22278253	salmon	181.5139	44.9867	0.961563	0.008994	24.462
gnl|UG|Ssc#S22278643	blue	940.8075	224.223	0.965484	0.007658	28.158
gnl|UG|Ssc#S22279857	pink	264.5653	75.5496	0.998359	7.98E-05	0.118
gnl|UG|Ssc#S22279946	paleturquoise	163.9317	15.7231	0.907863	0.033105	11.39
gnl|UG|Ssc#S22280355	paleturquoise	157.8937	17.3122	0.930052	0.021973	3.372
gnl|UG|Ssc#S22281680	green	175.0134	22.2124	0.338119	0.577843	15.124
gnl|UG|Ssc#S22281703	grey60	969.8012	60.2145	0.988084	0.001559	18.94
gnl|UG|Ssc#S22282893	black	978.7427	96.4135	0.992874	0.000721	26.404
gnl|UG|Ssc#S22283333	red	716.5362	83.6792	0.93745	0.018602	69.234
gnl|UG|Ssc#S22284067	red	423.3772	103.968	0.986109	0.001961	7.682
gnl|UG|Ssc#S22284475	black	961.8265	98.4879	0.998041	0.000104	16.12
gnl|UG|Ssc#S22284677	purple	214.8011	16.7712	-0.79634	0.106895	4.298
gnl|UG|Ssc#S22285435	turquoise	204.2432	65.3157	0.590327	0.294654	2.088
gnl|UG|Ssc#S22286222	red	591.5798	108.179	0.994256	0.000522	12.628
gnl|UG|Ssc#S22286260	grey	153.1669	14.0412	-0.8714	0.054279	58.916
gnl|UG|Ssc#S22286650	turquoise	358.0238	106.664	0.687196	0.199864	13.036
gnl|UG|Ssc#S22287254	green	96.79745	33.1652	0.793584	0.109024	31.188
gnl|UG|Ssc#S22287363	turquoise	898.7535	263.208	0.98604	0.001976	18.304
gnl|UG|Ssc#S22304929	turquoise	136.3614	44.3926	0.487819	0.404488	4.914
gnl|UG|Ssc#S22312937	lightyellow	965.1814	63.0342	0.998406	7.64E-05	9.104
gnl|UG|Ssc#S22313154	turquoise	873.5031	258.471	0.980621	0.003229	165.894
gnl|UG|Ssc#S22313956	white	185.4702	25.7445	0.888067	0.044192	6.708
gnl|UG|Ssc#S22316206	brown	70.9781	28.4077	0.531923	0.356188	25.136
gnl|UG|Ssc#S23689583	blue	682.1152	174.757	0.906644	0.033757	17.964
gnl|UG|Ssc#S23689697	tan	933.6324	68.6914	0.998057	0.000103	3.642
gnl|UG|Ssc#S23689787	blue	707.0078	156.443	0.88499	0.046004	10.332
gnl|UG|Ssc#S23689840	skyblue	193.6682	26.6485	0.952993	0.012148	37.052
gnl|UG|Ssc#S23689938	white	227.7394	22.5759	0.68016	0.206402	6.768
gnl|UG|Ssc#S23690002	green	153.3222	43.1909	0.90426	0.035046	36.126
gnl|UG|Ssc#S23690384	magenta	242.621	78.7745	0.990382	0.001131	5.924
gnl|UG|Ssc#S23690475	turquoise	737.4587	204.706	0.921194	0.026241	44.072
gnl|UG|Ssc#S23690683	lightyellow	987.0149	63.1196	0.998624	6.13E-05	36.352
gnl|UG|Ssc#S23690802	darkturquoise	158.8701	22.9112	0.890017	0.043055	28.062
gnl|UG|Ssc#S23690807	yellow	261.9429	87.0877	0.928145	0.022871	20.098
gnl|UG|Ssc#S23690967	blue	548.5393	126.195	0.836911	0.077099	28.766
gnl|UG|Ssc#S23691069	red	604.0217	108.616	0.995202	0.000399	32.576
gnl|UG|Ssc#S23691079	turquoise	913.2674	259.89	0.974375	0.004905	43.142
gnl|UG|Ssc#S23691136	red	525.8688	84.9376	0.940583	0.01723	25.016
gnl|UG|Ssc#S23691157	salmon	295.0042	43.5574	0.949212	0.013634	11.9
gnl|UG|Ssc#S23691372	yellow	189.2282	65.0935	0.84797	0.069513	9.936
gnl|UG|Ssc#S23691718	green	139.5693	19.0843	0.073036	0.90709	59.164
gnl|UG|Ssc#S23691805	brown	144.5294	17.4198	0.5925	0.292423	13.458
gnl|UG|Ssc#S23691900	turquoise	947.4292	263.823	0.985138	0.00217	11.438

gnl|UG|Ssc#S23691927	green	114.0344	18.2814	0.403652	0.500374	13.366
gnl|UG|Ssc#S23692548	salmon	136.2726	34.4176	0.891818	0.042013	9.048
gnl|UG|Ssc#S23692872	turquoise	843.4156	235.651	0.94544	0.015172	15.136
gnl|UG|Ssc#S23692986	black	621.6867	64.2012	0.911671	0.031092	3.822
gnl|UG|Ssc#S23693254	greenyellow	213.3963	47.452	0.959556	0.009704	21.43
gnl|UG|Ssc#S23693266	turquoise	235.6574	59.9209	0.594103	0.29078	16.376
gnl|UG|Ssc#S23693320	red	666.1172	97.5145	0.970798	0.005964	11.232
gnl|UG|Ssc#S23693357	red	433.0384	102.649	0.983362	0.00257	129.166
gnl|UG|Ssc#S23693410	royalblue	387.9215	32.5941	0.996028	0.0003	9.392
gnl|UG|Ssc#S23693441	darkgrey	178.5682	30.6687	0.966714	0.007254	13.424
gnl|UG|Ssc#S23694531	turquoise	295.461	65.5999	0.55984	0.326408	30.874
gnl|UG|Ssc#S23694535	blue	638.318	197.297	0.928118	0.022884	16.088
gnl|UG|Ssc#S23694970	yellow	208.8703	69.0823	0.862958	0.059632	25.742
gnl|UG|Ssc#S23695084	white	185.573	19.6073	0.639504	0.24529	22.286
gnl|UG|Ssc#S23695102	lightcyan	156.4116	10.9539	0.443009	0.454979	38.56
gnl|UG|Ssc#S23695121	turquoise	488.0582	117.805	0.772485	0.125731	4.628
gnl|UG|Ssc#S23695134	yellow	306.1996	118.789	0.997849	0.00012	3.742
gnl|UG|Ssc#S23695164	white	963.91	26.7195	0.772496	0.125722	5.272
gnl|UG|Ssc#S23695204	black	802.8999	78.0845	0.951186	0.012851	36.876
gnl|UG|Ssc#S23695295	grey	220.8452	11.4641	0.520916	0.368102	22.186
gnl|UG|Ssc#S23695328	turquoise	880.9723	249.652	0.961509	0.009013	13.812
gnl|UG|Ssc#S23695397	salmon	140.5725	38.3319	0.920082	0.026793	10.456
gnl|UG|Ssc#S23695759	green	70.45445	27.5925	0.731222	0.160358	25.906
gnl|UG|Ssc#S23695896	darkgreen	247.0396	36.9495	0.975798	0.004503	54.418
gnl|UG|Ssc#S23696553	blue	877.775	230.024	0.965796	0.007555	18.938
gnl|UG|Ssc#S23696616	yellow	291.0455	111.122	0.981413	0.003034	110.464
gnl|UG|Ssc#S23696619	turquoise	601.8067	167.959	0.84789	0.069567	15.508
gnl|UG|Ssc#S23696689	lightgreen	291.7922	59.0754	0.991761	0.000897	11.84
gnl|UG|Ssc#S23696700	darkorange	203.2458	19.9064	0.986737	0.00183	5.438
gnl|UG|Ssc#S23696826	purple	87.95389	18.2424	0.767498	0.129783	12.694
gnl|UG|Ssc#S23697216	turquoise	883.939	267.546	0.974014	0.005009	63.084
gnl|UG|Ssc#S23697230	magenta	238.5053	56.7764	0.921884	0.0259	26.986
gnl|UG|Ssc#S23697813	yellow	242.4116	82.4666	0.910001	0.03197	3.018
gnl|UG|Ssc#S23697932	pink	203.5017	39.0394	0.852135	0.06672	32.684
gnl|UG|Ssc#S23698058	blue	758.755	217.852	0.958177	0.010203	12.52
gnl|UG|Ssc#S23698110	paleturquoise	139.9493	12.149	0.824102	0.086183	19.898
gnl|UG|Ssc#S23698716	midnightblue	178.8196	40.7293	0.967586	0.006971	123.776
gnl|UG|Ssc#S23698735	sienna3	903.7303	25.1741	0.992671	0.000752	5.576
gnl|UG|Ssc#S23698752	darkgreen	257.3968	35.578	0.968509	0.006677	28.084
gnl|UG|Ssc#S23698821	lightcyan	248.7126	38.9036	0.965659	0.0076	10.344
gnl|UG|Ssc#S23699498	yellow	209.0617	73.3576	0.880739	0.048546	0.566
gnl|UG|Ssc#S23699782	navy	129.5499	40.8531	0.928742	0.022588	1.772
gnl|UG|Ssc#S23699859	darkturquoise	205.8758	30.7823	0.964496	0.007988	7.482
gnl|UG|Ssc#S23699941	navy	134.9967	3.33088	-0.66633	0.219424	2.348
gnl|UG|Ssc#S23700002	turquoise	515.6845	167.272	0.834617	0.078703	92.826
gnl|UG|Ssc#S23700016	yellowgreen	162.7663	16.576	0.966404	0.007355	11.53
gnl|UG|Ssc#S23700159	purple	54.36891	13.8586	0.819631	0.089426	67.062
gnl|UG|Ssc#S23700422	red	421.4231	103.336	0.984821	0.00224	118.48

gnl|UG|Ssc#S23701109	blue	563.5358	173.685	0.901978	0.036293	32.268
gnl|UG|Ssc#S23701196	greenyellow	48.8584	2.21865	0.279553	0.648754	18.47
gnl|UG|Ssc#S23701257	blue	862.4637	243.238	0.979516	0.003508	17.546
gnl|UG|Ssc#S23701295	blue	680.4684	190.699	0.923214	0.025246	8.122
gnl|UG|Ssc#S23701516	sienna3	948.9557	25.7602	0.997291	0.000169	13.826
gnl|UG|Ssc#S23755332	darkred	251.8575	40.8116	0.971386	0.005785	2.95
gnl|UG|Ssc#S23755373	brown	208.7836	61.67	0.957719	0.01037	27.07
gnl|UG|Ssc#S23755435	blue	831.7251	181.731	0.91624	0.028731	181.21
gnl|UG|Ssc#S23755492	violet	929.4696	74.6499	0.997668	0.000135	1.63
gnl|UG|Ssc#S23755550	green	157.0934	22.5296	0.297578	0.626781	23.54
gnl|UG|Ssc#S23755551	green	109.6375	31.4748	0.759949	0.135987	56.424
gnl|UG|Ssc#S23755976	darkorange	112.8165	17.3679	0.89747	0.038799	21.696
gnl|UG|Ssc#S23756098	purple	82.41435	16.9778	0.742178	0.150925	225.908
gnl|UG|Ssc#S23756321	black	945.7746	97.3701	0.995201	0.000399	8.934
gnl|UG|Ssc#S23756324	green	95.39442	40.9483	0.837006	0.077033	9.204
gnl|UG|Ssc#S23756535	darkturquoise	135.0747	6.88341	0.621767	0.262816	2.232
gnl|UG|Ssc#S23756582	pink	264.5653	75.5496	0.998359	7.98E-05	0.078
gnl|UG|Ssc#S23756822	white	814.0091	26.7511	0.809222	0.097116	7.792
gnl|UG|Ssc#S23756850	salmon	168.7954	38.5226	0.919425	0.027122	124.658
gnl|UG|Ssc#S23756909	turquoise	877.6614	245.434	0.958243	0.010179	29.996
gnl|UG|Ssc#S23757077	navy	129.5499	40.8531	0.928742	0.022588	0.272
gnl|UG|Ssc#S23757521	pink	277.9503	71.544	0.970311	0.006114	17.286
gnl|UG|Ssc#S23757556	navy	106.5974	25.2758	0.791274	0.110818	10.786
gnl|UG|Ssc#S23757653	pink	273.644	69.3339	0.969657	0.006316	12.716
gnl|UG|Ssc#S23757695	lightyellow	903.8376	61.2376	0.99262	0.00076	31.58
gnl|UG|Ssc#S23757705	blue	515.0101	133.552	0.841167	0.074151	11.538
gnl|UG|Ssc#S23757763	green	93.25576	35.2818	0.814554	0.093152	41.782
gnl|UG|Ssc#S23758125	royalblue	265.6713	13.3476	0.715456	0.174218	14.958
gnl|UG|Ssc#S23758297	orange	453.6493	32.8967	0.980664	0.003218	20.672
gnl|UG|Ssc#S23758340	magenta	273.7198	75.8915	0.982327	0.002813	3.594
gnl|UG|Ssc#S23758422	blue	817.3722	231.712	0.96563	0.007609	28.2
gnl|UG|Ssc#S23758705	greenyellow	122.3417	10.8903	0.659809	0.225636	143.996
gnl|UG|Ssc#S23758881	salmon	198.8034	50.2704	0.989658	0.001261	41.24
gnl|UG|Ssc#S23758884	red	447.4113	103.547	0.985149	0.002168	27.022
gnl|UG|Ssc#S23758955	turquoise	811.6879	254.492	0.950359	0.013178	100.722
gnl|UG|Ssc#S23759010	lightyellow	1001.445	60.0877	0.988602	0.001458	21.05
gnl|UG|Ssc#S23759049	darkmagenta	914.9029	26.558	0.990838	0.001051	6.57
gnl|UG|Ssc#S23759123	yellow	285.929	104.618	0.965604	0.007618	3.644
gnl|UG|Ssc#S23759125	royalblue	268.4968	25.4713	0.904126	0.035119	12.004
gnl|UG|Ssc#S23759297	midnightblue	115.1485	34.1572	0.937553	0.018556	0.608
gnl|UG|Ssc#S23759508	navy	206.7399	10.4098	-0.51997	0.369136	85.928
gnl|UG|Ssc#S23759619	green	73.10773	29.9809	0.744946	0.148568	45.99
gnl|UG|Ssc#S23759640	turquoise	778.158	247.182	0.93498	0.019707	40.926
gnl|UG|Ssc#S23759681	green	86.97867	47.7167	0.911504	0.03118	19.008
gnl|UG|Ssc#S23759759	blue	945.3182	210.653	0.9506	0.013082	33.95
gnl|UG|Ssc#S23760025	purple	149.4636	12.0264	-0.92129	0.026191	22.812
gnl|UG|Ssc#S23760280	blue	830.1191	211.837	0.947184	0.014455	17.908
gnl|UG|Ssc#S23760498	blue	897.0628	197.909	0.935535	0.019457	20.722

gnl|UG|Ssc#S23760821	green	99.89155	51.4852	0.920832	0.02642	10.72
gnl|UG|Ssc#S23761044	grey60	940.06	61.946	0.993775	0.000589	9.85
gnl|UG|Ssc#S23761475	yellow	308.9719	117.685	0.994582	0.000478	1.242
gnl|UG|Ssc#S23761814	purple	120.0137	13.1742	-0.60868	0.275949	22.336
gnl|UG|Ssc#S23761942	green	103.2762	27.9556	0.743212	0.150043	2.94
gnl|UG|Ssc#S23762132	yellow	275.7757	103.972	0.964382	0.008026	11.352
gnl|UG|Ssc#S23762352	blue	783.5289	196.5	0.928867	0.02253	5.446
gnl|UG|Ssc#S23762453	blue	857.2214	234.038	0.970188	0.006151	36.894
gnl|UG|Ssc#S23762471	brown	156.613	40.7668	0.805547	0.099877	19.502
gnl|UG|Ssc#S23762560	grey	142.6195	7.4064	0.887207	0.044696	13.776
gnl|UG|Ssc#S23762736	royalblue	314.8344	31.3159	0.975926	0.004468	70.506
gnl|UG|Ssc#S23763543	yellow	299.1772	107.427	0.972729	0.005384	13.16
gnl|UG|Ssc#S23763873	violet	951.9937	75.104	0.998919	4.26E-05	29.434
gnl|UG|Ssc#S23764274	magenta	273.2259	78.0096	0.987496	0.001675	6.886
gnl|UG|Ssc#S23764286	turquoise	392.8947	110.787	0.71136	0.177873	5.198
gnl|UG|Ssc#S23764351	red	725.4388	90.8548	0.955142	0.011328	16.234
gnl|UG|Ssc#S23764699	salmon	314.1365	42.7411	0.943124	0.016143	31.35
gnl|UG|Ssc#S23765090	turquoise	939.3573	269.828	0.989697	0.001254	18.54
gnl|UG|Ssc#S23765150	yellow	288.8633	101.388	0.957977	0.010276	11.228
gnl|UG|Ssc#S23765416	yellow	235.3629	75.7101	0.897856	0.038582	6.892
gnl|UG|Ssc#S23765932	navy	197.2487	10.8531	-0.60065	0.28409	184.246
gnl|UG|Ssc#S23765987	blue	884.0043	250.793	0.988151	0.001546	84.06
gnl|UG|Ssc#S23766507	magenta	203.2979	59.8779	0.934206	0.020058	9.714
gnl|UG|Ssc#S23766613	turquoise	560.3924	140.494	0.789147	0.112478	5.036
gnl|UG|Ssc#S23766626	lightgreen	301.1065	57.9469	0.98786	0.001603	2.36
gnl|UG|Ssc#S23766637	turquoise	837.6027	262.692	0.963284	0.008399	90.88
gnl|UG|Ssc#S23766713	turquoise	242.9831	63.3463	0.534881	0.353002	6.516
gnl|UG|Ssc#S23766741	lightgreen	301.1398	60.8023	0.997691	0.000133	3.062
gnl|UG|Ssc#S23767099	lightyellow	893.4107	61.425	0.993177	0.000676	7.096
gnl|UG|Ssc#S23767247	red	666.0672	101.38	0.980003	0.003384	11.522
gnl|UG|Ssc#S23768000	turquoise	904.6096	257.484	0.970805	0.005962	22.322
gnl|UG|Ssc#S23768055	turquoise	700.5173	221.819	0.900511	0.037103	20.562
gnl|UG|Ssc#S23768102	magenta	269.6816	80.4722	0.995085	0.000413	4.248
gnl|UG|Ssc#S23768212	lightgreen	280.9114	56.3541	0.982125	0.002861	13.56
gnl|UG|Ssc#S23768320	red	420.5364	92.8566	0.961251	0.009103	22.928
gnl|UG|Ssc#S23769437	green	102.5011	59.1967	0.971119	0.005866	27.726
gnl|UG|Ssc#S23769966	midnightblue	92.92207	36.0669	0.945037	0.01534	11.712
gnl|UG|Ssc#S23770591	turquoise	293.1723	84.0732	0.627441	0.257174	6.694
gnl|UG|Ssc#S23770850	pink	124.2573	8.72084	-0.19233	0.756639	70.526
gnl|UG|Ssc#S23770912	yellow	211.822	67.6496	0.860104	0.061477	1.788
gnl|UG|Ssc#S23770961	magenta	261.7777	75.4171	0.980834	0.003176	60.152
gnl|UG|Ssc#S23771584	yellow	200.5021	70.0749	0.865289	0.058138	2.674
gnl|UG|Ssc#S23772152	yellow	308.5696	118.297	0.99659	0.000239	42.976
gnl|UG|Ssc#S23772162	darkorange	97.76632	12.2524	0.817315	0.09112	4.652
gnl|UG|Ssc#S23772243	turquoise	330.8263	75.2862	0.60508	0.279595	25.456
gnl|UG|Ssc#S23772402	tan	965.6926	59.4407	0.965927	0.007511	26.584
gnl|UG|Ssc#S23773089	paleturquoise	190.7037	19.9301	0.955801	0.01108	1.124
gnl|UG|Ssc#S23775145	blue	742.6613	212.707	0.945003	0.015354	99.22

gnl|UG|Ssc#S23775247	royalblue	369.0636	32.9774	0.997605	0.000141	3917.98
gnl|UG|Ssc#S23775283	blue	690.7921	199.781	0.938321	0.018217	13.374
gnl|UG|Ssc#S23775351	magenta	265.4117	79.4882	0.992759	0.000739	9.304
gnl|UG|Ssc#S23775735	pink	219.3998	63.8867	0.961593	0.008983	45.668
gnl|UG|Ssc#S23775891	greenyellow	205.9696	44.6359	0.945137	0.015299	106.154
gnl|UG|Ssc#S23776629	green	93.64861	46.0764	0.890647	0.04269	31.7
gnl|UG|Ssc#S23776821	darkred	189.3895	33.614	0.928859	0.022533	14.766
gnl|UG|Ssc#S23776939	lightcyan	296.1664	43.7365	0.98603	0.001978	18.66
gnl|UG|Ssc#S24609641	blue	729.2138	209.525	0.946037	0.014926	28.49
gnl|UG|Ssc#S24610253	purple	105.6753	22.5078	0.926065	0.023863	3.144
gnl|UG|Ssc#S25134978	green	102.0927	42.2246	0.881187	0.048276	17.968
gnl|UG|Ssc#S25135543	lightcyan	95.01709	8.18056	0.633467	0.251219	28.362
gnl|UG|Ssc#S25135588	brown	85.30389	24.8782	0.437838	0.46089	15.264
gnl|UG|Ssc#S25135664	green	86.13036	49.7216	0.904929	0.034683	510.858
gnl|UG|Ssc#S25135824	green	106.0996	41.7424	0.867861	0.056504	33.136
gnl|UG|Ssc#S26390532	black	805.3261	77.3823	0.949556	0.013497	2.598
gnl|UG|Ssc#S26391783	darkgreen	215.7482	32.5666	0.930673	0.021683	10.132
gnl|UG|Ssc#S26394010	orange	599.7007	26.6614	0.934699	0.019834	5.88
gnl|UG|Ssc#S26395675	purple	169.7972	17.0619	-0.91197	0.030934	34.168
gnl|UG|Ssc#S26396713	blue	691.8858	170.638	0.89884	0.038032	18.342
gnl|UG|Ssc#S26399811	turquoise	194.7279	59.7136	0.532582	0.355477	63.96
gnl|UG|Ssc#S26400438	orange	391.0408	17.1944	0.839191	0.075515	34.6
gnl|UG|Ssc#S26400566	darkred	248.7499	45.3205	0.995562	0.000355	3.63
gnl|UG|Ssc#S26434913	turquoise	329.9492	71.0083	0.608683	0.275949	3.488
gnl|UG|Ssc#S26644701	yellowgreen	167.7709	17.747	0.987468	0.001681	53.628
gnl|UG|Ssc#S26645511	turquoise	350.3859	85.0139	0.604434	0.28025	23.892
gnl|UG|Ssc#S26646704	green	83.95104	38.1432	0.832059	0.080504	12.888
gnl|UG|Ssc#S26647295	turquoise	764.1955	246.665	0.933736	0.020272	44.842
gnl|UG|Ssc#S26648149	darkorange	147.5216	7.90431	0.334281	0.582446	14.82
gnl|UG|Ssc#S26648312	grey60	925.254	62.8138	0.99669	0.000228	4.464
gnl|UG|Ssc#S26648808	green	168.6164	20.9547	0.405196	0.498576	13.214
gnl|UG|Ssc#S26648854	grey60	988.9315	58.4059	0.981693	0.002965	12.09
gnl|UG|Ssc#S26649927	black	963.831	98.3017	0.99722	0.000176	11.126
gnl|UG|Ssc#S26650236	magenta	242.1394	77.9548	0.9871	0.001755	4.032
gnl|UG|Ssc#S26650700	violet	966.901	74.89	0.998342	8.10E-05	23.918
gnl|UG|Ssc#S26651783	magenta	259.6159	82.211	0.999655	7.69E-06	3.018
gnl|UG|Ssc#S26652589	greenyellow	218.9162	47.912	0.963952	0.008171	5.608
gnl|UG|Ssc#S26710643	lightgreen	300.0455	60.5339	0.996809	0.000216	4.538
gnl|UG|Ssc#S26710759	orange	640.4396	31.4317	0.97064	0.006012	89.226
gnl|UG|Ssc#S26710977	lightgreen	291.422	58.0067	0.988119	0.001552	2.204
gnl|UG|Ssc#S26711201	yellow	284.2709	106.321	0.97004	0.006197	6.666
gnl|UG|Ssc#S26711451	yellow	307.4795	114.263	0.987106	0.001754	8.266
gnl|UG|Ssc#S26711473	navy	147.3344	39.063	0.948802	0.013799	7.92
gnl|UG|Ssc#S26711650	turquoise	651.9807	189.497	0.889778	0.043194	80.92
gnl|UG|Ssc#S26712602	lightgreen	290.7708	57.8979	0.987729	0.001629	4.344
gnl|UG|Ssc#S26712901	yellow	308.9719	117.685	0.994582	0.000478	1.242
gnl|UG|Ssc#S26713258	darkgreen	252.4639	32.4556	0.937079	0.018767	5.08
gnl|UG|Ssc#S26713408	green	180.6036	17.3946	0.140431	0.821787	3.264

gnl|UG|Ssc#S26713534	lightgreen	257.2769	54.7886	0.976633	0.004273	3.408
gnl|UG|Ssc#S26713597	yellow	305.8833	113.351	0.984426	0.002328	7.586
gnl|UG|Ssc#S26713657	turquoise	777.777	250.276	0.950562	0.013097	26.824
gnl|UG|Ssc#S26713880	lightgreen	287.2031	60.2361	0.995809	0.000326	13.064
gnl|UG|Ssc#S26714423	red	386.6673	94.6126	0.965803	0.007552	30.218
gnl|UG|Ssc#S26714441	turquoise	523.1362	146.459	0.809172	0.097154	126.554
gnl|UG|Ssc#S26714520	navy	111.8184	36.9372	0.886905	0.044874	13.19
gnl|UG|Ssc#S26714587	green	175.5179	19.8366	0.139899	0.822458	1.77
gnl|UG|Ssc#S26714690	yellow	304.3285	112.935	0.984179	0.002383	4.192
gnl|UG|Ssc#S26714922	salmon	124.2756	30.3797	0.862081	0.060197	54.25
gnl|UG|Ssc#S26715431	darkred	241.6351	41.3051	0.97444	0.004887	3.084
gnl|UG|Ssc#S26715859	darkturquoise	210.467	32.9929	0.982265	0.002828	1.538
gnl|UG|Ssc#S26715866	yellow	287.75	101.342	0.955944	0.011027	9.566
gnl|UG|Ssc#S26716026	navy	80.57986	6.42668	-0.00489	0.99377	89.132
gnl|UG|Ssc#S26716062	darkred	276.2128	44.9182	0.997185	0.000179	3.514
gnl|UG|Ssc#S26716533	greenyellow	175.1041	50.8868	0.97799	0.003907	0.36
gnl|UG|Ssc#S26716874	yellow	309.3335	118.716	0.997255	0.000173	10.708
gnl|UG|Ssc#S26717032	yellow	300.7349	109.933	0.976418	0.004332	68.936
gnl|UG|Ssc#S26717661	blue	725.2208	184.463	0.920223	0.026723	5.046
gnl|UG|Ssc#S26717691	royalblue	320.0081	16.2178	0.797193	0.106239	8.072
gnl|UG|Ssc#S26718069	orange	279.6646	29.5618	0.955675	0.011128	34.946
gnl|UG|Ssc#S26718537	turquoise	384.5081	102.278	0.696716	0.191113	2.98
gnl|UG|Ssc#S26718597	tan	823.4019	63.7095	0.982725	0.002719	42.958
gnl|UG|Ssc#S26718943	midnightblue	163.1163	40.1971	0.969051	0.006506	11.49
gnl|UG|Ssc#S26718955	brown	93.3696	32.4135	0.796562	0.106724	2.844
gnl|UG|Ssc#S26719135	yellow	277.2389	96.4176	0.94596	0.014958	9.854
gnl|UG|Ssc#S26719371	pink	240.9872	72.1035	0.995064	0.000416	413.882
gnl|UG|Ssc#S26719435	darkred	223.6073	35.4726	0.94306	0.01617	259.056
gnl|UG|Ssc#S26719749	yellow	177.1865	45.8878	0.78309	0.117245	1.52
gnl|UG|Ssc#S26719780	lightgreen	278.9062	56.7056	0.98339	0.002563	6.338
gnl|UG|Ssc#S26719881	white	192.5646	20.8634	0.768717	0.128789	4.392
gnl|UG|Ssc#S26719960	lightgreen	273.1209	55.5241	0.979298	0.003565	2.382
gnl|UG|Ssc#S26720075	grey	143.0791	11.6687	0.397302	0.507782	0.382
gnl|UG|Ssc#S26720413	green	42.01771	12.7326	0.48531	0.407279	5.568
gnl|UG|Ssc#S26720420	turquoise	890.4278	251.973	0.965323	0.007711	34.194
gnl|UG|Ssc#S26720426	darkorange	216.1817	18.8879	0.980554	0.003246	9.136
gnl|UG|Ssc#S26720529	navy	94.84829	29.8311	0.811803	0.095191	4.3
gnl|UG|Ssc#S26720534	blue	889.7263	249.532	0.987983	0.001578	10.644
gnl|UG|Ssc#S26720630	blue	812.1503	167.248	0.900072	0.037346	6.778
gnl|UG|Ssc#S26720820	yellow	302.4393	115.801	0.990069	0.001186	3.518
gnl|UG|Ssc#S26721025	yellow	308.2459	116.985	0.992725	0.000744	61.448
gnl|UG|Ssc#S26721043	darkgreen	216.9529	15.8301	0.720006	0.170184	0.94
gnl|UG|Ssc#S26721109	darkturquoise	205.116	35.025	0.99736	0.000163	7.404
gnl|UG|Ssc#S26721489	lightyellow	959.812	62.653	0.997211	0.000177	16.868
gnl|UG|Ssc#S26721530	brown	66.69505	17.171	0.476324	0.417311	11.456
gnl|UG|Ssc#S26721534	yellow	309.2016	118.918	0.998134	9.67E-05	99.322
gnl|UG|Ssc#S26721589	lightgreen	304.4755	59.2048	0.992242	0.000819	1.688
gnl|UG|Ssc#S26721666	yellow	302.3932	116.021	0.990798	0.001058	4.402

gnl|UG|Ssc#S26721918	blue	932.0325	226.123	0.964733	0.007908	14.24
gnl|UG|Ssc#S26722141	paleturquoise	185.4271	19.8771	0.958074	0.01024	2.506
gnl|UG|Ssc#S26722216	purple	134.5153	20.9029	0.891165	0.04239	37.896
gnl|UG|Ssc#S26722459	purple	256.0804	7.94638	0.491912	0.399945	9.386
gnl|UG|Ssc#S26722558	brown	214.2581	50.2933	0.777458	0.121729	10.466
gnl|UG|Ssc#S26723217	turquoise	906.0338	259.388	0.977973	0.003911	5.416
gnl|UG|Ssc#S26723327	green	90.32893	51.4868	0.922477	0.025607	38.488
gnl|UG|Ssc#S26723508	violet	929.4696	74.6499	0.997668	0.000135	0.318
gnl|UG|Ssc#S26723801	white	213.7105	23.3128	0.749021	0.145119	2.774
gnl|UG|Ssc#S26724122	lightgreen	295.9503	58.8905	0.991177	0.000993	2.874
gnl|UG|Ssc#S26724291	lightgreen	294.7712	61.2079	0.999103	3.22E-05	10.732
gnl|UG|Ssc#S26724425	lightgreen	274.7209	58.4935	0.989864	0.001223	1.8
gnl|UG|Ssc#S26724464	yellow	275.4974	93.0095	0.940282	0.017361	9.196
gnl|UG|Ssc#S26724489	blue	871.0065	206.515	0.942385	0.016457	15.504
gnl|UG|Ssc#S26724539	midnightblue	198.9259	40.2274	0.961864	0.008889	16.048
gnl|UG|Ssc#S26724832	black	960.3862	96.6752	0.993487	0.00063	13.646
gnl|UG|Ssc#S26725357	darkgreen	283.4798	29.8681	0.942136	0.016563	2.582
gnl|UG|Ssc#S26725439	yellow	306.5128	115.809	0.991155	0.000997	9.444
gnl|UG|Ssc#S26725722	brown	172.0088	37.1157	0.745391	0.14819	57.49
gnl|UG|Ssc#S26725970	red	383.5987	96.044	0.968726	0.006608	0.208
gnl|UG|Ssc#S26726120	brown	180.3678	57.2973	0.859436	0.061911	24.19
gnl|UG|Ssc#S26726211	darkred	258.5628	41.8433	0.982179	0.002848	7.868
gnl|UG|Ssc#S26726238	darkred	224.5731	31.6032	0.917649	0.028016	5.724
gnl|UG|Ssc#S26726300	darkturquoise	213.647	32.1157	0.974286	0.004931	1.278
gnl|UG|Ssc#S26726336	red	405.4812	97.955	0.973189	0.005249	1.802
gnl|UG|Ssc#S26726474	red	596.4439	84.2782	0.940463	0.017282	13.32
gnl|UG|Ssc#S26726517	grey60	906.7858	62.0335	0.994126	0.00054	2.552
gnl|UG|Ssc#S26726607	midnightblue	129.8146	27.3378	0.852795	0.066281	3.962
gnl|UG|Ssc#S26726690	turquoise	511.5338	134.173	0.788763	0.112778	2.632
gnl|UG|Ssc#S26726753	yellow	303.1722	116.88	0.993234	0.000667	1.776
gnl|UG|Ssc#S26727199	brown	146.9458	44.7824	0.854152	0.06538	7.526
gnl|UG|Ssc#S26727288	yellow	224.8157	69.5299	0.87945	0.049326	1.288
gnl|UG|Ssc#S26727794	lightgreen	306.0872	59.6707	0.993877	0.000575	6.976
gnl|UG|Ssc#S26727819	blue	896.0655	237.986	0.97523	0.004662	14.316
gnl|UG|Ssc#S26727906	white	174.3338	21.4636	0.725768	0.165115	5.776
gnl|UG|Ssc#S26727931	yellow	303.5753	117.957	0.996872	0.00021	2.89
gnl|UG|Ssc#S26728087	white	941.2102	29.4749	0.803757	0.10123	14.476
gnl|UG|Ssc#S26728282	magenta	226.2826	73.9175	0.975823	0.004496	7.472
gnl|UG|Ssc#S26728690	turquoise	433.7127	145.995	0.811035	0.095762	14.588
gnl|UG|Ssc#S26729898	yellow	308.2809	115.12	0.988672	0.001445	17.932
gnl|UG|Ssc#S26731491	turquoise	175.9891	47.85	0.510625	0.379328	52.868
gnl|UG|Ssc#S26731505	white	896.3377	29.0552	0.827275	0.083904	2.54
gnl|UG|Ssc#S26732538	yellowgreen	188.0926	17.6343	0.982003	0.00289	5.038
gnl|UG|Ssc#S26732732	yellow	294.9456	106.356	0.970215	0.006143	8.4
gnl|UG|Ssc#S26732741	greenyellow	238.0478	47.0763	0.954865	0.011433	118.052
gnl|UG|Ssc#S26733049	pink	82.53162	16.9298	0.46015	0.435507	10.636
gnl|UG|Ssc#S26733269	tan	660.9035	50.3053	0.93255	0.020815	59.864
gnl|UG|Ssc#S26733360	darkorange	158.2259	7.14669	0.266389	0.664878	2.244

gnl|UG|Ssc#S26733687	violet	960.7978	72.969	0.993161	0.000678	23.122
gnl|UG|Ssc#S26733977	black	865.5135	84.3526	0.966408	0.007354	19.6
gnl|UG|Ssc#S26734385	yellow	234.0636	77.1102	0.901358	0.036635	11.004
gnl|UG|Ssc#S26734441	turquoise	394.3916	132.098	0.780759	0.119095	42.366
gnl|UG|Ssc#S26734833	magenta	275.2895	76.6895	0.983832	0.002462	7.172
gnl|UG|Ssc#S26735067	pink	228.7544	69.3203	0.985368	0.00212	5.472
gnl|UG|Ssc#S26735228	blue	700.2607	207.813	0.945771	0.015035	7.47
gnl|UG|Ssc#S26735265	magenta	211.2756	62.5063	0.942014	0.016615	6.202
gnl|UG|Ssc#S26735527	green	170.2101	18.0808	0.024888	0.968314	3.32
gnl|UG|Ssc#S27598976	turquoise	98.03708	27.3723	0.330175	0.587377	0.678
gnl|UG|Ssc#S27599980	blue	821.5472	242.762	0.978992	0.003644	35.236
gnl|UG|Ssc#S27600018	turquoise	711.8718	213.403	0.93465	0.019856	21.078
gnl|UG|Ssc#S27600022	pink	241.2608	66.0009	0.969974	0.006218	26.236
gnl|UG|Ssc#S27600466	violet	993.1651	71.3896	0.988629	0.001453	57.024
gnl|UG|Ssc#S27600517	grey60	965.3017	61.1066	0.99109	0.001008	54.14
gnl|UG|Ssc#S27600920	lightyellow	941.3649	63.218	0.998976	3.93E-05	17.05
gnl|UG|Ssc#S27600966	black	923.3123	86.6365	0.971713	0.005687	54.968
gnl|UG|Ssc#S27601215	turquoise	378.5071	120.878	0.744436	0.149002	18.118
gnl|UG|Ssc#S27602031	lightyellow	988.5575	62.3047	0.995998	0.000304	4.494
gnl|UG|Ssc#S27602037	red	446.8265	106.448	0.991246	0.000982	26.994
gnl|UG|Ssc#S27602440	turquoise	754.723	227.985	0.943948	0.015795	27.744
gnl|UG|Ssc#S27604329	red	430.9721	87.0908	0.947458	0.014343	10.864
gnl|UG|Ssc#S29456864	turquoise	831.9026	258.9	0.955723	0.011109	8.582
gnl|UG|Ssc#S29456965	brown	78.46765	30.4764	0.710713	0.178451	140.122
gnl|UG|Ssc#S29459150	darkgrey	174.6356	30.5818	0.968694	0.006618	9.91
gnl|UG|Ssc#S29461992	turquoise	799.8677	253.289	0.945014	0.01535	202.002
gnl|UG|Ssc#S29462637	orange	441.9662	34.5965	0.991154	0.000997	59.002
gnl|UG|Ssc#S29465510	pink	147.8	43.9855	0.877893	0.050272	3537.64
gnl|UG|Ssc#S29974082	darkred	280.6116	43.0117	0.989191	0.001347	6.394
gnl|UG|Ssc#S29975107	yellow	287.8253	109.958	0.977169	0.004127	1.5
gnl|UG|Ssc#S29975173	violet	892.5988	73.012	0.993171	0.000677	14.282
gnl|UG|Ssc#S29978365	lightcyan	140.6026	8.74796	0.663642	0.221978	27.912
gnl|UG|Ssc#S29978506	blue	820.9021	239.974	0.978846	0.003682	28.286
gnl|UG|Ssc#S29978907	lightgreen	279.1075	56.6706	0.983257	0.002594	2.596
gnl|UG|Ssc#S29979645	turquoise	278.5004	75.4266	0.590664	0.294308	4.344
gnl|UG|Ssc#S29979787	magenta	246.8134	77.5231	0.985787	0.00203	4.09
gnl|UG|Ssc#S29980641	yellow	296.6656	114.563	0.99063	0.001087	0.834
gnl|UG|Ssc#S29981643	turquoise	466.1095	112.109	0.723058	0.167494	61.202
gnl|UG|Ssc#S29983891	turquoise	412.6119	89.5117	0.703541	0.184908	9.934
gnl|UG|Ssc#S29984324	darkred	212.529	37.8656	0.947639	0.014269	121.736
gnl|UG|Ssc#S29988216	turquoise	472.5575	157.741	0.814865	0.092923	54.938
gnl|UG|Ssc#S29988897	salmon	181.5176	33.5321	0.884874	0.046073	12.456
gnl|UG|Ssc#S29989732	brown	203.4121	26.0973	0.569807	0.315935	69.002
gnl|UG|Ssc#S29989765	blue	653.8764	201.606	0.935593	0.019431	15.504
gnl|UG|Ssc#S29989903	salmon	145.5046	36.1715	0.90468	0.034818	13.042
gnl|UG|Ssc#S29990179	red	709.0695	95.0023	0.965042	0.007805	14.768
gnl|UG|Ssc#S31101544	brown	231.0961	19.9365	0.451558	0.445244	116.304
gnl|UG|Ssc#S31101703	skyblue	253.54	31.6862	0.990419	0.001124	17.69

gnl|UG|Ssc#S31101896	blue	819.9466	193.235	0.924578	0.024581	45.196
gnl|UG|Ssc#S31101963	turquoise	625.0038	199.044	0.886753	0.044963	4.286
gnl|UG|Ssc#S31102048	brown	94.29746	28.9709	0.642769	0.242099	4.792
gnl|UG|Ssc#S31102193	lightgreen	301.7578	58.372	0.989349	0.001317	2.832
gnl|UG|Ssc#S31102306	lightyellow	838.6326	58.9372	0.98483	0.002238	7.842
gnl|UG|Ssc#S31102841	darkgreen	267.3822	33.8002	0.954361	0.011624	1.53
gnl|UG|Ssc#S31102878	purple	224.5293	17.3501	-0.63376	0.250929	1.158
gnl|UG|Ssc#S31103021	yellow	309.9835	117.805	0.994847	0.000444	2.494
gnl|UG|Ssc#S31103335	darkred	255.3849	18.3564	0.784788	0.115902	17.284
gnl|UG|Ssc#S31104316	turquoise	515.3315	111.563	0.745324	0.148248	10.326
gnl|UG|Ssc#S31105561	salmon	157.1084	47.448	0.974617	0.004836	16.318
gnl|UG|Ssc#S31106386	blue	773.1439	170.267	0.896168	0.039532	12.53
gnl|UG|Ssc#S31106760	green	103.1718	37.6396	0.837047	0.077005	19.748
gnl|UG|Ssc#S31106968	navy	155.569	36.175	0.940866	0.017108	27.204
gnl|UG|Ssc#S31107252	sienna3	885.9463	24.8686	0.990368	0.001133	11.92
gnl|UG|Ssc#S31107413	sienna3	974.6493	25.9924	0.999026	3.65E-05	4.628
gnl|UG|Ssc#S31107757	red	367.5818	90.0479	0.954672	0.011506	56.602
gnl|UG|Ssc#S31110463	orange	650.0858	30.3804	0.962648	0.008617	5.164
gnl|UG|Ssc#S31111456	darkgreen	295.2207	34.8216	0.974724	0.004806	6.89
gnl|UG|Ssc#S31112286	darkturquoise	155.4227	22.9206	0.879125	0.049522	0.944
gnl|UG|Ssc#S31113853	black	870.1043	91.8726	0.983285	0.002588	26.368
gnl|UG|Ssc#S31114103	green	96.96647	39.7972	0.826904	0.084169	86.898
gnl|UG|Ssc#S31114178	turquoise	387.961	92.4965	0.670056	0.215892	17.474
gnl|UG|Ssc#S31114666	skyblue	236.4502	30.1751	0.978283	0.003829	20.854
gnl|UG|Ssc#S31115183	paleturquoise	219.8711	17.253	0.91314	0.030326	16.224
gnl|UG|Ssc#S31116110	darkgreen	277.0545	33.0915	0.955773	0.011091	8.528
gnl|UG|Ssc#S31116114	brown	45.67899	14.8572	0.442671	0.455365	49.594
gnl|UG|Ssc#S31116402	lightcyan	180.485	26.0683	0.869268	0.055616	14.596
gnl|UG|Ssc#S31116518	darkgrey	189.8588	34.9977	0.997247	0.000173	14.844
gnl|UG|Ssc#S31117514	green	87.48882	38.8553	0.828647	0.082924	2.19
gnl|UG|Ssc#S31117548	midnightblue	132.0562	43.0904	0.97855	0.003759	3.24
gnl|UG|Ssc#S31118802	blue	710.0202	186.973	0.922654	0.02552	12.272
gnl|UG|Ssc#S31119321	yellow	305.1557	115.861	0.990529	0.001105	7.134
gnl|UG|Ssc#S31119453	yellow	263.5425	89.6116	0.93166	0.021225	22.9
gnl|UG|Ssc#S31120438	grey	278.0216	11.0733	0.280837	0.647185	16.044
gnl|UG|Ssc#S31120524	darkgreen	259.2622	22.1274	0.845895	0.070918	3.904
gnl|UG|Ssc#S31120797	magenta	297.2288	51.1241	0.893514	0.04104	11.138
gnl|UG|Ssc#S31120805	turquoise	743.053	233.526	0.95109	0.012889	4.288
gnl|UG|Ssc#S31121045	midnightblue	127.8585	43.1335	0.975759	0.004514	359.504
gnl|UG|Ssc#S31121529	darkgreen	249.3967	34.2625	0.953966	0.011774	26.146
gnl|UG|Ssc#S31122375	lightgreen	290.8026	59.0561	0.991858	0.000881	2.356
gnl|UG|Ssc#S31122377	darkred	211.3632	12.7877	0.688287	0.198856	25.18
gnl|UG|Ssc#S31122485	darkgrey	175.723	30.4726	0.964775	0.007894	18.246
gnl|UG|Ssc#S31123459	brown	221.1788	44.661	0.747511	0.146394	10.346
gnl|UG|Ssc#S31123943	black	980.1157	99.1379	0.999054	3.49E-05	6.012
gnl|UG|Ssc#S31124004	royalblue	123.3307	1.39659	-0.24315	0.693494	44.904
gnl|UG|Ssc#S31124539	turquoise	276.5324	88.0642	0.622339	0.262246	11.722
gnl|UG|Ssc#S31124838	turquoise	634.3644	177.469	0.87902	0.049586	1.808

gnl|UG|Ssc#S31125030	paleturquoise	99.68591	6.98786	0.732337	0.15939	9.088
gnl|UG|Ssc#S31125151	turquoise	887.0193	264.507	0.978893	0.003669	38.376
gnl|UG|Ssc#S31129394	black	938.7055	97.5624	0.996156	0.000286	16.642
gnl|UG|Ssc#S31131447	turquoise	593.7216	151.73	0.818482	0.090265	15.338
gnl|UG|Ssc#S31133289	lightgreen	283.8522	60.4054	0.99641	0.000258	0.252
gnl|UG|Ssc#S31133660	greenyellow	185.5109	33.1824	0.869237	0.055635	15.784
gnl|UG|Ssc#S31133723	lightcyan	107.7592	10.5353	0.682688	0.204046	183.51
gnl|UG|Ssc#S31133867	green	91.93454	13.0798	0.37033	0.539492	1.144
gnl|UG|Ssc#S31133980	pink	91.8409	15.5846	0.171614	0.782572	6.454
gnl|UG|Ssc#S31986337	brown	179.4854	57.1034	0.940301	0.017352	42.794
gnl|UG|Ssc#S32382339	blue	790.6034	230.918	0.965706	0.007584	3.092
gnl|UG|Ssc#S32389412	brown	83.43805	35.7001	0.735027	0.157063	2.83
gnl|UG|Ssc#S32419644	midnightblue	140.8034	46.0205	0.993667	0.000604	399.53
gnl|UG|Ssc#S32771805	brown	221.7502	61.5938	0.950811	0.012999	10.662
gnl|UG|Ssc#S32800726	midnightblue	153.8628	36.3522	0.948967	0.013733	7.542
gnl|UG|Ssc#S32800732	navy	202.0015	9.90008	-0.66794	0.217895	98.376
gnl|UG|Ssc#S34494848	white	165.7491	20.4031	0.71982	0.170349	2.312
gnl|UG|Ssc#S34495212	blue	685.974	210.298	0.945838	0.015008	2.838
gnl|UG|Ssc#S34495586	magenta	190.6629	60.5537	0.932983	0.020616	3.526
gnl|UG|Ssc#S34496141	greenyellow	224.3289	46.582	0.957298	0.010525	1.19
gnl|UG|Ssc#S34497940	yellow	235.4245	77.7632	0.899161	0.037853	5.032
gnl|UG|Ssc#S34497945	darkturquoise	194.1598	34.8629	0.994658	0.000468	3.668
gnl|UG|Ssc#S34499792	lightgreen	288.7002	59.9329	0.994785	0.000452	2.11
gnl|UG|Ssc#S34500484	yellow	258.7949	93.2459	0.940919	0.017085	5.348
gnl|UG|Ssc#S34501706	midnightblue	210.2888	37.5196	0.937975	0.01837	54.564
gnl|UG|Ssc#S34501858	turquoise	153.4636	39.552	0.42515	0.475463	1.758
gnl|UG|Ssc#S34502088	tan	971.94	68.0118	0.995577	0.000353	25.91
gnl|UG|Ssc#S34504168	purple	161.5956	13.9757	-0.81019	0.096396	39.532
gnl|UG|Ssc#S34507193	yellow	187.3276	59.1244	0.824359	0.085997	1.652
gnl|UG|Ssc#S34507225	darkred	262.8989	45.2166	0.998256	8.74E-05	2.408
gnl|UG|Ssc#S34507478	pink	88.03299	16.7447	0.259017	0.673935	11.03
gnl|UG|Ssc#S34508066	brown	177.4394	47.4628	0.782218	0.117936	8.276
gnl|UG|Ssc#S34509005	paleturquoise	164.4035	15.6428	0.882283	0.047618	3.546
gnl|UG|Ssc#S34509766	midnightblue	183.7956	37.6607	0.947985	0.014129	52.672
gnl|UG|Ssc#S34509960	lightgreen	294.9034	60.7937	0.997707	0.000132	28.15
gnl|UG|Ssc#S34510245	salmon	384.3988	35.4125	0.896811	0.039169	46.672
gnl|UG|Ssc#S34511316	yellow	241.3126	85.0673	0.915278	0.029223	5.978
gnl|UG|Ssc#S34511666	blue	720.3936	219.611	0.953873	0.01181	16.588
gnl|UG|Ssc#S34512569	turquoise	279.5379	95.9684	0.671558	0.214474	26.752
gnl|UG|Ssc#S34512723	pink	254.4123	74.3373	0.996513	0.000247	5.968
gnl|UG|Ssc#S34512833	greenyellow	172.908	52.6377	0.987255	0.001724	17.978
gnl|UG|Ssc#S34512837	blue	609.873	171.552	0.904584	0.03487	3.504
gnl|UG|Ssc#S34513996	blue	757.5638	223.934	0.957081	0.010605	34.608
gnl|UG|Ssc#S34516183	brown	240.0799	56.4866	0.882093	0.047732	6.936
gnl|UG|Ssc#S34516266	grey60	926.6777	61.4843	0.99241	0.000793	10.68
gnl|UG|Ssc#S34516411	royalblue	151.4064	2.6849	0.179099	0.77319	13.76
gnl|UG|Ssc#S34517157	greenyellow	216.7802	49.3505	0.966615	0.007286	5.716
gnl|UG|Ssc#S34517367	navy	189.6252	33.2205	0.889497	0.043357	48.486

gnl|UG|Ssc#S34517412	turquoise	634.7996	177.73	0.864956	0.058351	30.182
gnl|UG|Ssc#S34517549	yellow	287.0104	107.483	0.972293	0.005513	12.152
gnl|UG|Ssc#S34517830	turquoise	480.3762	104.557	0.739882	0.152888	11.002
gnl|UG|Ssc#S34518344	midnightblue	199.3508	39.6594	0.958397	0.010123	5.94
gnl|UG|Ssc#S34521601	blue	620.1669	146.251	0.86171	0.060437	29.364
gnl|UG|Ssc#S34521876	green	138.4643	20.9194	0.230224	0.709481	8.104
gnl|UG|Ssc#S34521911	purple	176.9354	14.1152	-0.66826	0.217587	3.936
gnl|UG|Ssc#S34524449	yellow	302.7561	111.57	0.980134	0.003351	7.85
gnl|UG|Ssc#S34527542	yellow	306.1823	118.303	0.996223	0.000279	9.302
gnl|UG|Ssc#S34527926	purple	55.12121	11.0837	0.642902	0.24197	36.13
gnl|UG|Ssc#S34528484	white	187.2001	21.3929	0.666056	0.219682	3.986
gnl|UG|Ssc#S34528527	yellowgreen	204.6885	16.1232	0.963733	0.008245	52.902
gnl|UG|Ssc#S34529071	darkturquoise	203.7191	34.6316	0.993971	0.000561	2.208
gnl|UG|Ssc#S34529284	magenta	240.6062	75.2678	0.979647	0.003475	38.758
gnl|UG|Ssc#S34529469	turquoise	553.4605	128.663	0.789353	0.112316	7.304
gnl|UG|Ssc#S34529519	darkgreen	231.0326	16.2437	0.732387	0.159347	2.198
gnl|UG|Ssc#S34530765	blue	795.1638	235.727	0.971827	0.005652	11.952
gnl|UG|Ssc#S34530890	brown	88.82947	31.9919	0.639653	0.245144	99.448
gnl|UG|Ssc#S34531282	purple	209.0827	16.0185	-0.80342	0.101487	16.204
gnl|UG|Ssc#S34531946	lightgreen	299.6601	60.9994	0.998358	7.99E-05	1.3
gnl|UG|Ssc#S34532310	paleturquoise	152.4686	15.8573	0.888202	0.044113	2.92
gnl|UG|Ssc#S34534055	black	978.5305	98.202	0.997196	0.000178	22.07
gnl|UG|Ssc#S35167355	pink	284.4324	71.0961	0.969344	0.006414	10.614
gnl|UG|Ssc#S35167435	darkgrey	226.3774	28.4253	0.950971	0.012936	3.898
gnl|UG|Ssc#S35167565	magenta	208.3046	66.2459	0.95204	0.012517	20.37
gnl|UG|Ssc#S35169073	greenyellow	208.0021	39.9514	0.916887	0.028402	5.798
gnl|UG|Ssc#S35169280	brown	123.1408	37.2247	0.845453	0.071218	14.2
gnl|UG|Ssc#S35171904	blue	886.8388	196.94	0.934286	0.020021	132.418
gnl|UG|Ssc#S35173121	blue	904.0127	254.412	0.992297	0.000811	5.128
gnl|UG|Ssc#S35173188	salmon	125.5069	36.8783	0.913777	0.029996	5.85
gnl|UG|Ssc#S35173683	greenyellow	170.1435	38.918	0.908149	0.032952	35.368
gnl|UG|Ssc#S35321865	greenyellow	178.1697	46.8744	0.95923	0.009821	57.066
gnl|UG|Ssc#S35322082	midnightblue	75.5719	26.8853	0.877739	0.050366	61.242
gnl|UG|Ssc#S35322980	yellow	301.2055	109.332	0.975036	0.004717	5.53
gnl|UG|Ssc#S35323577	brown	203.1814	60.3555	0.847482	0.069843	38.184
gnl|UG|Ssc#S35323988	darkturquoise	197.661	34.4044	0.992016	0.000855	6.808
gnl|UG|Ssc#S35324071	green	91.49753	43.7864	0.871202	0.054403	6.018
gnl|UG|Ssc#S35324483	tan	902.2321	50.0961	0.934914	0.019737	3.832
gnl|UG|Ssc#S35325248	grey60	963.7581	62.3711	0.995226	0.000396	3.808
gnl|UG|Ssc#S35325791	darkturquoise	181.9611	29.6969	0.955091	0.011347	14.416
gnl|UG|Ssc#S35326006	darkturquoise	177.1159	24.2878	0.89815	0.038417	18.568
gnl|UG|Ssc#S35326333	turquoise	711.2042	223.339	0.91192	0.030962	13.14
gnl|UG|Ssc#S35326360	purple	224.4929	20.0592	-0.75241	0.142271	4.658
gnl|UG|Ssc#S35326767	violet	952.7028	73.9402	0.995788	0.000328	11.086
gnl|UG|Ssc#S35327002	midnightblue	121.2679	30.1973	0.884835	0.046096	13.03
gnl|UG|Ssc#S35327790	white	944.3869	29.7335	0.831677	0.080773	3.422
gnl|UG|Ssc#S35328709	turquoise	606.4171	161.29	0.837129	0.076948	15.332
gnl|UG|Ssc#S35329402	yellowgreen	187.8607	15.8187	0.958969	0.009915	333.972

gnl|UG|Ssc#S35329662	yellow	231.443	77.4348	0.897695	0.038672	7.6
gnl|UG|Ssc#S35330371	pink	147.338	11.527	0.48376	0.409005	15.256
gnl|UG|Ssc#S35331200	greenyellow	176.824	33.6098	0.876874	0.050894	37.008
gnl|UG|Ssc#S35331259	purple	173.9167	16.2623	-0.87683	0.050921	10.16
gnl|UG|Ssc#S35332512	greenyellow	193.6512	53.8785	0.990832	0.001052	4.73
gnl|UG|Ssc#S35332920	black	649.8045	65.9867	0.917051	0.028319	3.366
gnl|UG|Ssc#S35332961	darkturquoise	167.0211	22.2636	0.880796	0.048512	3.808
gnl|UG|Ssc#S35333026	yellowgreen	131.3336	10.3514	0.86214	0.060159	17.464
gnl|UG|Ssc#S35333447	black	956.6357	82.4975	0.961441	0.009036	2.024
gnl|UG|Ssc#S35334154	violet	820.9608	68.3984	0.979935	0.003402	7.19
gnl|UG|Ssc#S35334793	tan	946.7173	67.4805	0.993783	0.000588	50.986
gnl|UG|Ssc#S35337111	greenyellow	234.3855	39.9619	0.917491	0.028095	13.014
gnl|UG|Ssc#S35722094	brown	135.1209	44.1401	0.85336	0.065905	52.126
gnl|UG|Ssc#S35722541	lightgreen	293.0922	60.2522	0.995904	0.000314	0.978
gnl|UG|Ssc#S35723730	turquoise	637.15	149.863	0.825501	0.085176	26.526
gnl|UG|Ssc#S38479714	yellow	251.7269	89.5784	0.928091	0.022897	3.224
gnl|UG|Ssc#S38479741	salmon	130.4519	39.8499	0.931103	0.021483	3.936
gnl|UG|Ssc#S38479786	blue	912.3716	234.814	0.971985	0.005605	60.226
gnl|UG|Ssc#S38481918	pink	197.7624	3.3094	-0.87231	0.053713	20.198
gnl|UG|Ssc#S38482154	magenta	178.6893	55.6808	0.916202	0.028751	6.522
gnl|UG|Ssc#S38482329	blue	726.6445	141.883	0.861324	0.060686	26.004
gnl|UG|Ssc#S39762052	magenta	270.2545	81.0048	0.99605	0.000298	5.576
gnl|UG|Ssc#S39765406	red	534.0255	109.18	0.996669	0.000231	1.434
gnl|UG|Ssc#S39769221	purple	252.8571	16.438	-0.64155	0.243288	11.42
gnl|UG|Ssc#S39772429	red	619.7867	94.6898	0.966136	0.007443	31.94
gnl|UG|Ssc#S39773791	green	121.4672	45.9056	0.905201	0.034535	209.562
gnl|UG|Ssc#S39776911	midnightblue	171.2048	38.2785	0.937578	0.018545	43.092
gnl|UG|Ssc#S39777926	darkorange	119.6775	18.536	0.910402	0.031758	499.754
gnl|UG|Ssc#S39778104	pink	203.0373	60.6394	0.958975	0.009913	45.564
gnl|UG|Ssc#S39778806	blue	942.0153	216.696	0.95454	0.011556	19.222
gnl|UG|Ssc#S39781881	turquoise	975.4983	269.366	0.999052	3.50E-05	31.762
gnl|UG|Ssc#S39785088	pink	225.9812	67.617	0.979734	0.003453	15.99
gnl|UG|Ssc#S39789448	yellowgreen	190.6921	17.0706	0.978858	0.003678	1.628
gnl|UG|Ssc#S39791419	skyblue	284.3206	31.1351	0.986946	0.001787	3.454
gnl|UG|Ssc#S39793738	darkturquoise	192.0136	26.8795	0.928233	0.022829	1.836
gnl|UG|Ssc#S39794612	grey60	946.7049	61.5734	0.992646	0.000756	15.858
gnl|UG|Ssc#S39794773	orange	527.0395	31.024	0.966417	0.00735	229.574
gnl|UG|Ssc#S39796976	tan	976.4411	66.3166	0.989797	0.001235	8.206
gnl|UG|Ssc#S39797758	green	130.7919	49.408	0.929528	0.022219	6.962
gnl|UG|Ssc#S39801969	grey60	991.6185	59.469	0.985453	0.002101	15.488
gnl|UG|Ssc#S39803451	red	596.2598	90.6615	0.95469	0.011499	7.282
gnl|UG|Ssc#S39805483	lightcyan	262.0721	34.6038	0.953058	0.012123	21.486
gnl|UG|Ssc#S39808414	pink	279.1731	73.8233	0.984242	0.002369	5.34
gnl|UG|Ssc#S39812694	green	135.8693	19.8159	0.147907	0.812368	1.704
gnl|UG|Ssc#S39833372	tan	950.836	65.0015	0.986067	0.00197	11.446
gnl|UG|Ssc#S39833392	turquoise	683.5968	225.525	0.898765	0.038073	22.98
gnl|UG|Ssc#S39835484	pink	199.818	58.0342	0.951941	0.012556	10.286
gnl|UG|Ssc#S39835999	blue	678.8467	150.519	0.873436	0.053012	1.146

gnl|UG|Ssc#S39836829	turquoise	818.1038	237.515	0.952585	0.012305	73.178
gnl|UG|Ssc#S39836895	brown	151.8202	18.411	0.552161	0.334534	30.026
gnl|UG|Ssc#S39838237	orange	217.0726	27.237	0.935977	0.019258	31.512
gnl|UG|Ssc#S39839709	turquoise	503.1783	168.279	0.850238	0.067988	9.976
gnl|UG|Ssc#S39839769	turquoise	906.3585	259.931	0.972776	0.00537	4.58
gnl|UG|Ssc#S39840475	greenyellow	167.7066	44.6873	0.946578	0.014703	7.566
gnl|UG|Ssc#S39841126	violet	929.4696	74.6499	0.997668	0.000135	3.624
gnl|UG|Ssc#S39841622	brown	226.8707	21.4342	0.453099	0.443493	16.756
gnl|UG|Ssc#S39842447	turquoise	380.579	126.276	0.783247	0.11712	173.914
gnl|UG|Ssc#S39842527	magenta	249.6542	76.9568	0.984224	0.002373	7.066
gnl|UG|Ssc#S39844514	turquoise	779.9832	233.647	0.954182	0.011692	13.246
gnl|UG|Ssc#S39858087	turquoise	792.4283	253.897	0.947864	0.014178	30.932
gnl|UG|Ssc#S39861498	lightcyan	307.6268	42.4946	0.973523	0.005151	310.304
gnl|UG|Ssc#S39863250	blue	792.7405	222.865	0.96059	0.009336	4.552
gnl|UG|Ssc#S39866181	green	65.22481	34.235	0.771182	0.126786	2.06
gnl|UG|Ssc#S39871294	purple	224.7477	12.6171	-0.59707	0.287746	2.4
gnl|UG|Ssc#S39883528	turquoise	383.3612	87.1414	0.686237	0.200752	27.73
gnl|UG|Ssc#S39886474	darkred	284.115	43.9384	0.993642	0.000608	3.76
gnl|UG|Ssc#S39888860	turquoise	542.0468	157.14	0.823837	0.086374	19.186
gnl|UG|Ssc#S39889520	red	713.2749	95.4905	0.96597	0.007497	105.844
gnl|UG|Ssc#S39891101	midnightblue	121.6167	28.857	0.871544	0.054189	19.574
gnl|UG|Ssc#S39891679	navy	135.7055	42.5713	0.94988	0.013368	8.08
gnl|UG|Ssc#S39892044	turquoise	694.484	228.504	0.902358	0.036085	9.482
gnl|UG|Ssc#S39892248	lightgreen	258.0027	53.5481	0.971914	0.005626	10.304
gnl|UG|Ssc#S39929154	lightgreen	275.619	55.3653	0.978687	0.003723	9.202
gnl|UG|Ssc#S39943227	paleturquoise	151.8686	14.4228	0.87336	0.053059	5.828
gnl|UG|Ssc#S39943772	lightgreen	306.2232	59.8377	0.994451	0.000496	1.862
gnl|UG|Ssc#S39944894	darkred	241.632	44.3217	0.991825	0.000886	6.706
gnl|UG|Ssc#S39946921	orange	245.5991	29.1631	0.954676	0.011504	106.666
gnl|UG|Ssc#S39947174	green	191.4758	16.6121	0.132832	0.831371	3.75
gnl|UG|Ssc#S39950607	darkturquoise	169.8191	32.6573	0.97703	0.004164	0.85
gnl|UG|Ssc#S39951148	darkred	272.0494	42.5935	0.98609	0.001965	4.446
gnl|UG|Ssc#S39951768	navy	268.5991	26.7184	0.732999	0.158817	11.606
gnl|UG|Ssc#S39954477	grey	156.1286	14.8495	-0.94742	0.014357	5.69
gnl|UG|Ssc#S39957439	darkgrey	182.9559	1.26996	0.18041	0.771548	10.858
gnl|UG|Ssc#S39958727	darkgrey	216.1391	1.50605	0.253916	0.680212	13.712
gnl|UG|Ssc#S39959416	blue	595.3346	162.689	0.892199	0.041794	7.174
gnl|UG|Ssc#S39961595	blue	862.2	225.494	0.96441	0.008017	11.134
gnl|UG|Ssc#S39969872	yellow	297.6665	106.19	0.970332	0.006107	53.674
gnl|UG|Ssc#S39970588	midnightblue	91.95082	5.03951	-0.49563	0.395828	7.47
gnl|UG|Ssc#S39979082	blue	910.0972	228.784	0.964372	0.00803	32.234
gnl|UG|Ssc#S39979866	green	118.5485	56.3462	0.966339	0.007376	48.794
gnl|UG|Ssc#S39980362	darkgrey	154.982	23.9605	0.91632	0.028691	1.652
gnl|UG|Ssc#S39982552	midnightblue	130.564	37.8824	0.939859	0.017544	2343.14
gnl|UG|Ssc#S39984038	red	475.4838	101.101	0.979654	0.003473	40.432
gnl|UG|Ssc#S39984272	turquoise	497.688	166.453	0.833353	0.079591	9.624
gnl|UG|Ssc#S39984310	pink	264.5653	75.5496	0.998359	7.98E-05	0.078
gnl|UG|Ssc#S39984579	brown	151.3436	38.5216	0.728348	0.162859	1.998

gnl|UG|Ssc#S39991834	green	113.6992	46.7559	0.900585	0.037062	18.764
gnl|UG|Ssc#S39993494	darkorange	162.6142	9.31265	0.548256	0.338686	43.832
gnl|UG|Ssc#S39994120	brown	238.2651	20.282	0.500311	0.390659	13.306
gnl|UG|Ssc#S39995034	blue	712.2522	216.652	0.951518	0.012721	21.484
gnl|UG|Ssc#S39996844	brown	230.7681	52.9899	0.833236	0.079673	35.938
gnl|UG|Ssc#S39998732	blue	864.0924	247.475	0.982936	0.002669	6.786
gnl|UG|Ssc#S39998964	pink	275.2707	70.7803	0.975092	0.004701	2.818
gnl|UG|Ssc#S39998988	blue	879.0023	211.421	0.946033	0.014927	1232.612
gnl|UG|Ssc#S40003052	white	960.8431	28.7009	0.810678	0.096029	31.808
gnl|UG|Ssc#S40004648	blue	809.6943	214.213	0.946305	0.014815	21.434
gnl|UG|Ssc#S40006091	darkturquoise	187.2181	32.4511	0.973321	0.00521	8.362
gnl|UG|Ssc#S40016934	turquoise	721.3704	229.28	0.90906	0.032467	2.962
gnl|UG|Ssc#S40017556	brown	225.2884	61.0028	0.89206	0.041874	1.828
gnl|UG|Ssc#S40020159	turquoise	703.729	230.91	0.908138	0.032958	477.274
gnl|UG|Ssc#S40024674	pink	264.4305	69.5841	0.974196	0.004957	10.714
gnl|UG|Ssc#S40030592	lightyellow	995.6453	62.3941	0.996277	0.000273	12.456
gnl|UG|Ssc#S40034435	lightgreen	292.2049	58.94	0.991325	0.000969	3.156
gnl|UG|Ssc#S40034650	turquoise	823.7525	259.912	0.957613	0.010409	20.04
gnl|UG|Ssc#S40037863	grey	88.2473	8.17219	0.040937	0.947892	47.162
gnl|UG|Ssc#S40038955	darkmagenta	974.7107	26.1385	0.986924	0.001791	477.792
gnl|UG|Ssc#S40042081	magenta	269.657	79.8187	0.992527	0.000775	3.846
gnl|UG|Ssc#S40042631	skyblue	362.6148	27.3763	0.959866	0.009593	16.734
gnl|UG|Ssc#S40043958	lightyellow	951.9098	63.2435	0.999063	3.44E-05	10.402
gnl|UG|Ssc#S40045135	skyblue	180.8773	28.5425	0.97089	0.005936	7.794
gnl|UG|Ssc#S40045387	salmon	189.3859	27.7234	0.837171	0.076918	20.494
gnl|UG|Ssc#S40048435	lightgreen	302.064	60.0087	0.995047	0.000418	4.352
gnl|UG|Ssc#S40051540	brown	191.7803	57.1047	0.827499	0.083744	5.566
gnl|UG|Ssc#S40053273	violet	937.5565	74.8492	0.998218	9.03E-05	13.494
gnl|UG|Ssc#S40053418	lightyellow	931.819	62.9438	0.998105	9.90E-05	11.978
gnl|UG|Ssc#S40055771	lightgreen	291.1002	58.2112	0.988811	0.001418	5.282
gnl|UG|Ssc#S40056037	midnightblue	132.3563	37.9473	0.941245	0.016945	23.94
gnl|UG|Ssc#S40060840	darkgrey	183.0089	34.2125	0.992309	0.000809	9.066
gnl|UG|Ssc#S40064815	grey	133.6754	12.5247	0.155865	0.802353	3.218
gnl|UG|Ssc#S40064881	lightcyan	165.0676	11.843	0.422286	0.478766	2.412
gnl|UG|Ssc#S40066476	purple	213.7565	18.1884	0.757459	0.138052	41.524
gnl|UG|Ssc#S40068459	grey	180.4566	8.14983	0.451176	0.445678	18.954
gnl|UG|Ssc#S40069269	skyblue	256.5053	30.9921	0.987984	0.001578	57.792
gnl|UG|Ssc#S40070005	magenta	268.122	78.2753	0.989333	0.00132	10.326
gnl|UG|Ssc#S40072585	darkgrey	191.748	34.9655	0.996489	0.00025	6.556
gnl|UG|Ssc#S40073180	navy	127.0726	37.413	0.904477	0.034928	31.682
gnl|UG|Ssc#S40074206	turquoise	658.4103	217.93	0.889745	0.043213	3.334
gnl|UG|Ssc#S40077180	pink	280.7104	68.7258	0.963354	0.008375	8.07
gnl|UG|Ssc#S40079260	darkgrey	179.4654	31.707	0.97478	0.00479	1.172
gnl|UG|Ssc#S40081361	brown	180.3054	20.7318	0.617464	0.267116	9.336
gnl|UG|Ssc#S40081684	red	686.1551	88.4297	0.949087	0.013685	1.022
gnl|UG|Ssc#S40082945	black	927.9251	85.9188	0.970366	0.006097	7.034
gnl|UG|Ssc#S40084406	turquoise	823.9894	258.533	0.957528	0.01044	8.506
gnl|UG|Ssc#S40084794	orange	248.4084	29.012	0.948873	0.013771	4.096

gnl|UG|Ssc#S40087294	green	112.5104	44.8805	0.899306	0.037772	2.448
gnl|UG|Ssc#S40091442	brown	117.2646	10.1212	0.510191	0.379803	2.218
gnl|UG|Ssc#S40094285	turquoise	519.4333	175.364	0.828255	0.083204	8.262
gnl|UG|Ssc#S40094995	blue	799.9184	233.274	0.972041	0.005588	5.378
gnl|UG|Ssc#S40095300	magenta	228.7786	73.6686	0.97607	0.004428	5.154
gnl|UG|Ssc#S40095611	lightyellow	972.176	63.5058	0.999878	1.62E-06	6.906
gnl|UG|Ssc#S40096291	yellow	270.7602	99.9817	0.957922	0.010296	9.93
gnl|UG|Ssc#S40096829	greenyellow	167.647	42.2884	0.934157	0.02008	19.734
gnl|UG|Ssc#S40097075	brown	197.1947	58.1295	0.817045	0.091319	3.264
gnl|UG|Ssc#S40097726	lightcyan	185.4682	13.3017	0.565952	0.319976	2.116
gnl|UG|Ssc#S40098777	brown	257.9111	22.8381	0.611188	0.273421	13.236
gnl|UG|Ssc#S40099921	darkgrey	199.2988	31.9803	0.976027	0.00444	3.102
gnl|UG|Ssc#S40100350	brown	106.2904	37.7609	0.860953	0.060926	1.83
gnl|UG|Ssc#S40100454	pink	270.5845	73.3617	0.983931	0.002439	52.578
gnl|UG|Ssc#S40101480	brown	200.357	57.0705	0.864294	0.058775	2.014
gnl|UG|Ssc#S40103354	brown	125.4299	43.8102	0.887989	0.044237	50.35
gnl|UG|Ssc#S40104089	red	532.4573	96.4782	0.968776	0.006592	33.782
gnl|UG|Ssc#S40106980	magenta	270.7098	79.867	0.99343	0.000639	2.862
gnl|UG|Ssc#S40107769	greenyellow	194.255	48.7709	0.970779	0.00597	47.048
gnl|UG|Ssc#S40108865	purple	158.0998	20.0019	0.859791	0.061681	72.02
gnl|UG|Ssc#S40109312	brown	203.2752	61.2514	0.886311	0.045223	1.37
gnl|UG|Ssc#S40110548	pink	280.5678	70.651	0.971009	0.0059	7.464
gnl|UG|Ssc#S40110625	blue	800.6276	225.01	0.95884	0.009962	34.162
gnl|UG|Ssc#S40110683	pink	277.8142	53.8033	0.889225	0.043516	12.094
gnl|UG|Ssc#S40110774	lightcyan	168.5738	24.2165	0.843423	0.072603	8.188
gnl|UG|Ssc#S40111130	magenta	248.66	79.4091	0.991237	0.000983	4.032
gnl|UG|Ssc#S40117649	pink	286.5827	68.3241	0.95438	0.011617	12.958
gnl|UG|Ssc#S40118942	green	100.2479	41.1071	0.878249	0.050055	18.554
gnl|UG|Ssc#S40121987	green	158.3367	19.2833	0.054486	0.930661	0.682
gnl|UG|Ssc#S40127774	darkgrey	185.2413	33.3608	0.985571	0.002076	24.794
gnl|UG|Ssc#S40130919	turquoise	301.7529	99.6647	0.67864	0.207822	36.45
gnl|UG|Ssc#S40131166	purple	83.08105	14.6468	0.717345	0.17254	2.42
gnl|UG|Ssc#S40140172	magenta	268.9804	78.1733	0.987766	0.001621	9.29
gnl|UG|Ssc#S40141829	navy	112.0456	34.7298	0.866079	0.057635	241.138
gnl|UG|Ssc#S40142099	green	128.7579	36.7676	0.825452	0.085211	5.906
gnl|UG|Ssc#S40143536	green	109.8195	41.2052	0.856154	0.064059	73.064
gnl|UG|Ssc#S40143544	magenta	207.4227	66.6749	0.952309	0.012413	35.978
gnl|UG|Ssc#S40144447	brown	188.1969	57.4242	0.863083	0.059552	18.418
gnl|UG|Ssc#S40144457	red	525.0579	106.059	0.990126	0.001176	30.402
gnl|UG|Ssc#S40144865	tan	623.8899	48.1587	0.922757	0.02547	4.008
gnl|UG|Ssc#S40145713	green	139.8448	50.6424	0.937047	0.018781	7.96
gnl|UG|Ssc#S40146561	midnightblue	158.3892	33.1631	0.909621	0.032171	144.026
gnl|UG|Ssc#S40146833	greenyellow	156.2929	37.0362	0.899209	0.037826	62.798
gnl|UG|Ssc#S40149693	brown	211.5995	60.7289	0.858163	0.062742	65.264
gnl|UG|Ssc#S40151409	blue	760.4224	188.625	0.921143	0.026265	40.568
gnl|UG|Ssc#S40152348	yellow	216.9272	63.9747	0.845162	0.071417	55.592
gnl|UG|Ssc#S40152399	white	224.1118	22.4238	0.950125	0.01327	3.102
gnl|UG|Ssc#S40153033	brown	205.3722	60.7776	0.853888	0.065556	12.43

gnl|UG|Ssc#S40153466	turquoise	591.1654	177.091	0.872381	0.053668	154.336
gnl|UG|Ssc#S40153514	royalblue	335.1091	31.356	0.983173	0.002614	53.298
gnl|UG|Ssc#S40154325	black	859.6987	87.8562	0.974796	0.004785	3.174
gnl|UG|Ssc#S40154377	brown	179.7373	52.4401	0.814305	0.093337	1.554
gnl|UG|Ssc#S40154544	purple	194.2947	17.9019	0.808196	0.097884	87.51
gnl|UG|Ssc#S40155098	tan	865.2886	66.4521	0.99126	0.00098	3.198
gnl|UG|Ssc#S40155274	green	96.49012	33.8054	0.773338	0.125042	16.344
gnl|UG|Ssc#S40157076	purple	119.38	18.6648	0.726047	0.164871	19.152
gnl|UG|Ssc#S40157206	red	663.2862	102.703	0.982978	0.002659	9.24
gnl|UG|Ssc#S40158599	green	91.93634	41.0055	0.879732	0.049155	5.54
gnl|UG|Ssc#S40159132	turquoise	588.6338	182.675	0.85322	0.065999	81.572
gnl|UG|Ssc#S40160168	blue	458.8969	123.832	0.829811	0.082096	8.136
gnl|UG|Ssc#S40162079	darkred	141.9292	27.3527	0.867906	0.056475	3.764
gnl|UG|Ssc#S40162503	blue	816.1148	238.911	0.974081	0.00499	67.316
gnl|UG|Ssc#S40163341	violet	930.4593	74.1585	0.996361	0.000263	4.25
gnl|UG|Ssc#S40163661	turquoise	371.8022	125.055	0.749345	0.144846	73.71
gnl|UG|Ssc#S40166203	turquoise	769.7008	248.823	0.938147	0.018294	5.438
gnl|UG|Ssc#S40166987	yellow	135.1118	36.6802	0.720041	0.170153	2.408
gnl|UG|Ssc#S40169746	turquoise	640.3582	179.545	0.878603	0.04984	17.904
gnl|UG|Ssc#S40170485	turquoise	735.538	225.412	0.928899	0.022515	19.266
gnl|UG|Ssc#S40171578	magenta	199.6185	59.3054	0.931716	0.021199	2.352
gnl|UG|Ssc#S40171618	black	964.5842	99.0636	0.999	3.80E-05	20.564
gnl|UG|Ssc#S40171668	navy	140.0448	30.1286	0.896052	0.039597	45.858
gnl|UG|Ssc#S40172065	darkgreen	266.0058	23.4141	0.865222	0.058181	5.92
gnl|UG|Ssc#S40173479	yellow	263.3688	94.6904	0.94195	0.016643	2.026
gnl|UG|Ssc#S40173770	turquoise	688.4225	227.134	0.90576	0.034233	24.44
gnl|UG|Ssc#S40174139	purple	200.4217	18.1624	0.795925	0.107215	43.068
gnl|UG|Ssc#S40174218	turquoise	602.2084	190.221	0.88276	0.047332	7.426
gnl|UG|Ssc#S40174963	darkgreen	222.2241	26.68	0.92736	0.023244	622.918
gnl|UG|Ssc#S40175532	orange	238.3814	27.2081	0.940764	0.017152	16.836
gnl|UG|Ssc#S40177122	brown	194.9086	42.5339	0.685274	0.201644	15.626
gnl|UG|Ssc#S40180694	pink	270.2665	69.4914	0.962863	0.008543	22.488
gnl|UG|Ssc#S40181886	turquoise	592.7153	164.691	0.857445	0.063211	1.606
gnl|UG|Ssc#S40182904	blue	790.7	173.649	0.903211	0.035618	7.132
gnl|UG|Ssc#S40184013	paleturquoise	150.1143	16.9133	0.92203	0.025827	9.054
gnl|UG|Ssc#S40185643	yellow	299.5022	105.946	0.967485	0.007004	2.004
gnl|UG|Ssc#S40186233	brown	217.4293	60.1306	0.859306	0.061996	51.346
gnl|UG|Ssc#S40188565	turquoise	323.6825	86.3196	0.636577	0.24816	12.52
gnl|UG|Ssc#S40191217	yellow	278.5167	100.115	0.958408	0.010119	1.604
gnl|UG|Ssc#S40191742	blue	828.3551	226.676	0.961737	0.008933	11.766
gnl|UG|Ssc#S40195327	turquoise	464.4216	153.651	0.826026	0.084798	67.122
gnl|UG|Ssc#S40197378	black	946.4837	97.7375	0.996601	0.000238	21.53
gnl|UG|Ssc#S40198313	lightgreen	259.8448	55.7631	0.980154	0.003346	7.742
gnl|UG|Ssc#S40198369	brown	215.7478	62.0306	0.92094	0.026367	4.668
gnl|UG|Ssc#S40204438	magenta	211.2527	68.0166	0.956553	0.0108	11.046
gnl|UG|Ssc#S40204678	orange	361.0995	30.8365	0.967156	0.00711	31.968
gnl|UG|Ssc#S40204729	lightcyan	213.263	32.3291	0.926835	0.023494	14.19
gnl|UG|Ssc#S40205297	turquoise	941.4116	273.036	0.992026	0.000854	7.102

gnl|UG|Ssc#S40206941	blue	893.0359	236.414	0.972978	0.005311	55.272
gnl|UG|Ssc#S40206946	brown	118.0597	22.1629	0.505749	0.384675	3.582
gnl|UG|Ssc#S40208375	darkturquoise	169.7335	23.439	0.901277	0.03668	1.666
gnl|UG|Ssc#S40209535	turquoise	622.6289	204.685	0.900005	0.037383	8.038
gnl|UG|Ssc#S40210299	greenyellow	195.2685	53.2917	0.99017	0.001168	76.982
gnl|UG|Ssc#S40210420	turquoise	888.1734	259.024	0.968005	0.006837	23.158
gnl|UG|Ssc#S40212608	darkgreen	246.2063	36.9693	0.974288	0.00493	19.2
gnl|UG|Ssc#S40212638	turquoise	668.8484	168.779	0.858397	0.062589	64.672
gnl|UG|Ssc#S40213312	green	104.5995	54.6705	0.948407	0.013958	307.108
gnl|UG|Ssc#S40213383	green	81.02609	44.8224	0.868249	0.056259	12.524
gnl|UG|Ssc#S40214496	turquoise	696.0273	222.377	0.898345	0.038308	5.356
gnl|UG|Ssc#S40214545	greenyellow	238.8029	43.3283	0.930685	0.021677	17.204
gnl|UG|Ssc#S40214866	brown	100.8996	42.2315	0.826457	0.08449	44.874
gnl|UG|Ssc#S40215563	yellow	259.7954	86.2178	0.927801	0.023034	6.204
gnl|UG|Ssc#S40215755	pink	82.94664	8.1718	0.183795	0.76731	8.086
gnl|UG|Ssc#S40219335	midnightblue	187.5952	42.171	0.9729	0.005333	16.936
gnl|UG|Ssc#S40219489	navy	118.7763	20.5017	0.782845	0.117439	20.772
gnl|UG|Ssc#S40219525	purple	168.5344	17.7735	0.808767	0.097456	28.596
gnl|UG|Ssc#S40219983	royalblue	279.5716	29.2174	0.948971	0.013731	125.506
gnl|UG|Ssc#S40232606	darkgrey	179.1101	32.868	0.97971	0.003459	7.552
gnl|UG|Ssc#S40233232	magenta	276.1006	77.4887	0.986394	0.001901	10.106
gnl|UG|Ssc#S40235024	turquoise	562.1134	170.272	0.842422	0.073289	4.834
gnl|UG|Ssc#S40238117	lightgreen	291.984	54.6033	0.975864	0.004485	41.624
gnl|UG|Ssc#S40238461	midnightblue	175.1384	39.4181	0.945438	0.015173	7.27
gnl|UG|Ssc#S40239079	greenyellow	179.2253	36.6099	0.890045	0.043039	20.428
gnl|UG|Ssc#S40241735	magenta	224.5623	70.8565	0.968598	0.006648	0.692
gnl|UG|Ssc#S40243562	darkturquoise	118.52	1.80283	0.339752	0.575887	16.818
gnl|UG|Ssc#S40262955	lightyellow	948.5302	63.2689	0.999142	3.02E-05	2.512
gnl|UG|Ssc#S40267793	orange	261.5809	30.6348	0.965324	0.007711	91.89
gnl|UG|Ssc#S40268415	turquoise	627.4799	154.653	0.828813	0.082806	15.408
gnl|UG|Ssc#S40268599	greenyellow	161.4109	46.5866	0.960168	0.009486	8.666
gnl|UG|Ssc#S40268747	magenta	260.6386	80.724	0.995012	0.000423	16.542
gnl|UG|Ssc#S40270259	blue	640.6957	174.499	0.905308	0.034478	5.154
gnl|UG|Ssc#S40271107	red	626.6984	106.154	0.989897	0.001217	33.57
gnl|UG|Ssc#S40272618	royalblue	158.9417	15.5462	0.741384	0.151603	132.258
gnl|UG|Ssc#S40277991	green	93.34728	37.0501	0.852102	0.066742	7.978
gnl|UG|Ssc#S40278304	red	695.6668	85.0005	0.939973	0.017495	5.178
gnl|UG|Ssc#S40283901	royalblue	377.5923	33.3347	0.999607	9.37E-06	429.566
gnl|UG|Ssc#S40288024	green	107.8088	46.0335	0.905241	0.034514	13.848
gnl|UG|Ssc#S40288802	turquoise	800.5467	233.976	0.936775	0.018902	50.81
gnl|UG|Ssc#S40289417	navy	131.188	41.2517	0.940162	0.017412	4.13
gnl|UG|Ssc#S40306095	turquoise	626.7642	200.204	0.86611	0.057615	13.262
gnl|UG|Ssc#S40312019	brown	230.7449	59.207	0.881999	0.047788	2.678
gnl|UG|Ssc#S40314380	darkorange	65.27995	1.17516	0.284882	0.642245	0.678
gnl|UG|Ssc#S40315766	lightcyan	252.7633	37.8596	0.951922	0.012563	16.416
gnl|UG|Ssc#S40318462	black	998.8956	96.6889	0.993721	0.000597	165.918
gnl|UG|Ssc#S40320170	turquoise	443.8447	145.284	0.796463	0.106801	1.102
gnl|UG|Ssc#S40322346	turquoise	282.832	93.1234	0.662889	0.222695	62.142

gnl|UG|Ssc#S40324300	grey60	975.8525	60.5758	0.989133	0.001358	17.394
gnl|UG|Ssc#S40324547	lightgreen	286.0987	60.4912	0.996675	0.00023	6.182
gnl|UG|Ssc#S40324708	grey	116.1548	4.30598	0.282085	0.645659	15.736
gnl|UG|Ssc#S40326987	red	451.0511	105.806	0.989923	0.001213	91.6
gnl|UG|Ssc#S40327511	greenyellow	157.019	44.5	0.947724	0.014235	27.222
gnl|UG|Ssc#S40330478	skyblue	393.5242	25.5665	0.943137	0.016138	20.15
gnl|UG|Ssc#S40333225	brown	118.2411	34.0688	0.651736	0.233397	19.948
gnl|UG|Ssc#S40334954	blue	820.6163	204.385	0.937125	0.018746	12.014
gnl|UG|Ssc#S40335524	pink	259.1497	72.0783	0.980598	0.003235	15.364
gnl|UG|Ssc#S40342578	darkorange	163.8582	22.5683	0.988235	0.001529	16.61
gnl|UG|Ssc#S40349544	black	810.9816	77.3323	0.94925	0.013619	3.658
gnl|UG|Ssc#S40352145	blue	883.4161	222.696	0.957221	0.010553	2.284
gnl|UG|Ssc#S40362920	lightgreen	277.3457	58.9546	0.991417	0.000953	1.444
gnl|UG|Ssc#S40367565	blue	651.0492	158.5	0.884512	0.046288	4.466
gnl|UG|Ssc#S40369764	yellow	283.2897	107.287	0.974505	0.004868	0.828
gnl|UG|Ssc#S40370377	navy	77.37925	19.1763	0.702439	0.185906	42.442
gnl|UG|Ssc#S40376111	darkred	211.4186	12.5603	0.683263	0.203511	15.208
gnl|UG|Ssc#S40376675	grey60	979.2456	59.7988	0.9865	0.001879	14.098
gnl|UG|Ssc#S40377012	midnightblue	98.03554	34.3667	0.92498	0.024387	307.274
gnl|UG|Ssc#S40377740	yellow	278.088	94.8316	0.945199	0.015273	844.392
gnl|UG|Ssc#S40378245	green	102.1776	52.2118	0.933505	0.020377	9.068
gnl|UG|Ssc#S40380547	greenyellow	217.5491	49.0416	0.969608	0.006331	7.02
gnl|UG|Ssc#S40381468	yellow	213.9546	62.6467	0.854111	0.065407	4.424
gnl|UG|Ssc#S40390344	brown	197.3234	59.0612	0.845935	0.070891	2.8
gnl|UG|Ssc#S40396824	navy	148.1383	31.4297	0.898934	0.037979	134.674
gnl|UG|Ssc#S40401313	red	509.0115	109.994	0.998444	7.37E-05	247.67
gnl|UG|Ssc#S40406997	white	890.1054	26.5773	0.810899	0.095864	5.194
gnl|UG|Ssc#S40411000	purple	78.99574	17.3203	0.997211	0.000177	14.892
gnl|UG|Ssc#S40414470	brown	100.9307	26.7405	0.456868	0.439221	48.75
gnl|UG|Ssc#S40416576	pink	155.97	32.7457	0.829669	0.082196	12.204
gnl|UG|Ssc#S40417362	blue	798.5301	204.369	0.936826	0.018879	14.916
gnl|UG|Ssc#S40424734	lightcyan	180.6637	12.2814	0.47939	0.413882	15.056
gnl|UG|Ssc#S40426329	lightcyan	233.3995	36.6214	0.947134	0.014475	21.346
gnl|UG|Ssc#S40427363	magenta	270.139	79.2908	0.991956	0.000865	6.026
gnl|UG|Ssc#S40430134	navy	144.0539	11.5085	-0.35149	0.561861	1.114
gnl|UG|Ssc#S40431495	salmon	163.1042	44.8756	0.960699	0.009297	20.268
gnl|UG|Ssc#S40431582	blue	782.0429	223.512	0.960133	0.009498	44.472
gnl|UG|Ssc#S40432472	lightgreen	302.827	59.5144	0.993318	0.000655	8.032
gnl|UG|Ssc#S40434088	turquoise	681.5193	224.651	0.896668	0.03925	98.676
gnl|UG|Ssc#S40435203	skyblue	330.4071	25.3812	0.939403	0.017743	7.336
gnl|UG|Ssc#S40435713	darkorange	179.2904	20.3575	0.987197	0.001736	22.244
gnl|UG|Ssc#S40437142	brown	197.1755	60.2452	0.860244	0.061386	3.16
gnl|UG|Ssc#S40437415	grey	156.5681	14.6206	-0.89886	0.03802	12.82
gnl|UG|Ssc#S40439319	darkred	243.988	42.1263	0.977713	0.003981	4.256
gnl|UG|Ssc#S40439733	lightgreen	290.6379	54.8133	0.976533	0.0043	14.82
gnl|UG|Ssc#S40439791	darkturquoise	181.2867	32.3951	0.977381	0.00407	8.66
gnl|UG|Ssc#S40440480	greenyellow	117.6178	23.8698	0.784806	0.115888	273.272
gnl|UG|Ssc#S40442316	midnightblue	215.629	37.0064	0.94088	0.017102	18.478

gnl|UG|Ssc#S40442878	darkgrey	164.6781	27.9205	0.949783	0.013406	19.272
gnl|UG|Ssc#S40443028	lightgreen	302.4509	55.912	0.980698	0.00321	0.866
gnl|UG|Ssc#S40443068	darkgreen	246.6968	20.2558	0.810943	0.095831	15.706
gnl|UG|Ssc#S40443438	purple	87.09522	3.72639	-0.71464	0.174944	26.34
gnl|UG|Ssc#S40443581	navy	111.0619	36.9021	0.890815	0.042593	5.072
gnl|UG|Ssc#S40448602	pink	292.1082	63.8361	0.930466	0.02178	9.552
gnl|UG|Ssc#S40453605	pink	276.3523	71.7519	0.975078	0.004705	5.554
gnl|UG|Ssc#S40459875	yellow	288.3743	110.276	0.979654	0.003473	19.806
gnl|UG|Ssc#S40463925	darkturquoise	195.5587	29.7552	0.956725	0.010736	4.296
gnl|UG|Ssc#S40471332	greenyellow	209.6358	48.8017	0.966247	0.007406	43.356
gnl|UG|Ssc#S40472465	darkgrey	147.4954	26.7459	0.933288	0.020476	11.88
gnl|UG|Ssc#S40473833	pink	227.2504	68.4177	0.984385	0.002337	6.234
gnl|UG|Ssc#S40473912	lightcyan	276.4176	38.8659	0.960552	0.009349	1.082
gnl|UG|Ssc#S40474141	turquoise	809.5604	246.044	0.947389	0.014371	14.276
gnl|UG|Ssc#S40474257	darkturquoise	163.1063	24.7801	0.900159	0.037298	7.384
gnl|UG|Ssc#S40475010	darkmagenta	959.25	26.8513	0.992944	0.000711	13.432
gnl|UG|Ssc#S40475244	violet	982.817	73.3687	0.994216	0.000528	3.624
gnl|UG|Ssc#S40476333	turquoise	789.6602	253.553	0.950874	0.012974	36.336
gnl|UG|Ssc#S40477805	grey60	910.0558	61.3838	0.992061	0.000848	6.964
gnl|UG|Ssc#S40478006	blue	675.8771	130.954	0.842283	0.073384	2.336
gnl|UG|Ssc#S40478259	magenta	267.9464	81.6099	0.998022	0.000106	59.074
gnl|UG|Ssc#S40479242	tan	889.7059	67.2329	0.993774	0.000589	18.622
gnl|UG|Ssc#S40479246	purple	155.6831	20.714	0.865832	0.057792	11.822
gnl|UG|Ssc#S40480053	brown	113.9794	43.8888	0.815526	0.092435	11.578
gnl|UG|Ssc#S40480472	yellow	238.1562	78.5755	0.89959	0.037614	3.686
gnl|UG|Ssc#S40481511	white	185.1066	24.0677	0.865069	0.058279	46.666
gnl|UG|Ssc#S40482232	turquoise	934.5364	265.051	0.986947	0.001787	30.684
gnl|UG|Ssc#S40482424	salmon	228.896	48.0189	0.974805	0.004783	29.528
gnl|UG|Ssc#S40482896	turquoise	383.1343	122.572	0.723642	0.16698	10.84
gnl|UG|Ssc#S40483985	turquoise	840.8179	242	0.949864	0.013374	26.182
gnl|UG|Ssc#S40485583	grey60	858.3761	58.6124	0.982668	0.002732	4.73
gnl|UG|Ssc#S40485684	blue	703.4718	172.33	0.90114	0.036755	4.626
gnl|UG|Ssc#S40488267	turquoise	330.8549	68.7611	0.61729	0.26729	11.618
gnl|UG|Ssc#S40492797	lightgreen	283.8522	60.4054	0.99641	0.000258	0.102
gnl|UG|Ssc#S40492996	royalblue	178.7588	7.0416	0.37329	0.535994	0.72
gnl|UG|Ssc#S40494040	magenta	267.625	78.8677	0.989759	0.001242	6.788
gnl|UG|Ssc#S40494112	violet	999.0852	68.9031	0.981398	0.003037	10.068
gnl|UG|Ssc#S40494421	pink	78.22013	19.4597	0.42338	0.477503	33.228
gnl|UG|Ssc#S40494534	salmon	140.4631	31.9177	0.873934	0.052704	22.996
gnl|UG|Ssc#S40494554	yellowgreen	162.0235	16.2501	0.966974	0.007169	12.612
gnl|UG|Ssc#S40494954	white	963.4919	29.452	0.790415	0.111487	10.214
gnl|UG|Ssc#S40495537	turquoise	765.494	243.183	0.941761	0.016724	318.036
gnl|UG|Ssc#S40495944	turquoise	848.5993	259.123	0.960108	0.009507	48.206
gnl|UG|Ssc#S40496270	blue	835.7577	198.574	0.9333	0.02047	55.168
gnl|UG|Ssc#S40496326	blue	784.6384	234.977	0.971048	0.005888	21.332
gnl|UG|Ssc#S40496509	brown	94.46862	35.6499	0.786728	0.114375	6.736
gnl|UG|Ssc#S40499484	grey60	909.6132	59.998	0.987337	0.001707	5.522
gnl|UG|Ssc#S40499633	pink	280.7116	73.7062	0.981291	0.003063	6.422

gnl|UG|Ssc#S40500401	salmon	149.7757	46.7044	0.970384	0.006091	67.714
gnl|UG|Ssc#S40500672	sienna3	997.4918	25.3733	0.994134	0.000539	82.726
gnl|UG|Ssc#S40500952	midnightblue	99.70485	21.105	0.80501	0.100282	538.486
gnl|UG|Ssc#S40508283	darkorange	173.6042	21.5093	0.978661	0.00373	3.712
gnl|UG|Ssc#S40511352	skyblue	131.6092	19.521	0.887641	0.044441	1.17
gnl|UG|Ssc#S40512743	darkred	245.9058	42.7094	0.982481	0.002776	3.338
gnl|UG|Ssc#S40513578	turquoise	344.0956	99.1976	0.65858	0.226813	11.724
gnl|UG|Ssc#S40516238	turquoise	505.7162	129.543	0.75485	0.140226	7.988
gnl|UG|Ssc#S40516636	brown	84.75371	28.7515	0.753183	0.14162	7.868
gnl|UG|Ssc#S40524913	turquoise	888.045	256.253	0.970645	0.006011	331.772
gnl|UG|Ssc#S40526036	skyblue	108.26	18.4791	0.871648	0.054124	63.5
gnl|UG|Ssc#S40526482	royalblue	387.2958	33.0867	0.996618	0.000236	9.24
gnl|UG|Ssc#S40527077	lightcyan	180.0996	12.5622	0.474771	0.41905	9.666
gnl|UG|Ssc#S40527391	yellow	168.6051	44.9915	0.757494	0.138023	25.32
gnl|UG|Ssc#S40527867	greenyellow	194.6036	55.4558	0.998363	7.95E-05	79.33
gnl|UG|Ssc#S40528822	grey60	880.4136	60.2789	0.988288	0.001519	22.626
gnl|UG|Ssc#S40529248	grey	163.8939	14.8601	-0.96722	0.00709	346.352
gnl|UG|Ssc#S40529850	darkgreen	236.2217	33.8636	0.969362	0.006408	7.09
gnl|UG|Ssc#S40543101	brown	133.0059	37.7229	0.737856	0.154626	11.69
gnl|UG|Ssc#S40543607	midnightblue	153.0927	37.5	0.952552	0.012318	11.642
gnl|UG|Ssc#S40544230	salmon	225.4117	46.7079	0.9698	0.006272	4.79
gnl|UG|Ssc#S40546869	turquoise	686.5411	225.588	0.898996	0.037945	46.26
gnl|UG|Ssc#S40546945	lightgreen	274.662	58.321	0.98925	0.001336	2.776
gnl|UG|Ssc#S40550337	tan	794.1723	60.938	0.972599	0.005422	48.5
gnl|UG|Ssc#S40551167	darkgreen	217.1391	16.5488	0.737434	0.154989	2.896
gnl|UG|Ssc#S40553929	black	986.1048	95.6794	0.991305	0.000972	31.28
gnl|UG|Ssc#S40554875	royalblue	296.3661	31.1881	0.97226	0.005523	12.274
gnl|UG|Ssc#S40556021	turquoise	521.9258	148.116	0.817243	0.091173	18.694
gnl|UG|Ssc#S40558865	pink	264.5653	75.5496	0.998359	7.98E-05	0.468
gnl|UG|Ssc#S40559812	skyblue	183.3656	28.9058	0.973623	0.005122	54.09
gnl|UG|Ssc#S40559942	royalblue	248.5346	27.3357	0.932913	0.020648	8.342
gnl|UG|Ssc#S40560136	pink	156.1977	43.5332	0.885489	0.045709	21.278
gnl|UG|Ssc#S40570043	turquoise	862.8124	261.307	0.963849	0.008206	18.18
gnl|UG|Ssc#S40572845	turquoise	448.6003	150.152	0.83201	0.080538	7.952
gnl|UG|Ssc#S40583885	darkgrey	165.6368	29.088	0.951744	0.012633	12.818
gnl|UG|Ssc#S40584531	red	294.4154	68.5054	0.895663	0.039817	8.572
gnl|UG|Ssc#S40584674	turquoise	597.5306	172.122	0.859544	0.061841	7.172
gnl|UG|Ssc#S40588301	brown	179.3814	57.4359	0.917545	0.028068	382.472
gnl|UG|Ssc#S41446009	black	934.9686	97.2342	0.995241	0.000394	22.896
gnl|UG|Ssc#S41446582	black	893.8069	90.7775	0.981335	0.003053	12.638
gnl|UG|Ssc#S41575095	darkturquoise	181.9185	22.8815	0.885835	0.045504	12.49
gnl|UG|Ssc#S41575759	lightgreen	292.5917	56.945	0.984286	0.002359	2.372
gnl|UG|Ssc#S41576667	navy	84.42769	5.80895	0.032843	0.95819	12.288
gnl|UG|Ssc#S46917265	brown	92.98824	40.1364	0.784985	0.115747	37.314
gnl|UG|Ssc#S46917266	greenyellow	162.8034	45.6094	0.951117	0.012878	105.272
gnl|UG|Ssc#S5979288	yellow	194.9574	63.0018	0.84031	0.074742	4.428
gnl|UG|Ssc#S5983468	blue	848.9256	198.477	0.930527	0.021751	13.808
gnl|UG|Ssc#S5987022	red	485.6038	98.9288	0.974597	0.004842	16.726

gnl|UG|Ssc#S5987782	darkred	232.2522	44.5184	0.987882	0.001598	4.79
gnl|UG|Ssc#S5989761	lightgreen	287.2394	59.9807	0.994916	0.000435	2.836
gnl|UG|Ssc#S5991837	yellow	309.6478	116.295	0.991817	0.000887	4.466
gnl|UG|Ssc#S5993568	lightcyan	282.6863	41.7816	0.979076	0.003622	1.906
gnl|UG|Ssc#S5994539	royalblue	284.9657	30.7692	0.962006	0.008839	4.38
gnl|UG|Ssc#S5995101	turquoise	831.115	260.108	0.95973	0.009642	1.504
gnl|UG|Ssc#S5995435	grey	147.7297	6.82561	0.419036	0.48252	64.642
gnl|UG|Ssc#S5999093	white	194.143	25.0881	0.890564	0.042738	8.376
gnl|UG|Ssc#S5999899	magenta	257.8055	81.9705	0.999072	3.39E-05	16.552
gnl|UG|Ssc#S6001821	pink	286.3915	62.8591	0.929693	0.022141	10.222
gnl|UG|Ssc#S6008489	pink	150.7709	42.3119	0.868508	0.056095	23.3
gnl|UG|Ssc#S6014656	pink	103.9062	13.6625	0.068506	0.912844	81.562
gnl|UG|Ssc#S6030301	magenta	261.9439	82.2409	0.999509	1.31E-05	1.798
gnl|UG|Ssc#S6033944	paleturquoise	192.195	18.62	0.933632	0.020319	2.364
gnl|UG|Ssc#S6035331	brown	106.0503	33.1746	0.585402	0.299727	2.242
gnl|UG|Ssc#S6039711	royalblue	292.1123	29.8471	0.950895	0.012966	5.286
gnl|UG|Ssc#S6047303	royalblue	271.6765	21.7674	0.863172	0.059494	25.256
gnl|UG|Ssc#S6048843	grey	127.8147	9.165	-0.75882	0.136926	18.788
gnl|UG|Ssc#S6049456	green	94.22678	46.3979	0.908067	0.032996	32.924
gnl|UG|Ssc#S6052869	purple	193.7681	19.3342	-0.73254	0.159214	2.4
gnl|UG|Ssc#S6053340	darkgrey	205.8544	33.5726	0.988513	0.001475	0.37
gnl|UG|Ssc#S6054321	darkturquoise	130.0439	21.5727	0.863248	0.059445	1.96
gnl|UG|Ssc#S6054758	royalblue	333.5637	32.6185	0.9917	0.000907	0.616
gnl|UG|Ssc#S6055006	darkturquoise	220.2392	32.0235	0.975415	0.00461	3.096
gnl|UG|Ssc#S6055104	green	89.57042	44.5201	0.862427	0.059974	12.546
gnl|UG|Ssc#S6055465	lightcyan	184.9499	13.6727	0.568745	0.317048	16.562
gnl|UG|Ssc#S6056024	pink	165.6918	3.47046	-0.88564	0.045618	8.426
gnl|UG|Ssc#S6057089	darkmagenta	803.2996	22.9961	0.96142	0.009044	4.756
gnl|UG|Ssc#S6057322	navy	150.8495	9.45882	-0.62218	0.262407	4.686
gnl|UG|Ssc#S6057513	darkgreen	287.5072	36.1865	0.982222	0.002838	63.168
gnl|UG|Ssc#S6058884	blue	892.9207	218.035	0.953348	0.012011	21.018
gnl|UG|Ssc#S6058939	darkorange	200.2094	20.2268	0.96949	0.006368	31.788
gnl|UG|Ssc#S6059254	blue	747.6902	155.652	0.882418	0.047537	8.194
gnl|UG|Ssc#S6061125	lightgreen	266.5542	56.207	0.981725	0.002957	1.84
gnl|UG|Ssc#S6061185	yellow	298.0077	112.935	0.985006	0.002199	3.378
gnl|UG|Ssc#S6063101	brown	187.1726	52.5259	0.754154	0.140808	7.858
gnl|UG|Ssc#S6063403	magenta	266.6018	80.5353	0.994658	0.000468	16.77
gnl|UG|Ssc#S6063458	royalblue	117.6035	7.47448	0.505599	0.38484	19.924
gnl|UG|Ssc#S6063538	darkred	214.8388	32.5699	0.925018	0.024368	1.454
gnl|UG|Ssc#S6064814	yellow	303.3962	116.148	0.991843	0.000883	6.5
gnl|UG|Ssc#S6065016	darkgrey	203.0995	31.6841	0.973859	0.005054	19.71
gnl|UG|Ssc#S6065152	yellow	286.012	102.91	0.961154	0.009137	2.536
gnl|UG|Ssc#S6065267	lightgreen	297.7557	61.2203	0.999121	3.13E-05	1.034
gnl|UG|Ssc#S6072007	orange	356.0463	34.9345	0.994348	0.00051	8.376
gnl|UG|Ssc#S6072016	brown	212.1886	56.8213	0.815357	0.09256	7.934
gnl|UG|Ssc#S6072575	violet	901.7717	72.3418	0.991363	0.000962	8.354
gnl|UG|Ssc#S6072742	darkgreen	157.1181	23.7294	0.876044	0.051403	7.94
gnl|UG|Ssc#S6072986	magenta	270.9428	78.5974	0.989267	0.001333	45.654

gnl|UG|Ssc#S6073178	blue	554.9035	167.504	0.893303	0.041161	12.612
gnl|UG|Ssc#S6073996	red	405.6503	87.2187	0.948022	0.014114	12.152
gnl|UG|Ssc#S6074088	salmon	232.9913	47.0218	0.972745	0.005379	17.014
gnl|UG|Ssc#S6074397	yellow	293.7899	107.141	0.970477	0.006062	4.62
gnl|UG|Ssc#S6074613	green	164.5944	22.2401	0.384288	0.523034	10.232
gnl|UG|Ssc#S6074827	greenyellow	179.3854	46.6628	0.958233	0.010182	11.912
gnl|UG|Ssc#S6075131	darkorange	193.5403	22.1264	0.995586	0.000352	7.17
gnl|UG|Ssc#S6075522	pink	217.921	64.861	0.97546	0.004598	1.744
gnl|UG|Ssc#S6075829	darkred	221.7466	43.6378	0.982241	0.002833	47.498
gnl|UG|Ssc#S6076796	darkred	170.5599	28.436	0.892038	0.041887	1.628
gnl|UG|Ssc#S6076838	blue	843.9724	246.852	0.983962	0.002432	9.362
gnl|UG|Ssc#S6077148	lightyellow	977.5261	62.9986	0.998274	8.61E-05	3.126
gnl|UG|Ssc#S6077174	yellow	303.8102	117.985	0.996877	0.000209	3.658
gnl|UG|Ssc#S6078209	yellow	302.5129	117.356	0.994671	0.000467	1.866
gnl|UG|Ssc#S6078250	yellow	305.804	113.849	0.98645	0.001889	5.832
gnl|UG|Ssc#S6078867	lightgreen	294.7818	57.7942	0.987332	0.001708	2.834
gnl|UG|Ssc#S6078919	yellow	303.761	110.33	0.979032	0.003633	3.664
gnl|UG|Ssc#S6080666	darkgreen	152.481	21.4504	0.864022	0.058949	0.434
gnl|UG|Ssc#S6081209	black	935.6751	96.7083	0.993773	0.000589	5.332
gnl|UG|Ssc#S6081736	lightyellow	937.9645	62.8937	0.997956	0.000111	5.138
gnl|UG|Ssc#S6082387	black	882.9522	94.1821	0.988783	0.001424	4.226
gnl|UG|Ssc#S6083312	yellow	310.4556	117.911	0.995267	0.000391	3.864
gnl|UG|Ssc#S6084972	navy	108.4889	8.55761	0.282608	0.645021	34.002
gnl|UG|Ssc#S6085466	greenyellow	142.2819	39.1727	0.911171	0.031354	65.856
gnl|UG|Ssc#S6085536	green	101.3447	48.3873	0.902507	0.036003	35.634
gnl|UG|Ssc#S6085556	turquoise	685.4689	187.396	0.889118	0.043578	99.382
gnl|UG|Ssc#S6085589	salmon	129.4677	32.7951	0.881042	0.048364	21.554
gnl|UG|Ssc#S6085619	brown	106.6558	42.0152	0.748652	0.14543	5.708
gnl|UG|Ssc#S6085659	magenta	239.2236	75.5239	0.979976	0.003391	2.596
gnl|UG|Ssc#S6085740	brown	124.0677	44.7379	0.916645	0.028525	45.62
gnl|UG|Ssc#S6087197	red	452.4234	94.2537	0.963846	0.008207	11.114
gnl|UG|Ssc#S6088059	brown	224.2631	59.1888	0.889629	0.04328	5.064
gnl|UG|Ssc#S6088064	yellow	233.3464	76.6451	0.899517	0.037655	0.98
gnl|UG|Ssc#S6665112	turquoise	904.7865	270.264	0.980691	0.003212	11.318
gnl|UG|Ssc#S6665509	lightcyan	235.3838	36.0311	0.934749	0.019812	28.992
gnl|UG|Ssc#S6666019	lightyellow	941.7922	63.23	0.999015	3.71E-05	3.244
gnl|UG|Ssc#S6666342	brown	191.4475	53.6656	0.760789	0.135292	17.79
gnl|UG|Ssc#S6666751	brown	107.5584	43.7369	0.799726	0.104297	9.062
gnl|UG|Ssc#S6667357	yellow	174.1055	43.0922	0.753942	0.140985	9.328

Table S3-1 Calculation of the overlap for each possible pair of modules between LDE and LT in prenatal.

	black	blue	brown	darkgreen	darkgrey	darkmagenta	darkorange	darkred	darkturquoise	green	greenyellow	grey	grey60	lightcyan	lightgreen	lightyellow	magenta	midnightblue	navy	orange	paleturquoise	pink	purple	red	royalblue	salmon	sienna3	skyblue	skyblue3	tan	turquoise	violet	white	yellow	yellowgreen	(blank)	Total
black	0	1	9	1	1	2	1	0	0	0	3	0	0	0	0	1	5	0	2	0	1	6	0	3	0	0	0	0	0	0	15	2	0	5	1	2	59
blue	0	43	4	2	0	1	0	12	5	1	0	2	0	3	5	5	10	3	6	3	0	3	0	3	0	2	1	2	0	3	57	5	4	8	1	0	194
brown	1	100	4	1	0	0	0	3	3	1	0	0	1	1	9	5	4	2	2	0	0	4	0	0	0	1	1	0	1	1	28	0	2	1	0	1	176
darkgreen	0	8	0	0	1	0	0	0	1	2	0	0	1	0	2	1	2	2	0	0	0	0	0	0	0	0	2	0	0	0	4	0	2	2	0	1	30
darkred	0	0	0	2	0	0	0	1	0	1	1	0	0	0	0	2	0	0	2	0	0	0	0	1	0	0	0	0	0	0	19	0	1	2	0	0	32
darkturquoise	0	0	0	1	0	0	0	1	2	2	0	0	0	0	0	0	0	0	0	1	0	1	0	1	0	0	1	0	0	0	14	1	1	1	0	0	27
green	0	1	13	3	0	1	3	0	0	0	9	0	0	0	0	1	1	0	2	0	0	4	15	3	1	0	0	0	0	0	18	2	4	3	4	1	88
greenyellow	1	13	1	0	0	0	1	2	1	0	0	1	0	0	2	1	2	1	0	0	0	1	1	2	2	0	0	0	0	1	13	1	0	1	0	2	48
grey	0	4	4	0	1	0	0	1	1	0	2	0	0	0	1	0	3	0	2	0	0	4	2	1	0	0	0	0	0	0	11	2	3	6	1	0	49
grey60	0	0	7	0	2	1	1	0	0	0	2	0	0	0	0	0	0	0	1	0	0	1	2	2	0	0	0	0	0	0	13	4	0	3	0	0	39
lightcyan	0	5	3	0	0	0	0	2	0	0	1	0	0	0	1	1	2	2	2	0	0	1	2	0	2	0	0	0	1	1	12	1	1	2	0	0	42
lightgreen	2	1	4	1	1	0	0	0	2	0	2	0	2	2	0	1	0	0	0	1	0	2	0	0	0	0	0	0	0	0	12	0	0	3	0	1	36
lightyellow	0	0	2	0	2	1	0	1	1	0	0	0	0	0	0	1	4	0	0	0	0	2	0	1	0	0	0	0	0	0	14	3	1	2	1	0	36
magenta	0	8	4	2	1	0	1	1	3	0	1	1	0	0	1	1	4	0	1	0	0	3	0	0	1	0	0	0	0	0	17	2	1	1	0	0	54
midnightblue	1	0	6	1	0	0	1	0	0	0	5	0	0	0	0	0	0	0	3	0	0	0	14	1	1	0	0	0	0	0	3	0	5	0	1	0	42
navy	0	1	7	1	0	0	2	1	1	0	5	0	0	0	0	0	2	0	5	1	0	0	3	1	0	0	0	0	0	0	10	0	1	3	1	0	45
pink	0	0	14	4	0	2	5	0	0	0	3	0	0	0	0	0	0	0	1	0	0	1	4	3	2	0	0	0	0	0	9	2	5	3	1	0	59
purple	0	5	0	1	2	1	0	3	0	2	0	0	1	2	0	0	2	1	0	0	0	0	0	0	0	1	2	2	0	0	27	0	1	1	0	0	54
red	0	1	8	2	0	1	0	1	0	0	4	0	0	0	0	1	3	0	5	0	0	0	12	3	4	0	0	0	0	0	11	2	1	3	2	0	64
royalblue	0	6	3	1	0	2	2	1	1	0	2	0	0	0	1	1	2	0	3	0	0	0	0	0	0	0	0	0	0	0	6	0	2	0	0	0	33
salmon	0	0	14	3	0	0	1	0	0	0	2	0	2	0	0	0	3	0	1	0	0	1	0	1	0	0	0	0	0	0	10	0	2	4	1	0	45
tan	1	4	5	0	1	0	0	1	3	0	0	0	1	1	1	0	4	0	4	0	0	2	0	2	0	0	0	0	0	1	16	1	0	1	0	1	49
turquoise	69	58	23	31	25	11	15	5	8	104	20	28	42	41	21	18	31	36	33	30	22	61	13	55	29	54	14	29	19	51	1021	25	14	66	7	54	2129
yellow	1	2	7	5	2	1	0	4	4	2	4	2	0	0	3	4	9	3	3	1	3	7	1	3	1	2	1	0	1	2	42	3	4	10	0	1	137
(blank)	1	0	0	0	0	0	2	1	3	0	0	0	0	1	0	0	0	1	1	0	0	1	0	0	0	0	1	0	0	0	15	1	1	1	1	0	31
Total	77	261	##	62	39	24	35	41	39	115	66	34	50	51	47	44	93	51	79	37	26	105	69	86	43	60	23	33	22	60	1417	57	56	132	22	64	3662

Table S3-2 Calculation of the overlap for each possible pair of modules between LDE and LT in postnatal.

	black	blue	brown	darkgreen	darkgrey	darkmagenta	darkorange	darkred	darkturquoise	green	greenyellow	grey	grey60	lightcyan	lightgreen	lightyellow	magenta	midnightblue	navy	orange	paleturquoise	pink	purple	red	royalblue	salmon	sienna3	skyblue	tan	turquoise	violet	white	yellow	yellowgreen	(blank)
black	5	15	2	3	6	2	1	3	2	0	3	0	7	2	1	4	1	0	1	0	0	2	4	1	2	1	0	1	4	31	3	5	6	0	2
blue	11	24	3	1	2	6	0	2	3	0	0	1	11	4	4	10	0	1	6	4	0	0	1	8	4	1	3	2	7	43	9	8	6	0	4
brown	2	25	2	1	2	1	4	6	1	7	8	2	2	1	3	1	1	6	12	1	8	6	7	3	1	10	1	3	2	40	1	4	7	2	3
darkgreen	0	3	13	0	0	2	2	1	0	9	1	2	0	5	0	1	2	1	0	3	1	9	1	3	1	0	1	0	0	10	0	0	1	0	1
darkgrey	3	5	0	1	1	1	0	2	3	0	0	1	0	0	1	2	0	0	0	1	0	2	1	1	0	0	1	0	6	16	5	3	5	0	1
darkmagenta	0	5	1	0	1	0	1	1	0	0	0	0	1	1	0	0	2	0	1	0	0	2	1	0	0	2	0	0	0	3	0	0	0	0	0
darkorange	1	13	0	1	1	1	1	0	0	0	1	0	2	0	0	1	0	0	1	3	0	1	2	4	1	2	1	0	2	16	1	1	1	0	1
darkred	0	0	13	0	2	0	3	0	0	2	0	2	0	4	0	1	15	1	2	1	0	13	1	4	3	0	0	0	0	4	0	0	1	0	1
darkturquoise	2	8	3	2	1	0	0	0	1	4	0	3	1	1	0	2	1	2	1	1	0	1	3	5	2	3	0	0	2	13	1	1	0	0	0
green	5	31	4	1	2	1	0	3	1	5	2	1	1	1	2	3	0	1	9	2	4	2	2	1	4	5	0	1	6	25	2	5	5	0	2
greenyellow	0	15	2	1	0	0	1	0	1	5	1	4	4	0	1	0	0	5	1	1	0	2	4	1	3	2	1	0	4	23	5	2	1	0	2
grey	0	1	0	0	1	0	1	0	0	1	0	0	0	0	0	1	1	0	3	0	1	0	1	0	0	1	0	0	1	2	0	1	4	0	0
grey60	2	6	8	1	0	0	1	1	1	8	1	2	0	1	0	0	0	2	2	2	0	1	2	13	1	2	0	1	0	13	1	0	3	0	3
lightcyan	4	2	7	2	6	0	1	0	4	3	1	1	1	2	1	0	4	1	1	3	0	7	4	2	3	0	0	0	2	13	2	1	4	1	2
lightgreen	2	8	5	1	1	0	1	0	1	3	6	0	0	0	1	2	2	5	1	0	1	8	2	3	6	2	2	1	0	7	1	0	2	2	1
lightyellow	2	4	1	2	2	1	0	2	0	6	4	7	0	0	1	0	0	1	4	0	2	2	3	0	2	6	0	2	1	8	1	2	3	5	2
magenta	1	2	5	9	0	0	3	2	1	14	18	4	0	2	1	0	1	9	5	0	3	4	2	0	0	6	0	4	0	1	0	0	5	4	4
midnightblue	6	20	1	0	1	0	1	1	2	0	0	0	5	0	1	3	0	1	2	0	0	0	1	2	0	2	0	2	1	21	3	4	3	0	4
navy	4	7	4	6	2	0	1	1	1	12	2	2	0	2	3	1	1	7	5	1	2	5	4	4	2	3	3	4	0	10	1	3	2	3	2
orange	1	4	7	2	2	0	1	0	0	3	0	0	1	2	0	2	2	0	1	1	0	8	2	1	3	1	0	1	0	7	1	1	3	0	3
paleturquoise	0	0	6	0	2	0	0	0	0	0	0	4	1	6	0	0	8	0	0	0	0	7	0	2	0	0	0	1	0	1	0	0	0	0	1
pink	3	13	1	8	0	2	1	7	8	2	1	0	5	0	17	4	2	2	5	0	2	0	3	7	0	0	2	1	4	14	5	7	30	0	2
purple	0	11	13	0	0	0	4	0	0	7	0	0	0	4	1	0	1	7	5	3	1	7	4	13	2	0	0	2	1	11	0	0	1	0	3
red	7	9	0	2	0	0	0	13	7	2	1	1	3	0	11	4	0	0	0	1	4	1	2	0	1	0	0	1	4	21	10	3	27	1	1
royalblue	0	8	1	3	1	0	2	2	2	1	5	3	1	0	0	0	0	1	6	0	5	2	0	2	0	7	0	5	0	8	1	0	3	1	3
salmon	1	3	25	0	1	0	0	0	0	3	1	1	0	9	0	1	14	0	0	2	0	12	1	2	4	1	1	0	0	2	1	1	2	0	2
skyblue	0	0	3	0	0	0	0	3	0	6	3	2	0	1	0	0	1	2	4	0	0	0	4	1	1	3	0	1	1	5	0	2	6	1	1
tan	0	2	9	1	2	1	2	0	0	6	1	2	0	6	0	1	17	1	2	1	0	11	1	6	2	1	0	0	2	8	0	1	2	1	1
turquoise	34	81	14	4	10	9	4	2	6	8	7	8	16	11	5	13	18	4	19	4	1	11	7	35	10	8	8	4	23	101	20	17	6	1	12
violet	5	6	3	0	0	0	3	3	3	3	5	0	1	3	3	5	1	5	3	1	0	1	0	0	1	2	1	1	1	7	1	1	13	1	0
white	5	4	5	2	0	3	0	5	1	0	1	1	1	2	9	2	0	0	1	3	0	5	2	2	0	1	0	1	1	6	2	0	7	0	0
yellow	1	13	12	1	1	1	2	0	1	19	4	1	4	3	0	1	1	4	5	5	2	4	7	5	6	4	3	2	3	51	1	0	3	1	1
(blank)	1	0	3	2	0	0	1	0	0	2	1	0	0	0	0	1	0	4	1	0	0	2	1	0	0	0	0	1	0	2	1	0	2	0	0
total	108	348	176	57	50	31	42	60	50	141	78	55	68	73	66	66	96	73	109	44	37	138	80	131	65	76	28	42	78	543	79	73	164	24	65

Table S4-1 Module eigengene and its evolutionary rates in the common module between LT and LDE (prenatal_brown vs blue).

pig_Genebank	pig_Gene	LDE Module	kTotal	kWithin	eigencorr	eigenpval	meanExpr	LT Module	kTotal	kWithin	eigencorr	eigenpval	meanExpr	Ka/Ks
NM_001044620.1	RABGGTB	brown	249.04	124.8191	0.98893777	0.0013944	179.71	blue	318.3339	195.6248	0.990748	0.001067	121.186	0
AK231026.1	EIF1AX	brown	213.82	107.3751	0.95209124	0.0124971	6.882	blue	261.1911	167.7916	0.956577	0.010791	2.072	--
AK234386.1	SFT2D1	brown	212.62	120.2547	0.9813902	0.003039	19.588	blue	246.2399	150.7798	0.932176	0.020986	9.412	0.2254
AK234587.1	WDR41	brown	288.17	113.2748	0.95779445	0.0103424	2.758	blue	318.4464	192.5385	0.986809	0.001815	1.81	--
AK234725.1	GNB1	brown	213.46	94.77229	0.92165345	0.0260131	58.926	blue	325.8754	180.5844	0.970096	0.00618	51	--
AK234756.1		brown	253.82	129.0579	0.99642073	0.0002569	9.152	blue	346.4597	203.283	0.997839	0.000121	1.824	--
AK235272.1	PJA1	brown	164.45	89.47169	0.9028537	0.0358131	8.468	blue	356.6692	191.8201	0.983143	0.002621	13.528	0.2562
AK235462.1	PCBP1	brown	342.85	79.26756	0.85726182	0.0633315	167.73	blue	350.0833	166.1894	0.948729	0.013828	142.348	99
AK238658.1	SBDS	brown	193.57	110.4185	0.95951522	0.0097189	3.688	blue	346.4135	204.2818	0.999226	2.58E-05	3.912	--
AK236398.1	RNASEK	brown	222.3	110.2737	0.95911856	0.0098615	9.932	blue	298.0283	186.9441	0.980622	0.003229	3.968	0
AK236487.1		brown	114.22	49.46574	0.75852779	0.1371647	6.222	blue	357.2182	175.4628	0.961839	0.008897	3.304	--
AK239385.1	UTP18	brown	237.41	124.7103	0.98965298	0.0012615	49.456	blue	264.4885	149.1174	0.930825	0.021612	23.766	0.1679
AK239624.1	YWHAE	brown	280.02	107.8752	0.94950736	0.0135163	330.476	blue	205.7209	106.9822	0.853035	0.066121	245.668	4.6399
AK236671.1	ATP11A	brown	290.99	113.969	0.95818846	0.0101985	117.404	blue	325.6833	179.4151	0.968841	0.006572	85.648	--
AK236730.1	LMCD1	brown	199.63	99.96621	0.94285537	0.0162569	19.694	blue	339.8178	197.3246	0.990631	0.001087	13.482	0
AK239788.1	SMN1	brown	212.33	119.2819	0.97918908	0.0035926	13.296	blue	319.8552	195.6127	0.989758	0.001242	5.924	0.3603
AK240329.1	HSPA4	brown	172.18	82.67021	0.89685367	0.0391452	152.368	blue	266.4751	166.1274	0.954218	0.011678	132.538	99
AK234033.1	MRPL53	brown	180.13	108.7001	0.95704422	0.0106181	20.712	blue	352.2877	201.4777	0.995471	0.000366	21.762	--
AK237193.1	LOC100287741	brown	229.02	105.0811	0.94797778	0.0141318	4.17	blue	328.4439	202.9576	0.998799	4.99E-05	1.92	--
NM_001037146.2	RPSA	brown	267.45	127.3456	0.9909574	0.0010308	82.46	blue	333.354	202.314	0.997413	0.000158	11.95	--
NM_001097521.2	PGRMC2	brown	175.72	106.6943	0.95611742	0.0109621	50.094	blue	365.7034	182.6029	0.970617	0.006019	57.542	99
AK230942	ANXA1	brown	253.16	128.3025	0.9963858	0.0002607	5.85	blue	330.4868	179.2115	0.966796	0.007227	1.432	--
XM_001924669.1	APH1A	brown	214.49	118.5635	0.98131541	0.0030573	14.186	blue	226.5104	131.961	0.901225	0.036708	10.624	--
XM_001924618.1	TRAM1	brown	228	123.0102	0.98653055	0.0018727	128.9	blue	358.5112	183.6464	0.972315	0.005507	74.728	99
XM_001926198.1	PFDN5	brown	280.46	102.3997	0.93009181	0.0219543	42.51	blue	360.2501	195.3297	0.987204	0.001734	37.11	0
XM_001924658.1	IGF2BP2	brown	134.72	65.4409	0.84538504	0.0712645	6.894	blue	302.6184	189.4837	0.983541	0.002529	4.006	0
XM_001925435.1	RBM34	brown	288.85	117.8611	0.97008013	0.0061846	38.09	blue	347.7404	201.0551	0.995243	0.000394	6.956	0.3836
XM_001924505.1	PDGFRL	brown	214.67	115.0713	0.96959983	0.0063337	11.59	blue	222.1097	123.241	0.886324	0.045216	5.26	--
AJ000786.1	TMPRSS11F	brown	181.79	107.5492	0.95288038	0.0121911	65.132	blue	334.9394	199.4313	0.993603	0.000614	50.004	--
NM_213901.1	PCCB	brown	185.82	108.7788	0.95657893	0.0107904	4.214	blue	189.4151	108.1267	0.857876	0.062929	2.198	0
NM_214198.1	TGFB3	brown	290.68	95.41936	0.91020695	0.0318611	6.118	blue	324.113	171.6086	0.958423	0.010113	1.156	99
NM_214332.1	GNB2L1	brown	305.03	113.7983	0.95649466	0.0108216	505.572	blue	342.2337	203.629	0.998522	6.82E-05	152.87	--

AY609470.1	UFM1	brown	189.46	107.0403	0.95083522	0.0129893	41.19	blue	267.3106	135.3285	0.905021	0.034633	42.212	--
AY609565.1	MRPL1	brown	204.97	111.6087	0.96188346	0.0088819	6.312	blue	314.8444	163.2657	0.945166	0.015286	3.096	--
AY609717.1	CCT4	brown	228.74	120.7537	0.98204047	0.0028814	4.718	blue	308.1732	184.5448	0.9755	0.004587	1	0
AY609804.1	VMA21	brown	242.12	120.9104	0.98172491	0.0029575	3.918	blue	331.6564	200.566	0.995311	0.000385	4.796	99
AY610090.1	ELOF1	brown	223.61	126.9777	0.99593819	0.0003106	7.032	blue	361.9018	176.2346	0.96232	0.00873	3.19	--
AY610185.1	HSPB11	brown	255.27	122.1203	0.98062699	0.0032275	4.382	blue	241.5123	148.6904	0.927329	0.023259	1.02	99
CF363829		brown	219.55	106.2124	0.94971744	0.0134325	7.026	blue	356.1326	178.2647	0.964297	0.008055	1.6	--
BX666142		brown	224.41	112.2891	0.96561119	0.0076156	10.612	blue	350.8996	201.0348	0.994689	0.000464	9.65	99
BX667155		brown	253.55	110.6292	0.95643977	0.010842	3.59	blue	339.3718	198.3086	0.991844	0.000883	0.892	--
BX667181		brown	267.73	126.237	0.98855156	0.0014679	20.406	blue	324.8194	200.5935	0.995949	0.000309	3.754	--
BX667682		brown	275.3	126.4245	0.98853688	0.0014708	49.36	blue	267.5002	173.0413	0.96368	0.008264	33.854	0
BX670378		brown	156.14	84.08967	0.90419709	0.0350801	56.766	blue	249.5213	140.6268	0.914982	0.029375	53.774	--
BX676412		brown	216.23	106.5615	0.95414954	0.0117041	15.692	blue	347.7777	199.3441	0.992733	0.000743	14.174	--
BP160416		brown	256.85	128.5886	0.99531337	0.0003849	6.596	blue	345.5998	198.4721	0.991761	0.000897	1.24	--
CK449686		brown	317.57	98.846	0.91680523	0.0284435	8.85	blue	278.2547	173.3047	0.963499	0.008325	5.816	--
BX915109		brown	170.71	77.00479	0.88180825	0.0479028	3.55	blue	250.0752	148.196	0.927871	0.023001	2.386	0.3771
BX919706		brown	267.11	105.913	0.9399028	0.0175251	11.54	blue	269.1813	170.3647	0.958926	0.009931	5.776	--
BX923548		brown	287.8	118.6141	0.96881374	0.0065802	109.696	blue	349.3083	199.5289	0.992806	0.000732	38.686	0.2026
BX926488		brown	326.72	95.81403	0.90837408	0.0328323	595.22	blue	364.1545	183.9705	0.972627	0.005414	198.92	--
DN100858		brown	261.66	124.0349	0.98590792	0.0020039	7.258	blue	204.669	104.0649	0.847789	0.069635	2.65	99
DN101483		brown	262.12	123.8465	0.98437519	0.002339	3.594	blue	339.1724	188.2688	0.979092	0.003618	0.662	--
DN103747		brown	208	99.52193	0.93347664	0.0203898	10.962	blue	295.5339	186.3529	0.979478	0.003518	8.178	--
DN106205		brown	244.2	102.1945	0.9346465	0.0198579	55.258	blue	352.2045	201.0379	0.994884	0.000439	47.888	99
DN107719		brown	111.83	58.76644	0.82581866	0.0849475	127.484	blue	188.1681	91.01389	0.815179	0.092691	213.292	--
DN125721		brown	240.29	118.84	0.97897875	0.0036471	8.276	blue	332.3567	202.9288	0.998408	7.62E-05	3.72	--
AJ949964		brown	250.37	120.9851	0.97889436	0.003669	0.274	blue	358.2577	175.6559	0.962018	0.008835	12.588	--
AJ958642		brown	164.32	92.17831	0.91035605	0.0317825	54.132	blue	311.6592	175.4183	0.963422	0.008351	35.902	--
AJ944603		brown	226.19	119.0934	0.97834607	0.0038126	20.596	blue	312.6087	171.8152	0.95868	0.01002	8.724	--
CV876445		brown	261.43	123.4503	0.98547133	0.0020976	5.1	blue	348.8096	192.1888	0.984017	0.00242	1.562	--
DY424635		brown	322.16	93.92699	0.90643725	0.0338686	17.502	blue	256.7648	158.1641	0.942071	0.016591	8.336	0
DY684916		brown	179.45	94.6511	0.93063215	0.021702	7.724	blue	321.296	183.7266	0.974331	0.004918	6.382	--
CT863733		brown	316.34	94.89379	0.9040163	0.0351784	13.616	blue	366.3865	189.6238	0.979633	0.003479	6.94	--
AK236628	ATIC	brown	283.16	105.1602	0.93683932	0.0188732	5.838	blue	358.1656	191.1674	0.981712	0.002961	1.328	0.0443
DT321969		brown	297.27	110.5478	0.95390552	0.0117973	4.782	blue	285.3207	172.9709	0.962187	0.008776	8.93	0
DT324339		brown	252.99	125.449	0.98968275	0.001256	9.312	blue	339.9332	202.9129	0.998124	9.75E-05	3.736	--

EV857330	brown	246.35	129.7004	0.99915677	2.94E-05	13.762	blue	334.5526	197.1242	0.990602	0.001092	4.958	--
EW023671	brown	265.31	114.068	0.96084756	0.009245	2.774	blue	348.698	198.0497	0.991515	0.000937	0.468	--
EW083818	brown	206.13	91.89037	0.91357953	0.0300987	2.984	blue	300.5677	175.8191	0.965801	0.007553	0.612	--
EW093873	brown	257.62	124.6871	0.98901808	0.0013792	6.754	blue	298.6902	191.0741	0.985923	0.002001	3.92	--
EW136988	brown	246.5	127.2878	0.99442636	0.0004991	19.844	blue	336.8562	198.1804	0.992334	0.000805	7.116	--
EW159788	brown	190.11	108.3996	0.95396911	0.011773	17.614	blue	363.1592	193.4346	0.984447	0.002323	8.05	--
EW167492	brown	180.49	98.87471	0.94070302	0.0171784	7.038	blue	324.1792	195.909	0.989895	0.001217	7.222	--
EW189192	brown	142.65	66.90363	0.85392892	0.0655282	19.57	blue	273.5976	156.0351	0.939479	0.017709	20.838	--
EW191982	brown	247.75	122.505	0.9834288	0.0025544	3.514	blue	326.2109	193.5343	0.987211	0.001733	3.838	--
EW192633	brown	250.76	124.8443	0.98927654	0.0013309	4.216	blue	298.2098	186.6372	0.980186	0.003338	1.216	--
EW196387	brown	336.56	78.21285	0.85266758	0.0663656	6.33	blue	296.7011	188.0042	0.982224	0.002838	2.056	--
EW201887	brown	260.04	128.6991	0.99527079	0.0003901	8.12	blue	339.018	195.9195	0.988665	0.001446	3.834	--
EW206037	brown	236.99	120.5079	0.98116663	0.0030938	4.664	blue	332.4293	203.1793	0.998719	5.50E-05	3.77	--
EW247940	brown	228.31	124.0724	0.98872539	0.0014347	9.866	blue	351.058	199.3125	0.993059	0.000693	4.226	--
EW249284	brown	253.38	129.4209	0.99720333	0.0001775	3.47	blue	347.1372	200.746	0.994917	0.000435	0.544	--
EW310470	brown	154.56	90.16454	0.90550765	0.0343697	2.694	blue	367.12	189.5647	0.979463	0.003522	7.45	--
EW322856	brown	172.27	91.06197	0.9077414	0.0331697	10.148	blue	333.3411	187.6131	0.979035	0.003633	11.502	--
EW359956	brown	243.06	129.0984	0.9977287	0.0001299	6.334	blue	256.8004	142.8111	0.920372	0.026649	1.95	--
EW417768	brown	251.81	127.6816	0.99538165	0.0003765	10.086	blue	257.6308	162.0961	0.948666	0.013854	2.65	--
EW453494	brown	272.91	126.619	0.98881572	0.0014175	4.398	blue	323.8167	201.8187	0.997674	0.000135	1.726	--
EW519341	brown	236.46	129.4467	0.99953871	1.19E-05	33.396	blue	320.1792	198.9515	0.994662	0.000468	12.716	--
EW521970	brown	254.72	129.822	0.99819557	9.20E-05	12.6	blue	327.7698	188.4741	0.980739	0.0032	5.176	--
EW548211	brown	204.75	118.1622	0.9769652	0.0041822	10.562	blue	362.8624	184.089	0.97286	0.005345	4.894	--
EW572865	brown	264.42	128.1205	0.99355189	0.000621	162.468	blue	344.2656	201.3455	0.995745	0.000333	42.382	99
EW577626	brown	243.65	125.5164	0.99019579	0.0011636	9.46	blue	346.4934	182.7073	0.972589	0.005425	6.848	--
EW588646	brown	258.44	129.1304	0.99608783	0.0002936	11.696	blue	330.3357	198.2883	0.99277	0.000737	4.418	--
EW621683	brown	245.52	129.7997	0.9992501	2.46E-05	10.638	blue	336.3524	199.9156	0.994565	0.000481	5.416	--
BE233268	brown	282.99	106.8937	0.94016013	0.0174134	4.786	blue	354.6322	198.7065	0.992074	0.000846	1.134	--
BG896003	brown	276.13	122.7158	0.98183631	0.0029306	10.628	blue	155.5255	78.66299	0.789915	0.111878	4.126	--
BQ599187	brown	198.44	105.3165	0.94635394	0.014795	6.576	blue	305.3735	193.8136	0.988878	0.001406	4.19	--
BI399871	brown	249.34	126.9462	0.99290757	0.0007163	10.162	blue	339.1626	201.3121	0.996389	0.00026	7.798	--
BI181083	brown	223.43	124.436	0.98976331	0.0012414	9.148	blue	351.2506	197.1334	0.990198	0.001163	3.782	99
BM190542	brown	259.14	129.0709	0.99611186	0.0002909	12.012	blue	340.9245	204.1696	0.999415	1.70E-05	6.87	--

Table S4-2 Module eigengene and its evolutionary rates in the common module between LT and LDE (prenatal_turquoise vs turquoise).

pig_Genebank	pig_Gene	LDEModule	kTotal	kWithin	eigencorr	eigenpval	meanExpr	LTModule	kTotal	kWithin	eigencorr	eigenpval	meanExpr	Ka/Ks
NM_001044558.1	TMEM59	turquoise	1081.5	1080.4645	0.9090526	0.03247164	18.144	turquoise	977.388	922.92	0.9782425	0.0038399	32.088	0
NM_001044598.2	DIRAS3	turquoise	1553.6	1553.1829	0.9965278	0.00024548	2.692	turquoise	1005.67	979.158	0.9905181	0.0011068	16.292	99
AK230645.1	LIPA	turquoise	1236.5	1235.7116	0.9389475	0.01794204	11.15	turquoise	559.365	538.077	0.8446218	0.0717842	12.822	0.842
AK230737.1	FAM46A	turquoise	809.13	807.57706	0.8473851	0.06990828	32.576	turquoise	374.311	277.815	0.7015727	0.1866919	44.822	--
AK234167.1	HOXB7	turquoise	1050.5	1049.4588	0.9012849	0.03667514	1.09	turquoise	937.786	918.325	0.974139	0.0049729	4.532	99
AK234174.1	TMEM222	turquoise	816.27	815.51254	0.8586057	0.06245244	31.632	turquoise	277.926	178.596	0.592857	0.292057	26.714	99
AK234189.1	FDFT1	turquoise	1504.4	1503.9199	0.9881316	0.00154935	4.612	turquoise	979.329	904.481	0.9729634	0.0053149	8.492	0.034
AK234218.1	SNX17	turquoise	1391.3	1390.7561	0.9720354	0.00559007	17.362	turquoise	907.737	859.838	0.9634239	0.0083509	27.838	0
AK234327.1	TSC22D1	turquoise	814.68	811.68153	0.8482387	0.06933194	38.908	turquoise	844.141	798.116	0.9411075	0.017004	53.08	99
AK234333.1	CNN2	turquoise	1523.6	1523.0436	0.9935185	0.00062578	61.454	turquoise	1034.74	973.34	0.990694	0.0010761	55.392	99
AK234341.1	FXYD6	turquoise	326.87	306.32942	0.6003028	0.28444859	110.692	turquoise	1008.64	940.691	0.9824192	0.0027909	150.458	99
AK234347.1	ABHD14A	turquoise	975.91	973.15797	0.8906305	0.0426994	12.63	turquoise	803.44	783.003	0.9361852	0.0191652	8.996	0.41
AK237362.1	MARVELI	turquoise	1214.9	1213.1481	0.9404773	0.01727594	74.556	turquoise	1029.26	970.849	0.9898799	0.0012202	71.51	--
AK237390.1	FDFT1	turquoise	830.3	826.91594	0.855621	0.06441002	44.838	turquoise	959.778	925.32	0.9768476	0.0042142	49.372	--
AK237513.1	PLOD1	turquoise	174.67	81.931832	0.2240019	0.71719532	55.918	turquoise	492.339	371.651	0.7585681	0.1371313	37.77	0
AK237525.1	RP9P	turquoise	1368.6	1367.7254	0.9696075	0.00633132	34.53	turquoise	834.403	765.455	0.931019	0.021522	27.012	99
AK240476.1	MIR2277	turquoise	1473.2	1472.7531	0.9858082	0.00202518	5.86	turquoise	1020.55	992.401	0.9941309	0.0005393	7.646	0
AK240506.1	GAP43	turquoise	1505.4	1504.8222	0.990893	0.00104184	4.316	turquoise	1026.97	997.858	0.9954013	0.0003741	20.668	99
AK240553.1	S100B	turquoise	1319.7	1318.9251	0.9548266	0.01144702	25.808	turquoise	882.733	866.376	0.9594767	0.0097327	31.344	--
AK230973.1	DDX1	turquoise	1460.4	1459.6857	0.9838022	0.00246865	67.054	turquoise	315.018	264.575	0.6934088	0.1941405	81.49	0
AK234367.1	PTN	turquoise	272.89	234.78338	0.5153202	0.37419669	92.414	turquoise	456.482	419.781	0.7948687	0.1080303	54.692	--
AK234534.1	CSRP1	turquoise	1542.7	1542.2685	0.9951306	0.0004076	54.66	turquoise	905.776	851.344	0.9595967	0.0096897	51.03	0
AK237582.1	SRPRB	turquoise	1499.4	1498.8314	0.9896313	0.00126544	12.326	turquoise	967.681	918.84	0.9779268	0.0039236	18.184	0.418
AK240610.1	FZD5	turquoise	1427.1	1426.265	0.9788141	0.00368995	21.368	turquoise	704.15	594.254	0.8721817	0.0537919	20.56	99
AK231087.1	RGS4	turquoise	1430.1	1429.6119	0.9777297	0.00397619	3.168	turquoise	657.616	620.213	0.8795463	0.0492672	10.296	99
AK231100.1	COPB1	turquoise	1571.2	1570.8068	0.9996442	8.06E-06	2.112	turquoise	197.912	137.051	0.5656078	0.3203369	10.746	0
AK231154.1	C1orf35	turquoise	129.71	102.62942	0.3696599	0.5402849	25.346	turquoise	874.652	852.837	0.9573262	0.0105142	20.31	--
AK231177.1	CDK19	turquoise	1417.8	1417.3336	0.9731295	0.00526612	10.27	turquoise	88.6916	62.7865	0.3960835	0.5092061	20.248	--
AK231217.1	FADS1	turquoise	405.92	393.58023	0.6725998	0.21349156	15.708	turquoise	1046.38	994.889	0.9960211	0.0003011	11.298	0.038
AK234559.1	FAM176B	turquoise	956.74	953.23068	0.8827745	0.04732378	64.824	turquoise	734.296	613.244	0.8786462	0.0498134	67.114	--
AK234590.1	YPEL4	turquoise	1548.2	1547.7895	0.996807	0.00021649	1.214	turquoise	903.156	883.776	0.965699	0.0075866	2.006	0
AK234663.1	RAB3A	turquoise	1470.6	1470.0807	0.9817578	0.00294955	3.88	turquoise	1034.28	1003.66	0.996949	0.0002022	13.654	0
AK237762.1	DCTN1	turquoise	975.06	973.10739	0.8941586	0.04067235	55.238	turquoise	770.902	729.748	0.9260926	0.0238502	48.612	0
AK237827.1	MARCKS	turquoise	985.25	984.29528	0.89881	0.03804835	1002.29	turquoise	930.158	888.458	0.9677235	0.0069271	1112.53	--

AK237870.1		turquoise	1546.3	1545.8307	0.997151	0.00018247	81.3	turquoise	997.006	920.901	0.9772748	0.0040983	81.514 --
AK231257.1	ELAVL4	turquoise	1553.6	1553.1829	0.9965278	0.00024548	1.748	turquoise	1015.27	988.541	0.9929221	0.0007141	5.008 --
AK231416.1	C7orf36	turquoise	1128.4	1127.6659	0.9243975	0.02466897	18.944	turquoise	256.53	196.99	0.6197904	0.264789	21.152 0.457
AK234779.1	RAB1A	turquoise	1258.8	1257.4658	0.9504041	0.01315966	108.706	turquoise	937.333	885.019	0.9668265	0.0072168	117.212 99
AK234832.1	BTBD3	turquoise	1509	1508.4353	0.9916703	0.00091146	77.15	turquoise	1021.14	959.516	0.9874148	0.0016916	81.33 --
AK237968.1	ENO2	turquoise	1433.6	1433.2155	0.9779079	0.00392867	3.642	turquoise	1025.55	996.98	0.9951566	0.0004043	11.098 0.06
AK238007.1	KIAA0892	turquoise	1434.5	1434.1277	0.9766456	0.00426933	6.08	turquoise	841.137	805.351	0.9474823	0.0143331	8.876 --
AK231529.1	MAPK3	turquoise	879.1	876.89258	0.8562247	0.06401258	48.836	turquoise	993.757	966.72	0.9875325	0.001668	73.828 --
AK231444.1	BCL11A	turquoise	1530.5	1530.149	0.9931124	0.00068547	12.142	turquoise	1019.68	992.16	0.9938692	0.0005757	22.828 --
AK234973.1	CMAS	turquoise	1421.4	1420.7424	0.979466	0.00352129	28.906	turquoise	785.973	697.674	0.9093935	0.032291	42.862 99
AK231701.1	SNORA60	turquoise	156.49	150.73046	0.5174815	0.3718402	57.002	turquoise	1014.94	979.292	0.9916846	0.0009091	46.906 --
AK235000.1	TM9SF1	turquoise	1321.8	1320.9456	0.9630445	0.00848066	39.186	turquoise	304.288	218.005	0.6375396	0.2472149	59.764 99
AK235006.1	ERGIC1	turquoise	1351.8	1350.7921	0.9656324	0.00760863	25.418	turquoise	690.68	569.003	0.8594083	0.0619293	48.126 --
AK235026.1	CTSC	turquoise	1391.5	1390.9255	0.9713223	0.00580462	42.016	turquoise	1026.26	979.542	0.9921598	0.0008324	50.956 --
AK235041.1	CNRIP1	turquoise	590.25	585.25802	0.7665103	0.13058933	36.29	turquoise	948.726	907.491	0.9740266	0.0050053	51.542 0
AK235084.1	MORF4L2	turquoise	1091.5	1089.2888	0.9164592	0.02861959	218.056	turquoise	613.673	503.226	0.8301772	0.0818358	236.442 0
AK235131.1	U2AF1	turquoise	787.73	783.81118	0.8421263	0.07349149	73.72	turquoise	1051.04	1000.95	0.9971477	0.0001828	82.9 --
AK231548.1	PIGC	turquoise	1257.6	1256.1552	0.9491012	0.01367892	64.736	turquoise	820.495	720.032	0.9166274	0.0285339	102.65 0
AK231575.1	SCAMP4	turquoise	975.75	973.03587	0.8929328	0.0413731	23.474	turquoise	330.086	238.204	0.6528719	0.2323005	18.768 0.258
AK231676.1	BAMBI	turquoise	1095.3	1093.5323	0.9184648	0.0276037	4.392	turquoise	1006.23	967.808	0.9889208	0.0013976	6.458 0.146
AK235322.1	TRAPPC3	turquoise	1214.1	1212.7913	0.9367526	0.01891187	54.846	turquoise	566.939	516.788	0.8429721	0.0729114	54.528 0
AK235336.1	CLPTM1L	turquoise	1366.4	1365.5455	0.9674261	0.00702273	22.282	turquoise	519.745	450.599	0.8046377	0.1005634	28.988 99
AK235358.1		turquoise	1511.5	1511.1228	0.9899021	0.00121624	87.488	turquoise	904.061	807.05	0.9454098	0.0151851	103.698 0
AK235359.1	11-Sep	turquoise	1447.4	1446.6992	0.9818666	0.00292326	101.738	turquoise	956.659	867.003	0.9624796	0.0086751	125.804 0
AK238171.1	PTPRF	turquoise	1506.4	1505.8569	0.9899714	0.00120377	21.126	turquoise	1051.35	1011.93	0.999516	1.28E-05	29.864 --
AK238267.1	KIAA1737	turquoise	975.15	971.95203	0.8882382	0.04409159	25.586	turquoise	1035.32	998.623	0.9964904	0.0002495	25.792 --
AK231855.1	ABHD11	turquoise	1279	1277.6458	0.9525646	0.01231325	48.664	turquoise	884.152	776.769	0.936352	0.0190907	50.232 99
AK231895.1	NUDT21	turquoise	235.26	211.67323	0.519946	0.36915748	30.526	turquoise	1011.16	961.773	0.9872916	0.0017165	29.794 99
AK235196.1		turquoise	1546.2	1545.6807	0.9971914	0.0001786	64.394	turquoise	321.426	224.453	0.6358267	0.2488967	100.08 --
AK235227.1	FBXL2	turquoise	1159.4	1157.9101	0.9287392	0.0225898	5.128	turquoise	971.383	942.263	0.9818285	0.0029325	5.506 --
AK235381.1	NAP1L3	turquoise	1430.7	1430.2386	0.9762307	0.00438333	10.81	turquoise	902.266	872.469	0.962165	0.008784	22.288 0.266
AK235410.1	DDX6	turquoise	566.42	560.91524	0.7661381	0.13089375	12.432	turquoise	591.324	573.904	0.8648802	0.0583994	22.202 99
AK235434.1	EIF1B	turquoise	338.27	319.9875	0.6319611	0.2527033	58.656	turquoise	670.214	637.113	0.8854213	0.045749	58.6 99
AK235450.1	GNB2	turquoise	1435.3	1434.6779	0.9802268	0.00332779	247.09	turquoise	1049.02	1000.1	0.9968576	0.0002114	252.93 --
AK235476.1	RRAGB	turquoise	125.67	99.99391	0.39005	0.51626991	19.072	turquoise	1010.65	983.441	0.9916434	0.0009159	26.274 --
AK235477.1	TNRC6A	turquoise	1268.2	1267.6898	0.9517227	0.0126409	106.994	turquoise	1002.23	968.934	0.9885947	0.0014597	97.93 --

AK235508.1	PAPSS1	turquoise	1381	1380.0376	0.9709571	0.00591551	76.292	turquoise	873.659	760.305	0.9304201	0.0218009	69.356	--
AK235521.1	AUP1	turquoise	966.08	963.63862	0.8885387	0.04391596	16.928	turquoise	353.631	285.486	0.6947987	0.1928666	17.57	99
AK235558.1	PPM1M	turquoise	1373.5	1372.6713	0.9684662	0.0066901	8.904	turquoise	1041.19	1001.59	0.9969227	0.0002048	8.27	0
AK235559.1	HMGCS1	turquoise	1535.8	1535.3307	0.9954299	0.00037061	56.876	turquoise	1004.16	951.031	0.9845169	0.0023073	100.382	99
AK232310.1	ALDH1A1	turquoise	100.27	52.238913	0.1996886	0.74744859	0.284	turquoise	618.487	596.83	0.8685423	0.0560736	4.66	0
AK232326.1	WWP2	turquoise	1555.8	1555.4291	0.9968502	0.00021211	42.678	turquoise	1051.3	1012.41	0.9995577	1.12E-05	16.728	--
AK232329.1	CNDP2	turquoise	1458.4	1457.8276	0.98377	0.00247601	30.666	turquoise	743.099	605.066	0.8750617	0.0520075	37.664	0.145
AK235612.1	SERPINH1	turquoise	645.35	641.88888	0.8019326	0.10261482	561.656	turquoise	815.046	698.135	0.9098374	0.0320561	411.43	0
AK235633.1	TRIM54	turquoise	788.98	785.42839	0.8441136	0.07213087	7.53	turquoise	950.431	917.995	0.9752177	0.0046658	10.114	--
AK235634.1	SMAP1	turquoise	1322.5	1321.4	0.9582408	0.01017943	54.46	turquoise	924.668	851.832	0.958659	0.0100275	57.382	99
AK235644.1	RAB5C	turquoise	309.08	297.28238	0.6280223	0.25659794	99.152	turquoise	1022.91	991.458	0.9941631	0.0005348	86.814	99
AK235648.1	CD24	turquoise	1549.3	1548.8822	0.9957464	0.0003328	35.246	turquoise	1015.83	988.989	0.9930476	0.0006952	73.526	0.977
AK235657.1	CDK8	turquoise	1371.4	1370.801	0.9645176	0.00798047	25.382	turquoise	922.465	845.464	0.9573505	0.0105053	28.56	--
AK235709.1	FMNL3	turquoise	1335.6	1334.6255	0.9623393	0.00872364	35.576	turquoise	334.738	251.316	0.670739	0.2152468	28.362	--
AK238597.1	C16orf72	turquoise	378.19	367.24848	0.6672275	0.21856998	155.276	turquoise	1039.58	990.245	0.9943558	0.0005086	160.174	--
AK238611.1	CD200	turquoise	1491	1490.6302	0.9852805	0.002139	11.066	turquoise	576.755	560.264	0.8546241	0.0650681	19.624	--
AK238616.1	ADRBK1	turquoise	1469.8	1469.1267	0.986119	0.00195912	27.102	turquoise	737.711	648.921	0.8936497	0.0409628	33.42	--
AK238622.1	PA2G4	turquoise	1565.7	1565.2339	0.9994687	1.47E-05	50.96	turquoise	902.641	808.233	0.9467037	0.0146513	73.38	99
AK238655.1	hCG_3898	turquoise	453.1	448.4124	0.7266577	0.16433606	34.614	turquoise	975.812	949.493	0.983251	0.0025955	28.56	--
AK238706.1	LXN	turquoise	794.6	792.22095	0.8342224	0.07898004	61.432	turquoise	369.004	330.934	0.7281506	0.1630317	71.08	99
AK232354.1	SLC37A4	turquoise	1295.9	1294.7383	0.9548978	0.01142013	7.282	turquoise	1045.62	996.058	0.9959024	0.0003147	18.096	99
AK232477.1	PRKCZ	turquoise	1521.2	1520.8189	0.9911531	0.00099757	0.982	turquoise	1010.79	984.754	0.9919484	0.0008662	5.846	0
AK235788.1	RARRES1	turquoise	321.44	317.55097	0.6464913	0.23847643	3.758	turquoise	990.283	965.549	0.9870059	0.0017746	5.198	0
AK235829.1	KIAA0174	turquoise	1228.7	1227.7305	0.9468431	0.01459417	71.226	turquoise	617.239	560.685	0.8608854	0.06097	101.35	0
AK235833.1	BECN1	turquoise	407.89	400.22433	0.6911324	0.19623215	56.152	turquoise	921.263	824.15	0.9502412	0.0132242	63.248	0
AK235860.1	CDK9	turquoise	329.04	318.34785	0.6437645	0.24112922	63.136	turquoise	792.856	753.886	0.927944	0.0229662	67.562	0
AK235879.1	ANTXR1	turquoise	1457.4	1456.7396	0.9845914	0.00229072	11.168	turquoise	345.72	316.006	0.7430308	0.1501977	12.506	--
AK235881.1	PIRC34	turquoise	1115.1	1114.4304	0.9250588	0.02434847	56.246	turquoise	961.749	881.455	0.9665865	0.007295	107.178	99
AK235892.1	SEC11A	turquoise	1107.6	1105.8042	0.9232587	0.02522403	45.186	turquoise	1042.66	1008.49	0.9984843	7.08E-05	43.424	--
AK235949.1	GPR125	turquoise	1359.3	1358.4546	0.968024	0.00683088	57.056	turquoise	903.962	808.291	0.9454254	0.0151786	59.524	0.082
AK235971.1	GAS2	turquoise	1526	1525.4991	0.993469	0.00063296	90.356	turquoise	724.268	578.965	0.8640621	0.058923	87.472	--
AK238760.1	PPIL1	turquoise	1000.7	997.49471	0.892947	0.04136492	32.854	turquoise	1052.42	1003.09	0.9976686	0.0001351	34.748	0
AK238779.1	MARCKSl	turquoise	261.3	222.64196	0.5009871	0.38991415	60.228	turquoise	1009.72	982.821	0.9915124	0.0009375	43.584	--
AK238807.1	PRELID1	turquoise	292.95	258.33517	0.5465933	0.34045755	95.998	turquoise	950.845	859.997	0.9605845	0.0093379	83.75	--
AK238832.1	TNRC6A	turquoise	131.14	108.53454	0.3946681	0.51086146	41.966	turquoise	660.548	635.179	0.885056	0.0459653	36.522	--
AK238888.1	HP1BP3	turquoise	163.18	111.93457	0.3470361	0.56717657	20.862	turquoise	518.909	503.899	0.8339547	0.0791681	38.736	99

AK232622.1	GABARAI	turquoise	779.81	775.4585	0.8381612	0.07622959	18.582	turquoise	989.785	966.602	0.9870947	0.0017565	17.62	--
AK232649.1	STX12	turquoise	1105.3	1104.0661	0.9189823	0.0273435	35.31	turquoise	875.967	852.125	0.9560567	0.0109847	97.338	0.213
AK236042.1	RAD51L3	turquoise	1519.9	1519.4991	0.990114	0.0011782	8.302	turquoise	889.799	787.755	0.9392333	0.017817	9.17	0.258
AK236110.1	RPA1	turquoise	1481.5	1480.8763	0.9870932	0.00175679	40.794	turquoise	822.935	719.544	0.9199834	0.0268424	45.538	0
AK236160.1	CHST10	turquoise	1365.4	1364.5436	0.969466	0.00637544	51.184	turquoise	137.237	103.194	0.5176487	0.371658	60.764	0
AK238961.1	AES	turquoise	1355	1353.9119	0.9676264	0.00695826	198.012	turquoise	986.655	932.459	0.9801195	0.0033549	189.538	--
AK239055.1	RNF207	turquoise	1470.3	1469.6471	0.985299	0.00213499	21.432	turquoise	234.648	167.851	0.5807485	0.3045405	24.988	--
AK239094.1	SFRP1	turquoise	1446.4	1445.6583	0.981041	0.00312478	47.83	turquoise	1042.2	1008.22	0.9982801	8.56E-05	38.442	--
AK236193.1	ARF3	turquoise	375.35	356.97992	0.6376148	0.24714105	23.482	turquoise	980.799	954.877	0.9843812	0.0023377	15.072	--
AK236213.1	CDC42EP4	turquoise	1537.9	1537.5472	0.9944199	0.00049996	2.924	turquoise	402.388	296.477	0.7085529	0.1803895	4.306	99
AK236249.1	VAT1	turquoise	1398.6	1397.6478	0.9747364	0.00480201	75.79	turquoise	964.761	901.671	0.9725256	0.0054441	85.824	0
AK236339.1	PSMD2	turquoise	973.85	970.67084	0.8880222	0.04421804	91.254	turquoise	1052.63	1011.17	0.9993167	2.14E-05	83.81	99
AK239198.1	PIK3R3	turquoise	1031.8	1028.8306	0.9002687	0.03723706	14.132	turquoise	933.664	852.757	0.9579483	0.0102861	19.874	--
AK239277.1	LIMD2	turquoise	1110.4	1108.771	0.918885	0.02739236	9.066	turquoise	860.955	797.137	0.9434161	0.0160196	10.954	0.246
AK239294.1	RSL24D1	turquoise	1007.8	1005.5343	0.9004981	0.03710996	52.868	turquoise	1009.83	977.205	0.991137	0.0010003	49.97	--
AK239337.1	AFF3	turquoise	1319.2	1318.1551	0.9592062	0.0098299	10.818	turquoise	976.207	926.601	0.9783702	0.0038063	9.232	--
AK239352.1	PIAS3	turquoise	1442.9	1442.2126	0.9818321	0.00293159	24.794	turquoise	915.864	821.916	0.9495287	0.0135078	25.198	--
AK230496.1	CASP3	turquoise	938.65	935.34937	0.8794645	0.04931672	72.87	turquoise	839.294	808.58	0.9447682	0.0154521	60.462	0.172
AK230522.1	FUT8	turquoise	1527.9	1527.4485	0.9926244	0.00075954	14.82	turquoise	1042.22	992.211	0.9949966	0.0004245	14.592	0
AK230524.1	VPS4B	turquoise	1267.5	1266.1327	0.9506886	0.01304717	6.93	turquoise	711.329	588.325	0.8694186	0.0555215	12.24	--
AK230555.1	PPIL4	turquoise	1256.6	1255.4375	0.9444732	0.01557532	18.608	turquoise	328.05	291.34	0.7214926	0.1688719	25.69	0.497
AK230594.1	B4GALT4	turquoise	1516.5	1516.0572	0.9898564	0.0012245	3.29	turquoise	1025.22	990.345	0.9940561	0.0005496	3.838	0
AK230621.1	DCAF10	turquoise	1405.5	1404.9422	0.976987	0.00417626	44.86	turquoise	672.951	528.094	0.8424845	0.0732457	51.622	--
AK232758.1	HABP4	turquoise	814.1	810.11672	0.8476255	0.06974584	17.754	turquoise	945.528	887.298	0.9699522	0.0062242	20.234	--
AK236374.1	NUP93	turquoise	1143.9	1142.0118	0.9277435	0.02306136	32.044	turquoise	1049.75	1003.35	0.9979219	0.0001137	38.648	--
AK236407.1	ANO10	turquoise	1544.9	1544.4869	0.9964564	0.00025309	7.48	turquoise	267.798	225.404	0.6762366	0.2100726	8.818	0
AK236435.1	WDR13	turquoise	645.03	638.70145	0.7932789	0.10926061	34.206	turquoise	720.348	676.21	0.9038541	0.0352667	44.142	99
AK236453.1	FAM129A	turquoise	1443.4	1442.9479	0.9765255	0.00430223	14.526	turquoise	800.892	707.295	0.9132581	0.0302653	20.676	0.522
AK236499.1	SLC20A1	turquoise	1527.8	1527.2592	0.9944829	0.00049153	9.328	turquoise	704.433	586.631	0.8692225	0.0556449	21.882	--
AK232930.1	AMN1	turquoise	1483.1	1482.4659	0.9882016	0.00153567	14.884	turquoise	574.223	452.193	0.8144765	0.0932098	30.826	--
AK239359.1	HCFC1	turquoise	1355.1	1354.0818	0.9684147	0.00670644	36.628	turquoise	899.976	795.21	0.9418801	0.0166724	40.078	--
AK239497.1	SFRS3	turquoise	536.07	523.39729	0.7320339	0.15965292	232.17	turquoise	851.322	770.265	0.9337878	0.0202479	177.418	99
AK239620.1	ASXL1	turquoise	1139.8	1139.2752	0.9278074	0.02303104	69.82	turquoise	722.473	705.099	0.9093231	0.0323282	55.328	--
AK239666.1	MAN2B1	turquoise	1166.6	1164.973	0.9337175	0.02027995	22.234	turquoise	344.729	273.398	0.6909015	0.1964447	14.65	99
AK239676.1	RSL1D1	turquoise	643.29	642.55311	0.8060999	0.09945988	38.74	turquoise	1022.58	972.912	0.9904545	0.0011179	31.068	--
AK239723.1	BASP1	turquoise	1568.9	1568.5466	0.9993662	1.92E-05	58.992	turquoise	1031.47	998.938	0.9958177	0.0003245	91.154	99

AK232985.1	ETV1	turquoise	1460.8	1460.0958	0.9834954	0.00253901	6.554	turquoise	1039.34	993.398	0.9951362	0.0004069	12.92	--
AK236576.1	PDIA4	turquoise	134.26	76.24129	0.2472985	0.68836924	191.344	turquoise	1001.49	964.225	0.9883167	0.0015133	121.488	0.056
AK236599.1	NAE1	turquoise	1541.2	1540.7315	0.996345	0.00026511	8.912	turquoise	637.079	547.088	0.8566701	0.0637198	15.84	--
AK236654.1	ACTN1	turquoise	1466.4	1465.7069	0.9830492	0.00264247	42.71	turquoise	937.853	894.061	0.9694252	0.0063882	44.298	0
AK236655.1	ATP6V1B2	turquoise	1101.1	1099.9214	0.9090826	0.03245571	80.03	turquoise	991.734	963.744	0.9868588	0.0018048	88.468	--
AK236660.1	CSNK1E	turquoise	697.33	693.91906	0.808429	0.09770963	34.586	turquoise	1046.56	1005.54	0.9982061	9.12E-05	37.81	0
AK239758.1	RNPS1	turquoise	1498.6	1498.1953	0.9878686	0.00160106	37.594	turquoise	532.376	395.774	0.7740073	0.1245019	67.028	--
AK239777.1	NUP50	turquoise	969.11	967.25971	0.8880037	0.04422883	23.882	turquoise	596.247	447.919	0.8039438	0.1010885	33.034	0.101
AK239913.1	PTH	turquoise	1553.4	1553.0099	0.9968909	0.00020801	23.416	turquoise	502.242	471.359	0.8137261	0.0937646	33.19	--
AK236816.1	BACH2	turquoise	1366.4	1365.6735	0.9637937	0.00822501	18.052	turquoise	1024.5	982.653	0.9925527	0.0007706	16.782	--
AK233216.1	EFHA1	turquoise	61.945	52.519007	0.3001667	0.62363516	0.674	turquoise	327.278	285.105	0.7062884	0.1824274	5.9	--
AK233236.1	EPB41	turquoise	1368.6	1367.5526	0.9698726	0.0062489	40.63	turquoise	999.867	927.593	0.9794512	0.0035251	34.198	--
AK233255.1	C10orf35	turquoise	1553.6	1553.1829	0.9965278	0.00024548	0.378	turquoise	1031.01	1001.09	0.9962725	0.000273	2.378	--
AK233339.1	ZNF516	turquoise	1552	1551.6272	0.9972804	0.00017019	26.128	turquoise	956.803	900.341	0.9717726	0.0056688	34.04	--
AK239962.1	TWIST2	turquoise	1558.9	1558.5429	0.9979279	0.0001132	9.9	turquoise	1044.92	1006.69	0.9981719	9.38E-05	5.008	99
AK239969.1	TMEM127	turquoise	1122.5	1120.7757	0.925224	0.02426862	15.384	turquoise	196.297	151.348	0.5601645	0.3260653	22.32	--
AK240077.1	SUMO2	turquoise	274.6	240.8479	0.5283825	0.36000968	299.774	turquoise	1042.84	991.534	0.9948147	0.0004479	201.248	99
AK240079.1	PDGFRB	turquoise	115.58	89.566946	0.3291437	0.58861604	31.662	turquoise	186.808	122.817	0.5011542	0.38973	28.994	--
AK240098.1	PAFAH1B	turquoise	1321.9	1320.8368	0.9622008	0.00877161	28.906	turquoise	937.381	894.067	0.9695596	0.0063462	27.968	--
AK240146.1	CXCL12	turquoise	1503.2	1502.7606	0.9899194	0.00121313	40.826	turquoise	707.542	689.501	0.9054959	0.034376	75.204	--
AK233368.1	NAP1L1	turquoise	215.83	167.20457	0.4325895	0.46690621	78.666	turquoise	324.313	249.449	0.6621786	0.2233727	66.846	0
AK233462.1	FEZ1	turquoise	1558.6	1558.154	0.9979055	0.00011503	7.258	turquoise	1017	989.791	0.9933479	0.0006506	24.05	0
AK233502.1	TCFL5	turquoise	1518.5	1517.9607	0.9925653	0.00076868	25.046	turquoise	742.879	600.936	0.8743181	0.0524664	28.232	--
AK237028.1	FCHO1	turquoise	162.21	140.7328	0.4520484	0.44468637	5.362	turquoise	868.271	852.694	0.9554707	0.0112042	3.338	99
AK237091.1	RPS5P3	turquoise	1456.7	1455.9828	0.9837851	0.00247255	21.26	turquoise	946.681	870.733	0.9632368	0.0084148	23.008	--
AK233581.1	SERPING1	turquoise	1420.4	1419.9813	0.9734037	0.00518592	17.066	turquoise	872.297	813.651	0.9513554	0.0127847	13.222	--
AK233667.1	HS2ST1	turquoise	1104.2	1103.3298	0.9221114	0.02578722	22.766	turquoise	864.946	831.933	0.951076	0.0128945	18.58	--
AK233731.1	DPYSL3	turquoise	1120.4	1118.5308	0.9231645	0.02527011	94.22	turquoise	1045.03	1001.31	0.9969653	0.0002006	96.498	--
AK240176.1	APCDD1	turquoise	1133.6	1132.64	0.9167803	0.02845617	14.732	turquoise	660.256	630.376	0.8826221	0.0474149	13.942	--
AK240211.1	TGFBI	turquoise	1012.6	1010.4156	0.9028784	0.03579953	208.94	turquoise	284.318	223.2	0.6619745	0.2235674	186.958	0.036
AK240222.1	PTK2	turquoise	1221	1219.7011	0.9371363	0.01874112	55.294	turquoise	672.071	555.408	0.8606504	0.0611223	65.86	--
AK240296.1	CCT7	turquoise	235	209.77691	0.5112089	0.37868933	64.742	turquoise	857.821	822.384	0.9501943	0.0132428	79.196	0
AK240334.1	MPZL2	turquoise	1553.3	1552.9008	0.9966454	0.00023312	8.87	turquoise	1051.87	1012.67	0.9997064	6.04E-06	7.064	--
AK233921.1	PACSIN2	turquoise	457.92	455.05054	0.7229528	0.16758596	24.126	turquoise	972.989	929.042	0.9794995	0.0035127	29.856	0
AK233937.1	VKORC1	turquoise	440.42	432.17219	0.7072772	0.1815367	24.036	turquoise	202.864	152.905	0.5624395	0.323668	28.564	--
AK234108.1	PFKL	turquoise	1393.1	1392.4345	0.9703834	0.00609114	38.71	turquoise	754.109	694.983	0.9090018	0.0324986	43.138	0

AK237173.1	PSMA5	turquoise	133.77	115.38878	0.4236389	0.47720525	51.576	turquoise	1025.59	977.924	0.9923291	0.0008056	56.726	--
AK237286.1	RALB	turquoise	99.539	34.729103	0.1016	0.8708618	83.954	turquoise	145.815	120.393	0.5179051	0.3713787	85.946	0
AK237308.1	ATP6AP1	turquoise	858.49	854.21702	0.8573865	0.06324978	17.792	turquoise	304.81	259.142	0.6765508	0.2097779	28.796	--
AK237326.1	THOC4	turquoise	1355.5	1354.4502	0.9680132	0.00683431	57.284	turquoise	680.621	569.393	0.8606776	0.0611047	53.188	--
NM_214281.2	CFH	turquoise	1484.7	1484.2983	0.984849	0.0022336	6.718	turquoise	622.584	578.125	0.8758545	0.0515197	19.628	0.242
NM_001097419.1	PPPDE1	turquoise	1436	1435.2819	0.9804872	0.00326242	46.312	turquoise	288.048	243.485	0.6702494	0.2157093	51.11	0
NM_001097439.1	CPE	turquoise	1133.9	1131.8379	0.9245023	0.02461808	105.792	turquoise	962.086	901.172	0.9711807	0.0058475	124.864	0
NM_001097447.1	UIMC1	turquoise	1546.7	1546.2038	0.9970066	0.00019651	22.466	turquoise	750.961	671.777	0.9027492	0.0358703	21.956	99
NM_001099925.1	CARTPT	turquoise	1553.6	1553.1829	0.9965278	0.00024548	0.378	turquoise	1015.27	988.541	0.9929221	0.0007141	1.828	0
NM_001101026.1	SQLE	turquoise	1174	1172.1231	0.9328377	0.02068227	17.82	turquoise	1015.27	988.541	0.9929221	0.0007141	1.15	0.061
NM_001101031.1	BMP4	turquoise	1458.1	1457.5327	0.9844175	0.00232954	13.62	turquoise	985.935	954.122	0.9861006	0.001963	17.662	0
NM_001101827.1	MKI67IP	turquoise	224.13	184.18101	0.4586609	0.43719064	49.846	turquoise	961.876	885.471	0.9675485	0.0069833	55.384	0
NM_001105292.1	TBK1	turquoise	1472.8	1472.1793	0.9835534	0.00252565	9.4	turquoise	172.422	96.0425	0.4666074	0.4282207	23.182	--
NM_001105302.1	ACLY	turquoise	1477.2	1476.6443	0.9868177	0.00181327	24.644	turquoise	993.612	926.338	0.9786686	0.0037279	30.746	0
NM_001099936.1	BTG1	turquoise	165.81	151.63093	0.4986681	0.39247151	83.53	turquoise	311.374	210.147	0.621995	0.2625885	102.806	99
L10363.1	IBSP	turquoise	1553.6	1553.1829	0.9965278	0.00024548	8.314	turquoise	1015.27	988.541	0.9929221	0.0007141	1.082	--
NM_001113701.1	C20orf108	turquoise	363.68	352.65358	0.6643814	0.22127376	8.304	turquoise	1023.16	993.479	0.9945116	0.0004877	9.342	99
NM_001114275.1	NCOA2	turquoise	1514.3	1513.7822	0.9925616	0.00076926	18.392	turquoise	549.083	502.386	0.8424146	0.0732936	33.164	0.097
NM_001114670.1	TYK2	turquoise	1153.7	1152.4189	0.9216282	0.0260256	78.072	turquoise	591.306	544.037	0.8609427	0.0609329	74.032	0
NM_001114672.1	ATP9A	turquoise	376.91	371.34197	0.665921	0.21981004	34.362	turquoise	749.882	730.354	0.9177846	0.0279469	48.93	0.017
NM_001122990.1	NNAT	turquoise	251.45	229.48779	0.5369706	0.35075554	36.442	turquoise	1032.25	998.381	0.9957373	0.0003339	38.594	--
NM_001123131.1	ITM2C	turquoise	1473.2	1472.6358	0.9851082	0.00217663	10.508	turquoise	617.534	464.867	0.8162835	0.0918778	14.126	0
NM_001123111.1	EGFL8	turquoise	1024.2	1022.3959	0.8997149	0.03754441	3.882	turquoise	1013.61	981.558	0.9921251	0.0008379	6.146	--
NM_001123177.1	RAB33A	turquoise	1507.5	1507.0298	0.9896981	0.00125324	1.04	turquoise	1015.27	988.541	0.9929221	0.0007141	2.03	--
NM_001123160.1	PFDN4	turquoise	171.26	151.64247	0.4598123	0.43588828	53.418	turquoise	1047.87	998.044	0.9966684	0.0002307	45.34	0
NM_001123157.1	PBX2	turquoise	1237.2	1236.105	0.9474693	0.01433843	82.412	turquoise	789.103	775.174	0.9328693	0.0206677	79.262	99
NM_001123200.1	TES	turquoise	1498.4	1497.8077	0.9891921	0.00134661	17.032	turquoise	785.734	717.867	0.9156895	0.0290126	27.538	0
NM_001123096.1	CDC37	turquoise	1298.1	1297.2717	0.9576152	0.01040802	33.866	turquoise	433.836	390.133	0.76833	0.1291038	35.644	99
NM_001123090.1	C6orf153	turquoise	1486.3	1485.7934	0.9868531	0.00180597	49.468	turquoise	665.774	583.843	0.8744999	0.0523541	47.4	--
EU780792.1	CHD3	turquoise	1553.7	1553.3073	0.9967278	0.00022458	8.348	turquoise	889.834	870.37	0.9610214	0.0091837	11.232	--
NM_001128462.1	RAI14	turquoise	1513.6	1513.202	0.9907331	0.00106938	16.89	turquoise	969.623	922.658	0.9777094	0.0039816	21.844	0.614
NM_001129967.1	LEF1	turquoise	1056.1	1054.7551	0.9058205	0.03420077	2.908	turquoise	1019.79	984.416	0.9927553	0.0007394	3.504	0
XM_001927265.1	AKAP12	turquoise	1537.4	1536.983	0.9957973	0.00032685	66.866	turquoise	882.447	800.87	0.9433006	0.0160684	130.97	0.418
XM_001924731.1	MTHFD1L	turquoise	1522.3	1521.8852	0.9928212	0.00072937	5.918	turquoise	581.868	421.988	0.7921776	0.1101154	10.83	0.214
XM_001927696.1	PDCL	turquoise	1483.4	1482.7774	0.9885469	0.00146883	26.01	turquoise	1014.14	949.788	0.9851482	0.0021679	31.658	0
XM_001927813.1	PBX3	turquoise	1518.2	1517.804	0.9923476	0.00080266	7.738	turquoise	979.703	943.89	0.9820609	0.0028765	19.946	0

XM_001925557.1	GARNL3	turquoise	61.987	45.670377	0.2741926	0.6553123	3.528	turquoise	991.732	967.981	0.9874961	0.0016753	4.882	--
XM_001924714.1	FAM129B	turquoise	1514.6	1514.1128	0.9919819	0.00086082	26.79	turquoise	816.591	783.67	0.9364892	0.0190294	19.612	0
XM_001924823.1	STXBP1	turquoise	1000.3	998.23612	0.8913331	0.04229324	5.836	turquoise	931.335	900.002	0.971405	0.0057796	8.554	0.047
XM_001924991.1	NIPSNAP3	turquoise	1415.3	1414.5618	0.9775108	0.00403481	39.49	turquoise	570.076	423.668	0.7895125	0.1121921	39.032	0.358
XM_001924394.1	IKBKAP	turquoise	1420	1419.4727	0.9775455	0.00402552	4.916	turquoise	237.101	152.595	0.5656496	0.320293	11.928	0.344
XM_001926189.1	ZNF618	turquoise	1426.5	1425.8833	0.9775157	0.0040335	11.414	turquoise	986.648	910.892	0.9747889	0.0047871	17.652	0.085
AK232748	AMBP	turquoise	1553.6	1553.1829	0.9965278	0.00024548	1.558	turquoise	1015.27	988.541	0.9929221	0.0007141	0.406	--
XM_001924352.1	IGFBPL1	turquoise	1516.7	1516.3448	0.9909222	0.00103685	1.288	turquoise	1015.27	988.541	0.9929221	0.0007141	3.654	0.353
XM_001928221.1	RP11-35N(	turquoise	939.66	938.23388	0.889842	0.04315673	1.686	turquoise	1026.36	997.169	0.9953549	0.0003798	2.048	0
XM_001927070.1	SLC1A1	turquoise	844.67	839.69037	0.8510372	0.06745287	2.956	turquoise	1033.34	973.225	0.9905238	0.0011058	4.42	0
XM_001924292.1	KANK1	turquoise	811.09	808.84465	0.8379456	0.07637937	11.722	turquoise	758.314	733.781	0.9252726	0.0242452	9.586	0
XM_001924582.1	ELAVL2	turquoise	1553.6	1553.1829	0.9965278	0.00024548	2.786	turquoise	1015.27	988.541	0.9929221	0.0007141	10.962	0
XM_001925498.1	ATL1	turquoise	1468.4	1467.7122	0.9852906	0.00213681	2.852	turquoise	951.167	926.731	0.9776598	0.0039949	3.85	99
XM_001924194.1	WDHD1	turquoise	1267.9	1266.8757	0.9465366	0.01471992	11.724	turquoise	876.69	784.228	0.939892	0.0175298	14.144	0.957
XM_001925911.1	SCG3	turquoise	1556.4	1556.0725	0.9967712	0.00022013	3.202	turquoise	1022.04	993.969	0.99444	0.0004973	7.748	0.156
XM_001924603.1	DMXL2	turquoise	1443.6	1443.0915	0.979606	0.00348542	2.372	turquoise	1010.59	983.499	0.9917141	0.0009043	6.584	99
XM_001927452.1	SORD	turquoise	451.38	441.17458	0.7032013	0.18521597	7.334	turquoise	1004.76	974.677	0.9896374	0.0012643	10.48	99
XM_001928118.1	MFAP1	turquoise	1508.1	1507.6777	0.9890358	0.00137588	28.392	turquoise	795.45	685.277	0.9081329	0.0329608	35.614	--
XM_001925969.1	MAP1A	turquoise	173.86	154.40787	0.4748795	0.41892832	23.066	turquoise	858.271	841.911	0.952762	0.0122369	19.946	--
XM_001929418.1	FAM82A2	turquoise	1430.5	1429.8191	0.9786211	0.00374037	14.156	turquoise	907.738	824.884	0.950512	0.0131169	13.922	0.421
XM_001927442.1	THBS1	turquoise	1459.3	1458.6259	0.9840231	0.00241841	63.162	turquoise	864.153	814.4	0.9467927	0.0146148	73.574	0
XM_001928355.1	MEX3C	turquoise	1532.4	1531.9482	0.9932049	0.00067171	17.406	turquoise	903.041	866.712	0.9643834	0.0080256	29.998	0
XM_001927251.1	HERC1	turquoise	1450.6	1450.0349	0.9782253	0.00384448	59.61	turquoise	824.774	784.597	0.9372017	0.0187121	57.34	0.03
XM_001924962.1	TUBE1	turquoise	1410.1	1409.236	0.9767773	0.00423335	25.788	turquoise	651.773	524.753	0.8455633	0.0711433	25.03	99
XM_001925318.1	HDAC2	turquoise	1191.9	1190.217	0.9363808	0.01907778	112.7	turquoise	719.833	630.835	0.8850263	0.0459829	146.236	99
XM_001927441.1	TPBG	turquoise	1555.1	1554.6487	0.9977398	0.00012895	48.55	turquoise	909.514	841.969	0.9547948	0.0114591	55.388	0
XM_001925769.1	ELOVL4	turquoise	1297.9	1297.1925	0.949852	0.0133789	1.712	turquoise	992.811	968.208	0.9877362	0.0016273	3.296	0
XM_001926931.1	MOXD1	turquoise	1516.8	1516.2506	0.9919266	0.00086975	4.766	turquoise	985.985	951.766	0.9854927	0.002093	7.038	0.132
XM_001925476.1	ENPP1	turquoise	1534.1	1533.7041	0.9932252	0.00066872	22.126	turquoise	459.193	423.661	0.7899719	0.1118334	17.888	0.783
XM_001926912.1	HEY2	turquoise	1282.3	1281.4853	0.9470005	0.01452976	6.586	turquoise	815.858	801.339	0.941385	0.0168846	9.708	99
XM_001924744.1	ZNF34	turquoise	1484.9	1484.4522	0.9837944	0.00247044	6.326	turquoise	739.507	672.643	0.905031	0.0346275	10.964	99
XM_001924535.1	ASAP1	turquoise	1355.2	1354.1407	0.966234	0.00741039	32.684	turquoise	871.839	801.557	0.9437213	0.0158909	27.314	0
XM_001928185.1	FAM84B	turquoise	123.35	93.895763	0.3501165	0.56350051	4.064	turquoise	942.063	921.54	0.9751658	0.0046804	6.774	0
XM_001924955.1	KIAA0196	turquoise	1075.2	1073.0725	0.9144404	0.02965401	35.426	turquoise	867.875	810.627	0.9465137	0.0147293	40.308	0
XM_001928505.1	WDYHV1	turquoise	1476.3	1475.7085	0.9875457	0.00166532	78.814	turquoise	311.076	209.011	0.6246541	0.259941	86.834	0.077
XM_001927135.1	ZNF644	turquoise	1567.1	1566.6938	0.9994958	1.36E-05	53.61	turquoise	1035.58	997.515	0.9957194	0.000336	68.604	--

XM_001928869.1	PEX19	turquoise	1343.5	1342.8238	0.9600765	0.00951828	55.688	turquoise	233.127	153.711	0.5627361	0.3233558	83.506	0.177
XM_001929031.1	ATP1A2	turquoise	403	395.54918	0.6712493	0.21476503	3.046	turquoise	538.532	508.192	0.8310547	0.0812137	11.414	0
XM_001929177.1	CADM3	turquoise	1490.6	1490.2361	0.9864308	0.00189357	1.636	turquoise	1020.35	992.764	0.9940676	0.000548	7.132	0
XM_001925466.1		turquoise	298.34	277.52662	0.5787748	0.30658801	105.11	turquoise	752.459	674.748	0.9020129	0.0362743	60.794	99
XM_001926377.1	CCT3	turquoise	208.57	150.90591	0.3882789	0.51834711	133.048	turquoise	741.886	635.235	0.8906883	0.042666	103.6	0.134
XM_001929400.1	SHC1	turquoise	1356.3	1355.7663	0.9657168	0.00758072	21.056	turquoise	935.281	877.784	0.9662659	0.0073999	16.186	0
XM_001929662.1	MLLT11	turquoise	1258.5	1257.1638	0.949181	0.01364691	3.964	turquoise	1050.2	1010.34	0.9990896	3.30E-05	10.146	--
XM_001928686.1	SEC22B	turquoise	1428.4	1427.8484	0.9793587	0.00354887	28.404	turquoise	868.796	753.885	0.9285032	0.0227013	44.686	0
XM_001925127.1	MAGI3	turquoise	1560.4	1560.0183	0.9981706	9.39E-05	41.262	turquoise	880.893	791.781	0.9408456	0.0171168	64.414	0.086
XM_001928463.1	TOX	turquoise	420.08	411.34812	0.6961579	0.19162312	13.646	turquoise	951.907	908.575	0.9727738	0.0053707	11.616	99
XM_001927760.1	KIFAP3	turquoise	1112	1110.3469	0.9192136	0.02722747	21.606	turquoise	1001.13	970.245	0.9889845	0.0013855	36.332	0
XM_001927877.1	KLHDC9	turquoise	333.74	329.42253	0.6743873	0.21180921	2.126	turquoise	1048.57	1010.85	0.9991494	2.98E-05	3.626	99
XM_001927747.1	PABPC1	turquoise	901.24	897.09321	0.8678202	0.05652983	438.702	turquoise	883.251	848.074	0.9577804	0.0103475	562.674	99
XM_001928953.1	HRSP12	turquoise	1553.3	1552.857	0.9970309	0.00019413	15.404	turquoise	867.544	807.55	0.9440419	0.0157561	24.838	--
XM_001928669.1	3-Sep	turquoise	872.46	867.67893	0.8587147	0.06238132	1.148	turquoise	1038.17	1006.29	0.9976827	0.0001339	9.404	0
XM_001925128.1	HOXC4	turquoise	1374.1	1373.216	0.9704905	0.00605821	28.926	turquoise	1018.45	975.912	0.9907682	0.0010633	25.518	--
XM_001927351.1	EPYC	turquoise	1552.3	1551.917	0.9963903	0.0002602	20.116	turquoise	1015.27	988.541	0.9929221	0.0007141	2.64	0.144
XM_001927280.1	CSRP2	turquoise	320.4	291.5672	0.5789769	0.30637817	34.562	turquoise	1051.18	1006.77	0.9983712	7.89E-05	40.156	0
XM_001926886.1	PTPRR	turquoise	1542.6	1542.1788	0.994826	0.00044642	2.518	turquoise	957.389	931.719	0.979491	0.0035149	4.076	0
XM_001927835.1	RIMKLB	turquoise	1560.7	1560.2316	0.998822	4.85E-05	13.228	turquoise	921.299	824.6	0.9502334	0.0132273	18.744	99
XM_001928013.1	MLF2	turquoise	1442.9	1442.1254	0.9822273	0.00283663	88.838	turquoise	742.305	598.323	0.8727932	0.0534114	104.92	0
XM_001924188.1	CNTN1	turquoise	1447.1	1446.4002	0.9809466	0.0031481	3.072	turquoise	1030.72	1000.11	0.9960333	0.0002997	11.378	0
XM_001927045.1	FOXF2	turquoise	1553.6	1553.1829	0.9965278	0.00024548	0.756	turquoise	1015.27	988.541	0.9929221	0.0007141	4.33	--
XM_001928655.1	CD83	turquoise	1482.6	1482.0975	0.9876326	0.00164795	8.358	turquoise	804.067	746.397	0.92642	0.0236931	14.36	0
XM_001927909.1	DEK	turquoise	1354.8	1353.9804	0.966406	0.00735403	266.846	turquoise	473.505	385.056	0.7644632	0.1322664	285.724	0
XM_001927641.1	FLRT2	turquoise	1481	1480.6285	0.9835265	0.00253185	11.734	turquoise	126.84	78.6152	0.4089367	0.4942253	14.182	0
XM_001928187.1	CALM1	turquoise	1517.2	1516.8242	0.9907424	0.00106777	90.006	turquoise	999.604	934.581	0.9805566	0.0032451	125.396	99
XM_001926890.1	CPSF2	turquoise	1390.1	1389.1959	0.9721007	0.00557054	40.91	turquoise	699.461	624.13	0.889166	0.04355	51.154	0
XM_001926793.1	PLEKHG3	turquoise	465.88	457.53178	0.7211427	0.16918048	46.082	turquoise	322.325	282.047	0.6914668	0.1959246	28.57	0.042
XM_001928679.1	ARG2	turquoise	1413.7	1412.9821	0.9769889	0.00417575	7.408	turquoise	896.7	874.608	0.9624634	0.0086807	11.58	0
XM_001928767.1	RDH11	turquoise	672.54	666.13606	0.79893	0.10490641	26.262	turquoise	1029.27	968.819	0.9893759	0.0013124	45.028	0.154
XM_001928961.1	PNMA1	turquoise	1069.9	1068.3022	0.9164634	0.02861747	8.702	turquoise	1050.66	1011.6	0.9993118	2.17E-05	14.154	99
XM_001928512.1	LOXL1	turquoise	1452.9	1452.3139	0.9831221	0.00262548	27.482	turquoise	747.525	622.769	0.8828523	0.0472772	24.29	99
XM_001926092.1	NEO1	turquoise	1376.3	1375.7236	0.9657354	0.00757456	42.136	turquoise	580.44	558.909	0.8551065	0.0647494	47.672	99
XM_001924413.1	NOVA1	turquoise	142.42	120.16785	0.407708	0.49565345	11.748	turquoise	1028.54	998.245	0.9955336	0.0003581	27.472	--
XM_001927354.1	NFATC4	turquoise	1483.3	1482.6693	0.9881495	0.00154585	22.394	turquoise	969.623	920.841	0.9775283	0.0040301	20.368	99

XM_001928918.1	SLC22A17	turquoise	1461.6	1460.8548	0.9841001	0.00240097	18.144	turquoise	1017.38	977.04	0.9911576	0.0009968	19.628	0
XM_001925226.1	ADAMTS7	turquoise	1156.5	1154.7303	0.9307174	0.02166229	22.902	turquoise	844.792	817.8	0.9516137	0.0126835	16.532	0.105
XM_001929079.1	SH3GL3	turquoise	692.7	684.85372	0.8001877	0.10394468	12.566	turquoise	715.949	584.965	0.8676011	0.0566685	13.998	0
XM_001929124.1	HDGFRP3	turquoise	1263.5	1262.3235	0.9492375	0.01362426	32.994	turquoise	963.862	931.58	0.9797669	0.0034443	29.388	99
XM_001925448.1	SOX4	turquoise	1249.2	1248.657	0.9497745	0.01340975	54.768	turquoise	1031.91	997.356	0.9958074	0.0003257	71.254	0
XM_001927905.1	NRSN1	turquoise	1513.8	1513.2691	0.9894726	0.00129457	1.086	turquoise	793.225	769.814	0.9310866	0.0214906	5.314	0.395
XM_001929121.1		turquoise	1553.6	1553.1829	0.9965278	0.00024548	0.566	turquoise	1037.88	1002.84	0.9972042	0.0001774	3.008	99
XM_001929612.1	RING1	turquoise	248.85	230.73698	0.5514799	0.33525736	20.88	turquoise	684.363	648.062	0.8900371	0.0430434	14.616	0
XM_001927693.1	RGL2	turquoise	195.33	185.58593	0.5542764	0.33229044	34.68	turquoise	783.276	768.44	0.9309113	0.0215721	38.11	0.617
XM_001928400.1	HMGA1	turquoise	501.15	494.90917	0.7432633	0.14999956	39.014	turquoise	1020.72	960.257	0.9873424	0.0017062	32.332	99
XM_001926869.1	SCUBE3	turquoise	1553.6	1553.1829	0.9965278	0.00024548	2.362	turquoise	1015.27	988.541	0.9929221	0.0007141	0.204	--
XM_001929580.1	MTCH1	turquoise	1276.3	1274.9854	0.9517924	0.01261368	168.67	turquoise	1046.89	1001.61	0.9972324	0.0001747	198.746	99
XM_001927223.1	PAN3	turquoise	124.83	99.019573	0.3730146	0.53631897	62.712	turquoise	448.285	415.096	0.7830076	0.1173097	61.818	0
XM_001928150.1	TRIM13	turquoise	180.6	156.44046	0.4551623	0.44115299	29.566	turquoise	1006.57	951.219	0.9851982	0.002157	27.632	--
XM_001927466.1	DZIP1	turquoise	1546	1545.5319	0.9962813	0.00027207	68.772	turquoise	721.876	574.127	0.8627104	0.0597914	92.484	0.178
XM_001926132.1	CLN5	turquoise	1293.3	1292.0313	0.9560972	0.01096962	22.546	turquoise	844.201	803.419	0.9489867	0.0137249	23.604	99
XM_001926443.1	COL8A1	turquoise	1552.9	1552.5135	0.9964263	0.00025631	20.412	turquoise	977.97	899.494	0.9714472	0.0057668	15.964	0
XM_001924920.1	HSPA13	turquoise	1244.4	1243.0768	0.9485344	0.01390683	26.612	turquoise	996.764	937.627	0.9810016	0.0031345	50.476	0
XM_001925741.1	TMEM44	turquoise	364.4	347.43721	0.643294	0.24158777	59.64	turquoise	977.869	928.521	0.9783845	0.0038025	42.558	0
XM_001928236.1	LSG1	turquoise	1210.9	1210.1875	0.9389474	0.01794208	9.588	turquoise	501.871	440.885	0.7991823	0.1047132	29.39	0.727
XM_001925819.1	TMEM44	turquoise	1262.8	1262.2244	0.9455358	0.01513285	59.44	turquoise	483.372	405.99	0.7910627	0.1109827	58.11	--
XM_001928989.1	ITGB5	turquoise	1384.3	1383.4047	0.9713739	0.00578899	45.538	turquoise	989.133	918.603	0.9771823	0.0041233	40.422	--
XM_001925354.1	NISCH	turquoise	1311.8	1310.6542	0.9600305	0.00953467	54.018	turquoise	544.276	516.028	0.84895	0.0688528	41.228	0.161
XM_001927800.1	SETD5	turquoise	449.51	433.08118	0.683567	0.20322844	39.422	turquoise	831.396	814.565	0.944451	0.0155846	45.904	0
XM_001924481.1	SEC61A1	turquoise	1481.5	1480.8726	0.9873937	0.00169587	40.798	turquoise	396.894	311.269	0.7162287	0.1735309	54.382	0
XM_001929007.1	CDC42BPA	turquoise	1530.6	1530.0858	0.9947742	0.00045314	5.338	turquoise	1019.73	974.881	0.9909338	0.0010349	1.626	1.074
XM_001926755.1	XPO7	turquoise	1268	1266.7894	0.9477989	0.0142044	48.486	turquoise	248.832	192.443	0.6076033	0.2770401	52.394	0
XM_001924700.1	NEFL	turquoise	1560.1	1559.7568	0.9979207	0.00011378	20.418	turquoise	1034.89	1003.72	0.9969838	0.0001988	61.802	0
XM_001927592.1	BNIP3L	turquoise	1542	1541.5253	0.9964172	0.00025729	84.21	turquoise	853.085	735.999	0.9224282	0.0256313	114.108	99
XM_001926955.1	CALY	turquoise	1553.6	1553.1829	0.9965278	0.00024548	0.52	turquoise	1015.27	988.541	0.9929221	0.0007141	3.79	99
XM_001927529.1	ALDH18A	turquoise	1366.7	1365.9279	0.9688559	0.00656687	65.642	turquoise	285.231	237.148	0.6729051	0.213204	56.294	0
XM_001928803.1	EXOSC1	turquoise	1333.8	1333.215	0.9610534	0.00917244	21.236	turquoise	189.487	154.468	0.5961956	0.288639	27.29	0
XM_001926725.1	DPCD	turquoise	114.17	78.112205	0.2938854	0.63127183	10.51	turquoise	986.023	954.022	0.9851124	0.0021757	9.634	99
XM_001925953.1	PSD	turquoise	1559.3	1558.9086	0.9973212	0.00016636	2.112	turquoise	1031.2	1001.24	0.9963105	0.0002689	9.45	99
XM_001928812.1	CUEDC2	turquoise	912.01	909.03611	0.873349	0.05306639	53.46	turquoise	785.32	769.389	0.9305466	0.0217419	61.484	99
XM_001929320.1	INA	turquoise	1553.6	1553.1829	0.9965278	0.00024548	3.496	turquoise	1015.27	988.541	0.9929221	0.0007141	10.826	0

XM_001925277.1	MXI1	turquoise	907.95	906.23577	0.8667231	0.05722527	20.594	turquoise	346.516	325.221	0.7311085	0.1604562	26.854	99
XM_001927456.1	INPP5F	turquoise	1028.8	1026.8823	0.9020298	0.03626498	42.648	turquoise	1014.27	980.587	0.9918761	0.0008779	57.218	0.073
XM_001928522.1	CCAR1	turquoise	1402.8	1402.0092	0.9741438	0.00497151	65.996	turquoise	953.461	912.298	0.974454	0.0048825	90.556	99
XM_001927977.1	ATAD1	turquoise	95.118	52.83315	0.2169339	0.72597314	34.07	turquoise	108.973	80.5494	0.449232	0.4478876	42.646	0
XM_001928136.1		turquoise	1435.3	1434.6287	0.9797521	0.00344811	49.604	turquoise	910.266	859.522	0.9600797	0.0095171	64.116	99
XM_001928949.1	MYST4	turquoise	1567	1566.5606	0.9995328	1.21E-05	24.544	turquoise	978.387	915.419	0.9767841	0.0042315	28.548	0.256
XM_001929420.1	ARPC3	turquoise	401.12	392.54673	0.6944421	0.1931932	39.98	turquoise	1019.58	973.74	0.9906638	0.0010814	36.58	0
XM_001925272.1	GCN1L1	turquoise	1391.6	1390.8045	0.9745649	0.00485087	25.352	turquoise	469.952	431.647	0.8073727	0.0985023	27.33	0
XM_001928487.1	COX6A1P	turquoise	1446.6	1445.8813	0.9807368	0.00320014	97.438	turquoise	573.219	452.303	0.8042953	0.1008224	133.566	--
XM_001925356.1	RNF10	turquoise	1377.3	1376.7874	0.9710655	0.00588253	35.736	turquoise	953.142	886.491	0.9679782	0.0068455	46.92	0.43
XM_001926685.1	SART3	turquoise	1488	1487.4281	0.9874766	0.0016792	57.768	turquoise	472.296	325.822	0.727219	0.1638453	53.926	1.025
XM_001928908.1	NIPSNAP1	turquoise	877.25	876.09032	0.8629985	0.05960592	16.408	turquoise	980.961	955.022	0.9844188	0.0023293	16.446	--
XM_001926534.1	PIK3IP1	turquoise	204.79	191.74949	0.516353	0.37307018	3.398	turquoise	588.182	563.077	0.854599	0.0650847	6.948	99
XM_001928076.1	YWHAH	turquoise	830.24	827.54639	0.8501091	0.06807426	101.718	turquoise	979.538	941.314	0.9812613	0.0030706	127.152	0
XM_001927481.1	HIRA	turquoise	1505.1	1504.5613	0.989318	0.00132317	19.184	turquoise	916.735	830.756	0.9536804	0.0118834	19.768	0.351
XM_001926661.1	FAM101A	turquoise	1548.6	1548.1891	0.996179	0.00028337	22.294	turquoise	1032.44	978.616	0.9916015	0.0009228	17.014	0
XM_001928673.1	IDH1	turquoise	1541.1	1540.607	0.9964437	0.00025445	5.398	turquoise	843.502	744.361	0.9255926	0.0240908	11.984	--
XM_001928206.1	STK16	turquoise	1428.1	1427.4178	0.9792571	0.00357504	40.074	turquoise	711.073	591.983	0.8708875	0.0545999	40.812	0
XM_001928489.1	RESP18	turquoise	1553.6	1553.1829	0.9965278	0.00024548	0.236	turquoise	1015.27	988.541	0.9929221	0.0007141	2.91	0
XM_001925017.1	HOXD3	turquoise	749.53	745.68321	0.8266648	0.08434067	24.918	turquoise	405.966	390.418	0.7890772	0.1125325	20.802	0
XM_001927705.1	PRKRA	turquoise	185.2	118.72512	0.317258	0.6029371	24.388	turquoise	975.366	914.282	0.9750075	0.0047251	28.438	99
XM_001925245.1	FAM149A	turquoise	132.66	103.80267	0.3624367	0.54884342	15.082	turquoise	999.004	963.533	0.9877518	0.0016242	13.08	1.043
XM_001924403.1	MTMR7	turquoise	1530.3	1529.8112	0.9949078	0.00043587	2.32	turquoise	1018.87	990.529	0.9935485	0.0006214	4.262	0.118
XM_001924460.1	CNOT7	turquoise	1172.6	1171.0282	0.9307183	0.02166187	74.538	turquoise	517.989	470.929	0.8327745	0.0799986	76.66	0
XM_001925000.1	ISM1	turquoise	1537.8	1537.429	0.9932448	0.00066582	3.968	turquoise	981.059	946.016	0.9828658	0.0026854	4.336	0.068
XM_001929295.1	GGT7	turquoise	409.55	392.2548	0.6666461	0.21912157	5.54	turquoise	1052.59	1006.11	0.9984421	7.38E-05	6.63	0.164
XM_001925248.1	SAMHD1	turquoise	1548.3	1547.9255	0.9959623	0.0003078	21.818	turquoise	960.042	907.495	0.9737118	0.0050963	26.896	0
XM_001928614.1	SRC	turquoise	1528.9	1528.4623	0.9925541	0.00077042	16.428	turquoise	898.533	821.499	0.9495532	0.013498	23.13	0.119
XM_001928737.1	VSTM2L	turquoise	1553.6	1553.1829	0.9965278	0.00024548	2.598	turquoise	1015.27	988.541	0.9929221	0.0007141	5.482	99
XM_001927204.1	SULF2	turquoise	1434.9	1434.3825	0.9767059	0.00425283	38.918	turquoise	844.632	823.53	0.9474238	0.014357	27.36	0.216
XM_001924191.1	SYAP1	turquoise	64.889	51.523728	0.2782498	0.65034751	40.11	turquoise	1021.75	971.642	0.9900222	0.0011946	33.86	0
XM_001927440.1	SLC25A5	turquoise	1566.3	1565.9146	0.9992651	2.39E-05	24.44	turquoise	1023	959.837	0.9874482	0.0016849	32.732	0
XM_001927589.1	SLC25A43	turquoise	1511.3	1510.7872	0.9916915	0.00090797	11.198	turquoise	723.304	669.468	0.9067721	0.0336887	16.482	99
XM_001927811.1	NLGN3	turquoise	1467.2	1466.7177	0.9834781	0.00254298	5.184	turquoise	921.655	856.439	0.9606341	0.0093204	3.766	0
XM_001924869.1	TCEAL2	turquoise	1553.6	1553.1829	0.9965278	0.00024548	1.748	turquoise	1002.13	976.781	0.9898318	0.001229	6.338	--
XM_001925649.1	BEX5	turquoise	1553.6	1553.1829	0.9965278	0.00024548	1.04	turquoise	1015.27	988.541	0.9929221	0.0007141	3.924	0.168

NM_001130535.1	6-Mar	turquoise	315.19	305.19525	0.6377267	0.24703129	68.814	turquoise	234.623	211.3	0.6305118	0.2541344	107.038	0
X17058.1	SLC2A1	turquoise	1371.4	1370.5037	0.9695522	0.00634854	11.838	turquoise	819.002	699.089	0.9099229	0.032011	13.55	99
NM_001131045.1	SLC39A7	turquoise	1381.2	1380.3707	0.9722391	0.00552926	87.648	turquoise	661.25	595.637	0.8744664	0.0523748	87.352	0.334
AF490841.1	GLS	turquoise	139.83	107.0466	0.3960385	0.50925882	128.1	turquoise	889.917	816.979	0.9481868	0.0140472	124.256	99
NM_001134967.1	EPHA4	turquoise	1558.9	1558.4828	0.9975717	0.0001436	3.982	turquoise	951.771	912.093	0.9743203	0.0049208	4.648	0
X16638.1	PGD	turquoise	1468	1467.2829	0.9858668	0.00201267	35.81	turquoise	769.673	629.218	0.8844593	0.0463194	33.326	0
NM_001135680.1	USP7	turquoise	1435.4	1434.6101	0.979507	0.00351078	30.11	turquoise	339.52	322.751	0.7493724	0.1448224	51.706	0
NM_001135966.1	SLC26A11	turquoise	634.66	625.15082	0.7768822	0.12219068	7.194	turquoise	1030.47	995.738	0.9953811	0.0003766	9.786	--
NM_213921.1	HEXB	turquoise	1428	1427.4119	0.9804825	0.00326358	16.674	turquoise	987.145	939.23	0.9823131	0.0028162	18.928	0
NM_213911.1	PGRMC1	turquoise	1566.9	1566.5565	0.9988895	4.44E-05	275.984	turquoise	1042.35	991.125	0.9950022	0.0004238	308.788	0
NM_213971.1	CLU	turquoise	1057	1055.7414	0.9012462	0.03669648	11.086	turquoise	985.965	961.215	0.9860428	0.0019752	44.516	0.031
NM_213977.1	SEPW1	turquoise	1563.8	1563.3529	0.9988861	4.46E-05	31.994	turquoise	594.367	516.229	0.8358008	0.0778742	49.154	99
NM_214003.1	IGFBP2	turquoise	591.58	581.41508	0.762656	0.13375228	142.956	turquoise	575.919	493.15	0.8255057	0.0851724	117.826	--
NM_214024.1	PPP2R1A	turquoise	1163.9	1162.0729	0.9331799	0.02052546	78.79	turquoise	834.926	791.076	0.939511	0.0176957	100.862	0
NM_214025.1	PPP2R2B	turquoise	1175.8	1174.894	0.9356836	0.01939016	1.788	turquoise	945.41	920.382	0.9750406	0.0047158	5.054	0
NM_214054.1	PLAT	turquoise	1564.2	1563.756	0.9986215	6.14E-05	11.564	turquoise	1008.53	981.535	0.9913832	0.0009589	20.262	0.695
NM_214064.1	FMO1	turquoise	1039.9	1038.5749	0.8982559	0.03835804	2.84	turquoise	712.325	689.89	0.9054402	0.0344061	6.09	0.208
NM_213866.1	CEND1	turquoise	1553.6	1553.1829	0.9965278	0.00024548	0.33	turquoise	1031.01	1001.09	0.9962725	0.000273	2.378	--
NM_213815.1	CD55	turquoise	1549.6	1549.2232	0.9959694	0.000307	17.088	turquoise	933.924	891.572	0.9702777	0.0061237	2.884	0.632
NM_213781.1	SCD	turquoise	694.28	689.17878	0.8109045	0.09585983	2.17	turquoise	999.968	974.983	0.9894327	0.0013019	4.018	0
AY459532	UCHL1	turquoise	1555.4	1555.0523	0.9971385	0.00018367	68.082	turquoise	1030.26	1000.57	0.9961237	0.0002895	152.592	--
NM_213746.1	CALCB	turquoise	1532	1531.5603	0.9930566	0.0006938	3.89	turquoise	945.322	915.701	0.9740501	0.0049985	3.53	0
NM_213741.1	TRO	turquoise	1014.8	1011.6326	0.8962794	0.03946904	6.238	turquoise	1039.25	994.233	0.99549	0.0003633	8.126	--
NM_214230.1	TNC	turquoise	1489.5	1488.8975	0.9882137	0.00153331	92.214	turquoise	897.618	804.045	0.9440691	0.0157447	47.94	0.155
NM_214240.1	ATP6V1H	turquoise	1363.7	1362.7978	0.965151	0.00776847	37.614	turquoise	1013.41	970.316	0.9894723	0.0012946	42.236	0.297
NM_214267.1	CYBA	turquoise	1521.6	1521.0727	0.9930794	0.0006904	19.348	turquoise	557.159	459.453	0.8098774	0.0966261	19.358	--
NM_214306.1	HSD17B4	turquoise	1053.6	1052.2866	0.9124862	0.03066649	41.044	turquoise	865.929	832.124	0.9512331	0.0128327	49.454	0
NM_214317.1	AFP	turquoise	1553.6	1553.1829	0.9965278	0.00024548	7.086	turquoise	1015.27	988.541	0.9929221	0.0007141	0.474	99
NM_214328.1	GATA6	turquoise	158.56	136.75309	0.4345609	0.46464431	2.266	turquoise	917.231	899.209	0.9687918	0.0065871	8.388	0
NM_214333.1	RPN1	turquoise	321.9	316.9354	0.6570447	0.2282857	84.526	turquoise	1000.7	961.796	0.9873294	0.0017088	84.924	0.115
NM_214337.1	PPP1R14A	turquoise	1092.7	1091.4766	0.9151378	0.02929535	3.96	turquoise	985.338	960.161	0.9857277	0.0020424	6.382	--
NM_214366.1	PPP2CA	turquoise	426.71	408.33566	0.67008	0.21586935	98.04	turquoise	296.959	261.311	0.6879421	0.1991741	118.878	99
NM_214372.1	APP	turquoise	1549	1548.6535	0.9954519	0.00036795	241.998	turquoise	1009.14	978.306	0.9907429	0.0010677	427.722	0
NM_214382.1	PTH1R	turquoise	527.55	523.76589	0.7423418	0.1507851	61.498	turquoise	1044.73	992.96	0.9952497	0.0003927	38.16	0.14
NM_214408.1	PRDX6	turquoise	278.76	257.54061	0.5727676	0.3128415	81.484	turquoise	985.977	944.527	0.9823023	0.0028187	55.982	0
NM_214432.1	CYP51A1	turquoise	1326.7	1325.5651	0.9622763	0.00874546	22.266	turquoise	767.366	722.36	0.9180111	0.0278325	35.1	0

NM_214440.1	HYAL2	turquoise	1486.5	1485.9519	0.9871454	0.00174616	10.862	turquoise	941.436	920.28	0.9754519	0.0046	17.064	0
NM_213742.1	CALCB	turquoise	1263.8	1262.4168	0.9491665	0.01365273	1.576	turquoise	1040	1004.69	0.9977592	0.0001273	3.702	--
AY609441.1	APOO	turquoise	817.48	813.83698	0.8448858	0.07160431	17.476	turquoise	592.641	537.016	0.8484289	0.0692037	17.36	99
AY609490.1	CLDN11	turquoise	1503.5	1503.1447	0.9888032	0.00141985	7.414	turquoise	1012.47	985.968	0.9922843	0.0008126	7.33	0
AY609495.1	CCNI	turquoise	1401	1400.3582	0.9739832	0.00501779	268.918	turquoise	209.863	149.944	0.5508475	0.3359292	398.6	0
AY609516.1	LECT1	turquoise	1554.1	1553.7169	0.9965962	0.00023827	45.294	turquoise	1023.98	995.545	0.9947703	0.0004536	27.104	0.35
AY609525.1	CBX3	turquoise	159.69	126.26356	0.4059008	0.49775542	120.326	turquoise	313.851	218.874	0.6305386	0.254108	93.798	99
AY609579.1	C14orf37	turquoise	1370.3	1369.3508	0.9692549	0.00644147	15.78	turquoise	995.82	949.526	0.9841518	0.0023893	14.438	--
AY609735.1	GPR177	turquoise	1391.7	1391.1597	0.9690753	0.0064978	34.372	turquoise	925.261	901.099	0.9700576	0.0061916	31.114	0
AY609784.1	GSTM3	turquoise	102.92	77.241766	0.3411335	0.57423317	28.532	turquoise	275.216	190.595	0.5979169	0.2868809	23.84	0.184
AY609849.1	UBE2S	turquoise	485.4	470.59146	0.7072554	0.18155637	98.418	turquoise	740.088	698.541	0.9083056	0.0328687	106.416	--
AY609859.1	NECAP2	turquoise	949.91	948.72568	0.8882854	0.04406402	15.614	turquoise	896.588	874.789	0.9631118	0.0084576	18.31	0
AY609862.1	UBL7	turquoise	637.04	629.43413	0.7840517	0.11648364	37.714	turquoise	1002.13	961.028	0.9872424	0.0017264	37.97	--
AY609871.1	D4S234E	turquoise	1525.2	1524.6931	0.9931553	0.00067908	20.07	turquoise	1031.25	1001.18	0.996291	0.000271	65.096	--
AY609874.1	RAB18	turquoise	1462.9	1462.2663	0.983773	0.00247532	111.832	turquoise	972.237	889.256	0.9685321	0.0066692	147.482	0
AY609884.1	TRA2A	turquoise	216.93	185.28763	0.4761295	0.41752829	85.632	turquoise	996.491	971.226	0.9887058	0.0014384	79.11	--
AY609892.1	TMEM98	turquoise	302.4	287.73285	0.6059139	0.27874988	68.854	turquoise	1029.32	993.233	0.9946851	0.0004648	60.926	0
AY610017.1	CCT6A	turquoise	753.14	747.96232	0.8259744	0.08483575	106.458	turquoise	594.908	501.347	0.8361819	0.077608	127.482	--
AY610049.1	TSPAN7	turquoise	932.35	928.86868	0.8777816	0.05033989	24.394	turquoise	1015.27	988.541	0.9929221	0.0007141	2.098	--
AY610051.1	NUDC	turquoise	421.42	413.14169	0.6861997	0.20078603	66.024	turquoise	510.337	441.759	0.7995081	0.104464	66.064	--
AY610126.1	MEAF6	turquoise	1558.2	1557.7717	0.9985103	6.90E-05	8.656	turquoise	579.772	435.508	0.7963961	0.1068522	21.452	--
AY610316.1	OLFM1	turquoise	1522	1521.4669	0.9928179	0.00072986	4.306	turquoise	1019.09	991.564	0.9937292	0.0005955	13.874	--
AY610444.1	UBE2C	turquoise	1533.8	1533.3027	0.9951178	0.0004092	5.982	turquoise	310.626	221.295	0.6385601	0.2462143	7.566	0
AY610488.1	SNRPG	turquoise	1454.7	1453.9286	0.9836187	0.00251065	16.418	turquoise	336.502	236.039	0.6492887	0.2357633	27.326	--
AY609627.1	UBA5	turquoise	1402.3	1401.7144	0.9764952	0.00431054	8.366	turquoise	359.279	267.828	0.6867005	0.2003223	13.064	0.167
AY609641.1	MPPED2	turquoise	1532.9	1532.5221	0.9929308	0.00071274	7.252	turquoise	937.349	916.655	0.9742539	0.0049399	7.246	--
AY609650.1	REXO2	turquoise	1484.1	1483.6012	0.9864291	0.00189393	27.258	turquoise	1018.42	954.266	0.9860137	0.0019814	39.142	--
NM_001001264.1	WT1	turquoise	1334	1332.8881	0.9624075	0.00870003	0.69	turquoise	1034.47	1003.66	0.9969667	0.0002004	1.906	0
AY705919.1	UBE2R2	turquoise	1449.3	1448.6714	0.9790117	0.00363854	6.262	turquoise	492.844	424.02	0.7901376	0.111704	16.088	--
NM_001011727.1	AHCY	turquoise	118.34	62.959593	0.2304661	0.70918023	35.666	turquoise	996.101	937.512	0.9816107	0.0029852	28.39	0
NM_001012299.1	SCG2	turquoise	1553.6	1553.1829	0.9965278	0.00024548	0.426	turquoise	1015.27	988.541	0.9929221	0.0007141	7.512	--
AY803123.1	LOC10029	turquoise	1553.6	1553.1829	0.9965278	0.00024548	0.472	turquoise	1015.27	988.541	0.9929221	0.0007141	6.158	--
NM_001032355.1	DNMT1	turquoise	1406.7	1405.8402	0.9753117	0.00463933	21.748	turquoise	389.16	300.41	0.7104738	0.178666	25.116	0
NM_001032358.1	PIGK	turquoise	1470.4	1469.935	0.985809	0.00202501	23.64	turquoise	523.455	381.711	0.7678075	0.1295299	27.05	--
NM_001037145.1	SNCA	turquoise	914.91	913.72425	0.8720149	0.0538958	1.992	turquoise	758.42	741.016	0.921063	0.0263054	5.152	99
NM_001037147.1	PRPH	turquoise	1426	1425.5037	0.9754661	0.00459599	2.91	turquoise	294.351	235.308	0.6537674	0.2314373	3.316	0

NM_001037965.1	ID2	turquoise	1506.5	1506.1055	0.9892345	0.00133871	14.57	turquoise	1002.71	956.464	0.9856581	0.0020574	20.262	99
NM_001038636.1	PALM	turquoise	1508.2	1507.78	0.9905037	0.0011093	10.5	turquoise	837.538	751.722	0.927897	0.0229885	16.008	99
DQ351713.1	CRTAC1	turquoise	1351.3	1350.2355	0.9653116	0.00771504	2.51	turquoise	1005.59	977.733	0.9903828	0.0011305	3.138	--
NM_001039749.1	DKK3	turquoise	1483.9	1483.1907	0.9883426	0.00150826	9.4	turquoise	747.721	604.584	0.8746903	0.0522366	13.654	0.09
CB285786		turquoise	1553.6	1553.1829	0.9965278	0.00024548	1.086	turquoise	1015.27	988.541	0.9929221	0.0007141	5.616	--
CD572380		turquoise	1553.6	1553.1829	0.9965278	0.00024548	1.228	turquoise	1024.21	995.815	0.9948582	0.0004423	4.342	--
CD572468		turquoise	1553.6	1553.1829	0.9965278	0.00024548	0.85	turquoise	1004.25	979.276	0.9904644	0.0011162	3.336	--
AU295982		turquoise	1566.7	1566.3141	0.9992322	2.55E-05	2.764	turquoise	996.279	959.238	0.9869814	0.0017796	3.226	--
CF177631		turquoise	932.74	929.49189	0.8791127	0.04953007	9.798	turquoise	1005.78	949.837	0.9848387	0.0022359	12.612	--
CF178410		turquoise	1465.2	1464.7584	0.9810953	0.00311138	1.182	turquoise	1018.3	990.538	0.9935022	0.0006281	6.36	--
CF178517		turquoise	484.71	479.80555	0.7182335	0.1717522	28.26	turquoise	869.182	845.859	0.9542493	0.0116661	22.68	--
CF179479		turquoise	1505.4	1505.0215	0.988146	0.00154653	5.046	turquoise	981.643	956.676	0.9847975	0.002245	7.114	--
CF180093		turquoise	833.39	830.2785	0.850143	0.06805153	4.342	turquoise	538.886	476.942	0.8334548	0.0795195	5.944	--
CF359145		turquoise	845.93	843.77254	0.8497325	0.06832693	0.282	turquoise	1015.27	988.541	0.9929221	0.0007141	1.76	--
CF363170		turquoise	1553.6	1553.1829	0.9965278	0.00024548	0.142	turquoise	1015.27	988.541	0.9929221	0.0007141	1.692	--
CF366893		turquoise	1568.1	1567.7087	0.9994784	1.43E-05	2.816	turquoise	614.819	507.747	0.8401736	0.074836	5.26	--
CF367389		turquoise	1115.5	1113.8644	0.9209456	0.02636359	1.156	turquoise	1016.19	989.215	0.9931356	0.000682	4.562	--
CF367397		turquoise	1553.6	1553.1829	0.9965278	0.00024548	1.464	turquoise	1015.27	988.541	0.9929221	0.0007141	2.368	--
CF791017		turquoise	1543.4	1542.9666	0.9965448	0.00024368	0.604	turquoise	1000.17	975.2	0.9895506	0.0012802	2.074	--
CF791227		turquoise	1541.1	1540.7394	0.9950624	0.00041619	15.174	turquoise	829.197	707.382	0.9126179	0.0305979	28.228	--
BX665171		turquoise	1519.5	1518.9825	0.9920329	0.00085263	6.312	turquoise	537.906	508.751	0.8317847	0.0806972	9.566	--
BX665337		turquoise	1377	1376.0224	0.9717854	0.00566498	20.1	turquoise	870.582	839.029	0.9532101	0.012064	20.602	0
BX665422		turquoise	544.66	536.37852	0.7539834	0.14095041	103.442	turquoise	489.78	401.899	0.7748127	0.1238531	108.826	--
BX665515		turquoise	1333.7	1332.7863	0.9640027	0.00815416	30.71	turquoise	968.086	889.355	0.9686512	0.0066315	34.544	--
BX665627		turquoise	818.19	814.75268	0.8547508	0.06498434	15.808	turquoise	593.867	497.472	0.830038	0.0819346	17.906	--
BX666107		turquoise	1566.9	1566.484	0.9987289	5.44E-05	3.904	turquoise	1020.62	991.087	0.9940142	0.0005554	3.844	--
BX666112		turquoise	207.75	166.36673	0.4521666	0.44455209	23.298	turquoise	1042.1	990.883	0.9946168	0.0004737	18.444	--
BX667112		turquoise	253.94	214.40285	0.504613	0.38592354	86.772	turquoise	963.006	898.311	0.9714388	0.0057694	105.314	--
BX667489		turquoise	1553.6	1553.1829	0.9965278	0.00024548	0.284	turquoise	1010.22	984.289	0.9918412	0.0008836	3.122	--
BX667494		turquoise	433.45	416.99407	0.6808827	0.20572736	19.134	turquoise	1047.56	993.389	0.9954697	0.0003658	14.598	--
BX666727		turquoise	866.73	864.94405	0.8565286	0.06381279	8.978	turquoise	866.713	849.7	0.9549882	0.011386	8.982	--
BX666776		turquoise	1333.8	1332.9908	0.9612576	0.00910067	10.034	turquoise	1024.86	994.911	0.9946809	0.0004653	12.394	--
BX667130		turquoise	930.59	927.80585	0.8798166	0.04910346	28.676	turquoise	991.655	967.623	0.9874736	0.0016798	23.638	--
BX667206		turquoise	576.04	564.61064	0.7509324	0.143509	14.928	turquoise	1000.5	975.765	0.9896239	0.0012668	24.222	--
BX667724		turquoise	1553.6	1553.1829	0.9965278	0.00024548	0.094	turquoise	1015.27	988.541	0.9929221	0.0007141	2.03	--
BX668405		turquoise	273.84	268.95686	0.6111962	0.27341307	38.17	turquoise	194.04	156.093	0.5671019	0.318769	22.414	--

BX668485	turquoise	1554.9	1554.4992	0.9967423	0.00022309	30.222	turquoise	1026.5	997.622	0.9953409	0.0003815	15.512	--
BX669151	turquoise	1553.6	1553.1829	0.9965278	0.00024548	6.754	turquoise	1018.01	990.802	0.9935229	0.0006251	14.56	--
BX669234	turquoise	1553.6	1553.1829	0.9965278	0.00024548	0.142	turquoise	1015.27	988.541	0.9929221	0.0007141	2.3	--
BX669322	turquoise	1425.4	1424.5967	0.9777035	0.0039832	19.196	turquoise	1046.38	1007.48	0.9983255	8.22E-05	40.788	99
BX670093	turquoise	188.29	104.15361	0.2818851	0.64590421	3.854	turquoise	1043.16	1008.64	0.9984168	7.56E-05	4.052	99
BX670180	turquoise	1394.3	1393.5899	0.9747276	0.00480453	119.022	turquoise	730.149	606.637	0.8763964	0.051187	169.282	--
BX670270	turquoise	1484.1	1483.6872	0.9851047	0.00217739	14.356	turquoise	506.907	472.998	0.8333572	0.0795882	24.598	--
BX670323	turquoise	189.16	102.82891	0.2621294	0.67010897	23.876	turquoise	820.242	692.322	0.9078103	0.0331329	24.796	0
BX670437	turquoise	1392.3	1391.7539	0.9676894	0.00693801	6.112	turquoise	1010.23	984.192	0.9918494	0.0008822	12.802	--
BX670438	turquoise	128	105.34445	0.3878004	0.51890869	4.462	turquoise	735.816	718.24	0.913859	0.0299541	4.222	--
BX670600	turquoise	465.33	453.14829	0.7104975	0.1786447	7.858	turquoise	908.916	819.771	0.9485354	0.0139064	2.646	--
BX670766	turquoise	1553.6	1553.1829	0.9965278	0.00024548	0.566	turquoise	1018.71	991.371	0.9936743	0.0006034	11.582	0
BX670931	turquoise	273.54	236.93788	0.5250387	0.36362868	17.584	turquoise	1054.15	1011.11	0.999521	1.26E-05	16.744	--
BX671254	turquoise	1533.6	1533.1632	0.9947684	0.00045388	5.452	turquoise	1041.37	988.531	0.9941268	0.0005398	15.596	--
BX671284	turquoise	893.24	890.45675	0.8691231	0.05570749	1.112	turquoise	1018.53	984.68	0.9923004	0.0008101	3.69	--
BX671531	turquoise	1505.7	1505.2879	0.9876046	0.00165355	2.596	turquoise	753.884	703.16	0.911741	0.0310555	12.096	--
BX671826	turquoise	1319.9	1319.2268	0.9560031	0.01100475	3.708	turquoise	963.136	941.561	0.9807639	0.0031934	5.258	--
BX672205	turquoise	1553.6	1553.1829	0.9965278	0.00024548	6.754	turquoise	973.517	945.808	0.9827056	0.0027231	2.944	--
BX672270	turquoise	1478.9	1478.4813	0.9837619	0.00247785	3.372	turquoise	937.474	899.465	0.9705728	0.006033	6.036	0.432
BX672673	turquoise	1452.4	1451.7872	0.9796013	0.00348662	24.538	turquoise	768.907	706.319	0.9152808	0.0292219	27.486	--
BX672674	turquoise	1453.9	1453.3034	0.9791473	0.00360341	19.118	turquoise	857.911	795.297	0.9417122	0.0167443	52.556	--
BX673261	turquoise	563.01	552.91864	0.753879	0.14103773	5.262	turquoise	976.147	950.429	0.9833541	0.0025716	4.056	--
BX673328	turquoise	1100.7	1099.9117	0.9229626	0.025369	4.828	turquoise	668.453	647.183	0.8903038	0.0428887	6.204	--
BX673363	turquoise	1535.3	1534.8472	0.9932382	0.00066679	14.174	turquoise	332.134	256.647	0.6717036	0.2143364	21.148	99
BX673388	turquoise	1336.4	1335.5657	0.9619767	0.00884945	1.496	turquoise	1023.76	992.677	0.9941693	0.000534	4.038	--
BX673567	turquoise	862.91	861.5948	0.8620943	0.06018845	12.252	turquoise	957.09	935.878	0.9790444	0.0036301	14.046	--
BX673587	turquoise	1492.9	1492.3381	0.9888559	0.00140985	75.96	turquoise	527.405	389.701	0.7738595	0.1246211	181.912	--
BX673843	turquoise	1057.4	1056.3212	0.9107373	0.03158181	33.454	turquoise	961.15	873.597	0.9642402	0.0080739	36.516	--
BX674049	turquoise	1516.5	1515.9459	0.9919273	0.00086963	7.016	turquoise	931.59	848.879	0.957871	0.0103144	10.904	--
BX674094	turquoise	36.406	29.18535	0.2116853	0.73250063	88.892	turquoise	1023.95	994.375	0.9945688	0.0004801	76.426	--
BX674245	turquoise	158.92	137.05261	0.4495773	0.44749479	33.57	turquoise	853.741	797.812	0.9420499	0.0165998	28.972	0
BX674443	turquoise	1158.8	1157.3087	0.9311339	0.02146862	1.79	turquoise	1032.9	989.885	0.9940264	0.0005537	4.242	--
BX674924	turquoise	1082.7	1081.9528	0.9184457	0.02761333	1.196	turquoise	963.516	940.985	0.9807165	0.0032052	5.376	99
BX675092	turquoise	1479.1	1478.6309	0.984875	0.00222786	19.5	turquoise	691.843	635.987	0.8858646	0.0454868	17.272	--
BX675290	turquoise	1489.5	1488.9343	0.9879029	0.00159428	1.398	turquoise	1043.3	1009.63	0.9986144	6.19E-05	3.038	--
BX675465	turquoise	1451.3	1450.6315	0.9819085	0.00291315	14.828	turquoise	341.26	266.359	0.6767253	0.2096144	18.534	--

BX675792	turquoise	132.08	110.7675	0.3951871	0.51025436	112.632	turquoise	522.219	491.58	0.823978	0.0862723	92.492	--
BX675898	turquoise	1168.1	1167.2146	0.9280019	0.0229387	10.754	turquoise	1005.6	980.122	0.9907692	0.0010631	13.998	99
BX675902	turquoise	1553.6	1553.1829	0.9965278	0.00024548	3.402	turquoise	1015.27	988.541	0.9929221	0.0007141	14.548	--
BP156692	turquoise	1480.4	1479.9351	0.9876263	0.00164921	6.836	turquoise	941.862	862.222	0.9617232	0.0089378	7.898	--
BP156716	turquoise	1134.6	1132.9607	0.9270924	0.02337147	18.34	turquoise	1000.33	968.169	0.9885147	0.001475	23.024	--
BP164138	turquoise	447.1	439.36387	0.7053465	0.18327693	55.516	turquoise	959.361	936.385	0.9796484	0.0034746	63.162	--
BP165213	turquoise	1463.3	1462.5903	0.984879	0.00222699	7.12	turquoise	664.478	617.201	0.8793678	0.0493753	11.382	--
BP166941	turquoise	1456	1455.2799	0.9820791	0.00287213	2.184	turquoise	412.147	331.194	0.7403061	0.1525247	0.9	--
BP169101	turquoise	127.71	105.6723	0.3843044	0.52301489	1.758	turquoise	987.529	964.265	0.9865505	0.0018686	5.016	--
BP169289	turquoise	358.94	356.22801	0.6841766	0.20266223	11.88	turquoise	753.031	717.113	0.9174659	0.0281082	23.154	--
BP170588	turquoise	1492.3	1491.7272	0.9891082	0.0013623	54.598	turquoise	967.477	909.16	0.9752929	0.0046446	60.676	0.394
BP170821	turquoise	749.83	745.3353	0.8303924	0.08168311	4.604	turquoise	1023.26	993.656	0.994349	0.0005095	6.422	0
BP171491	turquoise	1507.3	1506.7814	0.9903954	0.0011283	16.57	turquoise	1042	1006.82	0.9980075	0.0001067	20.81	--
BP173206	turquoise	1566.3	1565.8526	0.9991557	2.94E-05	14.914	turquoise	1000.17	926.31	0.9785357	0.0037628	23.708	--
BP436950	turquoise	1523.8	1523.3263	0.9930891	0.00068894	65.68	turquoise	604.687	501.472	0.8315923	0.0808332	80.48	--
BP438990	turquoise	1409.1	1408.4669	0.9710859	0.00587633	21.354	turquoise	526.981	513.064	0.8376358	0.0765947	20.816	--
BP440821	turquoise	1291.7	1291.0767	0.9506984	0.01304329	4.036	turquoise	872.006	833.377	0.9524036	0.0123757	4.504	--
BP141462	turquoise	1564.9	1564.4665	0.9991211	3.13E-05	9.096	turquoise	41.9174	12.5864	0.126011	0.8399834	17.876	0
BP144557	turquoise	1525.8	1525.3859	0.9933492	0.00065045	3.86	turquoise	692.206	636.258	0.8936125	0.0409841	8.908	--
BP148354	turquoise	1568.2	1567.8575	0.9990179	3.69E-05	2.068	turquoise	890.331	806.981	0.9452713	0.0152426	2.402	--
BP153914	turquoise	1558.5	1558.1344	0.9970811	0.00018922	2.684	turquoise	996.818	960.685	0.9869137	0.0017935	8.928	--
CK449358	turquoise	1454	1453.3486	0.98217	0.00285034	10.244	turquoise	1028.28	993.235	0.9948395	0.0004447	18.186	--
CK450265	turquoise	1543.4	1542.9737	0.9960353	0.0002995	8.192	turquoise	1003.26	939.958	0.9820327	0.0028833	18.226	--
CK450911	turquoise	655.83	649.49586	0.7931683	0.10934637	14.738	turquoise	795.09	777.455	0.9366053	0.0189775	12.064	--
CK451122	turquoise	1549.9	1549.4394	0.9975517	0.00014537	16.78	turquoise	618.129	489.366	0.8262257	0.0846554	8.996	--
CK451295	turquoise	1276	1274.7178	0.954212	0.01168036	23.548	turquoise	986.315	935.195	0.9807557	0.0031954	20.83	--
CK451467	turquoise	1567.2	1566.7471	0.9994381	1.60E-05	18.308	turquoise	1044.47	1008.05	0.998321	8.26E-05	19.166	99
CK451551	turquoise	1143.1	1141.0577	0.9258056	0.02398819	79.476	turquoise	942.408	849.632	0.9576846	0.0103826	75.596	--
CK452729	turquoise	1105.3	1102.9051	0.9171194	0.02828389	27.88	turquoise	1043.88	992.651	0.9951884	0.0004004	32.63	--
CK452774	turquoise	1348.8	1347.7159	0.9659335	0.0075092	17.6	turquoise	1042.39	1005.37	0.9978943	0.000116	15.246	--
CK452818	turquoise	1219	1218.363	0.9421278	0.01656658	4.234	turquoise	997.126	963.92	0.9879252	0.0015899	10.684	--
CK452842	turquoise	1308.5	1307.771	0.9564142	0.01085156	6.334	turquoise	948.599	922.259	0.9761083	0.0044172	6.064	--
CK452952	turquoise	910.33	907.37344	0.8777018	0.05038857	136.606	turquoise	428.612	331.015	0.7300868	0.1613445	94.798	--
CK452970	turquoise	1571.4	1571.0325	0.9997393	5.05E-06	1.924	turquoise	937.837	858.929	0.960262	0.0094523	3.152	--
CK452980	turquoise	1322.2	1321.4826	0.9637749	0.00823142	13.508	turquoise	353.615	228.089	0.6557709	0.2295093	20.812	0.15
CK453021	turquoise	1340.9	1339.916	0.9634823	0.00833097	22.298	turquoise	681.854	582.816	0.8670909	0.0569918	33.072	99

CK454052	turquoise	598.42	590.35082	0.7707728	0.12711766	2.354	turquoise	1032.78	1000.94	0.9962882	0.0002713	4.412	--
CK454514	turquoise	615.56	610.26563	0.7810684	0.11884849	53.26	turquoise	938.47	914.486	0.9740481	0.0049991	43.462	--
CK454991	turquoise	1469.2	1468.6119	0.9853948	0.00211418	38.736	turquoise	862.245	755.868	0.9301224	0.02194	47.804	--
CK455126	turquoise	1481.7	1481.2482	0.9848163	0.00224083	49.388	turquoise	248.686	196.346	0.6189095	0.2656695	71.286	--
CK455184	turquoise	1304.7	1303.6486	0.9584305	0.01011041	24.018	turquoise	203.765	118.391	0.5309738	0.3572112	38.158	--
CK455320	turquoise	77.403	51.400012	0.2431458	0.69349515	4.188	turquoise	1005.15	952.532	0.9847682	0.0022515	8.16	--
CK455502	turquoise	1550.5	1550.0676	0.9956518	0.00034397	4.784	turquoise	547.5	532.49	0.848513	0.069147	4.258	--
CK455968	turquoise	190.76	165.9108	0.497607	0.39364296	6.898	turquoise	904.854	879.334	0.9646123	0.0079487	7.82	--
CK456799	turquoise	1521.1	1520.7294	0.9911154	0.00100394	2.75	turquoise	1051.45	1012.64	0.999701	6.21E-06	6.734	--
CK456945	turquoise	1554.9	1554.4797	0.9970531	0.00019195	5.212	turquoise	1001.4	976.424	0.9897691	0.0012403	12.076	--
CK458238	turquoise	1333	1331.8174	0.962473	0.00867741	15.708	turquoise	1020.55	959.242	0.9868046	0.001816	11.296	--
CK458354	turquoise	1562.1	1561.7248	0.9982378	8.88E-05	10.322	turquoise	509.9	383.472	0.7701555	0.1276187	14.57	--
CK458731	turquoise	368.4	344.75517	0.6266217	0.25798671	0.728	turquoise	1020.88	993.049	0.9941423	0.0005377	2.27	--
CK458744	turquoise	935.51	931.51797	0.8760914	0.0513742	32.046	turquoise	787.714	659.635	0.896853	0.0391456	29.738	99
CK459071	turquoise	1444.7	1443.9641	0.981849	0.00292751	14.756	turquoise	844.661	744.07	0.9255128	0.0241292	9.788	0
CK459081	turquoise	601.94	595.64335	0.7776696	0.12155995	71.884	turquoise	978.02	921.99	0.9777907	0.0039599	67.7	--
CK459449	turquoise	1564.8	1564.3642	0.9992058	2.69E-05	1.634	turquoise	965.099	936.428	0.9809871	0.0031381	5.36	--
CK460198	turquoise	1132.7	1131.1413	0.9212089	0.02623306	1.226	turquoise	1011.25	982.917	0.991849	0.0008823	4.844	--
CK461258	turquoise	1553.6	1553.1829	0.9965278	0.00024548	0.566	turquoise	1015.27	988.541	0.9929221	0.0007141	3.586	--
CK461875	turquoise	1512	1511.6306	0.9885909	0.00146038	3.642	turquoise	657.791	635.208	0.8846065	0.046232	5.884	--
CK462022	turquoise	779.66	776.22375	0.8453497	0.07128859	3.7	turquoise	982.577	950.197	0.9846629	0.0022748	3.62	--
CK462358	turquoise	1507.4	1506.9445	0.9901349	0.00117447	1.504	turquoise	1021.31	992.243	0.9940312	0.0005531	7.754	--
CK462854	turquoise	1490.4	1490.0325	0.987529	0.00166867	23.462	turquoise	1040.27	1006.68	0.9978953	0.0001159	27.63	--
CK463343	turquoise	1561.1	1560.6266	0.9990325	3.61E-05	15.106	turquoise	1020.88	975.716	0.9909538	0.0010314	9.706	99
CK465210	turquoise	1413.2	1412.7453	0.9750951	0.00470036	8.948	turquoise	841.557	775.055	0.9389622	0.0179356	9.54	--
CK466505	turquoise	1145.8	1144.1783	0.9294311	0.02226394	16.828	turquoise	1043.29	994.672	0.9954824	0.0003642	16.412	--
CK466890	turquoise	721.4	714.11036	0.8106699	0.09603467	1.842	turquoise	1037.49	1002.36	0.9968293	0.0002142	7.386	--
CK467233	turquoise	1339.3	1338.7236	0.9589045	0.0099387	0.756	turquoise	1015.27	988.541	0.9929221	0.0007141	3.384	--
CK467614	turquoise	1498.8	1498.1999	0.989537	0.00128273	40.584	turquoise	635.656	583.483	0.8657364	0.057853	37.77	--
CK468440	turquoise	1551.2	1550.8342	0.9965866	0.00023927	4.264	turquoise	556.272	407.565	0.7812596	0.1186965	13.258	--
BX914375	turquoise	1553.6	1553.1829	0.9965278	0.00024548	1.89	turquoise	1005.41	980.176	0.9907131	0.0010728	4.366	--
BX914760	turquoise	1553.6	1553.1829	0.9965278	0.00024548	0.804	turquoise	1015.27	988.541	0.9929221	0.0007141	3.518	--
BX914900	turquoise	161.86	144.91294	0.4705464	0.42379009	14.344	turquoise	649.212	634.788	0.8830835	0.047139	17.346	--
BX914973	turquoise	1571.1	1570.748	0.9996267	8.66E-06	1.406	turquoise	977.993	918.488	0.9769811	0.0041779	1.396	--
BX915431	turquoise	1555.8	1555.4234	0.9978084	0.00012312	9.74	turquoise	1016.66	982.999	0.9918482	0.0008824	28.666	--
BX915705	turquoise	1453.8	1453.0907	0.9823162	0.00281542	2.426	turquoise	1007.91	982.278	0.9913196	0.0009696	5.18	--

BX915997	turquoise	1493	1492.5153	0.9865947	0.00185941	17.558	turquoise	841.446	776.582	0.9356732	0.0193949	19.434	--
BX917118	turquoise	1258.5	1257.3402	0.952055	0.01251124	42.718	turquoise	457.523	354.394	0.7565221	0.1388318	40.236	--
BX917311	turquoise	1070.1	1067.3775	0.9093819	0.0322971	126.948	turquoise	545.816	392.587	0.7724181	0.1257851	133.716	--
BX917504	turquoise	1553.6	1553.1829	0.9965278	0.00024548	0.708	turquoise	1008.06	982.377	0.9913589	0.000963	5.872	--
BX917585	turquoise	475.07	468.07365	0.7315336	0.16008712	33.224	turquoise	674.681	520.29	0.8404378	0.0746537	34.478	99
BX917665	turquoise	513.91	510.63403	0.7370203	0.15534473	0.588	turquoise	795.15	778.155	0.9347568	0.019808	3.282	--
BX918032	turquoise	1553.6	1553.1829	0.9965278	0.00024548	0.142	turquoise	1015.27	988.541	0.9929221	0.0007141	1.556	--
BX918173	turquoise	593.5	582.51004	0.7594173	0.13642727	17.148	turquoise	1026.03	973.844	0.9905859	0.0010949	12.954	--
BX918187	turquoise	157.38	83.348877	0.2331709	0.70583017	165.034	turquoise	1008.35	939.735	0.9819762	0.0028969	144.09	0.404
BX918528	turquoise	1553.6	1553.1829	0.9965278	0.00024548	0.756	turquoise	1015.27	988.541	0.9929221	0.0007141	9.068	--
BX918613	turquoise	1322.5	1321.8734	0.9561346	0.01095566	12.638	turquoise	682.513	599.9	0.8725967	0.0535336	30.818	--
BX918737	turquoise	1327.3	1326.7036	0.9553791	0.0112386	19.348	turquoise	737.459	652.451	0.8937059	0.0409307	27.558	--
BX918832	turquoise	316.98	304.6101	0.6268074	0.25780241	1.19	turquoise	1015.27	988.541	0.9929221	0.0007141	2.774	0
BX919298	turquoise	174.98	158.21865	0.4918255	0.4000403	2.492	turquoise	918.372	898.839	0.9692911	0.0064301	2.952	--
BX919676	turquoise	136.18	121.96163	0.4480729	0.4492065	24.406	turquoise	998.243	967.233	0.988562	0.0014659	31.16	--
BX919821	turquoise	1345.6	1344.9948	0.9586317	0.01003743	2.232	turquoise	865.971	802.224	0.9455562	0.0151244	4.474	--
BX920452	turquoise	847.98	845.88179	0.8485763	0.06910444	102.958	turquoise	972.495	908.019	0.9742284	0.0049472	143.614	--
BX920732	turquoise	1252.4	1251.3773	0.9502187	0.01323317	14.848	turquoise	993.355	938.176	0.9819702	0.0028983	13.548	--
BX921084	turquoise	255.22	233.78845	0.5694528	0.3163061	19.226	turquoise	989.294	946.583	0.9831466	0.0026198	46.104	99
BX921278	turquoise	1137.4	1135.6715	0.9286666	0.02262408	43.956	turquoise	128.107	77.3441	0.4384696	0.4601668	57.126	--
BX921846	turquoise	1421.8	1420.9375	0.9774369	0.00405467	130.464	turquoise	339.26	221.884	0.6342823	0.2504155	127.936	--
BX922199	turquoise	1554	1553.473	0.9980179	0.0001059	62.802	turquoise	556.365	427.354	0.8010447	0.1032909	43.902	--
BX922273	turquoise	1548.7	1548.2519	0.9970448	0.00019276	24.558	turquoise	1043.15	988.886	0.9943162	0.000514	42.66	99
BX922394	turquoise	433.54	426.12918	0.7087231	0.18023656	17.15	turquoise	970.974	945.883	0.9823505	0.0028072	10.072	--
BX922568	turquoise	1543.8	1543.4253	0.9950151	0.00042218	2.706	turquoise	1017.09	989.967	0.9933698	0.0006474	5.37	--
BX922590	turquoise	1540.9	1540.5458	0.9937491	0.0005927	18.862	turquoise	349.153	276.162	0.692138	0.1953074	16.442	--
BX922774	turquoise	900.34	898.35297	0.8601638	0.06143807	11.556	turquoise	1014.17	985.201	0.9923845	0.0007969	19.42	--
BX922846	turquoise	891.25	889.90018	0.8679988	0.0564169	159.298	turquoise	1052.97	1011.87	0.9995056	1.32E-05	226.234	--
BX922933	turquoise	155.49	136.56422	0.4278334	0.4723728	25.106	turquoise	789.518	765.093	0.9299878	0.0220029	16.608	--
BX923391	turquoise	1048.2	1045.7925	0.9083435	0.03284854	19.804	turquoise	764.009	709.518	0.9133061	0.0302403	27.978	--
BX923394	turquoise	1477.7	1477.1571	0.9845562	0.00229857	4.048	turquoise	1006.86	980.591	0.9909575	0.0010308	8.01	--
BX923690	turquoise	1539.5	1539.037	0.9957672	0.00033037	32.738	turquoise	1037.13	999.142	0.99675	0.0002223	38.046	--
BX923706	turquoise	1501.4	1500.7932	0.9910854	0.00100903	22.152	turquoise	616.259	479.785	0.8201508	0.089047	40.998	--
BX923820	turquoise	1506.6	1506.1707	0.9883907	0.00149895	1.312	turquoise	997.435	973.321	0.9889052	0.0014005	3.35	--
BX923928	turquoise	154.35	96.725017	0.323839	0.5950001	21.836	turquoise	1011.38	974.469	0.9903654	0.0011336	23.184	--
BX924061	turquoise	1555.1	1554.6858	0.9972161	0.00017625	58.646	turquoise	938.041	849.171	0.9572839	0.0105298	56.584	--

BX924264	turquoise	962.94	960.66684	0.8875719	0.0444819	24.054	turquoise	348.872	278.664	0.6911434	0.196222	26.39	0
BX925008	turquoise	1564.1	1563.6999	0.9993827	1.84E-05	24.986	turquoise	980.761	900.764	0.9716526	0.0057049	39.96	--
BX925280	turquoise	1550.1	1549.6882	0.9956158	0.00034825	2.168	turquoise	1027.15	997.565	0.9953572	0.0003795	6.568	--
BX925298	turquoise	1064.7	1062.9895	0.9147224	0.02950879	1.382	turquoise	1040.48	1007.86	0.9981511	9.54E-05	5.912	--
BX925827	turquoise	1553.6	1553.1829	0.9965278	0.00024548	0.992	turquoise	1010.32	983.693	0.9918977	0.0008744	3.058	--
BX925865	turquoise	1462	1461.575	0.9800459	0.00337347	13.58	turquoise	562.147	428.586	0.7948754	0.1080251	27.444	--
BX925898	turquoise	1525.1	1524.6283	0.9936628	0.00060502	41.234	turquoise	357.278	290.694	0.6978904	0.1900414	67.11	--
BX926176	turquoise	946.94	945.31914	0.8726708	0.0534875	20.288	turquoise	1034.03	1001.11	0.9964268	0.0002563	37.862	--
BX926404	turquoise	1435.9	1435.33	0.9792381	0.00357995	9.854	turquoise	1043.18	1000.39	0.9967202	0.0002254	17.164	--
BX926703	turquoise	1535.2	1534.7105	0.9946951	0.00046345	2.62	turquoise	1009.79	942.226	0.9825265	0.0027654	2.434	--
BX926738	turquoise	1533.8	1533.4418	0.9933537	0.00064979	6.49	turquoise	962.156	879.594	0.965731	0.007576	11.54	--
BX926797	turquoise	1513.5	1512.9115	0.9927291	0.00074344	6.608	turquoise	299.32	225.115	0.6396702	0.2451273	9.42	--
BX926895	turquoise	715.51	711.44811	0.8125092	0.09466653	4.02	turquoise	1018.5	984.274	0.9924808	0.0007818	4.382	--
BX926938	turquoise	1532	1531.6105	0.9932286	0.00066821	1.714	turquoise	863.257	777.359	0.9374541	0.0186001	4.724	--
BX926955	turquoise	1489.6	1489.0542	0.9873055	0.00171367	13.872	turquoise	277.009	225.324	0.6521551	0.2329922	10.502	--
CN154817	turquoise	868.98	866.88242	0.8668749	0.0571289	0.804	turquoise	1015.27	988.541	0.9929221	0.0007141	1.692	--
CN154979	turquoise	417.38	410.19371	0.6813513	0.20529053	13.426	turquoise	540.389	512.715	0.832676	0.080068	22.648	--
CN155399	turquoise	1564.6	1564.2305	0.9985754	6.45E-05	5.124	turquoise	1037.14	1005.05	0.9973528	0.0001634	8.092	--
CN155773	turquoise	1564.9	1564.5476	0.9989255	4.23E-05	63.076	turquoise	1048.59	1005.12	0.997871	0.0001179	57.64	--
CN156021	turquoise	1472.5	1472.0498	0.984514	0.00230797	48.504	turquoise	754.046	649.556	0.8935215	0.0410361	45.198	--
CN156223	turquoise	1476.6	1476.0506	0.9833702	0.0025679	0.944	turquoise	994.237	970.24	0.9881179	0.001552	2.672	--
CN156290	turquoise	1109.1	1107.3334	0.9224371	0.02562693	5.086	turquoise	1008.16	980.723	0.9912093	0.0009881	8.224	--
CN156664	turquoise	1563.7	1563.3192	0.9982295	8.94E-05	3.542	turquoise	1011.71	985.579	0.9921675	0.0008311	4.136	--
CN156970	turquoise	595.79	592.75292	0.7716924	0.12637233	0.814	turquoise	1004.31	976.897	0.990236	0.0011565	4.2	--
CN157016	turquoise	1553.6	1553.1829	0.9965278	0.00024548	0.708	turquoise	1033.9	1003.25	0.9968539	0.0002117	1.972	--
CN157134	turquoise	1501.8	1501.3865	0.9890541	0.00137245	31.836	turquoise	978.785	946.434	0.9829373	0.0026686	38.886	--
CN157778	turquoise	1553.6	1553.1829	0.9965278	0.00024548	2.55	turquoise	1015.27	988.541	0.9929221	0.0007141	7.038	--
CN157824	turquoise	1214.4	1213.2163	0.9354898	0.01947729	21.472	turquoise	569.212	542.337	0.8547218	0.0650035	27.176	--
CN157905	turquoise	725.08	718.75928	0.8142353	0.09338796	17.276	turquoise	1045.09	1009.53	0.9987019	5.61E-05	14.794	--
CN157954	turquoise	1507.4	1506.9445	0.9901349	0.00117447	1.504	turquoise	1009.67	983.801	0.9917181	0.0009036	4.24	--
CN158074	turquoise	546.05	541.22333	0.7614401	0.13475468	6.302	turquoise	1017.96	988.693	0.9931235	0.0006838	8.3	--
CN158100	turquoise	421.37	409.44359	0.6938234	0.1937603	82.262	turquoise	812.952	751.032	0.927483	0.0231853	65.706	--
CN158117	turquoise	705.17	699.17207	0.8120919	0.09497637	16.028	turquoise	1013.82	987.167	0.9925542	0.0007704	16.682	--
CN158744	turquoise	1406.5	1405.6224	0.9749295	0.0047472	9.86	turquoise	1000.8	953.879	0.9850616	0.0021868	11.532	--
CN158770	turquoise	1570.6	1570.2451	0.9998803	1.57E-06	10.214	turquoise	785.286	738.742	0.9263456	0.0237288	15.746	--
CN158781	turquoise	1534.4	1533.8719	0.9952592	0.00039157	8.124	turquoise	711.276	695.854	0.9064439	0.033865	18.344	--

CN158983	turquoise	661.37	654.37735	0.7946307	0.1082142	0.464	turquoise	982.447	958.52	0.985326	0.0021291	1.706	--
CN159132	turquoise	1471.9	1471.5148	0.9816363	0.00297902	47.638	turquoise	977.723	903.604	0.9726281	0.0054138	51.418	--
CN160793	turquoise	1293.4	1292.1779	0.9571891	0.01056468	15.67	turquoise	939.696	912.804	0.9756388	0.0045477	11.026	--
CN161415	turquoise	1485.6	1485.051	0.9868506	0.00180648	2.44	turquoise	1009.15	982.918	0.9914494	0.0009479	10.642	--
CN161545	turquoise	1556.3	1555.8875	0.9983019	8.40E-05	45.494	turquoise	631.605	480.284	0.8222776	0.0875018	45.312	--
CN161554	turquoise	264.09	253.57089	0.5849578	0.30018552	18.886	turquoise	976.125	953.914	0.9838873	0.0024492	22.266	--
CN161683	turquoise	1509.8	1509.4403	0.9891782	0.00134921	2.248	turquoise	578.655	429.734	0.7954313	0.1075959	4.378	0
CN161880	turquoise	905.33	902.73193	0.8762069	0.05130329	16.086	turquoise	647.725	507.375	0.8320152	0.0805344	29.53	--
CN162042	turquoise	1553.6	1553.1829	0.9965278	0.00024548	2.126	turquoise	1043.12	991.769	0.9953233	0.0003837	1.638	--
CN162073	turquoise	60.709	40.989662	0.2149701	0.72841451	95.3	turquoise	829.404	774.972	0.9352368	0.0195912	116.874	--
CN162090	turquoise	1552.3	1551.869	0.9976084	0.00014035	3.232	turquoise	982.015	949.301	0.9843732	0.0023395	5.2	--
CN162295	turquoise	1553.6	1553.1829	0.9965278	0.00024548	1.654	turquoise	460.504	406.165	0.7780741	0.1212363	2.322	--
CN162405	turquoise	298.54	292.61508	0.6469429	0.23803784	9.454	turquoise	1032.74	1001.27	0.9966274	0.000235	12.138	--
CN162710	turquoise	1518	1517.3843	0.9931786	0.00067562	10.098	turquoise	713.585	633.974	0.8944452	0.0405091	7.522	--
CN163671	turquoise	1364.3	1363.233	0.9680073	0.00683621	60.588	turquoise	1050.36	1007.29	0.9984115	7.60E-05	48.652	99
CN163855	turquoise	1124.8	1123.0749	0.9220604	0.02581234	29.838	turquoise	503.199	489.001	0.8207504	0.0886105	26.968	--
CN164027	turquoise	1380.3	1379.5537	0.9731156	0.00527017	47.332	turquoise	690.601	663.01	0.8949332	0.0402315	54.226	--
CN164462	turquoise	1499.1	1498.5237	0.9905458	0.00110192	2.838	turquoise	1020.31	991.668	0.9937878	0.0005872	4.018	--
CN165181	turquoise	1553.6	1553.1829	0.9965278	0.00024548	3.024	turquoise	1021.62	993.743	0.9943055	0.0005154	18.71	--
CN165208	turquoise	1415.5	1414.9781	0.9739328	0.00503235	2.786	turquoise	1005.9	980.437	0.9908699	0.0010458	2.886	--
CN165377	turquoise	1028.9	1026.1668	0.9012552	0.0366915	10.746	turquoise	1040.19	1007.77	0.9981063	9.89E-05	11.768	--
CN165379	turquoise	151.45	132.87163	0.4615615	0.43391156	5.616	turquoise	969.208	944.381	0.9815642	0.0029965	6.446	--
CN166224	turquoise	1516	1515.4927	0.9924023	0.00079408	0.836	turquoise	1031.75	999.238	0.995978	0.000306	2.98	--
AJ657682	turquoise	1543.5	1543.0265	0.9965136	0.00024698	7.616	turquoise	525.327	441.926	0.8147539	0.0930049	8.294	--
AJ680676	turquoise	1553.6	1553.1829	0.9965278	0.00024548	0.566	turquoise	1005.52	980.098	0.9907845	0.0010605	1.904	--
CO938694	turquoise	1547.5	1547.1368	0.995807	0.00032572	3.728	turquoise	1040.96	1006.78	0.9979646	0.0001102	2.228	--
CJ008156	turquoise	175.55	140.6405	0.406152	0.49746308	29.64	turquoise	981.283	938.781	0.9807581	0.0031948	39.95	99
CJ008440	turquoise	64.214	39.017311	0.1956954	0.75243254	5.118	turquoise	575.368	551.062	0.8492514	0.0686501	4.69	0
CJ008695	turquoise	1277.9	1276.7971	0.9548638	0.01143297	18.748	turquoise	1041.15	1003.83	0.9976291	0.0001385	31.76	0
CJ010524	turquoise	1012.2	1009.9168	0.9019098	0.03633099	12.592	turquoise	989.739	964.681	0.9869998	0.0017759	11.53	--
CJ011443	turquoise	1489.5	1488.8769	0.9871542	0.00174435	18.65	turquoise	427.635	323.693	0.7365938	0.1557119	30.434	--
CJ012466	turquoise	1539.4	1538.9549	0.996081	0.00029434	3.342	turquoise	569.805	479.602	0.8267719	0.0842639	5.23	0.522
CJ012912	turquoise	1003.7	1000.9762	0.8949061	0.04024691	10.992	turquoise	778.99	749.356	0.9247963	0.0244755	21.358	--
CJ024171	turquoise	1317.4	1316.1596	0.9591912	0.00983532	283.24	turquoise	1034.78	983.422	0.9928463	0.0007255	132.862	--
CJ027205	turquoise	626.18	616.67078	0.774209	0.12433937	31.142	turquoise	723.554	674.23	0.9001544	0.0373004	43.52	99
CJ032354	turquoise	1350.8	1349.7353	0.966925	0.00718484	11.776	turquoise	946.077	857.253	0.9596785	0.0096604	12.69	0

CJ033142	turquoise	1422.8	1422.1764	0.9771778	0.00412457	14.64	turquoise	845.441	779.136	0.9353893	0.0195225	22.156	--
CJ020894	turquoise	1445.4	1444.6927	0.982652	0.00273575	602.086	turquoise	973.159	901.104	0.9722407	0.0055288	381.264	99
DN100878	turquoise	1454.3	1453.8331	0.9839047	0.0024453	2.99	turquoise	954.589	933.11	0.9787055	0.0037183	7.016	99
DN100934	turquoise	1548	1547.5849	0.996272	0.00027309	14.954	turquoise	1014.25	945.671	0.9835552	0.0025252	15.234	--
DN100970	turquoise	687.59	679.41797	0.798318	0.1053754	8.03	turquoise	1032.72	982.766	0.9928786	0.0007206	6.17	--
DN101534	turquoise	1553.6	1553.1829	0.9965278	0.00024548	1.558	turquoise	870.325	757.635	0.9301485	0.0219277	4.016	--
DN101612	turquoise	952.23	951.3044	0.8892822	0.04348234	9.674	turquoise	1012.63	949.858	0.9846586	0.0022758	15.752	99
DN101643	turquoise	1408.6	1408.1304	0.9723009	0.00551087	17.958	turquoise	978.106	933.261	0.9820773	0.0028726	23.3	--
DN102328	turquoise	1522.3	1521.8471	0.9925652	0.00076869	39.258	turquoise	989.336	915.362	0.9762133	0.0043881	25.998	--
DN102330	turquoise	1553.6	1553.1829	0.9965278	0.00024548	0.85	turquoise	1015.27	988.541	0.9929221	0.0007141	2.234	0
DN102924	turquoise	1553.6	1553.1829	0.9965278	0.00024548	1.748	turquoise	1034.95	1003.81	0.9969985	0.0001973	3.154	--
DN103647	turquoise	1526	1525.6204	0.9915673	0.0009284	10.994	turquoise	996.286	952.419	0.984908	0.0022206	20.666	--
DN104450	turquoise	1040.7	1038.8614	0.8977354	0.03864964	20.092	turquoise	858.546	831.103	0.9509489	0.0129445	13.342	--
DN106021	turquoise	1529.6	1529.265	0.9928746	0.00072125	1.62	turquoise	1015.27	988.541	0.9929221	0.0007141	3.79	--
DN106213	turquoise	1554.2	1553.8155	0.9971144	0.000186	10.436	turquoise	1011.6	942.889	0.9832998	0.0025842	13.436	--
DN106221	turquoise	1529	1528.5408	0.9949014	0.00043669	9.66	turquoise	1034.81	1003.78	0.9970411	0.0001931	23.12	--
DN106224	turquoise	643.35	640.30353	0.8002017	0.103934	5.8	turquoise	879.291	856.38	0.9597112	0.0096487	11.316	--
DN106274	turquoise	1546.8	1546.4126	0.9947691	0.0004538	8.014	turquoise	511.579	353.285	0.7539913	0.1409438	16.42	--
DN106323	turquoise	1259.7	1258.6201	0.9460117	0.01493602	3.636	turquoise	1020.1	968.288	0.9896512	0.0012618	7.1	--
DN106451	turquoise	1140	1137.9081	0.9253845	0.0241911	9.872	turquoise	959.849	925.303	0.9777316	0.0039757	10.366	--
DN106928	turquoise	1552	1551.5203	0.9975876	0.00014218	27.456	turquoise	1047.91	999.245	0.9968337	0.0002138	36.45	--
DN107015	turquoise	1523.1	1522.7085	0.9914403	0.00094942	2.528	turquoise	806.968	769.259	0.9345915	0.0198828	3.104	99
DN107136	turquoise	1425.9	1425.0967	0.9782501	0.00383794	13.172	turquoise	898.866	854.772	0.9600265	0.0095361	14.614	--
DN107769	turquoise	1554.2	1553.7791	0.9976348	0.00013803	20.81	turquoise	1038.85	991.402	0.9945691	0.0004801	50.696	--
DN107929	turquoise	1272.8	1271.5635	0.9510085	0.01292103	40.99	turquoise	843.261	787.204	0.9391372	0.017859	35.792	--
DN107937	turquoise	1552.5	1552.0854	0.9965244	0.00024584	24.928	turquoise	1027.94	997.786	0.9954366	0.0003698	27.472	--
DN108486	turquoise	536.58	531.79147	0.759105	0.13668607	46.946	turquoise	1037.69	1006.01	0.9976379	0.0001378	41.374	--
DN108517	turquoise	602.23	598.22134	0.7699125	0.12781607	6.894	turquoise	886.254	864.644	0.9594834	0.0097303	15.512	--
DN108763	turquoise	403.47	398.88086	0.7116997	0.17756855	13.504	turquoise	907.525	817.501	0.9477441	0.0142266	20.612	0
DN109089	turquoise	1554.6	1554.2035	0.9981356	9.66E-05	32.59	turquoise	944.738	920.462	0.9754199	0.004609	44.174	--
DN109161	turquoise	1228.8	1227.3208	0.9446887	0.01548526	18.248	turquoise	243.344	159.687	0.5650165	0.3209579	20.664	--
DN110430	turquoise	1571.7	1571.315	0.9998077	3.20E-06	5.302	turquoise	1017.3	979.727	0.9923672	0.0007996	16.342	--
DN110465	turquoise	1250.3	1248.9437	0.9470805	0.01449703	6.494	turquoise	1033.73	1001.48	0.9964477	0.000254	6.514	--
DN110896	turquoise	92.523	44.490624	0.21269	0.73125052	21.336	turquoise	1021.08	987.054	0.9932511	0.0006649	25.238	--
DN110907	turquoise	1529.2	1528.712	0.9946497	0.00046941	15.036	turquoise	1046.72	1007.43	0.9982633	8.69E-05	18.056	--
DN111558	turquoise	1546	1545.5745	0.9965623	0.00024183	18.534	turquoise	871.934	796.589	0.9413317	0.0169075	42.974	--

DN112204	turquoise	1522.1	1521.7206	0.9906734	0.00107972	11.02	turquoise	882.019	792.535	0.9415665	0.0168067	10.272	--
DN112212	turquoise	1466.2	1465.6521	0.9810204	0.00312986	51.358	turquoise	736.497	665.677	0.90046	0.037131	101.444	--
DN112252	turquoise	902.81	900.75892	0.880393	0.04875505	11.388	turquoise	641.146	583.994	0.8653154	0.0581215	10.956	--
DN112314	turquoise	1489.9	1489.2792	0.9888743	0.00140636	26.722	turquoise	862.235	756.618	0.9291571	0.0223928	22.4	--
DN112323	turquoise	1553.6	1553.1829	0.9965278	0.00024548	1.558	turquoise	948.414	927.812	0.9771766	0.0041249	3.768	--
DN112991	turquoise	1546	1545.5777	0.99707	0.0001903	28.528	turquoise	858.338	759.99	0.9308804	0.0215864	25.18	--
DN112996	turquoise	90.848	65.255491	0.2929267	0.63243874	3.322	turquoise	996.836	967.146	0.9879563	0.0015838	4.44	--
DN113159	turquoise	933.99	931.667	0.8745902	0.05229834	42.98	turquoise	763.159	672.587	0.9032407	0.0356014	37.108	--
DN113688	turquoise	1387.7	1386.8407	0.973127	0.00526683	5.316	turquoise	677.095	628.185	0.8947852	0.0403157	2.108	--
DN114373	turquoise	432.54	413.64358	0.6708819	0.21511185	16.35	turquoise	1034.78	993.356	0.995611	0.0003488	8.624	0.42
DN114544	turquoise	1343.4	1342.3383	0.9623219	0.00872965	19.36	turquoise	964.525	939.432	0.9808335	0.0031761	26.34	--
DN114642	turquoise	1557.6	1557.175	0.9974605	0.00015356	5.958	turquoise	680.754	548.164	0.8557909	0.0642981	10.612	--
DN115031	turquoise	1538	1537.5205	0.9960159	0.00030169	7.944	turquoise	1006.62	953.383	0.9853678	0.00212	12.938	--
DN115327	turquoise	1553.6	1553.1829	0.9965278	0.00024548	0.708	turquoise	1015.27	988.541	0.9929221	0.0007141	2.842	--
DN115687	turquoise	1460.5	1460.0059	0.9800097	0.00338264	8.088	turquoise	523.35	510.514	0.8442925	0.0720088	20.892	--
DN115744	turquoise	1294.6	1293.5713	0.9588391	0.00996236	220.986	turquoise	282.725	189.702	0.6068105	0.2778422	244.998	--
DN115925	turquoise	1553.6	1553.1829	0.9965278	0.00024548	1.322	turquoise	1015.27	988.541	0.9929221	0.0007141	9.542	--
DN116041	turquoise	123.49	106.71025	0.404389	0.49951521	75.588	turquoise	749.697	726.408	0.9171355	0.0282757	61.518	--
DN117853	turquoise	1514.2	1513.7027	0.9917086	0.00090518	15.22	turquoise	1043.44	992.686	0.9950321	0.00042	9.23	--
DN119461	turquoise	1488.1	1487.6905	0.9855861	0.00207283	5.674	turquoise	976.139	939.029	0.9817848	0.002943	9.24	--
DN120179	turquoise	208.79	149.37756	0.387311	0.51948307	41.082	turquoise	616.228	593.811	0.8709179	0.0545808	31.488	--
DN120816	turquoise	1503.3	1502.8406	0.988426	0.00149211	2.25	turquoise	933.904	859.871	0.9606369	0.0093194	7.548	--
DN121344	turquoise	1417.7	1416.8222	0.9782385	0.00384101	10.04	turquoise	869.911	793.013	0.9419276	0.0166521	9.46	--
DN121454	turquoise	134.41	109.70519	0.3972119	0.50788728	21.988	turquoise	948.719	924.34	0.9766256	0.0042748	25.2	--
DN121650	turquoise	1553.6	1553.1829	0.9965278	0.00024548	0.944	turquoise	910.125	889.809	0.9671628	0.0071078	3.39	--
DN121967	turquoise	1532.6	1532.2001	0.993566	0.00061892	2.666	turquoise	1026.32	997.48	0.9953031	0.0003861	5.26	0
DN122834	turquoise	1458.8	1458.0384	0.9837776	0.00247427	26.722	turquoise	880.163	862.167	0.9589136	0.0099354	26.618	99
DN122987	turquoise	1523.2	1522.7981	0.9918907	0.00087554	2.898	turquoise	71.2451	48.7239	0.3714506	0.5381673	2.366	--
DN123452	turquoise	1553.6	1553.1829	0.9965278	0.00024548	9.92	turquoise	1054.44	1012.46	0.999748	4.80E-06	0.998	0
DN123882	turquoise	1553.6	1553.1829	0.9965278	0.00024548	0.33	turquoise	1015.27	988.541	0.9929221	0.0007141	1.76	--
DN124029	turquoise	1555.7	1555.2403	0.9978732	0.0001177	3.596	turquoise	784.023	655.854	0.8945578	0.040445	3.19	--
DN125959	turquoise	1530.1	1529.7569	0.9922472	0.00081849	1.7	turquoise	1009.48	982.64	0.9914626	0.0009457	5.518	--
DN126017	turquoise	1481.9	1481.2504	0.9867362	0.00183008	11.218	turquoise	439.723	336.881	0.7514878	0.1430423	10.506	--
DN126098	turquoise	562.61	555.09203	0.7582491	0.13739598	14.758	turquoise	1047.6	999.957	0.9968363	0.0002135	11.356	--
DN126130	turquoise	1527.6	1527.0431	0.9943492	0.00050948	3.744	turquoise	750.579	652.971	0.8957774	0.0397528	3.606	--
DN126995	turquoise	1093.8	1092.0884	0.9105065	0.03170321	9.378	turquoise	790.574	770.341	0.9359079	0.0192895	10.14	--

DN128230	turquoise	1521.2	1520.8275	0.9906063	0.00109138	20.206	turquoise	385.178	283.395	0.7039819	0.1845097	25.742	--
DN129825	turquoise	1554.1	1553.6451	0.9979797	0.00010898	25.116	turquoise	861.203	800.45	0.9468004	0.0146117	28.896	--
DN133913	turquoise	1516.6	1516.1821	0.9908592	0.00104765	11.824	turquoise	778.487	758.105	0.9269565	0.0234364	7.624	--
DN134319	turquoise	906.25	903.98765	0.8751874	0.05193007	9.786	turquoise	1012.69	979.131	0.9910322	0.0010181	16.304	--
DN134561	turquoise	1483.7	1483.3254	0.9842742	0.00236171	1.796	turquoise	1029.59	997.644	0.9958234	0.0003238	2.27	--
CR939665	turquoise	1315.6	1314.6079	0.9586726	0.01002259	19.336	turquoise	862.184	805.653	0.9457863	0.0150291	24.58	0
CR940070	turquoise	1340.2	1339.5584	0.9601606	0.00948836	2.07	turquoise	451.645	333.17	0.7398835	0.1528865	5.71	--
CR939318	turquoise	1530.4	1529.9932	0.9929289	0.00071302	16.296	turquoise	924.895	889.123	0.96936	0.0064086	23.462	--
BW961291	turquoise	1549	1548.5525	0.9974887	0.00015101	2.928	turquoise	692.417	643.16	0.8922502	0.041765	7.066	--
BW960629	turquoise	1436.4	1435.9337	0.9812561	0.00307185	4.584	turquoise	268.213	181.399	0.5992298	0.2855417	6.868	0
BW973016	turquoise	1512	1511.542	0.9886226	0.0014543	15.856	turquoise	1015.24	950.123	0.9845278	0.0023049	22.07	--
BW967903	turquoise	329.29	305.54649	0.5978187	0.28698111	12.884	turquoise	1050.65	1009.69	0.9989135	4.30E-05	12.976	--
BW979660	turquoise	1436.7	1436.207	0.9773473	0.0040788	4.73	turquoise	192.469	156.297	0.5643318	0.3216774	20.184	--
BW980850	turquoise	1404.7	1404.098	0.9737138	0.00509571	5.916	turquoise	812.906	779.551	0.9358962	0.0192947	18.036	99
BW980990	turquoise	820.88	817.33839	0.8524114	0.06653608	27.818	turquoise	815.234	740.447	0.9266158	0.0235993	30.178	--
BW982750	turquoise	1491.1	1490.5129	0.9887011	0.00143931	6.452	turquoise	788.551	673.8	0.9030011	0.0357324	7.512	--
AJ951006	turquoise	1023.3	1021.2069	0.9007236	0.03698515	29.472	turquoise	922.219	873.597	0.9642116	0.0080835	32.302	--
AJ952587	turquoise	1504.9	1504.444	0.9883684	0.00150326	13.682	turquoise	664.546	594.986	0.8745272	0.0523372	17.204	--
AJ953775	turquoise	1503.3	1502.6652	0.9902526	0.00115354	17.356	turquoise	937.427	917.589	0.9741277	0.0049761	12.136	--
AJ956532	turquoise	1301.6	1300.3145	0.9562573	0.01090994	1.716	turquoise	1015.27	988.541	0.9929221	0.0007141	2.098	--
AJ957377	turquoise	1546.8	1546.3785	0.9958346	0.00032251	4.22	turquoise	985.395	913.059	0.9752718	0.0046506	2.606	--
AJ957942	turquoise	1478.8	1478.3341	0.9839216	0.00244145	6.936	turquoise	499.898	448.602	0.8021979	0.1024131	6.156	--
AJ958416	turquoise	1448.2	1447.7255	0.9796771	0.00346724	6.4	turquoise	981.953	953.383	0.9842154	0.0023749	12.402	--
AJ958520	turquoise	1571.6	1571.1741	0.9998108	3.12E-06	2.016	turquoise	653.316	634.16	0.8937118	0.0409273	3.526	--
AJ958543	turquoise	1098.5	1096.6154	0.9210952	0.02628943	29.068	turquoise	991.838	935.963	0.9804995	0.0032593	33.092	--
AJ959966	turquoise	888.97	884.36412	0.8630826	0.05955182	10.45	turquoise	900.697	855.108	0.9584816	0.0100919	6.714	--
AJ960294	turquoise	1553.6	1553.1829	0.9965278	0.00024548	0.708	turquoise	1015.27	988.541	0.9929221	0.0007141	1.962	--
AJ960623	turquoise	878.02	876.81231	0.8732587	0.05312238	5.896	turquoise	207.015	117.106	0.4989375	0.3921742	15.076	--
AJ960641	turquoise	1239.1	1238.3067	0.9397162	0.01760631	6.12	turquoise	1039.33	1002.13	0.997413	0.0001579	14.22	--
AJ962199	turquoise	1260.6	1259.3289	0.9514861	0.0127335	22.592	turquoise	743.283	632.007	0.8856711	0.0456012	18.786	--
AJ962658	turquoise	1490	1489.3942	0.9893914	0.00130957	7.502	turquoise	455.825	352.066	0.7461256	0.1475673	10.958	--
AJ964270	turquoise	1495.1	1494.6735	0.986069	0.0019697	9.43	turquoise	743.249	595.495	0.871428	0.0542619	12.958	--
AJ964761	turquoise	1566.3	1565.9012	0.9990226	3.67E-05	3.754	turquoise	162.469	110.645	0.518596	0.3706265	10.032	--
AJ964869	turquoise	1545.2	1544.6895	0.9964311	0.00025581	4.524	turquoise	1043.49	994.386	0.9954532	0.0003678	10.446	--
AJ944251	turquoise	202.83	195.97071	0.5458646	0.34123473	95.154	turquoise	535.544	496.753	0.8404449	0.0746488	115.204	--
AJ944277	turquoise	110.47	84.942829	0.3223446	0.59680073	7.47	turquoise	1046.41	998.672	0.9965247	0.0002458	7.662	--

AJ946247	turquoise	339.99	312.36099	0.5975096	0.28729662	32.958	turquoise	277.022	245.023	0.6671998	0.2185963	21.628	--
AJ946071	turquoise	1558.8	1558.461	0.9970919	0.00018817	15.098	turquoise	1020.88	988.66	0.9934944	0.0006293	18.658	--
DV224291	turquoise	1438.9	1438.4916	0.9786082	0.00374374	5.868	turquoise	901.978	876.774	0.9644922	0.007989	8.192	--
DV225307	turquoise	1212.5	1211.3519	0.9345468	0.01990307	82.618	turquoise	590.401	489.145	0.8251214	0.0854487	91.36	--
DV225844	turquoise	925.36	922.61204	0.8824093	0.04754238	74.128	turquoise	1049.88	999.963	0.9971429	0.0001832	76.636	--
DV226023	turquoise	1571	1570.5891	0.9995834	1.02E-05	1.454	turquoise	804.493	774.427	0.9389884	0.0179241	2.198	0.072
DV226542	turquoise	1197.7	1196.5223	0.9313138	0.02138513	31.94	turquoise	1037.39	977.339	0.9915953	0.0009238	39.3	--
DV227158	turquoise	1502.4	1501.8692	0.9891808	0.00134873	8.102	turquoise	270.547	237.29	0.6865559	0.2004562	17.892	--
DV227358	turquoise	550.24	549.28328	0.7722046	0.12595776	7.366	turquoise	1048.92	1011.37	0.9992398	2.52E-05	9.856	--
CV868318	turquoise	1289.1	1288.0367	0.9577568	0.01035615	4.056	turquoise	940.223	911.829	0.9732515	0.0052304	6.14	99
CV873038	turquoise	1451.3	1450.7303	0.9815934	0.00298946	8.624	turquoise	356.501	275.551	0.7072816	0.1815327	8.376	--
CV873720	turquoise	1549.4	1548.9605	0.9968621	0.0002109	1.738	turquoise	580.241	440.699	0.7995166	0.1044575	2.496	--
CX058016	turquoise	1045.1	1043.2111	0.904088	0.03513941	7.08	turquoise	841.897	811.1	0.9503846	0.0131674	10.94	99
CX065124	turquoise	1369	1368.1323	0.9688915	0.00655564	55.188	turquoise	938.139	861.303	0.9617333	0.0089342	59.062	--
DY406486	turquoise	51.3	37.767928	0.2324834	0.70668141	4.084	turquoise	1046.12	997.03	0.9960843	0.000294	5.23	--
DY407195	turquoise	1553.6	1553.1829	0.9965278	0.00024548	4.298	turquoise	1015.27	988.541	0.9929221	0.0007141	28.556	--
DY407390	turquoise	1458.7	1458.2434	0.9813831	0.00304074	1.034	turquoise	1002.06	977.188	0.9899453	0.0012085	3.97	--
DY411095	turquoise	1446.7	1446.1968	0.9779028	0.00393002	0.708	turquoise	1009.6	983.741	0.9917028	0.0009061	2.85	--
DY415087	turquoise	1294.3	1293.3544	0.9586373	0.01003539	4.96	turquoise	551.938	446.818	0.8078522	0.0981422	5.724	--
DY416230	turquoise	1464.5	1463.8416	0.9849866	0.00220329	6.066	turquoise	451.848	365.988	0.7609276	0.1351779	5.462	99
DY417568	turquoise	996.92	994.99611	0.8990785	0.03789859	2.552	turquoise	1052.78	1012.27	0.9996412	8.16E-06	6.196	--
DY418401	turquoise	1456.6	1456.0958	0.9795812	0.00349176	5.36	turquoise	1024.3	985.629	0.99353	0.0006241	10.15	--
DY418469	turquoise	1553.6	1553.1829	0.9965278	0.00024548	1.322	turquoise	1015.27	988.541	0.9929221	0.0007141	7.578	--
DY418643	turquoise	1456.9	1456.1648	0.9832958	0.00258513	2.6	turquoise	1017.71	986.106	0.9926075	0.0007621	7.138	--
DY418677	turquoise	1531	1530.5309	0.9947173	0.00046055	1.808	turquoise	798.22	665.489	0.898266	0.0383523	2.102	--
DY419075	turquoise	1536	1535.4727	0.9956081	0.00034917	1.312	turquoise	1011.49	985.052	0.9919962	0.0008585	3.97	--
DY419287	turquoise	1411.5	1410.7455	0.9756251	0.0045515	2.336	turquoise	1046.74	1007.64	0.9983386	8.13E-05	3.542	0
DY419575	turquoise	1553.6	1553.1829	0.9965278	0.00024548	0.426	turquoise	1009.43	983.5	0.9915942	0.000924	4.392	--
DY420354	turquoise	1553.6	1553.1829	0.9965278	0.00024548	0.188	turquoise	1015.27	988.541	0.9929221	0.0007141	1.894	0
DY422193	turquoise	1508.8	1508.4247	0.9880127	0.00157265	2.324	turquoise	1010.51	983.856	0.99182	0.000887	6.49	--
DY424276	turquoise	1387.4	1386.5304	0.9696636	0.00631385	37.018	turquoise	289.378	245.742	0.678806	0.2076664	26.262	--
DY425355	turquoise	346.98	339.2073	0.6629751	0.22261311	11.182	turquoise	776.29	744.248	0.9264616	0.0236731	10.906	--
DY426016	turquoise	1415.6	1414.8168	0.9759844	0.00445146	1.58	turquoise	1047.38	1010.18	0.9988975	4.39E-05	5.284	--
DY426090	turquoise	903.02	900.40147	0.8763747	0.05120037	25.532	turquoise	578.092	494.911	0.8287015	0.0828854	31.436	--
DY426166	turquoise	1172.4	1171.6481	0.9371956	0.01871476	14.25	turquoise	849.652	802.33	0.946778	0.0146209	16.632	--
DY428656	turquoise	1465.5	1464.7806	0.9857459	0.00203851	28.438	turquoise	725.184	707.525	0.9109739	0.0314575	32.214	--

DY432545		turquoise	1244.3	1243.6201	0.9487412	0.01382354	1.902	turquoise	1019.45	991.76	0.9937797	0.0005884	3.574	--
DY432870		turquoise	1553.6	1553.1829	0.9965278	0.00024548	0.33	turquoise	1015.27	988.541	0.9929221	0.0007141	2.842	--
DY434291		turquoise	1559.7	1559.2327	0.9987209	5.49E-05	4.676	turquoise	976.827	895.826	0.970339	0.0061048	8.168	--
DY434551		turquoise	1539.5	1539.0862	0.9939587	0.00056316	3.392	turquoise	433.503	358.023	0.763529	0.1330339	5.236	--
DY435434		turquoise	1553.6	1553.1829	0.9965278	0.00024548	1.558	turquoise	973.525	889.323	0.9689079	0.0065505	0.738	--
DY437503		turquoise	1174.2	1172.5047	0.9358593	0.01931129	15.906	turquoise	901.091	879.164	0.964877	0.00786	12.476	--
DY437556		turquoise	990.91	990.02992	0.900874	0.036902	0.78	turquoise	1043.87	1009.42	0.9987173	5.51E-05	2.506	--
DY438052		turquoise	1486.6	1486.1697	0.9858437	0.00201759	3.318	turquoise	269.262	240.653	0.6848624	0.2020257	7.482	--
DY438091		turquoise	1056.7	1054.5124	0.9092509	0.0323665	26.372	turquoise	1040.07	1003.62	0.9973679	0.000162	33.014	--
DB789553		turquoise	1547.6	1547.198	0.9952789	0.00038913	1.938	turquoise	910.626	863.013	0.9614602	0.0090297	5.196	--
DB789579		turquoise	1553.6	1553.1829	0.9965278	0.00024548	0.566	turquoise	1015.27	988.541	0.9929221	0.0007141	4.128	--
DB789650		turquoise	230.89	221.3952	0.5498323	0.3370085	11.054	turquoise	1001.43	951.221	0.9848324	0.0022373	15.384	0
DB789990		turquoise	1303.9	1302.754	0.9590759	0.00987686	12.652	turquoise	295.954	247.42	0.6958774	0.1918796	12.358	--
DB786410		turquoise	1487.7	1487.0537	0.9884035	0.00149646	34.92	turquoise	1050.74	999.612	0.9969206	0.000205	50.498	--
DB786768		turquoise	1568.5	1568.1379	0.9990706	3.40E-05	3.056	turquoise	1032.26	970.653	0.9901332	0.0011748	7.04	--
DB787689		turquoise	1178.4	1177.4389	0.9256989	0.02403954	13.034	turquoise	1000.24	945.376	0.9834856	0.0025413	18.954	--
DB788150		turquoise	1405	1404.285	0.9765443	0.00429707	21.632	turquoise	737.007	708.714	0.9150624	0.0293341	17.064	0
DB788467		turquoise	1559.5	1559.1204	0.9972444	0.00017358	6.778	turquoise	820.406	782.345	0.9367508	0.0189126	9.744	0.272
DB794356		turquoise	1389.6	1389.1292	0.9695325	0.00635469	6.288	turquoise	509.126	357.914	0.7500809	0.1442255	11.942	--
DB803904		turquoise	1329.9	1328.8577	0.9589506	0.00992206	3.416	turquoise	160.551	95.7031	0.4694848	0.4249831	3.966	--
DB805450		turquoise	232.19	180.63256	0.438152	0.46053029	36.654	turquoise	1014.16	986.135	0.9925596	0.0007696	27.066	--
DB801635		turquoise	1514.1	1513.7525	0.9897361	0.00124633	14.924	turquoise	1020.41	988.007	0.9937137	0.0005977	14.986	--
AK235649	PLTP	turquoise	429.67	424.20985	0.7156535	0.17404225	2.912	turquoise	1009.01	980.515	0.9911545	0.0009973	5.868	1.213
DT329775		turquoise	1562.6	1562.1992	0.9984439	7.37E-05	3.51	turquoise	1033.48	995.764	0.9956238	0.0003473	4.694	99
DT331358		turquoise	785.4	781.16759	0.83838	0.07607769	21.226	turquoise	1002.93	974.883	0.9895436	0.0012815	25.16	99
ES445611		turquoise	1570.6	1570.2246	0.9994948	1.36E-05	1.548	turquoise	1011.99	982.721	0.9919181	0.0008711	5.358	--
ES447414		turquoise	1435.3	1434.7981	0.9754809	0.00459183	23.64	turquoise	927.995	887.779	0.9694245	0.0063884	31.966	--
ES447504		turquoise	1448.6	1447.8458	0.982674	0.00273054	8.694	turquoise	851.976	739.083	0.9235113	0.0251006	18.796	--
ES447553		turquoise	1506.7	1506.2356	0.9896312	0.00126546	1.352	turquoise	1046.97	1010.37	0.9990092	3.74E-05	3.344	--
EV900222		turquoise	1562.5	1562.0281	0.9991765	2.84E-05	3.082	turquoise	1038.01	1001.56	0.9966576	0.0002318	6.562	--
EV910030		turquoise	1422.6	1421.8366	0.9790461	0.00362963	28.952	turquoise	1011.38	958.298	0.9876465	0.0016452	29.652	0
EV951008		turquoise	1496.4	1495.9457	0.9860854	0.00196622	2.03	turquoise	435.82	297.347	0.7075921	0.1812534	3.072	--
EV974686		turquoise	306.33	301.15386	0.6287013	0.25592544	3.464	turquoise	640.487	628.277	0.8813149	0.0481994	4.926	--
EW054481		turquoise	1426.6	1426.092	0.9763493	0.00435064	13.136	turquoise	477.182	399.676	0.7821138	0.1180182	18.594	--
EW054741		turquoise	1536.1	1535.6724	0.994826	0.00044641	2.666	turquoise	579.925	441.185	0.7991257	0.1047566	4.112	--
EW074147		turquoise	272.65	265.0662	0.5975428	0.28726279	106.744	turquoise	958.811	879.972	0.9664589	0.0073367	99.526	--

EW075751	turquoise	1553.6	1553.1829	0.9965278	0.00024548	0.142	turquoise	1015.27	988.541	0.9929221	0.0007141	1.894	--
EW078900	turquoise	1223.8	1222.5704	0.9461132	0.01489415	3.614	turquoise	736.086	706.734	0.9108962	0.0314983	7.704	--
EW079103	turquoise	182.84	134.82299	0.3725692	0.53684518	108.874	turquoise	1035.13	1001.53	0.9965502	0.0002431	102.004	--
EW079881	turquoise	1553.6	1553.1829	0.9965278	0.00024548	0.378	turquoise	1015.27	988.541	0.9929221	0.0007141	1.894	0.23
EW080318	turquoise	654.01	648.00093	0.7974804	0.10601824	3.788	turquoise	1010.27	979.925	0.991044	0.0010161	7.112	--
EW080828	turquoise	1553.6	1553.1829	0.9965278	0.00024548	0.898	turquoise	1024.21	995.815	0.9948582	0.0004423	4.342	--
EW082850	turquoise	1233.5	1231.9393	0.9433601	0.01604326	2.084	turquoise	1019.6	992.105	0.9938695	0.0005757	9.146	--
EW085361	turquoise	1571	1570.5891	0.9995834	1.02E-05	1.454	turquoise	812.98	798.266	0.9397703	0.0175827	5.39	--
EW085980	turquoise	550.69	543.15759	0.7538168	0.14108977	5.778	turquoise	1029.94	999.751	0.9960829	0.0002941	5.43	--
EW087115	turquoise	1553.6	1553.1829	0.9965278	0.00024548	3.07	turquoise	1015.27	988.541	0.9929221	0.0007141	16.376	--
EW088450	turquoise	1553.6	1553.1829	0.9965278	0.00024548	2.268	turquoise	1015.27	988.541	0.9929221	0.0007141	2.978	99
EW089070	turquoise	1458	1457.3674	0.9842466	0.00236793	12.308	turquoise	1030.23	978.707	0.9918516	0.0008819	18.004	--
EW089197	turquoise	1553.6	1553.1829	0.9965278	0.00024548	0.188	turquoise	1015.27	988.541	0.9929221	0.0007141	1.556	--
EW089920	turquoise	1355.6	1355.0556	0.9618604	0.00888992	0.52	turquoise	1015.27	988.541	0.9929221	0.0007141	4.128	--
EW090244	turquoise	724.11	720.63911	0.8276046	0.08366817	21.07	turquoise	1041.33	987.78	0.9940466	0.0005509	25.332	--
EW090454	turquoise	1553.6	1553.1829	0.9965278	0.00024548	2.786	turquoise	1040.61	1007.93	0.9981356	9.66E-05	2.08	--
EW093429	turquoise	1553.6	1553.1829	0.9965278	0.00024548	0.426	turquoise	1015.27	988.541	0.9929221	0.0007141	2.91	--
EW094407	turquoise	1553.6	1553.1829	0.9965278	0.00024548	0.566	turquoise	999.607	974.678	0.9894189	0.0013045	4.084	--
EW095754	turquoise	1531.6	1531.1827	0.9944401	0.00049724	1.794	turquoise	998.903	974.025	0.9892544	0.001335	3.3	99
EW097961	turquoise	1531.1	1530.6797	0.9939262	0.00056771	92.334	turquoise	767.727	643.544	0.8915675	0.042158	96.438	--
EW098042	turquoise	1568.5	1568.1238	0.9996061	9.38E-06	2.758	turquoise	1053.44	1013.32	0.9998675	1.83E-06	1.82	--
EW099557	turquoise	136.15	111.44921	0.401831	0.50249588	78.814	turquoise	704.233	644.655	0.8923372	0.0417149	50.79	--
EW101413	turquoise	1528.8	1528.4552	0.9927918	0.00073384	3.422	turquoise	615.365	511.878	0.8387032	0.0758535	5.49	--
EW107866	turquoise	1553.6	1553.1829	0.9965278	0.00024548	1.512	turquoise	306.773	254.009	0.6706162	0.2153628	1.348	--
EW109769	turquoise	1393.4	1392.641	0.9724651	0.00546208	9.792	turquoise	892.569	860.267	0.9589172	0.0099341	3.264	--
EW133912	turquoise	631.28	626.71699	0.7804147	0.11936852	10.99	turquoise	901.777	879.595	0.9656225	0.0076119	13.762	--
EW139582	turquoise	1495.2	1494.6647	0.9871464	0.00174595	11.35	turquoise	586.766	496.781	0.833476	0.0795046	10.572	--
EW141069	turquoise	1413.7	1412.8822	0.9756811	0.00453587	5.136	turquoise	1053.73	1013.44	0.9999381	5.84E-07	5.98	--
EW143820	turquoise	1464.8	1464.1983	0.9838495	0.00245786	28.18	turquoise	961.535	933.492	0.9796392	0.0034769	20.554	--
EW145802	turquoise	1546.4	1545.9374	0.9965637	0.00024169	4.612	turquoise	855.967	743.55	0.9258964	0.0239445	6.504	--
EW161383	turquoise	1261	1259.6921	0.9514663	0.01274124	80.588	turquoise	187.671	122.923	0.5180681	0.3712013	94.09	--
EW163138	turquoise	133.59	92.037929	0.3092657	0.61260103	15.052	turquoise	1038.94	1006.88	0.9978678	0.0001182	16.324	--
EW164836	turquoise	1434.6	1434.1644	0.978241	0.00384035	9.78	turquoise	555.822	449.146	0.8094908	0.0969149	6.616	--
EW172766	turquoise	143.56	118.47303	0.398076	0.50687783	0.866	turquoise	964.38	943.103	0.9808194	0.0031796	2.048	--
EW173726	turquoise	998.16	995.50359	0.8950883	0.04014345	29.618	turquoise	935.396	897.679	0.9702362	0.0061365	26.97	--
EW174492	turquoise	1009.9	1007.1426	0.8966816	0.03924217	35.346	turquoise	1013.23	954.319	0.9866006	0.0018582	29.394	--

EW177900	turquoise	765.93	760.90171	0.8304689	0.08162884	4.61	turquoise	997.966	972.329	0.9886978	0.0014399	7.556	--
EW179408	turquoise	1553.6	1553.1829	0.9965278	0.00024548	0.566	turquoise	1015.27	988.541	0.9929221	0.0007141	4.872	--
EW179426	turquoise	437.39	429.76509	0.6980169	0.18992607	6.878	turquoise	967.266	943.629	0.9818334	0.0029313	11.74	--
EW179891	turquoise	1066.5	1064.3501	0.9133352	0.03022525	7.33	turquoise	1015.84	978.272	0.9915371	0.0009334	6.046	--
EW180914	turquoise	1553.6	1553.1829	0.9965278	0.00024548	1.842	turquoise	1015.27	988.541	0.9929221	0.0007141	9.27	--
EW182797	turquoise	1203.1	1201.5887	0.9381078	0.01831107	3.076	turquoise	1047.54	1011.96	0.9992968	2.24E-05	2.496	--
EW186017	turquoise	458.17	455.86623	0.7363747	0.15590057	26.372	turquoise	1051.53	1009.59	0.9989343	4.18E-05	25.186	--
EW186377	turquoise	1390.8	1390.3204	0.970042	0.00619645	42.53	turquoise	969.42	935.661	0.980597	0.003235	34.294	--
EW187819	turquoise	1485.6	1485.1307	0.9848872	0.00222517	1.924	turquoise	809.409	794.488	0.9384223	0.0181726	2.278	--
EW189832	turquoise	1303.7	1302.4504	0.956844	0.01069213	2.366	turquoise	1032.84	996.397	0.9958321	0.0003228	3.336	--
EW189910	turquoise	1486.1	1485.4226	0.9878708	0.00160064	3.306	turquoise	906.081	884.041	0.9658782	0.0075274	6.056	--
EW190138	turquoise	1553.6	1553.1829	0.9965278	0.00024548	0.33	turquoise	1015.27	988.541	0.9929221	0.0007141	2.64	--
EW190356	turquoise	571.78	569.30257	0.7820332	0.11808215	2.84	turquoise	1019.19	990.563	0.9935104	0.000627	7.732	--
EW191736	turquoise	1183.3	1181.6534	0.9347001	0.01983362	49.04	turquoise	493.676	452.476	0.8036325	0.1013243	107.208	--
EW192631	turquoise	420.7	418.30055	0.7039806	0.18451092	3.51	turquoise	1048.71	1008.13	0.9985528	6.61E-05	5.368	--
EW193806	turquoise	1553.6	1553.1829	0.9965278	0.00024548	0.188	turquoise	1015.27	988.541	0.9929221	0.0007141	2.774	99
EW194119	turquoise	1553.6	1553.1829	0.9965278	0.00024548	0.094	turquoise	1015.27	988.541	0.9929221	0.0007141	2.3	--
EW195015	turquoise	1446.1	1445.3729	0.9814218	0.00303128	14.222	turquoise	931.981	848.44	0.9577998	0.0103404	10.376	--
EW197779	turquoise	1062.7	1060.2313	0.9078715	0.03310019	1.898	turquoise	1020.88	993.141	0.9941454	0.0005373	14.234	--
EW198502	turquoise	1553.6	1553.1829	0.9965278	0.00024548	0.142	turquoise	1015.27	988.541	0.9929221	0.0007141	1.76	--
EW198996	turquoise	1198.1	1197.1074	0.9297602	0.02210951	44.884	turquoise	902.824	855.684	0.9621825	0.008778	35.496	--
EW200082	turquoise	887.09	885.88736	0.8749998	0.05204568	0.572	turquoise	1034.47	1003.66	0.9969667	0.0002004	1.906	--
EW201053	turquoise	1494.7	1494.1566	0.9895103	0.00128765	31.522	turquoise	1024.21	991.905	0.9942466	0.0005234	43.906	--
EW201455	turquoise	1553.6	1553.1829	0.9965278	0.00024548	0.756	turquoise	1015.27	988.541	0.9929221	0.0007141	3.248	--
EW202873	turquoise	195.38	139.56078	0.3967708	0.50840272	4.52	turquoise	992.302	951.622	0.9844589	0.0023203	6.104	--
EW205532	turquoise	634.69	629.04924	0.79451	0.10830748	15.658	turquoise	1024.94	993.726	0.9949055	0.0004362	24.14	--
EW238270	turquoise	641.93	636.16175	0.7927233	0.10969161	34.224	turquoise	297.836	174.1	0.5855299	0.2995948	39.99	--
EW249451	turquoise	1538.9	1538.4311	0.9960554	0.00029722	151.586	turquoise	1034.96	985.687	0.9940923	0.0005446	156.742	0
EW251159	turquoise	1510	1509.5894	0.9883922	0.00149865	7.294	turquoise	1026.83	990.289	0.9944282	0.0004988	7.922	--
EW251531	turquoise	1471.1	1470.7081	0.9844133	0.0023305	2.272	turquoise	1013.88	987.119	0.9925436	0.000772	4.85	--
EW251561	turquoise	862.06	857.67655	0.8577866	0.06298778	3.532	turquoise	1034.66	1000.61	0.9965334	0.0002449	4.978	--
EW255075	turquoise	1565.6	1565.178	0.9987197	5.50E-05	7.422	turquoise	801.722	708.744	0.9148743	0.0294307	18.112	--
EW268677	turquoise	1186.6	1184.9712	0.9378285	0.01843441	195.028	turquoise	512.516	403.193	0.7839143	0.1165923	112.91	--
EW272029	turquoise	1082.8	1080.954	0.9130728	0.03036144	34.184	turquoise	733.074	676.856	0.9053588	0.0344501	35.708	--
EW277811	turquoise	970.48	968.23674	0.8929934	0.04133836	24.522	turquoise	921.619	885.103	0.9680042	0.0068372	17.326	0.359
EW299585	turquoise	1508.7	1508.2032	0.9908878	0.00104274	95.958	turquoise	840.426	735.561	0.9240897	0.0248186	67.774	--

EW302543	turquoise	1515.9	1515.469	0.9919744	0.00086203	1.79	turquoise	1053.3	1008.2	0.998927	4.22E-05	8.352	--
EW329662	turquoise	1523.9	1523.4643	0.9930154	0.00069999	4.004	turquoise	1017.7	961.932	0.9877829	0.001618	4.824	--
EW329931	turquoise	98.603	90.85533	0.4188342	0.48275352	30.086	turquoise	1017.71	951.815	0.9851659	0.002164	34.016	--
EW337334	turquoise	457.92	445.03224	0.6971173	0.19074672	1.586	turquoise	995.183	970.947	0.9883856	0.0014999	2.36	--
EW357378	turquoise	439.59	434.27339	0.7257581	0.16512344	0.178	turquoise	1005.91	980.319	0.9907929	0.001059	2.116	--
EW372898	turquoise	1464.6	1464.1223	0.9846393	0.00228006	10.982	turquoise	1013.75	967.888	0.9893073	0.0013251	7.348	--
EW390240	turquoise	1524.8	1524.321	0.9926093	0.00076187	0.996	turquoise	1015.27	988.541	0.9929221	0.0007141	1.556	--
EW395978	turquoise	687.45	686.4169	0.8200384	0.08912881	16.202	turquoise	1040.16	998.538	0.9963813	0.0002612	16.106	--
EW418907	turquoise	144.97	132.36715	0.4454197	0.45222879	42.096	turquoise	973.864	937.883	0.9816232	0.0029822	37.14	--
EW422073	turquoise	1554.6	1554.2423	0.9970789	0.00018944	4.044	turquoise	592.051	537.392	0.8468454	0.0702735	1.256	0.57
EW424869	turquoise	1554.1	1553.7345	0.996738	0.00022354	73.226	turquoise	1050.1	1013.03	0.9996175	8.98E-06	36.796	--
EW430401	turquoise	1561.8	1561.39	0.9978836	0.00011684	4.382	turquoise	1053.71	1013.2	0.9998576	2.04E-06	2.51	0.351
EW431129	turquoise	1534.2	1533.8616	0.9934403	0.00063713	4.15	turquoise	1036.67	1005.21	0.9973628	0.0001625	4.652	--
EW435434	turquoise	198.94	182.46469	0.5044437	0.38610968	52.56	turquoise	946.919	875.042	0.9645008	0.0079861	58.39	--
EW450885	turquoise	1506.4	1506.0298	0.9899776	0.00120264	16.836	turquoise	705.531	618.779	0.8846795	0.0461886	20.192	--
EW479707	turquoise	600.97	597.45205	0.7667073	0.13042826	18.302	turquoise	875.092	855.67	0.9572631	0.0105374	16.448	--
EW484432	turquoise	1553.7	1553.2404	0.9981483	9.56E-05	11.556	turquoise	616.124	600.077	0.8751977	0.0519237	11.888	--
EW495076	turquoise	1352.1	1351.0821	0.9664033	0.00735491	2.116	turquoise	1005.49	979.957	0.9907187	0.0010719	5.604	--
EW498151	turquoise	1553.6	1553.1829	0.9965278	0.00024548	0.426	turquoise	1015.27	988.541	0.9929221	0.0007141	2.368	99
EW524237	turquoise	1555.5	1555.0811	0.9968293	0.00021422	14.592	turquoise	1053.48	1013.53	0.9999022	1.16E-06	6.172	--
EW529657	turquoise	1488.4	1487.8123	0.9889906	0.00138439	0.694	turquoise	1010.32	984.293	0.9917963	0.0008909	3.334	--
EW532021	turquoise	739.35	736.61026	0.8154747	0.09247326	28.142	turquoise	899.416	809.971	0.9460308	0.0149282	29.024	--
EW534339	turquoise	453.58	448.94553	0.7130773	0.17633757	16.924	turquoise	188.767	136.53	0.5584914	0.3278311	40.994	--
EW535325	turquoise	387.65	379.65305	0.6728414	0.21326397	13.286	turquoise	990.064	959.083	0.9861717	0.001948	12.546	--
EW536534	turquoise	1231.9	1230.2988	0.9430962	0.0161549	0.784	turquoise	1034.42	1003.63	0.9969568	0.0002014	2.892	--
EW537255	turquoise	1553.6	1553.1829	0.9965278	0.00024548	1.04	turquoise	975.146	946.678	0.9825281	0.002765	3.18	--
EW537783	turquoise	1545.1	1544.7252	0.9958115	0.0003252	1.492	turquoise	1045.54	1009.46	0.9986609	5.88E-05	5.244	--
EW538044	turquoise	421.04	402.42237	0.6690963	0.2167996	91.56	turquoise	438.673	393.795	0.7732734	0.125094	82.044	--
EW561372	turquoise	1492.4	1491.9386	0.9877082	0.00163289	6.384	turquoise	1034.26	1002.18	0.996661	0.0002315	8.946	--
EW566285	turquoise	296.64	272.1102	0.5815512	0.30370867	12.918	turquoise	1048.2	1008.52	0.9986393	6.02E-05	7.528	--
EW566432	turquoise	1558.5	1558.0665	0.9976351	0.00013801	22.148	turquoise	1030.7	974.569	0.9912836	0.0009756	26.016	--
EW569764	turquoise	267.85	252.15599	0.5771416	0.30828496	21.038	turquoise	1006.93	971.486	0.9892918	0.001328	14.466	--
EW571676	turquoise	91.733	82.110037	0.3824515	0.52519372	1.704	turquoise	966.399	944.573	0.9814917	0.0030142	2.688	--
EW573852	turquoise	1529.9	1529.3913	0.9940927	0.00054455	20.458	turquoise	1028.55	981.257	0.9923316	0.0008052	24.002	--
EW575758	turquoise	1499.4	1498.8321	0.9905119	0.00110786	6.588	turquoise	613.219	580.114	0.8689797	0.0557978	3.112	--
EW576972	turquoise	1447.9	1447.4963	0.9814314	0.00302892	1.002	turquoise	1010.06	984.253	0.9917771	0.000894	5.238	--

EW577030	turquoise	346	334.52857	0.6518793	0.23325841	41.514	turquoise	1039.27	1004.38	0.9973158	0.0001669	43.88	--
EW577185	turquoise	300.45	289.77445	0.6303187	0.25432534	79.13	turquoise	920.231	869.128	0.9646324	0.0079419	73.338	--
EW577489	turquoise	1107.5	1105.98	0.9226762	0.02550946	47.644	turquoise	1031.93	995.519	0.9954167	0.0003722	37.47	--
EW577619	turquoise	1559.4	1558.9919	0.9988547	4.65E-05	8.982	turquoise	539.171	466.34	0.8117376	0.0952397	17.44	--
EW578058	turquoise	1269.7	1268.5601	0.9504962	0.01312322	1.212	turquoise	1018.78	991.441	0.9936789	0.0006027	11.952	0
EW578108	turquoise	1454.1	1453.4318	0.9822857	0.00282269	9.59	turquoise	964.652	917.07	0.9752052	0.0046693	14.822	--
EW578118	turquoise	1490.3	1489.7769	0.9883816	0.00150071	3.476	turquoise	1044.06	1010.1	0.9987808	5.11E-05	6.774	--
EW578417	turquoise	1463.6	1463.0543	0.9822072	0.00284144	3.978	turquoise	993.305	940.654	0.9820009	0.0028909	5.06	--
EW585518	turquoise	1437.3	1436.8616	0.977166	0.00412776	3.634	turquoise	619.214	595.382	0.8731116	0.0532137	6.122	--
EW618947	turquoise	208.7	149.66521	0.3858321	0.52121965	18.882	turquoise	939.286	857.942	0.960704	0.0092957	12.452	--
EW633611	turquoise	580.85	576.58031	0.7623497	0.13400459	0.236	turquoise	1015.27	988.541	0.9929221	0.0007141	1.828	--
EW637050	turquoise	1348.2	1347.3531	0.9647417	0.00790526	67.512	turquoise	830.99	717.025	0.9159879	0.02886	80.422	--
EW638124	turquoise	1212.9	1211.6894	0.938933	0.01794835	24.136	turquoise	996.877	948.941	0.9837075	0.0024903	20.972	--
EW645536	turquoise	1536	1535.4402	0.9956507	0.0003441	5.24	turquoise	866.206	785.536	0.9396337	0.0176422	3.164	--
EW651254	turquoise	669.74	664.51304	0.8045849	0.10060341	18.048	turquoise	1035.26	996.737	0.9956553	0.0003436	25.454	--
EW656756	turquoise	1012.6	1010.5805	0.8919919	0.0419135	29.098	turquoise	1032.61	995.145	0.9954142	0.0003725	26.232	--
EW660426	turquoise	1439.3	1438.5616	0.9799493	0.00339797	12.98	turquoise	900.26	795.734	0.9420732	0.0165899	7.302	--
EW662770	turquoise	1552.9	1552.4897	0.996255	0.00027496	5.112	turquoise	686.636	648.437	0.9022604	0.0361383	9.49	--
EW674892	turquoise	1400.9	1400.4828	0.9731331	0.00526505	36.762	turquoise	980.853	941.118	0.9819756	0.002897	32.176	--
EW677807	turquoise	1340.6	1339.5681	0.9653276	0.00770971	37.134	turquoise	865.716	770.963	0.9361662	0.0191738	33.258	--
EX654498	turquoise	1553.6	1553.1829	0.9965278	0.00024548	0.52	turquoise	1015.27	988.541	0.9929221	0.0007141	1.894	0
EX655280	turquoise	597.95	591.67963	0.777527	0.12167407	32.426	turquoise	1035.54	992.824	0.995116	0.0004094	32.84	--
EH005985	turquoise	720.93	717.9383	0.8156313	0.09235784	17.872	turquoise	339.063	278.336	0.6905135	0.1968019	24.898	--
AW785225	turquoise	1322.5	1321.4221	0.9579951	0.01026901	4.252	turquoise	320.811	216.274	0.6350904	0.2496204	5.548	--
BE232673	turquoise	1439.9	1439.2455	0.9797197	0.00345638	0.938	turquoise	1015.27	988.541	0.9929221	0.0007141	1.76	--
BE234968	turquoise	317.84	293.43533	0.5961223	0.28871394	2.41	turquoise	855.136	831.758	0.9508283	0.012992	3.896	--
BE235175	turquoise	1518.8	1518.3516	0.9922192	0.00082293	4.022	turquoise	1035.32	1004.28	0.997133	0.0001842	3.676	--
BG733282	turquoise	1539	1538.5351	0.9957508	0.00033229	2.802	turquoise	991.941	930.15	0.9795514	0.0034994	2.93	99
BG733214	turquoise	1537.5	1537.0585	0.9943439	0.0005102	3.24	turquoise	1017.09	989.899	0.9933715	0.0006472	4.626	--
BI338650	turquoise	1479.9	1479.2641	0.9868027	0.00181635	10.62	turquoise	935.709	900.339	0.9730666	0.0052845	8.2	99
AW312281	turquoise	1556.5	1556.0978	0.9978001	0.00012382	5.442	turquoise	641.33	620.353	0.879567	0.0492546	8.36	--
BF077739	turquoise	1486.7	1486.1357	0.9887241	0.00143491	5.51	turquoise	953.097	887.766	0.9682913	0.0067457	5.282	0
BF079211	turquoise	1553.6	1553.1829	0.9965278	0.00024548	0.378	turquoise	1021.09	993.274	0.9941673	0.0005343	4.494	--
BF190991	turquoise	1452.2	1451.5957	0.9829846	0.00265757	18.978	turquoise	529.187	512.243	0.8483011	0.0692899	10.64	--
BI341100	turquoise	1494.8	1494.2651	0.9895512	0.00128012	6.682	turquoise	1039.87	981.305	0.9924973	0.0007792	16.344	--
BI347030	turquoise	1553.6	1553.1829	0.9965278	0.00024548	0.472	turquoise	1000.17	975.2	0.9895506	0.0012802	2.074	--

BI360398	turquoise	1553.6	1553.1829	0.9965278	0.00024548	0.52	turquoise	1005.91	980.319	0.9907929	0.001059	2.116	--
BI360586	turquoise	1553.6	1553.1829	0.9965278	0.00024548	0.614	turquoise	1015.27	988.541	0.9929221	0.0007141	4.602	--
BI359639	turquoise	324.2	302.81884	0.6074556	0.27718953	15.898	turquoise	1039.79	999.165	0.9964974	0.0002487	14.848	--
BE241031	turquoise	1363.8	1363.1895	0.9661943	0.00742341	4.446	turquoise	835.078	799.481	0.9415849	0.0167988	7.428	--
BF712298	turquoise	1553.6	1553.1829	0.9965278	0.00024548	0.284	turquoise	1015.27	988.541	0.9929221	0.0007141	3.858	--
BF703663	turquoise	1473.9	1473.3196	0.9850851	0.00218168	7.396	turquoise	448.62	308.491	0.7129721	0.1764315	9.24	--
BI398876	turquoise	1500.2	1499.5241	0.9905043	0.00110918	2.01	turquoise	523.021	368.478	0.7633156	0.1332093	2.298	--
BF702205	turquoise	1407.1	1406.5446	0.9728862	0.00533759	16.772	turquoise	718.402	642.499	0.8969204	0.0391076	23.728	--
BI181520	turquoise	1483.1	1482.6116	0.9860614	0.00197129	1.956	turquoise	1039.51	981.647	0.9926006	0.0007632	5.114	99
BI186233	turquoise	1421	1420.3677	0.9772495	0.00410517	1.906	turquoise	1018.08	987.092	0.9930257	0.0006984	3.306	--
BI186417	turquoise	1152.5	1151.2523	0.9298541	0.02206553	35.704	turquoise	943.7	906.171	0.9729105	0.0053304	23.526	--
BI182177	turquoise	1312	1310.6997	0.9585944	0.01005096	10.206	turquoise	984.549	915.03	0.9754419	0.0046028	19.472	99
BI401500	turquoise	707.35	699.61072	0.8055134	0.09990205	0.548	turquoise	1047.27	1011.83	0.9992564	2.43E-05	6.088	--
BQ605037	turquoise	1256	1254.6513	0.9504492	0.0131418	6.266	turquoise	798.34	765.389	0.9303584	0.0218297	5.692	--
CA779544	turquoise	1386.1	1385.1941	0.9726534	0.00540629	8.006	turquoise	1022.9	978.959	0.9913228	0.000969	15.196	99

Table S4-3 Module eigengene and its evolutionary rates in the common module between LT and LDE (postnatal turquoise vs turquoise).

pig_Genebank	pig_Gene	LDE Module	kTotal	kWithin	eigencorr	eigenpval	meanExpr	LT Module	kTotal	kWithin	eigencorr	eigenpval	meanExpr	Ka/Ks
AK230747.1	DDX47	turquoise	155.67	49.5587	0.409985	0.4930075	16.238	turquoise	656.52	206.5127	0.922039	0.025823	15.87	--
AK234203.1	ALG3	turquoise	184.67	61.4281	0.567188	0.3186793	8.586	turquoise	687.49	176.2335	0.874767	0.052189	8.85	0.1747
AK240472.1	TMEM128	turquoise	449.44	147.738	0.899406	0.0377163	9.184	turquoise	903.17	267.9444	0.978633	0.003737	13.624	0.4455
AK230965.1	7-Mar	turquoise	233.82	90.9022	0.736796	0.1555374	48.586	turquoise	836.36	245.8626	0.94829	0.014005	63.508	0.958
AK237695.1	NOP14	turquoise	240.49	94.3804	0.683648	0.2031532	19.862	turquoise	800.45	248.5563	0.963971	0.008165	19.636	0.3033
AK234971.1	UBLCP1	turquoise	319.23	60.757	-0.10516	0.8663509	15.382	turquoise	472.65	140.0084	0.784258	0.11632	29.092	0
AK235143.1	SPRY1	turquoise	558.19	173.654	0.924788	0.0244795	20.974	turquoise	906.12	267.1722	0.980388	0.003287	20.914	--
AK235559.1	HMGCS1	turquoise	961.35	222.766	0.966183	0.0074271	12.178	turquoise	270.04	90.70309	0.661759	0.223773	13.35	99
AK232198.1	CHMP2A	turquoise	481.3	115.898	0.747602	0.1463172	44.358	turquoise	912.4	269.9884	0.990629	0.001088	46.164	--
AK238708.1	CHKB	turquoise	823.77	195.309	0.936451	0.0190465	14.924	turquoise	937.68	269.1982	0.986133	0.001956	14.814	--
AK235833.1	BECN1	turquoise	350.39	88.8043	0.666388	0.2193667	25.958	turquoise	716.15	196.0669	0.905068	0.034608	33.16	0
AK239232.1	WDR43	turquoise	1014.6	220.032	0.951644	0.0126717	3.422	turquoise	317.17	75.39473	0.578632	0.306736	8.03	0
AK239332.1	DNAJC2	turquoise	199	80.0624	0.673421	0.2127183	12.642	turquoise	619.93	191.2033	0.885837	0.045503	12.496	99
AK230619.1	PSMA2	turquoise	321.01	85.3654	0.634025	0.2506691	114.394	turquoise	435.1	144.7477	0.757608	0.137928	164.238	99
AK236414.1		turquoise	440.68	145.305	0.861773	0.0603959	3.124	turquoise	390.3	117.6271	0.733465	0.158413	0.77	--
AK236435.1	WDR13	turquoise	765.39	195.297	0.944809	0.0154352	28.692	turquoise	836.11	251.265	0.951495	0.01273	33.88	99
AK239497.1	SFRS3	turquoise	348.46	127.344	0.841002	0.0742647	93.74	turquoise	939.04	268.1922	0.985717	0.002045	80.656	99
AK239575.1	ALAS1	turquoise	901.04	207.91	0.944383	0.0156129	43.926	turquoise	568.6	159.0518	0.828777	0.082831	52.36	0
AK236576.1	PDIA4	turquoise	311.61	114.85	0.740524	0.1523385	54.728	turquoise	883.38	259.0503	0.976158	0.004404	58.488	0.0555
AK233711.1	CHRAC1	turquoise	588.65	178.271	0.926751	0.0235346	24.932	turquoise	895.79	265.724	0.97432	0.004921	32.948	0.4391
AK240234.1	CENPB	turquoise	275.11	56.8591	-0.14646	0.8141956	12.592	turquoise	249.12	74.46514	0.592759	0.292158	17.068	0
AK240271.1	MRPS7	turquoise	190.82	71.6527	0.551538	0.3351954	15.072	turquoise	424.09	140.9647	0.802686	0.102042	10.174	99
AK240329.1	HSPA4	turquoise	573.33	149.041	0.871368	0.0542996	89.024	turquoise	621.43	186.2646	0.897353	0.038864	135.398	99
AK233834.1		turquoise	338.71	121.772	0.799351	0.104584	5.984	turquoise	410.91	105.5495	0.708312	0.180606	2.866	--
AK233903.1	MTCH2	turquoise	577.26	140.299	0.833594	0.0794215	50.144	turquoise	779.18	218.7436	0.920961	0.026356	42.264	0
NM_001105302.1	ACLY	turquoise	1012.9	216.017	0.942766	0.0162951	4.48	turquoise	357.47	102.2602	0.675528	0.210738	10.65	0
NM_001114670.1	TYK2	turquoise	286.51	105.263	0.736359	0.1559139	23.504	turquoise	738.07	211.3445	0.929929	0.02203	20.664	0
NM_001123204.1	TNXB	turquoise	354.14	112.669	0.739038	0.1536116	28.176	turquoise	573.23	191.6279	0.842348	0.073339	23.908	0
XM_001927795.1	HSPA5	turquoise	975.1	217.967	0.95281	0.0122184	8.902	turquoise	609.24	141.9283	0.812571	0.094621	16.172	0
XM_001927936.1	PTGES2	turquoise	579.5	178.228	0.941561	0.0168091	35.348	turquoise	872.52	249.2187	0.958869	0.009951	28.282	0
XM_001926719.1	SETX	turquoise	430.95	59.723	0.084581	0.8924366	15.306	turquoise	283.01	89.53385	0.696456	0.191351	19.76	--
XM_001925347.1	HSDL2	turquoise	918.14	210.706	0.952252	0.0124345	100.61	turquoise	841.43	261.4295	0.960156	0.00949	173.606	0.0914
XM_001924679.1	AK3	turquoise	854.6	213.909	0.955485	0.0111988	40.152	turquoise	481.9	136.4363	0.776797	0.122259	54.886	--

XM_001926714.1	KIAA1370	turquoise	947.42	213.678	0.948532	0.0139078	11.468	turquoise	455.61	151.5248	0.774891	0.12379	9.778	0.1049
XM_001927594.1	SYNCRIP	turquoise	124.05	46.2392	0.554797	0.331739	56.57	turquoise	898.61	261.1916	0.97454	0.004858	65.772	--
XM_001927804.1	NDUFB9	turquoise	667.66	191.572	0.938471	0.0181511	186.116	turquoise	805.33	238.8847	0.948993	0.013722	260.366	0
XM_001928441.1	RAD21	turquoise	946.63	208.519	0.93816	0.018288	8.218	turquoise	251.03	54.84132	0.524011	0.364743	18.726	0
XM_001925389.1	EIF3E	turquoise	492.77	59.6107	0.222703	0.7188075	175.628	turquoise	345.91	105.15	0.714741	0.174854	312.33	0
XM_001927740.1	CYR61	turquoise	683.41	137.805	0.804247	0.1008593	34.222	turquoise	896.01	250.7414	0.968781	0.00659	34.93	0
XM_001928544.1	DDX20	turquoise	109.02	31.3034	0.442012	0.4561175	5.21	turquoise	935.63	269.3665	0.986139	0.001955	3.656	0.1079
XM_001925670.1	SARS	turquoise	792.35	204.756	0.964885	0.0078573	109.568	turquoise	305.02	70.20101	0.60927	0.275356	100.412	0.0838
XM_001929116.1	MTDH	turquoise	224.55	91.4636	0.688832	0.1983526	73.85	turquoise	483.21	97.82447	0.722893	0.167639	98.864	99
XM_001929293.1	PAN2	turquoise	221.64	82.8927	0.677987	0.2084329	33.104	turquoise	855.97	250.2532	0.968856	0.006567	27.42	0
XM_001927985.1	PSMC1	turquoise	209.35	81.444	0.596664	0.2881605	43.488	turquoise	676.22	223.6746	0.900047	0.03736	35.632	0
XM_001928723.1	THTPA	turquoise	456.51	60.3642	0.150325	0.809324	8.966	turquoise	407.84	130.382	0.761271	0.134894	8.2	99
XM_001928863.1	MESDC2	turquoise	991.77	206.097	0.927385	0.0232318	17.856	turquoise	735.82	239.7511	0.923612	0.025051	13.1	0
XM_001929558.1	CDKN1A	turquoise	947.47	221.843	0.967485	0.0070037	117.254	turquoise	555.5	155.7062	0.819394	0.089599	151.278	0.6194
XM_001928226.1	MTUS2	turquoise	1010.9	218.181	0.949981	0.0133275	9.51	turquoise	859.58	259.4316	0.961822	0.008903	2.08	0
XM_001925192.1	TM9SF3	turquoise	1001.3	211.528	0.934915	0.0197363	37.77	turquoise	415.34	100.9028	0.691268	0.196108	66.71	99
XM_001928393.1	GBF1	turquoise	176.6	52.8011	0.450719	0.446197	51.124	turquoise	524.57	173.6621	0.85884	0.062299	48.172	0
XM_001928522.1	CCAR1	turquoise	615.07	166.621	0.877884	0.0502775	28.306	turquoise	343.61	83.94843	0.624236	0.260356	36.238	99
XM_001927977.1	ATAD1	turquoise	1013.5	201.897	0.915951	0.0288789	33.294	turquoise	813.13	254.3394	0.954169	0.011697	33.974	0
XM_001925080.1	PPA1	turquoise	367.37	119.167	0.832565	0.0801461	126.154	turquoise	388.17	106.8504	0.704715	0.183847	143.644	0
XM_001929420.1	ARPC3	turquoise	464.91	139.74	0.81859	0.090186	20.542	turquoise	783.79	241.4019	0.960376	0.009412	14.136	0
XM_001929554.1	ACAD10	turquoise	830.99	211.194	0.966658	0.0072717	9.804	turquoise	802.05	247.9118	0.947847	0.014185	13.284	0
XM_001927490.1	EIF4ENIF1	turquoise	745.37	189.682	0.915638	0.0290389	7.862	turquoise	645.18	162.3767	0.847142	0.070073	10.028	0
XM_001927890.1	TLK1	turquoise	974.09	225.798	0.971459	0.0057633	9.248	turquoise	857.47	261.7199	0.975835	0.004493	11.026	0
XM_001928282.1	NXT1	turquoise	103.18	27.1654	0.397724	0.5072895	9.698	turquoise	287.52	95.77145	0.680425	0.206154	9.332	--
NM_214313.1	TXN	turquoise	423.17	145.167	0.86939	0.0555393	11.838	turquoise	892.06	269.3166	0.984846	0.002234	15.852	--
NM_214366.1	PPP2CA	turquoise	405.75	122.401	0.773052	0.1252732	75.766	turquoise	747.64	231.0484	0.92425	0.024741	68.288	99
NM_001004031.1	DSTN	turquoise	761.24	194.821	0.936418	0.0190614	14.142	turquoise	894.51	259.7755	0.970251	0.006132	18.472	99
AY609399.1	TMED7	turquoise	901.19	212.918	0.95689	0.010675	18.658	turquoise	261.07	76.14398	0.603127	0.281577	27.512	99
AY609974.1	FAM136A	turquoise	952.23	201.296	0.923115	0.0252941	20.48	turquoise	332.32	93.231	0.642966	0.241908	29.728	99
AY610044.1	MRPL35	turquoise	831.25	219.039	0.985161	0.002165	12.016	turquoise	873.36	264.4251	0.977083	0.00415	11.634	99
AY610394.1	NDUFS4	turquoise	357.19	87.9317	0.669838	0.2160983	140.36	turquoise	406.81	137.591	0.786651	0.114435	153.256	99
NM_001032379.1	MOBKL3	turquoise	884.66	204.636	0.934325	0.0200035	34.302	turquoise	896.85	255.6207	0.970243	0.006134	49.376	--
CF787035		turquoise	767.73	203.106	0.945091	0.0153175	13.918	turquoise	488.32	142.8108	0.814999	0.092824	0.542	--
BX668358		turquoise	903.27	220.914	0.971078	0.0058786	6.13	turquoise	219.14	55.46054	0.506428	0.38393	8.346	--
BX671573		turquoise	837.78	208.453	0.961252	0.0091026	15.058	turquoise	895.27	268.5184	0.981749	0.002952	13.14	--

BX672533	turquoise	1018.4	211.628	0.935379	0.0195273	21.412	turquoise	824.95	239.1237	0.946015	0.014935	23.752	--
BP157493	turquoise	183.26	74.4391	0.655374	0.2298913	41.746	turquoise	911.8	269.3984	0.981831	0.002932	41.806	99
CK450204	turquoise	946.51	218.673	0.954331	0.0116351	9.514	turquoise	896.13	266.9646	0.976668	0.004263	21.746	--
CK456864	turquoise	939.47	222.43	0.971485	0.0057553	4.276	turquoise	880.27	266.2789	0.974211	0.004952	4.634	--
CK461574	turquoise	516.33	164.157	0.898965	0.0379619	28.418	turquoise	141.14	36.62453	0.462705	0.432621	21.414	--
CK463357	turquoise	241.23	52.802	0.516411	0.3730068	88.486	turquoise	461.22	125.9089	0.750051	0.14425	108.706	--
BX920663	turquoise	255.97	102.206	0.723994	0.1666709	111.7	turquoise	825	257.2863	0.959166	0.009844	132.252	--
BX920963	turquoise	612.16	153.852	0.839214	0.0754993	25.242	turquoise	586.16	188.9481	0.859988	0.061552	21.55	99
CN158394	turquoise	704.72	173.949	0.905021	0.034633	10.67	turquoise	882.94	266.5531	0.978866	0.003677	19.516	--
CN158718	turquoise	177.48	43.6328	-0.33255	0.5845248	20.984	turquoise	635.9	163.9899	0.853326	0.065928	36.402	--
CN162178	turquoise	507.02	164.415	0.910593	0.0316577	18.264	turquoise	650.69	207.3475	0.920493	0.026589	15.312	--
CJ013877	turquoise	362.9	55.1492	0.031399	0.960028	51.278	turquoise	307.25	102.5066	0.658928	0.22648	43.496	--
DN106431	turquoise	982.9	217.308	0.948732	0.0138272	9.52	turquoise	880.97	249.652	0.961509	0.009013	13.812	--
DN111105	turquoise	187.7	67.4705	0.611662	0.2729441	133.324	turquoise	515.68	167.2715	0.834617	0.078703	92.826	--
DN116574	turquoise	637.84	164.927	0.881368	0.0481672	69.196	turquoise	811.69	254.4919	0.950359	0.013178	100.722	--
DV896278	turquoise	126.91	42.9393	0.517943	0.3713375	9.376	turquoise	831.9	258.8999	0.955723	0.011109	8.582	--
CX058367	turquoise	288.73	59.2928	-0.154	0.8047013	6.834	turquoise	412.61	89.51171	0.703541	0.184908	9.934	--
CX062690	turquoise	841.61	217.999	0.973528	0.0051496	50.454	turquoise	472.56	157.7407	0.814865	0.092923	54.938	--
DT327101	turquoise	261.21	57.0846	-0.22362	0.7176676	8.336	turquoise	606.42	161.2895	0.837129	0.076948	15.332	--
EV877159	turquoise	993.29	218.678	0.952368	0.0123896	23.764	turquoise	975.5	269.3662	0.999052	3.50E-05	31.762	99
EV987119	turquoise	190.7	70.8802	0.675028	0.2112075	9.686	turquoise	694.48	228.5037	0.902358	0.036085	9.482	--
EW115068	turquoise	652.49	176.079	0.91477	0.0294842	467.066	turquoise	703.73	230.9099	0.908138	0.032958	477.274	0
EW129557	turquoise	863.03	218.287	0.968869	0.0065628	27.338	turquoise	823.75	259.9122	0.957613	0.010409	20.04	--
EW189192	turquoise	892.82	224.555	0.982596	0.0027489	9.25	turquoise	519.43	175.3641	0.828255	0.083204	8.262	--
EW225826	turquoise	223.31	61.871	0.52192	0.3670119	26.222	turquoise	301.75	99.66471	0.67864	0.207822	36.45	--
EW329931	turquoise	247.21	93.8243	0.689396	0.1978323	10.782	turquoise	562.11	170.2719	0.842422	0.073289	4.834	--
EW582873	turquoise	210.18	48.4952	-0.30245	0.6208603	6.088	turquoise	330.85	68.76115	0.61729	0.26729	11.618	--
EW590143	turquoise	218.05	81.1919	0.667192	0.2186037	340.864	turquoise	765.49	243.1828	0.941761	0.016724	318.036	99
EW610844	turquoise	842.52	184.268	0.889791	0.0431865	5.16	turquoise	505.72	129.5425	0.75485	0.140226	7.988	--
EW656756	turquoise	809.58	213.473	0.973363	0.0051977	12.518	turquoise	862.81	261.3067	0.963849	0.008206	18.18	--
EW659558	turquoise	901.47	220.562	0.970079	0.006185	8.486	turquoise	448.6	150.1521	0.83201	0.080538	7.952	--
EW671387	turquoise	399.82	138.376	0.858713	0.0623821	4.5	turquoise	597.53	172.122	0.859544	0.061841	7.172	99

Table S5-1. GO annotation analysis of the module eigengene in the common module between LT and LDE (prenatal_brown vs blue).

GO ID	GO Name	Total Count	Diff Count	P Value	FDR	Gene
GO:0007220	Notch receptor processing	1	1	0.000843036	0.014612621	[APH1A]
GO:0031293	membrane protein intracellular domain proteolysis	1	1	0.000843036	0.014612621	[APH1A]
GO:0043148	mitotic spindle stabilization	1	1	0.000843036	0.014612621	[SBDS]
GO:0010611	regulation of cardiac muscle hypertrophy	1	1	0.000843036	0.014612621	[LMCD1]
GO:0070072	vacuolar proton-transporting V-type ATPase complex assembly	1	1	0.000843036	0.014612621	[VMA21]
GO:0032464	positive regulation of protein homooligomerization	2	1	0.00168538	0.010954971	[GNB2L1]
GO:0070886	positive regulation of calcineurin-NFAT signaling cascade	2	1	0.00168538	0.010954971	[LMCD1]
GO:0051302	regulation of cell division	2	1	0.00168538	0.010954971	[GNB2L1]
GO:0050765	negative regulation of phagocytosis	2	1	0.00168538	0.010954971	[GNB2L1]
GO:0042987	amyloid precursor protein catabolic process	2	1	0.00168538	0.010954971	[APH1A]
GO:0006364	rRNA processing	80	2	0.00209657	0.009911056	[SBDS, UTP18]
GO:0048539	bone marrow development	3	1	0.002527034	0.010108134	[SBDS]
GO:0042256	mature ribosome assembly	3	1	0.002527034	0.010108134	[SBDS]
GO:0031340	positive regulation of vesicle fusion	3	1	0.002527034	0.010108134	[ANXA1]
GO:0071569	protein ufmylation	4	1	0.003367996	0.010945989	[UFM1]
GO:0009303	rRNA transcription	4	1	0.003367996	0.010945989	[RNASEK]
GO:0016071	mRNA metabolic process	4	1	0.003367996	0.010945989	[PCBP1]
GO:0006950	response to stress	104	2	0.003508686	0.010136203	[HSPA4, HSPB11]
GO:0030595	leukocyte chemotaxis	5	1	0.00420827	0.011222052	[SBDS]
GO:0043547	positive regulation of GTPase activity	5	1	0.00420827	0.011222052	[GNB2L1]
GO:2000543	positive regulation of gastrulation	6	1	0.005047853	0.012499446	[GNB2L1]
GO:0032880	regulation of protein localization	7	1	0.005886748	0.013604929	[GNB2L1]
GO:2000114	regulation of establishment of cell polarity	7	1	0.005886748	0.013604929	[GNB2L1]
GO:0045332	phospholipid translocation	10	1	0.008399306	0.018198497	[ATP11A]
GO:0018149	peptide cross-linking	11	1	0.009235452	0.019209739	[ANXA1]
GO:0032436	positive regulation of proteasomal ubiquitin-dependent protein catabolic proces	12	1	0.010070911	0.020141822	[GNB2L1]
GO:0030282	bone mineralization	13	1	0.010905685	0.021003542	[SBDS]
GO:0017148	negative regulation of translation	14	1	0.011739774	0.021802438	[GNB2L1]
GO:2001244	positive regulation of intrinsic apoptotic signaling pathway	15	1	0.012573179	0.022162891	[GNB2L1]
GO:0048511	rhythmic process	15	1	0.012573179	0.022162891	[GNB2L1]
GO:0006164	purine nucleotide biosynthetic process	16	1	0.013405899	0.022487315	[ATIC]
GO:0030178	negative regulation of Wnt receptor signaling pathway	17	1	0.014237937	0.023136648	[GNB2L1]

GO:0001934	positive regulation of protein phosphorylation	21	1	0.017559265	0.027669145	[GNB2L1]
GO:0051726	regulation of cell cycle	22	1	0.018387894	0.028122662	[GNB2L1]
GO:0006464	protein modification process	24	1	0.020043113	0.02977834	[RABGGTB]
GO:0030216	keratinocyte differentiation	29	1	0.024169289	0.034911195	[ANXA1]
GO:0044419	interspecies interaction between organisms	315	2	0.028992994	0.04074691	[GNB2L1, YWHAE]
GO:0006986	response to unfolded protein	35	1	0.029098389	0.039818848	[HSPA4]
GO:0051781	positive regulation of cell division	41	1	0.034003261	0.045337682	[TGFB3]
GO:0030335	positive regulation of cell migration	46	1	0.038072231	0.0494939	[GNB2L1]
GO:0015031	protein transport	383	2	0.041389456	0.052493944	[SFT2D1, TRAM1]
GO:0040008	regulation of growth	51	1	0.042124523	0.052154171	[GNB2L1]
GO:0030308	negative regulation of cell growth	58	1	0.047769838	0.057768177	[GNB2L1]
GO:0043065	positive regulation of apoptotic process	83	1	0.067668638	0.079972027	[GNB2L1]
GO:0006916	anti-apoptosis	84	1	0.068456116	0.079104845	[ANXA1]
GO:0008283	cell proliferation	90	1	0.073167412	0.082710987	[SBDS]
GO:0006412	translation	130	1	0.103988828	0.115051469	[RPSA]
GO:0007165	signal transduction	141	1	0.112288379	0.121645743	[ANXA1]
GO:0006355	regulation of transcription, DNA-dependent	1254	2	0.285547206	0.303029688	[ELOF1, LMCD1]
GO:0006508	proteolysis	476	1	0.332117804	0.345402516	[TMPRSS11F]
GO:0008150	biological_process	588	1	0.393021433	0.400727736	[PDGFRL]
GO:0007275	multicellular organismal development	822	1	0.503333661	0.503333661	[GNB2L1]

Table S5-2. GO annotation analysis of the module eigengene in the common module between LT and LDE (prenatal_turquoise vs turquoise).

GO ID	GO Name	Total Count	Diff Count	P Value	FDR	Gene
GO:0044419	interspecies interaction between organisms	315	15	5.63104E-07	0.000402	[AMBP, ATP6V1H, BNIP3L, COPB1, FBXL2, HCFC1, HMGA1, MAGI3, MAPK3, SLC25A5, SRC, TBK1, TSPAN7, USP7, WWP2]
GO:0008219	cell death	136	10	9.37612E-07	0.000335	[ANO10, APP, ATL1, CLN5, DCTN1, HEXB, KIAA0196, PPP2R2B, SNCA, UCHL1]
GO:0043066	negative regulation of apoptotic process	118	9	2.42613E-06	0.000577	[BMP4, BNIP3L, FAM129B, GATA6, LEF1, PTK2, SFRP1, THBS1, WT1]
GO:0045892	negative regulation of transcription, DNA-dependent	284	13	4.8956E-06	0.000874	[AES, BASP1, BMP4, CBX3, DKK3, GATA6, HEY2, HMGA1, LEF1, RING1, SFRP1, TWIST2, WT1]
GO:0045595	regulation of cell differentiation	16	4	1.43891E-05	0.002055	[FADS1, PPP2CA, PPP2R1A, TCFL5]
GO:0030308	negative regulation of cell growth	58	6	2.10525E-05	0.002505	[BTG1, HYAL2, PPP2CA, PPP2R1A, SFRP1, WT1]
GO:0006355	regulation of transcription, DNA-dependent	1254	29	2.18593E-05	0.00223	[AFF3, ASXL1, BCL11A, BTG1, CCAR1, CDK8, CDK9, CHD3, DDX1, FADS1, HABP4, HCFC1, HDAC2, HOXB7, HOXC4, MEAF6, MORF4L2, MXI1, NCOA2, PIAS3, PPP2CA, PPP2R1A, TCEAL2, TCFL5, WT1, ZNF34, ZNF516, ZNF618, ZNF644]
GO:0015031	protein transport	383	14	2.69312E-05	0.002404	[ARF3, LSG1, NECAP2, NUP50, NUP93, RAB18, RAB1A, RAB3A, RAB5C, SCAMP4, SEC22B, SEC61A1, STXBP1, VPS4B]
GO:0008285	negative regulation of cell proliferation	102	7	6.36647E-05	0.005051	[BTG1, HMGA1, SFRP1, SOX4, TES, TMEM127, WT1]
GO:0007275	multicellular organismal development	822	21	6.4994E-05	0.004641	[CSRP2, DDX1, DKK3, DZIP1, EPHA4, FZD5, GAP43, HOXB7, HOXC4, HOXD3, ID2, INA, LECT1, NNAT, OLFM1, PDGFRB, RAB18, TCFL5, TRIM54, TWIST2, USP7]
GO:0016568	chromatin modification	221	10	6.64458E-05	0.004313	[ASXL1, CHD3, DEK, DNMT1, HDAC2, HIRA, MEAF6, MORF4L2, RING1, UIMC1]
GO:0007155	cell adhesion	420	14	7.25562E-05	0.004317	[AMBP, APP, CADM3, CNTN1, COL8A1, EPHA4, FLRT2, ITGB5, NEO1, NLGN3, PTPRF, SRC, TNC, TRO]
GO:0006464	protein modification process	24	4	7.90118E-05	0.00434	[CPE, MAN2B1, PLOD1, WDYHV1]
GO:0007356	thorax and anterior abdomen determination	2	2	9.35521E-05	0.004175	[BASP1, WT1]

GO:2000060	positive regulation of protein ubiquitination involved in ubiquitin-dependent protein catabolic process	2	2	9.35521E-05	0.004175	[CLU, PTK2]
GO:0060421	positive regulation of heart growth	2	2	9.35521E-05	0.004175	[BASP1, WT1]
GO:0010994	free ubiquitin chain polymerization	2	2	9.35521E-05	0.004175	[UBE2C, UBE2S]
GO:2001076	positive regulation of metanephric ureteric bud development	2	2	9.35521E-05	0.004175	[BASP1, WT1]
GO:0048661	positive regulation of smooth muscle cell proliferation	10	3	0.000102865	0.003766	[BMP4, ID2, PDGFRB]
GO:0006378	mRNA polyadenylation	10	3	0.000102865	0.003766	[APP, NUDT21, PABPC1]
GO:0051216	cartilage development	26	4	0.000109475	0.003722	[HOXD3, HYAL2, LECT1, SULF2]
GO:0010629	negative regulation of gene expression	27	4	0.000127534	0.004139	[HEY2, ID2, SFRP1, WWP2]
GO:0043065	positive regulation of apoptotic process	83	6	0.000160203	0.004973	[BMP4, CLU, MLLT11, MTCH1, SFRP1, SOX4]
GO:0016925	protein sumoylation	12	3	0.000185884	0.005417	[BCL11A, PIAS3, SUMO2]
GO:0032436	positive regulation of proteasomal ubiquitin-dependent protein catabolic process	12	3	0.000185884	0.005417	[CLU, CSNK1E, SUMO2]
GO:0007219	Notch signaling pathway	30	4	0.000194633	0.005345	[APP, CNTN1, HEY2, HOXD3]
GO:0072112	glomerular visceral epithelial cell differentiation	3	2	0.000278853	0.007111	[BASP1, WT1]
GO:0042518	negative regulation of tyrosine phosphorylation of Stat3 protein	3	2	0.000278853	0.007111	[PPP2CA, PPP2R1A]
GO:0019932	second-messenger-mediated signaling	3	2	0.000278853	0.007111	[PPP2CA, PPP2R1A]
GO:0001701	in utero embryonic development	14	3	0.000303154	0.007215	[EGFL8, FUT8, PTPRR]
GO:0045893	positive regulation of transcription, DNA-dependent	334	11	0.000466833	0.010752	[BMP4, FOXF2, HMGA1, ID2, LEF1, MLLT11, RNF10, SFRP1, SOX4, WT1, YWHAH]
GO:0016477	cell migration	67	5	0.000490579	0.010946	[BTG1, CD24, CDC42BPA, FMNL3, PDGFRB]
GO:0016192	vesicle-mediated transport	104	6	0.0005433	0.011755	[ARF3, COPB1, ERGIC1, RAB1A, SEC22B, TRAPPC3]
GO:0045730	respiratory burst	4	2	0.000554125	0.010551	[CD24, CD55]
GO:0060539	diaphragm development	4	2	0.000554125	0.010551	[BASP1, WT1]
GO:0008406	gonad development	4	2	0.000554125	0.010551	[BASP1, WT1]
GO:0051491	positive regulation of filopodium assembly	4	2	0.000554125	0.010551	[DPYSL3, PALM]
GO:0032410	negative regulation of transporter activity	4	2	0.000554125	0.010551	[SNCA, WWP2]
GO:0060231	mesenchymal to epithelial transition	4	2	0.000554125	0.010551	[BASP1, WT1]
GO:0031145	anaphase-promoting complex-dependent proteasomal ubiquitin-dependent protein catabolic process	4	2	0.000554125	0.010551	[UBE2C, UBE2S]
GO:0010458	exit from mitosis	4	2	0.000554125	0.010551	[UBE2C, UBE2S]

GO:0007049	cell cycle	405	12	0.00066839	0.011363	[BECN1, CCAR1, GAS2, MAPK3, MARVELD1, NAE1, RALB, SEPT11, SRC, UBE2C, UBE2S, VPS4B]
GO:0008380	RNA splicing	150	7	0.000682584	0.011334	[PABPC1, PPIL1, PPP2CA, PPP2R1A, SNRPG, TRA2A, WT1]
GO:0051291	protein heterooligomerization	5	2	0.000917616	0.014243	[SCUBE3, SEPT11]
GO:0008629	induction of apoptosis by intracellular signals	5	2	0.000917616	0.014243	[CD24, CLU]
GO:0051488	activation of anaphase-promoting complex activity	5	2	0.000917616	0.014243	[UBE2C, UBE2S]
GO:0071345	cellular response to cytokine stimulus	5	2	0.000917616	0.014243	[CDK9, DPYSL3]
GO:0045807	positive regulation of endocytosis	5	2	0.000917616	0.014243	[CALY, SNCA]
GO:0006917	induction of apoptosis	79	5	0.001041465	0.015176	[BNIP3L, PPP2CA, PPP2R1A, THBS1, WT1]
GO:0006958	complement activation, classical pathway	22	3	0.001210898	0.017292	[CD55, CLU, SERPING1]
GO:0006897	endocytosis	83	5	0.001300105	0.018201	[APP, ATP6V1H, NECAP2, RAB18, SH3GL3]
GO:0010628	positive regulation of gene expression	49	4	0.001301956	0.017877	[DNMT1, HCFC1, HOXD3, ID2]
GO:0030501	positive regulation of bone mineralization	6	2	0.001367601	0.017917	[BMP4, PTH]
GO:0030325	adrenal gland development	6	2	0.001367601	0.017917	[DKK3, WT1]
GO:0009267	cellular response to starvation	6	2	0.001367601	0.017917	[FADS1, SFRP1]
GO:0072075	metanephric mesenchyme development	6	2	0.001367601	0.017917	[BASP1, WT1]
GO:0040008	regulation of growth	51	4	0.001512466	0.018946	[GAP43, PPP2CA, PPP2R1A, SHC1]
GO:0043154	negative regulation of cysteine-type endopeptidase activity involved in apoptotic process	25	3	0.001770003	0.021789	[LEF1, SNCA, THBS1]
GO:0045453	bone resorption	7	2	0.001902378	0.021733	[PTH, SRC]
GO:0046718	entry of virus into host cell	7	2	0.001902378	0.021733	[HYAL2, WWP2]
GO:0000188	inactivation of MAPK activity	7	2	0.001902378	0.021733	[PPP2CA, PPP2R1A]
GO:0007017	microtubule-based process	7	2	0.001902378	0.021733	[KIFAP3, TRIM54]
GO:0006275	regulation of DNA replication	7	2	0.001902378	0.021733	[PPP2CA, PPP2R1A]
GO:0002244	hemopoietic progenitor cell differentiation	7	2	0.001902378	0.021733	[BMP4, SFRP1]
GO:0016358	dendrite development	7	2	0.001902378	0.021733	[APP, HDAC2]
GO:0030111	regulation of Wnt receptor signaling pathway	7	2	0.001902378	0.021733	[PPP2CA, PPP2R1A]
GO:0090090	negative regulation of canonical Wnt receptor signaling pathway	27	3	0.002218976	0.023647	[AES, LEF1, SFRP1]
GO:0015992	proton transport	28	3	0.002467534	0.025909	[ATP6AP1, ATP6V1B2, ATP6V1H]
GO:0032836	glomerular basement membrane development	8	2	0.00252027	0.026079	[SULF2, WT1]
GO:0006417	regulation of translation	59	4	0.002592476	0.026443	[APP, GCN1L1, PA2G4, TNRC6A]
GO:0000398	nuclear mRNA splicing, via spliceosome	98	5	0.002703039	0.027183	[PABPC1, PPIL1, SNRPG, TRA2A, U2AF1]

GO:0008284	positive regulation of cell proliferation	102	5	0.00321297	0.031862	[LEF1, PDGFRB, PTK2, SFRP1, SHC1]
GO:0007281	germ cell development	9	2	0.003219626	0.030857	[DZIP1, WT1]
GO:0010977	negative regulation of neuron projection development	9	2	0.003219626	0.030857	[DPYSL3, ITM2C]
GO:0006672	ceramide metabolic process	9	2	0.003219626	0.030857	[PPP2CA, PPP2R1A]
GO:0051493	regulation of cytoskeleton organization	9	2	0.003219626	0.030857	[MAPK3, PTK2]
GO:0018108	peptidyl-tyrosine phosphorylation	32	3	0.003630669	0.033666	[EPHA4, PDGFRB, PTK2]
GO:0055114	oxidation-reduction process	65	4	0.003685466	0.033736	[LOXL1, PGD, PLOD1, SNCA]
GO:0006461	protein complex assembly	33	3	0.003965333	0.035839	[HMGA1, MKI67IP, PPP2R1A]
GO:0071158	positive regulation of cell cycle arrest	10	2	0.003998816	0.035249	[GATA6, ID2]
GO:0043407	negative regulation of MAP kinase activity	10	2	0.003998816	0.035249	[BMP4, HYAL2]
GO:0030010	establishment of cell polarity	10	2	0.003998816	0.035249	[PRKCZ, PTK2]
GO:0008360	regulation of cell shape	67	4	0.004109789	0.035354	[CDC42EP4, FMNL3, GAS2, PTK2]
GO:0070936	protein K48-linked ubiquitination	34	3	0.004318159	0.036704	[MARCH6, UBE2C, UBE2R2]
GO:0006915	apoptotic process	321	9	0.004372371	0.036728	[CCAR1, CLPTM1L, FAM82A2, MAGI3, MAPK3, NISCH, PNMA1, PPP2R2B, RALB]
GO:0060548	negative regulation of cell death	11	2	0.004856237	0.040085	[BMP4, SOX4]
GO:0030177	positive regulation of Wnt receptor signaling pathway	11	2	0.004856237	0.040085	[SFRP1, SULF2]
GO:0009615	response to virus	114	5	0.005155864	0.041833	[BECN1, CLU, HMGA1, HYAL2, TBK1]
GO:0006470	protein dephosphorylation	37	3	0.005488364	0.04403	[PPM1M, PPP2CA, PPP2R1A]
GO:0006810	transport	395	10	0.005560954	0.044117	[APOO, CYBA, DCTN1, FADS1, FEZ1, HERC1, MTCH1, PLTP, SLC25A43, SLC37A4]
GO:0043392	negative regulation of DNA binding	12	2	0.005790307	0.044938	[HABP4, LEF1]
GO:0010033	response to organic substance	12	2	0.005790307	0.044938	[PPP2CA, PPP2R1A]
GO:0000245	spliceosome assembly	12	2	0.005790307	0.044938	[DDX1, SNRPG]
GO:0042493	response to drug	38	3	0.005916522	0.04494	[GATA6, SFRP1, THBS1]
GO:0006730	one-carbon metabolic process	13	2	0.006799469	0.050309	[AHCY, MTHFD1L]
GO:0006446	regulation of translational initiation	13	2	0.006799469	0.050309	[DDX1, EIF1B]
GO:0043552	positive regulation of phosphatidylinositol 3-kinase activity	13	2	0.006799469	0.050309	[PDGFRB, PTK2]
GO:0071347	cellular response to interleukin-1	13	2	0.006799469	0.050309	[HYAL2, SFRP1]
GO:0043406	positive regulation of MAP kinase activity	14	2	0.007882189	0.056562	[CD24, PDGFRB]
GO:0001658	branching involved in ureteric bud morphogenesis	14	2	0.007882189	0.056562	[BMP4, WT1]
GO:0051260	protein homooligomerization	43	3	0.008353025	0.05905	[ATL1, DPYSL3, SCUBE3]
GO:0007051	spindle organization	15	2	0.009036953	0.062342	[CHD3, UBE2C]

GO:0060326	cell chemotaxis	15	2	0.009036953	0.062342	[LEF1, PDGFRB]
GO:0043981	histone H4-K5 acetylation	15	2	0.009036953	0.062342	[HCFC1, MEAF6]
GO:0043982	histone H4-K8 acetylation	15	2	0.009036953	0.062342	[HCFC1, MEAF6]
GO:2000007	negative regulation of metanephric comma-shaped body morphogenesis	1	1	0.00968352	0.043213	[BMP4]
GO:2000005	negative regulation of metanephric S-shaped body morphogenesis	1	1	0.00968352	0.043213	[BMP4]
GO:0035441	cell migration involved in vasculogenesis	1	1	0.00968352	0.043213	[PDGFRB]
GO:0051164	L-xylitol metabolic process	1	1	0.00968352	0.043213	[SORD]
GO:0090194	negative regulation of glomerulus development	1	1	0.00968352	0.043213	[BMP4]
GO:0018277	protein deamination	1	1	0.00968352	0.043213	[LOXL1]
GO:0072101	specification of ureteric bud anterior/posterior symmetry by BMP signaling pathway	1	1	0.00968352	0.043213	[BMP4]
GO:0072104	glomerular capillary formation	1	1	0.00968352	0.043213	[BMP4]
GO:0048669	collateral sprouting in absence of injury	1	1	0.00968352	0.043213	[APP]
GO:0030223	neutrophil differentiation	1	1	0.00968352	0.043213	[LEF1]
GO:0045063	T-helper 1 cell differentiation	1	1	0.00968352	0.043213	[LEF1]
GO:0008090	retrograde axon cargo transport	1	1	0.00968352	0.043213	[NEFL]
GO:2000054	negative regulation of Wnt receptor signaling pathway involved in dorsal/ventral axis specification	1	1	0.00968352	0.043213	[SFRP1]
GO:2000052	positive regulation of non-canonical Wnt receptor signaling pathway	1	1	0.00968352	0.043213	[SFRP1]
GO:0048745	smooth muscle tissue development	1	1	0.00968352	0.043213	[BMP4]
GO:2000195	negative regulation of female gonad development	1	1	0.00968352	0.043213	[WT1]
GO:0072200	negative regulation of mesenchymal cell proliferation involved in ureter development	1	1	0.00968352	0.043213	[BMP4]
GO:2000178	negative regulation of neural precursor cell proliferation	1	1	0.00968352	0.043213	[ID2]
GO:0010807	regulation of synaptic vesicle priming	1	1	0.00968352	0.043213	[STXBP1]
GO:0055015	ventricular cardiac muscle cell development	1	1	0.00968352	0.043213	[HEY2]
GO:0050774	negative regulation of dendrite morphogenesis	1	1	0.00968352	0.043213	[YWHAH]
GO:0008088	axon cargo transport	1	1	0.00968352	0.043213	[APP]
GO:0045603	positive regulation of endothelial cell differentiation	1	1	0.00968352	0.043213	[BTG1]
GO:2000270	negative regulation of fibroblast apoptosis	1	1	0.00968352	0.043213	[SFRP1]

GO:2000271	positive regulation of fibroblast apoptosis	1	1	0.00968352	0.043213	[SFRP1]
GO:0070495	negative regulation of thrombin receptor signaling pathway	1	1	0.00968352	0.043213	[SNCA]
GO:0006713	glucocorticoid catabolic process	1	1	0.00968352	0.043213	[YWHAH]
GO:0042551	neuron maturation	1	1	0.00968352	0.043213	[CLN5]
GO:0003208	cardiac ventricle morphogenesis	1	1	0.00968352	0.043213	[HEY2]
GO:0010626	negative regulation of Schwann cell proliferation	1	1	0.00968352	0.043213	[RNF10]
GO:0048710	regulation of astrocyte differentiation	1	1	0.00968352	0.043213	[EPHA4]
GO:0071305	cellular response to vitamin D	1	1	0.00968352	0.043213	[SFRP1]
GO:0072302	negative regulation of metanephric glomerular mesangial cell proliferation	1	1	0.00968352	0.043213	[WT1]
GO:0061149	BMP signaling pathway involved in ureter morphogenesis	1	1	0.00968352	0.043213	[BMP4]
GO:0019066	translocation of virus into host cell	1	1	0.00968352	0.043213	[HYAL2]
GO:0072192	ureter epithelial cell differentiation	1	1	0.00968352	0.043213	[BMP4]
GO:2000080	negative regulation of canonical Wnt receptor signaling pathway involved in controlling type B pancreatic cell proliferation	1	1	0.00968352	0.043213	[SFRP1]
GO:0072193	ureter smooth muscle cell differentiation	1	1	0.00968352	0.043213	[BMP4]
GO:0061155	pulmonary artery endothelial tube morphogenesis	1	1	0.00968352	0.043213	[BMP4]
GO:0061151	BMP signaling pathway involved in renal system segmentation	1	1	0.00968352	0.043213	[BMP4]
GO:0006543	glutamine catabolic process	1	1	0.00968352	0.043213	[GLS]
GO:0046456	icosanoid biosynthetic process	1	1	0.00968352	0.043213	[FADS1]
GO:0019087	transformation of host cell by virus	1	1	0.00968352	0.043213	[HYAL2]
GO:0006062	sorbitol catabolic process	1	1	0.00968352	0.043213	[SORD]
GO:0009051	pentose-phosphate shunt, oxidative branch	1	1	0.00968352	0.043213	[PGD]
GO:0046851	negative regulation of bone remodeling	1	1	0.00968352	0.043213	[SFRP1]
GO:0061032	visceral serous pericardium development	1	1	0.00968352	0.043213	[WT1]
GO:0061031	endodermal digestive tract morphogenesis	1	1	0.00968352	0.043213	[ID2]
GO:0014070	response to organic cyclic compound	1	1	0.00968352	0.043213	[SFRP1]
GO:0006491	N-glycan processing	1	1	0.00968352	0.043213	[FUT8]
GO:0032600	chemokine receptor transport out of membrane raft	1	1	0.00968352	0.043213	[CD24]

GO:0061047	positive regulation of branching involved in lung morphogenesis	1	1	0.00968352	0.043213	[BMP4]
GO:0007176	regulation of epidermal growth factor-activated receptor activity	1	1	0.00968352	0.043213	[APP]
GO:0032606	type I interferon production	1	1	0.00968352	0.043213	[TBK1]
GO:0046947	hydroxylysine biosynthetic process	1	1	0.00968352	0.043213	[PLOD1]
GO:0033693	neurofilament bundle assembly	1	1	0.00968352	0.043213	[NEFL]
GO:0016072	rRNA metabolic process	1	1	0.00968352	0.043213	[MKI67IP]
GO:0022601	menstrual cycle phase	1	1	0.00968352	0.043213	[SFRP1]
GO:0021957	corticospinal tract morphogenesis	1	1	0.00968352	0.043213	[EPHA4]
GO:0072262	metanephric glomerular mesangial cell proliferation involved in metanephros development	1	1	0.00968352	0.043213	[PDGFRB]
GO:0043567	regulation of insulin-like growth factor receptor signaling pathway	1	1	0.00968352	0.043213	[IGFBP2]
GO:0071773	cellular response to BMP stimulus	1	1	0.00968352	0.043213	[SFRP1]
GO:0032597	B cell receptor transport into membrane raft	1	1	0.00968352	0.043213	[CD24]
GO:0048392	intermediate mesodermal cell differentiation	1	1	0.00968352	0.043213	[BMP4]
GO:2000623	negative regulation of nuclear-transcribed mRNA catabolic process, nonsense-mediated decay	1	1	0.00968352	0.043213	[PABPC1]
GO:0060732	positive regulation of inositol phosphate biosynthetic process	1	1	0.00968352	0.043213	[SNCA]
GO:0071380	cellular response to prostaglandin E stimulus	1	1	0.00968352	0.043213	[SFRP1]
GO:0051901	positive regulation of mitochondrial depolarization	1	1	0.00968352	0.043213	[MLLT11]
GO:0021522	spinal cord motor neuron differentiation	1	1	0.00968352	0.043213	[SOX4]
GO:0031056	regulation of histone modification	1	1	0.00968352	0.043213	[CDK9]
GO:0046370	fructose biosynthetic process	1	1	0.00968352	0.043213	[SORD]
GO:0051583	dopamine uptake	1	1	0.00968352	0.043213	[SNCA]
GO:0051585	negative regulation of dopamine uptake	1	1	0.00968352	0.043213	[SNCA]
GO:0032026	response to magnesium ion	1	1	0.00968352	0.043213	[SNCA]
GO:0007617	mating behavior	1	1	0.00968352	0.043213	[APP]
GO:2001108	positive regulation of Rho guanyl-nucleotide exchange factor activity	1	1	0.00968352	0.043213	[EPHA4]
GO:0072097	negative regulation of branch elongation involved in ureteric bud branching by BMP signaling pathway	1	1	0.00968352	0.043213	[BMP4]

GO:0032229	negative regulation of synaptic transmission, GABAergic	1	1	0.00968352	0.043213 [STXBP1]
GO:0060178	regulation of exocyst localization	1	1	0.00968352	0.043213 [RALB]
GO:0032367	intracellular cholesterol transport	1	1	0.00968352	0.043213 [VPS4B]
GO:0033993	response to lipid	1	1	0.00968352	0.043213 [VPS4B]
GO:0060160	negative regulation of dopamine receptor signaling pathway	1	1	0.00968352	0.043213 [PALM]
GO:0060981	cell migration involved in coronary angiogenesis	1	1	0.00968352	0.043213 [PDGFRB]
GO:0043111	replication fork arrest	1	1	0.00968352	0.043213 [CDK9]
GO:2000553	positive regulation of T-helper 2 cell cytokine production	1	1	0.00968352	0.043213 [PRKCZ]
GO:0051160	L-xylitol catabolic process	1	1	0.00968352	0.043213 [SORD]
GO:0035791	platelet-derived growth factor receptor-beta signaling pathway	1	1	0.00968352	0.043213 [PDGFRB]
GO:0071864	positive regulation of cell proliferation in bone marrow	1	1	0.00968352	0.043213 [LEF1]
GO:0071866	negative regulation of apoptotic process in bone marrow	1	1	0.00968352	0.043213 [LEF1]
GO:2000463	positive regulation of excitatory postsynaptic membrane potential	1	1	0.00968352	0.043213 [PRKCZ]
GO:0010517	regulation of phospholipase activity	1	1	0.00968352	0.043213 [SNCA]
GO:0071872	cellular response to epinephrine stimulus	1	1	0.00968352	0.043213 [SNCA]
GO:0051612	negative regulation of serotonin uptake	1	1	0.00968352	0.043213 [SNCA]
GO:0031639	plasminogen activation	1	1	0.00968352	0.043213 [PLAT]
GO:2000761	positive regulation of N-terminal peptidyl-lysine acetylation	1	1	0.00968352	0.043213 [SOX4]
GO:0051622	negative regulation of norepinephrine uptake	1	1	0.00968352	0.043213 [SNCA]
GO:0044154	histone H3-K14 acetylation	1	1	0.00968352	0.043213 [MEAF6]
GO:0060947	cardiac vascular smooth muscle cell differentiation	1	1	0.00968352	0.043213 [GATA6]
GO:0032911	negative regulation of transforming growth factor beta1 production	1	1	0.00968352	0.043213 [GATA6]
GO:0032912	negative regulation of transforming growth factor beta2 production	1	1	0.00968352	0.043213 [GATA6]
GO:0032913	negative regulation of transforming growth factor beta3 production	1	1	0.00968352	0.043213 [CD24]

GO:0001959	regulation of cytokine-mediated signaling pathway	1	1	0.00968352	0.043213	[CD24]
GO:2001181	positive regulation of interleukin-10 secretion	1	1	0.00968352	0.043213	[PRKCZ]
GO:0001928	regulation of exocyst assembly	1	1	0.00968352	0.043213	[RALB]
GO:0010594	regulation of endothelial cell migration	1	1	0.00968352	0.043213	[PTK2]
GO:0038007	netrin-activated signaling pathway	1	1	0.00968352	0.043213	[PTK2]
GO:0051764	actin crosslink formation	1	1	0.00968352	0.043213	[DPYSL3]
GO:0060218	hemopoietic stem cell differentiation	1	1	0.00968352	0.043213	[SFRP1]
GO:0071893	BMP signaling pathway involved in nephric duct formation	1	1	0.00968352	0.043213	[BMP4]
GO:0043627	response to estrogen stimulus	16	2	0.010262271	0.034001	[CD24, GSTM3]
GO:0031623	receptor internalization	16	2	0.010262271	0.034001	[ADRBK1, SNCA]
GO:0071356	cellular response to tumor necrosis factor	17	2	0.011556676	0.037851	[HYAL2, SFRP1]
GO:0030178	negative regulation of Wnt receptor signaling pathway	17	2	0.011556676	0.037851	[APCDD1, SFRP1]
GO:0001649	osteoblast differentiation	17	2	0.011556676	0.037851	[LEF1, SFRP1]
GO:0001822	kidney development	18	2	0.012918721	0.041737	[BMP4, WT1]
GO:0030155	regulation of cell adhesion	18	2	0.012918721	0.041737	[PPP2CA, PPP2R1A]
GO:0007409	axonogenesis	18	2	0.012918721	0.041737	[APP, ATL1]
GO:0016197	endosome transport	19	2	0.014346981	0.045326	[SNX17, VPS4B]
GO:0045727	positive regulation of translation	19	2	0.014346981	0.045326	[FAM129A, SOX4]
GO:0014068	positive regulation of phosphatidylinositol 3-kinase cascade	19	2	0.014346981	0.045326	[PDGFRB, PTK2]
GO:0090263	positive regulation of canonical Wnt receptor signaling pathway	19	2	0.014346981	0.045326	[SFRP1, SOX4]
GO:0042593	glucose homeostasis	19	2	0.014346981	0.045326	[SLC37A4, SOX4]
GO:0031214	biomineral tissue development	19	2	0.014346981	0.045326	[ENPP1, IBSP]
GO:0043984	histone H4-K16 acetylation	19	2	0.014346981	0.045326	[HCFC1, MEAF6]
GO:0051897	positive regulation of protein kinase B signaling cascade	20	2	0.015840052	0.049173	[PTK2, THBS1]
GO:0071456	cellular response to hypoxia	21	2	0.017396552	0.053424	[GATA6, SFRP1]
GO:0006006	glucose metabolic process	21	2	0.017396552	0.053424	[SLC37A4, SORD]
GO:0031532	actin cytoskeleton reorganization	21	2	0.017396552	0.053424	[ANTXR1, CDC42BPA]
GO:0001934	positive regulation of protein phosphorylation	21	2	0.017396552	0.053424	[FAM129A, PTK2]
GO:0045087	innate immune response	103	4	0.018096469	0.054982	[CD55, CLU, SERPING1, TBK1]
GO:0070534	protein K63-linked ubiquitination	22	2	0.019015117	0.057407	[UBE2S, WWP2]

GO:0008584	male gonad development	22	2	0.019015117	0.057407	[GATA6, WT1]
GO:0010739	positive regulation of protein kinase A signaling cascade	2	1	0.019273488	0.049235	[AKAP12]
GO:0008054	cyclin catabolic process	2	1	0.019273488	0.049235	[UBE2C]
GO:0090191	negative regulation of branching involved in ureteric bud morphogenesis	2	1	0.019273488	0.049235	[BMP4]
GO:0033598	mammary gland epithelial cell proliferation	2	1	0.019273488	0.049235	[ID2]
GO:0045161	neuronal ion channel clustering	2	1	0.019273488	0.049235	[MTCH1]
GO:0014846	esophagus smooth muscle contraction	2	1	0.019273488	0.049235	[SULF2]
GO:0014009	glial cell proliferation	2	1	0.019273488	0.049235	[SOX4]
GO:0010216	maintenance of DNA methylation	2	1	0.019273488	0.049235	[DNMT1]
GO:0072125	negative regulation of glomerular mesangial cell proliferation	2	1	0.019273488	0.049235	[BMP4]
GO:0035694	mitochondrial protein catabolic process	2	1	0.019273488	0.049235	[BNIP3L]
GO:0016188	synaptic vesicle maturation	2	1	0.019273488	0.049235	[STXBP1]
GO:0015891	siderophore transport	2	1	0.019273488	0.049235	[SLC22A17]
GO:0008089	anterograde axon cargo transport	2	1	0.019273488	0.049235	[NEFL]
GO:0002437	inflammatory response to antigenic stimulus	2	1	0.019273488	0.049235	[PNMA1]
GO:0070295	renal water absorption	2	1	0.019273488	0.049235	[HYAL2]
GO:0045777	positive regulation of blood pressure	2	1	0.019273488	0.049235	[ID2]
GO:0030856	regulation of epithelial cell differentiation	2	1	0.019273488	0.049235	[CD24]
GO:0030854	positive regulation of granulocyte differentiation	2	1	0.019273488	0.049235	[LEF1]
GO:0045630	positive regulation of T-helper 2 cell differentiation	2	1	0.019273488	0.049235	[PRKCZ]
GO:0046628	positive regulation of insulin receptor signaling pathway	2	1	0.019273488	0.049235	[PRKCZ]
GO:0019046	reactivation of latent virus	2	1	0.019273488	0.049235	[HCFC1]
GO:2000065	negative regulation of cortisol biosynthetic process	2	1	0.019273488	0.049235	[DKK3]
GO:0006097	glyoxylate cycle	2	1	0.019273488	0.049235	[IDH1]
GO:0051967	negative regulation of synaptic transmission, glutamatergic	2	1	0.019273488	0.049235	[ATAD1]
GO:0006282	regulation of DNA repair	2	1	0.019273488	0.049235	[CDK9]
GO:0097156	fasciculation of motor neuron axon	2	1	0.019273488	0.049235	[EPHA4]
GO:0097155	fasciculation of sensory neuron axon	2	1	0.019273488	0.049235	[EPHA4]

GO:0032510	endosome to lysosome transport via multivesicular body sorting pathway	2	1	0.019273488	0.049235	[VPS4B]
GO:0032696	negative regulation of interleukin-13 production	2	1	0.019273488	0.049235	[LEF1]
GO:0040037	negative regulation of fibroblast growth factor receptor signaling pathway	2	1	0.019273488	0.049235	[SULF2]
GO:0032516	positive regulation of phosphoprotein phosphatase activity	2	1	0.019273488	0.049235	[PDGFRB]
GO:0061030	epithelial cell differentiation involved in mammary gland alveolus development	2	1	0.019273488	0.049235	[ID2]
GO:0033689	negative regulation of osteoblast proliferation	2	1	0.019273488	0.049235	[SFRP1]
GO:0010040	response to iron(II) ion	2	1	0.019273488	0.049235	[SNCA]
GO:0019896	axon transport of mitochondrion	2	1	0.019273488	0.049235	[NEFL]
GO:0009950	dorsal/ventral axis specification	2	1	0.019273488	0.049235	[SFRP1]
GO:0014911	positive regulation of smooth muscle cell migration	2	1	0.019273488	0.049235	[PDGFRB]
GO:0046826	negative regulation of protein export from nucleus	2	1	0.019273488	0.049235	[SOX4]
GO:0006027	glycosaminoglycan catabolic process	2	1	0.019273488	0.049235	[HYAL2]
GO:0003156	regulation of organ formation	2	1	0.019273488	0.049235	[WT1]
GO:0072166	posterior mesonephric tubule development	2	1	0.019273488	0.049235	[WT1]
GO:0035990	tendon cell differentiation	2	1	0.019273488	0.049235	[BMP4]
GO:0071392	cellular response to estradiol stimulus	2	1	0.019273488	0.049235	[SFRP1]
GO:0035993	deltoid tuberosity development	2	1	0.019273488	0.049235	[BMP4]
GO:0070537	histone H2A K63-linked deubiquitination	2	1	0.019273488	0.049235	[UIMC1]
GO:0035802	adrenal cortex formation	2	1	0.019273488	0.049235	[WT1]
GO:0032317	regulation of Rap GTPase activity	2	1	0.019273488	0.049235	[EPHA4]
GO:2001243	negative regulation of intrinsic apoptotic signaling pathway	2	1	0.019273488	0.049235	[SRC]
GO:0090068	positive regulation of cell cycle process	2	1	0.019273488	0.049235	[LEF1]
GO:0008344	adult locomotory behavior	2	1	0.019273488	0.049235	[APP]
GO:0006002	fructose 6-phosphate metabolic process	2	1	0.019273488	0.049235	[PFKL]
GO:0016557	peroxisome membrane biogenesis	2	1	0.019273488	0.049235	[PEX19]
GO:0060749	mammary gland alveolus development	2	1	0.019273488	0.049235	[ID2]
GO:0032714	negative regulation of interleukin-5 production	2	1	0.019273488	0.049235	[LEF1]
GO:0032713	negative regulation of interleukin-4 production	2	1	0.019273488	0.049235	[LEF1]

GO:0035860	glial cell-derived neurotrophic factor receptor signaling pathway	2	1	0.019273488	0.049235	[SULF2]
GO:0001841	neural tube formation	2	1	0.019273488	0.049235	[SOX4]
GO:0060384	innervation	2	1	0.019273488	0.049235	[SULF2]
GO:0021782	glial cell development	2	1	0.019273488	0.049235	[SOX4]
GO:0090402	oncogene-induced senescence	2	1	0.019273488	0.049235	[HMGA1]
GO:0043408	regulation of MAPK cascade	2	1	0.019273488	0.049235	[CD24]
GO:0043401	steroid hormone mediated signaling pathway	2	1	0.019273488	0.049235	[BMP4]
GO:0001869	negative regulation of complement activation, lectin pathway	2	1	0.019273488	0.049235	[SERPING1]
GO:0051573	negative regulation of histone H3-K9 methylation	2	1	0.019273488	0.049235	[DNMT1]
GO:0043254	regulation of protein complex assembly	2	1	0.019273488	0.049235	[HCFC1]
GO:0032348	negative regulation of aldosterone biosynthetic process	2	1	0.019273488	0.049235	[DKK3]
GO:0015760	glucose-6-phosphate transport	2	1	0.019273488	0.049235	[SLC37A4]
GO:0031936	negative regulation of chromatin silencing	2	1	0.019273488	0.049235	[HMGA1]
GO:0031536	positive regulation of exit from mitosis	2	1	0.019273488	0.049235	[UBE2C]
GO:0051145	smooth muscle cell differentiation	2	1	0.019273488	0.049235	[GATA6]
GO:0002576	platelet degranulation	2	1	0.019273488	0.049235	[STXBP1]
GO:0045920	negative regulation of exocytosis	2	1	0.019273488	0.049235	[SNCA]
GO:0007412	axon target recognition	2	1	0.019273488	0.049235	[STXBP1]
GO:0048681	negative regulation of axon regeneration	2	1	0.019273488	0.049235	[EPHA4]
GO:2000664	positive regulation of interleukin-5 secretion	2	1	0.019273488	0.049235	[PRKCZ]
GO:2000667	positive regulation of interleukin-13 secretion	2	1	0.019273488	0.049235	[PRKCZ]
GO:0050803	regulation of synapse structure and activity	2	1	0.019273488	0.049235	[APP]
GO:0015991	ATP hydrolysis coupled proton transport	2	1	0.019273488	0.049235	[ATP6V1H]
GO:0003357	noradrenergic neuron differentiation	2	1	0.019273488	0.049235	[SOX4]
GO:0061298	retina vasculature development in camera-type eye	2	1	0.019273488	0.049235	[PDGFRB]
GO:0031643	positive regulation of myelination	2	1	0.019273488	0.049235	[RNF10]
GO:0071899	negative regulation of estrogen receptor binding	2	1	0.019273488	0.049235	[LEF1]
GO:0051271	negative regulation of cellular component movement	2	1	0.019273488	0.049235	[ACTN1]
GO:0097084	vascular smooth muscle cell development	2	1	0.019273488	0.049235	[HEY2]
GO:0043123	positive regulation of I-kappaB kinase/NF-kappaB cascade	105	4	0.019276367	0.042743	[S100B, SLC20A1, TBK1, TRIM13]

GO:0046777	protein autophosphorylation	107	4	0.020502762	0.045322	[EPHA4, PDGFRB, PTK2, STK16]
GO:0051028	mRNA transport	60	3	0.020539292	0.045263	[NUP50, NUP93, XPO7]
GO:0006749	glutathione metabolic process	25	2	0.024229888	0.053149	[GGT7, GSTM3]
GO:0048015	phosphatidylinositol-mediated signaling	25	2	0.024229888	0.053149	[PDGFRB, UBE2C]
GO:0042981	regulation of apoptotic process	65	3	0.02531203	0.055268	[ACTN1, BTG1, NAE1]
GO:0070979	protein K11-linked ubiquitination	26	2	0.026083496	0.056779	[UBE2C, UBE2S]
GO:0000122	negative regulation of transcription from RNA polymerase II promoter	117	4	0.027346068	0.059347	[GATA6, HCFC1, HEY2, PTH]
GO:0000184	nuclear-transcribed mRNA catabolic process, nonsense-mediated decay	27	2	0.027992658	0.060474	[PAN3, RNPS1]
GO:0051607	defense response to virus	27	2	0.027992658	0.060474	[BNIP3L, SAMHD1]
GO:0006508	proteolysis	476	10	0.028111622	0.060457	[ADAMTS7, CASP3, CPE, CTSC, PIGK, PLAT, SEC11A, SERPING1, UCHL1, USP7]
GO:0006457	protein folding	68	3	0.028436989	0.060973	[PFDN4, PPIL1, PPIL4]
GO:0033314	mitotic cell cycle DNA replication checkpoint	3	1	0.028770805	0.057542	[NAE1]
GO:0030201	heparan sulfate proteoglycan metabolic process	3	1	0.028770805	0.057542	[SULF2]
GO:0006636	unsaturated fatty acid biosynthetic process	3	1	0.028770805	0.057542	[FADS1]
GO:0030422	production of siRNA involved in RNA interference	3	1	0.028770805	0.057542	[PRKRA]
GO:0007194	negative regulation of adenylate cyclase activity	3	1	0.028770805	0.057542	[PALM]
GO:2000117	negative regulation of cysteine-type endopeptidase activity	3	1	0.028770805	0.057542	[SFRP1]
GO:0048663	neuron fate commitment	3	1	0.028770805	0.057542	[ID2]
GO:0009888	tissue development	3	1	0.028770805	0.057542	[WT1]
GO:0055003	cardiac myofibril assembly	3	1	0.028770805	0.057542	[PDGFRB]
GO:0002768	immune response-regulating cell surface receptor signaling pathway	3	1	0.028770805	0.057542	[CD24]
GO:0008634	negative regulation of survival gene product expression	3	1	0.028770805	0.057542	[BNIP3L]
GO:0003211	cardiac ventricle formation	3	1	0.028770805	0.057542	[SOX4]
GO:0032508	DNA duplex unwinding	3	1	0.028770805	0.057542	[DDX1]
GO:0042416	dopamine biosynthetic process	3	1	0.028770805	0.057542	[SNCA]
GO:0006268	DNA unwinding involved in replication	3	1	0.028770805	0.057542	[HMGA1]
GO:0009395	phospholipid catabolic process	3	1	0.028770805	0.057542	[PRDX6]
GO:0030539	male genitalia development	3	1	0.028770805	0.057542	[WT1]

GO:0072277	metanephric glomerular capillary formation	3	1	0.028770805	0.057542	[PDGFRB]
GO:0007035	vacuolar acidification	3	1	0.028770805	0.057542	[ATP6V1H]
GO:0072284	metanephric S-shaped body morphogenesis	3	1	0.028770805	0.057542	[WT1]
GO:0045578	negative regulation of B cell differentiation	3	1	0.028770805	0.057542	[SFRP1]
GO:0014059	regulation of dopamine secretion	3	1	0.028770805	0.057542	[SNCA]
GO:0061077	chaperone-mediated protein folding	3	1	0.028770805	0.057542	[CLU]
GO:0006467	protein thiol-disulfide exchange	3	1	0.028770805	0.057542	[PIGK]
GO:0035986	senescence-associated heterochromatin focus assembly	3	1	0.028770805	0.057542	[HMGA1]
GO:0006337	nucleosome disassembly	3	1	0.028770805	0.057542	[HMGA1]
GO:0061099	negative regulation of protein tyrosine kinase activity	3	1	0.028770805	0.057542	[HYAL2]
GO:0006102	isocitrate metabolic process	3	1	0.028770805	0.057542	[IDH1]
GO:0071371	cellular response to gonadotropin stimulus	3	1	0.028770805	0.057542	[WT1]
GO:0040014	regulation of multicellular organism growth	3	1	0.028770805	0.057542	[APP]
GO:0070779	D-aspartate import	3	1	0.028770805	0.057542	[SLC1A1]
GO:0007611	learning or memory	3	1	0.028770805	0.057542	[S100B]
GO:0071353	cellular response to interleukin-4	3	1	0.028770805	0.057542	[LEF1]
GO:0042167	heme catabolic process	3	1	0.028770805	0.057542	[AMBP]
GO:0051938	L-glutamate import	3	1	0.028770805	0.057542	[SLC1A1]
GO:0060766	negative regulation of androgen receptor signaling pathway	3	1	0.028770805	0.057542	[SFRP1]
GO:0048488	synaptic vesicle endocytosis	3	1	0.028770805	0.057542	[SNCA]
GO:0060174	limb bud formation	3	1	0.028770805	0.057542	[SOX4]
GO:0002237	response to molecule of bacterial origin	3	1	0.028770805	0.057542	[CD24]
GO:0060563	neuroepithelial cell differentiation	3	1	0.028770805	0.057542	[SOX4]
GO:0050732	negative regulation of peptidyl-tyrosine phosphorylation	3	1	0.028770805	0.057542	[SFRP1]
GO:0002063	chondrocyte development	3	1	0.028770805	0.057542	[SULF2]
GO:0035793	positive regulation of metanephric mesenchymal cell migration by platelet-derived growth factor receptor-beta signaling pathway	3	1	0.028770805	0.057542	[PDGFRB]
GO:0001775	cell activation	3	1	0.028770805	0.057542	[CD24]
GO:0051224	negative regulation of protein transport	3	1	0.028770805	0.057542	[WWP2]
GO:0048678	response to axon injury	3	1	0.028770805	0.057542	[DPYSL3]

GO:0090280	positive regulation of calcium ion import	3	1	0.028770805	0.057542	[PDGFRB]
GO:0007204	elevation of cytosolic calcium ion concentration	28	2	0.029956129	0.056138	[CD24, CD55]
GO:0042127	regulation of cell proliferation	29	2	0.031972684	0.059682	[PTK2, TCFL5]
GO:0016579	protein deubiquitination	29	2	0.031972684	0.059682	[UCHL1, USP7]
GO:0030307	positive regulation of cell growth	30	2	0.034041115	0.063213	[LEF1, SFRP1]
GO:0030336	negative regulation of cell migration	30	2	0.034041115	0.063213	[DPYSL3, SFRP1]
GO:0006468	protein phosphorylation	185	5	0.034694115	0.064175	[APP, CDC42BPA, CSNK1E, MAPK3, PRKCZ]
GO:0050821	protein stabilization	31	2	0.036160235	0.066714	[CLU, HCFC1]
GO:0009303	rRNA transcription	4	1	0.038176365	0.066564	[MKI67IP]
GO:0010719	negative regulation of epithelial to mesenchymal transition	4	1	0.038176365	0.066564	[SFRP1]
GO:0045047	protein targeting to ER	4	1	0.038176365	0.066564	[SEC61A1]
GO:0016199	axon midline choice point recognition	4	1	0.038176365	0.066564	[APP]
GO:0045725	positive regulation of glycogen biosynthetic process	4	1	0.038176365	0.066564	[PTH]
GO:0045663	positive regulation of myoblast differentiation	4	1	0.038176365	0.066564	[BTG1]
GO:0030214	hyaluronan catabolic process	4	1	0.038176365	0.066564	[HYAL2]
GO:0072001	renal system development	4	1	0.038176365	0.066564	[BMP4]
GO:0000085	G2 phase of mitotic cell cycle	4	1	0.038176365	0.066564	[APP]
GO:0071569	protein ufmylation	4	1	0.038176365	0.066564	[UBA5]
GO:0072584	caveolin-mediated endocytosis	4	1	0.038176365	0.066564	[MAPK3]
GO:0006942	regulation of striated muscle contraction	4	1	0.038176365	0.066564	[ATP1A2]
GO:0035519	protein K29-linked ubiquitination	4	1	0.038176365	0.066564	[UBE2S]
GO:0006561	proline biosynthetic process	4	1	0.038176365	0.066564	[ALDH18A1]
GO:0048806	genitalia development	4	1	0.038176365	0.066564	[FOXF2]
GO:0007042	lysosomal lumen acidification	4	1	0.038176365	0.066564	[CLN5]
GO:0032753	positive regulation of interleukin-4 production	4	1	0.038176365	0.066564	[PRKCZ]
GO:0048255	mRNA stabilization	4	1	0.038176365	0.066564	[PABPC1]
GO:0022408	negative regulation of cell-cell adhesion	4	1	0.038176365	0.066564	[LEF1]
GO:0022409	positive regulation of cell-cell adhesion	4	1	0.038176365	0.066564	[LEF1]
GO:0042921	glucocorticoid receptor signaling pathway	4	1	0.038176365	0.066564	[YWHAH]
GO:0032872	regulation of stress-activated MAPK cascade	4	1	0.038176365	0.066564	[MAPK3]
GO:0042104	positive regulation of activated T cell proliferation	4	1	0.038176365	0.066564	[CD24]
GO:0050921	positive regulation of chemotaxis	4	1	0.038176365	0.066564	[PDGFRB]
GO:0043010	camera-type eye development	4	1	0.038176365	0.066564	[WT1]

GO:0043206	fibril organization	4	1	0.038176365	0.066564	[SNCA]
GO:0048268	clathrin coat assembly	4	1	0.038176365	0.066564	[CALY]
GO:0060291	long-term synaptic potentiation	4	1	0.038176365	0.066564	[PRKCZ]
GO:0090026	positive regulation of monocyte chemotaxis	4	1	0.038176365	0.066564	[CXCL12]
GO:0031295	T cell costimulation	4	1	0.038176365	0.066564	[CD24]
GO:0035067	negative regulation of histone acetylation	4	1	0.038176365	0.066564	[SNCA]
GO:0032835	glomerulus development	4	1	0.038176365	0.066564	[WT1]
GO:0051571	positive regulation of histone H3-K4 methylation	4	1	0.038176365	0.066564	[DNMT1]
GO:0044314	protein K27-linked ubiquitination	4	1	0.038176365	0.066564	[UBE2S]
GO:0007530	sex determination	4	1	0.038176365	0.066564	[WT1]
GO:2000774	positive regulation of cellular senescence	4	1	0.038176365	0.066564	[HMGA1]
GO:0003337	mesenchymal to epithelial transition involved in metanephros morphogenesis	4	1	0.038176365	0.066564	[BMP4]
GO:0002092	positive regulation of receptor internalization	4	1	0.038176365	0.066564	[ATAD1]
GO:0031032	actomyosin structure organization	4	1	0.038176365	0.066564	[CDC42BPA]
GO:0090184	positive regulation of kidney development	4	1	0.038176365	0.066564	[BMP4]
GO:0051281	positive regulation of release of sequestered calcium ion into cytosol	4	1	0.038176365	0.066564	[SNCA]
GO:0090170	regulation of Golgi inheritance	4	1	0.038176365	0.066564	[MAPK3]
GO:2000641	regulation of early endosome to late endosome transport	4	1	0.038176365	0.066564	[MAPK3]
GO:0010575	positive regulation vascular endothelial growth factor production	4	1	0.038176365	0.066564	[SULF2]
GO:0031047	gene silencing by RNA	32	2	0.038328873	0.063203	[PRKRA, TNRC6A]
GO:0007218	neuropeptide signaling pathway	32	2	0.038328873	0.063203	[CARTPT, CPE]
GO:0001666	response to hypoxia	32	2	0.038328873	0.063203	[CD24, PLOD1]
GO:0006302	double-strand break repair	33	2	0.040545876	0.066475	[DDX1, UIMC1]
GO:0007420	brain development	33	2	0.040545876	0.066475	[CLN5, RAB18]
GO:0045766	positive regulation of angiogenesis	34	2	0.042810108	0.069946	[BTG1, GATA6]
GO:0008643	carbohydrate transport	35	2	0.045120452	0.073553	[SLC2A1, SLC37A4]
GO:0051168	nuclear export	5	1	0.047491052	0.073714	[LSG1]
GO:0021510	spinal cord development	5	1	0.047491052	0.073714	[SOX4]
GO:0010718	positive regulation of epithelial to mesenchymal transition	5	1	0.047491052	0.073714	[LEF1]

GO:0010863	positive regulation of phospholipase C activity	5	1	0.047491052	0.073714	[PDGFRB]
GO:0045667	regulation of osteoblast differentiation	5	1	0.047491052	0.073714	[PTK2]
GO:2000020	positive regulation of male gonad development	5	1	0.047491052	0.073714	[WT1]
GO:0045109	intermediate filament organization	5	1	0.047491052	0.073714	[NEFL]
GO:0045116	protein neddylation	5	1	0.047491052	0.073714	[NAE1]
GO:0042535	positive regulation of tumor necrosis factor biosynthetic process	5	1	0.047491052	0.073714	[THBS1]
GO:0072207	metanephric epithelium development	5	1	0.047491052	0.073714	[WT1]
GO:0072205	metanephric collecting duct development	5	1	0.047491052	0.073714	[BMP4]
GO:0003007	heart morphogenesis	5	1	0.047491052	0.073714	[PTK2]
GO:0006098	pentose-phosphate shunt	5	1	0.047491052	0.073714	[PGD]
GO:0046676	negative regulation of insulin secretion	5	1	0.047491052	0.073714	[SFRP1]
GO:0046488	phosphatidylinositol metabolic process	5	1	0.047491052	0.073714	[PDGFRB]
GO:0035235	ionotropic glutamate receptor signaling pathway	5	1	0.047491052	0.073714	[APP]
GO:0033235	positive regulation of protein sumoylation	5	1	0.047491052	0.073714	[PIAS3]
GO:0007029	endoplasmic reticulum organization	5	1	0.047491052	0.073714	[ATL1]
GO:0019367	fatty acid elongation, saturated fatty acid	5	1	0.047491052	0.073714	[ELOVL4]
GO:0006878	cellular copper ion homeostasis	5	1	0.047491052	0.073714	[APP]
GO:0007188	G-protein signaling, coupled to cAMP nucleotide second messenger	5	1	0.047491052	0.073714	[PTH1R]
GO:0009952	anterior/posterior pattern specification	5	1	0.047491052	0.073714	[HOXD3]
GO:0033673	negative regulation of kinase activity	5	1	0.047491052	0.073714	[PIK3IP1]
GO:0071493	cellular response to UV-B	5	1	0.047491052	0.073714	[HYAL2]
GO:0003094	glomerular filtration	5	1	0.047491052	0.073714	[SULF2]
GO:0071391	cellular response to estrogen stimulus	5	1	0.047491052	0.073714	[SFRP1]
GO:0008272	sulfate transport	5	1	0.047491052	0.073714	[SLC26A11]
GO:0071280	cellular response to copper ion	5	1	0.047491052	0.073714	[SNCA]
GO:0071363	cellular response to growth factor stimulus	5	1	0.047491052	0.073714	[SFRP1]
GO:0048557	embryonic digestive tract morphogenesis	5	1	0.047491052	0.073714	[ID2]
GO:0071504	cellular response to heparin	5	1	0.047491052	0.073714	[SFRP1]
GO:0071157	negative regulation of cell cycle arrest	5	1	0.047491052	0.073714	[CDK9]
GO:0007612	learning	5	1	0.047491052	0.073714	[ATAD1]
GO:0016322	neuron remodeling	5	1	0.047491052	0.073714	[APP]
GO:0071333	cellular response to glucose stimulus	5	1	0.047491052	0.073714	[SOX4]

GO:0070555	response to interleukin-1	5	1	0.047491052	0.073714	[SNCA]
GO:0016126	sterol biosynthetic process	5	1	0.047491052	0.073714	[SQLE]
GO:0016255	attachment of GPI anchor to protein	5	1	0.047491052	0.073714	[PIGK]
GO:0070848	response to growth factor stimulus	5	1	0.047491052	0.073714	[GATA6]
GO:0090398	cellular senescence	5	1	0.047491052	0.073714	[ID2]
GO:0010564	regulation of cell cycle process	5	1	0.047491052	0.073714	[SFRP1]
GO:0045839	negative regulation of mitosis	5	1	0.047491052	0.073714	[BMP4]
GO:0010642	negative regulation of platelet-derived growth factor receptor signaling pathway	5	1	0.047491052	0.073714	[SNCA]
GO:0007165	signal transduction	141	4	0.048752367	0.072218	[PDCL, PDGFRB, PSD, TRIM54]
GO:0006096	glycolysis	37	2	0.04987509	0.073728	[ENO2, PFKL]
GO:0006357	regulation of transcription from RNA polymerase II promoter	38	2	0.052317233	0.077179	[IKBKAP, WT1]
GO:0016042	lipid catabolic process	87	3	0.052682808	0.077558	[LIPA, PAFAH1B3, PRDX6]
GO:0070374	positive regulation of ERK1 and ERK2 cascade	39	2	0.054801185	0.080428	[PDGFRB, PRKCZ]
GO:0043161	proteasomal ubiquitin-dependent protein catabolic process	39	2	0.054801185	0.080428	[TRIM13, WWP2]
GO:0034976	response to endoplasmic reticulum stress	6	1	0.056715742	0.080587	[FAM129A]
GO:0008045	motor axon guidance	6	1	0.056715742	0.080587	[EPHA4]
GO:0055074	calcium ion homeostasis	6	1	0.056715742	0.080587	[SNCA]
GO:0045880	positive regulation of smoothened signaling pathway	6	1	0.056715742	0.080587	[SFRP1]
GO:0000186	activation of MAPKK activity	6	1	0.056715742	0.080587	[BMP4]
GO:0010862	positive regulation of pathway-restricted SMAD protein phosphorylation	6	1	0.056715742	0.080587	[BMP4]
GO:0045668	negative regulation of osteoblast differentiation	6	1	0.056715742	0.080587	[TWIST2]
GO:0048008	platelet-derived growth factor receptor signaling pathway	6	1	0.056715742	0.080587	[PDGFRB]
GO:0050777	negative regulation of immune response	6	1	0.056715742	0.080587	[AMBP]
GO:2000379	positive regulation of reactive oxygen species metabolic process	6	1	0.056715742	0.080587	[PDGFRB]
GO:0048706	embryonic skeletal system development	6	1	0.056715742	0.080587	[SULF2]
GO:0061001	regulation of dendritic spine morphogenesis	6	1	0.056715742	0.080587	[EPHA4]
GO:0008542	visual learning	6	1	0.056715742	0.080587	[APP]
GO:0051893	regulation of focal adhesion assembly	6	1	0.056715742	0.080587	[PTK2]

GO:0051489	regulation of filopodium assembly	6	1	0.056715742	0.080587	[GAP43]
GO:0032319	regulation of Rho GTPase activity	6	1	0.056715742	0.080587	[PTK2]
GO:0006200	ATP catabolic process	6	1	0.056715742	0.080587	[VPS4B]
GO:0006398	histone mRNA 3'-end processing	6	1	0.056715742	0.080587	[CPSF2]
GO:0032024	positive regulation of insulin secretion	6	1	0.056715742	0.080587	[SOX4]
GO:0035278	negative regulation of translation involved in gene silencing by miRNA	6	1	0.056715742	0.080587	[TNRC6A]
GO:0031274	positive regulation of pseudopodium assembly	6	1	0.056715742	0.080587	[CDC42EP4]
GO:0001890	placenta development	6	1	0.056715742	0.080587	[PTK2]
GO:2000573	positive regulation of DNA biosynthetic process	6	1	0.056715742	0.080587	[PDGFRB]
GO:0044344	cellular response to fibroblast growth factor stimulus	6	1	0.056715742	0.080587	[SFRP1]
GO:0017144	drug metabolic process	6	1	0.056715742	0.080587	[VKORC1]
GO:0001958	endochondral ossification	6	1	0.056715742	0.080587	[BMP4]
GO:0030279	negative regulation of ossification	6	1	0.056715742	0.080587	[SFRP1]
GO:0001921	positive regulation of receptor recycling	6	1	0.056715742	0.080587	[SNCA]
GO:0043983	histone H4-K12 acetylation	6	1	0.056715742	0.080587	[MEAF6]
GO:0001933	negative regulation of protein phosphorylation	6	1	0.056715742	0.080587	[FAM129A]
GO:0007399	nervous system development	214	5	0.058047358	0.080011	[DCTN1, EPHA4, GAP43, INA, NRSN1]
GO:0022900	electron transport chain	41	2	0.059890396	0.082234	[CYBA, FADS1]
GO:0006886	intracellular protein transport	41	2	0.059890396	0.082234	[SNX17, YWHAH]
GO:0000910	cytokinesis	41	2	0.059890396	0.082234	[BECN1, RALB]
GO:0006633	fatty acid biosynthetic process	42	2	0.062493634	0.085316	[ELOVL4, SCD]
GO:0071260	cellular response to mechanical stimulus	42	2	0.062493634	0.085316	[HABP4, MAPK3]
GO:0030182	neuron differentiation	42	2	0.062493634	0.085316	[FZD5, ITM2C]
GO:0045088	regulation of innate immune response	7	1	0.065851303	0.08772	[SAMHD1]
GO:0006625	protein targeting to peroxisome	7	1	0.065851303	0.08772	[PEX19]
GO:0051090	regulation of sequence-specific DNA binding transcription factor activity	7	1	0.065851303	0.08772	[USP7]
GO:0048870	cell motility	7	1	0.065851303	0.08772	[PTK2]
GO:0022008	neurogenesis	7	1	0.065851303	0.08772	[CLN5]
GO:0006707	cholesterol catabolic process	7	1	0.065851303	0.08772	[SNX17]
GO:0045600	positive regulation of fat cell differentiation	7	1	0.065851303	0.08772	[SFRP1]
GO:0050770	regulation of axonogenesis	7	1	0.065851303	0.08772	[EPHA4]
GO:0042769	DNA damage response, detection of DNA damage	7	1	0.065851303	0.08772	[SOX4]

GO:0042761	very long-chain fatty acid biosynthetic process	7	1	0.065851303	0.08772	[ELOVL4]
GO:0048704	embryonic skeletal system morphogenesis	7	1	0.065851303	0.08772	[HOXD3]
GO:0030878	thyroid gland development	7	1	0.065851303	0.08772	[HOXD3]
GO:0035518	histone H2A monoubiquitination	7	1	0.065851303	0.08772	[RING1]
GO:0042326	negative regulation of phosphorylation	7	1	0.065851303	0.08772	[BMP4]
GO:0032314	regulation of Rac GTPase activity	7	1	0.065851303	0.08772	[EPHA4]
GO:0006103	2-oxoglutarate metabolic process	7	1	0.065851303	0.08772	[IDH1]
GO:0048485	sympathetic nervous system development	7	1	0.065851303	0.08772	[SOX4]
GO:0043923	positive regulation by host of viral transcription	7	1	0.065851303	0.08772	[LEF1]
GO:0060395	SMAD protein signal transduction	7	1	0.065851303	0.08772	[BMP4]
GO:0042572	retinol metabolic process	7	1	0.065851303	0.08772	[RDH11]
GO:0085020	protein K6-linked ubiquitination	7	1	0.065851303	0.08772	[UBE2S]
GO:0050808	synapse organization	7	1	0.065851303	0.08772	[NLGN3]
GO:0034341	response to interferon-gamma	7	1	0.065851303	0.08772	[SNCA]
GO:0051092	positive regulation of NF-kappaB transcription factor activity	45	2	0.070526081	0.09189	[CLU, PRKCZ]
GO:0016567	protein ubiquitination	99	3	0.071754129	0.09332	[DCAF10, UBE2C, WWP2]
GO:0030335	positive regulation of cell migration	46	2	0.073274619	0.095037	[LEF1, PTK2]
GO:0006869	lipid transport	46	2	0.073274619	0.095037	[APOO, PLTP]
GO:0031397	negative regulation of protein ubiquitination	8	1	0.074898593	0.095752	[SOX4]
GO:0045664	regulation of neuron differentiation	8	1	0.074898593	0.095752	[YWHAH]
GO:0033628	regulation of cell adhesion mediated by integrin	8	1	0.074898593	0.095752	[PTK2]
GO:0042325	regulation of phosphorylation	8	1	0.074898593	0.095752	[CD24]
GO:0046326	positive regulation of glucose import	8	1	0.074898593	0.095752	[PTH]
GO:0035176	social behavior	8	1	0.074898593	0.095752	[NLGN3]
GO:0035810	positive regulation of urine volume	8	1	0.074898593	0.095752	[HYAL2]
GO:0070527	platelet aggregation	8	1	0.074898593	0.095752	[STXBP1]
GO:0006309	DNA fragmentation involved in apoptotic nuclear change	8	1	0.074898593	0.095752	[SFRP1]
GO:0007613	memory	8	1	0.074898593	0.095752	[ATAD1]
GO:2001237	negative regulation of extrinsic apoptotic signaling pathway	8	1	0.074898593	0.095752	[SRC]
GO:0071230	cellular response to amino acid stimulus	8	1	0.074898593	0.095752	[RRAGB]
GO:0071320	cellular response to cAMP	8	1	0.074898593	0.095752	[WT1]

GO:0001942	hair follicle development	8	1	0.074898593	0.095752	[APCDD1]
GO:0001525	angiogenesis	102	3	0.076943029	0.097062	[COL8A1, PTK2, TGFBI]
GO:0030100	regulation of endocytosis	9	1	0.083858464	0.104676	[SNX17]
GO:0045840	positive regulation of mitosis	9	1	0.083858464	0.104676	[PDGFRB]
GO:0030819	positive regulation of cAMP biosynthetic process	9	1	0.083858464	0.104676	[AKAP12]
GO:0045665	negative regulation of neuron differentiation	9	1	0.083858464	0.104676	[ID2]
GO:0006513	protein monoubiquitination	9	1	0.083858464	0.104676	[UBE2R2]
GO:0006611	protein export from nucleus	9	1	0.083858464	0.104676	[XPO7]
GO:0030513	positive regulation of BMP signaling pathway	9	1	0.083858464	0.104676	[BMP4]
GO:0001837	epithelial to mesenchymal transition	9	1	0.083858464	0.104676	[FOXF2]
GO:0042355	L-fucose catabolic process	9	1	0.083858464	0.104676	[FUT8]
GO:0090200	positive regulation of release of cytochrome c from mitochondria	9	1	0.083858464	0.104676	[MLLT11]
GO:0051402	neuron apoptosis	9	1	0.083858464	0.104676	[APP]
GO:0006811	ion transport	455	8	0.088679618	0.109545	[ANO10, ATP6AP1, ATP6V1B2, ATP6V1H, FXYD6, SLC22A17, SLC26A11, SLC39A7]
GO:0007283	spermatogenesis	174	4	0.089612007	0.110506	[CCNI, DZIP1, PNMA1, TCFL5]
GO:0006750	glutathione biosynthetic process	10	1	0.092731759	0.112603	[GGT7]
GO:0042632	cholesterol homeostasis	10	1	0.092731759	0.112603	[CD24]
GO:0045332	phospholipid translocation	10	1	0.092731759	0.112603	[ATP9A]
GO:0048754	branching morphogenesis of a tube	10	1	0.092731759	0.112603	[BMP4]
GO:0045787	positive regulation of cell cycle	10	1	0.092731759	0.112603	[HCFC1]
GO:0006099	tricarboxylic acid cycle	10	1	0.092731759	0.112603	[IDH1]
GO:0006957	complement activation, alternative pathway	10	1	0.092731759	0.112603	[CFH]
GO:0007026	negative regulation of microtubule depolymerization	10	1	0.092731759	0.112603	[TRIM54]
GO:0048167	regulation of synaptic plasticity	10	1	0.092731759	0.112603	[YWHAH]
GO:0006977	DNA damage response, signal transduction by p53 class mediator resulting in cell cycle arrest	10	1	0.092731759	0.112603	[SOX4]
GO:0009966	regulation of signal transduction	10	1	0.092731759	0.112603	[MTCH1]
GO:0001823	mesonephros development	10	1	0.092731759	0.112603	[BMP4]
GO:0032007	negative regulation of TOR signaling cascade	10	1	0.092731759	0.112603	[TMEM127]
GO:0001657	ureteric bud development	10	1	0.092731759	0.112603	[WT1]
GO:0001654	eye development	10	1	0.092731759	0.112603	[RAB18]
GO:0031648	protein destabilization	10	1	0.092731759	0.112603	[SNCA]

GO:0008299	isoprenoid biosynthetic process	10	1	0.092731759	0.112603	[FDFT1]
GO:0007059	chromosome segregation	55	2	0.099433705	0.118921	[PPP2R1A, SLC25A5]
GO:0007265	Ras protein signal transduction	11	1	0.101519311	0.120407	[RGL2]
GO:0030317	sperm motility	11	1	0.101519311	0.120407	[SORD]
GO:0034504	protein localization to nucleus	11	1	0.101519311	0.120407	[BMP4]
GO:0046677	response to antibiotic	11	1	0.101519311	0.120407	[HYAL2]
GO:0006891	intra-Golgi vesicle-mediated transport	11	1	0.101519311	0.120407	[COPB1]
GO:0046329	negative regulation of JNK cascade	11	1	0.101519311	0.120407	[AMBP]
GO:0051017	actin filament bundle assembly	11	1	0.101519311	0.120407	[DPYSL3]
GO:0007626	locomotory behavior	11	1	0.101519311	0.120407	[APP]
GO:0031647	regulation of protein stability	11	1	0.101519311	0.120407	[SOX4]
GO:0007229	integrin-mediated signaling pathway	57	2	0.105556982	0.124164	[ITGB5, PTK2]
GO:0034446	substrate adhesion-dependent cell spreading	12	1	0.110221947	0.128803	[ANTXR1]
GO:0006284	base-excision repair	12	1	0.110221947	0.128803	[HMGA1]
GO:0051898	negative regulation of protein kinase B signaling cascade	12	1	0.110221947	0.128803	[HYAL2]
GO:0048041	focal adhesion assembly	12	1	0.110221947	0.128803	[ACTN1]
GO:0043968	histone H2A acetylation	12	1	0.110221947	0.128803	[MEAF6]
GO:0032008	positive regulation of TOR signaling cascade	12	1	0.110221947	0.128803	[RRAGB]
GO:0060021	palate development	12	1	0.110221947	0.128803	[FOXF2]
GO:0018298	protein-chromophore linkage	13	1	0.118840487	0.137747	[AMBP]
GO:0034613	cellular protein localization	13	1	0.118840487	0.137747	[RRAGB]
GO:0002062	chondrocyte differentiation	13	1	0.118840487	0.137747	[BMP4]
GO:0007249	I-kappaB kinase/NF-kappaB cascade	14	1	0.127375741	0.146688	[TBK1]
GO:0006829	zinc ion transport	14	1	0.127375741	0.146688	[SLC39A7]
GO:0030163	protein catabolic process	14	1	0.127375741	0.146688	[CLN5]
GO:0001570	vasculogenesis	14	1	0.127375741	0.146688	[WT1]
GO:0017148	negative regulation of translation	14	1	0.127375741	0.146688	[WT1]
GO:0045860	positive regulation of protein kinase activity	15	1	0.135828512	0.154306	[PTK2]
GO:0055072	iron ion homeostasis	15	1	0.135828512	0.154306	[SLC22A17]
GO:0050680	negative regulation of epithelial cell proliferation	15	1	0.135828512	0.154306	[SFRP1]
GO:0050679	positive regulation of epithelial cell proliferation	15	1	0.135828512	0.154306	[SFRP1]
GO:0071560	cellular response to transforming growth factor beta stimulus	15	1	0.135828512	0.154306	[SFRP1]

GO:0006952	defense response	15	1	0.135828512	0.154306	[MLF2]
GO:0007031	peroxisome organization	15	1	0.135828512	0.154306	[PEX19]
GO:0008544	epidermis development	15	1	0.135828512	0.154306	[PLOD1]
GO:2001244	positive regulation of intrinsic apoptotic signaling pathway	15	1	0.135828512	0.154306	[CLU]
GO:0010595	positive regulation of endothelial cell migration	15	1	0.135828512	0.154306	[BMP4]
GO:0030198	extracellular matrix organization	15	1	0.135828512	0.154306	[APP]
GO:0007565	female pregnancy	15	1	0.135828512	0.154306	[AMBP]
GO:0050896	response to stimulus	589	2	0.137053021	0.154104	[PDCL, TGFBI]
GO:0051297	centrosome organization	16	1	0.144199594	0.161377	[CHD3]
GO:0045666	positive regulation of neuron differentiation	16	1	0.144199594	0.161377	[HOXD3]
GO:0007264	small GTPase mediated signal transduction	16	1	0.144199594	0.161377	[RAB18]
GO:0045931	positive regulation of mitotic cell cycle	16	1	0.144199594	0.161377	[APP]
GO:0051262	protein tetramerization	16	1	0.144199594	0.161377	[NUDT21]
GO:0030154	cell differentiation	484	8	0.151345164	0.168581	[CSRP2, DZIP1, FAM82A2, GAP43, INA, LECT1, NFATC4, TRIM54]
GO:0030855	epithelial cell differentiation	17	1	0.152489777	0.169065	[WT1]
GO:0043967	histone H4 acetylation	17	1	0.152489777	0.169065	[LEF1]
GO:0042476	odontogenesis	17	1	0.152489777	0.169065	[BMP4]
GO:0032496	response to lipopolysaccharide	17	1	0.152489777	0.169065	[SNCA]
GO:0001938	positive regulation of endothelial cell proliferation	17	1	0.152489777	0.169065	[BMP4]
GO:0045739	positive regulation of DNA repair	18	1	0.160699839	0.177067	[UIMC1]
GO:0000187	activation of MAPK activity	18	1	0.160699839	0.177067	[SHC1]
GO:0010976	positive regulation of neuron projection development	18	1	0.160699839	0.177067	[DPYSL3]
GO:0006874	cellular calcium ion homeostasis	19	1	0.168830553	0.185169	[PTH]
GO:0007160	cell-matrix adhesion	19	1	0.168830553	0.185169	[HOXD3]
GO:0007224	smoothened signaling pathway	19	1	0.168830553	0.185169	[BMP4]
GO:0019370	leukotriene biosynthetic process	20	1	0.176882685	0.193258	[GGT7]
GO:0016338	calcium-independent cell-cell adhesion	20	1	0.176882685	0.193258	[CLDN11]
GO:0006364	rRNA processing	80	2	0.181664171	0.198028	[EXOSC1, PA2G4]
GO:0000165	MAPK cascade	21	1	0.184856991	0.200895	[SHC1]
GO:0031572	G2/M transition DNA damage checkpoint	21	1	0.184856991	0.200895	[UIMC1]
GO:0031175	neuron projection development	21	1	0.184856991	0.200895	[APP]
GO:0048013	ephrin receptor signaling pathway	22	1	0.192754224	0.208683	[PTK2]

GO:0042254	ribosome biogenesis	22	1	0.192754224	0.208683	[RSL24D1]
GO:0045944	positive regulation of transcription from RNA polymerase II promoter	158	3	0.197915731	0.213785	[GATA6, RNF10, SOX4]
GO:0060070	canonical Wnt receptor signaling pathway	23	1	0.200575124	0.21633	[LEF1]
GO:0016055	Wnt receptor signaling pathway	87	2	0.206204425	0.222066	[AES, CD24]
GO:0051301	cell division	285	5	0.206463029	0.22201	[CDK19, SEPT11, UBE2C, UBE2S, VPS4B]
GO:0051865	protein autoubiquitination	25	1	0.215990869	0.231558	[TRIM13]
GO:0001501	skeletal system development	25	1	0.215990869	0.231558	[SOX4]
GO:0006338	chromatin remodeling	25	1	0.215990869	0.231558	[CBX3]
GO:0008610	lipid biosynthetic process	26	1	0.223587164	0.238805	[ACLY]
GO:0030433	ER-associated protein catabolic process	26	1	0.223587164	0.238805	[TRIM13]
GO:0010212	response to ionizing radiation	27	1	0.23111003	0.246104	[UIMC1]
GO:0043966	histone H3 acetylation	27	1	0.23111003	0.246104	[LEF1]
GO:0060271	cilium morphogenesis	28	1	0.238560174	0.253282	[ASAP1]
GO:0007411	axon guidance	28	1	0.238560174	0.253282	[PTK2]
GO:0007601	visual perception	97	2	0.241784814	0.256134	[PDCL, TGFBI]
GO:0030097	hemopoiesis	29	1	0.245938299	0.259763	[BCL11A]
GO:0016525	negative regulation of angiogenesis	29	1	0.245938299	0.259763	[THBS1]
GO:0007417	central nervous system development	29	1	0.245938299	0.259763	[PNMA1]
GO:0009790	embryo development	31	1	0.26048126	0.274312	[PTK2]
GO:0006950	response to stress	104	2	0.266856434	0.280612	[FAM129A, SERPINH1]
GO:0006919	activation of cysteine-type endopeptidase activity involved in apoptotic process	32	1	0.267647466	0.28103	[MTCH1]
GO:0007268	synaptic transmission	33	1	0.274744391	0.288058	[CARTPT]
GO:0009968	negative regulation of signal transduction	36	1	0.295626127	0.30927	[RGS4]
GO:0006914	autophagy	36	1	0.295626127	0.30927	[TM9SF1]
GO:0007507	heart development	37	1	0.302452545	0.315718	[WT1]
GO:0030521	androgen receptor signaling pathway	38	1	0.309212959	0.322304	[NCOA2]
GO:0001558	regulation of cell growth	39	1	0.315908006	0.328802	[MORF4L2]
GO:0006511	ubiquitin-dependent protein catabolic process	40	1	0.322538316	0.334971	[UBE2C]
GO:0006979	response to oxidative stress	40	1	0.322538316	0.334971	[PRDX6]
GO:0051781	positive regulation of cell division	41	1	0.329104514	0.341046	[PTN]
GO:0007267	cell-cell signaling	42	1	0.335607218	0.347281	[FADS1]
GO:0030036	actin cytoskeleton organization	43	1	0.342047042	0.353432	[EPB41]

GO:0008654	phospholipid biosynthetic process	44	1	0.34842459	0.359502	[FADS1]
GO:0006954	inflammatory response	128	2	0.352134297	0.362805	[PRKCZ, TBK1]
GO:0035556	intracellular signal transduction	48	1	0.373323971	0.383806	[MAGI3]
GO:0006351	transcription, DNA-dependent	48	1	0.373323971	0.383806	[HOXD3]
GO:0006631	fatty acid metabolic process	50	1	0.385415363	0.395099	[HSD17B4]
GO:0010923	negative regulation of phosphatase activity	50	1	0.385415363	0.395099	[MKI67IP]
GO:0016337	cell-cell adhesion	51	1	0.391373494	0.400345	[CD24]
GO:0007050	cell cycle arrest	52	1	0.397273997	0.405799	[GAS2]
GO:0006397	mRNA processing	144	2	0.407124384	0.415267	[DDX1, NUDT21]
GO:0007126	meiosis	60	1	0.442469072	0.450353	[PPP2CA]
GO:0030030	cell projection organization	60	1	0.442469072	0.450353	[ASAP1]
GO:0006935	chemotaxis	71	1	0.499142999	0.506593	[CXCL12]
GO:0007010	cytoskeleton organization	71	1	0.499142999	0.506593	[FMNL3]
GO:0006310	DNA recombination	73	1	0.508811826	0.515307	[RPA1]
GO:0006814	sodium ion transport	82	1	0.550068098	0.556301	[ATP1A2]
GO:0006916	anti-apoptosis	84	1	0.558756008	0.564288	[SNCA]
GO:0008283	cell proliferation	90	1	0.583828318	0.588776	[HDGFRP3]
GO:0006260	DNA replication	93	1	0.595826277	0.600028	[NAE1]
GO:0006813	potassium ion transport	102	1	0.629788868	0.633337	[ATP1A2]
GO:0008150	biological_process	588	4	0.669294047	0.672118	[ABHD11, FLRT2, FXYD6, MFAP1]
GO:0006955	immune response	220	1	0.728573407	0.73062	[SAMHD1]
GO:0007067	mitosis	159	1	1	1	[UBE2C]
GO:0006281	DNA repair	147	1	1	1	[MORF4L2]

Table S5-3. GO annotation analysis of the module eigengene in the common module between LT and LDE (postnatal_turquoise vs turquoise).

GO ID	GO Name	Total Coun	Diff Coun	P Value	FDR	Gene
GO:0008380	RNA splicing	150	4	7.5458E-05	0.007772147	[DDX20, DDX47, PPP2CA, SYNCRIP]
GO:0000184	nuclear-transcribed mRNA catabolic process, nonsense-mediated d	27	2	0.00074014	0.038117424	[EIF3E, PAN2]
GO:0006772	thiamine metabolic process	1	1	0.00148101	0.043583976	[THTPA]
GO:0000462	maturation of SSU-rRNA from tricistronic rRNA transcript	1	1	0.00148101	0.043583976	[NOP14]
GO:0006120	mitochondrial electron transport, NADH to ubiquinone	42	2	0.00178976	0.036869128	[NDUFB9, NDUFS4]
GO:0006810	transport	395	4	0.00283905	0.048737058	[MTCH2, NDUFB9, NDUFS4, TXN]
GO:0000480	endonucleolytic cleavage in 5'-ETS of tricistronic rRNA transcript	2	1	0.00295986	0.030486539	[NOP14]
GO:0051967	negative regulation of synaptic transmission, glutamatergic	2	1	0.00295986	0.030486539	[ATAD1]
GO:0032963	collagen metabolic process	2	1	0.00295986	0.030486539	[TNXB]
GO:0051083	'de novo' cotranslational protein folding	2	1	0.00295986	0.030486539	[DNAJC2]
GO:0000447	endonucleolytic cleavage in ITS1 to separate SSU-rRNA from 5.8S rRNA and LSU-rRNA from tricistronic rRNA transcript (SSU-rRNA, 5.8S rRNA, LSU-rRNA)	2	1	0.00295986	0.030486539	[NOP14]
GO:0000472	endonucleolytic cleavage to generate mature 5'-end of SSU-rRNA from (SSU-rRNA, 5.8S rRNA, LSU-rRNA)	2	1	0.00295986	0.030486539	[NOP14]
GO:0001672	regulation of chromatin assembly or disassembly	2	1	0.00295986	0.030486539	[TLK1]
GO:0048251	elastic fiber assembly	3	1	0.00443655	0.028560295	[TNXB]
GO:0042518	negative regulation of tyrosine phosphorylation of Stat3 protein	3	1	0.00443655	0.028560295	[PPP2CA]
GO:0019932	second-messenger-mediated signaling	3	1	0.00443655	0.028560295	[PPP2CA]
GO:0071168	protein localization to chromatin	3	1	0.00443655	0.028560295	[RAD21]
GO:0033158	regulation of protein import into nucleus, translocation	3	1	0.00443655	0.028560295	[TXN]
GO:0006646	phosphatidylethanolamine biosynthetic process	4	1	0.00591109	0.03122268	[CHKB]
GO:0002092	positive regulation of receptor internalization	4	1	0.00591109	0.03122268	[ATAD1]
GO:0006364	rRNA processing	80	2	0.0063354	0.031073642	[DDX47, NOP14]
GO:0007498	mesoderm development	5	1	0.00738348	0.033065143	[MESDC2]
GO:0007612	learning	5	1	0.00738348	0.033065143	[ATAD1]
GO:0070934	CRD-mediated mRNA stabilization	5	1	0.00738348	0.033065143	[SYNCRIP]
GO:0051591	response to cAMP	6	1	0.00885372	0.035074352	[NDUFS4]
GO:0010508	positive regulation of autophagy	6	1	0.00885372	0.035074352	[MTDH]
GO:0045947	negative regulation of translational initiation	6	1	0.00885372	0.035074352	[EIF3E]
GO:0000188	inactivation of MAPK activity	7	1	0.01032182	0.036660247	[PPP2CA]
GO:0006275	regulation of DNA replication	7	1	0.01032182	0.036660247	[PPP2CA]

GO:0030111	regulation of Wnt receptor signaling pathway	7	1	0.01032182	0.036660247	[PPP2CA]
GO:0006091	generation of precursor metabolites and energy	8	1	0.01178777	0.037358174	[THTPA]
GO:0006396	RNA processing	8	1	0.01178777	0.037358174	[SETX]
GO:0019933	cAMP-mediated signaling	8	1	0.01178777	0.037358174	[NDUFS4]
GO:0007613	memory	8	1	0.01178777	0.037358174	[ATAD1]
GO:0006915	apoptotic process	321	3	0.01212148	0.035671798	[CCAR1, DDX47, RAD21]
GO:0009615	response to virus	114	2	0.01250379	0.035774742	[BECN1, PSMA2]
GO:0048146	positive regulation of fibroblast proliferation	9	1	0.01325159	0.035918786	[NDUFS4]
GO:0006672	ceramide metabolic process	9	1	0.01325159	0.035918786	[PPP2CA]
GO:0042149	cellular response to glucose starvation	9	1	0.01325159	0.035918786	[HSPA5]
GO:0043066	negative regulation of apoptotic process	118	2	0.01335005	0.034376373	[HSPA5, MTDH]
GO:0043388	positive regulation of DNA binding	10	1	0.01471327	0.036082553	[TXN]
GO:0009314	response to radiation	10	1	0.01471327	0.036082553	[TXN]
GO:0016311	dephosphorylation	10	1	0.01471327	0.036082553	[THTPA]
GO:0006412	translation	130	2	0.01603325	0.037532384	[MRPL35, MRPS7]
GO:0042274	ribosomal small subunit biogenesis	11	1	0.01617283	0.035823677	[NOP14]
GO:0045333	cellular respiration	11	1	0.01617283	0.035823677	[NDUFS4]
GO:0031663	lipopolysaccharide-mediated signaling pathway	11	1	0.01617283	0.035823677	[MTDH]
GO:0032981	mitochondrial respiratory chain complex I assembly	11	1	0.01617283	0.035823677	[NDUFS4]
GO:0001516	prostaglandin biosynthetic process	12	1	0.01763025	0.036685161	[PTGES2]
GO:0010033	response to organic substance	12	1	0.01763025	0.036685161	[PPP2CA]
GO:0006446	regulation of translational initiation	13	1	0.01908554	0.038545313	[EIF3E]
GO:0015031	protein transport	383	3	0.01933365	0.038295507	[CHMP2A, NXT1, TMED7]
GO:0006397	mRNA processing	144	2	0.01942888	0.037758004	[DDX20, DDX47]
GO:0072593	reactive oxygen species metabolic process	14	1	0.02053872	0.039175701	[NDUFS4]
GO:0007049	cell cycle	405	3	0.02235448	0.04186385	[BECN1, CCAR1, TLK1]
GO:0045595	regulation of cell differentiation	16	1	0.02343871	0.04311048	[PPP2CA]
GO:0006486	protein glycosylation	17	1	0.02488553	0.044968591	[ALG3]
GO:0030155	regulation of cell adhesion	18	1	0.02633024	0.046758881	[PPP2CA]
GO:0051897	positive regulation of protein kinase B signaling cascade	20	1	0.02921335	0.050999577	[MTDH]
GO:0043154	negative regulation of cysteine-type endopeptidase activity involved in apoptotic process	25	1	0.03638439	0.061943673	[HSPA5]
GO:0006338	chromatin remodeling	25	1	0.03638439	0.061943673	[CHRAC1]
GO:0006413	translational initiation	26	1	0.03781233	0.062314715	[EIF3E]

GO:0008610	lipid biosynthetic process	26	1	0.03781233	0.062314715	[ACLY]
GO:0016568	chromatin modification	221	2	0.04267313	0.06867707	[DNAJC2, TLK1]
GO:0006302	double-strand break repair	33	1	0.04774971	0.075087336	[SETX]
GO:0007420	brain development	33	1	0.04774971	0.075087336	[NDUFS4]
GO:0045766	positive regulation of angiogenesis	34	1	0.04916107	0.075575967	[MTDH]
GO:0006986	response to unfolded protein	35	1	0.05057036	0.07659922	[HSPA4]
GO:0006470	protein dephosphorylation	37	1	0.05338278	0.079687334	[PPP2CA]
GO:0000910	cytokinesis	41	1	0.05898303	0.085566927	[BECN1]
GO:0022900	electron transport chain	41	1	0.05898303	0.085566927	[TXN]
GO:0006886	intracellular protein transport	41	1	0.05898303	0.085566927	[TLK1]
GO:0006633	fatty acid biosynthetic process	42	1	0.06037798	0.085190856	[PTGES2]
GO:0030036	actin cytoskeleton organization	43	1	0.06177091	0.085978424	[TNXB]
GO:0008654	phospholipid biosynthetic process	44	1	0.06316179	0.086742198	[CHKB]
GO:0051092	positive regulation of NF-kappaB transcription factor activity	45	1	0.06455065	0.087483122	[MTDH]
GO:0035556	intracellular signal transduction	48	1	0.06870507	0.091904191	[TLK1]
GO:0040008	regulation of growth	51	1	0.07284133	0.096187909	[PPP2CA]
GO:0007059	chromosome segregation	55	1	0.07832821	0.102124121	[RAD21]
GO:0030308	negative regulation of cell growth	58	1	0.08242238	0.106118809	[PPP2CA]
GO:0007126	meiosis	60	1	0.08514187	0.108266818	[PPP2CA]
GO:0055114	oxidation-reduction process	65	1	0.09190592	0.115442805	[TXN]
GO:0006457	protein folding	68	1	0.09594069	0.119058934	[MESDC2]
GO:0006935	chemotaxis	71	1	0.09995781	0.12256732	[CYR61]
GO:0006917	induction of apoptosis	79	1	0.11058441	0.134002286	[PPP2CA]
GO:0043065	positive regulation of apoptotic process	83	1	0.11585129	0.138752122	[DDX20]
GO:0006916	anti-apoptosis	84	1	0.1171632	0.138710454	[HSPA5]
GO:0016055	Wnt receptor signaling pathway	87	1	0.12108745	0.141727355	[MESDC2]
GO:0006974	response to DNA damage stimulus	93	1	0.12888449	0.14915846	[TLK1]
GO:0000398	nuclear mRNA splicing, via spliceosome	98	1	0.13532998	0.154877638	[SYNCRIP]
GO:0006950	response to stress	104	1	0.14300259	0.161860076	[HSPA4]
GO:0043123	positive regulation of I-kappaB kinase/NF-kappaB cascade	105	1	0.14427483	0.161525078	[MTDH]
GO:0000122	negative regulation of transcription from RNA polymerase II prom	117	1	0.15939736	0.176536864	[MTDH]
GO:0008219	cell death	136	1	0.18280488	0.200307471	[SETX]
GO:0006468	protein phosphorylation	185	1	0.24025052	0.260482147	[TLK1]
GO:0051301	cell division	285	1	0.34542821	0.370615683	[RAD21]
GO:0044419	interspecies interaction between organisms	315	1	0.37408641	0.397225771	[SYNCRIP]

GO:0045893	positive regulation of transcription, DNA-dependent	334	1	0.39159382	0.411573096	[DNAJC2]
GO:0006355	regulation of transcription, DNA-dependent	1254	3	0.43585304	0.453463266	[CCAR1, PPP2CA, TXN]
GO:0007155	cell adhesion	420	1	0.46499409	0.478943911	[TNXB]
GO:0006508	proteolysis	476	1	0.50802683	0.518086763	[PSMC1]
GO:0008150	biological_process	588	1	0.58412208	0.589848769	[TMEM128]
GO:0007275	multicellular organismal development	822	1	1	1	[SPRY1]

Table S6-1. Module eigengene and its evolutionary rates in six prenatal highly LDE-specific modules (LDE_blue).

pig_Genebank	pig_Gene	LDE Module	kTotal	kWithin	eigencor r	eigenpval	mean Expr	LTModule	Ka/Ks
NM_001044529.1	CAPN10	blue	280.497	135.4669	0.96413	0.0081128	20.23	magenta	--
NM_001044548.1	NTN1	blue	297.083	140.6561	0.97295	0.0053183	17.35	darkred	0
AK234245.1	CCDC23	blue	267.942	129.7176	0.95374	0.011862	48	turquoise	99
AK234254.1	AP2S1	blue	308.667	151.1919	0.98946	0.0012964	54.51	turquoise	99
AK230890.1	UBA3	blue	330.278	138.118	0.96988	0.0062458	99.04	turquoise	--
AK230981.1	TNFAIP6	blue	308.079	145.7172	0.9809	0.0031593	21.86	yellow	--
AK240575.1	C11orf58	blue	320.719	147.6085	0.98383	0.0024632	58.49	navy	99
AK240580.1	RCHY1	blue	343.356	145.0638	0.97938	0.0035429	13.96	blue	99
AK231033.1	MAPKSP1	blue	326.343	113.4681	0.92358	0.0250674	22.61	darkred	0
AK234825.1	SPCS2	blue	343.685	152.1234	0.99081	0.0010568	38.48	blue	0
AK235171.1	DDAH2	blue	221.799	100.9559	0.89788	0.0385701	43.53	turquoise	--
AK238491.1	PPP1R1B	blue	243.547	117.3866	0.93195	0.0210901	5.426	turquoise	99
AK235662.1	RAB23	blue	319.808	146.6854	0.98195	0.0029033	18.62	yellow	--
AK238657.1	FOXK2	blue	321.142	151.1261	0.98937	0.0013144	28.95	white	--
AK235782.1	RABAC1	blue	349.715	140.8495	0.97324	0.0052335	85.13	turquoise	--
AK238784.1	DNAJB6	blue	236.637	103.4066	0.90389	0.0352451	12.99	turquoise	99
AK239167.1	THAP3	blue	338.091	150.7948	0.98837	0.0015034	7.646	blue	99
AK239234.1	TPRG1L	blue	224.773	106.8526	0.91093	0.031479	14.56	blue	99
AK230559.1	PICALM	blue	301.813	132.3918	0.9594	0.0097589	120.3	turquoise	99
AK236416.1	TYMS	blue	254.927	122.3157	0.94112	0.0169966	57.48	green	--
AK239611.1	HNRPDL	blue	318.649	107.5245	0.91147	0.0311998	159.9	blue	--
AK236676.1	TGFB2	blue	332.113	99.89093	0.89508	0.040147	21.6	blue	99
AK239756.1	TERF2IP	blue	287.172	141.1846	0.97324	0.005234	89.18	lightgreen	0
AK239935.1	NUTF2	blue	328.478	150.9149	0.9889	0.0014023	36.94	blue	0
AK233427.1	RTN3	blue	188.623	83.2891	0.85607	0.0641123	24.14	turquoise	99
AK233469.1	AARSD1	blue	296.166	146.4449	0.98216	0.0028537	19.05	blue	--
AK237149.1	NCAPG2	blue	314.411	123.7218	0.9433	0.0160705	11.88	turquoise	--
AK233574.1	SPATS2L	blue	214.099	93.60382	0.8807	0.0485686	98.26	pink	0
AK233803.1	RNASEH2C	blue	310.38	144.9235	0.98006	0.0033711	41.64	turquoise	99
AK234140.1	EIF4E2	blue	331.211	105.4832	0.90744	0.0333301	24.8	violet	--
AB292846.1	NME1-NME	blue	344.419	114.7129	0.92573	0.0240248	28.85	turquoise	--
AJ416019.1	FABP3	blue	325.715	144.4368	0.97843	0.0037918	27.3	darkturquoise	99
NM_001123122.1	H2AFZ	blue	322.071	125.8388	0.94674	0.0146351	105.1	turquoise	99
XM_001927037.1	RRAGA	blue	301.63	146.4925	0.98203	0.0028832	65	turquoise	99
XM_001924578.1	CHST14	blue	330.676	145.5987	0.98125	0.0030726	57.34	turquoise	0
XM_001928960.1	PEA15	blue	323.343	151.5247	0.98964	0.0012637	106.9	turquoise	99
XM_001928648.1	KIAA0907	blue	326.947	142.8341	0.97724	0.004108	26.67	pink	0
XM_001929572.1	C1orf77	blue	340.871	135.4556	0.96544	0.0076728	17.21	yellow	0
XM_001929641.1	PSMB4	blue	308.423	150.2932	0.98807	0.0015613	75.73	blue	0
XM_001927218.1	KRT4	blue	209.505	73.97136	0.83017	0.0818405	2.41	salmon	0.074
XM_001929413.1	PTGES3	blue	235.989	94.16578	0.88202	0.0477763	63.24	darkmagenta	99
XM_001925133.1	ACIN1	blue	345.976	149.2027	0.98579	0.0020283	66.9	yellowgreen	0.461
XM_001926121.1	MRPL52	blue	331.809	145.3388	0.97994	0.0034006	22.85	darkturquoise	--
XM_001926913.1	POLR1D	blue	345.111	146.6876	0.98223	0.002836	81.41	darkred	0

XM_001926988.1	POLR1D	blue	267.851	125.894	0.94797	0.0141336	25.01	blue	0
XM_001924615.1	PDLIM1	blue	278.429	115.0891	0.92797	0.0229525	24.78	grey	0
XM_001925382.1	MRPL43	blue	322.59	130.1057	0.9556	0.0111557	23.18	darkred	--
XM_001928772.1	DDX21	blue	339.685	141.6328	0.97386	0.0050539	126.1	midnightblue	0.132
XM_001924660.1	C10orf72	blue	284.507	134.4953	0.96252	0.0086614	53.8	turquoise	0
XM_001924736.1	C10orf104	blue	283.923	140.7768	0.97264	0.0054105	81.02	white	0
XM_001925978.1	EWSR1	blue	274.99	127.6404	0.95132	0.0127982	52.77	turquoise	--
XM_001924074.1	HSBP1	blue	339.963	155.8771	0.99605	0.0002982	88.76	blue	--
XM_001928335.1	TUBA4A	blue	310.232	131.4381	0.9569	0.0106708	32.06	red	0
XM_001927332.1	AGA	blue	270.558	122.7094	0.94143	0.0168637	11.31	blue	0.413
XM_001927451.1		blue	240.137	108.7466	0.91518	0.0292713	8.412	skyblue	0
NM_214144.1	PRDX5	blue	278.414	137.3509	0.96689	0.0071964	69.4	turquoise	0
NM_214309.1	NUDT2	blue	307.642	151.3094	0.98956	0.0012783	33.84	lightcyan	0.395
NM_001001539.1	AKR1B1	blue	332.87	126.6476	0.94817	0.0140532	94.88	yellow	0
AY609657.1	PPID	blue	303.112	150.6835	0.98846	0.0014862	25.11	navy	99
AY609726.1	ARF6	blue	326.049	138.8151	0.96958	0.0063408	10.13	navy	0
AY609827.1	HSPBP1	blue	311.192	133.7068	0.96249	0.0086728	13.93	turquoise	99
AY610021.1	SNRPD1	blue	281.297	131.3991	0.9575	0.0104516	54.35	magenta	0
AY610024.1	PGP	blue	276.815	116.5524	0.92967	0.0221496	11.88	tan	99
NM_213748.1	TNNT1	blue	332.665	137.172	0.96777	0.0069134	1382	sienna3	99
NM_001012406.1	MYOG	blue	314.885	141.8359	0.97492	0.0047504	14.59	blue	99
CF178259		blue	270.797	132.2562	0.95891	0.0099374	57.22	lightgreen	--
CF360790		blue	325.482	132.504	0.95928	0.0098037	6.828	midnightblue	--
CF364773		blue	286.516	141.4599	0.97449	0.0048717	8.364	blue	--
BX667513		blue	301.207	150.2633	0.98769	0.0016362	51.88	turquoise	--
BX666658		blue	340.92	140.0014	0.97123	0.0058333	4.58	blue	--
BX666730		blue	253.182	118.4767	0.93429	0.0200195	32.11	blue	--
BX668263		blue	343.94	122.1753	0.9405	0.0172678	21.8	lightyellow	--
BX669236		blue	307.339	145.7387	0.98108	0.0031161	23.45	lightyellow	--
BX672167		blue	274.263	133.093	0.95963	0.0096791	11.86	violet	--
BX674271		blue	298.124	133.4459	0.9618	0.0089104	53.28	white	--
BX675385		blue	340.049	157.4072	0.99837	7.88E-05	5.376	darkred	--
BP158662		blue	273.009	133.0443	0.96007	0.0095189	26.13	navy	0.085
BP433436		blue	260.207	128.7692	0.9526	0.0122996	135.2	turquoise	--
BP142915		blue	260.457	118.7093	0.93596	0.0192654	19.98	turquoise	--
BP463816		blue	352.591	143.6254	0.97731	0.0040895	30.79	turquoise	0.163
CK449659		blue	258.23	117.5698	0.93366	0.0203041	17.99	turquoise	--
CK450104		blue	287.676	143.1703	0.97644	0.0043256	22.77	blue	--
CK451664		blue	257.159	116.625	0.93234	0.0209116	71.43	darkred	--
CK452762		blue	327.502	156.252	0.99678	0.0002195	38.73	blue	--
CK459703		blue	329.771	154.0763	0.9936	0.0006144	27.86	white	--
CK460762		blue	293.62	125.445	0.94625	0.0148378	22.32	turquoise	--
CK461229		blue	335.523	145.5552	0.98039	0.0032862	54.08	magenta	--
BX914521		blue	293.867	133.9085	0.96228	0.0087458	14.8	blue	99
BX915597		blue	248.704	118.9629	0.93405	0.02013	13.35	lightcyan	99
BX916011		blue	315.422	142.9286	0.97657	0.0042909	64.29	turquoise	--
BX917211		blue	311.929	148.6979	0.98586	0.0020136	46.51	blue	--
BX919071		blue	239.629	109.7798	0.9164	0.0286491	36.14	tan	--

BX919211	blue	329.794	155.2244	0.99524	0.0003934	15.08	lightgreen	--
BX920888	blue	292.433	110.3281	0.9171	0.0282962	31.72	turquoise	--
BX922414	blue	325.243	153.0529	0.99202	0.000855	11.77	blue	--
BX922807	blue	301.222	134.3475	0.96331	0.0083892	10.58	orange	--
BX923265	blue	318.137	145.0549	0.97987	0.0034188	8.062	navy	--
BX924131	blue	337.634	146.1673	0.98129	0.0030625	15.04	turquoise	--
BX924576	blue	321.911	139.0942	0.97038	0.0060908	18.27	magenta	--
CN154367	blue	308.411	148.4219	0.98487	0.0022279	4.618	blue	--
CN156524	blue	297.145	140.6479	0.97309	0.0052784	15.48	brown	--
CN156601	blue	331.756	157.6964	0.99886	4.59E-05	53.67	violet	--
CN156870	blue	326.613	154.4969	0.99404	0.0005517	55.41	turquoise	--
CN157291	blue	341.716	133.9114	0.96134	0.0090708	12.83	turquoise	--
CN158157	blue	347.714	149.9549	0.98747	0.0016805	174.1	darkgreen	--
CN158745	blue	340.543	154.9054	0.99489	0.0004382	192	blue	--
CN158757	blue	205.698	89.77913	0.8729	0.0533456	2.486	lightyellow	--
CO992371	blue	341.333	150.5814	0.98824	0.0015273	33.18	turquoise	--
CJ009488	blue	300.466	140.0243	0.97269	0.0053941	2.868	tan	--
CJ009624	blue	314.634	117.3299	0.93091	0.0215735	8.656	blue	--
CJ011598	blue	329.757	135.1172	0.96307	0.0084731	11.81	turquoise	--
CJ022587	blue	236.523	108.3223	0.9137	0.0300343	66.4	blue	--
DN100786	blue	332.969	151.6703	0.98991	0.0012144	12.63	turquoise	--
DN101634	blue	345.595	150.953	0.98887	0.0014064	46.72	blue	--
DN102168	blue	290.774	133.0497	0.96158	0.0089877	100.8	orange	--
DN106372	blue	305.446	129.6726	0.95498	0.0113874	17.37	turquoise	--
DN106398	blue	352.367	131.4102	0.95675	0.010728	37.58	turquoise	--
DN107111	blue	300.643	142.8556	0.97629	0.0043676	15.61	blue	--
DN109035	blue	341.792	141.7273	0.97484	0.0047717	82.27	blue	--
DN109946	blue	300.083	144.3081	0.97857	0.0037524	8.882	blue	--
DN112403	blue	315.849	131.1941	0.95672	0.0107396	25.29	blue	0
DN117304	blue	287.422	133.7347	0.96223	0.0087615	10.76	navy	--
DN120090	blue	318.367	146.5824	0.98217	0.0028505	18.91	darkred	--
DN121729	blue	306.529	126.5726	0.95048	0.0131285	9.292	red	--
DN124126	blue	300.142	137.4762	0.9679	0.0068699	37.05	blue	--
DN124198	blue	241.199	110.5873	0.91885	0.0274091	44.59	violet	--
DN125831	blue	271.627	113.047	0.92361	0.0250503	168.7	salmon	--
DN126201	blue	304.934	151.705	0.98991	0.0012147	90.63	lightgreen	--
BW957936	blue	266.575	114.3659	0.92629	0.0237554	2.774	blue	99
BW981299	blue	257.726	125.9921	0.9476	0.0142872	20.82	turquoise	--
AJ954193	blue	306.497	143.4441	0.97754	0.0040263	20.79	turquoise	--
AJ956714	blue	155.165	68.95951	0.81583	0.0922086	32.99	turquoise	--
AJ958305	blue	280.657	136.2701	0.96588	0.0075262	15.11	turquoise	--
AJ959876	blue	308.596	142.4706	0.97643	0.0043275	2.25	grey	--
AJ944988	blue	311.494	143.9463	0.97792	0.0039242	7.596	blue	--
DV227720	blue	271.512	130.1132	0.95509	0.0113465	18.72	turquoise	--
DV229300	blue	326.727	137.2064	0.96657	0.0073017	7.908	lightyellow	--
DV899239	blue	307.179	137.0201	0.96785	0.0068869	10.59	brown	--
DV902328	blue	286.487	131.7763	0.95862	0.0100409	2.074	orange	--
DR120938	blue	318.497	155.8417	0.99607	0.000296	28.78	darkred	--

CV876076		blue	348.545	149.7934	0.98676	0.0018247	3.164	magenta	--	
DY405629		blue	257.222	123.6141	0.94283	0.0162657	2.456	lightcyan	--	
DY410479		blue	330.781	157.8598	0.99907	3.41E-05	30.84	pink	--	
DY428559		blue	307.567	142.1329	0.97521	0.004667	9.29	magenta	--	
DY437892		blue	332.949	150.9344	0.9888	0.0014198	9.516	turquoise	--	
DY437999		blue	340.269	143.9776	0.97762	0.0040047	7.656	brown	--	
EB684343		blue	352.518	128.945	0.95251	0.0123363	83.21	darkred	--	
DB812814		blue	216.443	95.52792	0.88571	0.0455796	57.31	turquoise	--	
DB786707		blue	312.65	140.9349	0.97347	0.0051654	11.5	darkred	--	
AK236389	EPHX1	blue	339.052	150.6831	0.98839	0.0014999	13.33	darkred		0.192
AK232389	CTSB	blue	317.11	150.0496	0.98779	0.0016173	19.67	blue		0
ES446945		blue	249.649	104.0821	0.90455	0.0348888	7.082	turquoise	--	
ES446999		blue	269.991	130.7782	0.95642	0.0108486	22.48	blue	--	
ES447481		blue	207.293	86.13472	0.86298	0.0596174	50.81	turquoise	--	
EV984595		blue	343.733	150.4633	0.98821	0.0015334	170.7	turquoise	--	
EW074840		blue	322.539	156.754	0.9975	0.0001501	9.574	blue	--	
EW085190		blue	339.64	154.5347	0.99437	0.0005065	10.9	magenta	--	
EW087471		blue	315.855	153.2879	0.99244	0.0007877	7.464	blue	--	
EW154385		blue	279.747	138.8962	0.97013	0.006169	22.85	violet	--	
EW172087		blue	325.226	133.0039	0.96163	0.0089694	12.27	darkturquoise	--	
EW177532		blue	284.244	141.7259	0.97444	0.0048863	7.248	turquoise	--	
EW188932		blue	311.149	151.7868	0.99024	0.0011563	19.25	blue	--	
EW194624		blue	328.226	156.6312	0.99735	0.0001639	4.446	darkturquoise	--	
EW212556		blue	262.951	119.7422	0.93697	0.018813	21.33	blue	--	
EW238451		blue	345.904	140.0062	0.97243	0.0054739	27.8	turquoise	--	
EW244903		blue	318.4	155.3584	0.99547	0.0003656	15.31	blue	--	
EW249649		blue	299.504	142.6089	0.97617	0.0043988	7.128	turquoise	--	
EW281140		blue	300.666	131.3314	0.95669	0.0107505	753.8	darkred	--	
EW290234		blue	337.225	139.161	0.97118	0.0058465	161.3	turquoise	--	
EW291794		blue	314.102	145.0407	0.97992	0.0034049	29.41	yellow	--	
EW299345		blue	290.048	144.8304	0.97931	0.0035626	22.92	lightyellow	--	
EW301848		blue	337.612	124.7086	0.94725	0.0144262	81.84	turquoise	--	
EW385918		blue	346.586	114.7421	0.92624	0.0237786	12.25	turquoise	--	
EW407282		blue	312.696	146.0845	0.98161	0.002985	28.04	blue	--	
EW410373		blue	336.956	120.4213	0.93676	0.0189069	32.22	darkturquoise	--	
EW429561		blue	272.441	131.5127	0.95689	0.0106759	58.8	turquoise	--	
EW501346		blue	271.268	110.7609	0.9183	0.0276882	50.39	darkgreen	--	
EW511183		blue	346.031	125.8745	0.94702	0.0145207	6.818	yellow	--	
EW525048		blue	326.162	147.5469	0.98378	0.0024727	8.286	lightgreen	--	
EW528311		blue	330.778	135.3827	0.96403	0.0081466	16.15	midnightblue	--	
EW568747		blue	288.949	139.2027	0.97052	0.0060502	60.25	turquoise	--	
EW569367		blue	280.308	140.0472	0.97173	0.0056829	7.862	magenta	--	
EW570939		blue	201.074	88.29748	0.86829	0.0562336	209.3	turquoise	--	
EW581399		blue	302.552	118.9631	0.93422	0.0200517	3.638	red	--	
EW588718		blue	325.05	148.7798	0.98582	0.0020219	29.97	yellow		0
EW603701		blue	318.981	139.7935	0.97156	0.0057338	7.144	blue	--	
EW664676		blue	260.438	125.7814	0.94712	0.0144822	16.19	blue	--	
EW675014		blue	244.129	112.5156	0.9226	0.0255489	665.2	skyblue	--	

AW231897	blue	202.012	75.56107	0.83393	0.0791859	12.67	turquoise	--
BE014011	blue	286.858	123.3537	0.94269	0.0163256	6.196	magenta	--
BE233131	blue	274.993	113.6182	0.92497	0.0243921	14.36	yellow	--
BE233738	blue	305.019	148.6656	0.98544	0.0021052	27.49	magenta	--
BF703133	blue	344.896	135.1057	0.96461	0.007948	11.39	brown	--
BQ604210	blue	278.173	136.2975	0.96542	0.0076786	41.13	turquoise	--

Table S6-2. Module eigengene and its evolutionary rates in six prenatal highly LDE-specific modules (LDE_midnightblue).

pig_Genebank	pig_Gene	LDEModule	kTotal	kWithin	eigen corr	eigenpval	meanExpr	LTModule	Ka/Ks
XM_001925341.1	ASB2	midnightblue	226.629	36.619494	1	0.000228	12.226	white	0
XM_001926116.1	ASPH	midnightblue	258.652	33.5788	0.98	0.003922	49.27	white	--
XM_001929000.1	BAG3	midnightblue	253.613	32.80669	0.97	0.005177	51.128	white	0.152
XM_001927918.1	CASQ2	midnightblue	202.833	34.726675	0.99	0.00207	18.838	darkorange	0.065
AK235923.1	COQ9	midnightblue	244.291	36.81882	1	0.000128	57.084	brown	0
AY610225.1	COX7B	midnightblue	229.005	34.958883	0.99	0.001693	24.796	turquoise	99
NM_001048187.1	DLK1	midnightblue	244.763	36.201023	0.99	0.0005	6.374	brown	--
AK234424.1	DUSP26	midnightblue	219.047	36.575409	1	0.000253	63.068	greenyellow	0
AY609402.1	EIF1AY	midnightblue	282.17	28.201445	0.94	0.017226	23.018	brown	--
AK232843.1	GPD1	midnightblue	164.045	28.786626	0.95	0.014776	3.086	purple	0
AK232127.1	MLF1	midnightblue	223.54	32.394577	0.97	0.005761	24.136	greenyellow	0
NM_214374.2	MYL1	midnightblue	192.446	33.507286	0.98	0.003923	102.71	brown	--
AK231783.1	PDLIM3	midnightblue	209.632	32.248573	0.97	0.005938	68.094	purple	0
NM_001099932.1	PGK1	midnightblue	153.916	27.806131	0.94	0.018236	324.41	royalblue	0
NM_214213.1	PLN	midnightblue	223.006	36.446624	1	0.000326	9.668	purple	--
XM_001926917.1	PLXND1	midnightblue	193.887	33.7288	0.98	0.003588	108.79	purple	0.03
AK236976.1	PPP1R3B	midnightblue	264.717	30.850524	0.96	0.009563	16.28	purple	0.087
AK237414.1	PTP4A3	midnightblue	229.841	36.508053	1	0.000275	101.38	purple	--
X95846.1	RYR3	midnightblue	215.051	36.23146	0.99	0.000501	5.86	white	--
AK233419.1	SLC25A4	midnightblue	251.573	35.606469	0.99	0.001052	232.88	purple	0
AK233342.1	SLC6A12	midnightblue	203.247	33.49299	0.98	0.003918	6.814	purple	0
XM_001928874.1	SNTA1	midnightblue	236.144	34.870391	0.99	0.00189	12.122	purple	0
AK238056.1		midnightblue	240.157	37.116479	1	1.32E-05	19.294	purple	--
BX666448		midnightblue	251.825	36.191731	0.99	0.000545	524.67	red	--
BX666997		midnightblue	269.139	34.156695	0.98	0.003028	54.326	greenyellow	--
CK453765		midnightblue	226.423	36.768213	1	0.000143	31.66	turquoise	--
CN162187		midnightblue	224.594	35.259588	0.99	0.001417	27.744	purple	0
CJ014614		midnightblue	209.59	32.30199	0.97	0.005847	9.656	yellowgreen	--
DN101101		midnightblue	235.448	34.82037	0.99	0.001861	4.228	brown	--
DQ000205		midnightblue	249.938	36.431547	1	0.000357	5.794	navy	--
DY405382		midnightblue	228.855	35.096389	0.99	0.001617	112.3	purple	0
DB805355		midnightblue	249.305	36.531184	1	0.000288	14.2	purple	0
EV869069		midnightblue	232.442	36.227108	0.99	0.000503	87.732	greenyellow	--
EV983935		midnightblue	186.883	31.011915	0.96	0.008546	35.706	black	99
EW362700		midnightblue	260.679	32.420281	0.97	0.00592	93.876	purple	--
EW367525		midnightblue	267.434	29.922137	0.95	0.012045	69.936	greenyellow	--
EW472347		midnightblue	264.752	34.026331	0.98	0.003175	214.67	brown	--
EW569850		midnightblue	192.068	33.899298	0.98	0.003321	4.588	turquoise	99
EW590143		midnightblue	206.927	34.04328	0.98	0.002898	132.25	navy	99
EW595007		midnightblue	245.1	36.625046	1	0.00022	29.784	darkgreen	--
EW621997		midnightblue	149.731	27.038637	0.93	0.020894	28.236	navy	--
BI182553		midnightblue	235.401	35.415038	0.99	0.001257	30.484	white	--

Table S6-3. Module eigengene and its evolutionary rates in six prenatal highly LDE-specific modules (LDE_only_turquoise).

pig_Genebank	pig_Gene	LDE Module	kTotal	kWithin	eigencorr	eigenpval	meanExpr	Ka/Ks
AK230970.1	TAX1BP1	turquoise	1427.613	1427.123445	0.9748765	0.0047622	22.304	0
AK234734.1	PDCD6IP	turquoise	1555.892	1555.448701	0.9972045	0.0001774	99.81	0.2083
AK239273.1	TMEM106C	turquoise	1398.026	1397.511331	0.9727305	0.0053835	37.442	99
AK232798.1	FERMT2	turquoise	1440.917	1440.438605	0.9761009	0.0044192	7.156	--
AK237060.1	HNRNPC	turquoise	58.80791	22.08050441	0.1072513	0.8637057	15.64	--
AK237199.1	ATG4C	turquoise	145.959	45.99747442	0.1201419	0.8473993	3.008	--
NM_001097478.1	RPL14	turquoise	744.9099	740.8437241	0.8215075	0.0880603	526.736	--
NM_001122994.1	OAZ1	turquoise	1570.161	1569.767161	0.9995428	1.17E-05	69.454	99
X15073.1	ATP2A2	turquoise	1545.012	1544.626916	0.9952284	0.0003954	16.296	99
XM_001927571.1	PSIP1	turquoise	1094.761	1092.758528	0.9155102	0.0291044	98.244	0
XM_001926689.1	FAM189B	turquoise	1556.876	1556.468304	0.9968311	0.000214	1.79	99
XM_001924386.1	RARG	turquoise	1512.314	1511.93024	0.9898898	0.0012185	4.304	99
XM_001928732.1	ETFA	turquoise	948.9302	947.4394731	0.8822176	0.0476572	65.554	0.1286
XM_001924270.1	MAP4	turquoise	1516.785	1516.414475	0.9904094	0.0011258	7.634	--
XM_001924322.1	CHRNA1	turquoise	1340.321	1339.43201	0.9641764	0.0080955	19.902	0
XM_001924519.1	SRPX2	turquoise	291.5491	269.8480856	0.5719635	0.313681	20.188	0
AY610221.1	LRRC39	turquoise	561.853	556.5224047	0.769481	0.1281668	5.638	0
AY610266.1	FIS1	turquoise	1300.01	1299.296823	0.9500702	0.0132921	23.414	--
CF787005		turquoise	1015.585	1014.312342	0.8903647	0.0428534	5.582	--
BX673255		turquoise	1570.404	1570.019856	0.9994487	1.55E-05	1.596	--
BX674220		turquoise	1550.915	1550.517176	0.9968036	0.0002168	1.888	--
BP438382		turquoise	1530.25	1529.844444	0.9928751	0.0007212	2.484	0
BP464871		turquoise	1539.886	1539.518314	0.9939764	0.0005607	6.89	--
CN155806		turquoise	1119.332	1118.227009	0.9258588	0.0239626	6.44	--
CN157667		turquoise	1383.542	1383.043229	0.9669938	0.0071625	8.498	--
CN158497		turquoise	1542.276	1541.906429	0.9941934	0.0005307	4.824	--
CN164041		turquoise	1553.556	1553.182884	0.9965278	0.0002455	3.07	99
CO988234		turquoise	1517.708	1517.33643	0.9902104	0.001161	4.914	--
CJ015366		turquoise	1488.102	1487.694732	0.984956	0.00221	1.938	--
CJ000197		turquoise	1567.338	1566.945397	0.9989097	4.32E-05	14.152	--
CJ039155		turquoise	1414.531	1413.710978	0.974584	0.0048454	3.914	99
DN110566		turquoise	1550.354	1549.896149	0.9976456	0.0001371	12.738	--
DN116453		turquoise	1562.809	1562.400229	0.998281	8.55E-05	1.936	99
DN116548		turquoise	1557.426	1557.019782	0.9972723	0.0001709	18.958	--
DN123890		turquoise	1511.379	1510.964162	0.9911326	0.001001	2.416	--
DN125647		turquoise	341.7533	337.5968891	0.6667547	0.2190185	28.14	--
DN134303		turquoise	1366.245	1365.673502	0.9650152	0.0078138	2.618	--
BW975440		turquoise	1303.985	1302.695245	0.9572686	0.0105354	55.31	--
BW974451		turquoise	1367.794	1367.256001	0.9654576	0.0076665	5.056	--
AJ953116		turquoise	1518.292	1517.912805	0.991504	0.0009389	36.452	--
AJ956957		turquoise	1433.818	1433.336354	0.9775332	0.0040288	12.776	--
AJ963372		turquoise	1544.36	1543.917339	0.9967336	0.000224	9.754	--
DY431973		turquoise	1555.52	1555.064007	0.9980488	0.0001034	2.912	--
DB803190		turquoise	326.4111	298.7960394	0.5840532	0.30112	7.886	--

DB805308	turquoise	1517.783	1517.386105	0.9895927	0.0012725	15.212	--	
DT322380	turquoise	1475.709	1475.281009	0.982373	0.0028019	14.786		99
EV893970	turquoise	1121.396	1120.361801	0.9163036	0.0286989	9.78	--	
EW054351	turquoise	1508.531	1508.091003	0.9901383	0.0011739	3.964	--	
EW102856	turquoise	1515.392	1515.005452	0.9894589	0.0012971	22.536	--	
EW242994	turquoise	1536.614	1536.116018	0.9948648	0.0004414	13.178	--	
EW524057	turquoise	1498.231	1497.624765	0.988803	0.0014199	2.834	--	
AW360262	turquoise	1539.154	1538.780411	0.9944244	0.0004994	3.456	--	
BI183745	turquoise	1489.484	1488.931447	0.9870269	0.0017703	5.206	--	
BQ603067	turquoise	1499.108	1498.691662	0.988443	0.0014888	4.768	--	

Table S6-4. Module eigengene and its evolutionary rates in six prenatal highly LDE-specific modules (LDE_pink).

pig_Geneba	pig_Gene	LDEModule	kTotal	kWithin	eigencorr	eigenpval	meanExpr	Ka/Ks
NM_00104	CACNG1	pink	222.66791	45.605862	0.9884354	0.0014903	41.894	0
NM_00104	NRAP	pink	194.85748	44.968614	0.9851952	0.0021576	21.004	0.117
AK237778.1		pink	172.50745	41.768128	0.9679263	0.0068621	9.164	--
AK231853.	NFKBIB	pink	124.83246	30.550397	0.8949716	0.0402097	32.994	0
AK231968.	TCEA3	pink	246.21013	46.617622	0.9916924	0.0009078	44.57	--
AK232023.	PDLIM5	pink	264.13779	41.856132	0.9631439	0.0084466	89.204	--
NM_00100	FGL2	pink	230.89593	46.898997	0.9933682	0.0006477	32.382	0
NM_00109	ADSSL1	pink	271.07946	38.297432	0.941298	0.016922	37.162	--
NM_00112	AMPD1	pink	248.04252	44.712664	0.9808188	0.0031798	170.728	0.0238
NM_00112	TPM2	pink	282.17639	39.126512	0.9472233	0.0144387	4239.712	99
XM_00192	NDUFB9	pink	183.62776	38.79323	0.9527904	0.0122259	136.806	0
XM_00192	ZFP36L1	pink	284.22755	38.836413	0.9443871	0.0156114	788.818	0
XM_00192	APOBEC2	pink	265.18388	45.311153	0.9826421	0.0027381	270.856	--
XM_00192	C6orf62	pink	243.83199	44.116579	0.9802339	0.003326	403.634	99
XM_00192	MTMR14	pink	218.95052	46.520639	0.9922867	0.0008123	38.65	99
XM_00192	ACTN2	pink	248.98847	45.260147	0.9847626	0.0022527	652.286	0
XM_00192	ANK3	pink	200.03273	43.540449	0.9787203	0.0037144	72.954	0.1356
XM_00192	FBRSL1	pink	115.66024	24.424066	0.8500171	0.0681359	75.99	--
XM_00192	IGSF1	pink	210.95867	39.050031	0.9519906	0.0125363	62.096	0.7994
NM_00113	AGPAT2	pink	206.94134	45.040136	0.9852372	0.0021484	24.728	0
NM_00113	PGAM2	pink	276.60952	41.334226	0.9610084	0.0091883	300.808	0
NM_21383	FOLR1	pink	119.02351	28.849725	0.8822425	0.0476423	5.528	0.0744
NM_00100	DES	pink	264.71716	45.041149	0.9816667	0.0029716	2674.704	0
AY610291.	EIF4EBP1	pink	281.18637	39.985893	0.9512544	0.0128244	131.136	--
NM_00100	TNNT3	pink	232.16986	42.290755	0.9718416	0.0056481	1892.508	--
AY870324.	GYS1	pink	152.77554	36.463063	0.9375128	0.0185741	37.738	--
CF181210		pink	198.62551	46.158613	0.9909417	0.0010335	23.09	--
BX667493		pink	182.46358	43.025365	0.9752058	0.0046691	24.436	0
BX667667		pink	211.06293	36.010534	0.9328793	0.0206632	43.826	--
BX670880		pink	283.68867	38.889801	0.9449823	0.0153628	218.222	--
BX672685		pink	206.46187	42.131161	0.9718883	0.0056341	95.858	--
CK458789		pink	157.98382	37.848699	0.9471256	0.0144786	89.55	--
BX916224		pink	267.76899	40.175477	0.9557881	0.0110851	283.934	--
BX921034		pink	127.6131	31.298959	0.9034285	0.0354989	21.084	--
BX922054		pink	254.77606	45.56607	0.9846185	0.0022847	418.774	--
BX923137		pink	197.867	45.403096	0.9872477	0.0017254	42.48	--
CN157030		pink	148.37004	29.116446	0.8955294	0.0398932	41.954	0.4429
CN161458		pink	127.96985	31.111066	0.9017634	0.0364115	64.14	--
DN110939		pink	212.77254	46.898754	0.9941936	0.0005307	25.204	--
DN112227		pink	146.10712	32.491468	0.9148102	0.0294637	4.1	--
DN114469		pink	262.88262	45.501226	0.9836685	0.0024992	90.372	--
DN120355		pink	230.87838	45.363356	0.9869353	0.0017891	23.27	--
DN124256		pink	226.76652	47.875655	0.998756	5.27E-05	182.242	--
AJ964843		pink	263.47247	43.699468	0.9742527	0.0049402	61.292	--
CX064206		pink	243.22545	47.68931	0.9964545	0.0002533	86.912	--

DY433232	pink	238.68022	47.744941	0.9972109	0.0001767	247.776	--
DB784140	pink	213.76258	40.857727	0.9650424	0.0078047	31.14	--
DT329597	pink	234.04303	40.714296	0.9597588	0.0096316	13.93	0
DT332656	pink	173.78998	42.02167	0.9699696	0.0062188	22.234	--
EH412677	pink	266.91299	43.679849	0.9739402	0.0050302	43.444	--
EW075087	pink	146.70996	36.457469	0.9367171	0.0189277	18.472	--
EW238443	pink	223.99784	44.949748	0.9835656	0.0025229	57.318	--
EW308219	pink	264.86842	41.646575	0.9643413	0.0080398	100.3	--
EW319834	pink	193.88088	45.148426	0.9859224	0.0020008	5.624	--
EW491431	pink	227.27677	47.492738	0.9965225	0.000246	46.79	--
EW570202	pink	213.70909	45.601859	0.9880302	0.0015692	12.708	--
BM190349	pink	281.46571	38.185941	0.9424685	0.0164214	3.952	99
BM190547	pink	125.0224	31.066569	0.8981527	0.0384158	9.926	--
BM190692	pink	221.68114	46.597777	0.9931654	0.0006776	12.692	--

Table S6-5. Module eigengene and its evolutionary rates in six prenatal highly LDE-specific modules (LDE_red).

pig_Genebank	pig_Gene	LDE Module	kTotal	kWithin	eigencorr	eigenpval	meanExpr	LTModule	Ka/Ks
AK230715.1	MALAT1	red	265.89	45.0549	0.9596704	0.0096633	77.63	greenyellow	--
AK237425.1	AOC3	red	248.18	50.9308	0.9896226	0.001267	32.564	greenyellow	--
AK237684.1	PRKAG2	red	197.19	50.5139	0.9896888	0.0012549	61.634	royalblue	0
AK232067.1	MRPL45	red	244.37	51.5768	0.9926557	0.0007547	66.752	turquoise	--
AK232780.1	USP2	red	188.46	48.0601	0.9788494	0.0036807	9.784	red	0
AK239575.1	ALAS1	red	148.78	34.3256	0.9061264	0.0340359	19.912	turquoise	0
AK236674.1	WBSCR22	red	167.69	43.5627	0.9569751	0.0106437	46.508	turquoise	0
AK233231.1	NDUFS8	red	256.59	45.3366	0.9629729	0.0085052	152.784	navy	--
EU650276.1	SLC16A3	red	246.02	43.6677	0.9550598	0.0113589	8.976	red	0
NM_001128433.1	SLC2A4	red	181.42	47.8391	0.977446	0.0040522	26.03	purple	--
XM_001929195.1	PPP6C	red	254.38	49.7468	0.9833139	0.0025809	201.388	magenta	0
XM_001924679.1	AK3	red	204.15	50.0207	0.9878033	0.001614	26.202	turquoise	--
XM_001926680.1	FBXL4	red	132.71	32.9779	0.8955426	0.0398858	24.248	navy	0.135
XM_001928966.1	CASQ1	red	178.14	47.2082	0.9743814	0.0049033	43.322	purple	0
XM_001928924.1	RCSD1	red	232.3	50.1842	0.9879546	0.0015841	22.378	darkgreen	0.2
XM_001929272.1	COQ10A	red	210.71	49.7557	0.9865427	0.0018702	19.288	navy	--
XM_001927338.1	IDH3A	red	223.67	48.452	0.9802371	0.0033252	65.578	navy	0.176
XM_001929023.1	LPAR6	red	131.7	34.5756	0.9034058	0.0355112	56.558	greenyellow	0
XM_001927832.1	FAM190B	red	141.74	36.3026	0.9150798	0.0293251	13.25	yellow	99
XM_001929063.1	MAFB	red	144.09	39.1359	0.9309103	0.0215725	35.88	purple	--
XM_001925452.1	ATG4A	red	233	48.2948	0.9787616	0.0037036	8.698	brown	--
NM_001130733.1	ADSL	red	181.99	43.8187	0.9594474	0.0097432	84.19	brown	0.135
NM_001142669.1	TEAD1	red	153.5	37.939	0.9268099	0.0235064	7.152	brown	0
NM_213980.1	UGP2	red	155.8	39.9964	0.937392	0.0186276	45.766	magenta	0
NM_213986.1	MYOC	red	190.84	48.687	0.9822451	0.0028324	1.516	greenyellow	0.14
NM_214356.1	RGS5	red	235.39	52.5421	0.9974947	0.0001505	52.11	white	0
AY610012.1	C1GALT1C	red	262.93	45.1388	0.9600264	0.0095361	32.22	magenta	99
AY610394.1	NDUFS4	red	247.77	50.0307	0.985071	0.0021848	133.056	royalblue	99
NM_001006592.1	MYLPF	red	257.63	49.4895	0.9828621	0.0026863	4419.722	brown	0
NM_214136.1	MYH2	red	232.59	50.4905	0.9899538	0.0012069	18.784	purple	0
DQ058292		red	260.47	49.17	0.9809435	0.0031489	43.262	navy	--
CF360662		red	200.03	49.5241	0.9860683	0.0019698	6.648	royalblue	--
BX671489		red	272.55	45.2479	0.9604959	0.0093693	19.284	brown	--
BX673719		red	264.11	48.03	0.9746379	0.0048301	3.078	yellow	0
BX675832		red	269.84	44.9408	0.9581903	0.0101978	86.93	turquoise	--
CK449832		red	205.45	50.4924	0.9902067	0.0011617	44.278	violet	--
CK453720		red	154.99	37.7393	0.9267153	0.0235516	30.29	darkgreen	--
BX916417		red	232.05	51.6131	0.9941974	0.0005301	18.122	yellowgreen	--
BX918145		red	262.26	45.6448	0.9623204	0.0087302	85.238	red	--
BX920496		red	201.4	50.5198	0.9900022	0.0011982	2.12	purple	0
BX921928		red	149.17	40.9268	0.9408825	0.0171009	2.756	turquoise	--
BX924065		red	206.22	51.0515	0.9923812	0.0007974	25.374	purple	--
CN156385		red	258.96	47.2962	0.9714054	0.0057795	14.52	royalblue	--
DN104974		red	274.46	44.179	0.9542187	0.0116778	7.358	lightyellow	--
DN120811		red	164.36	42.7975	0.9528989	0.012184	4.118	turquoise	--
AJ947734		red	131.09	35.1865	0.9085637	0.0327313	13.95	purple	--

AJ954681		red	143.36	35.2945	0.911971	0.0309352	115.48	turquoise	--
AJ956925		red	279.26	41.8389	0.941503	0.0168339	22.08	blue	--
AJ961806		red	205.86	51.7625	0.9952051	0.0003983	10.256	purple	--
AJ962029		red	243.29	51.7878	0.994299	0.0005163	5.24	yellow	--
AJ945191		red	252.35	45.3417	0.961515	0.0090105	1.052	turquoise	--
DV901297		red	176.14	46.6156	0.9720362	0.0055898	5.2	yellowgreen	--
DY437561		red	257.44	49.5023	0.9828446	0.0026904	55.22	violet	--
DQ459258	SLN	red	184.13	40.4421	0.9416626	0.0167655	34.582	brown	--
DT331413		red	202.52	48.4116	0.980266	0.0033179	11.87	purple	--
EW088403		red	266.65	47.168	0.9702832	0.006122	94.282	purple	--
EW168087		red	218.14	46.9651	0.9735341	0.0051479	114.234	darkmagenta	--
EW189742		red	162.54	42.5166	0.9513595	0.0127831	3.632	turquoise	--
EW190405		red	261.98	44.8398	0.9581683	0.0102058	7.8	turquoise	--
EW269870		red	254.92	46.5423	0.9686493	0.0066321	136.212	brown	--
EW633584		red	192.96	47.3713	0.976259	0.0043755	35.772	purple	0.725
FL593097		red	181.97	46.5952	0.9723157	0.0055065	2.452	brown	--
BF190620		red	228.2	50.6379	0.9893035	0.0013259	3.036	purple	--
BI185477		red	244.47	42.084	0.9456684	0.0150779	5.082	darkred	0

Table S6-6. Module eigengene and its evolutionary rates in six prenatal highly LDE-specific modules (LDE_tan).

pig_Genebank	pig_Gene	Module	kTotal	kWithin	eigencorr	eigenpval	meanExpr	LTModule	Ka/Ks
AK237695.1	NOP14	tan	256.704	34.712864	0.9606412	0.0093178	37.326	brown	0.3033
AK235136.1	DECR2	tan	301.228	37.229474	0.9761727	0.0043993	16.876	turquoise	--
AK232459.1	GLTSCR2	tan	305.382	40.130737	0.9932518	0.0006648	61.57	red	0
AK238836.1	EIF4H	tan	329.71	35.426133	0.9637589	0.0082368	206.558	turquoise	--
AK239538.1	MEF2D	tan	340.833	35.40572	0.9633816	0.0083653	50.872	brown	--
AK233129.1	CCDC92	tan	245.781	32.301366	0.9462038	0.0148568	2.76	turquoise	0.3261
AK236617.1	G3BP2	tan	320.625	38.44607	0.9831321	0.0026231	31.808	darkred	99
NM_001113439.1	SFRS18	tan	340.698	35.762954	0.9669432	0.0071789	113.09	magenta	99
XM_001928798.1	OSR2	tan	272.344	37.347542	0.9772498	0.0041051	17.398	violet	0
XM_001925424.1	ANO6	tan	285.183	37.82541	0.9821233	0.0028615	31.746	magenta	--
XM_001928309.1	TBC1D7	tan	224.06	28.218216	0.9159158	0.0288968	17.818	blue	0
XM_001928393.1	GBF1	tan	241.249	30.711903	0.9332043	0.0205143	72.36	navy	0
XM_001928504.1	GATC	tan	308.616	36.342307	0.970885	0.0059375	13.16	turquoise	99
AY803094.1	SS18L1	tan	299.259	39.508533	0.9915737	0.0009273	22.096	turquoise	--
BX667249		tan	266.467	35.292984	0.9675986	0.0069672	10.552	blue	--
BX675350		tan	341.677	36.050758	0.9698412	0.0062586	13.634	lightgreen	99
BP173173		tan	231.795	25.693418	0.8979996	0.0385015	9.668	turquoise	99
BP440506		tan	292.17	35.409501	0.966546	0.0073083	10.772	brown	--
CK451418		tan	235.717	33.320479	0.9525894	0.0123036	55.092	turquoise	0
BX917182		tan	260.996	33.163519	0.9544042	0.0116072	26.948	turquoise	--
BX920877		tan	318.285	38.001197	0.9814942	0.0030136	18.202	navy	--
BX924453		tan	313.259	39.693992	0.9910016	0.0010233	18.038	darkgrey	--
BX924845		tan	320.63	40.531341	0.995816	0.0003247	37.976	turquoise	--
BX925452		tan	305.154	36.610243	0.9728426	0.0053504	21.764	darkturquoi	--
CN166292		tan	331.626	39.239134	0.9881784	0.0015402	4.236	yellow	--
DN115175		tan	312.215	40.733281	0.9971939	0.0001784	21.012	turquoise	--
DN116048		tan	324.516	38.172695	0.9812528	0.0030727	12.306	turquoise	0
BW973127		tan	350.525	35.158875	0.9632083	0.0084246	19.344	navy	99
AJ956510		tan	292.638	31.296257	0.9383351	0.0182109	87.396	tan	--
DY418504		tan	335.092	37.396201	0.9772215	0.0041127	32.828	darkturquoi	--
DY420049		tan	172.239	14.01251	0.7785082	0.1208893	3.74	turquoise	--
DY420192		tan	284.549	38.646306	0.9866842	0.0018408	30.22	black	--
DT324183		tan	322.58	38.415849	0.9828397	0.0026915	17.484	brown	0
EV871347		tan	278.719	34.853129	0.9631486	0.008445	112.226	grey60	--
EV947055		tan	326.859	38.644252	0.9839354	0.0024383	29.692	darkturquoi	--
EV956580		tan	332.485	40.56677	0.9955279	0.0003588	176.924	red	--
EW084349		tan	335.479	37.984471	0.9803337	0.0033009	14.036	navy	--
EW089029		tan	305.675	35.429961	0.9648077	0.0078831	39.494	turquoise	--
EW117408		tan	338.469	39.721314	0.9905984	0.0010928	8.198	lightcyan	--
EW129629		tan	293.715	38.185359	0.9837138	0.0024889	254.708	pink	--
EW137538		tan	238.379	32.476433	0.9494463	0.0135408	2607.526	blue	--
EW143863		tan	223.158	30.134707	0.9333086	0.0204666	49.726	turquoise	--
EW146447		tan	341.213	37.531213	0.9768459	0.0042147	44.178	blue	--

EW155191	tan	180.585	23.528778	0.8746816	0.0522419	4.768		--	
EW203772	tan	266.682	36.981542	0.9765682	0.0042905	53.896	turquoise		99
EW275601	tan	310.855	36.74347	0.9739089	0.0050393	53.082	magenta	--	
EW495920	tan	282.84	37.887738	0.9822768	0.0028248	945.06	brown		0
EW537674	tan	326.636	37.536406	0.9786353	0.0037367	165.472	pink	--	
EW577076	tan	329.428	36.679733	0.9718428	0.0056477	21.732	magenta		99
BE235135	tan	245.376	34.45583	0.9612675	0.0090972	61.35	turquoise	--	

Table S7-1. Module eigengene and its evolutionary rates in five prenatal highly LT-specific modules (LT_blue).

pig_Genebank	pig_Gene	LDE Module	kTotal	kWithin	eigencorr	eigenpval	mean Expr	LT Module	Ka/Ks
AK235372.1	3-Mar	turquoise	1312.5425	1311.6717	0.9616276	0.0089711	9.836	blue	0
AK233469.1	AARSD1	blue	296.1659	146.44487	0.9821559	0.0028537	19.05	blue	--
AK230529.1	ABI1	turquoise	994.59779	993.04999	0.8904415	0.0428089	19.208	blue	99
XM_001927332.1	AGA	blue	270.55781	122.70937	0.9414336	0.0168637	11.314	blue	0.4132
NM_001128455.1	AKIRIN1	turquoise	239.03453	198.84308	0.4712025	0.4230531	4.874	blue	--
AK230942	ANXA1	brown	253.15986	128.30247	0.9963858	0.0002607	5.85	blue	--
AK236330.1	ANXA4	greenyellow	209.80999	22.733135	0.9800312	0.0033772	5.752	blue	0.1941
XM_001924669.1	APH1A	brown	214.48815	118.56353	0.9813154	0.0030573	14.186	blue	--
AK233864.1	ARF5	turquoise	575.13309	567.49577	0.7621256	0.1341893	20.074	blue	99
XM_001926997.1	ARHGAP2!	turquoise	1360.6769	1359.9309	0.9688881	0.0065567	47.808	blue	0.3509
AY610050.1	ARL2BP	turquoise	271.07202	236.79743	0.5201895	0.3688927	42.84	blue	0
AK236628	ATIC	brown	283.15852	105.16021	0.9368393	0.0188732	5.838	blue	0.0443
AK236671.1	ATP11A	brown	290.98955	113.96896	0.9581885	0.0101985	117.404	blue	--
XM_001925710.1	BCAS2	turquoise	132.7937	69.037234	0.2691289	0.6615172	39.102	blue	0
AY609731.1	C6orf115	darkgreen	281.14403	22.694525	0.9865345	0.0018719	3.066	blue	99
XM_001925294.1	C9orf119	turquoise	172.61649	116.90623	0.3640762	0.5468985	18.762	blue	99
XM_001924741.1	CAB39	turquoise	108.79724	71.442373	0.3118515	0.6094715	19.492	blue	99
AK240220.1	CCDC124	turquoise	1543.317	1542.9268	0.994314	0.0005142	30.804	blue	99
AY609717.1	CCT4	brown	228.74223	120.75371	0.9820405	0.0028814	4.718	blue	0
AK230614	CD36	royalblue	150.11832	21.678685	0.9521029	0.0124926	2.418	blue	0.3146
XM_001928774.1	COQ10B	royalblue	205.08773	19.955998	0.9364927	0.0190278	37.174	blue	0.3627
XM_001927225.1	COX17	greenyellow	213.18719	22.351233	0.9912274	0.000985	36.108	blue	--
XM_001925488.1	CTNNBL1	lightcyan	150.31109	6.9939101	0.2877061	0.6387995	25.408	blue	--
AK232389	CTSB	blue	317.11005	150.04959	0.9877864	0.0016173	19.674	blue	0
AK230569.1	DHX15	purple	212.20666	36.275485	0.9978889	0.0001164	93.67	blue	0
XM_001928813.1	DNTTIP1	turquoise	133.86209	66.046359	0.2499808	0.6850614	24.616	blue	0
AK231026.1	EIF1AX	brown	213.82197	107.37512	0.9520912	0.0124971	6.882	blue	--
AF288822.1	EIF4G2	turquoise	335.34784	323.70307	0.6443971	0.2405131	41.59	blue	--
AY610090.1	ELOF1	brown	223.60538	126.97765	0.9959382	0.0003106	7.032	blue	--
AK234725.1	GNB1	brown	213.46397	94.772289	0.9216535	0.0260131	58.926	blue	--
NM_214332.1	GNB2L1	brown	305.0296	113.7983	0.9564947	0.0108216	505.572	blue	--
AK233386.1	GOLGB1	turquoise	480.53255	473.7115	0.7330659	0.1587585	24.874	blue	--
AK239611.1	HNRPDL	blue	318.64904	107.52453	0.9114652	0.0311998	159.888	blue	--
XM_001924074.1	HSBP1	blue	339.96288	155.8771	0.9960467	0.0002982	88.756	blue	--
XM_001929570.1	HSP90AB1	purple	185.47966	32.523844	0.9739796	0.0050188	646.798	blue	0
AK240329.1	HSPA4	brown	172.17955	82.67021	0.8968537	0.0391452	152.368	blue	99
AY610185.1	HSPB11	brown	255.26781	122.12031	0.980627	0.0032275	4.382	blue	99
XM_001924658.1	IGF2BP2	brown	134.71629	65.440898	0.845385	0.0712645	6.894	blue	0
AK234213.1	KIAA1191	turquoise	1500.808	1500.2591	0.9902978	0.0011455	7.072	blue	--
XM_001928810.1	KIAA1279	magenta	328.07076	42.969476	0.9846293	0.0022823	103.902	blue	0
AK234175.1	KIAA1467	greenyellow	201.69581	22.910172	0.9867411	0.0018291	8.424	blue	--
AK236730.1	LMCD1	brown	199.63297	99.966213	0.9428554	0.0162569	19.694	blue	0
AY705918.1	LOC10028(	lightgreen	85.761236	7.5093144	0.3958671	0.5094592	2.688	blue	--

AK237193.1	LOC10028:	brown	229.01771	105.08113	0.9479778	0.0141318	4.17	blue	--
AK233777.1	LOC10029:	turquoise	159.30509	121.11779	0.4177854	0.4839665	58.212	blue	--
AJ236938.1	LUC7L3	greenyellow	114.84568	14.065964	0.8925433	0.0415965	40.006	blue	--
XM_001927000.1	LUM	turquoise	1114.6794	1112.4033	0.9195729	0.0270475	79.74	blue	0.1529
XM_001925291.1	MAPK6	turquoise	1521.6024	1521.2206	0.9905804	0.0010959	14.392	blue	0
AK239435.1	MAT2A	greenyellow	182.77511	22.63111	0.9865951	0.0018593	4.008	blue	99
AY609565.1	MRPL1	brown	204.96949	111.60865	0.9618835	0.0088819	6.312	blue	--
AK234571.1	MRPL12	lightcyan	257.01276	24.717403	0.9854678	0.0020984	30.044	blue	--
AK234033.1	MRPL53	brown	180.12864	108.70009	0.9570442	0.0106181	20.712	blue	--
NM_001012406.1	MYOG	blue	314.88537	141.83586	0.9749181	0.0047504	14.59	blue	99
AK234198.1	N6AMT2	yellow	303.25171	62.456991	0.8921234	0.0418378	84.382	blue	--
AY609572.1	NDUFC2	turquoise	105.8614	42.034712	0.1591403	0.7982348	91.158	blue	--
AK239935.1	NUTF2	blue	328.47811	150.91491	0.9888958	0.0014023	36.944	blue	0
NM_214342.1	OXSR1	turquoise	1503.8578	1503.2354	0.9911009	0.0010064	29.856	blue	0
AK239095.1	PARN	turquoise	239.90292	220.29251	0.5609892	0.3251957	13.562	blue	99
AK235462.1	PCBP1	brown	342.85458	79.267558	0.8572618	0.0633315	167.73	blue	99
NM_213901.1	PCCB	brown	185.81711	108.7788	0.9565789	0.0107904	4.214	blue	0
XM_001924505.1	PDGFRL	brown	214.67194	115.0713	0.9695998	0.0063337	11.59	blue	--
XM_001926198.1	PFDN5	brown	280.46488	102.3997	0.9300918	0.0219543	42.51	blue	0
NM_001097521.2	PGRMC2	brown	175.72024	106.69426	0.9561174	0.0109621	50.094	blue	99
AK235272.1	PJA1	brown	164.44628	89.471691	0.9028537	0.0358131	8.468	blue	0.2562
XM_001927189.1	PLEKHA1	turquoise	1514.3382	1513.9397	0.9898733	0.0012214	10.972	blue	99
XM_001926988.1	POLR1D	blue	267.85059	125.89397	0.9479735	0.0141336	25.006	blue	0
NM_214184.2	PPP1CB	turquoise	244.9903	229.76347	0.5699165	0.3158209	269.218	blue	99
XM_001929641.1	PSMB4	blue	308.4231	150.29317	0.9880706	0.0015613	75.73	blue	0
AK239491	PSMB4	turquoise	1570.6606	1570.2558	0.9996554	7.68E-06	3.76	blue	--
XM_001927985.1	PSMC1	turquoise	336.18992	321.40988	0.641716	0.2431273	42.514	blue	0
XM_001927062.1	PTBP2	turquoise	1532.3002	1531.8177	0.9949281	0.0004333	18.184	blue	--
AY609534.1	RAB6A	magenta	304.82122	40.838439	0.9753712	0.0046226	9.2	blue	--
NM_001044620.1	RABGGTB	brown	249.036	124.81913	0.9889378	0.0013944	179.71	blue	0
XM_001925435.1	RBM34	brown	288.84612	117.86111	0.9700801	0.0061846	38.09	blue	0.3836
AK240580.1	RCHY1	blue	343.35614	145.06382	0.9793821	0.0035429	13.96	blue	99
XM_001928955.1	RCN2	turquoise	1310.5142	1309.3818	0.960769	0.0092727	107.684	blue	99
AK236398.1	RNASEK	brown	222.29614	110.27368	0.9591186	0.0098615	9.932	blue	0
XM_001929330.1	RNPC3	turquoise	1272.363	1271.6234	0.9457256	0.0150542	11.784	blue	--
AK231197.1	RPL36	grey	289.13408	14.087015	0.8630738	0.0595575	619.35	blue	99
NM_001037146.2	RPSA	brown	267.4518	127.34562	0.9909574	0.0010308	82.46	blue	--
AK238658.1	SBDS	brown	193.5692	110.41846	0.9595152	0.0097189	3.688	blue	--
XM_001926939.1	SDC2	lightcyan	152.0737	6.9697026	0.1744806	0.7789768	14.772	blue	0.2203
AK234386.1	SFT2D1	brown	212.62377	120.25471	0.9813902	0.003039	19.588	blue	0.2254
AK239788.1	SMN1	brown	212.33186	119.28194	0.9791891	0.0035926	13.296	blue	0.3603
XM_001928820.1	SNRPC	turquoise	1433.9013	1433.152	0.9784646	0.0037814	25.034	blue	99
AK234825.1	SPCS2	blue	343.68483	152.12339	0.9908058	0.0010568	38.476	blue	0
XM_001928309.1	TBC1D7	tan	224.06047	28.218216	0.9159158	0.0288968	17.818	blue	0
AK236676.1	TGFB2	blue	332.11328	99.890928	0.8950821	0.040147	21.6	blue	99
NM_214198.1	TGFB3	brown	290.68063	95.41936	0.9102069	0.0318611	6.118	blue	99
AK239167.1	THAP3	blue	338.09095	150.79479	0.9883679	0.0015034	7.646	blue	99
AJ000786.1	TMPRSS11	brown	181.79083	107.54916	0.9528804	0.0121911	65.132	blue	--

AK239234.1	TPRG1L	blue	224.77309	106.85259	0.9109328	0.031479	14.562	blue	99
XM_001924618.1	TRAM1	brown	228.00384	123.01017	0.9865305	0.0018727	128.9	blue	99
XM_001927870.1	TUFT1	turquoise	1357.0076	1356.5144	0.9666728	0.0072669	2.084	blue	0.385
AK236036.1	UBA2	turquoise	144.64724	96.41948	0.3486553	0.5652437	30.32	blue	99
NM_214289.1	UCP2	grey	225.77404	13.794692	0.8555386	0.0644644	31.388	blue	0
AY609470.1	UFM1	brown	189.46347	107.04026	0.9508352	0.0129893	41.19	blue	--
AK239385.1	UTP18	brown	237.41207	124.71035	0.989653	0.0012615	49.456	blue	0.1679
AY609804.1	VMA21	brown	242.12098	120.9104	0.9817249	0.0029575	3.918	blue	99
AK234587.1	WDR41	brown	288.17468	113.27477	0.9577945	0.0103424	2.758	blue	--
AK239624.1	YWHAE	brown	280.02325	107.87519	0.9495074	0.0135163	330.476	blue	4.6399
AK234756.1		brown	253.81595	129.05791	0.9964207	0.0002569	9.152	blue	--
AK236487.1		brown	114.21979	49.465741	0.7585278	0.1371647	6.222	blue	--
AK232832.1		turquoise	168.88679	132.06296	0.429563	0.4703833	2.124	blue	--
XM_001924115.1		turquoise	1350.429	1349.858	0.9609578	0.0092061	2.704	blue	--
CD572587		darkgreen	238.81649	22.901772	0.9810443	0.003124	7.806	blue	--
CF363829		brown	219.54766	106.21236	0.9497174	0.0134325	7.026	blue	--
CF364773		blue	286.51612	141.45986	0.974492	0.0048717	8.364	blue	--
BX664951		turquoise	1531.1386	1530.7482	0.9926709	0.0007524	13.808	blue	--
BX666082		turquoise	146.58385	60.941013	0.1812718	0.7704679	17.33	blue	--
BX666142		brown	224.40523	112.28906	0.9656112	0.0076156	10.612	blue	99
BX666658		blue	340.92032	140.00141	0.9712276	0.0058333	4.58	blue	--
BX666730		blue	253.18195	118.47665	0.9342899	0.0200195	32.114	blue	--
BX667155		brown	253.5533	110.62918	0.9564398	0.010842	3.59	blue	--
BX667181		brown	267.73003	126.237	0.9885516	0.0014679	20.406	blue	--
BX667249		tan	266.46739	35.292984	0.9675986	0.0069672	10.552	blue	--
BX667682		brown	275.29906	126.42451	0.9885369	0.0014708	49.36	blue	0
BX668814		darkgreen	325.58166	21.083574	0.9768386	0.0042166	34.842	blue	--
BX670378		brown	156.14186	84.089669	0.9041971	0.0350801	56.766	blue	--
BX670664		magenta	332.22321	41.437194	0.9790255	0.003635	22.31	blue	--
BX673036		turquoise	1412.3922	1411.6897	0.9749195	0.0047501	2.66	blue	--
BX674124		turquoise	157.25706	123.27742	0.4275894	0.4726537	17.956	blue	--
BX674131		turquoise	1446.5322	1446.0266	0.9803558	0.0032954	14.064	blue	--
BX675880		greenyellow	238.49875	19.919724	0.9718923	0.0056329	15.478	blue	--
BX675945		purple	192.14663	32.769127	0.9743666	0.0049076	37.834	blue	--
BX676412		brown	216.22818	106.56154	0.9541495	0.0117041	15.692	blue	--
BP160416		brown	256.85328	128.58861	0.9953134	0.0003849	6.596	blue	--
BP168099		turquoise	991.97893	989.64349	0.89302	0.0413231	15.64	blue	--
BP459855		turquoise	645.83886	640.31445	0.7948868	0.1080163	4.142	blue	--
CK449686		brown	317.56885	98.845998	0.9168052	0.0284435	8.85	blue	--
CK450104		blue	287.67639	143.17034	0.9764402	0.0043256	22.768	blue	--
CK452102		grey	78.110987	7.6924039	-0.001438	0.9981686	39.888	blue	--
CK452762		blue	327.50196	156.252	0.9967778	0.0002195	38.726	blue	--
CK454645		black	171.69421	24.48803	0.8599962	0.0615469	149.918	blue	0
CK459361		greenyellow	195.83717	22.850578	0.9942178	0.0005273	37.534	blue	--
CK467320		magenta	337.1785	42.87538	0.9848146	0.0022412	25.632	blue	--
CK467557		turquoise	1559.6177	1559.1894	0.9985049	6.94E-05	1.636	blue	--
BX914521		blue	293.8666	133.90848	0.9622753	0.0087458	14.796	blue	99
BX915109		brown	170.71369	77.004787	0.8818083	0.0479028	3.55	blue	0.3771

BX915128	purple	257.70119	31.954623	0.9722273	0.0055328	95.766	blue	--
BX917211	blue	311.92942	148.6979	0.9858626	0.0020136	46.514	blue	--
BX917406	turquoise	1525.4195	1524.9214	0.9937541	0.000592	14.692	blue	99
BX919706	brown	267.11272	105.91303	0.9399028	0.0175251	11.54	blue	--
BX922414	blue	325.2434	153.0529	0.9920183	0.000855	11.774	blue	--
BX923548	brown	287.80321	118.61407	0.9688137	0.0065802	109.696	blue	0.2026
BX926488	brown	326.71558	95.814032	0.9083741	0.0328323	595.22	blue	--
CN154367	blue	308.41054	148.42191	0.9848748	0.0022279	4.618	blue	--
CN155716	green	111.04859	31.065108	0.8438273	0.0723264	170.942	blue	--
CN157754	purple	226.36404	34.420117	0.9879351	0.0015879	21.17	blue	99
CN157783	darkgreen	224.43794	20.740756	0.9601026	0.009509	15.13	blue	--
CN158088	turquoise	1488.8465	1488.196	0.9885259	0.0014729	10.666	blue	--
CN158745	blue	340.54296	154.9054	0.9948899	0.0004382	191.984	blue	--
CN160327	turquoise	1448.9062	1448.4136	0.9792797	0.0035692	13.148	blue	--
CN162261	turquoise	1398.8456	1398.362	0.9737158	0.0050951	18.182	blue	--
CN165338	turquoise	1147.7516	1146.3137	0.928244	0.0228239	11.266	blue	--
AJ659652	darkgreen	266.79146	23.93027	0.9953292	0.0003829	10.62	blue	--
CJ009624	blue	314.63369	117.3299	0.9309083	0.0215735	8.656	blue	--
CJ022587	blue	236.52263	108.32231	0.9137039	0.0300343	66.396	blue	--
CJ039813	yellow	121.5933	46.570733	0.8149221	0.0928808	22.84	blue	--
DN100858	brown	261.66395	124.03487	0.9859079	0.0020039	7.258	blue	99
DN100920	greenyellow	202.28097	21.804428	0.988073	0.0015608	19.002	blue	--
DN101483	brown	262.11961	123.8465	0.9843752	0.002339	3.594	blue	--
DN101634	blue	345.59535	150.95304	0.9888742	0.0014064	46.724	blue	--
DN103747	brown	207.99658	99.521927	0.9334766	0.0203898	10.962	blue	--
DN106205	brown	244.20266	102.19451	0.9346465	0.0198579	55.258	blue	99
DN107111	blue	300.64262	142.85559	0.9762879	0.0043676	15.606	blue	--
DN107719	brown	111.8274	58.766435	0.8258187	0.0849475	127.484	blue	--
DN107843	turquoise	123.48544	82.503101	0.3109024	0.6106198	21.048	blue	--
DN109035	blue	341.79246	141.72728	0.9748431	0.0047717	82.268	blue	--
DN109946	blue	300.08343	144.30813	0.978575	0.0037524	8.882	blue	--
DN112403	blue	315.84924	131.19405	0.9567158	0.0107396	25.292	blue	0
DN115147	turquoise	1529.6161	1529.1633	0.9935409	0.0006225	10.62	blue	--
DN115959	lightcyan	180.1688	7.5415078	0.3339279	0.5828692	7.666	blue	0
DN120378	grey	284.78413	13.971588	0.8869952	0.0448205	26.69	blue	--
DN124126	blue	300.14172	137.47621	0.967902	0.0068699	37.052	blue	--
DN125721	brown	240.29075	118.84004	0.9789788	0.0036471	8.276	blue	--
DN126967	turquoise	623.05952	619.47962	0.783406	0.1169943	1.636	blue	--
DN129644	turquoise	1437.2268	1436.4838	0.9809149	0.0031559	3.456	blue	--
DN132970	darkgreen	313.20679	22.860561	0.9924695	0.0007836	21.824	blue	--
BW957936	blue	266.57508	114.36587	0.92629	0.0237554	2.774	blue	99
BW982846	greenyellow	201.68139	22.624055	0.9933572	0.0006493	20.654	blue	--
AJ949964	brown	250.36619	120.98507	0.9788944	0.003669	0.274	blue	--
AJ956925	red	279.25828	41.838923	0.941503	0.0168339	22.08	blue	--
AJ958642	brown	164.32022	92.17831	0.9103561	0.0317825	54.132	blue	--
AJ962204	magenta	316.26815	43.708837	0.9906596	0.0010821	23.66	blue	--
AJ943510	turquoise	1563.8019	1563.3825	0.9990634	3.44E-05	3.274	blue	0
AJ944988	blue	311.49416	143.94628	0.9779248	0.0039242	7.596	blue	--

AJ944603	brown	226.185	119.09338	0.9783461	0.0038126	20.596	blue	--	
CV876445	brown	261.43289	123.45033	0.9854713	0.0020976	5.1	blue	--	
DY410986	magenta	315.80061	35.7469	0.9470766	0.0144986	12.076	blue	--	
DY424272	royalblue	195.65849	26.188104	0.9957268	0.0003351	5.534	blue	--	
DY424635	brown	322.15939	93.92699	0.9064373	0.0338686	17.502	blue		0
DY684916	brown	179.44975	94.651096	0.9306322	0.021702	7.724	blue	--	
CT863733	brown	316.34478	94.893793	0.9040163	0.0351784	13.616	blue	--	
DB803157	greenyellow	196.96358	22.817495	0.9756758	0.0045373	33.684	blue	--	
DT321969	brown	297.27329	110.54777	0.9539055	0.0117973	4.782	blue		0
DT324339	brown	252.98859	125.44901	0.9896828	0.001256	9.312	blue	--	
ES446999	blue	269.99087	130.77817	0.956422	0.0108486	22.482	blue	--	
EV857330	brown	246.35374	129.70038	0.9991568	2.94E-05	13.762	blue	--	
EW023671	brown	265.31065	114.06801	0.9608476	0.009245	2.774	blue	--	
EW074840	blue	322.53893	156.75405	0.9974984	0.0001501	9.574	blue	--	
EW083818	brown	206.13457	91.890373	0.9135795	0.0300987	2.984	blue	--	
EW087471	blue	315.85518	153.28792	0.9924431	0.0007877	7.464	blue	--	
EW091753	turquoise	231.35744	217.88983	0.5583368	0.3279944	60.62	blue	--	
EW093873	brown	257.62232	124.68707	0.9890181	0.0013792	6.754	blue	--	
EW112465	royalblue	165.23884	23.82833	0.9739875	0.0050166	4.07	blue	--	
EW130595	darkgreen	229.78482	19.449722	0.9493961	0.0135608	3.628	blue	--	
EW136988	brown	246.50271	127.28781	0.9944264	0.0004991	19.844	blue	--	
EW137538	tan	238.37931	32.476433	0.9494463	0.0135408	2607.53	blue	--	
EW146447	tan	341.21265	37.531213	0.9768459	0.0042147	44.178	blue	--	
EW155747	darkgreen	308.96773	22.041818	0.9846472	0.0022783	75.742	blue	--	
EW159788	brown	190.11179	108.39955	0.9539691	0.011773	17.614	blue	--	
EW167492	brown	180.4887	98.874708	0.940703	0.0171784	7.038	blue	--	
EW188932	blue	311.1495	151.78681	0.9902369	0.0011563	19.254	blue	--	
EW189192	brown	142.65231	66.903632	0.8539289	0.0655282	19.57	blue	--	
EW191982	brown	247.75134	122.50497	0.9834288	0.0025544	3.514	blue	--	
EW192633	brown	250.75782	124.84429	0.9892765	0.0013309	4.216	blue	--	
EW196387	brown	336.56266	78.212845	0.8526676	0.0663656	6.33	blue	--	
EW201887	brown	260.04146	128.69912	0.9952708	0.0003901	8.12	blue	--	
EW205590	turquoise	153.11761	81.663749	0.2223707	0.7192198	40.778	blue	--	
EW206037	brown	236.99242	120.50788	0.9811666	0.0030938	4.664	blue	--	
EW212556	blue	262.95127	119.74219	0.9369746	0.018813	21.326	blue	--	
EW244600	magenta	278.7946	34.329013	0.9376575	0.01851	55.084	blue	--	
EW244903	blue	318.40002	155.35841	0.9954709	0.0003656	15.31	blue	--	
EW247940	brown	228.31106	124.07238	0.9887254	0.0014347	9.866	blue	--	
EW249284	brown	253.37941	129.42086	0.9972033	0.0001775	3.47	blue	--	
EW310470	brown	154.55768	90.164538	0.9055076	0.0343697	2.694	blue	--	
EW322856	brown	172.26734	91.061967	0.9077414	0.0331697	10.148	blue	--	
EW332614	turquoise	1515.7317	1515.3208	0.9900946	0.0011817	2.094	blue		99
EW334411	turquoise	176.50278	71.508509	0.1870116	0.7632847	12.454	blue	--	
EW359956	brown	243.05897	129.09843	0.9977287	0.0001299	6.334	blue	--	
EW363654	royalblue	164.51255	24.01654	0.9746762	0.0048191	4.754	blue	--	
EW407282	blue	312.69566	146.0845	0.9816117	0.002985	28.038	blue	--	
EW417768	brown	251.81178	127.68159	0.9953816	0.0003765	10.086	blue	--	
EW453494	brown	272.90858	126.61897	0.9888157	0.0014175	4.398	blue	--	

EW470987	lightcyan	226.28433	18.438948	0.8964565	0.0393691	22.354	blue	--	
EW483255	navy	151.42445	16.579301	0.8264355	0.084505	0.664	blue	--	
EW519341	brown	236.45858	129.44672	0.9995387	1.19E-05	33.396	blue	--	
EW521970	brown	254.71771	129.82197	0.9981956	9.20E-05	12.6	blue	--	
EW548211	brown	204.75052	118.16216	0.9769652	0.0041822	10.562	blue	--	
EW572865	brown	264.41687	128.12052	0.9935519	0.000621	162.468	blue		99
EW576918	turquoise	476.23876	461.92216	0.7070009	0.1817854	56.846	blue	--	
EW577626	brown	243.64883	125.51644	0.9901958	0.0011636	9.46	blue	--	
EW588646	brown	258.44322	129.13045	0.9960878	0.0002936	11.696	blue	--	
EW594177	turquoise	150.24602	138.9946	0.4790994	0.414206	0.998	blue	--	
EW603701	blue	318.98105	139.79351	0.9715566	0.0057338	7.144	blue	--	
EW621683	brown	245.51607	129.79972	0.9992501	2.46E-05	10.638	blue	--	
EW664676	blue	260.43839	125.78137	0.947117	0.0144822	16.19	blue	--	
EW670598	royalblue	237.89998	23.074848	0.9669989	0.0071609	21.662	blue	--	
BE030540	magenta	288.48277	37.247188	0.9554189	0.0112237	6.304	blue	--	
BE233268	brown	282.9881	106.89371	0.9401601	0.0174134	4.786	blue	--	
BG896003	brown	276.13403	122.71584	0.9818363	0.0029306	10.628	blue	--	
BQ599187	brown	198.43594	105.3165	0.9463539	0.014795	6.576	blue	--	
BQ601250	greenyellow	177.45028	22.451258	0.9861037	0.0019623	8.732	blue	--	
BI399871	brown	249.34061	126.94623	0.9929076	0.0007163	10.162	blue	--	
BQ597564	greenyellow	185.4114	22.509569	0.971464	0.0057618	10.262	blue	--	
BI181083	brown	223.43437	124.43599	0.9897633	0.0012414	9.148	blue		99
BI184747	turquoise	160.14483	113.253	0.335049	0.581524	77.348	blue	--	
BI181508	turquoise	1510.9799	1510.5698	0.9904147	0.0011249	2.748	blue		99
BQ604024	greenyellow	146.15289	19.243153	0.938039	0.0183414	5.694	blue	--	
BM190542	brown	259.14389	129.07085	0.9961119	0.0002909	12.012	blue	--	

Table S7-2. Module eigengene and its evolutionary rates in five prenatal highly LT-specific modules (LT_darkred).

pig_Genebank	pig_Gene	LDEModule	kTotal	kWithin	eigencorr	eigenpval	mean Expr	LT Module	Ka/Ks
NM_001044548.1	NTN1	blue	297.0833	140.6561	0.972952	0.005318	17.346	darkred	0
NM_001044606.1	ATP5O	magenta	333.135	43.79341	0.991024	0.001019	144.15	darkred	0.1786
AK231033.1	MAPKSP1	blue	326.3434	113.4681	0.923579	0.025067	22.608	darkred	0
AK232289.1	NOL7	greenyellow	218.8315	21.40189	0.983842	0.00246	12.222	darkred	--
AK235604.1	CMTM3	lightcyan	202.6861	6.436603	0.261776	0.670543	33.82	darkred	--
AK238860.1	RPF1	turquoise	119.3662	44.7817	0.087672	0.888515	58.664	darkred	--
AK236086.1	ALKBH5	grey	315.4755	17.24843	0.967176	0.007103	79.31	darkred	--
AK230619.1	PSMA2	purple	160.9621	27.64482	0.92814	0.022873	211.14	darkred	99
AK236420.1	RRAGD	lightyellow	298.5297	23.74369	0.977006	0.004171	11.102	darkred	0
AK236617.1	G3BP2	tan	320.6253	38.44607	0.983132	0.002623	31.808	darkred	99
XM_001925875.1	TPD52	yellow	131.7131	58.58274	0.897157	0.038974	55.446	darkred	0
XM_001926913.1	POLR1D	blue	345.1107	146.6876	0.98223	0.002836	81.41	darkred	0
XM_001925382.1	MRPL43	blue	322.5901	130.1057	0.9556	0.011156	23.184	darkred	--
XM_001925458.1	FAM21A	brown	145.0148	71.98678	0.84294	0.072933	11.906	darkred	0.5382
BX665103		greenyellow	235.0703	20.23032	0.938163	0.018287	40.664	darkred	--
BX665955		brown	279.5568	117.322	0.970932	0.005923	6.566	darkred	99
BX675385		blue	340.0489	157.4072	0.998373	7.88E-05	5.376	darkred	--
CK451664		blue	257.1589	116.625	0.932339	0.020912	71.426	darkred	--
CK454516		royalblue	209.4712	25.85391	0.993263	0.000663	5.85	darkred	--
CK464065		yellow	99.67795	42.60602	0.794768	0.108108	125.26	darkred	--
BX921473		purple	174.6442	27.51044	0.931203	0.021436	2.656	darkred	--
CN157594		brown	322.4954	101.4193	0.923754	0.024982	20.452	darkred	--
CN161590		turquoise	1534.085	1533.56	0.994954	0.00043	5.248	darkred	--
DN120090		blue	318.3672	146.5824	0.982169	0.002851	18.91	darkred	--
AJ948107		purple	76.21353	5.652905	0.532425	0.355647	1.612	darkred	--
DR120938		blue	318.4968	155.8417	0.996066	0.000296	28.778	darkred	--
CV875565		turquoise	120.4862	103.4161	0.416429	0.485536	4.124	darkred	--
DY437498		darkturquoise	146.4519	15.21459	0.954126	0.011713	16.398	darkred	99
EB684343		blue	352.518	128.945	0.952505	0.012336	83.21	darkred	--
DB786707		blue	312.6502	140.9349	0.973474	0.005165	11.496	darkred	--
AK236389	EPHX1	blue	339.0518	150.6831	0.988386	0.0015	13.328	darkred	0.1916
DT321372		turquoise	557.201	553.6229	0.757947	0.137647	2.992	darkred	--
EV873382		yellow	311.1789	57.24785	0.87106	0.054492	26.268	darkred	--
EW281140		blue	300.6658	131.3314	0.956686	0.01075	753.84	darkred	--
EW430131		navy	270.3831	32.86976	0.984972	0.002206	26.046	darkred	--
EW520628		darkred	62.86005	11.88912	0.909178	0.032405	34.334	darkred	--
EW567071		turquoise	34.30398	24.46049	0.161854	0.794824	27.974	darkred	--
BG385044		yellow	297.5939	72.49647	0.944403	0.015605	31.402	darkred	--
BF080208								darkred	--
BI185477		red	244.4728	42.084	0.945668	0.015078	5.082	darkred	0
CA780055		lightcyan	271.5258	22.52244	0.954638	0.011518	19.592	darkred	--

Table S7-3. Module eigengene and its evolutionary rates in five prenatal highly LT-specific modules (LT_only_turquoise).

pig_Geneba	pig_Gene	kTotal	kWithin	eigencorr	eigenpval	meanExpr	LTModule	Ka/Ks
XM_00192	LTA4H	1313.2006	1312.6078	0.9534027	0.0119899	3.936	turquoise	0
AY166682.	TMBIM6	224.59407	35.259588	0.9888177	0.0014171	27.744	turquoise	--
NM_21442	CYP11A1	1052.6105	1051.5399	0.9001052	0.0373277	13.586	turquoise	--
AY610411.	TPRKB	187.08178	48.624279	0.9814416	0.0030264	84.108	turquoise	0
AY825267.	ABCB1	131.09108	35.186501	0.9085637	0.0327313	13.95	turquoise	0.1414
NM_21375	STAR	205.85748	51.762488	0.9952051	0.0003983	10.256	turquoise	99
AU298693		328.07076	42.969476	0.9846293	0.0022823	103.902	turquoise	--
BX675384		201.69581	22.910172	0.9867411	0.0018291	8.424	turquoise	--
BX675925		199.63297	99.966213	0.9428554	0.0162569	19.694	turquoise	--
BX926015		85.761236	7.5093144	0.3958671	0.5094592	2.688	turquoise	--
CN164380		229.01771	105.08113	0.9479778	0.0141318	4.17	turquoise	--
DN118117		318.49676	155.84171	0.9960664	0.000296	28.778	turquoise	--
EW169810		120.4862	103.41614	0.4164294	0.4855356	4.124	turquoise	--
EW240620		146.45189	15.21459	0.9541262	0.011713	16.398	turquoise	--
EW421594		352.51804	128.945	0.9525051	0.0123363	83.21	turquoise	99

Table S7-4. Module eigengene and its evolutionary rates in five prenatal highly LT-specific modules (LT_purple).

pig_Genebank	pig_Gene	LDE Module	kTotal	kWithin	eigencorr	eigenpval	meanExpr	LT Module	Ka/Ks
NM_001044527.1	ENO3	green	241.36404	53.77627	0.989651	0.001262	428.998	purple	0
NM_001044594.1	MYOZ3	green	221.29959	47.242311	0.940534	0.017251	21.02	purple	--
NM_001044616.1	NRAP	pink	194.85748	44.968614	0.985195	0.002158	21.004	purple	0.117
AK237414.1	PTP4A3	midnightblue	229.84127	36.508053	0.996251	0.000275	101.384	purple	--
AK238056.1		midnightblue	240.15671	37.116479	0.999506	1.32E-05	19.294	purple	--
AK231783.1	PDLIM3	midnightblue	209.63159	32.248573	0.970883	0.005938	68.094	purple	0
AK238516.1	ITIH5	lightcyan	269.79797	22.788062	0.941017	0.017043	53.16	purple	0.412
AK238595.1	MALAT1	green	167.73067	34.570212	0.893264	0.041183	11.114	purple	--
AK236532.1	RPS6KA3	green	104.07577	28.504252	0.835992	0.077741	39.638	purple	0
AK232843.1	GPD1	midnightblue	164.04493	28.786626	0.9464	0.014776	3.086	purple	0
AK239600.1	DCI	grey60	268.49997	32.709955	0.994818	0.000447	24.166	purple	--
AK239646.1	HADHB	navy	247.71996	30.132073	0.96408	0.008128	83.924	purple	0
AK239858.1	CD99	yellow	221.78598	60.268196	0.910706	0.031598	171.656	purple	--
AK233342.1	SLC6A12	midnightblue	203.24666	33.49299	0.977947	0.003918	6.814	purple	0
AK233419.1	SLC25A4	midnightblue	251.57336	35.606469	0.990835	0.001052	232.884	purple	0
AK236976.1	PPP1R3B	midnightblue	264.71674	30.850524	0.959952	0.009563	16.28	purple	0.087
AK240262.1	CLEC3B	green	153.30118	31.769604	0.857884	0.062924	28.772	purple	--
AK237272.1	HSPB6	green	229.92393	53.270586	0.982111	0.002865	154.294	purple	--
NM_213946.2	FHL3	grey	273.65615	12.269239	0.816903	0.091423	50.504	purple	--
NM_001097516.1	SDHD	turquoise	1519.8096	1519.4059	0.99088	0.001044	3.378	purple	0.491
NM_001109945.1	CDH13	turquoise	1502.9416	1502.5711	0.988217	0.001533	2.378	purple	0.053
NM_001128433.1	SLC2A4	red	181.42021	47.839103	0.977446	0.004052	26.03	purple	--
XM_001927804.1	NDUFB9	pink	183.62776	38.79323	0.95279	0.012226	136.806	purple	0
XM_001928966.1	CASQ1	red	178.13503	47.208159	0.974381	0.004903	43.322	purple	0
XM_001929660.1	SEMA6C	green	190.45898	51.702221	0.989103	0.001363	41.324	purple	0.196
XM_001927213.1	ANKRD28	turquoise	1294.4525	1293.8259	0.953462	0.011967	23.146	purple	0
XM_001926917.1	PLXND1	midnightblue	193.88712	33.7288	0.979207	0.003588	108.794	purple	0.03
XM_001927272.1	ACTN2	pink	248.98847	45.260147	0.984763	0.002253	652.286	purple	0
XM_001929322.1	ANK3	pink	200.03273	43.540449	0.97872	0.003714	72.954	purple	0.136
XM_001928874.1	SNTA1	midnightblue	236.14427	34.870391	0.986448	0.00189	12.122	purple	0
XM_001929063.1	MAFB	red	144.09108	39.135852	0.93091	0.021573	35.88	purple	--
NM_213928.1	GOT2	turquoise	1493.0281	1492.6094	0.98608	0.001967	12.082	purple	0.078
NM_213954.1	ACO2	navy	253.27162	31.694912	0.977444	0.004053	41.276	purple	0
NM_214213.1	PLN	midnightblue	223.00568	36.446624	0.995808	0.000326	9.668	purple	--
NM_213855.1	MYH7	grey	200.03233	12.036919	0.805759	0.099717	623.904	purple	0
NM_214136.1	MYH2	red	232.58535	50.4905	0.989954	0.001207	18.784	purple	0
BX673614		green	215.62735	50.583426	0.969286	0.006432	22.06	purple	--
BP168899		turquoise	1570.9326	1570.5358	0.999793	3.56E-06	2.716	purple	0
CK463858		greenyellow	88.310184	5.1841377	-0.341433	0.573874	78.988	purple	--
BX920496		red	201.39973	50.51983	0.990002	0.001198	2.12	purple	0
BX924065		red	206.21917	51.051459	0.992381	0.000797	25.374	purple	--
BX926893		green	154.5316	42.800047	0.931367	0.021361	41.124	purple	--
CN156156		navy	207.87142	27.795967	0.950561	0.013098	10.44	purple	--
CN156511		turquoise	759.08699	756.4775	0.819666	0.0894	9.598	purple	99

CN157450	turquoise	1313.2006	1312.6078	0.953403	0.01199	3.936	purple	0
CN162187	midnightblue	224.59407	35.259588	0.988818	0.001417	27.744	purple	0
CN162365	turquoise	1052.6105	1051.5399	0.900105	0.037328	13.586	purple	0
DN113717	green	187.08178	48.624279	0.981442	0.003026	84.108	purple	--
AJ947734	red	131.09108	35.186501	0.908564	0.032731	13.95	purple	--
AJ961806	red	205.85748	51.762488	0.995205	0.000398	10.256	purple	--
DV226293	green	239.19398	51.37013	0.983492	0.00254	18.336	purple	0
DV227364	turquoise	161.37245	139.04102	0.464779	0.430281	7.414	purple	--
DY405382	midnightblue	228.85453	35.096389	0.98779	0.001617	112.296	purple	0
DY437756	lightcyan	286.57469	27.075539	0.998759	5.25E-05	21.308	purple	99
DB805355	midnightblue	249.30531	36.531184	0.996135	0.000288	14.2	purple	0
DT320474	turquoise	119.73692	85.690135	0.318672	0.60123	1.334	purple	--
DT325394	turquoise	1536.2533	1535.8493	0.993277	0.000661	5.436	purple	0.214
DT328391	turquoise	1544.2644	1543.8592	0.994949	0.000431	4.678	purple	0
DT331413	red	202.51883	48.41159	0.980266	0.003318	11.87	purple	--
EW088403	red	266.65463	47.167965	0.970283	0.006122	94.282	purple	--
EW132770	grey60	265.12374	32.565253	0.993788	0.000587	78.852	purple	--
EW142807	turquoise	873.33005	871.53257	0.860323	0.061335	9.81	purple	99
EW241468	green	145.43487	36.901154	0.903744	0.035327	17.814	purple	--
EW362700	midnightblue	260.67859	32.420281	0.970941	0.00592	93.876	purple	--
EW366014	green	267.48117	46.61252	0.946158	0.014876	18.514	purple	--
EW633584	red	192.96338	47.371267	0.976259	0.004376	35.772	purple	0.725
EW675702	green	196.82198	50.257772	0.966981	0.007167	17.716	purple	--
BF190620	red	228.19632	50.637891	0.989303	0.001326	3.036	purple	--
BI346349	green	253.24049	50.080944	0.957932	0.010292	74.176	purple	--

Table S7-5. Module eigengene and its evolutionary rates in five prenatal highly LT-specific modules (LT_yellowgreen).

pig_Genebank	pig_Gene	LDEModule	kTotal	kWithin	eigencorr	eigenpval	mean Expr	LTModule	Ka/Ks
DQ508264.1	PYGM	green	127.1	18.0861	0.7305	0.160949	1.25	yellowgreen	0.0327
AK240563.1	DAG1	grey	278.9	16.1809	0.9419	0.016651	72.038	yellowgreen	--
XM_001924280.1	SNTB1	salmon	96.33	18.2177	0.863	0.059616	26.326	yellowgreen	0.0986
XM_001925133.1	ACIN1	blue	346	149.203	0.9858	0.002028	66.896	yellowgreen	0.4614
XM_001929585.1	HSPB8	green	226.5	49.9531	0.9735	0.00517	40.122	yellowgreen	--
XM_001929468.1	CHCHD10	green	262.5	50.4352	0.9701	0.006181	31.734	yellowgreen	--
XM_001929645.1	DGCR8	turquoise	1337	1336.45	0.9637	0.008245	8.978	yellowgreen	0.1729
XM_001927848.1	MED12	turquoise	419.8	414.505	0.6859	0.201106	38.478	yellowgreen	0.0701
NM_001032359.1	TNNI2	green	218.7	52.432	0.9792	0.003584	72.186	yellowgreen	--
BX667460		navy	253.9	31.8617	0.9795	0.003523	101.39	yellowgreen	--
BX667493		pink	182.5	43.0254	0.9752	0.004669	24.436	yellowgreen	0
BX916417		red	232.1	51.6131	0.9942	0.00053	18.122	yellowgreen	--
CJ014614		midnightblue	209.6	32.302	0.9712	0.005847	9.656	yellowgreen	--
DN111814								yellowgreen	--
DN132764		lightyellow	282.2	24.1293	0.9686	0.006656	47.506	yellowgreen	--
BW978477		turquoise	1547	1547.02	0.9967	0.000224	3.07	yellowgreen	0
AJ950525		black	170.8	24.3766	0.8531	0.066089	150.35	yellowgreen	--
DV901297		red	176.1	46.6156	0.972	0.00559	5.2	yellowgreen	--
CV875164		turquoise	467.2	462.542	0.7096	0.179478	15.448	yellowgreen	--
CX064239		turquoise	1168	1166.82	0.925	0.024354	13.266	yellowgreen	--
BI182545		turquoise	1283	1282.26	0.9471	0.014489	10.258	yellowgreen	--
BI184076		turquoise	951.1	949.36	0.8739	0.052706	8.984	yellowgreen	--

Table S8-1. GO annotation analysis of the module eigengene in six prenatal highly LDE-specific modules (LDE_blue).

GO ID	GO Name	Total Count	Diff Count	P Value	FDR	Gene
GO:0006412	translation	130	3	0.0009699	0.1134748	[AARSD1, MRPL43, MRPL52]
GO:0045657	positive regulation of monocyte differentiation	1	1	0.001481	0.0266582	[ACIN1]
GO:0046325	negative regulation of glucose import	1	1	0.001481	0.0266582	[PEA15]
GO:0048239	negative regulation of DNA recombination at telomere	1	1	0.001481	0.0266582	[TERF2IP]
GO:0042637	catagen	1	1	0.001481	0.0266582	[TGFB2]
GO:0050655	dermatan sulfate proteoglycan metabolic process	1	1	0.001481	0.0266582	[CHST14]
GO:0045823	positive regulation of heart contraction	1	1	0.001481	0.0266582	[TGFB2]
GO:0045932	negative regulation of muscle contraction	1	1	0.001481	0.0266582	[TNNT1]
GO:0051795	positive regulation of catagen	1	1	0.001481	0.0266582	[TGFB2]
GO:0048103	somatic stem cell division	1	1	0.001481	0.0266582	[TGFB2]
GO:0006517	protein deglycosylation	1	1	0.001481	0.0266582	[AGA]
GO:0060998	regulation of dendritic spine development	2	1	0.0029599	0.0203708	[ARF6]
GO:0019439	aromatic compound catabolic process	2	1	0.0029599	0.0203708	[EPHX1]
GO:0060038	cardiac muscle cell proliferation	2	1	0.0029599	0.0203708	[TGFB2]
GO:0010002	cardioblast differentiation	2	1	0.0029599	0.0203708	[TGFB2]
GO:0035020	regulation of Rac protein signal transduction	2	1	0.0029599	0.0203708	[ARF6]
GO:0071786	endoplasmic reticulum tubular network organization	2	1	0.0029599	0.0203708	[RTN3]
GO:0048261	negative regulation of receptor-mediated endocytosis	2	1	0.0029599	0.0203708	[ARF6]
GO:0000052	citrulline metabolic process	2	1	0.0029599	0.0203708	[DDAH2]
GO:0050778	positive regulation of immune response	2	1	0.0029599	0.0203708	[TGFB2]
GO:0051891	positive regulation of cardioblast differentiation	2	1	0.0029599	0.0203708	[TGFB2]
GO:0031848	protection from non-homologous end joining at telomere	2	1	0.0029599	0.0203708	[TERF2IP]
GO:0042981	regulation of apoptotic process	65	2	0.00423	0.0215179	[CTSB, PEA15]
GO:0010833	telomere maintenance via telomere lengthening	3	1	0.0044366	0.0203559	[TERF2IP]
GO:0030263	apoptotic chromosome condensation	3	1	0.0044366	0.0203559	[ACIN1]
GO:0042416	dopamine biosynthetic process	3	1	0.0044366	0.0203559	[TGFB2]
GO:0045919	positive regulation of cytolysis	3	1	0.0044366	0.0203559	[RRAGA]
GO:0006457	protein folding	68	2	0.0046195	0.0193029	[DNAJB6, PPID]
GO:0046683	response to organophosphorus	4	1	0.0059111	0.0223096	[TYMS]
GO:0032205	negative regulation of telomere maintenance	4	1	0.0059111	0.0223096	[TERF2IP]
GO:0048268	clathrin coat assembly	4	1	0.0059111	0.0223096	[AP2S1]

GO:0070389	chaperone cofactor-dependent protein refolding	4	1	0.0059111	0.0223096	[PTGES3]
GO:0003009	skeletal muscle contraction	4	1	0.0059111	0.0223096	[TNNT1]
GO:0006916	anti-apoptosis	84	2	0.0069625	0.0239592	[DDAH2, PEA15]
GO:0031069	hair follicle morphogenesis	5	1	0.0073835	0.0239963	[TGFB2]
GO:0045109	intermediate filament organization	5	1	0.0073835	0.0239963	[DNAJB6]
GO:0048699	generation of neurons	5	1	0.0073835	0.0239963	[TGFB2]
GO:0051489	regulation of filopodium assembly	6	1	0.0088537	0.0265612	[ARF6]
GO:0010569	regulation of double-strand break repair via homologous recombination	6	1	0.0088537	0.0265612	[TERF2IP]
GO:0050777	negative regulation of immune response	6	1	0.0088537	0.0265612	[TGFB2]
GO:0031529	ruffle organization	7	1	0.0103218	0.0274467	[ARF6]
GO:0048666	neuron development	7	1	0.0103218	0.0274467	[TGFB2]
GO:0043525	positive regulation of neuron apoptosis	7	1	0.0103218	0.0274467	[TGFB2]
GO:0006401	RNA catabolic process	7	1	0.0103218	0.0274467	[RNASEH2C]
GO:0030866	cortical actin cytoskeleton organization	7	1	0.0103218	0.0274467	[ARF6]
GO:0019048	virus-host interaction	7	1	0.0103218	0.0274467	[RRAGA]
GO:0034614	cellular response to reactive oxygen species	7	1	0.0103218	0.0274467	[PRDX5]
GO:0006950	response to stress	104	2	0.0104964	0.0253213	[AKR1B1, RTN3]
GO:0016192	vesicle-mediated transport	104	2	0.0104964	0.0253213	[ARF6, RTN3]
GO:0044419	interspecies interaction between organisms	315	3	0.0115241	0.0269665	[PSMB4, RRAGA, RTN3]
GO:0001942	hair follicle development	8	1	0.0117878	0.02678	[TGFB2]
GO:0071230	cellular response to amino acid stimulus	8	1	0.0117878	0.02678	[RRAGA]
GO:0006915	apoptotic process	321	3	0.0121215	0.0267587	[NTN1, RRAGA, RTN3]
GO:0030100	regulation of endocytosis	9	1	0.0132516	0.0279358	[AP2S1]
GO:0016999	antibiotic metabolic process	9	1	0.0132516	0.0279358	[AKR1B1]
GO:0097067	cellular response to thyroid hormone stimulus	9	1	0.0132516	0.0279358	[CTSB]
GO:0009165	nucleotide biosynthetic process	9	1	0.0132516	0.0279358	[TYMS]
GO:0000723	telomere maintenance	10	1	0.0147133	0.0291772	[PTGES3]
GO:0001654	eye development	10	1	0.0147133	0.0291772	[TGFB2]
GO:0045787	positive regulation of cell cycle	10	1	0.0147133	0.0291772	[TGFB2]
GO:0030261	chromosome condensation	11	1	0.0161728	0.0307678	[NCAPG2]
GO:0030838	positive regulation of actin filament polymerization	11	1	0.0161728	0.0307678	[ARF6]
GO:0008219	cell death	136	2	0.0174542	0.032415	[RRAGA, TGFB2]
GO:0001516	prostaglandin biosynthetic process	12	1	0.0176302	0.0314922	[PTGES3]

GO:0032008	positive regulation of TOR signaling cascade	12	1	0.0176302	0.0314922	[RRAGA]
GO:0032436	positive regulation of proteasomal ubiquitin-dependent protein catabolic process	12	1	0.0176302	0.0314922	[RCHY1]
GO:0000902	cell morphogenesis	12	1	0.0176302	0.0314922	[TGFB2]
GO:0030218	erythrocyte differentiation	13	1	0.0190855	0.0325987	[ACIN1]
GO:0034613	cellular protein localization	13	1	0.0190855	0.0325987	[RRAGA]
GO:0042060	wound healing	15	1	0.0219898	0.0362367	[TGFB2]
GO:0050680	negative regulation of epithelial cell proliferation	15	1	0.0219898	0.0362367	[KRT4]
GO:0030593	neutrophil chemotaxis	15	1	0.0219898	0.0362367	[TGFB2]
GO:0009636	response to toxin	17	1	0.0248855	0.0396137	[EPHX1]
GO:0030855	epithelial cell differentiation	17	1	0.0248855	0.0396137	[KRT4]
GO:0042787	protein ubiquitination involved in ubiquitin-dependent protein catabolic process	18	1	0.0263302	0.0408032	[RCHY1]
GO:0006898	receptor-mediated endocytosis	18	1	0.0263302	0.0408032	[PICALM]
GO:0031398	positive regulation of protein ubiquitination	22	1	0.032088	0.0487572	[RCHY1]
GO:0006508	proteolysis	476	3	0.0337625	0.0506438	[CAPN10, CTSB, SPCS2]
GO:0051865	protein autoubiquitination	25	1	0.0363844	0.0532122	[RCHY1]
GO:0043154	negative regulation of cysteine-type endopeptidase activity involved in apoptotic process	25	1	0.0363844	0.0532122	[DNAJB6]
GO:0048015	phosphatidylinositol-mediated signaling	25	1	0.0363844	0.0532122	[TYMS]
GO:0030097	hemopoiesis	29	1	0.0420836	0.0600462	[TGFB2]
GO:0030307	positive regulation of cell growth	30	1	0.0435033	0.0613239	[TGFB2]
GO:0009790	embryo development	31	1	0.0449208	0.0621981	[TGFB2]
GO:0006928	cellular component movement	31	1	0.0449208	0.0621981	[ARF6]
GO:0008643	carbohydrate transport	35	1	0.0505704	0.0684015	[PEA15]
GO:0006986	response to unfolded protein	35	1	0.0505704	0.0684015	[DNAJB6]
GO:0007507	heart development	37	1	0.0533828	0.0709748	[TGFB2]
GO:0006979	response to oxidative stress	40	1	0.057586	0.075703	[PRDX5]
GO:0051781	positive regulation of cell division	41	1	0.058983	0.0766779	[TGFB2]
GO:0006633	fatty acid biosynthetic process	42	1	0.060378	0.0776288	[PTGES3]
GO:0051092	positive regulation of NF-kappaB transcription factor activity	45	1	0.0645507	0.0820916	[TERF2IP]
GO:0005975	carbohydrate metabolic process	58	1	0.0824224	0.1036927	[PGP]
GO:0006417	regulation of translation	59	1	0.0837831	0.1042832	[EIF4E2]
GO:0007010	cytoskeleton organization	71	1	0.0999578	0.1231059	[KRT4]

GO:0015031 protein transport	383	2	0.1105116	0.1346861	[ARF6, RAB23]
GO:0006917 induction of apoptosis	79	1	0.1105844	0.1333853	[NUDT2]
GO:0006355 regulation of transcription, DNA-dependent	1254	4	0.1151356	0.1374578	[EWSR1, FOXK2, HNRPDL, TERF2IP]
GO:0007155 cell adhesion	420	2	0.1284969	0.1518599	[ARF6, TNFAIP6]
GO:0006260 DNA replication	93	1	0.1288845	0.1507949	[TYMS]
GO:0000398 nuclear mRNA splicing, via spliceosome	98	1	0.13533	0.1567684	[SNRPD1]
GO:0016567 protein ubiquitination	99	1	0.1366134	0.1567036	[RCHY1]
GO:0008284 positive regulation of cell proliferation	102	1	0.1404525	0.1580091	[TGFB2]
GO:0008285 negative regulation of cell proliferation	102	1	0.1404525	0.1580091	[TGFB2]
GO:0001525 angiogenesis	102	1	0.1404525	0.1580091	[TGFB2]
GO:0043123 positive regulation of I-kappaB kinase/NF-kappaB cascade	105	1	0.1442748	0.1592467	[TERF2IP]
GO:0043066 negative regulation of apoptotic process	118	1	0.1606456	0.1756592	[PRDX5]
GO:0006954 inflammatory response	128	1	0.1730284	0.1874474	[PRDX5]
GO:0007165 signal transduction	141	1	0.1888573	0.2027184	[PPP1R1B]
GO:0006281 DNA repair	147	1	0.196062	0.2085387	[TYMS]
GO:0007067 mitosis	159	1	0.2102829	0.2216495	[NCAPG2]
GO:0007399 nervous system development	214	1	0.2723561	0.2845149	[ARF6]
GO:0051301 cell division	285	1	0.3454282	0.3576558	[NCAPG2]
GO:0006810 transport	395	1	0.4446061	0.4563063	[PEA15]
GO:0007049 cell cycle	405	1	0.4528515	0.4607272	[NCAPG2]
GO:0030154 cell differentiation	484	1	0.5138894	0.5183194	[ARF6]
GO:0007275 multicellular organismal development	822	1	1	1	[MYOG]

Table S8-2. GO annotation analysis of the module eigengene in six prenatal highly LDE-specific modules (LDE_midnightblue).

GO ID	GO Name	Total Count	Diff Count	P Value	FDR	Gene
GO:0071318	cellular response to ATP	1	1	0.000524	0.0101316	[RYR3]
GO:0070588	calcium ion transmembrane transport	1	1	0.000524	0.0101316	[RYR3]
GO:0071286	cellular response to magnesium ion	2	1	0.0010478	0.0086821	[RYR3]
GO:0002318	myeloid progenitor cell differentiation	2	1	0.0010478	0.0086821	[MLF1]
GO:0071313	cellular response to caffeine	3	1	0.0015714	0.0091139	[RYR3]
GO:0006744	ubiquinone biosynthetic process	6	1	0.0031404	0.0151784	[COQ9]
GO:0051924	regulation of calcium ion transport	7	1	0.0036628	0.014163	[PLN]
GO:0071526	semaphorin-plexin signaling pathway	7	1	0.0036628	0.014163	[PLXND1]
GO:0045765	regulation of angiogenesis	10	1	0.0052287	0.016848	[PLXND1]
GO:0043542	endothelial cell migration	13	1	0.0067922	0.0196973	[PLXND1]
GO:0005977	glycogen metabolic process	14	1	0.0073128	0.0192793	[PPP1R3B]
GO:0071277	cellular response to calcium ion	15	1	0.0078332	0.0189303	[RYR3]
GO:0007416	synapse assembly	20	1	0.0104312	0.0224078	[PLXND1]
GO:0016310	phosphorylation	20	1	0.0104312	0.0224078	[PGK1]
GO:0030334	regulation of cell migration	22	1	0.0114686	0.0221726	[PLXND1]
GO:0051289	protein homotetramerization	25	1	0.0130227	0.0236037	[RYR3]
GO:0007517	muscle organ development	38	1	0.0197303	0.0336575	[MYL1]
GO:0006351	transcription, DNA-dependent	48	1	0.0248602	0.0400526	[MLF1]
GO:0007050	cell cycle arrest	52	1	0.026905	0.0410655	[MLF1]
GO:0005975	carbohydrate metabolic process	58	1	0.0299645	0.0434485	[PPP1R3B]
GO:0006457	protein folding	68	1	0.0350432	0.048393	[BAG3]
GO:0006916	anti-apoptosis	84	1	0.0431163	0.0568351	[BAG3]
GO:0001525	angiogenesis	102	1	0.0521212	0.0657181	[PLXND1]
GO:0044419	interspecies interaction between organisms	315	1	0.1527102	0.1845248	[SLC25A4]
GO:0007049	cell cycle	405	1	0.1920693	0.2228004	[MLF1]
GO:0006811	ion transport	455	1	0.2131734	0.2377704	[RYR3]
GO:0030154	cell differentiation	484	1	0.2251711	0.2418505	[MLF1]
GO:0008150	biological_process	588	1	0.2667746	0.2763022	[ASPH]
GO:0007275	multicellular organismal development	822	1	0.3527118	0.3527118	[MLF1]

Table S8-3. GO annotation analysis of the module eigengene in six prenatal highly LDE-specific modules (LDE_only_turquoise).

GO ID	GO Name	Total Count	Diff Count	P Value	FDR	Gene
GO:0006915	apoptotic process	321	3	0.0002915	0.0116613	[FIS1, PDCD6IP, TAX1BP1]
GO:0090050	positive regulation of cell migration involved in sprouting angiogenesis	1	1	0.0004101	0.0082025	[SRPX2]
GO:0016559	peroxisome fission	2	1	0.0008201	0.0093725	[FIS1]
GO:0043653	mitochondrial fragmentation involved in apoptosis	2	1	0.0008201	0.0093725	[FIS1]
GO:0000422	mitochondrion degradation	3	1	0.0012299	0.0081993	[FIS1]
GO:0000395	nuclear mRNA 5'-splice site recognition	3	1	0.0012299	0.0081993	[PSIP1]
GO:0032471	reduction of endoplasmic reticulum calcium ion concentration	3	1	0.0012299	0.0081993	[FIS1]
GO:0035584	calcium-mediated signaling using intracellular calcium source	4	1	0.0016395	0.0069034	[FIS1]
GO:0001836	release of cytochrome c from mitochondria	4	1	0.0016395	0.0069034	[FIS1]
GO:0051561	elevation of mitochondrial calcium ion concentration	4	1	0.0016395	0.0069034	[FIS1]
GO:0000266	mitochondrial fission	4	1	0.0016395	0.0069034	[FIS1]
GO:0048870	cell motility	7	1	0.0028675	0.0095585	[SRPX2]
GO:0006612	protein targeting to membrane	8	1	0.0032766	0.0097083	[ATG4C]
GO:0042325	regulation of phosphorylation	8	1	0.0032766	0.0097083	[SRPX2]
GO:0042273	ribosomal large subunit biogenesis	10	1	0.0040941	0.0109176	[RPL14]
GO:0009408	response to heat	11	1	0.0045027	0.0109155	[PSIP1]
GO:0000045	autophagic vacuole assembly	11	1	0.0045027	0.0109155	[ATG4C]
GO:0043280	positive regulation of cysteine-type endopeptidase activity involved in apoptotic process	14	1	0.0057273	0.0127274	[FIS1]
GO:2001244	positive regulation of intrinsic apoptotic signaling pathway	15	1	0.0061352	0.0129163	[FIS1]
GO:0044419	interspecies interaction between organisms	315	2	0.0072821	0.0145642	[PDCD6IP, PSIP1]
GO:0015031	protein transport	383	2	0.0105959	0.0201826	[ATG4C, PDCD6IP]
GO:0007204	elevation of cytosolic calcium ion concentration	28	1	0.0114237	0.0207703	[FIS1]
GO:0006914	autophagy	36	1	0.0146648	0.0255041	[ATG4C]
GO:0006979	response to oxidative stress	40	1	0.0162817	0.0271361	[PSIP1]
GO:0030036	actin cytoskeleton organization	43	1	0.0174927	0.0279883	[FERMT2]
GO:0016337	cell-cell adhesion	51	1	0.0207151	0.0318693	[SRPX2]
GO:0008150	biological_process	588	2	0.0237901	0.0352445	[FAM189B, TMEM106C]

GO:0008360	regulation of cell shape	67	1	0.0271299	0.038757	[FERMT2]
GO:0006364	rRNA processing	80	1	0.0323128	0.0445693	[RPL14]
GO:0000398	nuclear mRNA splicing, via spliceosome	98	1	0.039446	0.0525946	[HNRNPC]
GO:0001525	angiogenesis	102	1	0.0410244	0.0529347	[SRPX2]
GO:0006412	translation	130	1	0.0520048	0.065006	[RPL14]
GO:0045944	positive regulation of transcription from RNA polymerase II promoter	158	1	0.0628663	0.0762016	[PSIP1]
GO:0051301	cell division	285	1	0.1106725	0.130203	[PDCD6IP]
GO:0006810	transport	395	1	0.1502088	0.1716672	[ETFA]
GO:0007049	cell cycle	405	1	0.1537195	0.1707995	[PDCD6IP]
GO:0007155	cell adhesion	420	1	0.1589599	0.1718485	[FERMT2]
GO:0006811	ion transport	455	1	0.1710684	0.180072	[CHRNA1]
GO:0006508	proteolysis	476	1	0.1782543	0.182825	[ATG4C]
GO:0006355	regulation of transcription, DNA-dependent	1254	1	0.4066022	0.4066022	[PSIP1]

Table S8-4. GO annotation analysis of the module eigengene in six prenatal highly LDE-specific modules (LDE_pink).

GO ID	GO Name	Total Count	Diff Count	P Value	FDR	Gene
GO:0043462	regulation of ATPase activity	2	2	3.11494E-07	9.96781E-06	[TNNT3, TPM2]
GO:0030035	microspike assembly	2	1	0.001138926	0.012148545	[ACTN2]
GO:0051963	regulation of synapse assembly	2	1	0.001138926	0.012148545	[PDLIM5]
GO:0002376	immune system process	2	1	0.001138926	0.012148545	[ADSSL1]
GO:0005978	glycogen biosynthetic process	3	1	0.001707922	0.009937001	[GYS1]
GO:0006167	AMP biosynthetic process	3	1	0.001707922	0.009937001	[ADSSL1]
GO:0006941	striated muscle contraction	4	1	0.002276607	0.009106428	[PGAM2]
GO:0006942	regulation of striated muscle contraction	4	1	0.002276607	0.009106428	[TNNT3]
GO:0003009	skeletal muscle contraction	4	1	0.002276607	0.009106428	[TNNT3]
GO:0045184	establishment of protein localization	6	1	0.003413044	0.010401657	[ANK3]
GO:0061001	regulation of dendritic spine morphogenesis	6	1	0.003413044	0.010401657	[PDLIM5]
GO:0006644	phospholipid metabolic process	7	1	0.003980796	0.010615456	[AGPAT2]
GO:0031929	TOR signaling cascade	8	1	0.004548238	0.011195662	[EIF4EBP1]
GO:0080111	DNA demethylation	9	1	0.005115369	0.011692272	[APOBEC2]
GO:0048041	focal adhesion assembly	12	1	0.006814903	0.01453846	[ACTN2]
GO:0045931	positive regulation of mitotic cell cycle	16	1	0.009076613	0.017603128	[EIF4EBP1]
GO:0006164	purine nucleotide biosynthetic process	16	1	0.009076613	0.017603128	[ADSSL1]
GO:0000082	G1/S transition of mitotic cell cycle	22	1	0.01245991	0.02215095	[EIF4EBP1]
GO:0009117	nucleotide metabolic process	23	1	0.013022713	0.02193299	[AMPD1]
GO:0051289	protein homotetramerization	25	1	0.014147396	0.022635834	[ACTN2]
GO:0006096	glycolysis	37	1	0.020869704	0.031801454	[PGAM2]
GO:0006120	mitochondrial electron transport, NADH to ubiquinone	42	1	0.023657659	0.03441114	[NDUFB9]
GO:0008654	phospholipid biosynthetic process	44	1	0.024770705	0.03446359	[AGPAT2]
GO:0042981	regulation of apoptotic process	65	1	0.036384391	0.048512521	[ACTN2]
GO:0008150	biological_process	588	2	0.043875874	0.056161119	[C6orf62, NRAP]
GO:0008219	cell death	136	1	0.074674478	0.09190705	[FOLR1]
GO:0007165	signal transduction	141	1	0.077315185	0.091632812	[IGSF1]
GO:0006397	mRNA processing	144	1	0.078896136	0.090167012	[APOBEC2]
GO:0006810	transport	395	1	0.202345827	0.223278153	[NDUFB9]
GO:0007155	cell adhesion	420	1	0.213732364	0.227981188	[ACTN2]

GO:0006811	ion transport	455	1	0.229411676	0.236812053	[CACNG1]
GO:0006355	regulation of transcription, DNA-dependent	1254	1	0.515630047	0.515630047	[IGSF1]

Table S8-5. GO annotation analysis of the module eigengene in six prenatal highly LDE-specific modules (LDE_red).

GO ID	GO Name	Total Count	Diff Count	P Value	FDR	Gene
GO:0032981	mitochondrial respiratory chain complex I assembly	11	2	2.645E-05	0.0010315	[NDUFS4, NDUFS8]
GO:0006120	mitochondrial electron transport, NADH to ubiquinone	42	2	0.0004084	0.0079647	[NDUFS4, NDUFS8]
GO:0009308	amine metabolic process	1	1	0.0007063	0.0091823	[AOC3]
GO:0006167	AMP biosynthetic process	3	1	0.0021175	0.020646	[ADSL]
GO:0045647	negative regulation of erythrocyte differentiation	4	1	0.0028224	0.0220148	[MAFB]
GO:0051591	response to cAMP	6	1	0.0042307	0.0253844	[NDUFS4]
GO:0032869	cellular response to insulin stimulus	6	1	0.0042307	0.0253844	[SLC2A4]
GO:0015758	glucose transport	7	1	0.0049342	0.0226391	[SLC2A4]
GO:0051924	regulation of calcium ion transport	7	1	0.0049342	0.0226391	[SLN]
GO:0019933	cAMP-mediated signaling	8	1	0.0056371	0.0219848	[NDUFS4]
GO:0048146	positive regulation of fibroblast proliferation	9	1	0.0063396	0.0224767	[NDUFS4]
GO:0046677	response to antibiotic	11	1	0.0077431	0.0232293	[AOC3]
GO:0035329	hippo signaling cascade	11	1	0.0077431	0.0232293	[TEAD1]
GO:0045333	cellular respiration	11	1	0.0077431	0.0232293	[NDUFS4]
GO:0006936	muscle contraction	12	1	0.0084441	0.0219547	[MYH2]
GO:0072593	reactive oxygen species metabolic process	14	1	0.0098448	0.0239966	[NDUFS4]
GO:0051262	protein tetramerization	16	1	0.0112435	0.0243609	[ADSL]
GO:0045931	positive regulation of mitotic cell cycle	16	1	0.0112435	0.0243609	[USP2]
GO:0006164	purine nucleotide biosynthetic process	16	1	0.0112435	0.0243609	[ADSL]
GO:0016310	phosphorylation	20	1	0.0140352	0.0273686	[UGP2]
GO:0016579	protein deubiquitination	29	1	0.0202886	0.0376789	[USP2]
GO:0050821	protein stabilization	31	1	0.0216731	0.0384204	[USP2]
GO:0007420	brain development	33	1	0.0230556	0.0390943	[NDUFS4]
GO:0008643	carbohydrate transport	35	1	0.0244363	0.0397089	[SLC2A4]
GO:0009968	negative regulation of signal transduction	36	1	0.0251259	0.0384278	[RGS5]
GO:0006914	autophagy	36	1	0.0251259	0.0384278	[ATG4A]
GO:0007517	muscle organ development	38	1	0.0265037	0.0382831	[USP2]
GO:0006979	response to oxidative stress	40	1	0.0278796	0.0388323	[NDUFS8]
GO:0006810	transport	395	2	0.0316386	0.0425485	[NDUFS4, NDUFS8]

GO:0007049	cell cycle	405	2	0.033119	0.0430547	[PPP6C, USP2]
GO:0006816	calcium ion transport	50	1	0.0347311	0.043694	[SLN]
GO:0007186	G-protein coupled receptor signaling pathway	51	1	0.0354137	0.0431604	[LPAR6]
GO:0006508	proteolysis	476	2	0.0443805	0.0524497	[ATG4A, USP2]
GO:0000122	negative regulation of transcription from RNA polymerase II promoter	117	1	0.0794454	0.0911286	[USP2]
GO:0006954	inflammatory response	128	1	0.0865923	0.0964886	[AOC3]
GO:0006412	translation	130	1	0.087886	0.0952098	[MRPL45]
GO:0015031	protein transport	383	1	0.2379964	0.250861	[ATG4A]
GO:0007155	cell adhesion	420	1	0.2578385	0.2646238	[AOC3]
GO:0008150	biological_process	588	1	0.3418166	0.3418166	[WBSCR22]

Table S8-6 GO annotation analysis of the module eigengene in six prenatal highly LDE-specific modules (LDE_tan).

GO ID	GO Name	Total Count	Diff Count	P Value	FDR	Gene
GO:0000462	maturation of SSU-rRNA from tricistronic rRNA transcript (SSU-rRNA, 5.8S rRNA, LSU-rRNA)	1	1	0.000318987	0.006379731	[NOP14]
GO:0097045	phosphatidylserine exposure on blood platelet	1	1	0.000318987	0.006379731	[ANO6]
GO:0002543	activation of blood coagulation via clotting cascade	1	1	0.000318987	0.006379731	[ANO6]
GO:0000480	endonucleolytic cleavage in 5'-ETS of tricistronic rRNA transcript (SSU-rRNA, 5.8S rRNA, LSU-rRNA)	2	1	0.000637879	0.004639117	[NOP14]
GO:0007512	adult heart development	2	1	0.000637879	0.004639117	[MEF2D]
GO:0000472	endonucleolytic cleavage to generate mature 5'-end of SSU-rRNA from (SSU-rRNA, 5.8S rRNA, LSU-rRNA)	2	1	0.000637879	0.004639117	[NOP14]
GO:0000447	endonucleolytic cleavage in ITS1 to separate SSU-rRNA from 5.8S rRNA and LSU-rRNA from tricistronic rRNA transcript (SSU-rRNA, 5.8S rRNA, LSU-rRNA)	2	1	0.000637879	0.004639117	[NOP14]
GO:0033687	osteoblast proliferation	3	1	0.000956676	0.004251894	[OSR2]
GO:0006636	unsaturated fatty acid biosynthetic process	3	1	0.000956676	0.004251894	[DECR2]
GO:0070681	glutaminyl-tRNAGln biosynthesis via transamidation	3	1	0.000956676	0.004251894	[GATC]
GO:0061029	eyelid development in camera-type eye	4	1	0.001275379	0.004081214	[OSR2]
GO:0042474	middle ear morphogenesis	4	1	0.001275379	0.004081214	[OSR2]
GO:0007253	cytoplasmic sequestering of NF-kappaB	4	1	0.001275379	0.004081214	[G3BP2]
GO:0060349	bone morphogenesis	4	1	0.001275379	0.004081214	[OSR2]
GO:0032543	mitochondrial translation	5	1	0.001593988	0.004250635	[GATC]
GO:0048704	embryonic skeletal system morphogenesis	7	1	0.002230922	0.005577306	[OSR2]
GO:0001656	metanephros development	8	1	0.002549248	0.005998231	[OSR2]
GO:0001823	mesonephros development	10	1	0.003185617	0.007079148	[OSR2]
GO:0007265	Ras protein signal transduction	11	1	0.003503659	0.007186994	[G3BP2]
GO:0042274	ribosomal small subunit biogenesis	11	1	0.003503659	0.007186994	[NOP14]
GO:0060021	palate development	12	1	0.003821608	0.007279253	[OSR2]
GO:0050679	positive regulation of epithelial cell proliferation	15	1	0.004774889	0.008681616	[OSR2]
GO:0045893	positive regulation of transcription, DNA-dependent	334	2	0.004946192	0.008602072	[OSR2, SS18L1]
GO:0042476	odontogenesis	17	1	0.005409938	0.009016564	[OSR2]
GO:0031398	positive regulation of protein ubiquitination	22	1	0.006995917	0.011193467	[TBC1D7]
GO:0009790	embryo development	31	1	0.009844762	0.015145788	[OSR2]

GO:0010628	positive regulation of gene expression	49	1	0.015519699	0.022992146	[OSR2]
GO:0051028	mRNA transport	60	1	0.018972832	0.027104045	[G3BP2]
GO:0006364	rRNA processing	80	1	0.025222458	0.034789597	[NOP14]
GO:0008284	positive regulation of cell proliferation	102	1	0.032054335	0.042739113	[OSR2]
GO:0045944	positive regulation of transcription from RNA polymerase II promoter	158	1	0.049244458	0.063541236	[MEF2D]
GO:0007399	nervous system development	214	1	0.06615074	0.082688426	[MEF2D]
GO:0016568	chromatin modification	221	1	0.068244285	0.082720345	[SS18L1]
GO:0044419	interspecies interaction between organisms	315	1	0.095938663	0.112869015	[EIF4H]
GO:0006915	apoptotic process	321	1	0.097680172	0.111634483	[MEF2D]
GO:0006810	transport	395	1	0.118904216	0.132115796	[G3BP2]
GO:0006811	ion transport	455	1	0.13577158	0.146780087	[ANO6]
GO:0008150	biological_process	588	1	0.17209761	0.181155379	[GLTSCR2]
GO:0007275	multicellular organismal development	822	1	0.232589221	0.238553047	[MEF2D]
GO:0006355	regulation of transcription, DNA-dependent	1254	1	0.333620463	0.333620463	[MEF2D]

Table S9-1. GO annotation analysis of the module eigengene in five prenatal highly LT-specific modules (LT_blue).

GO ID	GO Name	Total Count	Diff Count	P Value	FDR	Gene
GO:0006397	mRNA processing	144	5	2.078E-05	0.0026805	[BCAS2, DHX15, LUC7L3, PTBP2, RNPC3]
GO:0006950	response to stress	104	4	9.795E-05	0.0063176	[HSP90AB1, HSPA4, HSPB11, LUC7L3]
GO:0032436	positive regulation of proteasomal ubiquitin-dependent pr	12	2	0.0003342	0.0143708	[GNB2L1, RCHY1]
GO:0030282	bone mineralization	13	2	0.0003944	0.0127188	[SBDS, TUFT1]
GO:0031398	positive regulation of protein ubiquitination	22	2	0.0011525	0.0297335	[RCHY1, TBC1D7]
GO:0042637	catagen	1	1	0.0022785	0.0279927	[TGFB2]
GO:0043148	mitotic spindle stabilization	1	1	0.0022785	0.0279927	[SBDS]
GO:0007220	Notch receptor processing	1	1	0.0022785	0.0279927	[APH1A]
GO:0045823	positive regulation of heart contraction	1	1	0.0022785	0.0279927	[TGFB2]
GO:0051795	positive regulation of catagen	1	1	0.0022785	0.0279927	[TGFB2]
GO:0048103	somatic stem cell division	1	1	0.0022785	0.0279927	[TGFB2]
GO:0031293	membrane protein intracellular domain proteolysis	1	1	0.0022785	0.0279927	[APH1A]
GO:0010611	regulation of cardiac muscle hypertrophy	1	1	0.0022785	0.0279927	[LMCD1]
GO:0070072	vacuolar proton-transporting V-type ATPase complex assembly	1	1	0.0022785	0.0279927	[VMA21]
GO:0006517	protein deglycosylation	1	1	0.0022785	0.0279927	[AGA]
GO:0006986	response to unfolded protein	35	2	0.0029118	0.0234762	[HSP90AB1, HSPA4]
GO:0006412	translation	130	3	0.0033301	0.0252694	[AARSD1, RPL36, RPSA]
GO:0051781	positive regulation of cell division	41	2	0.0039774	0.0285045	[TGFB2, TGFB3]
GO:0006839	mitochondrial transport	2	1	0.0045518	0.024466	[KIAA1279]
GO:0032464	positive regulation of protein homooligomerization	2	1	0.0045518	0.024466	[GNB2L1]
GO:0060038	cardiac muscle cell proliferation	2	1	0.0045518	0.024466	[TGFB2]
GO:0010002	cardioblast differentiation	2	1	0.0045518	0.024466	[TGFB2]
GO:0000042	protein targeting to Golgi	2	1	0.0045518	0.024466	[RAB6A]
GO:0042987	amyloid precursor protein catabolic process	2	1	0.0045518	0.024466	[APH1A]
GO:0070886	positive regulation of calcineurin-NFAT signaling cascade	2	1	0.0045518	0.024466	[LMCD1]
GO:0050778	positive regulation of immune response	2	1	0.0045518	0.024466	[TGFB2]
GO:0051302	regulation of cell division	2	1	0.0045518	0.024466	[GNB2L1]
GO:0051891	positive regulation of cardioblast differentiation	2	1	0.0045518	0.024466	[TGFB2]
GO:0050765	negative regulation of phagocytosis	2	1	0.0045518	0.024466	[GNB2L1]
GO:0008380	RNA splicing	150	3	0.0049663	0.0213549	[BCAS2, LUC7L3, RNPC3]

GO:0016445	somatic diversification of immunoglobulins	3	1	0.00682	0.0266601	[CTNNBL1]
GO:0042256	mature ribosome assembly	3	1	0.00682	0.0266601	[SBDS]
GO:0042416	dopamine biosynthetic process	3	1	0.00682	0.0266601	[TGFB2]
GO:0048539	bone marrow development	3	1	0.00682	0.0266601	[SBDS]
GO:0031340	positive regulation of vesicle fusion	3	1	0.00682	0.0266601	[ANXA1]
GO:0071569	protein ufmylation	4	1	0.0090831	0.0312459	[UFM1]
GO:0009303	rRNA transcription	4	1	0.0090831	0.0312459	[RNASEK]
GO:0016071	mRNA metabolic process	4	1	0.0090831	0.0312459	[PCBP1]
GO:0051457	maintenance of protein location in nucleus	4	1	0.0090831	0.0312459	[ARL2BP]
GO:0031069	hair follicle morphogenesis	5	1	0.0113411	0.0344236	[TGFB2]
GO:0033119	negative regulation of RNA splicing	5	1	0.0113411	0.0344236	[PTBP2]
GO:0008154	actin polymerization or depolymerization	5	1	0.0113411	0.0344236	[ABI1]
GO:0030595	leukocyte chemotaxis	5	1	0.0113411	0.0344236	[SBDS]
GO:0043547	positive regulation of GTPase activity	5	1	0.0113411	0.0344236	[GNB2L1]
GO:0048699	generation of neurons	5	1	0.0113411	0.0344236	[TGFB2]
GO:0015031	protein transport	383	4	0.0116122	0.0325646	[ARF5, RAB6A, SFT2D1, TRAM1]
GO:0050777	negative regulation of immune response	6	1	0.013594	0.0365338	[TGFB2]
GO:0045184	establishment of protein localization	6	1	0.013594	0.0365338	[PLEKHA1]
GO:2000543	positive regulation of gastrulation	6	1	0.013594	0.0365338	[GNB2L1]
GO:0006364	rRNA processing	80	2	0.0144706	0.0373342	[SBDS, UTP18]
GO:0043065	positive regulation of apoptotic process	83	2	0.0155148	0.0392434	[CTNNBL1, GNB2L1]
GO:0031529	ruffle organization	7	1	0.0158418	0.0371562	[PLEKHA1]
GO:0032880	regulation of protein localization	7	1	0.0158418	0.0371562	[GNB2L1]
GO:0048666	neuron development	7	1	0.0158418	0.0371562	[TGFB2]
GO:2000114	regulation of establishment of cell polarity	7	1	0.0158418	0.0371562	[GNB2L1]
GO:0043525	positive regulation of neuron apoptosis	7	1	0.0158418	0.0371562	[TGFB2]
GO:0000387	spliceosomal snRNP assembly	7	1	0.0158418	0.0371562	[SNRPC]
GO:0045429	positive regulation of nitric oxide biosynthetic process	7	1	0.0158418	0.0371562	[HSP90AB1]
GO:0006916	anti-apoptosis	84	2	0.0158699	0.0346986	[ANXA1, ANXA4]
GO:0070301	cellular response to hydrogen peroxide	8	1	0.0180845	0.0385604	[PLEKHA1]
GO:0001942	hair follicle development	8	1	0.0180845	0.0385604	[TGFB2]
GO:0042517	positive regulation of tyrosine phosphorylation of Stat3 protein	9	1	0.0203222	0.0416121	[ARL2BP]
GO:0014065	phosphatidylinositol 3-kinase cascade	9	1	0.0203222	0.0416121	[PLEKHA1]

GO:0097067	cellular response to thyroid hormone stimulus	9	1	0.0203222	0.0416121	[CTSB]
GO:0045332	phospholipid translocation	10	1	0.0225548	0.0440845	[ATP11A]
GO:0001654	eye development	10	1	0.0225548	0.0440845	[TGFB2]
GO:0045787	positive regulation of cell cycle	10	1	0.0225548	0.0440845	[TGFB2]
GO:0016192	vesicle-mediated transport	104	2	0.0236784	0.0449193	[ARF5, RAB6A]
GO:0006508	proteolysis	476	4	0.0237133	0.0443336	[CTSB, PSMC1, SPCS2, TMPRSS11F]
GO:0018149	peptide cross-linking	11	1	0.0247824	0.0456705	[ANXA1]
GO:0016925	protein sumoylation	12	1	0.027005	0.048384	[UBA2]
GO:0051898	negative regulation of protein kinase B signaling cascade	12	1	0.027005	0.048384	[PLEKHA1]
GO:0000902	cell morphogenesis	12	1	0.027005	0.048384	[TGFB2]
GO:0006446	regulation of translational initiation	13	1	0.0292225	0.0506001	[EIF4G2]
GO:0006730	one-carbon metabolic process	13	1	0.0292225	0.0506001	[MAT2A]
GO:0005977	glycogen metabolic process	14	1	0.0314351	0.052664	[PPP1CB]
GO:0017148	negative regulation of translation	14	1	0.0314351	0.052664	[GNB2L1]
GO:0030199	collagen fibril organization	14	1	0.0314351	0.052664	[LUM]
GO:2001244	positive regulation of intrinsic apoptotic signaling pathway	15	1	0.0336427	0.0539118	[GNB2L1]
GO:0042060	wound healing	15	1	0.0336427	0.0539118	[TGFB2]
GO:0048511	rhythmic process	15	1	0.0336427	0.0539118	[GNB2L1]
GO:0030593	neutrophil chemotaxis	15	1	0.0336427	0.0539118	[TGFB2]
GO:0044419	interspecies interaction between organisms	315	3	0.0355689	0.0552819	[GNB2L1, PSMB4, YWHAE]
GO:0006164	purine nucleotide biosynthetic process	16	1	0.0358452	0.055048	[ATIC]
GO:0030178	negative regulation of Wnt receptor signaling pathway	17	1	0.0380428	0.057398	[GNB2L1]
GO:0042476	odontogenesis	17	1	0.0380428	0.057398	[TUFT1]
GO:0030155	regulation of cell adhesion	18	1	0.0402355	0.0593186	[PPP1CB]
GO:0042787	protein ubiquitination involved in ubiquitin-dependent protein catabolic process	18	1	0.0402355	0.0593186	[RCHY1]
GO:0007165	signal transduction	141	2	0.0413417	0.0599222	[ANXA1, ANXA4]
GO:0050853	B cell receptor signaling pathway	19	1	0.0424232	0.0608065	[PLEKHA1]
GO:0001934	positive regulation of protein phosphorylation	21	1	0.0467838	0.0663198	[GNB2L1]
GO:0051726	regulation of cell cycle	22	1	0.0489567	0.0682747	[GNB2L1]
GO:0000375	RNA splicing, via transesterification reactions	22	1	0.0489567	0.0682747	[BCAS2]
GO:0006464	protein modification process	24	1	0.0532878	0.073129	[RABGGTB]
GO:0051865	protein autoubiquitination	25	1	0.0554461	0.0752899	[RCHY1]
GO:0000184	nuclear-transcribed mRNA catabolic process	27	1	0.0597479	0.0802863	[PARN]

GO:0006810	transport	395	3	0.0618283	0.0822252	[CD36, NDUFC2, UCP2]
GO:0030216	keratinocyte differentiation	29	1	0.0640304	0.0838571	[ANXA1]
GO:0030097	hemopoiesis	29	1	0.0640304	0.0838571	[TGFB2]
GO:0030307	positive regulation of cell growth	30	1	0.0661644	0.0853521	[TGFB2]
GO:0006468	protein phosphorylation	185	2	0.0668901	0.0854339	[MAPK6, OXSR1]
GO:0009790	embryo development	31	1	0.0682936	0.08595	[TGFB2]
GO:0006928	cellular component movement	31	1	0.0682936	0.08595	[ABI1]
GO:0007507	heart development	37	1	0.0809681	0.1004316	[TGFB2]
GO:0022900	electron transport chain	41	1	0.0893228	0.1097394	[NDUFC2]
GO:0006120	mitochondrial electron transport, NADH to ubiquinone	42	1	0.0913997	0.1112317	[NDUFC2]
GO:0007243	intracellular protein kinase cascade	45	1	0.0976023	0.11767	[OXSR1]
GO:0030335	positive regulation of cell migration	46	1	0.0996605	0.1190389	[GNB2L1]
GO:0040008	regulation of growth	51	1	0.109882	0.1300438	[GNB2L1]
GO:0030308	negative regulation of cell growth	58	1	0.1239993	0.1447594	[GNB2L1]
GO:0005975	carbohydrate metabolic process	58	1	0.1239993	0.1447594	[PPP1CB]
GO:0042981	regulation of apoptotic process	65	1	0.137895	0.1588255	[CTSB]
GO:0008150	biological_process	588	3	0.1508998	0.1722662	[KIAA1191, PDGFRL, SDC2]
GO:0006897	endocytosis	83	1	0.1726327	0.1953475	[MARCH3]
GO:0008283	cell proliferation	90	1	0.1857643	0.2083791	[SBDS]
GO:0000398	nuclear mRNA splicing, via spliceosome	98	1	0.2005194	0.2229914	[SNRPC]
GO:0016567	protein ubiquitination	99	1	0.2023451	0.2230984	[RCHY1]
GO:0008284	positive regulation of cell proliferation	102	1	0.2077974	0.2252593	[TGFB2]
GO:0008285	negative regulation of cell proliferation	102	1	0.2077974	0.2252593	[TGFB2]
GO:0001525	angiogenesis	102	1	0.2077974	0.2252593	[TGFB2]
GO:0007049	cell cycle	405	2	0.2356928	0.2512758	[MAPK6, PPP1CB]
GO:0008219	cell death	136	1	0.2670698	0.2823935	[TGFB2]
GO:0007399	nervous system development	214	1	0.386969	0.4058455	[KIAA1279]
GO:0007275	multicellular organismal development	822	3	0.4392374	0.4569486	[GNB2L1, KIAA1279, MYOG]
GO:0051301	cell division	285	1	0.4791125	0.4944442	[PPP1CB]
GO:0006915	apoptotic process	321	1	0.5204517	0.5328434	[LUC7L3]
GO:0007155	cell adhesion	420	1	0.618124	0.6278582	[CD36]
GO:0006355	regulation of transcription, DNA-dependent	1254	3	0.7639818	0.7699504	[ELOF1, HNRPDL, LMCD1]
GO:0030154	cell differentiation	484	1	1	1	[KIAA1279]

Table S9-2. GO annotation analysis of the module eigengene in five prenatal highly LT-specific modules (LT_darkred).

GO ID	GO Name	Total Count	Diff Count	P Value	FDR	Gene
GO:0019439	aromatic compound catabolic process	2	1	0.000683434	0.015035538	[EPHX1]
GO:0046903	secretion	3	1	0.001024987	0.011274855	[TPD52]
GO:0007253	cytoplasmic sequestering of NF-kappaB	4	1	0.001366431	0.010020495	[G3BP2]
GO:0006200	ATP catabolic process	6	1	0.002048993	0.011269462	[ATP5O]
GO:0006754	ATP biosynthetic process	8	1	0.002731119	0.010924478	[ATP5O]
GO:0071230	cellular response to amino acid stimulus	8	1	0.002731119	0.010924478	[RRAGD]
GO:0007265	Ras protein signal transduction	11	1	0.003753493	0.011796693	[G3BP2]
GO:0032008	positive regulation of TOR signaling cascade	12	1	0.004094067	0.011258684	[RRAGD]
GO:0034613	cellular protein localization	13	1	0.004434532	0.010839968	[RRAGD]
GO:0042776	mitochondrial ATP synthesis coupled proton transport	14	1	0.004774889	0.010504755	[ATP5O]
GO:0009636	response to toxin	17	1	0.005795307	0.011590613	[EPHX1]
GO:0030183	B cell differentiation	18	1	0.006135229	0.01124792	[TPD52]
GO:0006810	transport	395	2	0.007851004	0.013286314	[FAM21A, G3BP2]
GO:0042147	retrograde transport, endosome to Golgi	24	1	0.008172486	0.012842478	[FAM21A]
GO:0015992	proton transport	28	1	0.009528491	0.01397512	[ATP5O]
GO:0051028	mRNA transport	60	1	0.020314407	0.02793231	[G3BP2]
GO:0006935	chemotaxis	71	1	0.023996677	0.031054523	[CMTM3]
GO:0006364	rRNA processing	80	1	0.02699983	0.032999792	[RPF1]
GO:0009615	response to virus	114	1	0.038267417	0.044309641	[PSMA2]
GO:0006412	translation	130	1	0.043527564	0.04788032	[MRPL43]
GO:0006915	apoptotic process	321	1	0.10428176	0.109247558	[NTN1]
GO:0006811	ion transport	455	1	0.144733949	0.144733949	[ATP5O]

Table S9-3. GO annotation analysis of the module eigengene in five prenatal highly LT-specific modules (LT_only_turquoise).

GO ID	GO Name	Total Count	Diff Count	P Value	FDR	Gene
GO:0042359	vitamin D metabolic process	1	1	0.0001367	0.000820251	[CYP11A1]
GO:0006700	C21-steroid hormone biosynthetic process	1	1	0.0001367	0.000820251	[CYP11A1]
GO:0043171	peptide catabolic process	3	1	0.0004101	0.001230236	[LTA4H]
GO:0030163	protein catabolic process	14	1	0.0019125	0.00430313	[TPRKB]
GO:0019370	leukotriene biosynthetic process	20	1	0.0027312	0.004916183	[LTA4H]
GO:0008203	cholesterol metabolic process	24	1	0.0032767	0.004915063	[CYP11A1]
GO:0006869	lipid transport	46	1	0.0062725	0.008064634	[STAR]
GO:0006810	transport	395	1	0.0528023	0.059402578	[STAR]
GO:0006508	proteolysis	476	1	0.0633377	0.063337684	[LTA4H]

Table S9-4. GO annotation analysis of the module eigengene in five prenatal highly LT-specific modules (LT_purple).

GO ID	GO Name	Total Count	Diff Count	P Value	FDR	Gene
GO:0071310	cellular response to organic substance	1	1	0.000797466	0.040670783	[CLEC3B]
GO:0043555	regulation of translation in response to stress	2	1	0.001594315	0.023231445	[RPS6KA3]
GO:0010756	positive regulation of plasminogen activation	2	1	0.001594315	0.023231445	[CLEC3B]
GO:0006531	aspartate metabolic process	2	1	0.001594315	0.023231445	[GOT2]
GO:0030035	microspike assembly	2	1	0.001594315	0.023231445	[ACTN2]
GO:0015908	fatty acid transport	3	1	0.002390546	0.018756592	[GOT2]
GO:0006533	aspartate catabolic process	3	1	0.002390546	0.018756592	[GOT2]
GO:0006950	response to stress	104	2	0.003144315	0.020045006	[HSPB6, RPS6KA3]
GO:0043620	regulation of DNA-dependent transcription in response to stress	4	1	0.00318616	0.017104651	[RPS6KA3]
GO:0045647	negative regulation of erythrocyte differentiation	4	1	0.00318616	0.017104651	[MAFB]
GO:0006810	transport	395	3	0.003824554	0.017732021	[GOT2, NDUFB9, SDHD]
GO:0007155	cell adhesion	420	3	0.004537771	0.019285526	[ACTN2, CD99, CDH13]
GO:0032869	cellular response to insulin stimulus	6	1	0.00477554	0.017396611	[SLC2A4]
GO:0002224	toll-like receptor signaling pathway	6	1	0.00477554	0.017396611	[RPS6KA3]
GO:0045184	establishment of protein localization	6	1	0.00477554	0.017396611	[ANK3]
GO:0045597	positive regulation of cell differentiation	7	1	0.005569307	0.015779703	[RPS6KA3]
GO:0015758	glucose transport	7	1	0.005569307	0.015779703	[SLC2A4]
GO:0051924	regulation of calcium ion transport	7	1	0.005569307	0.015779703	[PLN]
GO:0071526	semaphorin-plexin signaling pathway	7	1	0.005569307	0.015779703	[PLXND1]
GO:0006103	2-oxoglutarate metabolic process	7	1	0.005569307	0.015779703	[GOT2]
GO:0006536	glutamate metabolic process	9	1	0.007154995	0.016972314	[GOT2]
GO:0045471	response to ethanol	9	1	0.007154995	0.016972314	[GOT2]
GO:0006099	tricarboxylic acid cycle	10	1	0.007946918	0.017246503	[SDHD]
GO:0045765	regulation of angiogenesis	10	1	0.007946918	0.017246503	[PLXND1]
GO:0006936	muscle contraction	12	1	0.009528923	0.019057847	[MYH2]
GO:0048041	focal adhesion assembly	12	1	0.009528923	0.019057847	[ACTN2]
GO:0043542	endothelial cell migration	13	1	0.010319007	0.019137067	[PLXND1]
GO:0030282	bone mineralization	13	1	0.010319007	0.019137067	[CLEC3B]
GO:0005977	glycogen metabolic process	14	1	0.011108478	0.019535599	[PPP1R3B]

GO:0071560	cellular response to transforming growth factor beta stimulus	15	1	0.011897337	0.020225473 [CLEC3B]
GO:0032496	response to lipopolysaccharide	17	1	0.013473222	0.022165624 [RPS6KA3]
GO:0007416	synapse assembly	20	1	0.015832473	0.025233004 [PLXND1]
GO:0030334	regulation of cell migration	22	1	0.017402261	0.026894404 [PLXND1]
GO:0051289	protein homotetramerization	25	1	0.019752384	0.029199176 [ACTN2]
GO:0043154	negative regulation of cysteine-type endopeptidase activity involved in apoptotic process	25	1	0.019752384	0.029199176 [RPS6KA3]
GO:0001503	ossification	29	1	0.022877388	0.032409633 [CLEC3B]
GO:0030307	positive regulation of cell growth	30	1	0.023657125	0.03260847 [RPS6KA3]
GO:0008643	carbohydrate transport	35	1	0.027546756	0.036970646 [SLC2A4]
GO:0006096	glycolysis	37	1	0.029098389	0.03805174 [ENO3]
GO:0006120	mitochondrial electron transport, NADH to ubiquinone	42	1	0.032966958	0.042032872 [NDUFB9]
GO:0006631	fatty acid metabolic process	50	1	0.039125543	0.048668359 [HADHB]
GO:0005975	carbohydrate metabolic process	58	1	0.045246021	0.054941597 [PPP1R3B]
GO:0042981	regulation of apoptotic process	65	1	0.050570359	0.059978798 [ACTN2]
GO:0001525	angiogenesis	102	1	0.078237638	0.090684535 [PLXND1]
GO:0043066	negative regulation of apoptotic process	118	1	0.089957813	0.101952188 [RPS6KA3]
GO:0007399	nervous system development	214	1	0.157297823	0.174395413 [SEMA6C]
GO:0044419	interspecies interaction between organisms	315	1	0.222915164	0.241886668 [SLC25A4]
GO:0007049	cell cycle	405	1	0.277183291	0.294507246 [RPS6KA3]
GO:0030154	cell differentiation	484	1	0.321770097	0.33490357 [SEMA6C]
GO:0008150	biological_process	588	1	0.376411033	0.383939253 [NRAP]
GO:0007275	multicellular organismal development	822	1	0.484177504	0.484177504 [SEMA6C]

Table S9-5. GO annotation analysis of the module eigengene in five prenatal highly LT-specific modules (LT_yellowgreen).

GO ID	GO Name	Total Count	Diff Count	P Value	FDR	Gene
GO:0045657	positive regulation of monocyte differentiation	1	1	0.000227848	0.005012646	[ACIN1]
GO:0006607	NLS-bearing substrate import into nucleus	2	1	0.000455648	0.005012132	[DAG1]
GO:0043409	negative regulation of MAPK cascade	3	1	0.000683402	0.004295672	[DAG1]
GO:0030263	apoptotic chromosome condensation	3	1	0.000683402	0.004295672	[ACIN1]
GO:0003009	skeletal muscle contraction	4	1	0.00091111	0.004008883	[TNNI2]
GO:0034453	microtubule anchoring	5	1	0.00113877	0.0038543	[DAG1]
GO:0007016	cytoskeletal anchoring at plasma membrane	5	1	0.00113877	0.0038543	[DAG1]
GO:0019048	virus-host interaction	7	1	0.001593952	0.004383367	[DAG1]
GO:0006509	membrane protein ectodomain proteolysis	8	1	0.001821472	0.004452488	[DAG1]
GO:0045893	positive regulation of transcription, DNA-dependent	334	2	0.002495591	0.0054903	[MED12, TNNI2]
GO:0051898	negative regulation of protein kinase B signaling cascade	12	1	0.002731088	0.005224691	[DAG1]
GO:0030518	intracellular steroid hormone receptor signaling pathway	12	1	0.002731088	0.005224691	[MED12]
GO:0030218	erythrocyte differentiation	13	1	0.002958376	0.005006482	[ACIN1]
GO:0030178	negative regulation of Wnt receptor signaling pathway	17	1	0.003867059	0.006076807	[MED12]
GO:0006367	transcription initiation from RNA polymerase II promoter	23	1	0.005228687	0.007668741	[MED12]
GO:0030336	negative regulation of cell migration	30	1	0.006815135	0.009370811	[DAG1]
GO:0030521	androgen receptor signaling pathway	38	1	0.008625431	0.011162322	[MED12]
GO:0005975	carbohydrate metabolic process	58	1	0.013138184	0.01605778	[PYGM]
GO:0006950	response to stress	104	1	0.023447433	0.027149659	[HSPB8]
GO:0008219	cell death	136	1	0.03056181	0.033617991	[HSPB8]
GO:0044419	interspecies interaction between organisms	315	1	0.069504634	0.072814379	[DAG1]
GO:0008150	biological_process	588	1	0.126191315	0.126191315	[HSPB8]

Table S10-1. Module eigengene and its evolutionary rates in 15 postnatal highly LDE-specific modules (LDE_black).

pig_Genebank	pig_Gene	LDE Module	kTotal	kWithin	eigencorr	eigenpval	meanExpr	LTModule	Ka/Ks
AF288822.1	EIF4G2	black	781.53	107.525	0.99717	0.00018	50.062	darkgreen	--
NM_001042375.1	ITM2B	black	808.6	96.2529	0.97339	0.00519	259.792	turquoise	0.2074
AK230784.1	NDUFA5	black	928.86	96.2469	0.97207	0.00558	90.554	lightcyan	--
AK240469.1	CTNNA1	black	724.66	101.258	0.98481	0.00224	3.702	white	0
AK230971.1	MATR3	black	659.18	98.8046	0.97921	0.00359	11.158	darkred	0
AK237701.1	ZNF655	black	882.7	104.508	0.99042	0.00112	5.512	blue	99
AK231179.1	DDR1	black	694.8	100.285	0.98288	0.00268	12.568	lightyellow	--
AK235131.1	U2AF1	black	912.46	103.985	0.9894	0.00131	40.874	grey60	--
AK238804.1	DYNC1I2	black	935.14	100.554	0.98194	0.00291	12.454	white	0
AK232538.1	LONP1	black	728.21	90.4931	0.96185	0.00889	19.392	turquoise	99
AK232688.1	ABCF3	black	777.01	103.917	0.99002	0.00119	11.382	grey60	0
AK239087.1	MFF	black	797.74	105.035	0.99236	0.0008	4.398	yellow	99
AK236321.1	CCT5	black	931.44	100.761	0.98228	0.00283	4.848	turquoise	--
AK239257.1	CPSF7	black	843.74	104.21	0.99002	0.0012	11.37	turquoise	--
AK230529.1	ABI1	black	897.11	103.703	0.98856	0.00147	6.568	purple	99
AK236418.1	PAF1	black	684.95	96.1495	0.97387	0.00505	12.39	turquoise	99
AK239385.1	UTP18	black	722.17	100.247	0.98249	0.00277	17.748	darkorange	0.1679
AK239707.1	TMEM179B	black	891.09	103.381	0.98872	0.00144	19.85	turquoise	--
AK239710.1	PRPSAP1	black	648.01	95.8485	0.97348	0.00516	3.632	black	0
AK236573.1	SPTBN1	black	810.81	108.844	0.99967	7.18E-06	11.844	darkgreen	--
AK236660.1	CSNK1E	black	817.62	92.6698	0.96562	0.00761	17.412	tan	0
AK239758.1	RNPS1	black	882.68	106.681	0.99495	0.00043	19.42	blue	--
AK239858.1	CD99	black	842.02	108.279	0.99838	7.86E-05	38.728	turquoise	--
AK239890.1	SRP68	black	744.42	100.325	0.98295	0.00266	27.228	turquoise	0.1645
AK233343.1	RBM10	black	818.8	103.584	0.98928	0.00133	39.262	darkmagenta	0
AK233652.1	FZD4	black	617.77	94.2642	0.96959	0.00634	29.622	turquoise	--
AK240345.1	OGDH	black	703.19	94.2175	0.97014	0.00617	97.352	turquoise	99
NM_001044575.2	IDH3B	black	903.4	101.749	0.98527	0.00214	74.618	black	0
NM_001044582.1	ZRANB2	black	836.34	104.572	0.99134	0.00097	39.268	violet	99
NM_001114275.1	NCOA2	black	504.44	78.0044	0.92864	0.02264	4.098	blue	0.0967
NM_001123101.1	TBRG4	black	698.09	103.328	0.98874	0.00143	34.612	violet	99
NM_001129954.1	RPS20	black	637.38	90.1724	0.96093	0.00921	1460.098	blue	--
XM_001926549.1	FERMT2	black	814.44	105.164	0.99227	0.00081	58.406	red	0
XM_001927513.1	MPHOSPH10	black	804.04	107.103	0.99627	0.00027	8.916	grey60	99
XM_001924618.1	TRAM1	black	735.11	104.885	0.99199	0.00086	28.946	darkgrey	99
XM_001927666.1	NDUFS2	black	943.17	96.6511	0.97333	0.00521	155.17	blue	0
XM_001927000.1	LUM	black	914.05	102.742	0.98653	0.00187	15.938	darkgrey	0.1529
XM_001929118.1	TOX4	black	854.38	103.572	0.98918	0.00135	10.732	tan	0
XM_001929613.1	RXRB	black	771	105.182	0.9926	0.00076	10.72	violet	0
XM_001929571.1	PI16	black	872.65	107.192	0.996	0.0003	6.92	darkred	0.8208
XM_001924481.1	SEC61A1	black	859.27	107.216	0.99607	0.0003	11.298	turquoise	0
XM_001928536.1	GADD45G	black	615.87	93.58	0.96728	0.00707	32.348	turquoise	99
XM_001925435.1	RBM34	black	637.43	95.7831	0.9725	0.00545	6.358	brown	0.3836

XM_001929322.1	ANK3	black	687.46	100.465	0.98289	0.00268	39.034	royalblue	0.1356
XM_001928193.1	SEC24C	black	898.77	101.191	0.98315	0.00262	10.908	turquoise	0.4432
XM_001928374.1	NDST2	black	891.23	104.103	0.98943	0.0013	15.546	darkmagenta	99
XM_001927712.1	DDX55	black	723.78	100.43	0.98319	0.00261	24.2	turquoise	0.1968
XM_001926788.1	PRPF40A	black	684.23	91.9815	0.96528	0.00773	20.748	blue	99
EU714326.1	KDR	black	719.62	104.555	0.99116	0.001	3.06	yellow	0
AF490841.1	GLS	black	840.33	105.607	0.99295	0.00071	19.942	turquoise	99
NM_001136510.1	NFS1	black	858.19	91.6007	0.96239	0.0087	28.856	pink	0
NM_213928.1	GOT2	black	901.27	103.229	0.98807	0.00156	49.376	darkred	0.0782
NM_213927.1	GOT1	black	756.9	101.19	0.98461	0.00229	65.686	pink	0
NM_213901.1	PCCB	black	722.27	104.07	0.99021	0.00116	3.39	brown	0
NM_213976.1	CD163	black	729.95	100.994	0.98435	0.00235	6.192	blue	--
NM_213980.1	UGP2	black	769.21	102.228	0.98669	0.00184	63.336	turquoise	0
NM_214333.1	RPN1	black	831.64	108.689	0.99921	2.64E-05	33.152	skyblue	0.1147
NM_214364.1	VDAC3	black	768.03	107.949	0.99798	0.00011	28.87	greenyellow	--
NM_214372.1	APP	black	761.02	102.104	0.98644	0.00189	34.022	darkturquoise	0
AY609530.1	SC65	black	654.01	93.9533	0.96863	0.00664	8.09	turquoise	0
AY609970.1	BPNT1	black	850.29	108.157	0.99816	9.45E-05	4.302	white	0.0723
AY609644.1	CNIH4	black	859.04	102.935	0.98746	0.00168	17.168	blue	99
NM_001005726.1	ANXA2	black	835.65	100.979	0.98332	0.00258	56.298	turquoise	0.3622
NM_001038644.1	ANGPTL4	black	665.56	97.007	0.97534	0.00463	17.21	turquoise	99
BX666608		black	943.41	96.507	0.97272	0.00539	7.24	tan	--
BX669885		black	814.88	108.484	0.99895	4.08E-05	29.644	yellow	99
BX674131		black	645.62	98.3957	0.97806	0.00389	11.888	turquoise	--
BX674519		black	849.52	108.299	0.99843	7.45E-05	10.93	purple	0
BX675006		black	915.01	102.271	0.98619	0.00194	13.94	greenyellow	--
BX675465		black	848.79	107.593	0.99717	0.00018	3.01	yellow	--
BX675880		black	908.67	102.121	0.9858	0.00203	10.146	magenta	--
BP440119		black	795.55	108.042	0.99817	9.38E-05	2.46	white	--
CK453192		black	826.54	106.509	0.99519	0.0004	15.7	turquoise	--
CK459106		black	927.68	99.9988	0.98052	0.00326	9.106	grey60	99
CK459361		black	803.29	108.676	0.99936	1.96E-05	10.864	darkgrey	--
CK464458		black	852.39	106.166	0.99439	0.0005	8.462	lightcyan	--
CK467496		black	829.63	105.217	0.99267	0.00075	12.454	royalblue	--
BX915635		black	592.89	88.6193	0.9559	0.01104	1.26	black	--
BX917599		black	601.9	89.0649	0.95714	0.01058	23.852	black	0
BX917687		black	746.61	103.991	0.99029	0.00115	59.016	salmon	--
BX918145		black	871.24	106.844	0.99527	0.00039	72.476	grey60	--
BX919338		black	722.13	104.914	0.99205	0.00085	4.928	grey60	99
CN156156		black	691.16	100.58	0.98293	0.00267	2.88	white	--
CN156410		black	928.46	99.4945	0.97939	0.00354	8.488	navy	0
CN156471		black	781.52	92.6689	0.96574	0.00757	23.758	turquoise	--
CN156513		black	615.46	90.214	0.95988	0.00959	0.862	lightyello[illegible]	--
CN157824		black	893.88	103.045	0.98727	0.00172	20.872	turquoise	--
CN158780		black	836.85	102.307	0.98614	0.00195	44.948	turquoise	--
CN159900		black	714.97	94.4704	0.97069	0.006	3.824		--
CN161458		black	804.71	102.09	0.98602	0.00198	64.432	blue	--

CN161563		black	942.99	98.8177	0.97813	0.00387	24.364	blue	--
CJ009698		black	527.83	81.5103	0.93771	0.01849	5.854	turquoise	0.1364
CJ029380		black	646.89	96.5461	0.9745	0.00487	9.934	turquoise	--
DN101782		black	713.56	102.056	0.98615	0.00195	17.61	lightyellow	0
DN106073		black	930.89	95.0793	0.9694	0.00639	16.624	yellow	--
DN117259		black	933.47	100.418	0.98153	0.003	22.586	turquoise	--
DN117899		black	767.91	103.526	0.98932	0.00132	14.188	blue	99
DN119433		black	693.32	99.8669	0.98183	0.00293	16.842	purple	--
DN123035		black	835.82	108.186	0.99817	9.37E-05	3.398	yellow	--
AJ944251		black	865.15	96.8644	0.97421	0.00495	23.946	tan	--
CV876303		black	723.13	104.772	0.99173	0.0009	1.314	turquoise	--
DY406144		black	771.44	98.9338	0.97992	0.00341	5.19	lightyellow	--
DB784140		black	610.22	92.0229	0.96451	0.00798	9.19	blue	--
AK230614	CD36	black	533.74	82.7755	0.94093	0.01708	9.834	darkgrey	0.3146
DT324725		black	774	107.309	0.99676	0.00022	12.236	turquoise	--
DT324752		black	934.9	100.321	0.98141	0.00303	10.494	purple	--
DT331317		black	611.95	89.9945	0.95933	0.00978	3.58	black	0
EW056660		black	685.88	101.543	0.98496	0.00221	5.222	blue	--
EW155747		black	596.44	92.0835	0.96375	0.00824	6.484	darkgrey	--
EW266972		black	915.55	99.1845	0.97869	0.00372	3.772	darkgreen	--
EW301848		black	669.31	98.6173	0.97925	0.00358	39.996	blue	--
EW422118		black	905.38	100.851	0.98253	0.00276	23.73	greenyellow	--
EW526188		black	874.59	102.806	0.98681	0.00181	17.466	blue	--
EW619519		black	913.13	103.328	0.9878	0.00162	199.596	turquoise	--
EW623428		black	600.55	92.9563	0.96583	0.00754	12.102	grey60	--
EW633584		black	907.47	102.243	0.98627	0.00193	28.73	turquoise	0.7251
EH005985		black	941.84	98.83	0.97819	0.00385	6.618	darkturquoise	--
EH006647		black	819.77	107.896	0.99769	0.00013	1.112	lightgreen	--
BQ597564		black	741.94	105.504	0.99324	0.00067	9.492	darkgrey	--
BI186101		black	896.25	104.306	0.98985	0.00123	2.084		--

Table S10-2. Module eigengene and its evolutionary rates in 15 postnatal highly LDE-specific modules (LDE_blue).

pig_Genebank	pig_Gene	LDE Module	kTotal	kWithin	eigencorr	eigenpval	meanExpr	LTModule	Ka/Ks
NM_001044576.1	NCOR2	blue	711.8619	140.112	0.980535	0.00325	22.344	lightyellow	0.075
AK234174.1	TMEM222	blue	728.1961	115.905	0.935678	0.01939	14.574	violet	99
AK234195.1	LRPPRC	blue	947.6861	140.767	0.979587	0.00349	4.464	darkred	0.138
AK234286.1	PER1	blue	362.9855	87.2395	0.874123	0.05259	41.852	black	99
AK234337.1	TBC1D17	blue	582.4931	112.976	0.933048	0.02059	13.85	darkmagenta	99
AK230834.1	TAF12	blue	676.3208	136.625	0.976132	0.00441	21.91	black	--
AK230893.1	NECAP1	blue	695.4454	142.831	0.986556	0.00187	9.226	darkmagenta	--
AK234386.1	SFT2D1	blue	391.5872	90.253	0.883185	0.04708	16.108	grey	0.225
AK237684.1	PRKAG2	blue	919.0855	149.862	0.994116	0.00054	11.368	blue	0
AK237699.1	ZDHHC18	blue	312.4915	67.2627	0.819155	0.08977	4.73	turquoise	--
AK237762.1	DCTN1	blue	809.6531	151.673	0.998719	5.5E-05	22.28	turquoise	0
AK231325.1	DDX27	blue	954.1155	135.498	0.970078	0.00619	5.398	blue	0.35
AK234837.1	C2orf29	blue	675.402	84.0399	0.866152	0.05759	22.73	turquoise	--
AK238002.1	CDC42EP1	blue	368.8324	73.5205	0.840082	0.0749	11.468	blue	99
AK231733.1	NDUFA10	blue	917.9908	130.137	0.959991	0.00955	47.492	turquoise	0.263
AK235011.1	NET1	blue	794.1531	132.086	0.96537	0.0077	18.008	turquoise	0
AK235117.1	RIOK3	blue	568.9435	102.337	0.908546	0.03274	67.994	turquoise	--
AK231946.1	PXDN	blue	620.0112	88.9984	0.884354	0.04638	14.46	blue	--
AK235252.1	MRPS35	blue	509.9605	91.5051	0.884307	0.04641	14.144	red	0.386
AK235264.1	MLYCD	blue	817.7713	151.612	0.998176	9.4E-05	9.648	turquoise	--
AK235336.1	CLPTM1L	blue	885.076	142.402	0.982549	0.00276	10.612	blue	99
AK231959.1	CRIP2	blue	544.9288	111.063	0.929095	0.02242	26.11	red	--
AK238521.1	MCAT	blue	548.8683	122.463	0.950745	0.01302	3.912	violet	99
AK232242.1	KLHDC3	blue	584.0209	109.742	0.925868	0.02396	130.588	navy	--
AK232271.1	PHB2	blue	742.8461	136.125	0.97392	0.00504	74.618	grey60	0
AK235752.1	TRAP1	blue	803.5468	147.94	0.992665	0.00075	21.066	turquoise	99
AK238694.1	GRPEL1	blue	469.0041	73.383	0.837373	0.07678	30.546	blue	0
AK235894.1	GNL3L	blue	942.6867	139.755	0.977884	0.00394	13.824	grey60	0.102
AK238836.1	EIF4H	blue	522.0341	115.046	0.93761	0.01853	118.318	blue	--
AK236053.1	BRE	blue	782.8505	150.737	0.997499	0.00015	7.59	turquoise	--
AK238961.1	AES	blue	618.6693	128.335	0.962901	0.00853	75.646	orange	--
AK239104.1	PRPF8	blue	654.7377	77.8775	0.846449	0.07054	27.258	tan	0
AK236182.1	NDUFS1	blue	738.8087	109.95	0.924352	0.02469	116.754	brown	0.121
AK236235.1	ACP2	blue	684.6111	125.644	0.956321	0.01089	6.838	tan	0.171
AK236339.1	PSMD2	blue	860.8467	130.994	0.963278	0.0084	82.006	tan	99
AK239282.1	STARD7	blue	846.72	140.753	0.980758	0.00319	41.632	blue	0
AK236459.1	BOP1	blue	567.587	106.005	0.916603	0.02855	11.562	violet	99
AK232788.1	LONP2	blue	567.5199	114.82	0.937741	0.01847	26.182	white	99
AK232832.1		blue	935.1136	144.994	0.986217	0.00194	2.656	darkgrey	--
AK239601.1	ACOT1	blue	874.3797	133.502	0.966517	0.00732	31.152	black	--
AK239677.1	WIPI2	blue	979.0333	119.963	0.940314	0.01735	20.93	sienna3	0
AK232979.1	MRPS5	blue	962.9634	137.216	0.972387	0.00549	19.366	tan	0
AK233000.1	NAPA	blue	593.0269	119.866	0.945575	0.01512	56.898	blue	99
AK233005.1	ADPRHL2	blue	799.8887	138.132	0.97558	0.00456	4.982	tan	0
AK233047.1	BRP44L	blue	723.9722	137.355	0.977782	0.00396	314.876	turquoise	--
AK236699.1	FSCN1	blue	840.5049	150.044	0.995358	0.00038	0.79	lightgreen	99

AK233231.1	NDUFS8	blue	394.4282	77.0821	0.848024	0.06948	320.59	red	--
AK240009.1	NCLN	blue	972.2354	132.526	0.964749	0.0079	5.6	lightyellow	--
AK233419.1	SLC25A4	blue	842.1794	141.916	0.98253	0.00276	1366.408	turquoise	0
AK233680.1	MRPL36	blue	360.5506	81.6222	0.861188	0.06077	6.348	grey60	99
AK240262.1	CLEC3B	blue	645.6378	136.872	0.975959	0.00446	27.836	lightcyan	--
NM_001078679.1	CD81	blue	977.7803	130.92	0.961669	0.00896	5.358	grey60	--
NM_213946.2	FHL3	blue	908.6444	140.26	0.979039	0.00363	144.588	blue	--
NM_001097420.1	EPAS1	blue	815.6891	151.559	0.998147	9.6E-05	5.734	darkturquoise	0
NM_001105296.1	RAE1	blue	666.4391	131.064	0.966151	0.00744	9.986	tan	0
NM_001099936.1	BTG1	blue	674.2153	120.725	0.948204	0.01404	32.74	turquoise	99
EU009401.2	PNPLA2	blue	908.9084	150.44	0.995558	0.00036	13.862	blue	0.084
NM_001123213.1	VAPB	blue	907.5916	150.431	0.995572	0.00035	47.842	black	99
NM_001123198.1	ST7	blue	830.0159	148.855	0.994369	0.00051	8.504	blue	0
NM_001123104.1	CTSZ	blue	371.1919	77.1001	0.851079	0.06743	22.016	turquoise	0.135
XM_001926626.1	C9orf123	blue	964.9778	144.801	0.985307	0.00213	10.3	red	--
XM_001925462.1	CPSF1	blue	393.2081	91.4359	0.88621	0.04528	21.496	tan	0
XM_001924535.1	ASAP1	blue	478.7791	109.233	0.925152	0.0243	7.206	blue	0
XM_001929031.1	ATP1A2	blue	937.8728	141.384	0.980944	0.00315	32.292	darkturquoise	0
XM_001927806.1	PRPF38B	blue	892.6852	131.086	0.962651	0.00862	26.716	turquoise	0
XM_001927255.1	TCEA1	blue	988.2354	135.142	0.968591	0.00665	7.076	turquoise	0.131
XM_001925928.1	CEBPD	blue	631.4072	134.672	0.97242	0.00548	87.986	turquoise	0
XM_001925252.1	ITGA5	blue	918.406	142.652	0.982395	0.0028	5.07	lightyellow	0
XM_001927127.1	SLC38A2	blue	890.5909	152.079	0.998153	9.5E-05	48.608	turquoise	--
XM_001925445.1	KIAA0528	blue	917.7332	150.121	0.994833	0.00045	11.596	red	--
XM_001929074.1	PTGR2	blue	841.2543	149.025	0.994	0.00056	49.842	turquoise	0.202
XM_001925833.1	IPO4	blue	996.4562	132.951	0.964618	0.00795	9.872	white	0
XM_001929568.1	SLC29A1	blue	764.7073	149.348	0.995901	0.00031	18.646	blue	0.29
XM_001929570.1	HSP90AB1	blue	542.1271	88.7429	0.875714	0.05161	260.05	turquoise	0
XM_001927338.1	IDH3A	blue	960.3707	138.67	0.975287	0.00465	185.96	red	0.176
XM_001929415.1	FKBP5	blue	647.4752	136.028	0.976047	0.00443	7.018	violet	0
XM_001928742.1	POSTN	blue	902.5341	150.395	0.995115	0.00041	19.812	turquoise	0.041
XM_001927648.1	MTG1	blue	515.1122	72.8557	0.841192	0.07413	14.07	blue	99
XM_001929110.1	ZFYVE27	blue	757.6057	147.466	0.992831	0.00073	7.522	grey60	0.196
XM_001927395.1	SFXN4	blue	933.7867	143.407	0.983743	0.00248	19.998	darkmagenta	0.881
XM_001924717.1		blue	847.47	140.219	0.979986	0.00339	16.03	turquoise	99
XM_001925999.1	VPS26A	blue	311.4586	60.6712	0.794068	0.10865	17.798	turquoise	99
XM_001928164.1	NUP133	blue	867.6606	151.267	0.997242	0.00017	4.606	grey60	0.039
XM_001925659.1	REEP3	blue	760.3197	125.607	0.953456	0.01197	3.734	yellow	99
XM_001926896.1	ERP29	blue	933.6651	136.469	0.971843	0.00565	15.704	lightcyan	99
XM_001929345.1	CORO1C	blue	983.2891	132.629	0.964358	0.00803	8.904	blue	99
XM_001925978.1	EWSR1	blue	778.387	144.586	0.987742	0.00163	19.756	turquoise	--
XM_001929539.1	PPIL2	blue	839.4861	142.231	0.983017	0.00265	5.072	black	0
XM_001925613.1	USP40	blue	721.5942	145.148	0.989099	0.00136	1.824	white	--
XM_001926406.1	ACOT9	blue	944.1672	139.727	0.976776	0.00423	21.8	grey60	0
XM_001925991.1	STAG2	blue	913.5807	150.604	0.995628	0.00035	2.094	darkred	0
NM_001130531.1	ADAM10	blue	866.2629	148.062	0.99194	0.00087	1.246	lightgreen	99
NM_214223.2	THOP1	blue	582.2376	111.062	0.929779	0.0221	5.566	grey60	0
NM_001142668.1	DKC1	blue	848.5236	148.008	0.993013	0.0007	7.682	violet	99
NM_001142666.1	TEAD4	blue	532.3492	99.9048	0.905789	0.03422	19.924	skyblue	0
AY421755.1	HMOX2	blue	542.9362	124.145	0.954382	0.01162	3.106	grey60	--
NM_213904.1	GANAB	blue	921.1179	142.106	0.981189	0.00309	15.414	black	0

NM_213962.1	HADHA	blue	959.0588	146.038	0.987424	0.00169	121.492	turquoise	0.143
NM_214031.1	ENG	blue	905.8497	151.106	0.996268	0.00027	9.49	white	0.058
NM_214062.1	DLD	blue	958.2527	136.631	0.972355	0.00549	70.504	yellow	0
NM_214200.1	PLIN2	blue	946.6493	147.299	0.989617	0.00127	5.728	blue	0.13
NM_214230.1	TNC	blue	570.4484	119.125	0.943046	0.01618	1.164	blue	0.155
NM_214323.1	JUP	blue	639.7396	125.732	0.954969	0.01139	4.032	royalblue	0
NM_214375.1	FHL1	blue	938.4858	144.818	0.986295	0.00192	527.014	turquoise	0
NM_214397.1	DLST	blue	947.1611	140.074	0.978477	0.00378	40.92	violet	99
NM_214418.1	SLC25A6	blue	600.0459	130.629	0.96612	0.00745	54.658	sienna3	--
AY609425.1	NUDCD2	blue	472.4585	60.5893	0.80893	0.09733	32.048	purple	0
AY609525.1	CBX3	blue	997.0673	123.786	0.947698	0.01425	20.698	turquoise	99
AY609565.1	MRPL1	blue	760.3924	148.26	0.993668	0.0006	7.6	lightcyan	--
AY609735.1	GPR177	blue	905.5015	128.644	0.957572	0.01042	4.898	yellow	0
AY609803.1	ECSIT	blue	899.7688	146.721	0.989194	0.00135	53.04	turquoise	0
AY609860.1	SGCE	blue	882.4418	103.183	0.906495	0.03384	14.472	turquoise	99
AY609887.1	PFDN1	blue	625.3873	79.0397	0.853466	0.06584	59.952		99
AY610104.1	MED30	blue	824.3487	142.085	0.982702	0.00272	5.286	sienna3	0
AY610107.1	CBX5	blue	985.8474	125.115	0.950189	0.01324	39.346	black	--
NM_213855.1	MYH7	blue	468.3696	91.2454	0.88625	0.04526	1927.712	midnightblue	0
CF367219		blue	975.3702	116.249	0.932942	0.02063	14.214	turquoise	--
BX665422		blue	352.8921	84.1913	0.865388	0.05808	66.802	turquoise	--
BX666448		blue	897.3659	151.768	0.997589	0.00014	150.16	turquoise	--
BX666848		blue	903.3227	142.682	0.983118	0.00263	26.74	turquoise	--
BX668759		blue	423.8834	97.5999	0.89916	0.03785	1.036	lightyellow	--
BX675832		blue	974.209	136.401	0.971001	0.0059	201.596	blue	--
BX675898		blue	598.6325	111.877	0.928654	0.02263	11.84	red	99
BX676463		blue	811.2297	147.248	0.991509	0.00094	8.488	darkmagenta	--
BP163971		blue	488.9765	95.5892	0.899954	0.03741	2.732	blue	--
CK453021		blue	972.5377	140.605	0.978387	0.0038	16.7	blue	99
CK454008		blue	897.4859	150.817	0.996406	0.00026	2.03	skyblue	--
CK458262		blue	985.2861	132.358	0.96371	0.00825	12.23	white	--
CK458404		blue	869.2834	147.742	0.991404	0.00096	5.446	black	0
BX914656		blue	728.6866	98.5461	0.900672	0.03701	35.446	turquoise	99
BX919363		blue	871.0434	150.762	0.996975	0.0002	3.11	yellow	--
BX920145		blue	657.2293	118.576	0.943926	0.0158	25.27	turquoise	--
BX920222		blue	938.6872	148.758	0.992108	0.00084	22.328	white	--
BX920452		blue	836.2043	121.606	0.94492	0.01539	36.224	blue	--
BX924006		blue	986.2734	140	0.976973	0.00418	10.672	navy	--
BX924401		blue	706.6468	141.572	0.984932	0.00222	7.936		--
BX925530		blue	651.6807	136.773	0.977218	0.00411	2.252	violet	--
BX926950		blue	799.4294	149.502	0.994897	0.00044	30.594	turquoise	--
CN157619		blue	991.086	134.011	0.966442	0.00734	64.982	blue	--
CN158157		blue	783.2543	150.187	0.99683	0.00021	26.676	turquoise	--
CN159503		blue	774.8378	148.03	0.993627	0.00061	11.206	turquoise	--
CN162261		blue	823.7533	149.108	0.994162	0.00053	4.58	turquoise	--
CJ020677		blue	927.5247	148.493	0.991734	0.0009	7.208	lightyellow	99
DN103971		blue	879.2622	148.09	0.992467	0.00078	19.126	turquoise	--
DN104509		blue	740.4868	143.478	0.986406	0.0019	10.042	royalblue	--
DN106267		blue	745.4209	143.736	0.986106	0.00196	3.688	white	--
DN106307		blue	882.0214	140.741	0.980335	0.0033	35.044	black	--
DN113940		blue	749.9724	148.745	0.994845	0.00044	5.734	black	--

DN115314	blue	979.8983	139.845	0.976669	0.00426	30.196	lightyellow	--	
DN115744	blue	565.2152	114.496	0.93404	0.02013	13.576	royalblue	--	
DN115916	blue	859.9734	144.837	0.986306	0.00192	12.094	orange	--	
DN116041	blue	786.9382	126.377	0.954361	0.01162	21.328	blue	--	
DN116668	blue	430.1434	103.602	0.913194	0.0303	9.676	darkmagenta	--	
DN118663	blue	991.1468	136.271	0.970547	0.00604	3.284	grey60	--	
DN121492	blue	845.1703	131.47	0.964413	0.00802	7.656	violet	--	
DN122709	blue	384.5387	74.7872	0.841202	0.07413	16.25	turquoise	--	
DN123551	blue	593.7688	94.2905	0.889032	0.04363	122.164	navy		99
DN124718	blue	925.3166	149.368	0.993523	0.00063	2.884	lightyellow		0
DN125619	blue	567.5285	118.817	0.944528	0.01555	13.188	turquoise	--	
DN132866	blue	549.7049	105.553	0.916908	0.02839	4063.742	royalblue	--	
BW974788	blue	587.2749	85.9038	0.870695	0.05472	30.95	orange		0
AJ947557	blue	493.836	106.855	0.922764	0.02547	6.634	navy	--	
AJ956504	blue	957.9383	138.206	0.974742	0.0048	22.134	turquoise	--	
DV227358	blue	849.8925	135.956	0.972849	0.00535	2.154	lightyellow	--	
DV900662	blue	803.2575	150.527	0.996662	0.00023	248.594	turquoise	--	
CV875565	blue	774.2519	150.289	0.996736	0.00022	1.054	lightgreen	--	
CX064206	blue	710.0368	124.756	0.954724	0.01149	77.784	brown	--	
EB684343	blue	635.3383	103.688	0.912035	0.0309	116.238	navy	--	
DB789786	blue	965.4397	144.499	0.984873	0.00223	3.258	yellow	--	
DB795256	blue	941.396	148.466	0.991591	0.00092	5.584	yellow	--	
DT323640	blue	838.2138	142.015	0.982702	0.00272	5.902	grey60	--	
DT324398	blue	977.8395	140.145	0.977462	0.00405	11.66	darkturquoise	--	
EW041986	blue	973.3932	137.076	0.972521	0.00545	78.76	orange	--	
EW113181	blue	635.037	132.631	0.968747	0.0066	3.27		--	
EW138865	blue	639.4295	135.562	0.973629	0.00512	4.182	lightyellow	--	
EW150678	blue	926.663	148.649	0.992051	0.00085	3.336	lightgreen	--	
EW190518	blue	775.8715	135.896	0.973305	0.00521	6.278	lightyellow	--	
EW244600	blue	577.8565	112.72	0.934902	0.01974	25.89	brown	--	
EW258248	blue	941.8889	148.082	0.990939	0.00103	2.482	violet	--	
EW266525	blue	910.9474	144.368	0.985539	0.00208	13.024	black		99
EW299636	blue	681.148	90.2772	0.878093	0.05015	7.53	lightcyan	--	
EW501346	blue	640.4399	137.836	0.978201	0.00385	14.458		--	
EW537674	blue	553.257	86.8004	0.872234	0.05376	8.246	darkgreen	--	
EW569616	blue	662.0212	121.619	0.948775	0.01381	6.838	darkmagenta	--	
EW577030	blue	465.1693	108.593	0.923819	0.02495	29.92	salmon	--	
EW589560	blue	867.9835	131.323	0.964364	0.00803	22.042	white	--	
EW670598	blue	853.5275	143.401	0.984021	0.00242	9.212	darkgrey	--	
AW414891	blue	954.8295	144.24	0.984578	0.00229	11.744	red	--	
BQ601250	blue	592.3566	128.184	0.961356	0.00907	5.538	navy	--	

Table S10-3. Module eigengene and its evolutionary rates in 15 postnatal highly LDE-specific modules (LDE_darkgrey).

pig_Genebank	pig_Gene	LDE Module	kTotal	kWithin	eigencorr	eigenpval	meanExpr	LTModule	Ka/Ks
AK230667.1	RIOK2	darkgrey	863.31	50.9695	0.98544	0.002105	19.572	turquoise	0.1625
AK234301.1	RPUSD1	darkgrey	950.32	51.9505	0.98875	0.001429	9.692	tan	0
AK240542.1	CBX1	darkgrey	686.17	39.1613	0.93242	0.020874	25.588	blue	--
AK234463.1		darkgrey	881.37	48.2375	0.97413	0.004975	2.424	yellow	--
AK234534.1	CSRP1	darkgrey	826.44	51.2423	0.9866	0.001859	25.928	black	0
AK238822.1	SLC16A1	darkgrey	918.82	50.8563	0.98487	0.002229	48.594	turquoise	0.4149
AK232646.1	DHRS11	darkgrey	953.26	52.5469	0.99113	0.001002	5.452	violet	0.1299
AK233336.1		darkgrey	899.39	52.368	0.99129	0.000975	5.984	darkred	--
AK236998.1	HMGN2	darkgrey	767.8	48.2544	0.97397	0.005021	2.726	tan	--
AK240275.1	TRAK1	darkgrey	861.76	49.4512	0.97941	0.003536	9.754	yellow	99
AK237326.1	THOC4	darkgrey	895.49	50.0046	0.98166	0.002974	11.514	tan	--
NM_001078687.1	SMPX	darkgrey	666.9	38.8903	0.92996	0.022015	153.78	orange	--
NM_001123135.1	PLIN5	darkgrey	959.24	51.3116	0.98566	0.002056	6.244	black	99
XM_001925477.1	ITGA9	darkgrey	743.65	46.414	0.96596	0.0075	11.006	darkmagenta	99
XM_001927860.1	RBM8A	darkgrey	921.86	53	0.99343	0.000638	69.232	violet	0
XM_001927969.1	PEX11B	darkgrey	790.58	48.4536	0.97491	0.004752	8.112	tan	0
XM_001928010.1	ARFGEF1	darkgrey	1000.7	50.6221	0.98256	0.002758	18.394	tan	0.1897
XM_001927459.1	PRRX1	darkgrey	932.22	54.3136	0.9985	6.96E-05	2.318	turquoise	--
XM_001928732.1	ETFA	darkgrey	860.46	51.0273	0.98564	0.00206	154.422	turquoise	0.1286
XM_001927529.1	ALDH18A1	darkgrey	998.95	51.6342	0.98674	0.001829	6.774	turquoise	0
XM_001928156.1	RBM19	darkgrey	930.24	52.3751	0.99049	0.001111	5.016	lightyellow	0.1252
XM_001927481.1	HIRA	darkgrey	1015.4	49.9108	0.97926	0.003575	8.568	pink	0.3506
XM_001927396.1	TRMT6	darkgrey	753.73	47.5736	0.97097	0.005912	15.104	blue	0
NM_214407.1	GPX4	darkgrey	673.19	42.2101	0.94645	0.014755	85.436	grey	--
AY609612.1	GDE1	darkgrey	877.36	52.8029	0.99276	0.000739	56.918	black	0
AY609966.1	MRPL42	darkgrey	930.68	50.7137	0.98401	0.00242	23.156	turquoise	99
AY610041.1	RTN4IP1	darkgrey	901.88	52.7734	0.99253	0.000775	14.584	turquoise	0.3431
CF177919		darkgrey	851.26	49.4828	0.97942	0.003533	4.53	darkgrey	--
CF180385		darkgrey	1000.1	50.3463	0.98113	0.003104	5.122	violet	--
CF181098		darkgrey	992.54	51.852	0.98765	0.001644	9.042	violet	--
BX667460		darkgrey	825.18	49.9946	0.98132	0.003057	567.994	turquoise	--
BP144404		darkgrey	823.24	50.9983	0.98556	0.002079	6.348	pink	--
BP144484		darkgrey	945.6	53.9416	0.99684	0.000213	11.44	red	0.4075
BP153164		darkgrey	614.74	39.1846	0.93028	0.021867	8.536	yellow	--
CK453453		darkgrey	941.04	52.9107	0.9927	0.000748	3.416	yellow	--
CK454542		darkgrey	932.79	53.7252	0.99607	0.000296	9.812	darkturquoise	--
CK459522		darkgrey	645.87	41.2701	0.9411	0.017008	14.896	purple	--
CK460835		darkgrey	990.91	50.756	0.98335	0.002572	16.706	tan	--
CN165093		darkgrey	1002.2	50.7287	0.98289	0.00268	10.322	yellow	--
DN101901		darkgrey	1011.6	49.4363	0.97712	0.004141	22.296	darkturquoise	--
DN112398		darkgrey	991.98	49.3721	0.97741	0.004063	3.606	blue	--
AJ947734		darkgrey	919.8	48.5647	0.97502	0.004723	143.694	turquoise	--
AJ955965		darkgrey	972.97	52.0502	0.98876	0.001427	6.38	white	--
AJ957573		darkgrey	1019.3	48.7974	0.97423	0.004948	9.17	lightyellow	--
AJ959301		darkgrey	896.3	50.4158	0.98313	0.002625	7.804	turquoise	--
AJ959885		darkgrey	851.82	49.4211	0.97939	0.003542	4.822	white	--
CV871831		darkgrey	1010.4	50.132	0.9801	0.003359	7.292	violet	--

DY411251	darkgrey	807.1	49.2882	0.97843	0.003792	3.276	sienna3	--
DY416124	darkgrey	896.01	52.8548	0.99279	0.000733	3.194	darkturquoise	--
DY424643	darkgrey	924.54	51.6981	0.98826	0.001525	3.406	turquoise	--
DB798053	darkgrey	1001.9	50.6862	0.98257	0.002756	21.4	blue	--
DB803591	darkgrey	860.83	50.1499	0.98188	0.00292	4.182	darkgreen	--
DV937193	darkgrey	528.02	33.1496	0.89629	0.039463	2.696	lightgreen	--
EH412737	darkgrey	847.15	51.0016	0.98559	0.002072	5.746	turquoise	--
EW247306	darkgrey	758.77	46.2795	0.96542	0.007679	5.616	white	--
EW255075	darkgrey	1011.5	50.4113	0.98133	0.003053	7.332	blue	--
EW256986	darkgrey	894.23	50.6456	0.98421	0.002376	4.524	darkred	--
EW300204	darkgrey	997.17	50.7741	0.983	0.002655	6.094	turquoise	--
EW309403	darkgrey	960.29	51.907	0.98838	0.001502	4.58	turquoise	--
EW568747	darkgrey	774.74	44.0338	0.95606	0.010983	13.386	turquoise	--
EW577502	darkgrey	955.59	49.0747	0.97674	0.004243	7.664	turquoise	--
EH005907	darkgrey	841.2	51.5478	0.9878	0.001615	6.848		--

Table S10-4. Module eigengene and its evolutionary rates in 15 postnatal highly LDE-specific modules (LDE_green).

pig_Genebank	pig_Gene	LDE Module	kTotal	kWithin	eigencorr	eigenpval	meanExpr	LTModule	Ka/Ks
NM_001044616.1	NRAP	green	846.4	116.139	0.997944	0.000112	80.066	blue	0.117
AK230758.1	ATP6AP2	green	927.68	96.9054	0.955618	0.011149	22.462	white	0
AK234190.1	TGIF1	green	891.05	114.938	0.995896	0.000315	14.654	tan	99
AK237714.1	RUFY1	green	949.46	110.111	0.983348	0.002573	16.85	salmon	0.1455
AK234734.1	PDCD6IP	green	675.92	98.5002	0.963329	0.008383	60.448	blue	0.2083
AK231294.1	CUL2	green	500.93	62.3297	0.865456	0.058032	27.126	brown	0
AK231701.1	SNORA60	green	964.36	106.415	0.975153	0.004684	5.422	tan	--
AK235390.1	UBE2J2	green	852.75	116.689	0.999198	2.73E-05	5.768	blue	--
AK235521.1	AUP1	green	766.58	113.535	0.994131	0.000539	8.798	blue	99
AK235736.1	NUP37	green	653.22	103.367	0.973578	0.005135	14.25	navy	99
AK238622.1	PA2G4	green	953.07	105.227	0.972032	0.005591	31.668	violet	99
AK238659.1	TMEM123	green	569.23	92.4733	0.94907	0.013692	7.134	yellow	--
AK232425.1	GRWD1	green	847.51	113.763	0.994216	0.000528	5.004	blue	0
AK235881.1	PIRC34	green	974.66	101.403	0.963526	0.008316	20.878	blue	99
AK236069.1	RRN3	green	477.73	62.6502	0.866171	0.057576	10.458	tan	--
AK239116.1	DARS	green	954.96	100.277	0.963179	0.008435	142.774	turquoise	0.1844
AK236287.1	NOL10	green	945.41	112.558	0.990478	0.001114	4.846	blue	0
AK230590.1	SAT1	green	801.04	114.642	0.996446	0.000254	19.204	navy	99
AK236374.1	NUP93	green	872.37	115.524	0.996284	0.000272	2.934	blue	--
AK236554.1	CDC37L1	green	656.99	88.135	0.93858	0.018103	14.94	turquoise	99
AK232780.1	USP2	green	971.33	104.059	0.970984	0.005907	32.604	turquoise	0
AK239617.1	CCNDBP1	green	778.76	105.493	0.976019	0.004442	11.966	navy	--
AK239726.1	UBE2D3	green	827.65	109.358	0.98347	0.002545	22.718	brown	--
AK236572.1	UQCC	green	772.32	113.307	0.993935	0.000566	13.926	turquoise	--
AK236700.1	FKBP9	green	905.44	113.878	0.991835	0.000885	41.898	green	0
AK236976.1	PPP1R3B	green	712.36	107.127	0.981432	0.003029	46.522	navy	0.0869
AK237106.1	SLC25A12	green	772.71	78.8328	0.911431	0.031218	24.91	red	99
AK240240.1	SAPS3	green	699.68	96.5976	0.957782	0.010347	29.136	turquoise	--
AK237308.1	ATP6AP1	green	923.03	108.523	0.979848	0.003424	18.198	orange	--
NM_214068.2	FUT1	green	937.51	113.421	0.992306	0.000809	9.876	turquoise	99
NM_001097506.1	ZFAND5	green	774.97	108.254	0.982629	0.002741	23.364	white	--
NM_001097485.1	HSBP1	green	964.25	109.558	0.98306	0.00264	26.374	turquoise	--
NM_001097460.1	SEPX1	green	406.11	67.6992	0.877875	0.050283	24.042	royalblue	--
NM_001097475.1	NDUFV2	green	815	112.104	0.990459	0.001117	105.43	royalblue	99
NM_214343.2	QDPR	green	970.94	101.947	0.965735	0.007575	19.014	black	0
XM_001927669.1	KIAA0020	green	864.52	108.429	0.981207	0.003084	22.924	navy	99
XM_001925555.1	SGPP1	green	832.6	112.258	0.990258	0.001152	7.5	paleturquoise	--
XM_001925373.1	KIAA1468	green	816.39	87.5044	0.934163	0.020077	19.994	green	0.4208
XM_001928960.1	PEA15	green	904.67	104.11	0.970027	0.006201	36.472	blue	99
XM_001928649.1	RAP1A	green	863.15	106.306	0.975792	0.004505	67.268	turquoise	0
XM_001927243.1	LYPLA1	green	943.36	105.251	0.97201	0.005598	38.256	turquoise	--
XM_001925783.1	DCAF13	green	868.57	116.128	0.99745	0.000155	8.698	turquoise	0.173
XM_001927549.1	COPZ1	green	704.96	97.5625	0.961161	0.009135	15.442	blue	--
XM_001925059.1	RAB21	green	889.3	110.02	0.983485	0.002541	10.48	yellow	99
XM_001925531.1	CDC42BPB	green	871.7	111.702	0.988477	0.001482	14.398	blue	0.0615

XM_001927473.1		green	807.49	109.869	0.986082	0.001967	75.722	turquoise	0
XM_001926122.1	ACYP1	green	775.32	111.059	0.989156	0.001353	10.864	blue	--
XM_001928271.1	FAM158A	green	567.12	87.0596	0.936102	0.019202	47.53	purple	0.3437
XM_001929390.1	HDDC3	green	872.99	111.48	0.987515	0.001672	15.144	turquoise	99
XM_001929425.1	ZXDC	green	644.19	75.2283	0.905854	0.034183	12.44	tan	0.2171
XM_001927097.1	SPRY2	green	731.88	107.134	0.982314	0.002816	24.58	green	99
XM_001927213.1	ANKRD28	green	527.38	82.7704	0.924501	0.024619	15.226		0
XM_001924716.1	CLDND1	green	663.67	104.544	0.976175	0.004399	14.552	blue	99
XM_001926917.1	PLXND1	green	564.16	68.5364	0.886434	0.045151	21.758	brown	0.0304
XM_001926579.1	SMC3	green	782.64	114.014	0.994895	0.000437	26.504	white	0
XM_001929000.1	BAG3	green	678.84	104.704	0.976675	0.004261	56.38	turquoise	0.1523
XM_001927415.1	BMPR1A	green	814.28	113.616	0.994208	0.000529	14.278	darkturquoise	0
XM_001927868.1	ZNF22	green	530.89	87.6781	0.936526	0.019013	4.08	blue	99
XM_001929282.1	CCDC6	green	555.88	65.0829	0.87411	0.052595	6.946	violet	99
XM_001925326.1	TRIAP1	green	777.98	113.006	0.993492	0.00063	4.972	blue	--
XM_001928487.1	COX6A1P2	green	951.6	103.568	0.968056	0.006821	16.196	turquoise	--
XM_001924322.1	CHRNA1	green	446.67	66.9223	0.879112	0.049531	1.484	navy	0
EF113595.2	SEPN1	green	962.34	103.139	0.967417	0.007026	28.898	royalblue	99
AY368623.1	COL5A2	green	969.32	107.11	0.977184	0.004123	24.29	turquoise	--
NM_213944.1	DAD1	green	834.61	115.494	0.996785	0.000219	20.498	blue	0
NM_214024.1	PPP2R1A	green	913.31	112.104	0.987826	0.001609	29.146	tan	0
NM_214188.1	DDOST	green	521.7	71.3719	0.896148	0.039543	10.1	orange	0
NM_214245.1	SCAMP1	green	686.57	94.7526	0.954706	0.011493	12.126	darkred	0
NM_214361.1	MCL1	green	790.91	111.914	0.990558	0.0011	105.712	lightyellow	99
NM_214396.1	XIRP2	green	253.51	40.9239	0.775529	0.123277	701.55	pink	99
NM_001004045.1	S100A11	green	893.78	115.898	0.997742	0.000129	5.894	skyblue	0
AY609728.1	MRPL30	green	795	87.2771	0.931363	0.021362	36.134	royalblue	0
AY610012.1	C1GALT1C1	green	862.06	117.025	0.999887	1.45E-06	9.526	darkgrey	99
AY610221.1	LRRC39	green	790.48	114.126	0.994883	0.000439	12.622		0
AY610353.1	MRPL11	green	955.74	111.512	0.987858	0.001603	7.652	blue	0.6639
NM_001032358.1	PIGK	green	860.89	116.351	0.99809	0.0001	8.862	turquoise	--
NM_001038007.1	SFRS1	green	990.31	98.7942	0.958337	0.010144	63.646	grey60	99
BX665072		green	924.85	109.632	0.982118	0.002863	6.056	turquoise	--
BX665424		green	932.74	109.526	0.981799	0.00294	135.022	grey	0
BX670342		green	972.94	104.278	0.970238	0.006136	25.942	black	--
BX670411		green	763.19	112.377	0.992288	0.000812	10.278	yellow	--
BX672685		green	701.33	91.8697	0.948258	0.014019	11.064	pink	--
BP163106		green	960.61	109.758	0.983154	0.002618	8.034	black	--
CK451396		green	845.46	112.882	0.99278	0.000736	15.354	blue	99
CK456387		green	974.67	107.031	0.977047	0.00416	18.52	turquoise	--
CK463972		green	915.59	114.669	0.993746	0.000593	11.232	darkgreen	--
CK466682		green	821.74	116.202	0.998668	5.83E-05	0.934	lightyellow	--
CK467336		green	976.31	106.543	0.976068	0.004428	15.392	blue	--
BX914732		green	804.73	112.881	0.992009	0.000857	13.14	paleturquoise	--
BX915153		green	815.18	111.225	0.989305	0.001326	4.57	turquoise	99
BX921788		green	863.66	112.649	0.991712	0.000905	20.644	black	--
BX922232		green	942.86	107.373	0.976899	0.0042	34.422	paleturquoise	--
BX922274		green	750.83	110.75	0.988688	0.001442	6.888	salmon	0
BX923017		green	967.37	106.959	0.978219	0.003846	10.212	darkmagenta	99
BX923116		green	814.95	114.232	0.994501	0.000489	6.364	brown	--
BX925602		green	593.65	96.2463	0.957573	0.010423	24.568	navy	--

BX926249		green	796.39	108.713	0.984007	0.002422	7.946	darkred	--	
CN155716		green	486.41	80.0037	0.915554	0.029082	53.214	white	--	
CN157199		green	447.57	74.6244	0.900208	0.037271	13.406	turquoise	--	
CN158088		green	704.65	88.0638	0.935054	0.019674	11.024	greenyellow	--	
AJ604633		green	701.12	77.8161	0.905674	0.03428	3.922	blue	--	
CJ014614		green	706.88	106.205	0.979802	0.003435	9.55	salmon	--	
CJ022676		green	582.7	94.3647	0.953374	0.012001	5.244	paleturquoise	--	
DN102066		green	770.82	109.647	0.98635	0.001911	20.15	blue	--	
DN105634		green	813.71	109.843	0.98496	0.002209	17.318	turquoise	--	
DN107792		green	976.55	106.181	0.975062	0.00471	4.112	lightgreen	--	
DN112212		green	956.62	106.788	0.975697	0.004532	24.404	blue	--	
DN114469		green	406.63	59.3365	0.851215	0.067334	80.644	salmon	--	
DN116629		green	820.06	114.832	0.995641	0.000345	11.214	lightyellow	--	
DN119751		green	901.62	112.613	0.989152	0.001354	6.558	yellow	--	
DN132902		green	903.27	115.793	0.996767	0.000221	5.64	blue	--	
DQ000205		green	940.37	111.268	0.986135	0.001956	16.374	blue	--	
BW975963		green	870.16	115.227	0.996977	0.000199	17.374	purple		0
AJ954681		green	708.05	99.8851	0.966035	0.007476	33.678	tan	--	
AJ956714		green	659.14	102.35	0.971388	0.005785	2.29	blue	--	
AJ943714		green	963.79	105.472	0.972775	0.00537	4.116	yellow	--	
AJ946210		green	462.74	77.169	0.907553	0.03327	4.102	blue		0
DV225307		green	927.76	109.607	0.982039	0.002882	19.498	blue	--	
CV875023		green	555.54	90.9986	0.94468	0.015489	15.186	lightcyan	--	
CV878301		green	420.55	61.3701	0.859458	0.061897	34.642	turquoise	--	
CX064239		green	891.04	111.291	0.986463	0.001887	9.85	blue	--	
DY408154		green	881.58	109.375	0.982244	0.002833	6.004	turquoise	--	
DY417691		green	975.59	101.441	0.963798	0.008224	38.302	black	--	
DB788903		green	837.56	115.764	0.998264	8.68E-05	12.534	lightgreen	--	
DB789650		green	906.66	112.339	0.988409	0.001495	13.234	blue		0
DB787461		green	905.02	110.176	0.983599	0.002515	28.712	navy		99
AK234908	ARL6IP5	green	383.92	49.8428	0.816161	0.091968	48.102	blue		0
DT322463		green	459.49	76.6606	0.906151	0.034023	3.592	green	--	
EV978603		green	545.3	88.5285	0.938467	0.018153	22.844	turquoise	--	
EW052504		green	775.74	112.898	0.992774	0.000737	7.086	darkgrey	--	
EW065653		green	685.78	105.346	0.978186	0.003855	8.334	midnightblue	--	
EW086743		green	657.06	90.8389	0.946119	0.014892	12.444	green	--	
EW236736		green	928.65	105.804	0.973324	0.005209	134.056	navy	--	
EW363322		green	897.49	109.527	0.982199	0.002843	7.432	turquoise	--	
EW475154		green	641.45	102.106	0.970922	0.005926	4.542	greenyellow		0
EW533925		green	916.81	115.018	0.995601	0.00035	2.548	darkred	--	
EW590932		green	468.47	76.4165	0.905018	0.034635	21.488	blue	--	
EW630945		green	722.02	106.145	0.980303	0.003309	8.828	salmon	--	
BE232243		green	557.35	80.8729	0.920825	0.026423	7.234	white	--	

Table S10-5. Module eigengene and its evolutionary rates in 15 postnatal highly LDE-specific modules (LDE_grey60).

pig_Genebank	pig_Gene	LDE Module	kTotal	kWithin	eigencorr	eigenpval	meanExpr	LTModule	Ka/Ks
AK230683.1		grey60	296.4	64.178	0.98292	0.002671688	19.478	brown	--
AK234277.1	PDLIM7	grey60	303.16	67.69	0.99486	0.000442153	1790.29	brown	--
AK240479.1	UBAC1	grey60	296.12	62.877	0.97847	0.003779683	383.234	orange	--
AK231289.1	C16orf62	grey60	314.19	68.764	0.99782	0.00012236	6.634	turquoise	0.4
AK235136.1	DECR2	grey60	314.8	69.237	0.99951	1.30E-05	30.196	brown	--
AK238421.1	ANAPC13	grey60	282.38	60.553	0.9705	0.006055144	27.794	brown	--
AK238713.1	NFATC3	grey60	277.53	57.344	0.95921	0.009827438	62.19	green	--
AK235782.1	RABAC1	grey60	307.77	66.772	0.99161	0.000921642	93.34	red	--
AK232764.1	MAPK8IP1	grey60	284.32	54.258	0.94796	0.014139201	76.344	turquoise	--
AK236600.1	RTF1	grey60	300.72	65.707	0.98793	0.001587978	11.96	turquoise	99
AK239934.1	VAMP8	grey60	302.31	66.971	0.99226	0.000816062	30.098	grey	0
AK240107.1	MAP4	grey60	303.45	65.08	0.98581	0.002025504	125.76	red	--
AK233538.1	CDC34	grey60	298.91	66.183	0.99012	0.001177844	140.376	brown	--
AK233673.1	GPR157	grey60	265.59	54.73	0.94996	0.013337457	28.8		--
NM_001123127.1	HSPA6	grey60	315.05	69.206	0.99924	2.49E-05	5.114	violet	--
NM_001123136.1	LSM2	grey60	299	63.54	0.98058	0.003239737	39.052	blue	--
XM_001928928.1	ATP6V1G1	grey60	313.36	68.773	0.99809	1.00E-04	47.064		99
XM_001926863.1	DCAF6	grey60	311.9	69.087	0.99898	3.92E-05	174.856	brown	0
XM_001928892.1	CRELD1	grey60	308.51	67.552	0.99438	0.0005049	13.948	turquoise	0.331
XM_001929335.1	TAF5	grey60	290.69	58.26	0.96214	0.008794345	46.704	red	--
XM_001929055.1	DUSP13	grey60	255.92	52.198	0.93969	0.017616054	19.124	turquoise	0.302
XM_001928504.1	GATC	grey60	315.42	68.361	0.99652	0.000245946	12.764	red	99
XM_001929590.1	KLHL22	grey60	303.1	65.53	0.98759	0.001656383	16.642	red	--
NM_214204.1	PKIA	grey60	258.17	53.432	0.94354	0.015965395	385.558	red	--
AY609560.1	ZNF639	grey60	300.86	65.885	0.98935	0.001317109	45.722	midnightblue	--
AY609951.1	RNF130	grey60	274.2	60.098	0.96991	0.006236222	156.176	green	--
AY609997.1	CMBL	grey60	233.94	49.47	0.92797	0.022952655	22.476	green	--
AY610214.1	UBE2G1	grey60	285.69	63.415	0.98102	0.003129432	77.432	green	0
AY610223.1	RPL35A	grey60	283.57	56.985	0.95726	0.010538384	6.87	blue	0
AY610341.1	SEC11C	grey60	313.82	68.502	0.99732	0.00016676	9.334	turquoise	0
CF179222		grey60	314.9	68.42	0.99677	0.00022016	34.856	turquoise	--
BX670617		grey60	255.26	53.494	0.94461	0.015518177	54.216	blue	--
BX671397		grey60	307.56	68.164	0.99635	0.000264539	124.356	midnightblue	--
BX675592		grey60	272.2	59.705	0.96766	0.006947704	30.83	red	--
CK456926		grey60	307.27	65.28	0.98672	0.001834184	6.886	turquoise	--
BX914800		grey60	269.17	56.57	0.95572	0.011112072	78.546	turquoise	--
BX915506		grey60	253.42	55.423	0.95307	0.01211904	23.912	green	1.622
BX915599		grey60	312.28	68.868	0.99829	8.47E-05	14.914	turquoise	--
BX917456		grey60	308.67	68.257	0.99651	0.000247071	18.76	brown	99
BX926121		grey60	282.56	62.798	0.97922	0.003585294	37.094	orange	--
CN155050		grey60	289.39	60.484	0.97006	0.006190282	8.67	red	--
CN155836		grey60	286.28	56.976	0.95745	0.01046879	32.392	black	--
CN157668		grey60	250.79	50.881	0.93374	0.020270654	23.174	lightcyan	0
CN159661		grey60	306	67.239	0.99309	0.000689214	13.094	black	--
CN166351		grey60	237.42	48.871	0.92713	0.02335283	2.46	darkgreen	0.372

AJ656748	grey60	309.64	67.758	0.99462	0.000473013	113.82	red	--	
CJ012192	grey60	308.44	67.617	0.99433	0.000512515	10.022	grey	--	
CJ028165	grey60	298.26	62.356	0.97667	0.004262353	5.238	turquoise	--	
DN102471	grey60	296.97	61.304	0.97304	0.005292461	26.014	yellow	--	
DN114154	grey60	309.71	67.443	0.99393	0.000567694	8.554	darkturquoise	--	
DN114201	grey60	308.87	67.471	0.99368	0.000603127	3.444	pink	--	
DN115175	grey60	304.63	65.692	0.98791	0.001593526	16.582	navy	--	
DN119561	grey60	300.34	66.321	0.99065	0.00108457	9.758	green	--	
DN122318	grey60	267.83	57.704	0.96092	0.009217922	55.208	salmon	--	
DN125939	grey60	309.13	64.67	0.98482	0.002239071	60.632	red		0
DN127056	grey60	294.2	65.753	0.98884	0.001412095	54.24	green	--	
AJ958002	grey60	312.48	67.594	0.99408	0.000545913	16.74	blue	--	
DV229656	grey60	315.14	68.031	0.99575	0.000332416	23.316	red		99
DY435285	grey60	295.19	60.962	0.97188	0.005635694	40.804	turquoise	--	
DB808839	grey60	312.84	67.38	0.99338	0.000645913	12.136	blue	--	
DB786703	grey60	310.99	68.437	0.99696	0.000200747	20.81	royalblue		99
DB795640	grey60	307.42	66.889	0.99195	0.000865474	84.538	purple		0
EW064937	grey60	300.61	67.193	0.99323	0.000667928	96.436	yellow		99
EW176268	grey60	292.68	62.615	0.97771	0.003980118	12.666	brown	--	
EW202676	grey60	309.14	66.756	0.99132	0.000969279	67.304	greenyellow	--	
EW226073	grey60	315.11	68.82	0.99821	9.11E-05	6.112	purple	--	
EW252113	grey60	314.7	68.725	0.99784	0.000120708	13.052	red	--	
EW297931	grey60	314.72	69.104	0.99892	4.25E-05	38.772		--	
EW372898	grey60	263.34	57.086	0.95798	0.010274964	18.414	green	--	
EW373211	grey60	314.64	67.319	0.99332	0.000655229	9.144	red	--	
EW414777	grey60	312.98	68.47	0.99699	0.000198382	4.3	turquoise	--	
EW470718	grey60	220.77	46.454	0.91486	0.029436063	17.628	darkred		99
EW476075	grey60	292.24	58.278	0.96229	0.00873906	8.486	yellow	--	
EW529809	grey60	286.42	62.683	0.97841	0.003796602	11.334	skyblue	--	
EW572612	grey60	314.52	69.151	0.99908	3.36E-05	5.65	blue	--	
EW589140	grey60	228.85	47.384	0.92051	0.026579774	11.632	salmon	--	
BI184843	grey60	285.99	61.386	0.9743	0.004925559	13.388	darkorange	--	
BM083163	grey60	299.74	65.443	0.98793	0.001588771	85.844	navy	--	

Table S10-6. Module eigengene and its evolutionary rates in 15 postnatal highly LDE-specific modules (LDE_lightyellow).

pig_Genebank	pig_Gene	LDE Module	kTotal	kWithin	eigencorr	eigenpval	meanExpr	LTModule	Ka/Ks
NM_001044553.1	SELK	lightyellow	478.675	64.07508	0.998878	4.51E-05	30.714	purple	99
NM_001044558.1	TMEM59	lightyellow	662.916	54.61539	0.960446	0.009387	8.938	white	0
AK240439.1	TBCB	lightyellow	368.614	60.38818	0.985043	0.002191	19.886	salmon	99
AK234414.1	IPO11	lightyellow	531.998	61.21603	0.987573	0.00166	48.17	green	--
AK234948.1	STRAP	lightyellow	574.403	59.96359	0.982464	0.00278	143.722	darkred	0
AK232023.1	PDLIM5	lightyellow	425.554	63.27538	0.995689	0.00034	135.396	grey	--
AK232310.1	ALDH1A1	lightyellow	394.232	57.31386	0.973883	0.005047	1.22	yellow	0
AK232397.1	TMBIM6	lightyellow	625.03	58.16862	0.975421	0.004609	11.17	green	--
AK238821.1	NAP1L1	lightyellow	414.977	63.16115	0.995306	0.000386	6.312	salmon	99
AK240077.1	SUMO2	lightyellow	344.089	51.81924	0.954667	0.011508	86.374	black	99
AK240146.1	CXCL12	lightyellow	430.565	59.34853	0.981847	0.002928	9.592	grey	--
AK233765.1	FAM3A	lightyellow	489.408	63.98949	0.998524	6.81E-05	46.042	turquoise	--
NM_001078669.1	POMGNT1	lightyellow	572.708	53.18451	0.959389	0.009764	15.738	blue	0.044
NM_001128486.1	FRRS1	lightyellow	279.141	50.35325	0.943487	0.01599	6.392	darkgreen	0.17
X07617.1	PRKACA	lightyellow	530.772	53.98242	0.963017	0.00849	9.726	turquoise	--
XM_001929195.1	PPP6C	lightyellow	589.931	58.63094	0.977124	0.004139	114.69	navy	0
XM_001925794.1	VLDLR	lightyellow	312.449	55.92249	0.967469	0.007009	5.208	yellowgreen	--
XM_001928396.1	PNRC1	lightyellow	327.536	55.04495	0.964387	0.008024	5.426	grey	99
XM_001928966.1	CASQ1	lightyellow	213.189	41.18864	0.89916	0.037853	351.124	green	0
XM_001925288.1	SETD3	lightyellow	495.65	63.23007	0.99514	0.000406	23.108	turquoise	0
XM_001926312.1	PTPN21	lightyellow	575.509	60.23538	0.983326	0.002578	1.482	darkgreen	0
XM_001928665.1	NPTN	lightyellow	419.319	58.96962	0.981041	0.003125	76.942	violet	99
XM_001929228.1	IMP3	lightyellow	347.812	58.02646	0.976299	0.004364	44.868	skyblue	0
XM_001924631.1	N4BP2L2	lightyellow	666.831	47.43861	0.933099	0.020563	8.596	blue	0.241
XM_001928236.1	LSG1	lightyellow	302.607	52.24061	0.953768	0.01185	5.104	turquoise	0.727
XM_001926521.1	PPP1R3C	lightyellow	321.662	49.33518	0.944404	0.015604	39.796	salmon	0
NM_213891.1	VCAM1	lightyellow	458.318	63.71928	0.997451	0.000154	14.29	blue	0.093
NM_213945.1	PLAU	lightyellow	324.741	56.90799	0.972208	0.005538	6.286	skyblue	1.804
NM_213998.1	ITGA2B	lightyellow	457.236	55.43518	0.968832	0.006574	7.882	royalblue	0.082
NM_213785.1	F3	lightyellow	574.957	52.0774	0.95499	0.011385	1.184	tan	0
NM_001001537.1	ITIH4	lightyellow	517.607	55.46635	0.968755	0.006599	4.024	darkmagenta	0.17
NM_001006592.1	MYLPF	lightyellow	391.273	59.49712	0.98331	0.002582	8968.848	yellow	0
AY870324.1	GYS1	lightyellow	318.209	56.48219	0.969905	0.006239	49.048		--
AF116346.1	HRAS	lightyellow	638.835	53.85355	0.956982	0.010641	7.51	turquoise	--
CF360662		lightyellow	340.043	48.53029	0.940257	0.017371	5.044	pink	--
BX668887		lightyellow	514.011	54.46428	0.964955	0.007834	11.37	salmon	--
BX672674		lightyellow	287.484	51.32437	0.947943	0.014146	2.9	midnightblue	--
BX672750		lightyellow	393.497	62.5583	0.993367	0.000648	12.206	paleturquoise	0
BX674122		lightyellow	551.755	62.49046	0.992267	0.000815	14.526	turquoise	99
BP160020		lightyellow	525.817	60.20509	0.983812	0.002466	159.506	greenyellow	99
BP163665		lightyellow	673.881	51.87572	0.948186	0.014047	12.344	navy	--
CK455244		lightyellow	628.727	56.13107	0.966601	0.00729	9.382	salmon	--
CK458900		lightyellow	465.277	61.94577	0.990701	0.001075	29.936	greenyellow	--
CK459066		lightyellow	568.199	59.1021	0.97893	0.00366	4.966	grey	--
CK459225		lightyellow	375.359	60.01222	0.983719	0.002488	19.9	grey	--

CK464272	lightyellow	517.683	63.17233	0.995514	0.00036	3.552	lightgreen	--
BX919553	lightyellow	337.433	58.047	0.976718	0.00425	87.922	royalblue	--
BX925843	lightyellow	634.06	57.4473	0.972491	0.005454	4.412	green	99
CN158183	lightyellow	506.825	63.22344	0.995125	0.000408	11.318	navy	--
CN161737	lightyellow	344.648	57.80163	0.975956	0.004459	4.888	yellowgreen	--
CN162200	lightyellow	399.903	59.37003	0.981805	0.002938	6.838	black	0.492
CJ008695	lightyellow	637.901	52.03379	0.949698	0.01344	8.884	purple	0
CJ023085	lightyellow	310.371	45.71259	0.926914	0.023457	4.122	paleturquoise	--
CJ029984	lightyellow	396.382	52.40618	0.956807	0.010706	26.444	green	--
BW982564	lightyellow	344.763	54.20692	0.962735	0.008587	2.944		0
AJ951006	lightyellow	449.306	62.90551	0.994467	0.000494	36.128	salmon	--
AJ955519	lightyellow	428.99	59.42556	0.982312	0.002816	169.246	darkred	--
AJ964774	lightyellow	440.429	62.24257	0.991954	0.000865	8.918	turquoise	--
AJ943520	lightyellow	654.206	54.27846	0.95876	0.009991	1.906	yellowgreen	--
AJ943723	lightyellow	294.439	52.61109	0.953586	0.01192	54.518	greenyellow	--
AJ944031	lightyellow	492.988	61.85394	0.990139	0.001174	8.852	pink	--
DV227767	lightyellow	733.559	46.17422	0.921762	0.025959	16.854	turquoise	--
DY405734	lightyellow	562.469	59.00069	0.97859	0.003748	20.372	blue	0
DY426323	lightyellow	414.176	62.94484	0.995009	0.000423	9.028	darkgrey	--
DB798165	lightyellow	624.222	58.28982	0.976052	0.004433	3.32	purple	--
DB802598	lightyellow	703.375	49.9898	0.94027	0.017366	34.484	yellowgreen	--
DT327799	lightyellow	390.629	62.48525	0.992899	0.000717	137.426	yellowgreen	--
DT335508	lightyellow	354.105	55.98536	0.968543	0.006666	6.17	greenyellow	99
EW049542	lightyellow	242.777	45.62588	0.922172	0.025757	3.474	grey	--
EW382931	lightyellow	323.456	57.33085	0.973053	0.005289	7.702	green	--
EW532021	lightyellow	494.373	62.17967	0.991302	0.000972	14.21	grey	--
EW567071	lightyellow	632.471	54.8026	0.961472	0.009026	6.904	darkgrey	--
EW576117	lightyellow	240.986	38.08604	0.887511	0.044518	25.964	white	--
EW621997	lightyellow	518.04	60.41475	0.985373	0.002119	18.13	yellow	--
EW675014	lightyellow	298.501	48.13089	0.936593	0.018983	123.366	brown	--
EH007552	lightyellow	622.151	56.92877	0.969987	0.006213	9.078	navy	99

Table S10-7. Module eigengene and its evolutionary rates in 15 postnatal highly LDE-specific modules (LDE_magenta).

pig_Genebank	pig_Gene	LDE Module	kTotal	kWithin	eigencorr	eigenpval	meanExpr	LTModule	Ka/Ks
NM_001044584.1	CRYZ	magenta	398.538	78.77953	0.9733092	0.00521	33.084	green	0.0893
NM_001044594.1	MYOZ3	magenta	167.364	60.98798	0.9211055	0.02628	173.672	darkorange	--
AK230984.1	SEC62	magenta	365.162	83.27746	0.9880901	0.00156	66.902	skyblue	--
AK234909.1	NUB1	magenta	327.395	79.23191	0.9790986	0.00362	38.876	skyblue	--
AK235335.1	MLX	magenta	309.87	86.33648	0.9989808	3.91E-05	22.164	greenyellow	0
AK235497.1	ZADH2	magenta	264.977	73.68594	0.9650048	0.00782	68.216	purple	--
AK238441.1	MAP2K2	magenta	384.318	76.34244	0.9672064	0.00709	45.356		0
AK238555.1	CCNY	magenta	127.513	38.43605	0.8220382	0.08768	8.488	blue	99
AK232437.1	PACSIN3	magenta	357.739	79.11914	0.9767689	0.00424	25.548	green	0
AK238935.1	9-Mar	magenta	268.716	79.80436	0.9823774	0.0028	5.076	grey	--
AK232581.1	RABEP1	magenta	388.791	78.92312	0.9743697	0.00491	18.536	paleturquoise	--
AK236160.1	CHST10	magenta	419.765	77.22001	0.967384	0.00704	11.576	paleturquoise	0
AK230600.1	DYNLT3	magenta	117.112	34.55505	0.8032351	0.10163	11.092	darkturquoise	--
AK236496.1		magenta	281.924	85.74786	0.9980763	0.0001	8.988	greenyellow	--
AK232843.1	GPD1	magenta	256.403	84.54542	0.9949247	0.00043	15.054	midnightblue	0
AK233149.1	H2AFJ	magenta	423.27	75.52335	0.9617724	0.00892	14.668	skyblue	--
AK236681.1	CFDP1	magenta	310.643	81.86043	0.9868532	0.00181	11.708	greenyellow	--
AK236867.1	KLHL7	magenta	226.351	80.78573	0.9840591	0.00241	6.466	greenyellow	0
NM_001097509.1	ADSSL1	magenta	200.856	76.27581	0.9696809	0.00631	178.64	grey	--
NM_214374.2	MYL1	magenta	333.831	82.88642	0.9888691	0.00141	899.148	darkgreen	--
NM_001123078.1	ATF4	magenta	264.704	84.56027	0.9949961	0.00042	279.664	green	0
EU650276.1	SLC16A3	magenta	444.269	73.37769	0.953907	0.0118	18.88	brown	0
XM_001925693.1	VLDLR	magenta	438.379	75.15681	0.9594965	0.00973	61.882	darkorange	0
XM_001925096.1	LRRC28	magenta	299.983	80.36767	0.9841637	0.00239	4.634	darkgreen	99
XM_001924314.1	EYA4	magenta	216.469	77.22702	0.973585	0.00513	2	greenyellow	--
XM_001927929.1	GBP4	magenta	136.394	55.86493	0.8925295	0.0416	11.758	brown	1.2581
XM_001929090.1	PKM2	magenta	176.859	66.62668	0.9377471	0.01847	283.6	midnightblue	--
XM_001927339.1	FSD2	magenta	374.463	82.20519	0.9844894	0.00231	19.804	green	0.1717
XM_001929236.1	SNUPN	magenta	341.684	72.8877	0.9596619	0.00967	36.068	navy	0.1999
XM_001928810.1	KIAA1279	magenta	443.706	71.00527	0.9460184	0.01493	28.378	yellow	0
XM_001924401.1	GNPAT	magenta	572.404	52.74897	0.8677041	0.0566	23.704	yellow	0.0622
XM_001928874.1	SNTA1	magenta	422.043	77.65691	0.9682882	0.00675	39.924	midnightblue	0
NM_213938.1	OXCT1	magenta	215.531	79.05223	0.978532	0.00376	24.836	greenyellow	99
NM_213961.1	OPTN	magenta	224.535	78.55207	0.9778707	0.00394	5.392	yellow	0
NM_214006.1	CD9	magenta	168.184	62.92086	0.9286517	0.02263	16.626	navy	--
NM_214077.1	PRKAG3	magenta	289.54	79.83651	0.9822043	0.00284	4.57	greenyellow	0.1571
NM_214193.1	CAMK2G	magenta	264.802	72.76261	0.962448	0.00869	19.79	yellowgreen	0
AY609877.1	ABCF2	magenta	384.018	76.2133	0.967253	0.00708	35.67	salmon	0
AY609892.1	TMEM98	magenta	197.817	64.9586	0.9361852	0.01917	10.786	darkorange	0
AY610166.1	PTPLA	magenta	351.961	83.82072	0.9904281	0.00112	142.87	lightcyan	0
AY610168.1	KCMF1	magenta	95.2048	40.17559	0.8106254	0.09607	39.4	navy	99
NM_001008691.1	CXCL10	magenta	85.9469	34.70276	0.7749639	0.12373	18.066	brown	--
NM_001009582.1	STMN1	magenta	152.858	63.90266	0.9246518	0.02455	18.254	magenta	99
NM_001011727.1	AHCY	magenta	482.362	66.71617	0.9285309	0.02269	50.18	salmon	0

CF364671		magenta	339.278	84.39937	0.9925487	0.00077	43.86	greenyellow	--	
BX668548		magenta	400.58	76.77333	0.9672627	0.00708	109.992	green		0
BX668978		magenta	243.643	82.92726	0.9904278	0.00112	126.616		--	
BX669201		magenta	409.85	77.87258	0.9697822	0.00628	399.728	darkgreen		99
BX669236		magenta	434.31	72.14281	0.9503223	0.01319	3.254	lightcyan	--	
BX670633		magenta	316.372	85.30361	0.9958908	0.00032	4.746	darkred	--	
BX673276		magenta	183.444	68.52747	0.9451848	0.01528	15.52	green	--	
BX674344		magenta	128.231	44.43934	0.8456977	0.07105	3.938	darkgreen		99
BP158848		magenta	479.379	68.69662	0.9360449	0.01923	5.214	darkgreen		0
CK449475		magenta	207.355	76.66882	0.9714203	0.00577	2.48	darkred	--	
CK454028		magenta	494.974	64.34867	0.9201694	0.02675	8.456	lightgreen	--	
CK454705		magenta	106.468	42.52302	0.8340223	0.07912	65.608	brown	--	
CK458531		magenta	422.618	74.63314	0.9594272	0.00975	11.808	navy	--	
CK459166		magenta	171.579	66.52572	0.937796	0.01845	22.252	yellowgreen	--	
CK459461		magenta	335.147	77.2301	0.9727493	0.00538	3.558	blue		0
CK463551		magenta	398.12	78.09632	0.9713285	0.0058	13.556	midnightblue	--	
CK466654		magenta	566.215	50.93102	0.8599095	0.0616	21.344	purple	--	
BX915041		magenta	128.106	44.32965	0.8499468	0.06818	101.666	skyblue		99
BX917005		magenta	413.297	76.0685	0.9641788	0.00809	6.548	darkgreen	--	
BX922054		magenta	190.336	73.34472	0.9599694	0.00956	507.954	brown	--	
BX922270		magenta	229.632	79.38155	0.9802642	0.00332	3.682	darkgreen		0.0705
BX922273		magenta	343.35	84.55012	0.9928356	0.00073	10.568	green		99
BX922590		magenta	234.293	82.09696	0.9879344	0.00159	7.384	green	--	
BX923105		magenta	230.524	81.32764	0.9855992	0.00207	2.95	green		99
CN155590		magenta	174.584	62.78963	0.9277259	0.02307	1.884	yellow	--	
CN157249		magenta	339.308	82.02525	0.986116	0.00196	6.46	darkgreen	--	
CN159908		magenta	349.261	75.9609	0.9689334	0.00654	21.192		--	
CJ024410		magenta	404.466	75.44604	0.9633126	0.00839	10.132	green	--	
CJ028990		magenta	282.563	81.95423	0.9880963	0.00156	17.074	grey		99
DN103026		magenta	412.405	79.50428	0.9743809	0.0049	5.262	green	--	
DN103647		magenta	380.175	79.60717	0.9771871	0.00412	6.262	salmon	--	
DN111119		magenta	321.149	75.43135	0.96798	0.00684	5.844	yellowgreen	--	
DN134248		magenta	317.484	86.0562	0.9978989	0.00012	15.24	green		0
BW955252		magenta	273.4	83.91217	0.9934017	0.00064	15.848	yellowgreen	--	
BW984102		magenta	390.429	76.05714	0.9657993	0.00755	7.274	turquoise		99
AJ956613		magenta	174.655	61.03814	0.9205137	0.02658	1.286	navy	--	
AJ944959		magenta	200.295	65.83834	0.9382754	0.01824	45.068	black	--	
CX063371		magenta	242.49	66.2102	0.9418521	0.01668	5.22	salmon	--	
CX064377		magenta	288.423	86.07853	0.9988542	4.65E-05	6.77	salmon	--	
DY410598		magenta	137.939	49.90806	0.8799184	0.04904	20.26	green	--	
DY417941		magenta	168.369	67.37491	0.939643	0.01764	59.888	green	--	
DY424883		magenta	184.044	62.8977	0.9274855	0.02318	317.736	midnightblue	--	
DY428868		magenta	197.862	56.52223	0.9112902	0.03129	6.606	paleturquoise	--	
DQ459258	SLN	magenta	256.158	77.34185	0.9754933	0.00459	128.41	midnightblue	--	
DB788488		magenta	356.961	74.9749	0.9648551	0.00787	2.308	midnightblue	--	
AK235841	OPTN	magenta	262.02	81.33387	0.9865889	0.00186	3.772	greenyellow	--	
AK240390	BIN1	magenta	467.353	67.63773	0.9327673	0.02071	28.814	greenyellow		0
DT328059		magenta	213.743	78.13643	0.9759917	0.00445	2.582	yellow		0

DT329597	magenta	228.311	77.40066	0.9746646	0.00482	21.122	greenyellow	0
DT330909	magenta	227.613	81.19224	0.985094	0.00218	12.136	greenyellow	0
EW055180	magenta	467.699	66.44649	0.9297934	0.02209	15.272		--
EW305206	magenta	427.017	74.17809	0.9574965	0.01045	34.934	greenyellow	--
EW307515	magenta	157.972	63.3543	0.9239307	0.0249	7.214	darkgreen	99
EW309452	magenta	513.973	63.04315	0.9129236	0.03044	5.706	greenyellow	0.4165
EW310662	magenta	247.675	82.98405	0.9908034	0.00106	4.976	pink	--
EW333368	magenta	207.696	76.46818	0.9707127	0.00599	4.288	midnightblue	--
EW333986	magenta	289.362	71.5651	0.9577543	0.01036	9.486	greenyellow	--
EW589027	magenta	181.52	72.10074	0.9552086	0.0113	24.706	pink	--
EW622473	magenta	199.032	76.02412	0.9688956	0.00655	30.312	greenyellow	--
EW630322	magenta	437.705	76.40801	0.9633109	0.00839	8.662	midnightblue	--
EW645578	magenta	100.27	42.00027	0.8198795	0.08924	23.23	pink	--
BI337552	magenta	352.302	66.72673	0.9394587	0.01772	36.04	pink	--
BI360377	magenta	365.493	81.37314	0.982584	0.00275	12.478	grey	--
BI184391	magenta	466.459	70.09684	0.9415526	0.01681	3.834	greenyellow	99
BM190547	magenta	459.991	72.89798	0.9508734	0.01297	52.122	greenyellow	--
BM190416	magenta	515.475	59.51977	0.8995221	0.03765	12.226	salmon	--

Table S10-8. Module eigengene and its evolutionary rates in 15 postnatal highly LDE-specific modules (LDE_midnightblue).

pig_Genebank	pig_Gene	LDEModule	kTotal	kWithin	eigencorr	eigenpval	meanExpr	LTModule	Ka/Ks
NM_001044599.1	GADD45A	midnightblue	965.46	77.103	0.99462	0.00047	18.506	violet	--
NM_001048072.1	ARF4	midnightblue	971.1	77.174	0.99477	0.00045	35.736	navy	0
AK234260.1	DDX39	midnightblue	730.26	69.528	0.97339	0.00519	8.862	blue	0
AK231014.1	C11orf54	midnightblue	892.09	75.348	0.99015	0.00117	3.58	blue	0.176
AK234713.1	GTF2I	midnightblue	956.81	73.869	0.98543	0.00211	25.004	turquoise	0
AK231556.1	HSF2	midnightblue	804.74	74.364	0.98744	0.00169	34.466	black	99
AK235325.1	SFRS2	midnightblue	893.49	76.405	0.9931	0.00069	144.162	lightyellow	0
AK238246.1	WDR26	midnightblue	843.9	76.159	0.99215	0.00083	14.806	blue	99
AK238334.1	SLC35D2	midnightblue	916.32	74.621	0.98811	0.00155	5.798	violet	99
AK231915.1	STIP1	midnightblue	922.94	76.303	0.9926	0.00076	42.672	blue	0.2
AK235234.1	RAB10	midnightblue	624.14	63.701	0.95477	0.01147	112.428	turquoise	99
AK235982.1	CWC27	midnightblue	811.94	72.559	0.98254	0.00276	9.6	lightyellow	0.504
AK236052.1	PTX3	midnightblue	892.34	77.285	0.99543	0.00037	7.484	turquoise	0.361
AK236154.1	TADA2A	midnightblue	897.17	77.562	0.99609	0.00029	12.04	white	0
AK236189.1	DNAJB9	midnightblue	979.7	75.351	0.9896	0.00127	10.506	lightyellow	--
AK239186.1	MCM6	midnightblue	867.08	72.304	0.98112	0.00311	10.344	navy	--
AK230591.1	AZIN1	midnightblue	626.85	65.194	0.95967	0.00966	45.446	blue	99
AK230605.1	CCNC	midnightblue	901.19	70.85	0.97681	0.00422	13.254	grey60	0
AK239563.1	CCT5	midnightblue	950.73	74.536	0.98766	0.00164	70.788	grey60	--
AK236584.1	MRPS27	midnightblue	880.08	64.868	0.95817	0.0102	19.168	turquoise	0
AK236655.1	ATP6V1B2	midnightblue	938.94	78.656	0.99892	4.26E-05	27.908	turquoise	--
AK236674.1	WBSCR22	midnightblue	923.88	77.422	0.99556	0.00035	25.398	blue	0
AK239906.1	HSPB1	midnightblue	694.69	64.92	0.95917	0.00984	117.038	blue	--
AK236899.1	SH3KBP1	midnightblue	805.44	74.779	0.98839	0.0015	8.348	turquoise	0.381
AK240079.1	PDGFRB	midnightblue	909.62	69.721	0.97342	0.00518	7.382	black	--
AK233373.1	TSPAN12	midnightblue	903.3	77.628	0.99628	0.00027	9.318	blue	0
NM_001113442.1	UNC45B	midnightblue	1000	70.778	0.97624	0.00438	20.97	turquoise	0.015
X15073.1	ATP2A2	midnightblue	903.46	76.966	0.99455	0.00048	13.336		99
XM_001925674.1	ATP5A1	midnightblue	997.68	71.917	0.97972	0.00346	1093.45	turquoise	0
XM_001927095.1	NT5E	midnightblue	947.62	67.75	0.96715	0.00711	6.846	turquoise	0.104
XM_001928505.1	WDYHV1	midnightblue	704.25	70.621	0.97648	0.00431	10.196	black	0.077
XM_001926398.1	SMG5	midnightblue	944.48	71.164	0.97772	0.00398	5.498	yellow	0
XM_001928851.1	DAP3	midnightblue	764.87	59.031	0.9397	0.01761	18.688	blue	99
XM_001926489.1	SF3B4	midnightblue	989.17	69.037	0.97107	0.00588	7.184	tan	99
XM_001928686.1	SEC22B	midnightblue	812.76	74.576	0.98812	0.00155	10.568	salmon	0
XM_001927665.1	UBE2W	midnightblue	901.25	76.946	0.99465	0.00047	11.282	blue	99
XM_001927466.1	DZIP1	midnightblue	948.83	78.085	0.99734	0.00016	5.952	darkorange	0.178
XM_001928597.1	DNAJC19	midnightblue	582.99	56.126	0.92929	0.02233	45.276	blue	0
XM_001924202.1	SEC31B	midnightblue	823.88	75.835	0.99142	0.00095	6.23		--
XM_001927832.1	FAM190B	midnightblue	955.63	77.371	0.99534	0.00038	14.928	turquoise	99
XM_001927955.1	HNRNPH3	midnightblue	839.04	76.096	0.992	0.00086	64.744	blue	0.393
XM_001924771.1	RPP30	midnightblue	936.32	78.463	0.99837	7.88E-05	18.616	turquoise	0
XM_001929407.1	ANAPC7	midnightblue	969.78	73.816	0.98522	0.00215	7.302	skyblue	0
XM_001925356.1	RNF10	midnightblue	900.66	69.811	0.9737	0.0051	26.668	grey60	0.43

XM_001926134.1	CLCN3	midnightblue	775.52	71.595	0.97914	0.00361	4.45	blue	0
XM_001924460.1	CNOT7	midnightblue	802.86	63.303	0.95354	0.01194	37.84	grey60	0
NM_214183.1	CACNA2D1	midnightblue	658.23	67.496	0.96706	0.00714	9.8	yellow	0
NM_214213.1	PLN	midnightblue	862.4	71.342	0.97829	0.00383	23.266	brown	--
NM_214240.1	ATP6V1H	midnightblue	712.6	66.899	0.96552	0.00765	25.354	blue	0.297
AY609472.1	PNO1	midnightblue	900.22	67.491	0.96646	0.00733	13.428	white	99
AY609474.1	RPF2	midnightblue	802.73	69.433	0.97348	0.00516	8.818	blue	99
AY609500.1	CEBPG	midnightblue	865.18	77.762	0.99673	0.00022	18.722	blue	--
AY610132.1	TXNDC9	midnightblue	897.81	78.45	0.99846	7.26E-05	26.78	turquoise	99
AY610180.1	MRPL13	midnightblue	911.68	77.767	0.99665	0.00023	24.578	white	0
CF362171		midnightblue	851.18	74.311	0.98718	0.00174	5.306		--
BX673587		midnightblue	901.72	73.42	0.9844	0.00233	9.424	white	--
CK455500		midnightblue	988.47	75.531	0.99017	0.00117	9.066	turquoise	--
CK464622		midnightblue	817.13	67.54	0.96706	0.00714	8.328	blue	--
BX925865		midnightblue	742.89	71.933	0.98051	0.00326	3.39		--
BX926942		midnightblue	979.84	73.955	0.98567	0.00206	15.092	black	--
CN155564		midnightblue	932	77.229	0.99502	0.00042	20.276	skyblue	--
CN156505		midnightblue	925.08	78.547	0.99863	6.06E-05	7.874	violet	--
CN157030		midnightblue	761.26	71.968	0.98057	0.00324	15.258	turquoise	0.443
CN157667		midnightblue	687.19	69.076	0.97166	0.0057	8.938	yellow	--
CN158958		midnightblue	731.56	72.132	0.98099	0.00314	9.528	turquoise	--
CN162073		midnightblue	923.58	73.892	0.98577	0.00203	81.978	blue	--
CN164027		midnightblue	788.09	70.592	0.97652	0.0043	26.28	grey60	--
CJ030093		midnightblue	920.25	78.346	0.99809	0.0001	7.9	turquoise	--
DN102178		midnightblue	929.08	77.83	0.99681	0.00022	40.656	turquoise	--
DN107722		midnightblue	898.5	78.587	0.99881	4.91E-05	10.564	turquoise	--
DN125831		midnightblue	841.49	77.673	0.99644	0.00025	13.906	lightgreen	--
BW956903		midnightblue	714.6	70.35	0.97574	0.00452	1.266	black	--
AJ960573		midnightblue	840.82	72.238	0.98091	0.00316	11.038	blue	--
CV875164		midnightblue	906.91	78.518	0.99858	6.45E-05	8.258	blue	--
CX058800		midnightblue	930.32	78.16	0.99756	0.00015	84.47	darkred	--
CX064653		midnightblue	854.34	62.704	0.95121	0.01284	10.446	red	--
DY418016		midnightblue	881.44	77.525	0.99593	0.00031	10.198	turquoise	--
DB805153		midnightblue	989.49	75.197	0.9892	0.00135	10.908	purple	0.2
DT320474		midnightblue	800.44	75.877	0.9915	0.00094	52.236	midnightblue	--
DT331358		midnightblue	934.18	77.271	0.99532	0.00038	1.66	darkturquoise	99
EV898729		midnightblue	724.74	71.67	0.97976	0.00345	6.724	red	--
EV932107		midnightblue	844.8	76.677	0.99383	0.00058	43.566	turquoise	--
EV983935		midnightblue	965.48	76.649	0.99328	0.00066	10.702	turquoise	99
EW053792		midnightblue	742.76	71.264	0.97867	0.00373	5.922	darkgrey	--
EW177852		midnightblue	720.51	66.148	0.96311	0.00846	6.134	black	--
EW568863		midnightblue	989.43	71.492	0.97836	0.00381	7.018	darkturquoise	--
		midnightblue							

Table S10-9. Module eigengene and its evolutionary rates in 15 postnatal highly LDE-specific modules (LDE_pink).

pig_Genebank	pig_Gene	LDE Module	kTotal	kWithin	eigencorr	eigenpval	meanExpr	LTModule	Ka/Ks
DQ508264.1	PYGM	pink	860.8	147.68	0.99708	0.00019	1.592	yellow	0.033
NM_001044541.1	MTX2	pink	899.2	146.4	0.99521	0.0004	5.546	blue	0
AK234182.1	SERHL2	pink	894.6	149.63	0.99969	6.63E-06	1.476	lightgreen	--
AK234671.1		pink	900.2	147.5	0.99679	0.00022	2.112	magenta	--
AK234693.1	MOSC2	pink	913.4	149.63	0.99967	7.34E-06	5.216	darkturquoise	99
AK234702.1	ZNF154	pink	922.4	148.66	0.99826	8.69E-05	8.516	white	--
AK237778.1		pink	1006	128.14	0.96704	0.00715	74.428	turquoise	--
AK234813.1		pink	935.5	148.3	0.99772	0.00013	52.302	yellow	--
AK231815.1		pink	894.6	149.63	0.99969	6.63E-06	1.668	lightgreen	--
AK238195.1		pink	927.3	148.27	0.9977	0.00013	3.414	yellow	99
AK235496.1	RUVBL2	pink	895.1	145.08	0.99343	0.00064	2.396	darkgreen	0
AK232205.1	HSPB7	pink	978.2	135.54	0.97897	0.00365	3.664	blue	--
AK238597.1	C16orf72	pink	894.6	149.63	0.99969	6.63E-06	0.45	blue	--
AK232480.1		pink	895	145.04	0.99338	0.00065	1.598	turquoise	--
AK238947.1	FSTL4	pink	895.1	145.08	0.99343	0.00064	2.396	sienna3	--
AK236316.1	TRPC3	pink	973.2	142.71	0.98954	0.00128	8.786	turquoise	--
AK239290.1	SERP1	pink	833.2	145.02	0.99339	0.00064	4.222	darkturquoise	--
AK236487.1		pink	731.3	134.62	0.97825	0.00384	27.486	turquoise	--
AK236532.1	RPS6KA3	pink	918.9	149.59	0.99958	1.03E-05	3.456	darkgreen	0
AK232861.1	GPT	pink	844.9	147.28	0.99654	0.00024	3.412	paleturquoise	--
AK233062.1		pink	941.8	148.27	0.99767	0.00013	17.092	yellow	--
AK233154.1		pink	970	145	0.9929	0.00072	5.034	yellow	--
AK236758.1		pink	976.8	142.41	0.9891	0.00136	4.278	navy	99
AK236782.1	ELP4	pink	894.6	149.63	0.99969	6.63E-06	1.412		0.199
AK233820.1	SETD2	pink	760.5	137.28	0.98216	0.00285	3.87	white	0
AK234126.1	TIMM8A	pink	919.3	149.7	0.99973	5.44E-06	14.578	darkgreen	99
AK234149.1	CD320	pink	894.6	149.63	0.99969	6.63E-06	0.128	grey60	--
NM_001105294.1	KCNAB1	pink	865.4	141.99	0.98916	0.00135	31.594	violet	99
NM_001099931.1	FABP3	pink	695.8	131.86	0.97381	0.00507	30.618	blue	99
NM_001123162.1	PHGDH	pink	621.7	120.33	0.95522	0.0113	5.156	yellow	0
XM_001924525.1	C18orf22	pink	1004	130.92	0.97146	0.00576	45.272	lightgreen	99
XM_001924580.1	C15orf61	pink	854.3	148.29	0.99792	0.00011	47.78	grey60	--
XM_001924870.1	IBTK	pink	928.5	148.16	0.99753	0.00015	3.286	lightgreen	0.068
XM_001926476.1	STX7	pink	850.7	147.6	0.99695	0.0002	3.17	darkred	0.409
XM_001929668.1	SETDB1	pink	903.3	149.81	0.99992	8.03E-07	21.228	lightgreen	0
XM_001927918.1	CASQ2	pink	766.8	136.98	0.98177	0.00295	6.14	black	0.065
XM_001925028.1	PRKD1	pink	894.6	149.63	0.99969	6.63E-06	1.732	green	0
XM_001928468.1	PCK2	pink	694.8	122.05	0.95872	0.01	3.196	darkturquoise	0.311
XM_001925622.1	HAPLN3	pink	920.9	148.82	0.99849	7.03E-05	2.898	lightgreen	0
XM_001928454.1	GNL3	pink	894.6	149.63	0.99969	6.63E-06	1.54	blue	0.565
XM_001925662.1	PITX3	pink	970.5	143.65	0.99092	0.00104	7.08	blue	0
XM_001927272.1	ACTN2	pink	747.9	138.03	0.98319	0.00261	108.904	blue	0
XM_001929445.1	MMP11	pink	894.6	149.63	0.99969	6.63E-06	1.348	darkturquoise	99
XM_001926068.1	ESF1	pink	894.6	149.63	0.99969	6.63E-06	1.604	darkturquoise	99
XM_001927947.1	SRXN1	pink	838.6	145.44	0.99397	0.00056	10.006	darkturquoise	--
XM_001928889.1	ACOT8	pink	957	146.62	0.99525	0.00039	17.638	black	0.067

XM_001929453.1		pink	946.5	145.64	0.9939	0.00057	4.67	darkred	0
NM_213791.1	MYL2	pink	847.9	147.7	0.99711	0.00019	5.272	lightgreen	--
NM_214189.1	INHA	pink	895.2	149.33	0.99928	2.30E-05	9.284	red	0
NM_214339.1	DNAJA4	pink	718.4	131.02	0.97279	0.00537	17.852	blue	99
AY609581.1	C4orf46	pink	629.3	118.45	0.95204	0.01252	4.978	darkred	99
AY609808.1	GALT	pink	993.4	131.41	0.97243	0.00547	15.138	brown	99
AY609968.1	COMMD3	pink	983.2	139.7	0.98507	0.00219	55.714	darkgreen	99
AY610017.1	CCT6A	pink	894.6	149.63	0.99969	6.63E-06	1.604	darkgreen	--
AY610106.1	PLA2G7	pink	894.6	149.63	0.99969	6.63E-06	1.796	lightgreen	0.083
NM_001012613.1	SLC7A1	pink	894.6	149.63	0.99969	6.63E-06	3.53	darkred	99
NM_001033009.1	ITGB1BP2	pink	936.7	149.01	0.9987	5.62E-05	88.628	yellow	--
NM_001037322.1	ST8SIA4	pink	952.9	147.18	0.99608	0.00029	11.766	yellow	0
CF177183		pink	892.5	144.09	0.99204	0.00085	5.94	lightyellow	--
CF178442		pink	872.1	148.91	0.99876	5.26E-05	77.926		--
CF179357		pink	887.3	140.85	0.98733	0.00171	7.674	lightgreen	--
CF180095		pink	1016	127.99	0.96673	0.00725	19.756	white	--
CF181085		pink	842.4	140.95	0.98771	0.00163	15.742	violet	--
CF360725		pink	710.7	130.9	0.97264	0.00541	10.314	midnightblue	--
CF362081		pink	863.6	148.75	0.99855	6.65E-05	11.062	white	--
CF366098		pink	931.2	148.22	0.99763	0.00014	5.694	yellow	--
CF368599		pink	851.3	146.17	0.995	0.00042	3.474	turquoise	--
CF792402		pink	894.6	149.63	0.99969	6.63E-06	1.412	yellow	--
CF793177		pink	952.9	145.89	0.99424	0.00052	3.4	yellow	--
CF793308		pink	974.7	144.85	0.99264	0.00076	15.73	turquoise	--
CF795779		pink	908.1	149.57	0.9996	9.68E-06	9.938	yellow	--
BX666875		pink	981	142.57	0.9893	0.00133	4.81	yellow	--
BX667822		pink	984.4	141.92	0.98837	0.0015	23.446	blue	--
BX670717		pink	949.2	147.96	0.99719	0.00018	3.622	darkred	--
BX674049		pink	926.8	148.33	0.99778	0.00013	3.478	grey60	--
BX675434		pink	567.9	103.34	0.92532	0.02422	25.746	darkmagenta	--
BP169289		pink	892.5	144.93	0.99319	0.00067	44.208	violet	--
BP172747		pink	895	145.04	0.99338	0.00065	1.598	darkturquoise	--
BP440567		pink	815.6	135.95	0.98032	0.0033	10.864	red	99
CK455612		pink	742.3	135.67	0.97971	0.00346	16.96	turquoise	--
CK457857		pink	911.3	149.78	0.99987	1.77E-06	4.632	turquoise	--
CK459217		pink	894.6	149.63	0.99969	6.63E-06	1.54	lightgreen	--
CK460594		pink	836.6	146.02	0.99481	0.00045	17.188	grey60	--
CK460617		pink	864.4	144.68	0.99296	0.00071	1.65	blue	--
CK461051		pink	607.9	116.07	0.94785	0.01418	110.26	tan	--
CK461743		pink	976.4	137.43	0.98183	0.00293	1.286	tan	--
CK462736		pink	894.6	149.63	0.99969	6.63E-06	1.668	darkturquoise	--
CK466076		pink	894.6	149.63	0.99969	6.63E-06	1.476	turquoise	--
CK467678		pink	858.8	147.49	0.99681	0.00022	1.528	darkorange	99
BX917298		pink	895	145.04	0.99338	0.00065	1.598	darkgreen	0.074
BX926632		pink	931.5	148.88	0.99854	6.72E-05	17.44	yellow	--
CN167094		pink	880.7	149.24	0.9992	2.74E-05	3.196	lightgreen	--
CJ009195		pink	832.4	146.16	0.995	0.00042	13.632	tan	--
CJ013911		pink	915.5	146.31	0.995	0.00042	4.044	yellow	--
CJ021696		pink	901.6	149.46	0.99943	1.61E-05	5.644	yellow	--
DN101037		pink	934.9	143.15	0.9904	0.00113	3.692	white	--
DN102999		pink	891.7	149.37	0.99934	2.05E-05	5.476	blue	--

DN106999	pink	601.6	116.48	0.94876	0.01382	9.964	white	99
DN111044	pink	940.9	148.79	0.99837	7.89E-05	13.004	yellow	--
DN112956	pink	665.2	117.05	0.95049	0.01312	56.998	white	--
DN114696	pink	932.7	149.2	0.99899	3.87E-05	5.738	yellow	--
DN121970	pink	911.6	147.52	0.99678	0.00022	4.748	green	0.574
DN129771	pink	812.1	144.97	0.99331	0.00066	7.588	lightgreen	--
AJ946727	pink	847.6	146.29	0.99517	0.0004	2.544	paleturquoise	--
AJ947285	pink	926.9	148.32	0.99776	0.00013	2.32	tan	--
AJ947409	pink	908.4	149.02	0.9988	4.97E-05	15.058	lightgreen	--
AJ948686	pink	762.2	132.37	0.975	0.00473	155.31	sienna3	--
AJ951943	pink	947.1	147.76	0.9969	0.00021	23.78	yellow	--
AJ951950	pink	990.1	141.4	0.98755	0.00166	4.99	skyblue	--
AJ953116	pink	894.6	149.63	0.99969	6.63E-06	1.862	navy	--
AJ956904	pink	813.2	138.03	0.98346	0.00255	5.25	navy	--
AJ957127	pink	850.6	148.1	0.99766	0.00014	3.666	yellow	--
AJ960206	pink	936.5	145.35	0.99357	0.00062	18.072	red	--
AJ961441	pink	583.5	113.03	0.94235	0.01647	45.134	turquoise	--
AJ962837	pink	824.2	143.39	0.99111	0.00101	6.784	midnightblue	--
AJ963372	pink	940.7	148.63	0.99816	9.50E-05	42.192	yellow	--
AJ963878	pink	930.3	149.18	0.99896	4.04E-05	8.584	lightyellow	--
AJ964171	pink	897.9	148.1	0.99763	0.00014	17.29	darkgreen	--
AJ940806	pink	885.3	148.17	0.99769	0.00013	16.784	lightgreen	--
AJ944342	pink	893.1	149.64	0.99971	5.87E-06	22.29	yellow	--
DY406031	pink	842.3	141.85	0.989	0.00138	3.172	navy	--
DY406679	pink	828.6	146.09	0.9949	0.00044	7.874	turquoise	--
DY415294	pink	925	149.27	0.9991	3.22E-05	27.704	red	--
DY437127	pink	967	143.9	0.99135	0.00096	7.316	turquoise	--
DB808485	pink	923.9	149.07	0.99882	4.88E-05	14.528	yellow	--
DB809778	pink	926.4	149.32	0.99917	2.88E-05	35.868	yellow	--
DB812928	pink	990.3	139.84	0.98522	0.00215	2.844	violet	--
DT322875	pink	961.8	133.69	0.97616	0.0044	645.53	darkmagenta	--
EV860684	pink	906.5	149.82	0.99992	8.38E-07	8.908	lightgreen	--
EV867707	pink	936.8	147.99	0.99727	0.00017	4.94	lightgreen	--
EV907972	pink	915.6	148.31	0.9978	0.00012	3.402	yellow	--
EV931277	pink	894.6	149.63	0.99969	6.63E-06	1.476	greenyellow	--
EV936208	pink	925	148.5	0.99802	0.00011	4.96	darkgreen	--
EV987323	pink	894.6	149.63	0.99969	6.63E-06	0.514	violet	--
EW038837	pink	972.6	143.6	0.99083	0.00105	5.378	lightyellow	--
EW097961	pink	894.6	149.63	0.99969	6.63E-06	1.796	blue	--
EW216894	pink	958.5	145.39	0.9935	0.00063	6.636	yellow	--
EW239772	pink	894.6	149.63	0.99969	6.63E-06	0.128	grey60	--
EW261894	pink	897	145.9	0.99458	0.00048	1.726	yellow	--
EW280550	pink	885.5	148.79	0.99856	6.58E-05	4.336	purple	--
EW286124	pink	974.4	142.46	0.98918	0.00135	4.718	turquoise	--
EW303282	pink	925.5	143.89	0.99158	0.00093	3.346	yellow	--
EW305327	pink	894.6	149.63	0.99969	6.63E-06	2.182	darkred	--
EW333024	pink	927.4	148.09	0.99747	0.00015	27.404	red	--
EW418907	pink	688.9	121.39	0.95774	0.01036	6.476	blue	--
EW464371	pink	797	143.27	0.9909	0.00104	40.888	turquoise	--
EW595278	pink	618.4	118.28	0.95189	0.01258	14.35	black	99
EX655853	pink	847.6	146.29	0.99517	0.0004	2.544	darkred	--

BF198544	pink	938.8	146.69	0.99543	0.00037	1.614	yellow	--
BQ598384	pink	942	144.79	0.99282	0.00073	7.256	yellow	--
BF710584	pink	804.5	141.39	0.98822	0.00153	2.892	purple	--
BQ597815	pink	897.8	146.26	0.99507	0.00042	1.79	purple	--
BI183745	pink	668.6	125.49	0.96377	0.00823	2.966	red	--
BI184334	pink	897.1	148.68	0.99837	7.90E-05	3.52	magenta	--
BI401798	pink	1003	132.19	0.97357	0.00514	4.676	navy	--
BI401840	pink	894.6	149.63	0.99969	6.63E-06	1.09	lightyellow	--
BI402468	pink	982.1	132.55	0.97427	0.00493	10.164	red	--
BM659726	pink	925.3	148.58	0.99815	9.55E-05	3.646	lightgreen	--

Table S10-10. Module eigengene and its evolutionary rates in 15 postnatal highly LDE-specific modules (LDE_purple).

pig_Genebank	pig_Gene	LDE Module	kTotal	kWithin	eigencorr	eigenpval	meanExpr	LTModule	Ka/Ks
AK237496.1	C16orf61	purple	212.45	46.2877	0.87598	0.051445	10.888		--
AK237517.1	MRPS6	purple	233.95	44.741	0.86967	0.055362	24.31	lightcyan	--
AK231197.1	RPL36	purple	231.63	49.807	0.89025	0.042918	426.966	red	99
AK237867.1	SMYD2	purple	182.66	42.5058	0.86284	0.059707	52.864	green	0
AK231453.1		purple	293.43	77.8571	0.99249	0.00078	7.62	turquoise	--
AK235913.1	FHOD1	purple	279.84	76.295	0.98816	0.001545	135.132	orange	1.2135
AK232557.1	CAST	purple	303.72	75.569	0.98457	0.002296	20.778	red	--
AK236151.1	LEPROT	purple	257.42	64.6297	0.94986	0.013375	6.67	darkorange	--
AK232929.1	LMAN2L	purple	303.95	75.3182	0.98446	0.002321	44.658	pink	--
AK239728.1	DNAJB6	purple	285.69	77.6231	0.99213	0.000836	73.714	red	99
AK236587.1	GYG1	purple	276.44	74.0479	0.98111	0.003108	235.772	green	99
AK236686.1	ZNF134	purple	223.09	50.5495	0.88963	0.043279	18.196	turquoise	99
AK236973.1	PLCL2	purple	197.61	43.7296	0.86454	0.058619	40.214	lightcyan	--
AB292846.1	NME1-NME2	purple	302.05	78.5426	0.994	0.000558	107.41	red	--
NM_001097474.1	COX7C	purple	241.59	49.7914	0.89124	0.042347	553.29	pink	0
NM_001112690.1	TNFSF13	purple	288.95	79.9821	0.9994	1.78E-05	11.448	navy	--
XM_001924715.1		purple	258.59	73.7403	0.98307	0.002636	23.472	darkorange	0
XM_001928143.1	ELL3	purple	290.59	79.5371	0.99782	0.000122	6.112	brown	0.2297
XM_001924332.1	SLC22A16	purple	148.37	39.6767	0.84219	0.07345	9.742	paleturquoise	0
XM_001926279.1	C1orf183	purple	285.98	79.2637	0.99744	0.000155	8.68	darkorange	0
XM_001925875.1	TPD52	purple	225.68	64.1194	0.95272	0.012253	19.742	pink	0
XM_001927180.1	ATP6V1E1	purple	256.83	57.8645	0.92249	0.025601	24.806	red	0
XM_001929541.1	ABCF1	purple	304.21	74.4423	0.98161	0.002985	31.47	blue	0
XM_001926225.1		purple	270.31	68.1145	0.95905	0.009887	70.666	blue	0
XM_001929009.1	ZRANB1	purple	145.28	36.2319	0.81551	0.092445	39.698	blue	0.1315
XM_001925019.1	LOC388789	purple	255.03	69.9215	0.96927	0.006436	16.212	skyblue	0
XM_001927209.1	CETN2	purple	233.11	45.0677	0.86841	0.056155	21.206	lightcyan	--
NM_213781.1	SCD	purple	299.32	79.0799	0.99586	0.00032	11.118	turquoise	0
NM_214391.1	FCGR3B	purple	240.3	63.8995	0.94792	0.014155	24.604	midnightblue	--
AY609726.1	ARF6	purple	272.91	77.0326	0.99221	0.000825	15.618	red	0
AY609732.1	PPP2CB	purple	250.59	66.7863	0.95679	0.01071	46.658	royalblue	0
AY610127.1	AK1	purple	275.88	67.2125	0.95815	0.010212	2090.66	brown	0
AY610374.1	RPL27A	purple	307.32	72.7516	0.97614	0.004408	130.65	blue	99
AY803100.1	CDC25B	purple	294.16	77.9776	0.99203	0.000854	3.562	brown	--
CF175213		purple	207.05	58.4349	0.93083	0.021611	138.328	blue	--
CF791685		purple	148.07	39.8696	0.84005	0.074918	25.174	blue	--
BX675792		purple	199.8	54.5783	0.91456	0.029591	31.928	skyblue	--
BX676378		purple	268.23	65.8664	0.95464	0.011519	75.458	red	99
BP169130		purple	283.81	78.0372	0.99417	0.000534	13.2	darkorange	--
BX915963		purple	276.49	75.7093	0.98632	0.001916	21.44	purple	--
BX918525		purple	254.32	64.6739	0.95049	0.013125	9.776	brown	--
BX918740		purple	305.68	74.8577	0.98253	0.002765	7.004	navy	--
BX925093		purple	229.39	51.4208	0.89382	0.040868	10.42	blue	--
CN156474		purple	262.82	66.3498	0.95482	0.011449	21.944	orange	--
CN156870		purple	274.11	71.4233	0.97181	0.005658	35.916	brown	--

DN101101	purple	218.59	61.9663	0.94133	0.016908	31.89	green	--	
DN104456	purple	264.29	62.9407	0.9421	0.016578	205.098	red	--	
DN111525	purple	288.93	79.0532	0.99629	0.000271	495.626	red	--	
DN116503	purple	113.37	21.153	0.72451	0.166215	25.598	red	--	
DN117644	purple	212.99	55.4514	0.91784	0.02792	19.614	purple	--	
DN118440	purple	289.74	77.3558	0.99118	0.000993	24.35	green	--	
DN121905	purple	286.48	79.7926	0.99907	3.42E-05	54.07	turquoise	--	
DR082636	purple	215.01	52.9985	0.90371	0.035348	29.486	green	--	
DR082404	purple	230.21	64.7959	0.95133	0.012796	1781.64	green	--	
AJ962420	purple	307.93	74.7262	0.9823	0.002818	7.252	red	--	
AJ962691	purple	248.36	59.4246	0.93024	0.021885	6.106	midnightblue	--	
DV904076	purple	300.62	76.2315	0.98648	0.001882	5564.096	pink	--	
DY437818	purple	252.3	59.0443	0.92975	0.022113	17.926	pink	--	
EB684337	purple	239.33	68.7068	0.96631	0.007386	27.248	midnightblue	--	
DB809172	purple	251.46	66.1762	0.95849	0.010089	13.638	magenta	--	
DB814005	purple	292.52	74.456	0.9822	0.002842	188.322	midnightblue	--	
DB814157	purple	297.62	78.2311	0.99286	0.000723	8.46	turquoise	--	
DB816263	purple	297.25	78.9782	0.99545	0.000368	25.656	brown	--	
DB805355	purple	228.8	64.0629	0.9481	0.014082	87.934	brown		0
EV892254	purple	262.95	66.4372	0.95592	0.011034	16.632	tan	--	
EV928670	purple	202.04	46.6025	0.88003	0.048973	32.198	turquoise	--	
EH412677	purple	296.48	79.5411	0.99709	0.000188	29.262	turquoise	--	
EV936704	purple	291.86	80.2206	0.99973	5.20E-06	36.304	brown	--	
EV958332	purple	299.64	74.5542	0.98228	0.002824	11.506	blue	--	
EV984595	purple	279.36	74.8607	0.98271	0.002722	94.166	red	--	
EV986754	purple	168.61	44.3608	0.87053	0.054825	5.518	navy	--	
EW079337	purple	281.02	77.9718	0.99443	0.000498	20.676	turquoise	--	
EW089029	purple	310.57	72.5515	0.97575	0.004517	15.91	brown	--	
EW093873	purple	281.38	69.6179	0.96588	0.007526	5.994	pink	--	
EW111843	purple	299.26	77.7003	0.99123	0.000984	8.294	turquoise	--	
EW150944	purple	138.96	32.8185	0.79317	0.109346	45.506	midnightblue	--	
EW161383	purple	204.6	52.5072	0.90614	0.034027	36.374	purple	--	
EW169113	purple	294	75.8805	0.9866	0.001858	7.782	turquoise	--	
EW176591	purple	284.15	78.599	0.99544	0.000369	8.656	red	--	
EW241204	purple	261.26	65.5736	0.9504	0.013163	13.952		--	
EW249451	purple	220.82	56.6486	0.92116	0.026257	112.576	purple		0
EW261110	purple	295.27	78.7221	0.99505	0.000417	13.864	turquoise	--	
EW268386	purple	305.1	76.1283	0.98644	0.001892	10.708	yellow		0
EW299585	purple	240.66	54.1439	0.91332	0.030232	26.768	orange	--	
EW301853	purple	294.41	76.8223	0.98916	0.001352	5.302	brown	--	
EW314396	purple	273.28	76.9921	0.9912	0.00099	38.5	navy	--	
EW384324	purple	271.46	76.1914	0.98872	0.001436	5.09	navy	--	
EW446752	purple	304.4	76.2283	0.98709	0.001758	6.024	blue	--	
EW471619	purple	237.76	67.476	0.96268	0.008605	731.748	midnightblue	--	
EW472852	purple	267.04	74.7903	0.9847	0.002266	19.74	green		99
EW528311	purple	306.42	76.3256	0.98734	0.001706	25.312		--	
EW587403	purple	282.75	78.6253	0.99613	0.000289	5.746	lightgreen	--	
EW595558	purple	191.69	46.493	0.87319	0.053163	1008.516	midnightblue	--	
EW611242	purple	289.68	78.5487	0.99426	0.000521	33.242	brown	--	
EW629816	purple	203.07	49	0.89242	0.041667	14.254	brown	--	
AW231897	purple	295.24	79.9108	0.9986	6.29E-05	15.684	blue	--	

BI400253	purple	306.74	74.5825	0.9821	0.002867	60.938	royalblue	--
BI182836	purple	241.85	52.8867	0.9072	0.033461	24.142	blue	99
BI185434	purple	285	78.8425	0.99645	0.000253	8.93	pink	--
BM190470	purple	281.4	71.974	0.97407	0.004994	4.432	brown	--
CA779010	purple	281.47	77.3017	0.99125	0.000981	38.982	lightcyan	--

Table S10-11. Module eigengene and its evolutionary rates in 15 postnatal highly LDE-specific modules (LDE_red).

pig_Genebank	pig_Gene	LDE Module	kTotal	kWithin	eigencorr	eigenpval	meanExpr	LTModule	Ka/Ks
AF319661.1	DDX58	red	913.65807	125.1059	0.995004	0.000424	2.626	yellow	--
AK234216.1		red	901.00883	126.8797	0.998079	0.000101	4.036	yellow	--
AK240376.1	RPL17P4	red	994.74738	115.0161	0.976786	0.004231	61.678	black	--
AK240398.1	ACAD9	red	957.63475	123.3656	0.991777	0.000894	9.194	blue	0
AK230922.1	METTL1	red	691.43369	104.5757	0.958691	0.010016	4.176	blue	--
AK240599.1	FTSJ2	red	917.55432	124.9813	0.994814	0.000448	11.08	blue	--
AK234682.1	APH1A	red	956.98073	121.0054	0.987676	0.001639	0.972	grey60	--
AK231373.1		red	864.47075	125.7806	0.99635	0.000265	4.38	yellow	99
AK238068.1		red	478.32021	79.20459	0.902978	0.035745	7.648	darkred	--
AK238113.1		red	931.76331	126.7145	0.997617	0.00014	3.212	lightgreen	--
AK234969.1		red	893.18817	124.7317	0.994526	0.000486	5.554	darkred	--
AK234990.1		red	758.83614	116.2891	0.980419	0.003279	1.954	yellow	--
AK235023.1		red	945.23832	124.8724	0.994387	0.000504	1.358	lightgreen	--
AK235076.1	MFSD8	red	860.27555	124.4075	0.994091	0.000545	3.702	yellow	0.184
AK231855.1	ABHD11	red	893.15251	126.5318	0.997562	0.000144	20.452	turquoise	99
AK231927.1		red	917.20999	127.2863	0.998598	6.30E-05	3.082	lightgreen	--
AK235508.1	PAPSS1	red	825.84961	121.2253	0.988976	0.001387	5.18	turquoise	--
AK238751.1	RNF8	red	799.45382	116.3184	0.98061	0.003232	2.174	royalblue	--
AK238837.1	ZBTB8OS	red	987.66865	117.1821	0.980895	0.003161	35.526	turquoise	--
AK236288.1	DIS3L	red	888.14051	124.5843	0.994239	0.000525	5.596	yellow	--
AK236349.1		red	852.28699	125.8689	0.996559	0.000242	5.452	yellow	--
AK230543.1	CLEC7A	red	894.62975	127.5842	0.999251	2.46E-05	2.438	darkred	0.2755
AK236478.1	PRMT5	red	824.65027	122.5679	0.991097	0.001007	67.94	violet	--
AK236482.1		red	760.06663	115.6475	0.979203	0.003589	5.59	turquoise	--
AK239676.1	RSL1D1	red	767.45465	114.0883	0.976372	0.004344	23.138	white	--
AK239731.1	AFF3	red	872.67918	126.5108	0.997608	0.00014	8.55	darkred	0.2063
AK239743.1	PLEKHO2	red	932.04923	127.0653	0.998211	9.08E-05	7.638	darkgreen	--
AK233007.1		red	921.04355	127.7156	0.999334	2.06E-05	200.636	yellow	--
AK236743.1	SGPL1	red	902.07225	125.177	0.995277	0.000389	13.696	darkred	--
AK237272.1	HSPB6	red	837.64075	123.3596	0.992532	0.000774	91.996	turquoise	--
NM_213824.2	TP53	red	688.9285	108.1017	0.96545	0.007669	6.396	blue	--
NM_001005152.2	FGL2	red	976.68448	119.4312	0.984954	0.00221	9.006	white	0
AB271920.1	ADAMTS2	red	923.25583	123.8154	0.992852	0.000725	4.388	turquoise	0.6218
EF468461.1		red	740.53342	113.5805	0.975552	0.004572	3.644	violet	--
NM_001101827.1	MKI67IP	red	965.55813	121.3097	0.988291	0.001518	31.556	white	0
NM_001111257.1	LMNA	red	932.29467	125.7967	0.996146	0.000287	27.128	violet	0.1866
XM_001927375.1	NSMAF	red	784.19887	114.9359	0.978128	0.00387	5.724	black	0.0736
XM_001925784.1	USP5	red	901.22843	126.7253	0.997773	0.000126	11.164	tan	0
XM_001928014.1	GMPR2	red	917.25604	125.3738	0.995437	0.00037	29.94	orange	99
XM_001929510.1	GTPBP2	red	953.13388	120.7126	0.987239	0.001727	10.718	grey60	--
XM_001925300.1	OXNAD1	red	867.37383	125.0467	0.995109	0.00041	31.006	black	0.2142
XM_001928803.1	EXOSC1	red	667.02878	99.74286	0.950039	0.013304	9.73	turquoise	0
XM_001929155.1	COMTD1	red	609.36606	95.76364	0.940901	0.017093	8.17	black	--
XM_001926296.1	C22orf36	red	873.43045	126.7124	0.997942	0.000112	14.72	black	0.2048

XM_001928131.1	ABCB6	red	819.82323	120.9218	0.988451	0.001487	10.556	blue	0.0452
NM_214115.1	LOC10029:	red	962.08587	123.0578	0.991194	0.000991	45.688	tan	0
NM_001001860.1	MAGED1	red	887.98496	126.3951	0.997273	0.000171	38.73	black	0
AY609561.1	UTP11L	red	878.09122	126.1448	0.997	0.000197	14.376	violet	0.4604
AY609709.1	NAA20	red	962.98044	120.3744	0.986786	0.00182	39.988	lightyellow	99
AY610308.1	MED31	red	577.04404	87.94755	0.924292	0.02472	26.934	turquoise	0
AY609650.1	REXO2	red	697.84066	107.557	0.964653	0.007935	64.99	violet	--
NM_001039750.1	MTX1	red	936.02577	123.191	0.991663	0.000913	4.418	turquoise	99
CB471233		red	785.58494	119.6048	0.986169	0.001949	2.59	lightgreen	--
CF365202		red	908.49305	127.6441	0.999264	2.40E-05	5.658	yellow	--
CF367186		red	953.52131	122.6398	0.990525	0.001106	4.854	turquoise	--
BX670323		red	913.6448	124.918	0.994715	0.000461	7.496	violet	0
BX672681		red	952.16383	123.1204	0.991359	0.000963	3.364	turquoise	--
BX674366		red	969.36198	120.1458	0.986232	0.001935	4.434	violet	--
BX674823		red	921.20767	125.9042	0.996351	0.000264	72.126	lightyellow	0.088
BP166381		red	750.81837	115.2453	0.978577	0.003752	2.204	darkturquois	--
CK458901		red	519.14329	81.97965	0.910176	0.031878	10.664	blue	--
CK459204		red	950.81714	123.5404	0.992086	0.000844	1.746	lightyellow	--
CK463769		red	938.19307	125.9801	0.996299	0.00027	2.452	violet	--
BX915248		red	900.72637	125.3658	0.995534	0.000358	2.238	yellow	--
BX920265		red	938.82636	125.8987	0.996158	0.000286	1.614	blue	0.3097
BX926893		red	901.54092	126.395	0.997281	0.00017	4.706	tan	--
CN156385		red	1001.434	113.8719	0.974677	0.004819	8.632	turquoise	--
CN159132		red	973.24303	117.774	0.98221	0.002841	21.38	blue	--
CJ010970		red	853.99243	123.2111	0.99206	0.000848	6.866	purple	--
CJ013003		red	775.65153	117.783	0.983059	0.00264	3.644	darkred	--
DN100796		red	958.36023	120.3065	0.986515	0.001876	2.482	tan	--
DN106237		red	901.00883	126.8797	0.998079	0.000101	4.036	yellow	--
DN110962		red	1010.9087	112.478	0.972145	0.005557	7.148	darkturquois	0
DN113111		red	894.62975	127.5842	0.999251	2.46E-05	0.256	violet	--
DN119094		red	887.26571	126.6702	0.997816	0.000122	2.934	yellow	--
DN124332		red	632.07488	95.59982	0.941173	0.016976	7.014	turquoise	--
DN124360		red	918.06453	127.1301	0.998333	8.17E-05	5.78	lightgreen	--
DN129203		red	824.62724	120.8235	0.988326	0.001511	4.478	yellow	--
DN130708		red	894.62975	127.5842	0.999251	2.46E-05	1.668	paleturquois	--
AJ939015		red	926.13641	126.1948	0.996852	0.000212	4.082	turquoise	--
BW981763		red	830.50899	122.707	0.991373	0.000961	12	violet	99
AJ947061		red	854.43061	125.4607	0.995883	0.000317	4.39	lightgreen	--
AJ949618		red	910.76223	124.3372	0.99368	0.000602	4.284	lightgreen	--
AJ950671		red	694.03523	108.2362	0.965815	0.007548	3.604	green	--
AJ951515		red	887.63265	126.7277	0.997908	0.000115	2.998	darkred	--
AJ952146		red	907.4193	126.7065	0.997703	0.000132	5.376	darkred	--
AJ952617		red	898.47308	123.8237	0.993019	0.000699	1.854	greenyellow	--
AJ955833		red	883.66829	126.0942	0.996891	0.000208	2.484	yellow	--
AJ955864		red	896.47829	126.9214	0.998098	9.96E-05	9.07	lightgreen	--
AJ957109		red	864.12154	126.3702	0.997392	0.00016	91.158	yellow	--
AJ958225		red	821.89563	122.1914	0.990604	0.001092	2.718	paleturquois	--
AJ961523		red	904.56782	124.8292	0.994564	0.000481	21.452	yellow	99

AJ962295	red	891.86133	127.1187	0.99853	6.76E-05	13.744	darkred	--	
AJ962384	red	893.75392	127.0423	0.998321	8.25E-05	2.43	darkturquois	--	
AJ962774	red	900.90829	125.2543	0.995397	0.000375	2.304	turquoise	--	
AJ964015	red	872.48026	126.1095	0.996964	0.000201	6.71	yellow	--	
DV224303	red	948.99039	124.0389	0.992948	0.00071	2.458	turquoise		0
CV870740	red	962.68352	120.5729	0.987066	0.001762	7.658	darkred	--	
CV871765	red	928.58718	126.9062	0.99791	0.000115	2.192	yellow	--	
DY406859	red	945.70932	124.7793	0.994226	0.000526	2.002	yellow	--	
DY424276	red	488.76196	79.13985	0.903882	0.035252	23.314	grey	--	
DY426213	red	927.30232	126.2258	0.996845	0.000213	3.946	lightgreen	--	
DY428676	red	763.09945	116.2536	0.980286	0.003313	2.64	turquoise	--	
EB411464	red	879.02141	125.7344	0.996309	0.000269	4.202	blue		99
DB814812	red	923.51728	126.9443	0.997999	0.000107	5.948	darkturquois	--	
DB812092	red	823.65106	120.0077	0.986841	0.001809	3.838	lightgreen	--	
DB807707	red	882.95563	125.9781	0.996704	0.000227	2.42	yellow	--	
DB807739	red	901.29773	125.6264	0.996013	0.000302	2.496	darkred	--	
DB786410	red	1003.8475	115.0659	0.976826	0.00422	6.858	grey60	--	
DB792839	red	924.30271	126.6343	0.997553	0.000145	9.862	yellow	--	
DB803541	red	609.83584	97.43844	0.944267	0.015661	7.042	turquoise	--	
DB800389	red	941.82462	122.9671	0.991167	0.000995	3.16	paleturquois	--	
EV884726	red	843.63929	123.6743	0.993037	0.000697	1.952	yellowgreen	--	
EV889016	red	927.51955	126.2899	0.996876	0.000209	3.088	darkturquois	--	
EV953169	red	990.78034	116.6299	0.97972	0.003456	32.846	turquoise	--	
EW038292	red	912.24855	125.7788	0.996207	0.00028	6.652	paleturquois	--	
EW042239	red	941.77689	125.8893	0.996198	0.000281	4.64	green	--	
EW191198	red	681.6595	106.3191	0.962009	0.008838	12.108	yellow	--	
EW304442	red	832.05018	120.256	0.987312	0.001712	6.848	turquoise	--	
EW357862	red	894.62975	127.5842	0.999251	2.46E-05	1.156	lightyellow	--	
EW383709	red	1010.0172	113.2408	0.973513	0.005154	24.956	turquoise		99
EW444151	red	884.33261	125.7215	0.996291	0.000271	4.904	black	--	
EW554481	red	1007.1251	115.0577	0.976904	0.004199	20.888	yellow	--	
EW558531	red	899.16037	126.0221	0.99669	0.000229	5.712	darkturquois	--	
EW605958	red	930.45374	126.765	0.997662	0.000136	2.064	skyblue	--	
EW607349	red	918.23482	124.5059	0.993996	0.000558	3.572	darkred	--	
EW633660	red	934.90685	125.9826	0.996318	0.000268	5.218	lightgreen	--	
EH007086	red	944.03277	123.5989	0.992417	0.000792	8.292		--	
AW431074	red	929.51148	126.8383	0.997791	0.000125	4.254	darkred	--	
AW785426	red	861.14398	126.0898	0.996915	0.000206	6.406	yellow	--	
BF702023	red	871.61577	125.8054	0.996471	0.000252	2.542	purple	--	
BQ598728	red	869.17738	126.7372	0.997935	0.000113	3.178	darkturquois	--	
BF712024	red	962.96654	122.2425	0.989963	0.001205	7.74	pink	--	
BQ597362	red	891.7913	127.4722	0.999067	3.42E-05	10.792	yellow	--	
BQ597700	red	932.31159	125.901	0.996196	0.000281	3.604	yellow	--	
BI404153	red	894.62975	127.5842	0.999251	2.46E-05	1.926	darkgreen	--	
BQ602547	red	937.17013	126.3104	0.996916	0.000206	5.588	yellow	--	

Table S10-12. Module eigengene and its evolutionary rates in 15 postnatal highly LDE-specific modules (LDE_royalblue).

pig_Genebank	pig_Gene	LDEModule	kTotal	kWithin	eigencorr	eigenpval	meanExpr	LTModule	Ka/Ks
NM_001044615.1	MED7	royalblue	555.24	61.4815	0.990139	0.00117366	6.016	blue	0
NM_001044620.1	RABGGTB	royalblue	851.19	51.4262	0.947371	0.01437837	106.52	darkorange	0
AK230737.1	FAM46A	royalblue	757.61	59.2258	0.979929	0.0034031	8.89	turquoise	--
AK230850.1	FEM1C	royalblue	592.4	62.462	0.993571	0.00061822	48.964	darkturquoi	--
AK237827.1	MARCKS	royalblue	694.85	56.7432	0.972789	0.00536635	67.862	yellow	--
AK231783.1	PDLIM3	royalblue	441.61	55.0558	0.964724	0.00791129	352.914	blue	0
AK231793.1	ZC3H11A	royalblue	360.43	46.21	0.924798	0.02447465	8.67	paleturquois	0
AK232067.1	MRPL45	royalblue	580.52	62.5318	0.99355	0.00062117	58.134	skyblue	--
AK238675.1	AHCYL2	royalblue	673.33	63.9553	0.998104	9.90E-05	1.61	greenyellow	--
AK236197.1	CHMP1B	royalblue	603.03	63.0982	0.995698	0.00033852	9.426		--
AK230525.1	TRIP12	royalblue	719.28	63.1366	0.995046	0.00041827	3.64	paleturquois	--
AK232987.1	SLC25A46	royalblue	619.42	64.2073	0.999225	2.59E-05	13.542	paleturquois	0
AK234088.1	ISCU	royalblue	618.56	64.3135	0.999558	1.11E-05	16.364	turquoise	99
NM_001097504.1	HSPH1	royalblue	392.93	50.8143	0.945736	0.01504983	9.998	navy	0
NM_001097435.1	P4HA1	royalblue	368.09	47.9616	0.932808	0.02069606	24.026	red	0.7591
NM_001105304.1	COPS4	royalblue	569.14	63.2297	0.995735	0.00033412	36.054	salmon	0
NM_001113053.1	IKBKG	royalblue	634.58	62.5714	0.994002	0.00055718	3.93	blue	--
NM_001122994.1	OAZ1	royalblue	547.45	62.3114	0.992383	0.00079706	72.584	greenyellow	99
NM_001123124.1	HIF1A	royalblue	474.49	56.8853	0.972016	0.00559588	4.5	darkred	0
XM_001926625.1	CTAGE5	royalblue	742	59.1842	0.980791	0.00318663	12.708	blue	--
XM_001928709.1	CCPG1	royalblue	769.08	59.7965	0.98224	0.00283358	9.4	salmon	0.1642
XM_001925291.1	MAPK6	royalblue	429.33	54.4914	0.961604	0.00897923	8.566	yellow	0
XM_001928869.1	PEX19	royalblue	712.48	61.9351	0.991114	0.00100414	44.664	grey60	0.1768
XM_001924342.1	UBR5	royalblue	665.15	63.1219	0.995471	0.00036563	11.508	salmon	0
XM_001926975.1	PTDSS1	royalblue	676.24	61.4331	0.989511	0.00128752	5.482		0.3803
XM_001928879.1	DNAJC14	royalblue	634.15	59.0368	0.981771	0.00294649	16.17	skyblue	0
XM_001928398.1	TTRAP	royalblue	545.72	61.4945	0.989617	0.00126811	8.662	paleturquois	99
XM_001928389.1	C6orf62	royalblue	744.17	62.0995	0.991277	0.00097676	152.064	salmon	99
XM_001928820.1	SNRPC	royalblue	825.01	53.7224	0.957111	0.01059347	18.634	navy	99
XM_001925268.1	FILIP1L	royalblue	576.85	63.1386	0.995633	0.00034617	11.228	darkred	--
NM_213973.1	HSP90AA1	royalblue	515.91	59.2303	0.981452	0.00302384	7.17	salmon	0
NM_213884.1	TOP2A	royalblue	398.43	50.4191	0.944665	0.01549497	0.988	brown	0.026
AY459532	UCHL1	royalblue	764.31	59.5693	0.981598	0.00298827	5.3	turquoise	--
NM_214331.1	HADH	royalblue	834.76	51.0571	0.945794	0.01502578	65.558	pink	0
NM_001001637.1	PDLIM3	royalblue	695.87	63.7116	0.997264	0.00017175	27.854	grey	0
NM_001001770.1	CYB5A	royalblue	633.03	58.6057	0.980181	0.0033393	56.194	navy	--
AY609477.1	MRPS36	royalblue	653.65	58.1423	0.978503	0.00377128	48.514	red	--
AY609755.1	TCEAL8	royalblue	817.73	57.0412	0.971343	0.00579844	28.266	blue	0
AY610051.1	NUDC	royalblue	680.25	62.9031	0.994462	0.00049425	26.502	blue	--
AY610485.1	CHMP5	royalblue	468.09	57.0524	0.972379	0.0054876	5.11	grey	99
NM_001009576.1	RDX	royalblue	498.16	59.5367	0.981957	0.00290144	10.516	darkgreen	0
CF181210		royalblue	393.91	50.2657	0.943765	0.01587235	228.74	paleturquois	--
CK452729		royalblue	710.6	55.9919	0.969604	0.00633232	8.048	turquoise	--
CK463560		royalblue	610.69	63.9509	0.998368	7.91E-05	11.612	pink	--
BX914689		royalblue	860.46	50.6981	0.944034	0.01575934	9.102	darkgrey	--

BX920496	royalblue	824.45	54.6895	0.961234	0.00910896	49.928	darkgreen		0
BX926630	royalblue	456.86	56.7753	0.971119	0.00586633	7.39	greenyellow	--	
CN156511	royalblue	766.02	60.1042	0.983223	0.00260195	4.262	navy		99
CN157146	royalblue	521.89	60.9494	0.987433	0.00168793	9.712	grey	--	
CN158127	royalblue	429.38	54.2368	0.960755	0.00927756	84.09	darkorange	--	
CN161882	royalblue	836.37	54.7678	0.961706	0.00894372	9.156	turquoise	--	
CN162353	royalblue	491.4	59.1304	0.980561	0.00324392	24.882	turquoise	--	
CJ011443	royalblue	688.98	63.4547	0.996444	0.00025441	8.51	salmon	--	
DN106500	royalblue	627.27	63.1767	0.995951	0.00030904	6.256	salmon	--	
DN113169	royalblue	766.27	58.8792	0.978516	0.003768	14.334	green		0
DN116324	royalblue	723.25	58.9473	0.979531	0.00350457	86.13	greenyellow	--	
DN118117	royalblue	592.54	59.8843	0.985088	0.00218106	8.178	blue	--	
BW961190	royalblue	774.85	60.5602	0.985313	0.00213191	5.708	darkgreen	--	
BW980850	royalblue	433.55	54.5993	0.96234	0.00872346	6.778			99
DV226542	royalblue	649.01	62.2045	0.992779	0.00073575	13.446	turquoise	--	
DY410806	royalblue	789.64	56.2061	0.967876	0.00687811	8.28	navy	--	
DB790069	royalblue	565.48	61.3361	0.989714	0.00125039	9.338	greenyellow	--	
DB787693	royalblue	564.93	57.5573	0.977078	0.00415169	9.456	yellow	--	
DT331423	royalblue	481.13	57.1685	0.973305	0.00521475	6.848	yellowgreen		99
DT332551	royalblue	749.53	60.4963	0.984834	0.00223688	4.198	violet	--	
DV938382	royalblue	650.8	63.6948	0.997431	0.00015621	12.11	turquoise	--	
EW241468	royalblue	400.73	51.5958	0.949147	0.01366066	89.386	midnightblu	--	
EW425085	royalblue	737.98	57.8444	0.975054	0.00471194	12.292	skyblue	--	
EW464984	royalblue	837.6	54.8923	0.962291	0.00874048	24.256	navy	--	
EW534397	royalblue	743.9	61.6944	0.989393	0.00130923	6.454	darkturquoi	--	
EW620642	royalblue	537.67	58.8261	0.981116	0.00310622	28.368	skyblue	--	
EW646525	royalblue	723.6	61.5283	0.989467	0.00129558	29.382	skyblue	--	
BF711302	royalblue	434.27	53.0581	0.957829	0.01032974	14.328	blue	--	

Table S10-13. Module eigengene and its evolutionary rates in 15 postnatal highly LDE-specific modules (LDE_salmon).

pig_Genebank	pig_Gene	LDEModule	kTotal	kWithin	eigencorr	eigenpval	meanExpr	LTModule	Ka/Ks
NM_213912.2	TNNI1	salmon	220.0595	71.2416	0.97987	0.0034182	550.324	brown	0
AK230715.1	MALAT1	salmon	186.0012	57.2749	0.93659	0.01898344	56.482	lightcyan	--
AK237384.1	MAPKAP1	salmon	165.4178	63.7533	0.95778	0.01034785	2.266	pink	99
AK240401.1	STAT5B	salmon	223.9655	77.4233	0.99829	8.50E-05	6.526	lightcyan	--
AK240417.1	ADI1	salmon	190.5261	66.4651	0.9666	0.00729203	22.01	pink	99
AK231006.1	IGJ	salmon	227.895	76.8968	0.99647	0.00025215	13.18	brown	--
AK231172.1	NDUFB7	salmon	241.5812	73.3676	0.98587	0.00201207	49.874	lightcyan	99
AK234615.1	NSD1	salmon	196.341	72.0289	0.98367	0.00249873	6.428	lightcyan	0
AK237832.1	LY6E	salmon	210.4273	57.3329	0.93164	0.02123333	3.486	pink	--
AK231412.1	SS18L2	salmon	162.1063	61.9465	0.95207	0.01250523	21.722	brown	99
AK238051.1	TOX	salmon	212.1188	69.1754	0.974	0.00501321	3.396	magenta	--
AK235232.1	USF2	salmon	191.4208	67.68	0.97058	0.00603074	26.928	brown	0
AK235609.1	LOC10012€	salmon	227.4921	77.5	0.9985	6.99E-05	11.912	pink	--
AK235749.1	MPHOSPH	salmon	228.0379	74.954	0.99078	0.00106052	8.802	brown	99
AK238850.1	KCTD2	salmon	217.7294	71.5611	0.98134	0.00305145	31.154		--
AK239106.1	CRKL	salmon	219.4323	73.2441	0.98597	0.00199039	88.07	red	--
AK236398.1	RNASEK	salmon	228.7277	72.9076	0.98419	0.00238145	20.292	magenta	0
AK239550.1	COPS8	salmon	209.5655	73.2576	0.98702	0.00177167	14.654	purple	0
AK232969.1	CLASP1	salmon	248.7166	67.4029	0.96766	0.00694864	39.42	orange	--
AK236727.1	C2orf64	salmon	158.7119	55.9628	0.9306	0.0217147	11.184	brown	--
AK239841.1		salmon	226.6611	71.0063	0.97897	0.00364951	4.924	brown	--
AK239935.1	NUTF2	salmon	205.366	69.3311	0.97502	0.00472095	40.856	brown	0
AK236863.1	PPP3CA	salmon	98.88597	39.8034	0.85851	0.06251493	17.924	brown	--
AK233340.1	MAPRE3	salmon	236.7717	75.7693	0.99296	0.00070888	31.4	blue	--
AK233423.1	WIPI1	salmon	219.5609	76.8745	0.99699	0.00019835	5.18	pink	0
AK233960.1	BCL2L14	salmon	220.0704	76.7583	0.99681	0.00021598	1.548	pink	0.392
XM_001924881.1	CA14	salmon	194.3165	62.9613	0.95461	0.01153001	50.076	pink	--
XM_001926198.1	PFDN5	salmon	217.2981	70.3534	0.97703	0.00416497	52.78	magenta	0
XM_001928655.1	CD83	salmon	99.29967	39.0639	0.8543	0.06528081	7.126	salmon	0
XM_001925495.1	GPHN	salmon	106.5485	41.9281	0.86915	0.05569046	13.894	sienna3	0
XM_001926121.1	MRPL52	salmon	227.0132	75.8519	0.99416	0.00053517	30.168	magenta	--
XM_001928656.1	TBCC	salmon	225.5724	77.699	0.99903	3.64E-05	5.78	brown	0.524
XM_001926988.1	POLR1D	salmon	233.5142	73.5302	0.98697	0.00178235	13.678	pink	0
XM_001927225.1	COX17	salmon	231.405	76.4857	0.99503	0.00041999	86.848	magenta	--
XM_001929344.1	CUTC	salmon	240.5771	74.1108	0.9881	0.0015563	16.526	royalblue	0.412
XM_001925381.1	IDE	salmon	165.3417	54.5175	0.92521	0.02427528	67.726	yellow	0
XM_001924074.1	HSBP1	salmon	240.0326	72.203	0.98189	0.00291742	68.692	lightcyan	--
NM_214039.1	ACADM	salmon	226.6346	64.9702	0.95851	0.01008214	85.154	pink	0
NM_214332.1	GNB2L1	salmon	226.1713	71.678	0.98086	0.00316884	355.768	lightcyan	--
AY609590.1	TXNL1	salmon	231.5141	70.0785	0.97543	0.00460495	11.61	turquoise	0
AY609717.1	CCT4	salmon	224.502	70.4743	0.97745	0.00405107	3.866	brown	0
AY609883.1	MRPL48	salmon	253.1887	65.5111	0.96046	0.00938183	15.078	orange	0
CB288239		salmon	216.4011	72.7888	0.985	0.00220126	5.7	brown	--
CF792873		salmon	239.3696	71.814	0.98147	0.00301969	21.244	white	--

BX666142	salmon	223.6251	72.4364	0.98356	0.00252304	11.56	magenta		99
BX666730	salmon	195.7066	72.7243	0.98592	0.00200127	5.518	magenta	--	
BX672343	salmon	166.843	65.0062	0.96216	0.00878575	8.344	green	--	
BX921221	salmon	230.6475	73.0269	0.98594	0.00199625	130.354	magenta	--	
BX921650	salmon	195.2692	70.0272	0.97776	0.00396752	18.358	royalblue	--	
CN161508	salmon	217.6747	67.449	0.96829	0.00674643	6.842	lightcyan	--	
AJ656502	salmon	205.5885	69.7549	0.97622	0.00438697	6.48	magenta	--	
CJ008036	salmon	162.628	59.408	0.94354	0.01596576	10.676	brown	--	
CJ009624	salmon	175.8258	53.3415	0.91933	0.02717104	5.83	brown	--	
CJ011598	salmon	235.4404	75.6912	0.99291	0.00071603	16.19	royalblue	--	
DN101483	salmon	211.8551	68.4055	0.97105	0.00588787	6.712	magenta	--	
DN109924	salmon	242.031	68.598	0.97055	0.0060409	9.064	lightcyan	--	
DN113943	salmon	199.1531	71.5818	0.98287	0.00268415	10.964	green	--	
DN115140	salmon	222.6352	76.7208	0.99654	0.00024404	45.472	pink	--	
DN120090	salmon	212.6175	76.0261	0.99491	0.00043596	15.48	brown	--	
DN128580	salmon	223.0864	68.9722	0.9735	0.00515921	62.25	magenta	--	
AJ954621	salmon	220.1494	75.087	0.99209	0.00084375	9.996	turquoise	--	
AJ956159	salmon	220.0704	76.7583	0.99681	0.00021598	1.438	grey	--	
AJ957614	salmon	227.302	70.7821	0.97923	0.0035826	20.71	brown	--	
AJ962054	salmon	231.0199	74.543	0.98991	0.00121392	4.072	red	--	
AJ962204	salmon	217.2313	72.3595	0.98344	0.00255225	36.902	brown	--	
AJ946071	salmon	192.3476	69.5445	0.97642	0.00433022	2.558	green	--	
CV877299	salmon	238.7673	74.8248	0.99046	0.0011161	15.874	yellow	--	
DY422640	salmon	239.785	68.1986	0.97024	0.00613541	30.376	blue	--	
CT863733	salmon	214.3111	75.7809	0.99413	0.0005399	21.34	brown	--	
DB815923	salmon	220.0704	76.7583	0.99681	0.00021598	1.492		--	
DT321969	salmon	200.3452	66.9192	0.96756	0.00697987	46.294	brown		0
EW112465	salmon	236.0096	72.6731	0.98355	0.00252614	6.776	brown	--	
EW125499	salmon	230.363	74.9473	0.99143	0.00095188	7.74	lightyellov		0
EW172087	salmon	246.4512	68.503	0.97086	0.00594554	12.616	pink	--	
EW195257	salmon	205.0976	71.9718	0.98321	0.00260495	3.638	brown	--	
EW196387	salmon	194.2183	58.7479	0.93888	0.01797357	4.47	brown	--	
EW212556	salmon	199.4484	65.9787	0.96427	0.00806333	12.008	pink	--	
EW241740	salmon	105.1686	39.3439	0.85744	0.06321375	49.154	greenyello	--	
EW247940	salmon	227.6226	77.8055	0.99927	2.37E-05	26.916	brown	--	
EW249232	salmon	252.2943	66.506	0.96379	0.00822756	11.49	black	--	
EW266485	salmon	227.5518	72.5204	0.9837	0.00249145	2.536	magenta		0
EW336642	salmon	180.1552	67.3753	0.97032	0.00611023	1.88	magenta	--	
EW365166	salmon	172.4542	66.0465	0.9652	0.0077511	6.01	blue	--	
EW588718	salmon	188.7809	69.5405	0.97599	0.00445033	8.478	violet		0
FL593096	salmon	222.5842	74.5085	0.99067	0.0010803	138.208	brown	--	
BF192577	salmon	221.6329	72.7901	0.98467	0.00227389	2.974	magenta	--	
BF712213	salmon	219.1419	75.5324	0.993	0.00070271	1.786	darkgrey	--	
BF711853	salmon	243.0613	71.8524	0.98096	0.00314396	4.4	royalblue	--	
BQ599187	salmon	215.8463	76.2957	0.99551	0.00036142	28.204	lightcyan	--	
BI181083	salmon	239.5978	71.3984	0.9791	0.00361545	22.114	brown		99

Table S10-14. Module eigengene and its evolutionary rates in 15 postnatal highly LDE-specific modules (LDE_skyblue).

pig_Genebank	pig_Gene	LDE Module	kTotal	kWithin	eigencorr	eigenpval	meanExpr	LTModule	Ka/Ks
AK240428.1	JUN	skyblue	467.24	44.12717	0.997942	0.00011	4.698	yellow	99
AK234533.1	UBXN4	skyblue	569.379	39.61232	0.973197	0.00525	64.332	skyblue	0
AK231233.1	USP24	skyblue	566.692	43.084276	0.991294	0.00097	28.652	salmon	0
AK231450.1	N4BP1	skyblue	479.335	44.382447	0.999099	3.25E-05	7.714	salmon	0.429
AK235378.1	C16orf5	skyblue	574.159	42.37714	0.987439	0.00169	14.834	green	--
AK235438.1	CAPRIN1	skyblue	627.884	39.929977	0.973089	0.00528	2.896	darkred	0
AK232237.1	SPARC	skyblue	403.372	42.044621	0.987174	0.00174	16.962	yellow	--
AK235771.1	LRRC57	skyblue	615.186	40.65116	0.977494	0.00404	23.278	royalblue	99
AK235963.1	TNFRSF1B	skyblue	505.948	43.192405	0.993232	0.00067	3.788	green	--
AK238834.1	PARP8	skyblue	290.191	31.813978	0.924094	0.02482	2.6	yellow	0
AK238998.1	ASNA1	skyblue	357.002	38.807828	0.969161	0.00647	56.894	purple	99
AK230486.1		skyblue	547.344	43.574733	0.994113	0.00054	1821.55	midnightblue	--
AK230621.1	DCAF10	skyblue	343.636	36.677224	0.957704	0.01038	26.226	turquoise	--
AK232881.1	ACAD8	skyblue	654.651	37.149394	0.956465	0.01083	26.306	navy	0.104
AK239745.1	C1orf174	skyblue	271.661	29.989971	0.910731	0.03159	17.766	navy	99
AK239944.1	CMPK1	skyblue	485.426	43.912914	0.996912	0.00021	17.25	green	0
AK233368.1	NAP1L1	skyblue	602.535	39.028518	0.969296	0.00643	21.496	white	0
NM_001077227.1	UCHL3	skyblue	508.977	44.024736	0.997201	0.00018	8.3	purple	0.336
NM_001123194.1	SERPING1	skyblue	700.157	34.426755	0.938736	0.01803	6.224	navy	0.041
XM_001929120.1	GMPR	skyblue	512.173	42.76928	0.991207	0.00099	63.398	greenyellow	0
XM_001924308.1	ZFP36L1	skyblue	495.231	42.948798	0.992296	0.00081	131.502	navy	0
XM_001924784.1	HECTD1	skyblue	638.257	37.797957	0.960863	0.00924	70.276	turquoise	--
XM_001928342.1	EIF3A	skyblue	651.759	38.349	0.963688	0.00826	126.964	white	0
XM_001925803.1	TTN	skyblue	360.954	37.847906	0.965231	0.00774	3064.368	green	--
NM_001130733.1	ADSL	skyblue	563.346	43.154678	0.991773	0.00089	74.936	salmon	0.135
NM_001142669.1	TEAD1	skyblue	497.605	44.332118	0.998688	5.70E-05	6.856	yellowgreen	0
NM_214144.1	PRDX5	skyblue	333.508	35.804342	0.951567	0.0127	22.21	brown	0
AY610454.1	ATP5G2	skyblue	403.473	42.095053	0.987389	0.0017	20.708	turquoise	--
CF787473		skyblue	651.119	38.392302	0.963945	0.00817	8.016	turquoise	--
BX665551		skyblue	491.964	44.562338	0.999844	2.34E-06	44.822	green	--
BX917182		skyblue	551.99	42.742191	0.9899	0.00122	12.306	red	--
BX918252		skyblue	616.667	40.130908	0.974627	0.00483	6.006	darkred	--
CN161493		skyblue	430.19	43.376659	0.994089	0.00055	235.046	yellow	--
CN162120		skyblue	442.6	42.756601	0.991446	0.00095	13.186	tan	--
CJ010759		skyblue	494.154	44.19388	0.998156	9.50E-05	10.974	midnightblue	--
CJ038862		skyblue	520.756	44.099292	0.997327	0.00017	13.52	brown	--
DN106398		skyblue	472.027	41.130205	0.983556	0.00253	10.712	grey	--
DN107605		skyblue	570.612	42.835213	0.989879	0.00122	22.116		0
DN107719		skyblue	544.196	43.3845	0.993292	0.00066	55.488	yellow	--
DN107929		skyblue	378.943	39.067438	0.971762	0.00567	8.832	purple	--
DN113717		skyblue	631.772	39.698691	0.971806	0.00566	224.852	purple	--
AJ956497		skyblue	421.359	42.616749	0.990397	0.00113	6.698	green	--
AJ964366		skyblue	329.397	34.818877	0.947886	0.01417	5.014	magenta	--
AJ945367		skyblue	644.281	38.782611	0.966454	0.00734	4.712	yellow	--

DV225345	skyblue	468.126	43.575738	0.995409	0.00037	10.512	turquoise		99
DY426215	skyblue	314.099	34.854221	0.94446	0.01558	11.978	darkred	--	
DY437498	skyblue	407.308	40.527168	0.980282	0.00331	8.862	greenyellow		99
DY437561	skyblue	262.947	28.91035	0.902661	0.03592	82.858	lightcyan	--	
EV935557	skyblue	411.941	41.319168	0.983884	0.00245	5.552	greenyellow	--	
EW239354	skyblue	439.063	43.250376	0.993753	0.00059	9.484	brown	--	
EW623854	skyblue	406.566	41.64748	0.985511	0.00209	163.098	grey	--	

Table S10-15. Module eigengene and its evolutionary rates in 15 postnatal highly LDE-specific modules (LDE_yellow).

pig_Genebank	pig_Gene	LDE Module	kTotal	kWithin	eigencorr	eigenpval	meanExpr	LTModule	Ka/Ks
NM_001044527.1	ENO3	yellow	150.35	69.68024	0.8490825	0.0687637	14058.178	green	0
NM_001044535.1	PSMD4	yellow	272.64	123.2125	0.9791383	0.0036057	56.038	turquoise	0
AK230718.1	SON	yellow	299.73	126.3428	0.9830424	0.0026441	2.622	turquoise	--
AK237425.1	AOC3	yellow	239.19	113.081	0.9602501	0.0094565	23.528	red	--
AK237433.1	TBC1D10B	yellow	310.27	127.8898	0.9879732	0.0015804	46.362	turquoise	0
AK230890.1	UBA3	yellow	217.75	81.86102	0.8835561	0.046857	43.832	red	--
AK234448.1	ACTR10	yellow	220.22	73.66665	0.8548278	0.0649334	22.672	tan	0
AK234624.1	TAP1	yellow	224.47	76.85822	0.8769221	0.050865	18.496	brown	--
AK234832.1	BTBD3	yellow	292.23	124.2921	0.9790574	0.0036267	14.936	turquoise	--
AK231509.1	FJX1	yellow	255.58	95.95749	0.9164941	0.0286018	2.134	lightyellow	0.178
AK234955.1	MAFG	yellow	313.32	129.048	0.9889041	0.0014007	18.378	turquoise	--
AK231548.1	PIGC	yellow	173.39	80.01532	0.8817118	0.0479608	145.702	greenyellow	0
AK231759.1	MAZ	yellow	198.27	90.30882	0.907552	0.0332709	57.926	green	99
AK231765.1	CAPN3	yellow	149.45	62.80127	0.8230331	0.0869549	33.966	green	--
AK231943.1	SERINC2	yellow	278.98	130.6087	0.9927755	0.0007363	22.928	darkgrey	0
AK235345.1	MLLT3	yellow	253.41	114.9926	0.9639104	0.0081854	8.196	grey60	0
AK231905.1	DNAJC5	yellow	279.91	123.3362	0.9780571	0.003889	49.066	blue	--
AK235380.1	STAT5B	yellow	303.88	121.1517	0.9738551	0.0050548	15.048	purple	--
AK238495.1	ZNF219	yellow	250.24	113.6208	0.9617572	0.0089259	14.206	lightcyan	--
AK232277.1	HSPB2	yellow	277.63	110.4317	0.9499479	0.0133407	81.342	turquoise	0
AK232326.1	WWP2	yellow	190.99	78.15219	0.8746578	0.0522566	3.706	turquoise	--
AK235638.1	HSPA1A	yellow	279.88	130.299	0.9919463	0.0008666	160.858	turquoise	--
AK235644.1	RAB5C	yellow	298.39	132.5922	0.9960409	0.0002989	53.794	turquoise	99
AK232367.1	EIF3I	yellow	295.08	129.9432	0.9913982	0.0009564	20.528	turquoise	0
AK232423.1	UBE2D4	yellow	120.95	52.77209	0.7879894	0.1133842	157.048	green	--
AK232491.1	PNPO	yellow	271.14	117.261	0.9664834	0.0073287	21.062	greenyellow	99
AK238776.1	TGIF1	yellow	272.61	85.27287	0.8896631	0.0432607	5.222	turquoise	99
AK232614.1	MAP7D1	yellow	281.53	122.8075	0.9769932	0.0041746	49.676	turquoise	99
AK236127.1	PABPC4	yellow	212.61	100.8208	0.9334858	0.0203856	175.8	blue	99
AK236301.1	G3BP1	yellow	260.6	104.9462	0.9395119	0.0176953	20.162	turquoise	--
AK236404.1	HNRNPUL1	yellow	272.43	92.41776	0.9079084	0.0330805	29.664	sienna3	99
AK236564.1	SPNS1	yellow	265.89	120.1303	0.9732843	0.0052208	15.784	tan	0
AK239436.1	MYOM1	yellow	198.09	89.36607	0.9033985	0.0355152	654.514	brown	0
AK239459.1	LOC390834	yellow	223.34	79.13654	0.8810003	0.0483888	302.608	brown	--
AK239526.1	TNFRSF13E	yellow	274.34	120.2084	0.9727702	0.0053718	16.896	turquoise	--
AK239597.1	DDB2	yellow	295.48	128.4236	0.9892914	0.0013281	15.094	navy	0
AK239729.1	TMEM100	yellow	195.35	75.3778	0.8656799	0.057889	11.824	orange	0
AK236609.1	CCDC85B	yellow	281.28	123.8628	0.9788894	0.0036703	12.642	turquoise	--
AK233229.1	MRPS2	yellow	242.02	102.4779	0.9346872	0.0198395	4.58	turquoise	99
AK239956.1	UTP14A	yellow	161.04	61.73221	0.8276705	0.0836211	31.54	turquoise	99
AK240023.1	TOP1	yellow	293.71	133.87	0.9977322	0.0001296	8.846	turquoise	--
AK240042.1	PDE6D	yellow	291.14	130.9967	0.9931899	0.0006739	6.692	navy	0
AK233383.1	CDC42BPA	yellow	303.26	133.4376	0.9966065	0.0002372	9.506	turquoise	--
AK233420.1	SCAND1	yellow	292.86	120.8428	0.9714359	0.0057703	73.478	turquoise	99

AK233465.1	NAA38	yellow	306.85	131.0274	0.9916412	0.0009162	8.352	blue	--	
AK240338.1	TMEM126B	yellow	256.71	115.6948	0.9639561	0.00817	8.99	turquoise		0.165
AK233778.1	GTF2A2	yellow	275.59	109.2782	0.9489619	0.0137348	36.06	turquoise		99
NM_001097419.1	PPPDE1	yellow	259.14	102.7603	0.9342677	0.0200296	46.69			0
NM_001097486.1	ATPIF1	yellow	311.62	125.9452	0.9824534	0.0027828	103.824	red		99
NM_001123203.1	PMEPA1	yellow	309.87	121.3457	0.9732352	0.0052351	10.174	grey60	--	
XM_001926129.1	PTGS1	yellow	310.75	130.6676	0.9920962	0.0008425	5.458	turquoise		0.132
XM_001925915.1	SMARCA2	yellow	244.95	117.2256	0.9679641	0.00685	54.804	pink		0.2
XM_001929399.1	GCHFR	yellow	256.94	122.0937	0.9767024	0.0042538	22.824	brown		99
XM_001927620.1	CYHR1	yellow	227.73	84.07104	0.8924751	0.0416357	26.878	pink		99
XM_001929384.1	CCBL2	yellow	258.41	121.3386	0.9762051	0.0043904	87.38	purple		99
XM_001928548.1	ARHGDIB	yellow	274.18	129.1361	0.9901892	0.0011648	8.506	turquoise	--	
XM_001925621.1	COPS7A	yellow	205.16	90.93051	0.9087516	0.0326314	86.31	royalblue		99
XM_001926748.1		yellow	292.71	130.6354	0.9924175	0.0007917	7.518	turquoise	--	
XM_001926160.1	ERH	yellow	310.66	127.2738	0.9854801	0.0020957	22.55	turquoise		99
XM_001924445.1	PSMB5	yellow	291.29	132.1887	0.9946441	0.0004702	178.25	darkmagenta		99
XM_001929172.1	HMG20A	yellow	238.67	73.0493	0.8540116	0.0654734	36.354	blue		0
XM_001927655.1	PFDN6	yellow	310.1	124.6682	0.9795862	0.0034905	5.028	purple	--	
XM_001928486.1	FXR1	yellow	147.5	66.37493	0.8407036	0.0744703	331.414	brown		0
XM_001924973.1	FBXW4	yellow	241.48	105.4772	0.9431859	0.0161169	66.414	green		0.166
XM_001928329.1	C1orf57	yellow	272.2	127.4598	0.9865489	0.0018689	46.458	pink	--	
XM_001927802.1	ANXA7	yellow	227.07	105.9774	0.9426674	0.0163367	184.598	skyblue		99
XM_001928616.1	NOC4L	yellow	282.9	113.4736	0.9564608	0.0108342	5.662	turquoise		0.133
NM_001135960.1	ABRA	yellow	236.81	110.0199	0.9515798	0.0126968	213.644	turquoise		0.08
NM_213954.1	ACO2	yellow	287.71	117.3438	0.9661605	0.0074345	372.544	royalblue		0
NM_213983.1	PSMC5	yellow	251.2	91.48086	0.90838	0.0328291	234.298	blue		0
NM_214157.1	SREBF1	yellow	268.02	125.6642	0.983462	0.0025467	10.046	darkgreen	--	
NM_214249.1	ATP1A1	yellow	176.65	82.23433	0.8844176	0.0463441	18.002	salmon		0
NM_214342.1	OXSR1	yellow	310.62	130.038	0.9906292	0.0010874	26.838	orange		0
NM_214381.1	CAMK2D	yellow	139.41	56.56788	0.800619	0.1036155	52.706	navy		99
AY609689.1	FH	yellow	248.68	99.34133	0.9278739	0.0229995	120.21	orange		0.136
AY610172.1	CAPZA2	yellow	228.87	103.8455	0.9387711	0.0180193	38.57	midnightblue	--	
NM_214042.1	NTAN1	yellow	262.98	118.6505	0.9702777	0.0061237	58.81	turquoise	--	
NM_001008688.1	CA3	yellow	165.31	72.3418	0.8549065	0.0648814	5923.556	green		0
NM_001037151.1	TPI1	yellow	239.57	112.6318	0.9579238	0.0102951	12.162	greenyellow		0
CB469596		yellow	292.49	122.0024	0.9744985	0.0048698	2.872	lightcyan	--	
CF179281		yellow	250.07	116.1927	0.9645336	0.0079751	21.078	sienna3	--	
CF365654		yellow	306.85	127.8551	0.9880101	0.0015732	28.05	yellowgreen	--	
CF367795		yellow	278.44	125.2798	0.9822155	0.0028394	15.526	purple	--	
BX666997		yellow	256.76	119.9654	0.9735468	0.0051442	349.08	green	--	
BX667232		yellow	262.01	79.056	0.8812521	0.0482371	5.872	brown	--	
BX669608		yellow	299.26	127.0128	0.9844446	0.0023235	8.298	brown	--	
BX670880		yellow	238.41	109.7413	0.9508722	0.0129747	416.9	turquoise	--	
BX671641		yellow	300.42	130.0715	0.9906018	0.0010922	4.114	salmon		0
BX672877		yellow	269.1	117.991	0.9685933	0.0066498	18.75	turquoise	--	
BX672955		yellow	315.56	122.475	0.9776034	0.00401	163.926	purple	--	
BX673135		yellow	299.96	134.8919	0.9994749	1.44E-05	36.39	turquoise		0
BX673343		yellow	301.67	131.5153	0.9942907	0.0005174	40.37	paleturquoise	--	

BX675885		yellow	312.92	127.9113	0.9876647	0.0016415	19.942	blue	--	
CK451444		yellow	243.8	108.5181	0.9492408	0.013623	8.734	turquoise	--	
CK453505		yellow	181.06	84.91013	0.8951572	0.0401043	44.296	turquoise	--	
CK454638		yellow	231.13	106.6568	0.9464514	0.0147549	7.118	red	--	
CK458661		yellow	300.3	134.6069	0.9988473	4.70E-05	44.074	turquoise	--	
CK459914		yellow	304.6	130.6498	0.9910994	0.0010067	6.404	turquoise	--	
CK461249		yellow	261.94	124.6444	0.9820805	0.0028718	3.42	salmon	--	
CK463421		yellow	293.2	133.2237	0.9967039	0.000227	19.078	turquoise	--	
CK467233		yellow	208.76	86.25977	0.9001293	0.0373143	12.398	green	--	
CK467821		yellow	283.44	128.1575	0.9880101	0.0015732	98.382	green	--	
BX916347		yellow	313.54	122.6011	0.9783581	0.0038095	10.952	turquoise	--	
BX921055		yellow	294.55	134.6428	0.9993096	2.18E-05	24.054	royalblue	--	
BX921809		yellow	285.72	121.4485	0.9772958	0.0040927	64.642	green	--	
BX922353		yellow	135.31	61.325	0.8218586	0.0878055	17.572	grey	--	
BX926193		yellow	251.06	101.6859	0.9326362	0.0207748	29.664	turquoise		99
BX926422		yellow	289.78	132.6136	0.9958964	0.0003154	2.772	yellow	--	
CN153435		yellow	300.6	124.5212	0.9800091	0.0033828	7.506	green	--	
CN154075		yellow	181.29	57.53216	0.8137062	0.0937794	12.772	darkturquois	--	
CN158750		yellow	285.79	112.1137	0.9530605	0.0121216	5.86	turquoise	--	
CO946908		yellow	263.82	115.8307	0.9641506	0.0081042	7.47	turquoise	--	
DN100886		yellow	181.31	83.85524	0.8881884	0.0441207	35.788	blue	--	
DN100939		yellow	294.25	125.8159	0.9817167	0.0029595	34.43	skyblue	--	
DN104085		yellow	309.56	119.8472	0.9712165	0.0058367	11.616	black		99
DN104365		yellow	310.32	125.3929	0.9818273	0.0029328	19.37	turquoise	--	
DN108916		yellow	290.68	133.3357	0.9969047	0.0002066	6.828	yellow	--	
DN111262		yellow	305.49	124.1805	0.9815683	0.0029955	191.884	purple	--	
DN112619		yellow	308.04	123.3268	0.976765	0.0042367	17.738	sienna3	--	
DN116744		yellow	232.5	104.7066	0.9419965	0.0166227	5.142	royalblue	--	
DN117127		yellow	225.96	67.68163	0.8347634	0.0786005	84.39	navy	--	
DN120072		yellow	165.29	58.51015	0.8125876	0.0946083	37.442	blue		99
DN124256		yellow	226.62	69.97051	0.8425312	0.0732136	59.888	turquoise	--	
BW960627		yellow	285.13	132.1649	0.995312	0.000385	15.582	orange	--	
BW979917		yellow	244.98	100.1102	0.9281143	0.0228854	16.132	grey60	--	
AJ955027		yellow	309.58	129.0931	0.9881533	0.0015451	18.932	midnightblue		99
AJ955039		yellow	218.66	99.70795	0.9324243	0.0208722	14.254	brown	--	
DV225793		yellow	243.04	105.4319	0.9416066	0.0167895	71.006	violet	--	
DY405801		yellow	244.68	114.894	0.9633704	0.0083692	15.048	turquoise	--	
DY423291		yellow	214	97.49306	0.9230685	0.0253171	19.1	yellow	--	
EB418537		yellow	312.05	124.1557	0.9813429	0.0030506	11.194	brown	--	
DB786327		yellow	227.4	105.4613	0.9448779	0.0154063	11.536	brown		99
AK230483	HLA-DQA1	yellow	278.55	116.6748	0.9672089	0.0070928	8.408	magenta	--	
ES444893		yellow	303.03	117.1927	0.9662687	0.007399	9.016	salmon	--	
EV872189		yellow	216.36	100.7294	0.9317023	0.0212052	79.704	midnightblue	--	
EV873204		yellow	298.22	122.6214	0.9783122	0.0038216	1144.25	darkorange	--	
EV889890		yellow	284.97	127.324	0.9867332	0.0018307	21.582	grey60	--	
EV890051		yellow	307.26	122.7174	0.9760711	0.0044275	289.064	orange		99
EV893036		yellow	259.9	121.7594	0.9768124	0.0042238	10.302	green	--	
EV928650		yellow	228.95	84.33437	0.8903253	0.0428763	9.948	tan	--	

EV986176	yellow	301.2	132.4954	0.9948198	0.0004472	24.334	midnightblue	--	
EW074931	yellow	165.52	63.78309	0.8307772	0.0814103	64.968	green	--	
EW079375	yellow	315.45	123.6314	0.979667	0.0034698	3.702	pink	--	
EW079644	yellow	206.62	91.88704	0.9141338	0.0298121	4.198	brown	--	
EW093641	yellow	267.7	116.739	0.9678708	0.0068799	13.466	blue		99
EW182201	yellow	269.3	118.3241	0.9685539	0.0066623	4.82	green	--	
EW189902	yellow	296.21	134.2089	0.9982238	8.98E-05	8.524	blue	--	
EW205532	yellow	307.91	124.5599	0.9822976	0.0028199	61.324	blue	--	
EW237006	yellow	243.3	112.508	0.9591166	0.0098622	10.412	green	--	
EW238443	yellow	230.18	103.0099	0.9373776	0.018634	71.61	green	--	
EW240620	yellow	309.98	128.539	0.9890708	0.0013693	12.748	green	--	
EW246316	yellow	179.21	75.28159	0.8647758	0.0584661	72.332	blue	--	
EW253506	yellow	299.72	127.3242	0.9852628	0.0021429	16.392	green	--	
EW254039	yellow	294.15	115.9939	0.9641214	0.008114	246.866	turquoise	--	
EW264653	yellow	240.41	107.3145	0.9480987	0.0140829	19.336	turquoise	--	
EW269125	yellow	306.59	128.7591	0.9890841	0.0013668	14.922	turquoise	--	
EW276793	yellow	313.96	128.7243	0.9884068	0.0014958	5.882	turquoise	--	
EW278920	yellow	252.68	117.3597	0.9675591	0.0069799	19.888	paleturquoise	--	
EW367525	yellow	242.26	111.2104	0.956681	0.0107525	203.338	royalblue	--	
EW408987	yellow	289.51	133.0718	0.9965631	0.0002417	2.122	darkorange	--	
EW429561	yellow	307.5	130.7392	0.9921974	0.0008264	15.368	blue	--	
EW524740	yellow	273.4	105.5872	0.9390797	0.0178841	2.674	navy	--	
EW528694	yellow	269.89	103.8707	0.9361291	0.0191904	78.944	turquoise	--	
EW565938	yellow	284.46	130.0728	0.9914998	0.0009396	120.698	greenyellow	--	
EW568518	yellow	268.66	123.1864	0.9794117	0.0035352	5.49	lightcyan	--	
EW573852	yellow	276.27	128.0981	0.9879604	0.0015829	17.43	purple	--	
EW590550	yellow	313.09	129.5389	0.9902171	0.0011598	102.718	turquoise	--	
EW641588	yellow	154.06	60.85641	0.8211611	0.0883119	20.542	royalblue	--	
EW671244	yellow	236	84.44784	0.886904	0.0448742	15.364	red	--	
BF441130	yellow	275.57	125.6439	0.9833469	0.0025733	3.448	brown	--	
BQ598826	yellow	306.09	128.5338	0.9883906	0.001499	31.642	green	--	
BM190355	yellow	210.3	97.10964	0.9220485	0.0258182	93.966	turquoise		0

Table S11-1. Module eigengene and its evolutionary rates in 13 postnatal highly LT-specific modules (LT_black).

pig_Genebank	pig_Gene	LT Module	kTotal	kWithin	eigencorr	eigenpval	meanExpr	LDEModule	Ka/Ks
NM_001044593.1	MYOZ2	black	980.75	96.831	0.9938335	0.000581	518.232	white	99
NM_001044614.1	TXNIP	black	956.94	96.175	0.9928753	0.000721	144.45	turquoise	0
AK234286.1	PER1	black	958.4	98.866	0.9986797	5.76E-05	30.558	blue	99
AK230834.1	TAF12	black	836.9	90.376	0.9802317	0.003327	27.676	blue	--
AK240376.1	RPL17P4	black	937.68	97.145	0.9947677	0.000454	92.706	red	--
AK234534.1	CSRP1	black	943.76	98.184	0.9973253	0.000166	21.136	darkgrey	0
AK237626.1	C1orf69	black	964.33	94.415	0.9887768	0.001425	9.05	navy	99
AK237638.1	PLEKHJ1	black	950.22	86.276	0.9711145	0.005868	13.312	turquoise	99
AK231149.1	DNAJC24	black	846.54	91.14	0.9819183	0.002911	15.298	turquoise	--
AK237784.1	TBC1D1	black	988.96	94.287	0.9882496	0.001526	10.368	turquoise	0
AK231556.1	HSF2	black	857.88	82.185	0.9614733	0.009025	52.83	midnightblue	99
AK235255.1	TMEM131	black	862.4	90.056	0.9794751	0.003519	45.962	darkturquoise	99
AK232059.1	TIMM44	black	931.78	95.858	0.9919891	0.00086	16.086	white	99
AK235887.1	WDR74	black	917.68	76.482	0.9469944	0.014532	7.918	turquoise	0.091
AK238807.1	PRELID1	black	925.82	85.379	0.9687536	0.006599	29.456	turquoise	--
AK232622.1	GABARAPL1	black	948.19	96.899	0.9941135	0.000542	16.154	turquoise	--
AK236159.1	ATP6V0E1	black	980.77	96.031	0.9921035	0.000841	16.602		--
AK236249.1	VAT1	black	925.55	95.286	0.9911009	0.001006	14.344	turquoise	0
AK239346.1	UBE2F	black	972.53	98.89	0.9986697	5.82E-05	10.284	navy	0
AK236497.1	PREB	black	980.98	99.086	0.9991551	2.95E-05	15.732	darkorange	0.401
AK239601.1	ACOT1	black	956	97.405	0.9952451	0.000393	21.456	blue	--
AK239710.1	PRPSAP1	black	730.47	75.764	0.9448006	0.015439	5.234	black	0
AK240077.1	SUMO2	black	947.23	87.205	0.9729412	0.005321	104.51	lightyellow	99
AK240079.1	PDGFRB	black	889.92	87.252	0.9734332	0.005177	9.056	midnightblue	--
AK240201.1	EIF4G1	black	962.12	97.589	0.9959864	0.000305	18.326	turquoise	--
NM_214014.2	FOXO1	black	747.99	77.724	0.9499353	0.013346	3.906	lightcyan	99
NM_214343.2	QDPR	black	980.2	98.19	0.9968723	0.00021	34.384	green	0
NM_001044575.2	IDH3B	black	943.64	97.248	0.9949259	0.000434	102.608	black	0
NM_001123135.1	PLIN5	black	930.96	96.798	0.994578	0.000479	3.848	darkgrey	99
NM_001123213.1	VAPB	black	972.6	97.644	0.9960558	0.000297	58.106	blue	99
XM_001928505.1	WDYHV1	black	982.32	98.688	0.9980029	0.000107	16.798	midnightblue	0.077
XM_001927918.1	CASQ2	black	893.81	90.778	0.9813347	0.003053	12.638	pink	0.065
XM_001927375.1	NSMAF	black	956.21	94.545	0.9893576	0.001316	9.32	red	0.074
XM_001928852.1	C6orf145	black	953.79	98.524	0.9980035	0.000107	30.15	turquoise	99
XM_001927771.1	FBXL3	black	935.79	96.674	0.9938572	0.000577	25.316	turquoise	99
XM_001925300.1	OXNAD1	black	960.21	96.504	0.9931408	0.000681	33.384	red	0.214
XM_001929115.1	ANXA8	black	977.24	99.251	0.9993493	1.99E-05	6.576	turquoise	0.263
XM_001926555.1	LOC100287932	black	970.44	98.55	0.9977315	0.00013	46.264	turquoise	--
XM_001929155.1	COMTD1	black	988.21	97.15	0.994595	0.000477	9.058	red	--
XM_001929270.1	ACACB	black	989.98	98.797	0.9982989	8.42E-05	57.244	turquoise	0.024
XM_001928076.1	YWHAH	black	991.78	97.85	0.9962396	0.000277	44.674	white	0
XM_001926296.1	C22orf36	black	919.13	95.694	0.9922125	0.000824	6.556	red	0.205
XM_001929539.1	PPIL2	black	958.69	93.676	0.9870542	0.001765	6.796	blue	0
XM_001924998.1	SSB	black	982.46	98.286	0.9972302	0.000175	53.948	turquoise	0

XM_001928889.1	ACOT8	black	967.24	98.896	0.9985421	6.68E-05	20.48	pink	0.067
XM_001927440.1	SLC25A5	black	993.32	96.204	0.9925977	0.000764	15.008	navy	0
XM_001926010.1	IGSF1	black	964.62	97.344	0.9949983	0.000424	2.638	violet	0.799
NM_213904.1	GANAB	black	691.81	67.444	0.9219954	0.025844	13.828	blue	0
NM_213792.1	HOPX	black	920.2	96.698	0.9941837	0.000532	12.212	violet	99
NM_001001860.1	MAGED1	black	985.93	94.902	0.9897991	0.001235	55.85	red	0
AY609612.1	GDE1	black	980.61	97.019	0.9942509	0.000523	66.864	darkgrey	0
AY609874.1	RAB18	black	968.49	99.232	0.9993618	1.94E-05	86.276	brown	0
AY610021.1	SNRPD1	black	982.26	99.216	0.9992396	2.52E-05	16.258	violet	0
AY610107.1	CBX5	black	948.95	87.086	0.972772	0.005371	43.348	blue	--
AY610175.1	MAGOH	black	981.43	98.184	0.9969973	0.000197	10.502	turquoise	99
AY610191.1	TIMM17A	black	963.52	96.419	0.9929198	0.000714	27.32	turquoise	--
CF178224		black	905.97	92.551	0.9848432	0.002235	6.756	turquoise	0
BX667112		black	961.78	90.194	0.9801984	0.003335	97.84	violet	--
BX670270		black	892.26	94.016	0.9885193	0.001474	27.612	turquoise	--
BX670342		black	977.76	91.477	0.9820887	0.00287	50.314	green	--
BX673567		black	977.39	98.946	0.9986131	6.20E-05	29.604	lightcyan	--
BP159931		black	885.92	92.667	0.9851318	0.002171	16.164	turquoise	--
BP163106		black	936.59	92.179	0.9838603	0.002455	9.164	green	--
CK454369		black	991.68	95.334	0.9905934	0.001094	15.158	navy	--
CK458404		black	807.57	87.683	0.9740609	0.004995	5.108	blue	0
CK463600		black	843.15	88.594	0.9765763	0.004288	31.506	brown	--
CK467671		black	993.38	94.713	0.9892806	0.00133	24.012	turquoise	--
BX915635		black	944.05	97.944	0.9969916	0.000198	3.692	black	--
BX917599		black	976.41	97.086	0.9945891	0.000477	22.194	black	0
BX921788		black	965.42	96.063	0.9923173	0.000807	43.786	green	--
BX923137		black	926.75	84.515	0.9669714	0.00717	12.986	turquoise	--
BX923538		black	955.25	91.934	0.9835644	0.002523	45.992	darkturquoise	--
BX926942		black	993.79	95.544	0.9910961	0.001007	23.4	midnightblue	--
CN155836		black	978.26	96.887	0.993946	0.000565	19.508	grey60	--
CN158795		black	978.57	98.528	0.9977143	0.000131	17.786	turquoise	--
CN159661		black	981.35	94.554	0.988761	0.001428	10.178	grey60	--
CN162200		black	867.23	79.967	0.9561563	0.010948	10.074	lightyellow	0.492
CN163978		black	948.7	96.274	0.9928891	0.000719	9.656	turquoise	--
CN164046		black	906.03	94.358	0.9887736	0.001425	2.944	lightgreen	--
CJ010554		black	960.74	98.581	0.997886	0.000117	9.566	turquoise	99
CJ012319		black	953.82	98.513	0.997979	0.000109	29.456	turquoise	--
CJ025623		black	978.74	96.414	0.9928735	0.000721	26.404	turquoise	--
CJ027205		black	961.83	98.488	0.9980414	0.000104	16.12	turquoise	99
DN104085		black	621.69	64.201	0.9116713	0.031092	3.822	yellow	99
DN106307		black	802.9	78.084	0.9511863	0.012851	36.876	blue	--
DN113940		black	945.77	97.37	0.9952009	0.000399	8.934	blue	--
BW956903		black	805.33	77.382	0.9495562	0.013497	2.598	midnightblue	--
BW980990		black	963.83	98.302	0.9972205	0.000176	11.126	turquoise	--
AJ960916		black	960.39	96.675	0.9934868	0.00063	13.646	turquoise	--
AJ944959		black	865.51	84.353	0.9664075	0.007354	19.6	magenta	--
DV226293		black	923.31	86.636	0.9717132	0.005687	54.968	turquoise	0
DY417691		black	870.1	91.873	0.9832853	0.002588	26.368	green	--

DY427781	black	980.12	99.138	0.9990538	3.49E-05	6.012	lightgreen	--	
DY433232	black	938.71	97.562	0.9961556	0.000286	16.642	white	--	
DB802144	black	978.53	98.202	0.9971961	0.000178	22.07	turquoise		0
DT331317	black	649.8	65.987	0.9170508	0.028319	3.366	black		0
DT331844	black	956.64	82.497	0.9614408	0.009036	2.024	orange	--	
EW177852	black	927.93	85.919	0.9703656	0.006097	7.034	midnightblue	--	
EW249232	black	859.7	87.856	0.9747957	0.004785	3.174	salmon	--	
EW266525	black	964.58	99.064	0.9989998	3.80E-05	20.564	blue		99
EW292285	black	946.48	97.738	0.9966014	0.000238	21.53	turquoise	--	
EW413069	black	998.9	96.689	0.9937209	0.000597	165.918	turquoise	--	
EW444151	black	810.98	77.332	0.9492504	0.013619	3.658	red	--	
EW595278	black	976	88.699	0.9764099	0.004334	22.202	pink		99
EW640642	black	986.1	95.679	0.9913047	0.000972	31.28	lightcyan	--	
EX655280	black	934.97	97.234	0.9952413	0.000394	22.896	lightcyan	--	
BQ603893	black	935.68	96.708	0.9937732	0.000589	5.332	violet	--	
BQ605071	black	882.95	94.182	0.9887828	0.001424	4.226	white	--	

Table S11-2. Module eigengene and its evolutionary rates in 13 postnatal highly LT-specific modules (LT_darkmagenta).

pig_Genebank	pig_Gene	LDE Module	LTModule	kTotal	kWithin	eigencorr	eigenpval	meanExpr	Ka/Ks
NM_001044545.1	PLAGL1	white	darkmagenta	973.9	26.857	0.99297	0.000707	21.52	0
AK234337.1	TBC1D17	blue	darkmagenta	794.34	23.263	0.96422	0.008082	18.468	99
AK230893.1	NECAP1	blue	darkmagenta	972.55	26.196	0.98738	0.001698	14.17	--
AK231707.1	FBL	turquoise	darkmagenta	907.48	25.348	0.98155	0.003001	32.212	99
AK236196.1	DDA1	tan	darkmagenta	967.21	24.979	0.97859	0.003749	23.924	--
AK236528.1	RHEB	darkorange	darkmagenta	962.48	26.916	0.99329	0.000659	100.946	0
AK233343.1	RBM10	black	darkmagenta	805.8	23.314	0.96434	0.00804	53.59	0
AK240018.1	PQLC2	turquoise	darkmagenta	949.23	26.909	0.99342	0.00064	4.338	0
NM_001123090.1	C6orf153	turquoise	darkmagenta	956.76	26.518	0.99006	0.001188	22.168	--
XM_001925477.1	ITGA9	darkgrey	darkmagenta	961.58	26.916	0.99343	0.000638	15.556	99
XM_001929169.1	RANGAP1	turquoise	darkmagenta	886.13	25.264	0.9805	0.00326	9.87	0
XM_001929457.1	SHMT2	turquoise	darkmagenta	893.67	25.098	0.97969	0.003464	12.368	--
XM_001924445.1	PSMB5	yellow	darkmagenta	962.09	26.738	0.99177	0.000895	191.306	99
XM_001927395.1	SFXN4	blue	darkmagenta	909.67	25.835	0.985	0.0022	14.356	0.881
XM_001928374.1	NDST2	black	darkmagenta	985.76	25.499	0.98216	0.002853	27.514	99
XM_001925471.1	GPC4	turquoise	darkmagenta	947.5	26.669	0.99158	0.000927	5.252	--
NM_214063.1	HMGB2	turquoise	darkmagenta	982.01	26.574	0.99079	0.00106	9.066	0.823
NM_001001537.1	ITIH4	lightyellow	darkmagenta	831.11	23.583	0.96706	0.00714	5.638	0.17
AY610003.1	ISCA2	turquoise	darkmagenta	973.07	26.246	0.98825	0.001527	11.498	99
BX675434		pink	darkmagenta	989.59	26.518	0.99002	0.001196	27.728	--
BX676463		blue	darkmagenta	933.87	26.489	0.99002	0.001196	10.248	--
BP164138		brown	darkmagenta	983.15	26.085	0.98655	0.001868	20.47	--
BP171209		turquoise	darkmagenta	939.32	26.955	0.99373	0.000596	8.774	--
CK466455		white	darkmagenta	987.17	27.198	0.99534	0.000382	1.27	--
BX923017		green	darkmagenta	955.21	25.048	0.9792	0.003589	34.182	99
CN155162		white	darkmagenta	928.78	25.633	0.98342	0.002556	18.712	--
DN116668		blue	darkmagenta	914.9	26.558	0.99084	0.001051	6.57	--
DT322875		pink	darkmagenta	987.52	26.695	0.99138	0.00096	978.536	--
EW133862		darkgreen	darkmagenta	974.71	26.139	0.98692	0.001791	477.792	--
EW569616		blue	darkmagenta	959.25	26.851	0.99294	0.000711	13.432	--
BF712794		darkgreen	darkmagenta	803.3	22.996	0.96142	0.009044	4.756	--

Table S11-3. Module eigengene and its evolutionary rates in 13 postnatal highly LT-specific modules (LT_grey60).

pig_Genebank	pig_Gene	LDEModule	LT Module	kTotal	kWithin	eigencorr	eigenpval	meanExpr	Ka/Ks
AK231088.1	NMD3	turquoise	grey60	923.1	62.471	0.9956	0.00035	40.222	0
AK234682.1	APH1A	red	grey60	971.7	62.824	0.99674	0.000223	4.054	--
AK235131.1	U2AF1	black	grey60	954.6	62.961	0.99719	0.000179	81.826	--
AK235345.1	MLLT3	yellow	grey60	855	58.325	0.98157	0.002996	12.862	0
AK232271.1	PHB2	blue	grey60	974.7	61.168	0.99136	0.000962	100.97	0
AK235894.1	GNL3L	blue	grey60	981.2	59.967	0.98721	0.001733	16.17	0.102
AK232688.1	ABCF3	black	grey60	839.6	57.383	0.97833	0.003816	15.008	0
AK236271.1	MED9	turquoise	grey60	835.9	58.309	0.98157	0.002995	5.462	--
AK239254.1	GAR1	turquoise	grey60	940.3	62.813	0.99666	0.000232	7.26	99
AK230605.1	CCNC	midnightblue	grey60	946.7	60.674	0.98955	0.001281	17.05	0
AK239563.1	CCT5	midnightblue	grey60	974.8	63.141	0.99772	0.000131	81.628	--
AK239689.1	PSMD8	darkorange	grey60	976.6	59.978	0.98724	0.001727	72.864	0
AK240022.1	MRTO4	violet	grey60	964.8	62.17	0.9945	0.000489	32.852	0.508
AK233680.1	MRPL36	blue	grey60	950.1	59.842	0.98684	0.001809	7.5	99
AK237314.1	DNAJB5	darkmagenta	grey60	929.9	62.942	0.99713	0.000184	17.914	0
AK234149.1	CD320	pink	grey60	950.7	63.494	0.9989	4.39E-05	2.378	--
NM_001078679.1	CD81	blue	grey60	924.3	61.706	0.99302	0.000699	10.154	--
EU561660.1	CFL2	turquoise	grey60	876.2	61.074	0.99099	0.001026	476.374	99
NM_001123203.1	PMEPA1	yellow	grey60	951.1	61.938	0.99376	0.000591	12.782	--
XM_001928937.1	SIX1	darkorange	grey60	998.3	59.541	0.98572	0.002043	126.722	0
XM_001927513.1	MPHOSPH10	black	grey60	961.8	60.815	0.98995	0.001208	9.534	99
XM_001924580.1	C15orf61	pink	grey60	924.6	62.291	0.99495	0.00043	70.518	--
XM_001928869.1	PEX19	royalblue	grey60	956.5	62.974	0.99726	0.000172	55.492	0.177
XM_001926890.1	CPSF2	turquoise	grey60	941.9	62.29	0.99506	0.000417	35.566	0
XM_001929510.1	GTPBP2	red	grey60	804.5	54.648	0.96835	0.006728	9.754	--
XM_001927951.1	PPP1R11	greenyellow	grey60	975.5	61.86	0.99351	0.000627	3.166	--
XM_001928445.1	C6orf1	greenyellow	grey60	961.9	60.355	0.98838	0.0015	1.838	0
XM_001928861.1	MUSTN1	darkturquoise	grey60	958.6	61.979	0.99397	0.000562	565.862	--
XM_001929110.1	ZFYVE27	blue	grey60	968.4	61.195	0.99146	0.000946	11.394	0.196
XM_001928164.1	NUP133	blue	grey60	950.5	63.05	0.99742	0.000157	8.012	0.039
XM_001925079.1	ARID5B	turquoise	grey60	947.9	63.262	0.99816	9.49E-05	12.518	0.585
XM_001924558.1	TRAFD1	turquoise	grey60	920.3	61.479	0.99233	0.000806	8.584	0
XM_001925356.1	RNF10	midnightblue	grey60	960.7	60.451	0.98887	0.001408	44.912	0.43
XM_001924460.1	CNOT7	midnightblue	grey60	961.3	60.795	0.99002	0.001195	50.348	0
XM_001926406.1	ACOT9	blue	grey60	977.8	61.075	0.99086	0.001048	34.47	0
NM_214223.2	THOP1	blue	grey60	916.1	62.507	0.99573	0.000335	4.636	0
AY421755.1	HMOX2	blue	grey60	918.7	61.001	0.99072	0.001071	3.3	--
NM_213911.1	PGRMC1	turquoise	grey60	979.1	58.674	0.98262	0.002744	64.316	0
AY609773.1	OTUB1	greenyellow	grey60	964.2	62.039	0.99411	0.000542	5.726	99
AY609821.1	TMEM208	turquoise	grey60	980.5	62.862	0.99686	0.000211	12.87	99
NM_001038007.1	SFRS1	green	grey60	990.9	61.828	0.99343	0.000639	77.798	99
BX674049		pink	grey60	866.8	60.6	0.9894	0.001307	2.966	--
BX675793		orange	grey60	986.9	62.703	0.99635	0.000264	5.414	--
BP158663		brown	grey60	932.5	62.16	0.99457	0.00048	16.424	99

CK452102	turquoise	grey60	886.6	60.057	0.98754	0.001666	45.718	--	
CK459106	black	grey60	799.3	57.012	0.97696	0.004183	12.886		99
CK460594	pink	grey60	975.9	60.576	0.98913	0.001358	17.394	--	
BX918046	lightcyan	grey60	983.4	59.243	0.98468	0.002271	24.074	--	
BX918145	black	grey60	814.8	56.743	0.97601	0.004444	9.968	--	
BX919338	black	grey60	846.8	59.222	0.98467	0.002272	8.148		99
BX922019	turquoise	grey60	976.3	61.616	0.99264	0.000757	16.28	--	
CN164027	midnightblue	grey60	906.6	62.234	0.99482	0.000447	31.818	--	
CJ024433	brown	grey60	969.8	60.215	0.98808	0.001559	18.94	--	
DN118663	blue	grey60	940.1	61.946	0.99378	0.000589	9.85	--	
BW979375	turquoise	grey60	925.3	62.814	0.99669	0.000228	4.464	--	
BW979917	yellow	grey60	988.9	58.406	0.98169	0.002965	12.09	--	
AJ962601	turquoise	grey60	906.8	62.034	0.99413	0.00054	2.552	--	
DV225844	greenyellow	grey60	965.3	61.107	0.99109	0.001008	54.14	--	
DB786410	red	grey60	926.7	61.484	0.99241	0.000793	10.68	--	
DT323640	blue	grey60	963.8	62.371	0.99523	0.000396	3.808	--	
EV889890	yellow	grey60	946.7	61.573	0.99265	0.000756	15.858	--	
EV897247	paleturquoise	grey60	991.6	59.469	0.98545	0.002101	15.488	--	
EW239772	pink	grey60	872.3	60.17	0.98794	0.001587	1.918	--	
EW471282	turquoise	grey60	979.2	59.799	0.9865	0.001879	14.098	--	
EW572411	turquoise	grey60	910.1	61.384	0.99206	0.000848	6.964	--	
EW580189	white	grey60	858.4	58.612	0.98267	0.002732	4.73	--	
EW594090	turquoise	grey60	909.6	59.998	0.98734	0.001707	5.522	--	
EW623428	black	grey60	880.4	60.279	0.98829	0.001519	22.626	--	

Table S11-4. Module eigengene and its evolutionary rates in 13 postnatal highly LT-specific modules (LT_lightgreen).

pig_Genebank	pig_Gene	LDEModule	LTModule	kTotal	kWithin	eigencorr	eigenpval	meanExpr	Ka/Ks
AK234182.1	SERHL2	pink	lightgreen	304.9	58.661	0.99036	0.001135	0.794	--
AK237852.1		white	lightgreen	296.6	60.577	0.99698	0.000199	5.428	--
AK237969.1		turquoise	lightgreen	292.2	60.472	0.9966	0.000238	21.056	--
AK238113.1		red	lightgreen	284.7	55.238	0.97823	0.003843	1.348	--
AK235023.1		red	lightgreen	290.9	60.452	0.99651	0.000248	1.884	--
AK231815.1		pink	lightgreen	283.9	60.405	0.99641	0.000258	0.252	--
AK231927.1		red	lightgreen	261.8	55.213	0.97809	0.003881	1.202	--
AK239218.1		turquoise	lightgreen	300.7	60	0.995	0.000424	7.096	--
AK236699.1	FSCN1	blue	lightgreen	291.2	58.913	0.99135	0.000965	3.794	99
XM_001924525.1	C18orf22	pink	lightgreen	292	54.603	0.97586	0.004485	41.624	99
XM_001924870.1	IBTK	pink	lightgreen	294.8	57.794	0.98733	0.001708	2.834	0.068
XM_001929668.1	SETDB1	pink	lightgreen	303	60.827	0.99779	0.000124	7.636	0
XM_001925622.1	HAPLN3	pink	lightgreen	300	60.534	0.99681	0.000216	4.538	0
NM_001130531.1	ADAM10	blue	lightgreen	303.5	56.259	0.98186	0.002924	3.26	99
NM_213791.1	MYL2	pink	lightgreen	290.8	57.898	0.98773	0.001629	4.344	--
AY610106.1	PLA2G7	pink	lightgreen	297.8	61.22	0.99912	3.13E-05	1.034	0.083
CB471233		red	lightgreen	291.9	60.071	0.9952	0.000398	2.244	--
CF179357		pink	lightgreen	306.1	59.671	0.99388	0.000575	6.976	--
CK454028		magenta	lightgreen	302.4	57.787	0.98729	0.001718	13.642	--
CK459217		pink	lightgreen	283.9	60.405	0.99641	0.000258	0.102	--
CK464272		lightyellow	lightgreen	291.4	57.643	0.9869	0.001796	7.628	--
BX915038		lightgreen	lightgreen	287.7	55.133	0.97793	0.003922	13.354	--
CN153338		white	lightgreen	306.3	58.996	0.99157	0.000927	58.408	--
CN153908		turquoise	lightgreen	274.1	58.799	0.99097	0.001029	1.528	--
CN159109		turquoise	lightgreen	253.8	53.726	0.97266	0.005404	8.634	--
CN167094		pink	lightgreen	289.8	58.953	0.9914	0.000957	1.67	--
CO939672		white	lightgreen	308.1	59.314	0.99263	0.000759	8.71	--
DN107792		green	lightgreen	291.8	59.075	0.99176	0.000897	11.84	--
DN124245		violet	lightgreen	301.1	57.947	0.98786	0.001603	2.36	--
DN124360		red	lightgreen	301.1	60.802	0.99769	0.000133	3.062	--
DN125831		midnightblue	lightgreen	280.9	56.354	0.98212	0.002861	13.56	--
DN129771		pink	lightgreen	280.4	54.171	0.97425	0.004942	4.154	--
AJ947061		red	lightgreen	291.4	58.007	0.98812	0.001552	2.204	--
AJ947409		pink	lightgreen	258	53.548	0.97191	0.005626	10.304	--
AJ949618		red	lightgreen	257.3	54.789	0.97663	0.004273	3.408	--
AJ949964		brown	lightgreen	287.2	60.236	0.99581	0.000326	13.064	--
AJ955864		red	lightgreen	278.9	56.706	0.98339	0.002563	6.338	--
AJ956044		white	lightgreen	273.1	55.524	0.9793	0.003565	2.382	--
AJ957673		navy	lightgreen	304.5	59.205	0.99224	0.000819	1.688	--
AJ960375		white	lightgreen	294.8	61.208	0.9991	3.22E-05	10.732	--
AJ960509		white	lightgreen	274.7	58.493	0.98986	0.001223	1.8	--
AJ940806		pink	lightgreen	270.4	56.249	0.98178	0.002943	8.334	--
CV875565		blue	lightgreen	279.1	56.671	0.98326	0.002594	2.596	--
DY426213		red	lightgreen	290.8	59.056	0.99186	0.000881	2.356	--
DB812092		red	lightgreen	288.7	59.933	0.99478	0.000452	2.11	--

DB788903	green	lightgreen	294.9	60.794	0.99771	0.000132	28.15	--	
DB805764	navy	lightgreen	299.7	60.999	0.99836	7.99E-05	1.3	--	
DV937193	darkgrey	lightgreen	293.1	60.252	0.9959	0.000314	0.978	--	
EV860684	pink	lightgreen	301.8	58.372	0.98935	0.001317	2.832	--	
EV867707	pink	lightgreen	306.2	59.838	0.99445	0.000496	1.862	--	
EW024219	white	lightgreen	275.6	55.365	0.97869	0.003723	9.202	--	
EW129342	violet	lightgreen	292.2	58.94	0.99132	0.000969	3.156	--	
EW143342	brown	lightgreen	302.1	60.009	0.99505	0.000418	4.352		0
EW150678	blue	lightgreen	291.1	58.211	0.98881	0.001418	5.282	--	
EW293220	greenyellow	lightgreen	259.8	55.763	0.98015	0.003346	7.742	--	
EW419154	white	lightgreen	286.1	60.491	0.99667	0.00023	6.182	--	
EW457527	violet	lightgreen	277.3	58.955	0.99142	0.000953	1.444	--	
EW527078	white	lightgreen	302.8	59.514	0.99332	0.000655	8.032	--	
EW534339	brown	lightgreen	290.6	54.813	0.97653	0.0043	14.82	--	
EW537634	navy	lightgreen	302.5	55.912	0.9807	0.00321	0.866	--	
EW587403	purple	lightgreen	283.9	60.405	0.99641	0.000258	0.102	--	
EW633660	red	lightgreen	274.7	58.321	0.98925	0.001336	2.776	--	
EH006647	black	lightgreen	292.6	56.945	0.98429	0.002359	2.372	--	
AW656641	turquoise	lightgreen	287.2	59.981	0.99492	0.000435	2.836	--	
BF710522	lightcyan	lightgreen	266.6	56.207	0.98173	0.002957	1.84	--	
BM659726	pink	lightgreen	296	58.891	0.99118	0.000993	2.874	--	

Table S11-5. Module eigengene and its evolutionary rates in 13 postnatal highly LT-specific modules (LT_lightyellow).

pig_Genebank	pig_Gene	LDEModule	LTModule	kTotal	kWithin	eigencorr	eigenpval	meanExpr	Ka/Ks
NM_001044576.1	LOC733643	blue	lightyellow	852.4	59.973	0.98831	0.001515	15.828	0.075
AK231179.1	DDR1	black	lightyellow	836.8	59.141	0.98551	0.002089	15.838	--
AK231509.1		yellow	lightyellow	976.8	63.027	0.99835	8.05E-05	3.03	0.178
AK235325.1	SFRS2	midnightblue	lightyellow	999	59.705	0.98731	0.001712	249.932	0
AK235558.1		orange	lightyellow	990	62.652	0.99711	0.000186	9.398	0
AK235599.1		turquoise	lightyellow	993.3	62.422	0.99638	0.000261	28.626	99
AK232498.1		grey	lightyellow	993.6	61.511	0.99337	0.000647	6.636	--
AK232704.1		navy	lightyellow	972.6	63.258	0.9991	3.26E-05	1533.93	0.359
AK235982.1		midnightblue	lightyellow	979.3	63.171	0.9988	4.99E-05	12.236	0.504
AK236189.1		midnightblue	lightyellow	865	60.495	0.99009	0.001183	13.904	--
AK239620.1		brown	lightyellow	1001	60.795	0.99097	0.001029	22.308	--
AK240009.1		blue	lightyellow	866.3	60.545	0.99026	0.001153	8.184	--
AK240109.1		turquoise	lightyellow	987.1	62.995	0.99824	8.87E-05	64.456	--
AK234130.1		violet	lightyellow	874.7	60.659	0.99065	0.001083	3.18	0.302
NM_001032376.2	HPRT1	turquoise	lightyellow	970.3	63.303	0.99924	2.53E-05	20.716	--
NM_001101824.1	SBAB-707F1.11	turquoise	lightyellow	973.4	63.252	0.99907	3.41E-05	16.432	99
XM_001929334.1	LOC100155784	violet	lightyellow	905.3	62.18	0.99563	0.000347	5.54	99
XM_001926377.1	LOC100154317	turquoise	lightyellow	997.1	61.851	0.99448	0.000492	96.44	0.134
XM_001929654.1	LOC100153061	darkgreen	lightyellow	963.3	63.324	0.99931	2.17E-05	19.302	0
XM_001925252.1	LOC100155091	blue	lightyellow	961.6	63.184	0.99887	4.54E-05	15.102	0
XM_001928638.1	LOC100155796	darkorange	lightyellow	944.5	63.127	0.99869	5.66E-05	4.084	0
XM_001927577.1	LOC100154266	violet	lightyellow	969.1	63.383	0.9995	1.36E-05	31.654	0.08
XM_001927602.1	LOC100156140	turquoise	lightyellow	956.4	63.086	0.99857	6.46E-05	22.242	0
XM_001928156.1	LOC100156794	darkgrey	lightyellow	996.8	61.634	0.99377	0.000589	6.44	0.125
XM_001924775.1	LOC100154322	darkred	lightyellow	1002	61.178	0.99224	0.000819	33.352	0
XM_001927107.1	LOC100157894	white	lightyellow	999.8	61.793	0.9943	0.000516	5.538	--
AY216477.1	GLUL	tan	lightyellow	976.5	62.475	0.99663	0.000235	42.086	--
NM_213914.1	FAAH	violet	lightyellow	973.7	63.426	0.99963	8.61E-06	6.474	0.194
NM_213910.1	SERPINE1	darkturquoise	lightyellow	969.3	63.409	0.99957	1.06E-05	3.268	--
NM_214320.1	CDKN3	turquoise	lightyellow	922.4	62.634	0.9971	0.000187	8.312	99
NM_214361.1	MCL1	green	lightyellow	1002	60.866	0.99123	0.000984	117.71	99
AY609709.1		red	lightyellow	974.2	62.842	0.9978	0.000124	51.864	99
CF177183		pink	lightyellow	803.2	57.382	0.97941	0.003535	2.34	--
BX668759		blue	lightyellow	949.8	63.239	0.99905	3.52E-05	2.284	--
BX674823		red	lightyellow	972.4	62.844	0.99779	0.000125	213.61	0.088
BP143526		lightgreen	lightyellow	922	62.638	0.99712	0.000185	10.42	--
CK454645			lightyellow	950.7	63.209	0.99895	4.06E-05	3.224	0
CK455184		turquoise	lightyellow	946.9	62.866	0.99788	0.000117	30.572	--
CK459204		red	lightyellow	985	62.485	0.99658	0.00024	7.77	--
CK466682		green	lightyellow	867.1	60.408	0.9898	0.001235	1.81	--
BX923234		violet	lightyellow	975.8	63.198	0.9989	4.40E-05	2.946	0
BX924202		turquoise	lightyellow	911.8	61.765	0.99433	0.000512	24.158	--
CN156236		darkturquoise	lightyellow	998.9	60.821	0.99105	0.001014	1.902	99
CN156513		black	lightyellow	895.9	61.773	0.9943	0.000517	2.808	--
CN156728		orange	lightyellow	946.9	63.283	0.99919	2.79E-05	3.548	0.791

CN162101		lightgreen	lightyellow	989.5	62.4	0.99633	0.000267	9.964	--
CJ020677		blue	lightyellow	965.2	63.034	0.99841	7.64E-05	9.104	99
DN101782	HO2	black	lightyellow	987	63.12	0.99862	6.13E-05	36.352	0
DN115314		blue	lightyellow	903.8	61.238	0.99262	0.00076	31.58	--
DN116629		green	lightyellow	1001	60.088	0.9886	0.001458	21.05	--
DN124718		blue	lightyellow	893.4	61.425	0.99318	0.000676	7.096	0
AJ957573		darkgrey	lightyellow	959.8	62.653	0.99721	0.000177	16.868	--
AJ963878		pink	lightyellow	995	62.667	0.99715	0.000183	21.938	--
DV226247		turquoise	lightyellow	941.4	63.218	0.99898	3.93E-05	17.05	--
DV227358		blue	lightyellow	988.6	62.305	0.996	0.000304	4.494	--
DY406144		black	lightyellow	838.6	58.937	0.98483	0.002238	7.842	--
EW038837		pink	lightyellow	997	62.437	0.9964	0.00026	9.51	--
EW125499		salmon	lightyellow	995.6	62.394	0.99628	0.000273	12.456	0
EW138865		blue	lightyellow	951.9	63.243	0.99906	3.44E-05	10.402	--
EW148325		white	lightyellow	931.8	62.944	0.9981	9.90E-05	11.978	--
EW190518		blue	lightyellow	972.2	63.506	0.99988	1.62E-06	6.906	--
EW357862		red	lightyellow	948.5	63.269	0.99914	3.02E-05	2.512	--
BI184060		turquoise	lightyellow	977.5	62.999	0.99827	8.61E-05	3.126	0
BI401840		pink	lightyellow	898.9	61.193	0.99246	0.000785	3.808	--
BQ604420		turquoise	lightyellow	938	62.894	0.99796	0.000111	5.138	--
CA779520		turquoise	lightyellow	941.8	63.23	0.99901	3.71E-05	3.244	--

Table S11-6. Module eigengene and its evolutionary rates in 13 postnatal highly LT-specific modules (LT_magenta).

pig_Genebank	pig_Gene	LDEModule	LT Module	kTotal	kWithin	eigencorr	eigenpval	meanExpr	Ka/Ks
NM_001044606.1	ATP5O	darkgreen	magenta	258.99	71.063	0.96696	0.007175	315.914	0.1786
AK230796.1	PAFAH1B1	tan	magenta	205.09	64.981	0.94872	0.013831	20.168	--
AK234327.1	TSC22D1	tan	magenta	265.18	81.917	0.99855	6.66E-05	26.018	99
AK240551.1	DAP	darkred	magenta	267.87	75.429	0.9798	0.003435	6.328	99
AK234571.1	MRPL12	darkred	magenta	262.22	81.949	0.99902	3.71E-05	93.782	--
AK234671.1		pink	magenta	291.13	54.827	0.90937	0.032305	1.248	--
AK234756.1		darkred	magenta	245.9	75.963	0.98149	0.003016	2.278	--
AK238051.1	TOX	salmon	magenta	242.79	75.517	0.97992	0.003404	0.838	--
AK238120.1	LOC100130153	paleturquoise	magenta	261.54	82.224	0.99943	1.61E-05	4.538	--
AK235272.1	PJA1	paleturquoise	magenta	231.13	65.183	0.94982	0.013392	1.936	0.2562
AK232127.1	MLF1	lightcyan	magenta	242.59	74.195	0.9767	0.004253	72.732	0
AK238391.1		darkturquoise	magenta	242.04	72.559	0.97141	0.005777	4.038	--
AK235604.1	CMTM3	tan	magenta	212.3	61.549	0.93631	0.019109	3.838	--
AK235806.1	FUNDC2	lightcyan	magenta	277.1	76.521	0.98333	0.002578	19.134	99
AK235923.1	COQ9	darkmagenta	magenta	257.29	77.114	0.98658	0.001862	226.978	0
AK236330.1	ANXA4	darkred	magenta	244.25	76.267	0.98213	0.00286	3.43	0.1941
AK239272.1	ADA	grey	magenta	209.18	67.569	0.95573	0.011107	5.856	0.2632
AK236398.1	RNASEK	salmon	magenta	266.63	79.591	0.99185	0.000882	7.458	0
AK233669.1	CABC1	lightgreen	magenta	287.27	55.803	0.91327	0.03026	544.134	0
AK233777.1	LOC100291183	violet	magenta	219.77	69.869	0.96232	0.008731	81.068	--
AK237193.1	LOC100287741	darkred	magenta	244.68	77.673	0.98623	0.001935	3.798	--
NM_001113439.1	SFRS18	lightcyan	magenta	275.29	78.413	0.98902	0.001379	48.158	99
XM_001927231.1	RAB13	turquoise	magenta	253.79	75.148	0.97984	0.003426	4.618	0
XM_001926198.1	PFDN5	salmon	magenta	271.36	77.114	0.98477	0.002251	27.72	0
XM_001926121.1	MRPL52	salmon	magenta	250.58	77.127	0.98667	0.001843	14.434	--
XM_001927225.1	COX17	salmon	magenta	270.18	79.541	0.99262	0.00076	61.502	--
XM_001928724.1	IFIT1L	turquoise	magenta	241.04	76.36	0.98418	0.002383	21.684	0.6742
XM_001927332.1	AGA	tan	magenta	207.81	64.009	0.94335	0.016047	3.6	0.4132
AJ000786.1	TMPRSS11F	turquoise	magenta	279.57	73.639	0.97462	0.004835	29.632	--
NM_213898.1	ACADS	turquoise	magenta	259.29	77.199	0.98586	0.002014	92.02	0.1354
NM_214116.1	MGP	turquoise	magenta	260.29	78.553	0.98885	0.00141	197.894	0
NM_001001539.1	AKR1B1	turquoise	magenta	209.6	67.885	0.95726	0.010537	63.718	0
AY609804.1	VMA21	darkred	magenta	252.73	77.373	0.9855	0.002092	4.536	99
AY610147.1	LOC730107	turquoise	magenta	270.75	78.867	0.99078	0.001061	6.68	99
AY610185.1	HSPB11	turquoise	magenta	293.5	52.487	0.89965	0.037579	0.968	99
NM_001009582.1	STMN1	magenta	magenta	225.92	68.122	0.95772	0.010369	19.554	99
NM_001008689.2	SAR1B	tan	magenta	198.34	62.273	0.93748	0.01859	116.54	99
BX666142		salmon	magenta	248.97	77.198	0.98512	0.002173	5.668	99
BX666730		salmon	magenta	277.62	54.522	0.90976	0.032096	7.496	--
BX667181		tan	magenta	257.88	75.766	0.98118	0.003091	2.776	--
BX670664		tan	magenta	223.92	71.255	0.96892	0.006547	17.684	--
BX675880		black	magenta	264.27	79.37	0.99134	0.000967	13.762	--
BX675945		tan	magenta	245.27	77.152	0.98515	0.002168	23.61	--
BX676412		darkred	magenta	255.92	78.57	0.98894	0.001395	12.678	--
BX915349		paleturquoise	magenta	243.34	78.852	0.99043	0.001123	2.286	--
BX921221		salmon	magenta	209.63	65.314	0.95019	0.013245	51.11	--

BX923253		orange	magenta	239.89	78.093	0.98829	0.001519	7.222	--
BX923548		lightgreen	magenta	280.66	74.67	0.9776	0.004012	13.41	0.2026
BX923655		darkred	magenta	292.84	56.822	0.91683	0.028431	42.864	--
CN162158		tan	magenta	223.89	70.383	0.96394	0.008175	3.984	--
CN162187		tan	magenta	289.25	51.566	0.89639	0.039409	22.016	0
AJ656502		salmon	magenta	207.79	64.126	0.94703	0.014517	3.676	--
AJ657091		tan	magenta	196.4	60.193	0.93233	0.020914	1.58	0
CJ011098		orange	magenta	183.83	55.715	0.91807	0.027802	2.878	--
DN101483		salmon	magenta	242.62	78.775	0.99038	0.001131	5.924	--
DN108333		brown	magenta	238.51	56.776	0.92188	0.0259	26.986	--
DN115959		turquoise	magenta	273.72	75.891	0.98233	0.002813	3.594	0
DN121893		darkmagenta	magenta	273.23	78.01	0.9875	0.001675	6.886	99
DN124126		tan	magenta	203.3	59.878	0.93421	0.020058	9.714	--
DN125721		paleturquoise	magenta	269.68	80.472	0.99508	0.000413	4.248	--
DN128580		salmon	magenta	261.78	75.417	0.98083	0.003176	60.152	--
DN132970		paleturquoise	magenta	265.41	79.488	0.99276	0.000739	9.304	--
BW981299		turquoise	magenta	242.14	77.955	0.9871	0.001755	4.032	--
BW982846		turquoise	magenta	259.62	82.211	0.99966	7.69E-06	3.018	--
AJ964366		skyblue	magenta	226.28	73.917	0.97582	0.004496	7.472	--
AJ944603		turquoise	magenta	275.29	76.69	0.98383	0.002462	7.172	--
AJ946247		tan	magenta	211.28	62.506	0.94201	0.016615	6.202	--
CV876445		turquoise	magenta	246.81	77.523	0.98579	0.00203	4.09	--
DY424635		tan	magenta	297.23	51.124	0.89351	0.04104	11.138	0
DB809172		purple	magenta	190.66	60.554	0.93298	0.020616	3.526	--
DB803157		tan	magenta	240.61	75.268	0.97965	0.003475	38.758	--
AK230483	HLA-DQA1	yellow	magenta	208.3	66.246	0.95204	0.012517	20.37	--
ES447306		navy	magenta	178.69	55.681	0.9162	0.028751	6.522	--
EV857330		darkred	magenta	270.25	81.005	0.99605	0.000298	5.576	--
EV937609		darkred	magenta	249.65	76.957	0.98422	0.002373	7.066	--
EW136988		turquoise	magenta	269.66	79.819	0.99253	0.000775	3.846	--
EW164912		turquoise	magenta	268.12	78.275	0.98933	0.00132	10.326	--
EW190207		turquoise	magenta	228.78	73.669	0.97607	0.004428	5.154	--
EW201887		tan	magenta	270.71	79.867	0.99343	0.000639	2.862	--
EW206037		paleturquoise	magenta	248.66	79.409	0.99124	0.000983	4.032	--
EW235079		darkgreen	magenta	268.98	78.173	0.98777	0.001621	9.29	--
EW238451		darkred	magenta	207.42	66.675	0.95231	0.012413	35.978	--
EW266485		salmon	magenta	199.62	59.305	0.93172	0.021199	2.352	0
EW299345		tan	magenta	211.25	68.017	0.95655	0.0108	11.046	--
EW328139		turquoise	magenta	276.1	77.489	0.98639	0.001901	10.106	--
EW336642		salmon	magenta	224.56	70.856	0.9686	0.006648	0.692	--
EW363654		darkred	magenta	260.64	80.724	0.99501	0.000423	16.542	--
EW521970		darkred	magenta	270.14	79.291	0.99196	0.000865	6.026	--
EW572865		paleturquoise	magenta	267.95	81.61	0.99802	0.000106	59.074	99
EW588646		turquoise	magenta	267.63	78.868	0.98976	0.001242	6.788	--
BE233738		darkred	magenta	257.81	81.97	0.99907	3.39E-05	16.552	--
BF192577		salmon	magenta	261.94	82.241	0.99951	1.31E-05	1.798	--
BI400195		paleturquoise	magenta	266.6	80.535	0.99466	0.000468	16.77	--
BI182553		lightcyan	magenta	270.94	78.597	0.98927	0.001333	45.654	--
BI184334		pink	magenta	259.4	79.604	0.99183	0.000886	7.264	--
BM190542		darkred	magenta	239.22	75.524	0.97998	0.003391	2.596	--

Table S11-7. Module eigengene and its evolutionary rates in 13 postnatal highly LT-specific modules (LT_orange).

pig_Genebank	pig_Gene	LDEModule	LT Module	kTotal	kWithin	eigencorr	eigenpval	meanExpr	Ka/Ks
AK240479.1	UBAC1	grey60	orange	286.3	30.853	0.968767	0.006595	213.696	--
AK238619.1	PNRC1	tan	orange	348.3	22.108	0.896843	0.039151	92.03	--
AK235913.1	FHOD1	purple	orange	506.3	34.053	0.989849	0.001226	96.458	1.2135
AK238961.1	AES	blue	orange	330	32.466	0.977855	0.003943	100.144	--
AK239070.1	MED24	violet	orange	463.1	35.113	0.996433	0.000256	11.86	--
AK230500.1	DAZAP2	turquoise	orange	594.1	31.175	0.968266	0.006754	24.222	99
AK239729.1	TMEM100	yellow	orange	476.6	30.684	0.964257	0.008068	10.526	0
AK232969.1	CLASP1	salmon	orange	385.8	35.434	0.997503	0.00015	40.374	--
AK240094.1	HYI	lightcyan	orange	555.4	33.87	0.988008	0.001574	25.912	0
AK233469.1	AARSD1	lightcyan	orange	432.7	16.904	0.835546	0.078053	15.358	--
AK240159.1	CALCOCO2	navy	orange	440.1	35.171	0.99603	0.0003	20.988	0.0832
AK237308.1	ATP6AP1	green	orange	392.1	33.206	0.983461	0.002547	18.28	--
NM_001078687.1	SMPX	darkgrey	orange	347	34.366	0.989057	0.001372	173.502	--
NM_001113698.1	TH1L	lightcyan	orange	242	28.66	0.947785	0.01421	52.258	99
XM_001927866.1	RAD23B	darkgreen	orange	476.6	33.559	0.987032	0.001769	81.928	99
XM_001926740.1	MFSD3	darkgreen	orange	568.2	33.017	0.982011	0.002888	4.98	0
XM_001928014.1	GMPR2	red	orange	349.5	31.623	0.970099	0.006179	15.852	99
XM_001924615.1	PDLIM1	darkorange	orange	352.6	33.225	0.980918	0.003155	23.942	0
NM_214188.1	DDOST	green	orange	207.2	25.723	0.921092	0.026291	12.904	0
NM_214342.1	OXSR1	yellow	orange	402.6	32.081	0.97294	0.005322	13.848	0
AY609689.1	FH	yellow	orange	561.1	29.165	0.955172	0.011317	95.278	0.1362
AY609883.1	MRPL48	salmon	orange	301.8	30.386	0.966355	0.007371	3.552	0
AY610193.1	C7orf30	turquoise	orange	625.6	24.467	0.915192	0.029268	49.146	--
BX666495		white	orange	562.4	32.565	0.97801	0.003902	27.218	--
BX671690		greenyellow	orange	504.5	34.118	0.990385	0.00113	3.518	--
CK460216		brown	orange	572.6	32.957	0.98225	0.002831	91.478	--
BX926121		grey60	orange	278.9	29.627	0.960506	0.009366	25.64	--
CN156363		turquoise	orange	544.8	31.576	0.971464	0.005762	7.918	--
CN156474		purple	orange	303.9	32.472	0.978354	0.00381	11.562	--
DN115916		blue	orange	453.6	32.897	0.980664	0.003218	20.672	--
BW960627		yellow	orange	599.7	26.661	0.934699	0.019834	5.88	--
BW974788		blue	orange	391	17.194	0.839191	0.075515	34.6	0
AJ946843		white	orange	640.4	31.432	0.97064	0.006012	89.226	--
AJ954153		darkgreen	orange	279.7	29.562	0.955675	0.011128	34.946	--
DV901297		white	orange	442	34.596	0.991154	0.000997	59.002	--
DY414301		turquoise	orange	650.1	30.38	0.962648	0.008617	5.164	--
EV890051		yellow	orange	527	31.024	0.966417	0.00735	229.574	99
EV933515		orange	orange	217.1	27.237	0.935977	0.019258	31.512	--
EW041986		blue	orange	245.6	29.163	0.954676	0.011504	106.666	--
EW179701		darkturquoise	orange	248.4	29.012	0.948873	0.013771	4.096	--
EW270439		darkred	orange	238.4	27.208	0.940764	0.017152	16.836	--
EW299585		purple	orange	361.1	30.836	0.967156	0.00711	31.968	--
EW362700		darkorange	orange	261.6	30.635	0.965324	0.007711	91.89	--
BI181072		darkorange	orange	356	34.935	0.994348	0.00051	8.376	--

Table S11-8. Module eigengene and its evolutionary rates in 13 postnatal highly LT-specific modules (LT_red).

pig_Genebank	pig_Gene	LDEModule	LT Module	kTotal	kWithin	eigencorr	eigenpval	meanExpr	Ka/Ks
AK237425.1	AOC3	yellow	red	329.59	73.6241	0.91096	0.031465	22.494	--
AK230890.1	UBA3	yellow	red	616.99	93.5819	0.963067	0.008473	43.102	--
AK234461.1	LUC7L3	brown	red	465.53	82.7269	0.935321	0.019553	133.084	0.5285
AK240575.1	C11orf58	turquoise	red	606.59	105.49	0.989104	0.001363	34.718	99
AK231197.1	RPL36	purple	red	543.03	109.199	0.996743	0.000223	290.914	99
AK234577.1	TSFM	turquoise	red	679.79	101.592	0.980033	0.003377	38.958	--
AK231558.1	UQCRFS1	turquoise	red	631.66	105.145	0.987978	0.001579	659.306	0.5812
AK231604.1	LAP3	navy	red	623.11	93.4894	0.961142	0.009141	59.15	0
AK235252.1	MRPS35	blue	red	449.39	83.2272	0.936758	0.018909	19.668	0.3859
AK235434.1	EIF1B	turquoise	red	381.56	91.2418	0.957197	0.010562	37.732	99
AK235564.1	PRMT7	tan	red	444.17	100.375	0.97825	0.003838	14.096	0.2927
AK231959.1	CRIP2	blue	red	478.43	105.566	0.989193	0.001347	34.546	--
AK235782.1	RABAC1	grey60	red	382.26	90.5215	0.956063	0.010982	61.082	--
AK232557.1	CAST	purple	red	392.11	78.9708	0.926014	0.023888	10.42	--
AK236065.1	RAB14	brown	red	550.68	87.8012	0.948398	0.013962	29.51	99
AK239106.1	CRKL	salmon	red	449.26	102.112	0.982038	0.002882	60.56	--
AK230559.1	PICALM	lightcyan	red	346.83	77.0967	0.919953	0.026858	56.238	99
AK239509.1	PECR	turquoise	red	325.57	78.5902	0.925755	0.024013	23.264	99
AK239646.1	HADHB	turquoise	red	482.86	109.288	0.996928	0.000204	236.196	0
AK239728.1	DNAJB6	purple	red	624.36	95.2383	0.965279	0.007726	43.712	99
AK233231.1	NDUFS8	blue	red	370.88	85.7884	0.944304	0.015646	311.794	--
AK240107.1	MAP4	grey60	red	641.9	104.038	0.985272	0.002141	133.064	--
AK237044.1	UBE2Z	tan	red	559.96	100.913	0.979278	0.00357	10.674	0
AK237106.1	SLC25A12	green	red	424.67	102.223	0.982534	0.002764	32.406	99
AK240181.1	MAP1LC3A	darkred	red	513.6	104.742	0.987693	0.001636	183.398	99
AB292846.1	NME1-NME2	purple	red	425.36	98.1751	0.973615	0.005124	12.65	--
NM_001097426.1	DDO	turquoise	red	587.27	108.998	0.996158	0.000286	12.292	0.177
NM_001097486.1	ATPIF1	yellow	red	565.85	108.747	0.995612	0.000349	67.136	99
NM_001097469.1	HIGD2A	darkturquoise	red	311.97	75.8018	0.918038	0.027819	66.77	0.6964
NM_001097435.1	P4HA1	royalblue	red	480.02	93.4772	0.962145	0.008791	26.816	0.7591
NM_001113287.1	LDHB	navy	red	699.32	94.7022	0.96406	0.008135	221.662	0
XM_001926626.1	C9orf123	blue	red	598.45	105.59	0.989447	0.001299	9.484	--
XM_001926549.1	FERMT2	black	red	446.89	103.026	0.984366	0.002341	95.66	0
XM_001929410.1	ATP5B	greenyellow	red	484.87	109.634	0.997698	0.000133	867.136	0
XM_001925445.1	KIAA0528	blue	red	539.77	107.228	0.992871	0.000722	21.686	--
XM_001927180.1	ATP6V1E1	purple	red	442.15	103.4	0.984878	0.002227	18.88	0
XM_001928042.1	MED6	turquoise	red	632.6	99.4023	0.975238	0.00466	19.136	99
XM_001926123.1	GSTZ1	turquoise	red	323.19	74.4386	0.91451	0.029618	9.818	99
XM_001929246.1	MRPL2	turquoise	red	682.04	101.288	0.979307	0.003562	23.744	0.0637
XM_001927338.1	IDH3A	blue	red	647.64	100.761	0.9789	0.003668	221.372	0.176
XM_001925339.1	COL21A1	turquoise	red	408.08	86.1942	0.944252	0.015668	8.666	--
XM_001926876.1	IFT122	tan	red	489.41	107.95	0.994444	0.000497	4.638	99
XM_001929335.1	TAF5	grey60	red	593.3	101.317	0.979378	0.003544	15.932	--
XM_001928978.1	CASP7	darkorange	red	479.75	96.167	0.96854	0.006667	12.256	--

XM_001925275.1	DDIT4	tan	red	450.2	101.938	0.981368	0.003044	54.314	--
XM_001928504.1	GATC	grey60	red	637.62	93.9335	0.963267	0.008405	6.454	99
XM_001929273.1	C22orf13	turquoise	red	669.42	100.972	0.979248	0.003577	31.996	99
XM_001929590.1	KLHL22	grey60	red	371.73	70.8624	0.904606	0.034858	7.736	--
XM_001925965.1	APOOL	darkorange	red	517.9	110.213	0.998648	5.97E-05	11.792	0.3466
AY374470.1	ETFB	turquoise	red	429.7	104.559	0.98737	0.001701	49.288	0
AJ236938.1	LUC7L3	turquoise	red	322.92	73.65	0.909887	0.03203	32.97	--
NM_213923.1	APEH	turquoise	red	656.26	93.0536	0.96008	0.009517	14.528	0.0766
NM_213896.1	ACY1	turquoise	red	460.85	98.1623	0.97281	0.00536	27.86	0.1093
NM_214049.1	UCP3	lightgreen	red	441.02	103.799	0.985737	0.00204	15.944	0.0618
NM_214189.1	INHA	pink	red	452.42	94.2537	0.963846	0.008207	11.114	0
NM_214204.1	PKIA	grey60	red	436.05	105.77	0.989858	0.001224	313.884	--
NM_001001645.1	C19orf56	darkred	red	378.69	95.1264	0.96655	0.007307	51.248	99
AY609477.1	MRPS36	royalblue	red	521.47	110.738	0.999778	3.97E-06	71.29	--
AY609572.1	NDUFC2	white	red	560.59	105.907	0.990061	0.001188	121.252	--
AY609726.1	ARF6	purple	red	530.31	100.536	0.978045	0.003892	4.632	0
AY609765.1	NDUFV1	lightcyan	red	427.69	103.805	0.985589	0.002072	26.416	0
AY610069.1	ACOT13	turquoise	red	401.96	82.3605	0.93502	0.019689	28.168	99
AY610163.1	IFRD1	turquoise	red	470.56	77.574	0.922135	0.025775	9.598	0
CF787137		darkorange	red	485.94	109.702	0.997756	0.000128	315.204	99
BX668263		paleturquoise	red	479.44	109.246	0.996839	0.000213	9.21	--
BX668638		turquoise	red	397.93	99.0413	0.975632	0.00455	9.722	--
BX675592		grey60	red	447.53	98.066	0.973202	0.005245	18.298	--
BX675898		blue	red	486.25	97.5362	0.971671	0.005699	15.994	99
BX676378		purple	red	554.65	91.8124	0.957564	0.010427	68.984	99
BP440567		pink	red	725.44	90.8548	0.955142	0.011328	16.234	99
BP142174		turquoise	red	409.04	101.359	0.980497	0.00326	427.76	--
BP144484		darkgrey	red	487.21	98.5013	0.973584	0.005133	16.992	0.4075
CK452952		turquoise	red	564.93	103.625	0.985555	0.00208	34.46	--
CK453720		turquoise	red	401.85	93.7076	0.962638	0.00862	62.898	--
CK454638		yellow	red	563.83	99.4294	0.975781	0.004508	6.744	--
CK457989		turquoise	red	459.83	106.64	0.991624	0.000919	8.6	--
CK462660		navy	red	426.65	77.6273	0.924527	0.024606	14.42	0
CK463770		turquoise	red	605.9	103.143	0.983653	0.002503	29.272	--
CK467191		darkorange	red	601.14	107.319	0.992532	0.000774	31.366	--
BX914725		turquoise	red	750.7	79.3313	0.925022	0.024366	18.394	--
BX916011		turquoise	red	517.94	100.679	0.978627	0.003739	15.772	--
BX917182		skyblue	red	244.78	50.3855	0.834315	0.078915	15.136	--
BX917819		lightgreen	red	394.02	93.6763	0.962616	0.008628	21.986	--
BX921896		turquoise	red	488.58	103.706	0.985829	0.002021	29.382	--
BX923516		darkgreen	red	637.39	105.657	0.988864	0.001408	14.896	--
BX926350		brown	red	360.52	85.557	0.944172	0.015702	21.374	--
CN155050		grey60	red	434.85	87.085	0.948091	0.014086	2.716	--
CN158322		darkturquoise	red	370.85	92.244	0.960202	0.009474	30.29	--
CN162142		darkgreen	red	302.91	73.2572	0.909929	0.032008	6.092	--
CN162202		turquoise	red	402.97	76.2983	0.917639	0.02802	45.622	--
CN165277		turquoise	red	537.88	105.493	0.989306	0.001325	16.796	--
AJ656748		grey60	red	573.43	93.1993	0.962492	0.008671	69.23	--

CJ011361	turquoise	red	395.88	95.8181	0.967765	0.006914	28.666	0
CJ026063	turquoise	red	716.54	83.6792	0.93745	0.018602	69.234	--
CJ026797	tan	red	423.38	103.968	0.986109	0.001961	7.682	--
CJ028952	orange	red	591.58	108.179	0.994256	0.000522	12.628	--
DN102168	navy	red	604.02	108.616	0.995202	0.000399	32.576	--
DN102235	darkturquoise	red	525.87	84.9376	0.940583	0.01723	25.016	0
DN104419	lightgreen	red	666.12	97.5145	0.970798	0.005964	11.232	0
DN104456	purple	red	433.04	102.649	0.983362	0.00257	129.166	--
DN111525	purple	red	421.42	103.336	0.984821	0.00224	118.48	--
DN116503	purple	red	447.41	103.547	0.985149	0.002168	27.022	--
DN124866	darkred	red	666.07	101.38	0.980003	0.003384	11.522	--
DN125939	grey60	red	420.54	92.8566	0.961251	0.009103	22.928	0
AJ950507	turquoise	red	386.67	94.6126	0.965803	0.007552	30.218	--
AJ960206	pink	red	657.98	102.813	0.983227	0.002601	23.76	--
AJ962054	salmon	red	383.6	96.044	0.968726	0.006608	0.208	--
AJ962420	purple	red	405.48	97.955	0.973189	0.005249	1.802	--
AJ962558	darkgreen	red	596.44	84.2782	0.940463	0.017282	13.32	--
DV227364	turquoise	red	446.83	106.448	0.991246	0.000982	26.994	--
DV229656	grey60	red	430.97	87.0908	0.947458	0.014343	10.864	99
CX064653	midnightblue	red	709.07	95.0023	0.965042	0.007805	14.768	--
DY411595	darkturquoise	red	367.58	90.0479	0.954672	0.011506	56.602	99
DY415294	pink	red	619.79	94.6898	0.966136	0.007443	31.94	--
EV898729	midnightblue	red	596.26	90.6615	0.95469	0.011499	7.282	--
EV984595	purple	red	713.27	95.4905	0.96597	0.007497	105.844	--
EW079103	darkred	red	475.48	101.101	0.979654	0.003473	40.432	--
EW176591	purple	red	686.16	88.4297	0.949087	0.013685	1.022	--
EW198996	darkturquoise	red	532.46	96.4782	0.968776	0.006592	33.782	--
EW239364	turquoise	red	525.06	106.059	0.990126	0.001176	30.402	--
EW252113	grey60	red	663.29	102.703	0.982978	0.002659	9.24	--
EW333024	pink	red	531.29	105.368	0.9884	0.001497	31.924	--
EW366014	white	red	626.7	106.154	0.989897	0.001217	33.57	--
EW373211	grey60	red	695.67	85.0005	0.939973	0.017495	5.178	--
EW421594	turquoise	red	451.05	105.806	0.989923	0.001213	91.6	99
EW495920	paleturquoise	red	509.01	109.994	0.998444	7.37E-05	247.67	0
EW671244	yellow	red	294.42	68.5054	0.895663	0.039817	8.572	--
AW414891	blue	red	485.6	98.9288	0.974597	0.004842	16.726	--
BI182094	tan	red	405.65	87.2187	0.948022	0.014114	12.152	--
BI183745	pink	red	534.03	109.18	0.996669	0.000231	1.434	--
BI402468	pink	red	563.78	98.8987	0.975267	0.004652	7.304	--

Table S11-9. Module eigengene and its evolutionary rates in 13 postnatal highly LT-specific modules (LT_sienna3).

pig_Genebank	pig_Gene	LDEModule	LT Module	kTotal	kWithin	eigencorr	eigenpval	meanExpr	Ka/Ks
AK230724.1	MTHFD2	turquoise	sienna3	885.06	25.0755	0.99184	0.000884	23.766	99
AK235285.1	HDLBP	brown	sienna3	996.32	25.4324	0.994685	0.000465	116.038	--
AK238947.1	FSTL4	pink	sienna3	927.54	23.1195	0.976321	0.004358	2.134	--
AK236153.1	C1QBP	turquoise	sienna3	972.74	25.8454	0.997887	0.000117	138.358	--
AK236404.1	HNRNPUL1	yellow	sienna3	965.83	25.9492	0.998704	5.60E-05	26.816	99
AK239524.1	FKBP5	lightgreen	sienna3	962.5	25.9292	0.998554	6.60E-05	55.616	--
AK239677.1	WIPI2	blue	sienna3	989.72	25.9517	0.998669	5.83E-05	28.858	0
AK236724.1	TSSC1	turquoise	sienna3	983.06	25.8549	0.99793	0.000113	14.646	0
NM_001001546.2	MBP	lightgreen	sienna3	779.99	22.7133	0.972174	0.005549	5.35	--
XM_001928451.1	KLF10	navy	sienna3	971.22	23.2389	0.976713	0.004251	15.93	0.431
XM_001925495.1	GPHN	salmon	sienna3	919.34	25.633	0.996229	0.000278	18.714	0
XM_001926227.1	PSMA4	turquoise	sienna3	995.97	25.364	0.994064	0.000549	82.714	0
XM_001926112.1	C20orf7	violet	sienna3	999.72	24.4937	0.987146	0.001746	8.882	0.174
NM_214114.1	JAK1	greenyellow	sienna3	967.04	26.0435	0.999415	1.70E-05	87.804	0
NM_214418.1	SLC25A6	blue	sienna3	959.97	26.018	0.999208	2.67E-05	36.814	--
AY609865.1	C3orf26	turquoise	sienna3	934.06	25.8051	0.997593	0.000142	6.686	99
AY610104.1	MED30	blue	sienna3	984.33	26.0055	0.99911	3.19E-05	11.082	0
CF179281		yellow	sienna3	985.02	26.0117	0.999135	3.05E-05	15.44	--
BX673234		turquoise	sienna3	976.22	25.8601	0.998007	0.000107	19.346	--
CK462749		navy	sienna3	958.96	25.975	0.998888	4.45E-05	18.52	99
BX915715		turquoise	sienna3	819.17	23.5621	0.979497	0.003513	6.578	--
CN165379		turquoise	sienna3	960.18	26.0823	0.999709	5.96E-06	9.342	--
AJ650064		darkgreen	sienna3	997.56	25.2344	0.993028	0.000698	10.914	--
DN109838		darkorange	sienna3	903.73	25.1741	0.992671	0.000752	5.576	--
DN112619		yellow	sienna3	948.96	25.7602	0.997291	0.000169	13.826	--
AJ948686		pink	sienna3	997.49	25.3733	0.994134	0.000539	82.726	--
DY411090		navy	sienna3	885.95	24.8686	0.990368	0.001133	11.92	--
DY411251		darkgrey	sienna3	974.65	25.9924	0.999026	3.65E-05	4.628	--

Table S11-10. Module eigengene and its evolutionary rates in 13 postnatal highly LT-specific modules (LT_skyblue).

pig_Genebank	pig_Gene	LDEModule	LT Module	kTotal	kWithin	eigencorr	eigenpval	meanExpr	Ka/Ks
AK230984.1	SEC62	magenta	skyblue	158.2	21.416	0.908692	0.032663	122.188	--
AK230996.1	SCFD1	turquoise	skyblue	200.61	29.772	0.977567	0.00402	33.306	0
AK234533.1	UBXN4	skyblue	skyblue	133.05	21.12	0.903914	0.035234	107.052	0
AK237795.1	CCM2	turquoise	skyblue	134.34	24.277	0.934058	0.020125	63.48	0.095
AK234909.1	NUB1	magenta	skyblue	193.57	28.326	0.96578	0.00756	58.084	--
AK232067.1	MRPL45	royalblue	skyblue	285.14	30.753	0.981894	0.002917	102.95	--
AK235860.1	CDK9	navy	skyblue	385.28	24.382	0.923699	0.025009	32.598	0
AK233082.1	MCTS1	paleturquoise	skyblue	138.47	25.688	0.946564	0.014709	30.648	--
AK233149.1	H2AFJ	magenta	skyblue	196.25	30.244	0.980971	0.003142	29.952	--
XM_001928879.1	DNAJC14	royalblue	skyblue	182.41	30.63	0.987173	0.001741	22.122	0
XM_001924251.1	MYF6	navy	skyblue	261.11	30.288	0.977752	0.00397	14.84	0
XM_001929228.1	IMP3	lightyellow	skyblue	208.15	28.247	0.96505	0.007802	174.49	0
XM_001924661.1	SORBS1	turquoise	skyblue	259.74	30.359	0.982862	0.002686	3.762	0.231
XM_001927802.1	ANXA7	yellow	skyblue	252.91	31.914	0.992973	0.000706	191.054	99
XM_001929407.1	ANAPC7	midnightblue	skyblue	191.65	28.969	0.971433	0.005771	7.474	0
XM_001925019.1	LOC388789	purple	skyblue	95.051	15.907	0.835781	0.077888	1.84	0
NM_001142666.1	TEAD4	blue	skyblue	168.87	28.984	0.973826	0.005063	19.042	0
AY166682.1	TMBIM6		skyblue	132.63	21.629	0.908613	0.032705	6.524	--
NM_213945.1	PLAU	lightyellow	skyblue	158.22	28.814	0.973236	0.005235	16.65	1.804
NM_213830.1	FOLR1	brown	skyblue	178.75	30.126	0.981908	0.002913	6.136	0.074
NM_214201.1	GPX1	navy	skyblue	382.6	27.701	0.957763	0.010354	100.962	--
NM_214333.1	RPN1	black	skyblue	465.35	22.955	0.913831	0.029968	51.702	0.115
NM_001004045.1	S100A11	green	skyblue	232.16	32.194	0.995984	0.000305	11.934	0
BX671974		brown	skyblue	154.95	24.315	0.937373	0.018636	17.88	--
BX675792		purple	skyblue	107.6	17.629	0.862718	0.059787	26.508	--
BP160266		brown	skyblue	297.47	27.923	0.956479	0.010827	29.658	--
CK454008		blue	skyblue	220.37	31.517	0.989849	0.001226	12.35	--
BX915041		magenta	skyblue	293.86	30.525	0.980927	0.003153	150.048	99
CN155564		midnightblue	skyblue	292.24	27.557	0.960036	0.009533	23.276	--
DN100939		yellow	skyblue	193.67	26.649	0.952993	0.012148	37.052	--
AJ951950		pink	skyblue	323.22	29.338	0.970831	0.005954	4.27	--
DY405541		lightgreen	skyblue	253.54	31.686	0.990419	0.001124	17.69	0
DY418504		orange	skyblue	236.45	30.175	0.978283	0.003829	20.854	--
EV886697		navy	skyblue	284.32	31.135	0.986946	0.001787	3.454	--
EW137538		violet	skyblue	362.61	27.376	0.959866	0.009593	16.734	--
EW140042		white	skyblue	180.88	28.542	0.97089	0.005936	7.794	--
EW164176		turquoise	skyblue	256.51	30.992	0.987984	0.001578	57.792	--
EW425085		royalblue	skyblue	393.52	25.567	0.943137	0.016138	20.15	--
EW529809		grey60	skyblue	330.41	25.381	0.939403	0.017743	7.336	--
EW605958		red	skyblue	131.61	19.521	0.887641	0.044441	1.17	--
EW620642		royalblue	skyblue	108.26	18.479	0.871648	0.054124	63.5	--
EW646525		royalblue	skyblue	183.37	28.906	0.973623	0.005122	54.09	--

Table S11-11. Module eigengene and its evolutionary rates in 13 postnatal highly LT-specific modules (LT_tan).

pig_Genebank	pig_Gene	LDEModule	LT Module	kTotal	kWithin	eigencorr	eigenpval	meanExpr	Ka/Ks
AK234190.1	TGIF1	green	tan	859.8	66.2259	0.99055	0.001101	13.238	99
AK234301.1	RPUSD1	darkgrey	tan	948.21	59.4798	0.966342	0.007375	8.424	0
AK234448.1	ACTR10	yellow	tan	956.61	63.5046	0.979963	0.003394	17.49	0
AK234779.1	RAB1A	turquoise	tan	878.89	58.16	0.963613	0.008287	64.818	99
AK231701.1	SNORA60	green	tan	968.1	62.1502	0.97545	0.0046	16.754	--
AK235733.1	B3GNT4	tan	tan	628.35	43.8202	0.904887	0.034706	29.846	--
AK238586.1	SCAMP2	turquoise	tan	971.16	58.4439	0.962695	0.008601	29.312	--
AK238867.1	UBXN7	turquoise	tan	971.59	66.8404	0.991471	0.000944	13.51	--
AK236069.1	RRN3	green	tan	887.61	66.6943	0.991716	0.000904	16.896	--
AK239104.1	PRPF8	blue	tan	868.72	60.4809	0.972055	0.005584	36.388	0
AK236235.1	ACP2	blue	tan	946.07	61.1264	0.971961	0.005612	9.444	0.171
AK236339.1	PSMD2	blue	tan	954.04	68.7753	0.998495	7.00E-05	96.72	99
AK236564.1	SPNS1	yellow	tan	816.92	62.7948	0.978958	0.003652	14.748	0
AK232979.1	MRPS5	blue	tan	870	63.2854	0.980977	0.00314	18.478	0
AK233005.1	ADPRHL2	blue	tan	897.41	66.8972	0.992596	0.000764	11.506	0
AK236660.1	CSNK1E	black	tan	859.04	65.4552	0.987944	0.001586	14.702	0
AK233221.1	MRPL54	violet	tan	887.36	67.3853	0.994395	0.000503	27.338	--
AK236998.1	HMGN2	darkgrey	tan	697.28	49.8637	0.933007	0.020605	4.57	--
[truncated: 197,226 more chars]
